# Benefits and harms of drug treatment for type 2 diabetes: systematic review and network meta-analysis of randomised controlled trials

**DOI:** 10.1136/bmj-2022-074068

**Published:** 2023-04-06

**Authors:** Qingyang Shi, Kailei Nong, Per Olav Vandvik, Gordon H Guyatt, Oliver Schnell, Lars Rydén, Nikolaus Marx, Frank C Brosius, Reem A Mustafa, Arnav Agarwal, Xinyu Zou, Yunhe Mao, Aminreza Asadollahifar, Saifur Rahman Chowdhury, Chunjuan Zhai, Sana Gupta, Ya Gao, João Pedro Lima, Kenji Numata, Zhi Qiao, Qinlin Fan, Qinbo Yang, Yinghui Jin, Long Ge, Qiuyu Yang, Hongfei Zhu, Fan Yang, Zhe Chen, Xi Lu, Siyu He, Xiangyang Chen, Xiafei Lyu, Xingxing An, Yaolong Chen, Qiukui Hao, Eberhard Standl, Reed Siemieniuk, Thomas Agoritsas, Haoming Tian, Sheyu Li

**Affiliations:** 1 Department of Endocrinology and Metabolism, Division of Guideline and Rapid Recommendation, Cochrane China Centre, MAGIC China Centre, Chinese Evidence-Based Medicine Centre, West China Hospital, Sichuan University, Chengdu, China; 2 Department of Medicine, Lovisenberg Diaconal Hospital, Oslo, Norway; 3 Department of Health Research Methods, Evidence and Impact, McMaster University, ON, Canada; 4 Forschergruppe Diabetes eV at the Helmholtz Centre, Munich-Neuherberg, Germany; 5 Department of Medicine K2, Karolinska Institutet, Stockholm, Sweden; 6 Clinic for Cardiology, Angiology, and Intensive Care Medicine, RWTH Aachen University, University Hospital Aachen, Aachen, Germany; 7 Division of Nephrology, University of Arizona College of Medicine Tucson, Tucson, AZ, USA; 8 Department of Internal Medicine, Division of Nephrology and Hypertension, University of Kansas, Kansas City, MI, USA; 9 Department of Medicine, McMaster University, Hamilton, ON, Canada; 10 Department of Orthopedics, Orthopedic Research Institute, West China Hospital, Sichuan University, Chengdu, China; 11 Digestive Disease Research Institute, Shariati Hospital, Tehran University of Medical Sciences, Tehran, Iran; 12 Department of Cardiology, Shandong Provincial Hospital affiliated to Shandong First Medical University, Jinan, China; 13 Evidence-Based Medicine Centre, School of Basic Medical Sciences, Lanzhou University, Lanzhou, China; 14 Department of Emergency Medicine, St Marianna University School of Medicine, Kawasaki, Japan; 15 West China School of Medicine, Sichuan University, Chengdu, China; 16 Department of Nephrology, National Clinical Research Centre for Geriatrics, West China Hospital, Sichuan University, Chengdu, China; 17 Center for Evidence-Based and Translational Medicine, Zhongnan Hospital of Wuhan University, Wuhan, China; 18 Evidence-Based Social Science Research Centre, School of Public Health, Lanzhou University, Lanzhou, China; 19 Evidence-Based Nursing Centre, School of Nursing, Lanzhou University, Lanzhou, China; 20 Department of Social Medicine and Health Management, School of Public Health, Lanzhou University, Lanzhou, China; 21 Department of Endocrinology and Metabolism, Chengdu Fifth People’s Hospital, Chengdu, China; 22 Evidence-Based Medicine Centre, Tianjin University of Traditional Chinese Medicine, Tianjin, China; 23 Department of Cardiovascular Surgery, West China Hospital, Sichuan University, Chengdu, China; 24 Department of Endocrinology and Metabolism, First People’s Hospital of Shuangliu District, Chengdu, China; 25 Department of Radiology, West China Hospital, Sichuan University, Chengdu, China; 26 School of Rehabilitation Science, McMaster University, Hamilton, ON, Canada; 27 Division of General Internal Medicine, Division of Clinical Epidemiology, University Hospitals of Geneva, Geneva, Switzerland

## Abstract

**Objective:**

To compare the benefits and harms of drug treatments for adults with type 2 diabetes, adding non-steroidal mineralocorticoid receptor antagonists (including finerenone) and tirzepatide (a dual glucose dependent insulinotropic polypeptide (GIP)/glucagon-like peptide-1 (GLP-1) receptor agonist) to previously existing treatment options.

**Design:**

Systematic review and network meta-analysis.

**Data sources:**

Ovid Medline, Embase, and Cochrane Central up to 14 October 2022.

**Eligibility criteria for selecting studies:**

Eligible randomised controlled trials compared drugs of interest in adults with type 2 diabetes. Eligible trials had a follow-up of 24 weeks or longer. Trials systematically comparing combinations of more than one drug treatment class with no drug, subgroup analyses of randomised controlled trials, and non-English language studies were deemed ineligible. Certainty of evidence was assessed following the GRADE (grading of recommendations, assessment, development and evaluation) approach.

**Results:**

The analysis identified 816 trials with 471 038 patients, together evaluating 13 different drug classes; all subsequent estimates refer to the comparison with standard treatments. Sodium glucose cotransporter-2 (SGLT-2) inhibitors (odds ratio 0.88, 95% confidence interval 0.83 to 0.94; high certainty) and GLP-1 receptor agonists (0.88, 0.82 to 0.93; high certainty) reduce all cause death; non-steroidal mineralocorticoid receptor antagonists, so far tested only with finerenone in patients with chronic kidney disease, probably reduce mortality (0.89, 0.79 to 1.00; moderate certainty); other drugs may not. The study confirmed the benefits of SGLT-2 inhibitors and GLP-1 receptor agonists in reducing cardiovascular death, non-fatal myocardial infarction, admission to hospital for heart failure, and end stage kidney disease. Finerenone probably reduces admissions to hospital for heart failure and end stage kidney disease, and possibly cardiovascular death. Only GLP-1 receptor agonists reduce non-fatal stroke; SGLT-2 inhibitors are superior to other drugs in reducing end stage kidney disease. GLP-1 receptor agonists and probably SGLT-2 inhibitors and tirzepatide improve quality of life. Reported harms were largely specific to drug class (eg, genital infections with SGLT-2 inhibitors, severe gastrointestinal adverse events with tirzepatide and GLP-1 receptor agonists, hyperkalaemia leading to admission to hospital with finerenone). Tirzepatide probably results in the largest reduction in body weight (mean difference −8.57 kg; moderate certainty). Basal insulin (mean difference 2.15 kg; moderate certainty) and thiazolidinediones (mean difference 2.81 kg; moderate certainty) probably result in the largest increases in body weight. Absolute benefits of SGLT-2 inhibitors, GLP-1 receptor agonists, and finerenone vary in people with type 2 diabetes, depending on baseline risks for cardiovascular and kidney outcomes (https://matchit.magicevidence.org/230125dist-diabetes).

**Conclusions:**

This network meta-analysis extends knowledge beyond confirming the substantial benefits with the use of SGLT-2 inhibitors and GLP-1 receptor agonists in reducing adverse cardiovascular and kidney outcomes and death by adding information on finerenone and tirzepatide. These findings highlight the need for continuous assessment of scientific progress to introduce cutting edge updates in clinical practice guidelines for people with type 2 diabetes.

**Systematic review registration:**

PROSPERO CRD42022325948.

## Introduction

People with type 2 diabetes face an elevated risk of cardiovascular and kidney disease, resulting in impaired quality of life and reduced life expectancy. In the light of increased recognition of these risks and the failure of intensive glycaemic control to provide substantial risk reduction, regulatory agencies and researchers have increasingly shifted away from a glucose centric paradigm. Instead, reductions in cardiovascular disease and chronic kidney disease are now, through new and effective treatment options, priority treatment objectives.^1^ Two classes of drugs, sodium glucose cotransporter-2 (SGLT-2) inhibitors and glucagon-like peptide-1 (GLP-1) receptor agonists, provide cardiovascular and kidney benefits, particularly in patients with established cardiovascular or kidney disease,^2^ with trustworthy guidelines providing recommendations stratified by baseline risks.^2^ ^3^

Recently, two novel agents have become available to treat patients with type 2 diabetes: finerenone, a non-steroidal mineralocorticoid receptor antagonist, and tirzepatide, a dual glucose dependent insulinotropic polypeptide (GIP)/GLP-1 receptor agonist. In two randomised trials looking at the cardiovascular outcomes of finerenone, findings suggested cardiovascular and kidney benefits in people with type 2 diabetes and chronic kidney disease,^4^ ^5^ while several randomised trials suggested benefits of tirzepatide in weight loss and quality of life.^6^ ^7^ ^8^ ^9^ ^10^ A recent large non-industry funded trial has provided insight regarding older drugs for diabetes treatment, including sulfonylureas and basal insulin, by comparing their long term cardiovascular effects with liraglutide, a GLP-1 receptor agonist.^11^ ^12^ ^13^

Clinicians now face the challenge of guiding their patients with type 2 diabetes in whether to add SGLT-2 inhibitors, GLP-1 receptor agonists, or finerenone and tirzepatide to their ongoing therapeutic regimens. Expanding on our previous large network meta-analysis^2^ that focused on SGLT-2 inhibitors and GLP-1 receptor agonists, this synthesis of the best current evidence on clinically relevant benefits and harms of all available drugs for people with type 2 diabetes included finerenone and tirzepatide, which are new to clinicians. Beyond informing a current update of the *BMJ* Rapid Recommendations for diabetes drugs, we designed this network meta-analysis to inform professional societies and healthcare systems in updating their health technology assessments, clinical practice guidelines, and other decision support.^2^ ^3^

## Methods

The taskforce of the guideline workshop (represented by OS, LR, NM, FCB, and ES), an international multidisciplinary team including endocrinologists, cardiologists, and nephrologists,^14^ helped to formulate the clinical questions and provided input for the study protocol. The reports of this study followed the PRISMA (preferred reporting items for systematic reviews and meta-analyses) 2020 and PRISMA network meta-analysis statement standards.^15^ ^16^ A protocol detailing predefined eligibility criteria, which differed slightly from the previously published network meta-analysis,^2^ was registered with PROSPERO (CRD42022325948).

### Eligibility criteria

Eligible randomised controlled trials compared drugs used to treat adults with type 2 diabetes. We considered the following drug classes: SGLT-2 inhibitors, GLP-1 receptor agonists, dipeptidyl peptidase-4 (DPP-4) inhibitors, thiazolidinediones, sulfonylureas, metformin, α-glucosidase inhibitors, meglitinides, insulins, dual GIP/GLP-1 receptor agonists, and non-steroidal mineralocorticoid receptor antagonists. Appendix 1.3 describes the detailed drug names and definitions of control arms (typically standard treatment at the time the trials were conducted, representing the treatment regimens the patient received before the clinician considered adding a new drug). Eligible trials had a follow-up of 24 weeks or longer. Trials systematically comparing combinations of more than one drug treatment class with no drug treatment, subgroup analyses of randomised controlled trials, and non-English language studies were deemed ineligible.

### Search strategy and information sources

We used comprehensive literature search strategies from previously published network meta-analyses in Ovid Medline, Embase, and Cochrane Central to 14 October 2022 (see appendix 1.1 for search strategies). The search added terms for non-steroidal mineralocorticoid receptor antagonists and dual GIP/GLP-1 receptor agonists. The search included reference lists of other identified systematic reviews evaluating cardiovascular and kidney outcomes associated with drugs of interest.

### Study selection

The study performed a pilot test for the study selection process before screening. Pairs of reviewers (QS, KNo, QF, ZQ, and FY) independently screened identified hits at the title and abstract and full text levels, with discrepancies resolved by a senior reviewer (SL).

### Data collection and data items

Using a standardised extraction form, the paired trained reviewers (QS, KNo, YM, QF, ZQ, XZ, XC, ZC, XL, and SH) independently extracted the following data:

- Study characteristics (year, countries, setting, funding, length of follow-up);
- Baseline characteristics of included participants (personal characteristics, number of participants, age, sex, body mass index, haemoglobin A1c (HbA1c), duration of diabetes, and complications or comorbidities including cardiovascular diseases, chronic kidney diseases, and obesity);
- Interventions (drug name, dose, frequency, and cointerventions); and
- Outcomes (trial specific definition, number of events and participants for binary outcomes, quality of life score change, and body weight change).

A senior reviewer (SL) resolved discrepancies. We prioritised study reported intention-to-treat results or modified intention-to-treat results over per protocol results.

### Risk-of-bias assessment

Pairs of reviewers independently assessed the risk of bias (QS, KNo, LG, YJ, YM, AAs, CZ, JPL, KNu, SRC, SG, YG, HZ, QiuY, XL, QinY, and XA). The Cochrane risk-of-bias tool, modified by the CLARITY group at McMaster University, informed risk-of-bias assessments^17^ for the following six domains: random sequence generation, allocation concealment, blinding to allocated interventions, missing outcome data, selective outcome reporting, and other concerns. Response options for each item were definitely yes (low risk of bias), probably yes, probably no, and no (high risk of bias). A third team (KNo, QinY, and QS) cross checked the pairs of assessments and summarised the final results, with residual discrepancies resolved by a senior reviewer (SL).

### Outcomes and effect measures

We judged the following outcomes as critical: all cause death, cardiovascular death, non-fatal stroke, end stage kidney disease, and amputation; and the following outcomes as important: non-fatal myocardial infarction, admission to hospital for heart failure, body weight change, health related quality of life, severe hypoglycaemia, severe gastrointestinal events, genital infection, ketoacidosis due to diabetes, and hyperkalaemia leading to admission to hospital. We evaluated the impact on end stage kidney disease using a composite of long term dialysis, kidney transplantation, a sustained estimated glomerular filtration rate <15 mL per minute per 1.73 m^2^, a sustained percentage decline in estimated glomerular filtration rate of at least 40% or a doubling of serum creatinine, or kidney related death.^18^ Appendix 1.2 details the definition of outcomes.

We measured the binary outcomes using odds ratios. For continuous outcomes, we measured health related quality of life as standardised mean difference, and body weight in kg as mean difference.

### Data synthesis

We conducted random effect network meta-analysis using a frequentist graph theoretical approach with the weighted least square estimator and Moore-Penrose pseudoinverse.^19^ In principle, we started with the assumption that relative effects were similar across drugs in the same class unless evidence indicated otherwise, and the network nodes are therefore in most cases grouped into drug classes based on their mechanisms. The sole exception in which evidence suggests the starting assumption is inaccurate is the impact of GLP-1 receptor agonists on body weight change.^20^ The analysis used the continuity correction to account for zero event by adding 0.5 to all cells of groups for the trials with at least one zero event.^21^ The global heterogeneity was evaluated with generalised methods of moments estimate of variance between studies and tested by the design based decomposition of Cochran’s Q statistic.^22^ We calculated indirect estimates from the network by node splitting and back calculation methods.^23^ For each network loop, we judged the local incoherence considering the clinical and statistical significance of the ratio of direct and indirect estimates. Comparison adjusted funnel plots evaluated global small study effects, which could reflect publication bias. We judged the intransitivity based on distribution comparisons of potential effect modifiers (ie, baseline age, sex, body mass index, HbA1c, the proportion of cardiovascular disease, and duration of diabetes) for each direct comparison and outcome, as well as meta-regressions of these parameters with the treatment effect for each drug and outcome. 

We performed sensitivity analyses, including a bayesian network meta-analysis adjusted by trial duration^24^; a Mantel-Haenszel fixed effect network meta-analysis for rare events^25^; a meta-analysis excluding trials with high risks of bias; a meta-analysis for end stage kidney disease that restricted the definition to a composite of long term dialysis, kidney transplantation, and death from kidney failure; a meta-analysis excluding phase 2 or phase 3 trials; and a meta-analysis pooling study reported hazard ratios for the trials with ≥2 years’ follow-up.

### Meta-regression

For trial and aggregated patient characteristics measured as continuous variables, we performed the following four meta-regressions:

- Proportion of patients with established cardiovascular diseases (hypothesising a larger relative effect in reducing death and cardiovascular and kidney outcomes in trials with a higher proportion of patients with cardiovascular diseases).
- Mean patients’ estimated glomerular filtration rate at baseline (hypothesising a larger relative effect in reducing death and cardiovascular and kidney outcomes in patients with lower estimated glomerular filtration rate).
- Mean patients’ body mass index at baseline (hypothesising a larger relative effect in reducing death and cardiovascular and kidney outcomes in patients with higher body mass index).
- Trial follow-up length (hypothesising a larger relative effect in reducing death and cardiovascular and kidney outcomes in studies with longer follow-up).

The credibility of any apparent subgroup effect (regression coefficient’s credible interval excludes null effect) was rated using the ICEMAN tool.^26^ If no credible subgroup effect was indicated, we assumed the constancy of relative effects across populations.

### GRADE certainty of evidence assessment

Following GRADE (grading of recommendations assessment, development and evaluation) guidance, evidence from direct comparisons started as high certainty evidence and could be rated down for risk of bias, inconsistency, indirectness, and publication bias.^27^ Evidence from indirect comparisons could be further rated down for intransitivity. A contribution matrix quantified the proportional contribution of each direct comparison with each indirect and network comparison using the random walk approach.^28^ The final certainty for network evidence was rated down for incoherence or imprecision.^29^ We rated imprecision following the GRADE guidance.^30^ When point estimates proved less than specified minimal important differences established by a previous guideline panel,^3^ we rated certainty in little or no effect, otherwise in non-zero effect (ie, null effect threshold). We rated down for imprecision by two levels when the 95% confidence interval crossed more than one threshold of importance (appendices 1.4 and 5).^31^

To categorise the relative impact of interventions, we chose the null effect as the decision threshold and standard treatments as the reference intervention.^32^ ^33^ We initially categorised treatments as different or not different from standard treatments, and subsequently as different or not different from at least one of those with an established difference from standard treatments. This process established five categories of interventions from among the best to among the worst. We then separated these drugs as high or moderate versus low or very low certainty of evidence according to the certainty of evidence relative to standard treatments.

### Absolute effect estimations

To better inform clinical decision making, we estimated the anticipated absolute effects of all drugs on the cardiovascular, kidney, and safety outcomes. If valid, we adopted baseline risk estimates applied in a clinical practice guideline that included a systematic review of risk prediction models.^34^ We calculated the absolute benefits (number of events per 1000 patients in five years) by applying the relative effects to the baseline risks in five tiers of adults with type 2 diabetes at varying risks of cardiovascular and kidney outcomes: three or fewer cardiovascular risk factors, more than three cardiovascular risk factors, established cardiovascular disease but not chronic kidney disease, established chronic kidney disease but not cardiovascular disease, and established cardiovascular disease and chronic kidney disease. For outcomes not included in the guideline we anticipated baseline risks by pooling the incidence rate in the control arm across trials via the random effect single arm meta-analysis (appendix 5.3), not further stratified by individual risk profiles (eg, risk of genital infections).

Given the complexity of presenting 9770 estimates of effect from this network meta-analysis, we elected to primarily present relative and absolute estimates of effect, certainty, and more detailed network meta-analysis results (eg, number of participants and trials for each comparison) through an interactive GRADE summary of findings table, the MATCH-IT tool (https://matchit.magicevidence.org/230125dist-diabetes). This tool also allows end users to compare any of the treatment options, including change of comparator (eg, finerenone *v* SGLT-2 inhibitors or GLP-1 receptor agonists for key cardiovascular and kidney outcomes).

### Patient and public involvement

For the outcome selection and importance rating as well as the minimal important difference for each outcome, this systematic review referred to a previous guideline and its company systematic review, where the patient partners informed their values and preferences.

## Results

### Study selection and study characteristics

**Fig 1:**
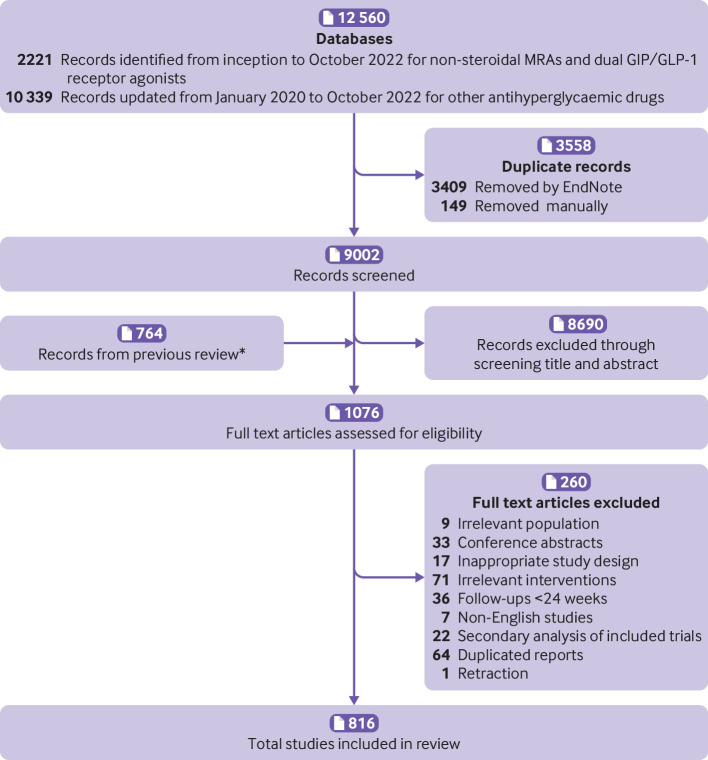
Flow diagram for trial screen and selection. MRA=non-steroidal mineralocorticoid receptor antagonists; GIP/GLP-1=glucose dependent insulinotropic polypeptide/glucagon-like peptide-1. *Previous review refers to reference 2

The 816 trials that proved eligible enrolled 471 038 participants with a typical mean age in the late 50s, over 50% men, with a mean body mass index of about 30, and a mean HbA1c of about 8.0%. About 60% of the participants had confirmed cardiovascular disease at baseline (fig 1, table 1, appendix 2.1, and appendix 2.2).

**Table 1:** Baseline characteristics of included trials and participants

Characteristic	No/median/pooled mean*	Interquartile or 95% CI	Range or 95% PI
**Study settings (of eligible studies)**			
Total No of trials	816	—	—
No of participants	471 038	—	—
Follow-up (months)**†**	6.0	5.5 to 12.0	5.5 to 212.0
**Study characteristics (of participants)**			
Age (years)**‡**	57.7	57.4 to 58.1	47.6 to 68.0
No of men (%)‡	56.6	55.8 to 57.5	34.1 to 76.7
Body mass index‡	29.5	29.3 to 29.8	22.7 to 36.4
HbA1c (%)**‡**	8.1	8.1 to 8.2	6.5 to 9.7
Cardiovascular disease (%)**‡ **	58.9	40.9 to 74.9	0.0 to 100.0
Duration of diabetes (years)*	7.4	5.2 to 10.1	0.0 to 20.7

CI=confidence interval; HbA1c=haemoglobin A1c; PI=prediction interval.

* Pooled mean was estimated using the single mean/proportion meta-analyses via a random effect model.

† Data are median, interquartile, and range.

‡ Data are pooled mean, 95% confidence interval, and 95% prediction interval.

### Risk of bias, global inconsistency, global publication bias, intransitivity, and incoherence

Of the 816 trials, 223 proved at high risk of bias for at least one of six domains, most commonly because of lack of blinding (62%), missing outcome data (26%), and allocation concealment (25%) (appendix 3). The evidence did not suggest global publication bias and intransitivity for any outcome (appendix 4.7), nor did the results suggest relevant global inconsistency or incoherence in outcomes except for health related quality of life, body weight change, and amputation (appendices 4.4, 4.5, and 4.6).

### Comparative effectiveness of drugs

**Fig 2:**
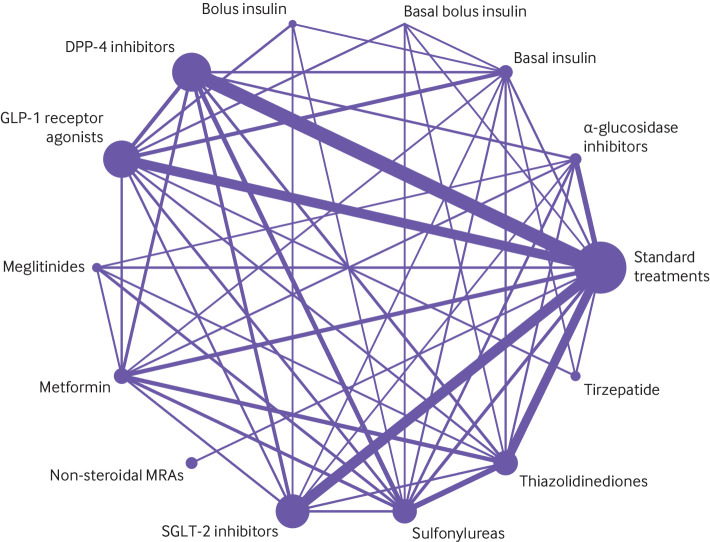
Network plot for all included studies, by drug treatments. Drug treatments were grouped by their drug classes. Network plots consist of the drug nodes with node size being proportional to the sample size and the comparison edges with line thickness being proportional to the number of trials. MRA=non-steroidal mineralocorticoid receptor antagonists; GLP-1=glucagon-like peptide-1; SGLT-2=sodium glucose cotransporter-2; DPP-4=dipeptidyl peptidase-4

**Fig 3:**
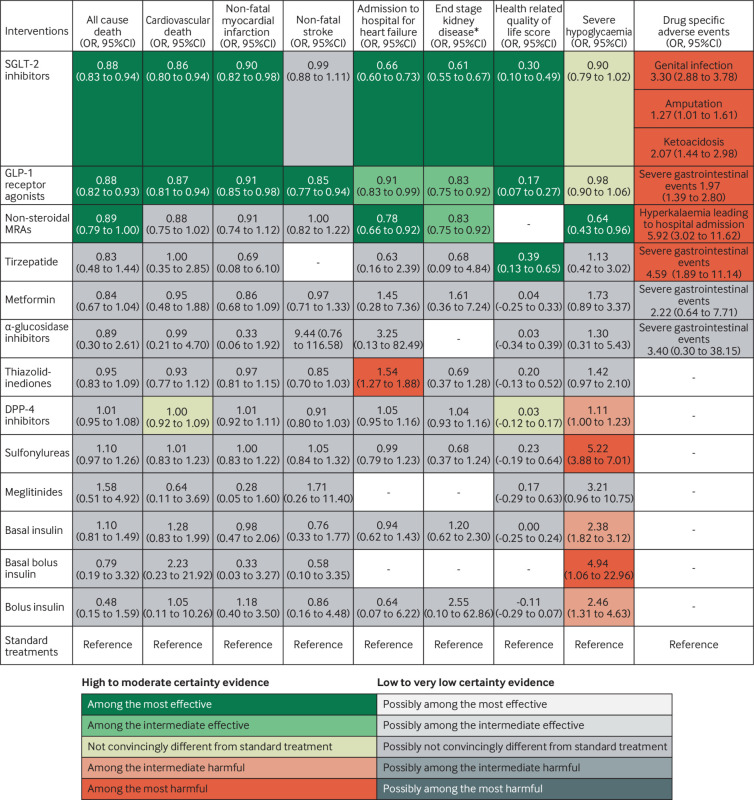
Benefits and harms of drug treatments for type 2 diabetes. Figure shows benefits and harms of the drugs for diabetes with the estimates that represent the comparative effects of the drugs compared with standard treatments. The GRADE (grading of recommendations, assessment, development, and evaluations) approach was used with a null effect threshold to rate and categorise drugs from among the most effective to among the most harmful. Any 95% confidence intervals touching but not crossing the decision threshold (ie, the null effect), were not rated down for imprecision. Drugs that were superior to (or inferior to) standard treatments (ie, point estimate exceeding (or falling below) the null effect and the 95% confidence interval not crossing) were first categorised into the most effective group (or the most harmful group). Drugs among the most effective (or most harmful) but inferior to (ie, point estimate falling below and 95% confidence interval not crossing) at least one drug in that group were then categorised into the intermediate effective group (or the intermediate harmful group). Non-steroidal mineralocorticoid receptor antagonists (MRAs) mainly refer to finerenone. *End stage kidney disease was defined as a composite of a long term dialysis, kidney transplantation, sustained estimated glomerular filtration rate <15 mL per min per 1.73 m^2^ for ≥30 days, sustained percent decline in estimated glomerular filtration rate of at least 40% for ≥30 days or a doubling of serum creatinine, or renal death; effects on end stage kidney disease were rated down owing to indirectness. CI=confidence interval; GLP-1=glucagon-like peptide-1; OR=odds ratio; SGLT-2=sodium glucose cotransporter-2

**Fig 4:**
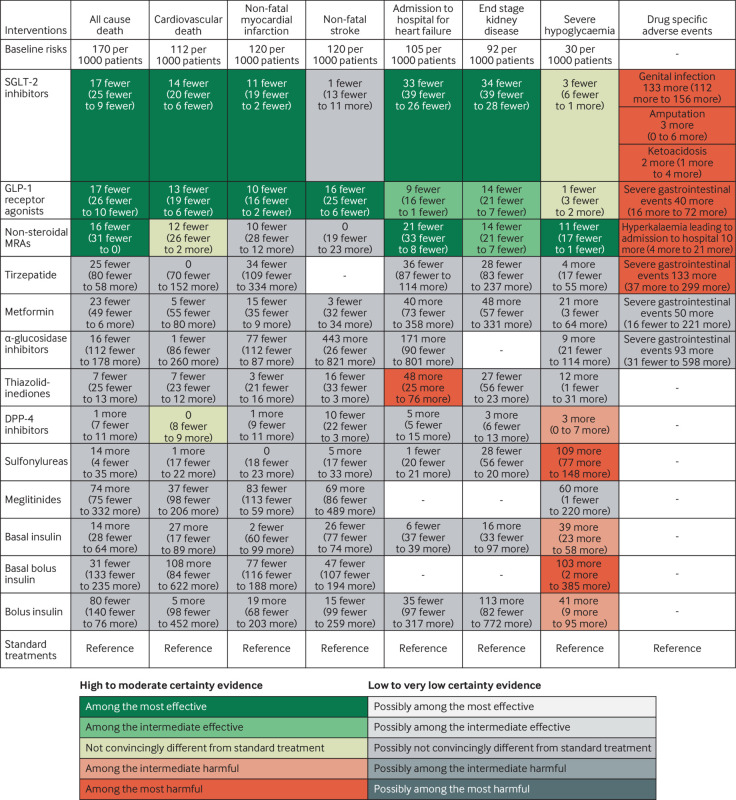
Anticipated absolute effects for patients with type 2 diabetes and chronic kidney disease, by drug treatment. Figure shows absolute benefits and harms of the drugs for patients with type 2 diabetes and chronic kidney disease. Estimates represent risk differences per 1000 patients in five years compared with standard treatments. Absolute effects were anticipated by applying the relative effects to the baseline risks adopted from a previous guideline panel. Figure is restricted to adults with type 2 diabetes and chronic kidney disease as an example, with the full populations in appendix 6 and the online tool (https://qingys.shinyapps.io/data_visualization) or the MATCH-IT tool (https://matchit.magicevidence.org/230125dist-diabetes). Non-steroidal mineralocorticoid receptor antagonists (MRAs) mainly refer to finerenone. GLP-1=glucagon-like peptide-1; SGLT-2=sodium glucose cotransporter-2

**Fig 5:**
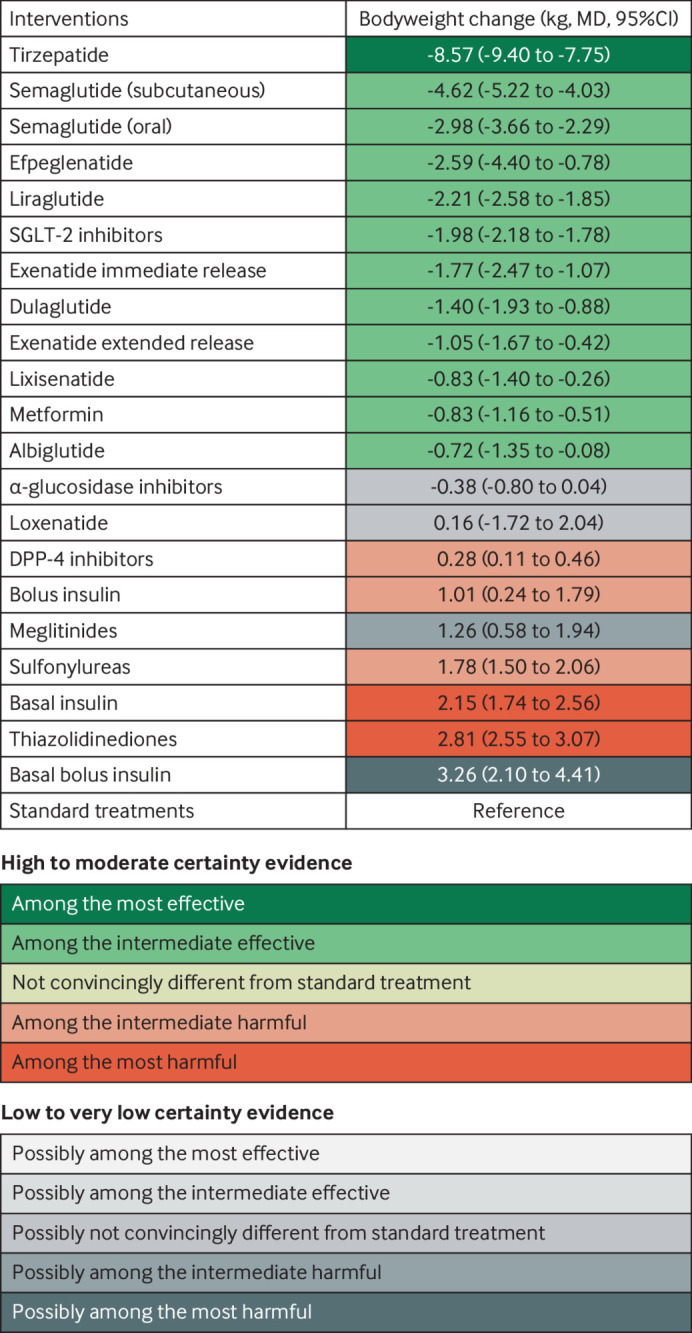
Body weight impact of drug treatment for type 2 diabetes by drug treatment. Figure shows body weight changes of the drugs for diabetes with the estimates that represent the comparative effects of the drugs compared with standard treatments. The GRADE (grading of recommendations, assessment, development, and evaluations) approach was used with a null effect threshold to rate and categorise drugs from among the most effective to among the most harmful. Any 95% confidence intervals touching but not crossing the decision threshold (ie, the null effect) were not rated down for imprecision. Drugs that were superior to (or inferior to) standard treatments (ie, point estimate exceeding (or falling below) the null effect and the 95% confidence interval not crossing) were first categorised into the most effective group (or the most harmful group). Drugs among the most effective (or the most harmful) but inferior to (ie, point estimate falling below and 95% confidence interval not crossing) at least one drug in that group were then categorised into the intermediate effective group (or the intermediate harmful group). CI=confidence interval; DPP-4=dipeptidyl peptidase-4; MD=mean difference; SGLT-2=sodium glucose cotransporter-2

Figure 2 shows the network plot with connections between each drug in all included trials for any outcome, appendix 4.1 shows other network plots for each outcome, and appendix 4.3 shows the network estimates with certainty of evidence for each comparison for each outcome. Appendix 5 details the assessments of GRADE certainty of evidence for the direct, indirect, and network comparisons. Figure 3 shows the comparative benefits and harms (excluding weight change, see below) of all drugs of interest through their relative estimates of effect, categorised from the most effective to the most harmful, taking certainty of evidence into account. Figure 4 illustrates the anticipated absolute benefits and harms of all drugs for adults with type 2 diabetes and chronic kidney disease (selected because randomised trials on finerenone were restricted to this population). Figure 5 shows the effects of these drugs on weight change.

 The MATCH-IT tool (https://matchit.magicevidence.org/230125dist-diabetes) provides an interactive view of the anticipated absolute effects for all populations at varying risks of cardiovascular and kidney outcomes. These data are also available in appendix 6 and - with even more details, in another interactive online tool (https://qingys.shinyapps.io/data_visualization). Below, we summarise the relative effects for cardiovascular, kidney, and harm outcomes for all drugs and provide examples of anticipated absolute effects for selected outcomes; all subsequent estimates refer to the comparison with standard treatments.

### All cause death and cardiovascular death

The analysis included 257 trials with 342 237 participants and 15 371 events for all cause mortality, and 144 trials with 275 679 participants and 9120 events for cardiovascular death. SGLT-2 inhibitors (odds ratio 0.88, 95% confidence interval 0.83 to 0.94; high certainty) and GLP-1 receptor agonists (0.88, 0.82 to 0.93; high certainty) reduce all cause mortality, and cardiovascular death (SGLT-2 inhibitors: 0.86, 0.80 to 0.94; GLP-1 receptor agonists: 0.87, 0.81 to 0.94; both high certainty). Non-steroidal mineralocorticoid receptor antagonists probably reduce all cause mortality (0.89, 0.79 to 1.00; moderate certainty) and possibly reduce cardiovascular death (0.88, 0.75 to 1.02; low certainty). Metformin possibly reduces all cause mortality (0.84, 0.67 to 1.04; low certainty) and might have little or no effect on cardiovascular death. DPP-4 inhibitors probably have little or no effect on cardiovascular death (moderate certainty). Sulfonylureas possibly increase all cause mortality (low certainty) and might have little or no effect on cardiovascular death. Other drugs might have little or no or uncertain effect on mortal outcomes (low to very low certainty; fig 3 and appendix 5).

### Non-fatal myocardial infarction and non-fatal stroke

This analysis included 209 trials with 293 042 participants and 8906 events for non-fatal myocardial infarction, and 178 trials with 283 728 participants and 4878 events for non-fatal stroke. SGLT-2 inhibitors reduce non-fatal myocardial infarction (odds ratio 0.90, 95% confidence interval 0.82 to 0.98; high certainty)—as, probably, do GLP-1 receptor agonists (0.91, 0.85 to 0.98; moderate certainty) and, possibly, metformin (0.86, 0.68 to 1.09; low certainty). GLP-1 receptor agonists are the only drug class that convincingly reduces non-fatal stroke (0.85, 0.77 to 0.94; high certainty). Other drugs might have little or no or uncertain effects on non-fatal myocardial infarction or stroke, relative to standard treatments (low to very low certainty; fig 3 and appendix 5).

### Admission to hospital for heart failure

The analysis included 142 trials with 252 055 participants and 6681 events. SGLT-2 inhibitors (odds ratio 0.66, 95% confidence interval 0.60 to 0.73; high certainty) decrease admission to hospital for heart failure as, probably, do GLP-1 receptor agonists (0.91, 0.83 to 0.99; moderate certainty) and finerenone (0.78, 0.66 to 0.92; moderate certainty). SGLT-2 inhibitors and finerenone are among the most effective drugs in this regard and SGLT-2 inhibitors are probably superior to GLP-1 receptor agonists (moderate certainty). Thiazolidinediones probably increase admission to hospital due to heart failure (1.54, 1.27 to 1.88; moderate certainty). Metformin and other drugs might have little or no effect, or uncertain effects (low or very low certainty; fig 3 and appendix 5).

### End stage kidney disease

The analysis included 54 trials with 209 754 participants and 6972 events. Compared with standard treatments, SGLT-2 inhibitors (odds ratio 0.61, 95% confidence interval 0.55 to 0.67; moderate certainty), GLP-1 receptor agonists (0.83, 0.75 to 0.92; moderate certainty), and finerenone (0.83, 0.75 to 0.92; moderate certainty) probably reduce end stage kidney disease. We rated down the certainty of evidence to moderate owing to indirectness, a result of our composite outcome of end stage kidney disease driven by variable reporting of kidney outcomes in the trials. SGLT-2 inhibitors are among the most effective drugs and are possibly superior to GLP-1 receptor agonists and finerenone (low certainty). Other drugs might have little or no effect, or uncertain effects on end stage kidney disease, relative to standard treatment (very low to low certainty; fig 3 and appendix 5).

### Health related quality of life

We analysed 33 trials with 18 588 participants using 13 types of questionaries (appendix 1.2). SGLT-2 inhibitors, GLP-1 receptor agonists, and tirzepatide probably improve health related quality of life with standardised mean differences ranging from 0.17 to 0.39 (moderate certainty), which did not surpass the minimal important difference (1.7 to 3.9 points in the 36-item short form survey; minimal important difference 10 points). DPP-4 inhibitors probably have little or no effect, and other drugs might have little or uncertain impact on health related quality of life (low or very low certainty; fig 3 and appendix 5).

### Body weight change

We analysed 531 trials with 279 118 participants. Figure 5 shows that tirzepatide is the most effective drug for reducing body weight (mean reduction 8.57 kg, 95% confidence interval 7.75 to 9.40), followed by individual GLP-1 receptor agonists, SGLT-2 inhibitors (class effect), and metformin with intermediate effects (mean reduction, range 4.62 to 0.72 kg), all high to moderate certainty). Two classes of drugs probably have the biggest effect size in increasing body weight: thiazolidinediones (2.81 kg, moderate certainty) and basal insulin (2.15 kg, moderate certainty). A third, basal bolus insulin, may have a similar effect (increase 3.26 kg, low certainty). Another four drugs have intermediate effects on body weight: sulfonylureas probably increase body weight by 1.78 kg (moderate certainty), meglitinides may increase body weight by 1.26 kg (low certainty), bolus insulin probably increases body weight by 1.01 kg (moderate certainty), and DPP-4 inhibitors probably increase body weight minimally by 0.28 kg (moderate certainty). Other drugs might have little or no effect on body weight (low to very low certainty; fig 5 and appendix 5).

### Severe hypoglycaemia

We analysed 202 trials with 302 457 participants and 5595 events. Sulfonylureas (odds ratio 5.22, 95% confidence interval 3.88 to 7.01) and basal bolus insulin (4.94, 1.06 to 22.96) probably increase the risk of severe hypoglycaemic events (moderate certainty), with likely smaller increases in risk with basal insulin (2.38, 1.82 to 3.12), bolus insulin (2.46, 1.31 to 4.63), and DPP-4 inhibitors (1.11, 1.00 to 1.23), with and without the contamination of other treatments. Meglitinides and thiazolidinediones may increase the risk of severe hypoglycaemic events (low certainty). SGLT-2 inhibitors and GLP-1 receptor agonists do not increase the risk of severe hypoglycaemic events (high certainty). Finerenone is probably associated with fewer severe hypoglycaemia than the standard treatments (0.64, 0.43 to 0.96, moderate certainty). Other drugs might have little to no effect compared with standard treatments (low to very low certainty; fig 3 and appendix 5).

### Severe gastrointestinal events

We analysed 37 trials with 65 283 participants and 1661 events. Tirzapetide (odds ratio 4.59, 95% confidence interval 1.89 to 11.14) and GLP-1 receptor agonists (1.97, 1.39 to 2.80) probably increase the risk of severe gastrointestinal adverse events (moderate certainty). Other drugs might have little or no effects compared with standard treatments (low to very low certainty; fig 3 and appendix 5).

### Genital infection

We analysed 94 trials with 103 111 participants and 2396 events. SGLT-2 inhibitors increase genital infection (odds ratio 3.30, 95% confidence interval 2.88 to 3.78; high certainty). Sulfonylureas may reduce the risk of a genital infection (0.52, 0.36 to 0.75; low certainty). Other drugs might have little to no effect compared with standard treatments (low to very low certainty; fig 3 and appendix 5).

### Amputation

We analysed 18 trials with 107 503 participants and 1150 events. SGLT-2 inhibitors probably increase the risk of amputation (odds ratio 1.27, 95% confidence interval 1.01 to 1.61, moderate certainty); other drugs do not (high to very low certainty; fig 3 and appendix 5). With an estimated baseline risk of 1%, treatment with SGLT-2 inhibitors in 1000 patients for five years probably results in three additional amputations (95% confidence interval 0 to 6; fig 4 and appendix 6).

### Ketoacidosis due to diabetes

We analysed 36 trials with 138 322 participants and 265 events. SGLT-2 inhibitors increase the risk of ketoacidosis due to diabetes (odds ratio 2.07, 95% confidence interval 1.44 to 2.98; high certainty); other drugs do not (high to very low certainty; fig 3 and appendix 5). With an estimated baseline risk of 0.2%, treatment with SGLT-2 inhibitors in 1000 patients for five years probably results in two more events with ketoacidosis due to diabetes (95% confidence interval 1 to 4; fig 4 and appendix 6).

### Hyperkalaemia leading to admission to hospital

We analysed two trials with 12 999 participants and 71 events for the non-steroidal mineralocorticoid receptor antagonists, which probably increase the risk of hyperkalaemia leading to admission to hospital (odds ratio 5.92, 95% confidence interval 3.02 to 11.62; moderate certainty). With an estimated baseline risk of 0.2%, treatment with non-steroidal mineralocorticoid receptor antagonists in 1000 patients for five years probably results in 10 additional events (95% confidence interval 4 to 21; fig 4 and appendix 6).

### Subgroup analyses and sensitivity analyses

Our study did not identify any credible subgroup effects (appendix 7) and all sensitivity analyses confirmed the robustness of our findings (appendix 8).

## Discussion

### Principal findings

This network meta-analysis comprehensively summarises the benefits and harms of available drug treatments for type 2 diabetes, including the two recently available drugs finerenone and tirzepatide. Among all drug classes, SGLT-2 inhibitors, GLP-1 receptor agonists, and finerenone show benefits in reducing all cause mortality, admission to hospital due to heart failure (SGLT-2 inhibitors and—probably—finerenone are the most effective drug treatments), and end stage kidney disease (SGLT-2 inhibitors are the most effective drug treatments). Only GLP-1 receptor agonists convincingly reduce non-fatal stroke. SGLT-2 inhibitors, GLP-1 receptor agonists, and tirzepatide improve health related quality of life, but did not reach the threshold for minimal important differences, suggesting trivial effects. As illustrated in the MATCH-IT tool, the absolute benefits of these drugs vary greatly in people with type 2 diabetes depending on baseline risks for cardiovascular and kidney outcomes 2 3 (appendix 6). Trustworthy clinical practice guidelines should provide risk stratified recommendations that could differ in direction and strength, reserving these drugs to adults at elevated risk for cardiovascular and kidney outcomes.^3^

### Strengths and limitations

Our work represents the most comprehensive systematic review and network meta-analysis assessing all pertinent drug classes for type 2 diabetes treatment. An international multidisciplinary team shaped the study question and protocol, optimising its relevance to current clinical practice. Our study incorporated current and rigorous approaches to network meta-analysiss and GRADE assessment.^31^ ^32^ Results provide policy makers and healthcare professionals with quick access to summaries of the effectiveness and safety of all available drug classes for diabetes treatments.

Limitations of our systematic review and network meta-analysis are largely driven by the available evidence. Firstly, for many outcomes that are important to patients, we found low to very low certainty evidence for the oldest as well as the newest classes of drugs, including metformin, sulfonylureas, tirzepatide, and non-steroidal mineralocorticoid receptor antagonists. Secondly, this network meta-analysis cannot answer a highly relevant question: what are the benefits and harms of coadministration of SGLT-2 inhibitors, GLP1 receptor agonists, and finerenone? Nevertheless, observational studies and post hoc analyses of trials suggest cardiovascular and kidney benefits of the combination of SGLT-2 inhibitors, finerenone, and GLP-1 receptor agonists.^35^ ^36^ Thirdly, owing to sparse direct evidence and limited reporting of outcomes important to patients, we adopted a composite outcome definition for end stage kidney disease that included a surrogate component. This resulted in moderate certainty of evidence for the effects of all drugs on end stage kidney disease, given the inherent indirectness. Fourthly, this study did not consider the dose-response of each drug. In future studies it could be interesting to explore potential dose-response effects for drugs such as GLP-1 receptor agonists. Fifthly, the exclusion of all non-English language literature might introduce potential publication bias, which in this study was adjusted by trim-and-fill analyses.

### Clinical interpretation

Finerenone is the first non-steroidal mineralocorticoid receptor antagonist likely to reduce all cause mortality and admission to hospital for heart failure and improve kidney outcomes in people living with type 2 diabetes. Finerenone is an alternative to SGLT-2 inhibitors and GLP-1 receptor agonists in patients with concomitant chronic kidney disease, but results provide only indirect evidence for other populations.^4^ ^5^ A recent analysis, however, suggests that the relative effect on all cause mortality of finerenone is not associated with either baseline estimated glomerular filtration rate or urine albumin-creatinine ratio; thus, effects of finerenone could also apply to patients with type 2 diabetes without chronic kidney disease.^37^ ^38^ Regarding the use of finerenone in patients without diabetes or kidney disease, an ongoing cardiovascular outcome trial of participants with congestive heart failure with or without type 2 diabetes or chronic kidney disease (FINEARTS-HF) is expected to conclude in 2024 and will provide relevant evidence.^39^

Although finerenone results in a fivefold relative increase in hyperkalaemia leading to admission to hospital, the absolute numbers of admissions to hospitals were very low. The absolute effects of hyperkalaemia (10 additional events per 1000 patients treated for five years) are, for most patients with type 2 diabetes and chronic kidney disease, of less importance than the benefits of 16 fewer deaths, 21 fewer admissions to hospital for heart failure, and 14 fewer cases of end stage kidney disease. Nevertheless, for patients at elevated baseline risk of hyperkalaemia, clinicians should closely monitor serum potassium when prescribing finerenone. Such patients include those taking drug treatments that could elevate serum potassium such as angiotensin converting enzyme inhibitors, angiotensin receptor blockers, and angiotensin receptor-neprilysin inhibitors.^40^

Tirzepatide, the only dual GIP/GLP-1 receptor agonist currently available, improves quality of life and reduces body weight with a greater effect than any other drugs or drug classes. GLP-1 receptor agonists also reduce body weight, with semaglutide being the most effective drug in people with type 2 diabetes, as previously established in patients with overweight and obesity.^20^ Whereas tirzepatide might be particularly attractive for people with type 2 diabetes seeking body weight loss, we could not show its benefits for cardiovascular and kidney outcomes; these are being explored in an ongoing cardiovascular outcome trial with results expected in 2025.^41^ GLP-1 receptor agonists are safe except that an average of 40 patients per 1000 withdraw from these drug treatments because of severe gastrointestinal adverse events. Tirzepatide also causes 133 patients per 1000 to withdraw owing to severe gastrointestinal adverse events. These findings exemplify the need to carefully balance benefits and harms across all patient outcomes for new diabetes drugs, individualised to patients’ characteristics, values, and preferences.

Our results raise concerns about the use of some well established drugs for glucose lowering in adults with type 2 diabetes. In particular, sulfonylureas may increase all cause mortality (low certainty), and thiazolidinediones probably increase admission to hospital due to heart failure (moderate certainty). Clinicians should be cautious in prescribing these drugs, especially to those at higher baseline risks for such outcomes. An additional new finding in our review is that DPP-4 inhibitors, in addition to sulfonylurea and insulin, show a probable intermediate increased risk of severe hypoglycaemia (moderate certainty). Since laboratory studies do not support the hypoglycaemic risk caused by the monotherapy of DPP-4 inhibitors,^42^ for people receiving combined therapy, clinicians should consider reducing the dose of insulin or sulfonylureas when adding these drugs.

Consistent with previous systematic reviews,^2^ ^43^ ^44^ SGLT-2 inhibitors increase the risk of genital infection, ketoacidosis due to diabetes, and, probably, amputation. The minimal absolute increase we have estimated (two additional ketoacidoses and three additional amputations among 1000 people treated with SGLT-2 inhibitors for five years) warrants a trade-off against established cardiovascular and kidney benefits, again most pronounced in patients at increased cardiorenal risk. This finding is in line with the decision made in 2020 by the US Food and Drug Administration to remove the boxed warning for canagliflozin.

### Conclusions

Keeping pace with the growing number of published randomised trials in adults with type 2 diabetes, this network meta-analysis finds highly heterogeneous benefits and drug specific harms across 13 drug classes. Beyond confirming the substantial cardiovascular and kidney benefits of SGLT-2 inhibitors and GLP-1 receptor agonists—with absolute effects highly dependent on patient risk profiles—we find that finerenone, the drug recently made available, displays quite similar benefits to SGLT-2 inhibitors and GLP-1 receptor agonists. Tirzepatide shows superior benefits on weight loss than SGLT-2 inhibitors and GLP-1 receptor agonists. These results and other key findings of our comprehensive systematic review highlight the need for continuous assessment of scientific progress to introduce cutting edge updates in clinical practice guidelines for people with type 2 diabetes.

**What is already known on this topic**

Sodium glucose cotransporter-2 (SGLT-2) inhibitors and glucagon-like peptide-1 (GLP-1) receptor agonists have proven benefits in cardiovascular and kidney outcomes, with some notable differences in outcomes such as heart failure and stroke
Recent randomised trials report both cardiovascular and kidney benefits with finerenone, a novel non-steroidal mineralocorticoid receptor antagonist, and improvements in quality of life and weight loss with tirzepatide, a dual glucose dependent insulinotropic polypeptide (GIP)/GLP-1 receptor agonist

**What this study adds**

Compared with standard treatments, adding finerenone probably reduces all cause mortality, admission to hospital for heart failure, and end stage kidney disease, while adding tirzepatide could reduce body weight
Compared with standard treatments, findings indicate that adding SGLT-2 inhibitors or GLP-1 receptor agonists reduces all cause mortality, cardiovascular death, non-fatal myocardial infarction, admission to hospital for heart failure, and end stage kidney disease, while adding only GLP-1 receptor agonists reduces non-fatal stroke
Compared with standard treatments, adding metformin possibly reduces all cause mortality and non-fatal myocardial infarction, adding sulfonylureas possibly increases all cause mortality, and adding thiazolidinediones probably increases admission to hospital due to heart failure

## Data Availability

No additional data available.

## Web extra

Extra material supplied by authors

Web appendix: Supplementary appendix

## Notes

Contributors: QS and KNo contributed equally to this study. QS, SL, POV, GHG, OS, FCB, LR, NM, ES, QH, RAM, RS, TA, and HT conceived and designed the study. SL, POV, GHG, OS, FCB, LR, NM, ES, QH, RAM, AAg, and HT discussed and drafted the study protocol. QS, KNo, QF, ZQ, and FY screened and selected the articles. QS, KNo, YM, QF, ZQ, XZ, XC, ZC, XL, and SH extracted the data. QS, KNo, LG, YJ, YM, AAs, CZ, JPL, KNu, SRC, SG, YG, HZ, QiuY, XL, QinY, and XA assessed the risk of bias of included trials. QS analysed the data. QS, KNo, SL, and GHG rated and revised the GRADE certainty of evidence. QS, SL, POV, GHG, OS, FCB, LR, NM, ES, RAM, AAg, RS, YC, and TA interpreted the results. QS, SL, and GHG drafted the manuscript. QS, SL, GHG, POV, OS, FCB, LR, NM, ES, RAM, and AAg critically revised the manuscript. All authors contributed to revising the manuscript. All authors had full access to all the data in the study and had final responsibility for the decision to submit for publication. SL, POV, GHG, OS, FCB, LR, NM, ES, QH, RAM, RS, TA, and HT supervised the study. SL is the guarantor. The corresponding author attests that all listed authors meet authorship criteria and that no others meeting the criteria have been omitted.

The manuscript’s guarantors (SL) affirm that the manuscript is an honest, accurate and transparent account of the study being reported; that no important aspects of the study have been omitted; and that any discrepancies from the study as planned and registered have been explained.

Dissemination to participants and related patient and public communities: This study will support the update of a *BMJ* Rapid Recommendation (https://www.bmj.com/rapid-recommendations).

Provenance and peer review: Not commissioned; externally peer reviewed.
