# Supplementary material for: Benefits and harms of drug treatment for type 2 diabetes: systematic review and network meta-analysis of randomised controlled trials
Source: BMJ. 2023 Apr 6;381:e074068. doi: 10.1136/bmj-2022-074068 (PMC10077111; doi:10.1136/bmj-2022-074068)
Supplement: Supplementary file 1 — Web appendix: Supplementary appendix [file shiq074068.ww.pdf]

Supplementary appendix

Supplement to:

Benefits and harms of pharmacotherapy for type 2 diabetes: a systematic review and network meta-analysis of randomised controlled trials

Qingyang Shi, Kailei Nong, Per Olav Vandvik, Gordon H Guyatt, Oliver Schnell, Lars Rydén, Nikolaus Marx, Frank C Brosius III, Reem A Mustafa, Arnav Agarwal, Xinyu Zou, Yunhe Mao, Aminreza Asadollahifar, Saifur Rahman Chowdhury, Chunjuan Zhai, Sana Gupta, Ya Gao, João Pedro Lima, Kenji Numata, Zhi Qiao, Qinlin Fan, Qinbo Yang, Yinghui Jin, Long Ge, Qiuyu Yang, Hongfei Zhu, Fan Yang, Zhe Chen, Xi Lu, Siyu He, Xiangyang Chen, Xiafei Lyu, Xingxing An, Yaolong Chen, Qiukui Hao, Eberhard Standl, Reed Siemieniuk, Thomas Agoritsas, Haoming Tian, Sheyu Li

Table of contents

APPENDIX 1 SUPPLEMENT TO METHODS 4

1.1 SEARCH STRATEGY 4

1.2 DEFINITION OF OUTCOMES 25

1.3 SUMMARY OF INCLUDED INTERVENTIONS 27

1.4 DETAILS OF GRADE APPROACHES 28

APPENDIX 2 LIST OF INCLUDED STUDIES 30

2.1 STUDY SETTINGS 30

2.2 PATIENTS' CHARACTERISTICS 313

APPENDIX 3 RISK OF BIAS ASSESSMENTS 411

APPENDIX 4 SUPPLEMENT TO RESULTS 476

4.1 NETWORK PLOTS FOR THE OUTCOMES 476

4.2 FOREST PLOTS 489

4.3 LEAGUE TABLES 503

4.4 HETEROGENEITY (INCONSISTENCY) ASSESSMENTS 518

4.5 INTRANSITIVITY ASSESSMENTS 565

4.6 INCOHERENCE ASSESSMENTS 644

4.7 PUBLICATION BIAS ASSESSMENTS 684

4.8 BETWEEN-STUDY VARIANCE 745

APPENDIX 5 GRADE CERTAINTY OF EVIDENCE ASSESSMENTS 746

5.1 GRADE ASSESSMENT TABLE 746

5.2 PERCENTAGE CONTRIBUTION MATRIX 835

5.3 BASELINE RISK ESTIMATION 849

APPENDIX 6 ANTICIPATED ABSOLUTE EFFECTS 855

6.1 IN PATIENTS WITH 3 OR FEWER CARDIOVASCULAR RISK FACTORS 855
6.2 IN PATIENTS WITH MORE THAN 3 CARDIOVASCULAR RISK FACTORS 947
6.3 IN PATIENTS WITH ESTABLISHED CARDIOVASCULAR DISEASE 1038
6.4 IN PATIENTS WITH CHRONIC KIDNEY DISEASE 1129
6.5 IN PATIENTS WITH ESTABLISHED CARDIOVASCULAR DISEASE AND CHRONIC KIDNEY DISEASE 1220

APPENDIX 7 META-REGRESSION ANALYSES 1311

7.1 REGRESSION ANALYSES FOR PATIENTS' BODY MASS INDEX AT BASELINE 1311
7.2 REGRESSION ANALYSES FOR PROPORTION OF PATIENTS WITH ESTABLISHED CARDIOVASCULAR DISEASE AT BASELINE 1317
7.3 REGRESSION ANALYSES FOR PATIENTS' EGFR AT BASELINE 1321
7.4 REGRESSION ANALYSES FOR TRIAL'S FOLLOW-UP 1324
7.5 ICEMAN ASSESSMENTS 1332

APPENDIX 8 SENSITIVITY ANALYSES 1343

8.1 RESULTS OF BAYESIAN LOG TIME REGRESSION FOR BINARY OUTCOMES 1343
8.2 RESULTS OF FREQUENTIST SENSITIVITY META-ANALYSES 1371

APPENDIX 9 REFERENCE LIST FOR INCLUDED STUDIES 1439

Appendix 1 Supplement to methods

1.1 Search strategy

Search strategies for non-steroidal MRAs and dual GIP/GLP-1 receptor agonists from inception to October 2022

Embase (via OVID)

1 exp Diabetes Mellitus Type 2/ 309094

2 Diabetes Mellitus/ 646929

3 ((diabetes or diabetes mellitus or diabetic*) adj1 (type 2 or type II or type ii or non-insulin dependent or noninsulin dependent or adult onset or mature onset or late onset)).tw. 275418

4 NIDDM.tw. 8072

5 Diabetic Nephropathies/ 25921

6 (diabetic nephropath* or diabetic kidney disease).tw. 34660

7 or/1-6 964617

8 exp mineralocorticoid receptor antagonists/ 100683

9 (nonsteroidal mineralocorticoid receptor antagonist* or nonsteroidal mineralocorticoid receptor inhibitor* or nonsteroidal mineralocorticoid antagonist* or nonsteroidal mineralocorticoid inhibitor* or non-steroidal mineralocorticoid receptor antagonist* or non-steroidal mineralocorticoid receptor inhibitor* or non-steroidal mineralocorticoid antagonist* or non-steroidal mineralocorticoid inhibitor*).tw. 170

10 (Finerenone* or kerendia* or BAY-94-8868 or BAY948862 or BAY-948862 or BAY94-8862 or BAY 948862).tw. 331

11 (AZD9977 or AZD-9977 or AZD 9977).tw. 18

12 (Apararenone* or MT-3995 or MT3995 or MT 3995).tw. 24

13 (KBP5074 or KBP-5074 or KBP 5074).tw. 15

14 (Esaxerenone* or CS3150 or CS-3150 or CS 3150).tw. 95

15 glucose-dependent insulinotropic polypeptide/ 6290

16 (glucose-dependent insulinotropic polypeptide receptor inhibitors* or glucose-dependent insulinotropic polypeptide receptor agonist* or glucose-dependent insulinotropic polypeptide inhibitor* or glucose-dependent insulinotropic polypeptide agonist* or GIP receptor inhibitors* or GIP receptor agonist* or GIP inhibitors* or GIP agonist*).tw. 110

17 (tirzepatide* or LY3298176 or LY-3298176 or LY 3298176).tw. 229

18 or/8-17 107187

19 and/7,18 11695

20 randomized.ab. 839436

21 randomised.ab. 165959

22 placebo.ab. 338950

23 clinical trials as topic/ 90728

24 randomly.ab. 520538

25 trial.ti. 372236

26 or/20-25 1755185

27 animals/ not (humans/ and animals/) 1017743

28 26 not 27 1724949

29 and/19,28 1761

MEDLINE (via OVID)

1 exp Diabetes Mellitus Type 2/ 162040

2 Diabetes Mellitus/ 133185

3 ((diabetes or diabetes mellitus or diabetic*) adj1 (type 2 or type II or type ii or non-insulin dependent or noninsulin dependent or adult onset or mature onset or late onset)).tw. 156382

4 NIDDM.tw. 6775

5 Diabetic Nephropathies/ 28587

6 (diabetic nephropath* or diabetic kidney disease).tw. 21055

7 or/1-6 345031

8 (nonsteroidal mineralocorticoid receptor antagonist* or nonsteroidal mineralocorticoid receptor inhibitor* or nonsteroidal mineralocorticoid antagonist* or nonsteroidal mineralocorticoid inhibitor* or non-steroidal mineralocorticoid receptor antagonist* or non-steroidal mineralocorticoid receptor inhibitor* or non-steroidal mineralocorticoid antagonist* or non-steroidal mineralocorticoid inhibitor*).tw. 97

9 (Finerenone* or kerendia* or BAY-94-8868 or BAY948862 or BAY-948862 or BAY94-8862 or BAY 948862).tw. 171

10 (AZD9977 or AZD-9977 or AZD 9977).tw. 7

11 (Apararenone* or MT-3995 or MT3995 or MT 3995).tw. 9

12 (KBP5074 or KBP-5074 or KBP 5074).tw. 6

13 (Esaxerenone* or CS3150 or CS-3150 or CS 3150).tw. 62

14 glucose-dependent insulinotropic polypeptide/ 0

15 ((glucose-dependent insulinotropic polypeptide adj5 receptor inhibitors*) or (glucose-dependent insulinotropic polypeptide adj5 receptor agonist*) or (glucose-dependent insulinotropic polypeptide adj5 inhibitor*) or (glucose-dependent insulinotropic polypeptide adj5 agonist*) or (GIP adj5 receptor inhibitors*) or (GIP adj5 receptor agonist*) or (GIP adj5 inhibitors*) or (GIP adj5 agonist*)).tw. 229

16 (tirzepatide* or LY3298176 or LY-3298176 or LY 3298176).tw. 90

17 or/8-16 554

18 and/7,17 327

19 randomized controlled trial.pt. 577473

20 controlled clinical trial.pt. 95029

21 pragmatic clinical trial.pt. 2145

22 randomized.ab. 507551

23 randomised.ab. 103652

24 placebo.ab. 212881

25 clinical trials as topic/ 200361

26 randomly.ab. 334494

27 trial.ti. 238934

28 or/19-27 1373150

29 animals/ not (humans/ and animals/) 5017107

30 28 not 29 1253710

31 and/18,30 91

Cochrane library

#1 MeSH descriptor: [Diabetes Mellitus] this term only 10807

#2 ((diabetes or diabetic* or "diabetes mellitus") near/1 ("type 2" or "type ii" or "non-insulin dependent" or "non insulin dependent" or "noninsulin dependent" or "adult onset" or "mature onset" or "late onset")):ti,ab,kw 48592

#3 ("diabetes mellitus" not "insulin dependent diabetes mellitus"):ti,ab,kw 50542

#4 #3 and EMBASE 16625

#5 NIDDM:ti,ab,kw 1117

#6 ((diabetic next nephropath*) or "diabetic kidney disease"):ti,ab,kw 4687

#7 {or #1-#2, #4-#6} 70233

#8 ("nonsteroidal mineralocorticoid receptor antagonist*" or "nonsteroidal mineralocorticoid receptor inhibitor*" or "nonsteroidal mineralocorticoid antagonist*" or "nonsteroidal mineralocorticoid inhibitor*" or "non-steroidal mineralocorticoid receptor antagonist*" or "non-steroidal mineralocorticoid receptor inhibitor*" or "non-steroidal mineralocorticoid antagonist*" or "non-steroidal mineralocorticoid inhibitor*"):ti,ab,kw 36

#9 Finerenone:ti,ab,kw 93

#10 kerendia:ti,ab,kw 1

#11 ("BAY-94-8868" or BAY948862 or BAY-948862 or "BAY94-8862" or "BAY 948862"):ti,ab,kw 12

#12 (AZD9977 or AZD-9977 or "AZD 9977"):ti,ab,kw 14

#13 Apararenone:ti,ab,kw 3

#14 (MT-3995 or MT3995 or “MT 3995”):ti,ab,kw 11

#15 (KBP5074 or KBP-5074 or "KBP 5074"):ti,ab,kw 7

#16 Esaxerenone:ti,ab,kw 33

#17 (CS3150 or CS-3150 or “CS 3150”):ti,ab,kw 24

#18 "glucose-dependent insulinotropic polypeptide":ti,ab,kw 422

#19 (("glucose-dependent insulinotropic polypeptide" near/5 "receptor inhibitors*") or ("glucose-dependent insulinotropic polypeptide" near/5 "receptor agonist*") or ("glucose-dependent insulinotropic polypeptide" near/5 inhibitor*) or ("glucose-dependent insulinotropic polypeptide" near/5 agonist*) or (GIP near/5 "receptor inhibitors*") or (GIP near/5 "receptor agonist*") or (GIP near/5 inhibitors*) or (GIP near/5 agonist*)):ti,ab,kw 62

#20 tirzepatide:ti,ab,kw 92

#21 (LY3298176 or LY-3298176 or "LY 3298176"):ti,ab,kw 38

#22 {or #8-#21} 692

#23 {and #7, #22} 369

Search strategies for other antihyperglycemic drugs from January 2020 to October 2022

Embase (via OVID)

1 diabetes mellitus/ 646929

2 non insulin dependent diabetes mellitus/ 309094

3 ((diabetes or diabetes mellitus or diabetic*) adj1 (type 2 or type II or type ii or non-insulin dependent or noninsulin dependent or adult onset or mature onset or late onset)).tw. 275418

4 NIDDM.tw. 8072

5 Diabetic Nephropathy/ 49734

6 (diabetic nephropath* or diabetic kidney disease).tw. 34660

7 or/1-6 969154

8 long acting insulin/ 2222

9 ((long acting or longer acting or intermediate acting) adj insulin*).tw. 2366

10 insulin degludec/ 2007

11 (insulin adj1 degludec).tw. 1484

12 insulin detemir/ 4149

13 (insulin adj1 detemir).tw. 1300

14 insulin glargine/ 11869

15 (insulin adj1 glargine).tw. 5042

16 insulin zinc suspension/ 2852

17 (insulin adj1 zinc).tw. 392

18 insulin aspart/ 5869

19 (insulin adj1 aspart).tw. 1942

20 insulin lispro/ 6266

21 (insulin adj1 lispro).tw. 1578

22 isophane insulin/ 7963

23 (insulin adj1 isophane).tw. 190

24 (insulin adj1 lente).tw. 388

25 (insulin adj1 ultralente).tw. 347

26 meglitinide/ 2173

27 meglitinide*.tw. 524

28 mitiglinide/ 439

29 mitiglinide.tw. 181

30 nateglinide/ 2759

31 nateglinide.tw. 741

32 repaglinide/ 4187

33 repaglinide.tw. 1290

34 amylin derivative/ 332

35 (amylin analogue* or amylin derivative*).tw. 138

36 pramlintide/ 1555

37 pramlintide.tw. 540

38 biguanide derivative/ 4010

39 biguanide*.tw. 4212

40 metformin.tw,sh. 81688

41 sulphonylurea derivative/ 10091

42 sulphonylurea*.tw. 3839

43 acetohexamide/ 866

44 carbutamide/634

45 chlorpropamide/ 5000

46 glibenclamide/ 26743

47 glibornuride/474

48 gliclazide/ 6846

49 glimepiride.sh,tw. 8138

50 glipizide/ 6285

51 gliquidone/ 740

52 tolazamide/ 1031

53 acetohexamide.tw. 140

54 carbutamide.tw. 60

55 chlorpropamide.tw. 1044

56 glibenclamide.tw. 11815

57 gliclazide.tw. 2273

58 glimepiride.tw. 2749

59 glibornuride.tw. 87

60 glipizide.tw. 1846

61 gliquidone.tw. 229

62 glyburide.tw. 2436

63 glycopyramide.tw. 1

64 tolazamide.tw. 135

65 alpha glucosidase inhibitor/ 6455

66 alpha glucosidase inhibitor*.tw. 4523

67 acarbose/ 9749

68 miglitol/1653

69 voglibose/ 1335

70 acarbose.tw. 4627

71 miglitol.tw. 489

72 voglibose.tw. 545

73 glitazone derivative/ 4814

74 (glitazone adj1 (derivative* or analogue*)).tw. 2

75 thiazolidinedione*.tw. 8242

76 pioglitazone/20817

77 rivoglitazone/ 74

78 rosiglitazone/ 19444

79 pioglitazone.tw. 9394

80 rivoglitazone.tw. 22

81 rosiglitazone.tw. 8391

82 glucagon like peptide 1 receptor agonist/ 8094

83 (glucagon-like peptide 1 receptor inhibitor* or glucagon-like peptide 1 receptor agonist* or glucagon-like peptide 1 inhibitor* or glucagon-like peptide 1 agonist* or GLP-1 receptor inhibitor* or GLP-1 receptor agonist* or GLP-1 inhibitor* or GLP-1 agonist*).tw. 8836

84 albiglutide/ 1242

85 dulaglutide/ 2253

86 exendin 4/ 11532

87 liraglutide/ 11091

88 lixisenatide/ 1863

89 semaglutide/ 2647

90 taspoglutide/ 263

91 albiglutide.tw. 391

92 dulaglutide.tw. 1155

93 (exenatide or exendin 4).tw. 6730

94 liraglutide.tw. 6436

95 lixisenatide.tw. 850

96 semaglutide.tw. 1519

97 taspoglutide.tw. 103

98 dipeptidyl peptidase IV inhibitor/ 12494

99 (dipeptidyl-peptidase IV Inhibitor* or dipeptidyl-peptidase 4 Inhibitor* or ((DPP4 or DPP 4 or DPP IV) adj inhibitor*)).tw. 10338

100 alogliptin/ 2206

101 anagliptin/ 297

102 gemigliptin/ 241

103 linagliptin/ 3363

104 omarigliptin/ 184

105 saxagliptin/ 3646

106 sitagliptin/ 9870

107 teneligliptin/ 583

108 vildagliptin/ 4434

109 alogliptin.tw. 860

110 anagliptin.tw. 164

111 gemigliptin.tw. 138

112 linagliptin.tw. 1705

113 omarigliptin.tw. 92

114 saxagliptin.tw. 1414

115 sitagliptin.tw. 4951

116 teneligliptin.tw. 350

117 vildagliptin.tw. 2033

118 evogliptin.tw. 73

119 dutogliptin.tw. 31

120 retagliptin.tw. 9

121 sodium glucose cotransporter 2 inhibitor/ 9411

122 (sodium glucose transporter 2 inhibitor* or sodium glucose transporter ii inhibitor* or SGLT 2 inhibitor*).tw. 1997

123 (sodium glucose cotransporter adj3 inhibitor*).tw. 4600

124 (sodium glucose co transporter adj3 inhibitor*).tw. 2528

125 canagliflozin/ 4692

126 dapagliflozin.tw. 3769

127 empagliflozin/ 6478

128 ertugliflozin/892

129 tofogliflozin/ 489

130 canagliflozin.tw. 2424

131 dapagliflozin.tw. 3769

132 empagliflozin.tw. 3739

133 ertugliflozin.tw. 344

134 tofogliflozin.tw. 233

135 bexagliflozin.tw. 14

136 henagliflozin.tw. 10

137 ipragliflozin.tw. 415

138 licogliflozin.tw. 16

139 luseogliflozin.tw.238

140 remogliflozin.tw.91

141 sergliflozin.tw. 17

142 sotagliflozin.tw. 214

143 antidiabetic agent/ 58349

144 oral antidiabetic agent/ 21090

145 or/8-144258776

146 and/7,145 145592

147 randomized controlled trial/ 732195

148 double-blind procedure/ 199691

149 single-blind procedure/ 47925

150 random$.tw. 1845386

151 factorial$.tw. 44928

152 placebo$.tw. 350022

153 (double$ adj blind$).tw. 234635

154 (singl$ adj blind$).tw. 29692

155 assign$.tw. 462905

156 allocat$.tw. 188260

157 or/147-156 2479317

158 and/146,157 27312

159 MEDLINE.cr. 8979033

160 158 not 159 25988

161 (mouse or mice or murine or rat or rats or dog or dogs or animal*).ti. 1886819

162 160 not 161 24823

163 limit 162 to yr="2020 -Current" 4881

MEDLINE (via OVID)

1 exp Diabetes Mellitus Type 2/ 162040

2 Diabetes Mellitus/ 133185

3 ((diabetes or diabetes mellitus or diabetic*) adj1 (type 2 or type II or type ii or non-insulin dependent or noninsulin dependent or adult onset or mature onset or late onset)).tw. 156382

4 NIDDM.tw. 6775

5 Diabetic Nephropathies/ 28587

6 (diabetic nephropath* or diabetic kidney disease).tw. 21055

7 or/1-6 345031

8 exp Insulin, Long Acting/ 4471

9 ((long acting or longer acting or intermediate acting) adj insulin*).tw. 1225

10 (insulin adj1 degludec).tw. 457

11 (insulin adj1 glargine).tw. 2069

12 (insulin adj1 detemir).tw. 560

13 (insulin adj1 zinc).tw. 439

14 (insulin adj1 isophane).tw. 109

15 (insulin adj1 aspart).tw. 888

16 (insulin adj1 lispro).tw. 822

17 (insulin adj1 lente).tw. 149

18 (insulin adj1 ultralente).tw. 115

19 Sulfonylurea Compounds/ 6575

20 Acetohexamide/ 240

21 Carbutamide/ 533

22 Chlorpropamide/ 1825

23 Gliclazide/ 960

24 Glipizide/ 755

25 Glyburide/ 6401

26 Tolazamide/ 168

27 (sulphonylurea or sulphonylureas).tw. 2407

28 acetohexamide.tw. 186

29 carbutamide.tw. 247

30 chlorpropamide.tw. 1317

31 glibenclamide.tw. 8100

32 gliclazide.tw. 1193

33 glimepiride.tw. 1235

34 glibornuride.tw. 83

35 glipizide.tw. 1022

36 gliquidone.tw. 145

37 glyburide.tw. 1553

38 glycopyramide.tw. 1

39 tolazamide.tw. 149

40 Biguanides/ 3395

41 biguanides.tw. 1159

42 Metformin/ 16892

43 metformin.tw. 21032

44 alpha-Glucosidases/ai [Antagonists & Inhibitors] 0

45 alpha glucosidase inhibitor*.tw. 2890

46 Acarbose/ 1491

47 acarbose.tw. 2741

48 miglitol.tw. 273

49 voglibose.tw. 290

50 Thiazolidinediones/ 11549

51 thiazolidinedione*.tw. 5508

52 pioglitazone.tw. 5181

53 rivoglitazone.tw. 13

54 rosiglitazone.tw. 5464

55 meglitinide*.tw. 288

56 repaglinide.tw. 737

57 nateglinide.tw. 446

58 mitiglinide.tw. 104

59 Receptors, Glucagon/ag 770

60 Glucagon-like Peptide 1/ag 542

61 (glucagon-like peptide 1 receptor inhibitor* or glucagon-like peptide 1 receptor agonist* or glucagon-like peptide 1 inhibitor* or glucagon-like peptide 1 agonist* or GLP-1 receptor inhibitor* or GLP-1 receptor agonist* or GLP-1 inhibitor* or GLP-1 agonist*).tw. 4374

62 dulaglutide.tw. 388

63 (exenatide or exendin 4).tw. 3352

64 liraglutide.tw. 2741

65 semaglutide.tw. 633

66 taspoglutide.tw. 51

67 lixisenatide.tw. 368

68 albiglutide.tw. 167

69 Dipeptidyl-peptidase IV Inhibitors/ 4993

70 (dipeptidyl-peptidase IV Inhibitor* or dipeptidyl-peptidase 4 Inhibitor* or ((DPP4 or DPP 4 or DPP IV) adj inhibitor*)).tw. 5240

71 omarigliptin.tw. 59

72 vildagliptin.tw. 931

73 sitagliptin.tw. 2105

74 saxagliptin.tw. 628

75 linagliptin.tw. 729

76 alogliptin.tw. 436

77 septagliptin.tw. 0

78 gemigliptin.tw. 66

79 anagliptin.tw. 76

80 teneligliptin.tw. 141

81 evogliptin.tw. 44

82 dutogliptin.tw. 17

83 retagliptin.tw. 3

84 Sodium-Glucose Transporter 2/ai 0

85 (sodium glucose transporter 2 inhibitor* or sodium glucose transporter ii inhibitor* or SGLT 2 inhibitor*).tw. 848

86 (sodium glucose cotransporter adj3 inhibitor*).tw. 2598

87 (sodium glucose co transporter adj3 inhibitor*).tw. 1319

88 canagliflozin.tw. 1042

89 dapagliflozin.tw. 1534

90 empagliflozin.tw. 1579

91 ertugliflozin.tw. 145

92 tofogliflozin.tw. 113

93 bexagliflozin.tw. 10

94 henagliflozin.tw. 9

95 ipragliflozin.tw. 201

96 licogliflozin.tw. 12

97 luseogliflozin.tw.110

98 remogliflozin.tw.33

99 sergliflozin.tw. 14

100 sotagliflozin.tw. 110

101 (amylin adj1 (analogue* or derivative*)).tw. 93

102 pramlintide.tw. 341

103 Insulins/1260

104 Hypoglycemic Agents/ 75089

105 or/8-104122620

106 and/7,105 56777

107 randomized controlled trial.pt.577473

108 controlled clinical trial.pt.95029

109 pragmatic clinical trial.pt.2145

110 randomized.ab. 507551

111 randomised.ab. 103652

112 placebo.ab. 212881

113 clinical trials as topic/ 200361

114 randomly.ab. 334494

115 trial.ti. 238934

116 or/107-115 1373150

117 animals/ not (humans/ and animals/) 5017107

118 116 not 117 1253710

119 and/106,118 12485

120 limit 119 to yr="2020 -Current" 2218

Cochrane library

#1 MeSH descriptor: [Diabetes Mellitus] this term only 10807

#2 ((diabetes or diabetic* or "diabetes mellitus") near/1 ("type 2" or "type ii" or "non-insulin dependent" or "non insulin dependent" or "noninsulin dependent" or "adult onset" or "mature onset" or "late onset")):ti,ab,kw 48592

#3 ("diabetes mellitus" not "insulin dependent diabetes mellitus"):kw 39345

#4 #3 and EMBASE 15466

#5 NIDDM:ti,ab,kw 1117

#6 ((diabetic next nephropath*) or "diabetic kidney disease"):ti,ab,kw 4687

#7 {or #1-#2, #4-#6} 69425

#8 ((long acting or longer acting or intermediate acting) near/1 insulin*):ti,ab,kw 2202

#9 insulin near/1 degludec:ti,ab,kw 1041

#10 insulin near/1 detemir:ti,ab,kw 668

#11 insulin near/1 glargine:ti,ab,kw 2813

#12 insulin near/1 zinc 561

#13 insulin near/1 aspart:ti,ab,kw 1508

#14 insulin near/1 lispro:ti,ab,kw 1099

#15 insulin near/1 isophane:ti,ab,kw 761

#16 insulin near/1 ultralente:ti,ab,kw 46

#17 insulin near/1 lente 26

#18 meglitinide*:ti,ab,kw 89

#19 mitiglinide:ti,ab,kw 87

#20 nateglinide:ti,ab,kw 215

#21 repaglinide:ti,ab,kw 344

#22 (amylin next analogue* or amylin next derivative*):ti,ab,kw 29

#23 pramlintide:ti,ab,kw 161

#24 biguanide*:ti,ab,kw 621

#25 metformin:ti,ab,kw 12149

#26 (sulfonylurea* or sulphonylurea*):ti,ab,kw 3159

#27 acetohexamide:ti,ab,kw 6

#28 carbutamide:ti,ab,kw 5

#29 chlopropramide:ti,ab,kw 0

#30 glibenclamide:ti,ab,kw 986

#31 gliclazide:ti,ab,kw 565

#32 glimepiride:ti,ab,kw 1280

#33 glibornuride:ti,ab,kw 11

#34 glipizide:ti,ab,kw397

#35 gliquidone:ti,ab,kw 23

#36 glyburide:ti,ab,kw 804

#37 glycopyramide:ti,ab,kw 0

#38 tolazimide:ti,ab,kw 0

#39 MeSH descriptor: [alpha-Glucosidases] this term only 32

#40 alpha next glucosidase next inhibitor*:ti,ab,kw 424

#41 acarbose:ti,ab,kw845

#42 miglitol:ti,ab,kw 167

#43 voglibose:ti,ab,kw 244

#44 (glitazone near/1 (derivative* or analogue*)):ti,ab,kw 70

#45 thiazolidinedione:ti,ab,kw630

#46 pioglitazone:ti,ab,kw 2537

#47 rivoglitazone:ti,ab,kw 13

#48 rosiglitazone:ti,ab,kw1181

#49 MeSH descriptor: [Receptors, Glucagon] explode all trees and with qualifier(s): [agonists - AG]38

#50 MeSH descriptor: [Glucagon-Like Peptide 1] explode all trees and with qualifier(s): [antagonists & inhibitors - AI] 7

#51 ("glucagon-like peptide 1 receptor inhibitor" or "glucagon-like peptide 1 receptor agonist" or "glucagon-like peptide 1 inhibitor" or "glucagon-like
peptide 1 agonist" or "GLP-1 receptor inhibitor" or "GLP-1 receptor agonist" or "GLP-1 inhibitor" or "GLP-1 agonist"):ti,ab,kw 1144

#52 ("glucagon-like peptide 1 receptor inhibitors" or "glucagon-like peptide 1 receptor agonists" or "glucagon-like peptide 1 inhibitors" or "glucagon-like
peptide 1 agonists" or "GLP-1 receptor inhibitors" or "GLP-1 receptor agonists" or "GLP-1 inhibitors" or "GLP-1 agonists"):ti,ab,kw 744

#53 albiglutide:ti,ab,kw 136

#54 dulaglutide:ti,ab,kw 463

#55 (exenatide or "exendin 4"):ti,ab,kw 1336

#56 liraglutide:ti,ab,kw 2152

#57 lixisenatide:ti,ab,kw 336

#58 semaglutide:ti,ab,kw 759

#59 taspoglutide:ti,ab,kw 42

#60 ("dipeptidyl-peptidase IV Inhibitor" or "dipeptidylpeptidase 4 Inhibitor" or "dipeptidyl-peptidase IV Inhibitors" or "dipeptidyl-peptidase 4 Inhibitors"):ti,ab,kw 1068

#61 ((DPP4 or DPP 4 or DPP IV) next inhibitor*):ti,ab,kw 2910

#62 alogliptin:ti,ab,kw 295

#63 anagliptin:ti,ab,kw 61

#64 gemigliptin:ti,ab,kw 91

#65 linagliptin:ti,ab,kw 683

#66 omarigliptin:ti,ab,kw 75

#67 saxagliptin:ti,ab,kw 495

#68 sitagliptin:ti,ab,kw 2105

#69 teneligliptin:ti,ab,kw 149

#70 vildagliptin:ti,ab,kw 768

#71 evogliptin:ti,ab,kw 46

#72 dutogliptin:ti,ab,kw 24

#73 retagliptin:ti,ab,kw 7

#74 MeSH descriptor: [Sodium-Glucose Transporter 2] explode all trees 115

#75 ("sodium glucose transporter 2 inhibitor" or "sodium glucose transporter ii inhibitor" or "SGLT 2 inhibitor" or "sodium glucose transporter 2 inhibitors"
or "sodium glucose transporter ii inhibitors" or "SGLT 2 inhibitors"):ti,ab,kw 823

#76 ("sodium glucose cotransporter" near/3 inhibitor*):ti,ab,kw 830

#77 ("sodium glucose co-transporter" near/3 inhibitor*):ti,ab,kw 538

#78 canagliflozin:ti,ab,kw677

#79 dapagliflozin:ti,ab,kw 1565

#80 empagliflozin:ti,ab,kw 1444

#81 ertugliflozin:ti,ab,kw 167

#82 tofogliflozin:ti,ab,kw 89

#83 bexagliflozin:ti,ab,kw 22

#84 henagliflozin:ti,ab,kw 6

#85 ipragliflozin:ti,ab,kw 158

#86 licogliflozin:ti,ab,kw 14

#87 luseogliflozin:ti,ab,kw 96

#88 remogliflozin:ti,ab,kw 30

#89 sergliflozin:ti,ab,kw 2

#90 sotagliflozin:ti,ab,kw 110

#91 MeSH descriptor: [Insulins] this term only 34

#92 MeSH descriptor: [Hypoglycemic Agents] this term only 8550

#93 (antidiabetic next agent*):kw 1344

#94 (oral next antidiabetic next agent*):kw 514

#95 {or #8-#94} 33183

#96 {and #7, #95} 21533

#97 Limits year first published = 2020-2022 3240

1.2 Definition of outcomes

**Outcome**	**Definition**	**Effect measure**
All-cause death	Number of patients who died due to any reason within follow-ups.	Odds ratio
Cardiovascular death	Number of patients who died due to acute myocardial infarction, sudden cardiac death, heart failure, stroke, cardiovascular procedures, and any other determined cardiovascular cause within follow-ups.	Odds ratio
Non-fatal myocardial infarction	Number of patients who suffered an acute myocardial infarction but not died within follow-ups.	Odds ratio
Non-fatal stroke	Number of patients who suffered a stroke but not died within follow-ups.	Odds ratio
Hospitalization for heart failure	Number of patients who are admitted to the hospital because of the worsening heart failure, with documented new or worsening symptoms of heart failure on presentation, physical examination and laboratory tests supporting the worsening of heart failure, and they stay in hospital for at least 12 hours (or a change in calendar date if the hospital admission and discharge times are unavailable) to receive initiation or intensification of treatment specifically for heart failure.	Odds ratio
End-stage kidney disease	Number of patients who were with a long-term dialysis, kidney transplantation, a sustained eGFR <15 ml per minute per 1.73 m2, a sustained percent decline in eGFR of at least 40% or a doubling of serum creatinine, or kidney-related death.	Odds ratio
Health-related quality of life	Change of health-related quality of life score from baseline to follow-up end measured by several questionaries, including Diabetes Treatment Satisfaction Questionnaire, Diabetes Therapy-Related Quality of Life, Psychological General Well-Being index, Medical Outcomes Study 36-Item Short-Form Health Survey, Diabetes Medication Satisfaction Tool, Impact of Weight on Quality of Life-Lite, Short Form 12-Item survey, Five-Dimension European Quality of Life Questionnaire, Diabetes Treatment Satisfaction Questionnaire, Diabetes Care Profile, TRIM-Diabetes Questionnaire Total Score, Kansas City Cardiomyopathy Questionnaire Total Symptom Score, Modified Diabetes Quality Of Life Clinical Trial Questionnaire.	Standardised mean difference
Severe hypoglycaemia	Number of patients who suffered at least one hypoglycaemia event which led to a medical assistance. The study prioritises the definition above but adopted any other study-reported definition if unavailable.	Odds ratio
Severe gastrointestinal events	Number of patients who suffered at least one severe gastrointestinal event or gastrointestinal event leading to discontinuation. The study prioritises the definition above but adopted any other study-reported definition if unavailable.	Odds ratio
Genital infection	Number of patients who suffered at least one genital infection event.	Odds ratio
Amputation	Number of patients who suffered at least one amputation event.	Odds ratio

Ketoacidosis due to diabetes	Number of patients who suffered at least one ketoacidosis event due to diabetes.	Odds ratio
Hyperkalaemia leading to hospitalization	Number of patients who suffered at least one hyperkalaemia leading to hospitalization.	Odds ratio
Body weigh change	Change of absolute bodyweight (kg) from baseline to follow-up end.	Mean difference

1.3 Summary of included interventions

**Drug classes**	**Individual drugs**
Alpha-glucosidases inhibitors	Acarbose, Miglitol, Voglibose
Basal insulin	Insulin detemir, Insulin degludec, Insulin glargine, NPH insulin, Isophane insulin
Basal-Bolus insulin	Insulin aspart 70/30, Premixed lispro/lispro protamine, Humalog Mix25, Intermittent intensive insulin therapy (basal insulin glargine at bedtime and premeal lispro)
Bolus insulin	Insulin glulisine, Insulin lispro, Insulin aspart
DPP-4 inhibitors	Alogliptin, Sitagliptin, Vildagliptin, Linagliptin, Gemigliptin, Tenegliptin, Omargliptin, Saxagliptin, Evogliptin, Trelagliptin, Prusogliptin
Dual GIP/GLP-1 receptor agonists	Tirzepatide (LY3298176)
Meglitinides	Repaglinide, Nateglinide
GLP-1 receptor agonists	Semaglutide (Subcutaneous), Semaglutide (Oral), Efpeglenatide, Liraglutide, Exenatide (immediate-release), Exenatide (extended-release), Dulaglutide, Albiglutide, Lixisenatide, Loxenatide
Metformin	Metformin
Non-steroidal MRAs	Finerenone, Esaxerenone
SGLT2 inhibitors	Dapagliflozin, Bexagliflozin, Canagliflozin, Empagliflozin, Sotagliflozin, Ipragliflozin, Luseogliflozin, Henagliflozin, Tofogliflozin, Ertugliflozin
Sulfonylureas	Glyburide, Glibenclamide, Glipizide, Glimepiride, Gliclazide
Thiazolidinediones	Pioglitazone, Rosiglitazone, Rivoglitazone, Lobeglitazone
Standard treatments	Standard treatments include standard care (i.e., lifestyle modification) and standard drug treatments (e.g., metformin and/or sulfonylureas) other than the drug of interest in the randomised trial.

1.4 Details of GRADE approaches

The study followed the GRADE guideline to assess the certainty of evidence for network meta-analysis. Evidence from direct comparisons started as high certainty evidence and was rated down for severe risk of bias (judged by the proportion of high risk of bias across all domains and studies), heterogeneity, indirectness, and local publication bias (i.e., pairwise publication bias) [Balshem et al, 2011]. Evidence from indirect comparisons was further rated down for intransitivity. A contribution matrix quantified the proportional contribution of each direct comparison to each indirect and network comparison using the random-walk approach [Davies et al, 2022] which determines the start of the certainty for indirect and network comparisons. The final certainty for network evidence was rated down if there were incoherence or imprecision [Puhan et al, 2014].

We rated down for heterogeneity based on similarity in point estimates and overlap in confidence intervals (CIs), which were estimated using frequentist random-effect pairwise meta-analysis with an inverse-variance estimator for pooling and a restricted maximum likelihood estimator for heterogeneity. For comparisons with five to nine trials, we tested local publication bias if indicated by Harbord's score test [Harbord et al, 2006] for binary outcomes or Egger's test [Egger et al, 1997] for continuous outcomes with the significant level being 0.05. For comparisons involving 10 or more trials, we assessed the publication bias using Harbord's tests for binary outcomes and Egger's tests for continuous outcomes. For comparisons indicated significant publication bias by the above statistical analyses, we rated down the certainty of evidence if the trim and fill analysis suggested the publication bias changed the direction of the results [Duval and Tweedie, 2000].

We rated imprecision by utilising a GRADE approach with a null effect decision threshold [Zeng et al, 2021]. When point estimates proved less than specified minimal important differences (MIDs) established by the previous guideline panel [Li et al, 2021], we rated certainty in little or no effect. Absolute effects were calculated by applying relative effects to baseline risks from the previous guideline panel (for cardiovascular and kidney outcomes and adopting baseline risks in high-risk group) [Li et al, 2021] or pooled single-rate random-effect meta-analysis of control arms (i.e., standard treatments) with at least one year follow up (for other outcomes). We rated down for imprecision by two levels when 95%CI crossed more than one threshold of importance [Zeng et al, 2022].

The study adopted the MIDs for binary outcomes from previous guideline [Li et al, 2021], and set the MID for body weight change as change in 5 kg as well as the MID for quality-of-life score as 10% of total score. We transformed the standardised mean difference back to mean difference following the guide [Murad et al,

2019] by multiplying the standardised standard deviation (i.e., 10) for a specific questionary, i.e., 36-Item Short Form Survey [SF-36]. Hence, the MID for the quality-of-life score is same as one standard deviation.

References

Balshem H, Helfand M, Schünemann HJ, et al. GRADE guidelines: 3. Rating the quality of evidence. J Clin Epidemiol 2011;64:401–6. doi:10.1016/j.jclinepi.2010.07.015

Davies AL, Papakonstantinou T, Nikolakopoulou A, et al. Network meta-analysis and random walks. Stat Med 2022;41:2091–114. doi:10.1002/sim.9346

Puhan MA, Schünemann HJ, Murad MH, et al. A GRADE Working Group approach for rating the quality of treatment effect estimates from network meta-analysis. BMJ 2014;349:g5630. doi:10.1136/bmj.g5630

Harbord RM, Egger M, Sterne JAC. A modified test for small-study effects in meta-analyses of controlled trials with binary endpoints. Statistics in Medicine 2006;25:3443–57. doi:10.1002/sim.2380

Egger M, Davey Smith G, Schneider M, et al. Bias in meta-analysis detected by a simple, graphical test. BMJ 1997;315:629–34. doi:10.1136/bmj.315.7109.629

Duval S, Tweedie R. Trim and fill: A simple funnel-plot-based method of testing and adjusting for publication bias in meta-analysis. Biometrics 2000;56:455–63. doi:10.1111/j.0006-341x.2000.00455.x

Zeng L, Brignardello-Petersen R, Hultcrantz M, et al. GRADE guidelines 32: GRADE offers guidance on choosing targets of GRADE certainty of evidence ratings. J Clin Epidemiol 2021;137:163–75. doi:10.1016/j.jclinepi.2021.03.026

Li S, Vandvik PO, Lytvyn L, et al. SGLT-2 inhibitors or GLP-1 receptor agonists for adults with type 2 diabetes: a clinical practice guideline. BMJ 2021;373:n1091. doi:10.1136/bmj.n1091

Zeng L, Brignardello-Petersen R, Hultcrantz M, et al. GRADE Guidance 34: update on rating imprecision using a minimally contextualized approach. J Clin Epidemiol 2022;:S0895-4356(22)00187-1. doi:10.1016/j.jclinepi.2022.07.014

Murad MH, Wang Z, Chu H, Lin L. When continuous outcomes are measured using different scales: guide for meta-analysis and interpretation. BMJ. 2019 Jan 22;364:k4817. doi: 10.1136/bmj.k4817.

Appendix 2 List of included studies

2.1 Study settings

Study ID	Registration	Year	Country	Intervention	N	Drug	Dose	Cointervention	Follow -up*	Settings	Fundings
**1860-LIRA-DPP-4**	NCT00700817	2010	Multinational	DPP-4 inhibitors	219	Sitagliptin	100 mg daily	Metformin	6		Novo Nordisk
**1860-LIRA-DPP-4**	NCT00700817	2010	Multinational	GLP-1 receptor agonists	221	Liraglutide	1.8 mg daily	Metformin	6		Novo Nordisk
**1860-LIRA-DPP-4**	NCT00700817	2010	Multinational	GLP-1 receptor agonists	225	Liraglutide	1.2 mg daily	Metformin	6		Novo Nordisk
**1860-LIRA-DPP-4**	NCT00700817	2010	Multinational	GLP-1 receptor agonists	446	Liraglutide	1.2-1.8 mg daily	Metformin	6		Novo Nordisk
**3D**	UMIN000003784	2015	Japan	Alpha-glucosidase inhibitors	40	Voglibose	0.6 mg daily	Metformin, sulfonylurea, or pioglitazone	5.5		Osaka Foundation for the Prevention of Cancer and Cardiovascular Diseases.
**3D**	UMIN000003784	2015	Japan	DPP-4 inhibitors	40	Sitagliptin	50 mg daily	Metformin, sulfonylurea, or pioglitazone	5.5	Heart failure	Osaka Foundation for the Prevention of Cancer and Cardiovascular Diseases.

Study ID	Registration	Year	Country	Intervention	N	Drug	Dose	Cointervention	Follow -up*	Settings	Fundings
**4B**	NCT00960661	2014	Multinational	Bolus insulin	312	Insulin lispro	Titrated	Metformin + insulin glargine	7.5		Eli Lilly; Amylin Pharmaceuticals Alliance; Bristol-Myers Squibb; AstraZeneca Alliance
**4B**	NCT00960661	2014	Multinational	GLP-1 receptor agonists	315	Exenatide	10-20 mcg daily	Metformin + insulin glargine	7.5		Eli Lilly; Amylin Pharmaceuticals Alliance; Bristol-Myers Squibb; AstraZeneca Alliance
**4T**	ISCRTN51125379	2007	Multinational	Basal insulin	234	Insulin detemir	Titrated	Metformin + sulfonylurea	12		Novo Nordisk
**4T**	ISCRTN51125379	2007	Multinational	Bolus insulin	239	Insulin aspart	Titrated	Metformin + sulfonylurea	12		Novo Nordisk
**Abdullah-2021**	Not provied	2021	Pakistan	DPP-4 inhibitors	110	Sitagliptin	100mg q.d	metformin	5.5		No provided
**Abdullah-2021**	Not provied	2021	Pakistan	SGLT2 inhibitors	110	Dapagliflozin	10mg qd	metformin	5.5		No provided
**Abe 2008**	Not provided	2008	Japan	Standard treatments	32	Control		Alpha glucosidase inhibitor meglitinide	22		Not reported
**Abe 2008**	Not provided	2008	Japan	Thiazolidinediones	31	Pioglitazone	15-30 mg daily	Alpha glucosidase inhibitor meglitinide	22	Haemodialysis	Not reported

Study ID	Registration	Year	Country	Intervention	N	Drug	Dose	Cointervention	Follow-up*	Settings	Fundings
**Abe 2016**	UMIN000018445	2016	Japan	DPP-4 inhibitors	41	Saxagliptin	2.5 mg daily	Oral drugs and/or insulin	5.5	Haemodialysis	Not reported
**Abe 2016**	UMIN000018445	2016	Japan	Standard treatments	41	Control		Oral drugs and/or insulin	5.5		Not reported
**ACTION-J**	UMIN 000002333	2011	Japan	Standard treatments	28	Control		Insulin	11		Not reported
**ACTION-J**	UMIN 000002333	2011	Japan	Thiazolidinediones	26	Pioglitazone	15-30 mg daily	Insulin	11		Not reported
**Adel-2022**	Not provided	2022	Iran	SGLT2 inhibitors	52	Empagliflozin	10 mg daily	Insulin	6	Acute coronary syndrome	Vice Chancellor for Research of Ahvaz Jundishapur University, Iran
**Adel-2022**	Not provided	2022	Iran	Standard treatments	54	Placebo		Insulin	6		Vice Chancellor for Research of Ahvaz Jundishapur University, Iran
**ADOPT**	NCT00279045	2006	Multinational	Metformin	1455	Metformin	500-2000 mg daily	None	48		GlaxoSmithKline
**ADOPT**	NCT00279045	2006	Multinational	Sulfonylureas	1447	Glyburide	2.5-15 mg daily	None	48		GlaxoSmithKline
**ADOPT**	NCT00279045	2006	Multinational	Thiazolidinediones	1458	Rosiglitazone	4-8 mg daily	None	48		GlaxoSmithKline
**Ahmann 2015**	NCT01617434	2015	Multinational	GLP-1 receptor agonists	225	Liraglutide	1.8 mg daily	Metformin + basal insulin	5.5		Novo Nordisk

**Study ID**	**Registration**	**Year**	**Country**	**Intervention**	**N**	**Drug**	**Dose**	**Cointervention**	**Follow -up***	**Settings**	**Fundings**
**Ahmann 2015**	NCT01617434	2015	Multinational	Standard treatments	225	Placebo		Metformin + basal insulin	5.5		Novo Nordisk
**Ahrén 2004**	Not applicable	2004	Sweden	DPP-4 inhibitors	42	Vildagliptin	50 mg daily	Metformin	12		Novartis
**Ahrén 2004**	Not applicable	2004	Sweden	Standard treatments	29	Placebo		Metformin	12		Novartis
**Allegretti 2019**	NCT02836873	2019	Multinational	SGLT2 inhibitors	157	Bexagliflozin	20 mg daily	Oral hypoglycaemic agents	6	Chronic kidney disease	Theracos
**Allegretti 2019**	NCT02836873	2019	Multinational	Standard treatments	155	Placebo		Oral hypoglycaemic agents	6		Theracos
**Alogliptin Study 007**	NCT00286468	2009	Multinational	DPP-4 inhibitors	198	Alogliptin	25 mg daily	Sulfonylurea	6		Takeda
**Alogliptin Study 007**	NCT00286468	2009	Multinational	DPP-4 inhibitors	203	Alogliptin	12.5 mg daily	Sulfonylurea	6		Takeda
**Alogliptin Study 007**	NCT00286468	2009	Multinational	DPP-4 inhibitors	401	Alogliptin	12.5-25 mg daily	Sulfonylurea	6		Takeda
**Alogliptin Study 007**	NCT00286468	2009	Multinational	Standard treatments	99	Placebo		Sulfonylurea	6		Takeda
**Alogliptin Study 009**	NCT00286494	2009	Multinational	DPP-4 inhibitors	199	Alogliptin	25 mg daily	Thiazolidinedione metformin or sulfonylurea	6		Takeda
**Alogliptin Study 009**	NCT00286494	2009	Multinational	DPP-4 inhibitors	197	Alogliptin	12.5 mg daily	Thiazolidinedione metformin or sulfonylurea	6		Takeda

Study ID	Registration	Year	Country	Intervention	N	Drug	Dose	Cointervention	Follow -up*	Settings	Fundings
**Alogliptin Study 009**	NCT00286494	2009	Multinational	DPP-4 inhibitors	396	Alogliptin	12.5-25 mg daily	Thiazolidinedione metformin or sulfonylurea	6		Takeda
**Alogliptin Study 009**	NCT00286494	2009	Multinational	Standard treatments	97	Placebo		Thiazolidinedione metformin or sulfonylurea	6		Takeda
**Alogliptin Study 010**	NCT00286455	2008	Multinational	DPP-4 inhibitors	131	Alogliptin	25 mg daily	None	6		Takeda
**Alogliptin Study 010**	NCT00286455	2008	Multinational	DPP-4 inhibitors	133	Alogliptin	12.5 mg daily	None	6		Takeda
**Alogliptin Study 010**	NCT00286455	2008	Multinational	DPP-4 inhibitors	263	Alogliptin	12.5-25 mg daily	None	6		Takeda
**Alogliptin Study 010**	NCT00286455	2008	Multinational	Standard treatments	64	Placebo		None	6		Takeda
**Alvarsson 2003**	Not applicable	2003	Sweden	Basal-Bolus insulin	23	Mixtard 30/70 insulin	Titrated	None	12		Novo Nordisk
**Alvarsson 2003**	Not applicable	2003	Sweden	Sulfonylureas	28	Glibenclamide	1.75-3.5 mg daily	None	12		Novo Nordisk
**AMPLITUDE-M**	NCT03353350	2022	Multinational	GLP-1 receptor agonists	100	Efpeglenatide	2 mg	diet and exercise	12.9		Sanofi and Hanmi Pharmaceutical Co., Ltd.
**AMPLITUDE-M**	NCT03353350	2022	Multinational	GLP-1 receptor agonists	101	Efpeglenatide	4 mg	diet and exercise	12.9		Sanofi and Hanmi Pharmaceutical Co., Ltd.

Study ID	Registration	Year	Country	Intervention	N	Drug	Dose	Cointervention	Follow-up*	Settings	Fundings
**AMPLITUDE-M**	NCT03353350	2022	Multinational	GLP-1 receptor agonists	103	Efpeglenatide	6 mg	diet and exercise	12.9		Sanofi and Hanmi Pharmaceutical Co., Ltd.
**AMPLITUDE-M**	NCT03353350	2022	Multinational	GLP-1 receptor agonists	304	Efpeglenatide	2-6 mg	diet and exercise	12.9		Sanofi and Hanmi Pharmaceutical Co., Ltd.
**AMPLITUDE-M**	NCT03353350	2022	Multinational	Standard treatments	102	Placebo		diet and exercise	12.9		Sanofi and Hanmi Pharmaceutical Co., Ltd.
**AMPLITUDE-O**	NCT03496298	2021	Multinational	GLP-1 receptor agonists	2717	Efpeglenatide	4 or 6 mg weekly	each participant's current therapy	21.72	cardiovascular disease	Sanofi
**AMPLITUDE-O**	NCT03496298	2021	Multinational	Standard treatments	1359	Placebo		each participant's current therapy	21.72		Sanofi
**Ando-2021**	UMIN000019382	2021	Japan	GLP-1 receptor agonists	20	Liraglutide	0.3 mg/day	basal insulin therapy	5.5		Unclear
**Ando-2021**	UMIN000019382	2021	Japan	SGLT2 inhibitors	20	Canagliflozin	100 mg/day	basal insulin therapy	5.5		Unclear
**APOLLO**	NCT00311818	2008	Multinational	Basal insulin	205	Insulin glargine	Titrated	Oral agents excluding alpha-glucosidase inhibitors	12		Sanofi-Aventis
**APOLLO**	NCT00311818	2008	Multinational	Bolus insulin	210	Insulin lispro	Titrated	Oral agents excluding alpha-	12		Sanofi-Aventis

Study ID	Registration	Year	Country	Intervention	N	Drug	Dose	Cointervention	Follow-up*	Settings	Fundings
								glucosidase inhibitors			
**Apovian 2010**	Not provided	2010	USA	GLP-1 receptor agonists	96	Exenatide	10 mcg daily	Metformin or sulfonylurea	5.5		Eli Lilly
**Apovian 2010**	Not provided	2010	USA	Standard treatments	98	Placebo		Metformin or sulfonylurea	5.5		Eli Lilly
**APPROACH**	NCT00116831	2010	Multinational	Sulfonylureas	339	Glipizide	5-15 mg daily	1-2 oral agents	18	Coronary artery disease	GlaxoSmithKline
**APPROACH**	NCT00116831	2010	Multinational	Thiazolidinediones	333	Rosiglitazone	4-8 mg daily	1-2 oral agents	18		GlaxoSmithKline
**APRIME**	UMIN000004367	2011	Japan	Metformin	31	Metformin	500-750 mg daily	Insulin or oral hypoglycemic agents other than thiazolidinediones/ metformin	12	Chronic kidney disease	Takeda
**APRIME**	UMIN000004367	2011	Japan	Thiazolidinediones	32	Pioglitazone	15 mg daily	Insulin or oral hypoglycemic agents other than thiazolidinediones/ metformin	12		Takeda
**Araki 2015**	NCT01368081	2015	Japan	Metformin	63	Metformin	500-2250 mg daily	Monotherapy with sulfonylurea, biguanide, thiazolidinedione,	12		Boehringer Ingelheim/Eli Lilly

Study ID	Registration	Year	Country	Intervention	N	Drug	Dose	Cointervention	Follow -up*	Settings	Fundings
								alpha glucosidase inhibitor, DPP-4 inhibitor or glinide			
**Araki 2015**	NCT01368081	2015	Japan	SGLT2 inhibitors	137	Empagliflozin	25 mg daily	Monotherapy with sulfonylurea, biguanide, thiazolidinedione, alpha glucosidase inhibitor, DPP-4 inhibitor or glinide	12		Boehringer Ingelheim/Eli Lilly
**Araki 2015**	NCT01368081	2015	Japan	SGLT2 inhibitors	136	Empagliflozin	10 mg daily	Monotherapy with sulfonylurea, biguanide, thiazolidinedione, alpha glucosidase inhibitor, DPP-4 inhibitor or glinide	12		Boehringer Ingelheim/Eli Lilly
**Araki 2015**	NCT01368081	2015	Japan	SGLT2 inhibitors	273	Empagliflozin	10-25 mg daily	Monotherapy with sulfonylurea, biguanide, thiazolidinedione, alpha glucosidase inhibitor, DPP-4 inhibitor or glinide	12		Boehringer Ingelheim/Eli Lilly

Study ID	Registration	Year	Country	Intervention	N	Drug	Dose	Cointervention	Follow -up*	Settings	Fundings
**Araki 2015a**	NCT01584232	2015	Japan	Basal insulin	180	Insulin glargine	Titrated	Biguanide and/or sulphonylurea	5.5		Eli Lilly
**Araki 2015a**	NCT01584232	2015	Japan	GLP-1 receptor agonists	181	Dulaglutide	0.75 mg weekly	Biguanide and/or sulphonylurea	5.5		Eli Lilly
**Arechavaleta 2011**	NCT00701090	2011	Multinational	DPP-4 inhibitors	516	Sitagliptin	100 mg daily	Metformin	7		Merck Sharp and Dohme
**Arechavaleta 2011**	NCT00701090	2011	Multinational	Sulfonylureas	519	Glimepiride	1-6 mg daily	Metformin	7		Merck Sharp and Dohme
**Arjona Ferreira 2013**	NCT00509236	2013	Multinational	DPP-4 inhibitors	64	Sitagliptin	25 mg daily	None	12.4		Merck Sharp and Dohme
**Arjona Ferreira 2013**	NCT00509236	2013	Multinational	Sulfonylureas	65	Glipizide	2.5-20 mg daily	None	12.4	Dialysis	Merck Sharp and Dohme
**Arjona Ferreira 2013a**	NCT00509262	2013	Multinational	DPP-4 inhibitors	211	Sitagliptin	25-50 mg daily	None	12.4		Merck Sharp and Dohme
**Arjona Ferreira 2013a**	NCT00509262	2013	Multinational	Sulfonylureas	212	Glipizide	2.5-20 mg daily	None	12.4	Chronic kidney disease	Merck Sharp and Dohme
**Arturi 2017**	Not provided	2017	Italy	Basal insulin	12	Insulin glargine	Titrated	Metformin +/- sulfonylurea	12	Heart failure	None
**Arturi 2017**	Not provided	2017	Italy	DPP-4 inhibitors	10	Sitagliptin	100 mg daily	Metformin +/- sulfonylurea	12		None
**Arturi 2017**	Not provided	2017	Italy	GLP-1 receptor agonists	10	Liraglutide	1.8 mg daily	Metformin +/- sulfonylurea	12		None

Study ID	Registration	Year	Country	Intervention	N	Drug	Dose	Cointervention	Follow -up*	Settings	Fundings
**Asian Acarbose Study**	Not provided	1998	Multinational	Alpha-glucosidase inhibitors	59	Acarbose	300 mg daily	None	5.5		Bayer
**Asian Acarbose Study**	Not provided	1998	Multinational	Standard treatments	62	Placebo		None	5.5		Bayer
**Aso 2019**	Not provided	2019	Unclear	SGLT2 inhibitors	33	Dapagliflozin	5 mg daily	Unclear	5.5		Not reported
**Aso 2019**	Not provided	2019	Unclear	Standard treatments	24	Control		Unclear	5.5		Not reported
**Avilés-Santa 1999**	Not provided	1999	USA	Metformin	21	Metformin	2000 mg daily	Insulin	5.5		Bristol-Myers Squibb
**Avilés-Santa 1999**	Not provided	1999	USA	Standard treatments	22	Placebo		Insulin	5.5		Bristol-Myers Squibb
**AWARD-1**	NCT01064687	2014	Multinational	GLP-1 receptor agonists	276	Exenatide	10-20 mcg daily	Metformin + thiazolidinedione	6		Eli Lilly
**AWARD-1**	NCT01064687	2014	Multinational	GLP-1 receptor agonists	279	Dulaglutide	1.5 mg weekly	Metformin + thiazolidinedione	6		Eli Lilly
**AWARD-1**	NCT01064687	2014	Multinational	GLP-1 receptor agonists	280	Dulaglutide	0.75 mg weekly	Metformin + thiazolidinedione	6		Eli Lilly

Study ID	Registration	Year	Country	Intervention	N	Drug	Dose	Cointervention	Follow -up*	Settings	Fundings
**AWARD-1**	NCT01064687	2014	Multinational	GLP-1 receptor agonists	835	Exenatide or dulaglutide	10-20 mcg weekly/0.75-1.5 mg weekly	Metformin + thiazolidinedione	6		Eli Lilly
**AWARD-1**	NCT01064687	2014	Multinational	Standard treatments	141	Placebo		Metformin + thiazolidinedione	6		Eli Lilly
**AWARD-10**	NCT02597049	2018	Multinational	GLP-1 receptor agonists	142	Dulaglutide	1.5 mg weekly	SGLT2 inhibitor +/- metformin	5.5		Eli Lilly
**AWARD-10**	NCT02597049	2018	Multinational	GLP-1 receptor agonists	142	Dulaglutide	0.75 mg weekly	SGLT2 inhibitor +/- metformin	5.5		Eli Lilly
**AWARD-10**	NCT02597049	2018	Multinational	GLP-1 receptor agonists	284	Dulaglutide	0.75-1.5 mg weekly	SGLT2 inhibitor +/- metformin	5.5		Eli Lilly
**AWARD-10**	NCT02597049	2018	Multinational	Standard treatments	140	Placebo		SGLT2 inhibitor +/- metformin	5.5		Eli Lilly
**AWARD-2**	NCT01075282	2015	Multinational	Basal insulin	262	Insulin glargine	Titrated	Metformin + sulfonylurea	17.7		Eli Lilly
**AWARD-2**	NCT01075282	2015	Multinational	GLP-1 receptor agonists	273	Dulaglutide	1.5 mg weekly	Metformin + sulfonylurea	17.7		Eli Lilly
**AWARD-2**	NCT01075282	2015	Multinational	GLP-1 receptor agonists	272	Dulaglutide	0.75 mg weekly	Metformin + sulfonylurea	17.7		Eli Lilly

Study ID	Registration	Year	Country	Intervention	N	Drug	Dose	Cointervention	Follow -up*	Settings	Fundings
**AWARD-2**	NCT01075282	2015	Multinational	GLP-1 receptor agonists	545	Dulaglutide	0.75-1.5 mg weekly	Metformin + sulfonylurea	17.7		Eli Lilly
**AWARD-3**	NCT01126580	2014	Multinational	GLP-1 receptor agonists	269	Dulaglutide	1.5 mg weekly	None	12		Eli Lilly
**AWARD-3**	NCT01126580	2014	Multinational	GLP-1 receptor agonists	270	Dulaglutide	0.75 mg weekly	None	12		Eli Lilly
**AWARD-3**	NCT01126580	2014	Multinational	GLP-1 receptor agonists	539	Dulaglutide	0.75-1.5 mg weekly	None	12		Eli Lilly
**AWARD-3**	NCT01126580	2014	Multinational	Metformin	268	Metformin	2000 mg daily	None	12		Eli Lilly
**AWARD-4**	NCT01191268	2015	Multinational	Basal insulin	296	Insulin glargine	Titrated	Insulin + metformin	12		Eli Lilly
**AWARD-4**	NCT01191268	2015	Multinational	GLP-1 receptor agonists	295	Dulaglutide	1.5 mg weekly	Insulin + metformin	12		Eli Lilly
**AWARD-4**	NCT01191268	2015	Multinational	GLP-1 receptor agonists	293	Dulaglutide	0.75 mg weekly	Insulin + metformin	12		Eli Lilly
**AWARD-4**	NCT01191268	2015	Multinational	GLP-1 receptor agonists	588	Dulaglutide	0.75-1.5 mg weekly	Insulin + metformin	12		Eli Lilly

Study ID	Registration	Year	Country	Intervention	N	Drug	Dose	Cointervention	Follow-up*	Settings	Fundings
**AWARD-5**	NCT00734474	2014	Multinational	DPP-4 inhibitors	315	Sitagliptin	100 mg daily	Metformin	6		Eli Lilly
**AWARD-5**	NCT00734474	2014	Multinational	GLP-1 receptor agonists	304	Dulaglutide	1.5 mg weekly	Metformin	6		Eli Lilly
**AWARD-5**	NCT00734474	2014	Multinational	GLP-1 receptor agonists	302	Dulaglutide	0.75 mg weekly	Metformin	6		Eli Lilly
**AWARD-5**	NCT00734474	2014	Multinational	GLP-1 receptor agonists	606	Dulaglutide	0.75-1.5 mg weekly	Metformin	6		Eli Lilly
**AWARD-5**	NCT00734474	2014	Multinational	Standard treatments	177	Placebo		Metformin	6		Eli Lilly
**AWARD-7**	NCT01621178	2018	Multinational	Basal insulin	194	Insulin glargine	Titrated	Insulin +/- hypoglycaemic agent	5.5		Eli Lilly
**AWARD-7**	NCT01621178	2018	Multinational	GLP-1 receptor agonists	193	Dulaglutide	1.5 mg weekly	Insulin +/- hypoglycaemic agent	5.5	Chronic kidney disease	Eli Lilly
**AWARD-7**	NCT01621178	2018	Multinational	GLP-1 receptor agonists	190	Dulaglutide	0.75 mg weekly	Insulin +/- hypoglycaemic agent	5.5		Eli Lilly
**AWARD-7**	NCT01621178	2018	Multinational	GLP-1 receptor agonists	383	Dulaglutide	0.75-1.5 mg weekly	Insulin +/- hypoglycaemic agent	5.5		Eli Lilly

Study ID	Registration	Year	Country	Intervention	N	Drug	Dose	Cointervention	Follow -up*	Settings	Fundings
**AWARD-8**	NCT01769378	2016	Multinational	GLP-1 receptor agonists	239	Dulaglutide	1.5 mg weekly	Sulfonylurea	5.5		Eli Lilly
**AWARD-8**	NCT01769378	2016	Multinational	Standard treatments	60	Placebo		Sulfonylurea	5.5		Eli Lilly
**AWARD-9**	NCT02152371	2017	Multinational	GLP-1 receptor agonists	150	Dulaglutide	1.5 mg weekly	Insulin glargine metformin	6		Eli Lilly
**AWARD-9**	NCT02152371	2017	Multinational	Standard treatments	150	Placebo		Insulin glargine metformin	6		Eli Lilly
**AWARD-CHN1**	NCT01644500	2018	China, South Korea and Taiwan	GLP-1 receptor agonists	244	Dulaglutide	1.5 mg weekly	None	6		Eli Lilly
**AWARD-CHN1**	NCT01644500	2018	China, South Korea and Taiwan	GLP-1 receptor agonists	248	Dulaglutide	0.75 mg weekly	None	6		Eli Lilly
**AWARD-CHN1**	NCT01644500	2019	China, South Korea and Taiwan	GLP-1 receptor agonists	492	Dulaglutide	0.75-1.5 mg weekly	None	6		Eli Lilly
**AWARD-CHN1**	NCT01644500	2018	China, South Korea and Taiwan	Sulfonylureas	245	Glimepiride	1-3 mg daily	None	6		Eli Lilly
**Ba 2017**	NCT01590771	2017	China	DPP-4 inhibitors	249	Sitagliptin	100 mg daily	Sulfonylurea metformin	5.5		Merck

Study ID	Registration	Year	Country	Intervention	N	Drug	Dose	Cointervention	Follow -up*	Settings	Fundings
**Ba 2017**	NCT01590771	2017	China	Standard treatments	249	Placebo		Sulfonylurea metformin	5.5		Merck
**Babar-2021**	Not provied	2021	Pakistan	SGLT2 inhibitors	120	Empagliflozin	10 mg twice a day	metformin, Sitagliptin	5.5	Obese	Unclear
**Babar-2021**	Not provied	2021	Pakistan	Standard treatments	120	Placebo		metformin, Sitagliptin	5.5		Unclear
**Bachmann 2003**	Not applicable	2003	Europe	Alpha-glucosidase inhibitors	184	Acarbose	100 mg daily	Sulfonylurea	18		Bayer
**Bachmann 2003**	Not applicable	2003	Europe	Standard treatments	189	Placebo		Sulfonylurea	18		Bayer
**Bae-2021**	NCT04013581	2021	South Korea	SGLT2 inhibitors	60	Empagliflozin	10 mg once daily	pre-existing triple therapy, metformin,sulfonyl urea,DPP-4 inhibitor	5.5		Yuhan Corporation
**Bae-2021**	NCT04013581	2021	South Korea	Thiazolidinediones	59	Pioglitazone	15 mg once daily	pre-existing triple therapy, metformin,sulfonyl urea,DPP-4 inhibitor	5.5		Yuhan Corporation
**Bailey 2010**	NCT00528879	2010	Multinational	SGLT2 inhibitors	135	Dapagliflozin	10 mg daily	Metformin	5.5		Bristol-Myers Squibb/AstraZeneca
**Bailey 2010**	NCT00528879	2010	Multinational	SGLT2 inhibitors	137	Dapagliflozin	5 mg daily	Metformin	5.5		Bristol-Myers Squibb/AstraZeneca

Study ID	Registration	Year	Country	Intervention	N	Drug	Dose	Cointervention	Follow-up*	Settings	Fundings
**Bailey 2010**	NCT00528879	2010	Multinational	SGLT2 inhibitors	137	Dapagliflozin	2.5 mg daily	Metformin	5.5		Bristol-Myers Squibb/AstraZeneca
**Bailey 2010**	NCT00528879	2010	Multinational	SGLT2 inhibitors	409	Dapagliflozin	2.5-10 mg daily	Metformin	5.5		Bristol-Myers Squibb/AstraZeneca
**Bailey 2010**	NCT00528879	2010	Multinational	Standard treatments	137	Placebo		Metformin	5.5		Bristol-Myers Squibb/AstraZeneca
**Bailey 2012**	Not provided	2012	Multinational	SGLT2 inhibitors	68	Dapagliflozin	5 mg daily	None	5.5		Bristol-Myers Squibb/AstraZeneca
**Bailey 2012**	Not provided	2012	Multinational	SGLT2 inhibitors	74	Dapagliflozin	2.5 mg daily	None	5.5		Bristol-Myers Squibb/AstraZeneca
**Bailey 2012**	Not provided	2012	Multinational	SGLT2 inhibitors	72	Dapagliflozin	1 mg daily	None	5.5		Bristol-Myers Squibb/AstraZeneca
**Bailey 2012**	Not provided	2012	Multinational	SGLT2 inhibitors	214	Dapagliflozin	1-5 mg daily	None	5.5		Bristol-Myers Squibb/AstraZeneca
**Bailey 2012**	Not provided	2012	Multinational	Standard treatments	68	Placebo		None	5.5		Bristol-Myers Squibb/AstraZeneca
**Bajaj 2014**	NCT00996658	2014	Multinational	DPP-4 inhibitors	183	Linagliptin	5 mg daily	Metformin + thiazolidinedione	5.5		Boehringer Ingelheim
**Bajaj 2014**	NCT00996658	2014	Multinational	Standard treatments	89	Placebo		Metformin + thiazolidinedione	5.5		Boehringer Ingelheim
**Bakris 2006**	Not provided	2006	Multinational	Sulfonylureas	185	Glyburide	5 mg daily	Metformin	7.4	Chronic kidney disease	GlaxoSmithKline
**Bakris 2006**	Not provided	2006	Multinational	Thiazolidinediones	204	Rosiglitazone	4 mg daily	Metformin	7.4		GlaxoSmithKline

Study ID	Registration	Year	Country	Intervention	N	Drug	Dose	Cointervention	Follow -up*	Settings	Fundings
**BALANCE**	NCT02126358	2018	Korea	DPP-4 inhibitors	96	Gemigliptin	50 mg daily	Metformin	5.5		LG Chem
**BALANCE**	NCT02126358	2018	Korea	Standard treatments	97	Standard care		Metformin	5.5		LG Chem
**Banerji 1995**	Not applicable	1995	United Kingdom	Standard treatments	10	Placebo		None	36		Pfizer Pharmaceuticals
**Banerji 1995**	Not applicable	1995	United Kingdom	Sulfonylureas	10	Glipizide		None	36		Pfizer Pharmaceuticals
**Barnett 2003**	Not applicable	2003	United Kingdom	Standard treatments	87	Placebo		Sulphonylurea	5.5		SmithKlineBeecham Pharmaceuticals
**Barnett 2003**	Not applicable	2003	United Kingdom	Thiazolidinediones	84	Rosiglitazone	4 mg daily	Sulphonylurea	5.5		SmithKlineBeecham Pharmaceuticals
**Barnett 2012**	NCT00757588	2012	Multicentre	DPP-4 inhibitors	304	Saxagliptin	5 mg daily	Metformin insulin	5.5		Bristol-Myers Squibb/AstraZeneca
**Barnett 2012**	NCT00757588	2012	Multicentre	Standard treatments	151	Placebo		Metformin insulin	5.5		Bristol-Myers Squibb/AstraZeneca
**Barnett 2013**	NCT01084005	2013	Multinational	DPP-4 inhibitors	162	Linagliptin	5 mg daily	Metformin sulfonylurea basal insulin	6		Boehringer Ingelheim
**Barnett 2013**	NCT01084005	2013	Multinational	Standard treatments	79	Placebo		Metformin sulfonylurea basal insulin	6		Boehringer Ingelheim
**Barzilai 2011**	NCT00305604	2011	USA	DPP-4 inhibitors	102	Sitagliptin	50-100 mg daily	None	5.5		Merck & Co Inc

Study ID	Registration	Year	Country	Intervention	N	Drug	Dose	Cointervention	Follow -up*	Settings	Fundings
**Barzilai 2011**	NCT00305604	2011	USA	Standard treatments	104	Placebo		None	5.5		Merck & Co Inc
**Bayrasheva 2020**	Not provided	2020	Russia	DPP-4 inhibitors	23	Vildagliptin	50 mg daily	Insulin	6		Russia Science Foundation
**Bayrasheva 2020**	Not provided	2020	Russia	Standard treatments	21	Control		Insulin	6		Russia Science Foundation
**BEGIN: ADD TO GLP-1**	NCT01664247	2016	Multinational	Basal insulin	174	Insulin degludec	Titrated	Metformin sulfonylurea, glinide, DPP-4 inhibitor, or exenatide plus liraglutide	6		Novo Nordisk
**BEGIN: ADD TO GLP-1**	NCT01664247	2016	Multinational	Standard treatments	172	Placebo		Metformin sulfonylurea, glinide, DPP-4 inhibitor, or exenatide plus liraglutide	6		Novo Nordisk
**BEGIN: VICTOZA ADD-ON**	NCT01388361	2014	Multinational	Bolus insulin	89	Insulin aspart	Titrated	Metformin + basal insulin	6		Novo Nordisk
**BEGIN: VICTOZA ADD-ON**	NCT01388361	2014	Multinational	GLP-1 receptor agonists	88	Liraglutide	1.8 mg daily	Metformin + basal insulin	6		Novo Nordisk

Study ID	Registration	Year	Country	Intervention	N	Drug	Dose	Cointervention	Follow -up*	Settings	Fundings
**Berberoglu 2010**	Not provided	2010	Turkey	Standard treatments	23	Control		None	24		Not reported
**Berberoglu 2010**	Not provided	2010	Turkey	Thiazolidinediones	26	Rosiglitazone	4 mg daily	None	24		Not reported
**Bergenstal 2009**	NCT00097877	2009	USA	Basal-Bolus insulin	124	Insulin aspart 70/30	Titrated	Metformin + sulfonylurea	6		Novo Nordisk
**Bergenstal 2009**	NCT00097877	2009	USA	Basal-Bolus insulin	124	Insulin aspart 70/30	Titrated	Metformin + sulfonylurea	6		Novo Nordisk
**Bergenstal 2009**	NCT00097877	2009	USA	Basal-Bolus insulin	248	Insulin aspart 70/30	Titrated	Metformin + sulfonylurea	6		Novo Nordisk
**Bergenstal 2009**	NCT00097877	2009	USA	GLP-1 receptor agonists	124	Exenatide	10-20 mcg daily	Metformin + sulfonylurea	6		Novo Nordisk
**Berndt-Zipfel 2013**	Not provided	2013	Germany	DPP-4 inhibitors	22	Vildagliptin	100 mg daily	Metformin	5.5		Novartis
**Berndt-Zipfel 2013**	Not provided	2013	Germany	Sulfonylureas	22	Glimepiride	4 mg daily	Metformin	5.5		Novartis
**BEST**	NCT00420511	2010	Canada	DPP-4 inhibitors	10	Sitagliptin	100 mg daily	Metformin	11.2		Merck Frosst Canada
**BEST**	NCT00420511	2010	Canada	Standard treatments	11	Placebo		Metformin	11.2		Merck Frosst Canada
**BETA**	NCT00562172	2010	Republic of Korea	Basal insulin	39	Insulin glargine	Titrated	Metformin	12		Sanofi Korea
**BETA**	NCT00562172	2010	Republic of Korea	Sulfonylureas	36	Glimepiride	8 mg daily	Metformin	12		Sanofi Korea

Study ID	Registration	Year	Country	Intervention	N	Drug	Dose	Cointervention	Follow -up*	Settings	Fundings
**BEYOND**	NCT04196231	2021	Italy	Bolus insulin	101	Rapid insulin analog	three injections at meals	basal insulin injection at bedtime	6		Associazione “Salute con Stile”
**BEYOND**	NCT04196231	2021	Italy	SGLT2 inhibitors	102	Canagliflozin, empagliflozin, or dapagliflozin	a single full dose at the main meal of the day	basal insulin injection at bedtime	6		Associazione “Salute con Stile”
**Bi 2013**	ChiCTR-TRC-10000941	2013	China	Metformin	80	Metformin	1500 mg daily	None	5.5		Pfizer
**Bi 2013**	ChiCTR-TRC-10000941	2013	China	Sulfonylureas	80	Glipizide	15 mg daily	None	5.5		Pfizer
**Bilezikian 2013**	NCT00679939	2013	USA	Metformin	111	Metformin	1000-2000 mg daily	None	12		GlaxoSmithKline
**Bilezikian 2013**	NCT00679939	2013	USA	Thiazolidinediones	114	Rosiglitazone	4-8 mg daily	None	12		GlaxoSmithKline
**Birkeland 1994a**	Not applicable	1994	Norway	Basal insulin	18	Basal and bolus insulin	Titrated	None	12		Novo Nordisk
**Birkeland 1994a**	Not applicable	1994	Norway	Sulfonylureas	16	Glibenclamide	10.5 mg daily	None	12		Novo Nordisk
**Bode 2013**	NCT01106651	2013	Multinational	SGLT2 inhibitors	236	Canagliflozin	300 mg daily	None or monotherapy/combination therapy (including metformin,	6		Janssen

Study ID	Registration	Year	Country	Intervention	N	Drug	Dose	Cointervention	Follow-up*	Settings	Fundings
								sulfonylurea, DPP-4 inhibitor, alpha glucosidase inhibitor, GLP-1 agonist, or insulin)			
**Bode 2013**	NCT01106651	2013	Multinational	SGLT2 inhibitors	241	Canagliflozin	100 mg daily	None or monotherapy/combination therapy (including metformin, sulfonylurea, DPP-4 inhibitor, alpha glucosidase inhibitor, GLP-1 agonist, or insulin)	6		Janssen
**Bode 2013**	NCT01106651	2013	Multinational	SGLT2 inhibitors	477	Canagliflozin	100-300 mg daily	None or monotherapy/combination therapy (including metformin, sulfonylurea, DPP-4 inhibitor, alpha glucosidase inhibitor, GLP-1 agonist, or insulin)	6		Janssen

Study ID	Registration	Year	Country	Intervention	N	Drug	Dose	Cointervention	Follow -up*	Settings	Fundings
**Bode 2013**	NCT01106651	2013	Multinational	Standard treatments	237	Placebo		None or monotherapy/combination therapy (including metformin, sulfonylurea, DPP-4 inhibitor, alpha glucosidase inhibitor, GLP-1 agonist, or insulin)	6		Janssen
**Bolinder 2012**	NCT00855166	2012	Multinational	SGLT2 inhibitors	91	Dapagliflozin	10 mg daily	Metformin	24		Bristol-Myers Squibb/AstraZeneca
**Bolinder 2012**	NCT00855166	2012	Multinational	Standard treatments	91	Placebo		Metformin	24		Bristol-Myers Squibb/AstraZeneca
**Bolli 2009**	Not provided	2009	Italy	DPP-4 inhibitors	295	Vildagliptin	100 mg daily	Metformin	12		Novartis
**Bolli 2009**	Not provided	2009	Italy	Thiazolidinediones	281	Pioglitazone	30 mg daily	Metformin	12		Novartis
**Borges 2011**	NCT00386100	2011	Multinational	Standard treatments	340	Control		None	20		GlaxoSmithKline
**Borges 2011**	NCT00386100	2011	Multinational	Thiazolidinediones	348	Rosiglitazone	4-8 mg daily	None	20		GlaxoSmithKline
**Bosi 2007**	NCT00099892	2007	Multinational	DPP-4 inhibitors	185	Vildagliptin	100 mg daily	Metformin	5.5		Novartis

Study ID	Registration	Year	Country	Intervention	N	Drug	Dose	Cointervention	Follow-up*	Settings	Fundings
**Bosi 2007**	NCT00099892	2007	Multinational	DPP-4 inhibitors	177	Vildagliptin	50 mg daily	Metformin	5.5		Novartis
**Bosi 2007**	NCT00099892	2007	Multinational	DPP-4 inhibitors	362	Vildagliptin	50-100 mg daily	Metformin	5.5		Novartis
**Bosi 2007**	NCT00099892	2007	Multinational	Standard treatments	182	Placebo		Metformin	5.5		Novartis
**Bosi 2009**	NCT00382096 and NCT00468039	2009	Multinational	DPP-4 inhibitors	295	Vildagliptin	100 mg daily	None	6		Novartis
**Bosi 2009**	NCT00382096 and NCT00468039	2009	Multinational	Standard treatments	294	Control		None	6		Novartis
**Bosi 2011**	NCT00432276	2011	Multinational	DPP-4 inhibitors	404	Alogliptin	25 mg daily	Metformin + thiazolidinedione	12		Takeda
**Bosi 2011**	NCT00432276	2011	Multinational	Standard treatments	399	Control		Metformin + thiazolidinedione	12		Takeda
**Bouchi 2017**	UMIN000015867	2017	Japan	GLP-1 receptor agonists	9	Liraglutide	0.9 mg daily	Insulin	5.5		Government
**Bouchi 2017**	UMIN000015867	2017	Japan	Standard treatments	10	Control		Insulin	5.5		Government
**Braun 1996**	Not applicable	1996	Germany	Alpha-glucosidase inhibitors	80	Acarbose	300 mg daily	None	5.5		None reported
**Braun 1996**	Not applicable	1996	Germany	Standard treatments	72	Placebo		None	5.5		None reported

Study ID	Registration	Year	Country	Intervention	N	Drug	Dose	Cointervention	Follow-up*	Settings	Fundings
**BRL-049653/334**	Not provided	2007	Sweden	Standard treatments	101	Placebo		Metformin or sulfonylurea	12		GlaxoSmithKline
**BRL-049653/334**	Not provided	2007	Sweden	Thiazolidinediones	99	Rosiglitazone	4 mg daily	Metformin or sulfonylurea	12	Coronary artery disease	GlaxoSmithKline
**Bryson 2016**	Not provided	2016	Multinational	DPP-4 inhibitors	88	Tenegliptin	40 mg daily	Metformin	5.5		Mitsubishi Tanabe Pharma
**Bryson 2016**	Not provided	2016	Multinational	DPP-4 inhibitors	91	Tenegliptin	20 mg daily	Metformin	5.5		Mitsubishi Tanabe Pharma
**Bryson 2016**	Not provided	2016	Multinational	DPP-4 inhibitors	93	Tenegliptin	10 mg daily	Metformin	5.5		Mitsubishi Tanabe Pharma
**Bryson 2016**	Not provided	2016	Multinational	DPP-4 inhibitors	87	Tenegliptin	5 mg daily	Metformin	5.5		Mitsubishi Tanabe Pharma
**Bryson 2016**	Not provided	2016	Multinational	DPP-4 inhibitors	359	Tenegliptin	5-40 mg daily	Metformin	5.5		Mitsubishi Tanabe Pharma
**Bryson 2016**	Not provided	2016	Multinational	Standard treatments	88	Placebo		Metformin	5.5		Mitsubishi Tanabe Pharma
**Bunck 2009**	NCT00097500	2009	Multinational	Basal insulin	33	Insulin glargine	Titrated	Metformin	12		Amylin Pharmaceuticals/Eli Lilly
**Bunck 2009**	NCT00097500	2009	Multinational	GLP-1 receptor agonists	36	Exenatide	60 mcg daily	Metformin	12		Amylin Pharmaceuticals/Eli Lilly
**Buse 2011**	NCT00765817	2011	Multinational	GLP-1 receptor agonists	138	Exenatide	20 mcg daily	Metformin thiazolidinedione insulin	7		Amylin Pharmaceuticals/Eli Lilly

Study ID	Registration	Year	Country	Intervention	N	Drug	Dose	Cointervention	Follow -up*	Settings	Fundings
**Buse 2011**	NCT00765817	2011	Multinational	Standard treatments	122	Placebo		Metformin thiazolidinedione insulin	7		Amylin Pharmaceuticals/Eli Lilly
**Cai-2021**	NCT01648582	2021	China	Basal insulin	10	Insulin glargine	6 unit/day	original therapeutic drug type and dose	12		National Key R&D Program of China
**Cai-2021**	NCT01648582	2021	China	GLP-1 receptor agonists	19	Exenatide	2 mg/week	original therapeutic drug type and dose	12		National Key R&D Program of China
**Cai-2021**	NCT01648582	2021	China	GLP-1 receptor agonists	19	Dulaglutide	1.5mg/wee k	original therapeutic drug type and dose	12		National Key R&D Program of China
**Cai-2021**	NCT01648582	2021	China	GLP-1 receptor agonists	38	Exenatide or Dulaglutide	2 mg or 1.5 mg weekly	original therapeutic drug type and dose	12		National Key R&D Program of China
**Cai-2021**	NCT01648582	2021	China	Standard treatments	17	Placebo		original therapeutic drug type and dose	12		National Key R&D Program of China
**Camerini-Davalos 1988**	Not applicable	1988	USA	Standard treatments	31	Placebo		None	24		Pfizer
**Camerini-Davalos 1988**	Not applicable	1988	USA	Sulfonylureas	46	Glipizide	20-40 mg daily	None	24		Pfizer
**Camerini-Davalos 1994**	Not applicable	1984	USA	Standard treatments	30	Placebo		Oral drugs insulin	36		Roerig-Pfizer Pharmaceuticals
**Camerini-Davalos 1994**	Not applicable	1984	USA	Sulfonylureas	40	Glipizide	5 mg daily	Oral drugs insulin	36		Roerig-Pfizer Pharmaceuticals

**Study ID**	**Registration**	**Year**	**Country**	**Intervention**	**N**	**Drug**	**Dose**	**Cointervention**	**Follow -up***	**Settings**	**Fundings**
**Campbell 1994**	Not applicable	1994	UK	Metformin	24	Metformin	3000 mg daily	None	12		Not reported
**Campbell 1994**	Not applicable	1994	UK	Sulfonylureas	24	Glipizide	30 mg daily	None	12		Not reported
**CANDLE**	UMIN000017669	2020	Japan	SGLT2 inhibitors	122	Canagliflozin	100 mg daily	Oral drugs insulin	5.5		Mitsubishi Tanabe Pharma Corporation
**CANDLE**	UMIN000017669	2020	Japan	Sulfonylureas	123	Glimepiride	6 mg daily	Oral drugs insulin	5.5	Heart failure	Mitsubishi Tanabe Pharma Corporation
**CANTATA-D**	NCT01106677	2013	Multinational	DPP-4 inhibitors	366	Sitagliptin	100 mg daily	Metformin	6		Janssen
**CANTATA-D**	NCT01106677	2013	Multinational	SGLT2 inhibitors	367	Canagliflozin	300 mg daily	Metformin	6		Janssen
**CANTATA-D**	NCT01106677	2013	Multinational	SGLT2 inhibitors	368	Canagliflozin	100 mg daily	Metformin	6		Janssen
**CANTATA-D**	NCT01106677	2013	Multinational	SGLT2 inhibitors	735	Canagliflozin	100-300 mg daily	Metformin	6		Janssen
**CANTATA-D2**	NCT01137812	2013	Multinational	DPP-4 inhibitors	378	Sitagliptin	100 mg daily	Metformin + sulfonylurea	12		Janssen
**CANTATA-D2**	NCT01137812	2013	Multinational	SGLT2 inhibitors	378	Canagliflozin	300 mg daily	Metformin + sulfonylurea	12		Janssen
**CANTATA-M**	NCT01081834	2013	Multinational	SGLT2 inhibitors	197	Canagliflozin	300 mg daily	None	6		Janssen
**CANTATA-M**	NCT01081834	2013	Multinational	SGLT2 inhibitors	195	Canagliflozin	100 mg daily	None	6		Janssen

Study ID	Registration	Year	Country	Intervention	N	Drug	Dose	Cointervention	Follow -up*	Settings	Fundings
**CANTATA-M**	NCT01081834	2013	Multinational	SGLT2 inhibitors	392	Canagliflozin	100-300 mg daily	None	6		Janssen
**CANTATA-M**	NCT01081834	2013	Multinational	Standard treatments	192	Placebo		None	6		Janssen
**CANTATA-MSU**	NCT01106625	2013	Multinational	SGLT2 inhibitors	156	Canagliflozin	300 mg daily	Metformin + sulfonylurea	5.5		Janssen
**CANTATA-MSU**	NCT01106625	2013	Multinational	SGLT2 inhibitors	157	Canagliflozin	100 mg daily	Metformin + sulfonylurea	5.5		Janssen
**CANTATA-MSU**	NCT01106625	2013	Multinational	SGLT2 inhibitors	313	Canagliflozin	100-300 mg daily	Metformin + sulfonylurea	5.5		Janssen
**CANTATA-MSU**	NCT01106625	2013	Multinational	Standard treatments	156	Placebo		Metformin + sulfonylurea	5.5		Janssen
**CANTATA-SU**	NCT00968812	2013	Multinational	SGLT2 inhibitors	485	Canagliflozin	300 mg daily	Metformin	12		Janssen
**CANTATA-SU**	NCT00968812	2013	Multinational	SGLT2 inhibitors	483	Canagliflozin	100 mg daily	Metformin	12		Janssen
**CANTATA-SU**	NCT00968812	2013	Multinational	SGLT2 inhibitors	968	Canagliflozin	100-300 mg daily	Metformin	12		Janssen
**CANTATA-SU**	NCT00968812	2013	Multinational	Sulfonylureas	482	Glimepiride	6-8 mg daily	Metformin	12		Janssen
**CANVAS/CANVAS-R**	NCT01032629/NCT01989754	2017	Multinational	SGLT2 inhibitors	5795	Canagliflozin	100-300 mg daily	Monotherapy or combination therapy of any approved agent	43	High cardiovascular risk	Janssen

Study ID	Registration	Year	Country	Intervention	N	Drug	Dose	Cointervention	Follow -up*	Settings	Fundings
**CANVAS/CANVAS-R**	NCT01032629/NCT01989754	2017	Multinational	Standard treatments	4347	Placebo		Monotherapy or combination therapy of any approved agent	43		Janssen
**Carlson 2019**	Not provided	2019	USA	Basal insulin	17	Insulin glargine		Metformin and/or sulfonylurea	7.8		Sanofi-Aventis
**Carlson 2019**	Not provided	2019	USA	GLP-1 receptor agonists	16	Exenatide	10-20 mcg	Metformin and/or sulfonylurea	7.8		Sanofi-Anetis
**CARMELINA**	NCT01897532	2019	Multinational	DPP-4 inhibitors	3494	Linagliptin	5 mg daily	Additional medications except DPP-4 inhibitors, GLP 1 receptor agonists or SGLT-2 inhibitors	26.4	High cardiovascular /renal risk	Boehringer Ingelheim and Eli Lilly.
**CARMELINA**	NCT01897532	2019	Multinational	Standard treatments	3485	Placebo		Additional medications except DPP-4 inhibitors, GLP 1 receptor agonists or SGLT-2 inhibitors	26.4		Boehringer Ingelheim and Eli Lilly.
**CAROLINA**	NCT01243424	2019	Multinational	DPP-4 inhibitors	3023	Linagliptin	5 mg daily	Additiona medications including adjustment of	75.6		Boehringer Ingelheim and Eli Lilly.

Study ID	Registration	Year	Country	Intervention	N	Drug	Dose	Cointervention	Follow -up*	Settings	Fundings
								background therapy, or addition of pioglitazone, metformin, alpha glucosidase inhibitor or basal insulin			
**CAROLINA**	NCT01243424	2019	Multinational	Sulfonylureas	3010	Glimepiride	1-4 mg daily	Additiona medications including adjustment of background therapy, or addition of pioglitazone, metformin, alpha glucosidase inhibitor or basal insulin	75.6	High cardiovascular risk	Boehringer Ingelheim and Eli Lilly.
**Casner 1988**	Not applicable	1988	USA	Standard treatments	33	Placebo		Insulin	12		Not reported
**Casner 1988**	Not applicable	1988	USA	Sulfonylureas	31	Glyburide	5 mg daily	Insulin	12		Not reported
**Cefalu 2015**	NCT01031680	2015	Multinational	SGLT2 inhibitors	460	Dapagliflozin	10 mg daily	Drugs excluding thiazolidinedione	12	Cardiovascular disease	Astra Zeneca / Bristol- Myers Squibb

Study ID	Registration	Year	Country	Intervention	N	Drug	Dose	Cointervention	Follow -up*	Settings	Fundings
**Cefalu 2015**	NCT01031680	2015	Multinational	Standard treatments	462	Placebo		Drugs excluding thiazolidinedione	12		Astra Zeneca / Bristol- Myers Squibb
**Chacra 2017**	NCT01698775	2017	Multinational	DPP-4 inhibitors	107	Omargliptin	12.5-25 mg daily	None, 1, or 2 oral agents	5.5	Chronic kidney disease	Merck Sharp & Dohme
**Chacra 2017**	NCT01698775	2017	Multinational	Standard treatments	106	Placebo		None, 1, or 2 oral agents	5.5		Merck Sharp & Dohme
**Chakraborty 2011**	Not provided	2011	India	Metformin	110	Metformin	850-2000 mg daily	None	5.5		RSSDI
**Chakraborty 2011**	Not provided	2011	India	Standard treatments	98	Placebo		None	5.5		RSSDI
**Charbonnel 2013**	NCT01296412	2013	Multinational	DPP-4 inhibitors	326	Sitagliptin	100 mg daily	Metformin	6		Merck Sharp & Dohme
**Charbonnel 2013**	NCT01296412	2013	Multinational	GLP-1 receptor agonists	327	Liraglutide	0.6-1.2 mg daily	Metformin	6		Merck Sharp & Dohme
**Charpentier 2009**	Not provided	2009	France	Standard treatments	154	Placebo		Metformin + sulfonylurea or metiglinide	7		Takeda
**Charpentier 2009**	Not provided	2009	France	Thiazolidinediones	145	Pioglitazone	30-45 mg daily	Metformin + sulfonylurea or metiglinide	7		Takeda
**Chehrehgosha-2021**	IRCT20190122042450N3	2021	Iran	SGLT2 inhibitors	35	Empagliflozin	10 mg once daily	None	5.5	NAFLD	Unclear

Study ID	Registration	Year	Country	Intervention	N	Drug	Dose	Cointervention	Follow -up*	Settings	Fundings
**Chehrehgosha-2021**	IRCT20190122042450N3	2021	Iran	Standard treatments	37	Placebo		None	5.5		Unclear
**Chehrehgosha-2021**	IRCT20190122042450N3	2021	Iran	Thiazolidinediones	34	Pioglitazone	30 mg once daily	None	5.5		Unclear
**Chen 2015**	NCT01214239	2015	Multinational	DPP-4 inhibitors	200	Linagliptin	5 mg daily	None	5.5		Boehringer Ingelheim, Pharma
**Chen 2015**	NCT01214239	2015	Multinational	Standard treatments	99	Placebo		None	5.5		Boehringer Ingelheim, Pharma
**Chen 2016**	NCT01175486	2016	Taiwan	Alpha-glucosidase inhibitors	30	Acarbose	150 mg daily	Metformin + sulfonylurea	6		Taipei Veterans General Hospital
**Chen 2016**	NCT01175486	2016	Taiwan	Thiazolidinediones	30	Pioglitazone	30 mg daily	Metformin + sulfonylurea	6		Taipei Veterans General Hospital
**Cherney-2021**	NCT03242018	2021	Multinational	SGLT2 inhibitors	92	Sotagliflozin	200 mg once a day	prior background antihyperglycaemic treatments	12.92	CKD4	Lexicon Pharmaceuticals, Inc., and Sanofi
**Cherney-2021**	NCT03242018	2021	Multinational	SGLT2 inhibitors	92	Sotagliflozin	400 mg once a day	prior background antihyperglycaemic treatments	12.92		Lexicon Pharmaceuticals, Inc., and Sanofi
**Cherney-2021**	NCT03242018	2021	Multinational	SGLT2 inhibitors	184	Sotagliflozin	200-400 mg daily	prior background antihyperglycaemic treatments	12.92		Lexicon Pharmaceuticals, Inc., and Sanofi
**Cherney-2021**	NCT03242018	2021	Multinational	Standard treatments	93	Placebo		prior background antihyperglycaemic treatments	12.92		Lexicon Pharmaceuticals, Inc., and Sanofi

Study ID	Registration	Year	Country	Intervention	N	Drug	Dose	Cointervention	Follow-up*	Settings	Fundings
**Chiasson 1994**	Not applicable	1994	Canada	Alpha-glucosidase inhibitors	172	Acarbose	600 mg daily	Metformin, sulfonylurea, and insulin	12		Miles Canada
**Chiasson 1994**	Not applicable	1994	Canada	Standard treatments	182	Placebo		Metformin, sulfonylurea, and insulin	12		Miles Canada
**Chiasson 2001**	Not applicable	2001	Canada	Alpha-glucosidase inhibitors	82	Miglitol	300 mg daily	None	8.3		Bayer
**Chiasson 2001**	Not applicable	2001	Canada	Metformin	83	Metformin	1500 mg daily	None	8.3		Bayer
**Chiasson 2001**	Not applicable	2001	Canada	Standard treatments	83	Placebo		None	8.3		Bayer
**CHICAGO**	NCT00225264	2006	USA	Sulfonylureas	230	Glimepiride	1-4 mg daily	Metformin sulfonylurea insulin	18		Takeda
**CHICAGO**	NCT00225264	2006	USA	Thiazolidinediones	232	Pioglitazone	15-45 mg daily	Metformin sulfonylurea insulin	18		Takeda
**Chien 2011**	Not provided	2011	Taiwan	DPP-4 inhibitors	49	Sitagliptin	100 mg daily	Metformin, sulfonylurea, alpha-glucosidase inhibitors	5.5		Not reported
**Chien 2011**	Not provided	2011	Taiwan	Standard treatments	48	Standard care		Metformin, sulfonylurea, alpha-	5.5		Not reported

Study ID	Registration	Year	Country	Intervention	N	Drug	Dose	Cointervention	Follow -up*	Settings	Fundings
								glucosidase inhibitors			
**Cho 2019**	UMIN000022804	2019	Japan	SGLT2 inhibitors	36	Dapagliflozin	5 mg daily	Other hypoglycemic agents	5.5		None
**Cho 2019**	UMIN000022804	2019	Japan	Thiazolidinediones	35	Pioglitazone	15-30 mg daily	Other hypoglycemic agents	5.5		None
**Choi 2004**	Not applicable	2004	Republic of Korea	Standard treatments	48	Standard care		Metformin, sulphonylurea, and/or alpha-glucosidase inhibitor or insulin	6		Basic Research Program of the Korea Science & Engineering Foundation
**Choi 2004**	Not applicable	2004	Republic of Korea	Thiazolidinediones	47	Rosiglitazone	4 mg daily	Metformin, sulphonylurea, and/or alpha-glucosidase inhibitor or insulin	6	Coronary artery disease	Basic Research Program of the Korea Science & Engineering Foundation<br><br>Korea Science & Engineering Foundation.
**Chou 2008**	SB-797620/004	2008	Multinational	Sulfonylureas	225	Glimepiride	4 mg daily	None	6.4		GlaxoSmithKline
**Chou 2008**	SB-797620/004	2008	Multinational	Thiazolidinediones	232	Rosiglitazone	8 mg daily	None	6.4		GlaxoSmithKline

Study ID	Registration	Year	Country	Intervention	N	Drug	Dose	Cointervention	Follow -up*	Settings	Fundings
**Chou 2012**	NCT00484198	2012	Multinational	Standard treatments	137	Placebo		None	6		Daiichi Sankyo
**Chou 2012**	NCT00484198	2012	Multinational	Thiazolidinediones	750	Rivoglitazone	1.5 mg daily	None	6		Daiichi Sankyo
**Chou 2012**	NCT00484198	2012	Multinational	Thiazolidinediones	274	Rivoglitazone	1.0 mg daily	None	6		Daiichi Sankyo
**Chou 2012**	NCT00484198	2012	Multinational	Thiazolidinediones	751	Pioglitazone	45 mg daily	None	6		Daiichi Sankyo
**Chou 2012**	NCT00484198	2012	Multinational	Thiazolidinediones	1775	Rivoglitazone/Pioglitazone	1.0-1.5 mg/45 mg daily	None	6		Daiichi Sankyo
**Chuang-2020**	Not provied	2020	Taiwan	Metformin	48	Metformin		insulin glargine	11.08		Unclear
**Chuang-2020**	Not provied	2020	Taiwan	Sulfonylureas	47	Sulfonylurea		insulin glargine	11.08		Unclear
**CIMT**	NCT00657943	2016	Denmark	Metformin	206	Metformin	2000 mg daily	Oral drugs insulin	18		Novo Nordisk
**CIMT**	NCT00657943	2016	Denmark	Standard treatments	206	Placebo		Oral drugs insulin	18		Novo Nordisk
**Civera 2008**	Not provided	2008	Spain	Meglitinides	12	Repaglinide	6 mg daily	Metformin + insulin	5.5		Not reported
**Civera 2008**	Not provided	2008	Spain	Standard treatments	12	Standard care		Metformin + insulin	5.5		Not reported
**COMPASS**	UMIN000004716	2013	Japan	DPP-4 inhibitors	65	Sitagliptin	100 mg daily	Metformin sulfonylurea	5.5		Ministry of Education,Culture, Sports, Science andTechnology(MEXT)of Japan, a Grant

Study ID	Registration	Year	Country	Intervention	N	Drug	Dose	Cointervention	Follow -up*	Settings	Fundings
											for the Strategic Japanese-Danish Cooperative Program on Molecular Diabetology from the Japan Science and Technology Agency
**COMPASS**	UMIN000004716	2013	Japan	Thiazolidinediones	65	Pioglitazone	30 mg daily	Metformin sulfonylurea	5.5		Ministry of Education,Culture, Sports, Science andTechnology(MEXT)of Japan, a Grant for the Strategic Japanese-Danish Cooperative Program on Molecular Diabetology from the Japan Science and Technology Agency
**CompoSIT-I**	NCT02738879	2018	Multinational	DPP-4 inhibitors	374	Sitagliptin	100 mg daily	Metformin + DPP-4 inhibitor and/or sulphonylurea	7		Merck & Co
**CompoSIT-I**	NCT02738879	2018	Multinational	Standard treatments	372	Placebo		Metformin + DPP-4 inhibitor and/or sulphonylurea	7		Merck & Co

Study ID	Registration	Year	Country	Intervention	N	Drug	Dose	Cointervention	Follow -up*	Settings	Fundings
**CompoSIT-R**	NCT02532855	2018	Multinational	DPP-4 inhibitors	307	Sitagliptin	100 mg daily	Metformin alone or with sulfonylurea	5.5		Merck, Sharpe and Dohme
**CompoSIT-R**	NCT02532855	2018	Multinational	SGLT2 inhibitors	307	Dapagliflozin	10 mg daily	Metformin alone or with sulfonylurea	5.5		Merck, Sharpe and Dohme
**CONFIDENCE**	NCT01147627	2015	China	Basal-Bolus insulin	138	Premixed lispro/lispro protamine	Titrated	None	11		Government/Eli Lilly/Amylin Pharmaceuticals
**CONFIDENCE**	NCT01147627	2015	China	GLP-1 receptor agonists	142	Exenatide	10-20 mcg daily	None	11		Government/Eli Lilly/Amylin Pharmaceuticals
**CONFIDENCE**	NCT01147627	2015	China	Thiazolidinediones	136	Pioglitazone	30-45 mg daily	None	11		Government/Eli Lilly/Amylin Pharmaceuticals
**Coniff 1994**	Not applicable	1994	USA	Alpha-glucosidase inhibitors	91	Acarbose	900 mg daily	None	8.4		Miles Inc
**Coniff 1994**	Not applicable	1994	USA	Standard treatments	98	Placebo		None	8.4		Miles Inc
**Coniff 1995**	Not applicable	1995	USA	Alpha-glucosidase inhibitors	67	Acarbose	600 mg daily	None	5.5		Not reported
**Coniff 1995**	Not applicable	1995	USA	Standard treatments	62	Placebo		None	5.5		Not reported

Study ID	Registration	Year	Country	Intervention	N	Drug	Dose	Cointervention	Follow -up*	Settings	Fundings
**Coniff 1995a**	Not applicable	1995	USA	Alpha-glucosidase inhibitors	103	Acarbose	900 mg daily	Insulin	8.4		Miles Pharmaceutical
**Coniff 1995a**	Not applicable	1995	USA	Standard treatments	104	Placebo		Insulin	8.4		Miles Pharmaceutical
**Costa 1997**	Not applicable	1997	Spain	Alpha-glucosidase inhibitors	36	Acarbose	300 mg daily	None	6		Bristol-Myers Squibb/AstraZeneca
**Costa 1997**	Not applicable	1997	Spain	Standard treatments	29	Placebo		None	6		Bristol-Myers Squibb/AstraZeneca
**CREDENCE**	NCT02065791	2019	Multinational	SGLT2 inhibitors	2202	Canagliflozin	100 mg daily	Background therapy consistent with guidelines	31.4	Chronic kidney disease	Janssen Research and Development
**CREDENCE**	NCT02065791	2019	Multinational	Standard treatments	2199	Placebo		Background therapy consistent with guidelines	31.4		Janssen Research and Development
**Cusi 2019**	NCT02009488	2019	USA	SGLT2 inhibitors	26	Canagliflozin	300 mg daily	Metformin DPP-4 inhibitor	5.5		Janssen Research and Development
**Cusi 2019**	NCT02009488	2019	USA	Standard treatments	30	Placebo		Metformin DPP-4 inhibitor	5.5		Janssen Research and Development
**CV181-011**	NCT00121641	2009	Multinational	DPP-4 inhibitors	98	Saxagliptin	10 mg daily	None	5.5		Bristol-Myers Squibb/AstraZeneca
**CV181-011**	NCT00121641	2009	Multinational	DPP-4 inhibitors	106	Saxagliptin	5 mg daily	None	5.5		Bristol-Myers Squibb/AstraZeneca

Study ID	Registration	Year	Country	Intervention	N	Drug	Dose	Cointervention	Follow -up*	Settings	Fundings
**CV181-011**	NCT00121641	2009	Multinational	DPP-4 inhibitors	102	Saxagliptin	2.5 mg daily	None	5.5		Bristol-Myers Squibb/AstraZeneca
**CV181-011**	NCT00121641	2009	Multinational	DPP-4 inhibitors	306	Saxagliptin	2.5-10 mg daily	None	5.5		Bristol-Myers Squibb/AstraZeneca
**CV181-011**	NCT00121641	2009	Multinational	Standard treatments	95	Placebo		None	5.5		Bristol-Myers Squibb/AstraZeneca
**CV181-013**	NCT00295633	2011	Multinational	DPP-4 inhibitors	186	Saxagliptin	5 mg daily	Thiazolidinedione	17.5		Bristol-Myers Squibb/AstraZeneca
**CV181-013**	NCT00295633	2011	Multinational	DPP-4 inhibitors	195	Saxagliptin	2.5 mg daily	Thiazolidinedione	17.5		Bristol-Myers Squibb/AstraZeneca
**CV181-013**	NCT00295633	2011	Multinational	DPP-4 inhibitors	381	Saxagliptin	2.5-5 mg daily	Thiazolidinedione	17.5		Bristol-Myers Squibb/AstraZeneca
**CV181-013**	NCT00295633	2011	Multinational	Standard treatments	184	Placebo		Thiazolidinedione	17.5		Bristol-Myers Squibb/AstraZeneca
**CV181-039**	NCT00327015	2011	Multinational	DPP-4 inhibitors	323	Saxagliptin	10 mg daily	Metformin	18		Bristol-Myers Squibb/AstraZeneca
**CV181-039**	NCT00327015	2011	Multinational	DPP-4 inhibitors	320	Saxagliptin	5 mg daily	Metformin	18		Bristol-Myers Squibb/AstraZeneca
**CV181-039**	NCT00327015	2011	Multinational	DPP-4 inhibitors	643	Saxagliptin	5-10 mg daily	Metformin	18		Bristol-Myers Squibb/AstraZeneca
**CV181-039**	NCT00327015	2011	Multinational	Standard treatments	328	Placebo		Metformin	18		Bristol-Myers Squibb/AstraZeneca
**D-LIFT**	NCT03590626	2020	India	GLP-1 receptor agonists	32	Dulaglutide	1.5mg weekly	other treatments(metformin, DPP-4 inhibitors	5.5	NAFLD	Medanta–The Medicity's departmental research

Study ID	Registration	Year	Country	Intervention	N	Drug	Dose	Cointervention	Follow -up*	Settings	Fundings
								in the control group, sulfonylureas and/or insulin)			fund and a grant from the Endocrine and Diabetes Foundation (EDF), India.
**D-LIFT**	NCT03590626	2020	India	Standard treatments	32	Standard treatment		other treatments(metformin, DPP-4 inhibitors in the control group, sulfonylureas and/or insulin)	5.5		Medanta–The Medicity's departmental research fund and a grant from the Endocrine and Diabetes Foundation (EDF), India.
**da Silva 2016**	NCT02607410	2016	Brazil	Basal insulin	17	Insulin NPH		Metformin + sulfonylurea	12		Sao Paulo Research Foundation
**da Silva 2016**	NCT02607410	2016	Brazil	DPP-4 inhibitors	18	Sitagliptin	100 mg daily	Metformin + sulfonylurea	12		Sao Paulo Research Foundation
**Dailey 2004**	Not applicable	2004	USA	Standard treatments	184	Placebo		Metformin + sulfonylurea	5.5		Bristol-Myers Squibb
**Dailey 2004**	Not applicable	2004	USA	Thiazolidinediones	181	Rosiglitazone	4-8 mg daily	Metformin + sulfonylurea	5.5		Bristol-Myers Squibb
**DAPA-LVH**	NCT02956811	2020	UK	SGLT2 inhibitors	32	Dapagliflozin	10 mg once daily	None	12	left ventricular hypertrophy	Astra Zeneca
**DAPA-LVH**	NCT02956811	2020	UK	Standard treatments	34	Placebo		None	12		Astra Zeneca
**Dapagliflozin 006**	NCT00673231	2012	Multinational	SGLT2 inhibitors	196	Dapagliflozin	10 mg daily	Insulin	11		Bristol-Myers Squibb/AstraZeneca

Study ID	Registration	Year	Country	Intervention	N	Drug	Dose	Cointervention	Follow -up*	Settings	Fundings
**Dapagliflozin 006**	NCT00673231	2012	Multinational	SGLT2 inhibitors	212	Dapagliflozin	5 mg daily	Insulin	11		Bristol-Myers Squibb/AstraZeneca
**Dapagliflozin 006**	NCT00673231	2012	Multinational	SGLT2 inhibitors	202	Dapagliflozin	2.5 mg daily	Insulin	11		Bristol-Myers Squibb/AstraZeneca
**Dapagliflozin 006**	NCT00673231	2012	Multinational	SGLT2 inhibitors	610	Dapagliflozin	2.5-10 mg daily	Insulin	11		Bristol-Myers Squibb/AstraZeneca
**Dapagliflozin 006**	NCT00673231	2012	Multinational	Standard treatments	197	Placebo		Insulin	11		Bristol-Myers Squibb/AstraZeneca
**Dargie 2007**	GSK 49653/211	2007	Europe	Standard treatments	114	Placebo		Antidiabetes treatment except thiazolidinedione or insulin + acarbose	12		GlaxoSmithKline
**Dargie 2007**	GSK 49653/211	2007	Europe	Thiazolidinediones	110	Rosiglitazone	4-8 mg daily	Antidiabetes treatment except thiazolidinedione or insulin + acarbose	12	Heart failure	GlaxoSmithKline
**Davidson 2007**	Not provided	2007	USA	Standard treatments	124	Placebo		Sulfonylurea	5.5		GlaxoSmithKline
**Davidson 2007**	Not provided	2007	USA	Thiazolidinediones	121	Rosiglitazone	8 mg daily	Sulfonylurea	5.5		GlaxoSmithKline
**Davies 2013**	NCT01003184	2013	United Kingdom/Ireland	Basal insulin	105	Insulin detemir	Titrated	Metformin sulfonylurea	5.5		Amylin Pharmaceuticals/Eli Lilly

Study ID	Registration	Year	Country	Intervention	N	Drug	Dose	Cointervention	Follow -up*	Settings	Fundings
**Davies 2013**	NCT01003184	2013	United Kingdom/Ireland	GLP-1 receptor agonists	111	Exenatide	2 mg weekly	Metformin sulfonylurea	5.5		Amylin Pharmaceuticals/Eli Lilly
**Davies 2017**	NCT01923181	2017	Multinational	GLP-1 receptor agonists	71	Semaglutide	40 mg daily	Metformin	5.5		Novo Nordisk
**Davies 2017**	NCT01923181	2017	Multinational	GLP-1 receptor agonists	70	Semaglutide	20 mg daily	Metformin	5.5		Novo Nordisk
**Davies 2017**	NCT01923181	2017	Multinational	GLP-1 receptor agonists	69	Semaglutide	10 mg daily	Metformin	5.5		Novo Nordisk
**Davies 2017**	NCT01923181	2017	Multinational	GLP-1 receptor agonists	70	Semaglutide	5 mg daily	Metformin	5.5		Novo Nordisk
**Davies 2017**	NCT01923181	2017	Multinational	GLP-1 receptor agonists	70	Semaglutide	2.5 mg daily	Metformin	5.5		Novo Nordisk
**Davies 2017**	NCT01923181	2017	Multinational	GLP-1 receptor agonists	69	Semaglutide	1mg, subcutaneous	Metformin	5.5		Novo Nordisk
**Davies 2017**	NCT01923181	2017	Multinational	GLP-1 receptor agonists	419	Semaglutide	2.5-40 mg daily or subcutaneous 1mg	Metformin	5.5		Novo Nordisk

Study ID	Registration	Year	Country	Intervention	N	Drug	Dose	Cointervention	Follow -up*	Settings	Fundings
**Davies 2017**	NCT01923181	2017	Multinational	Standard treatments	71	Placebo		Metformin	5.5		Novo Nordisk
**DECLARE-TIMI 58**	NCT01730534	2019	Multinational	SGLT2 inhibitors	8582	Dapagliflozin	10 mg daily	Use of other glucose lowering agents at discretion (other than SGLT-2 inhibitor, pioglitazone or rosiglitazone	50.4	High cardiovascular risk	AstraZeneca
**DECLARE-TIMI 58**	NCT01730534	2019	Multinational	Standard treatments	8578	Placebo		Use of other glucose lowering agents at discretion (other than SGLT-2 inhibitor, pioglitazone or rosiglitazone	50.4		AstraZeneca
**DeFronzo 1995**	Not applicable	1995	USA	Metformin	210	Metformin	500-2500 mg daily	None	6.7		Lipha Pharmaceuticals
**DeFronzo 1995**	Not applicable	1995	USA	Sulfonylureas	209	Glyburide	10-20 mg daily	None	6.7		Lipha Pharmaceuticals
**DeFronzo 2005**	Not applicable	2005	USA	GLP-1 receptor agonists	113	Exenatide	20 mcg daily	Metformin	7		Amylin Pharmaceuticals/Eli Lilly

Study ID	Registration	Year	Country	Intervention	N	Drug	Dose	Cointervention	Follow-up*	Settings	Fundings
**DeFronzo 2005**	Not applicable	2005	USA	GLP-1 receptor agonists	110	Exenatide	10 mcg daily	Metformin	7		Amylin Pharmaceuticals/Eli Lilly
**DeFronzo 2005**	Not applicable	2005	USA	GLP-1 receptor agonists	223	Exenatide	10-20 mcg daily	Metformin	7		Amylin Pharmaceuticals/Eli Lilly
**DeFronzo 2005**	Not applicable	2005	USA	Standard treatments	113	Placebo		Metformin	7		Amylin Pharmaceuticals/Eli Lilly
**DeFronzo 2012**	NCT00328627	2012	Multinational	DPP-4 inhibitors	129	Alogliptin	25 mg daily	Metformin	6		Takeda
**DeFronzo 2012**	NCT00328627	2012	Multinational	DPP-4 inhibitors	128	Alogliptin	12.5 mg daily	Metformin	6		Takeda
**DeFronzo 2012**	NCT00328627	2012	Multinational	DPP-4 inhibitors	257	Alogliptin	12.5-25 mg daily	Metformin	6		Takeda
**DeFronzo 2012**	NCT00328627	2012	Multinational	Standard treatments	129	Placebo		Metformin	6		Takeda
**DeFronzo 2015**	NCT01422876	2015	Multinational	DPP-4 inhibitors	128	Linagliptin	5 mg daily	Metformin	12		Boehringer Ingelheim/Eli Lilly
**DeFronzo 2015**	NCT01422876	2015	Multinational	SGLT2 inhibitors	140	Empagliflozin	25 mg daily	Metformin	12		Boehringer Ingelheim/Eli Lilly
**DeFronzo 2015**	NCT01422876	2015	Multinational	SGLT2 inhibitors	137	Empagliflozin	10 mg daily	Metformin	12		Boehringer Ingelheim/Eli Lilly
**DeFronzo 2015**	NCT01422876	2015	Multinational	SGLT2 inhibitors	277	Empagliflozin	10-25 mg daily	Metformin	12		Boehringer Ingelheim/Eli Lilly

Study ID	Registration	Year	Country	Intervention	N	Drug	Dose	Cointervention	Follow -up*	Settings	Fundings
**Dei Cas 2017**	NCT01822548	2017	Italy	DPP-4 inhibitors	40	Vildagliptin	100 mg daily	Metformin	12		Italy Ministry of Health
**Dei Cas 2017**	NCT01822548	2017	Italy	Sulfonylureas	24	Glibenclamide	5-20 mg daily	Metformin	12		Italy Ministry of Health
**Dejager 2007**	NCT00099905	2007	Multinational	DPP-4 inhibitors	157	Vildagliptin	100 mg daily	None	5.5		Novartis
**Dejager 2007**	NCT00099905	2007	Multinational	DPP-4 inhibitors	152	Vildagliptin	100 mg daily	None	5.5		Novartis
**Dejager 2007**	NCT00099905	2007	Multinational	DPP-4 inhibitors	163	Vildagliptin	50 mg daily	None	5.5		Novartis
**Dejager 2007**	NCT00099905	2007	Multinational	DPP-4 inhibitors	472	Vildagliptin	50-100 mg daily	None	5.5		Novartis
**Dejager 2007**	NCT00099905	2007	Multinational	Standard treatments	160	Placebo		None	5.5		Novartis
**Del Prato 2003**	Not applicable	2003	Multinational	Metformin	284	Metformin	2550 mg daily	None	6		Servier
**Del Prato 2003**	Not applicable	2003	Multinational	Standard treatments	144	Placebo		None	6		Servier
**Del Prato 2011**	Not provided	2011	Multinational	DPP-4 inhibitors	336	Linagliptin	5 mg daily	None	5.5		Boehringer Ingelheim.
**Del Prato 2011**	Not provided	2011	Multinational	Standard treatments	167	Placebo		None	5.5		Boehringer Ingelheim.
**Del Prato 2014**	NCT00856284	2014	Multinational	DPP-4 inhibitors	885	Alogliptin	25 mg daily	Metformin	24		Takeda

Study ID	Registration	Year	Country	Intervention	N	Drug	Dose	Cointervention	Follow-up*	Settings	Fundings
**Del Prato 2014**	NCT00856284	2014	Multinational	DPP-4 inhibitors	880	Alogliptin	12.5 mg daily	Metformin	24		Takeda
**Del Prato 2014**	NCT00856284	2014	Multinational	DPP-4 inhibitors	1765	Alogliptin	12.5-25 mg daily	Metformin	24		Takeda
**Del Prato 2014**	NCT00856284	2014	Multinational	Sulfonylureas	874	Glipizide	5 mg daily	Metformin	24		Takeda
**DELIGHT**	NCT02547935	2018	Multinational	SGLT2 inhibitors	145	Dapagliflozin	10 mg daily	Stable glucose-lowering therapy	5.5	Chronic kidney disease	AstraZeneca
**DELIGHT**	NCT02547935	2018	Multinational	Standard treatments	148	Placebo		Stable glucose-lowering therapy	5.5		AstraZeneca
**Deng 2017**	Not provided	2017	China	DPP-4 inhibitors	36	Sitagliptin	100 mg daily	None	12		Natural Science Foundation of Xinjiang Uygur Autonomous Region
**Deng 2017**	Not provided	2017	China	Standard treatments	36	Standard care		None	12		Natural Science Foundation of Xinjiang Uygur Autonomous Region
**DERIVE**	NCT02413398	2019	Multinational	SGLT2 inhibitors	160	Dapagliflozin	10 mg daily	Stable glucose-lowering therapy	5.5	Chronic kidney disease	AstraZeneca
**DERIVE**	NCT02413398	2019	Multinational	Standard treatments	161	Placebo		Stable glucose-lowering therapy	5.5		AstraZeneca
**Derosa 2003**	Not applicable	2003	Italy	Meglitinides	62	Repaglinide	1 mg daily	None	6		Not reported
**Derosa 2003**	Not applicable	2003	Italy	Sulfonylureas	62	Glimepiride	1 mg daily	None	6		Not reported
**Derosa 2003a**	Not applicable	2003	Italy	Meglitinides	56	Repaglinide	2-4 mg daily	None	12		Not reported

Study ID	Registration	Year	Country	Intervention	N	Drug	Dose	Cointervention	Follow -up*	Settings	Fundings
**Derosa 2003a**	Not applicable	2003	Italy	Metformin	56	Metformin	1500-2500 mg daily	None	12		Not reported
**Derosa 2004**	Not applicable	2004	Italy	Metformin	83	Metformin	3000 mg daily	None	12		Not reported
**Derosa 2004**	Not applicable	2004	Italy	Sulfonylureas	81	Glimepiride	4 mg daily	None	12		Not reported
**Derosa 2005**	Not applicable	2005	Italy	Sulfonylureas	47	Glimepiride	2 mg daily	Metformin	12		Not reported
**Derosa 2005**	Not applicable	2005	Italy	Thiazolidinediones	48	Rosiglitazone	4 mg daily	Metformin	12		Not reported
**Derosa 2007**	Not provided	2007	Italy	Meglitinides	124	Nateglinide	300 mg daily	Metformin	6		Not reported
**Derosa 2007**	Not provided	2007	Italy	Sulfonylureas	124	Glibenclamide	12.5 mg daily	Metformin	6		Not reported
**Derosa 2009**	Not provided	2009	Italy	Alpha-glucosidase inhibitors	175	Acarbose	300 mg daily	Oral hypoglycaemic agents	6		Not reported
**Derosa 2009**	Not provided	2009	Italy	Thiazolidinediones	175	Pioglitazone	45 mg daily	Oral hypoglycaemic agents	6		Not reported
**Derosa 2009a_1**	Not provided	2009	Italy	Metformin	67	Metformin	3000 mg daily	None	15		Not reported
**Derosa 2009a_1**	Not provided	2009	Italy	Thiazolidinediones	69	Pioglitazone	45 mg daily	None	15		Not reported
**Derosa 2009a_2**	Not provided	2009	Italy	Sulfonylureas	66	Glimepiride	6 mg daily	Metformin	15		Not reported
**Derosa 2009a_2**	Not provided	2009	Italy	Thiazolidinediones	69	Pioglitazone	45 mg daily	Metformin	15		Not reported

Study ID	Registration	Year	Country	Intervention	N	Drug	Dose	Cointervention	Follow -up*	Settings	Fundings
**Derosa 2010**	Not provided	2010	Italy	DPP-4 inhibitors	75	Sitagliptin	100 mg daily	Pioglitazone	12		University of Pavia
**Derosa 2010**	Not provided	2010	Italy	Metformin	76	Metformin	1700 mg daily	Pioglitazone	12		University of Pavia
**Derosa 2010a**	Not provided	2010	Italy	GLP-1 receptor agonists	63	Exenatide	20 mcg daily	Metformin	12		Not reported
**Derosa 2010a**	Not provided	2010	Italy	Sulfonylureas	65	Glibenclamide	15 mg daily	Metformin	12		Not reported
**Derosa 2011**	Not provided	2011	Italy	GLP-1 receptor agonists	57	Exenatide	20 mcg daily	Metformin	12		Not reported
**Derosa 2011**	Not provided	2011	Italy	Sulfonylureas	54	Glimepiride	6 mg daily	Metformin	12		Not reported
**Derosa 2012**	Not provided	2012	Italy	DPP-4 inhibitors	91	Sitagliptin	100 mg daily	Metformin	12		University of Pavia
**Derosa 2012**	Not provided	2012	Italy	Standard treatments	87	Placebo		Metformin	12		University of Pavia
**Derosa 2012a**	Not provided	2012	Italy	DPP-4 inhibitors	84	Vildagliptin	100 mg daily	Metformin	12		Not reported
**Derosa 2012a**	Not provided	2012	Italy	Standard treatments	83	Placebo		Metformin	12		Not reported
**Derosa 2013**	Not provided	2013	Italy	GLP-1 receptor agonists	86	Exenatide	20 mcg daily	Metformin	12		Not reported

Study ID	Registration	Year	Country	Intervention	N	Drug	Dose	Cointervention	Follow -up*	Settings	Fundings
**Derosa 2013**	Not provided	2013	Italy	Standard treatments	85	Placebo		Metformin	12		Not reported
**Derosa 2014**	Not provided	2014	Italy	DPP-4 inhibitors	86	Vildagliptin	100 mg daily	Metformin	5.5		Not reported
**Derosa 2014**	Not provided	2014	Italy	Sulfonylureas	81	Glimepiride	6 mg daily	Metformin	5.5		Not reported
**Derosa 2014a**	Not provided	2014	Italy	DPP-4 inhibitors	102	Sitagliptin	100 mg daily	Oral antidiabetic drugs	24		Not reported
**Derosa 2014a**	Not provided	2014	Italy	Standard treatments	103	Placebo		Oral antidiabetic drugs	24		Not reported
**DIA3004**	NCT01064414	2014	Multinational	SGLT2 inhibitors	89	Canagliflozin	300 mg daily	AHA monotherapy or combination therapy	12	Chronic kidney disease	Janssen Research and Development
**DIA3004**	NCT01064414	2014	Multinational	SGLT2 inhibitors	90	Canagliflozin	100 mg daily	AHA monotherapy or combination therapy	12		Janssen Research and Development
**DIA3004**	NCT01064414	2014	Multinational	SGLT2 inhibitors	179	Canagliflozin	100-300 mg daily	AHA monotherapy or combination therapy	12		Janssen Research and Development
**DIA3004**	NCT01064414	2014	Multinational	Standard treatments	90	Placebo		AHA monotherapy or combination therapy	12		Janssen Research and Development
**Distiller 2014**	Not provided	2014	South Africa	GLP-1 receptor agonists	14	Exenatide	20 mcg daily	Metformin + insulin	6		Eli Lilly/Merck Serono

Study ID	Registration	Year	Country	Intervention	N	Drug	Dose	Cointervention	Follow -up*	Settings	Fundings
**Distiller 2014**	Not provided	2014	South Africa	Standard treatments	14	Standard care		Metformin + insulin	6		Eli Lilly/Merck Serono
**DIVERSITY-CVR**	UMIN000028014	2020	Japan	DPP-4 inhibitors	170	Sitagliptin	50-100 mg daily	Metformin or no treatment	5.5		AstraZeneca Japan K.K
**DIVERSITY-CVR**	UMIN000028014	2020	Japan	SGLT2 inhibitors	170	Dapagliflozin	5-10 mg daily	Metformin or no treatment	5.5		AstraZeneca Japan K.K
**Dobs 2013**	NCT00350779	2013	Multinational	DPP-4 inhibitors	181	Sitagliptin	100 mg daily	Metformin + PPAR gamma agonist, metformin + sulfonylurea, sulfonylurea + PPAR gamma agonist	12.4		Merck Sharp & Dohme
**Dobs 2013**	NCT00350779	2013	Multinational	Standard treatments	97	Control		Metformin + PPAR gamma agonist, metformin + sulfonylurea, sulfonylurea + PPAR gamma agonist	12.4		Merck Sharp & Dohme
**Dorkhan 2009**	Not provided	2009	Sweden	Basal insulin	15	Insulin glargine		Metformin with or without sulfonylurea /meglitinide	6		Sanofi-Aventis, The Crafoord Foundation, and The Swedish Heart and Lung Association

Study ID	Registration	Year	Country	Intervention	N	Drug	Dose	Cointervention	Follow -up*	Settings	Fundings
**Dorkhan 2009**	Not provided	2009	Sweden	Thiazolidinediones	15	Pioglitazone	45 mg daily	Metformin with or without sulfonylurea /meglitinide	6		Sanofi-Aventis, The Crafoord Foundation, and The Swedish Heart and Lung Association
**Douek 2005**	Not provided	2005	United Kingdom	Metformin	92	Metformin	2000 mg daily	Sulfonylurea thiazolidinedione	12		United Bristol Hospitals/NHS Executive Southwest
**Douek 2005**	Not provided	2005	United Kingdom	Standard treatments	91	Placebo		Sulfonylurea thiazolidinedione	12		United Bristol Hospitals/NHS Executive Southwest
**Drent 2002**	Not applicable	2002	The Netherlands	Alpha-glucosidase inhibitors	93	Miglitol	600 mg daily	None	5.5		Not reported
**Drent 2002**	Not applicable	2002	The Netherlands	Alpha-glucosidase inhibitors	94	Miglitol	300 mg daily	None	5.5		Not reported
**Drent 2002**	Not applicable	2002	The Netherlands	Alpha-glucosidase inhibitors	93	Miglitol	150 mg daily	None	5.5		Not reported
**Drent 2002**	Not applicable	2002	The Netherlands	Alpha-glucosidase inhibitors	92	Miglitol	75 mg daily	None	5.5		Not reported

Study ID	Registration	Year	Country	Intervention	N	Drug	Dose	Cointervention	Follow -up*	Settings	Fundings
**Drent 2002**	Not applicable	2002	The Netherlands	Alpha-glucosidase inhibitors	372	Miglitol	75-600 mg daily	None	5.5		Not reported
**Drent 2002**	Not applicable	2002	The Netherlands	Standard treatments	93	Placebo		None	5.5		Not reported
**DUAL-I**	NCT01336023	2014	Multinational	Basal insulin	414	Insulin degludec	Titrated	Metformin thiazolidinedione	6		Novo Nordisk
**DUAL-I**	NCT01336023	2014	Multinational	GLP-1 receptor agonists	415	Liraglutide	1.8 mg daily	Metformin thiazolidinedione	6		Novo Nordisk
**DUAL-I JAPAN**	NCT02607306	2019	Japan	Basal insulin	271	Insulin degludec		Oral antidiabetic drugs	12		Novo Nordisk
**DUAL-I JAPAN**	NCT02607306	2019	Japan	GLP-1 receptor agonists	273	Liraglutide	1.8 mg daily	Oral antidiabetic drugs	12		Novo Nordisk
**DUAL-IX**	NCT02773368	2019	Multinational	GLP-1 receptor agonists	210	Liraglutide		SGLT2 inhibitor	6		Novo Nordisk
**DUAL-IX**	NCT02773368	2019	Multinational	Standard treatments	210	Control		SGLT2 inhibitor	6		Novo Nordisk
**DUAL-VIII**	NCT02501161	2019	Multinational	GLP-1 receptor agonists	506	Liraglutide	1.8 mg daily	Antidiabetic treatment except DPP-4 inhibitor or glinide	24		Novo Nordisk

Study ID	Registration	Year	Country	Intervention	N	Drug	Dose	Cointervention	Follow -up*	Settings	Fundings
**DUAL-VIII**	NCT02501161	2019	Multinational	Standard treatments	506	Control		Antidiabetic treatment except DPP-4 inhibitor or glinide	24		Novo Nordisk
**DURATION-2**	NCT00637273	2010	Multinational	DPP-4 inhibitors	166	Sitagliptin	100 mg daily	Metformin	6		Amylin Pharmaceuticals/Eli Lilly
**DURATION-2**	NCT00637273	2010	Multinational	GLP-1 receptor agonists	160	Exenatide	2 mg weekly	Metformin	6		Amylin Pharmaceuticals/Eli Lilly
**DURATION-2**	NCT00637273	2010	Multinational	Thiazolidinediones	165	Pioglitazone	45 mg daily	Metformin	6		Amylin Pharmaceuticals/Eli Lilly
**DURATION-3**	NCT00641056	2010	Multinational	Basal insulin	223	Insulin glargine	Titrated	Metformin sulfonylurea	6		Amylin Pharmaceuticals/Eli Lilly
**DURATION-3**	NCT00641056	2010	Multinational	GLP-1 receptor agonists	233	Exenatide	2 mg weekly	Metformin sulfonylurea	6		Amylin Pharmaceuticals/Eli Lilly
**DURATION-4**	NCT00676338	2012	Multinational	DPP-4 inhibitors	163	Sitagliptin	100 mg daily	None	6		Amylin Pharmaceuticals/Eli Lilly
**DURATION-4**	NCT00676338	2012	Multinational	GLP-1 receptor agonists	248	Exenatide	2.0 mg weekly	None	6		Amylin Pharmaceuticals/Eli Lilly

Study ID	Registration	Year	Country	Intervention	N	Drug	Dose	Cointervention	Follow -up*	Settings	Fundings
**DURATION-4**	NCT00676338	2012	Multinational	Metformin	246	Metformin	2000 mg daily	None	6		Amylin Pharmaceuticals/Eli Lilly
**DURATION-4**	NCT00676338	2012	Multinational	Thiazolidinediones	163	Pioglitazone	45 mg daily	None	6		Amylin Pharmaceuticals/Eli Lilly
**DURATION-7**	NCT02229383	2018	Multinational	GLP-1 receptor agonists	233	Exenatide	2.0 mg weekly	Metformin sulfonylurea	6.8		AstraZeneca
**DURATION-7**	NCT02229383	2018	Multinational	Standard treatments	231	Placebo		Metformin sulfonylurea	6.8		AstraZeneca
**DURATION-8**	NCT02229396	2020	Multinational	GLP-1 receptor agonists	230	Exenatide	2mg QW	Metformin	26.31		AstraZeneca
**DURATION-8**	NCT02229396	2020	Multinational	SGLT2 inhibitors	233	Dapagliflozin	10 mg daily	Metformin	26.31		AstraZeneca
**DURATION-NEO-2**	NCT01652729	2017	USA	DPP-4 inhibitors	122	Sitagliptin	100 mg daily	Metformin	6		AstraZeneca
**DURATION-NEO-2**	NCT01652729	2017	USA	GLP-1 receptor agonists	181	Exenatide	2 mg weekly	Metformin	6		AstraZeneca
**DURATION-NEO-2**	NCT01652729	2017	USA	Standard treatments	61	Placebo		Metformin	6		AstraZeneca
**EAGLE**	Not provided	2015	Multinational	Basal insulin	489	Insulin glargine	Titrated	Metformin sulfonylurea	5.5		Sanofi

Study ID	Registration	Year	Country	Intervention	N	Drug	Dose	Cointervention	Follow -up*	Settings	Fundings
**EAGLE**	Not provided	2015	Multinational	GLP-1 receptor agonists	489	Liraglutide	1.8 mg daily	Metformin sulfonylurea	5.5		Sanofi
**EASIE**	NCT00751114	2012	Multinational	Basal insulin	250	Insulin glargine	Titrated	Metformin	5.5		Sanofi
**EASIE**	NCT00751114	2012	Multinational	DPP-4 inhibitors	265	Sitagliptin	100 mg daily	Metformin	5.5		Sanofi
**Ebato 2009**	Not provided	2009	Japan	Standard treatments	12	Control		Insulin with or without biguanides and/or alpha glucosidase inhibitors	5.5		Novo Nordisk
**Ebato 2009**	Not provided	2009	Japan	Sulfonylureas	14	Glimepiride	3 mg daily	Insulin with or without biguanides and/or alpha glucosidase inhibitors	5.5		Novo Nordisk
**EDIT**	UMIN000004678	2015	Japan	DPP-4 inhibitors	25	Sitagliptin	50 mg daily	Insulin	5.5		Research Foundation for Community Medicine.
**EDIT**	UMIN000004678	2015	Japan	Standard treatments	25	Control		Insulin	5.5		Research Foundation for Community Medicine.
**ELAID study**	UTN 1111-1181-9567	2022	Australia	GLP-1 receptor agonists	7	Liraglutide	1.8 mg daily	None	6		Novo Nordisk

Study ID	Registration	Year	Country	Intervention	N	Drug	Dose	Cointervention	Follow -up*	Settings	Fundings
**ELAID study**	UTN 1111-1181-9567	2022	Australia	Standard treatments	9	Placebo		None	6		Novo Nordisk
**ELEGANT**	NCT01392898	2014	The Netherlands	GLP-1 receptor agonists	26	Liraglutide	1.8 mg daily	Antidiabetic medications (metformin, sulfonylurea, insulin)	12		Novo Nordisk
**ELEGANT**	NCT01392898	2014	The Netherlands	Standard treatments	24	Standard care		Antidiabetic medications (metformin, sulfonylurea, insulin)	12		Novo Nordisk
**ELITE**	NCT03208465	2021	Korea	DPP-4 inhibitors	50	Sitagliptin	100 mg once daily	Other conventional oral antidiabetic drugs, including metformin, sulfonylurea and pioglitazone	6		Boehringer Ingelheim and Eli Lilly
**ELITE**	NCT03208465	2021	Korea	SGLT2 inhibitors	50	Empagliflozin	10 mg once daily	Other conventional oral antidiabetic drugs, including metformin, sulfonylurea and pioglitazone	6	coronary artery disease	Boehringer Ingelheim and Eli Lilly

Study ID	Registration	Year	Country	Intervention	N	Drug	Dose	Cointervention	Follow-up*	Settings	Fundings
**ELIXA**	NCT01147250	2015	Multinational	GLP-1 receptor agonists	3034	Lixisenatide	10-20 mcg daily	Antidiabetic medications with the exception of other incretin therapies	25	Coronary artery disease	Sanofi
**ELIXA**	NCT01147250	2015	Multinational	Standard treatments	3034	Placebo		Antidiabetic medications with the exception of other incretin therapies	25		Sanofi
**ELLENA-IT**	UMIN000027614	2020	Japan	GLP-1 receptor agonists	30	Liraglutide	0.9 mg daily	Standard therapy	5.5		Not reported
**ELLENA-IT**	UMIN000027614	2020	Japan	SGLT2 inhibitors	31	Empagliflozin	10 mg daily	Standard therapy	5.5		Not reported
**EMBLEM**	UMIN000024502	2019	Japan	SGLT2 inhibitors	58	Empagliflozin	10 mg daily	Standard therapy	5.5	Cardiovascular disease	Boehringer Ingelheim and Eli Lilly and Company
**EMBLEM**	UMIN000024502	2019	Japan	Standard treatments	59	Placebo		Standard therapy	5.5		Boehringer Ingelheim and Eli Lilly and Company
**EMBODY**	UMIN000030158	2020	Japan	SGLT2 inhibitors	52	Empagliflozin	10 mg once daily	conventional therapy	5.5	acute myocardial infarction	Boehringer Ingelheim and Eli Lilly and Company

Study ID	Registration	Year	Country	Intervention	N	Drug	Dose	Cointervention	Follow -up*	Settings	Fundings
**EMBODY**	UMIN000030158	2020	Japan	Standard treatments	53	Placebo		conventional therapy	5.5		Boehringer Ingelheim and Eli Lilly and Company
**EMIT**	NCT01242215	2015	Japan	SGLT2 inhibitors	166	Ipragliflozin	50 mg daily	Sulphonylurea	5.5		Astellas
**EMIT**	NCT01242215	2015	Japan	Standard treatments	77	Placebo		Sulphonylurea	5.5		Astellas
**EMLIFA001**	NCT02637973	2020	Germany	SGLT2 inhibitors	42	Empagliflozin	25 mg daily	None	5.5		German Federal Ministry of Health
**EMLIFA001**	NCT02637973	2020	Germany	Standard treatments	42	Placebo		None	5.5		German Federal Ministry of Health
**EMPA-HEART**	EUDRACT Code 2016-002225-10	2022	Italy	SGLT2 inhibitors	27	Empagliflozin	10 mg daily	None	6		Boehringer Ingelheim
**EMPA-HEART**	EUDRACT Code 2016-002225-10	2022	Italy	DPP-4 inhibitors	29	Sitagliptin	100 mg daily	None	6		Boehringer Ingelheim
**EMPA-HEART CardioLink-6**	NCT02998970	2019	Canada	SGLT2 inhibitors	49	Empagliflozin	10 mg daily	Stable glucose-lowering therapy	6	Coronary artery disease	Boehringer Ingelheim
**EMPA-HEART CardioLink-6**	NCT02998970	2019	Canada	Standard treatments	48	Placebo		Stable glucose-lowering therapy	6		Boehringer Ingelheim
**EMPA-REG BASAL**	NCT01011868	2015	Multinational	SGLT2 inhibitors	155	Empagliflozin	25 mg daily	Insulin metformin sulphonylurea	18		Boehringer Ingelheim
**EMPA-REG BASAL**	NCT01011868	2015	Multinational	SGLT2 inhibitors	169	Empagliflozin	10 mg daily	Insulin metformin sulphonylurea	18		Boehringer Ingelheim

Study ID	Registration	Year	Country	Intervention	N	Drug	Dose	Cointervention	Follow-up*	Settings	Fundings
**EMPA-REG BASAL**	NCT01011868	2015	Multinational	SGLT2 inhibitors	324	Empagliflozin	10-25 mg daily	Insulin metformin sulphonylurea	18		Boehringer Ingelheim
**EMPA-REG BASAL**	NCT01011868	2015	Multinational	Standard treatments	170	Placebo		Insulin metformin sulphonylurea	18		Boehringer Ingelheim
**EMPA-REG H2H-SU**	NCT01167881	2014	Multinational	SGLT2 inhibitors	769	Empagliflozin	25 mg daily	Metformin	24		Boehringer Ingelheim/Eli Lilly
**EMPA-REG H2H-SU**	NCT01167881	2014	Multinational	Sulfonylureas	780	Glimepiride	1-4 mg daily	Metformin	24		Boehringer Ingelheim/Eli Lilly
**EMPA-REG MDI**	NCT01306214	2014	Multinational	SGLT2 inhibitors	189	Empagliflozin	25 mg daily	Insulin metformin	12		Boehringer Ingelheim/Eli Lilly
**EMPA-REG MDI**	NCT01306214	2014	Multinational	SGLT2 inhibitors	186	Empagliflozin	10 mg daily	Insulin metformin	12		Boehringer Ingelheim/Eli Lilly
**EMPA-REG MDI**	NCT01306214	2014	Multinational	SGLT2 inhibitors	375	Empagliflozin	10-25 mg daily	Insulin metformin	12		Boehringer Ingelheim/Eli Lilly
**EMPA-REG MDI**	NCT01306214	2014	Multinational	Standard treatments	188	Empagliflozin		Insulin metformin	12		Boehringer Ingelheim/Eli Lilly
**EMPA-REG MET**	NCT01159600	2014	Multinational	SGLT2 inhibitors	213	Empagliflozin	25 mg daily	Metformin	5.5		Boehringer Ingelheim/Eli Lilly
**EMPA-REG MET**	NCT01159600	2014	Multinational	SGLT2 inhibitors	217	Empagliflozin	10 mg daily	Metformin	5.5		Boehringer Ingelheim/Eli Lilly
**EMPA-REG MET**	NCT01159600	2014	Multinational	SGLT2 inhibitors	430	Empagliflozin	10-25 mg daily	Metformin	5.5		Boehringer Ingelheim/Eli Lilly
**EMPA-REG MET**	NCT01159600	2014	Multinational	Standard treatments	207	Placebo		Metformin	5.5		Boehringer Ingelheim/Eli Lilly

Study ID	Registration	Year	Country	Intervention	N	Drug	Dose	Cointervention	Follow -up*	Settings	Fundings
**EMPA-REG METSU**	NCT01159600	2013	Multinational	SGLT2 inhibitors	218	Empagliflozin	25 mg daily	Metformin + sulfonylurea	5.5		Boehringer Ingelheim
**EMPA-REG METSU**	NCT01159600	2013	Multinational	SGLT2 inhibitors	226	Empagliflozin	10 mg daily	Metformin + sulfonylurea	5.5		Boehringer Ingelheim
**EMPA-REG METSU**	NCT01159600	2013	Multinational	SGLT2 inhibitors	444	Empagliflozin	10-25 mg daily	Metformin + sulfonylurea	5.5		Boehringer Ingelheim
**EMPA-REG METSU**	NCT01159600	2013	Multinational	Standard treatments	225	Placebo		Metformin + sulfonylurea	5.5		Boehringer Ingelheim
**EMPA-REG MONO**	NCT01177813	2013	Multinational	DPP-4 inhibitors	223	Sitagliptin	100 mg daily	None	5.5		Boehringer Ingelheim/Eli Lilly
**EMPA-REG MONO**	NCT01177813	2013	Multinational	SGLT2 inhibitors	224	Empagliflozin	25 mg daily	None	5.5		Boehringer Ingelheim/Eli Lilly
**EMPA-REG MONO**	NCT01177813	2013	Multinational	SGLT2 inhibitors	224	Empagliflozin	10 mg daily	None	5.5		Boehringer Ingelheim/Eli Lilly
**EMPA-REG MONO**	NCT01177813	2013	Multinational	SGLT2 inhibitors	448	Empagliflozin	10-25 mg daily	None	5.5		Boehringer Ingelheim/Eli Lilly
**EMPA-REG MONO**	NCT01177813	2013	Multinational	Standard treatments	228	Placebo		None	5.5		Boehringer Ingelheim/Eli Lilly
**EMPA-REG OUTCOME**	NCT01131676	2015	Multinational	SGLT2 inhibitors	4687	Empagliflozin	10-25 mg daily	No or stable glucose lowering therapy	36	Cardiovascular disease	Boehringer Ingelheim/Eli Lilly
**EMPA-REG OUTCOME**	NCT01131676	2015	Multinational	Standard treatments	2333	Placebo		No or stable glucose lowering therapy	34.8		Boehringer Ingelheim/Eli Lilly

Study ID	Registration	Year	Country	Intervention	N	Drug	Dose	Cointervention	Follow -up*	Settings	Fundings
**EMPA-REG PIO**	NCT01210001	2014	Multinational	SGLT2 inhibitors	168	Empagliflozin	25 mg daily	Thiazolidinedione metformin	5.5		Boehringer Ingelheim
**EMPA-REG PIO**	NCT01210001	2014	Multinational	SGLT2 inhibitors	165	Empagliflozin	10 mg daily	Thiazolidinedione metformin	5.5		Boehringer Ingelheim
**EMPA-REG PIO**	NCT01210001	2014	Multinational	SGLT2 inhibitors	333	Empagliflozin	10-25 mg daily	Thiazolidinedione metformin	5.5		Boehringer Ingelheim
**EMPA-REG PIO**	NCT01210001	2014	Multinational	Standard treatments	165	Placebo		Thiazolidinedione metformin	5.5		Boehringer Ingelheim
**EMPA-REG RENAL - CKD2**	NCT01164501	2014	Multinational	SGLT2 inhibitors	97	Empagliflozin	25 mg daily	Antidiabetes treatment excluding SGLT-2 inhibitors	12	Chronic kidney disease	Boehringer Ingelheim/Eli Lilly
**EMPA-REG RENAL - CKD2**	NCT01164501	2014	Multinational	SGLT2 inhibitors	98	Empagliflozin	10 mg daily	Antidiabetes treatment excluding SGLT-2 inhibitors	12		Boehringer Ingelheim/Eli Lilly
**EMPA-REG RENAL - CKD2**	NCT01164501	2014	Multinational	SGLT2 inhibitors	195	Empagliflozin	10-25 mg daily	Antidiabetes treatment excluding SGLT-2 inhibitors	12		Boehringer Ingelheim/Eli Lilly
**EMPA-REG RENAL - CKD2**	NCT01164501	2014	Multinational	Standard treatments	97	Placebo		Antidiabetes treatment excluding SGLT-2 inhibitors	12		Boehringer Ingelheim/Eli Lilly
**EMPA-REG RENAL - CKD3**	NCT01164501	2014	Multinational	SGLT2 inhibitors	188	Empagliflozin	25 mg daily	Antidiabetes treatment excluding SGLT-2 inhibitors	12		Boehringer Ingelheim/Eli Lilly

Study ID	Registration	Year	Country	Intervention	N	Drug	Dose	Cointervention	Follow -up*	Settings	Fundings
**EMPA-REG RENAL - CKD3**	NCT01164501	2014	Multinational	Standard treatments	187	Placebo		Antidiabetes treatment excluding SGLT-2 inhibitors	12		Boehringer Ingelheim/Eli Lilly
**EMPA-REG RENAL - CKD4**	NCT01164501	2014	Multinational	SGLT2 inhibitors	37	Empagliflozin	25 mg daily	Antidiabetes treatment excluding SGLT-2 inhibitors	12		Boehringer Ingelheim/Eli Lilly
**EMPA-REG RENAL - CKD4**	NCT01164501	2014	Multinational	Standard treatments	37	Placebo		Antidiabetes treatment excluding SGLT-2 inhibitors	12		Boehringer Ingelheim/Eli Lilly
**Engelbrechtsen 2016**	NCT02094183	2016	Denmark	GLP-1 receptor agonists	22	Liraglutide	1.2 mg daily	None	12		Novo Nordisk
**Engelbrechtsen 2016**	NCT02094183	2016	Denmark	Standard treatments	20	Control		None	12		Novo Nordisk
**Erem 2014**	Not provided	2015	Turkey	Metformin	19	Metformin	2000 mg daily	None	12		Karadeniz Technical University
**Erem 2014**	Not provided	2015	Turkey	Sulfonylureas	19	Gliclazide	60 mg daily	None	12		Karadeniz Technical University
**Erem 2014**	Not provided	2015	Turkey	Thiazolidinediones	19	Pioglitazone	30 mg daily	None	12		Karadeniz Technical University
**ESAX-DN**	JapicCTI-173695	2020	Japan	Non-steroidal MRAs	226	Esaxerenone	1.25-2.5 mg/d	renin-angiotensin system inhibitors	12	hypertension	Daiichi Sankyo Co., Ltd
**ESAX-DN**	JapicCTI-173695	2020	Japan	Standard treatments	229	Placebo		renin-angiotensin system inhibitors	12		Daiichi Sankyo Co., Ltd

Study ID	Registration	Year	Country	Intervention	N	Drug	Dose	Cointervention	Follow-up*	Settings	Fundings
**ESPECIAL-ACS**	UMIN000007900	2017	Japan	DPP-4 inhibitors	21	Sitagliptin	50-100 mg daily	None	6	Acute coronary syndrome	Japan Kidney Foundation
**ESPECIAL-ACS**	UMIN000007900	2017	Japan	Standard treatments	20	Control		None	6		Japan Kidney Foundation
**Esposito 2004**	Not applicable	2004	Italy	Meglitinides	88	Repaglinide	12 mg daily	Oral drugs	12		Second University of Naples, the Cardiovascular Research Center, and the Regione Campania
**Esposito 2004**	Not applicable	2004	Italy	Sulfonylureas	87	Glyburide	20 mg daily	Oral drugs	12		Second University of Naples, the Cardiovascular Research Center, and the Regione Campania
**Esposito 2011**	Not provided	2011	Italy	Metformin	55	Metformin	2000 mg daily	None	5.5		Takeda Pharmaceuticals; Second University of Naples
**Esposito 2011**	Not provided	2011	Italy	Thiazolidinediones	55	Pioglitazone	45 mg daily	None	5.5		Takeda Pharmaceuticals; Second University of Naples

Study ID	Registration	Year	Country	Intervention	N	Drug	Dose	Cointervention	Follow -up*	Settings	Fundings
**Essen Study**	Not applicable	1994	Germany	Alpha-glucosidase inhibitors	28	Acarbose	300 mg daily	None	5.5		Bayer AG
**Essen Study**	Not applicable	1994	Germany	Standard treatments	30	Placebo		None	5.5		Bayer AG
**Essen Study**	Not applicable	1994	Germany	Sulfonylureas	27	Glibenclamide	10.5 mg daily	None	5.5		Bayer AG
**EUREXA**	NCT00359762	2012	Multinational	GLP-1 receptor agonists	515	Exenatide	10-20 mcg daily	Metformin	23.4		Eli Lilly
**EUREXA**	NCT00359762	2012	Multinational	Sulfonylureas	514	Glimepiride	Titrated	Metformin	26		Eli Lilly
**EUREXA extension**	NCT00359762	2015	Multinational	Sulfonylureas	77	Glimepiride	Titrated	Metformin + exenatide	24		Eli Lilly/Bristol-Myers Squibb
**EUREXA extension**	NCT00359762	2015	Multinational	Thiazolidinediones	77	Pioglitazone	45 mg daily	Metformin + exenatide	24		Eli Lilly/Bristol-Myers Squibb
**EXAMINE**	NCT00968708	2013	Multinational	DPP-4 inhibitors	2701	Alogliptin	6.25-25mg daily	Anti-diabetic therapy other than DPP-4 inhibitor or GLP-1 receptor agonist	19	Acute coronary syndrome	Takeda
**EXAMINE**	NCT00968708	2013	Multinational	Standard treatments	2679	Placebo		Anti-diabetic therapy other than DPP-4 inhibitor or GLP-1 receptor agonist	18		Takeda

Study ID	Registration	Year	Country	Intervention	N	Drug	Dose	Cointervention	Follow -up*	Settings	Fundings
**Exenatide-113**	Not applicable	2004	USA	GLP-1 receptor agonists	129	Exenatide	20 mcg daily	Sulfonylurea	7		Amylin Pharmaceuticals/Eli Lilly
**Exenatide-113**	Not applicable	2004	USA	GLP-1 receptor agonists	125	Exenatide	10 mcg daily	Sulfonylurea	7		Amylin Pharmaceuticals/Eli Lilly
**Exenatide-113**	Not applicable	2004	USA	GLP-1 receptor agonists	254	Exenatide	10-20 mcg daily	Sulfonylurea	7		Amylin Pharmaceuticals/Eli Lilly
**Exenatide-113**	Not applicable	2004	USA	Standard treatments	123	Placebo		Sulfonylurea	7		Amylin Pharmaceuticals/Eli Lilly
**EXENDA**	EudraCT 2016-000574-38	2021	Austria	GLP-1 receptor agonists	16	Exenatide	2mg weekly	Dapagliflozin	5.5		AstraZeneca
**EXENDA**	EudraCT 2016-000574-38	2021	Austria	Standard treatments	14	Placebo		Dapagliflozin	5.5		AstraZeneca
**EXEPUMP**	NCT01140893	2021	France	GLP-1 receptor agonists	28	Exenatide	5 to 10 µg BID	Insulin	6		AstraZeneca
**EXEPUMP**	NCT01140893	2021	France	Standard treatments	18	Placebo		Insulin	6		AstraZeneca
**EXSCEL**	NCT01144338	2017	Multinational	GLP-1 receptor agonists	7356	Exenatide	2 mg weekly	Up to three oral glucose-lowering agents or insulin	38.4		Amylin Pharmaceuticals

Study ID	Registration	Year	Country	Intervention	N	Drug	Dose	Cointervention	Follow -up*	Settings	Fundings
								plus two oral glucose-lowering agents			
**EXSCEL**	NCT01144338	2017	Multinational	Standard treatments	7396	Placebo		Up to three oral glucose-lowering agents or insulin plus two oral glucose-lowering agents	38.4		Amylin Pharmaceuticals
**Feng 2017**	NCT03068065	2017	China	GLP-1 receptor agonists	30	Liraglutide	1.8 mg daily	None	5.5		NGOs
**Feng 2017**	NCT03068065	2017	China	Metformin	31	Metformin	1000 mg twice daily	None	5.5		NGOs
**Feng 2017**	NCT03068065	2017	China	Sulfonylureas	32	Gliclazide	120 mg daily	None	5.5		NGOs
**Feng-2020**	IRSCTN2013L01573, IRSCTN2014L00001	2020	China	DPP-4 inhibitors	19	Saxagliptin	5 mg	conventional therapy (insulin with or without oral antidiabetic agents, except for SGLT2 inhibitors, DPP4 inhibitors and GLP1 analogues)	5.5		Jiangsu Natural Science Foundation (BK20171121), Jiangsu Planned Projects of Postdoctoral Research Funds, the Peak of Six Personnel in Jiangsu, and the

Study ID	Registration	Year	Country	Intervention	N	Drug	Dose	Cointervention	Follow-up*	Settings	Fundings
											Nanjing Medical Science and Technique Development Foundation.
**Feng-2020**	IRSCTN2013L01573, IRSCTN2014L00001	2020	China	SGLT2 inhibitors	19	Dapagliflozin	10 mg	conventional therapy (insulin with or without oral antidiabetic agents, except for SGLT2 inhibitors, DPP4 inhibitors and GLP1 analogues)	5.5		Jiangsu Natural Science Foundation (BK20171121), Jiangsu Planned Projects of Postdoctoral Research Funds, the Peak of Six Personnel in Jiangsu, and the Nanjing Medical Science and Technique Development Foundation.
**Feng-2020**	IRSCTN2013L01573, IRSCTN2014L00001	2020	China	Standard treatments	18	Placebo		conventional therapy (insulin with or without oral antidiabetic agents, except for SGLT2 inhibitors, DPP4	5.5		Jiangsu Natural Science Foundation (BK20171121), Jiangsu Planned Projects of Postdoctoral

Study ID	Registration	Year	Country	Intervention	N	Drug	Dose	Cointervention	Follow -up*	Settings	Fundings
								inhibitors and GLP1 analogues)			Research Funds, the Peak of Six Personnel in Jiangsu, and the Nanjing Medical Science and Technique Development Foundation.
**Ferdinand 2019**	NCT02182830	2019	USA	SGLT2 inhibitors	78	Empagliflozin	10-25 mg daily	None or stable oral hypoglycemic drug	5.5		Boehringer Ingelheim and Eli Lilly and Company
**Ferdinand 2019**	NCT02182830	2019	USA	Standard treatments	72	Placebo		None or stable oral hypoglycemic drug	5.5		Boehringer Ingelheim and Eli Lilly and Company
**Fernandez 2008**	Not provided	2008	USA	Standard treatments	10	Placebo		Insulin	8.4		Takeda Pharmaceuticals and American Diabetes Association
**Fernandez 2008**	Not provided	2008	USA	Thiazolidinediones	10	Pioglitazone	45 mg daily	Insulin	8.4		Takeda Pharmaceuticals and American Diabetes Association
**Ferrannini 2010**	NCT00528372	2010	Multinational	SGLT2 inhibitors	146	Dapagliflozin	10 mg daily	None	5.5		Bristol-Myers Squibb/AstraZeneca

Study ID	Registration	Year	Country	Intervention	N	Drug	Dose	Cointervention	Follow-up*	Settings	Fundings
**Ferrannini 2010**	NCT00528372	2010	Multinational	SGLT2 inhibitors	132	Dapagliflozin	5 mg daily	None	5.5		Bristol-Myers Squibb/AstraZeneca
**Ferrannini 2010**	NCT00528372	2010	Multinational	SGLT2 inhibitors	132	Dapagliflozin	2.5 mg daily	None	5.5		Bristol-Myers Squibb/AstraZeneca
**Ferrannini 2010**	NCT00528372	2010	Multinational	SGLT2 inhibitors	410	Dapagliflozin	2.5-10 mg daily	None	5.5		Bristol-Myers Squibb/AstraZeneca
**Ferrannini 2010**	NCT00528372	2010	Multinational	Standard treatments	75	Placebo		None	5.5		Bristol-Myers Squibb/AstraZeneca
**FIDELIO-DKD**	NCT02540993	2020	Multinational	Non-steroidal MRAs	2833	Finerenone	10-20 mg daily	ACEI or ARB	31.2	Chronic kidney disease	Bayer AG
**FIDELIO-DKD**	NCT02540993	2020	Multinational	Standard treatments	2841	Placebo		ACEI or ARB	31.2		Bayer AG
**FIGARO-DKD**	NCT02545049	2021	Multinational	Non-steroidal MRAs	3686	Finerenone	10-20 mg daily	ACEI or ARB	40.8	Chronic kidney disease	Bayer AG
**FIGARO-DKD**	NCT02545049	2021	Multinational	Standard treatments	3666	Placebo		ACEI or ARB	40.8		Bayer AG
**Filozof 2010**	Not provided	2010	Multinational	DPP-4 inhibitors	513	Vildagliptin	50 mg daily	Metformin	12		Novartis
**Filozof 2010**	Not provided	2010	Multinational	Sulfonylureas	494	Gliclazide	80-320 mg daily	Metformin	12		Novartis
**Finn 2009**	NCT00116792	2009	USA	Standard treatments	33	Placebo		Thiazolidinediones excluded	8		Abbott
**Finn 2009**	NCT00116792	2009	USA	Thiazolidinediones	32	Rosiglitazone	4 mg daily	Thiazolidinediones excluded	8	Coronary artery disease	Abbott

Study ID	Registration	Year	Country	Intervention	N	Drug	Dose	Cointervention	Follow -up*	Settings	Fundings
**Fischer 1998**	Not applicable	1998	Germany	Alpha-glucosidase inhibitors	98	Acarbose	75 mg daily	None	5.5		Bayer Corporation
**Fischer 1998**	Not applicable	1998	Germany	Alpha-glucosidase inhibitors	99	Acarbose	150 mg daily	None	5.5		Bayer Corporation
**Fischer 1998**	Not applicable	1998	Germany	Alpha-glucosidase inhibitors	99	Acarbose	300 mg daily	None	5.5		Bayer Corporation
**Fischer 1998**	Not applicable	1998	Germany	Alpha-glucosidase inhibitors	102	Acarbose	600 mg daily	None	5.5		Bayer Corporation
**Fischer 1998**	Not applicable	1998	Germany	Alpha-glucosidase inhibitors	398	Acarbose	75-600 mg daily	None	5.5		Bayer Corporation
**Fischer 1998**	Not applicable	1998	Germany	Standard treatments	97	Placebo		None	5.5		Bayer Corporation
**Foley 2009**	NCT00102388	2009	Multinational	DPP-4 inhibitors	546	Vildagliptin	100 mg daily	None	24		Novartis
**Foley 2009**	NCT00102388	2009	Multinational	Sulfonylureas	546	Gliclazide	80-320 mg daily	None	24		Novartis
**Foley 2011**	NCT00260156	2011	The Netherlands	DPP-4 inhibitors	29	Vildagliptin	100 mg daily	None	12		Novartis
**Foley 2011**	NCT00260156	2011	The Netherlands	Standard treatments	30	Placebo		None	12		Novartis

Study ID	Registration	Year	Country	Intervention	N	Drug	Dose	Cointervention	Follow -up*	Settings	Fundings
**Fonseca 2000**	Not applicable	2000	USA	Standard treatments	116	Placebo		Metformin	6		SmithKline Beecham
**Fonseca 2000**	Not applicable	2000	USA	Thiazolidinediones	113	Rosiglitazone	8 mg daily	Metformin	6		SmithKline Beecham
**Fonseca 2000**	Not applicable	2000	USA	Thiazolidinediones	119	Rosiglitazone	4 mg daily	Metformin	6		SmithKline Beecham
**Fonseca 2000**	Not applicable	2000	USA	Thiazolidinediones	232	Rosiglitazone	4-8 mg daily	Metformin	6		SmithKline Beecham
**Fonseca 2003**	Not applicable	2003	USA	Meglitinides	200	Nateglinide	120 mg daily	Rosiglitazone	5.5		Novartis
**Fonseca 2003**	Not applicable	2003	USA	Standard treatments	202	Placebo		Rosiglitazone	5.5		Novartis
**Fonseca 2007**	NCT00099931	2007	Multinational	DPP-4 inhibitors	144	Vildagliptin	50 mg twice daily	Insulin	5.5		Novartis
**Fonseca 2007**	NCT00099931	2007	Multinational	Standard treatments	152	Placebo		Insulin	5.5		Novartis
**Fonseca 2013**	NCT00885352	2013	Multinational	DPP-4 inhibitors	157	Sitagliptin	100 mg daily	Metformin + PPAR-gamma or sulfonylurea	6		Merck Sharp & Dohme
**Fonseca 2013**	NCT00885352	2013	Multinational	Standard treatments	156	Placebo		Metformin + PPAR-gamma or sulfonylurea	6		Merck Sharp & Dohme
**Forst 2003**	Not applicable	2003	Germany, Sweden and Switzerland	Bolus insulin	75	Insulin lispro	Titrated	Oral antidiabetic drugs	6		Eli Lilly and Company

Study ID	Registration	Year	Country	Intervention	N	Drug	Dose	Cointervention	Follow -up*	Settings	Fundings
**Forst 2003**	Not applicable	2003	Germany, Sweden and Switzerland	Sulfonylureas	68	Glibenclamide	3.5-10.5 mg daily	Oral antidiabetic drugs	6		Eli Lilly and Company
**Forst 2014**	NCT01106690	2014	Multinational	SGLT2 inhibitors	114	Canagliflozin	300 mg daily	Metformin + pioglitazone	6		Janssen Research and Development
**Forst 2014**	NCT01106690	2014	Multinational	SGLT2 inhibitors	113	Canagliflozin	100 mg daily	Metformin + pioglitazone	6		Janssen Research and Development
**Forst 2014**	NCT01106690	2014	Multinational	SGLT2 inhibitors	227	Canagliflozin	100-300 mg daily	Metformin + pioglitazone	6		Janssen Research and Development
**Forst 2014**	NCT01106690	2014	Multinational	Standard treatments	115	Placebo		Metformin + pioglitazone	6		Janssen Research and Development
**Forst 2015**	NCT01649466	2015	Germany	Basal insulin	79	NPH insulin	Titrated	Sulfonylurea	5.5		Novartis
**Forst 2015**	NCT01649466	2015	Germany	DPP-4 inhibitors	82	Vildagliptin	50 mg daily	Sulfonylurea	5.5		Novartis
**Frederich 2012**	NCT00316082	2012	Multinational	DPP-4 inhibitors	74	Saxagliptin	5 mg daily	None	5.5		Bristol-Myers Squibb/AstraZeneca
**Frederich 2012**	NCT00316082	2012	Multinational	DPP-4 inhibitors	72	Saxagliptin	5 mg daily	None	5.5		Bristol-Myers Squibb/AstraZeneca
**Frederich 2012**	NCT00316082	2012	Multinational	DPP-4 inhibitors	74	Saxagliptin	2.5 mg daily	None	5.5		Bristol-Myers Squibb/AstraZeneca
**Frederich 2012**	NCT00316082	2012	Multinational	DPP-4 inhibitors	71	Saxagliptin	2.5-5mg daily	None	5.5		Bristol-Myers Squibb/AstraZeneca
**Frederich 2012**	NCT00316082	2012	Multinational	DPP-4 inhibitors	291	Saxagliptin	2.5-5mg daily	None	5.5		Bristol-Myers Squibb/AstraZeneca

Study ID	Registration	Year	Country	Intervention	N	Drug	Dose	Cointervention	Follow -up*	Settings	Fundings
**Frederich 2012**	NCT00316082	2012	Multinational	Standard treatments	74	Placebo		None	5.5		Bristol-Myers Squibb/AstraZeneca
**FREEDOM CVO**	NCT01455896	2022	Multinational	GLP-1 receptor agonists	2075	ITCA 650	20-60 mcg daily	Standard treatment	16		Intarcia Therapeutics
**FREEDOM CVO**	NCT01455896	2022	Multinational	Standard treatments	2081	Placebo		Standard treatment	16		Intarcia Therapeutics
**FREEDOM-1**	NCT01455857	2018	USA	GLP-1 receptor agonists	153	Exenatide	60 mcg daily	Diet and exercise alone or with metformin, sulfonylureas or pioglitazone monotherapy or in combination	9.1		Intarcia Therapeutics
**FREEDOM-1**	NCT01455857	2018	USA	GLP-1 receptor agonists	153	Exenatide	40 mcg daily	Diet and exercise alone or with metformin, sulfonylureas or pioglitazone monotherapy or in combination	9.1		Intarcia Therapeutics
**FREEDOM-1**	NCT01455857	2018	USA	GLP-1 receptor agonists	306	Exenatide	40-60 mcg daily	Diet and exercise alone or with metformin, sulfonylureas or	9.1		Intarcia Therapeutics

Study ID	Registration	Year	Country	Intervention	N	Drug	Dose	Cointervention	Follow -up*	Settings	Fundings
								pioglitazone monotherapy or in combination			
**FREEDOM-1**	NCT01455857	2018	USA	Standard treatments	154	Placebo		Diet and exercise alone or with metformin, sulfonylureas or pioglitazone monotherapy or in combination	9.1		Intarcia Therapeutics
**Frias-2018**	NCT03131687	2018	Multinational	Dual GIP/GLP-1 receptor agonists	53	LY3298176	1 mg	metformin	6.9		Eli Lilly
**Frias-2018**	NCT03131687	2018	Multinational	Dual GIP/GLP-1 receptor agonists	55	LY3298176	5 mg	metformin	6.9		Eli Lilly
**Frias-2018**	NCT03131687	2018	Multinational	Dual GIP/GLP-1 receptor agonists	52	LY3298176	10 mg	metformin	6.9		Eli Lilly
**Frias-2018**	NCT03131687	2018	Multinational	Dual GIP/GLP-1	53	LY3298176	15 mg	metformin	6.9		Eli Lilly

Study ID	Registration	Year	Country	Intervention	N	Drug	Dose	Cointervention	Follow-up*	Settings	Fundings
				receptor agonists							
**Frias-2018**	NCT03131687	2018	Multinational	Dual GIP/GLP-1 receptor agonists	213	LY3298176	1-15 mg	metformin	6.9		Eli Lilly
**Frias-2018**	NCT03131687	2018	Multinational	GLP-1 receptor agonists	54	Dulaglutide	1.5 mg	metformin	6.9		Eli Lilly
**Frias-2018**	NCT03131687	2018	Multinational	Standard treatments	51	Placebo		metformin	6.9		Eli Lilly
**Gaal 2001**	Not applicable	2001	Multinational	Alpha-glucosidase inhibitors	77	Miglitol	300 mg daily	Metformin	6.4		Bayer and Sano-Synthelabo
**Gaal 2001**	Not applicable	2001	Multinational	Standard treatments	75	Placebo		Metformin	6.4		Bayer and Sano-Synthelabo
**Gallwitz 2011**	NCT00434954	2011	Germany	Basal-Bolus insulin	246	Insulin aspart 70/30	Titrated	Metformin	6		Eli Lilly
**Gallwitz 2011**	NCT00434954	2011	Germany	GLP-1 receptor agonists	248	Exenatide	10-20 mcg daily	Metformin	6		Eli Lilly
**Gallwitz 2012**	NCT00622284	2012	Multinational	DPP-4 inhibitors	777	Linagliptin	5 mg daily	Metformin	24		Boehringer Ingelheim
**Gallwitz 2012**	NCT00622284	2012	Multinational	Sulfonylureas	775	Glimepiride	1 mg daily	Metformin	24		Boehringer Ingelheim

Study ID	Registration	Year	Country	Intervention	N	Drug	Dose	Cointervention	Follow -up*	Settings	Fundings
**Gantz 2017**	NCT01703208	2017	Multinational	DPP-4 inhibitors	2100	Omarigliptin	25 mg daily	Diet and exercise alone or on monotherapy or dual combination therapy with metformin, pioglitzone, alpha glucosidase inhibitor, or SGLT2 inhibitor	22.3	Cardiovascular disease	Merck and Co Inc
**Gantz 2017**	NCT01703208	2017	Multinational	Standard treatments	2102	Placebo		Diet and exercise alone or on monotherapy or dual combination therapy with metformin, pioglitzone, alpha glucosidase inhibitor, or SGLT2 inhibitor	22.3		Merck and Co Inc
**Gantz 2017a**	NCT01814748	2017	Multinational	DPP-4 inhibitors	102	Omarigliptin	25 mg daily	Background therapy	5.5		Merck and Co Inc
**Gantz 2017a**	NCT01814748	2017	Multinational	Standard treatments	101	Placebo		Background therapy	5.5		Merck and Co Inc

Study ID	Registration	Year	Country	Intervention	N	Drug	Dose	Cointervention	Follow-up*	Settings	Fundings
**Gao-2020**	NCT02477969	2020	China	GLP-1 receptor agonists	191	PEX168	100 ug	Metformin	5.5		the Shanghai Key Clinical Center for Metabolic Disease (2017ZZ01013) and China Jiangsu Hansoh Phar-maceutical Group Co., Ltd (Hansoh Pharma)
**Gao-2020**	NCT02477969	2020	China	GLP-1 receptor agonists	192	PEX168	200 ug	Metformin	5.5		the Shanghai Key Clinical Center for Metabolic Disease (2017ZZ01013) and China Jiangsu Hansoh Phar-maceutical Group Co., Ltd (Hansoh Pharma)
**Gao-2020**	NCT02477969	2020	China	GLP-1 receptor agonists	383	PEX168	100-200 ug	Metformin	5.5		the Shanghai Key Clinical Center for Metabolic Disease (2017ZZ01013) and China Jiangsu Hansoh Phar-maceutical Group

Study ID	Registration	Year	Country	Intervention	N	Drug	Dose	Cointervention	Follow -up*	Settings	Fundings
											Co., Ltd (Hansoh Pharma)
**Gao-2020**	NCT02477969	2020	China	Standard treatments	190	Placebo		Metformin	5.5		the Shanghai Key Clinical Center for Metabolic Disease (2017ZZ01013) and China Jiangsu Hansoh Phar-maceutical Group Co., Ltd (Hansoh Pharma)
**Garber 2006**	Not provided	2006	USA	Sulfonylureas	158	Glibenclamide	5 mg daily	Metformin	5.5		Bristol-Myers Squibb
**Garber 2006**	Not provided	2006	USA	Thiazolidined iones	160	Rosiglitazone	4 mg daily	Metformin	5.5		Bristol-Myers Squibb
**Garber 2007**	NCT00099853	2007	Multinational	DPP-4 inhibitors	158	Vildagliptin	100 mg daily	Thiazolidinedione	5.5		Novartis
**Garber 2007**	NCT00099853	2007	Multinational	DPP-4 inhibitors	147	Vildagliptin	50 mg daily	Thiazolidinedione	5.5		Novartis
**Garber 2007**	NCT00099853	2007	Multinational	DPP-4 inhibitors	305	Vildagliptin	50-100 mg daily	Thiazolidinedione	5.5		Novartis
**Garber 2007**	NCT00099853	2007	Multinational	Standard treatments	158	Placebo		Thiazolidinedione	5.5		Novartis
**Garber 2008**	NCT00099944	2008	Multinational	DPP-4 inhibitors	169	Vildagliptin	100 mg daily	Sulfonylurea	5.5		Novartis

Study ID	Registration	Year	Country	Intervention	N	Drug	Dose	Cointervention	Follow -up*	Settings	Fundings
**Garber 2008**	NCT00099944	2008	Multinational	DPP-4 inhibitors	170	Vildagliptin	50 mg daily	Sulfonylurea	5.5		Novartis
**Garber 2008**	NCT00099944	2008	Multinational	DPP-4 inhibitors	339	Vildagliptin	50-100 mg daily	Sulfonylurea	5.5		Novartis
**Garber 2008**	NCT00099944	2008	Multinational	Standard treatments	176	Placebo		Sulfonylurea	5.5		Novartis
**Gastaldelli 2014**	NCT00381342	2014	USA	GLP-1 receptor agonists	29	Exenatide	10 mcg daily	None	5.5		Amylin Pharmaceuticals/Eli Lilly
**Gastaldelli 2014**	NCT00381342	2014	USA	GLP-1 receptor agonists	26	Exenatide	5 mcg daily	None	5.5		Amylin Pharmaceuticals/Eli Lilly
**Gastaldelli 2014**	NCT00381342	2014	USA	GLP-1 receptor agonists	55	Exenatide	5-10 mcg daily	None	5.5		Amylin Pharmaceuticals/Eli Lilly
**Gastaldelli 2014**	NCT00381342	2014	USA	Standard treatments	24	Placebo		None	5.5		Amylin Pharmaceuticals/Eli Lilly
**GENERATIO N**	NCT01006603	2015	Multinational	DPP-4 inhibitors	360	Saxagliptin	5 mg daily	Metformin	12		AstraZeneca and Bristol-Myers Squibb.
**GENERATIO N**	NCT01006603	2015	Multinational	Sulfonylureas	360	Glimepiride	1-6 mg daily	Metformin	12		AstraZeneca and Bristol-Myers Squibb.

Study ID	Registration	Year	Country	Intervention	N	Drug	Dose	Cointervention	Follow -up*	Settings	Fundings
**Gentile 2001**	Not applicable	2001	Italy	Alpha-glucosidase inhibitors	52	Acarbose	300 mg daily	Insulin	6.5		Italian Ministry for Scientific ad Technological Research
**Gentile 2001**	Not applicable	2001	Italy	Standard treatments	48	Placebo		Insulin	6.5		Italian Ministry for Scientific ad Technological Research
**GetGoal-Duo-1**	NCT00975286	2013	Multinational	GLP-1 receptor agonists	223	Lixisenatide	20 mcg daily	Metformin thiazolidinedione + insulin	5.5		Sanofi
**GetGoal-Duo-1**	NCT00975286	2013	Multinational	Standard treatments	223	Placebo		Metformin thiazolidinedione + insulin	5.5		Sanofi
**GetGoal-Duo-2**	NCT01768559	2016	Multinational	Bolus insulin	298	Insulin glulisine		Metformin + insulin glargine	5.5		Sanofi
**GetGoal-Duo-2**	NCT01768559	2016	Multinational	Bolus insulin	298	Insulin glulisine		Metformin + insulin glargine	5.5		Sanofi
**GetGoal-Duo-2**	NCT01768559	2016	Multinational	Bolus insulin	596	Insulin glulisine		Metformin + insulin glargine	5.5		Sanofi
**GetGoal-Duo-2**	NCT01768559	2016	Multinational	GLP-1 receptor agonists	298	Lixisenatide	20 mcg daily	Metformin + insulin glargine	5.5		Sanofi

Study ID	Registration	Year	Country	Intervention	N	Drug	Dose	Cointervention	Follow -up*	Settings	Fundings
**GetGoal-F1**	NCT00763451	2014	Multinational	GLP-1 receptor agonists	161	Lixisenatide	10-20 mcg daily	Metformin	17.5		Sanofi
**GetGoal-F1**	NCT00763451	2014	Multinational	GLP-1 receptor agonists	161	Lixisenatide	10-20 mcg daily	Metformin	17.5		Sanofi
**GetGoal-F1**	NCT00763451	2014	Multinational	GLP-1 receptor agonists	322	Lixisenatide	10-20 mcg daily	Metformin	17.5		Sanofi
**GetGoal-F1**	NCT00763451	2014	Multinational	Standard treatments	160	Placebo		Metformin	17.5		Sanofi
**GetGoal-L**	NCT00715624	2013	Multinational	GLP-1 receptor agonists	329	Lixisenatide	10-20 mcg daily	Insulin metformin	6		Sanofi
**GetGoal-L**	NCT00715624	2013	Multinational	Standard treatments	167	Placebo		Insulin metformin	6		Sanofi
**GetGoal-L Asia**	NCT00866658	2012	Multinational	GLP-1 receptor agonists	154	Lixisenatide	10-20 mcg daily	Insulin sulfonylurea	6		Sanofi
**GetGoal-L Asia**	NCT00866658	2012	Multinational	Standard treatments	157	Placebo		Insulin sulfonylurea	6		Sanofi
**GetGoal-L-C**	NCT01632163	2018	Multinational	GLP-1 receptor agonists	224	Lixisenatide	10-20 mcg daily	Insulin with or without metformin	5.5		Sanofi

Study ID	Registration	Year	Country	Intervention	N	Drug	Dose	Cointervention	Follow -up*	Settings	Fundings
**GetGoal-L-C**	NCT01632163	2018	Multinational	Standard treatments	224	Placebo		Insulin with or without metformin	5.5		Sanofi
**GetGoal-M**	NCT00712673	2013	Multinational	GLP-1 receptor agonists	255	Lixisenatide	20 mcg daily	Metformin	5.5		Sanofi
**GetGoal-M**	NCT00712673	2013	Multinational	GLP-1 receptor agonists	255	Lixisenatide	20 mcg daily	Metformin	5.5		Sanofi
**GetGoal-M**	NCT00712673	2013	Multinational	GLP-1 receptor agonists	510	Lixisenatide	20 mcg daily	Metformin	5.5		Sanofi
**GetGoal-M**	NCT00712673	2013	Multinational	Standard treatments	170	Placebo		Metformin	5.5		Sanofi
**GetGoal-M-Asia**	NCT01169779	2014	Multinational	GLP-1 receptor agonists	196	Lixisenatide	10-20 mcg daily	Metformin sulfonylurea	5.5		Sanofi
**GetGoal-M-Asia**	NCT01169779	2014	Multinational	Standard treatments	194	Placebo		Metformin sulfonylurea	5.5		Sanofi
**GetGoal-O**	NCT01798706	2017	Multinational	GLP-1 receptor agonists	176	Lixisenatide	20 mcg daily	Permitted therapies were metformin, sulfonylurea (except glibenclamide >10 mg and gliclazide >160	5.5		Sanofi

Study ID	Registration	Year	Country	Intervention	N	Drug	Dose	Cointervention	Follow -up*	Settings	Fundings
								mg), meglitininde (excet repaglinide >6 mg), pioglitazone, and basal insulin)			
**GetGoal-O**	NCT01798706	2017	Multinational	Standard treatments	174	Placebo		Permitted therapies were metformin, sulfonylurea (except glibenclamide >10 mg and gliclazide >160 mg), meglitininde (excet repaglinide >6 mg), pioglitazone, and basal insulin)	5.5		Sanofi
**GetGoal-P**	NCT00763815	2013	Multinational	GLP-1 receptor agonists	323	Lixisenatide	10-20 mcg daily	Thiazolidinedione metformin	5.5		Sanofi
**GetGoal-P**	NCT00763815	2013	Multinational	Standard treatments	161	Placebo		Thiazolidinedione metformin	5.5		Sanofi
**GetGoal-S**	NCT00713830	2014	Multinational	GLP-1 receptor agonists	574	Lixisenatide	10-20 mcg daily	Sulfonylurea metformin	5.5		Sanofi

Study ID	Registration	Year	Country	Intervention	N	Drug	Dose	Cointervention	Follow -up*	Settings	Fundings
**GetGoal-S**	NCT00713830	2014	Multinational	Standard treatments	285	Placebo		Sulfonylurea metformin	5.5		Sanofi
**Giles 2008**	NCT00521820	2008	Multinational	Sulfonylureas	256	Glyburide	10-15 mg daily	Thiazolidinedione or sulfonylurea insulin	6	Heart failure	Takeda
**Giles 2008**	NCT00521820	2008	Multinational	Thiazolidinediones	262	Pioglitazone	30-45 mg daily	Thiazolidinedione or sulfonylurea insulin	6		Takeda
**Giugliano 1993**	Not applicable	1993	Italy	Metformin	27	Metformin	1500 mg daily	Insulin	6		Not reported
**Giugliano 1993**	Not applicable	1993	Italy	Standard treatments	23	Placebo		Insulin	6		Not reported
**GLAC**	Not applicable	2004	Multinational	Sulfonylureas	109	Glibenclamide	10.5 mg daily	No therapy or monotherapy	9		Eli Lilly
**GLAC**	Not applicable	2004	Multinational	Thiazolidinediones	91	Pioglitazone	45 mg daily	No therapy or monotherapy	9		Eli Lilly
**GLAD**	Not applicable	2004	Mexico	Sulfonylureas	123	Glimepiride	8 mg daily	None	12		Eli Lilly and Company
**GLAD**	Not applicable	2004	Mexico	Thiazolidinediones	121	Pioglitazone	45 mg daily	None	12		Eli Lilly and Company
**GLAL**	Not applicable	2005	Multinational	Sulfonylureas	297	Gliclazide	320 mg daily	None	12		Takeda and Eli Lilly
**GLAL**	Not applicable	2005	Multinational	Thiazolidinediones	270	Pioglitazone	45 mg daily	None	12		Takeda and Eli Lilly

Study ID	Registration	Year	Country	Intervention	N	Drug	Dose	Cointervention	Follow -up*	Settings	Fundings
**Glimepiride Combination Group 1998**	Not applicable	1998	USA	Standard treatments	73	Placebo		None	5.5		Hoechst Marion Roussel
**Glimepiride Combination Group 1998**	Not applicable	1998	USA	Sulfonylureas	72	Glimepiride	8 mg twice daily	None	5.5		Hoechst Marion Roussel
**Göke 2002**	Not applicable	2002	Germany	Alpha-glucosidase inhibitors	136	Acarbose	300 mg daily	None	6		Takeda
**Göke 2002**	Not applicable	2002	Germany	Thiazolidinediones	129	Pioglitazone	45 mg daily	None	6		Takeda
**Göke 2010**	Not provided	2010	Multinational	DPP-4 inhibitors	428	Saxagliptin	5 mg daily	Metformin	12		Bristol-Myers Squibb/AstraZeneca
**Göke 2010**	Not provided	2010	Multinational	Sulfonylureas	430	Glipizide	5-20 mg daily	Metformin	12		Bristol-Myers Squibb/AstraZeneca
**Gomez-Perez 2002**	Not applicable	2002	Mexico	Standard treatments	34	Placebo		Metformin	5.5		GlaxoSmithKline
**Gomez-Perez 2002**	Not applicable	2002	Mexico	Thiazolidinediones	36	Rosiglitazone	8 mg daily	Metformin	5.5		GlaxoSmithKline
**Gomez-Perez 2002**	Not applicable	2002	Mexico	Thiazolidinediones	35	Rosiglitazone	4 mg daily	Metformin	5.5		GlaxoSmithKline
**Gomez-Perez 2002**	Not applicable	2002	Mexico	Thiazolidinediones	71	Rosiglitazone	4-8 mg daily	Metformin	5.5		GlaxoSmithKline
**Gomis 2011**	NCT00641043	2011	Multinational	DPP-4 inhibitors	259	Linagliptin	5 mg daily	Pioglitazone	5.5		Boehringer Ingelheim

Study ID	Registration	Year	Country	Intervention	N	Drug	Dose	Cointervention	Follow -up*	Settings	Fundings
**Gomis 2011**	NCT00641043	2011	Multinational	Standard treatments	130	Placebo		Pioglitazone	5.5		Boehringer Ingelheim
**Goodman 2009**	Not provided	2009	Multinational	DPP-4 inhibitors	123	Vildagliptin	100 mg daily	Metformin	5.5		Novartis
**Goodman 2009**	Not provided	2009	Multinational	DPP-4 inhibitors	125	Vildagliptin	100 mg daily	Metformin	5.5		Novartis
**Goodman 2009**	Not provided	2009	Multinational	DPP-4 inhibitors	248	Vildagliptin	100 mg daily	Metformin	5.5		Novartis
**Goodman 2009**	Not provided	2009	Multinational	Standard treatments	122	Placebo		Metformin	5.5		Novartis
**GRADE study**	NCT01794143	2022	Multinational	BASAL	1263	Glargine	20 U	Metformin	60		NIH
**GRADE study**	NCT01794143	2022	Multinational	SU	1254	Glimepiride	8 mg per day	Metformin	60		NIH
**GRADE study**	NCT01794143	2022	Multinational	GLP-1RA	1262	Liraglutide	1.8 mg daily	Metformin	60		NIH
**GRADE study**	NCT01794143	2022	Multinational	DPP-4-i	1268	Sitagliptin	100 mg	Metformin	60		NIH
**Grant 1996**	Not applicable	1996	UK	Metformin	27	Metformin	3000 mg daily	None	6		Lipha Pharmaceuticals
**Grant 1996**	Not applicable	1996	UK	Metformin	25	Metformin	1500 mg daily	None	6		Lipha Pharmaceuticals
**Grant 1996**	Not applicable	1996	UK	Metformin	52	Metformin	1500-3000 mg daily	None	6		Lipha Pharmaceuticals
**Grant 1996**	Not applicable	1996	UK	Standard treatments	23	Placebo		None	6		Lipha Pharmaceuticals

Study ID	Registration	Year	Country	Intervention	N	Drug	Dose	Cointervention	Follow -up*	Settings	Fundings
**GRAVITAS**	ISCTRN13643081	2019	UK	GLP-1 receptor agonists	53	Liraglutide	1.8 mg daily	Glucose lowering drugs based on guidelines	6		JP Moulton Foundation
**GRAVITAS**	ISCTRN13643081	2019	UK	Standard treatments	27	Placebo		Glucose lowering drugs based on guidelines	6		JP Moulton Foundation
**Grey 2014**	ACTRN12607000610437	2014	New Zealand	Standard treatments	43	Placebo		Insulin; thiazolidinedione excluded	12		Government
**Grey 2014**	ACTRN12607000610437	2014	New Zealand	Thiazolidinediones	43	Pioglitazone	15-30 mg daily	Insulin; thiazolidinedione excluded	12		Government
**Gudipaty 2014**	NCT00775684	2014	USA	DPP-4 inhibitors	13	Sitagliptin	100 mg daily	None	5.5		Government
**Gudipaty 2014**	NCT00775684	2014	USA	GLP-1 receptor agonists	17	Exenatide	20 mcg daily	None	5.5		Government
**Gudipaty 2014**	NCT00775684	2014	USA	Sulfonylureas	17	Glimepiride	4 mg daily	None	5.5		Government
**Guo-2020**	ChiCTR2000035091	2020	China	Basal insulin	32	Insulin glargine	10 U daily	Metformin	6	NAFLD	Natural Science Foundation of Fujian Province (2017Y0068) and 900 Hospital of the Joint Logistics Team

Study ID	Registration	Year	Country	Intervention	N	Drug	Dose	Cointervention	Follow -up*	Settings	Fundings
											Internal Hospital Project (2019L19)
**Guo-2020**	ChiCTR2000035091	2020	China	GLP-1 receptor agonists	32	Liraglutide	1.8mg weekly	Metformin	6		Natural Science Foundation of Fujian Province (2017Y0068) and 900 Hospital of the Joint Logistics Team Internal Hospital Project (2019L19)
**Guo-2020**	ChiCTR2000035091	2020	China	Standard treatments	32	Placebo		Metformin	6		Natural Science Foundation of Fujian Province (2017Y0068) and 900 Hospital of the Joint Logistics Team Internal Hospital Project (2019L19)
**Gurkan 2014**	Not provided	2014	Turkey	Basal insulin	17	Insulin glargine	Titrated	Metformin	6		None
**Gurkan 2014**	Not provided	2014	Turkey	GLP-1 receptor agonists	17	Exenatide	20 mcg daily	Metformin	6		None
**Gutniak 1987**	Not applicable	1987	Sweden	Standard treatments	10	Placebo		Insulin	12		Swedish Medical Council Grant

Study ID	Registration	Year	Country	Intervention	N	Drug	Dose	Cointervention	Follow -up*	Settings	Fundings
**Gutniak 1987**	Not applicable	1987	Sweden	Sulfonylureas	10	Glyburide	10.5 mg daily	Insulin	12		Swedish Medical Council Grant
**Guvener 1999**	Not applicable	1999	Turkey	Alpha-glucosidase inhibitors	20	Acarbose	150 mg daily	Insulin	6		None reported
**Guvener 1999**	Not applicable	1999	Turkey	Sulfonylureas	20	Gliclazide	320 mg daily	Insulin	6		None reported
**Guzman 2017**	NCT02111096	2017	Multinational	DPP-4 inhibitors	65	Sitagliptin	100 mg daily	Metformin + sulfonylurea	6		Eli Lilly
**Guzman 2017**	NCT02111096	2017	Multinational	Standard treatments	68	Placebo		Metformin + sulfonylurea	6		Eli Lilly
**Haak 2012_1**	NCT00798161	2012	Multinational	DPP-4 inhibitors	143	Linagliptin	2.5 mg daily	Metformin	6		Boehringer Ingelheim
**Haak 2012_1**	NCT00798161	2012	Multinational	DPP-4 inhibitors	143	Linagliptin	2.5 mg daily	Metformin	6		Boehringer Ingelheim
**Haak 2012_1**	NCT00798161	2012	Multinational	DPP-4 inhibitors	286	Linagliptin	2.5 mg daily	Metformin	6		Boehringer Ingelheim
**Haak 2012_1**	NCT00798161	2012	Multinational	Standard treatments	147	Standard care		Metformin	6		Boehringer Ingelheim
**Haak 2012_1**	NCT00798161	2012	Multinational	Standard treatments	144	Standard care		Metformin	6		Boehringer Ingelheim
**Haak 2012_1**	NCT00798161	2012	Multinational	Standard treatments	291	Standard care		Metformin	6		Boehringer Ingelheim
**Haak 2012_2**	NCT00798161	2012	Multinational	DPP-4 inhibitors	142	Linagliptin	5 mg	None	6		Boehringer Ingelheim

Study ID	Registration	Year	Country	Intervention	N	Drug	Dose	Cointervention	Follow -up*	Settings	Fundings
**Haak 2012_2**	NCT00798161	2012	Multinational	Standard treatments	72	Placebo		None	6		Boehringer Ingelheim
**Hadjadj 2016**	NCT01719003	2016	Multinational	Metformin	164	Metformin	1000 mg daily	None	5.5		Boehringer Ingelheim
**Hadjadj 2016**	NCT01719003	2016	Multinational	Metformin	168	Metformin	2000 mg daily	None	5.5		Boehringer Ingelheim
**Hadjadj 2016**	NCT01719003	2016	Multinational	Metformin	332	Metformin	1000-2000 mg daily	None	5.5		Boehringer Ingelheim
**Hadjadj 2016**	NCT01719003	2016	Multinational	SGLT2 inhibitors	164	Empagliflozin	10 mg daily	None	5.5		Boehringer Ingelheim
**Hadjadj 2016**	NCT01719003	2016	Multinational	SGLT2 inhibitors	169	Empagliflozin	25 mg daily	None	5.5		Boehringer Ingelheim
**Hadjadj 2016**	NCT01719003	2016	Multinational	SGLT2 inhibitors	333	Empagliflozin	10-25 mg daily	None	5.5		Boehringer Ingelheim
**Halimi 2000**	Not applicable	2000	France	Alpha-glucosidase inhibitors	74	Acarbose	300 mg daily	None	6		Not reported
**Halimi 2000**	Not applicable	2000	France	Standard treatments	78	Placebo		None	6		Not reported
**Hällsten 2002**	Not applicable	2002	Finland	Metformin	13	Metformin	1000-2000 mg daily	None	6		Academy of Finland; the Novo Nordisk Foundation; the Finnish Diabetes Research Society; GlaxoSmithKline

Study ID	Registration	Year	Country	Intervention	N	Drug	Dose	Cointervention	Follow -up*	Settings	Fundings
**Hällsten 2002**	Not applicable	2002	Finland	Standard treatments	14	Placebo		None	6		Academy of Finland; the Novo Nordisk Foundation; the Finnish Diabetes Research Society; GlaxoSmithKline
**Hällsten 2002**	Not applicable	2002	Finland	Thiazolidinediones	14	Rosiglitazone	4-8 mg daily	None	6		Academy of Finland; the Novo Nordisk Foundation; the Finnish Diabetes Research Society; GlaxoSmithKline
**Halvorsen 2019**	NCT01377844	2019	Mexico and Colombia	SGLT2 inhibitors	145	Bexagliflozin	20 mg daily	None	22.3		Theracos Sub LLC
**Halvorsen 2019**	NCT01377844	2019	Mexico and Colombia	Standard treatments	141	Placebo		None	22.3		Theracos Sub LLC
**Halvorsen 2019a**	NCT03115112	2019	Multinational	DPP-4 inhibitors	193	Sitagliptin	100 mg daily	Metformin	5.5		Theracos Sub LLC
**Halvorsen 2019a**	NCT03115112	2019	Multinational	SGLT2 inhibitors	191	Bexagliflozin	20 mg daily	Metformin	5.5		Theracos Sub LLC
**Han 2018**	NCT02452632	2018	Republic of Korea	SGLT2 inhibitors	74	Ipragliflozin	50 mg daily	Metformin + sitagliptin	5.5		Astellas
**Han 2018**	NCT02452632	2018	Republic of Korea	Standard treatments	69	Placebo		Metformin + sitagliptin	5.5		Astellas

Study ID	Registration	Year	Country	Intervention	N	Drug	Dose	Cointervention	Follow-up*	Settings	Fundings
**Han-2020**	NCT02875821	2020	Korea	SGLT2 inhibitors	30	Ipragliflozin	50 mg/day	metformin and pioglitazone	5.5		Astellas Pharma Korea, Inc.
**Han-2020**	NCT02875821	2020	Korea	Standard treatments	15	Control		metformin and pioglitazone	5.5	NAFLD	Astellas Pharma Korea, Inc.
**Han-2021**	NCT03910361	2021	Korea	DPP-4 inhibitors	25	Evogliptin	5 mg/day	None	5.5	NAFLD	DongA ST
**Han-2021**	NCT03910361	2021	Korea	Thiazolidinediones	26	Pioglitazone	15 mg/day	None	5.5		DongA ST
**Handelsman 2017**	NCT01682759	2017	Multinational	DPP-4 inhibitors	376	Omarigliptin	25 mg weekly	Metformin	12		Merck
**Handelsman 2017**	NCT01682759	2017	Multinational	Sulfonylureas	375	Glimepiride	6 mg daily	Metformin	12		Merck
**Handelsman 2019**	NCT02284893	2019	Multinational	SGLT2 inhibitors	232	Dapagliflozin	10 mg daily	Metformin plus saxagliptin	5.5		Bristol-Myers Squibb and AstraZeneca
**Handelsman 2019**	NCT02284893	2019	Multinational	Standard treatments	229	Control		Metformin plus sitagliptin	5.5		Bristol-Myers Squibb and AstraZeneca
**Haneda 2016**	JapicCTI-111507	2016	Japan	SGLT2 inhibitors	95	Luseogliflozin	2.5-5 mg daily	No therapy or 1-2 oral drugs	5.5		Taisho Pharmaceuticals
**Haneda 2016**	JapicCTI-111507	2016	Japan	Standard treatments	50	Placebo		No therapy or 1-2 oral drugs	5.5		Taisho Pharmaceuticals
**Hanefeld 1991**	Not applicable	1991	Germany	Alpha-glucosidase inhibitors	50	Acarbose	300 mg daily	None	5.5		Bayer
**Hanefeld 1991**	Not applicable	1991	Germany	Standard treatments	50	Placebo		None	5.5		Bayer

Study ID	Registration	Year	Country	Intervention	N	Drug	Dose	Cointervention	Follow-up*	Settings	Fundings
**Hanefeld 2004**	Not applicable	2004	Multinational	Metformin	320	Metformin	850-2550 mg daily	Sulfonylurea	12		Takeda/Eli Lilly
**Hanefeld 2004**	Not applicable	2004	Multinational	Thiazolidinediones	319	Pioglitazone	15-45 mg daily	Sulfonylurea	12		Takeda/Eli Lilly
**Hanefeld 2007**	Not provided	2007	Multinational	Sulfonylureas	207	Glibenclamide	15 mg daily	None	12		SmithKline Beecham
**Hanefeld 2007**	Not provided	2007	Multinational	Thiazolidinediones	191	Rosiglitazone	8 mg daily	None	12		SmithKline Beecham
**Hanefeld 2007**	Not provided	2007	Multinational	Thiazolidinediones	200	Rosiglitazone	4 mg daily	None	12		SmithKline Beecham
**Hanefeld 2007**	Not provided	2007	Multinational	Thiazolidinediones	391	Rosiglitazone	4-8 mg daily	None	12		SmithKline Beecham
**Hao-2022**	ChiCTR1800019864	2022	China	GLP-1 receptor agonist	180	Liraglutide	1.2 mg/d	None	5.5		Tianjin Fourth Central Hospital
**Hao-2022**	ChiCTR1800019864	2022	China	SGLT2 inhibitors	180	Dapagliflozin	10 mg/d	None	5.5		Tianjin Fourth Central Hospital
**HARMONY 1**	NCT00849056	2014	Multinational	GLP-1 receptor agonists	155	Albiglutide	30 mg weekly	Thiazolidinedione metformin	12		GlaxoSmithKline
**HARMONY 1**	NCT00849056	2014	Multinational	Standard treatments	155	Placebo		Thiazolidinedione metformin	12		GlaxoSmithKline
**HARMONY 2**	NCT00849017	2016	Multinational	GLP-1 receptor agonists	99	Albiglutide	50 mg weekly	None	12		GlaxoSmithKline

Study ID	Registration	Year	Country	Intervention	N	Drug	Dose	Cointervention	Follow -up*	Settings	Fundings
**HARMONY 2**	NCT00849017	2016	Multinational	GLP-1 receptor agonists	101	Albiglutide	30 mg weekly	None	12		GlaxoSmithKline
**HARMONY 2**	NCT00849017	2016	Multinational	GLP-1 receptor agonists	200	Albiglutide	30-50 mg weekly	None	12		GlaxoSmithKline
**HARMONY 2**	NCT00849017	2016	Multinational	Standard treatments	101	Placebo		None	12		GlaxoSmithKline
**HARMONY 3**	NCT00838903	2014	Multinational	DPP-4 inhibitors	302	Sitagliptin	100 mg daily	Metformin	24		GlaxoSmithKline
**HARMONY 3**	NCT00838903	2014	Multinational	GLP-1 receptor agonists	302	Albiglutide	30-50 mg weekly	Metformin	24		GlaxoSmithKline
**HARMONY 3**	NCT00838903	2014	Multinational	Standard treatments	101	Placebo		Metformin	24		GlaxoSmithKline
**HARMONY 3**	NCT00838903	2014	Multinational	Sulfonylureas	307	Glimepiride	2-4 mg daily	Metformin	24		GlaxoSmithKline
**HARMONY 4**	NCT00838916	2014	Multinational	Basal insulin	241	Insulin glargine	Titrated	Metformin sulfonylurea	12		GlaxoSmithKline
**HARMONY 4**	NCT00838916	2014	Multinational	GLP-1 receptor agonists	504	Albiglutide	30 mg weekly	Metformin sulfonylurea	12		GlaxoSmithKline
**HARMONY 5**	NCT00839527	2015	Multinational	GLP-1 receptor agonists	281	Albiglutide	30 mg weekly	Metformin + sulfonylurea	12		GlaxoSmithKline

Study ID	Registration	Year	Country	Intervention	N	Drug	Dose	Cointervention	Follow -up*	Settings	Fundings
**HARMONY 5**	NCT00839527	2015	Multinational	Standard treatments	116	Placebo		Metformin + sulfonylurea	12		GlaxoSmithKline
**HARMONY 5**	NCT00839527	2015	Multinational	Thiazolidinediones	288	Pioglitazone	30 mg daily	Metformin + sulfonylurea	12		GlaxoSmithKline
**HARMONY 6**	NCT00976391	2014	Mutlinational	Bolus insulin	281	Lispro Insulin	Titrated	Insulin oral drugs	12		GlaxoSmithKline
**HARMONY 6**	NCT00976391	2014	Multinational	GLP-1 receptor agonists	285	Albiglutide	30-50 mg weekly	Insulin oral drugs	12		GlaxoSmithKline
**HARMONY OUTCOMES**	NCT02465515	2018	Mutlinational	GLP-1 receptor agonists	4731	Albuglutide	30-50 mg weekly	Standard therapy	19.2	Cardiovascular disease	GlaxoSmithKline
**HARMONY OUTCOMES**	NCT02465515	2018	Mutlinational	Standard treatments	4732	Placebo		Standard therapy	19.2		GlaxoSmithKline
**Hartemann-Heurtier 2009**	NCT00159211	2009	France	Basal insulin	13	NPH insulin	0.2 IU/kg/day	Metformin + sulfonylurea	5.5		Takeda; Assistance Publique des Hopitaux de Paris
**Hartemann-Heurtier 2009**	NCT00159211	2009	France	Thiazolidinediones	14	Pioglitazone	30 mg daily	Metformin + sulfonylurea	5.5		Takeda; Assistance Publique des Hopitaux de Paris
**Hartley 2015**	NCT01189890	2015	USA	DPP-4 inhibitors	241	Sitagliptin	50-100 mg daily	None	7		Merck & Co., Inc., Kenilworth, NJ, USA.
**Hartley 2015**	NCT01189890	2015	USA	Sulfonylureas	239	Glimepiride	Titrated	None	7		Merck & Co., Inc.,

Study ID	Registration	Year	Country	Intervention	N	Drug	Dose	Cointervention	Follow -up*	Settings	Fundings
											Kenilworth, NJ, USA.
**Hasche 1999**	Not applicable	1999	Germany	Alpha-glucosidase inhibitors	36	Acarbose	100-600 mg daily	None	24		Not reported
**Hasche 1999**	Not applicable	1999	Germany	Standard treatments	38	Placebo		None	24		Not reported
**Hattori 2017**	UMIN000004674	2017	Japan	Alpha-glucosidase inhibitors	37	Voglibose or miglitol	0.2 mg or 150 mg daily	Sulphonylurea	5.5		Not reported
**Hattori 2017**	UMIN000004674	2017	Japan	DPP-4 inhibitors	37	Sitagliptin	50 mg daily	Sulphonylurea	5.5		Not reported
**Hattori 2018**	UMIN000021552	2018	Japan	SGLT2 inhibitors	51	Empagliflozin	10 mg daily	Medical treatment other than SGLT2 inhibitors	12		None
**Hattori 2018**	UMIN000021552	2018	Japan	Standard treatments	51	Placebo		Medical treatment other than SGLT2 inhibitors	12		None
**Hattori-2021**	UMIN000038459	2021	Japan	DPP-4 inhibitors	30	Sitagliptin	50 mg	oral glucose-lowering drugs (sulfonylureas, metformin, or pioglitazone)	120		None
**Hattori-2021**	UMIN000038459	2021	Japan	Standard treatments	22	Placebo		oral glucose-lowering drugs	120		None

Study ID	Registration	Year	Country	Intervention	N	Drug	Dose	Cointervention	Follow-up*	Settings	Fundings
								(sulfonylureas, metformin, or pioglitazone)			
**HEELA**	Not provided	2009	UK	Basal insulin	117	Insulin glargine	Titrated	2-3 oral drugs (metformin, sulfonylurea, thiazolidinedione)	6		Eli Lilly
**HEELA**	Not provided	2009	UK	GLP-1 receptor agonists	118	Exenatide	10-20 mcg daily	2-3 oral drugs (metformin, sulfonylurea, thiazolidinedione)	6		Eli Lilly
**Hegele 1995**	Not applicable	1995	Canada	Alpha-glucosidase inhibitors	70	Acarbose	Not reported	None	12		Heart and Stroke Foundation of Ontario, Miles Pharmaceuticals Canada
**Hegele 1995**	Not applicable	1995	Canada	Standard treatments	71	Placebo		None	12		Heart and Stroke Foundation of Ontario, Miles Pharmaceuticals Canada
**Heine 2005**	NCT00082381	2005	Multinational	Basal insulin	267	Insulin glargine	Titrated	Metformin + sulfonylurea	6		Eli Lilly/Amylin Pharmaceuticals

Study ID	Registration	Year	Country	Intervention	N	Drug	Dose	Cointervention	Follow-up*	Settings	Fundings
**Heine 2005**	NCT00082381	2005	Multinational	GLP-1 receptor agonists	282	Exenatide	20 mcg daily	Metformin + sulfonylurea	6		Eli Lilly/Amylin Pharmaceuticals
**Heise-2022**	NCT03951753	2022	Germany	Dual GIP/GLP-1 receptor agonists	45	Tirzepatide	15 mg weekly	Metformin	6.5		Eli Lilly
**Heise-2022**	NCT03951753	2022	Germany	GLP-1 receptor agonists	44	Semaglutide	1 mg weekly	Metformin	6.5		Eli Lilly
**Heise-2022**	NCT03951753	2022	Germany	Standard treatments	28	Placebo		Metformin	6.5		Eli Lilly
**Heliövaara 2007**	Not provided	2007	Multinational	Sulfonylureas	30	Glibenclamide	1.75-10.5 mg daily	None	12		Not reported
**Heliövaara 2007**	Not provided	2007	Multinational	Thiazolidinediones	29	Pioglitazone	30-45 mg daily	None	12		Not reported
**Henry 2012_1**	NCT00643851	2012	USA	Metformin	201	Metformin	500-2000 mg daily	None	5.5		Bristol-Myers Squibb/AstraZeneca
**Henry 2012_1**	NCT00643851	2012	USA	SGLT2 inhibitors	203	Dapagliflozin	5 mg daily	None	5.5		Bristol-Myers Squibb/AstraZeneca
**Henry 2012_2**	NCT00859898	2012	USA	Metformin	208	Metformin	500-2000 mg daily	None	5.5		Bristol-Myers Squibb/AstraZeneca
**Henry 2012_2**	NCT00859898	2012	USA	SGLT2 inhibitors	219	Dapagliflozin	10 mg daily	None	5.5		Bristol-Myers Squibb/AstraZeneca

Study ID	Registration	Year	Country	Intervention	N	Drug	Dose	Cointervention	Follow -up*	Settings	Fundings
**Henry 2014**	NCT00722371	2014	USA	DPP-4 inhibitors	186	Sitagliptin	100 mg daily	None	12		Merck Sharp & Dohme
**Henry 2014**	NCT00722371	2014	USA	Thiazolidinediones	188	Pioglitazone	45 mg daily	None	12		Merck Sharp & Dohme
**Henry 2014**	NCT00722371	2014	USA	Thiazolidinediones	194	Pioglitazone	30 mg daily	None	12		Merck Sharp & Dohme
**Henry 2014**	NCT00722371	2014	USA	Thiazolidinediones	183	Pioglitazone	15 mg daily	None	12		Merck Sharp & Dohme
**Henry 2014**	NCT00722371	2014	USA	Thiazolidinediones	565	Pioglitazone	15-45 mg daily	None	12		Merck Sharp & Dohme
**Hermann 1999**	Not applicable	1999	Sweden	Metformin	16	Metformin	1700 mg daily	Insulin	12		Not reported
**Hermann 1999**	Not applicable	1999	Sweden	Standard treatments	19	Placebo		Insulin	12		Not reported
**Hiramatsu 2018**	Not provided	2018	Japan	DPP-4 inhibitors	49	Sitagliptin	50 mg daily	None, insulin, alphaglucosidase inhibitor, or glinide	48	Chronic kidney disease	Not reported
**Hiramatsu 2018**	Not provided	2018	Japan	DPP-4 inhibitors	45	Linagliptin	5 mg daily	None, insulin, alphaglucosidase inhibitor, or glinide	48		Not reported
**Hiramatsu 2018**	Not provided	2018	Japan	DPP-4 inhibitors	45	Sitagliptin/Linagliptin		None, insulin, alphaglucosidase inhibitor, or glinide	48		Not reported

Study ID	Registration	Year	Country	Intervention	N	Drug	Dose	Cointervention	Follow -up*	Settings	Fundings
**Hiramatsu 2018**	Not provided	2018	Japan	GLP-1 receptor agonists	90	Liraglutide	0.9 mg daily	None, insulin, alphaglucosidase inhibitor, or glinide	48		Not reported
**Hirano 2009**	Not provided	2009	Japan	Standard treatments	30	Placebo		Anti-diabetic medications (sulfonylurea; alpha-glucosidase inhibitor; biguanide; insulin)	6		Ministry of Education, Culture, Sports, Science, and Technology, and by Health and Labor Sciences Research Grants for Comprehensive Research on Aging and Health (H15-Choju-012), Tokyo, Japan
**Hirano 2009**	Not provided	2009	Japan	Thiazolidinediones	31	Pioglitazone	15-30 mg daily	Anti-diabetic medications (sulfonylurea; alpha-glucosidase inhibitor; biguanide; insulin)	6	Acute coronary syndrome	Ministry of Education, Culture, Sports, Science, and Technology, and by Health and Labor Sciences Research Grants for Comprehensive

Study ID	Registration	Year	Country	Intervention	N	Drug	Dose	Cointervention	Follow -up*	Settings	Fundings
											Research on Aging and Health (H15-Choju-012), Tokyo, Japan
**Hirano 2012**	Not provided	2012	Japan	Alpha-glucosidase inhibitors	22	Acarbose	150-300 mg daily	OADs except alpha-glucosidase inhibitor	6	Acute coronary syndrome	Ministry of Education, Culture, Sports, Science, and Technology, and<br><br>by Health and Labor Sciences Research Grants for Comprehensive Research<br><br>on Aging and Health (H15-Choju-012), Tokyo, Japan.
**Hirano 2012**	Not provided	2012	Japan	Standard treatments	22	Control		OADs except alpha-glucosidase inhibitor	6		Ministry of Education, Culture, Sports, Science, and Technology, and<br><br>by Health and Labor Sciences Research

Study ID	Registration	Year	Country	Intervention	N	Drug	Dose	Cointervention	Follow -up*	Settings	Fundings
											Grants for Comprehensive Research on Aging and Health (H15-Choju-012), Tokyo, Japan.
**Hollander 2007**	NCT00054782	2007	USA	Standard treatments	212	Placebo		Insulin	5.5		GlaxoSmithKline
**Hollander 2007**	NCT00054782	2007	USA	Thiazolidinediones	209	Rosiglitazone	4 mg daily	Insulin	5.5		GlaxoSmithKline
**Hollander 2007**	NCT00054782	2007	USA	Thiazolidinediones	209	Rosiglitazone	2 mg daily	Insulin	5.5		GlaxoSmithKline
**Hollander 2007**	NCT00054782	2007	USA	Thiazolidinediones	418	Rosiglitazone	2-4 mg daily	Insulin	5.5		GlaxoSmithKline
**HOME**	NCT00375388	2009	Netherlands	Metformin	196	Metformin	850-2550 mg daily	Insulin	51.6		Altana;/Lifesca/Merck/Sante/ Merck, Sharpe, & Dohme/Novo Nordisk
**HOME**	NCT00375388	2009	Netherlands	Standard treatments	194	Placebo		Insulin	51.6		Altana;/Lifesca/Merck/Sante/ Merck, Sharpe, & Dohme/Novo Nordisk

Study ID	Registration	Year	Country	Intervention	N	Drug	Dose	Cointervention	Follow -up*	Settings	Fundings
**Home 2018**	NCT01717313	2018	Multinational	DPP-4 inhibitors	165	Omarigliptin	25 mg weekly	None	5.5		Merck and Co
**Home 2018**	NCT01717313	2018	Multinational	Standard treatments	164	Placebo		None	5.5		Merck and Co
**Hong 2015**	Not provided	2015	Republic of Korea	Standard treatments	36	Control		Treated/untreated	9		Government
**Hong 2015**	Not provided	2015	Republic of Korea	Thiazolidinediones	36	Pioglitazone	15 mg daily	Treated/untreated	9	Coronary artery disease	Government
**Hong 2016**	Not provided	2016	Republic of Korea	DPP-4 inhibitors	99	Tenegliptin	20 mg daily	None	5.5		Handok Inc
**Hong 2016**	Not provided	2016	Republic of Korea	Standard treatments	43	Placebo		None	5.5		Handok Inc
**Horton 2000**	Not applicable	2000	USA	Meglitinides	179	Nateglinide	360 mg daily	None	5.5		Novartis
**Horton 2000**	Not applicable	2000	USA	Metformin	178	Metformin	1500 mg daily	None	5.5		Novartis
**Horton 2000**	Not applicable	2000	USA	Standard treatments	172	Placebo		None	5.5		Novartis
**Hotta 1993**	Not applicable	1993	Japan	Alpha-glucosidase inhibitors	19	Acarbose	300 mg daily	None	5.5		Not reported
**Hotta 1993**	Not applicable	1993	Japan	Standard treatments	18	Placebo		None	5.5		Not reported

Study ID	Registration	Year	Country	Intervention	N	Drug	Dose	Cointervention	Follow -up*	Settings	Fundings
**Hsieh 2011**	Not provided	2011	Taiwan	Alpha-glucosidase inhibitors	52	Miglitol	150-300 mg daily	Sulfonylurea	5.5		Standard Chem. & Pharm. Co., Ltd.
**Hsieh 2011**	Not provided	2011	Taiwan	Standard treatments	53	Placebo		Sulfonylurea	5.5		Standard Chem. & Pharm. Co., Ltd.
**Hwang 2008**	Not provided	2008	Korea	Standard treatments	47	Placebo		Glibenclamide	12		GlaxoSmithKline Korea and GlaxoSmithKline
**Hwang 2008**	Not provided	2008	Korea	Thiazolidinediones	43	Rosiglitazone	4-8 mg daily	Glibenclamide	12		GlaxoSmithKline Korea and GlaxoSmithKline
**Hygum 2020**	NCT02473809	2020	Denmark	GLP-1 receptor agonists	30	Liraglutide	1.8 mg daily	Background therapy	6		Novo Nordisk Scandinavia
**Hygum 2020**	NCT0243809	2020	Denmark	Standard treatments	30	Placebo		Background therapy	6		Novo Nordisk Scandinavia
**Iacobellis 2017**	NCT02014740	2017	USA	GLP-1 receptor agonists	54	Liraglutide	1.8 mg daily	Metformin	6		Not reported
**Iacobellis 2017**	NCT02014740	2017	USA	Standard treatments	41	Control		Metformin	6		Not reported
**Iacobellis-2020**	NCT02235298	2020	USA	SGLT2 inhibitors	50	Dapagliflozin	5-10 mg daily	Metformin	5.5	overweight and/or obesity	AstraZeneca
**Iacobellis-2020**	NCT02235298	2020	USA	Standard treatments	50	Placebo		Metformin	5.5		AstraZeneca

Study ID	Registration	Year	Country	Intervention	N	Drug	Dose	Cointervention	Follow -up*	Settings	Fundings
**IDDIA**	NCT02751398	2021	Korea	SGLT2 inhibitors	30	Dapagliflozin	10 mg once daily	None	5.5	LV diastolic dysfunction	AstraZeneca, Ministry of Health and Welfare, Republic of Korea
**IDDIA**	NCT02751398	2021	Korea	Standard treatments	30	Placebo		None	5.5		AstraZeneca, Ministry of Health and Welfare, Republic of Korea
**Ikonomidis 2020**	NCT03878706	2020	Greece	GLP-1 receptor agonists	40	Liraglutide	1.8 mg daily	Metformin + basal insulin	12	High cardiovascular risk	None
**Ikonomidis 2020**	NCT03878706	2020	Greece	SGLT2 inhibitors	40	Empagliflozin	25 mg daily	Metformin + basal insulin	12	High cardiovascular risk	None
**ILLUMINATE**	NCT01135433	2015	Japan	SGLT2 inhibitors	112	Ipragliflozin	50 mg daily	Metformin	5.5		Astellas
**ILLUMINATE**	NCT01135433	2015	Japan	Standard treatments	56	Placebo		Metformin	5.5		Astellas
**Inagaki 2012**	NCT00935532	2012	Japan	Basal insulin	212	Insulin glargine	Titrated	Metformin sulfonylurea thiazolidinedione	6		Amylin Pharmaceuticals/Eli Lilly
**Inagaki 2012**	NCT00935532	2012	Japan	GLP-1 receptor agonists	215	Exenatide	2 mg weekly	Metformin sulfonylurea thiazolidinedione	6		Amylin Pharmaceuticals/Eli Lilly

Study ID	Registration	Year	Country	Intervention	N	Drug	Dose	Cointervention	Follow -up*	Settings	Fundings
**Inagaki 2013**	NCT01204294	2013	Japan	DPP-4 inhibitors	143	Linagliptin	5 mg daily	Monotherapy with an SU, biguanide, TZD, alpha-glucosidase inhibitor, DPP-4 inhibitor or glinide	12		Boehringer Ingelheim
**Inagaki 2013**	NCT01204294	2013	Japan	Metformin	63	Metformin	500-2250 mg daily	Monotherapy with an SU, biguanide, TZD, alpha glucosidase inhibitor, DPP-4 inhibitor or glinide	12		Boehringer Ingelheim
**Inagaki 2014**	NCT01413204	2014	Japan	SGLT2 inhibitors	89	Canaglifozin	200 mg daily	None	5.5		Mitsubishi Tanabe Pharma
**Inagaki 2014**	NCT01413204	2014	Japan	SGLT2 inhibitors	90	Canaglifozin	100 mg daily	None	5.5		Mitsubishi Tanabe Pharma
**Inagaki 2014**	NCT01413204	2014	Japan	SGLT2 inhibitors	179	Canaglifozin	100-200 mg daily	None	5.5		Mitsubishi Tanabe Pharma
**Inagaki 2014**	NCT01413204	2014	Japan	Standard treatments	93	Placebo		None	5.5		Mitsubishi Tanabe Pharma
**Inagaki 2015**	NCT01632007	2015	Japan	DPP-4 inhibitors	102	Trelagliptin	100 mg daily	None	5.5		Takeda Pharmaceutical Company

Study ID	Registration	Year	Country	Intervention	N	Drug	Dose	Cointervention	Follow -up*	Settings	Fundings
**Inagaki 2015**	NCT01632007	2015	Japan	DPP-4 inhibitors	92	Alogliptin	25 mg daily	None	5.5		Takeda Pharmaceutical Company
**Inagaki 2015**	NCT01632007	2015	Japan	DPP-4 inhibitors	194	Trelagliptin or alogliptin	100 mg or 25 mg daily	None	5.5		Takeda Pharmaceutical Company
**Inagaki 2015**	NCT01632007	2015	Japan	Standard treatments	51	Placebo		None	5.5		Takeda Pharmaceutical Company
**INICOM**	NCT01787396	2016	Republic of Korea	DPP-4 inhibitors	142	Gemigliptin	50 mg daily	None	5.5		LG Life Sciences
**INICOM**	NCT01787396	2016	Republic of Korea	Metformin	150	Metformin	1000-2000 mg daily	None	5.5		LG Life Sciences
**Inoue 2019**	UMIN000018839	2018	Japan	SGLT2 inhibitors	25	Ipragliflozin	50 mg daily	Insulin therapy alone or with insulin plus oral hypoglycemic agents other than SGLT2 inhibitors	5.5		Astellas Pharma
**Inoue 2019**	UMIN000018839	2018	Japan	Standard treatments	24	Control		Insulin therapy alone or with insulin plus oral hypoglycemic agents other than SGLT2 inhibitors	5.5		Astellas Pharma

Study ID	Registration	Year	Country	Intervention	N	Drug	Dose	Cointervention	Follow -up*	Settings	Fundings
**Insulin Glargine 4014**	Not provided	2006	USA	Basal insulin	104	Insulin glargine	Titrated	Metformin + sulfonylurea	5.5		Aventis Pharmaceuticals
**Insulin Glargine 4014**	Not provided	2006	USA	Thiazolidined iones	112	Rosiglitazone	8 mg daily	Metformin + sulfonylurea	5.5		Aventis Pharmaceuticals
**INTERVAL**	NCT01257451	2013	Europe	DPP-4 inhibitors	139	Vildagliptin	50-100 mg daily	Oral drugs	5.5		Novartis
**INTERVAL**	NCT01257451	2013	Europe	Standard treatments	139	Placebo		Oral drugs	5.5		Novartis
**Ito 2011**	Not provided	2011	Japan	DPP-4 inhibitors	30	Vildagliptin	50-100 mg daily	Mitiglinide or Voglibose	5.5	Haemodialysis	Not reported
**Ito 2011**	Not provided	2011	Japan	Standard treatments	30	Conrol		Mitiglinide or Voglibose	5.5		Not reported
**Ito 2017**	UMIN000022651	2017	Japan	SGLT2 inhibitors	34	Ipragliflozin	50 mg daily	Diet and exercise therapy alone or with oral hypoglycemic agents other than SGLT2 inhibitors and thiazolidinediones and/or insulin	5.5		None reported
**Ito 2017**	UMIN000022651	2017	Japan	Thiazolidined iones	32	Pioglitazone	15-30 mg daily	Diet and exercise therapy alone or with oral hypoglycemic	5.5		None reported

Study ID	Registration	Year	Country	Intervention	N	Drug	Dose	Cointervention	Follow -up*	Settings	Fundings
								agents other than SGLT2 inhibitors and thiazolidinediones and/or insulin			
**J-BOND Study**	UMIN000030514, jRCTs051180165	2020	Japan	DPP-4 inhibitors	127	Sitagliptin, vildagliptin, alogliptin, linagliptin, teneligliptin, anagliptin, or saxagliptin		None	5.5		AstraZeneca
**J-BOND Study**	UMIN000030514, jRCTs051180165	2020	Japan	SGLT2 inhibitors	126	Dapagliflozin	5 mg/day	None	5.5		AstraZeneca
**Jabbour 2014**	NCT00984867	2014	Multinational	SGLT2 inhibitors	225	Dapagliflozin	10 mg daily	DPP-4 inhibitor metformin	11		AstraZeneca and Bristol-Myers Squibb.
**Jabbour 2014**	NCT00984867	2014	Multinational	Standard treatments	226	Placebo		DPP-4 inhibitor metformin	11		AstraZeneca and Bristol-Myers Squibb.
**Jacob 2007**	Not provided	2007	USA	Metformin	18	Metformin	2000 mg daily	Insulin	6		Takeda Pharmaceuticals North America, Eli Lilly and Company, Amylin

Study ID	Registration	Year	Country	Intervention	N	Drug	Dose	Cointervention	Follow -up*	Settings	Fundings
											Pharmaceuticals Inc., Merck & Co., Inc., GlaxoSmithKline
**Jacob 2007**	Not provided	2007	USA	Standard treatments	21	Standard care		Insulin	6		Takeda Pharmaceuticals North America, Eli Lilly and Company, Amylin Pharmaceuticals Inc., Merck & Co., Inc., GlaxoSmithKline
**Jacob 2007**	Not provided	2007	USA	Thiazolidinediones	18	Pioglitazone	30 mg daily	Insulin	6		Takeda Pharmaceuticals North America, Eli Lilly and Company, Amylin Pharmaceuticals Inc., Merck & Co., Inc., GlaxoSmithKline
**Jain 2006**	Not provided	2006	Multinational	Sulfonylureas	251	Glyburide	5-15 mg daily	None	12.9		Takeda Pharmaceuticals North America, Eli Lilly and Company, Amylin Pharmaceuticals Inc.,

Study ID	Registration	Year	Country	Intervention	N	Drug	Dose	Cointervention	Follow -up*	Settings	Fundings
											Merck & Co., Inc., GlaxoSmithKline
**Jain 2006**	Not provided	2006	Multinational	Thiazolidinediones	251	Pioglitazone	15-45 mg daily	None	12.9		Takeda Pharmaceuticals North America, Eli Lilly and Company, Amylin Pharmaceuticals Inc., Merck & Co., Inc., GlaxoSmithKline
**JEDIS-1**	Not provided	2017	Japan	Alpha-glucosidase inhibitors	12	Acarbose or miglitol	300 mg daily/225 mg daily	None	24		Japan Diabetes Foundation
**JEDIS-1**	Not provided	2017	Japan	Metformin	12	Metformin	2250 mg daily	None	24		Japan Diabetes Foundation
**JEDIS-1**	Not provided	2017	Japan	Standard treatments	13	Standard care		None	24		Japan Diabetes Foundation
**JEDIS-1**	Not provided	2017	Japan	Sulfonylureas	12	Gliclazide	120 mg daily	None	24		Japan Diabetes Foundation
**Jeon 2011**	Not provided	2011	Republic of Korea	DPP-4 inhibitors	51	Vildagliptin	100 mg daily	Metformin	7.5		Chungbuk National University
**Jeon 2011**	Not provided	2011	Republic of Korea	Sulfonylureas	51	Glimepiride	4 mg daily	Metformin	7.5		Chungbuk National University

Study ID	Registration	Year	Country	Intervention	N	Drug	Dose	Cointervention	Follow -up*	Settings	Fundings
**Jeon 2018**	Not provided	2018	Republic of Korea	Basal insulin	148	Insulin glargine		Metformin, glimepiride + DPP-4 inhibitor	5.5		Not reported
**Jeon 2018**	Not provided	2018	Republic of Korea	SGLT2 inhibitors	162	Dapagliflozin	10 mg daily	Metformin, glimepiride + DPP-4 inhibitor	5.5		Not reported
**Jerums 1987**	Not applicable	1987	Germany;Australia	Standard treatments	17	Placebo		Insulin	24		Servier Laboratories
**Jerums 1987**	Not applicable	1987	Germany;Australia	Sulfonylureas	23	Gliclazide	Not provided	Insulin	24	Chronic kidney disease	Servier Laboratories
**Ji 2014**	NCT01095653	2014	China	SGLT2 inhibitors	133	Dapagliflozin	10 mg daily	None	5.5		Bristol-Myers Squibb, Astra-Zeneca
**Ji 2014**	NCT01095653	2014	China	SGLT2 inhibitors	128	Dapagliflozin	5 mg daily	None	5.5		Bristol-Myers Squibb, Astra-Zeneca
**Ji 2014**	NCT01095653	2014	China	SGLT2 inhibitors	261	Dapagliflozin	5-10 mg daily	None	5.5		Bristol-Myers Squibb, Astra-Zeneca
**Ji 2014**	NCT01095653	2014	China	Standard treatments	132	Placebo		None	5.5		Bristol-Myers Squibb, Astra-Zeneca
**Ji 2016**	Not provided	2016	China	DPP-4 inhibitors	120	Sitagliptin	100 mg daily	None	5.5		Merck and Co Inc
**Ji 2016**	Not provided	2016	China	Metformin	124	Metformin	1700 mg daily	None	5.5		Merck and Co Inc
**Ji 2016**	Not provided	2016	China	Metformin	126	Metformin	1000 mg daily	None	5.5		Merck and Co Inc

Study ID	Registration	Year	Country	Intervention	N	Drug	Dose	Cointervention	Follow-up*	Settings	Fundings
**Ji 2016**	Not provided	2016	China	Metformin	250	Metformin	1000-1700 mg daily	None	5.5		Merck and Co Inc
**Ji 2016**	Not provided	2016	China	Standard treatments	127	Placebo		None	5.5		Merck and Co Inc
**Ji 2017**	Not provided	2017	Multinational	DPP-4 inhibitors	162	Alogliptin	25 mg daily	None	5.5		Takeda Pharmaceutical Company
**Ji 2017**	Not provided	2017	Multinational	Metformin	161	Metformin	1000 mg daily	None	5.5		Takeda Pharmaceutical Company
**Ji 2017**	Not provided	2017	Multinational	Standard treatments	161	Placebo		None	5.5		Takeda Pharmaceutical Company
**Ji-2021**	NCT02924064	2021	China	DPP-4 inhibitors	122	Teneligliptin	20 mg daily	Metformin	6		Mitsubishi Tanabe Pharma, Osaka, Japan
**Ji-2021**	NCT02924064	2021	China	Standard treatments	124	Placebo		Metformin	6		Mitsubishi Tanabe Pharma, Osaka, Japan
**Ji-2021a**	NCT02916706	2021	China	DPP-4 inhibitors	125	Teneligliptin	20 mg daily	None	6		Mitsubishi Tanabe Pharma, Osaka, Japan
**Ji-2021a**	NCT02916706	2021	China	Standard treatments	126	Placebo		None	6		Mitsubishi Tanabe Pharma, Osaka, Japan
**Jian 2018**	Not provided	2018	China	SGLT2 inhibitors	63	Dapagliflozin		Insulin	6		Not reported
**Jian 2018**	Not provided	2018	China	Standard treatments	63	Control		Insulin	6		Not reported

Study ID	Registration	Year	Country	Intervention	N	Drug	Dose	Cointervention	Follow -up*	Settings	Fundings
**Jiang-2021**	CTR20131268, CTR20150102	2021	China	SGLT2 inhibitors	19	Dapagliflozin	10 mg daily	None	5.5		Jiangsu Natural Science Foundation (BK20171121) and Nanjing Medical Science and Technique Development Foundation (YKK18230)
**Jiang-2021**	CTR20131268, CTR20150102	2021	China	Standard treatments	10	Placebo		None	5.5		Jiangsu Natural Science Foundation (BK20171121) and Nanjing Medical Science and Technique Development Foundation (YKK18230)
**Jibran 2006**	Not provided	2006	Pakistan	Meglitinides	50	Repaglinide	0.5-2 mg three times daily	None	12		Not reported
**Jibran 2006**	Not provided	2006	Pakistan	Sulfonylureas	50	Glibenclamide	5-15 mg daily	None	12		Not reported

Study ID	Registration	Year	Country	Intervention	N	Drug	Dose	Cointervention	Follow -up*	Settings	Fundings
**Johnston 1998**	Not applicable	1997	USA	Alpha-glucosidase inhibitors	131	Miglitol	150-600 mg daily	No treatment or sulfonylurea	12		Bayer Corporation
**Johnston 1998**	Not applicable	1997	USA	Standard treatments	254	Placebo		No treatment or sulfonylurea	12		Bayer Corporation
**Johnston 1998a**	Not applicable	1998	USA	Alpha-glucosidase inhibitors	224	Miglitol	150-300 mg daily	No treatment or sulfonylurea	12		Bayer Corporation
**Johnston 1998a**	Not applicable	1998	USA	Standard treatments	116	Placebo		No treatment or sulfonylurea	12		Bayer Corporation
**Johnston 1998b**	Not applicable	1998	USA	Alpha-glucosidase inhibitors	85	Miglitol	150 mg daily	None	12		Bayer Corporation
**Johnston 1998b**	Not applicable	1998	USA	Alpha-glucosidase inhibitors	95	Miglitol	75 mg daily	None	12		Bayer Corporation
**Johnston 1998b**	Not applicable	1998	USA	Alpha-glucosidase inhibitors	180	Miglitol	75-150 mg daily	None	12		Bayer Corporation
**Johnston 1998b**	Not applicable	1998	USA	Standard treatments	92	Placebo		None	12		Bayer Corporation
**Johnston 1998b**	Not applicable	1998	USA	Sulfonylureas	92	Glyburide	1.25-20 mg daily	None	12		Bayer Corporation

Study ID	Registration	Year	Country	Intervention	N	Drug	Dose	Cointervention	Follow -up*	Settings	Fundings
**Josse 2003**	Not applicable	2003	Canada	Alpha-glucosidase inhibitors	93	Acarbose	300 mg daily	None	12		Bayer Canada
**Josse 2003**	Not applicable	2003	Canada	Standard treatments	99	Placebo		None	12		Bayer Canada
**Jovanovic 2000**	Not applicable	2000	USA	Meglitinides	146	Repaglinide	4 mg daily	None	5.5		Novo Nordisk
**Jovanovic 2000**	Not applicable	2000	USA	Meglitinides	140	Repaglinide	1 mg daily	None	5.5		Novo Nordisk
**Jovanovic 2000**	Not applicable	2000	USA	Meglitinides	286	Repaglinide	1-4 mg daily	None	5.5		Novo Nordisk
**Jovanovic 2000**	Not applicable	2000	USA	Standard treatments	75	Placebo		None	5.5		Novo Nordisk
**Jovanovic 2004**	Not applicable	2004	USA	Meglitinides	61	Repaglinide	12 mg daily	None	5.5		Novo Nordisk
**Jovanovic 2004**	Not applicable	2004	USA	Thiazolidinediones	62	Pioglitazone	30 mg daily	None	5.5		Novo Nordisk
**Jung 2005**	Not applicable	2005	Korea	Metformin	15	Metformin	500 mg twice daily	Glimepiride	6		Korea Health 21 R&D Project; Ministry of Health & Welfare; Republic of Korea
**Jung 2005**	Not applicable	2005	Korea	Thiazolidinediones	15	Rosiglitazone	4 mg daily	Glimepiride	6		Korea Health 21 R&D Project; Ministry of Health & Welfare; Republic of Korea

Study ID	Registration	Year	Country	Intervention	N	Drug	Dose	Cointervention	Follow -up*	Settings	Fundings
**Juurinen 2009**	Not provided	2009	Finland	Meglitinides	40	Nateglinide	360 mg daily	Metformin + insulin	5.5		Academy of Flinland; Novartis Finland
**Juurinen 2009**	Not provided	2009	Finland	Standard treatments	41	Placebo		Metformin + insulin	5.5		Academy of Flinland; Novartis Finland
**Kadoglou 2007**	NCT00306176	2007	Greece	Standard treatments	25	Control		Metformin; Gliclazide	8		Pythagoras I project (Greek Ministry of National Education and Religious Affairs and the European Union)
**Kadoglou 2007**	NCT00306176	2007	Greece	Thiazolidined iones	25	Rosiglitazone	8 mg daily	Metformin; Gliclazide	8		Pythagoras I project (Greek Ministry of National Education and Religious Affairs and the European Union)
**Kadoglou 2008**	Not provided	2008	Greece	Standard treatments	35	Control		Metformin; Gliclazide	6		Empirikion Foundation and Herakleitos and Pythagoras I projects (Greek Ministry of National Education and Religious Affairs and the European Union)

Study ID	Registration	Year	Country	Intervention	N	Drug	Dose	Cointervention	Follow -up*	Settings	Fundings
**Kadoglou 2008**	Not provided	2008	Greece	Thiazolidinediones	35	Rosiglitazone	8 mg daily	Metformin; Gliclazide	6		Empirikion Foundation and Herakleitos and Pythagoras I projects (Greek Ministry of National Education and Religious Affairs and the European Union)
**Kadoglou 2011**	Not provided	2011	Greece	Standard treatments	70	Control		Metformin	6		European Social Fund and National Resources (EPEAEK II, PYTHAGORAS II). Nikolaos P.E. Kadoglou was awarded a grant by the Alexander S. Onassis Public Benefit Foundation
**Kadoglou 2011**	Not provided	2011	Greece	Thiazolidinediones	70	Rosiglitazone	4 mg daily	Metformin	6		European Social Fund and National Resources (EPEAEK II, PYTHAGORAS II).

Study ID	Registration	Year	Country	Intervention	N	Drug	Dose	Cointervention	Follow -up*	Settings	Fundings
											Nikolaos P.E. Kadoglou was awarded a grant by the Alexander S. Onassis Public Benefit Foundation
**Kadowaki 2011**	NCT00577824	2011	Japan	GLP-1 receptor agonists	72	Exenatide	20 mcg daily	Sulphonylurea biguanide or thiazolidinedione	5.5		Eli Lilly and Company; Amylin Pharmaceuticals
**Kadowaki 2011**	NCT00577824	2011	Japan	GLP-1 receptor agonists	72	Exenatide	10 mcg daily	Sulphonylurea biguanide or thiazolidinedione	5.5		Eli Lilly and Company; Amylin Pharmaceuticals
**Kadowaki 2011**	NCT00577824	2011	Japan	GLP-1 receptor agonists	144	Exenatide	10-20 mcg daily	Sulphonylurea biguanide or thiazolidinedione	5.5		Eli Lilly and Company; Amylin Pharmaceuticals
**Kadowaki 2011**	NCT00577824	2011	Japan	Standard treatments	35	Placebo		Sulphonylurea biguanide or thiazolidinedione	5.5		Eli Lilly and Company; Amylin Pharmaceuticals
**Kadowaki 2017**	NCT02354235	2017	Japan	SGLT2 inhibitors	70	Canaglifozin	100 mg daily	DPP-4 inhibitor	5.5		Mitsubishi Tanabe Pharma
**Kadowaki 2017**	NCT02354235	2017	Japan	Standard treatments	68	Placebo		DPP-4 inhibitor	5.5		Mitsubishi Tanabe Pharma
**Kadowaki 2018**	NCT02354222	2018	Japan	DPP-4 inhibitors	77	Tenegliptin	20 mg daily	Canagliflozin	5.5		Mitsubishi Tanabe Pharma

Study ID	Registration	Year	Country	Intervention	N	Drug	Dose	Cointervention	Follow -up*	Settings	Fundings
**Kadowaki 2018**	NCT02354222	2018	Japan	Standard treatments	77	Placebo		Canagliflozin	5.5		Mitsubishi Tanabe Pharma
**Kaku 2009**	UMIN 000001110	2009	Japan	Standard treatments	86	Control		Metformin	6.5		Takeda Pharmaceutical Company
**Kaku 2009**	UMIN 000001110	2009	Japan	Thiazolidinediones	83	Pioglitazone	15-30 mg daily	Metformin	6.5		Takeda Pharmaceutical Company
**Kaku 2009a**	UMIN000001363	2009	Japan	Standard treatments	294	Control		None	12		Takeda, Novo Nordisk, Daiichi-Sankyo, Astellas, Sanofiaventis, Novartis, Banyu, Dainippon-Sumitomo, AstraZeneca, Schering-Plough, Mochida, Shionogi, Boehringer Ingelheim, Kowa, Pfizer, GlaxoSmithKline, Lilly, Sanwa, Pfeiser, Bayer

Study ID	Registration	Year	Country	Intervention	N	Drug	Dose	Cointervention	Follow -up*	Settings	Fundings
**Kaku 2009a**	UMIN000001363	2009	Japan	Thiazolidinediones	293	Pioglitazone	15-30 mg daily	None	12	Cardiovascular disease	Takeda, Novo Nordisk, Daiichi-Sankyo, Astellas, Sanofiaventis, Novartis, Banyu, Dainippon-Sumitomo, AstraZeneca, Schering-Plough, Mochida, Shionogi, Boehringer Ingelheim, Kowa, Pfizer, GlaxoSmithKline, Lilly, Sanwa, Pfeiser, Bayer
**Kaku 2011**	NCT00393718	2011	Japan	GLP-1 receptor agonists	268	Liraglutide	0.3-0.9 mg daily	None	12		Novo Nordisk
**Kaku 2011**	NCT00393718	2011	Japan	Sulfonylureas	132	Glibenclamide	1.25-2.5 mg daily	None	12		Novo Nordisk
**Kaku 2014**	Not provided	2014	Japan	SGLT2 inhibitors	88	Dapagliflozin	10 mg daily	None	5.5		Bristol-Myers Squibb, Astra-Zeneca
**Kaku 2014**	Not provided	2014	Japan	SGLT2 inhibitors	86	Dapagliflozin	5 mg daily	None	5.5		Bristol-Myers Squibb, Astra-Zeneca

Study ID	Registration	Year	Country	Intervention	N	Drug	Dose	Cointervention	Follow -up*	Settings	Fundings
**Kaku 2014**	Not provided	2014	Japan	SGLT2 inhibitors	174	Dapagliflozin	5-10 mg daily	None	5.5		Bristol-Myers Squibb, Astra-Zeneca
**Kaku 2014**	Not provided	2014	Japan	Standard treatments	87	Placebo		None	5.5		Bristol-Myers Squibb, Astra-Zeneca
**Kaku 2017**	Not provided	2017	Japan	Metformin	152	Metformin	500 mg daily	DPP-4 inhibitor	5.5		Takeda Pharmaceutical Company
**Kaku 2017**	Not provided	2017	Japan	Metformin	151	Metformin	500 mg daily	DPP-4 inhibitor	5.5		Takeda Pharmaceutical Company
**Kaku 2017**	Not provided	2017	Japan	Metformin	303	Metformin	500 mg daily	DPP-4 inhibitor	5.5		Takeda Pharmaceutical Company
**Kaku 2017**	Not provided	2017	Japan	Standard treatments	71	Control		DPP-4 inhibitor	5.5		Takeda Pharmaceutical Company
**Kaku 2019_1**	NCT02489968	2019	Japan	DPP-4 inhibitors	107	Linagliptin	5 mg daily	Empaglifozin	5.5		Boehringer Ingelheim and Eli Lilly and Co
**Kaku 2019_1**	NCT02489968	2019	Japan	Standard treatments	108	Control		Empaglifozin	5.5		Boehringer Ingelheim and Eli Lilly and Co
**Kaku 2019_2**	NCT02489968	2019	Japan	DPP-4 inhibitors	116	Linagliptin	5 mg daily	Empaglifozin	12		Boehringer Ingelheim and Eli Lilly and Co
**Kaku 2019_2**	NCT02489968	2019	Japan	Standard treatments	116	Control		Empaglifozin	12		Boehringer Ingelheim and Eli Lilly and Co

Study ID	Registration	Year	Country	Intervention	N	Drug	Dose	Cointervention	Follow-up*	Settings	Fundings
**Kanazawa 2010**	UMIN000001997	2010	Japan	Metformin	23	Metformin	500-750 mg daily	Insulin, Sulphonylurea, Alpha-glucosidase inhibitor	12		Alumni Association of Shimane University School of Medicine; Ministry of Science, Education and Culture of Japan
**Kanazawa 2010**	UMIN000001997	2010	Japan	Thiazolidinediones	22	Pioglitazone	15-30 mg daily	Insulin, Sulphonylurea, Alpha-glucosidase inhibitor	12		Alumni Association of Shimane University School of Medicine; Ministry of Science, Education and Culture of Japan
**Kanazawa 2011**	UMIN000002099	2011	Japan	Metformin	22	Metformin	2250 mg daily	Sulphonylurea, Insulin, Alpha-glucosidase inhibitor	6		Not reported
**Kanazawa 2011**	UMIN000002099	2011	Japan	Standard treatments	22	Control		Sulphonylurea, Insulin, Alpha-glucosidase inhibitor	6		Not reported
**Kanazawa 2011**	UMIN000002099	2011	Japan	Thiazolidinediones	22	Pioglitazone	30 mg daily	Sulphonylurea, Insulin, Alpha-glucosidase inhibitor	6		Not reported

Study ID	Registration	Year	Country	Intervention	N	Drug	Dose	Cointervention	Follow -up*	Settings	Fundings
**Kang-2021**	ChiCTR-IPR-17010825	2021	China	Basal insulin	80	Insulin glargine	once a day	None	5.5		Project 2014YLC20 of the Xinqiao Hospital, Project ctstc2015shmszx120014 and ctstc2015jcsf10003 of the Chongqing Science and Technology Bureau
**Kang-2021**	ChiCTR-IPR-17010825	2021	China	GLP-1 receptor agonists	79	Exenatide	10 μg b.i.d	None	5.5	Overweight or obesity	Project 2014YLC20 of the Xinqiao Hospital, Project ctstc2015shmszx120014 and ctstc2015jcsf10003 of the Chongqing Science and Technology Bureau
**Kashyap-2020**	NCT02912455	2020	USA	SGLT2 inhibitors	11	Canagliflozin	300 mg	None	6	bariatric surgery >1 but <15 years	Janssen Scientific Affairs, LLC
**Kashyap-2020**	NCT02912455	2020	USA	Standard treatments	5	Placebo		None	6		Janssen Scientific Affairs, LLC
**Kato 2015**	UMIN000013356	2015	Japan	DPP-4 inhibitors	10	Sitagliptin	50 mg daily	None	5.5		Not reported

Study ID	Registration	Year	Country	Intervention	N	Drug	Dose	Cointervention	Follow -up*	Settings	Fundings
**Kato 2015**	UMIN000013356	2015	Japan	Sulfonylureas	10	Glimepiride	1 mg daily	None	5.5		Not reported
**Kawamori 2018**	NCT02453555	2018	Japan	SGLT2 inhibitors	182	Empagliflozin	25 mg daily	None or oral antidiabetic drug (one drug at stable dosage)	5.5		Boehringer Ingelheim
**Kawamori 2018**	NCT02453555	2018	Japan	Standard treatments	93	Placebo		None or oral antidiabetic drug (one drug at stable dosage)	5.5		Boehringer Ingelheim
**Kayano-2020**	UMIN000023834	2020	Japan	SGLT2 inhibitors	36	Dapagliflozin	5 mg/day	None	6	hypertension (grade 1 or 2) and/or a history of ischemic heart disease	AstraZeneca PLC and Ono Pharmaceutical Co., Ltd.
**Kayano-2020**	UMIN000023834	2020	Japan	Standard treatments	38	Anti-diabetic drugs		None	6		AstraZeneca PLC and Ono Pharmaceutical Co., Ltd.
**Kelly 2007**	NCT00123643	2007	USA, India, Mexico	Sulfonylureas	16	Glyburide	20 mg daily	Metformin	6		GlaxoSmithKline
**Kelly 2007**	NCT00123643	2007	USA, India, Mexico	Thiazolidinediones	20	Rosiglitazone	8 mg daily	Metformin	6		GlaxoSmithKline
**Kendall 2005**	Not applicable	2005	USA	GLP-1 receptor agonists	241	Exenatide	20 mcg daily	Metformin + sulfonylurea	6		Eli Lilly and Company; Amylin Pharmaceuticals

Study ID	Registration	Year	Country	Intervention	N	Drug	Dose	Cointervention	Follow -up*	Settings	Fundings
**Kendall 2005**	Not applicable	2005	USA	GLP-1 receptor agonists	245	Exenatide	10 mcg daily	Metformin + sulfonylurea	6		Eli Lilly and Company; Amylin Pharmaceuticals
**Kendall 2005**	Not applicable	2005	USA	GLP-1 receptor agonists	486	Exenatide	10-20 mcg daily	Metformin + sulfonylurea	6		Eli Lilly and Company; Amylin Pharmaceuticals
**Kendall 2005**	Not applicable	2005	USA	Standard treatments	247	Placebo		Metformin + sulfonylurea	6		Eli Lilly and Company; Amylin Pharmaceuticals
**Khaloo 2019**	NCT03125694	2019	Iran	DPP-4 inhibitors	125	Sitagliptin	100 mg daily	Metformin + Gliclazide	12		None
**Khaloo 2019**	NCT03125694	2019	Iran	Thiazolidinediones	125	Pioglitazone	30 mg daily	Metformin + Gliclazide	12		None
**Khan-2022**	NCT05359432	2022	Pakistan	SGLT2 inhibitors	60	Empagliflozin	10 mg daily	Metformin	5.5		Primary Care Diabetes Association, Pakistan
**Khan-2022**	NCT05359432	2022	Pakistan	DPP-4 inhibitors	60	Vildagliptin	50 mg daily	Metformin	5.5		Primary Care Diabetes Association, Pakistan
**Khanolkar 2008**	Not provided	2008	UK	Sulfonylureas	25	Gliclazide	80 mg daily	Metformin	6		Not reported
**Khanolkar 2008**	Not provided	2008	UK	Thiazolidinediones	25	Rosiglitazone	4 mg daily	Metformin	6		Not reported
**Kikuchi 2012**	NCT00297063	2012	Japan	Standard treatments	54	Placebo		None	7		GlaxoSmithKline

Study ID	Registration	Year	Country	Intervention	N	Drug	Dose	Cointervention	Follow -up*	Settings	Fundings
**Kikuchi 2012**	NCT00297063	2012	Japan	Thiazolidinediones	159	Rosiglitazone	4 mg daily	None	7		GlaxoSmithKline
**Kikuchi 2012**	NCT00297063	2012	Japan	Thiazolidinediones	159	Pioglitazone	30 mg daily	None	7		GlaxoSmithKline
**Kikuchi 2012**	NCT00297063	2012	Japan	Thiazolidinediones	318	Rosiglitazone/Pioglitazone	4 mg/30 mg daily	None	7		GlaxoSmithKline
**Kim 2014**	NCT01001611	2014	Republic of Korea	Standard treatments	58	Placebo		None	5.5		Chong Kun Dang Pharmaceutical Corp
**Kim 2014**	NCT01001611	2014	Republic of Korea	Thiazolidinediones	115	Lobeglitazone	0.5 mg daily	None	5.5		Chong Kun Dang Pharmaceutical Corp
**Kim 2019**	NCT02426294	2019	Republic of Korea	Sulfonylureas	66	Glimepiride	4 mg daily	Metformin + alogliptin	6		Takeda Pharmaceuticals
**Kim 2019**	NCT02426294	2019	Republic of Korea	Thiazolidinediones	69	Pioglitazone	30 mg daily	Metformin + alogliptin	6		Takeda Pharmaceuticals
**Kim 2020**	Not provided	2020	Republic of Korea	DPP-4 inhibitors	125	Sitagliptin	100 mg daily	Metformin	5.5		Chong Kun Dang Pharmaceutical Corp
**Kim 2020**	Not provided	2020	Republic of Korea	Thiazolidinediones	118	Lobeglitazone	0.5 mg daily	Metformin	5.5		Chong Kun Dang Pharmaceutical Corp
**Kim-2022**	NCT04470310	2022	Korea	Sulfonylureas	35	Glimepiride	2 mg daily	Metformin	5.5		Takeda Pharmaceutical Company limited, Japan and Takeda Pharmaceutical Company limited, Korea

Study ID	Registration	Year	Country	Intervention	N	Drug	Dose	Cointervention	Follow -up*	Settings	Fundings
**Kim-2022**	NCT04470310	2022	Korea	DPP-4 inhibitors	31	Alogliptin	25 mg daily	Metformin	5.5		Takeda Pharmaceutical Company limited, Japan and Takeda Pharmaceutical Company limited, Korea
**KIND-LM**	UMIN000004243	2015	Japan	GLP-1 receptor agonists	22	Liraglutide	0.9 mg daily	None	5.5		Keio University; Novo Nordisk Pharma Limited
**KIND-LM**	UMIN000004243	2015	Japan	Metformin	24	Metformin	2250 mg daily	None	5.5		Keio University; Novo Nordisk Pharma Limited
**Kinoshita 2020**	UMIN000021291	2020	Japan	SGLT2 inhibitors	32	Dapagliflozin	5 mg daily	Antidiabetic drugs	6.8		Kawasaki Medical School
**Kinoshita 2020**	UMIN000021291	2020	Japan	Sulfonylureas	33	Glimepiride	0.5-1 mg daily	Antidiabetic drugs	6.8		Kawasaki Medical School
**Kinoshita 2020**	UMIN000021291	2020	Japan	Thiazolidinediones	33	Pioglitazone	7.5-15 mg daily	Antidiabetic drugs	6.8		Kawasaki Medical School
**Kiyici 2009**	Not provided	2009	Turkey	Metformin	16	Metformin	850 mg daily	Standard care	12		Not reported
**Kiyici 2009**	Not provided	2009	Turkey	Standard treatments	15	Control		Standard care	12		Not reported
**Kiyici 2009**	Not provided	2009	Turkey	Thiazolidinediones	19	Rosiglitzone	4mg daily	Standard care	12		Not reported

Study ID	Registration	Year	Country	Intervention	N	Drug	Dose	Cointervention	Follow -up*	Settings	Fundings
**Ko 2006**	Not provided	2006	China	Basal insulin	56	Isophane insulin	Titrated	Oral antidiabetic drugs	12		Not reported
**Ko 2006**	Not provided	2006	China	Thiazolidinediones	56	Rosiglitazone	8 mg daily	Oral antidiabetic drugs	12		Not reported
**Koffert 2017**	NCT02526615	2017	Finland	Metformin	13	Metformin	2000 mg daily	None	6		Finnish Centre of Excellence in Cardiovascular and Metabolic Diseases supported by the Academy of Finland, and grants by GSK, the Finnish Medical Foundation, the Emil Aaltonen Foundation, the Finnish Cultural Foundation, the Kyllikki and Uolevi Lehikoinen foundation and the Maud Kuistila Foundation
**Koffert 2017**	NCT02526615	2017	Finland	Standard treatments	14	Placebo		None	6		Finnish Centre of Excellence in Cardiovascular and Metabolic Diseases

Study ID	Registration	Year	Country	Intervention	N	Drug	Dose	Cointervention	Follow -up*	Settings	Fundings
											supported by the Academy of Finland, and grants by GSK, the Finnish Medical Foundation, the Emil Aaltonen Foundation, the Finnish Cultural Foundation, the Kyllikki and Uolevi Lehikoinen foundation and the Maud Kuistila Foundation
**Koffert 2017**	NCT02526615	2017	Finland	Thiazolidinediones	14	Rosiglitazone	8 mg daily	None	6		Finnish Centre of Excellence in Cardiovascular and Metabolic Diseases supported by the Academy of Finland, and grants by GSK, the Finnish Medical Foundation, the Emil Aaltonen Foundation, the Finnish Cultural Foundation, the

Study ID	Registration	Year	Country	Intervention	N	Drug	Dose	Cointervention	Follow -up*	Settings	Fundings
											Kyllikki and Uolevi Lehikoinen foundation and the Maud Kuistila Foundation
**Kohan 2014**	NCT00663260	2014	Multinational	SGLT2 inhibitors	85	Dapagliflozin	10 mg daily	Antidiabetic drugs including insulin	24	Chronic kidney disease	Bristol-Myers Squibb, Astra-Zeneca
**Kohan 2014**	NCT00663260	2014	Multinational	SGLT2 inhibitors	83	Dapagliflozin	5 mg daily	Antidiabetic drugs including insulin	24		Bristol-Myers Squibb, Astra-Zeneca
**Kohan 2014**	NCT00663260	2014	Multinational	SGLT2 inhibitors	168	Dapagliflozin	5-10 mg daily	Antidiabetic drugs including insulin	24		Bristol-Myers Squibb, Astra-Zeneca
**Kohan 2014**	NCT00663260	2014	Multinational	Standard treatments	84	Placebo		Antidiabetic drugs including insulin	24		Bristol-Myers Squibb, Astra-Zeneca
**Kondo 2016**	UMIN00004791	2016	Japan	DPP-4 inhibitors	86	Sitagliptin	100 mg daily	None	12		Japanese Diabetes Foundation
**Kondo 2016**	UMIN00004791	2016	Japan	Sulfonylureas	85	Glimepiride	1 mg daily	None	12		Japanese Diabetes Foundation
**Kothny 2013**	Not provided	2013	Multinational	DPP-4 inhibitors	228	Vildagliptin	100 mg daily	Insulin metformin	6		Novartis
**Kothny 2013**	Not provided	2013	Multinational	Standard treatments	221	Placebo		Insulin metformin	6		Novartis
**Krawczyk 2005**	Not applicable	2005	Poland	Metformin	20	Metformin	1500 mg daily	Insulin	6		Not reported
**Krawczyk 2005**	Not applicable	2005	Poland	Standard treatments	20	Control		Insulin	6		Not reported

Study ID	Registration	Year	Country	Intervention	N	Drug	Dose	Cointervention	Follow -up*	Settings	Fundings
**Kudo-Fujimaki 2014**	UMIN000004010	2014	Japan	Meglitinides	20	Nateglinide	270 mg daily	Vildagliptin	5.5		Not reported
**Kudo-Fujimaki 2014**	UMIN000004010	2014	Japan	Standard treatments	20	Standard care		Vildagliptin	5.5		Not reported
**Lam 1998**	Not applicable	1998	China	Alpha-glucosidase inhibitors	44	Acarbose	300 mg daily	Metformin + sulfonylurea	5.5		Bayer China
**Lam 1998**	Not applicable	1998	China	Standard treatments	45	Placebo		Metformin + sulfonylurea	5.5		Bayer China
**Lambadiari 2018**	NCT03010683	2018	Greece	GLP-1 receptor agonists	30	Liraglutide	1.8 mg daily	None	6		None
**Lambadiari 2018**	NCT03010683	2018	Greece	Metformin	30	Metformin	2000 mg daily	None	6		None
**LANTERN**	NCT01316094	2015	Japan	SGLT2 inhibitors	119	Ipragliflozin	50 mg daily	AGI, sulphonylurea or pioglitazone	5.5	Chronic kidney disease	Astellas Pharma Inc
**LANTERN**	NCT01316094	2015	Japan	Standard treatments	46	Placebo		AGI, sulphonylurea or pioglitazone	5.5		Astellas Pharma Inc
**Lawrence 2004**	Not applicable	2004	United Kingdom	Metformin	21	Metformin	1000-3000 mg daily	Metformin, sulfonylurea or no treatment	6		Takeda Pharmaceutical
**Lawrence 2004**	Not applicable	2004	United Kingdom	Sulfonylureas	22	Gliclazide	80-160 mg daily	Metformin, sulfonylurea or no treatment	6		Takeda Pharmaceutical

Study ID	Registration	Year	Country	Intervention	N	Drug	Dose	Cointervention	Follow-up*	Settings	Fundings
**Lawrence 2004**	Not applicable	2004	United Kingdom	Thiazolidinediones	21	Pioglitazone	30-45 mg daily	Metformin, sulfonylurea or no treatment	6		Takeda Pharmaceutical
**LEAD-1**	Not provided	2009	Multinational	GLP-1 receptor agonists	234	Liraglutide	1.8 mg daily	Oral drugs	5.5		Novo Nordisk
**LEAD-1**	Not provided	2009	Multinational	GLP-1 receptor agonists	228	Liraglutide	1.2 mg daily	Oral drugs	5.5		Novo Nordisk
**LEAD-1**	Not provided	2009	Multinational	GLP-1 receptor agonists	233	Liraglutide	0.6 mg daily	Oral drugs	5.5		Novo Nordisk
**LEAD-1**	Not provided	2009	Multinational	GLP-1 receptor agonists	695	Liraglutide	0.6-1.8 mg daily	Oral drugs	5.5		Novo Nordisk
**LEAD-1**	Not provided	2009	Multinational	Standard treatments	114	Placebo		Oral drugs	5.5		Novo Nordisk
**LEAD-1**	Not provided	2009	Multinational	Thiazolidinediones	232	Rosiglitazone	4 mg daily	Oral drugs	5.5		Novo Nordisk
**LEAD-2**	NCT00318461	2009	Multinational	GLP-1 receptor agonists	242	Liraglutide	1.8 mg daily	Oral drugs	6		Novo Nordisk
**LEAD-2**	NCT00318461	2009	Multinational	GLP-1 receptor agonists	241	Liraglutide	1.2 mg daily	Oral drugs	6		Novo Nordisk

Study ID	Registration	Year	Country	Intervention	N	Drug	Dose	Cointervention	Follow -up*	Settings	Fundings
**LEAD-2**	NCT00318461	2009	Multinational	GLP-1 receptor agonists	242	Liraglutide	0.6 mg daily	Oral drugs	6		Novo Nordisk
**LEAD-2**	NCT00318461	2009	Multinational	GLP-1 receptor agonists	725	Liraglutide	0.6-1.8 mg daily	Oral drugs	6		Novo Nordisk
**LEAD-2**	NCT00318461	2009	Multinational	Standard treatments	122	Placebo		Oral drugs	6		Novo Nordisk
**LEAD-2**	NCT00318461	2009	Multinational	Sulfonylureas	244	Glimepiride	4mg daily	Oral drugs	6		Novo Nordisk
**LEAD-3**	NCT00294723	2009	Multinational	GLP-1 receptor agonists	246	Liraglutide	1.8 mg daily	Monotherapy with an SU, biguanide, TZD, alpha glucosidase inhibitor, DPP-4 inhibitor or glinide	12		Novo Nordisk A/S.
**LEAD-3**	NCT00294723	2009	Multinational	GLP-1 receptor agonists	251	Liraglutide	1.2 mg daily	Monotherapy with an SU, biguanide, TZD, alpha glucosidase inhibitor, DPP-4 inhibitor or glinide	12		Novo Nordisk A/S.
**LEAD-3**	NCT00294723	2009	Multinational	GLP-1 receptor agonists	497	Liraglutide	1.2-1.8 mg daily	Monotherapy with an SU, biguanide, TZD, alpha glucosidase	12		Novo Nordisk A/S.

Study ID	Registration	Year	Country	Intervention	N	Drug	Dose	Cointervention	Follow -up*	Settings	Fundings
								inhibitor, DPP-4 inhibitor or glinide			
**LEAD-3**	NCT00294723	2009	Multinational	Sulfonylureas	248	Glimepiride	8 mg daily	Monotherapy with an SU, biguanide, TZD, alpha glucosidase inhibitor, DPP-4 inhibitor or glinide	12		Novo Nordisk A/S.
**LEAD-4**	NCT00333151	2009	USA	GLP-1 receptor agonists	178	Liraglutide	1.8 mg daily	Metformin + rosiglitazone	6		Novo Nordisk
**LEAD-4**	NCT00333151	2009	USA	GLP-1 receptor agonists	178	Liraglutide	1.2 mg daily	Metformin + rosiglitazone	6		Novo Nordisk
**LEAD-4**	NCT00333151	2009	USA	GLP-1 receptor agonists	356	Liraglutide	1.2-1.8 mg daily	Metformin + rosiglitazone	6		Novo Nordisk
**LEAD-4**	NCT00333151	2009	USA	Standard treatments	177	Placebo		Metformin + rosiglitazone	6		Novo Nordisk
**LEAD-5**	NCT00331851	2009	Multinational	Basal insulin	234	Insulin glargine	Titrated	Metformin + glimepiride	6		Novo Nordisk
**LEAD-5**	NCT00331851	2009	Multinational	GLP-1 receptor agonists	232	Liraglutide	1.8 mg daily	Metformin + glimepiride	6		Novo Nordisk

Study ID	Registration	Year	Country	Intervention	N	Drug	Dose	Cointervention	Follow -up*	Settings	Fundings
**LEAD-5**	NCT00331851	2009	Multinational	Standard treatments	115	Placebo		Metformin + glimepiride	6		Novo Nordisk
**LEADER**	NCT01179048	2016	Multinational	GLP-1 receptor agonists	4668	Liraglutide	1.8 mg daily	One or more drugs, insulin, or combination	42	High cardiovascular risk	Novo Nordisk/NIH
**LEADER**	NCT01179048	2016	Multinational	Standard treatments	4672	Placebo		One or more drugs, insulin, or combination	42		Novo Nordisk/NIH
**Lebovitz 2001**	Not applicable	2001	USA	Standard treatments	158	Placebo		None	6		SmithKline Beecham
**Lebovitz 2001**	Not applicable	2001	USA	Thiazolidinediones	169	Rosiglitazone	8 mg daily	None	6		SmithKline Beecham
**Lebovitz 2001**	Not applicable	2001	USA	Thiazolidinediones	166	Rosiglitazone	4 mg daily	None	6		SmithKline Beecham
**Lebovitz 2001**	Not applicable	2001	USA	Thiazolidinediones	335	Rosiglitazone	4-8 mg daily	None	6		SmithKline Beecham
**Ledesma 2019**	NCT02240680	2019	Multinational	DPP-4 inhibitors	151	Linagliptin	5 mg daily	Basal insulin	5.5		Boehringer Ingelheim & Eli Lilly and Company Diabetes Alliance
**Ledesma 2019**	NCT02240680	2019	Multinational	Standard treatments	151	Placebo		Basal insulin	5.5		Boehringer Ingelheim & Eli Lilly and Company Diabetes Alliance

Study ID	Registration	Year	Country	Intervention	N	Drug	Dose	Cointervention	Follow -up*	Settings	Fundings
**Lee 2013**	Not provided	2013	Korea	Standard treatments	61	Placebo		One or more drugs, insulin, or combination	12		Korean Heath Technology R&D Project, Ministry of Health and Welfare, Republic of Korea
**Lee 2013**	Not provided	2013	Korea	Thiazolidinediones	60	Pioglitazone	15 mg daily	One or more drugs, insulin, or combination	12	Coronary artery disease	Korean Heath Technology R&D Project, Ministry of Health and Welfare, Republic of Korea
**Lee 2017**	NCT01704261	2017	Multinational	DPP-4 inhibitors	154	Omarigliptin	25 mg weekly	Metformin + glimepiride	5.5		Merck
**Lee 2017**	NCT01704261	2017	Multinational	Standard treatments	153	Placebo		Metformin + glimepiride	5.5		Merck
**Lee-2013a**	Not provied	2013	Korea	Standard treatments	25	Control		None	6		the 2011 grant from the Korean Society of Hypertension
**Lee-2013a**	Not provied	2013	Korea	Thiazolidinediones	25	Pioglitazone	15 mg	None	6	hypertension	the 2011 grant from the Korean Society of Hypertension
**Lee-2022**	NCT03959501	2022	China	SGLT2 inhibitors	30	Dapagliflozin	10 mg daily	None	5.5		Astra Zeneca
**Lee-2022**	NCT03959501	2022	China	DPP-4 inhibitors	30	Sitagliptin	100 mg daily	None	5.5		Astra Zeneca

Study ID	Registration	Year	Country	Intervention	N	Drug	Dose	Cointervention	Follow -up*	Settings	Fundings
**Leiter 2014**	NCT01098539	2014	Multinational	DPP-4 inhibitors	246	Sitagliptin	25-100 mg daily	Oral antihyperglycaemic drugs (metformin, thiazolidinedione, sulfonylurea, or combination)	12		GlaxoSmithKline
**Leiter 2014**	NCT01098539	2014	Multinational	GLP-1 receptor agonists	249	Albiglutide	30-50 mg weekly	Oral antihyperglycaemic drugs (metformin, thiazolidinedione, sulfonylurea, or combination)	12		GlaxoSmithKline
**Leiter 2014a**	NCT01042977	2014	Multinational	SGLT2 inhibitors	482	Dapagliflozin	10 mg daily	Pre-existing treatment excluding rosiglitazone	6		AstraZeneca
**Leiter 2014a**	NCT01042977	2014	Multinational	Standard treatments	483	Placebo		Pre-existing treatment excluding rosiglitazone	6		AstraZeneca
**Leonhardt 1991**	Not applicable	1991	Germany	Alpha-glucosidase inhibitors	47	Acarbose	300 mg daily	None	5.5		Bayer AG
**Leonhardt 1991**	Not applicable	1991	Germany	Standard treatments	47	Placebo		None	5.5		Bayer AG
**Lewin 2007**	Not provided	2007	USA	Metformin	146	Metformin	2000 mg daily	Sulphonylurea	5.5		Not reported

Study ID	Registration	Year	Country	Intervention	N	Drug	Dose	Cointervention	Follow -up*	Settings	Fundings
**Lewin 2007**	Not provided	2007	USA	Metformin	141	Metformin	2000 mg daily	Sulphonylurea	5.5		Not reported
**Lewin 2007**	Not provided	2007	USA	Metformin	144	Metformin	1500 mg daily	Sulphonylurea	5.5		Not reported
**Lewin 2007**	Not provided	2007	USA	Metformin	431	Metformin	1500-2000 mg daily	Sulphonylurea	5.5		Not reported
**Lewin 2007**	Not provided	2007	USA	Standard treatments	144	Standard care		Sulphonylurea	5.5		Not reported
**Lewin 2015**	NCT01422876	2015	Multinational	DPP-4 inhibitors	133	Linagliptin	5 mg daily	None	5.5		Boehringer Ingelheim Pharma
**Lewin 2015**	NCT01422876	2015	Multinational	SGLT2 inhibitors	133	Empagliflozin	25 mg daily	None	5.5		Boehringer Ingelheim Pharma
**Lewin 2015**	NCT01422876	2015	Multinational	SGLT2 inhibitors	132	Empagliflozin	10 mg daily	None	5.5		Boehringer Ingelheim Pharma
**Lewin 2015**	NCT01422876	2015	Multinational	SGLT2 inhibitors	265	Empagliflozin	10-25 mg daily	None	5.5		Boehringer Ingelheim Pharma
**Li 2014**	Not provided	2014	China	DPP-4 inhibitors	67	Vildagliptin	100 mg daily	Metformin sulfonylurea alpha-glucosidase inhibitor or thiazolidinedione	5.5		Not reported
**Li 2014**	Not provided	2014	China	DPP-4 inhibitors	68	Saxagliptin	5 mg daily	Metformin sulfonylurea alpha-glucosidase	5.5		Not reported

Study ID	Registration	Year	Country	Intervention	N	Drug	Dose	Cointervention	Follow -up*	Settings	Fundings
								inhibitor or thiazolidinedione			
**Li 2014**	Not provided	2014	China	DPP-4 inhibitors	135	Vildagliptin or saxagliptin	100 mg or 5 mg daily	Metformin sulfonylurea alpha-glucosidase inhibitor or thiazolidinedione	5.5		Not reported
**Li 2014**	Not provided	2014	China	GLP-1 receptor agonists	68	Liraglutide	1.2 mg daily	Metformin sulfonylurea alpha-glucosidase inhibitor or thiazolidinedione	5.5		Not reported
**Li 2014a**	Not provided	2014	China	Basal insulin	22	Insulin glargine	Titrated	None or oral agents	77	High cardiovascular risk	Not reported
**Li 2014a**	Not provided	2014	China	Standard treatments	20	Standard care		None or oral agents	77		Not reported
**Li 2017**	Not provided	2017	China	DPP-4 inhibitors	17	Vildagliptin	100 mg daily	Metformin	5.5		Not reported
**Li 2017**	Not provided	2017	China	Standard treatments	16	Placebo		Metformin	5.5		Not reported
**Li 2019**	Not provided	2019	China	DPP-4 inhibitors	31	Saxagliptin	5 mg daily	None	5.5		Not reported
**Li 2019**	Not provided	2019	China	Sulfonylureas	33	Glimepiride	2 mg daily	None	5.5		Not reported

Study ID	Registration	Year	Country	Intervention	N	Drug	Dose	Cointervention	Follow-up*	Settings	Fundings
**Li 2019a**	NCT01644500	2019	China	GLP-1 receptor agonists	13	Dulaglutide	0.75-1.5 mg weekly	None or single oral agent	6		Scientific and Technological Development Program of Jiangsu Province of China
**Li 2019a**	NCT01644500	2019	China	Sulfonylureas	10	Glimepiride		None or single oral agent	6		Scientific and Technological Development Program of Jiangsu Province of China
**Li 2020**	NCT02655757	2020	China	DPP-4 inhibitors	75	Sitagliptin	100 mg daily	0, 1 or 2 oral agents	18	Coronary artery disease	Chinese PLA General Hospital.
**Li 2020**	NCT02655757	2020	China	Standard treatments	74	Control		0, 1 or 2 oral agents	18		Chinese PLA General Hospital.
**LIBRA**	NCT01270789	2017	USA	GLP-1 receptor agonists	26	Liraglutide	1.8 mg daily	Metformin	11.2		Novo Nordisk
**LIBRA**	NCT01270789	2017	USA	Standard treatments	25	Placebo		Metformin	11.2		Novo Nordisk
**Lim 2017**	Not applicable	2017	Republic of Korea	Standard treatments	58	Placebo		None, sulfonylurea, metformin, DPP-4 inhibitor or alphaglucosidase inhibitor	5.5		Not reported

Study ID	Registration	Year	Country	Intervention	N	Drug	Dose	Cointervention	Follow -up*	Settings	Fundings
**Lim 2017**	Not applicable	2017	Republic of Korea	Thiazolidinediones	112	Lobeglitazone	0.5 mg daily	None, sulfonylurea, metformin, DPP-4 inhibitor or alphaglucosidase inhibitor	5.5		Not reported
**Lin 2003**	Not applicable	2003	Multinational	Alpha-glucosidase inhibitors	33	Acarbose	300 mg daily	Sulfonylurea	5.5		Bayer Asia-Pacific
**Lin 2003**	Not applicable	2003	Multinational	Standard treatments	32	Placebo		Sulfonylurea	5.5		Bayer Asia-Pacific
**Linagliptin-LTC Trial**	NCT02061969	2018	USA	Basal insulin	73	Insulin glargine		Metformin	6		American Diabetes Association
**Linagliptin-LTC Trial**	NCT02061969	2018	USA	DPP-4 inhibitors	67	Linagliptin	5 mg daily	Metformin	6		American Diabetes Association
**Lindstrom 2000**	Not applicable	2000	Finland	Alpha-glucosidase inhibitors	55	Acarbose	300 mg daily	Biguianide sulfonylurea	5.5		Not reported
**Lindstrom 2000**	Not applicable	2000	Finland	Standard treatments	52	Placebo		Biguianide sulfonylurea	5.5		Not reported
**Lingvay 2018**	NCT02461589	2018	Multinational	GLP-1 receptor agonists	65	Liraglutide	1.8 mg daily	None or metformin	6		Novo Nordisk
**Lingvay 2018**	NCT02461589	2018	Multinational	GLP-1 receptor agonists	64	Liraglutide	1.2 mg daily	None or metformin	6		Novo Nordisk

Study ID	Registration	Year	Country	Intervention	N	Drug	Dose	Cointervention	Follow-up*	Settings	Fundings
**Lingvay 2018**	NCT02461589	2018	Multinational	GLP-1 receptor agonists	64	Liraglutide	0.6 mg daily	None or metformin	6		Novo Nordisk
**Lingvay 2018**	NCT02461589	2018	Multinational	GLP-1 receptor agonists	64	Liraglutide	0.3 mg daily	None or metformin	6		Novo Nordisk
**Lingvay 2018**	NCT02461589	2018	Multinational	GLP-1 receptor agonists	63	Semaglutide	0.3 mg daily	None or metformin	6		Novo Nordisk
**Lingvay 2018**	NCT02461589	2018	Multinational	GLP-1 receptor agonists	65	Semaglutide	0.2 mg daily	None or metformin	6		Novo Nordisk
**Lingvay 2018**	NCT02461589	2018	Multinational	GLP-1 receptor agonists	63	Semaglutide	0.1 mg daily	None or metformin	6		Novo Nordisk
**Lingvay 2018**	NCT02461589	2018	Multinational	GLP-1 receptor agonists	64	Semaglutide	0.05 mg daily	None or metformin	6		Novo Nordisk
**Lingvay 2018**	NCT02461589	2018	Multinational	GLP-1 receptor agonists	512	Semaglutide/liraglutide	Multiple doses	None or metformin	6		Novo Nordisk
**Lingvay 2018**	NCT02461589	2018	Multinational	Standard treatments	129	Placebo		None or metformin	6		Novo Nordisk

Study ID	Registration	Year	Country	Intervention	N	Drug	Dose	Cointervention	Follow-up*	Settings	Fundings
**LIPER2**	EudraCT number 2012-005197-63	2019	Spain	GLP-1 receptor agonists	12	Liraglutide	Liraglutide	Metformin if tolerated and not contraindicated) or a maximum of two intermediate- or long-acting insulin injections per day, or a combination of both	6		Novo Nordisk
**LIPER2**	EudraCT number 2012-005197-63	2019	Spain	Standard treatments	12	Placebo		Metformin if tolerated and not contraindicated) or a maximum of two intermediate- or long-acting insulin injections per day, or a combination of both	6		Novo Nordisk
**LIRA-ADD2SGLT2i**	NCT02964247	2020	Multinational	GLP-1 receptor agonists	203	Liraglutide	1.8 mg daily	Metformin SGLT-2 inhibitor	6		Novo Nordisk
**LIRA-ADD2SGLT2i**	NCT02964247	2020	Multinational	Standard treatments	100	Placebo		Metformin SGLT-2 inhibitor	6		Novo Nordisk

Study ID	Registration	Year	Country	Intervention	N	Drug	Dose	Cointervention	Follow -up*	Settings	Fundings
**LIRA-RENAL**	NCT01620489	2016	Multinational	GLP-1 receptor agonists	140	Liraglutide	1.8 mg daily	Monotherapy or dual therapy combinations with metformin, sulfonylurea, thiazolidinedione, or insulin	6	Chronic kidney disease	Novo Nordisk
**LIRA-RENAL**	NCT01620489	2016	Multinational	Standard treatments	137	Placebo		Monotherapy or dual therapy combinations with metformin, sulfonylurea, thiazolidinedione, or insulin	6		Novo Nordisk
**LIRA-SWITCH**	NCT01907854	2016	Multinational	DPP-4 inhibitors	202	Sitagliptin	100 mg daily	Metformin	5.5		Novo Nordisk
**LIRA-SWITCH**	NCT01907854	2016	Multinational	GLP-1 receptor agonists	204	Liraglutide	1.8 mg daily	Metformin	5.5		Novo Nordisk
**LIRAFLAME**	NCT03449654	2021	Denmark	GLP-1 receptor agonists	51	Liraglutide	1.8 mg/d	None	6		Novo Nordisk A/S
**LIRAFLAME**	NCT03449654	2021	Denmark	Standard treatments	51	Placebo		None	6		Novo Nordisk A/S

Study ID	Registration	Year	Country	Intervention	N	Drug	Dose	Cointervention	Follow -up*	Settings	Fundings
**Liraglutide-Detemir Study**	NCT00856986	2012	Multinational	Basal insulin	162	Insulin detemir	Titrated	Metformin sulfonylurea	6		Novo Nordisk
**Liraglutide-Detemir Study**	NCT00856986	2012	Multinational	Standard treatments	161	Control		Metformin sulfonylurea	6		Novo Nordisk
**Liu 2013**	NCT01195090	2013	Taiwan	DPP-4 inhibitors	60	Sitagliptin	100 mg daily	Metformin or sulfonylurea	5.5		Mackay Memorial Hospital
**Liu 2013**	NCT01195090	2013	Taiwan	Thiazolidinediones	59	Pioglitazone	30 mg daily	Metformin or sulfonylurea	5.5		Mackay Memorial Hospital
**Liu 2014**	Not provided	2016	China	Standard treatments	76	Placebo		Unclear	14		Not reported
**Liu 2014**	Not provided	2016	China	Thiazolidinediones	70	Pioglitazone	30 mg daily	Unclear	14	Atrial fibrillation	Not reported
**Liu 2020**	NCT02303730	2020	China	Basal insulin	36	Insulin glargine		None	5.5		AstraZeneca China and 3SBio Inc.
**Liu 2020**	NCT02303730	2020	China	GLP-1 receptor agonists	35	Exenatide	20 mcg daily	None	5.5		AstraZeneca China and 3SBio Inc.
**Liu-2021**	NCT03458715	2020	Taiwan	DPP-4 inhibitors	53	Linagliptin	5 mg once daily	Premixed insulin regimen	6		None
**Liu-2021**	NCT03458715	2020	Taiwan	SGLT2 inhibitors	53	Empagliflozin	25 mg once daily	Premixed insulin regimen	6		None
**Liutkus 2010**	Not provided	2010	Multinational	GLP-1 receptor agonists	111	Exenatide	20 mcg daily	Metformin and thiazolidinedione	6		Eli Lilly Co/Amylin Pharmaceuticals

Study ID	Registration	Year	Country	Intervention	N	Drug	Dose	Cointervention	Follow -up*	Settings	Fundings
**Liutkus 2010**	Not provided	2010	Multinational	Standard treatments	54	Placebo		Metformin and thiazolidinedione	6		Eli Lilly Co/Amylin Pharmaceuticals
**Lou 2020**	Not reported	2020	China	GLP-1 receptor agonists	43	Liraglutide	1.2 mg daily	Unclear	6		Not reported
**Lou 2020**	Not reported	2020	China	Metformin	43	Metformin	1500 mg daily	Unclear	6		Not reported
**Lu 2016**	NCT01505426	2016	Multinational	SGLT2 inhibitors	87	Ipragliflozin	50 mg daily	Metformin	5.5		Astellas Pharma
**Lu 2016**	NCT01505426	2016	Multinational	Standard treatments	83	Placebo		Metformin	5.5		Astellas Pharma
**Lu-2021**	NCT03159052	2021	China	SGLT2 inhibitors	156	Henagliflozin	5 mg once daily	None	5.5		Jiangsu Hengrui Medicine Co.Ltd
**Lu-2021**	NCT03159052	2021	China	SGLT2 inhibitors	156	Henagliflozin	10 mg once daily	None	5.5		Jiangsu Hengrui Medicine Co.Ltd
**Lu-2021**	NCT03159052	2021	China	SGLT2 inhibitors	312	Henagliflozin	5-10 mg daily	None	5.5		Jiangsu Hengrui Medicine Co.Ltd
**Lu-2021**	NCT03159052	2021	China	Standard treatments	156	Placebo		None	5.5		Jiangsu Hengrui Medicine Co.Ltd
**Lukashevich 2011_1**	Not provided	2011	Finland	DPP-4 inhibitors	165	Vildagliptin	50 mg daily	Untreated or treated with sulfonylurea, alpha glucosidase inhibitor, thiazolidinedione,	5.5	Chronic kidney disease	Novartis

Study ID	Registration	Year	Country	Intervention	N	Drug	Dose	Cointervention	Follow -up*	Settings	Fundings
								insulin, meglitinide, or combination			
**Lukashevich 2011_1**	Not provided	2011	Finland	Standard treatments	129	Placebo		Untreated or treated with sulfonylurea, alpha glucosidase inhibitor, thiazolidinedione, insulin, meglitinide, or combination	5.5		Novartis
**Lukashevich 2011_2**	Not provided	2011	Finland	DPP-4 inhibitors	124	Vildagliptin	50 mg daily	Untreated or treated with sulfonylurea, alpha glucosidase inhibitor, thiazolidinedione, insulin, meglitinide, or combination	5.5		Novartis
**Lukashevich 2011_2**	Not provided	2011	Finland	Standard treatments	97	Placebo		Untreated or treated with sulfonylurea, alpha glucosidase inhibitor, thiazolidinedione, insulin, meglitinide, or combination	5.5		Novartis
**Lund 2009**	NCT00118963	2009	Denmark	Meglitinides	49	Repaglinide	6 mg daily	Biphasic insulin	12		Novo Nordisk

Study ID	Registration	Year	Country	Intervention	N	Drug	Dose	Cointervention	Follow -up*	Settings	Fundings
**Lund 2009**	NCT00118963	2009	Denmark	Metformin	52	Metformin	2000 mg daily	Biphasic insulin	12		Novo Nordisk
**LYDIA**	NCT02043054	2020	UK	DPP-4 inhibitors	38	Sitagliptin	100 mg once dailty	metformin and/or any sulphonylurea	6		NovoNordisk
**LYDIA**	NCT02043054	2020	UK	GLP-1 receptor agonists	38	Liraglutide	1.8 mg once daily	metformin and/or any sulphonylurea	6	young obese adults	NovoNordisk
**Macauley 2015**	NCT01356381	2015	United Kingdom	DPP-4 inhibitors	22	Vildagliptin	100 mg daily	Metformin	6		Novartis Pharma AG
**Macauley 2015**	NCT01356381	2015	United Kingdom	Standard treatments	22	Placebo		Metformin	6		Novartis Pharma AG
**Madsbad 2001**	Not applicable	2001	Europe	Meglitinides	175	Repaglinide	1.5-12 mg daily	None or oral agents	12		Novo Nordisk
**Madsbad 2001**	Not applicable	2001	Europe	Sulfonylureas	81	Glipizide	5-15 mg daily	None or oral agents	12		Novo Nordisk
**Maffioli 2013**	Not provided	2013	Italy	Sulfonylureas	84	Glibenclamide	10 mg daily	Metformin	6		Not reported
**Maffioli 2013**	Not provided	2013	Italy	Thiazolidined iones	86	Pioglitazone	30 mg daily	Metformin	6		Not reported
**MAGNA VICTORIA**	NCT01761318	2019	The Netherlands	GLP-1 receptor agonists	23	Liraglutide	1.8 mg daily	Metformin with or without sulfonylurea and/or insulin	6		Novo Nordisk
**MAGNA VICTORIA**	NCT01761318	2019	The Netherlands	Standard treatments	26	Placebo		Metformin with or without	6		Novo Nordisk

Study ID	Registration	Year	Country	Intervention	N	Drug	Dose	Cointervention	Follow -up*	Settings	Fundings
								sulfonylurea and/or insulin			
**Marbury 1999**	Not applicable	1999	USA	Meglitinides	383	Repaglinide	1.5-12 mg daily	None or oral agents	12		Novo Nordisk
**Marbury 1999**	Not applicable	1999	USA	Sulfonylureas	193	Glyburide	2.5-15 mg daily	None or oral agents	12		Novo Nordisk
**MARCH**	ChiCTR-TRC-08000231	2014	China	Alpha-glucosidase inhibitors	391	Acarbose	300 mg daily	None	11		Bayer Healthcare (China) and Double Crane Phama.
**MARCH**	ChiCTR-TRC-08000231	2014	China	Metformin	393	Metformin	1500 mg daily	None	11		Bayer Healthcare (China) and Double Crane Phama.
**Mari 2008**	NCT00101712	2008	Multinational	DPP-4 inhibitors	156	Vildagliptin	50 mg daily	None	12		Novartis
**Mari 2008**	NCT00101712	2008	Multinational	Standard treatments	150	Placebo		None	12		Novartis
**MARLINA-T2D**	NCT01792518	2017	Multinational	DPP-4 inhibitors	182	Linagliptin	5 mg daily	Antidiabetic drugs including insulin	5.5	Chronic kidney disease	Boehringer Ingelheim
**MARLINA-T2D**	NCT01792518	2017	Multinational	Standard treatments	178	Placebo		Antidiabetic drugs including insulin	5.5		Boehringer Ingelheim
**Marre 2002**	Not applicable	2002	Multinational	Meglitinides	160	Nateglinide	360 mg daily	Metformin	5.5		Novartis
**Marre 2002**	Not applicable	2002	Multinational	Meglitinides	155	Nateglinide	180 mg daily	Metformin	5.5		Novartis

Study ID	Registration	Year	Country	Intervention	N	Drug	Dose	Cointervention	Follow-up*	Settings	Fundings
**Marre 2002**	Not applicable	2002	Multinational	Meglitinides	315	Nateglinide	180-360 mg daily	Metformin	5.5		Novartis
**Marre 2002**	Not applicable	2002	Multinational	Standard treatments	152	Placebo		Metformin	5.5		Novartis
**MASTER**	UMIN000001671	2014	Japan	Alpha-glucosidase inhibitors	14	Miglitol	150 mg daily	Metformin or sulphonylurea or no treatment	5.5		Japanese Ministry of Education, Culture, Sports, Science, and Technology and from the Japanese Ministry of Health, Labour and Welfare
**MASTER**	UMIN000001671	2014	Japan	DPP-4 inhibitors	14	Sitagliptin	50 mg daily	Metformin or sulphonylurea or no treatment	5.5		Japanese Ministry of Education, Culture, Sports, Science, and Technology and from the Japanese Ministry of Health, Labour and Welfare
**Mathieu 2015**	NCT01462266	2015	Unclear	DPP-4 inhibitors	329	Sitagliptin	100 mg daily	Insulin metformin	5.5		Merck Sharp & Dohme
**Mathieu 2015**	NCT01462266	2015	Unclear	Standard treatments	329	Placebo		Insulin metformin	5.5		Merck Sharp & Dohme
**Mathieu 2016**	NCT01646320	2016	Mexico	SGLT2 inhibitors	160	Dapagliflozin	10 mg daily	Metformin DPP-4 inhibitor	12		Bristol-Myers Squibb and AstraZeneca

Study ID	Registration	Year	Country	Intervention	N	Drug	Dose	Cointervention	Follow -up*	Settings	Fundings
**Mathieu 2016**	NCT01646320	2016	Mexico	Standard treatments	160	Placebo		Metformin DPP-4 inhibitor	12		Bristol-Myers Squibb and AstraZeneca
**Matthaei 2015**	NCT01619059	2015	Multinational	DPP-4 inhibitors	160	Saxagliptin	5 mg daily	Metformin + dapagliflozin	12		Bristol-Myers Squibb and AstraZeneca
**Matthaei 2015**	NCT01619059	2015	Multinational	Standard treatments	162	Placebo		Metformin + dapagliflozin	12		Bristol-Myers Squibb and AstraZeneca
**Matthaei 2015a**	NCT01392677	2015	Multinational	SGLT2 inhibitors	109	Dapagliflozin	10 mg daily	Metformin + sulfonylurea	5.5		Bristol-Myers Squibb and AstraZeneca
**Matthaei 2015a**	NCT01392677	2015	Multinational	Standard treatments	109	Placebo		Metformin + sulfonylurea	5.5		Bristol-Myers Squibb and AstraZeneca
**Matthews 2005**	Not applicable	2005	Multinational	Sulfonylureas	313	Gliclazide	80-320 mg daily	Metformin	12		Eli Lilly and Company
**Matthews 2005**	Not applicable	2005	Multinational	Thiazolidinediones	317	Pioglitazone	15-45 mg daily	Metformin	12		Eli Lilly and Company
**Matthews 2010**	EudraCT 2004-004559-21	2010	Multicentre	DPP-4 inhibitors	1562	Vildagliptin	50 mg twice daily	Metformin	24		Novartis
**Matthews 2010**	EudraCT 2004-004559-21	2010	Multicentre	Sulfonylureas	1556	Glimepiride	2-6 mg daily	Metformin	24		Novartis
**Mattoo 2005**	Not applicable	2005	Multinational	Standard treatments	147	Placebo		None or oral agents	6		Eli Lilly and Company
**Mattoo 2005**	Not applicable	2005	Multinational	Thiazolidinediones	142	Pioglitazone	30 mg daily	None or oral agents	6		Eli Lilly and Company
**McCluskey 2004**	Not applicable	2004	USA	Standard treatments	15	Placebo		Rosiglitazone	6		Aventis Pharmaceuticals

Study ID	Registration	Year	Country	Intervention	N	Drug	Dose	Cointervention	Follow -up*	Settings	Fundings
**McCluskey 2004**	Not applicable	2004	USA	Sulfonylureas	25	Glimepiride	2-8 mg daily	Rosiglitazone	6		Aventis Pharmaceuticals
**McGill 2013**	Not provided	2013	Multinational	DPP-4 inhibitors	68	Linagliptin	5 mg daily	Insulin, sulfonylurea, glinides, pioglitazone, and alpha -glucosidase inhibitors	12	Chronic kidney disease	Boehringer Ingelheim
**McGill 2013**	Not provided	2013	Multinational	Standard treatments	65	Placebo		Insulin, sulfonylurea, glinides, pioglitazone, and alpha -glucosidase inhibitors	12		Boehringer Ingelheim
**McGuire 2010**	NCT00424762	2010	USA	Standard treatments	76	Placebo		Existing treatment except thiazolidinediones	6		Glaxo Smith Kline; Biosite, Inc.; the Donald W. Reynolds Foundation; and the United States Public Health Services General Clinical Research Center (grant #M01-RR00633) from National Institutes of

Study ID	Registration	Year	Country	Intervention	N	Drug	Dose	Cointervention	Follow-up*	Settings	Fundings
											Health/National Center Research Resources-Clinical Research
**McGuire 2010**	NCT00424762	2010	USA	Thiazolidinediones	74	Rosiglitazone	4-8 mg daily	Existing treatment except thiazolidinediones	6	High cardiovascular risk	Glaxo Smith Kline; Biosite, Inc.; the Donald W. Reynolds Foundation; and the United States Public Health Services General Clinical Research Center (grant #M01-RR00633) from National Institutes of Health/National Center Research Resources-Clinical Research
**MDI Liraglutide**	EudraCT 2012-001941-42	2015	Sweden	GLP-1 receptor agonists	64	Liraglutide	1.8 mg daily	Insulin	5.5		Novo Nordisk
**MDI Liraglutide**	EudraCT 2012-001941-42	2015	Sweden	Standard treatments	60	Placebo		Insulin	5.5		Novo Nordisk

Study ID	Registration	Year	Country	Intervention	N	Drug	Dose	Cointervention	Follow -up*	Settings	Fundings
**Meneghini 2010**	Not provided	2010	USA	Basal insulin	121	Insulin glargine	Titrated	Metformin or sulphonylurea or no treatment	11		Sanofi -Aventis
**Meneghini 2010**	Not provided	2010	USA	Thiazolidinediones	126	Pioglitazone	45 mg daily	Metformin or sulphonylurea or no treatment	11		Sanofi -Aventis
**Mita 2007**	Not provided	2007	Japan	Meglitinides	34	Nateglinide	270 mg daily	None	12		Not reported
**Mita 2007**	Not provided	2007	Japan	Standard treatments	36	Control		None	12		Not reported
**Mita 2019**	UMIN000022953	2019	Japan	DPP-4 inhibitors	21	Linagliptin	5 mg daily	None or insulin	5.5		Nippon Boehringer Ingelheim Co., Ltd. and Eli Lilly and Company
**Mita 2019**	UMIN000022953	2019	Japan	Metformin	22	Metformin	2250 mg daily	None or insulin	5.5		Nippon Boehringer Ingelheim Co., Ltd. and Eli Lilly and Company
**Mitrakou 1998**	Not applicable	1998	Greece	Alpha-glucosidase inhibitors	60	Miglitol	300 mg daily	Insulin	5.5		University of Athens/Bayer AG
**Mitrakou 1998**	Not applicable	1998	Greece	Standard treatments	60	Placebo		Insulin	5.5		University of Athens/Bayer AG

Study ID	Registration	Year	Country	Intervention	N	Drug	Dose	Cointervention	Follow -up*	Settings	Fundings
**Miyagawa 2015**	NCT01558271	2015	Japan	GLP-1 receptor agonists	281	Dulaglutide	0.75 mg weekly	None	6		Eli Lilly and Company
**Miyagawa 2015**	NCT01558271	2015	Japan	GLP-1 receptor agonists	141	Liraglutide	0.9 mg daily	None	6		Eli Lilly and Company
**Miyagawa 2015**	NCT01558271	2015	Japan	GLP-1 receptor agonists	422	Dulaglutide or liraglutide	0.75 mg weekly or 0.9 mg daily	None	6		Eli Lilly and Company
**Miyagawa 2015**	NCT01558271	2015	Japan	Standard treatments	70	Placebo		None	6		Eli Lilly and Company
**Miyazaki 2002**	Not applicable	2002	USA	Standard treatments	11	Placebo		None	6		Takeda
**Miyazaki 2002**	Not applicable	2002	USA	Thiazolidinediones	11	Pioglitazone	45 mg daily	None	6		Takeda
**Miyazaki 2002**	Not applicable	2002	USA	Thiazolidinediones	11	Pioglitazone	30 mg daily	None	6		Takeda
**Miyazaki 2002**	Not applicable	2002	USA	Thiazolidinediones	12	Pioglitazone	15 mg daily	None	6		Takeda
**Miyazaki 2002**	Not applicable	2002	USA	Thiazolidinediones	13	Pioglitazone	7.5 mg daily	None	6		Takeda
**Miyazaki 2002**	Not applicable	2002	USA	Thiazolidinediones	47	Pioglitazone	7.5-45 mg daily	None	6		Takeda

Study ID	Registration	Year	Country	Intervention	N	Drug	Dose	Cointervention	Follow-up*	Settings	Fundings
**MK-0431J-842**	NCT02577016	2021	Japan	DPP-4 inhibitors	70	Sitagliptin	50 mg once daily	Ipragliflozin	5.5		Merck & Co., Inc.
**MK-0431J-842**	NCT02577016	2021	Japan	Standard treatments	71	Placebo		Ipragliflozin	5.5		Merck & Co., Inc.
**Moeinzadeh-2021**	IRCT20090905002417N23	2021	Iran	DPP-4 inhibitors	68	Linagliptin	5 mg	None	6	Severely Increased Albuminuria	Alhavi pharma company
**Moeinzadeh-2021**	IRCT20090905002417N23	2021	Iran	Standard treatments	68	Placebo		None	6		Alhavi pharma company
**Mokta 2018**	Not provided	2018	India	DPP-4 inhibitors	106	Vildagliptin	100 mg daily	Metformin	30		Not reported
**Mokta 2018**	Not provided	2018	India	Sulfonylureas	111	Glimepiride	2 mg daily	Metformin	30		Not reported
**Moretto 2008**	NCT00381342	2008	USA	GLP-1 receptor agonists	78	Exenatide	10 mcg daily	None	5.5		Eli Lilly and Company
**Moretto 2008**	NCT00381342	2008	USA	GLP-1 receptor agonists	77	Exenatide	5 mcg daily	None	5.5		Eli Lilly and Company
**Moretto 2008**	NCT00381342	2008	USA	GLP-1 receptor agonists	155	Exenatide	5-10 mcg daily	None	5.5		Eli Lilly and Company
**Moretto 2008**	NCT00381342	2008	USA	Standard treatments	78	Placebo		None	5.5		Eli Lilly and Company
**Morino 2018**	Not provided	2018	Japan	SGLT2 inhibitors	22	Ipragliflozin		Insulin	5.5		Not reported

Study ID	Registration	Year	Country	Intervention	N	Drug	Dose	Cointervention	Follow -up*	Settings	Fundings
**Morino 2018**	Not provided	2018	Japan	Standard treatments	24	Control		Insulin	5.5		Not reported
**Moriwaki 2018**	UMIN000012562	2018	Japan	Alpha-glucosidase inhibitors	14	Voglibose	0.9 mg daily	None or fixed doses of glucose-lowering drugs	5.5		Waksman Foundation of Japan Inc
**Moriwaki 2018**	UMIN000012562	2018	Japan	DPP-4 inhibitors	14	Sitagliptin	100 mg daily	None or fixed doses of glucose-lowering drugs	5.5	Coronary artery disease	Waksman Foundation of Japan Inc
**Moses 2014**	Not provided	2014	Multinational	DPP-4 inhibitors	129	Saxagliptin	5 mg daily	Metformin + sulfonylurea	5.5		Bristol-Myers Squibb; AstraZeneca
**Moses 2014**	Not provided	2014	Multinational	Standard treatments	128	Placebo		Metformin + sulfonylurea	5.5		Bristol-Myers Squibb; AstraZeneca
**Moses 2016**	NCT01076075	2016	Multinational	DPP-4 inhibitors	213	Sitagliptin	100 mg daily	Metformin + sulfonylurea	5.5		Merck and Company
**Moses 2016**	NCT01076075	2016	Multinational	Standard treatments	214	Placebo		Metformin + sulfonylurea	5.5		Merck and Company
**Mu 2017**	NCT01708902	2017	China	DPP-4 inhibitors	147	Linagliptin	5 mg daily	None	5.5		Boehringer Ingelheim, and Eli Lilly & Company
**Mu 2017**	NCT01708902	2017	China	Metformin	144	Metformin	2000 mg daily	None	5.5		Boehringer Ingelheim, and Eli Lilly & Company
**Mu 2017**	NCT01708902	2017	China	Metformin	145	Metformin	1000 mg daily	None	5.5		Boehringer Ingelheim, and Eli Lilly & Company

Study ID	Registration	Year	Country	Intervention	N	Drug	Dose	Cointervention	Follow -up*	Settings	Fundings
**Mu 2017**	NCT01708902	2017	China	Metformin	289	Metformin	1000-2000 mg daily	None	5.5		Boehringer Ingelheim, and Eli Lilly & Company
**Müller-Wieland 2018**	NCT02471404	2018	Multinational	SGLT2 inhibitors	314	Dapagliflozin	10 mg daily	Metformin	12		AstraZeneca
**Müller-Wieland 2018**	NCT02471404	2018	Multinational	Sulfonylureas	313	Glimepiride	6 mg daily	Metformin	12		AstraZeneca
**MUSCAT-HF**	UMIN000018395	2020	Japan	Alpha-glucosidase inhibitors	83	Voglibose	0.2 mg 3 times daily	diet and exercise therapy, a lifestyle modification program, and hypoglycemic medications based on standard guidelines of the Japan Diabetes Society	5.5		This work was supported by Novartis Pharma K. K.
**MUSCAT-HF**	UMIN000018395	2020	Japan	SGLT2 inhibitors	86	luseogliflozin	2.5 mg once daily	diet and exercise therapy, a lifestyle modification program, and hypoglycemic medications based on standard guidelines of the	5.5	heart failure with preserved ejection fraction	This work was supported by Novartis Pharma K. K.

Study ID	Registration	Year	Country	Intervention	N	Drug	Dose	Cointervention	Follow -up*	Settings	Fundings
								Japan Diabetes Society			
**N-ISM**	jRCTs031180205	2020	Japan	DPP-4 inhibitors	58	Sitagliptin	50 mg/d	treatment with metformin therapy (≥500 mg/d) with or without other antidiabetic agents	12		This study was funded by Astellas Pharma Inc.<br><br>The company was not involved in study design,<br><br>patient selection, data aggregation/analysis,<br><br>interpretation of results, or preparation of the<br><br>manuscript.
**N-ISM**	jRCTs031180205	2020	Japan	SGLT2 inhibitors	57	Ipragliflozin	50 mg/d	treatment with metformin therapy (≥500 mg/d) with or without other antidiabetic agents	12	without prior ASCVD	This study was funded by Astellas Pharma Inc.<br><br>The company was not involved in study design,

Study ID	Registration	Year	Country	Intervention	N	Drug	Dose	Cointervention	Follow -up*	Settings	Fundings
											patient selection, data aggregation/analysis, interpretation of results, or preparation of the manuscript.
**Nahra-2021**	NCT03235050	2021	120 sites in eight countries: Bulgaria, Canada, Czech Republic, Germany, Mexico, Russia, Slovakia, and the U.S.	GLP-1 receptor agonists	110	Liraglutide	1.8 mg once daily	on a background of stable metformin treatment that was maintained for the study duration	13.4		All aspects of the study and manuscript preparation were sponsored by AstraZeneca.
**Nahra-2021**	NCT03235050	2021	120 sites in eight countries: Bulgaria,	Standard treatments	112	Placebo		on a background of stable metformin treatment that was	13.4		All aspects of the study and manuscript preparation were

Study ID	Registration	Year	Country	Intervention	N	Drug	Dose	Cointervention	Follow -up*	Settings	Fundings
			Canada, Czech Republic, Germany, Mexico, Russia, Slovakia, and the U.S.					maintained for the study duration			sponsored by AstraZeneca.
**Nakamura 2001**	Not applicable	2001	Japan	Standard treatments	14	Placebo		Sulfonylurea	6		Not reported
**Nakamura 2001**	Not applicable	2001	Japan	Thiazolidinediones	14	Pioglitazone	30mg daily	Sulfonylurea	6	Chronic kidney disease	Not reported
**Nakamura 2004**	Not applicable	2004	Japan	Alpha-glucosidase inhibitors	15	Voglibose	0.6mg daily	None	12		Not reported
**Nakamura 2004**	Not applicable	2004	Japan	Sulfonylureas	15	Glibenclamide (Macroalbuminuria)	5-20 mg daily	None	12	Chronic kidney disease	Not reported
**Nakamura 2004**	Not applicable	2004	Japan	Thiazolidinediones	15	Pioglitazone	30 mg daily	None	12		Not reported
**Nakamura 2006**	Not provided	2006	Japan	Alpha-glucosidase inhibitors	17	Voglibose	0.6mg daily	None	12		Not reported
**Nakamura 2006**	Not provided	2006	Japan	Meglitinides	16	Netaglinide	270mg daily	None	12		Not reported

Study ID	Registration	Year	Country	Intervention	N	Drug	Dose	Cointervention	Follow -up*	Settings	Fundings
**Nakamura 2006**	Not provided	2006	Japan	Sulfonylureas	18	Glibenclamide (Macroalbuminuria)	5-20 mg daily	None	12	Chronic kidney disease	Not reported
**Nakamura 2006**	Not provided	2006	Japan	Thiazolidinediones	17	Pioglitazone	30 mg daily	None	12		Not reported
**Nar 2009**	Not provided	2009	Turkey	Metformin	19	Metformin	850-1700 mg daily	None	6		Not reported
**Nar 2009**	Not provided	2009	Turkey	Standard treatments	15	Control		None	6		Not reported
**Nathan 1988**	Not applicable	1988	USA	Basal insulin	15	NPH Insulin	Titrated	None	9		Upjohn, Eli Lilly and Miles Laboratories
**Nathan 1988**	Not applicable	1988	USA	Sulfonylureas	16	Glyburide	10.5 mg daily	None	9		Upjohn, Eli Lilly and Miles Laboratories
**Nauck 2007a**	Not provided	2007	Multinational	Basal-Bolus insulin	248	Biphasic Insulin Aspartate 30/70	Titrated	Metformin + sulfonylurea	12		Lilly and Amylin
**Nauck 2007a**	Not provided	2007	Multinational	GLP-1 receptor agonists	253	Exenatide	10-20 mcg daily	Metformin + sulfonylurea	12		Lilly and Amylin
**Nauck 2007b**	NCT00094770	2007	Multinational	DPP-4 inhibitors	588	Sitagliptin	100 mg daily	None, monotherapy or metformin + monotherapy	12		Merck
**Nauck 2007b**	NCT00094770	2007	Multinational	Sulfonylureas	584	Glipizide	5-20 mg daily	None, monotherapy or metformin + monotherapy	12		Merck

Study ID	Registration	Year	Country	Intervention	N	Drug	Dose	Cointervention	Follow -up*	Settings	Fundings
**Nauck 2009**	NCT00286442	2009	Multinational	DPP-4 inhibitors	210	Alogliptin	25mg daily	Metformin	6		Takeda
**Nauck 2009**	NCT00286442	2009	Multinational	DPP-4 inhibitors	213	Alogliptin	12.5mg daily	Metformin	6		Takeda
**Nauck 2009**	NCT00286442	2009	Multinational	DPP-4 inhibitors	423	Alogliptin	12.5-25 mg daily	Metformin	6		Takeda
**Nauck 2009**	NCT00286442	2009	Multinational	Standard treatments	104	Placebo		Metformin	6		Takeda
**Nauck 2011**	NCT00660907	2011	Multinational	SGLT2 inhibitors	406	Dapagliflozin	2.5-10 mg daily	Metformin oral agent	12		AstraZeneca and Bristol-Myers Squibb
**Nauck 2011**	NCT00660907	2011	Multinational	Sulfonylureas	408	Glipizide	5-20 mg daily	Metformin oral agent	12		AstraZeneca and Bristol-Myers Squibb
**Nauck 2016**	NCT01183013	2016	Multinational	DPP-4 inhibitors	135	Linagliptin	5 mg daily	None	7		Boehringer Ingelheim
**Nauck 2016**	NCT01183013	2016	Multinational	Thiazolidinediones	138	Pioglitazone	45 mg daily	None	7		Boehringer Ingelheim
**Nauck 2016**	NCT01183013	2016	Multinational	Thiazolidinediones	140	Pioglitazone	30 mg daily	None	7		Boehringer Ingelheim
**Nauck 2016**	NCT01183013	2016	Multinational	Thiazolidinediones	131	Pioglitazone	15 mg daily	None	7		Boehringer Ingelheim
**Nauck 2016**	NCT01183013	2016	Multinational	Thiazolidinediones	409	Pioglitazone	15-45 mg daily	None	7		Boehringer Ingelheim
**Negro 2005**	Not applicable	2005	Italy	Standard treatments	19	Placebo		Metformin	12		None reported

Study ID	Registration	Year	Country	Intervention	N	Drug	Dose	Cointervention	Follow -up*	Settings	Fundings
**Negro 2005**	Not applicable	2005	Italy	Thiazolidinediones	19	Rosiglitazone	8 mg daily	Metformin	12		None reported
**Ning 2016**	NCT01582230	2016	Multinational	DPP-4 inhibitors	146	Vildagliptin	100 mg daily	Metformin insulin	5.5		Novartis Pharma
**Ning 2016**	NCT01582230	2016	Multinational	Standard treatments	147	Placebo		Metformin insulin	5.5		Novartis Pharma
**Nino 2017**	NCT01733758	2017	Japan	GLP-1 receptor agonists	150	Albiglutide	50 mg weekly	Single oral drug	5.5		GlaxoSmithKline
**Nino 2017**	NCT01733758	2017	Japan	GLP-1 receptor agonists	160	Albiglutide	30 mg weekly	Single oral drug	5.5		GlaxoSmithKline
**Nino 2017**	NCT01733758	2017	Japan	GLP-1 receptor agonists	310	Albiglutide	30-50 mg weekly	Single oral drug	5.5		GlaxoSmithKline
**Nino 2017**	NCT01733758	2017	Japan	Standard treatments	77	Placebo		Single oral drug	5.5		GlaxoSmithKline
**Nishimura 2016**	UMIN000011420	2016	Japan	DPP-4 inhibitors	20	Sitagliptin	50 mg daily	Metformin pioglitazone + repaglinide	5.5		Not reported
**Nishimura 2016**	UMIN000011420	2016	Japan	Standard treatments	20	Control		Metformin pioglitazone + repaglinide	5.5		Not reported

Study ID	Registration	Year	Country	Intervention	N	Drug	Dose	Cointervention	Follow -up*	Settings	Fundings
**Nishio 2006**	Not provided	2006	Japan	Standard treatments	28	Control		alpha -glucosidase inhibitor sulfonylurea	6		Not reported
**Nishio 2006**	Not provided	2006	Japan	Thiazolidinediones	26	Pioglitazone	30 mg daily	alpha -glucosidase inhibitor sulfonylurea	6	Coronary artery disease	Not reported
**Nogueira 2014**	Not provided	2014	Brazil	Basal insulin	17	NPH Insulin	Titrated	Metformin; Glyburide	5.5		Fundacao de Amparo a Pesquisa do Estado de Sau Paulo
**Nogueira 2014**	Not provided	2014	Brazil	DPP-4 inhibitors	18	Sitagliptin	100 mg daily	Metformin; Glyburide	5.5		Fundacao de Amparo a Pesquisa do Estado de Sau Paulo
**Nowicki 2011**	NCT00614939	2011	Multinational	DPP-4 inhibitors	85	Saxagliptin	2.5 mg daily	Excluded metformin therapy, and previous or current DPP-4 inhibitor or GLP1 receptor agonist	12	Chronic kidney disease	Bristol-Myers Squibb; AstraZeneca
**Nowicki 2011**	NCT00614939	2011	Multinational	Standard treatments	85	Placebo		Excluded metformin therapy, and previous or current DPP-4 inhibitor or GLP1 receptor agonist	12		Bristol-Myers Squibb; AstraZeneca

Study ID	Registration	Year	Country	Intervention	N	Drug	Dose	Cointervention	Follow -up*	Settings	Fundings
**Ogasawara 2009**	Not provided	2009	Japan	Standard treatments	27	Control		Oral antidigabetic treatments	6		Not reported
**Ogasawara 2009**	Not provided	2009	Japan	Thiazolidinediones	27	Pioglitazone	15 mg daily	Oral antidigabetic treatments	6	Coronary artery disease	Not reported
**Oh 2019**	NCT01370707	2019	Republic of Korea	Alpha-glucosidase inhibitors	95	Voglibose	0.6 mg daily	Metformin	5.5		CJ Healthcare Co.
**Oh 2019**	NCT01370707	2019	Republic of Korea	Standard treatments	92	Control		Metformin	5.5		CJ Healthcare Co.
**Ohira 2014**	Not provided	2014	Japan	DPP-4 inhibitors	35	Sitagliptin	50 mg daily	Metformin	6		Not reported
**Ohira 2014**	Not provided	2014	Japan	Metformin	35	Metformin	500 mg daily	Metformin	6		Not reported
**Ohira 2014a**	Not provided	2014	Japan	Sulfonylureas	30	Glimepiride	1 mg daily	Metformin	6		Not reported
**Ohira 2014a**	Not provided	2014	Japan	Thiazolidinediones	30	Pioglitazone	15 mg daily	Metformin	6		Not reported
**Omarigliptin Protocol 020**	NCT01703221	2017	Japan	DPP-4 inhibitors	166	Omarigliptin	25 mg daily	None	5.5		MSD KK
**Omarigliptin Protocol 020**	NCT01703221	2017	Japan	DPP-4 inhibitors	165	Sitagliptin	50 mg daily	None	5.5		MSD KK
**Omarigliptin Protocol 020**	NCT01703221	2017	Japan	DPP-4 inhibitors	331	Omarigliptin/sitaglipin	25 mg/50 mg daily	None	5.5		MSD KK
**Omarigliptin Protocol 020**	NCT01703221	2017	Japan	Standard treatments	83	Placebo		None	5.5		MSD KK

Study ID	Registration	Year	Country	Intervention	N	Drug	Dose	Cointervention	Follow -up*	Settings	Fundings
**Omariglitin Study 015**	NCT01697592	2017	Japan	DPP-4 inhibitors	389	Omarigliptin	25 mg daily	Stable dose of an sulfonylurea, glinide, biguanide, thiazolidinedione, or alpha glucosidase inhibitor or dual treatment or additional other treatment depnding on HbA1C	5.5		MSD KK
**Omariglitin Study 015**	NCT01697592	2017	Japan	Standard treatments	196	Placebo		Stable dose of an sulfonylurea, glinide, biguanide, thiazolidinedione, or alpha glucosidase inhibitor or dual treatment or additional other treatment depnding on HbA1C	5.5		MSD KK
**Onoue 2020**	UMIN000014531	2019	Japan	DPP-4 inhibitors	12	Anagliptin	400 mg daily	No or stable glucose lowering therapy	5.5		Not reported

Study ID	Registration	Year	Country	Intervention	N	Drug	Dose	Cointervention	Follow -up*	Settings	Fundings
**Onoue 2020**	UMIN000014531	2019	Japan	Standard treatments	12	Control		No or stable glucose lowering therapy	5.5		Not reported
**Onuchin 2010_1**	None	2010	Russia	Basal insulin	45	Long acting Insulin	Titrated	None	12		Not reported
**Onuchin 2010_1**	None	2010	Russia	Metformin	46	Metformin	2500-5000 mg daily	None	12		Not reported
**Onuchin 2010_2**	None	2010	Russia	Basal insulin	44	Long-acting insulins	Titrated	Metformin	12		Not reported
**Onuchin 2010_2**	None	2010	Russia	Sulfonylureas	47	Glicliazide	30-90 mg daily	Metformin	12		Not reported
**Osman 2004**	Not applicable	2004	USA	Standard treatments	8	Placebo		Treatment not otherwise specified	6		NIH
**Osman 2004**	Not applicable	2004	USA	Thiazolidined iones	8	Rosiglitazone	8mg daily	Treatment not otherwise specified	6	Coronary artery disease	NIH
**Ovalle 2004**	Not applicable	2004	USA	Basal-Bolus insulin	8	30/70 Mixed Insulin	Titrated	Sulphonylurea; Metformin	6		GlaxoSmith-Kline
**Ovalle 2004**	Not applicable	2004	USA	Thiazolidined iones	9	Rosiglitazone	8 mg daily	Sulphonylurea; Metformin	6		GlaxoSmith-Kline
**Owens 2011**	NCT00602472	2011	Multinational (Argentina, Belgium, Canada, China, Germany,	DPP-4 inhibitors	793	Linagliptin	5 mg daily	Metformin + sulfonylurea	5.5		Boehringer Ingelheim

Study ID	Registration	Year	Country	Intervention	N	Drug	Dose	Cointervention	Follow -up*	Settings	Fundings
			Korea, Philippines, Russia, Taiwan, Turkey, UK)								
**Owens 2011**	NCT00602472	2011	Multinational (Argentina, Belgium, Canada, China, Germany, Korea, Philippines, Russia, Taiwan, Turkey, UK)	Standard treatments	265	Placebo		Metformin + sulfonylurea	5.5		Boehringer Ingelheim
**Oyama 2008**	Not provided	2008	Japan	Alpha-glucosidase inhibitors	41	Acarbose	300 mg daily	Glibenclamide	12		Not reported
**Oyama 2008**	Not provided	2008	Japan	Standard treatments	43	Control		Glibenclamide	12		Not reported
**Pagano 1995**	Not applicable	1995	Italy	Alpha-glucosidase inhibitors	49	Miglitol	300 mg daily	Metformin	6		Bayer SpA
**Pagano 1995**	Not applicable	1995	Italy	Sulfonylureas	47	Glibenclamide	5 mg daily	Metformin	6		Bayer SpA

Study ID	Registration	Year	Country	Intervention	N	Drug	Dose	Cointervention	Follow-up*	Settings	Fundings
**Pan 2008**	NCT00110240	2008	Multinational (Argentina, Belgium, Canada, China, Germany, Korea, Philippines, Russia, Taiwan, Turkey, UK)	Alpha-glucosidase inhibitors	220	Acarbose	300 mg daily	None	5.5		Novartis
**Pan 2008**	NCT00110240	2008	Multinational (Argentina, Belgium, Canada, China, Germany, Korea, Philippines, Russia, Taiwan, Turkey, UK)	DPP-4 inhibitors	441	Vildagliptin	100 mg daily	None	5.5		Novartis
**Pan 2012**	Not provided	2012	China	DPP-4 inhibitors	146	Vildagliptin	100 mg daily	Metformin	5.5		Novartis

Study ID	Registration	Year	Country	Intervention	N	Drug	Dose	Cointervention	Follow-up*	Settings	Fundings
**Pan 2012**	Not provided	2012	China	DPP-4 inhibitors	148	Vildagliptin	50 mg daily	Metformin	5.5		Novartis
**Pan 2012**	Not provided	2012	China	DPP-4 inhibitors	294	Vildagliptin	50-100 mg daily	Metformin	5.5		Novartis
**Pan 2012**	Not provided	2012	China	Standard treatments	144	Placebo		Metformin	5.5		Novartis
**Pan 2012a**	NCT00698932	2012	Multinational	DPP-4 inhibitors	284	Saxagliptin	5 mg daily	None	5.5		AstraZenca; Bristol-Myers Squibb
**Pan 2012a**	NCT00698932	2012	Multinational	Standard treatments	284	Placebo		None	5.5		AstraZenca; Bristol-Myers Squibb
**Park 2011**	Not provided	2011	Korea	Metformin	34	Metformin	1000 mg daiy	Sulfonylurea	5.5		Yonsei University College of Medicine
**Park 2011**	Not provided	2011	Korea	Thiazolidinediones	34	Pioglitazone	15 mg daily	Sulfonylurea	5.5		Yonsei University College of Medicine
**Park 2014**	NCT00708578	2014	Republic of Korea	Metformin	33	Metformin	1500 mg daily	Oral antidiabetic drugs	5.5		Sanofi-Korea
**Park 2014**	NCT00708578	2014	Republic of Korea	Sulfonylureas	34	Glimepiride	4 mg daily	Oral antidiabetic drugs	5.5		Sanofi-Korea
**Park 2017**	NCT02946541	2017	Republic of Korea	DPP-4 inhibitors	80	Evogliptin	5 mg daily	None	5.5		Dong-A ST Co
**Park 2017**	NCT02946541	2017	Republic of Korea	Standard treatments	80	Placebo		None	5.5		Dong-A ST Co
**Parmar Vinendra 2019**	Not provided	2019	India	DPP-4 inhibitors	65	Tenegliptin	20 mg daily	Metformin + glimepiride	5.5		None reported

Study ID	Registration	Year	Country	Intervention	N	Drug	Dose	Cointervention	Follow -up*	Settings	Fundings
**Parmar Vinendra 2019**	Not provided	2019	India	Standard treatments	65	Control		Metformin + glimepiride	5.5		None reported
**Parthan 2018**	NCT02097342	2018	India	Alpha-glucosidase inhibitors	10	Voglibose	0.6 mg daily	Metformin	6		None reported
**Parthan 2018**	NCT02097342	2018	India	DPP-4 inhibitors	10	Linagliptin	5 mg daily	Metformin	6		None reported
**Parthan 2018**	NCT02097342	2018	India	Standard treatments	10	Control		Metformin	6		None reported
**Pasquel-2021**	NCT01919489	2021	US	Basal insulin	147	Insulin glargine		diabetes regimen included diet and/or OADs including sulphonylureas, repaglinide, nateglinide, or metformin, either as monotherapy or in combination therapy, or taking a low total daily dose of insulin (≤0.4 unit/kg/d)	6		The study was an investigator-initiated study funded by Novo Nordisk

Study ID	Registration	Year	Country	Intervention	N	Drug	Dose	Cointervention	Follow -up*	Settings	Fundings
**Pasquel-2021**	NCT01919489	2021	US	GLP-1 receptor agonists	140	Liraglutide	1.8 mg once daily	diabetes regimen included diet and/or OADs including sulphonylureas, repaglinide, nateglinide, or metformin, either as monotherapy or in combination therapy, or taking a low total daily dose of insulin (≤0.4 unit/kg/d)	6	at the time of hospital discharge	The study was an investigator-initiated study funded by Novo Nordisk
**Patel 2013**	Not provided	2013	USA	Alpha-glucosidase inhibitors	109	Acarbose		None	60		Bayer; NIH
**Patel 2013**	Not provided	2013	USA	Standard treatments	110	Placebo		None	60		Bayer; NIH
**Pavithra 2019**	Not provided	2019	India	DPP-4 inhibitors	43	Sitagliptin	50 mg daily	Metformin	6		Not reported
**Pavithra 2019**	Not provided	2019	India	GLP-1 receptor agonists	37	Liraglutide	1.8 mg daily	Metformin	6		Not reported

Study ID	Registration	Year	Country	Intervention	N	Drug	Dose	Cointervention	Follow-up*	Settings	Fundings
**Pavo 2003**	Not applicable	2003	Multinational	Metformin	100	Metformin	2550 mg daily	None	7.5		Eli Lilly and Company
**Pavo 2003**	Not applicable	2003	Multinational	Thiazolidinediones	106	Pioglitazone	45 mg daily	None	7.5		Eli Lilly and Company
**Perez 2009**	NCT00727857	2009	Multinational	Metformin	210	Metformin	1500 mg daily	None	5.5		Takeda Global Research & Development Center, Inc.
**Perez 2009**	NCT00727857	2009	Multinational	Thiazolidinediones	189	Pioglitazone	30 mg daily	None	5.5		Takeda Global Research & Development Center, Inc.
**PERISCOPE**	NCT00225277	2008	Multinational	Sulfonylureas	273	Glimepiride	1-4 mg daily	1-2 oral drugs excluding thiazolidinedione	18	Coronary artery disease	Takeda
**PERISCOPE**	NCT00225277	2008	Multinational	Thiazolidinediones	274	Pioglitazone	15-45 mg daily	1-2 oral drugs excluding thiazolidinedione	18		Takeda
**Perriello 2006**	Not provided	2006	Italy	Sulfonylureas	137	Gliclazide	320 mg daily	None	12		Takeda Italy
**Perriello 2006**	Not provided	2006	Italy	Thiazolidinediones	146	Pioglitazone	45 mg daily	None	12		Takeda Italy
**Petrica 2009**	Not provided	2009	Romania	Sulfonylureas	22	Glimepiride	4 mg daily	Metformin	6		Not reported
**Petrica 2009**	Not provided	2009	Romania	Thiazolidinediones	22	Rosiglitazone	4 mg daily	Metformin	6		Not reported

Study ID	Registration	Year	Country	Intervention	N	Drug	Dose	Cointervention	Follow -up*	Settings	Fundings
**Petrica 2011**	Not provided	2011	Romania	Sulfonylureas	39	Glimepiride	4 mg daily	Oral antidiabetic drugs	12		NR
**Petrica 2011**	Not provided	2011	Romania	Thiazolidinediones	39	Pioglitazone	30 mg daily	Oral antidiabetic drugs	12		NR
**Philis-Tsimikas 2013**	NCT01046110	2013	Multinational	Basal insulin	229	Insulin degludev	Titrated	1-2 oral drugs including metformin, sulfonylureas, glinide or pioglitazone	6		Novo Nordisk A/S,
**Philis-Tsimikas 2013**	NCT01046110	2013	Multinational	DPP-4 inhibitors	229	Sitagliptin	100 mg daily	1-2 oral drugs including metformin, sulfonylureas, glinide or pioglitazone	6		Novo Nordisk A/S,
**Phillips 2001**	Not applicable	2001	USA	Standard treatments	173	Placebo		None	5.5		SmithKline Beecham Pharmaceuticals
**Phillips 2001**	Not applicable	2001	USA	Thiazolidinediones	187	Rosiglitazone	8 mg daily	None	5.5		SmithKline Beecham Pharmaceuticals
**Phillips 2001**	Not applicable	2001	USA	Thiazolidinediones	181	Rosiglitazone	8 mg daily	None	5.5		SmithKline Beecham Pharmaceuticals
**Phillips 2001**	Not applicable	2001	USA	Thiazolidinediones	186	Rosiglitazone	4 mg daily	None	5.5		SmithKline Beecham Pharmaceuticals

Study ID	Registration	Year	Country	Intervention	N	Drug	Dose	Cointervention	Follow -up*	Settings	Fundings
**Phillips 2001**	Not applicable	2001	USA	Thiazolidinediones	181	Rosiglitazone	4 mg daily	None	5.5		SmithKline Beecham Pharmaceuticals
**Phillips 2001**	Not applicable	2001	USA	Thiazolidinediones	735	Rosiglitazone	4-8 mg daily	None	5.5		SmithKline Beecham Pharmaceuticals
**Phillips 2003**	Not applicable	2003	Multinational	Alpha-glucosidase inhibitors	40	Acarbose	300 mg daily	Metformin	5.5		Bayer
**Phillips 2003**	Not applicable	2003	Multinational	Standard treatments	43	Placebo		Metformin	5.5		Bayer
**Phrommintikul 2019**	NCT03178591	2019	Thailand	DPP-4 inhibitors	24	Vildagliptin	100 mg daily	Metformin and or sulfonylurea and or thiazoledinedione	6	Coronary artery disease	Thailand Research Fund
**Phrommintikul 2019**	NCT03178591	2019	Thailand	SGLT2 inhibitors	25	Dapagliflozin	10 mg daily	Metformin and or sulfonylurea and or thiazoledinedione	6		Thailand Research Fund
**Pi-Sunyer 2007**	NCT00120536	2007	Multinational	DPP-4 inhibitors	91	Vildagliptin	100 mg daily	None	5.5		Novartis Pharmaceuticals
**Pi-Sunyer 2007**	NCT00120536	2007	Multinational	DPP-4 inhibitors	83	Vildagliptin	100 mg daily	None	5.5		Novartis Pharmaceuticals
**Pi-Sunyer 2007**	NCT00120536	2007	Multinational	DPP-4 inhibitors	88	Vildagliptin	50 mg daily	None	5.5		Novartis Pharmaceuticals
**Pi-Sunyer 2007**	NCT00120536	2007	Multinational	DPP-4 inhibitors	262	Vildagliptin	50-100 mg daily	None	5.5		Novartis Pharmaceuticals
**Pi-Sunyer 2007**	NCT00120536	2007	Multinational	Standard treatments	92	Placebo		None	5.5		Novartis Pharmaceuticals

Study ID	Registration	Year	Country	Intervention	N	Drug	Dose	Cointervention	Follow-up*	Settings	Fundings
**PIOCOMB**	Not provided	2011	Germany	Metformin	40	Metformin	1700 mg daily	Insulin glargine	6		Takeda Pharma
**PIOCOMB**	Not provided	2011	Germany	Thiazolidinediones	42	Pioglitazone	30 mg daily	Insulin glargine	6		Takeda Pharma
**PIOfix**	NCT00770653	2011	Germany	Sulfonylureas	150	Glimepiride	4 mg daily	Metformin	5.5		Takeda Pharma, Germany
**PIOfix**	NCT00770653	2011	Germany	Thiazolidinediones	155	Pioglitazone	30 mg daily	Metformin	5.5		Takeda Pharma, Germany
**Pioglitazone 001**	Not applicable	2000	USA	Standard treatments	79	Placebo		None	6		Takeda America
**Pioglitazone 001**	Not applicable	2000	USA	Thiazolidinediones	76	Pioglitazone	45 mg daily	None	6		Takeda America
**Pioglitazone 001**	Not applicable	2000	USA	Thiazolidinediones	85	Pioglitazone	30 mg daily	None	6		Takeda America
**Pioglitazone 001**	Not applicable	2000	USA	Thiazolidinediones	79	Pioglitazone	15 mg daily	None	6		Takeda America
**Pioglitazone 001**	Not applicable	2000	USA	Thiazolidinediones	80	Pioglitazone	7.5 mg daily	None	6		Takeda America
**Pioglitazone 001**	Not applicable	2000	USA	Thiazolidinediones	320	Pioglitazone	7.5-40 mg daily	None	6		Takeda America
**PIONEER**	Not applicable	2005	Germany	Sulfonylureas	84	Glimepiride	1-6 mg daily	Excluding thiazolidinedione treatment	6		Takeda Pharma

Study ID	Registration	Year	Country	Intervention	N	Drug	Dose	Cointervention	Follow-up*	Settings	Fundings
**PIONEER**	Not applicable	2005	Germany	Thiazolidinediones	89	Pioglitazone	45 mg daily	Excluding thiazolidinedione treatment	6		Takeda Pharma
**PIONEER 1**	NCT02906930	2019	Multinational	GLP-1 receptor agonists	175	Semaglutide	14 mg daily	None	5.5		Novo Nordisk
**PIONEER 1**	NCT02906930	2019	Multinational	GLP-1 receptor agonists	175	Semaglutide	7 mg daily	None	5.5		Novo Nordisk
**PIONEER 1**	NCT02906930	2019	Multinational	GLP-1 receptor agonists	175	Semaglutide	3 mg daily	None	5.5		Novo Nordisk
**PIONEER 1**	NCT02906930	2019	Multinational	GLP-1 receptor agonists	525	Semaglutide	3-14 mg daily	None	5.5		Novo Nordisk
**PIONEER 1**	NCT02906930	2019	Multinational	Standard treatments	178	Placebo		None	5.5		Novo Nordisk
**PIONEER 2**	NCT02863328	2019	Multinational	GLP-1 receptor agonists	412	Semaglutide	14 mg daily	Metformin	12		Novo Nordisk
**PIONEER 2**	NCT02863328	2019	Multinational	SGLT2 inhibitors	410	Empagliflozin	25 mg daily	Metformin	12		Novo Nordisk
**PIONEER 3**	NCT02607865	2019	Multinational	DPP-4 inhibitors	467	Sitagliptin	100 mg daily	Metformin with or without sulfonylurea	18		Novo Nordisk

Study ID	Registration	Year	Country	Intervention	N	Drug	Dose	Cointervention	Follow -up*	Settings	Fundings
**PIONEER 3**	NCT02607865	2019	Multinational	GLP-1 receptor agonists	465	Semaglutide	14 mg daily	Metformin with or without sulfonylurea	18		Novo Nordisk
**PIONEER 3**	NCT02607865	2019	Multinational	GLP-1 receptor agonists	466	Semaglutide	7 mg daily	Metformin with or without sulfonylurea	18		Novo Nordisk
**PIONEER 3**	NCT02607865	2019	Multinational	GLP-1 receptor agonists	466	Semaglutide	3 mg daily	Metformin with or without sulfonylurea	18		Novo Nordisk
**PIONEER 3**	NCT02607865	2019	Multinational	GLP-1 receptor agonists	1397	Semaglutide	3-14 mg daily	Metformin with or without sulfonylurea	18		Novo Nordisk
**PIONEER 4**	NCT02863419	2019	Multinational	GLP-1 receptor agonists	285	Semaglutide	14 mg daily	Metformin with or without SGLT-2 inhibitor	12		Novo Nordisk
**PIONEER 4**	NCT02863419	2019	Multinational	GLP-1 receptor agonists	284	Liraglutide	1.8 mg daily	Metformin with or without SGLT-2 inhibitor	12		Novo Nordisk
**PIONEER 4**	NCT02863419	2019	Multinational	GLP-1 receptor agonists	569	Semaglutide/liraglutide	14 mg daily/1.8 mg daily	Metformin with or without SGLT-2 inhibitor	12		Novo Nordisk
**PIONEER 4**	NCT02863419	2019	Multinational	Standard treatments	142	Placebo		Metformin with or without SGLT-2 inhibitor	12		Novo Nordisk

Study ID	Registration	Year	Country	Intervention	N	Drug	Dose	Cointervention	Follow -up*	Settings	Fundings
**PIONEER 5**	NCT02827708	2019	Multinational	GLP-1 receptor agonists	163	Semaglutide	14 mg daily	Metformin, a sulfonylurea, or both; or basal insulin with or without metformin	6		Novo Nordisk
**PIONEER 5**	NCT02827708	2019	Multinational	Standard treatments	161	Placebo		Metformin, a sulfonylurea, or both; or basal insulin with or without metformin	6		Novo Nordisk
**PIONEER 6**	NCT02692716	2019	Multinational	GLP-1 receptor agonists	1591	Semaglutide	14 mg daily	Standard of care	15.9		Novo Nordisk
**PIONEER 6**	NCT02692716	2019	Multinational	Standard treatments	1592	Placebo		Standard of care	15.9		Novo Nordisk
**PIONEER 7**	NCT02849080	2019	Multinational	DPP-4 inhibitors	251	Sitagliptin	100 mg daily	One or two glucose-lowering drugs (metformin, sulphonylureas, sodium glucose co-transporter-2 [SGLT2] inhibitors, or thiazolidinediones)	12		Novo Nordisk

Study ID	Registration	Year	Country	Intervention	N	Drug	Dose	Cointervention	Follow -up*	Settings	Fundings
**PIONEER 7**	NCT02849080	2019	Multinational	GLP-1 receptor agonists	253	Semaglutide	14 mg daily	One or two glucose-lowering drugs (metformin, sulphonylureas, sodium glucose co-transporter-2 [SGLT2] inhibitors, or thiazolidinediones)	12		Novo Nordisk
**PIONEER 8**	NCT03021187	2019	Multinational	GLP-1 receptor agonists	181	Semaglutide	14 mg daily	Insulin with or without metformin	12		Novo Nordisk
**PIONEER 8**	NCT03021187	2019	Multinational	GLP-1 receptor agonists	182	Semaglutide	7 mg daily	Insulin with or without metformin	12		Novo Nordisk
**PIONEER 8**	NCT03021187	2019	Multinational	GLP-1 receptor agonists	184	Semaglutide	3 mg daily	Insulin with or without metformin	12		Novo Nordisk
**PIONEER 8**	NCT03021187	2019	Multinational	GLP-1 receptor agonists	547	Semaglutide	3-14 mg daily	Insulin with or without metformin	12		Novo Nordisk
**PIONEER 8**	NCT03021187	2019	Multinational	Standard treatments	184	Placebo		Insulin with or without metformin	12		Novo Nordisk

Study ID	Registration	Year	Country	Intervention	N	Drug	Dose	Cointervention	Follow -up*	Settings	Fundings
**PIONEER 9**	NCT03018028	2020	Japan	GLP-1 receptor agonists	48	Semaglutide	14 mg daily	Oral drug monotherapy or no therapy	6		Novo Nordisk
**PIONEER 9**	NCT03018028	2020	Japan	GLP-1 receptor agonists	48	Semaglutide	7 mg daily	Oral drug monotherapy or no therapy	6		Novo Nordisk
**PIONEER 9**	NCT03018028	2020	Japan	GLP-1 receptor agonists	49	Semaglutide	3 mg daily	Oral drug monotherapy or no therapy	6		Novo Nordisk
**PIONEER 9**	NCT03018028	2020	Japan	GLP-1 receptor agonists	49	Liraglutide	0.9 mg daily	Oral drug monotherapy or no therapy	6		Novo Nordisk
**PIONEER 9**	NCT03018028	2020	Japan	GLP-1 receptor agonists	194	Semaglutide 3-14 mg/Liraglutide 0.9 mg		Oral drug monotherapy or no therapy	6		Novo Nordisk
**PIONEER 9**	NCT03018028	2020	Japan	Standard treatments	49	Placebo		Oral drug monotherapy or no therapy	6		Novo Nordisk
**PioRAGE**	UMIN000002055	2014	Japan	Sulfonylureas	32	Glimepiride	0.5-2 mg daily	Sulfonylurea or glinide or no treatment	6		Grant-in-Aid for Scientific Research; Sanofi; Takeda
**PioRAGE**	UMIN000002055	2014	Japan	Thiazolidinediones	31	Pioglitazone	15-30 mg daily	Sulfonylurea or glinide or no treatment	6		Grant-in-Aid for Scientific Research; Sanofi; Takeda

Study ID	Registration	Year	Country	Intervention	N	Drug	Dose	Cointervention	Follow -up*	Settings	Fundings
**PIRAMID**	ISRCTN53177482	2009	Netherlands	Metformin	39	Metformin	500-1000 mg daily	Glimepiride	5.5		Eli Lilly
**PIRAMID**	ISRCTN53177482	2009	Netherlands	Thiazolidined iones	39	Pioglitazone	15-30 mg daily	Glimepiride	5.5		Eli Lilly
**Pistrosch 2012**	NCT00324675	2012	Germany	Standard treatments	14	Placebo		Insulin sulfonylurea glinide alpha - glucosidase inhibitor therapy	12		GlaxoSmithKline
**Pistrosch 2012**	NCT00324675	2012	Germany	Thiazolidined iones	14	Rosiglitazone	8 mg daily	Insulin sulfonylurea glinide alpha - glucosidase inhibitor therapy	12	Chronic kidney disease	GlaxoSmithKline
**Pistrosch 2013**	NCT00857870	2013	Germany	Basal insulin	39	Insulin glargine	Titrated	None	8.4		Sanofi-Aventis
**Pistrosch 2013**	NCT00857870	2013	Germany	Metformin	36	Metformin	2000 mg daily	None	8.4		Sanofi-Aventis
**Pop-Busui 2009**	NCT00549874	2009	USA	Sulfonylureas	13	Glyburide	10 mg daily	Sulfonylurea	6		GlaxoSmithKline
**Pop-Busui 2009**	NCT00549874	2009	USA	Thiazolidined iones	14	Rosiglitazone	8 mg daily	Sulfonylurea	6		GlaxoSmithKline
**POPPS**	Not provided	2009	Japan	Standard treatments	49	Control		Excluding pioglitazone	6		Not reported
**POPPS**	Not provided	2009	Japan	Thiazolidined iones	48	Pioglitazone	30 mg daily	Excluding pioglitazone	6	Coronary artery disease	Not reported
**Pratley 2014**	NCT01023581	2014	Multinational	DPP-4 inhibitors	112	Alogliptin	25 mg daily	None	6		Takeda Development Center

Study ID	Registration	Year	Country	Intervention	N	Drug	Dose	Cointervention	Follow -up*	Settings	Fundings
**Pratley 2014**	NCT01023581	2014	Multinational	DPP-4 inhibitors	113	Alogliptin	25 mg daily	None	6		Takeda Development Center
**Pratley 2014**	NCT01023581	2014	Multinational	DPP-4 inhibitors	225	Alogliptin	25 mg daily	None	6		Takeda Development Center
**Pratley 2014**	NCT01023581	2014	Multinational	Metformin	111	Metformin	2000 mg daily	None	6		Takeda Development Center
**Pratley 2014**	NCT01023581	2014	Multinational	Metformin	114	Metformin	1000 mg daily	None	6		Takeda Development Center
**Pratley 2014**	NCT01023581	2014	Multinational	Metformin	225	Metformin	1000-2000 mg daily	None	6		Takeda Development Center
**Pratley 2014**	NCT01023581	2014	Multinational	Standard treatments	109	Placebo		None	6		Takeda Development Center
**PRESERVE-beta**	Not applicable	2005	USA	Meglitinides	219	Nateglinide	360 mg daily	Metformin	24		Novartis
**PRESERVE-beta**	Not applicable	2005	USA	Sulfonylureas	209	Glyburide	1.25-10 mg daily	Metformin	24		Novartis
**PREVENT-J**	UMIN000021552	2018	Japan	Standard treatments	84	Control		None, metformin or alpha glucosidase inhibitor	18		Japan Cardiovascular Research Foundation
**PREVENT-J**	UMIN000021552	2018	Japan	Sulfonylureas	93	Sulfonylurea (Gliclazide, glimepiride, tolbutamide)		None, metformin or alpha glucosidase inhibitor	18		Japan Cardiovascular Research Foundation

Study ID	Registration	Year	Country	Intervention	N	Drug	Dose	Cointervention	Follow -up*	Settings	Fundings
**PREVENT-J**	UMIN000021552	2018	Japan	Thiazolidined iones	101	Pioglitazone	30-45 mg daily	None, metformin or alpha glucosidase inhibitor	18		Japan Cardiovascular Research Foundation
**PRIDE**	Not provided	2018	Japan	Standard treatments	46	Control		Oral hypoglycemic agents	6		JSPS KAKENHI
**PRIDE**	Not provided	2018	Japan	Thiazolidined iones	48	Pioglitazone	30 mg daily	Oral hypoglycemic agents	6	Coronary artery disease	JSPS KAKENHI
**PRIME-V**	UMIN000015170	2019	Japan	Metformin	50	Metformin	1000-1500 mg daily	Sitagliptin	5.5		Astellas Pharma
**PRIME-V**	UMIN000015170	2019	Japan	SGLT2 inhibitors	48	Ipragliflozin	50 mg daily	Sitagliptin	5.5		Astellas Pharma
**PRISMA**	NCT00772174	2013	Italy	Standard treatments	103	Placebo		Metformin	5.5		Takeda Italia
**PRISMA**	NCT00772174	2013	Italy	Thiazolidined iones	110	Pioglitazone	45 mg daily	Metformin	5.5		Takeda Italia
**PROactive**	NCT00174993	2005	Multinational	Standard treatments	2633	Placebo		Oral agents insulin	34.5		Takeda Pharmaceuticals; Eli Lilly
**PROactive**	NCT00174993	2005	Multinational	Thiazolidined iones	2605	Pioglitazone	15-45 mg daily	Oral agents insulin	34.5	Cardiovascular disease	Takeda Pharmaceuticals; Eli Lilly
**PROLOGUE**	UMIN000004490	2016	Japan	DPP-4 inhibitors	232	Sitagliptin	100 mg daily	Antidiabetic drugs	24		Clinical Research Promotion Foundation

Study ID	Registration	Year	Country	Intervention	N	Drug	Dose	Cointervention	Follow -up*	Settings	Fundings
**PROLOGUE**	UMIN000004490	2016	Japan	Standard treatments	231	Placebo		Antidiabetic drugs	24		Clinical Research Promotion Foundation
**QUARTER**	Not applicable	2004	Europe	Metformin	597	Metformin	2550 mg daily	None	12		Not reported
**QUARTER**	Not applicable	2004	Europe	Thiazolidinediones	597	Pioglitazone	45 mg daily	None	12		Not reported
**Quatraro 1986**	Not applicable	1986	Italy	Standard treatments	15	Control		Insulin	12		Not reported
**Quatraro 1986**	Not applicable	1986	Italy	Sulfonylureas	15	Glicazide	40-240 mg daily	Insulin	12		Not reported
**Raghuveer 2020**	Not provided	2020	India	DPP-4 inhibitors	50	Tenegliptin	20 mg daily	Metformin	12		Not reported
**Raghuveer 2020**	Not provided	2020	India	Standard treatments	50	Control		Metformin	12		Not reported
**Rahman 2010**	NCT00489229	2010	Malaysia	Standard treatments	11	Placebo		None	12		Ministry of Science and Technology and Environment, the Government of Malaysia
**Rahman 2010**	NCT00489229	2010	Malaysia	Thiazolidinediones	11	Rosiglitazone	4 mg daily	None	12		Ministry of Science and Technology and Environment, the

Study ID	Registration	Year	Country	Intervention	N	Drug	Dose	Cointervention	Follow-up*	Settings	Fundings
											Government of Malaysia
**Rahman 2011**	Not provided	2011	Pakistan	Metformin	102	Metformin	2000 mg daily	None	12		Not reported
**Rahman 2011**	Not provided	2011	Pakistan	Sulfonylureas	102	Glimepiride	8 mg daily	None	12		Not reported
**Raskin 2001**	Not applicable	2001	USA	Standard treatments	107	Placebo		Insulin	6		SmithKline Beecham Pharmaceuticals
**Raskin 2001**	Not applicable	2001	USA	Thiazolidinediones	105	Rosiglitazone	8 mg daily	Insulin	6		SmithKline Beecham Pharmaceuticals
**Raskin 2001**	Not applicable	2001	USA	Thiazolidinediones	107	Rosiglitazone	4 mg daily	Insulin	6		SmithKline Beecham Pharmaceuticals
**Raskin 2001**	Not applicable	2001	USA	Thiazolidinediones	212	Rosiglitazone	4-8 mg daily	Insulin	6		SmithKline Beecham Pharmaceuticals
**Raskin 2004**	Not applicable	2004	USA	Meglitinides	63	Repaglinide	12 mg daily	None	5.5		Novo Nordisk
**Raskin 2004**	Not applicable	2004	USA	Thiazolidinediones	62	Rosiglitazone	8 mg daily	None	5.5		Novo Nordisk
**Raskin 2009**	Not provided	2009	USA	Meglitinides	187	Repaglinide	10 mg daily	Oral agents	6		Novo Nordisk
**Raskin 2009**	Not provided	2009	USA	Meglitinides	187	Repaglinide	8 mg daily	Oral agents	6		Novo Nordisk
**Raskin 2009**	Not provided	2009	USA	Meglitinides	374	Repaglinide	8-10 mg daily	Oral agents	6		Novo Nordisk
**Raskin 2009**	Not provided	2009	USA	Thiazolidinediones	187	Rosiglitazone	4 mg daily	Oral agents	6		Novo Nordisk

Study ID	Registration	Year	Country	Intervention	N	Drug	Dose	Cointervention	Follow -up*	Settings	Fundings
**Raz 2008**	NCT00337610	2008	Multinational	DPP-4 inhibitors	96	Sitagliptin	100 mg daily	Metformin oral monotherapy	6.9		Merck & Co
**Raz 2008**	NCT00337610	2008	Multinational	Standard treatments	94	Placebo		Metformin oral monotherapy	6.9		Merck & Co
**RECORD**	NCT00379769	2009	Multinational	Standard treatments	2227	Standard care		Metformin	66		GlaxoSmithKline
**RECORD**	NCT00379769	2009	Multinational	Thiazolidinediones	2220	Rosiglitazone	8 mg daily	Metformin	66		GlaxoSmithKline
**REFORM**	NCT02397421	2020	United Kingdom	SGLT2 inhibitors	28	Dapagliflozin	10 mg daily	Stable therapy	12	Heart failure	European Foundation for the Study of Diabetes, Clinical Diabetes Research Programme in Macrovascular Complications of Diabetes.
**REFORM**	NCT02397421	2020	United Kingdom	Standard treatments	28	Placebo		Stable therapy	12		European Foundation for the Study of Diabetes, Clinical Diabetes Research Programme in Macrovascular Complications of Diabetes.

Study ID	Registration	Year	Country	Intervention	N	Drug	Dose	Cointervention	Follow -up*	Settings	Fundings
**REGO-F**	UMIN000018321	2020	Japan	Meglitinides	36	Repaglinide	0.75 mg daily	Antidiabetic agents	11		Sumitomo Dainippon Pharma.
**REGO-F**	UMIN000018321	2020	Japan	Sulfonylureas	36	Glimepiride	1 mg daily	Antidiabetic agents	11		Sumitomo Dainippon Pharma.
**RELEASE**	NCT02015299	2017	The Netherlands	DPP-4 inhibitors	22	Linagliptin	5 mg daily	None	6		Boehringer Ingelheim
**RELEASE**	NCT02015299	2017	The Netherlands	Standard treatments	22	Placebo		None	6		Boehringer Ingelheim
**RESET-IT Main**	NCT02192424	2021	Canada	Basal-Bolus insulin	55	Intermittent intensive insulin therapy (basal insulin glargine at bedtime and premeal lispro)	ITT: 2 weeks every 3 months (during which metformin was stopped)	Metformin	24		Canadian Institutes of Health Research, Grant/Award Number: MOP 133701
**RESET-IT Main**	NCT02192424	2021	Canada	Standard treatments	53	Control		Metformin	24		Canadian Institutes of Health Research, Grant/Award Number: MOP 133701
**RESULT**	Not provided	2006	USA, Canada	Standard treatments	111	Placebo		Sulfonylurea	24		GlaxoSmithKline

Study ID	Registration	Year	Country	Intervention	N	Drug	Dose	Cointervention	Follow-up*	Settings	Fundings
**RESULT**	Not provided	2006	USA, Canada	Thiazolidinediones	116	Rosiglitazone	4-8 mg daily	Sulfonylurea	24		GlaxoSmithKline
**REWIND**	NCT01394952	2019	Multinational	GLP-1 receptor agonists	4949	Dulaglutide	1.5 mg weekly	Antihyperglycemic therapy except DPP-4 inhibitor or GLP-1 receptor agonist	64.8		Eli Lilly and Company
**REWIND**	NCT01394952	2019	Multinational	Standard treatments	4952	Placebo		Antihyperglycemic therapy except DPP-4 inhibitor or GLP-1 receptor agonist	64.8		Eli Lilly and Company
**Reynolds 2002**	Not applicable	2002	USA	Standard treatments	10	Placebo		None	5.5		GlaxoSmithKline
**Reynolds 2002**	Not applicable	2002	USA	Thiazolidinediones	8	Rosiglitazone	4 mg daily	None	5.5		GlaxoSmithKline
**Reynolds 2007**	Not provided	2007	USA	Basal insulin	20	Insulin glargine	Titrated	Metformin + sulfonylurea	5.5		GlaxoSmithKline
**Reynolds 2007**	Not provided	2007	USA	Thiazolidinediones	20	Rosiglitazone	4 mg daily	Metformin + sulfonylurea	5.5		GlaxoSmithKline
**Ristic 2006**	Not provided	2006	Multinational	Meglitinides	133	Nateglinide	540 mg daily	Metformin	5.5		Novartis Pharma
**Ristic 2006**	Not provided	2006	Multinational	Sulfonylureas	129	Gliclazide	240 mg daily	Metformin	5.5		Novartis Pharma

Study ID	Registration	Year	Country	Intervention	N	Drug	Dose	Cointervention	Follow -up*	Settings	Fundings
**Robbins 2007**	NCT00191464	2007	Multinational	Basal insulin	158	Insulin glargine	Titrated	Metformin sulfonylurea	5.5		Eli Lilly
**Robbins 2007**	NCT00191464	2007	Multinational	Basal-Bolus insulin	159	Insulin protamine/lispro	Titrated	Metformin sulfonylurea	5.5		Eli Lilly
**Roberts 2005**	Not applicable	2005	USA, Canada	Standard treatments	84	Placebo		Metformin + thiazolidinedione	7		Sanofi -Aventis
**Roberts 2005**	Not applicable	2005	USA, Canada	Sulfonylureas	84	Glimepiride	2-8 mg daily	Metformin + thiazolidinedione	7		Sanofi -Aventis
**Rodbard 2016**	Not provided	2016	Multinational	SGLT2 inhibitors	108	Canagliflozin	300 mg daily	Metformin and sitagliptin	6		Janssen Research and Development
**Rodbard 2016**	Not provided	2016	Multinational	Standard treatments	108	Placebo		Metformin and sitagliptin	6		Janssen Research and Development
**Rosenstock 1998**	Not applicable	1998	USA	Alpha-glucosidase inhibitors	74	Acarbose	150-300 mg daily	Metformin	5.5		Bayer Corporation
**Rosenstock 1998**	Not applicable	1998	USA	Standard treatments	74	Placebo		Metformin	5.5		Bayer Corporation
**Rosenstock 2006**	Not provided	2006	Multinational	Metformin	154	Metformin	500-2000 mg daily	None	7.4		GlaxoSmithKline
**Rosenstock 2006**	Not provided	2006	Multinational	Thiazolidinediones	159	Rosiglitazone	4-8 mg daily	None	7.4		GlaxoSmithKline
**Rosenstock 2007**	NCT00099918	2007	Multinational	DPP-4 inhibitors	519	Vildagliptin	100 mg daily	None	5.5		Novartis Pharma, USA and Switzerland
**Rosenstock 2007**	NCT00099918	2007	Multinational	Thiazolidinediones	267	Rosiglitazone	8 mg daily	None	5.5		Novartis Pharma, USA and Switzerland

Study ID	Registration	Year	Country	Intervention	N	Drug	Dose	Cointervention	Follow -up*	Settings	Fundings
**Rosenstock 2007a**	NCT00101803	2007	Multinational	DPP-4 inhibitors	154	Vildagliptin	100 mg daily	None	5.5		Novartis Pharmaceuticals Corporation
**Rosenstock 2007a**	NCT00101803	2007	Multinational	Thiazolidinediones	161	Pioglitazone	30 mg daily	None	5.5		Novartis Pharmaceuticals Corporation
**Rosenstock 2009**	NCT00286429	2009	Multinational	DPP-4 inhibitors	129	Alogliptin	25 mg daily	Insulin metformin	6		Takeda Pharmaceuticals
**Rosenstock 2009**	NCT00286429	2009	Multinational	DPP-4 inhibitors	131	Alogliptin	12.5 mg daily	Insulin metformin	6		Takeda Pharmaceuticals
**Rosenstock 2009**	NCT00286429	2009	Multinational	DPP-4 inhibitors	260	Alogliptin	12.5-25 mg daily	Insulin metformin	6		Takeda Pharmaceuticals
**Rosenstock 2009**	NCT00286429	2009	Multinational	Standard treatments	130	Placebo		Insulin metformin	6		Takeda Pharmaceuticals
**Rosenstock 2010**	NCT00395512	2010	Multinational	DPP-4 inhibitors	164	Alogliptin	25 mg daily	None	6		Takeda Pharmaceuticals
**Rosenstock 2010**	NCT00395512	2010	Multinational	Thiazolidinediones	163	Pioglitazone	30 mg daily	None	6		Takeda Pharmaceuticals
**Rosenstock 2012**	NCT00683878	2012	Argentina, Canada, India, Mexico, Peru, Philippines, Taiwan, USA	SGLT2 inhibitors	140	Dapagliflozin	10 mg daily	None or metformin or sulfonylurea or thiazolidinedione	11		Bristol-Myers Squibb and AstraZeneca

Study ID	Registration	Year	Country	Intervention	N	Drug	Dose	Cointervention	Follow -up*	Settings	Fundings
**Rosenstock 2012**	NCT00683878	2012	Argentina, Canada, India, Mexico, Peru, Philippines, Taiwan, USA	SGLT2 inhibitors	141	Dapagliflozin	5 mg daily	None or metformin or sulfonylurea or thiazolidinedione	11		Bristol-Myers Squibb and AstraZeneca
**Rosenstock 2012**	NCT00683878	2012	Argentina, Canada, India, Mexico, Peru, Philippines, Taiwan, USA	SGLT2 inhibitors	281	Dapagliflozin	5-10 mg daily	None or metformin or sulfonylurea or thiazolidinedione	11		Bristol-Myers Squibb and AstraZeneca
**Rosenstock 2012**	NCT00683878	2012	Argentina, Canada, India, Mexico, Peru, Philippines, Taiwan, USA	Standard treatments	139	Placebo		None or metformin or sulfonylurea or thiazolidinedione	11		Bristol-Myers Squibb and AstraZeneca
**Rosenstock 2013**	NCT00707993	2013	Multinational	DPP-4 inhibitors	222	Alogliptin	25 mg daily	None	12		Takeda Pharmaceuticals
**Rosenstock 2013**	NCT00707993	2013	Mutlinational	Sulfonylureas	219	Glipizide	5-10 mg daily	None	12		Takeda Pharmaceuticals
**Rosenstock 2015**	NCT01606007	2015	USA	DPP-4 inhibitors	176	Saxagliptin	5 mg daily	Metformin	5.5		Bristol-Myers Squibb and AstraZeneca

Study ID	Registration	Year	Country	Intervention	N	Drug	Dose	Cointervention	Follow -up*	Settings	Fundings
**Rosenstock 2015**	NCT01606007	2015	USA	SGLT2 inhibitors	179	Dapagliflozin	10 mg daily	Metformin	5.5		Bristol-Myers Squibb and AstraZeneca
**Rosenstock 2016**	NCT01809327	2016	Multinational	Metformin	237	Metformin	500-2000 mg daily	None	5.5		Janssen Research and Development
**Rosenstock 2016**	NCT01809327	2016	Multinational	SGLT2 inhibitors	238	Canagliflozin	300 mg daily	None	5.5		Janssen Research and Development
**Rosenstock 2016**	NCT01809327	2016	Multinational	SGLT2 inhibitors	237	Canagliflozin	100 mg daily	None	5.5		Janssen Research and Development
**Rosenstock 2016**	NCT01809327	2016	Multinational	SGLT2 inhibitors	475	Canagliflozin	100-300 mg daily	None	5.5		Janssen Research and Development
**Rosenstock 2019**	NCT02681094	2019	Multinational	DPP-4 inhibitors	296	Saxagliptin	5 mg daily	Metformin	5.5		AstraZeneca
**Rosenstock 2019**	NCT02681094	2019	Multinational	SGLT2 inhibitors	294	Dapagliflozin	5 mg daily	Metformin	5.5		AstraZeneca
**Rosenstock-2020**	NCT02229227	2020	Brazil, Canada, France, Germany, Hungary, Italy, Korea, Republic of, Mexico, Philippines, Poland,	Bolus insulin	412	Insulin lispro	adjusting insulin according to provided algorithms	Insulin glargine	6.9		GlaxoSmithKline (GSK)

Study ID	Registration	Year	Country	Intervention	N	Drug	Dose	Cointervention	Follow -up*	Settings	Fundings
			South Africa, Spain, United Kingdom, United States								
**Rosenstock-2020**	NCT02229227	2020	Brazil, Canada, France, Germany, Hungary, Italy, Korea, Republic of, Mexico, Philippines, Poland, South Africa, Spain, United Kingdom, United States	GLP-1 receptor agonists	402	Albiglutide	albiglutide: 50 mg once weekly; adjusting insulin according to provided algorithms	Insulin glargine	6.9		GlaxoSmithKline (GSK)
**Ross 2015**	NCT01512979	2015	Multinational	Metformin	159	Metformin	1500-2000 mg daily	Linagliptin	5.5		Boehringer Ingelheim
**Ross 2015**	NCT01512979	2015	Multinational	Standard treatments	157	Standard care		Linagliptin	5.5		Boehringer Ingelheim

Study ID	Registration	Year	Country	Intervention	N	Drug	Dose	Cointervention	Follow -up*	Settings	Fundings
**SAIS1**	UMIN 000004955	2016	Japan	DPP-4 inhibitors	48	Sitagliptin	50-100 mg daily	Metformin	6		Japan Waksman Foundation of Japan, and Hokkaido Diabetes Clinical Research Organization
**SAIS1**	UMIN 000004955	2016	Japan	Sulfonylureas	55	Glimepiride	0.5-2.0 mg daily	Metformin	6		Japan Waksman Foundation of Japan, and Hokkaido Diabetes Clinical Research Organization
**Saleem 2011**	Not provided	2011	Pakistan	Meglitinides	50	Repaglinide	4.27 mg daily	None	12		Not reported
**Saleem 2011**	Not provided	2011	Pakistan	Sulfonylureas	50	Glibenclamide	8.8 mg daily	None	12		Not reported
**Salman 2001**	Not applicable	2001	Turkey	Alpha-glucosidase inhibitors	27	Acarbose	200-300 mg daily	None	5.5		Bayer Turk
**Salman 2001**	Not applicable	2001	Turkey	Sulfonylureas	30	Gliclazide	80-160 mg daily	None	5.5		Bayer Turk
**Saloranta 2002**	Not applicable	2002	Multinational	Meglitinides	171	Nateglinide	360 mg daily	None	5.5		Novartis Pharma
**Saloranta 2002**	Not applicable	2002	Multinational	Meglitinides	175	Nateglinide	180 mg daily	None	5.5		Novartis Pharma

Study ID	Registration	Year	Country	Intervention	N	Drug	Dose	Cointervention	Follow -up*	Settings	Fundings
**Saloranta 2002**	Not applicable	2002	Multinational	Meglitinides	166	Nateglinide	60 mg daily	None	5.5		Novartis Pharma
**Saloranta 2002**	Not applicable	2002	Multinational	Meglitinides	512	Nateglinide	90-360 mg daily	None	5.5		Novartis Pharma
**Saloranta 2002**	Not applicable	2002	Multinational	Standard treatments	163	Placebo		None	5.5		Novartis Pharma
**Samson 2011**	NCT01432405	2011	USA	GLP-1 receptor agonists	11	Exenatide	20 ug daily	Pioglitazone	12		Amylin Pharmaceuticals and Eli-Lilly.
**Samson 2011**	NCT01432405	2011	USA	Standard treatments	10	Placebo		Pioglitazone	12		Amylin Pharmaceuticals and Eli-Lilly.
**Sato 2018**	Not applicable	2018	Japan	SGLT2 inhibitors	20	Dapagliflozin		Oral hypoglycemic drugs except SGLT-2 inhibitors	6		None reported
**Sato 2018**	Not applicable	2018	Japan	Standard treatments	20	Conventional treatment groups		Oral hypoglycemic drugs except SGLT-2 inhibitors	6		None reported
**Sato 2019**	UMIN000004985	2019	Japan	DPP-4 inhibitors	19	Sitagliptin	50 mg daily	Oral hypoglycemic agents except sulfonylurea, glinide or DPP-4 inhibitor	5.5		None reported

Study ID	Registration	Year	Country	Intervention	N	Drug	Dose	Cointervention	Follow-up*	Settings	Fundings
**Sato 2019**	UMIN000004985	2019	Japan	Sulfonylureas	22	Glimepiride	6 mg daily	Oral hypoglycemic agents except sulfonylurea, glinide or DPP-4 inhibitor	5.5		None reported
**SAVOR-TIMI 53**	NCT01107886	2013	Multinational	DPP-4 inhibitors	8280	Saxagliptin	2.5-5 mg daily	Excluded incretin-based therapy	25.2	High cardiovascular risk	AstraZeneca and Bristol-Myers Squibb
**SAVOR-TIMI 53**	NCT01107886	2013	Multinational	Standard treatments	8212	Placebo		Excluded incretin-based therapy	25.2		AstraZeneca and Bristol-Myers Squibb
**Saxagliptin 014**	NCT00121667	2009	Multinational	DPP-4 inhibitors	181	Saxagliptin	10 mg daily	Metformin	5.5		Bristol-Myers-Squibb
**Saxagliptin 014**	NCT00121667	2009	Multinational	DPP-4 inhibitors	191	Saxagliptin	5 mg daily	Metformin	5.5		Bristol-Myers-Squibb
**Saxagliptin 014**	NCT00121667	2009	Multinational	DPP-4 inhibitors	192	Saxagliptin	2.5 mg daily	Metformin	5.5		Bristol-Myers-Squibb
**Saxagliptin 014**	NCT00121667	2009	Multinational	DPP-4 inhibitors	564	Saxagliptin	2.5-10 mg daily	Metformin	5.5		Bristol-Myers-Squibb
**Saxagliptin 014**	NCT00121667	2009	Multinational	Standard treatments	179	Placebo		Metformin	5.5		Bristol-Myers-Squibb
**SCALE Diabetes**	NCT01272232	2015	Multinational	GLP-1 receptor agonists	432	Liraglutide	3 mg daily	1 to 3 drugs (metformin, thiazolidinedione, sulfonylurea)	13		Novo Nordisk

Study ID	Registration	Year	Country	Intervention	N	Drug	Dose	Cointervention	Follow -up*	Settings	Fundings
**SCALE Diabetes**	NCT01272232	2015	Multinational	GLP-1 receptor agonists	211	Liraglutide	1.8 mg daily	1 to 3 drugs (metformin, thiazolidinedione, sulfonylurea)	13		Novo Nordisk
**SCALE Diabetes**	NCT01272232	2015	Multinational	GLP-1 receptor agonists	643	Liraglutide	1.8-3 mg daily	1 to 3 drugs (metformin, thiazolidinedione, sulfonylurea)	13		Novo Nordisk
**SCALE Diabetes**	NCT01272232	2015	Multinational	Standard treatments	212	Placebo		1 to 3 drugs (metformin, thiazolidinedione, sulfonylurea)	13		Novo Nordisk
**SCALE insulin**	NCT02963922	2020	Canada, Germany, Israel, Italy, Mexico, Turkey, United States	GLP-1 receptor agonists	198	Liraglutide	3 mg once daily	intensive behavioral therapy, basal insulin and up to 2 OADs.	13.8	overweight or obesity (with a BMI>=27 kg/m2 ,)	Novo Nordisk A/S
**SCALE insulin**	NCT02963922	2020	Canada, Germany, Israel, Italy, Mexico,	Standard treatments	198	Placebo		intensive behavioral therapy, basal insulin and up to 2 OADs.	13.8		Novo Nordisk A/S

Study ID	Registration	Year	Country	Intervention	N	Drug	Dose	Cointervention	Follow -up*	Settings	Fundings
			Turkey, United States								
**Scherbaum 2002**	Not applicable	2002	Germany	Standard treatments	78	Placebo		None	6		Takeda Pharmaceuticals
**Scherbaum 2002**	Not applicable	2002	Germany	Thiazolidinediones	72	Pioglitazone	30 mg daily	None	6		Takeda Pharmaceuticals
**Scherbaum 2002**	Not applicable	2002	Germany	Thiazolidinediones	83	Pioglitazone	15 mg daily	None	6		Takeda Pharmaceuticals
**Scherbaum 2002**	Not applicable	2002	Germany	Thiazolidinediones	155	Pioglitazone	15-30 mg daily	None	6		Takeda Pharmaceuticals
**Scherbaum 2008**	NCT00101712	2008	Multinational	DPP-4 inhibitors	156	Vildagliptin	50 mg daily	None	12		Novartis Pharmaceuticals Corporation.
**Scherbaum 2008**	NCT00101712	2008	Multinational	Standard treatments	150	Placebo		None	12		Novartis Pharmaceuticals Corporation.
**Schiavon-2021**	NCT02973321	2021		GLP-1 receptor agonists	67	Liraglutide	1.8 mg once daily	On diet/exercise and/or treatment with metformin (stable dose of ≥1500 mg/day or maximal tolerated dose) for at least 3 months prior to screening	6		MIUR (Italian Minister for Education), Grant/ Award Number: “Departments of Excellence” (Law 232/2016);

Study ID	Registration	Year	Country	Intervention	N	Drug	Dose	Cointervention	Follow -up*	Settings	Fundings
											Sanofi, Grant/Award Number: NCT02973321
**Schiavon-2021**	NCT02973321	2021		Standard treatments	33	Placebo		On diet/exercise and/or treatment with metformin (stable dose of ≥1500 mg/day or maximal tolerated dose) for at least 3 months prior to screening	6		MIUR (Italian Minister for Education), Grant/ Award Number: “Departments of Excellence” (Law 232/2016); Sanofi, Grant/Award Number: NCT02973321
**Schweizer 2007**	NCT00099866	2007	Multinational	DPP-4 inhibitors	526	Vildagliptin	100 mg daily	None	12		Novartis Pharmaceuticals Corporation
**Schweizer 2007**	NCT00099866	2007	Multinational	Metformin	254	Metformin	2000 mg daily	None	12		Novartis Pharmaceuticals Corporation

Study ID	Registration	Year	Country	Intervention	N	Drug	Dose	Cointervention	Follow-up*	Settings	Fundings
**Schweizer 2009**	NCT00246619	2009	Multinational	DPP-4 inhibitors	169	Vildagliptin	100 mg daily	None	5.5		Novartis Pharmaceuticals Corporation
**Schweizer 2009**	NCT00246619	2009	Multinational	Metformin	166	Metformin	500-1500 mg daily	None	5.5		Novartis Pharmaceuticals Corporation
**SCORED**	NCT03315143	2020	750 sites in 44 countries	SGLT2 inhibitors	5292	Sotagliflozin	200 mg once daily, with an increase to 400 mg once daily if unacceptable side effects did not occur	metformin	15.9	chronic kidney disease (eGFR, 25 to 60 ml per minute per 1.73 m² of body-surface area), and additional cardiovascular risk factors	Sanofi and Lexicon Pharmaceuticals
**SCORED**	NCT03315143	2020	750 sites in 44 countries	Standard treatments	5292	Placebo		metformin	11.9		Sanofi and Lexicon Pharmaceuticals
**SEESAW_1**	NCT02798744	2022	UK	SGLT2 inhibitors	17	Empagliflozin	25 mg daily	None	5.5		Boehringer-Ingelheim
**SEESAW_1**	NCT02798744	2022	UK	Standard treatments	17	Placebo		None	5.5		Boehringer-Ingelheim

Study ID	Registration	Year	Country	Intervention	N	Drug	Dose	Cointervention	Follow -up*	Settings	Fundings
**SEESAW_2**	NCT02798744	2022	UK	SGLT2 inhibitors	17	Empagliflozin	25 mg daily	energy- restricted diet	5.5		Boehringer-Ingelheim
**SEESAW_2**	NCT02798744	2022	UK	Standard treatments	17	Placebo		energy- restricted diet	5.5		Boehringer-Ingelheim
**Segal 1997**	Not applicable	1997	Multinational	Alpha-glucosidase inhibitors	40	Miglitol	300 mg daily	None	5.5		Bayer
**Segal 1997**	Not applicable	1997	Multinational	Standard treatments	42	Placebo		None	5.5		Bayer
**Segal 1997**	Not applicable	1997	Multinational	Sulfonylureas	37	Glibenclamide	3.5 mg daily	None	5.5		Bayer
**Segal 2005**	Not applicable	2005	Israel	Alpha-glucosidase inhibitors	69	Acarbose	150-900 mg daily	Mainly sulfonylureas	18		Bayer
**Segal 2005**	Not applicable	2005	Israel	Standard treatments	70	Placebo		Mainly sulfonylureas	18		Bayer
**Seino 2011**	NCT00395746	2011	Japan	GLP-1 receptor agonists	88	Liraglutide	0.9 mg daily	Sulfonylurea	12		Novo Nordisk Pharma
**Seino 2011**	NCT00395746	2011	Japan	GLP-1 receptor agonists	88	Liraglutide	0.6 mg daily	Sulfonylurea	12		Novo Nordisk Pharma
**Seino 2011**	NCT00395746	2011	Japan	GLP-1 receptor agonists	176	Liraglutide	0.6-0.9 mg daily	Sulfonylurea	12		Novo Nordisk Pharma

Study ID	Registration	Year	Country	Intervention	N	Drug	Dose	Cointervention	Follow -up*	Settings	Fundings
**Seino 2011**	NCT00395746	2011	Japan	Standard treatments	88	Placebo		Sulfonylurea	12		Novo Nordisk Pharma
**Seino 2014**	JapicCTI-111661	2014	Japan	SGLT2 inhibitors	79	Luseogliflozin	2.5 mg daily	None	5.5		Taisho Pharmaceuticals
**Seino 2014**	JapicCTI-111661	2014	Japan	Standard treatments	79	Placebo		None	5.5		Taisho Pharmaceuticals
**Seino 2015**	JapicCTI-111507	2015	Japan	SGLT2 inhibitors	150	Luseogliflozin	2.5 mg daily	Sulfonylurea	5.5		Taisho Pharmaceuticals
**Seino 2015**	JapicCTI-111507	2015	Japan	Standard treatments	71	Placebo		Sulfonylurea	5.5		Taisho Pharmaceuticals
**Seino 2016**	NCT01572740	2016	Japan	GLP-1 receptor agonists	127	Liraglutide	0.9 mg daily	Insulin	8.4		Novo Nordisk Pharma
**Seino 2016**	NCT01572740	2016	Japan	Standard treatments	130	Placebo		Insulin	8.4		Novo Nordisk Pharma
**Seino 2018**	NCT02254291	2018	Japan	DPP-4 inhibitors	103	Sitagliptin	100 mg daily	None	7		Novo Nordisk Pharma
**Seino 2018**	NCT02254291	2018	Japan	GLP-1 receptor agonists	102	Semaglutide	1.0 mg weekly	None	7		Novo Nordisk Pharma
**Seino 2018**	NCT02254291	2018	Japan	GLP-1 receptor agonists	103	Semaglutide	0.5 mg weekly	None	7		Novo Nordisk Pharma

Study ID	Registration	Year	Country	Intervention	N	Drug	Dose	Cointervention	Follow-up*	Settings	Fundings
**Seino 2018**	NCT02254291	2018	Japan	GLP-1 receptor agonists	205	Semaglutide	0.5-1.0 mg weekly	None	7		Novo Nordisk Pharma
**Shah 2011**	Not provided	2011	Pakistan	Meglitinides	100	Repaglinide	0.5-2 mg three times daily	None	12		Not reported
**Shah 2011**	Not provided	2011	Pakstan	Sulfonylureas	100	Glibenclamide	5-15 mg daily	None	12		Not reported
**Shankar 2017**	NCT01590797	2017	China	DPP-4 inhibitors	234	Sitagliptin	100 mg daily	Insulin	5.5		Merck and Co
**Shankar 2017**	NCT01590797	2017	China	Standard treatments	233	Placebo		Insulin	5.5		Merck and Co
**Shankar 2017a**	NCT01755156	2017	Multinational	DPP-4 inhibitors	201	Omarigliptin	25 mg daily	Metformin	5.5		Merck and Co
**Shankar 2017a**	NCT01755156	2017	Multinational	Standard treatments	201	Placebo		Metformin	5.5		Merck and Co
**Shestakova 2018**	NCT02794792	2018	Russia	SGLT2 inhibitors	110	Ipragliflozin	50 mg daily	Metformin	5.5		Astellas Pharma
**Shestakova 2018**	NCT02794792	2018	Russia	Standard treatments	55	Placebo		Metformin	5.5		Astellas Pharma
**Shibuya 2018**	UMIN000016090	2018	Japan	Metformin	16	Metformin	1500 mg daily	Oral agents	6		No funding
**Shibuya 2018**	UMIN000016090	2018	Japan	SGLT2 inhibitors	16	Luseogliflozin	2.5 mg daily	Oral agents	6		No funding

Study ID	Registration	Year	Country	Intervention	N	Drug	Dose	Cointervention	Follow-up*	Settings	Fundings
**Shuai-2021**	NCT02477865	2021	China	GLP-1 receptor agonists	134	polyethylene glycol loxenatide	100 ug once weekly	None	5.5		This study was funded by Jiangsu Hansoh Pharmaceutical Group Co., Ltd. (Hansoh Pharma).
**Shuai-2021**	NCT02477865	2021	China	GLP-1 receptor agonists	136	polyethylene glycol loxenatide	200 ug once weekly	None	5.5		This study was funded by Jiangsu Hansoh Pharmaceutical Group Co., Ltd. (Hansoh Pharma).
**Shuai-2021**	NCT02477865	2021	China	GLP-1 receptor agonists	270	Loxenatide	100-200 ug weekly	None	5.5		This study was funded by Jiangsu Hansoh Pharmaceutical Group Co., Ltd. (Hansoh

Study ID	Registration	Year	Country	Intervention	N	Drug	Dose	Cointervention	Follow -up*	Settings	Fundings
											Pharma).
**Shuai-2021**	NCT02477865	2021	China	Standard treatments	136	Placebo		None	5.5		This study was funded by Jiangsu Hansoh Pharmaceutical Group Co., Ltd. (Hansoh Pharma).
**SIMPLE**	NCT01966978	2019	USA	Bolus insulin	61	Insulin aspart		Any treatment plus insulin detemir	6		Novo Nordisk Pharma
**SIMPLE**	NCT01966978	2019	USA	GLP-1 receptor agonists	59	Liraglutide	1.8 mg daily	Any treatment plus insulin detemir	6		Novo Nordisk Pharma
**Sit2Mix**	Not provided	2015	Multinational	DPP-4 inhibitors	195	Sitagliptin	100 mg daily	Metformin oral antidiabetic drugs	5.5		Novo Nordisk Pharma
**Sit2Mix**	Not provided	2015	Multinational	Standard treatments	194	Standard care		Metformin oral antidiabetic drugs	5.5		Novo Nordisk Pharma
**Sitagliptin 019**	NCT00086502	2006	Multinational	DPP-4 inhibitors	175	Sitagliptin	100 mg daily	Pioglitazone	5.5		Merck & Co., Whitehouse Station, USA

Study ID	Registration	Year	Country	Intervention	N	Drug	Dose	Cointervention	Follow -up*	Settings	Fundings
**Sitagliptin 019**	NCT00086502	2006	Multinational	Standard treatments	178	Placebo		Pioglitazone	5.5		Merck & Co., Whitehouse Station, USA
**Sitagliptin 020**	NCT0086515	2006	Multinational	DPP-4 inhibitors	464	Sitagliptin	100 mg daily	Metformin	5.5		Merck
**Sitagliptin 020**	NCT0086515	2006	Multinational	Standard treatments	237	Placebo		Metformin	5.5		Merck
**Sitagliptin 021**	NCT00087516	2006	Multinational	DPP-4 inhibitors	250	Sitagliptin	200 mg daily	None	5.5		Merck & Co., Whitehouse Station, USA
**Sitagliptin 021**	NCT00087516	2006	Multinational	DPP-4 inhibitors	238	Sitagliptin	100 mg daily	None	5.5		Merck & Co., Whitehouse Station, USA
**Sitagliptin 021**	NCT00087516	2006	Multinational	DPP-4 inhibitors	488	Sitagliptin	100-200 mg daily	None	5.5		Merck & Co., Whitehouse Station, USA
**Sitagliptin 021**	NCT00087516	2006	Multinational	Standard treatments	253	Placebo		None	5.5		Merck & Co., Whitehouse Station, USA
**Sitagliptin 024**	NCT00094770	2007	Multinational	DPP-4 inhibitors	588	Sitagliptin	100 mg daily	None, monotherapy or metformin + monotherapy	12		Merck
**Sitagliptin 024**	NCT00094770	2007	Multinational	Sulfonylureas	584	Glipizide	5-20 mg daily	None, monotherapy or metformin + monotherapy	12		Merck

Study ID	Registration	Year	Country	Intervention	N	Drug	Dose	Cointervention	Follow -up*	Settings	Fundings
**Sitagliptin 035**	Not provided	2007	Multinational	DPP-4 inhibitors	222	Sitagliptin	100 mg daily	None, sulfonylurea metformin, or mono-, dual, or triple therapy	5.5		Merck & Co., Whitehouse Station, USA
**Sitagliptin 035**	Not provided	2007	Multinational	Standard treatments	219	Placebo		None, sulfonylurea metformin, or mono-, dual, or triple therapy	5.5		Merck & Co., Whitehouse Station, USA
**Sitagliptin 036**	NCT00103857	2007	Multinational	DPP-4 inhibitors	179	Sitagliptin	100 mg daily	None	5.5		Merck & Company
**Sitagliptin 036**	NCT00103857	2007	Multinational	Standard treatments	176	Placebo		None	5.5		Merck & Company
**Sitagliptin 049**	NCT00449930	2010	Multinational	DPP-4 inhibitors	528	Sitagliptin	100 mg daily	None	6		Merck and Co
**Sitagliptin 049**	NCT00449930	2010	Multinational	Metformin	522	Metformin	2000 mg daily	None	6		Merck and Co
**Sitagliptin 051**	NCT00395343	2010	Multinational	DPP-4 inhibitors	322	Sitagliptin	100 mg daily	Insulin metformin	5.5		Merck
**Sitagliptin 051**	NCT00395343	2010	Multinational	Standard treatments	319	Placebo		Insulin metformin	5.5		Merck
**SLIM**	UMIN000006511	2020	Japan	DPP-4 inhibitors	97	Sitagliptin	50 mg once daily	sulfonylurea	6		This study was financially supported by The Kidney Foundation, Japan.

Study ID	Registration	Year	Country	Intervention	N	Drug	Dose	Cointervention	Follow -up*	Settings	Fundings
**SLIM**	UMIN000006511	2020	Japan	Standard treatments	92	Non-sitagliptin		sulfonylurea	6		This study was financially supported by The Kidney Foundation, Japan.
**SMART**	NCT02243176	2017	China	Alpha-glucosidase inhibitors	244	Acarbose	300 mg daily	Metformin	5.5		AstraZeneca
**SMART**	NCT02243176	2017	China	DPP-4 inhibitors	241	Saxagliptin	5 mg daily	Metformin	5.5		AstraZeneca
**Smith 2005**	Not applicable	2005	USA	Standard treatments	24	Placebo		Metformin; sulfonylurea	6		Takeda Pharmaceuticals
**Smith 2005**	Not applicable	2005	USA	Thiazolidinediones	24	Pioglitazone	30-45 mg/day	Metformin; sulfonylurea	6		Takeda Pharmaceuticals
**Softeland 2017**	NCT01734785	2017	Multinational	SGLT2 inhibitors	111	Empagliflozin	25 mg daily	Meformin + linagliptin	5.5		Boehringer Ingelheim Pharma
**Softeland 2017**	NCT01734785	2017	Multinational	SGLT2 inhibitors	112	Empagliflozin	10 mg daily	Meformin + linagliptin	5.5		Boehringer Ingelheim Pharma
**Softeland 2017**	NCT01734785	2017	Multinational	SGLT2 inhibitors	223	Empagliflozin	10-25 mg daily	Meformin + linagliptin	5.5		Boehringer Ingelheim Pharma
**Softeland 2017**	NCT01734785	2017	Multinational	Standard treatments	110	Placebo		Meformin + linagliptin	5.5		Boehringer Ingelheim Pharma
**Sohn 2008**	Not provided	2006	Republic of Korea	Metformin	8	Metformin	2000 mg daily	None	7.5		Not reported
**Sohn 2008**	Not provided	2006	Republic of Korea	Thiazolidinediones	11	Rosiglitazone	8 mg daily	None	7.5		Not reported

Study ID	Registration	Year	Country	Intervention	N	Drug	Dose	Cointervention	Follow -up*	Settings	Fundings
**SOLOIST-WHF**	NCT03521934	2020	306 sites in 32 countries	SGLT2 inhibitors	608	sotagliflozin	200 mg once daily, with a dose increase to 400 mg, depending on side effects	metformin and other treatment	9.2	recently hospitalized for worsening heart failure	Funded by Sanofi and Lexicon Pharmaceuticals
**SOLOIST-WHF**	NCT03521934	2020	306 sites in 32 countries	Standard treatments	614	placebo		metformin and other treatment	8.9		Funded by Sanofi and Lexicon Pharmaceuticals
**Sone 2019**	NCT02589639	2019	Japan	SGLT2 inhibitors	90	Empagliflozin	25 mg daily	Insulin	12		Boehringer Ingelheim Pharma
**Sone 2019**	NCT02589639	2019	Japan	SGLT2 inhibitors	86	Empagliflozin	10 mg daily	Insulin	12		Boehringer Ingelheim Pharma
**Sone 2019**	NCT02589639	2019	Japan	SGLT2 inhibitors	176	Empagliflozin	10-25 mg daily	Insulin	12		Boehringer Ingelheim Pharma
**Sone 2019**	NCT02589639	2019	Japan	Standard treatments	90	Placebo		Insulin	12		Boehringer Ingelheim Pharma
**South Danish Diabetes Study**	Not provided	2011	Denmark	Metformin	45	Metformin	1000-2000 mg daily	Oral agents insulin	24		Southern counties of Denmark and the Danish Medical Research Council, Novo Nordisk

Study ID	Registration	Year	Country	Intervention	N	Drug	Dose	Cointervention	Follow -up*	Settings	Fundings
**South Danish Diabetes Study**	Not provided	2011	Denmark	Standard treatments	48	Placebo		Oral agents insulin	24		Southern counties of Denmark and the Danish Medical Research Council, Novo Nordisk
**South Danish Diabetes Study**	Not provided	2011	Denmark	Thiazolidinediones	47	Rosiglitazone	4-8 mg daily	Oral agents insulin	24		Southern counties of Denmark and the Danish Medical Research Council, Novo Nordisk
**SPEAD-A**	UMIN000005311	2016	Japan	DPP-4 inhibitors	172	Alogliptin	25 mg daily	Treatment excluding DPP-4 inhibitors	24		Astellas Pharma Inc., AstraZeneca K.K., Bayer Holding, Daiichi Sankyo Co., Sumitomo Dainippon Pharma Co., Eli Lilly Japan K.K., MSD K.K., Nippon Boehringer Ingelheim, Novartis Pharma K.K., Novo Nordisk Pharma Ltd., Pfizer Japan Inc., Sano K.K., Sanwa Kagaku Kenkyusho

Study ID	Registration	Year	Country	Intervention	N	Drug	Dose	Cointervention	Follow -up*	Settings	Fundings
											Co., Shionogi & Co., Ltd., and Takeda Pharmaceutical Co
**SPEAD-A**	UMIN000005311	2016	Japan	Standard treatments	169	Standard care		Treatment excluding DPP-4 inhibitors	24		Astellas Pharma Inc., AstraZeneca K.K., Bayer Holding, Daiichi Sankyo Co., Sumitomo Dainippon Pharma Co., Eli Lilly Japan K.K., MSD K.K., Nippon Boehringer Ingelheim, Novartis Pharma K.K., Novo Nordisk Pharma Ltd., Pfizer Japan Inc., Sano K.K., Sanwa Kagaku Kenkyusho Co., Shionogi & Co., Ltd., and Takeda Pharmaceutical Co
**SPECIFY**	NCT02280486	2019	China	DPP-4 inhibitors	194	saxagliptin	5 mg once daily	metformin	11.1		The study was funded by AstraZeneca
**SPECIFY**	NCT02280486	2019	China	Sulfonylureas	194	glimepiride	1-6 mg once daily	metformin	11.1		The study was funded by AstraZeneca

Study ID	Registration	Year	Country	Intervention	N	Drug	Dose	Cointervention	Follow -up*	Settings	Fundings
**SPIKE**	UMIN000007396	2016	Japan	DPP-4 inhibitors	142	Sitagliptin	25-100 mg daily	Treatment excluding DPP-4 inhibitors	24		Novo Nordisk
**SPIKE**	UMIN000007396	2016	Japan	Standard treatments	140	Standard care		Treatment excluding DPP-4 inhibitors	24		Novo Nordisk
**SPOTLIGHT**	NCT01225081	2015	Japan	SGLT2 inhibitors	98	Ipragliflozin	50 mg daily	Pioglitazone	12		Astellas Pharma
**SPOTLIGHT**	NCT01225081	2015	Japan	Standard treatments	54	Placebo		Pioglitazone	12		Astellas Pharma
**SPREAD-DIMCAD**	NCT00513630	2013	China	Metformin	156	Metformin	750-1500 mg daily	Excluding insulin	36	Coronary artery disease	National Natural Science Foundation of China (30971077, 81170784), the Shanghai Shenkang Hospital Development Center (Shdc12007309), the Key Laboratory for Endocrine and Metabolic Diseases of Ministry of Chinese Public Health (1994DP131044), the

Study ID	Registration	Year	Country	Intervention	N	Drug	Dose	Cointervention	Follow -up*	Settings	Fundings
											National Key New Drug Creation and Manufacturing Program (2008ZX09312/019), the Shanghai Committee on Science and Technology (10dz1920802), the Program for Innovative Research Team of Shanghai Municipal Education Commission, the Sector Funds of Ministry of Health (No. 201002002), and the National Key New Drug Creation and Manufacturing Program of Ministry of Science and Technology (No.

Study ID	Registration	Year	Country	Intervention	N	Drug	Dose	Cointervention	Follow-up*	Settings	Fundings
											2012ZX09303006-001).
**SPREAD-DIMCAD**	NCT00513630	2013	China	Sulfonylureas	148	Glipizide	15-30 mg daily	Excluding insulin	36		National Natural Science Foundation of China (30971077, 81170784), the Shanghai Shenkang Hospital Development Center (Shdc12007309), the Key Laboratory for Endocrine and Metabolic Diseases of Ministry of Chinese Public Health (1994DP131044), the National Key New Drug Creation and Manufacturing Program (2008ZX09312/019), the Shanghai Committee on Science and

Study ID	Registration	Year	Country	Intervention	N	Drug	Dose	Cointervention	Follow-up*	Settings	Fundings
											Technology (10dz1920802), the Program for Innovative Research Team of Shanghai Municipal Education Commission, the Sector Funds of Ministry of Health (No. 201002002), and the National Key New Drug Creation and Manufacturing Program of Ministry of Science and Technology (No. 2012ZX09303006-001).
**Sridhar 2013**	NCT01206400	2013	India	Standard treatments	25	Placebo		Glimepiride and metformin	6		Sun Pharmaceuticals
**Sridhar 2013**	NCT01206400	2013	India	Thiazolidinediones	25	Pioglitazone	30 mg daily	Glimepiride and metformin	6		Sun Pharmaceuticals
**St. John Sutton 2002**	Not applicable	2002	USA	Sulfonylureas	99	Glyburide	20 mg daily	None	12		Not reported

Study ID	Registration	Year	Country	Intervention	N	Drug	Dose	Cointervention	Follow -up*	Settings	Fundings
**St. John Sutton 2002**	Not applicable	2002	USA	Thiazolidinediones	104	Rosiglitazone	8 mg daily	None	12		Not reported
**Standl 1999**	Not applicable	1999	Germany	Alpha-glucosidase inhibitors	24	Acarbose	50 mg twice daily-200 mg three times daily	Sulphonylurea, Insulin	5.5		Not reported
**Standl 1999**	Not applicable	1999	Germany	Standard treatments	24	Placebo		Sulphonylurea, Insulin	5.5		Not reported
**Standl 2001**	Not applicable	2001	Multinational	Alpha-glucosidase inhibitors	65	Miglitol	300 mg daily	Metformin + glibenclamide	5.5		Bayer; Sanofi-Synthelabo
**Standl 2001**	Not applicable	2001	Multinational	Standard treatments	68	Placebo		Metformin + glibenclamide	5.5		Bayer; Sanofi-Synthelabo
**START**	NCT02273050	2018	China	DPP-4 inhibitors	214	Saxagliptin	5 mg daily	None	5.5		AstraZeneca
**START**	NCT02273050	2018	China	Metformin	210	Metformin	2000 mg daily	None	5.5		AstraZeneca
**START-J**	NCT01183104; UMIN000004047	2016	Japan	DPP-4 inhibitors	153	Sitagliptin	25-100 mg daily	None	12		Merck Sharpe & Dohme
**START-J**	NCT01183104; UMIN000004047	2016	Japan	Sulfonylureas	152	Glimepiride	0.25-3 mg daily	None	12		Merck Sharpe & Dohme
**STEP 2**	NCT03552757	2021	149 outpatient	GLP-1 receptor agonists	403	semaglutide	1 mg once weekly	lifestyle intervention,metfor	17.3	overweight or obesity (with a	Novo Nordisk

Study ID	Registration	Year	Country	Intervention	N	Drug	Dose	Cointervention	Follow -up*	Settings	Fundings
			clinics in 12 countries					min and other treatment		BMI>=27 kg/m2)	
**STEP 2**	NCT03552757	2021	149 outpatient clinics in 12 countries	GLP-1 receptor agonists	404	semaglutide	2.4 mg once weekly	lifestyle intervention,metformin and other treatment	17.3		Novo Nordisk
**STEP 2**	NCT03552757	2021	149 outpatient clinics in 12 countries	GLP-1 receptor agonists	807	semaglutide	1-2.4 mg weekly	lifestyle intervention,metformin and other treatment	17.3		Novo Nordisk
**STEP 2**	NCT03552757	2021	149 outpatient clinics in 12 countries	Standard treatments	403	placebo		lifestyle intervention,metformin and other treatment	17.3		Novo Nordisk
**Stewart 2006**	Not provided	2006	Europe	Standard treatments	272	Control		None or monotherapy excluding insulin	7.4		Glaxo
**Stewart 2006**	Not provided	2006	Europe	Thiazolidinediones	254	Rosiglitazone	4-8 mg daily	None or monotherapy excluding insulin	7.4		Glaxo
**Stocker 2007**	Not provided	2007	USA	Metformin	47	Metformin	1500 mg daily	None	5.5		GlaxoSmithKline
**Stocker 2007**	Not provided	2007	USA	Thiazolidinediones	45	Rosiglitazone	4 mg daily	None	5.5		GlaxoSmithKline

Study ID	Registration	Year	Country	Intervention	N	Drug	Dose	Cointervention	Follow -up*	Settings	Fundings
**STOP-OB**	UMIN000026161	2020	Japan	SGLT2 inhibitors	33	Tofogliflozin	20 mg daily	Metformin + DPP-4 inhibitor	5.5		Kowa Co
**STOP-OB**	UMIN000026161	2020	Japan	Sulfonylureas	31	Glimepiride	0.5 mg daily	Metformin + DPP-4 inhibitor	5.5		Kowa Co
**Strojek 2009**	NCT00469092	2009	Multinational	Basal insulin	241	Insulin glargine	Titrated	Metformin + secretagogue (no more than 3 oral drugs)	6		Novo Nordisk
**Strojek 2009**	NCT00469092	2009	Multinational	Basal-Bolus insulin	239	biphasic insulin aspart 70/30 (BIAsp 30)	Titrated	Metformin + secretagogue (no more than 3 oral drugs)	6		Novo Nordisk
**Strojek 2011**	NCT00680745	2011	Multinational	SGLT2 inhibitors	151	Dapagliflozin	10 mg daily	Sulfonylurea	5.5		AstraZeneca and Bristol-Myers Squibb
**Strojek 2011**	NCT00680745	2011	Multinational	SGLT2 inhibitors	145	Dapagliflozin	5 mg daily	Sulfonylurea	5.5		AstraZeneca and Bristol-Myers Squibb
**Strojek 2011**	NCT00680745	2011	Multinational	SGLT2 inhibitors	154	Dapagliflozin	2.5 mg daily	Sulfonylurea	5.5		AstraZeneca and Bristol-Myers Squibb
**Strojek 2011**	NCT00680745	2011	Multinational	SGLT2 inhibitors	450	Dapagliflozin	2.5-10 mg daily	Sulfonylurea	5.5		AstraZeneca and Bristol-Myers Squibb
**Strojek 2011**	NCT00680745	2011	Multinational	Standard treatments	146	Placebo		Sulfonylurea	5.5		AstraZeneca and Bristol-Myers Squibb
**Strøm Halden 2019**	NCT03157414	2019	Norway	SGLT2 inhibitors	22	Empagliflozin	10 mg daily	None	5.5	Chronic kidney disease	South-Eastern Norway Regional Health Authority, the

Study ID	Registration	Year	Country	Intervention	N	Drug	Dose	Cointervention	Follow -up*	Settings	Fundings
											Norwegian Diabetes Association, and Oslo Diabetes Research Centre
**Strøm Halden 2019**	NCT03157414	2019	Norway	Standard treatments	22	Placebo		None	5.5		South-Eastern Norway Regional Health Authority, the Norwegian Diabetes Association, and Oslo Diabetes Research Centre
**Su 2014**	Not provided	2014	China	DPP-4 inhibitors	300	Vildagliptin	100 mg daily	Metformin + alpha glucosidase inhibitor	5.5		Not reported
**Su 2014**	Not provided	2014	China	Standard treatments	300	Placebo		Metformin + alpha glucosidase inhibitor	5.5		Not reported
**SUCCESS**	UMIN 000004675	2015	Japan	Alpha-glucosidase inhibitors	59	Alpha glucosidase inhibitor	0.2 mg voglibose or 150 mg miglitol daily	Metformin or pioglitazone	5.5		Japanese Ministry of Health, Labor and Welfare, and the Ministry of Education, Culture, Sports, Science and Technology

Study ID	Registration	Year	Country	Intervention	N	Drug	Dose	Cointervention	Follow-up*	Settings	Fundings
**SUCCESS**	UMIN 000004675	2015	Japan	DPP-4 inhibitors	60	Sitagliptin	50 mg daily	Metformin or pioglitazone	5.5		Japanese Ministry of Health, Labor and Welfare, and the Ministry of Education, Culture, Sports, Science and Technology
**SUGAR-DM-HF**	NCT03485092	2021	15 hospitals in Scotland, United Kingdom	SGLT2 inhibitors	52	empagliflozin	10 mg once daily	metformin and other treatment	9.2	heart failure with New York Heart Association functional class II to IV with a left ventricular (LV) ejection fraction =<40%	This trial was supported by an investigator-initiated study grant from Boehringer Ingelheim
**SUGAR-DM-HF**	NCT03485092	2021	15 hospitals in Scotland, United Kingdom	Standard treatments	53	placebo		metformin and other treatment	9.2		This trial was supported by an investigator-initiated study grant from Boehringer Ingelheim
**Sun 2006**	Not provided	2006	China	Sulfonylureas	30	Glipizide	5 mg daily	None	6		Social Development Fund of Science and

Study ID	Registration	Year	Country	Intervention	N	Drug	Dose	Cointervention	Follow -up*	Settings	Fundings
											Technology Committee of Jiangsu Province (BS2000408); Science and Technology Fund of Southeast University (ZJ002666)
**Sun 2006**	Not provided	2006	China	Thiazolidinediones	30	Pioglitazone	30 mg daily	None	6		Social Development Fund of Science and Technology Committee of Jiangsu Province (BS2000408); Science and Technology Fund of Southeast University (ZJ002666)
**Sun 2016**	ChiCTR-TRC-11001808	2016	China	Alpha-glucosidase inhibitors	54	Acarbose	300 mg daily	None	5.5		None
**Sun 2016**	ChiCTR-TRC-11001808	2016	China	Metformin	54	Metformin	1500 mg daily	None	5.5		None
**SUPER**	NCT02104804	2018	China	DPP-4 inhibitors	232	Saxagliptin	5 mg daily	Insulin with or without metformin	5.5		AstraZeneca

Study ID	Registration	Year	Country	Intervention	N	Drug	Dose	Cointervention	Follow-up*	Settings	Fundings
**SUPER**	NCT02104804	2018	China	Standard treatments	230	Placebo		Insulin with or without metformin	5.5		AstraZeneca
**SURPASS J-mono**	NCT03861052	2022	Japan	Dual GIP/GLP-1 receptor agonists	159	Tirzepatide	5 mg once weekly	Oral antihyperglycemic drugs	12		Eli Lilly
**SURPASS J-mono**	NCT03861052	2022	Japan	Dual GIP/GLP-1 receptor agonists	158	Tirzepatide	10 mg once weekly	Oral antihyperglycemic drugs	12		Eli Lilly
**SURPASS J-mono**	NCT03861052	2022	Japan	Dual GIP/GLP-1 receptor agonists	160	Tirzepatide	15 mg once weekly	Oral antihyperglycemic drugs	12		Eli Lilly
**SURPASS J-mono**	NCT03861052	2022	Japan	Dual GIP/GLP-1 receptor agonists	477	Tirzepatide	5-15 mg weekly	Oral antihyperglycemic drugs	12		Eli Lilly
**SURPASS J-mono**	NCT03861052	2022	Japan	GLP-1 receptor agonists	159	Dulaglutide	0.75 mg weekly	Oral antihyperglycemic drugs	12		Eli Lilly
**SURPASS-1**	NCT03954834	2021	52 medical research centres and hospitals in	Dual GIP/GLP-1 receptor agonists	121	tirzepatide	5 mg once weekly	metformin and other treatment	10.2		Eli Lilly and Company

Study ID	Registration	Year	Country	Intervention	N	Drug	Dose	Cointervention	Follow -up*	Settings	Fundings
			India, Japan, Mexico, and the USA								
**SURPASS-1**	NCT03954834	2021	52 medical research centres and hospitals in India, Japan, Mexico, and the USA	Dual GIP/GLP-1 receptor agonists	121	tirzepatide	10 mg once weekly	metformin and other treatment	10.2		Eli Lilly and Company
**SURPASS-1**	NCT03954834	2021	52 medical research centres and hospitals in India, Japan, Mexico, and the USA	Dual GIP/GLP-1 receptor agonists	121	tirzepatide	15 mg once weekly	metformin and other treatment	10.2		Eli Lilly and Company
**SURPASS-1**	NCT03954834	2021	52 medical research centres and hospitals in India, Japan, Mexico, and the USA	Dual GIP/GLP-1 receptor agonists	363	tirzepatide	5-15 mg	metformin and other treatment	10.2		Eli Lilly and Company

Study ID	Registration	Year	Country	Intervention	N	Drug	Dose	Cointervention	Follow -up*	Settings	Fundings
**SURPASS-1**	NCT03954834	2021	52 medical research centres and hospitals in India, Japan, Mexico, and the USA	Standard treatments	115	placebo		metformin and other treatment	10.2		Eli Lilly and Company
**SURPASS-2**	NCT03987919	2021	128 sites in the United States, Argentina, Australia, Brazil, Canada, Israel, Mexico, and the United Kingdom	Dual GIP/GLP-1 receptor agonists	471	tirzepatide	5 mg once weekly	metformin alone	10.2	with a BMI>=25 kg/m2	Eli Lilly
**SURPASS-2**	NCT03987919	2021	128 sites in the United States, Argentina, Australia, Brazil, Canada,	Dual GIP/GLP-1 receptor agonists	469	tirzepatide	10 mg once weekly	metformin alone	10.2		Eli Lilly

Study ID	Registration	Year	Country	Intervention	N	Drug	Dose	Cointervention	Follow -up*	Settings	Fundings
			Israel, Mexico, and the United Kingdom								
**SURPASS-2**	NCT03987919	2021	128 sites in the United States, Argentina, Australia, Brazil, Canada, Israel, Mexico, and the United Kingdom	Dual GIP/GLP-1 receptor agonists	470	tirzepatide	15 mg once weekly	metformin alone	10.2		Eli Lilly
**SURPASS-2**	NCT03987919	2021	128 sites in the United States, Argentina, Australia, Brazil, Canada, Israel, Mexico, and	Dual GIP/GLP-1 receptor agonists	1410	tirzepatide	5-15 mg	metformin alone	10.2		Eli Lilly

Study ID	Registration	Year	Country	Intervention	N	Drug	Dose	Cointervention	Follow -up*	Settings	Fundings
			the United Kingdom								
**SURPASS-2**	NCT03987919	2021	128 sites in the United States, Argentina, Australia, Brazil, Canada, Israel, Mexico, and the United Kingdom	GLP-1 receptor agonists	469	semaglutide	1 mg once weekly	metformin alone	10.2		Eli Lilly
**SURPASS-3**	NCT03882970	2021	122 medical research centres and hospitals in Argentina, Austria, Greece, Hungary, Italy, Poland, Puerto Rico, Romania, South Korea,	Basal insulin	365	insulin degludec	once daily	metformin alone or in combination with an SGLT2 inhibitor	12.9		Eli Lilly and Company

Study ID	Registration	Year	Country	Intervention	N	Drug	Dose	Cointervention	Follow -up*	Settings	Fundings
			Spain, Taiwan, Ukraine, and the USA.								
**SURPASS-3**	NCT03882970	2021	122 medical research centres and hospitals in Argentina, Austria, Greece, Hungary, Italy, Poland, Puerto Rico, Romania, South Korea, Spain, Taiwan, Ukraine, and the USA.	Dual GIP/GLP-1 receptor agonists	359	tirzepatide	5 mg once weekly	metformin alone or in combination with an SGLT2 inhibitor	12.9	with a BMI>=25 kg/m2	Eli Lilly and Company
**SURPASS-3**	NCT03882970	2021	122 medical research centres and hospitals in Argentina,	Dual GIP/GLP-1 receptor agonists	361	tirzepatide	10 mg once weekly	metformin alone or in combination with an SGLT2 inhibitor	12.9		Eli Lilly and Company

Study ID	Registration	Year	Country	Intervention	N	Drug	Dose	Cointervention	Follow-up*	Settings	Fundings
			Austria, Greece, Hungary, Italy, Poland, Puerto Rico, Romania, South Korea, Spain, Taiwan, Ukraine, and the USA.								
**SURPASS-3**	NCT03882970	2021	122 medical research centres and hospitals in Argentina, Austria, Greece, Hungary, Italy, Poland, Puerto Rico, Romania, South Korea, Spain, Taiwan,	Dual GIP/GLP-1 receptor agonists	359	tirzepatide	15 mg once weekly	metformin alone or in combination with an SGLT2 inhibitor	12.9		Eli Lilly and Company

Study ID	Registration	Year	Country	Intervention	N	Drug	Dose	Cointervention	Follow -up*	Settings	Fundings
			Ukraine, and the USA.								
**SURPASS-3**	NCT03882970	2021	122 medical research centres and hospitals in Argentina, Austria, Greece, Hungary, Italy, Poland, Puerto Rico, Romania, South Korea, Spain, Taiwan, Ukraine, and the USA.	Dual GIP/GLP-1 receptor agonists	1079	tirzepatide	5-15 mg	metformin alone or in combination with an SGLT2 inhibitor	12.9		Eli Lilly and Company
**SURPASS-4**	NCT03730662	2021	187 sites in 14 countries (Argentina, Australia, Brazil, Canada, Greece,	Basal insulin	1005	insulin glargine	once daily	any of three oral glucose-lowering medications (ie, metformin, sulfonylurea, or sodium-glucose cotransporter-2	12.9		Eli Lilly and Company

Study ID	Registration	Year	Country	Intervention	N	Drug	Dose	Cointervention	Follow-up*	Settings	Fundings
			Israel, Mexico, Poland, Romania, Russia, Slovakia, Spain, Taiwan, and the USA)					[SGLT-2] inhibitor) either alone or in any combination			
**SURPASS-4**	NCT03730662	2021	187 sites in 14 countries (Argentina, Australia, Brazil, Canada, Greece, Israel, Mexico, Poland, Romania, Russia, Slovakia, Spain, Taiwan, and the USA)	Dual GIP/GLP-1 receptor agonists	329	tirzepatide	5 mg once weekly	any of three oral glucose-lowering medications (ie, metformin, sulfonylurea, or sodium-glucose cotransporter-2 [SGLT-2] inhibitor) either alone or in any combination	12.9	with a BMI>=25 kg/m2; increased risk of cardiovascular events, defined as known coronary, peripheral arterial, or cerebrovascular disease, or aged 50 years or older with either history	Eli Lilly and Company

Study ID	Registration	Year	Country	Intervention	N	Drug	Dose	Cointervention	Follow -up*	Settings	Fundings
										of chronic kidney disease and an estimated glomerular filtration rate (eGFR) of less than 60 mL/min per 1·73 m² or history of congestive heart failure (New York Heart Association Class II or III).	
**SURPASS-4**	NCT03730662	2021	187 sites in 14 countries (Argentina, Australia, Brazil, Canada, Greece, Israel,	Dual GIP/GLP-1 receptor agonists	330	tirzepatide	10 mg once weekly	any of three oral glucose-lowering medications (ie, metformin, sulfonylurea, or sodium-glucose cotransporter-2 [SGLT-2] inhibitor)	12.9		Eli Lilly and Company

Study ID	Registration	Year	Country	Intervention	N	Drug	Dose	Cointervention	Follow -up*	Settings	Fundings
			Mexico, Poland, Romania, Russia, Slovakia, Spain, Taiwan, and the USA)					either alone or in any combination			
**SURPASS-4**	NCT03730662	2021	187 sites in 14 countries (Argentina, Australia, Brazil, Canada, Greece, Israel, Mexico, Poland, Romania, Russia, Slovakia, Spain, Taiwan, and the USA)	Dual GIP/GLP-1 receptor agonists	338	tirzepatide	15 mg once weekly	any of three oral glucose-lowering medications (ie, metformin, sulfonylurea, or sodium-glucose cotransporter-2 [SGLT-2] inhibitor) either alone or in any combination	12.9		Eli Lilly and Company

Study ID	Registration	Year	Country	Intervention	N	Drug	Dose	Cointervention	Follow -up*	Settings	Fundings
**SURPASS-4**	NCT03730662	2021	187 sites in 14 countries (Argentina, Australia, Brazil, Canada, Greece, Israel, Mexico, Poland, Romania, Russia, Slovakia, Spain, Taiwan, and the USA)	Dual GIP/GLP-1 receptor agonists	997	tirzepatide	5-15 mg	any of three oral glucose-lowering medications (ie, metformin, sulfonylurea, or sodium-glucose cotransporter-2 [SGLT-2] inhibitor) either alone or in any combination	12.9		Eli Lilly and Company
**SURPASS-5**	NCT04039503	2022	45 medical research centers and hospitals in the US, Japan, CzechRepublic, Germany, Poland,	Dual GIP/GLP-1 receptor agonists	116	tirzepatide	5 mg once weekly	insulin glargine with or without metformin	10.2	with a BMI>=23 kg/m2	Eli Lilly and Company

Study ID	Registration	Year	Country	Intervention	N	Drug	Dose	Cointervention	Follow -up*	Settings	Fundings
			PuertoRico, Slovakia, and Spain								
**SURPASS-5**	NCT04039503	2022	45 medical research centers and hospitals in the US, Japan, CzechRepublic, Germany, Poland, PuertoRico, Slovakia, and Spain	Dual GIP/GLP-1 receptor agonists	119	tirzepatide	10 mg once weekly	insulin glargine with or without metformin	10.2		Eli Lilly and Company
**SURPASS-5**	NCT04039503	2022	45 medical research centers and hospitals in the US, Japan, CzechRepublic, Germany, Poland, PuertoRico,	Dual GIP/GLP-1 receptor agonists	120	tirzepatide	15 mg once weekly	insulin glargine with or without metformin	10.2		Eli Lilly and Company

Study ID	Registration	Year	Country	Intervention	N	Drug	Dose	Cointervention	Follow-up*	Settings	Fundings
			Slovakia, and Spain								
**SURPASS-5**	NCT04039503	2022	45 medical research centers and hospitals in the US, Japan, CzechRepublic, Germany, Poland, PuertoRico, Slovakia, and Spain	Dual GIP/GLP-1 receptor agonists	355	tirzepatide	5-15 mg	insulin glargine with or without metformin	10.2		Eli Lilly and Company
**SURPASS-5**	NCT04039503	2022	45 medical research centers and hospitals in the US, Japan, CzechRepublic, Germany, Poland, PuertoRico,	Standard treatments	120	placebo		insulin glargine with or without metformin	10.2		Eli Lilly and Company

Study ID	Registration	Year	Country	Intervention	N	Drug	Dose	Cointervention	Follow-up*	Settings	Fundings
			Slovakia, and Spain								
**SUSTAIN 1**	NCT02054897	2014	Multinational	GLP-1 receptor agonists	130	Semaglutide	1.0 mg weekly	None	6		Novo Nordisk A/S
**SUSTAIN 1**	NCT02054897	2014	Multinational	GLP-1 receptor agonists	128	Semaglutide	0.5 mg weekly	None	6		Novo Nordisk A/S
**SUSTAIN 1**	NCT02054897	2014	Multinational	GLP-1 receptor agonists	258	Semaglutide	0.5-1.0 mg weekly	None	6		Novo Nordisk A/S
**SUSTAIN 1**	NCT02054897	2014	Multinational	Standard treatments	129	Placebo		None	6		Novo Nordisk A/S
**SUSTAIN 2**	NCT01930188	2017	Multinational	DPP-4 inhibitors	407	Sitagliptin	100 mg daily	Metformin thiazolidinedione	12		Novo Nordisk A/S
**SUSTAIN 2**	NCT01930188	2017	Multinational	GLP-1 receptor agonists	409	Semaglutide	1.0 mg weekly	Metformin thiazolidinedione	12		Novo Nordisk A/S
**SUSTAIN 2**	NCT01930188	2017	Multinational	GLP-1 receptor agonists	409	Semaglutide	0.5 mg weekly	Metformin thiazolidinedione	12		Novo Nordisk A/S
**SUSTAIN 2**	NCT01930188	2017	Multinational	GLP-1 receptor agonists	818	Semaglutide	0.5-1.0 mg weekly	Metformin thiazolidinedione	12		Novo Nordisk A/S

Study ID	Registration	Year	Country	Intervention	N	Drug	Dose	Cointervention	Follow -up*	Settings	Fundings
**SUSTAIN 4**	NCT02128932	2017	Multinational	Basal insulin	360	Insulin glargine	Titrated	Metformin sulfonylurea	6		Novo Nordisk A/S
**SUSTAIN 4**	NCT02128932	2017	Multinational	GLP-1 receptor agonists	360	Semaglutide	1.0 mg weekly	Metformin sulfonylurea	6		Novo Nordisk A/S
**SUSTAIN 4**	NCT02128932	2017	Multinational	GLP-1 receptor agonists	362	Semaglutide	0.5 mg weekly	Metformin sulfonylurea	6		Novo Nordisk A/S
**SUSTAIN 4**	NCT02128932	2017	Multinational	GLP-1 receptor agonists	722	Semaglutide	0.5-1.0 mg weekly	Metformin sulfonylurea	6		Novo Nordisk A/S
**SUSTAIN 5**	NCT02305381	2018	Multinational	GLP-1 receptor agonists	131	Semaglutide	1.0 mg weekly	Insulin alone or in combination with metformin	6		Novo Nordisk A/S
**SUSTAIN 5**	NCT02305381	2018	Multinational	GLP-1 receptor agonists	132	Semaglutide	0.5 mg weekly	Insulin alone or in combination with metformin	6		Novo Nordisk A/S
**SUSTAIN 5**	NCT02305381	2018	Multinational	GLP-1 receptor agonists	263	Semaglutide	0.5-1.0 mg weekly	Insulin alone or in combination with metformin	6		Novo Nordisk A/S
**SUSTAIN 5**	NCT02305381	2018	Multinational	Standard treatments	133	Placebo		Insulin alone or in combination with metformin	6		Novo Nordisk A/S

Study ID	Registration	Year	Country	Intervention	N	Drug	Dose	Cointervention	Follow -up*	Settings	Fundings
**SUSTAIN 6**	NCT01720446	2016	Multinational	GLP-1 receptor agonists	1648	Semaglutide	0.5-1.0 mg weekly	No more than two oral hypoglycemic drugs basal or premixed insulin	24		Novo Nordisk A/S
**SUSTAIN 6**	NCT01720446	2016	Multinational	Standard treatments	1649	Placebo		No more than two oral hypoglycemic drugs basal or premixed insulin	24		Novo Nordisk A/S
**SUSTAIN 8**	NCT03136484	2019	Multinational	GLP-1 receptor agonists	394	Semaglutide	1 mg weekly	Metformin	12		Novo Nordisk A/S
**SUSTAIN 8**	NCT03136484	2019	Multinational	SGLT2 inhibitors	394	Canagliflozin	300 mg daily	Metformin	12		Novo Nordisk A/S
**SUSTAIN 9**	NCT03086330	2019	Multinational	GLP-1 receptor agonists	151	Semaglutide	1.0 mg weekly	SGLT-2 inhibitor as monotherapy or with a sulfonylurea or metformin	6		Novo Nordisk A/S
**SUSTAIN 9**	NCT03086330	2019	Multinational	Standard treatments	151	Placebo		SGLT-2 inhibitor as monotherapy or with a sulfonylurea or metformin	6		Novo Nordisk A/S
**SUSTAIN 11**	NCT03689374	2022	Multinational	GLP-1 receptor agonists	874	Semaglutide	1 mg weekly	Metformin	12		Novo Nordisk A/S

Study ID	Registration	Year	Country	Intervention	N	Drug	Dose	Cointervention	Follow -up*	Settings	Fundings
**SUSTAIN 11**	NCT03689374	2022	Multinational	Bolus insulin	874	Insulin aspart	100 U/ml TID	Metformin	12		Novo Nordisk A/S
**SUSTAIN China**	NCT03061214	2021	65 sites in Brazil, the China region (consisting of mainland China,Taiwan and Hong Kong), the Republic of Korea, South Africa and Ukraine	DPP-4 inhibitors	290	sitagliptin	100mg once daily	metformin monotherapy	8.1		Novo Nordisk A/S, Denmark
**SUSTAIN China**	NCT03061214	2021	65 sites in Brazil, the China region (consisting of mainland China,Taiwan and Hong Kong), the Republic of Korea, South	GLP-1 receptor agonists	288	semaglutide	0.5 mg once weekly	metformin monotherapy	8.1		Novo Nordisk A/S, Denmark

Study ID	Registration	Year	Country	Intervention	N	Drug	Dose	Cointervention	Follow -up*	Settings	Fundings
			Africa and Ukraine								
**SUSTAIN China**	NCT03061214	2021	65 sites in Brazil, the China region (consisting of mainland China,Taiwan and Hong Kong), the Republic of Korea, South Africa and Ukraine	GLP-1 receptor agonists	290	semaglutide	1 mg once weekly	metformin monotherapy	8.1		Novo Nordisk A/S, Denmark
**SUSTAIN China**	NCT03061214	2021	65 sites in Brazil, the China region (consisting of mainland China,Taiwan and Hong Kong), the Republic of Korea, South	GLP-1 receptor agonists	578	semaglutide	0.5-1 mg weekly	metformin monotherapy	8.1		Novo Nordisk A/S, Denmark

Study ID	Registration	Year	Country	Intervention	N	Drug	Dose	Cointervention	Follow-up*	Settings	Fundings
			Africa and Ukraine								
**Suzuki 2014**	Not provided	2014	Japan	DPP-4 inhibitors	16	Sitagliptin	50 mg daily	None	6	Coronary artery disease	None
**Suzuki 2014**	Not provided	2014	Japan	GLP-1 receptor agonists	24	Liraglutide	0.9 mg daily	None	6		None
**SWIM**	NCT01341717	2017	India	DPP-4 inhibitors	219	Sitagliptin	100 mg daily	Metformin and insulin	5.5		Merck and Co
**SWIM**	NCT01341717	2017	India	Sulfonylureas	221	Glimepiride	3 mg daily	Metformin and insulin	5.5		Merck and Co
**Takagi 2003**	Not applicable	2003	Japan	Standard treatments	21	Control		Oral hypoglycemic agents or insulin	6		Not reported
**Takagi 2003**	Not applicable	2003	Japan	Thiazolidinediones	23	Pioglitazone	30 mg daily	Oral hypoglycemic agents or insulin	6		Not reported
**Takahara-2020**	UMIN000030208/jRCTs051180036	2020	Japan	SGLT2 inhibitors	20	canagliflozin	100 mg once daily plus	metformin, teneligliptin, glimepiride	5.8		This study was funded by Mitsubishi Tanabe Pharma Corporation
**Takahara-2020**	UMIN000030208/jRCTs051180036	2020	Japan	Standard treatments	20	Control		metformin, teneligliptin, glimepiride	5.8		This study was funded by Mitsubishi Tanabe Pharma Corporation
**Takahashi-2022**	UMIN000015727/jRCTs071180069	2022	Japan	SGLT2 inhibitors	27	ipragliflozin	50 mg once daily	with the exception of SGLT2is,	16.8	NAFLD	Astellas Pharma Inc

Study ID	Registration	Year	Country	Intervention	N	Drug	Dose	Cointervention	Follow -up*	Settings	Fundings
								pioglitazone, or GLP-1 analogs			
**Takahashi-2022**	UMIN000015727/jRCTs071180069	2022	Japan	Standard treatments	28	lifestyle modifications, including diet and exercise therapy, and/or took antidiabetic drugs, with the exception of SGLT2is, pioglitazone, or GLP-1 analogs		with the exception of SGLT2is, pioglitazone, or GLP-1 analogs	16.8		Astellas Pharma Inc
**Takase 2007**	Not provided	2007	Japan	Alpha-glucosidase inhibitors	19	Voglibose	0.6 mg daily	None	6		Not reported
**Takase 2007**	Not provided	2007	Japan	Thiazolidinediones	20	Pioglitazone	15 mg daily	None	6		Not reported
**Takashima 2018**	UMIN000031454	2018	Japan	SGLT2 inhibitors	20	Canagliflozin	100 mg daily	Oral hypoglycemic agents with or without insulin	12	Chronic kidney disease	None
**Takashima 2018**	UMIN000031454	2018	Japan	Standard treatments	20	Control		Oral hypoglycemic agents with or without insulin	12		None

Study ID	Registration	Year	Country	Intervention	N	Drug	Dose	Cointervention	Follow -up*	Settings	Fundings
**Takeshita-2022**	NCT02649465	2022	Japan	SGLT2 inhibitors	20	Tofogliflozin	20 mg daily	None	11		Ministry of Education, Culture, Sports, Science and Japan Agency for Medical Research and Development
**Takeshita-2022**	NCT02649465	2022	Japan	Sulfonylureas	20	Glimepiride	6 mg daily	None	11		Ministry of Education, Culture, Sports, Science and Japan Agency for Medical Research and Development
**Tao 2018**	ChiCTR-IPR-17011120	2018	China	DPP-4 inhibitors	21	Saxagliptin	5 mg daily	None	5.5		National Natural Science Foundation of China [grant number 81200628]; the Chinese Medical Association Clinical Research and Special Funds - Squibb Endocrinology Diabetes Research projects [2012]; the Natural Science Foundation of

Study ID	Registration	Year	Country	Intervention	N	Drug	Dose	Cointervention	Follow-up*	Settings	Fundings
											Shanghai, China [grant number 12ZR1417800]; and the Shanghai Science and Technology Development Fund [grant number 08411953000].
**Tao 2018**	ChiCTR-IPR-17011120	2018	China	Metformin	21	Metformin	2000 mg daily	None	5.5		National Natural Science Foundation of China [grant number 81200628]; the Chinese Medical Association Clinical Research and Special Funds - Squibb Endocrinology Diabetes Research projects [2012]; the Natural Science Foundation of Shanghai, China [grant number 12ZR1417800]; and the Shanghai Science

Study ID	Registration	Year	Country	Intervention	N	Drug	Dose	Cointervention	Follow -up*	Settings	Fundings
											and Technology Development Fund [grant number 08411953000].
**Taskinen 2011**	NCT00601250	2011	Multinational	DPP-4 inhibitors	523	Linagliptin	5 mg daily	Metformin + maximum one other drug	5.5		Boehringer Ingelheim
**Taskinen 2011**	NCT00601250	2011	Multinational	Standard treatments	177	Placebo		Metformin + maximum one other drug	5.5		Boehringer Ingelheim
**TECOS**	NCT00790205	2015	Multinational	DPP-4 inhibitors	7332	Sitagliptin	50-100 mg daily	1-2 oral drugs (metformin, pioglitazone, or sulfonylurea) or insulin (metformin)	36		Merck Sharpe & Dohme
**TECOS**	NCT00790205	2015	Multinational	Standard treatments	7339	Placebo		1-2 oral drugs (metformin, pioglitazone, or sulfonylurea) or insulin (metformin)	36		Merck Sharpe & Dohme
**Teramoto 2007**	Not provided	2007	Japan	Sulfonylureas	46	Glibenclamide	2.5 mg daily	None	5.5		Not reported
**Teramoto 2007**	Not provided	2007	Japan	Thiazolidinediones	46	Pioglitazone	30 mg daily	None	5.5		Not reported

Study ID	Registration	Year	Country	Intervention	N	Drug	Dose	Cointervention	Follow -up*	Settings	Fundings
**Teupe 1991**	Not applicable	1991	Germany	Metformin	50	Metformin	1700 mg daily	None	24		Not reported
**Teupe 1991**	Not applicable	1991	Germany	Standard treatments	50	Control		None	24		Not reported
**Thrasher 2014**	NCT01194830	2014	USA	DPP-4 inhibitors	106	Linagliptin	5 mg daily	None or 1 oral drug	5.5		Boehringer Ingelheim
**Thrasher 2014**	NCT01194830	2014	USA	Standard treatments	120	Placebo		None or 1 oral drug	5.5		Boehringer Ingelheim
**TIDE**	NCT00879970	2012	Multinational	Standard treatments	541	Placebo		Two or fewer glucose-lowering drugs	5.5		GlaxoSmithKline
**TIDE**	NCT00879970	2012	Multinational	Thiazolidinediones	399	Rosiglitazone	8 mg daily	Two or fewer glucose-lowering drugs	5.5		GlaxoSmithKline
**TIDE**	NCT00879970	2012	Multinational	Thiazolidinediones	392	Pioglitazone	45 mg daily	Two or fewer glucose-lowering drugs	5.5		GlaxoSmithKline
**TIDE**	NCT00879970	2012	Multinational	Thiazolidinediones	791	Rosiglitazone or pioglitazone	8 mg or 45 mg daily	Two or fewer glucose-lowering drugs	5.5		GlaxoSmithKline
**Tinahones 2017_1**	NCT01778049	2017	Multinational	DPP-4 inhibitors	126	Linagliptin	5 mg daily	Metformin and emplagliflozin	5.5		Boehringer Ingelheim
**Tinahones 2017_1**	NCT01778049	2017	Multinational	Standard treatments	128	Placebo		Metformin and emplagliflozin	5.5		Boehringer Ingelheim

Study ID	Registration	Year	Country	Intervention	N	Drug	Dose	Cointervention	Follow -up*	Settings	Fundings
**Tinahones 2017_2**	NCT01778049	2017	Multinational	DPP-4 inhibitors	112	Linagliptin	5 mg daily	Metformin and emplagliflozin	5.5		Boehringer Ingelheim
**Tinahones 2017_2**	NCT01778049	2017	Multinational	Standard treatments	112	Placebo		Metformin and emplagliflozin	5.5		Boehringer Ingelheim
**Tofogliflozin 003**	Japic CTI-101349	2014	Japan	SGLT2 inhibitors	55	Tofogliflozin	40 mg daily	None	5.5		Chugai Pharmaceutical Company
**Tofogliflozin 003**	Japic CTI-101349	2014	Japan	SGLT2 inhibitors	55	Tofogliflozin	20 mg daily	None	5.5		Chugai Pharmaceutical Company
**Tofogliflozin 003**	Japic CTI-101349	2014	Japan	SGLT2 inhibitors	55	Tofogliflozin	10 mg daily	None	5.5		Chugai Pharmaceutical Company
**Tofogliflozin 003**	Japic CTI-101349	2014	Japan	SGLT2 inhibitors	165	Tofogliflozin	10-40 mg daily	None	5.5		Chugai Pharmaceutical Company
**Tofogliflozin 003**	Japic CTI-101349	2014	Japan	Standard treatments	55	Placebo		None	5.5		Chugai Pharmaceutical Company
**Tolman 2009**	NCT00494312	2009	USA	Sulfonylureas	1057	Glibenclamide	5-15 mg daily	Metformin sulfonylurea	36		Takeda
**Tolman 2009**	NCT00494312	2009	USA	Thiazolidinediones	1063	Pioglitazone	15-45 mg daily	Metformin sulfonylurea	36		Takeda

Study ID	Registration	Year	Country	Intervention	N	Drug	Dose	Cointervention	Follow -up*	Settings	Fundings
**ToPiND**	jRCTs031180159	2021	Japan	SGLT2 inhibitors	21	tofogliflozin	20mg once daily	metformin and other treatment	5.5	nonalcoholic fatty liver disease	Kowa Co Ltd (Tokyo, Japan)
**ToPiND**	jRCTs031180159	2021	Japan	Thiazolidinediones	19	pioglitazone	15-30 mg once daily	metformin and other treatment	5.5		Kowa Co Ltd (Tokyo, Japan)
**TOPSCORE**	UMIN000017861	2017	Japan	DPP-4 inhibitors	38	Sitagliptin	50 mg daily	Insulin, biguanide, and/or thiazolidine	8.9	Coronary artery disease	Ministry of Education, Culture, Sports, Science and Technology of Japan
**TOPSCORE**	UMIN000017861	2017	Japan	Standard treatments	37	Control		Insulin, biguanide, and/or thiazolidine	8.5		Ministry of Education, Culture, Sports, Science and Technology of Japan
**TOSCA.IT**	NCT00700856	2017	Italy	Sulfonylureas	1493	Sulfonylurea		Metformin	57.3		Italian Medicines Agency, Diabete Ricerca, and Italian Diabetes Society.
**TOSCA.IT**	NCT00700856	2017	Italy	Thiazolidinediones	1535	Pioglitazone	15-45 mg daily	Metformin	57.3		Italian Medicines Agency, Diabete Ricerca, and Italian Diabetes Society.
**Trakarnvanich -2021**	NCT04705506	2021	Thailand	DPP-4 inhibitors	100	gemigliptin	50 mg once daily	metformin and other treatment	6	CKD stages 3 to 4 (estimated glomerular filtration rate	This trial was funded by the Navamindradhiraj University Research

Study ID	Registration	Year	Country	Intervention	N	Drug	Dose	Cointervention	Follow-up*	Settings	Fundings
										(eGFR) 15 to 60 mL/min/1.73 m2), persistent micro- or macroalbuminuria	Fund, and the LG Life Science Company provided the medicine (gemigliptin) for the wholestudy
**Trakarnvanich-2021**	NCT04705506	2021	Thailand	Standard treatments	101	standard treatment		metformin and other treatment	6		This trial was funded by the Navamindradhiraj University Research Fund, and the LG Life Science Company provided the medicine (gemigliptin) for the wholestudy
**Trial 843**	NCT02577003	2021	Japan	SGLT2 inhibitors	73	ipragliflozin	50 mg once daily	sitagliptin and other OHAs	5.5		The study was funded and conducted by MSD K.K., Tokyo, Japan, as a joint development

Study ID	Registration	Year	Country	Intervention	N	Drug	Dose	Cointervention	Follow -up*	Settings	Fundings
											programme with Astellas Pharma Inc.
**Trial 843**	NCT02577003	2021	Japan	Standard treatments	70	placebo		sitagliptin and other OHAs	5.5		The study was funded and conducted by MSD K.K., Tokyo, Japan, as a joint development programme with Astellas Pharma Inc.
**Tripathy 2013**	NCT01223196	2013	USA	Standard treatments	14	Placebo		Metformin and or sulfonylurea	6		Takeda
**Tripathy 2013**	NCT01223196	2013	USA	Thiazolidined iones	15	Pioglitazone	15 mg daily	Metformin and or sulfonylurea	6		Takeda
**TROICA**	NCT01990469	2017	Republic of Korea	DPP-4 inhibitors	108	Gemgliptin	50 mg daily	Metformin + glimepiride	5.5		LG Life Sciences
**TROICA**	NCT01990469	2017	Republic of Korea	Standard treatments	110	Placebo		Metformin + glimepiride	5.5		LG Life Sciences
**Truitt 2010**	NCT00143520	2010	USA	Standard treatments	92	Placebo		Antidiabetes monotherapy or combinations of sulfonylureas, meglitinides, metformin and/or alpha-glucosidase inhibitors	6		Daiichi Sankyo Pharma

Study ID	Registration	Year	Country	Intervention	N	Drug	Dose	Cointervention	Follow-up*	Settings	Fundings
**Truitt 2010**	NCT00143520	2010	USA	Thiazolidinediones	91	Pioglitazone	45 mg daily	Antidiabetes monotherapy or combinations of sulfonylureas, meglitinides, metformin and/or alpha-glucosidase inhibitors	6		Daiichi Sankyo Pharma
**Truitt 2010**	NCT00143520	2010	USA	Thiazolidinediones	86	Rivoglitazone	3 mg daily	Antidiabetes monotherapy or combinations of sulfonylureas, meglitinides, metformin and/or alpha-glucosidase inhibitors	6		Daiichi Sankyo Pharma
**Truitt 2010**	NCT00143520	2010	USA	Thiazolidinediones	85	Rivoglitazone	2 mg daily	Antidiabetes monotherapy or combinations of sulfonylureas, meglitinides, metformin and/or alpha-glucosidase inhibitors	6		Daiichi Sankyo Pharma

Study ID	Registration	Year	Country	Intervention	N	Drug	Dose	Cointervention	Follow -up*	Settings	Fundings
**Truitt 2010**	NCT00143520	2010	USA	Thiazolidined iones	87	Rivoglitazone	1 mg daily	Antidiabetes monotherapy or combinations of sulfonylureas, meglitinides, metformin and/or alpha-glucosidase inhibitors	6		Daiichi Sankyo Pharma
**Truitt 2010**	NCT00143520	2010	USA	Thiazolidined iones	349	Rivoglitazone	1-3 mg daily	Antidiabetes monotherapy or combinations of sulfonylureas, meglitinides, metformin and/or alpha-glucosidase inhibitors	6		Daiichi Sankyo Pharma
**Türkmen Kemal 2007**	Not provided	2009	Turkey	Metformin	16	Metformin	1700 mg daily	None	6		NR
**Türkmen Kemal 2007**	Not provided	2007	Turkey	Standard treatments	17	Control		None	6		NR
**Türkmen Kemal 2007**	Not provided	2008	Turkey	Thiazolidined iones	13	Rosiglitazone	8 mg daliy	None	6		NR
**Tuttolomondo-2021**	NCT03824002	2021	Italy	GLP-1 receptor agonists	60	traditional antidiabetic	5 mg once weekly	metformin and other treatment	9		Unclear

Study ID	Registration	Year	Country	Intervention	N	Drug	Dose	Cointervention	Follow -up*	Settings	Fundings
						treatment plus dulaglutide					
**Tuttolomondo-2021**	NCT03824002	2021	Italy	Standard treatments	64	traditional antidiabetic treatment alone		metformin and other treatment	9		Unclear
**UKPDS 44**	Not applicable	1999	UK	Alpha-glucosidase inhibitors	973	Acarbose	50 mg daily to 100 mg thrice daily	Insulin;Metformin, Sulfonylurea - monotherapy or combination	36		Bayer UK
**UKPDS 44**	Not applicable	1999	UK	Standard treatments	973	Placebo		Insulin;Metformin, Sulfonylurea - monotherapy or combination	36		Bayer UK
**UKPDS 80**	ISRCTN75451837	2008	UK	Metformin	342	Metformin		Unclear	212.4		Medical Research Council, University of Oxford
**UKPDS 80**	ISRCTN75451837	2008	UK	Standard treatments	411	conventional treatment		Unclear	212.4		Medical Research Council, University of Oxford
**Umpierrez 2006**	Not provided	2006	USA	Sulfonylureas	96	Glimepiride	8 mg daily	Metformin	6		Sanofi -Aventis
**Umpierrez 2006**	Not provided	2006	USA	Thiazolidinediones	107	Pioglitazone	45 mg daily	Metformin	6		Sanofi-Aventis
**UTOPIA**	UMIN000017607	2020	Japan	SGLT2 inhibitors	169	tofogliflozin	20 mg once daily	metformin and other treatment	24		Financial support for this study, including

Study ID	Registration	Year	Country	Intervention	N	Drug	Dose	Cointervention	Follow -up*	Settings	Fundings
											the fees for the assistances and for processing of the article by the journal, was provided by Kowa Co., Ltd., Tokyo, Japan
**UTOPIA**	UMIN000017607	2020	Japan	Standard treatments	171	conventional treatment		metformin and other treatment	24		Financial support for this study, including the fees for the assistances and for processing of the article by the journal, was provided by Kowa Co., Ltd., Tokyo, Japan
**Vähätalo 2007**	Not provided	2007	Finland	Metformin	26	Metformin	2500 mg daily	Insulin	12		NR
**Vähätalo 2007**	Not provided	2007	Finland	Sulfonylureas	15	Glipizide	10 mg daily	Insulin	12		NR
**Van Eyk 2019**	NCT02660047	2019	The Netherlands	GLP-1 receptor agonists	22	Liraglutide	1.8 mg daily	Concomitant treatment with metformin, sulfonylurea	5.5		Novo Nordisk A/S

Study ID	Registration	Year	Country	Intervention	N	Drug	Dose	Cointervention	Follow -up*	Settings	Fundings
								derivatives and insulin was optional			
**Van Eyk 2019**	NCT02660047	2019	The Netherlands	Standard treatments	25	Placebo		Concomitant treatment with metformin, sulfonylurea derivatives and insulin was optional	5.5		Novo Nordisk A/S
**Van Gaal 2014**	NCT00976937	2014	Belgium	DPP-4 inhibitors	161	Sitagliptin	100 mg daily	Metformin	5.5		Sanofi
**Van Gaal 2014**	NCT00976937	2014	Belgium	GLP-1 receptor agonists	158	Lixisenatide	20 ug daily	Metformin	5.5		Sanofi
**Vanderheiden 2016**	NCT01505673	2016	USA	GLP-1 receptor agonists	35	Liraglutide	1.8 mg daily	Oral drugs and insulin	6		Novo Nordisk
**Vanderheiden 2016**	NCT01505673	2016	USA	Standard treatments	36	Placebo		Oral drugs and insulin	6		Novo Nordisk
**Varghese 2009**	Not provided	2009	UK	Standard treatments	29	Placebo		Metformin or sulfonylurea	12		Siemens Research support group Novartis
**Varghese 2009**	Not provided	2009	UK	Thiazolidinediones	28	Pioglitazone	15 mg daily	Metformin or sulfonylurea	12	Coronary artery disease	Siemens Research support group Novartis

Study ID	Registration	Year	Country	Intervention	N	Drug	Dose	Cointervention	Follow -up*	Settings	Fundings
**Veleba 2015_1**	EudraCT number 2009-011106-42	2015	Czech Republic	Standard treatments	17	Placebo		Metformin	5.5		Ministry of Health of the Czech Republic (NT13763-4)
**Veleba 2015_1**	EudraCT number 2009-011106-42	2015	Czech Republic	Thiazolidinediones	18	Pioglitazone	15 mg daily	Metformin	5.5		Ministry of Health of the Czech Republic (NT13763-4)
**Veleba 2015_2**	EudraCT number 2009-011106-42	2015	Czech Republic	Standard treatments	17	Placebo		Metformin	5.5		Ministry of Health of the Czech Republic (NT13763-4)
**Veleba 2015_2**	EudraCT number 2009-011106-42	2015	Czech Republic	Thiazolidinediones	17	Pioglitazone	15 mg daily	Metformin	5.5		Ministry of Health of the Czech Republic (NT13763-4)
**VERIFY**	NCT01528254	2019	254 centres across 34 countries	DPP-4 inhibitors	998	vildagliptin	50 mg	metformin	59.8		Novartis
**VERIFY**	NCT01528254	2019	254 centres across 34 countries	Standard treatments	1003	placebo		metformin monotherapy	59.8		Novartis
**VERTIS Asia**	NCT02630706	2019	Multinational	SGLT2 inhibitors	169	Ertugliflozin	15 mg daily	Metformin	6		Merck Sharp and Dohme
**VERTIS Asia**	NCT02630706	2019	Multinational	SGLT2 inhibitors	170	Ertugliflozin	5 mg daily	Metformin	6		Merck Sharp and Dohme
**VERTIS Asia**	NCT02630706	2019	Multinational	SGLT2 inhibitors	339	Ertugliflozin	5-15 mg daily	Metformin	6		Merck Sharp and Dohme

Study ID	Registration	Year	Country	Intervention	N	Drug	Dose	Cointervention	Follow -up*	Settings	Fundings
**VERTIS Asia**	NCT02630706	2019	Multinational	Standard treatments	167	Placebo		Metformin	6		Merck Sharp and Dohme
**VERTIS CV**	NCT01986881	2020	567 centers in 34 countries	SGLT2 inhibitors	2752	ertugliflozin	5 mg once daily	metformin and other treatment	36	established atherosclerotic cardiovascular disease involving the coronary, cerebrovascular, or peripheral arterial systems	Funded by Merck Sharp & Dohme and Pfizer
**VERTIS CV**	NCT01986881	2020	567 centers in 34 countries	SGLT2 inhibitors	2747	ertugliflozin	15 mg once daily	metformin and other treatment	36		Funded by Merck Sharp & Dohme and Pfizer
**VERTIS CV**	NCT01986881	2020	567 centers in 34 countries	SGLT2 inhibitors	5499	ertugliflozin	5-15 mg daily	metformin and other treatment	36		Funded by Merck Sharp & Dohme and Pfizer
**VERTIS CV**	NCT01986881	2020	567 centers in 34 countries	Standard treatments	2747	placebo		metformin and other treatment	36		Funded by Merck Sharp & Dohme and Pfizer
**VERTIS FACTORIAL**	NCT02099110	2017	Multinational	DPP-4 inhibitors	247	Sitagliptin	100 mg daily	Metformin	6		Merck Sharp and Dohme
**VERTIS FACTORIAL**	NCT02099110	2017	Multinational	SGLT2 inhibitors	248	Ertugliflozin	15 mg daily	Metformin	6		Merck Sharp and Dohme

Study ID	Registration	Year	Country	Intervention	N	Drug	Dose	Cointervention	Follow -up*	Settings	Fundings
**VERTIS FACTORIAL**	NCT02099110	2017	Multinational	SGLT2 inhibitors	250	Ertugliflozin	5 mg daily	Metformin	6		Merck Sharp and Dohme
**VERTIS FACTORIAL**	NCT02099110	2017	Multinational	SGLT2 inhibitors	498	Ertugliflozin	5-15 mg daily	Metformin	6		Merck Sharp and Dohme
**VERTIS MET**	NCT02033889	2019	14 countries	SGLT2 inhibitors	207	ertugliflozin	5 mg once daily	Metformin	6		Merck & Co., Inc.
**VERTIS MET**	NCT02033889	2019	14 countries	SGLT2 inhibitors	205	ertugliflozin	15 mg once daily	Metformin	6		Merck & Co., Inc.
**VERTIS MET**	NCT02033889	2019	14 countries	SGLT2 inhibitors	412	ertugliflozin	5-15 mg daily	Metformin	6		Merck & Co., Inc.
**VERTIS MET**	NCT02033889	2019	14 countries	Standard treatments	209	placebo		Metformin	6		Merck & Co., Inc.
**VERTIS MONO**	NCT01958671	2017	Multinational	SGLT2 inhibitors	152	Ertugliflozin	15 mg daily	None	6		Merck Sharp and Dohme
**VERTIS MONO**	NCT01958671	2017	Multinational	SGLT2 inhibitors	156	Ertugliflozin	5 mg daily	None	6		Merck Sharp and Dohme
**VERTIS MONO**	NCT01958671	2017	Multinational	SGLT2 inhibitors	308	Ertugliflozin	5-15 mg daily	None	6		Merck Sharp and Dohme
**VERTIS MONO**	NCT01958671	2017	Multinational	Standard treatments	153	Placebo		None	6		Merck Sharp and Dohme
**VERTIS RENAL**	NCT01986855	2018	Multinational	SGLT2 inhibitors	155	Ertugliflozin	15 mg daily	Antihyperglycemic therapy (monotherapy or combination therapy including	12	Chronic kidney disease	Pfizer/Merck and Co

Study ID	Registration	Year	Country	Intervention	N	Drug	Dose	Cointervention	Follow -up*	Settings	Fundings
								sulfonylureas or insulin)			
**VERTIS RENAL**	NCT01986855	2018	Multinational	SGLT2 inhibitors	158	Ertugliflozin	5 mg daily	Antihyperglycemic therapy (monotherapy or combination therapy including sulfonylureas or insulin)	12		Pfizer/Merck and Co
**VERTIS RENAL**	NCT01986855	2018	Multinational	SGLT2 inhibitors	313	Ertugliflozin	5-15 mg daily	Antihyperglycemic therapy (monotherapy or combination therapy including sulfonylureas or insulin)	12		Pfizer/Merck and Co
**VERTIS RENAL**	NCT01986855	2018	Multinational	Standard treatments	154	Placebo		Antihyperglycemic therapy (monotherapy or combination therapy including sulfonylureas or insulin)	12		Pfizer/Merck and Co
**VERTIS SITA2**	NCT02036515	2018	Multinational	SGLT2 inhibitors	153	Ertugliflozin	15 mg daily	Metformin and sitagliptin	12		Pfizer/Merck and Co

Study ID	Registration	Year	Country	Intervention	N	Drug	Dose	Cointervention	Follow -up*	Settings	Fundings
**VERTIS SITA2**	NCT02036515	2018	Multinational	SGLT2 inhibitors	156	Ertugliflozin	5 mg daily	Metformin and sitagliptin	12		Pfizer/Merck and Co
**VERTIS SITA2**	NCT02036515	2018	Multinational	SGLT2 inhibitors	309	Ertugliflozin	5-15 mg daily	Metformin and sitagliptin	12		Pfizer/Merck and Co
**VERTIS SITA2**	NCT02036515	2018	Multinational	Standard treatments	153	Placebo		Metformin and sitagliptin	12		Pfizer/Merck and Co
**VERTIS SU**	NCT01999218	2018	Multinational	SGLT2 inhibitors	441	Ertugliflozin	15 mg daily	Metformin and sitagliptin	12		Pfizer/Merck and Co
**VERTIS SU**	NCT01999218	2018	Multinational	SGLT2 inhibitors	448	Ertugliflozin	5 mg daily	Metformin and sitagliptin	12		Pfizer/Merck and Co
**VERTIS SU**	NCT01999218	2018	Multinational	SGLT2 inhibitors	889	Ertugliflozin	5-15 mg daily	Metformin and sitagliptin	12		Pfizer/Merck and Co
**VERTIS SU**	NCT01999218	2018	Multinational	Sulfonylureas	437	Glimepiride	8 mg daily	Metformin and sitagliptin	12		Pfizer/Merck and Co
**Vianna 2017**	NCT01679899	2017	Brazil	DPP-4 inhibitors	21	Vildagliptin	100 mg daily	Metformin	12		Novartis Pharmaceuticals
**Vianna 2017**	NCT01679899	2017	Brazil	Sulfonylureas	21	Gliclazide	120 mg daily	Metformin	12		Novartis Pharmaceuticals
**VICTORY**	NCT00169832	2010	Multinational	Standard treatments	95	Placebo		Oral drugs	12		GlaxoSmithKline
**VICTORY**	NCT00169832	2010	Multinational	Thiazolidinediones	98	Rosiglitazone	8 mg daily	Oral drugs	12		GlaxoSmithKline
**VISION**	NCT01541956	2016	China	DPP-4 inhibitors	2501	Vildagliptin	100 mg daily	Metformin	5.5		Novartis Pharmaceuticals

Study ID	Registration	Year	Country	Intervention	N	Drug	Dose	Cointervention	Follow -up*	Settings	Fundings
**VISION**	NCT01541956	2016	China	Standard treatments	484	Standard care		Metformin	5.5		Novartis Pharmaceuticals
**VIVIDD**	NCT00894868	2018	Multinational	DPP-4 inhibitors	128	Vildagliptin	100 mg daily	Drug therapy, if any	12	Heart failure	Novartis Pharmaceuticals
**VIVIDD**	NCT00894868	2018	Multinational	Standard treatments	126	Placebo		Drug therapy, if any	12		Novartis Pharmaceuticals
**Vongthavaravat 2002**	Not applicable	2002	Multinational	Standard treatments	173	Control		Sulfonylurea	6		GlaxoSmithKline
**Vongthavaravat 2002**	Not applicable	2002	Multinational	Thiazolidinediones	175	Rosiglitazone	4 mg daily	Sulfonylurea	6		GlaxoSmithKline
**Wada-2022**	NCT03436693	2022	Japan	SGLT2 inhibitors	154	Canagliflozin	100 mg daily	None	24		Mitsubishi Tanabe Pharma Corporation
**Wada-2022**	NCT03436693	2022	Japan	Standard treatments	154	Placebo		None	24		Mitsubishi Tanabe Pharma Corporation
**Wajcberg 2007**	Not provided	2007	USA	Standard treatments	15	Placebo		None	5.5		Dept of Medicine University of Texas, Takeda Pharmaceuticals
**Wajcberg 2007**	Not provided	2007	USA	Thiazolidinediones	16	Pioglitazone	15-45 mg daily	None	5.5		Dept of Medicine University of Texas, Takeda Pharmaceuticals
**Wang 2005**	N	2005	China	Standard treatments	35	Control		Other antidiabetic drugs	6		Major National Basic Research Program of PR China

Study ID	Registration	Year	Country	Intervention	N	Drug	Dose	Cointervention	Follow -up*	Settings	Fundings
**Wang 2005**	Not applicable	2005	China	Thiazolidinediones	35	Rosiglitazone	4 mg daily	Other antidiabetic drugs	6	Coronary artery disease	Major National Basic Research Program of PR China
**Wang 2013**	Not provided	2013	China	Alpha-glucosidase inhibitors	30	Acarbose	300 mg daily	None	6		Pudong Health Bureau of Shanghai
**Wang 2013**	Not provided	2013	China	Metformin	30	Metformin	1700 mg daily	None	6		Pudong Health Bureau of Shanghai
**Wang 2013**	Not provided	2013	China	Sulfonylureas	30	Gliclazide	120 mg daily	None	6		Pudong Health Bureau of Shanghai
**Wang 2015**	Not provided	2015	China	Alpha-glucosidase inhibitors	45	Acarbose	150 mg daily	Metformin	12		Guangdong Pharmaceutical Association
**Wang 2015**	Not provided	2015	China	DPP-4 inhibitors	45	Saxagliptin	5 mg daily	Metformin	12		Guangdong Pharmaceutical Association
**Wang 2016**	NCT01215097	2016	Multinational	DPP-4 inhibitors	205	Linagliptin	5 mg daily	Metformin	5.5		Boehringer Ingelheim
**Wang 2016**	NCT01215097	2016	Multinational	Standard treatments	101	Placebo		Metformin	5.5		Boehringer Ingelheim
**Wang 2016a**	Not provided	2016	China	SGLT2 inhibitors	18	Dapagliflozin		None	5.5		National Natural Science Foundation of China, Jiangsu Planned Projects of Postdoctoral

Study ID	Registration	Year	Country	Intervention	N	Drug	Dose	Cointervention	Follow -up*	Settings	Fundings
											Research Funds, the Peak of Six Personnel in Jiangsu, and Nanjing Medical Science and Technique Development Foundation
**Wang 2016a**	Not provided	2016	China	Standard treatments	10	Placebo		None	5.5		National Natural Science Foundation of China, Jiangsu Planned Projects of Postdoctoral Research Funds, the Peak of Six Personnel in Jiangsu, and Nanjing Medical Science and Technique Development Foundation
**Wang 2017**	NCT01177384	2017	Multinational	DPP-4 inhibitors	191	Sitagliptin	100 mg daily	Acarbose	5.5		Merck and Co
**Wang 2017**	NCT01177384	2017	Multinational	Standard treatments	190	Placebo		Acarbose	5.5		Merck and Co

Study ID	Registration	Year	Country	Intervention	N	Drug	Dose	Cointervention	Follow-up*	Settings	Fundings
**Wang 2019**	NCT01648582	2018	Multinational	Basal insulin	259	Insulin glargine	Titrated	Metformin and/or sulfonylurea	6		Eli Lilly and Company
**Wang 2019**	NCT01648582	2018	Multinational	GLP-1 receptor agonists	258	Dulaglutide	1.5 mg weekly	Metformin and/or sulfonylurea	6		Eli Lilly and Company
**Wang 2019**	NCT01648582	2018	Multinational	GLP-1 receptor agonists	257	Dulaglutide	0.75 mg weekly	Metformin and/or sulfonylurea	6		Eli Lilly and Company
**Wang 2019**	NCT01648582	2018	Multinational	GLP-1 receptor agonists	515	Dulaglutide	0.75-1.5 mg weekly	Metformin and/or sulfonylurea	6		Eli Lilly and Company
**Wang 2020**	Not provided	2020	China	DPP-4 inhibitors	30	Sitagliptin	100 mg daily	Metformin, sulfonylurea or insulin	6	Stroke	Not reported
**Wang 2020**	Not provided	2020	China	GLP-1 receptor agonists	30	Liraglutide	1.2 mg daily	Metformin, sulfonylurea or insulin	6		Not reported
**Wang 2020b**	ChiCTR-IPR-14005568	2020	China	Basal-Bolus insulin	46	humalog Mix25	The starting doses of humalog Mix25 were 0.4 IU/kg per day (twice	metformin and other treatment	5.5		This study was funded by AstraZeneca and 3SBio Inc.

Study ID	Registration	Year	Country	Intervention	N	Drug	Dose	Cointervention	Follow -up*	Settings	Fundings
							daily) and were then gradually adjusted for target glucose values (fasting plasma glucose (FPG) < 7.0 mmol/L and 2 h plasma blood glucose (2hPBG) < 10.0 mmol/L) by the investigators.				

Study ID	Registration	Year	Country	Intervention	N	Drug	Dose	Cointervention	Follow -up*	Settings	Fundings
**Wang 2020b**	ChiCTR-IPR-14005568	2020	China	GLP-1 receptor agonists	49	exenatide	A 5 μg dose was injected subcutaneously twice daily for 4 weeks, after which a 10 μg dose was injected subcutaneously twice daily for 20 additional weeks	metformin and other treatment	5.5	Normal BMI Patients with Type 2 Diabetes and Visceral Adiposity	This study was funded by AstraZeneca and 3SBio Inc.
**Wang-2020a**	NCT02690883	2020	China	Bolus insulin	46	insulin lispro		insulin glargine, and metformin and other treatment	5.5		We thank AstraZeneca China and 3SBio Inc. for covering the study fees and providing drugs and examination items during the follow-up

Study ID	Registration	Year	Country	Intervention	N	Drug	Dose	Cointervention	Follow -up*	Settings	Fundings
**Wang-2020a**	NCT02690883	2020	China	GLP-1 receptor agonists	46	exenatide	5–10 µg twice daily	insulin glargine, and metformin and other treatment	5.5	Diabetic Kidney Disease	We thank AstraZeneca China and 3SBio Inc. for covering the study fees and providing drugs and examination items during the follow-up
**Wang-2022**	NCT02118376	2022	China	Standard treatments	14	Control group			5.5		Shanghai Tenth People’s Hospital and National Nature Science Foundation of China
**Wang-2022**	NCT02118376	2022	China	DPP-4 inhibitors	17	Sitagliptin	100 mg daily		5.5		Shanghai Tenth People’s Hospital and National Nature Science Foundation of China
**Wang-2022**	NCT02118376	2022	China	Metformin	17	Metformin	500 mg tid		5.5		Shanghai Tenth People’s Hospital and National Nature Science Foundation of China
**Watanabe 2005**	Not applicable	2005	Japan	Sulfonylureas	14	Glibenclamide		None	6		Not reported

Study ID	Registration	Year	Country	Intervention	N	Drug	Dose	Cointervention	Follow-up*	Settings	Fundings
**Watanabe 2005**	Not applicable	2005	Japan	Thiazolidinediones	13	Pioglitazone		None	6		Not reported
**Weng-2021**	NCT04390295	2021	China	SGLT2 inhibitors	166	henagliflozin	5 mg once daily	background metformin treatment (1500 mg/d) with lifestyle (diet and exercise) modification	5.5		This study was funded by Jiangsu Hengrui Medicine
**Weng-2021**	NCT04390295	2021	China	SGLT2 inhibitors	164	henagliflozin	10 mg once daily	background metformin treatment (1500 mg/d) with lifestyle (diet and exercise) modification	5.5		This study was funded by Jiangsu Hengrui Medicine
**Weng-2021**	NCT04390295	2021	China	SGLT2 inhibitors	330	henagliflozin	5-15 mg daily	background metformin treatment (1500 mg/d) with lifestyle (diet and exercise) modification	5.5		This study was funded by Jiangsu Hengrui Medicine
**Weng-2021**	NCT04390295	2021	China	Standard treatments	165	placebo		background metformin treatment (1500 mg/d) with lifestyle	5.5		This study was funded by Jiangsu Hengrui Medicine

Study ID	Registration	Year	Country	Intervention	N	Drug	Dose	Cointervention	Follow -up*	Settings	Fundings
								(diet and exercise) modification			
**Wolffenbuttel 1999**	Not applicable	1999	Multinational	Meglitinides	211	Repaglinide	1.5-12 mg daily	None	12		Not reported
**Wolffenbuttel 1999**	Not applicable	1999	Multinational	Sulfonylureas	109	Glyburide	1.75-10.5 mg daily	None	12		Not reported
**Wolffenbuttel 2000**	Not applicable	2000	Multinational	Standard treatments	192	Placebo		Sulfonylurea	6		SmithKlineBeecham Pharmaceuticals
**Wolffenbuttel 2000**	Not applicable	2000	Multinational	Thiazolidinediones	183	Rosiglitazone	4 mg daily	Sulfonylurea	6		SmithKlineBeecham Pharmaceuticals
**Wolffenbuttel 2000**	Not applicable	2000	Multinational	Thiazolidinediones	199	Rosiglitazone	2 mg daily	Sulfonylurea	6		SmithKlineBeecham Pharmaceuticals
**Wolffenbuttel 2000**	Not applicable	2000	Multinational	Thiazolidinediones	382	Rosiglitazone	2-4 mg daily	Sulfonylurea	6		SmithKlineBeecham Pharmaceuticals
**Wong 2005**	Not applicable	2005	Hong Kong	Standard treatments	26	Control		Insulin	5.5		Not reported
**Wong 2005**	Not applicable	2005	Hong Kong	Thiazolidinediones	26	Rosiglitazone	4 mg daily	Insulin	5.5		Not reported
**Wu 2014**	Not applicable	2014	China	Metformin	47	Metformin	1500 mg daily	None	12		Not reported
**Wu 2014**	Not applicable	2014	China	Thiazolidinediones	46	Pioglitazone	15 mg daily	None	12		Not reported
**Wu 2015**	Not provided	2015	China	DPP-4 inhibitors	34	Linagliptin	5 mg daily	None	5.5		Not reported

Study ID	Registration	Year	Country	Intervention	N	Drug	Dose	Cointervention	Follow -up*	Settings	Fundings
**Wu 2015**	Not provided	2015	China	Standard treatments	23	Placebo		None	5.5		Not reported
**Xiao 2015**	Not provided	2015	China	Sulfonylureas	40	Glipizide	5-10 mg daily	Metformin	5.5		Natural Science Foundation of Anhui Province
**Xiao 2015**	Not provided	2015	China	Thiazolidinediones	40	Pioglitazone	15-45 mg daily	Metformin	5.5		Natural Science Foundation of Anhui Province
**Xiao 2016**	Not provided	2016	China	DPP-4 inhibitors	18	Sitaglipitin	100 mg daily	Metformin	5.5		Chinese Medical Association; Medicine Health Care Science and Technology Development Project Program of Shandong Provience; Science Foundation of Qilu Hospital of Shandong University; Fundamental Research Funds of Shandong University
**Xiao 2016**	Not provided	2016	China	Sulfonylureas	23	Glimepiride	4 mg daily	Metformin	5.5		Chinese Medical Association; Medicine Health Care

Study ID	Registration	Year	Country	Intervention	N	Drug	Dose	Cointervention	Follow-up*	Settings	Fundings
											Science and Technology Development Project Program of Shandong Provience; Science Foundation of Qilu Hospital of Shandong University; Fundamental Research Funds of Shandong University
**Xu 2017**	NCT01709305	2017	China	Alpha-glucosidase inhibitors	551	Acarbose	300 mg daily	Metformin + sitagliptin	5.5		Merck and Company
**Xu 2017**	NCT01709305	2017	China	Meglitinides	545	Repaglinide	16 mg daily	Metformin + sitagliptin	5.5		Merck and Company
**Xu 2017**	NCT01709305	2017	China	Sulfonylureas	549	Glimepiride	6 mg daily	Metformin + sitagliptin	5.5		Merck and Company
**Xu 2017**	NCT01709305	2017	China	Sulfonylureas	550	Gliclazide	120 mg daily	Metformin + sitagliptin	5.5		Merck and Company
**Xu 2017**	NCT01709305	2017	China	Sulfonylureas	1099	Glimepiride or gliclazide	6 mg; 20 mg daily	Metformin + sitagliptin	5.5		Merck and Company
**Xu-2022**	NCT04218734	2022	China	DPP-4 inhibitors	143	Prusogliptin	100 mg daily	Metformin	5.5		CSPC Zhongqi Pharmaceutical Technology

Study ID	Registration	Year	Country	Intervention	N	Drug	Dose	Cointervention	Follow-up*	Settings	Fundings
											(Shijiazhuang) Co., Ltd
**Xu-2022**	NCT04218734	2022	China	Standard treatments	71	Placebo		Metformin	5.5		CSPC Zhongqi Pharmaceutical Technology (Shijiazhuang) Co., Ltd
**Yabiku2017**	ISCTRN33414972	2017	Japan	DPP-4 inhibitors	92	Sitagliptin	100 mg daily	None	6		Not reported
**Yabiku2017**	ISCTRN33414972	2017	Japan	Metformin	92	Metformin	1000 mg daily	None	6		Not reported
**Yabiku2017**	ISCTRN33414972	2017	Japan	Standard treatments	91	Control		None	6		Not reported
**Yabiku2017**	ISCTRN33414972	2017	Japan	Thiazolidinediones	91	Pioglitazone	30 mg daily	None	6		Not reported
**Yagoglu-2020**	Not provided	2020	Turkey	DPP-4 inhibitors	90	linagliptin	5 mg once daily	background insulin therapy	12	chronic kidney disease	Unclear
**Yagoglu-2020**	Not provided	2020	Turkey	Standard treatments	86	Control		background insulin therapy	12		Unclear
**Yale 2013**	Not provided	2013	Multinational	SGLT2 inhibitors	89	Canagliflozin	300 mg daily	None, monotherapy or combination therapy	6	Chronic kidney disease	Janssen Research & Development
**Yale 2013**	Not provided	2013	Multinational	SGLT2 inhibitors	90	Canagliflozin	100 mg daily	None, monotherapy or combination therapy	6		Janssen Research & Development

Study ID	Registration	Year	Country	Intervention	N	Drug	Dose	Cointervention	Follow -up*	Settings	Fundings
**Yale 2013**	Not provided	2013	Multinational	SGLT2 inhibitors	179	Canagliflozin	100-300 mg daily	None, monotherapy or combination therapy	6		Janssen Research & Development
**Yale 2013**	Not provided	2013	Multinational	Standard treatments	90	Placebo		None, monotherapy or combination therapy	6		Janssen Research & Development
**Yamakage 2019**	UMIN000021479	2019	Japan	SGLT2 inhibitors	27	Dapagliflozin	5 mg daily	Background glucose lowering therapy	5.5		AstraZeneca and<br><br>ONO PHARMACEUTICAL CO
**Yamakage 2019**	UMIN000021479	2019	Japan	Standard treatments	27	Control		Background glucose lowering therapy	5.5		AstraZeneca and<br><br>ONO PHARMACEUTICAL CO
**Yamamoto 2018**	UMIN000028313	2018	Japan	Bolus insulin	13	Insulin lispro/Aspart/Glulisine	Titrated	Basal insulin with or without oral hypoglycemic agents	5.5		None
**Yamamoto 2018**	UMIN000028313	2018	Japan	GLP-1 receptor agonists	12	Liraglutide	0.9 mg daily	Basal insulin with or without oral hypoglycemic agents	5.5		None

Study ID	Registration	Year	Country	Intervention	N	Drug	Dose	Cointervention	Follow -up*	Settings	Fundings
**Yamanouchi 2005**	Not applicable	2005	Japan	Metformin	39	Metformin	750 mg daily	None	12		Not reported
**Yamanouchi 2005**	Not applicable	2005	Japan	Sulfonylureas	37	Glimepiride	1-2 mg daily	None	12		Not reported
**Yamanouchi 2005**	Not applicable	2005	Japan	Thiazolidinediones	38	Pioglitazone	30-45 mg daily	None	12		Not reported
**Yamasaki 2005**	Not applicable	2005	Japan	Alpha-glucosidase inhibitors	53	Voglibose	0.4-0.6 mg daily	Diet; Sulphonylurea; Insulin	39.6		Not reported
**Yamasaki 2005**	Not applicable	2005	Japan	Standard treatments	50	Control		Diet; Sulphonylurea; Insulin	39.6		Not reported
**Yan 2019**	NCT02147925	2019	China	Basal insulin	24	Insulin glargine	Titrated	Metformin	6		Novo Nordisk
**Yan 2019**	NCT02147925	2019	China	DPP-4 inhibitors	27	Sitagliptin	100 mg daily	Metformin	6		Novo Nordisk
**Yan 2019**	NCT02147925	2019	China	GLP-1 receptor agonists	24	Liraglutide	1.8 mg daily	Metformin	6		Novo Nordisk
**Yang 2002**	Not applicable	2002	Unclear	Standard treatments	34	Placebo		Sulphonylurea	6		Smith-Kline Beecham; Department of Education of Republic of China
**Yang 2002**	Not applicable	2002	Unclear	Thiazolidinediones	30	Rosiglitazone	2 mg twice daily	Sulphonylurea	6		Smith-Kline Beecham;

Study ID	Registration	Year	Country	Intervention	N	Drug	Dose	Cointervention	Follow -up*	Settings	Fundings
											Department of Education of Republic of China
**Yang 2011**	NCT00661362	2011	Multinational	DPP-4 inhibitors	283	Saxagliptin	5 mg daily	Metformin	5.5		AstraZeneca; Bristol-Meyers Squibb
**Yang 2011**	NCT00661362	2011	Multinational	Standard treatments	287	Placebo		Metformin	5.5		AstraZeneca; Bristol-Meyers Squibb
**Yang 2012**	NCT00813995	2012	China	DPP-4 inhibitors	197	Sitagliptin	100 mg daily	Metformin	5.5		Merck Sharp & Dohme
**Yang 2012**	NCT00813995	2012	China	Standard treatments	198	Placebo		Metformin	5.5		Merck Sharp & Dohme
**Yang 2013**	Not provided	2013	Republic of Korea and India	DPP-4 inhibitors	90	Gemgliptin	50 mg daily	None	5.5		LG Life Sciences Ltd
**Yang 2013**	Not provided	2013	Republic of Korea and India	Standard treatments	92	Placebo		None	5.5		LG Life Sciences Ltd
**Yang 2015**	Not provided	2015	Korea	DPP-4 inhibitors	37	Anagliptin	200 mg daily	None	5.5		JW Pharmaceutical Corporation
**Yang 2015**	Not provided	2015	Korea	DPP-4 inhibitors	33	Anagliptin	100 mg daily	None	5.5		JW Pharmaceutical Corporation
**Yang 2015**	Not provided	2015	Korea	DPP-4 inhibitors	70	Anagliptin	100-200 mg daily	None	5.5		JW Pharmaceutical Corporation
**Yang 2015**	Not provided	2015	Korea	Standard treatments	39	Placebo		None	5.5		JW Pharmaceutical Corporation

Study ID	Registration	Year	Country	Intervention	N	Drug	Dose	Cointervention	Follow -up*	Settings	Fundings
**Yang 2015a**	NCT01357252	2015	China	DPP-4 inhibitors	143	Vildagliptin	50 mg daily	Sulfonylurea	5.5		Novartis Pharma
**Yang 2015a**	NCT01357252	2015	China	Standard treatments	136	Placebo		Sulfonylurea	5.5		Novartis Pharma
**Yang 2016**	NCT01095666	2016	Multinational	SGLT2 inhibitors	152	Dapagliflozin	10 mg daily	Metformin	5.5		Bristol-Myers Squibb; AstraZeneca
**Yang 2016**	NCT01095666	2016	Multinational	SGLT2 inhibitors	147	Dapagliflozin	5 mg daily	Metformin	5.5		Bristol-Myers Squibb; AstraZeneca
**Yang 2016**	NCT01095666	2016	Multinational	SGLT2 inhibitors	299	Dapagliflozin	5-10 mg daily	Metformin	5.5		Bristol-Myers Squibb; AstraZeneca
**Yang 2016**	NCT01095666	2016	Multinational	Standard treatments	145	Placebo		Metformin	5.5		Bristol-Myers Squibb; AstraZeneca
**Yang 2018**	NCT02096705	2018	Multinational	SGLT2 inhibitors	139	Dapagliflozin	10 mg daily	Insulin with or without oral antidiabetic drugs	5.5		AstraZeneca
**Yang 2018**	NCT02096705	2018	Multinational	Standard treatments	133	Placebo		Insulin with or without oral antidiabetic drugs	5.5		AstraZeneca
**Yang-2021**	NCT02897349	2021	China	DPP-4 inhibitors	104	linagliptin	5 mg once daily	background insulin (basal: insulin glargine, insulin detemir, neutral protamine Hagedorn [NPH] insulin; premixed:	5.5		Boehringer Ingelheim

Study ID	Registration	Year	Country	Intervention	N	Drug	Dose	Cointervention	Follow -up*	Settings	Fundings
								25/75 or 30/70 ratio), with or without metformin			
**Yang-2021**	NCT02897349	2021	China	Standard treatments	102	placebo		background insulin (basal: insulin glargine, insulin detemir, neutral protamine Hagedorn [NPH] insulin; premixed: 25/75 or 30/70 ratio), with or without metformin	5.5		Boehringer Ingelheim
**Yee 2010**	NCT00231387	2010	England	Standard treatments	29	Placebo		1-2 drug therapy excluding thiazolidinedione or insulin	12		GlaxoSmithKline
**Yee 2010**	NCT00231387	2010	England	Thiazolidinediones	28	Rosiglitazone	4-8 mg daily	1-2 drug therapy excluding thiazolidinedione or insulin	12	High cardiovascular risk	GlaxoSmithKline
**Yki-Jarvinen 1999**	Not applicable	1999	Finland	Metformin	19	Metformin	2000 mg daily	Sulfonylurea	12		Academy of Finland
**Yki-Jarvinen 1999**	Not applicable	1999	Finland	Sulfonylureas	22	Glyburide	10.5 mg daily	Sulfonylurea	12		Academy of Finland

Study ID	Registration	Year	Country	Intervention	N	Drug	Dose	Cointervention	Follow -up*	Settings	Fundings
**Yki-Jarvinen 2013**	NCT00954447	2013	Multinational	DPP-4 inhibitors	631	Linagliptin	5 mg daily	Insulin metformin and/or thiazolidinedione	12		Boehringer Ingelheim
**Yki-Jarvinen 2013**	NCT00954447	2013	Multinational	Standard treatments	630	Placebo		Insulin metformin and/or thiazolidinedione	12		Boehringer Ingelheim
**Yoon 2011**	NCT00397631	2011	Multinational	DPP-4 inhibitors	261	Sitagliptin	100 mg daily	None	5.5		Merck & Co
**Yoon 2011**	NCT00397631	2011	Multinational	Standard treatments	259	Placebo		None	5.5		Merck & Co
**Yoon 2011a**	Not provided	2011	Republic of Korea	Metformin	114	Metformin	2000 mg daily	None	11		Korean Diabetes Association
**Yoon 2011a**	Not provided	2011	Republic of Korea	Sulfonylureas	118	Glimepiride	8 mg daily	None	11		Korean Diabetes Association
**Yoon 2011a**	Not provided	2011	Republic of Korea	Thiazolidinediones	117	Rosiglitazone	8 mg daily	None	11		Korean Diabetes Association
**Yuan 2012**	Not provided	2012	China	GLP-1 receptor agonists	33	Exenatide	20 ug daily	None	6		Not reported
**Yuan 2012**	Not provided	2012	China	Metformin	26	Metformin	1500 mg daily	None	6		Not reported
**Zang 2016**	NCT02008682	2016	China	DPP-4 inhibitors	184	Sitagliptin	100 mg daily	Metformin	6		Novo Nordisk

Study ID	Registration	Year	Country	Intervention	N	Drug	Dose	Cointervention	Follow-up*	Settings	Fundings
**Zang 2016**	NCT02008682	2016	China	GLP-1 receptor agonists	184	Liraglutide	1.8 mg daily	Metformin	6		Novo Nordisk
**ZEUS II**	NCT02831361	2020	Korea and Thailand	DPP-4 inhibitors	188	Gemgliptin	50 mg daily	Insulin with or without metformin	5.5		LG Chem Ltd
**ZEUS II**	NCT02831361	2020	Korea and Thailand	Standard treatments	95	Placebo		Insulin with or without metformin	5.5		LG Chem Ltd
**Zhang 2020**	Not provided	2020	China	GLP-1 receptor agonists	30	Liraglutide	1.2 mg daily	Metformin	5.5		Yantai Affiliated Hospital ofBinzhou Medical University.
**Zhang 2020**	Not provided	2020	China	Thiazolidinediones	30	Piolglitazone	30 mg daily	Metformin	5.5		Yantai Affiliated Hospital ofBinzhou Medical University.
**Zhang 2020a**	ChiCTR-1800015658	2020	China	Basal-Bolus insulin	33	aspartate 70/30 insulin	The initial dose of insulin was 0.2–0.4 IU/kg per day and was titrated according to self-monitoring	All of the included patients were free to take hypoglycemic drugs except for sulfonylureas and nateglinide drugs	12		This study was sponsored by AstraZeneca China and 3SBio Inc.

Study ID	Registration	Year	Country	Intervention	N	Drug	Dose	Cointervention	Follow -up*	Settings	Fundings
							blood glucose				
**Zhang 2020a**	ChiCTR-1800015658	2020	China	GLP-1 receptor agonists	33	exenatide	5–10 μg twice daily	All of the included patients were free to take hypoglycemic drugs except for sulfonylureas and nateglinide drugs	12		This study was sponsored by AstraZeneca China and 3SBio Inc.
**Zhou-2022**	Not provided	2022	USA	Thiazolidinediones	16	Pioglitazone	10 mg daily	Metformin	5.5		Not provided
**Zhou-2022**	Not provided	2022	USA	Standard treatments	16	Control		Metformin	5.5		Not provided
**Zhu 2003**	Not applicable	2003	China	Standard treatments	112	Placebo		Sulfonylurea	5.5		SmithKline Beecham
**Zhu 2003**	Not applicable	2003	China	Thiazolidinediones	221	Rosiglitazone	8 mg daily	Sulfonylurea	5.5		SmithKline Beecham
**Zhu 2003**	Not applicable	2003	China	Thiazolidinediones	221	Rosiglitazone	4 mg daily	Sulfonylurea	5.5		SmithKline Beecham
**Zhu 2003**	Not applicable	2003	China	Thiazolidinediones	442	Rosiglitazone	4-8 mg daily	Sulfonylurea	5.5		SmithKline Beecham
**Zib 2007**	Not provided	2007	USA	Standard treatments	16	Control		Insulin	6		NIH, American diabetes Association, Takeda Pharmaceuticals, Heart and Stroke

Study ID	Registration	Year	Country	Intervention	N	Drug	Dose	Cointervention	Follow-up*	Settings	Fundings
											Foundation of Canada, Canadian Institue for Health Research, Canadian Diabetes Association
**Zib 2007**	Not provided	2007	USA	Thiazolidinediones	16	Pioglitazone	15-30 mg daily	Insulin	6		NIH, American diabetes Association, Takeda Pharmaceuticals, Heart and Stroke Foundation of Canada, Canadian Institue for Health Research, Canadian Diabetes Association

Note: *Follow-ups were shown as months; N, no. of patients.

2.2 Patients' characteristics

Study ID	N	Intervention	Age (mean)	Age (SD)	% of males	HbA1c (mean)	HbA1c (SD)	BMI (mean)	BMI (SD)	Duration of diabetes	% of CV disease	eGFR	ACR	ACR unit
**1860-LIRA-DPP-4**	219	DPP-4 inhibitors	55	9	55	8.5	0.7	32.6	5.4	6.3				
**1860-LIRA-DPP-4**	221	GLP-1 receptor agonists	55	9.1	52	8.4	0.7	33.1	5.1	6.4				
**1860-LIRA-DPP-4**	225	GLP-1 receptor agonists	55.9	9.6	52	8.4	0.8	32.6	5.2	6				
**3D**	40	DPP-4 inhibitors	67.8	10.5	50	7.1	0.7	27.7	4.1	48	13	75		
**3D**	40	Alpha-glucosidase inhibitors	66.7	9.8	65	6.9	0.5	25.7	4.3	38.5	5	71		
**4B**	315	GLP-1 receptor agonists	59.5	9.6	52	8.3	1	32.7	4.7	12				
**4B**	312	Bolus insulin	59.4	9.3	51	8.2	1	32.3	4.7	11				
**4T**	234	Basal insulin	61.9	10	61.1	8.4	0.8	29.7	4.6	9				
**4T**	239	Bolus insulin	61.6	10.5	63.6	8.6	0.8	29.6	4.5	9				
**Abdullah-2021**	110	DPP-4 inhibitors	55	14.2	60	7.8	0.96			6	1	72		
**Abdullah-2021**	110	SGLT2 inhibitors	57	12.8	60.4	7.8	0.73			6	1	76		
**Abe 2008**	31	Thiazolidinediones	65.2	12.1	67.7			22.2	2.5	16.6				
**Abe 2008**	32	Standard treatments	67.2	9.4	68.9			22.2	2.7	16.3				
**Abe 2016**	41	DPP-4 inhibitors	66.9	9.4	65.9	6.5	0.8	22.8	3.7		17.1			
**Abe 2016**	41	Standard treatments	66.3	9.3	68.3	6.5	0.7	22.9	3.7		14.6			
**ACTION-J**	26	Thiazolidinediones	56	9.7	81.8	8.6	1.3	25.5	3.1	13.4				
**ACTION-J**	28	Standard treatments	57.2	11.3	69.2	8.6	1.2	26.9	4.4	14.8				
**Adel-2022**	52	SGLT2 inhibitors												
**Adel-2022**	54	Standard treatments												

Study ID	N	Intervention	Age (mean)	Age (SD)	% of males	HbA1c (mean)	HbA1c (SD)	BMI (mean)	BMI (SD)	Duration of diabetes	% of CV disease	eGFR	ACR	ACR unit
**ADOPT**	1455	Metformin	57.9	9.9	59.4	7.4	0.9	32.1	6.1					
**ADOPT**	1447	Sulfonylureas	56.4	10.2	58	7.4	0.9	32.2	6.3					
**ADOPT**	1458	Thiazolidinediones	56.3	10	55.7	7.4	0.9	32.2	6.7					
**Ahmann 2015**	225	GLP-1 receptor agonists	59.3	9.2	53.3	8.2	0.8	32.3	5.6	12.1				
**Ahmann 2015**	225	Standard treatments	57.5	11.1	60.4	8.3	0.9	32.2	5.7	12.1				
**Ahrén 2004**	42	DPP-4 inhibitors	58.4	9.2	61.9	7.6	0.6	29.6	3.7	5.8				
**Ahrén 2004**	29	Standard treatments	54.3	12.2	75.9	7.8	0.6	29.9	3.6	4.6				
**Allegretti 2019**	157	SGLT2 inhibitors	69.3	8.4	58.6	8	0.8	30.3	6	15.5		45		
**Allegretti 2019**	155	Standard treatments	69.9	8.3	67.1	8	0.8	30.1	5.8	16.3		45		
**Alogliptin Study 007**	198	DPP-4 inhibitors	56.5	11.7	50	8.1	0.9	30	4.8	7.6				
**Alogliptin Study 007**	203	DPP-4 inhibitors	56.5	11.1	54.7	8.1	0.8	30.2	4.8	7.8				
**Alogliptin Study 007**	99	Standard treatments	57.1	10	51.5	8.2	0.9	30	5.3	7.7				
**Alogliptin Study 009**	199	DPP-4 inhibitors	55.4	10.2	62.8	8	0.8	33.1	5.4	7.4				
**Alogliptin Study 009**	197	DPP-4 inhibitors	55.5	9.4	55.3	8.1	0.9	32.3	5.7	7.7				
**Alogliptin Study 009**	97	Standard treatments	55.2	10.8	54.6	8	0.8	33.2	6.2	7.8				
**Alogliptin Study 010**	131	DPP-4 inhibitors												
**Alogliptin Study 010**	133	DPP-4 inhibitors												
**Alogliptin Study 010**	64	Standard treatments												
**Alvarsson 2003**	28	Sulfonylureas	55.6	7.3	71.4	6.9	0.9	27.8	3.7					
**Alvarsson 2003**	23	Basal-Bolus insulin	51.1	7.2	61.1	7.3	1.7	27.3	3					
**AMPLITUDE-M**	100	GLP-1 receptor agonists	58.6	10.5	55	8.1	0.9	34.4	6.4	5.3		101.3		

Study ID	N	Intervention	Age (mean)	Age (SD)	% of males	HbA1c (mean)	HbA1c (SD)	BMI (mean)	BMI (SD)	Duration of diabetes	% of CV disease	eGFR	ACR	ACR unit
**AMPLITUDE-M**	101	GLP-1 receptor agonists	56.3	11.5	51.5	8.1	0.9	33.8	6.6	4.9		106.1		
**AMPLITUDE-M**	103	GLP-1 receptor agonists	59.6	10.7	59.2	8.1	1	33.8	6.9	5.2		102.3		
**AMPLITUDE-M**	304	GLP-1 receptor agonists												
**AMPLITUDE-M**	102	Standard treatments	59.5	11.7	50	8	0.9	34.8	7.1	5		95.3		
**AMPLITUDE-O**	2717	GLP-1 receptor agonists	64.6	8.2	66	8.9	1.46	32.9	6.2	15.6	89.1	72.2	52.2	
**AMPLITUDE-O**	1359	Standard treatments	64.4	8.3	69.2	8.94	1.52	32.4	6	15.1	90.5	72.9	48.2	
**Ando-2021**	20	SGLT2 inhibitors	55.9	13	76.5	6.8	0.7	27	3.3	10.4		74.1		
**Ando-2021**	20	GLP-1 receptor agonists	58.2	11.5	76.5	6.4	0.6	26.8	3.1	7.8		74.8		
**APOLLO**	205	Basal insulin	60	9	52	8.7	1	29.2	3.7	9				
**APOLLO**	210	Bolus insulin	59.7	9	59	8.7	1	29.4	3.5	8.5				
**Apovian 2010**	96	GLP-1 receptor agonists	54.5	10	37	7.7	0.9	33.6	3.7	5.7				
**Apovian 2010**	98	Standard treatments	55.1	9	38	7.5	0.8	33.9	4.3	5.3				
**APPROACH**	339	Sulfonylureas	60.2	9	65.8	7.2	0.9	29.8	5.3					
**APPROACH**	333	Thiazolidinediones	61.8	8.4	70	7.1	0.8	29.3	5.5					
**APRIME**	31	Metformin	62.5	10.2	75	7.9	1.1	24.5	3.4	9.5	0	75	111	
**APRIME**	32	Thiazolidinediones	62.4	8.4	67.7	8	1.1	25.6	3.3	11.6	0	79	143	
**Araki 2015**	63	Metformin	60	10.2	75	7.9	0.8	25.2	3.6			87		
**Araki 2015**	137	SGLT2 inhibitors	61.8	9.6	70	8.1	0.8	25.2	4.2			86		

Study ID	N	Intervention	Age (mean)	Age (SD)	% of males	HbA1c (mean)	HbA1c (SD)	BMI (mean)	BMI (SD)	Duration of diabetes	% of CV disease	eGFR	ACR	ACR unit
**Araki 2015**	136	SGLT2 inhibitors	61.3	9.9	73	8	0.7	24.6	3.8			87		
**Araki 2015a**	181	GLP-1 receptor agonists	57.5	10.5	69	8.1	0.8	26.1	3.6	8.9				
**Araki 2015a**	180	Basal insulin	56.1	11.3	74	8	0.9	25.9	3.9	8.8				
**Arechavaleta 2011**	519	Sulfonylureas	56.2	10.1	53.8	7.5	0.8	30.2	4.4	6.7				
**Arechavaleta 2011**	516	DPP-4 inhibitors	56.3	9.7	55	7.5	0.7	29.7	4.5	6.8				
**Arjona Ferreira 2013**	65	Sulfonylureas	58.5	9.9	56.9	7.8	0.7	26.3	4.3		27.7			
**Arjona Ferreira 2013**	64	DPP-4 inhibitors	60.5	9.1	62.5	7.9	0.7	27.3	5.7		32.8			
**Arjona Ferreira 2013a**	212	Sulfonylureas	64.3	9.2	54.9	7.8	0.7	27	4.8	10.1				
**Arjona Ferreira 2013a**	211	DPP-4 inhibitors	64.8	10.6	59.3	7.8	0.7	26.5	4.8	10.7				
**Arturi 2017**	12	Basal insulin	60	8	75	7.9	0.8	30.8	6					
**Arturi 2017**	10	DPP-4 inhibitors	60.5	10	60	8.3	0.9	30.9	2.8					
**Arturi 2017**	10	GLP-1 receptor agonists	59.5	9	70	8.2	1	33.2	2					
**Asian Acarbose Study**	59	Alpha-glucosidase inhibitors	54	10	51.6	8.6	1.1	25.6	3.8	2.1				
**Asian Acarbose Study**	62	Standard treatments	52.8	10.2	51.6	8.2	1	25.4	3.9	2.7				
**Aso 2019**	33	SGLT2 inhibitors												
**Aso 2019**	24	Standard treatments												
**Avilés-Santa 1999**	21	Metformin	53.1	9.4	28.6	9	1.4			9.2				
**Avilés-Santa 1999**	22	Standard treatments	54.6	7.8	45.5	9.1	1.5			10.1				

Study ID	N	Intervention	Age (mean)	Age (SD)	% of males	HbA1c (mean)	HbA1c (SD)	BMI (mean)	BMI (SD)	Duration of diabetes	% of CV disease	eGFR	ACR	ACR unit
**AWARD-1**	276	GLP-1 receptor agonists	55	10	57	8.1	1.3	34	5	9				
**AWARD-1**	279	GLP-1 receptor agonists	56	10	58	8.1	1.3	33	5	9				
**AWARD-1**	280	GLP-1 receptor agonists	56	9	60	8.1	1.2	33	6	9				
**AWARD-1**	141	Standard treatments	55	10	59	8.1	1.3	33	6	9				
**AWARD-10**	142	GLP-1 receptor agonists	56.2	9.3	54	8	0.7	32.9	5.6	9.2				
**AWARD-10**	142	GLP-1 receptor agonists	58.6	9.1	49	8	0.6	32.77	6.3	10.1				
**AWARD-10**	140	Standard treatments	57.1	9.6	47	8.1	0.7	32.39	5	8.9				
**AWARD-2**	273	GLP-1 receptor agonists	56	10	53	8.2	1	31	5	9				
**AWARD-2**	272	GLP-1 receptor agonists	57	9	50	8.1	1	32	5	9				
**AWARD-2**	262	Basal insulin	57	9	52	8.1	1	32	6	9				
**AWARD-3**	268	Metformin	55	10	45	7.6	0.8	33	5	3				
**AWARD-3**	269	GLP-1 receptor agonists	56	10	42	7.6	0.9	34	6	3				
**AWARD-3**	270	GLP-1 receptor agonists	56	11	44	7.6	0.9	33	6	3				
**AWARD-4**	295	GLP-1 receptor agonists	58.9	9.6	54	8.5	1.1	32	5.1	12.8				

Study ID	N	Intervention	Age (mean)	Age (SD)	% of males	HbA1c (mean)	HbA1c (SD)	BMI (mean)	BMI (SD)	Duration of diabetes	% of CV disease	eGFR	ACR	ACR unit
**AWARD-4**	293	GLP-1 receptor agonists	59.3	9	50	8.4	1	33.1	5.2	12.4				
**AWARD-4**	296	Basal insulin	59.9	9.1	56	8.5	1	32.4	5.3	13				
**AWARD-5**	315	DPP-4 inhibitors	54	10	48	8.1	1.1	31	4	7				
**AWARD-5**	304	GLP-1 receptor agonists	54	10	48	8.1	1.1	31	5	7				
**AWARD-5**	302	GLP-1 receptor agonists	54	10	44	8.2	1.1	31	4	7				
**AWARD-5**	177	Standard treatments	55	9	51	8.1	1.1	31	4	7				
**AWARD-7**	193	GLP-1 receptor agonists	64.7	8.8	54	8.6	0.9	32.1	4.8	17.6		38.1	375.8333	mg/g
**AWARD-7**	190	GLP-1 receptor agonists	64.7	8.6	55	8.6	1.1	33	5.5	18		38.3	405.6	mg/g
**AWARD-7**	194	Basal insulin	64.3	8.4	48	8.6	1	32.4	5.3	18.7		38.5	413.6	mg/g
**AWARD-8**	239	GLP-1 receptor agonists	57.7	10.2	43.5	8.4	0.7	30.9	5.2	7.8				
**AWARD-8**	60	Standard treatments	58.2	7.4	46.7	8.4	0.7	32.4	5.9	6.8				
**AWARD-9**	150	GLP-1 receptor agonists	60.2	9.5	56.7	8.4	0.9	32.8	4.9	13				
**AWARD-9**	150	Standard treatments	60.6	10.1	58.7	8.3	0.8	32.6	4.9	13.3				
**AWARD-CHN1**	245	Sulfonylureas	52	10.1	53.7	7.9	1	25.7	3.1	3.8				
**AWARD-CHN1**	244	GLP-1 receptor agonists	52.7	10.8	56.1	8	1	25.8	3.4	4				

Study ID	N	Intervention	Age (mean)	Age (SD)	% of males	HbA1c (mean)	HbA1c (SD)	BMI (mean)	BMI (SD)	Duration of diabetes	% of CV disease	eGFR	ACR	ACR unit
**AWARD-CHN1**	248	GLP-1 receptor agonists	53.8	10.1	53.1	8	1	26.2	3.5	3.5				
**Ba 2017**	249	DPP-4 inhibitors	57.5	9.5	47	8.6	1.1	25.4	3.2	7.1				
**Ba 2017**	249	Standard treatments	56.5	9.3	53	8.5	0.9	25.3	3.2	6.9				
**Babar-2021**	120	SGLT2 inhibitors	53.4	9.1	91.7	7.9	0.9							
**Babar-2021**	120	Standard treatments	52.5	8.6	88.3	8	0.7							
**Bachmann 2003**	184	Alpha-glucosidase inhibitors	63.8	7.1	47.6	9.4	0.7	29	3.1					
**Bachmann 2003**	189	Standard treatments	63.3	7.2	43.4	9.4	0.7	29	2.9					
**Bae-2021**	60	SGLT2 inhibitors	61.83	10.15	60	7.94	0.74	26.7	3.73	13.86		86.88		
**Bae-2021**	59	Thiazolidinediones	61.88	9.7	55.9	8	0.78	26.24	3.71	13.92		86.32		
**Bailey 2010**	135	SGLT2 inhibitors	52.7	9.9	57	7.9	0.8	31.2	5.1	6.1				
**Bailey 2010**	137	SGLT2 inhibitors	54.3	9.4	50	8.2	1	31.4	5	6.4				
**Bailey 2010**	137	SGLT2 inhibitors	55	9.3	51	8	0.9	31.6	4.8	6				
**Bailey 2010**	137	Standard treatments	53.7	10.3	55	8.1	1	31.8	5.3	5.8				
**Bailey 2012**	68	SGLT2 inhibitors	51.3	11.5	47.1	7.9	1	31	5.7	1.4				
**Bailey 2012**	74	SGLT2 inhibitors	53.5	10.6	45.9	8.1	1.1	31.1	5.5	1.5				
**Bailey 2012**	72	SGLT2 inhibitors	53.7	9	52.8	7.8	1	32.5	5.7	1.6				
**Bailey 2012**	68	Standard treatments	53.5	11.1	54.4	7.8	1.1	32.5	4.9	1.1				
**Bajaj 2014**	183	DPP-4 inhibitors	53.1	9.7	45.4	8.4	0.8	28.2	5.2					
**Bajaj 2014**	89	Standard treatments	55.2	8.4	55.1	8.5	0.8	28.1	5.5					
**Bakris 2006**	185	Sulfonylureas	58.8	9.8	69	8.3	1.6	31.8	6	7.6			63.5	
**Bakris 2006**	204	Thiazolidinediones	60	9.6	63	8.5	1.7	31.6	5.6	8			65.9	
**BALANCE**	96	DPP-4 inhibitors	55.5	11	56.4	7.8	0.8	25.6	3.6	6.2				

Study ID	N	Intervention	Age (mean)	Age (SD)	% of males	HbA1c (mean)	HbA1c (SD)	BMI (mean)	BMI (SD)	Duration of diabetes	% of CV disease	eGFR	ACR	ACR unit
**BALANCE**	97	Standard treatments	56.2	9.2	51	7.8	0.8	25.2	3.3	6.8				
**Banerji 1995**	10	Sulfonylureas			70	14	3	26.7	2.5					
**Banerji 1995**	10	Standard treatments			30	12	2	27.5	3.2					
**Barnett 2003**	84	Thiazolidinediones	54.1	9.391714	75	9.1	1.3	26.4	5.104192	6.5				
**Barnett 2003**	87	Standard treatments	54.3	9.850848	80	9.2	1.3	26.8	3.694068	6.5				
**Barnett 2012**	304	DPP-4 inhibitors	57.2	9.4	40	8.7	0.9	32.6	5.7	11.8				
**Barnett 2012**	151	Standard treatments	57.3	9.3	45	8.6	0.9	31.8	4.8	12.2				
**Barnett 2013**	162	DPP-4 inhibitors	74.9	4.4	71.6	7.8	0.8	29.6	4.7					
**Barnett 2013**	79	Standard treatments	74.9	4.2	62	7.7	0.7	29.8	4.5					
**Barzilai 2011**	102	DPP-4 inhibitors	71.6	6.1	47	7.8	0.8	30.8	5.9	7.2		70		
**Barzilai 2011**	104	Standard treatments	72.1	6	47	7.8	0.7	31.1	7.2	7		72		
**Bayrasheva 2020**	23	DPP-4 inhibitors	61	3.950862	39.1	7.266667	0.553121	32.26667	3.634793	8		90	27.2	mg/g
**Bayrasheva 2020**	21	Standard treatments	60	9.540404	42.9	7.133333	0.795034	31.63333	5.883249	10		91	26.7	mg/g
**BEGIN: ADD TO GLP-1**	174	Basal insulin	57	10	56.3	7.6	0.6	32	5.7	9.7				
**BEGIN: ADD TO GLP-1**	172	Standard treatments	57.3	9.4	60.5	7.6	0.6	32.4	5.4	9.3				
**BEGIN: VICTOZA ADD-ON**	88	GLP-1 receptor agonists	61.1	9.5	71.6	7.7	0.6	32.5	5.4	12.9				
**BEGIN: VICTOZA ADD-ON**	89	Bolus insulin	60.9	8.8	59.6	7.7	0.8	32	4.8	11.8				
**Berberoglu 2010**	26	Thiazolidinediones	60.7	6.4	0	6.3	0.5	35	4.1					
**Berberoglu 2010**	23	Standard treatments	59.8	7.6	0	6	0.5	34.6	3.3					

Study ID	N	Intervention	Age (mean)	Age (SD)	% of males	HbA1c (mean)	HbA1c (SD)	BMI (mean)	BMI (SD)	Duration of diabetes	% of CV disease	eGFR	ACR	ACR unit
**Bergenstal 2009**	124	GLP-1 receptor agonists	52.5	10.6	48.4	10.2	1.5	34.2	7.1	8.6				
**Bergenstal 2009**	124	Basal-Bolus insulin	51.8	10.9	48.4	10.1	1.8	33.7	7.1	8.4				
**Bergenstal 2009**	124	Basal-Bolus insulin	53.4	10	47.6	10.3	1.9	33.5	7.4	9.9				
**Berndt-Zipfel 2013**	22	Sulfonylureas	60	7	59	7.3	0.6	33.3	6.7	6.1				
**Berndt-Zipfel 2013**	22	DPP-4 inhibitors	57	0.9	68	7.4	0.7	34.6	5.9	8.4				
**BEST**	10	DPP-4 inhibitors	58.76667	20.64008	60	6.3	0.946004	34.73333	13.15805	2.8				
**BEST**	11	Standard treatments	57.83333	8.567224	72.7	6.3	0.84824	34.93333	8.567224	3.5				
**BETA**	36	Sulfonylureas	54.9	8.6	34	8.9	1.3	25.5	3.6	8				
**BETA**	39	Basal insulin	51.3	8.1	38	8.8	1.2	24.4	2.7	6.58				
**BEYOND**	101	Bolus insulin	62.1	10.2	39.6	8.5	1.1	31.9	3.7	17.3	33.6	73.6		
**BEYOND**	102	SGLT2 inhibitors	60.5	10.3	43.1	8.7	1.1	31.6	4.3	16.9	31.3	81.7		
**Bi 2013**	80	Metformin	55.6	9.9		8.1	1.6	24.6	2.5					
**Bi 2013**	80	Sulfonylureas	54.5	9.9		8	1.6	25.2	2.5					
**Bilezikian 2013**	111	Metformin	64	6.5	0	6.8	0.7	31.5	5.8	3.3				
**Bilezikian 2013**	114	Thiazolidinediones	63.6	6.6	0	6.8	0.7	31.2	5.9	3.9				
**Birkeland 1994a**	16	Sulfonylureas				8.6	1.2	26.3	4					
**Birkeland 1994a**	18	Basal insulin				8.8	1.3	26.4	3					
**Bode 2013**	236	SGLT2 inhibitors	63.4	6	54.7	7.7	0.8	31.5	4.6	11.3		79		
**Bode 2013**	241	SGLT2 inhibitors	64.3	6.5	51.5	7.8	0.8	31.4	4.4	12.3		78		
**Bode 2013**	237	Standard treatments	63.2	6.2	60.3	7.8	0.8	31.8	4.8	11.4		76		
**Bolinder 2012**	91	SGLT2 inhibitors	60.6	8.2	55.1	7.2	0.4	32.1	3.9	6	23.6			
**Bolinder 2012**	91	Standard treatments	60.8	6.9	56	7.2	0.5	31.7	3.9	5.5	28.6			
**Bolli 2009**	281	Thiazolidinediones	57	9.7	64.1	8.4	0.9	32.1	5.1	6.4				

Study ID	N	Intervention	Age (mean)	Age (SD)	% of males	HbA1c (mean)	HbA1c (SD)	BMI (mean)	BMI (SD)	Duration of diabetes	% of CV disease	eGFR	ACR	ACR unit
**Bolli 2009**	295	DPP-4 inhibitors	56.3	9.3	61.7	8.4	1	32.2	5.6	6.4				
**Borges 2011**	348	Thiazolidinediones	51.5	10.5	53	8.6	0.9	32.2	6.8	2.3				
**Borges 2011**	340	Standard treatments	50.7	10.5	53	8.6	0.9	33.1	7.1	2.6				
**Bosi 2007**	185	DPP-4 inhibitors	53.9	9.5	61.5	8.4	1	32.9	5	5.8				
**Bosi 2007**	177	DPP-4 inhibitors	54.3	9.7	57.3	8.4	0.9	32.1	5.3	6.8				
**Bosi 2007**	182	Standard treatments	54.5	10.3	53.1	8.3	0.9	33.2	6.1	6.2				
**Bosi 2009**	295	DPP-4 inhibitors	52.8	10.8	58	8.7	1	31.4	4.8	1.9				
**Bosi 2009**	294	Standard treatments	52.4	10.7	58.2	8.6	0.9	31.3	4.6	2.2				
**Bosi 2011**	404	DPP-4 inhibitors	54.3	9.9	52	8.3	0.8	31.5	5.3	7.5				
**Bosi 2011**	399	Standard treatments	55.9	9.9	51.1	8.1	0.8	31.6	5.2	6.9				
**Bouchi 2017**	9	GLP-1 receptor agonists	57	16	8	8.2	0.5	27.7	2.5			71	220	
**Bouchi 2017**	10	Standard treatments	60	22	9	7.9	0.7	28.2	2.5			66	254	
**Braun 1996**	80	Alpha-glucosidase inhibitors	60	6.435031	61.9	10	1.5	26	2.75787	16				
**Braun 1996**	72	Standard treatments	61	7.293225	54.5	9.9	1.5	26	2.05122	17				
**BRL-049653/334**	99	Thiazolidinediones	67	6	51	6.9	0.8	30	4	3.7				
**BRL-049653/334**	101	Standard treatments	66	8	59	6.9	0.8	29	5	4.5				
**Bryson 2016**	88	DPP-4 inhibitors	58.2		59.1	7.9		32.2		5.1				
**Bryson 2016**	91	DPP-4 inhibitors	58.3		61.5	8		33		5.3				
**Bryson 2016**	93	DPP-4 inhibitors	58.5		54.8	7.8		32.2		4.8				
**Bryson 2016**	87	DPP-4 inhibitors	58.8		52.9	8		32.2		4.7				
**Bryson 2016**	88	Standard treatments	58.9		53.4	7.9		32		4.8				

Study ID	N	Intervention	Age (mean)	Age (SD)	% of males	HbA1c (mean)	HbA1c (SD)	BMI (mean)	BMI (SD)	Duration of diabetes	% of CV disease	eGFR	ACR	ACR unit
**Bunck 2009**	36	GLP-1 receptor agonists	58.4	1.4	63.9	7.6	0.6	30.9	4.2	5.7				
**Bunck 2009**	33	Basal insulin	58.3	1.3	66.7	7.4	0.6	30.1	3.6	4				
**Buse 2011**	138	GLP-1 receptor agonists	59	9	51.1	8.3	0.9	33.8	5.8	12				
**Buse 2011**	122	Standard treatments	59	10	63.9	8.5	1	33.1	6.2	12				
**Cai-2021**	19	GLP-1 receptor agonists	62.95	7.41	47.4	8.11	1.05	27.11	3.97					
**Cai-2021**	19	GLP-1 receptor agonists	57.42	7.89	57.9	8.77	1.61	25.34	3.23					
**Cai-2021**	10	Basal insulin	64.36	9.27	70	8.57	0.76	25.79	2.97					
**Cai-2021**	17	Standard treatments	62	4.99	52.9	8.01	0.95	27.04	3.42					
**Camerini-Davalos 1994**	40	Sulfonylureas	45.2	10.8	44.1	11.3	1.9	25.1	1.3	10.8				
**Camerini-Davalos 1994**	30	Standard treatments	46.4	10.8	37	10.3	1.6	24.3	2.2	14.5				
**Camerini-Davalos 1988**	46	Sulfonylureas	46.8	6.8		12.7	1.4	25.4	2	4.8				
**Camerini-Davalos 1988**	31	Standard treatments	47.1	8.4		12.9	1.7	25.4	2	5.6				
**Campbell 1994**	24	Metformin	57	10	33.3	11.5	1.9	29.6	5.6	2.3				
**Campbell 1994**	24	Sulfonylureas	57	9	33.3	11.8	2.1	31.2	6.6	2.8				
**CANDLE**	123	Sulfonylureas	68.9	10.4	71.7	7.1	0.9	25.4	4.8					
**CANDLE**	122	SGLT2 inhibitors	68.3	9.8	77.9	6.9	0.7	24.1	6.4					

Study ID	N	Intervention	Age (mean)	Age (SD)	% of males	HbA1c (mean)	HbA1c (SD)	BMI (mean)	BMI (SD)	Duration of diabetes	% of CV disease	eGFR	ACR	ACR unit
**CANTATA-D**	366	DPP-4 inhibitors	55.5	9.6	47	7.9	0.9	32	6.1	6.8				
**CANTATA-D**	367	SGLT2 inhibitors	55.3	9.2	45	7.9	0.9	31.4	6.3	7.1				
**CANTATA-D**	368	SGLT2 inhibitors	55.5	9.4	47.3	7.9	0.9	32.4	6.4	6.7				
**CANTATA-D2**	378	DPP-4 inhibitors	56.7	9.3	56.9	8.1	0.9	31.7	6.9	9.7				
**CANTATA-D2**	378	SGLT2 inhibitors	56.6	9.6	54.9	8.1	0.9	31.5	6.9	9.4				
**CANTATA-M**	197	SGLT2 inhibitors	55.3	10.2	45.2	8	1	31.7	6	4.3				
**CANTATA-M**	195	SGLT2 inhibitors	55.1	10.8	41.5	8.1	1	31.3	6.6	4.5				
**CANTATA-M**	192	Standard treatments	55.7	10.9	45.8	8	1	31.8	6.2	4.2				
**CANTATA-MSU**	156	SGLT2 inhibitors	56.1	8.9	55.8	8.1	0.9	33.2	6.3	9.4				
**CANTATA-MSU**	157	SGLT2 inhibitors	57.4	10.5	48.4	8.1	0.9	33.3	6.3	9				
**CANTATA-MSU**	156	Standard treatments	56.8	8.3	48.7	8.1	0.9	32.7	6.8	10.3				
**CANTATA-SU**	482	Sulfonylureas	56.3	9	54.6	7.8	0.8	30.9	5.5	6.6				
**CANTATA-SU**	485	SGLT2 inhibitors	55.8	9.2	49.6	7.8	0.8	31.2	5.4	6.7				
**CANTATA-SU**	483	SGLT2 inhibitors	54.6	9.5	52.1	7.8	0.8	31	5.3	6.5				
**CANVAS/CANVAS-R**	5795	SGLT2 inhibitors	63.2	8.3	64.9	8.2	0.9	31.9	5.9	13.5	55.8	77	12.1	
**CANVAS/CANVAS-R**	4347	Standard treatments	63.4	8.2	63.3	8.2	0.9	32	6	13.7	57.2	76	12.4	
**Carlson 2019**	16	GLP-1 receptor agonists	58.6	7.8	62.5	6.4	0.2	32.6	4.7	7.5				
**Carlson 2019**	17	Basal insulin	53.8	7.4	41.2	7.5	0.4	33.7	5.9	6.8				
**CARMELINA**	3494	DPP-4 inhibitors	66.1	9.1	61.5	7.9	1	31.4	5.3	15	58.1	55	162	
**CARMELINA**	3485	Standard treatments	65.6	9.1	64.3	8	1	31.3	5.4	14.5	58.9	55	162	
**CAROLINA**	3010	Sulfonylureas	63.9	9.5	60.8	7.2	0.6	30.2	5.2	6.3	42.2	77	9.7	

Study ID	N	Intervention	Age (mean)	Age (SD)	% of males	HbA1c (mean)	HbA1c (SD)	BMI (mean)	BMI (SD)	Duration of diabetes	% of CV disease	eGFR	ACR	ACR unit
**CAROLINA**	3023	DPP-4 inhibitors	64.2	9.5	59.2	7.2	0.6	30	5.1	6.2	41.7	77	9.7	
**Casner 1988**	31	Sulfonylureas				10.9	0.5							
**Casner 1988**	33	Standard treatments				10.1	0.4							
**Cefalu 2015**	460	SGLT2 inhibitors	62.8	7	67.9	8.2	0.8	32.6	5.9	12.6	100			
**Cefalu 2015**	462	Standard treatments	63	7.7	67.9	8.1	0.8	32.9	6.1	12.3	100			
**Chacra 2017**	107	DPP-4 inhibitors	65.9	9.4	63.6	8.3	0.8	29.4	4.5	14.9				
**Chacra 2017**	106	Standard treatments	64.5	9.7	59.4	8.3	0.8	30.7	6.8	15.1				
**Chakraborty 2011**	110	Metformin			55.5	8.7	1.4	27	2.4					
**Chakraborty 2011**	98	Standard treatments			46	8.7	1.5	27	1.3					
**Charbonnel 2013**	326	DPP-4 inhibitors	56.9	10	55	8.2	1.1	32.6	5.9					
**Charbonnel 2013**	327	GLP-1 receptor agonists	57.6	10.8	55	8.1	0.9	32.7	6.1					
**Charpentier 2009**	145	Thiazolidinediones	59.2	9.6	66.2	8.2	0.6	29.2	3.1	12.1				
**Charpentier 2009**	154	Standard treatments	60.2	9.3	64.6	8.1	0.7	29.1	3.3	12.5				
**Chehrehgosha-2021**	35	SGLT2 inhibitors	50.5	8.4	42.9	8.08	0.92	30.9	3.3	5.5				
**Chehrehgosha-2021**	34	Thiazolidinediones	52.5	7.9	50	8.23	0.79	29.4	3.7	7.3				
**Chehrehgosha-2021**	37	Standard treatments	51.8	7.8	37.8	7.96	0.62	30.2	4.4	7				
**Chen 2015**	200	DPP-4 inhibitors	54.6	10.1	58	8	0.9	25.5	3.3					
**Chen 2015**	99	Standard treatments	54.1	9.3	59.6	8.1	0.9	25.1	3.4					
**Chen 2016**	30	Thiazolidinediones	66.3	8.8	26.7	8.3	0.8	26	3.4	11.6				
**Chen 2016**	30	Alpha-glucosidase inhibitors	67.2	7.6	30	8.2	0.8	30.1	18.4	12.3				
**Cherney-2021**	92	SGLT2 inhibitors	66.8	10	47.8	8.3	1	30.9	5.4	19.6		23.8	2537.5	mg/mmol
**Cherney-2021**	92	SGLT2 inhibitors	67.3	9.6	53.3	8.3	0.9	32.1	6.2	18.5		23.9	2491.75	mg/mmol

Study ID	N	Intervention	Age (mean)	Age (SD)	% of males	HbA1c (mean)	HbA1c (SD)	BMI (mean)	BMI (SD)	Duration of diabetes	% of CV disease	eGFR	ACR	ACR unit
**Cherney-2021**	93	Standard treatments	68	8.3	45.2	8.4	1.1	31.7	5.7	20.7		24.1	2573	mg/mmol
**Chiasson 1994**	172	Alpha-glucosidase inhibitors												
**Chiasson 1994**	182	Standard treatments												
**Chiasson 2001**	83	Metformin	57.9	8.6	73.5	8.2	0.9	30.7	5.1	7.5				
**Chiasson 2001**	82	Alpha-glucosidase inhibitors	57.3	9	78	8.2	0.9	31.1	4.5	5.2				
**Chiasson 2001**	83	Standard treatments	57.7	9.9	67.5	8.1	0.7	31.1	4.4	5.1				
**CHICAGO**	230	Sulfonylureas	59.9	8.2	62.7	7.4	1	31.9	5	7.5	13.6			
**CHICAGO**	232	Thiazolidinediones	59.3	8	63.5	7.4	1	32	5.2	8	7.8			
**Chien 2011**	49	DPP-4 inhibitors	73.5	5.9	36.7	9.5	1.3	26.2	2.2	13.2				
**Chien 2011**	48	Standard treatments	72.5	5.2	47.9	10	1.6	26	2.3	14.2				
**Cho 2019**	35	Thiazolidinediones	63.6	10.2	54.3	6.9	0.6	28.5	4.2		17.1	67	26.5	
**Cho 2019**	36	SGLT2 inhibitors	63.1	10	63.9	6.9	0.6	28.7	6.2		13.9	72	22.1	
**Choi 2004**	47	Thiazolidinediones	60.9	9.3	63.2	7.8	1.3	24.9	2.96	7.5	100			
**Choi 2004**	48	Standard treatments	59.9	9.3	75.6	7.7	1.1	24.8	3.35	7.2	100			
**Chou 2008**	225	Sulfonylureas	53	11	57.7	9	1.3	31.8	7.2					
**Chou 2008**	232	Thiazolidinediones	53.6	10.7	60	9.1	1.3	31.3	5.8					
**Chou 2012**	750	Thiazolidinediones	55.1	10.6	50.9	7.7	0.6	29.6	5.3	4.3				
**Chou 2012**	274	Thiazolidinediones	55	10.5	48.2	7.7	0.5	29.7	5.6	5				
**Chou 2012**	751	Thiazolidinediones	55	10.8	53	7.7	0.6	30	5.8	4.4				
**Chou 2012**	137	Standard treatments	55.4	12.3	48.9	7.7	0.5	30.1	5.4	4.9				
**Chuang-2020**	48	Metformin	59.3	9.7	36.2	10.3	1.6	25.7	4	11.2		74.6		
**Chuang-2020**	47	Sulfonylureas	55	14.1	32.6	10.3	1.5	25.2	4.3	9.9		70.6		

Study ID	N	Intervention	Age (mean)	Age (SD)	% of males	HbA1c (mean)	HbA1c (SD)	BMI (mean)	BMI (SD)	Duration of diabetes	% of CV disease	eGFR	ACR	ACR unit
**CIMT**	206	Metformin	61	8.7	68	8.6	1.1	32.3	4.2	13.5	22	44		
**CIMT**	206	Standard treatments	60.3	9.1	68	8.5	1	32.1	4.2	12.2	27	45		
**Civera 2008**	12	Meglitinides	60.3	7.7	50	9.6	0.6	30.6	4.8	8				
**Civera 2008**	12	Standard treatments	61.6	9.2	60	9.6	0.7	27.9	3.8	7.9				
**COMPASS**	65	Thiazolidinediones	60.7	9.5	56.1	7.4	0.6	25.8	4.8			88	80.8	
**COMPASS**	65	DPP-4 inhibitors	60.3	7.5	62.1	7.5	0.7	24.6	3.3			88	100.5	
**CompoSIT-I**	374	DPP-4 inhibitors	58.6	9.5	45.6	8.8	0.9	31.2	5.8	10.4		104		
**CompoSIT-I**	372	Standard treatments	58.1	9.7	51.4	8.8	10	31.1	5.7	11.1		106		
**CompoSIT-R**	307	DPP-4 inhibitors	67.7	8.5	55	7.7	0.7	31.8	5.7	10.5		79		
**CompoSIT-R**	307	SGLT2 inhibitors	66.6	8.6	60.8	7.8	0.7	31.5	5.3	10.7		77		
**CONFIDENCE**	136	Thiazolidinediones	50	9.3	61	8	1.2	25.8	3.5					
**CONFIDENCE**	142	GLP-1 receptor agonists	50	9.5	69	8	1.2	26.1	3.6					
**CONFIDENCE**	138	Basal-Bolus insulin	51	9.4	61.6	8.1	1.2	25.6	3.5					
**Coniff 1994**	91	Alpha-glucosidase inhibitors	56	9.5	45	6.8		32		4				
**Coniff 1994**	98	Standard treatments	55.6	9.9	54	6.7		31.5		3				
**Coniff 1995**	67	Alpha-glucosidase inhibitors	56.2		39	6.9		29.7		5.1				
**Coniff 1995**	62	Standard treatments			52	7.1		29.9		5.5				
**Coniff 1995a**	103	Alpha-glucosidase inhibitors												
**Coniff 1995a**	104	Standard treatments												

Study ID	N	Intervention	Age (mean)	Age (SD)	% of males	HbA1c (mean)	HbA1c (SD)	BMI (mean)	BMI (SD)	Duration of diabetes	% of CV disease	eGFR	ACR	ACR unit
**Costa 1997**	36	Alpha-glucosidase inhibitors	60.2	8.4	33.3	9	1.2	28.7	4.2					
**Costa 1997**	29	Standard treatments	61.7	9.7	31	8.8	0.5	27.4	3.2					
**CREDENCE**	2202	SGLT2 inhibitors	62.9	9.2	65.4	8.3	1.3	31.4	6.2	15.5	50.5	56	923	
**CREDENCE**	2199	Standard treatments	63.2	9.2	66.7	8.3	1.3	31.3	6.2	16	50.3	56	931	
**Cusi 2019**	26	SGLT2 inhibitors	58	9	62	7.6	0.8	32.2	4.1			92		
**Cusi 2019**	30	Standard treatments	58	10	70	7.7	0.7	31	4.9			89		
**CV181-011**	98	DPP-4 inhibitors	52.7	11.3	45.9	7.8	0.9	31.7	4.7	2.3				
**CV181-011**	106	DPP-4 inhibitors	53.9	11.6	50.9	8	1.1	32.2	4.5	2.5				
**CV181-011**	102	DPP-4 inhibitors	53.3	10.1	56.9	7.9	0.9	31.9		3.1				
**CV181-011**	95	Standard treatments	53.9	12.3	49.5	7.9	0.9	31.7		2.3				
**CV181-013**	186	DPP-4 inhibitors	53.2	10.6	47.8	8.4	1.1	29.8	5.3	5.2				
**CV181-013**	195	DPP-4 inhibitors	54.9	9.7	54.4	8.3	1.1	30	5.8	5.3				
**CV181-013**	184	Standard treatments	54	10.1	46.2	8.2	1.1	30.3	5.8	5.2				
**CV181-039**	323	DPP-4 inhibitors	52.1	11.6	45.2	9.5	1.2	30.4	4.9	1.4				
**CV181-039**	320	DPP-4 inhibitors	52	10.4	51.6	9.4	1.2	29.9	4.5	2				
**CV181-039**	328	Standard treatments	51.8	10.7	49.7	9.4	1.3	30.2	4.9	1.7				
**da Silva 2016**	18	DPP-4 inhibitors	55.1	6.7	50	8	0.6	26.5	2.7	10.9				
**da Silva 2016**	17	Basal insulin	58.4	6.9	35	8.1	0.7	27.5	2.5	10.9				
**Dailey 2004**	181	Thiazolidinediones	57	9	58	8.1	0.9	32	5	9				
**Dailey 2004**	184	Standard treatments	57	10	61	8.1	0.8	32	5	9				
**Dapagliflozin 006**	196	SGLT2 inhibitors	59.3	8.8	44.8	8.6	0.8	33.4	5.1	14.2				
**Dapagliflozin 006**	212	SGLT2 inhibitors	59.3	7.9	47.4	8.6	0.9	33	5.3	13.1				
**Dapagliflozin 006**	202	SGLT2 inhibitors	59.8	7.6	49.5	8.5	0.8	33	5	13.6				

Study ID	N	Intervention	Age (mean)	Age (SD)	% of males	HbA1c (mean)	HbA1c (SD)	BMI (mean)	BMI (SD)	Duration of diabetes	% of CV disease	eGFR	ACR	ACR unit
**Dapagliflozin 006**	197	Standard treatments	58.8	8.6	49.2	8.5	0.8	33.1	5.9	13.5				
**DAPA-LVH**	32	SGLT2 inhibitors	64.25	7.01	62.5			32.3	4.66	9.42	3.1	107.53		
**DAPA-LVH**	34	Standard treatments	66.74	6.62	52.9			32.59	4.22	10.83	17.6	96.56		
**Dargie 2007**	110	Thiazolidinediones	64.3	8.8	84.3	7.8	1.3	28.8	3.7	4.5	100			
**Dargie 2007**	114	Standard treatments	63.9	8.6	79.1	7.8	1.3	28.6	3.5		100			
**Davidson 2007**	121	Thiazolidinediones	52	11.9	45.3	9.2	1.3	31.3	5.7	6				
**Davidson 2007**	124	Standard treatments	53	10.4	48.3	9.4	1.4	31.9	5.6	6.2				
**Davies 2013**	111	GLP-1 receptor agonists				8.4	0.9	33.7	4.7					
**Davies 2013**	105	Basal insulin				8.4	0.9	33.7	4.7					
**Davies 2017**	71	GLP-1 receptor agonists	56.5	10.2	60.6	8	0.7	31.1	4.1	7.7				
**Davies 2017**	70	GLP-1 receptor agonists	58.3	10.4	62.9	7.9	0.7	32	4.5	7				
**Davies 2017**	69	GLP-1 receptor agonists	56.5	10.1	62.3	7.8	0.7	31.9	4.4	5.8				
**Davies 2017**	70	GLP-1 receptor agonists	55.7	11	67.1	7.8	0.6	31.6	4.9	5.3				
**Davies 2017**	70	GLP-1 receptor agonists	56.7	9.9	64.3	8	0.7	31.7	4.1	6.1				
**Davies 2017**	69	GLP-1 receptor agonists	56.8	11.8	69.6	7.8	0.7	30.7	4	5.6				
**Davies 2017**	71	Standard treatments	58.9	10.3	56.3	8	0.8	32.6	4.5	6.7				
**DECLARE-TIMI 58**	8582	SGLT2 inhibitors	63.9	6.8	63.1	8.3	1.2	32.1	6	11	40.5	85		

Study ID	N	Intervention	Age (mean)	Age (SD)	% of males	HbA1c (mean)	HbA1c (SD)	BMI (mean)	BMI (SD)	Duration of diabetes	% of CV disease	eGFR	ACR	ACR unit
**DECLARE-TIMI 58**	8578	Standard treatments	64	6.8	62.1	8.3	1.2	32	6.1	10	40.8	85		
**DeFronzo 1995**	210	Metformin	55	14.5	45.7	8.4	5.8	29.4	4.3	8.4				
**DeFronzo 1995**	209	Sulfonylureas	56	14.5	49.3	8.5	1.4	29.1	4.3	8.7				
**DeFronzo 2005**	113	GLP-1 receptor agonists	52	11	60.2	8.2	1	34	6	4.9				
**DeFronzo 2005**	110	GLP-1 receptor agonists	53	11	51.8	8.3	1.1	34	6	6.2				
**DeFronzo 2005**	113	Standard treatments	54	9	59.3	8.2	1	34	6	6.6				
**DeFronzo 2012**	129	DPP-4 inhibitors	53.7	9.3	38.8	8.6	0.7	31.5	5.7	5.6				
**DeFronzo 2012**	128	DPP-4 inhibitors	53.1	9.6	52.3	8.6	0.7	31	5.1	6.2				
**DeFronzo 2012**	129	Standard treatments	55.2	9.9	47.3	8.5	0.6	30.6	4.8	6				
**DeFronzo 2015**	128	DPP-4 inhibitors	56.2	10	50	8	0.9	30.6	5.4			90		
**DeFronzo 2015**	140	SGLT2 inhibitors	55.5	10	46.4	8	0.8	31.8	5.3			90		
**DeFronzo 2015**	137	SGLT2 inhibitors	56.1	10.5	56.9	8	0.9	30.9	5.4			91		
**Dei Cas 2017**	24	Sulfonylureas	63	10	71	7.7		28.9		5				
**Dei Cas 2017**	40	DPP-4 inhibitors	61	9	65	7.7		29		7				
**Dejager 2007**	157	DPP-4 inhibitors	53.6	10.8	53.3	8.4	0.8	32.4	6.1	2.4				
**Dejager 2007**	152	DPP-4 inhibitors	52.8	9.6	46.7	8.6	0.8	33.3	4.8	2.1				
**Dejager 2007**	163	DPP-4 inhibitors	55.3	11.4	41.3	8.2	0.8	32.9	6	2.1				
**Dejager 2007**	160	Standard treatments	52.2	11.2	47.9	8.4	0.8	32.6	5.6	1.6				
**Del Prato 2003**	284	Metformin	56	9	59	7.8	1.6	29.7	4.2					
**Del Prato 2003**	144	Standard treatments	56	9	63	7.4		29.9	3.9					
**Del Prato 2011**	336	DPP-4 inhibitors	56.4	10.1	48.8	8	0.9	29	4.8					
**Del Prato 2011**	167	Standard treatments	54.4	10.3	47.3	8	0.9	29.1	4.8					

Study ID	N	Intervention	Age (mean)	Age (SD)	% of males	HbA1c (mean)	HbA1c (SD)	BMI (mean)	BMI (SD)	Duration of diabetes	% of CV disease	eGFR	ACR	ACR unit
**Del Prato 2014**	874	Sulfonylureas	55.4	9.6	50.5	7.6	0.6	31.1	5.3	5.5				
**Del Prato 2014**	885	DPP-4 inhibitors	55.5	9.8	51.1	7.6	0.6	31.3	5.3	5.4				
**Del Prato 2014**	880	DPP-4 inhibitors	55.2	9.6	47.6	7.6	0.6	31.3	5.4	5.7				
**DELIGHT**	145	SGLT2 inhibitors	64.7	8.6	70	8.4	1	30.2	5.3	17.6	40		270	
**DELIGHT**	148	Standard treatments	64.7	8.5	71	8.6	1.2	30.3	5.6	17.7	28		257.5	
**Deng 2017**	36	DPP-4 inhibitors	63.7	10.7	77.8	7.3	0.7							
**Deng 2017**	36	Standard treatments	64.1	11.2	72.2	7.4	0.8							
**DERIVE**	160	SGLT2 inhibitors	65.3		56.9	8.3	1.1	32.6	4.7	14.3		53	23.5	
**DERIVE**	161	Standard treatments	66.2		56.5	8	1.1	31.6	5	14.5		54	29	
**Derosa 2003**	62	Sulfonylureas	54	10	48.4	7.8	1.2	26.4	7.9					
**Derosa 2003**	62	Meglitinides	56	9	50	8	1.2	26.1	9.5					
**Derosa 2003a**	56	Metformin	52	9	48.2	7.4	0.9	24.7	1.2					
**Derosa 2003a**	56	Meglitinides	55	10	51.8	7.6	0.9	25.2	1.1					
**Derosa 2004**	83	Metformin												
**Derosa 2004**	81	Sulfonylureas												
**Derosa 2005**	47	Sulfonylureas	52	5	48.9	7.9	0.6	26.8	1.5	4				
**Derosa 2005**	48	Thiazolidinediones	54	4	52.1	8	0.7	26.6	1.3	5				
**Derosa 2007**	124	Sulfonylureas	56	4	50.9	8.2	1.1	26.5	1.5	4				
**Derosa 2007**	124	Meglitinides	55	5	48.7	8.1	1	26.4	1.4	5				
**Derosa 2009**	175	Thiazolidinediones	55	8	49.1	7.9	0.5	26.2	0.6					
**Derosa 2009**	175	Alpha-glucosidase inhibitors	57	6	49.7	8	0.6	27	0.8					
**Derosa 2009a_1**	67	Metformin	55	5	50.7	9.1	1.2	27.2	1.5					
**Derosa 2009a_1**	69	Thiazolidinediones	54	6	46.3	9.2	1.3	27.5	1.7					

Study ID	N	Intervention	Age (mean)	Age (SD)	% of males	HbA1c (mean)	HbA1c (SD)	BMI (mean)	BMI (SD)	Duration of diabetes	% of CV disease	eGFR	ACR	ACR unit
**Derosa 2009a_2**	66	Sulfonylureas	57.7	7	48.5	9	1.1	27.1	1.4					
**Derosa 2009a_2**	69	Thiazolidinediones	57	7	49.3	9.3	1.4	27.4	1.6					
**Derosa 2010**	76	Metformin	58	6	51.3	8.4	0.8	27.7	1.3	6				
**Derosa 2010**	75	DPP-4 inhibitors	57	5	49.3	8.5	0.9	27.9	1.5	5				
**Derosa 2010a**	65	Sulfonylureas	56	7	50.8	8.9	0.8	28.5	1.4					
**Derosa 2010a**	63	GLP-1 receptor agonists	57	8	47.6	8.8	0.7	28.7	1.5					
**Derosa 2011**	54	Sulfonylureas	55	6	48.1	8.8	0.8	28.5	1.4					
**Derosa 2011**	57	GLP-1 receptor agonists	56.7	7	49.1	8.7	0.7	28.4	1.3					
**Derosa 2012**	91	DPP-4 inhibitors	55.9	8.8	46	8.1	0.8	28.1	1.2					
**Derosa 2012**	87	Standard treatments	54.8	7.9	50.6	8	0.7	28.9	2					
**Derosa 2012a**	84	DPP-4 inhibitors	54.2	8.3	50	8.1	0.6	27.9	1.5	6.1				
**Derosa 2012a**	83	Standard treatments	52.4	7.1	51.8	8.2	0.7	27.8	1.4	6.3				
**Derosa 2013**	86	GLP-1 receptor agonists			50	8.1	0.8	31.9	1.7				98.2	
**Derosa 2013**	85	Standard treatments			48.2	7.9	0.6	31.7	1.5				93.6	
**Derosa 2014**	81	Sulfonylureas	57.2	9	49.4	7.7	0.7	27.6	1.3	0.57				
**Derosa 2014**	86	DPP-4 inhibitors	59.8	9.9	48.8	7.9	0.9	27.9	1.6	0.58				
**Derosa 2014a**	102	DPP-4 inhibitors			49	8	0.8	29.2	3.1					
**Derosa 2014a**	103	Standard treatments			48.5	8.1	0.9	29.4	3.2					
**DIA3004**	89	SGLT2 inhibitors	67.9	8.2	53.9	8	0.8	33.4	6.5	17		39	228.1	
**DIA3004**	90	SGLT2 inhibitors	69.5	8.2	64.4	7.9	0.9	32.4	5.5	15.6		40	285.9	
**DIA3004**	90	Standard treatments	68.2	8.4	63.3	8	0.9	33.1	6.5	16.4		40	255.1	

Study ID	N	Intervention	Age (mean)	Age (SD)	% of males	HbA1c (mean)	HbA1c (SD)	BMI (mean)	BMI (SD)	Duration of diabetes	% of CV disease	eGFR	ACR	ACR unit
**Distiller 2014**	14	GLP-1 receptor agonists	49.1	8.491683	50	8.7	1.083422	41.9	4.011588	12.3				
**Distiller 2014**	14	Standard treatments	54.3	8.7845	50	9.2	1.083422	40.2	3.543082	12.4				
**DIVERSITY-CVR**	170	DPP-4 inhibitors	57.9	12.1	58.3	7.8	0.8	27.9	4.2		2.5	79		
**DIVERSITY-CVR**	170	SGLT2 inhibitors	58.3	12.4	61.9	7.8	0.8	27.8	4		4.2	79		
**D-LIFT**	32	GLP-1 receptor agonists	46.6	9.1	72	8.4	1	29.6	3.6	4.9	0			
**D-LIFT**	32	Standard treatments	48.1	8.9	69	8.4	1	29.9	3.9	5.7	3.1			
**Dobs 2013**	181	DPP-4 inhibitors	54.4	8.8	56	8.8	1	30.1	6.2	9.3				
**Dobs 2013**	97	Standard treatments	54.8	9.5	60	8.7	1	30.8	5.6	9.4				
**Dorkhan 2009**	15	Thiazolidinediones	60.8	7.1	73.3	8.1	1.5	30.8	5.5	11.1				
**Dorkhan 2009**	15	Basal insulin	61.5	8.2	60	8.3	1.4	31.6	6.1	9.5				
**Douek 2005**	92	Metformin	58	8.9	67.4	9.7	1.3	30.9	4.5	9				
**Douek 2005**	91	Standard treatments	58	7.7	62.6	10	1.5	31.5	4.3	10				
**Drent 2002**	93	Alpha-glucosidase inhibitors	64	10	63.8	8.1	1	26.9	3.5					
**Drent 2002**	94	Alpha-glucosidase inhibitors	63	11	52.1	8	1.2	27.8	3.2					
**Drent 2002**	93	Alpha-glucosidase inhibitors	63	9	56	8.1	1.2	26.8	3.2					
**Drent 2002**	92	Alpha-glucosidase inhibitors	64	10	48.8	8.1	1.1	27.4	3.7					
**Drent 2002**	93	Standard treatments	63	11	56.3	7.9	1.2	27.2	3.4					

Study ID	N	Intervention	Age (mean)	Age (SD)	% of males	HbA1c (mean)	HbA1c (SD)	BMI (mean)	BMI (SD)	Duration of diabetes	% of CV disease	eGFR	ACR	ACR unit
**DUAL-I**	415	GLP-1 receptor agonists	55	10.2	50	8.3	0.9	31.3	4.8	7.2				
**DUAL-I**	414	Basal insulin	54.9	9.7	48	8.3	1	31.2	5.3	7				
**DUAL-I JAPAN**	273	GLP-1 receptor agonists	56.8	10.1	70.3	8.3	1	26.5	4.5	9.4				
**DUAL-I JAPAN**	271	Basal insulin	57.8	9.9	72	8.5	1.1	26.6	4.8	9.7				
**DUAL-IX**	210	GLP-1 receptor agonists	56.1	10.4	57.6	8.2	0.9	31.5	4.8	9.8				
**DUAL-IX**	210	Standard treatments	57.2	10.2	60	8.4	1.1	30.9	4.8	9.3				
**DUAL-VIII**	506	GLP-1 receptor agonists	56.8	10	55	8.4	1	32	6.2	10				
**DUAL-VIII**	506	Standard treatments	56.4	10.1	54	8.6	1	31.9	5.8	10.2				
**DURATION-2**	165	Thiazolidinediones	53	10	48	8.5	1.1	32	6	6				
**DURATION-2**	166	DPP-4 inhibitors	52	11	52	8.5	1.2	32	5	5				
**DURATION-2**	160	GLP-1 receptor agonists	52	10	56	8.6	1.2	32	5	6				
**DURATION-3**	233	GLP-1 receptor agonists	58	10	52	8.3	1.1	32	5	8				
**DURATION-3**	223	Basal insulin	58	9	55	8.3	1	32	5	7.8				
**DURATION-4**	246	Metformin	54	11	62.6	8.6	1.2	30.7	5.5	2.6				
**DURATION-4**	163	Thiazolidinediones	55	11	59.5	8.5	1.2	31.1	5.3	2.7				
**DURATION-4**	163	DPP-4 inhibitors	52	11	57.7	8.5	1.3	31.8	5.4	2.7				
**DURATION-4**	248	GLP-1 receptor agonists	54	11	56	8.5	1.2	31.4	5.3	2.7				

Study ID	N	Intervention	Age (mean)	Age (SD)	% of males	HbA1c (mean)	HbA1c (SD)	BMI (mean)	BMI (SD)	Duration of diabetes	% of CV disease	eGFR	ACR	ACR unit
**DURATION-7**	233	GLP-1 receptor agonists	57.8	9	49.4	8.5	0.9	33.3	6.1	11.5				
**DURATION-7**	231	Standard treatments	57.6	10.3	46.5	8.5	0.9	34.1	6.6	11.1				
**DURATION-8**	230	GLP-1 receptor agonists	54.2	9.62	51.1	9.3	1.1	32	5.9	7.4		99.4		
**DURATION-8**	233	SGLT2 inhibitors	54.5	9.16	47.8	9.3	1	33	6.1	7.1		97.5		
**DURATION-NEO-2**	122	DPP-4 inhibitors	54.3	9	54.1	8.5	1	31.6	5.8	7.9				
**DURATION-NEO-2**	181	GLP-1 receptor agonists	53.4	9.8	49.2	8.4	1	32.1	5.4	8.5				
**DURATION-NEO-2**	61	Standard treatments	53.4	9.5	60.7	8.5	1	31.5	5.1	8.7				
**EAGLE**	489	GLP-1 receptor agonists	57.4	8.9	56			31.8	4.1	8.4	4			
**EAGLE**	489	Basal insulin	57.1	8.8	52.7			32	4.2	8.5	4			
**EASIE**	265	DPP-4 inhibitors	53.3	8.7	52.2	8.5	1.1	31.3	4.9		6			
**EASIE**	250	Basal insulin	53.9	8.9	50.2	8.5	1	31.1	4.9		5			
**Ebato 2009**	14	Sulfonylureas	62.4	7.8	50	8.3	1.1	25.3	2.4	14.2		75		
**Ebato 2009**	12	Standard treatments	60.5	8.2	58.3	8.3	0.7	25.7	2.4	13.1		75		
**EDIT**	25	DPP-4 inhibitors	66	8	64	7.9	1	24.5	2	19	56	64		
**EDIT**	25	Standard treatments	66	13	75	7.8	0.7	26.8	4.3	20	46	64		
**ELAID study**	7	GLP-1 receptor agonists	57.1	5.7	71.4	6.9	1.1	31.4	4.9			79		
**ELAID study**	9	Standard treatments	63	6.7	55.6	7.6	0.9	32.6	7.5			84		
**ELEGANT**	26	GLP-1 receptor agonists	57	10	61.5	7.2	0.6	34	7	8.3				

Study ID	N	Intervention	Age (mean)	Age (SD)	% of males	HbA1c (mean)	HbA1c (SD)	BMI (mean)	BMI (SD)	Duration of diabetes	% of CV disease	eGFR	ACR	ACR unit
**ELEGANT**	24	Standard treatments	59	8	60	7.5	0.7	32	5	7.6				
**ELITE**	50	SGLT2 inhibitors	63.6	9.2	85.4	7.1	0.8	25.9	3.2	6.27		76.1		
**ELITE**	50	DPP-4 inhibitors	66.3	7.6	89.8	7.2	1	26.2	2.6	6.1		43.1		
**ELIXA**	3034	GLP-1 receptor agonists	59.9	9.7	69.6	7.7	1.3	30.1	5.6	9.2				
**ELIXA**	3034	Standard treatments	60.6	9.6	69.1	7.6	1.3	30.2	5.8	9.4				
**ELLENA-IT**	31	SGLT2 inhibitors	66.3	9.5	67.7	8.1	0.8	25.8	3.9	19				
**ELLENA-IT**	30	GLP-1 receptor agonists	67.2	9	70	8	0.8	26.4	4.6	18.8				
**EMBLEM**	58	SGLT2 inhibitors	65	11	69.2	7.2	0.8	26	5	14	100		67	
**EMBLEM**	59	Standard treatments	64	10	67.9	7.2	0.9	27	6	13	100		69	
**EMBODY**	52	SGLT2 inhibitors	63.9	10.4	82.6	6.82	1	25.2	3.7	3.19		64.6		
**EMBODY**	53	Standard treatments	64.6	11.6	78	6.89	0.92	25.2	4.1	2.7		66.1		
**EMIT**	166	SGLT2 inhibitors	59.6	10	67.3	8.4	0.6	25.8	3.6	10.3				
**EMIT**	77	Standard treatments	59.8	8.6	62.7	8.3	0.7	24.2	3	10.8				
**EMLIFA001**	42	SGLT2 inhibitors	62.7	7	69	6.7	0.7	32.1	4.6	3				
**EMLIFA001**	42	Standard treatments	61.5	10	69	6.8	0.5	32.4	4.2	3.3				
**EMPA-HEART**	27	SGLT2 inhibitors	61.6	9.6	86	7.4	0.59	27.8	4.7	7.8		91.5	3.7	mg/g
**EMPA-HEART**	29	DPP-4 inhibitors	61.8	10.1	86	7.7	0.57	29.6	5.7	11.1		87.7	16.0	mg/g
**EMPA-HEART CardioLink-6**	49	SGLT2 inhibitors			90	7.9	0.8	27.7	4.7			88		
**EMPA-HEART CardioLink-6**	48	Standard treatments			96	8	0.9	27.4	5.4			88		
**EMPA-REG BASAL**	155	SGLT2 inhibitors	59.9	10.5	60	8.2	0.8	32.7	5.9			83		

Study ID	N	Intervention	Age (mean)	Age (SD)	% of males	HbA1c (mean)	HbA1c (SD)	BMI (mean)	BMI (SD)	Duration of diabetes	% of CV disease	eGFR	ACR	ACR unit
**EMPA-REG BASAL**	169	SGLT2 inhibitors	58.6	9.8	55	8.3	0.8	32.1	5.8			85		
**EMPA-REG BASAL**	170	Standard treatments	58.1	9.4	53	8.2	0.8	31.8	6			84		
**EMPA-REG H2H-SU**	780	Sulfonylureas	55.7	10.4	54	7.9	0.9	30.3	5.3			88	39.8	
**EMPA-REG H2H-SU**	769	SGLT2 inhibitors	56.2	10.3	56	7.9	0.8	30	5.3			88	40.7	
**EMPA-REG MDI**	189	SGLT2 inhibitors	58	9.4	44	8.3	0.7	35	4			84		
**EMPA-REG MDI**	186	SGLT2 inhibitors	56.7	8.7	52	8.4	0.7	34.7	3.8			84		
**EMPA-REG MDI**	188	Standard treatments	55.3	10.1	40	8.3	0.7	34.7	4.3			83		
**EMPA-REG MET**	213	SGLT2 inhibitors	55.6	10.2	56	7.9	0.9	29.7	5.7			88		
**EMPA-REG MET**	217	SGLT2 inhibitors	55.5	9.9	58	7.9	0.8	29.1	5.5			90		
**EMPA-REG MET**	207	Standard treatments	56	9.7	56	7.9	0.9	28.7	5.2			90		
**EMPA-REG METSU**	218	SGLT2 inhibitors	57.4	9.3	53	8.1	0.8	28.3	5.5			88		
**EMPA-REG METSU**	226	SGLT2 inhibitors	57	9.2	50	8.1	0.8	28.3	5.4			87		
**EMPA-REG METSU**	225	Standard treatments	56.9	9.2	50	8.2	0.8	27.9	4.9			87		
**EMPA-REG MONO**	223	DPP-4 inhibitors	55.1	9.9	63	7.9	0.8	28.2	5.2			88		
**EMPA-REG MONO**	224	SGLT2 inhibitors	53.8	11.6	65	7.9	0.9	28.2	5.5			88		
**EMPA-REG MONO**	224	SGLT2 inhibitors	56.2	11.6	63	7.9	0.9	28.3	5.5			88		
**EMPA-REG MONO**	228	Standard treatments	54.9	10.9	54	7.9	0.8	28.7	6.2			87		
**EMPA-REG OUTCOME**	4687	SGLT2 inhibitors	63.1	8.6	71.2	8.1	0.9	30.6	5.3		75.6	74		
**EMPA-REG OUTCOME**	2333	Standard treatments	63.2	8.8	72	8.1	0.8	30.7	5.2		75.6	74		
**EMPA-REG PIO**	168	SGLT2 inhibitors	54.2	8.9	50.6	8.1	0.8	29.1	5.5			87		
**EMPA-REG PIO**	165	SGLT2 inhibitors	54.7	9.9	50.3	8.1	0.9	29.2	5.6			84		
**EMPA-REG PIO**	165	Standard treatments	54.6	10.5	44.2	8.2	0.9	29.3	5.4			86		

Study ID	N	Intervention	Age (mean)	Age (SD)	% of males	HbA1c (mean)	HbA1c (SD)	BMI (mean)	BMI (SD)	Duration of diabetes	% of CV disease	eGFR	ACR	ACR unit
**EMPA-REG RENAL - CKD2**	97	SGLT2 inhibitors	62	8.4	62.9	8	0.7	31.3	5.8			72		
**EMPA-REG RENAL - CKD2**	98	SGLT2 inhibitors	63.2	8.5	61.2	8	0.8	32.4	5.4			71		
**EMPA-REG RENAL - CKD2**	97	Standard treatments	62.6	8.1	58.9	8.1	0.8	30.8	5.6			72		
**EMPA-REG RENAL - CKD3**	188	SGLT2 inhibitors	64.6	8.9	57.2	8	0.8	30.2	5.3			45		
**EMPA-REG RENAL - CKD3**	187	Standard treatments	65.1	8.2	56.7	8.1	0.8	30.3	5.3			44		
**EMPA-REG RENAL - CKD4**	37	SGLT2 inhibitors	65.4	10.2	56.8	8.1	1.1	29	4.9			24		
**EMPA-REG RENAL - CKD4**	37	Standard treatments	62.9	11.9	51.4	8.2	1	31.8	6			22		
**Engelbrechtsen 2016**	22	GLP-1 receptor agonists	46	2	18.5									
**Engelbrechtsen 2016**	20	Standard treatments	45	2	12									
**Erem 2014**	19	Metformin	52.2	10.5	31.6	7.6	1.1	33.6	4.6		10.5			
**Erem 2014**	19	Sulfonylureas	55	8.7	36.8	8.3	1.7	32.7	3.9		10.5			
**Erem 2014**	19	Thiazolidinediones	53	5.2	26.3	8	1.7	31.3	4.7		0			
**ESAX-DN**	226	Non-steroidal MRAs	66	10	74	7	0.6	26.2	4.1	14	9	69	139.5	mg/g creatinine
**ESAX-DN**	229	Standard treatments	66	9	79	7	0.6	25.9	4	14	8	69	136.3	mg/g creatinine

Study ID	N	Intervention	Age (mean)	Age (SD)	% of males	HbA1c (mean)	HbA1c (SD)	BMI (mean)	BMI (SD)	Duration of diabetes	% of CV disease	eGFR	ACR	ACR unit
**ESPECIAL-ACS**	21	DPP-4 inhibitors			95.2	6.7	0.5	25.1	3.2		100			
**ESPECIAL-ACS**	20	Standard treatments			100	6.8	0.6	24.3	2.9		100			
**Esposito 2004**	87	Sulfonylureas	51.3	5.9	52.9	7.4	1.1	28.3	4.1					
**Esposito 2004**	88	Meglitinides	52	6.4	53.4	7.5	1.1	28.5	4.3					
**Esposito 2011**	55	Metformin	54.9	6.6	50.9	8.1	1	29.1	3.3					
**Esposito 2011**	55	Thiazolidinediones	54.2	6.1	54.5	8	1	28.9	3.5					
**Essen Study**	27	Sulfonylureas	59.5	5.7	48	8.3	0.4	26.5	2.1	1.5				
**Essen Study**	28	Alpha-glucosidase inhibitors	58.8	6.9	46	8.3	0.4	26.5	1.6	1.1				
**Essen Study**	30	Standard treatments	56.9	6.7	40	8.3	0.4	26.8	1.5	1				
**EUREXA**	514	Sulfonylureas	56.8	9.1	52	7.4	0.7	32.3	3.9	5.5				
**EUREXA**	515	GLP-1 receptor agonists	56.1	10	56	7.5	0.7	32.6	4.2	5.8				
**EUREXA extension**	77	Sulfonylureas	56	10	54.1	7.6	0.6	32.6	4.1	7.2				
**EUREXA extension**	77	Thiazolidinediones	55.7	9.2	65.8	7.7	0.7	32.8	4.3	6.9				
**EXAMINE**	2701	DPP-4 inhibitors			67.7	8	1.1	32.25	5.73904	7.65	87.5	71		
**EXAMINE**	2679	Standard treatments			68	8	1.1	35.325	7.53016	7.775	88.4	71		
**Exenatide-113**	129	GLP-1 receptor agonists	56	11	57.4	8.6	1.2	33	6	6.6				
**Exenatide-113**	125	GLP-1 receptor agonists	55	10	59.2	8.5	1.1	33	6	6.3				
**Exenatide-113**	123	Standard treatments	55	11	62.6	8.7	1.2	34	5	5.7				
**EXENDA**	16	GLP-1 receptor agonists	59.4	8.5	62.5	7.8	1.3	31.9	4.6	7.3				

Study ID	N	Intervention	Age (mean)	Age (SD)	% of males	HbA1c (mean)	HbA1c (SD)	BMI (mean)	BMI (SD)	Duration of diabetes	% of CV disease	eGFR	ACR	ACR unit
**EXENDA**	14	Standard treatments	60.9	7.4	71.4	7.3	0.6	30.7	3.5	5.8				
**EXEPUMP**	28	GLP-1 receptor agonists	61	7	39	8.8	0.8	35.5	4.2	19.3	36			
**EXEPUMP**	18	Standard treatments	58	8	50	8.9	0.9	35.1	4.4	18.4	33			
**EXSCEL**	7356	GLP-1 receptor agonists	62		62	8		31.8		12	73.3	77		
**EXSCEL**	7396	Standard treatments			62	8		31.7		12	72.9	76		
**Feng 2017**	31	Metformin	46.3	12.3	65.5	9.4	1.8	26.8	3.7					
**Feng 2017**	32	Sulfonylureas	48.1	12.6	68.9	9	1.2	27.9	3					
**Feng 2017**	30	GLP-1 receptor agonists	46.8	9.7	72.4	8.9	1.7	28.1	3					
**Feng-2020**	18	Standard treatments	60.7	7	50	8.52	0.6	24.31	3.51	14.4				
**Feng-2020**	19	SGLT2 inhibitors	58.3	8	52.6	8.31	0.74	24.82	3.14	9.4				
**Feng-2020**	19	DPP-4 inhibitors	60.7	7.1	47.4	8.48	0.44	25.93	2.08	12.4				
**Ferdinand 2019**	78	SGLT2 inhibitors	56.5	9.3	55.1	8.7	0.9			9.3	7.7	91		
**Ferdinand 2019**	72	Standard treatments	57.2	9.3	50	8.5	1.1			9.3	6.9	91		
**Fernandez 2008**	10	Thiazolidinediones				9	0.7							
**Fernandez 2008**	10	Standard treatments				9.2	0.4							
**Ferrannini 2010**	146	SGLT2 inhibitors	50.6	10	48.6	8	1	33.6	5.4					
**Ferrannini 2010**	132	SGLT2 inhibitors	52.6	10.9	48.4	7.9	0.9	31.9	4.8					
**Ferrannini 2010**	132	SGLT2 inhibitors	53	11.7	55.4	7.9	0.9	32.6	5.5					
**Ferrannini 2010**	75	Standard treatments	52.7	10.3	41.3	7.8	0.9	32.3	5.5					
**FIDELIO-DKD**	2833	Non-steroidal MRAs	65.4	8.9	68.9	7.7	1.3	31.1	6	16.6	46	44.4	967.33	mg/g
**FIDELIO-DKD**	2841	Standard treatments	65.7	9.2	71.5	7.7	1.4	31.1	6	16.6	45.8	44.3	988.33	mg/g

Study ID	N	Intervention	Age (mean)	Age (SD)	% of males	HbA1c (mean)	HbA1c (SD)	BMI (mean)	BMI (SD)	Duration of diabetes	% of CV disease	eGFR	ACR	ACR unit
**FIGARO-DKD**	3686	Non-steroidal MRAs	64.1	9.7	68.6	7.7	1.4	31.5	6	14.5	45.5	67.6	385.33	mg/g
**FIGARO-DKD**	3666	Standard treatments	64.1	10	70.3	7.7	1.4	31.4	5.9	14.4	45.1	68	385.67	mg/g
**Filozof 2010**	494	Sulfonylureas	59.7	10.2	51.8	8.5	1	30.8	5	6.8				
**Filozof 2010**	513	DPP-4 inhibitors	59.2	9.9	52.2	8.5	1	31.2	5	6.4				
**Finn 2009**	32	Thiazolidinediones	65.7	9.6	75			30.9	6.2					
**Finn 2009**	33	Standard treatments	59.7	9.3	78.8			32.7	6.5					
**Fischer 1998**	98	Alpha-glucosidase inhibitors	59.4	8.6	51	7.5	1.1	27.2	3.3	1.8				
**Fischer 1998**	99	Alpha-glucosidase inhibitors	56.8	9.4	59	7.4	1.1	27.6	3.7	1.4				
**Fischer 1998**	99	Alpha-glucosidase inhibitors	55.5	8.4	49	7.5	1.1	27.6	3.5	1.7				
**Fischer 1998**	102	Alpha-glucosidase inhibitors	58.5	8.4	53	7.4	1.1	27.3	3.5	2.2				
**Fischer 1998**	97	Standard treatments	52.7	8.7	53	7.3	1.1	26.9	2.9	2				
**Foley 2009**	546	Sulfonylureas	54.3	10.4	52.7	8.7	1.1	30.8	5.5	1.9				
**Foley 2009**	546	DPP-4 inhibitors	55.2	10.6	58.8	8.6	1	30.6	5	2.4				
**Foley 2011**	29	DPP-4 inhibitors	57.4	9.4	58.6	6	0.7	29.9	4.9	1.4				
**Foley 2011**	30	Standard treatments	57	6.7	60	6	0.7	29.2	4.4	0.6				
**Fonseca 2000**	113	Thiazolidinediones	58.3	8.8	68.2	8.9	1.5	29.8	3.9	8.3				
**Fonseca 2000**	119	Thiazolidinediones	57.5	10.5	62.1	8.9	1.3	30.2	4.2	7.5				
**Fonseca 2000**	116	Standard treatments	58.8	9.2	74.3	8.6	1.3	30.3	4.4	7.3				
**Fonseca 2003**	200	Meglitinides	56.5	11.3		8.3	1.4	31.6	4.2	5.9				
**Fonseca 2003**	202	Standard treatments	56.8	11.4		8.4	1.4	30.5	4.3	5.8				

Study ID	N	Intervention	Age (mean)	Age (SD)	% of males	HbA1c (mean)	HbA1c (SD)	BMI (mean)	BMI (SD)	Duration of diabetes	% of CV disease	eGFR	ACR	ACR unit
**Fonseca 2007**	144	DPP-4 inhibitors	59.6	10.3	47.9	8.4	1	33.3	5.2	14.4				
**Fonseca 2007**	152	Standard treatments	58.9	10.8	54.6	8.4	1.1	32.9	5.9	14.9				
**Fonseca 2013**	157	DPP-4 inhibitors	55.7	8.7	61.8	8.8	1	29.9	5.2	9.4				
**Fonseca 2013**	156	Standard treatments	56.4	9.4	62.8	8.7	1	30	5.2	10.2				
**Forst 2003**	68	Sulfonylureas	56.6	8.6	57.4	7.7	1.2	28.7	3.9	4.3				
**Forst 2003**	75	Bolus insulin	58.7	7.3	68	7.5	1	29.7	3.6	4.4				
**Forst 2014**	114	SGLT2 inhibitors	57	10.2	55.3	7.9	0.9	32.8	7.7	11		87		
**Forst 2014**	113	SGLT2 inhibitors	56.7	10.4	68.1	8	0.9	32.3	6.2	10.5		85		
**Forst 2014**	115	Standard treatments	58.3	9.6	66.1	8	1	32.5	6.4	10.1		87		
**Forst 2015**	82	DPP-4 inhibitors	65.9	9.8	56.1	7.6	0.5	29.7	4.5	7.3				
**Forst 2015**	79	Basal insulin	67.6	11.9	60.8	7.7	0.5	31.4	5.2	8.6				
**Frederich 2012**	74	DPP-4 inhibitors	54.7	9.7	51.4	8	0.9	31	5.2	1.7	10.8			
**Frederich 2012**	72	DPP-4 inhibitors	55.1	10.4	45.8	7.9	0.9	29.6	5.4	2	11.1			
**Frederich 2012**	74	DPP-4 inhibitors	55.2	10.4	33.8	8	0.8	30.4	4.8	1.2	12.2			
**Frederich 2012**	71	DPP-4 inhibitors	54.3	10.9	52.1	8	1.1	30.6	4.7	2	21.1			
**Frederich 2012**	74	Standard treatments	55.6	10.3	47.3	7.8	1	31.1	4.5	1.7	12.2			
**FREEDOM CVO**	2075	GLP-1 receptor agonists	63	7.42	62.5	8.17	1.56	32.6	5.79	10.63	49.7	80		
**FREEDOM CVO**	2081	Standard treatments	62.7	8.16	64.1	8.13	1.48	32.2	5.56	10.39	48.2	80.3		
**FREEDOM-1**	153	GLP-1 receptor agonists	54.7	9.6	59.5	8.5	0.8	33.8	5.2	8.9		88		
**FREEDOM-1**	153	GLP-1 receptor agonists	55.5	10.3	58.2	8.5	0.8	33.1	5.1	9.1		87		
**FREEDOM-1**	154	Standard treatments	54.7	9.1	59.7	8.5	0.8	33.7	5.5	8.6		89		

Study ID	N	Intervention	Age (mean)	Age (SD)	% of males	HbA1c (mean)	HbA1c (SD)	BMI (mean)	BMI (SD)	Duration of diabetes	% of CV disease	eGFR	ACR	ACR unit
**Frias-2018**	51	Standard treatments	56.6	8.9	57	8	0.9	32.4	6	8.6		95.3		
**Frias-2018**	53	Dual GIP/GLP-1 receptor agonists	57.4	8.9	56	8.2	0.9	32.9	6.1	7.8		95.6		
**Frias-2018**	55	Dual GIP/GLP-1 receptor agonists	57.9	8.2	62	8.2	1	32.9	5.7	8.9		92.2		
**Frias-2018**	52	Dual GIP/GLP-1 receptor agonists	56.5	9.9	59	8.2	1.1	32.6	5.8	7.9		93.7		
**Frias-2018**	53	Dual GIP/GLP-1 receptor agonists	56	7.6	42	8.1	1.1	32.2	6.2	8.5		91.8		
**Frias-2018**	54	GLP-1 receptor agonists	58.7	7.8	44	8.1	1	32.4	5.4	9.3		90.7		
**Gaal 2001**	77	Alpha-glucosidase inhibitors	57.9	10	41.6	8.5	1	30	4					
**Gaal 2001**	75	Standard treatments	57.9	8.5	49.3	8.4	1	29.7	3.9					
**Gallwitz 2011**	248	GLP-1 receptor agonists	57.2	10	59.7	7.9	0.8	33.4	4.2	4.8				
**Gallwitz 2011**	246	Basal-Bolus insulin	56.9	9.9	55.5	7.9	0.9	32.9	4.4	5.2				
**Gallwitz 2012**	775	Sulfonylureas	59.8	9.4	61	7.7	0.9	30.3	4.6					
**Gallwitz 2012**	777	DPP-4 inhibitors	59.8	9.4	60	7.7	0.9	30.2	4.8					
**Gantz 2017**	2100	DPP-4 inhibitors	63.7	8.5	69.6	8	0.9	31.2	5.5	12		86		
**Gantz 2017**	2102	Standard treatments	63.6	8.5	70.7	8	0.9	31.4	5.6	12.1		87		
**Gantz 2017a**	102	DPP-4 inhibitors	38.8	4.7	65.7	7.9	0.8	32.9	5.8	2.9				
**Gantz 2017a**	101	Standard treatments	39.5	4.5	59.4	8.1	0.9	32.1	5	3.3				

Study ID	N	Intervention	Age (mean)	Age (SD)	% of males	HbA1c (mean)	HbA1c (SD)	BMI (mean)	BMI (SD)	Duration of diabetes	% of CV disease	eGFR	ACR	ACR unit
**Gao-2020**	191	GLP-1 receptor agonists	53.6	10.5	57	8.5	0.9	26	3.5	4.3				
**Gao-2020**	192	GLP-1 receptor agonists	52.8	10.6	60.6	8.5	0.9	26.6	3.8	4.8				
**Gao-2020**	190	Standard treatments	52.3	10.7	54.7	8.6	0.9	26.9	3.9	4.7				
**Garber 2006**	158	Sulfonylureas	56	8.832616	56	8.5	1.2	32	5	5				
**Garber 2006**	160	Thiazolidinediones	56	10.16428	65	8.4	1.1	32	5	6				
**Garber 2007**	158	DPP-4 inhibitors	54	9.2	44.9	8.7	1.2	32.2	5.8	4.6				
**Garber 2007**	147	DPP-4 inhibitors	54	8.2	54.8	8.6	1	32.6	5	4.7				
**Garber 2007**	158	Standard treatments	54.8	10.6	50.7	8.7	1.2	32.3	5.8	4.8				
**Garber 2008**	169	DPP-4 inhibitors	58.2	11.1	59.8	8.6	1	30.8	5.3	6.7				
**Garber 2008**	170	DPP-4 inhibitors	58.6	10.6	59.1	8.5	0.9	32.2	4.9	6.9				
**Garber 2008**	176	Standard treatments	57.9	10.5	58.3	8.5	1	31	5.5	7.8				
**Gastaldelli 2014**	29	GLP-1 receptor agonists	59	10.8	37.9	7.7	0.9	30.3	5.4					
**Gastaldelli 2014**	26	GLP-1 receptor agonists	57	10.2	38.5	7.78	0.92	31.2	4.6					
**Gastaldelli 2014**	24	Standard treatments	54	9.8	50	7.8	0.8	32.9	4.9					
**GENERATION**	360	Sulfonylureas	72.7	5.4	63.3	7.6	0.7	29.9	5	7.6	10			
**GENERATION**	360	DPP-4 inhibitors	72.5	5.7	60.3	7.6	0.7	29.3	4.7	7.6	8.6			
**Gentile 2001**	52	Alpha-glucosidase inhibitors				8.9	0.8							
**Gentile 2001**	48	Standard treatments				8.7	0.8							

Study ID	N	Intervention	Age (mean)	Age (SD)	% of males	HbA1c (mean)	HbA1c (SD)	BMI (mean)	BMI (SD)	Duration of diabetes	% of CV disease	eGFR	ACR	ACR unit
**GetGoal-Duo-1**	223	GLP-1 receptor agonists	56	10	49	7.6	0.5	32	6.6	9.6				
**GetGoal-Duo-1**	223	Standard treatments	56	10	51	7.6	0.5	31.7	6	8.7				
**GetGoal-Duo-2**	298	GLP-1 receptor agonists	59.8	8.6	46.3	8.5	0.7	32.3	4.6	11.9				
**GetGoal-Duo-2**	298	Bolus insulin	60.2	8.6	45.3	8.5	0.7	31.9	4.4	12.3				
**GetGoal-Duo-2**	298	Bolus insulin	59.4	9.5	44.3	8.5	0.8	32.5	4.6	12.4				
**GetGoal-F1**	161	GLP-1 receptor agonists	54.6	8.9	45	8.1	0.9	32.1	4.8	6				
**GetGoal-F1**	161	GLP-1 receptor agonists	55.4	8.9	44	8	0.9	33	5.8	5.8				
**GetGoal-F1**	160	Standard treatments	58.2	9.8	45	8	0.8	32.4	5.5	6.2				
**GetGoal-L**	329	GLP-1 receptor agonists	57	10	45	8.4	0.9	31.9	6.2	12.5				
**GetGoal-L**	167	Standard treatments	57	10	49	8.4	0.8	32.6	6.3	12.4				
**GetGoal-L Asia**	154	GLP-1 receptor agonists	58.7	10.2	44.8	8.5	0.7	25.4	3.7	13.7				
**GetGoal-L Asia**	157	Standard treatments	58	10.1	51	8.5	0.8	25.2	3.9	14.1				
**GetGoal-L-C**	224	GLP-1 receptor agonists	53.9	9.9	46.9	7.9	0.7	27.5	4.4	10.3				
**GetGoal-L-C**	224	Standard treatments	56.2	9.1	43.8	8.6	0.9	27.9	4.5	10.2				
**GetGoal-M**	255	GLP-1 receptor agonists	54.8	10.4	38.4	8.1	0.9	32.5	5.8	6.2				

Study ID	N	Intervention	Age (mean)	Age (SD)	% of males	HbA1c (mean)	HbA1c (SD)	BMI (mean)	BMI (SD)	Duration of diabetes	% of CV disease	eGFR	ACR	ACR unit
**GetGoal-M**	255	GLP-1 receptor agonists	54.5	9.2	44.7	8	0.9	33.2	6.9	6.2				
**GetGoal-M**	170	Standard treatments	55	9.4	47.6	8.1	0.9	33.1	6.5	5.9				
**GetGoal-M-Asia**	196	GLP-1 receptor agonists	54.5	10.3	51.5	8	0.8	26.8	3.9	6.5				
**GetGoal-M-Asia**	194	Standard treatments	55.1	10.5	46.9	7.9	0.7	27.1	3.8	6.8				
**GetGoal-O**	176	GLP-1 receptor agonists	74	4	52.3	8.1	0.7	29.9	3.7	13.6				
**GetGoal-O**	174	Standard treatments	74.4	3.8	51.7	8.1	0.7	30.1	4.5	14.6				
**GetGoal-P**	323	GLP-1 receptor agonists	56	9.5	53	8.1	0.9	33.7	6.7	8.1				
**GetGoal-P**	161	Standard treatments	55.3	9.5	51	8.1	0.8	34.4	7	8.1				
**GetGoal-S**	574	GLP-1 receptor agonists	57	9.8	49.5	8.3	0.9	30.1	6.6	9.1				
**GetGoal-S**	285	Standard treatments	57.8	10.1	52.6	8.2	0.8	30.4	6.6	9.8				
**Giles 2008**	256	Sulfonylureas	63.4	9.4	77	8	1.8	29.7	5.4	11.7	100			
**Giles 2008**	262	Thiazolidinediones	64.2	9.9	70.2	8.7	1.6	29.6	5.2	11.8	100			
**Giugliano 1993**	27	Metformin	60	1	37	11.5	1.2	33	3.1	11.9				
**Giugliano 1993**	23	Standard treatments	60.8	1.1	39.1	11.7	1.3	32.7	3.2	11.5				
**GLAC**	109	Sulfonylureas	57.9	9.2	73	8.5	0.8	29.6	4.8					
**GLAC**	91	Thiazolidinediones	60	8.5	62	8.4	0.7	30.2	5.6					
**GLAD**	123	Sulfonylureas	55.7	9.3	53	8.5	1	28.8	3.2	6.8				
**GLAD**	121	Thiazolidinediones	55.1	8	45	8.5	0.9	29.3	3.3	6.5				
**GLAL**	297	Sulfonylureas	56	9.9	61.3			31	5.6	2.9				

Study ID	N	Intervention	Age (mean)	Age (SD)	% of males	HbA1c (mean)	HbA1c (SD)	BMI (mean)	BMI (SD)	Duration of diabetes	% of CV disease	eGFR	ACR	ACR unit
**GLAL**	270	Thiazolidinediones	57	9.8	63.3			32	6.4	2.7				
**Glimepiride Combination Group 1998**	72	Sulfonylureas	58	8	62.5	9.7		32.2	4.4	4				
**Glimepiride Combination Group 1998**	73	Standard treatments	58	8	54.8	9.9		33.7	5.4	7				
**Göke 2002**	129	Thiazolidinediones	58.9	9.1	53.5	9	1.2	30.9	5.3	4.8				
**Göke 2002**	136	Alpha-glucosidase inhibitors	58.8	9.1	54.5	9	1.3	30.8	4.4	4.9				
**Göke 2010**	430	Sulfonylureas	57.6	10.4	54	7.7	0.9	31.3	6.2	5.4				
**Göke 2010**	428	DPP-4 inhibitors	57.5	10.3	49.5	7.7	0.9	31.5	5.7	5.5				
**Gomez-Perez 2002**	36	Thiazolidinediones	54.2	9.3	19.4			27.6	3.2	10.7				
**Gomez-Perez 2002**	35	Thiazolidinediones	51.7	8.6	28.6			28	4	11.1				
**Gomez-Perez 2002**	34	Standard treatments	53.4	7.5	29.4			28.5	3.9	9.1				
**Gomis 2011**	259	DPP-4 inhibitors	57.7	9.6	58.7	8.6	0.8	28.7	4.8					
**Gomis 2011**	130	Standard treatments	57.1	10.1	65.4	8.6	0.9	29.7	4.8					
**Goodman 2009**	123	DPP-4 inhibitors	54.7	10.3	52.8	8.5	1	31.7	4.6					
**Goodman 2009**	125	DPP-4 inhibitors	55.2	11.4	52.8	8.5	0.9	31	4.8					
**Goodman 2009**	122	Standard treatments	54.5	9.7	67.2	8.7	1.1	31.7	4.3					
**GRADE study**	1263	Basal insulin	57	9.9	64.2	7.5	0.5	34.4	6.8	4.2	6.6			
**GRADE study**	1254	Sulfonylureas	57.1	10.1	62	7.5	0.5	34.3	6.9	4.3	6.6			
**GRADE study**	1262	GLP-1 receptor agonists	57.4	9.9	65.2	7.5	0.5	34.3	6.7	4.2	6.6			

Study ID	N	Intervention	Age (mean)	Age (SD)	% of males	HbA1c (mean)	HbA1c (SD)	BMI (mean)	BMI (SD)	Duration of diabetes	% of CV disease	eGFR	ACR	ACR unit
**GRADE study**	1268	DPP-4 inhibitors	57.2	10.1	62.9	7.5	0.5	34.1	6.8	4.2	6.6			
**Grant 1996**	27	Metformin												
**Grant 1996**	25	Metformin												
**Grant 1996**	23	Standard treatments												
**GRAVITAS**	53	GLP-1 receptor agonists	55		38	7.9	1.4	36.1	7.8	16.4				
**GRAVITAS**	27	Standard treatments	57		48	7.4	0.8	37	7.7	19.6				
**Grey 2014**	43	Thiazolidinediones			49	7.4	3.5	31	5					
**Grey 2014**	43	Standard treatments			53	7.5	3.2	31	5					
**Gudipaty 2014**	17	Sulfonylureas	52	7.4	57	6.7	0.4	31	7.5	3.4				
**Gudipaty 2014**	13	DPP-4 inhibitors	57	10.4	66	6.5	0.4	33	3.5	5.3				
**Gudipaty 2014**	17	GLP-1 receptor agonists	57	7.5	64	6.4	0.4	33	7.5	3.3				
**Guo-2020**	32	Basal insulin	52	8.7	60	7.4	0.9	28.3	3.8					
**Guo-2020**	32	GLP-1 receptor agonists	53.1	6.3	52	7.5	1.3	29.2	4.2					
**Guo-2020**	32	Standard treatments	52.6	3.9	67	7.4	1	28.6	3.7					
**Gurkan 2014**	17	GLP-1 receptor agonists	52.2	7.3	29.4	8	0.8	35.9	3.7		12			
**Gurkan 2014**	17	Basal insulin	53.1	7	42	8.1	0.8	33.2	4.5		6			
**Gutniak 1987**	10	Sulfonylureas	56.1	4.7		11.1	0.4							
**Gutniak 1987**	10	Standard treatments	58	2		10.3	0.5							
**Guvener 1999**	20	Sulfonylureas	53.1	6.1	22.2	8.32	1.1	28.04	4.29	11.86				

Study ID	N	Intervention	Age (mean)	Age (SD)	% of males	HbA1c (mean)	HbA1c (SD)	BMI (mean)	BMI (SD)	Duration of diabetes	% of CV disease	eGFR	ACR	ACR unit
**Guvener 1999**	20	Alpha-glucosidase inhibitors	59.61	9.3	20	8.6	0.67	27.6	5.41	10.15				
**Guzman 2017**	65	DPP-4 inhibitors	57.1	9	75.6	8.3	0.9	31.8	6.1	10.9				
**Guzman 2017**	68	Standard treatments	57.8	8.2	54.4	8.3	0.9	31.2	4.9	12.4				
**Haak 2012_1**	143	DPP-4 inhibitors	55.6	11.2	51	8.7	1	29.7	5.3					
**Haak 2012_1**	143	DPP-4 inhibitors	56.4	10.7	53.8	8.7	1	28.6	4.8					
**Haak 2012_1**	147	Standard treatments	55.2	10.6	53.1	8.5	0.9	29.5	5.3					
**Haak 2012_1**	144	Standard treatments	52.9	10.4	56.9	8.7	0.9	28.9	4.8					
**Haak 2012_2**	142	DPP-4 inhibitors	56.2	10.8	56.3	8.7	1	29	4.7					
**Haak 2012_2**	72	Standard treatments	55.7	11	50	8.7	1	28.6	5.2					
**Hadjadj 2016**	164	Metformin	51.6	10.8	51.2	8.7	1	30.3	5.8			91		
**Hadjadj 2016**	168	Metformin	53.4	10.9	56.1	8.6	1.1	30.5	5.9			93		
**Hadjadj 2016**	164	SGLT2 inhibitors	53.1	10.7	57.4	8.6	1.2	30.3	5.2			94		
**Hadjadj 2016**	169	SGLT2 inhibitors	53.3	10.7	50.6	8.9	1.3	30.6	5.9			92		
**Halimi 2000**	74	Alpha-glucosidase inhibitors	56	9.2	47.5	8.6	1.1	30.1	3.3	9.5				
**Halimi 2000**	78	Standard treatments	55	10	62.9	8.5	1.1	29.7	3.3	9				
**Hällsten 2002**	13	Metformin	57.8	7.9	61.5	6.9	0.7	29.9	4					
**Hällsten 2002**	14	Thiazolidinediones	58.6	7.5	71.4	6.8	0.7	29.3	3.7					
**Hällsten 2002**	14	Standard treatments	57.7	7.1	71.4	6.3	0.4	30.3	4.5					
**Halvorsen 2019**	145	SGLT2 inhibitors	56.2	10.9	46.2			29.7	5.4	7.55				
**Halvorsen 2019**	141	Standard treatments	54.9	10.3	35.5			30.6	5.5	7.38				
**Halvorsen 2019a**	193	DPP-4 inhibitors	59.6	9.8	65.3	8	0.9	31.4	5.3	9.36				
**Halvorsen 2019a**	191	SGLT2 inhibitors	59.3	9.7	62.8	7.9	0.8	32.1	6.1	8.22				

Study ID	N	Intervention	Age (mean)	Age (SD)	% of males	HbA1c (mean)	HbA1c (SD)	BMI (mean)	BMI (SD)	Duration of diabetes	% of CV disease	eGFR	ACR	ACR unit
**Han 2018**	74	SGLT2 inhibitors	57.6	8.3	50.7	7.9	0.7	25.5	3.1	11.62		89		
**Han 2018**	69	Standard treatments	57.4	7.9	48.5	7.9	0.8	26.1	3.8	11.3		91		
**Han-2020**	15	Standard treatments	56.7	11.8	60	6.6	0.6	30.2	2.5	10.1		94.8		
**Han-2020**	30	SGLT2 inhibitors	52.5	10.3	63.3	6.7	0.7	30.4	5.4	9.1		101.9		
**Han-2021**	25	DPP-4 inhibitors	54.24	10.91	80	7.16	0.64	28.76	3.18	5.08		93.98		
**Han-2021**	26	Thiazolidinediones	50.5	11.6	50	7.19	0.63	28.46	4.41	4.12		96.14		
**Handelsman 2017**	375	Sulfonylureas	58	9	56.3	7.4	0.7	31.7	6	7.7				
**Handelsman 2017**	376	DPP-4 inhibitors	58	10	54	7.5	0.8	31.2	5.3	7.6				
**Handelsman 2019**	232	SGLT2 inhibitors	55.9	8.9	43.1	8.8	0.8	33.3	6.1	7.9		92		
**Handelsman 2019**	229	Standard treatments	55.8	9.6	48	8.9	0.9	32.8	6.3	8.2		93		
**Haneda 2016**	95	SGLT2 inhibitors	67.9	8.9	75.8	7.7	0.7	25.5	4.2	10.4	20.2	52	335.7	
**Haneda 2016**	50	Standard treatments	68.4	8.9	78	7.7	0.7	25.8	4	12.6	20	52	231.9	
**Hanefeld 1991**	50	Alpha-glucosidase inhibitors	60	6.082544	49	9.2		27.4		5.8				
**Hanefeld 1991**	50	Standard treatments	59	6.082544	47	9.5		27.7		4.1				
**Hanefeld 2004**	320	Metformin	60	8	54.7	8.8	1	30	4.6	7.1			0.11	
**Hanefeld 2004**	319	Thiazolidinediones	60	8.8	53.6	8.8	1	30.2	4.4	7			0.07	
**Hanefeld 2007**	207	Sulfonylureas	60.1	8.3	70.4	8.2	1.3	28.7	3.9	6.4				
**Hanefeld 2007**	191	Thiazolidinediones	60.4	8.2	57.1	8.1	1.3	28.7	3.7	5.9				
**Hanefeld 2007**	200	Thiazolidinediones	60.6	9.2	68.2	8.2	1.4	28.8	3.7	6				
**Hao-2022**	180	GLP-1 receptor agonist	51.9	11.1	58	8.92	1.49	30.2	4.3	6.5				
**Hao-2022**	180	SGLT2 inhibitors	51.8	11.4	64.5	8.8	1.41	29.6	4.2	6.1				

Study ID	N	Intervention	Age (mean)	Age (SD)	% of males	HbA1c (mean)	HbA1c (SD)	BMI (mean)	BMI (SD)	Duration of diabetes	% of CV disease	eGFR	ACR	ACR unit
**HARMONY 1**	155	GLP-1 receptor agonists	55.2	10	61.3	8.1	1	33.6	5.9	8				
**HARMONY 1**	155	Standard treatments	54.9	9.4	58.3	8.1	0.9	34.7	5.6	7.9				
**HARMONY 2**	99	GLP-1 receptor agonists	52	11.8	50.5	8.2	0.9	33.9	5.5	4.2				
**HARMONY 2**	101	GLP-1 receptor agonists	53.6	10.9	57.4	8	0.8	33.7	5.1	3.4				
**HARMONY 2**	101	Standard treatments	53.1	11.7	57.4	8	0.9	33	5.4	4.3				
**HARMONY 3**	307	Sulfonylureas	54.4	10	51.5	8.1	0.8	32.5	5.5	6				
**HARMONY 3**	302	DPP-4 inhibitors	54.3	9.8	46	8.1	0.8	32.5	5.4	5.8				
**HARMONY 3**	302	GLP-1 receptor agonists	54.3	10.1	44.7	8.1	0.8	32.7	5.6	6				
**HARMONY 3**	101	Standard treatments	56.1	10	49.5	8.2	0.9	32.8	5.4	6.7				
**HARMONY 4**	504	GLP-1 receptor agonists	55.8	9.3	56.7	8.3	0.9	33.2	5.6	8.9	5.3			
**HARMONY 4**	241	Basal insulin	54.7	9.8	54.8	8.4	1	33	5.4	8.4	4.6			
**HARMONY 5**	288	Thiazolidinediones	55.7	9.4	53.4	8.3	0.9	32.2	5.7	9.2	5.1			
**HARMONY 5**	281	GLP-1 receptor agonists	54.5	9.5	49.8	8.2	0.9	32.4	5.5	8.5	3.7			
**HARMONY 5**	116	Standard treatments	55.7	9.6	60.9	8.3	1	31.8	4.9	9.3	3.5			
**HARMONY 6**	285	GLP-1 receptor agonists	54.8	9.1	46	8.5	0.9			11	7.7			
**HARMONY 6**	281	Bolus insulin	56.3	8.9	48	8.4	0.9			11	9.6			

Study ID	N	Intervention	Age (mean)	Age (SD)	% of males	HbA1c (mean)	HbA1c (SD)	BMI (mean)	BMI (SD)	Duration of diabetes	% of CV disease	eGFR	ACR	ACR unit
**HARMONY OUTCOMES**	4731	GLP-1 receptor agonists	64.1	8.7	70	8.8	1.5	32.3	5.9	14.1	70	79		
**HARMONY OUTCOMES**	4732	Standard treatments	64.2	8.7	69	8.7	1.5	32.3	5.9	14.2	71	79		
**Hartemann-Heurtier 2009**	14	Thiazolidinediones	62	10	64.2	8.3	0.5	30	5	12				
**Hartemann-Heurtier 2009**	13	Basal insulin	58	10	53.8	8.6	0.5	32	4	12				
**Hartley 2015**	239	Sulfonylureas	70.8	4.9	40.3	7.8	0.7	29.7	5.1	9.4				
**Hartley 2015**	241	DPP-4 inhibitors	70.6	4.8	47.2	7.8	0.7	29.7	4	8				
**Hasche 1999**	36	Alpha-glucosidase inhibitors	63.8	9.8	47.2	8.5	0.7	26.1	2.9					
**Hasche 1999**	38	Standard treatments	63.1	10.5	50	8.3	0.7	26.7	2.8					
**Hattori 2017**	37	DPP-4 inhibitors	65.7	5.4	51.5	7.3	0.6	23.4	3.4					
**Hattori 2017**	37	Alpha-glucosidase inhibitors	63.1	9.8	72.9	7	0.5	24.5	3.9					
**Hattori 2018**	51	SGLT2 inhibitors	57.4	12.3	74.5	7	1.1	30	4.9					
**Hattori 2018**	51	Standard treatments	58.1	9.7	80.4	6.8	0.9	30	4.4					
**Hattori-2021**	30	DPP-4 inhibitors				6.9	0.92	22.4	3.4	9.1			205	mg/g
**Hattori-2021**	22	Standard treatments				6.8	0.25	22.1	3.3	9.5			99	mg/g
**HEELA**	118	GLP-1 receptor agonists	56.8	10.2	70.3	8.7	0.7	34.6	5.7	9	13.6			
**HEELA**	117	Basal insulin	56.2	7.9	66.4	8.5	0.7	33.7	4.9	8.4	18.1			

Study ID	N	Intervention	Age (mean)	Age (SD)	% of males	HbA1c (mean)	HbA1c (SD)	BMI (mean)	BMI (SD)	Duration of diabetes	% of CV disease	eGFR	ACR	ACR unit
**Hegele 1995**	70	Alpha-glucosidase inhibitors	53.7	4.8	48.6	12.2	2.1							
**Hegele 1995**	71	Standard treatments	55.5	5.1	46.5	12.4	2.9							
**Heine 2005**	282	GLP-1 receptor agonists	59.8	8.8	55	8.2	1	31.4	4.4	9.9				
**Heine 2005**	267	Basal insulin	58	9.5	56.6	8.3	1	31.3	4.6	9.2				
**Heise-2022**	45	Dual GIP/GLP-1 receptor agonists	61.1	7.1	69	7.83	0.72	31.28	5.01	10.24				
**Heise-2022**	44	GLP-1 receptor agonists	63.7	5.9	77	7.7	0.6	30.82	3.84	12.73				
**Heise-2022**	28	Standard treatments	60.4	7.6	75	7.9	0.51	32.24	3.96	10.95				
**Heliövaara 2007**	30	Sulfonylureas	57.2	9.9	73.3	8.35	0.7	30.5	4.9					
**Heliövaara 2007**	29	Thiazolidinediones	57.4	7.5	58.6	8.18	0.5	32.3	7					
**Henry 2012_1**	201	Metformin	51.8	9.8	47.3	9.2	1.3			1.6				
**Henry 2012_1**	203	SGLT2 inhibitors	52.3	10.2	45.3	9.1	1.4			1.6				
**Henry 2012_2**	208	Metformin	52.7	10.4	46.6	9.1	1.3			1.9				
**Henry 2012_2**	219	SGLT2 inhibitors	51.1	11.5	47.9	9.1	1.3			2.1				
**Henry 2014**	188	Thiazolidinediones	52.5		50.5	8.7	1.2	31.2	5.1	3.7				
**Henry 2014**	194	Thiazolidinediones	51.8		54.1	8.9	1.1	30.9	5.6	3.9				
**Henry 2014**	183	Thiazolidinediones	50.3		65	8.8	1	30.7	5.2	3.7				
**Henry 2014**	186	DPP-4 inhibitors	51		60.2	8.6	1.2	31.4	5.7	4.5				
**Hermann 1999**	16	Metformin	56.9	10.2	43.8	9.1	1.3	33.6	3.5	13				
**Hermann 1999**	19	Standard treatments	58.1	9.7	63.2	8.7	1	32.6	3.8	13				
**Hiramatsu 2018**	49	DPP-4 inhibitors	69.9	8.5		6.7	0.7	24.2	3.8	8.8		46	300	

Study ID	N	Intervention	Age (mean)	Age (SD)	% of males	HbA1c (mean)	HbA1c (SD)	BMI (mean)	BMI (SD)	Duration of diabetes	% of CV disease	eGFR	ACR	ACR unit
**Hiramatsu 2018**	45	DPP-4 inhibitors	69	7.7		6.7	0.8	23.8	4.8	8.3		45	289	
**Hiramatsu 2018**	90	GLP-1 receptor agonists	70.5	5.7		6.8	0.6	23.5	3.5	9.2		40	380	
**Hirano 2009**	31	Thiazolidinediones	65	13	77			24.6	3.3		100			
**Hirano 2009**	30	Standard treatments	62	12	70			24.9	3		100			
**Hirano 2012**	22	Alpha-glucosidase inhibitors	65	10	82	8	1.8	25	3.9		100			
**Hirano 2012**	22	Standard treatments	65	11	77	8.1	1.7	24.9	3.8		100			
**Hollander 2007**	209	Thiazolidinediones	52.6	10.1	48.1	9	1.2	33.7	7.1	13				
**Hollander 2007**	209	Thiazolidinediones	52.7	9.6	57	8.9	1.1	32.8	7.4	12.5				
**Hollander 2007**	212	Standard treatments	53.8	10.2	46.2	9.1	1.3	33	6.5	12.6				
**HOME**	196	Metformin	64	10	41.3	7.9	1.2	30	5	14				
**HOME**	194	Standard treatments	59	11	50	7.9	1.2	30	5	12				
**Home 2018**	165	DPP-4 inhibitors	57.4	9.2	57.6	8	0.9	31.1	6.1	5.4				
**Home 2018**	164	Standard treatments	57	9.7	59.1	8.1	1	30.8	6.6	5.7				
**Hong 2015**	36	Thiazolidinediones	58.3	11.8	52.8	7.6	1.6	24.6	3.9	2.3	100			
**Hong 2015**	36	Standard treatments	60	12.7	58.3	7.5	1.9	24.4	3.5	2.2	100			
**Hong 2016**	99	DPP-4 inhibitors	56.6	10.1	52.5	7.7	0.6	25	2.5	55.1				
**Hong 2016**	43	Standard treatments	57.9	11.9	65.1	7.7	0.6	25.1	3.2	55.1				
**Horton 2000**	178	Metformin	56.8	10.9	68	8.4	1.2	29.6	4.3	4.5				
**Horton 2000**	179	Meglitinides	58.6	10.7	61.5	8.3	1	29.6	3.8	4.7				
**Horton 2000**	172	Standard treatments	59.6	10.9	60.5	8.3	1.1	29.2	3.9	4.6				
**Hotta 1993**	19	Alpha-glucosidase inhibitors	49.8		73.6	11.1	2	23.5		4.6				

Study ID	N	Intervention	Age (mean)	Age (SD)	% of males	HbA1c (mean)	HbA1c (SD)	BMI (mean)	BMI (SD)	Duration of diabetes	% of CV disease	eGFR	ACR	ACR unit
**Hotta 1993**	18	Standard treatments	47.9		77.8	10.4	1.6	22.9		4.8				
**Hsieh 2011**	52	Alpha-glucosidase inhibitors	58.4	10.5	44	8.1	0.7	25.3	3.1					
**Hsieh 2011**	53	Standard treatments	59	10.7	34	8.1	0.8	26.1	2.9					
**Hwang 2008**	43	Thiazolidinediones	55.1	10	53.5	7.81	0.7	26.5	2.8					
**Hwang 2008**	47	Standard treatments	53.4	9.7	45.7	7.9	1.1	26.6	2.5					
**Hygum 2020**	30	GLP-1 receptor agonists	62	8.383509	53.3			33	5.868456	6				
**Hygum 2020**	30	Standard treatments	64	8.383509	46.7			31.3	5.868456	7				
**Iacobellis 2017**	54	GLP-1 receptor agonists	50	10	41	6.6	0.8	37.8	7.3					
**Iacobellis 2017**	41	Standard treatments	52	10	37	6.4	0.6	32.6	6.7					
**Iacobellis-2020**	50	SGLT2 inhibitors	52	9	42	6.8	0.9	36.6	7.8	5		90		
**Iacobellis-2020**	50	Standard treatments	51	11	40	6.7	0.8	34.7	6	5		91		
**IDDIA**	30	SGLT2 inhibitors								4.7				
**IDDIA**	30	Standard treatments								4.7				
**Ikonomidis 2020**	40	SGLT2 inhibitors	58	10	75	7.8	0.9	29.8	3	6.6		85		
**Ikonomidis 2020**	40	GLP-1 receptor agonists	57	9	67.5	8	1.1	30	4	5.9		85		
**ILLUMINATE**	112	SGLT2 inhibitors	57.7	9.2	58.9	8.3	0.7	26	4.4	7.5				
**ILLUMINATE**	56	Standard treatments	56.2	10.7	58.9	8.4	0.7	25.5	3.1	8.1				
**Inagaki 2012**	215	GLP-1 receptor agonists	57.1	10.4	66	8.5	0.8	26.1	4	8.9				
**Inagaki 2012**	212	Basal insulin	56.4	11.2	69.8	8.5	0.8	26.2	3.8	9.2				

Study ID	N	Intervention	Age (mean)	Age (SD)	% of males	HbA1c (mean)	HbA1c (SD)	BMI (mean)	BMI (SD)	Duration of diabetes	% of CV disease	eGFR	ACR	ACR unit
**Inagaki 2013**	63	Metformin	61.2	10.6	69.8	8	0.1	25.8	4					
**Inagaki 2013**	143	DPP-4 inhibitors	61.8	10.8	73.4	8.1	0.8	24.5	4.2					
**Inagaki 2014**	89	SGLT2 inhibitors	57.4	11.1	81.8	8	0.8	25.4	4.2	5.9		87		
**Inagaki 2014**	90	SGLT2 inhibitors	58.4	10.4	65.6	8	0.7	25.6	4.2	4.7		81		
**Inagaki 2014**	93	Standard treatments	58.2	11	64.5	8	0.7	25.9	4.4	5.6		85		
**Inagaki 2015**	102	DPP-4 inhibitors	58		72	7.7	0.9	25.4	4.4	6.3		113		
**Inagaki 2015**	92	DPP-4 inhibitors	60		75	7.9	0.9	24.7	3.8	7.1		109		
**Inagaki 2015**	51	Standard treatments	62		86	7.7	0.8	24.6	4.3	7.5		98		
**INICOM**	150	Metformin	54	11.3	60.1	8.7	0.9			4.1				
**INICOM**	142	DPP-4 inhibitors	53.4	11	57.1	8.7	0.9			3.5				
**Inoue 2019**	25	SGLT2 inhibitors	60.5	9.8	54.2	8.1	0.9	27.9	4	15.9		91		
**Inoue 2019**	24	Standard treatments	60.8	12.1	58.3	8.3	0.7	27.7	4.5	19.1		83		
**Insulin Glargine 4014**	112	Thiazolidinediones	55.3	11.4	58	8.7	1	33.6	6.3	8.1				
**Insulin Glargine 4014**	104	Basal insulin	55.9	10.5	45	8.8	1	34.6	7	8.5				
**INTERVAL**	139	DPP-4 inhibitors	75.1	4.3	52.5	7.9	0.8	29.1	3.8	12.2				
**INTERVAL**	139	Standard treatments	74.4	4	38.1	7.9	0.7	30.5	4.8	10.6				
**Ito 2011**	30	DPP-4 inhibitors	67	10.95	70	6.7	0.55	22.7	2.74			15		
**Ito 2011**	30	Standard treatments	68	9.17	67	6.7	0.46	22.4	2.29			15		
**Ito 2017**	32	Thiazolidinediones	59.1	9.8	53	8.3	1.4	29.9	6.2	9.5				
**Ito 2017**	34	SGLT2 inhibitors	57.3	12.1	44	8.5	1.5	30.7	5	8.7				
**Jabbour 2014**	225	SGLT2 inhibitors	54.8	10.4	57	7.9	0.8			5.7				
**Jabbour 2014**	226	Standard treatments	55	10.2	52.7	8	0.8			5.6				
**Jacob 2007**	18	Metformin	47	10	61	11.1	1.4	31.9	7.1	8				
**Jacob 2007**	18	Thiazolidinediones	47	11	72	11.2	1.7	30.7	8.5	5				

Study ID	N	Intervention	Age (mean)	Age (SD)	% of males	HbA1c (mean)	HbA1c (SD)	BMI (mean)	BMI (SD)	Duration of diabetes	% of CV disease	eGFR	ACR	ACR unit
**Jacob 2007**	21	Standard treatments	44	13	57	11.2	1.8	32.3	5.6	9				
**Jain 2006**	251	Sulfonylureas	52.1	12.4	56.2	9.2	1.3	32.8	5.7	0.8				
**Jain 2006**	251	Thiazolidinediones	52.1	11.3	53	9.2	1.2	32.5	5.8	0.8				
**J-BOND Study**	126	SGLT2 inhibitors	54.9	11.9	57.3	7.8	1.4	28.4	5	3.5	4			
**J-BOND Study**	127	DPP-4 inhibitors	53.6	11.7	62.1	8	1.6	28.3	4.5	3.1	1.6			
**JEDIS-1**	12	Metformin												
**JEDIS-1**	12	Sulfonylureas												
**JEDIS-1**	12	Alpha-glucosidase inhibitors												
**JEDIS-1**	13	Standard treatments												
**Jeon 2011**	51	Sulfonylureas	55.4	11	60.8	8.1	0.9	23.1	4.2	5.9				
**Jeon 2011**	51	DPP-4 inhibitors	53.5	10.4	68.6	8	1.2	22.7	7.8	5.9				
**Jeon 2018**	162	SGLT2 inhibitors	59.2	12.7	54.3	8.5	0.9			12.1		100		
**Jeon 2018**	148	Basal insulin	56.1	15.2	47.3	8.8	1.4			12.8		99		
**Jerums 1987**	23	Sulfonylureas	49	4	58.8	10.5	2.1			12.7				
**Jerums 1987**	17	Standard treatments	53	5	37.5	9.6	1.9			11.7				
**Ji 2014**	133	SGLT2 inhibitors	51.2	9.9	64.7	8.3	0.9	25.8	3.4	1.7				
**Ji 2014**	128	SGLT2 inhibitors	53	11.1	65.6	8.1	0.7	25.2	3.3	1.2				
**Ji 2014**	132	Standard treatments	49.9	10.9	65.9	8.4	0.9	25.9	3.6	1.3				
**Ji 2016**	124	Metformin	53	10.3	60.5	8.7	1.1	25.8	3.5	1.1				
**Ji 2016**	126	Metformin	52.6	9.5	54.8	8.7	1	26	3.6	1				
**Ji 2016**	120	DPP-4 inhibitors	51.7	10.2	61.7	8.7	1.1	26	3.5	1.1				
**Ji 2016**	127	Standard treatments	53.6	9.7	68.5	9	1.1	25.4	3.4	1.1				
**Ji 2017**	161	Metformin	53.6	9.9	50.6	8.4	0.8	26.3	3.6					

Study ID	N	Intervention	Age (mean)	Age (SD)	% of males	HbA1c (mean)	HbA1c (SD)	BMI (mean)	BMI (SD)	Duration of diabetes	% of CV disease	eGFR	ACR	ACR unit
**Ji 2017**	162	DPP-4 inhibitors	55.4	9.6	60.1	8.5	0.7	26.2	3.9					
**Ji 2017**	161	Standard treatments	52.2	10.2	58.3	8.2	0.8	26.6	4.2					
**Ji-2021**	122	DPP-4 inhibitors	56	9.8	66.4	7.9	0.68	26	3.15	5.05				
**Ji-2021**	124	Standard treatments	54.7	10.1	54	7.87	0.72	26.19	3.22	5.41				
**Ji-2021a**	125	DPP-4 inhibitors	52.1	10.2	68	7.89	0.8	25.64	3.43	2.66				
**Ji-2021a**	126	Standard treatments	56	10.2	62.7	8.04	0.71	25.33	3	2.86				
**Jian 2018**	63	SGLT2 inhibitors												
**Jian 2018**	63	Standard treatments												
**Jiang-2021**	19	SGLT2 inhibitors	58.32	8.01	47	8.23	0.96	26.6	1.32					
**Jiang-2021**	10	Standard treatments	59.3	9.03	20	8.49	0.75	25.64	1.41					
**Jibran 2006**	50	Sulfonylureas	45.8	8.8	20	10.2	1.6	30.4	5.6					
**Jibran 2006**	50	Meglitinides	46.6	10.5	32	9.9	1.6	27.1	3.5					
**Johnston 1998**	254	Standard treatments	52.9		60	8.7		31.8		5.7				
**Johnston 1998**	131	Alpha-glucosidase inhibitors	53.9		52	8.5		30.6		4.8				
**Johnston 1998a**	224	Alpha-glucosidase inhibitors	55	14.3	50	8.7	2	31.9	10	5.1			158	
**Johnston 1998a**	116	Standard treatments	56.9	13.3	51	8.6	1.8	32	9.2	4.5			215	
**Johnston 1998b**	92	Sulfonylureas	67.7	5.8	64.1	8.4	1	29.3	5.8	7.2				
**Johnston 1998b**	85	Alpha-glucosidase inhibitors	67.8	6.5	71.8	8.4	0.9	29.4	5.5	6.8				
**Johnston 1998b**	95	Alpha-glucosidase inhibitors	67.2	5.9	63.2	8.3	1	29.7	5.9	7.5				
**Johnston 1998b**	92	Standard treatments	68.5	5.8	71.7	8.4	1	30.4	5.8	7				

Study ID	N	Intervention	Age (mean)	Age (SD)	% of males	HbA1c (mean)	HbA1c (SD)	BMI (mean)	BMI (SD)	Duration of diabetes	% of CV disease	eGFR	ACR	ACR unit
**Josse 2003**	93	Alpha-glucosidase inhibitors	69.7	5	69.9	7.4	1	28.3	4	5.8				
**Josse 2003**	99	Standard treatments	70.3	5	60.6	7.3	1	28.6	4	4.8				
**Jovanovic 2000**	146	Meglitinides	57.6		60	8.7	1.7	29.5	4.4	6.3				
**Jovanovic 2000**	140	Meglitinides	57.9		69	8.9	1.9	29.4	4.5	6.6				
**Jovanovic 2000**	75	Standard treatments	58.5		65	8.6	1.4	29.8	4.3	6.8				
**Jovanovic 2004**	62	Thiazolidinediones	56.2	12.2	50	9.3		32.1	5.3	6.1				
**Jovanovic 2004**	61	Meglitinides	57.8	13.1	59	9		31.2	5.3	6.9				
**Jung 2005**	15	Metformin	54	14	46.15	9	0.8	24.6	2.4	7	15.4			
**Jung 2005**	15	Thiazolidinediones	60	8	42.86	9.3	0.9	23.3	2.6	9	14.3			
**Juurinen 2009**	40	Meglitinides	55.3	8.2	60	7.5	0.6	33.6	4.4	9.5				
**Juurinen 2009**	41	Standard treatments	56.6	8.3	51	7.4	0.6	32.1	4.5	9.3				
**Kadoglou 2007**	25	Thiazolidinediones	59.04	7.35	40	8.53	1.26	30.04	2.99	5.29				
**Kadoglou 2007**	25	Standard treatments	60.32	9.28	39.1	8.03	0.91	28.96	1.03	5.78				
**Kadoglou 2008**	35	Thiazolidinediones	63.8	7.3	40	8.2	1.2	29.5	3.82	8.5				
**Kadoglou 2008**	35	Standard treatments	66.7	9.6	45.7	8	0.8	29.9	4.3	7.5				
**Kadoglou 2011**	70	Thiazolidinediones	62	8.3	27	7.58	0.6	29.68	2.47	1.8				
**Kadoglou 2011**	70	Standard treatments	62.7	6.8	28	7.56	0.64	30.04	4.85	2.7				
**Kadowaki 2011**	72	GLP-1 receptor agonists	59.4	89.8	68.1	8.2	1	25.8	3.9	11.6				
**Kadowaki 2011**	72	GLP-1 receptor agonists	58.5	9.3	68.1	8.3	0.8	25	4.1	12.2				
**Kadowaki 2011**	35	Standard treatments	56.3	11.4	68.6	8.1	0.9	25.8	4.2	12.4				
**Kadowaki 2017**	70	SGLT2 inhibitors	58.4	8.9	77.1	8.2	0.9	25.5	4.2	8.3				

Study ID	N	Intervention	Age (mean)	Age (SD)	% of males	HbA1c (mean)	HbA1c (SD)	BMI (mean)	BMI (SD)	Duration of diabetes	% of CV disease	eGFR	ACR	ACR unit
**Kadowaki 2017**	68	Standard treatments	56	9.5	77.9	7.9	0.8	26.4	3.9	6.5				
**Kadowaki 2018**	77	DPP-4 inhibitors	55.9	8.3	83.1	8	0.8	25.5	4	8.2				
**Kadowaki 2018**	77	Standard treatments	54.1	10.2	75.3	8.1	0.9	26.5	4.8	7.3				
**Kaku 2009**	83	Thiazolidinediones	52	8.6	66.3	7.6	1	25.6	4.2	4.5				
**Kaku 2009**	86	Standard treatments	53	7.5	43	7.6	0.9	25.4	3.6	5.6				
**Kaku 2009a**	293	Thiazolidinediones	58.1		63	7.6		26.5			9.6			
**Kaku 2009a**	294	Standard treatments	57.9		62	7.5		26.9			7.5			
**Kaku 2011**	132	Sulfonylureas	58.5	10.4	65.2	9.2	1	24.6	3.8	8.5				
**Kaku 2011**	268	GLP-1 receptor agonists	58.2	10.4	68.3	9.3	1.1	24.9	3.7	8.1				
**Kaku 2014**	88	SGLT2 inhibitors	57.5	9.3	60.2	7.5	0.6	26.1	4.5	4.9	50	67		
**Kaku 2014**	86	SGLT2 inhibitors	58.6	10.4	58.1	7.5	0.7	24.9	3.9	4.6	44.2	67		
**Kaku 2014**	87	Standard treatments	60.4	9.7	59.8	7.5	0.6	25.2	4.4	5.3	42.5	68		
**Kaku 2017**	152	Metformin	57.6	9.7	70.2	7.9	0.8			7				
**Kaku 2017**	151	Metformin	56.9	8.8	72.4	7.8	0.8			7				
**Kaku 2017**	71	Standard treatments	57.2	10.1	73.2	7.8	0.8			7.7				
**Kaku 2019_1**	107	DPP-4 inhibitors	58	9.3	79.4	8.3	0.5	24.9	4	8.4		97		
**Kaku 2019_1**	108	Standard treatments	56.3	9.9	78.7	8.4	0.7	25.1	3.6	7.6		96		
**Kaku 2019_2**	116	DPP-4 inhibitors	56.8	10.6	75	8.3	0.6	25	4.3	8.5		96		
**Kaku 2019_2**	116	Standard treatments	58.4	9.2	68.1	8.3	0.7	24.7	3.7	8.3		95		
**Kanazawa 2010**	23	Metformin	66	10	56.5	7.9	1.3	24.9	3.7	12				
**Kanazawa 2010**	22	Thiazolidinediones	67	10	63.6	7.9	1.7	22	2.3	14				
**Kanazawa 2011**	22	Metformin	64	6.9	50	7.7	1.9	24.4	4.2	13.8				
**Kanazawa 2011**	22	Thiazolidinediones	65.2	10.1	63.6	7.9	1.4	24.6	3.9	13.9				

Study ID	N	Intervention	Age (mean)	Age (SD)	% of males	HbA1c (mean)	HbA1c (SD)	BMI (mean)	BMI (SD)	Duration of diabetes	% of CV disease	eGFR	ACR	ACR unit
**Kanazawa 2011**	22	Standard treatments	65.4	9.1	54.5	7.9	1.7	23.7	3.7	14				
**Kang-2021**	79	GLP-1 receptor agonists	49.37	8.43	53.16	9.73	1.97	27.61	2.03	3				
**Kang-2021**	80	Basal insulin	47.63	9.65	56.25	9.64	1.55	27.06	1.94	3				
**Kashyap-2020**	11	SGLT2 inhibitors	57.25	11.61171	36.4	7.3	0.564894	39.3	6.527666					
**Kashyap-2020**	5	Standard treatments	45.25	10.59537	20	8.325	1.313825	38.1	5.424827					
**Kato 2015**	10	Sulfonylureas	53	17.20007	50	7.166667	0.516002	28	6.364026					
**Kato 2015**	10	DPP-4 inhibitors	62.66667	12.04005	60	7.233333	0.430002	27.6	6.708027					
**Kawamori 2018**	182	SGLT2 inhibitors	60	9.9	78	8.3	0.7	26	3.8	9		89		
**Kawamori 2018**	93	Standard treatments	59.8	10.8	77.4	8.4	0.7	26.6	4.5	8.7		86		
**Kayano-2020**	36	SGLT2 inhibitors	69.4	7.1	86.1	7.6	0.7	25.6	4.2	8.9	72.2		184.7	mg/g
**Kayano-2020**	38	Standard treatments	66	9.5	92.1	7.5	0.6	25.8	3.9	8.1	63.3		117	mg/g
**Kelly 2007**	16	Sulfonylureas	63.1	7.8	62.5	7.3	0.7							
**Kelly 2007**	20	Thiazolidinediones	57.9	8.2	45	7.8	1.1							
**Kendall 2005**	241	GLP-1 receptor agonists	55	10	59.3	8.5	1.1	34	6	8.7				
**Kendall 2005**	245	GLP-1 receptor agonists	55	9	59.2	8.5	1	33	6	8.7				
**Kendall 2005**	247	Standard treatments	56	10	55.9	8.5	1	34	5	9.4				
**Khaloo 2019**	125	Thiazolidinediones	62.7	8.2	41.5	9	1.2	29	4.8	14.3				
**Khaloo 2019**	125	DPP-4 inhibitors	60.8	8.1	58.5	8.8	1.1	29.7	4.3	11.3				
**Khan-2022**	60	SGLT2 inhibitors	52.28	7.55	46.7	9.42	1.48							
**Khan-2022**	60	DPP-4 inhibitors	49.4	9.48	35	9.17	1.36							
**Khanolkar 2008**	25	Sulfonylureas	56	37.55	60	7.08	0.7	33.66	7.76					

Study ID	N	Intervention	Age (mean)	Age (SD)	% of males	HbA1c (mean)	HbA1c (SD)	BMI (mean)	BMI (SD)	Duration of diabetes	% of CV disease	eGFR	ACR	ACR unit
**Khanolkar 2008**	25	Thiazolidinediones	59	37.55	56	7.33	0.5	34.55	8.38					
**Kikuchi 2012**	159	Thiazolidinediones	55	10.6	62.9	8.9	1.3	24.5	3.7	5				
**Kikuchi 2012**	159	Thiazolidinediones	56	10.3	62.3	8.8	1.3	24.9	3.5	4.2				
**Kikuchi 2012**	54	Standard treatments	53.9	10	61.1	9	1.4	25	3.6	4.2				
**Kim 2014**	115	Thiazolidinediones	56.4	9.3	57.4	8	1	25.3	2.8	4.3				
**Kim 2014**	58	Standard treatments	54.7	9.7	55.2	8.1	0.9	25.1	2.2	4.9				
**Kim 2019**	66	Sulfonylureas	58.5	10.4	45.5	8.2	0.6	24.9	2.8	9.7				
**Kim 2019**	69	Thiazolidinediones	60.7	9.1	49.3	8.3	0.6	24.4	3.2	10.6				
**Kim 2020**	125	DPP-4 inhibitors	58	9.9	44	7.8	0.7	26.5	3.3	7				
**Kim 2020**	118	Thiazolidinediones	57.8	8.8	54.2	7.9	0.7	26.3	3.9	7.9				
**Kim-2022**	35	Sulfonylureas	56.46	9.17	54.29	8.15	0.58	26.03	3.17	4.42				
**Kim-2022**	31	DPP-4 inhibitors	52.68	11.93	45.16	8.33	0.73	25.85	3.94	4.68				
**KIND-LM**	24	Metformin	51	11	66.7			28.7	3.7	4.7				
**KIND-LM**	22	GLP-1 receptor agonists	55	11	59.1			28.6	4.2	5.6				
**Kinoshita 2020**	33	Sulfonylureas	58	13.2	45.5	7.6	1.1	28.4	4	7.2				
**Kinoshita 2020**	33	Thiazolidinediones	59	10.9	45.5	7.4	1.1	28.7	5.2	7.9				
**Kinoshita 2020**	32	SGLT2 inhibitors	58.7	9.1	46.9	7.4	1.1	29.5	4.5	6.6				
**Kiyici 2009**	16	Metformin	52.4	8.3		6.7	0.9	31.6	3.6					
**Kiyici 2009**	19	Thiazolidinediones	50.7	6.4		7.1	0.9	31.2	5.9					
**Kiyici 2009**	15	Standard treatments	52.1	5.1		6.4	0.6	30	3.2					
**Ko 2006**	56	Thiazolidinediones	56.6	10.7	57.1	10.1	1	25.3	3.8	11.8				
**Ko 2006**	56	Basal insulin	59.8	11.2	42.9	9.6	0.9	24	2.7	13.6				
**Koffert 2017**	13	Metformin	57.8	7.9	61.5	6.9	0.7	29.9	13.7					

Study ID	N	Intervention	Age (mean)	Age (SD)	% of males	HbA1c (mean)	HbA1c (SD)	BMI (mean)	BMI (SD)	Duration of diabetes	% of CV disease	eGFR	ACR	ACR unit
**Koffert 2017**	14	Thiazolidinediones	58.6	7.5	71.4	6.8	0.8	29.1	14.2					
**Koffert 2017**	14	Standard treatments	57.5	7.1	71.4	6.3	0.4	30.3	16.3					
**Kohan 2014**	85	SGLT2 inhibitors	68	7.7	65.9	8.2	1			18.2		44	73	
**Kohan 2014**	83	SGLT2 inhibitors	66	8.9	66.3	8.3	1			16.9		44	79	
**Kohan 2014**	84	Standard treatments	67	8.6	63.1	8.5	1.3			15.7		46	67	
**Kondo 2016**	85	Sulfonylureas	64	8	72.1	7.5	0.5	24.7	3.3	6		75		
**Kondo 2016**	86	DPP-4 inhibitors	63	9	75.4	7.4	0.5	24.1	3.8	6.2		76		
**Kothny 2013**	228	DPP-4 inhibitors	59.3	9.9	47.8	8.8	1	28.9	4.4	12.9		78		
**Kothny 2013**	221	Standard treatments	59.1	10.1	52	8.8	1	29	4.6	13.2		82		
**Krawczyk 2005**	20	Metformin	55.8	8.14	40	8.58	1.89	35.4	3.83	12.2				
**Krawczyk 2005**	20	Standard treatments	58.4	5.96	60	8.44	1.64	33	3.67	11.9				
**Kudo-Fujimaki 2014**	20	Meglitinides	66.3	10	58.8	7.6	0.6	25	4.1	11.8				
**Kudo-Fujimaki 2014**	20	Standard treatments	63	13.2	78.9	7.5	0.6	24.7	2.6	11.6				
**Lam 1998**	44	Alpha-glucosidase inhibitors	57.8	8.7	44.4	9.4	0.7	24.8	3.4	10.1				
**Lam 1998**	45	Standard treatments	56.9	8.6	43.2	9.4	0.7	24.1	2.6	10.2				
**Lambadiari 2018**	30	Metformin	50	12	66.7	8.4	1.2	27.7	2			83		
**Lambadiari 2018**	30	GLP-1 receptor agonists	51	10	66.7	8.6	2	32.9	5			85		
**LANTERN**	119	SGLT2 inhibitors	63.9	6.6	78	7.5	0.5	25.8	3.5	9.5		60		
**LANTERN**	46	Standard treatments	65.7	6.9	78.3	7.6	0.5	25	3.4	9.4		63		
**Lawrence 2004**	21	Metformin	59.5	9.3	60	8	0.9	29.63333	2.792461					
**Lawrence 2004**	22	Sulfonylureas	63.5	11.4	65	8	0.9	30.46667	4.866861					
**Lawrence 2004**	21	Thiazolidinediones	60.4	7.5	70	7.4	0.9	31.73333	4.627507					

Study ID	N	Intervention	Age (mean)	Age (SD)	% of males	HbA1c (mean)	HbA1c (SD)	BMI (mean)	BMI (SD)	Duration of diabetes	% of CV disease	eGFR	ACR	ACR unit
**LEAD-1**	232	Thiazolidinediones	55.6	9.8	47	8.4	1	29	4.8	6.6				
**LEAD-1**	234	GLP-1 receptor agonists	55.6	10	53	8.5	0.9	30	5	6.5				
**LEAD-1**	228	GLP-1 receptor agonists	57.7	9	45	8.5	1.1	29.8	5.1	6.7				
**LEAD-1**	233	GLP-1 receptor agonists	55.7	9.9	54	8.4	1	30	5	6.5				
**LEAD-1**	114	Standard treatments	56	9.8	47	8.4	1	29.4	4.8	6.6				
**LEAD-2**	244	Sulfonylureas	57	9	57	8.4	1	31.2	4.6	8				
**LEAD-2**	242	GLP-1 receptor agonists	57	9	57	8.4	1	30.9	4.6	8				
**LEAD-2**	241	GLP-1 receptor agonists	57	9	54	8.3	1	31.1	4.8	7				
**LEAD-2**	242	GLP-1 receptor agonists	56	11	62	8.4	0.9	30.5	4.8	7				
**LEAD-2**	122	Standard treatments	56	9	60	8.4	1.1	31.6	4.4	8				
**LEAD-3**	248	Sulfonylureas	53.4	10.9	54	8.4	1.2	33.2	5.6	5.6				
**LEAD-3**	246	GLP-1 receptor agonists	52	10.8	49	8.3	1.1	32.8	6.3	5.3				
**LEAD-3**	251	GLP-1 receptor agonists	53.7	11	47	8.3	1	33.2	5.6	5.2				
**LEAD-4**	178	GLP-1 receptor agonists	55	11	51	8.6	1.2	33.5	5.1	9				

Study ID	N	Intervention	Age (mean)	Age (SD)	% of males	HbA1c (mean)	HbA1c (SD)	BMI (mean)	BMI (SD)	Duration of diabetes	% of CV disease	eGFR	ACR	ACR unit
**LEAD-4**	178	GLP-1 receptor agonists	55	10	57	8.5	1.2	33.2	5.4	9				
**LEAD-4**	177	Standard treatments	55	10	62	8.4	1.2	33.9	5.2	9				
**LEAD-5**	232	GLP-1 receptor agonists	57.6	9.5	57	8.3	0.9	30.4	5.3	9.2				
**LEAD-5**	234	Basal insulin	57.5	10.5	60	8.2	0.9	30.3	5.3	9.1				
**LEAD-5**	115	Standard treatments	57.5	9.6	49	8.3	0.9	31.3	5	9.4				
**LEADER**	4668	GLP-1 receptor agonists	64.2	7.2	64.5	8.7	1.6	32.5	6.3	12.8	82.1			
**LEADER**	4672	Standard treatments	64.4	7.2	64	8.7	1.5	32.5	6.3	12.9	80.6			
**Lebovitz 2001**	169	Thiazolidinediones	61	9.5	66.9	8.8	1.6	29.1	3.9	5.4				
**Lebovitz 2001**	166	Thiazolidinediones	60	9.8	64.5	9	1.5	30.2	4.4	4.8				
**Lebovitz 2001**	158	Standard treatments	59	10.9	65.8	9	1.7	29.9	4.1	4.6				
**Ledesma 2019**	151	DPP-4 inhibitors	72.3	5.1	60.9	8.2	0.8	28.3	5.6			66		
**Ledesma 2019**	151	Standard treatments	72.5	5.6	60.3	8.1	0.7	27.9	5.7			70		
**Lee 2013**	60	Thiazolidinediones	60.3	9.5	71.7	8.1	1.6	24	3	6	100			
**Lee 2013**	61	Standard treatments	61.9	8.8	75.4	7.4	1.8	23.8	3.2	5.5	100			
**Lee 2017**	154	DPP-4 inhibitors	57.2	8.4	47	8.5	0.8	31.8	6.2	9.8				
**Lee 2017**	153	Standard treatments	58.4	9.4	48	8.6	0.8	30.6	5.8	10.4				
**Lee-2013a**	25	Thiazolidinediones	61.4	12.4	64			24.8	4.1					
**Lee-2013a**	25	Standard treatments	59.5	11.8	56			24.1	3					
**Lee-2022**	30	SGLT2 inhibitors	56.9	10.7	53.3	8.94	0.6	26.4	3.87	17.1	13.3	90.4		
**Lee-2022**	30	DPP-4 inhibitors	60.6	7.03	66.7	8.79	0.56	26.9	2.9	19.3	13.3	81.2		
**Leiter 2014**	246	DPP-4 inhibitors	63.5	9	52.8	8.2	0.9	30.4	5.8	11.6	8.9			

Study ID	N	Intervention	Age (mean)	Age (SD)	% of males	HbA1c (mean)	HbA1c (SD)	BMI (mean)	BMI (SD)	Duration of diabetes	% of CV disease	eGFR	ACR	ACR unit
**Leiter 2014**	249	GLP-1 receptor agonists	63.2	8.4	54.6	8.1	1	30.4	5.5	10.8	8.4			
**Leiter 2014a**	482	SGLT2 inhibitors	63.9	7.6	66.9	8	0.8	33	5.3	13.5				
**Leiter 2014a**	483	Standard treatments	63.6	7	67	8.1	0.8	32.7	5.7	13				
**Leonhardt 1991**	47	Alpha-glucosidase inhibitors												
**Leonhardt 1991**	47	Standard treatments												
**Lewin 2007**	146	Metformin	53	10.9	51.4			34	7.1	4.9				
**Lewin 2007**	141	Metformin	53	10.5	58.9			34	6.3	5				
**Lewin 2007**	144	Metformin	54	10.3	53.5			35	7.1	5.1				
**Lewin 2007**	144	Standard treatments	53	10.7	54.9			34	7.1	5.8				
**Lewin 2015**	133	DPP-4 inhibitors	53.8	11.5	56.4	8.1	0.9	31.9	5.9			90		
**Lewin 2015**	133	SGLT2 inhibitors	56	9.3	57.9	8	1	31.2	5.7			89		
**Lewin 2015**	132	SGLT2 inhibitors	53.9	10.5	48.5	8.1	1	31.5	5.7			88		
**Li 2014**	67	DPP-4 inhibitors	46.4	9.8	60	8.6	0.8	25.9	1.8	5.4				
**Li 2014**	68	DPP-4 inhibitors	47	11.3	65	8.6	0.9	26.3	2.2	5.4				
**Li 2014**	68	GLP-1 receptor agonists	47.9	10.8	59	8.5	0.8	26.7	2.4	5.8				
**Li 2014a**	22	Basal insulin												
**Li 2014a**	20	Standard treatments												
**Li 2017**	17	DPP-4 inhibitors	58.9	9	35.3	8.5	0.8	26.1	3.1	8.6				
**Li 2017**	16	Standard treatments	59.9	7.7	56.3	8.8	0.7	26.1	3.4	8.6				
**Li 2019**	33	Sulfonylureas	47.4	9.4	51.5	7.8	0.6	26.5	3.2	0.9				
**Li 2019**	31	DPP-4 inhibitors	46.6	8.2	46.7	7.8	0.5	27.2	4.1	0.9				

Study ID	N	Intervention	Age (mean)	Age (SD)	% of males	HbA1c (mean)	HbA1c (SD)	BMI (mean)	BMI (SD)	Duration of diabetes	% of CV disease	eGFR	ACR	ACR unit
**Li 2019a**	10	Sulfonylureas	53.3	6.6	60	7.9	1							
**Li 2019a**	13	GLP-1 receptor agonists	54.8	5.3	53.8	8.4	0.9							
**Li 2020**	75	DPP-4 inhibitors	60.6	9.5	73.3	8.2	1.5	25.1	3.7	6.3	100			
**Li 2020**	74	Standard treatments	59.7	10.3	79.7	7.9	0.9	24.9	3.1	9.9	100			
**LIBRA**	26	GLP-1 receptor agonists	58.9	8.7	61.5	6.2	0.4	30	4.3	3				
**LIBRA**	25	Standard treatments	57.4	7.4	64	6.4	0.5	30.4	5.8	1.5				
**Lim 2017**	112	Thiazolidinediones	56.4	9.3	58	7.7	0.8	25.3	2.8	4.3				
**Lim 2017**	58	Standard treatments	54.7	9.7	55.2	7.9	0.9	25.1	2.2	4.9				
**Lin 2003**	33	Alpha-glucosidase inhibitors	57.7	7.3	53.1	9	1.2	24.8	3	7				
**Lin 2003**	32	Standard treatments	55.4	8.5	37.5	9	1	25.1	2.8	5				
**Linagliptin-LTC Trial**	67	DPP-4 inhibitors	68	14	48	7.9	2.2	29.7	7	10.9				
**Linagliptin-LTC Trial**	73	Basal insulin	71.5	13	34	7.8	2.2	30	8	10.6				
**Lindstrom 2000**	55	Alpha-glucosidase inhibitors	58.3	9		9.8	1.3	29.1	3.3	6.3				
**Lindstrom 2000**	52	Standard treatments	61.8	5.2		10.1	1.5	28.3	3.7	6				
**Lingvay 2018**	65	GLP-1 receptor agonists	55.8	9.2	33	8.1	0.8	32.1	4.5	6.6				
**Lingvay 2018**	64	GLP-1 receptor agonists	53.7	11.4	53.1	8.1	0.9	33.3	4.3	6.9				

Study ID	N	Intervention	Age (mean)	Age (SD)	% of males	HbA1c (mean)	HbA1c (SD)	BMI (mean)	BMI (SD)	Duration of diabetes	% of CV disease	eGFR	ACR	ACR unit
**Lingvay 2018**	64	GLP-1 receptor agonists	59.5	9.8	50	8.1	0.8	33	4	6.8				
**Lingvay 2018**	64	GLP-1 receptor agonists	57.2	10.8	45.3	8.1	0.9	32.9	3.9	8.1				
**Lingvay 2018**	63	GLP-1 receptor agonists	54.8	9.7	50.8	8.2	0.8	33.1	4.7	6.5				
**Lingvay 2018**	65	GLP-1 receptor agonists	58.4	9.6	50.8	8	0.8	32.8	4.5	7.2				
**Lingvay 2018**	63	GLP-1 receptor agonists	57.5	10	55.6	7.9	0.8	32.4	4.5	8.1				
**Lingvay 2018**	64	GLP-1 receptor agonists	57.5	9.8	51.6	7.9	0.7	32.3	4.6	6.5				
**Lingvay 2018**	129	Standard treatments	57.1	9.2	55.8	8.1	0.9	32.8	4.2	7.2				
**LIPER2**	12	GLP-1 receptor agonists	53.2	9.7	41.7	8	0.5	35	6.2	8.7				
**LIPER2**	12	Standard treatments	52.6	13.8	33.3	8.4	0.8	35	6.2	8.7				
**LIRA-ADD2SGLT2i**	203	GLP-1 receptor agonists	54.7	10.1	62	8	0.7	32	6	10.1				
**LIRA-ADD2SGLT2i**	100	Standard treatments	56	9.9	58	8	0.6	32.6	6.5	9.6				
**LIRAFLAME**	51	GLP-1 receptor agonists	65.9	8.6	88.2	7.5	0.88	30.5	5.3	11.93	21.6	82.7	8.17	mg/g
**LIRAFLAME**	51	Standard treatments	66.9	7.8	80.4	7.5	0.97	29.3	3.8	11.7	15.7	83.7	8	mg/g
**Liraglutide-Detemir Study**	162	Basal insulin	56.8	9.4	54.3	7.6	0.6	34.9	6.3	8.6				

Study ID	N	Intervention	Age (mean)	Age (SD)	% of males	HbA1c (mean)	HbA1c (SD)	BMI (mean)	BMI (SD)	Duration of diabetes	% of CV disease	eGFR	ACR	ACR unit
**Liraglutide-Detemir Study**	161	Standard treatments	57.3	9.8	55.3	7.6	0.7	33.9	6	8.5				
**LIRA-RENAL**	140	GLP-1 receptor agonists	68	8.3	53.6	8.1	0.8	33.4	5.4	15.9				
**LIRA-RENAL**	137	Standard treatments	66.3	8	47.4	8	0.9	34.5	5.4	14.2				
**LIRA-SWITCH**	202	DPP-4 inhibitors	56.5	9.7	61	8.2	0.6	32.2	6.2	7.6				
**LIRA-SWITCH**	204	GLP-1 receptor agonists	56.3	10.6	57.4	8.3	0.6	31.7	6	7.9				
**Liu 2013**	59	Thiazolidinediones	58.1	8.4	38.3	8.5	1	25.7	3.7	7.8				
**Liu 2013**	60	DPP-4 inhibitors	60.1	8.9	36.7	8.3	0.9	26.6	4.6	7.8				
**Liu 2014**	70	Thiazolidinediones	60.7	12.3	74.3	6.4	1.8							
**Liu 2014**	76	Standard treatments	62.3	12.4	65.8	6.2	1.5							
**Liu 2020**	35	GLP-1 receptor agonists	47.6	10.1	54.3	8.3	0.9	28.5	3	0.3				
**Liu 2020**	36	Basal insulin	50.6	11.8	52.8	8.6	0.9	27.8	3.1	0.5				
**Liu-2021**	53	DPP-4 inhibitors	59.1	10.2	28.3	9	1.2	27.9	3.5	11.1		83.9	145	mg/g
**Liu-2021**	53	SGLT2 inhibitors	58	10.4	49.1	9.4	1.5	27.8	4.8	12.6		81.4	233	mg/g
**Liutkus 2010**	111	GLP-1 receptor agonists	55	8	60	8.2	0.9	34	6	6.3				
**Liutkus 2010**	54	Standard treatments	54	9	57	8.3	0.9	33	5	6.4				
**Lou 2020**	43	Metformin	62.2	7.2	69			31.2	1.5	3.3				
**Lou 2020**	43	GLP-1 receptor agonists	61.8	7.7	71.4			31.1	1.7	3.5				
**Lu 2016**	87	SGLT2 inhibitors	53.9	11.3	50.6	7.7	0.8	26.6	4.3	6.5				

Study ID	N	Intervention	Age (mean)	Age (SD)	% of males	HbA1c (mean)	HbA1c (SD)	BMI (mean)	BMI (SD)	Duration of diabetes	% of CV disease	eGFR	ACR	ACR unit
**Lu 2016**	83	Standard treatments	53.4	11.3	39.8	7.8	0.7	27	4.1	5.8				
**Lu-2021**	156	SGLT2 inhibitors	53.3	9.6	58.7	8.59	0.89	25.4	3.1	2.15		120		
**Lu-2021**	156	SGLT2 inhibitors	52.2	9.4	76.2	8.59	0.91	25.5	3.1	2.22		122		
**Lu-2021**	156	Standard treatments	52.4	10.2	66.2	8.63	0.89	26	2.9	2.4		124		
**Lukashevich 2011_1**	165	DPP-4 inhibitors	67.7	8.8	58.2	7.8	1	30.2	5.1	15				
**Lukashevich 2011_1**	129	Standard treatments	69.7	7.3	62	7.8	0.9	30	5	15.2				
**Lukashevich 2011_2**	124	DPP-4 inhibitors	64.1	9.2	52.4	7.7	1	30.2	5.6	17.3				
**Lukashevich 2011_2**	97	Standard treatments	64.5	10.8	54.6	7.7	1	29.5	5	19				
**Lund 2009**	52	Metformin	63	7.8	59.6	7.8	1	24.5	2.3	8				
**Lund 2009**	49	Meglitinides	63.7	7.9	63.3	7.8	1.2	24.9	2.5	12				
**LYDIA**	38	GLP-1 receptor agonists	43.4	7	52.6	7.5	0.8	35.7	7	4.5		87.6		
**LYDIA**	38	DPP-4 inhibitors	44.8	5.9	39.5	7.6	0.8	34.9	5.3	4.4		89.1		
**Macauley 2015**	22	DPP-4 inhibitors	65.2	3.3		6.6	0.5	29.4	3.8					
**Macauley 2015**	22	Standard treatments	58.9	7.5		6.5	0.5	31.1	2.8					
**Madsbad 2001**	81	Sulfonylureas	62	8.8	64.2	7.2	1.4	28	3.5	7				
**Madsbad 2001**	175	Meglitinides	60.2	8.1	61.1	7.3	1.2	28	3.6	8.1				
**Maffioli 2013**	84	Sulfonylureas	61.4	5.6	50			30.2	2.9					
**Maffioli 2013**	86	Thiazolidinediones	62.8	6.3	47.7			30	3					
**MAGNA VICTORIA**	23	GLP-1 receptor agonists	60	6	61	8.4	1.1	32.6	4.4	11				
**MAGNA VICTORIA**	26	Standard treatments	59	7	58	8.2	1	31.6	3.4	11				
**Marbury 1999**	193	Sulfonylureas	58.7	9	66	8.9	1.6	29.1	3.7	8.3				
**Marbury 1999**	383	Meglitinides	58.3	9.4	67	8.7	1.7	29.4	3.7	7.2				

Study ID	N	Intervention	Age (mean)	Age (SD)	% of males	HbA1c (mean)	HbA1c (SD)	BMI (mean)	BMI (SD)	Duration of diabetes	% of CV disease	eGFR	ACR	ACR unit
**MARCH**	393	Metformin	50.2	9.3	60	7.6	1.2	25.7	2.7	0.3				
**MARCH**	391	Alpha-glucosidase inhibitors	50.6	9.2	61	7.5	1.3	25.5	2.7	0.2				
**Mari 2008**	156	DPP-4 inhibitors	63.3	10.2	59.6	6.7	0.4	30.4	4.9	2.5				
**Mari 2008**	150	Standard treatments	62.8	11	59.3	6.8	0.4	30	4.9	2.7				
**MARLINA-T2D**	182	DPP-4 inhibitors										103	120.8	
**MARLINA-T2D**	178	Standard treatments										94	131.9	
**Marre 2002**	160	Meglitinides	57.3	10.5	61.3	8.2		29.3	3.5	6.8				
**Marre 2002**	155	Meglitinides	57.9	9.9	61.3	8		29.4	3.7	7.2				
**Marre 2002**	152	Standard treatments	56.4	10.3	55.3	8.2		29.6	3.9	6.5				
**MASTER**	14	DPP-4 inhibitors	59.2	11.8	78.5	7.5	0.9	28.8	2.5	7.6				
**MASTER**	14	Alpha-glucosidase inhibitors	58.7	7	78.5	6.9	0.5	29.5	5.5	9.3				
**Mathieu 2015**	329	DPP-4 inhibitors	59.3	8.9	45.9	8.7	1	31.9	5.8	13.2				
**Mathieu 2015**	329	Standard treatments	58.3	9.7	49.8	8.8	1	32.2	6.6	13.7				
**Mathieu 2016**	160	SGLT2 inhibitors	55.2	8.6	43.7	8.24	0.96	31.2	4.7	7.2		94		
**Mathieu 2016**	160	Standard treatments	55	9.6	47.5	8.17	0.98	32.2	5.3	8		92		
**Matthaei 2015**	160	DPP-4 inhibitors	54.7	9.8	47.7	7.97	0.83	31.4	5.2	8.1		93		
**Matthaei 2015**	162	Standard treatments	54.5	9.3	46.9	7.86	0.93	31.4	5.3	7.4		94		
**Matthaei 2015a**	109	SGLT2 inhibitors	61.1	9.7	42.6	8.08	0.91	31.9	4.8	9.3	84.3			
**Matthaei 2015a**	109	Standard treatments	60.9	9.2	55.6	8.24	0.87	32	4.6	9.6	88			
**Matthews 2005**	313	Sulfonylureas	57	9	49.2	8.5	0.9	32.6	5.8	5.5				
**Matthews 2005**	317	Thiazolidinediones	56	9.2	50.8	8.7	1	32.6	5	5.8				
**Matthews 2010**	1556	Sulfonylureas	57.5	9.2	53.9	7.3	0.7	31.7	5.3	5.7				

Study ID	N	Intervention	Age (mean)	Age (SD)	% of males	HbA1c (mean)	HbA1c (SD)	BMI (mean)	BMI (SD)	Duration of diabetes	% of CV disease	eGFR	ACR	ACR unit
**Matthews 2010**	1562	DPP-4 inhibitors	57.5	9.1	53.1	7.3	0.7	31.9	5.3	5.7				
**Mattoo 2005**	142	Thiazolidinediones	58.8	7.4	43.7	8.9	1.3	32.5	4.8	13.6				
**Mattoo 2005**	147	Standard treatments	58.9	6.9	42.9	8.8	1.2	31.8	5	13.4				
**McCluskey 2004**	25	Sulfonylureas	60.2	7.8	44	7.9	0.6			7.2				
**McCluskey 2004**	15	Standard treatments	50.8	9.7	40	8.4	0.7			4.6				
**McGill 2013**	68	DPP-4 inhibitors	64	10.9	66.2	8.2	1.1	32.3	5.8			22		
**McGill 2013**	65	Standard treatments	64.9	9.6	53.8	8.2	0.9	31.7	5.9			25		
**McGuire 2010**	74	Thiazolidinediones	57	8.7	57.4	7.6	1.8	34.1	6.4	9.5				
**McGuire 2010**	76	Standard treatments	55.7	8.3	61.1	7.6	1.8	34.1	7.3	8.7				
**MDI Liraglutide**	64	GLP-1 receptor agonists	63.7	8.2	62.5	9	1	33.7	4.3	17.3	9.4			
**MDI Liraglutide**	60	Standard treatments	63.5	7.7	66.7	9	1.1	33.5	4	17	16.7			
**Meneghini 2010**	126	Thiazolidinediones	51.8	9.8	49	9.5	1.4	33.5	7.1	5.8				
**Meneghini 2010**	121	Basal insulin	52.9	10.6	48	9.4	1.2	33.7	6.9	6.5				
**Mita 2007**	34	Meglitinides	61.3	8.3	52.9	6.1	0.4	23.6	2.7	4.5				
**Mita 2007**	36	Standard treatments	61.8	6	52.8	6	0.4	23.6	2.7	4.8				
**Mita 2019**	22	Metformin	58.1	13.8	59	7.5	1.5	26.3	4.9	3.3		92		
**Mita 2019**	21	DPP-4 inhibitors	61.3	8.7	62	7.1	0.7	25.7	4.5	3.4		77		
**Mitrakou 1998**	60	Alpha-glucosidase inhibitors	57.4	5.6	48.3	9.9	0.5	24.4	3.1	8.5				
**Mitrakou 1998**	60	Standard treatments	57.4	5.8	61.7	9.9	0.4	24.5	3.4	7.9				
**Miyagawa 2015**	281	GLP-1 receptor agonists	57.2	9.6	81	8.2	0.8	25.6	3.6	6.8				

Study ID	N	Intervention	Age (mean)	Age (SD)	% of males	HbA1c (mean)	HbA1c (SD)	BMI (mean)	BMI (SD)	Duration of diabetes	% of CV disease	eGFR	ACR	ACR unit
**Miyagawa 2015**	141	GLP-1 receptor agonists	57.9	10.4	83	8.1	0.9	25.5	3.5	6.3				
**Miyagawa 2015**	70	Standard treatments	57.7	8.3	79	8.2	0.8	25.2	3.2	6.3				
**Miyazaki 2002**	11	Thiazolidinediones	55	6.6	45.4	9.1	1	30.4	3.3					
**Miyazaki 2002**	11	Thiazolidinediones	51	6.6	72.7	8.5	1.7	32.2	5.3					
**Miyazaki 2002**	12	Thiazolidinediones	57	13.9	66.6	8	1	30.8	4.5					
**Miyazaki 2002**	13	Thiazolidinediones	51	10.8	76.9	8.9	1.4	31.3	4.3					
**Miyazaki 2002**	11	Standard treatments	58	9.9	27.3	8.6	1.7	32.8	5.3					
**MK-0431J-842**	70	DPP-4 inhibitors	57	11.6	77.1	8	0.8	26.8	4.4	10		89.7		
**MK-0431J-842**	71	Standard treatments	54	9.5	63.4	8.1	0.8	27.1	4.6	8.3		93.9		
**Moeinzadeh-2021**	68	DPP-4 inhibitors	62.16	12.82	58.8	7.28	1.63			13.68		57.45		
**Moeinzadeh-2021**	68	Standard treatments	58.06	13.15	47.7	7.35	1.41			10.87		54.47		
**Mokta 2018**	111	Sulfonylureas	49.4	8.9	59	9		26.4	2.7					
**Mokta 2018**	106	DPP-4 inhibitors	50.9	9.3	60	9.1		26.8	3.2					
**Moretto 2008**	78	GLP-1 receptor agonists	54	10	63	7.9	1	31	5	2				
**Moretto 2008**	77	GLP-1 receptor agonists	55	10	52	7.8	1	32	5	2				
**Moretto 2008**	78	Standard treatments	53	9	55	7.8	0.9	32	5	1				
**Morino 2018**	22	SGLT2 inhibitors				8.1	0.9							
**Morino 2018**	24	Standard treatments				8.3	0.7							
**Moriwaki 2018**	14	DPP-4 inhibitors	69	9	79	6.6	0.5			3.1	100	71		
**Moriwaki 2018**	14	Alpha-glucosidase inhibitors	68	10	71	6.6	0.5			1.3	100	73		

Study ID	N	Intervention	Age (mean)	Age (SD)	% of males	HbA1c (mean)	HbA1c (SD)	BMI (mean)	BMI (SD)	Duration of diabetes	% of CV disease	eGFR	ACR	ACR unit
**Moses 2014**	129	DPP-4 inhibitors	57.2	9.6	62	8.4	0.9	29.4	5.3					
**Moses 2014**	128	Standard treatments	56.8	11.5	57.8	8.2	0.8	29.1	4.9					
**Moses 2016**	213	DPP-4 inhibitors	54.4	9.6	45.2	8.4	0.8	29.5	4.8	7.5				
**Moses 2016**	214	Standard treatments	55.4	10.2	46.2	8.4	0.9	28.8	5	8				
**Mu 2017**	144	Metformin	51.4	10.4	63.2	8.6	1	26.1	3.3	8.8				
**Mu 2017**	145	Metformin	52.1	9.6	62.8	8.7	1.1	25.8	3.3	9.8				
**Mu 2017**	147	DPP-4 inhibitors	50.8	10.5	51.7	8.7	0.9	26.2	3.9	9.2				
**Müller-Wieland 2018**	313	Sulfonylureas	58.6	8.4	66.5	8.3	0.8	33	5.1	6.7				
**Müller-Wieland 2018**	314	SGLT2 inhibitors	57.4	9.4	64.3	8.3	0.7	33.1	5.2	6.9				
**MUSCAT-HF**	86	SGLT2 inhibitors	71.7	7.7	66	7	0.7	25.4	4.3	6.2	59	60.6		
**MUSCAT-HF**	83	Alpha-glucosidase inhibitors	74.6	7.7	59	6.9	0.8	25.3	4.4	6.8	62	56.8		
**Nahra-2021**	110	GLP-1 receptor agonists	55.5	9.8	46	8.1	1	35.4	6.1	7.6				
**Nahra-2021**	112	Standard treatments	57.3	9.5	51	8.2	1.1	34.2	5.1	7.6				
**Nakamura 2001**	14	Thiazolidinediones				8.4	1.3					104		
**Nakamura 2001**	14	Standard treatments				8	1					106		
**Nakamura 2004**	15	Sulfonylureas	55	11.5	53.3	7.9	1.4			17				
**Nakamura 2004**	15	Thiazolidinediones	56.5	12	60	7.9	1.3			17.5				
**Nakamura 2004**	15	Alpha-glucosidase inhibitors	55	11	53.3	8.1	1.6			16.8				
**Nakamura 2006**	18	Sulfonylureas	53.5	12	55.6	7.8	1.3			16.5		109	136.5	mcg/min
**Nakamura 2006**	17	Thiazolidinediones	56	13	52.9	8	1.4			16		110	142.5	mcg/min

Study ID	N	Intervention	Age (mean)	Age (SD)	% of males	HbA1c (mean)	HbA1c (SD)	BMI (mean)	BMI (SD)	Duration of diabetes	% of CV disease	eGFR	ACR	ACR unit
**Nakamura 2006**	17	Alpha-glucosidase inhibitors	55	12.8	58.8	7.6	1.3			16.2		117	140.8	mcg/min
**Nakamura 2006**	16	Meglitinides	53.5	12.2	56.25	7.7	1.4			16.6		114	134.6	mcg/min
**Nar 2009**	19	Metformin	49.4	8.6	21.1	6.9	1.4	31	4	0				
**Nar 2009**	15	Standard treatments	44.5	5.9	33.3	6.1	1.1	33.7	6	0				
**Nathan 1988**	16	Sulfonylureas	50.3	5.9	56.3	10.5	2.41	30.2	5.7	5.9				
**Nathan 1988**	15	Basal insulin	53.5	7.1	53.3	10.3	1.5	28.6	5.1	3.7				
**Nauck 2007a**	253	GLP-1 receptor agonists	59	9	53	8.6	1	30.6	4	9.8				
**Nauck 2007a**	248	Basal-Bolus insulin	58	9	49	8.6	1.1	30.2	4.2	10				
**Nauck 2007b**	584	Sulfonylureas	56.5	9.8	61.3	7.6	0.9	31.3	5.2	6.2				
**Nauck 2007b**	588	DPP-4 inhibitors	56.8	9.3	57.1	7.7	0.9	31.5	5	6.5				
**Nauck 2009**	210	DPP-4 inhibitors	54	11	54.3	7.9	0.8	32	5	6				
**Nauck 2009**	213	DPP-4 inhibitors	55	11	47.4	7.9	0.7	32	5	6				
**Nauck 2009**	104	Standard treatments	56	11	48	8	0.9	32	6	6				
**Nauck 2011**	408	Sulfonylureas	59	10	54.9	7.7	0.9	31.2	5.1	7	19.5			
**Nauck 2011**	406	SGLT2 inhibitors	58	9	55.3	7.7	0.9	31.7	5.1	6	18			
**Nauck 2016**	138	Thiazolidinediones	56.5	11	52.2	8.1	0.9	33.9	5.5					
**Nauck 2016**	140	Thiazolidinediones	57	11.5	52.1	8	0.9	32.2	5.3					
**Nauck 2016**	131	Thiazolidinediones	56.3	10.4	55.7	8.3	0.9	32.3	5.6					
**Nauck 2016**	135	DPP-4 inhibitors	56	10.4	61.5	8	0.9	32.7	5.3					
**Negro 2005**	19	Thiazolidinediones	60.3	6.4	52.6	8.4	0.6	28.3	1.7	7.1				
**Negro 2005**	19	Standard treatments	59	8	63.2	8.1	0.5	28.7	1.9	6.6				
**Ning 2016**	146	DPP-4 inhibitors	57.8	9.1	41.8	8.6	0.9	26.2	3.2	11.2				

Study ID	N	Intervention	Age (mean)	Age (SD)	% of males	HbA1c (mean)	HbA1c (SD)	BMI (mean)	BMI (SD)	Duration of diabetes	% of CV disease	eGFR	ACR	ACR unit
**Ning 2016**	147	Standard treatments	58.4	9.6	44.9	8.7	0.9	26	3	11.4				
**Nino 2017**	150	GLP-1 receptor agonists	57.7	9.5	76	8.2	0.8	26.1	3.7	6.9				
**Nino 2017**	160	GLP-1 receptor agonists	59.6	9	78.1	8.1	0.8	25.3	4.2	7.2				
**Nino 2017**	77	Standard treatments	57.3	11.3	67.5	8.2	0.9	25.6	3.4	6.7				
**Nishimura 2016**	20	DPP-4 inhibitors	65.2	6.4	75	7.6	0.4	26.6	3.9	6.8	10	72		
**Nishimura 2016**	20	Standard treatments	59.4	8.5	70	7.6	0.4	25.2	3.3	8.5	20	71		
**Nishio 2006**	26	Thiazolidinediones	66.2	8.6	73.1	7.7	2.2	24.6	3.9		100			
**Nishio 2006**	28	Standard treatments	67.5	10.3	71.4	6.9	1.6	24.6	3.5		100			
**N-ISM**	57	SGLT2 inhibitors	60	9.1	61.1	7.54	0.75	26.3	3.1	10.2	0		279.3	mg/g
**N-ISM**	58	DPP-4 inhibitors	58.4	12.5	61.4	7.53	0.69	26.8	4.5	9.3	0		57.8	mg/g
**Nogueira 2014**	18	DPP-4 inhibitors	55.1	6.7	50	8	0.6	26.5	2.7	10.9				
**Nogueira 2014**	17	Basal insulin	58.4	6.9	35	8.1	0.7	27.5	2.5	10.9				
**Nowicki 2011**	85	DPP-4 inhibitors	66.8	8.3	37.6	8.5	1.2	31.2	6.1	15.1		32		
**Nowicki 2011**	85	Standard treatments	66.2	9.1	48.2	8.1	1.1	30.2	6.8	18.2		30		
**Ogasawara 2009**	27	Thiazolidinediones	68.6	7.9	72.7	7.2	0.7	23.8	3					
**Ogasawara 2009**	27	Standard treatments	66.8	8.1	58.3	6.8	0.9	24.1	2.1					
**Oh 2019**	95	Alpha-glucosidase inhibitors	51.9	8.8	62.5	8.1	1	24.5	2.4			91.3		
**Oh 2019**	92	Standard treatments	53.4	8.8	52.4	8.2	1	25.1	2.5			100.4		
**Ohira 2014**	35	Metformin	60.7	11	75	8.8	1.3	25.2	4.5					
**Ohira 2014**	35	DPP-4 inhibitors	60	12.4	60	9.1	1.7	25.2	3.1					
**Ohira 2014a**	30	Sulfonylureas	62.23	12.26	50	8.64	1.3	24.49	4.54					

Study ID	N	Intervention	Age (mean)	Age (SD)	% of males	HbA1c (mean)	HbA1c (SD)	BMI (mean)	BMI (SD)	Duration of diabetes	% of CV disease	eGFR	ACR	ACR unit
**Ohira 2014a**	30	Thiazolidinediones	63.7	7.94	63.3	8.54	1.1	23.61	4.06					
**Omarigliptin Protocol 020**	166	DPP-4 inhibitors	60	11	62.7	7.9	0.7	25.2	3.7	7.4				
**Omarigliptin Protocol 020**	165	DPP-4 inhibitors	60	9	69.7	8	0.8	25.4	4.2	7.4				
**Omarigliptin Protocol 020**	83	Standard treatments	61	9	68.7	8.1	0.7	24.3	3.3	8.6				
**Omariglitin Study 015**	389	DPP-4 inhibitors												
**Omariglitin Study 015**	196	Standard treatments												
**Onoue 2020**	12	DPP-4 inhibitors	64.8	12.4	40	7.1	0.5	26.9	6	7.7				
**Onoue 2020**	12	Standard treatments	62.9	14.7	33	7.3	0.5	25.6	3.5	7.4				
**Onuchin 2010_1**	46	Metformin	59.4	9.2	0	10.4	1.6	32.8	6.4	7				
**Onuchin 2010_1**	45	Basal insulin	61.1	8.5	0	11.03	1.9	31.1	7.6	9				
**Onuchin 2010_2**	47	Sulfonylureas	62.3	8.5	0	10.6	1.8	32.1	4.6	7				
**Onuchin 2010_2**	44	Basal insulin	61.4	8	0	10.8	1.6	32.3	5.7	8				
**Osman 2004**	8	Thiazolidinediones	53.5		14.3	10.3	3.2			7.8	100			
**Osman 2004**	8	Standard treatments	57.3		62.5	8.7	1.9			3.3	100			
**Ovalle 2004**	9	Thiazolidinediones	47	12		8.7		31.5	6.9	7.6				
**Ovalle 2004**	8	Basal-Bolus insulin	56	14.1		9		30.8	4.8	7.6				
**Owens 2011**	793	DPP-4 inhibitors	58.3	9.9	46.8	8.2	0.8	28.4	4.8					
**Owens 2011**	265	Standard treatments	57.6	9.7	48.3	8.1	0.8	28.2	4.5					
**Oyama 2008**	41	Alpha-glucosidase inhibitors	65	6	29.3	7.5	0.8	23.4	2.5	11.7				
**Oyama 2008**	43	Standard treatments	63	4	41.9	7.4	0.7	23.1	3.2	11.3				

Study ID	N	Intervention	Age (mean)	Age (SD)	% of males	HbA1c (mean)	HbA1c (SD)	BMI (mean)	BMI (SD)	Duration of diabetes	% of CV disease	eGFR	ACR	ACR unit
**Pagano 1995**	47	Sulfonylureas	59	7.5	51.1	7.8	0.7	26.7	2.7	7				
**Pagano 1995**	49	Alpha-glucosidase inhibitors	57	8.4	67.3	8.2	1.4	26.4	2.8	5				
**Pan 2008**	441	DPP-4 inhibitors	51.8	10.1	60.1	8.6	0.9	26.4	3.6	1.2				
**Pan 2008**	220	Alpha-glucosidase inhibitors	51.9	10.3	63.2	8.6	1	25.8	3.5	1.3				
**Pan 2012**	146	DPP-4 inhibitors	54.2	9.6	50	8.1	0.9	26	3.3	4.9				
**Pan 2012**	148	DPP-4 inhibitors	53.7	10	44.6	8.1	0.8	25	3.1	5				
**Pan 2012**	144	Standard treatments	54.5	9.7	45.8	8	0.8	25.5	3.1	5.2				
**Pan 2012a**	284	DPP-4 inhibitors	51.2	10	56.3	8.1	0.8	25.9	3.4	0.8		103		
**Pan 2012a**	284	Standard treatments	51.6	10.3	54.6	8.2	0.8	25.9	3.7	1.2		102		
**Park 2011**	34	Metformin	63.1	8.4	51.5	8.2	0.4	25.2	3	4.9				
**Park 2011**	34	Thiazolidinediones	62.3	8	52.9	8.3	0.3	25.5	3.8	5.1				
**Park 2014**	33	Metformin	55.8	10.5	60.6	8.4	0.9	25.1	3.6	11.3				
**Park 2014**	34	Sulfonylureas	57.3	9.2	58.8	8.4	1	25.6	2.7	13				
**Park 2017**	80	DPP-4 inhibitors	57.6	11	48.8	7.2	0.6	25.6	3.2	4.7		89		
**Park 2017**	80	Standard treatments	56.8	9.8	57.5	7.2	0.6	25.4	3.4	4.3		89		
**Parmar Vinendra 2019**	65	DPP-4 inhibitors	50.7	12.1	50	10.8	2.1	25.8	4	4				
**Parmar Vinendra 2019**	65	Standard treatments	49.8	14.3	56.7	9.9	1.7	25.4	4	3.5				
**Parthan 2018**	10	DPP-4 inhibitors	48.83333	12.04005	50	6.766667	0.602002	26.73333	6.450026	2.5				
**Parthan 2018**	10	Alpha-glucosidase inhibitors	49.66667	7.872121	77.8	7.066667	0.524808	25.43333	2.449104	2.5				

Study ID	N	Intervention	Age (mean)	Age (SD)	% of males	HbA1c (mean)	HbA1c (SD)	BMI (mean)	BMI (SD)	Duration of diabetes	% of CV disease	eGFR	ACR	ACR unit
**Parthan 2018**	10	Standard treatments	54	10.10396	71	6.966667	0.275563	26.9	6.154231	2				
**Pasquel-2021**	140	GLP-1 receptor agonists	56.1	9.5	65	8.3	0.9	33.5	5.3	9.5				
**Pasquel-2021**	147	Basal insulin	55.9	11.2	55	8.4	0.8	33.3	5.3	9.8				
**Patel 2013**	109	Alpha-glucosidase inhibitors	53.7	11	33	6.4	0.7	35.1	7.2					
**Patel 2013**	110	Standard treatments	53.7	11.7	34.6	6.3	0.6	35.2	7.1					
**Pavithra 2019**	43	DPP-4 inhibitors	47.6	4.7	65	8.6	5.2	25.3	8.2	7.1				
**Pavithra 2019**	37	GLP-1 receptor agonists	43.2	6.2	49	8.3	3.5	28.2	5.9	6.4				
**Pavo 2003**	100	Metformin	55.8	8.4	56	8.6		31.1	4.4	0.5				
**Pavo 2003**	106	Thiazolidinediones	54.2	9.1	43.8	8.6		31.3	4.2	0.5				
**Perez 2009**	210	Metformin	53.7	12	46.7	8.7	1	30.8	5.7					
**Perez 2009**	189	Thiazolidinediones	54	12	40.7	8.7	1	31.2	5.5					
**Perriello 2006**	137	Sulfonylureas	59	7	64.2	8.7	0.9	28.8	2.8	8.5				
**Perriello 2006**	146	Thiazolidinediones	58	8	66.4	8.8	0.9	29.2	3.1	9.8				
**PERISCOPE**	273	Sulfonylureas	59.7	9.1	65.9	7.4	1	32	5.2		100			
**PERISCOPE**	274	Thiazolidinediones	60	9.4	68.9	7.4	1	32.1	5.3		100			
**Petrica 2009**	22	Sulfonylureas	63.2	7.19	41.2	7.58	1.01	33.58	4.93	10.4				
**Petrica 2009**	22	Thiazolidinediones	63	8.07	41.2	7.72	1.2	33.55	4.84	10.53				
**Petrica 2011**	39	Sulfonylureas	58.82	7.78		7.49	1	32.1	5.95	10.17				
**Petrica 2011**	39	Thiazolidinediones	56.88	6.44		7.7	0.8	33.71	6.37	10				
**Philis-Tsimikas 2013**	229	DPP-4 inhibitors	54.9	11.4	54.5	9	1	30.8	5.2	7.7				
**Philis-Tsimikas 2013**	229	Basal insulin	56.4	10.2	62.7	8.8	1	30	5.1	7.8				

Study ID	N	Intervention	Age (mean)	Age (SD)	% of males	HbA1c (mean)	HbA1c (SD)	BMI (mean)	BMI (SD)	Duration of diabetes	% of CV disease	eGFR	ACR	ACR unit
**Phillips 2001**	187	Thiazolidinediones	56.5	9.7	65.2	9	1.5	29.9	4.3	5.9				
**Phillips 2001**	181	Thiazolidinediones	58.9	9.9	65.7	8.9	1.5	30	4.3	6.1				
**Phillips 2001**	186	Thiazolidinediones	56.8	9.4	59.1	8.9	1.5	30	4.2	5.5				
**Phillips 2001**	181	Thiazolidinediones	57.5	9.9	58.6	8.9	1.6	29.9	4.1	5.4				
**Phillips 2001**	173	Standard treatments	57.7	9.2	68.8	8.9	1.5	29.1	4.2	6.6				
**Phillips 2003**	40	Alpha-glucosidase inhibitors	58.4	10.7	65	8.1	0.9	30.8	3	5.3				
**Phillips 2003**	43	Standard treatments	62.4	8.02	77	7.8	0.8	30.1	2.9	6.1				
**Phrommintikul 2019**	24	DPP-4 inhibitors	63.9	7.65	50	8.3	1.1	24.9	3.2		75	70		
**Phrommintikul 2019**	25	SGLT2 inhibitors	62.6	8.27	56	8.2	1.4	25.6	3		60	74		
**PIOCOMB**	40	Metformin	64.2	7.3	55	7.4	0.5	31.8	5	12.3		114	11.4	
**PIOCOMB**	42	Thiazolidinediones	61.5	7.1	63	7.3	0.5	31.7	4.3	9.8		116	34.7	
**PIOfix**	150	Sulfonylureas	59	10	64.1	7.4	0.7	32.5	5.3	5.9				
**PIOfix**	155	Thiazolidinediones	59	10	65.8	7.1	0.6	32.6	4.9	6.2				
**Pioglitazone 001**	76	Thiazolidinediones												
**Pioglitazone 001**	85	Thiazolidinediones												
**Pioglitazone 001**	79	Thiazolidinediones												
**Pioglitazone 001**	80	Thiazolidinediones												
**Pioglitazone 001**	79	Standard treatments												
**PIONEER**	84	Sulfonylureas	63	7.4	62	7.4	0.9	31.8	4.3	6.9				
**PIONEER**	89	Thiazolidinediones	62.2	8.4	62	7.5	0.9	31.7	5	7.4				
**PIONEER 1**	175	GLP-1 receptor agonists	54	11	49.1	8	0.7	31.7	6.6	3.4		97		

Study ID	N	Intervention	Age (mean)	Age (SD)	% of males	HbA1c (mean)	HbA1c (SD)	BMI (mean)	BMI (SD)	Duration of diabetes	% of CV disease	eGFR	ACR	ACR unit
**PIONEER 1**	175	GLP-1 receptor agonists	56	11	53.1	8	0.6	31.6	6.4	3.6		95		
**PIONEER 1**	175	GLP-1 receptor agonists	55	11	50.9	7.9	0.7	31.8	6.3	3.8		99		
**PIONEER 1**	178	Standard treatments	55	11	50	7.9	0.7	32.2	6.9	3.4		100		
**PIONEER 2**	412	GLP-1 receptor agonists	58	10	51	8.1	0.9	32.8	5.9	7.7		95		
**PIONEER 2**	410	SGLT2 inhibitors	57	10	50.1	8.1	0.9	32.9	6.3	7.2		96		
**PIONEER 3**	467	DPP-4 inhibitors	58	10	51	8.3	0.9	32.5	6.2	8.8	17.3	96		
**PIONEER 3**	465	GLP-1 receptor agonists	57	10	53.1	8.3	0.9	32.3	6.3	8.7	16.6	95		
**PIONEER 3**	466	GLP-1 receptor agonists	58	10	52.7	8.4	1	32.6	6.4	8.3	16.3	96		
**PIONEER 3**	466	GLP-1 receptor agonists	58	10	54.5	8.3	1	32.6	6.7	8.4	15.7	96		
**PIONEER 4**	285	GLP-1 receptor agonists	56	10	52	8	0.7	32.5	5.9	7.8		96		
**PIONEER 4**	284	GLP-1 receptor agonists	56	10	52	8	0.7	33.4	6.7	7.3		96		
**PIONEER 4**	142	Standard treatments	57	10	52	7.9	0.7	32.9	6.1	7.8		95		
**PIONEER 5**	163	GLP-1 receptor agonists	71	8	51	8	0.7	32.2	5.4	14.1		47	19.2	
**PIONEER 5**	161	Standard treatments	70	8	45	7.9	0.7	32.6	5.5	13.9		48	14.1	

Study ID	N	Intervention	Age (mean)	Age (SD)	% of males	HbA1c (mean)	HbA1c (SD)	BMI (mean)	BMI (SD)	Duration of diabetes	% of CV disease	eGFR	ACR	ACR unit
**PIONEER 6**	1591	GLP-1 receptor agonists	66	7	68.1	8.2	1.6	32.3	6.6	14.7	35.3	74		
**PIONEER 6**	1592	Standard treatments	66	7	68.6	8.2	1.6	32.3	6.4	15.1	37	74		
**PIONEER 7**	251	DPP-4 inhibitors	56.9	9.7	57	8.3	0.6	31.5	6.5	8.6		97		
**PIONEER 7**	253	GLP-1 receptor agonists	57.9	10.1	56	8.3	0.6	31.5	6.1	9		95		
**PIONEER 8**	181	GLP-1 receptor agonists	61	10	47	8.2	0.7	30.8	6.3	14.1		91		
**PIONEER 8**	182	GLP-1 receptor agonists	60	10	56.6	8.2	0.7	31.1	7	16.2		92		
**PIONEER 8**	184	GLP-1 receptor agonists	61	9	55.4	8.2	0.7	31	6.8	15.1		92		
**PIONEER 8**	184	Standard treatments	61	10	57.1	8.2	0.7	31	6.7	14.8		91		
**PIONEER 9**	48	GLP-1 receptor agonists	58	9	73.5	8.1	0.8	26.5	4.6	7.4				
**PIONEER 9**	48	GLP-1 receptor agonists	60	10	73.5	8.3	1	26.3	3.5	7.4				
**PIONEER 9**	49	GLP-1 receptor agonists	61	9	83.3	8	0.9	24.7	4.1	7.9				
**PIONEER 9**	49	GLP-1 receptor agonists	59	10	81.3	8.3	0.8	26.9	4.8	6.7				
**PIONEER 9**	49	Standard treatments	59	9	81.6	8.3	1.1	25.1	3.9	8.4				
**PioRAGE**	32	Sulfonylureas	65.2	9.3	66.7	8	0.8	24.4	18.1	6	16.7			
**PioRAGE**	31	Thiazolidinediones	64.6	10.4	74.1	7.9	0.7	25.8	5.7	8	11.1			

Study ID	N	Intervention	Age (mean)	Age (SD)	% of males	HbA1c (mean)	HbA1c (SD)	BMI (mean)	BMI (SD)	Duration of diabetes	% of CV disease	eGFR	ACR	ACR unit
**PIRAMID**	39	Metformin	56.4	5.6	100	7	0.6	29.3	3.7	3				
**PIRAMID**	39	Thiazolidinediones	56.8	6.2	100	7.1	1.2	28.2	3.1	4.333333				
**Pistrosch 2012**	14	Thiazolidinediones	65.4	9.6	85.7	7	3	33.5	4.8	15		42	130	
**Pistrosch 2012**	14	Standard treatments	66.5	8.5	85.7	7.3	3	30.1	5.4	14.7		40	180	
**Pistrosch 2013**	36	Metformin	62	9.4	50	6.9	0.4	29.9	5.3	2.6				
**Pistrosch 2013**	39	Basal insulin	60	9.3	66.6	7.2	0.7	29.2	4.6	2.8				
**Pi-Sunyer 2007**	91	DPP-4 inhibitors	52	11.7	53.8	8.3	0.8	31.9	5	2.1				
**Pi-Sunyer 2007**	83	DPP-4 inhibitors	50.2	12.7	56.6	8.4	0.9	32.2	6	2.4				
**Pi-Sunyer 2007**	88	DPP-4 inhibitors	50.6	10.4	55.7	8.4	0.9	31.9	5.4	1.8				
**Pi-Sunyer 2007**	92	Standard treatments	52	12	54.3	8.5	0.8	32.7	6.4	2.5				
**Pop-Busui 2009**	13	Sulfonylureas				7.5	1.9				23			
**Pop-Busui 2009**	14	Thiazolidinediones				6.5	1				18			
**POPPS**	48	Thiazolidinediones	64	8.8	83	7.5	1.8				100			
**POPPS**	49	Standard treatments	62.4	9.8	86	7	1.5				100			
**Pratley 2014**	111	Metformin	52.6	11.3	56.6	8.4		30.5	5	4.1				
**Pratley 2014**	114	Metformin	54.6	10.2	53.6	8.5		30.2	4.8	3.8				
**Pratley 2014**	112	DPP-4 inhibitors	52.6	9.4	53.8	8.3		30.8	5.2	3.7				
**Pratley 2014**	113	DPP-4 inhibitors	53.7	9.7	71.2	8.4		30.4	5.2	4				
**Pratley 2014**	109	Standard treatments	53.1	9.6	60	8.5		31.2	5.3	4.3				
**PRESERVE-beta**	209	Sulfonylureas	53.5	11.6	48	8.3	1.1	33.5	5.6	2				
**PRESERVE-beta**	219	Meglitinides	52.6	11.6	51	8.4	1.2	33.3	6	1.5				
**PREVENT-J**	93	Sulfonylureas	58.8	8	49.5	6.9	0.6	24.7	4	4.3			37.8	
**PREVENT-J**	101	Thiazolidinediones	58.5	9	53.9	7	0.5	24.4	4	3.6			60.7	
**PREVENT-J**	84	Standard treatments	58.9	7.5	52.8	6.9	0.5	24.2	3.8	3.6			28.9	

Study ID	N	Intervention	Age (mean)	Age (SD)	% of males	HbA1c (mean)	HbA1c (SD)	BMI (mean)	BMI (SD)	Duration of diabetes	% of CV disease	eGFR	ACR	ACR unit
**PRIDE**	48	Thiazolidinediones	66	13.04896	83.3	6.766667	1.381655	24.7	3.147102		87.5			
**PRIDE**	46	Standard treatments	69.66667	9.245025	63.2	6.566667	0.924503	24.63333	2.157173		82.6			
**PRIME-V**	50	Metformin	55.7	12.2	56	8.1	0.9	28.8	5.3	5.3	0			
**PRIME-V**	48	SGLT2 inhibitors	56.6	11.9	64.6	8	0.7	27.6	4.2	5.4	0			
**PRISMA**	110	Thiazolidinediones	57	8.6	59.2	6.9	0.4	32.4	5.4	5.8			41.3	
**PRISMA**	103	Standard treatments	57.8	8.2	60.2	7	0.5	32.6	5.3	5.7			50	
**PROactive**	2605	Thiazolidinediones	61.9	7.6	67			30.7	4.7		100			
**PROactive**	2633	Standard treatments	61.6	7.8	66			31	4.8		100			
**PROLOGUE**	232	DPP-4 inhibitors	69.2	9.3	65.8	7	0.6	25.3	4.1		20.7	67		
**PROLOGUE**	231	Standard treatments	69.5	9.2	68.6	7	0.6	24.9	4		25	67		
**QUARTER**	597	Metformin	56	9.3	57.8	8.7	1	31.4	5.2	3.1				
**QUARTER**	597	Thiazolidinediones	57	9.4	52.6	8.7	1	31.2	4.9	3.4				
**Quatraro 1986**	15	Sulfonylureas	56	7.4		12	2.3	28.6	3.1	12.1				
**Quatraro 1986**	15	Standard treatments	57	7.4		11.8	1.5	28.9	2.7	11.8				
**Raghuveer 2020**	50	DPP-4 inhibitors												
**Raghuveer 2020**	50	Standard treatments												
**Rahman 2010**	11	Thiazolidinediones				7.5	1.7	26.6	1.4					
**Rahman 2010**	11	Standard treatments				8.3	2.3	27	3.2					
**Rahman 2011**	102	Metformin	51.9	14.1	49	10.5	2.5	26.6	6.2					
**Rahman 2011**	102	Sulfonylureas	52	15.4	47	10.6	2.9	26	5.2					
**Raskin 2001**	105	Thiazolidinediones	57.7	10.2	54.4	9	1.3	32.3	4.9	12.5				
**Raskin 2001**	107	Thiazolidinediones	57.1	10	56.6	9.1	1.3	32.1	4.8	12.7				
**Raskin 2001**	107	Standard treatments	55.6	10.3	55.8	8.9	1.1	32.7	4.5	11.7				
**Raskin 2004**	62	Thiazolidinediones	56.6	10.8	53.2				9	7.4				

Study ID	N	Intervention	Age (mean)	Age (SD)	% of males	HbA1c (mean)	HbA1c (SD)	BMI (mean)	BMI (SD)	Duration of diabetes	% of CV disease	eGFR	ACR	ACR unit
**Raskin 2004**	63	Meglitinides	58.5	10.1	61.9				9.3	7.2				
**Raskin 2009**	187	Thiazolidinediones	55.5	10.3	50.8	8.5	1.1	32.3	5.3	7.1				
**Raskin 2009**	187	Meglitinides	54.8	11.5	57.8	8.5	1.1	32.9	5.5	7.4				
**Raskin 2009**	187	Meglitinides	54.5	10.5	58.8	8.3	1	32.5	5.5	7.3				
**Raz 2008**	96	DPP-4 inhibitors	53.6	9.5	51	9.3	0.9	30.1	4.4	8.4				
**Raz 2008**	94	Standard treatments	56.1	9.5	41.5	9.1	0.8	30.4	5.3	7.3				
**RECORD**	2220	Thiazolidinediones			51.5						17.3			
**RECORD**	2227	Standard treatments			51.7						17.4			
**REFORM**	28	SGLT2 inhibitors	66.9	7	64.3			33	5.5		100	68		
**REFORM**	28	Standard treatments	67.4	6.8	67.9			32	5.2		100	76		
**REGO-F**	36	Sulfonylureas	62.4	11.3	63	7.4	0.6	25.1	2.7	10.3				
**REGO-F**	36	Meglitinides	63.5	9.4	61	7.6	0.8	25.5	3	8.1				
**RELEASE**	22	DPP-4 inhibitors	60.33333	11.09483	59	6.3	0.4	32.76667	8.241874	1.5		88		
**RELEASE**	22	Standard treatments	62.33333	10.30234	64	6.2	0.5	30.2	5.388917	1		81		
**RESET-IT Main**	55	Basal-Bolus insulin	56	9	54.6	6.5	0.7	33.7	7.2	1.6				
**RESET-IT Main**	53	Standard treatments	56	10	49.1	6.6	0.6	34.1	7.4	1.6				
**RESULT**	111	Standard treatments	68.2	6.3	71.8	7.7	1	30.5	4.9	6.8				
**RESULT**	116	Thiazolidinediones	68.7	6.1	74.8	7.7	1	30.2	4.5	6.8				
**REWIND**	4949	GLP-1 receptor agonists	66.2	6.5	53.4	7.3	1.1	32.3	5.7	10.5	31.5	75.3	1.8	
**REWIND**	4952	Standard treatments	66.2	6.5	53.9	7.4	1.1	32.3	5.8	10.6	31.4	74.7	1.9	
**Reynolds 2002**	8	Thiazolidinediones				8	0.8	36.4	5.1					
**Reynolds 2002**	10	Standard treatments				9.8	1.6	36.3	5.7					
**Reynolds 2007**	20	Thiazolidinediones				9.1	0.9							

Study ID	N	Intervention	Age (mean)	Age (SD)	% of males	HbA1c (mean)	HbA1c (SD)	BMI (mean)	BMI (SD)	Duration of diabetes	% of CV disease	eGFR	ACR	ACR unit
**Reynolds 2007**	20	Basal insulin				8.9	0.9							
**Ristic 2006**	129	Sulfonylureas	61.6	10.1	50.4	7.6	0.6	29.5	3.6	6.7				
**Ristic 2006**	133	Meglitinides	62	11	54.1	7.7	0.6	28.5	3.5	7.2				
**Robbins 2007**	158	Basal insulin	57.4	9.2	50.3	7.8	0.9	32.1	6.3	11.3				
**Robbins 2007**	159	Basal-Bolus insulin	58.1	8.9	49.4	7.8	1	32	6	12.5				
**Roberts 2005**	84	Sulfonylureas	56.5	9.8	61	8.2	0.7	34	5.2	7.9				
**Roberts 2005**	84	Standard treatments	56.4	10	62.3	8.2	0.8	32.8	5.1	8.7				
**Rodbard 2016**	108	SGLT2 inhibitors	57.4	9.3	61.7	8.5	0.9	32.3	5.8	9.8		91.4		
**Rodbard 2016**	108	Standard treatments	57.5	10.1	51.9	8.4	0.8	31.7	5.5	10.1		89.6		
**Rosenstock 1998**	74	Alpha-glucosidase inhibitors	57.2		61	8.5		32.4		7.2				
**Rosenstock 1998**	74	Standard treatments	55.9		49	8.2		32.3		7.8				
**Rosenstock 2006**	154	Metformin	51.5	10.4	56	8.8	1	32.5	7	2.9				
**Rosenstock 2006**	159	Thiazolidinediones	50.6	10.3	58	8.8	1	32.8	7.1	2.7				
**Rosenstock 2007**	267	Thiazolidinediones	54.2	11.6	57.6	8.7	1.1	32.9	6	2.7				
**Rosenstock 2007**	519	DPP-4 inhibitors	54.5	11.7	57.5	8.7	1.1	32.2	5.7	2.3				
**Rosenstock 2007a**	161	Thiazolidinediones	52.4	10.3	64	8.7	1	28.9	5.5	2.2				
**Rosenstock 2007a**	154	DPP-4 inhibitors	51.4	10.8	63.6	8.6	1	29.4	5.8	1.9				
**Rosenstock 2009**	129	DPP-4 inhibitors	55.9	10.2	34	9.3	1.1	32.3	5.6	13.4				
**Rosenstock 2009**	131	DPP-4 inhibitors	55.4	9.8	42	9.3	1.1	32.7	5.5	12.1				
**Rosenstock 2009**	130	Standard treatments	55	10.6	48	9.3	1.1	32.4	5.6	12.2				
**Rosenstock 2010**	163	Thiazolidinediones												
**Rosenstock 2010**	164	DPP-4 inhibitors												
**Rosenstock 2012**	140	SGLT2 inhibitors	53.8	10.4	42.1	8.4	1			5.8				

Study ID	N	Intervention	Age (mean)	Age (SD)	% of males	HbA1c (mean)	HbA1c (SD)	BMI (mean)	BMI (SD)	Duration of diabetes	% of CV disease	eGFR	ACR	ACR unit
**Rosenstock 2012**	141	SGLT2 inhibitors	53.2	10.9	55.3	8.4	1			5.6				
**Rosenstock 2012**	139	Standard treatments	53.5	11.4	51.1	8.3	1			5.1				
**Rosenstock 2013**	219	Sulfonylureas	69.8	4.1	43.8	7.5	0.6	30	4.5	5.9		72.9		
**Rosenstock 2013**	222	DPP-4 inhibitors	70.1	4.4	45.9	7.5	0.7	29.6	4.3	6.3		73.6		
**Rosenstock 2015**	176	DPP-4 inhibitors	54	10	50	8.9	1.2	31.5	5.3	7.4		92.5		
**Rosenstock 2015**	179	SGLT2 inhibitors	55	10	53	9	1.1	31.8	5.1	8.2		93.9		
**Rosenstock 2016**	237	Metformin	55.2	9.8	48.9	8.8	1.2	33	6	3.3		87		
**Rosenstock 2016**	238	SGLT2 inhibitors	55.8	9.6	52.5	8.8	1.2	32.6	5.8	3.3		85		
**Rosenstock 2016**	237	SGLT2 inhibitors	54	10.7	44.3	8.8	1.2	32.4	5.4	3.5		90		
**Rosenstock 2019**	296	DPP-4 inhibitors	32.4	5.5	54	8.3	1	32.4	5.5	7.8				
**Rosenstock 2019**	294	SGLT2 inhibitors	31.8	5.2	52.6	8.2	0.9	31.8	5.2	7.6				
**Rosenstock-2020**	402	GLP-1 receptor agonists	58	9.4	43.3	7.7	0.6	32.1	4.5	15.2				
**Rosenstock-2020**	412	Bolus insulin	58.1	9.5	48.1	7.7	0.6	32.5	4.7	14.7				
**Ross 2015**	159	Metformin	49	10.9	43.4	9.8	1.2	29.8	5.8		42.1			
**Ross 2015**	157	Standard treatments	48.6	11.2	49	9.9	1.1	29.6	5.4		45.9			
**SAIS1**	55	Sulfonylureas	52	18.27052	52.7	7.833333	0.837399	25.2	3.5					
**SAIS1**	48	DPP-4 inhibitors	59	34.39074	68.8	7.5	1.299206	25.7	3.9					
**Saleem 2011**	50	Sulfonylureas	45.8	8.8	20	10.2	1.6	30.4	5.6					
**Saleem 2011**	50	Meglitinides	46.6	10.5	32	9.9	1.6	27.1	3.5					
**Salman 2001**	30	Sulfonylureas	56.1	8.7	53.3	8.7	0.6	29.2	2.8	4.7				
**Salman 2001**	27	Alpha-glucosidase inhibitors	52.6	9.1	63	8.9	0.7	30.2	3.8	4.2				
**Saloranta 2002**	171	Meglitinides	59.6	9.9	65.5	6.6	0.7	29.1	3.7	3.7				

Study ID	N	Intervention	Age (mean)	Age (SD)	% of males	HbA1c (mean)	HbA1c (SD)	BMI (mean)	BMI (SD)	Duration of diabetes	% of CV disease	eGFR	ACR	ACR unit
**Saloranta 2002**	175	Meglitinides	61.1	9.3	61.1	6.5	0.6	28.9	3.7	3.6				
**Saloranta 2002**	166	Meglitinides	61	10.1	63.3	6.6	0.6	29	3.6	3.8				
**Saloranta 2002**	163	Standard treatments	59.1	10.6	60.1	6.5	0.6	28.8	3.5	3.2				
**Samson 2011**	11	GLP-1 receptor agonists												
**Samson 2011**	10	Standard treatments												
**Sato 2018**	20	SGLT2 inhibitors	68	4	0.8	7.2	0.6	26.6	4.6				21	mg/g
**Sato 2018**	20	Standard treatments	66	6	0.7	7.4	1.1	25	3.1				38	mg/g
**Sato 2019**	22	Sulfonylureas	62.1	9		7.8	0.5	24.6	3.5	11.9			43.7	
**Sato 2019**	19	DPP-4 inhibitors	63.4	5.9		8	0.6	24	2.4	12.9			33.8	
**SAVOR-TIMI 53**	8280	DPP-4 inhibitors	65.1	8.5	66.6	8	1.4	31.1	5.5		78.4	73	1.8	mg/mmol
**SAVOR-TIMI 53**	8212	Standard treatments	65	8.6	67.3	8	1.4	31.2	5.7		78.7	73	1.9	mg/mmol
**Saxagliptin 014**	181	DPP-4 inhibitors	54.2	10.1	52.5	8	1	31.1	4.8	6.4				
**Saxagliptin 014**	191	DPP-4 inhibitors	54.7	9.6	53.9	8.1	0.8	31.2	4.7	6.4				
**Saxagliptin 014**	192	DPP-4 inhibitors	54.7	10.1	42.2	8.1	1	31.7	5.2	6.7				
**Saxagliptin 014**	179	Standard treatments	54.8	10.2	53.6	8.1	0.9	31.6	4.8	6.7				
**SCALE Diabetes**	432	GLP-1 receptor agonists	55	10.8	52	7.9	0.8	37.1	6.5	7.5	16.4			
**SCALE Diabetes**	211	GLP-1 receptor agonists	54.9	10.7	51.2	8	0.8	37	6.9	7.4	14.8			
**SCALE Diabetes**	212	Standard treatments	54.7	9.8	45.8	7.9	0.8	37.4	7.1	6.7	12.3			
**SCALE insulin**	198	GLP-1 receptor agonists	55.9	11.3	45.5	7.9	1.1	35.9	6.5	11.4				
**SCALE insulin**	198	Standard treatments	57.6	10.4	50	8	1	35.3	5.8	12.8				

Study ID	N	Intervention	Age (mean)	Age (SD)	% of males	HbA1c (mean)	HbA1c (SD)	BMI (mean)	BMI (SD)	Duration of diabetes	% of CV disease	eGFR	ACR	ACR unit
**Scherbaum 2002**	72	Thiazolidinediones	59.6		41	9.1		29.3		4.6				
**Scherbaum 2002**	83	Thiazolidinediones	58		62.9	9.3		29.9		5.4				
**Scherbaum 2002**	78	Standard treatments	59.1		56	8.8		29.2		5.6				
**Scherbaum 2008**	156	DPP-4 inhibitors	63.3	10.2	59.6	6.7	0.4	30.4	4.9	2.5				
**Scherbaum 2008**	150	Standard treatments	62.8	11	59.3	6.8	0.4	30	4.9	2.7				
**Schiavon-2021**	67	GLP-1 receptor agonists	56.1	11.4	46.3	8.11	0.86	34.23	5.51					
**Schiavon-2021**	33	Standard treatments	56.2	9.3	54.5	8.04	0.86	32.07	4.41					
**Schweizer 2007**	254	Metformin	53.6	10.2	57.5	8.7	1.1	32.5	5.7					
**Schweizer 2007**	526	DPP-4 inhibitors	52.8	11.7	52.9	8.7	1.1	32.4	5.7					
**Schweizer 2009**	166	Metformin	70.2	5.1	53	7.7	0.6	29.4	4.6	3				
**Schweizer 2009**	169	DPP-4 inhibitors	71.6	5.2	44.4	7.8	0.6	29.8	4.4	2.9				
**SCORED**	5292	SGLT2 inhibitors	68.7	8.2	55.7	8.4	1.3	32.1	6		51.6	44.2	192.7	mg/g
**SCORED**	5292	Standard treatments	68.7	8.2	54.5	8.4	1.3	31.9	6		51.1	44.4	189.7	mg/g
**SEESAW_1**	17	SGLT2 inhibitors	62.0	8.9	47.1	6.8	0.49	32.0	4.3	6.5		89.3		
**SEESAW_1**	17	Standard treatments	62.7	10.5	76.5	7.0	0.65	33.0	5.0	7.2		87.7		
**SEESAW_2**	17	SGLT2 inhibitors	63.0	11.3	58.8	6.8	0.40	31.4	4.4	7.7		89.0		
**SEESAW_2**	17	Standard treatments	64.0	7.3	76.5	6.9	0.73	32.4	6.8	5.2		88.3		
**Segal 1997**	37	Sulfonylureas	56		62.2	8		29.2						
**Segal 1997**	40	Alpha-glucosidase inhibitors	61		55	8		28.6						
**Segal 1997**	42	Standard treatments	59		57	8.3		29.1						
**Segal 2005**	69	Alpha-glucosidase inhibitors	53.7	8	53.6	11	2.3	28.1	4.2	10				

Study ID	N	Intervention	Age (mean)	Age (SD)	% of males	HbA1c (mean)	HbA1c (SD)	BMI (mean)	BMI (SD)	Duration of diabetes	% of CV disease	eGFR	ACR	ACR unit
**Segal 2005**	70	Standard treatments	55.7	8.1	53.2	11.4	2.9	28	4.1	11				
**Seino 2011**	88	GLP-1 receptor agonists	66.8	13.7	67	8.6	0.8	24.4	3.8	11.6				
**Seino 2011**	88	GLP-1 receptor agonists	64.5	12	60.2	9	0.9	25.3	3.6	9.3				
**Seino 2011**	88	Standard treatments	59.1	10.3	64.8	8.9	1	24.9	4	10.1				
**Seino 2014**	79	SGLT2 inhibitors	58.9	10.1	75.9	8.1	0.9	26	4.9	6.5				
**Seino 2014**	79	Standard treatments	59.6	9.3	70.9	8.2	0.8	25.3	4.2	6.1				
**Seino 2015**	150	SGLT2 inhibitors	61.2	8.4	74.7	8.1	0.9	24.8	3.6	7.4		82		
**Seino 2015**	71	Standard treatments	59.9	10.5	67.6	8	0.7	24.7	3.3	7.9		84		
**Seino 2016**	127	GLP-1 receptor agonists	61.3	11	54.3	8.8	0.9	26.2	4.9	14.3				
**Seino 2016**	130	Standard treatments	59.8	11.3	57.7	8.8	0.9	25.2	4	14.7				
**Seino 2018**	103	DPP-4 inhibitors	57.9	10.1	78.6	8.2	0.9	25.1	3.6	8.1				
**Seino 2018**	102	GLP-1 receptor agonists	58.1	11.6	73.5	8	0.9	26.1	5.2	7.8				
**Seino 2018**	103	GLP-1 receptor agonists	58.8	10.4	76.7	8.2	1	25.1	3.8	8				
**Shah 2011**	100	Sulfonylureas	45.2	8.2	31			30.2	5.5					
**Shah 2011**	100	Meglitinides	46	10.1	20			27.2	3.2					
**Shankar 2017**	234	DPP-4 inhibitors	58.6	8.4	55.6	8.7	0.9	25.9	3	11				
**Shankar 2017**	233	Standard treatments	56.7	9.1	49.8	8.8	0.9	26.1	2.9	11.3				
**Shankar 2017a**	201	DPP-4 inhibitors	57.5	8.1	50.2	8.1	0.9	32.4	5.8	8.2				
**Shankar 2017a**	201	Standard treatments	56.8	9.1	50.7	8	0.9	32.6	6.1	7.4				

Study ID	N	Intervention	Age (mean)	Age (SD)	% of males	HbA1c (mean)	HbA1c (SD)	BMI (mean)	BMI (SD)	Duration of diabetes	% of CV disease	eGFR	ACR	ACR unit
**Shestakova 2018**	110	SGLT2 inhibitors	58.9	9.3	43.6	8.4	0.9	32.8	4.8	6.7				
**Shestakova 2018**	55	Standard treatments	58	9.5	40	8.5	1	32	4.2	6.6				
**Shibuya 2018**	16	Metformin	59.66667	10.56668	50	7.333333	0.650257	28.03333	5.771034	0.8				
**Shibuya 2018**	16	SGLT2 inhibitors	53.33333	12.19232	62.5	7.633333	0.568975	27.6	2.032054	0.4				
**Shuai-2021**	134	GLP-1 receptor agonists	50.5	10.4	66.9	8.5	0.9	27	3.7	1.8				
**Shuai-2021**	136	GLP-1 receptor agonists	52.4	11.5	55.2	8.5	0.9	26.4	3.3	2.3				
**Shuai-2021**	136	Standard treatments	51.5	10.9	72.7	8.6	1	26.3	3.4	2.6				
**SIMPLE**	59	GLP-1 receptor agonists	46.7	9	32.2	12.1	1.4	36.1	9.5	10				
**SIMPLE**	61	Bolus insulin	48.1	10	26.2	12	1.5	38.3	11	10				
**Sit2Mix**	195	DPP-4 inhibitors	56.3	10.2	48.2	8.4	0.8	29.4	4.5					
**Sit2Mix**	194	Standard treatments	54.8	9.5	57.2	8.4	0.8	29.3	4.3					
**Sitagliptin 019**	175	DPP-4 inhibitors	55.6	10.4	53.1	8.1	0.8	32	5.2	6.1				
**Sitagliptin 019**	178	Standard treatments	56.9	11.1	57.9	8	0.8	31	5	6.1				
**Sitagliptin 020**	464	DPP-4 inhibitors	54.4	10.4	55.8	8	0.8	30.9	5.3	6				
**Sitagliptin 020**	237	Standard treatments	54.7	9.7	59.5	8	0.8	31.5	4.9	6.6				
**Sitagliptin 021**	250	DPP-4 inhibitors												
**Sitagliptin 021**	238	DPP-4 inhibitors												
**Sitagliptin 021**	253	Standard treatments												
**Sitagliptin 024**	584	Sulfonylureas	56.5	9.8	61.3	7.6	0.9	31.3	5.2	6.2				
**Sitagliptin 024**	588	DPP-4 inhibitors	56.8	9.3	57.1	7.7	0.9	31.5	5	6.5				
**Sitagliptin 035**	222	DPP-4 inhibitors	55.6	9.6	52.7	8.3	0.8	31.2	6.3	8.3				

Study ID	N	Intervention	Age (mean)	Age (SD)	% of males	HbA1c (mean)	HbA1c (SD)	BMI (mean)	BMI (SD)	Duration of diabetes	% of CV disease	eGFR	ACR	ACR unit
**Sitagliptin 035**	219	Standard treatments	56.5	9.6	53.4	8.3	0.7	30.7	6.3	9.3				
**Sitagliptin 036**	179	DPP-4 inhibitors	53.3	10.2	52	8.9	1	31.2	5.9	4.4				
**Sitagliptin 036**	176	Standard treatments	53.6	10	52.8	8.7	1	32.5	6.7	4.6				
**Sitagliptin 049**	522	Metformin	55.7	10.3	44	7.2	0.7	30.9	4.9	2.1				
**Sitagliptin 049**	528	DPP-4 inhibitors	56.3	10.7	48	7.2	0.7	30.7	4.7	2.6				
**Sitagliptin 051**	322	DPP-4 inhibitors	58.3	9.1	49	8.7	0.9	31	5	13				
**Sitagliptin 051**	319	Standard treatments	57.2	9.3	53	8.6	0.9	31	5	12				
**SLIM**	97	DPP-4 inhibitors	64	10	54	7.5	0.8						133	mg/g
**SLIM**	92	Standard treatments	64	12	56	7.5	0.7						55	mg/g
**SMART**	241	DPP-4 inhibitors	54.7	10.5	61.8	8.2	0.9	26.4	3.5	5.1				
**SMART**	244	Alpha-glucosidase inhibitors	56.5	10.8	56.8	8.2	0.8	26.3	3.5	5.3				
**Smith 2005**	24	Thiazolidinediones	56.2	9.7	47.6	6.88	1.4	32.1	5.6					
**Smith 2005**	24	Standard treatments	53.1	9.3	71.4	6.46	0.7	31.9	5					
**Softeland 2017**	111	SGLT2 inhibitors	55.4	9.9	64.5	8	0.8	29.9	5.3			93		
**Softeland 2017**	112	SGLT2 inhibitors	54.3	9.6	60.6	8	0.8	31.2	5.9			91		
**Softeland 2017**	110	Standard treatments	55.9	9.7	55.6	8	0.9	29.6	5.7			93		
**Sohn 2008**	8	Metformin	52	10.7	50	9	1.3	22.8	2	1.1				
**Sohn 2008**	11	Thiazolidinediones	48.1	9.9	63.6	8.8	1	24.6	3	1.8				
**SOLOIST-WHF**	608	SGLT2 inhibitors	69.3	9.66	67.4	7.3	1.4	30.3	5.9	10.7		50		
**SOLOIST-WHF**	614	Standard treatments	70	8.92	65.1	7.3	1.3	31	5.4	10.8		52		
**Sone 2019**	90	SGLT2 inhibitors	58.6	9.5	67.8	8.7	0.7	26.8	3.3	14.6		84		
**Sone 2019**	86	SGLT2 inhibitors	58.3	10	73.3	8.8	0.7	27	3	14.4		85		
**Sone 2019**	90	Standard treatments	59.1	10.7	76.7	8.7	0.7	26.9	3.4	12.4		83		

Study ID	N	Intervention	Age (mean)	Age (SD)	% of males	HbA1c (mean)	HbA1c (SD)	BMI (mean)	BMI (SD)	Duration of diabetes	% of CV disease	eGFR	ACR	ACR unit
**South Danish Diabetes Study**	45	Metformin	56.1	8.2	62.2	8.5	1.2	33.7	6.1	8.7				
**South Danish Diabetes Study**	47	Thiazolidinediones	56.1	8.3	57.4	8.3	1	32.7	4.7	9.4				
**South Danish Diabetes Study**	48	Standard treatments	57.1	8.5	47.9	8.5	1.2	33.7	5	9.1				
**SPEAD-A**	172	DPP-4 inhibitors	64.4	9.8	63	7.3	0.8	24.6	4.3	9				
**SPEAD-A**	169	Standard treatments	64.8	9.1	61	7.2	0.8	24.9	3.7	8.2				
**SPECIFY**	194	DPP-4 inhibitors	54.2	9.4	63.6	8	0.7	25.3	2.3	5.2		102.3	13.5	mg/g
**SPECIFY**	194	Sulfonylureas	52.8	9.5	58.6	8	0.7	25.6	2.9	4.9		101.8	12	mg/g
**SPIKE**	142	DPP-4 inhibitors	63.8	9.7	61	8.1	1.1	25	4.3	17.2	0	78	20.3	mg/g
**SPIKE**	140	Standard treatments	63.6	10	60	8	1	25	3.8	17.3	0	80	19	mg/g
**SPOTLIGHT**	98	SGLT2 inhibitors	56.2	10.2	77.3	8.2	0.7	27.1	3.9	6.3				
**SPOTLIGHT**	54	Standard treatments	56.1	11.9	68.5	8.4	0.6	27.1	4.3	7.7				
**SPREAD-DIMCAD**	156	Metformin	62.8	8.5	78.2	7.6	1.7	25.2	3	5.6	100			
**SPREAD-DIMCAD**	148	Sulfonylureas	63.8	9.4	77	7.6	1.7	25.1	2.9	5.6	100			
**Sridhar 2013**	25	Thiazolidinediones	47.9	5.8	100	6.8	0.4	25.3	2.7	2.2				
**Sridhar 2013**	25	Standard treatments	44	7.2	100	6.8	0.4	25.1	3.2	2.9				
**St. John Sutton 2002**	99	Sulfonylureas	56.1	8.9	72	9.5	1.6			6.2				
**St. John Sutton 2002**	104	Thiazolidinediones	55.1	9	72.1	9.1	1.7			5.3				
**Standl 1999**	24	Alpha-glucosidase inhibitors	59.3	8.5	58.3	10.9	1	25.2	2.2	11.5				
**Standl 1999**	24	Standard treatments	62.9	9.4	41.7	11	1.2	24.1	2	12.2				

Study ID	N	Intervention	Age (mean)	Age (SD)	% of males	HbA1c (mean)	HbA1c (SD)	BMI (mean)	BMI (SD)	Duration of diabetes	% of CV disease	eGFR	ACR	ACR unit
**Standl 2001**	65	Alpha-glucosidase inhibitors	62	8	50.8	8.8	0.9	27.7	3.7	8				
**Standl 2001**	68	Standard treatments	61	8	54.4	8.8	0.7	27.9	3.5	9				
**START**	210	Metformin	50.1	11	63.8	9.5	1	26.5	3.6	0.7				
**START**	214	DPP-4 inhibitors	49.5	10.9	70.9	9.4	1	26.5	3.2	0.7				
**START-J**	152	Sulfonylureas	70.8	5.5	61.2	7.5	0.7	24.2	3.6	3.1				
**START-J**	153	DPP-4 inhibitors	70.2	5.4	51.7	7.5	0.7	24.1	3.3	4.2				
**STEP 2**	403	GLP-1 receptor agonists	56	10	49.6	8.1	0.8	35.3	5.9	7.7	9.9	93.43		
**STEP 2**	404	GLP-1 receptor agonists	55	11	44.8	8.1	0.8	35.9	6.4	8.2	6.4	94.25		
**STEP 2**	403	Standard treatments	55	11	52.9	8.1	0.8	35.9	6.5	8.2	8.2	92.32		
**Stewart 2006**	254	Thiazolidinediones	58.9	8.4	55	7.2	0.6	30.9	5.4	3.7				
**Stewart 2006**	272	Standard treatments	59	7.9	56	7.2	0.6	30.6	5.5	3.7				
**Stocker 2007**	47	Metformin	65	10	53.2	8.5	1.8	29.7	5.1					
**Stocker 2007**	45	Thiazolidinediones	64	11	71.1	8.5	1.9	29.4	4.5					
**STOP-OB**	31	Sulfonylureas	57.6	9.3	61.3	7.5	0.4	25.4	3.8	7.7		89		
**STOP-OB**	33	SGLT2 inhibitors	57.3	11.4	54.5	7.4	0.5	25.3	3.9	11.3		92		
**Strojek 2009**	241	Basal insulin	56.1	10	41.2	8.5	1.1	29.2	4.5	9.5				
**Strojek 2009**	239	Basal-Bolus insulin	55.9	9.7	46.8	8.5	1	29	4.6	9.1				
**Strojek 2011**	151	SGLT2 inhibitors	58.9	8.3	43.7	8.1	0.8	29.8	5.6	7.2	30.5			
**Strojek 2011**	145	SGLT2 inhibitors	60.2	9.7	50	8.1	0.8	29.8	5.2	7.4	38.7			
**Strojek 2011**	154	SGLT2 inhibitors	59.9	10.1	50	8.1	0.8	30	5.2	7.7	36.4			
**Strojek 2011**	146	Standard treatments	60.3	10.2	49	8.2	0.7	29.7	4.6	7.4	37.9			

Study ID	N	Intervention	Age (mean)	Age (SD)	% of males	HbA1c (mean)	HbA1c (SD)	BMI (mean)	BMI (SD)	Duration of diabetes	% of CV disease	eGFR	ACR	ACR unit
**Strøm Halden 2019**	22	SGLT2 inhibitors	57.25	10.73502	77.3	7.125	0.44511	30.4	3.822713			66		
**Strøm Halden 2019**	22	Standard treatments	53.5	14.1388	77.3	6.725	0.288013	30.8	6.126814			59		
**Su 2014**	300	DPP-4 inhibitors	47.6	14.4		8.8	1.7							
**Su 2014**	300	Standard treatments	48.7	11.5		8.4	1.4							
**SUCCESS**	60	DPP-4 inhibitors	57.6	12.9	65.5	7.6	0.7	25.9	4.3	6.7				
**SUCCESS**	59	Alpha-glucosidase inhibitors	59.3	11.3	62.1	7.6	0.7	26.3	4.6	6.8				
**SUGAR-DM-HF**	52	SGLT2 inhibitors	68.2	11.7	65.4	7.5	1.6	30.9	5.9	8.5	75	69.5		
**SUGAR-DM-HF**	53	Standard treatments	69.2	10.6	81.1	7	1.4	30.4	5.1	9.6	86.8	65.1		
**Sun 2006**	30	Sulfonylureas				8.9	3	26.09	1.57				64.05	
**Sun 2006**	30	Thiazolidinediones				8.8	3.1	26.07	1.06				64.01	
**Sun 2016**	54	Metformin	52	6	55.6	8.5	0.9	27	1.9					
**Sun 2016**	54	Alpha-glucosidase inhibitors	53	8	50	8.2	0.8	27.1	2					
**SUPER**	232	DPP-4 inhibitors	59.3	7.9	47	8.5	0.7	26.1	3.3	13.4				
**SUPER**	230	Standard treatments	58.9	8.2	43.5	8.5	0.7	26.3	3.1	13.3				
**SURPASS J-mono**	159	Dual GIP/GLP-1 receptor agonists	56.8	10.1	71	8.2	0.9	28.6	5.4	4.7		77.3		
**SURPASS J-mono**	158	Dual GIP/GLP-1 receptor agonists	56.2	10.3	75	8.2	0.9	28	4.1	5.2		79.3		
**SURPASS J-mono**	160	Dual GIP/GLP-1 receptor agonists	56	10.7	83	8.2	0.9	28.1	4.4	5.2		79.0		
**SURPASS J-mono**	477	Dual GIP/GLP-1 receptor agonists												

Study ID	N	Intervention	Age (mean)	Age (SD)	% of males	HbA1c (mean)	HbA1c (SD)	BMI (mean)	BMI (SD)	Duration of diabetes	% of CV disease	eGFR	ACR	ACR unit
**SURPASS J-mono**	159	GLP-1 receptor agonists	57.5	10.2	74	8.2	0.9	27.8	3.7	5.1		78.7		
**SURPASS-1**	121	Dual GIP/GLP-1 receptor agonists	54.1	11.9	46	7.97	0.84	32.2	7	4.6		94.7		
**SURPASS-1**	121	Dual GIP/GLP-1 receptor agonists	55.8	10.4	60	7.9	0.78	32.2	7.6	4.9		92.1		
**SURPASS-1**	121	Dual GIP/GLP-1 receptor agonists	52.9	12.3	52	7.85	1.02	31.5	5.5	4.8		96.2		
**SURPASS-1**	115	Standard treatments	53.6	12.8	49	8.05	0.8	31.7	6.1	4.5		93.4		
**SURPASS-2**	471	Dual GIP/GLP-1 receptor agonists	56.3	10	43.6	8.32	1.08	33.8	6.85	9.1		96.6	14	g/kg
**SURPASS-2**	469	Dual GIP/GLP-1 receptor agonists	57.2	10.5	50.7	8.3	1.02	34.3	6.6	8.4		95.5	13.1	g/kg
**SURPASS-2**	470	Dual GIP/GLP-1 receptor agonists	55.9	10.4	45.5	8.26	1	34.5	7.11	8.7		96.3	13.5	g/kg
**SURPASS-2**	469	GLP-1 receptor agonists	56.9	10.8	48	8.25	1.01	34.2	7.15	8.3		95.6	12.4	g/kg
**SURPASS-3**	359	Dual GIP/GLP-1 receptor agonists	57.2	10.1	56	8.17	0.89	33.6	5.9	8.5		95.1		
**SURPASS-3**	361	Dual GIP/GLP-1 receptor agonists	57.4	9.7	54	8.18	0.89	33.4	6.2	8.4		93.7		
**SURPASS-3**	359	Dual GIP/GLP-1 receptor agonists	57.5	10.2	54	8.21	0.94	33.7	6.1	8.5		93.1		
**SURPASS-3**	365	Basal insulin	57.5	10.1	59	8.12	0.94	33.4	6.1	8.1		94.6		

Study ID	N	Intervention	Age (mean)	Age (SD)	% of males	HbA1c (mean)	HbA1c (SD)	BMI (mean)	BMI (SD)	Duration of diabetes	% of CV disease	eGFR	ACR	ACR unit
**SURPASS-4**	329	Dual GIP/GLP-1 receptor agonists	62.9	8.6	60	8.52	0.84	32.6	6.06	10.4	84	80.3		
**SURPASS-4**	330	Dual GIP/GLP-1 receptor agonists	63.7	8.7	64	8.59	0.91	32.8	5.51	11.1	90	81.4		
**SURPASS-4**	338	Dual GIP/GLP-1 receptor agonists	63.7	8.6	60	8.52	0.98	32.5	5.02	10.5	87	81.6		
**SURPASS-4**	1005	Basal insulin	63.8	8.5	64	8.5	0.85	32.5	5.55	11.2	87	81.5		
**SURPASS-5**	116	Dual GIP/GLP-1 receptor agonists	62	10	53	8.3	0.88	33.6	5.9	14.1		86.1		
**SURPASS-5**	119	Dual GIP/GLP-1 receptor agonists	60	10	61	8.36	0.83	33.4	6.2	12.6		87.1		
**SURPASS-5**	120	Dual GIP/GLP-1 receptor agonists	61	10	54	8.23	0.86	33.4	5.9	13.7		84.1		
**SURPASS-5**	120	Standard treatments	60	10	55	8.37	0.84	33.2	6.3	12.9		84.7		
**SUSTAIN 1**	130	GLP-1 receptor agonists	52.7	11.9	62	8.1	0.8	33.9	8.4	3.6		101		
**SUSTAIN 1**	128	GLP-1 receptor agonists	54.6	11.1	47	8.1	0.9	32.5	7.6	4.8		96		
**SUSTAIN 1**	129	Standard treatments	53.9	11	54	8	0.9	32.4	6.9	4.1		100		
**SUSTAIN 2**	407	DPP-4 inhibitors	54.6	10.4	51	8.2	0.9	32.5	5.8	6.6		98		
**SUSTAIN 2**	409	GLP-1 receptor agonists	56	9.4	50	8	0.9	32.5	6.6	6.7		97		
**SUSTAIN 2**	409	GLP-1 receptor agonists	54.8	10.2	51	8	0.9	32.4	6.2	6.4		97		

Study ID	N	Intervention	Age (mean)	Age (SD)	% of males	HbA1c (mean)	HbA1c (SD)	BMI (mean)	BMI (SD)	Duration of diabetes	% of CV disease	eGFR	ACR	ACR unit
**SUSTAIN 4**	360	GLP-1 receptor agonists	56.2	10.6	51	8.3	0.9	33	6.5	9.3		98		
**SUSTAIN 4**	362	GLP-1 receptor agonists	56.7	10.4	54	8.1	0.8	33.1	6.5	7.8		98		
**SUSTAIN 4**	360	Basal insulin	56.2	10.6	54	8.1	0.9	33	6.5	8.6		100		
**SUSTAIN 5**	131	GLP-1 receptor agonists	57.5	9.064066	58.8	8.575	0.752125	33.775	6.190564	13.7				
**SUSTAIN 5**	132	GLP-1 receptor agonists	57.55	10.78888	56.1	8.525	0.635773	34.525	5.837554	12.9				
**SUSTAIN 5**	133	Standard treatments	55.65	12.89526	53.4	8.675	0.827606	33.35	5.350572	13.3				
**SUSTAIN 6**	1648	GLP-1 receptor agonists									60			
**SUSTAIN 6**	1649	Standard treatments									61			
**SUSTAIN 8**	394	SGLT2 inhibitors	57.5	10.7	51	8.2	1							
**SUSTAIN 8**	394	GLP-1 receptor agonists	55.7	11.1	56.6	8.3	1							
**SUSTAIN 9**	151	GLP-1 receptor agonists	57.5	8.9	58.9	8	0.8	31.1	6.2	9.8	5.3	95		
**SUSTAIN 9**	151	Standard treatments									5.3	96		
**SUSTAIN 11**	874	GLP-1 receptor agonists	60.8	9.4	50.9	8.6	0.7	31.4	5.5	13.4	5.8			
**SUSTAIN 11**	874	Bolus insulin	61.5	9.5	51.4	8.5	0.7	31.7	5.5	13.4	13.4			
**SUSTAIN China**	288	GLP-1 receptor agonists	53	11.4	55.6	8.1	0.9	28.2	5	6.3		118.3		

Study ID	N	Intervention	Age (mean)	Age (SD)	% of males	HbA1c (mean)	HbA1c (SD)	BMI (mean)	BMI (SD)	Duration of diabetes	% of CV disease	eGFR	ACR	ACR unit
**SUSTAIN China**	290	GLP-1 receptor agonists	53	10.6	53.1	8.1	0.9	27.9	5	6.7		138.8		
**SUSTAIN China**	290	DPP-4 inhibitors	53.1	10.4	63.8	8.1	0.9	27.3	4.7	6.1		125		
**Suzuki 2014**	16	DPP-4 inhibitors	56.1	15.3	56	9.1	1.6	26.3	7.2	1.9		74	14	ug/g
**Suzuki 2014**	24	GLP-1 receptor agonists	58.6	15.9	62.5	9.8	2.2	28.2	7.2	2.4		73	19	ug/g
**SWIM**	221	Sulfonylureas	50.1	7.8	51.1	7.9		25.2	3.7	15.7				
**SWIM**	219	DPP-4 inhibitors	51.1	6.6	47	8		26	3.3	15				
**Takagi 2003**	23	Thiazolidinediones	64	10	86.9	6.8	0.6	25.6	2.8		100			
**Takagi 2003**	21	Standard treatments	65	9	66.7	6.7	1.2	24.5	2.9		100			
**Takahara-2020**	20	SGLT2 inhibitors	59.4	8.66	47.4	7.95	0.76	26.05	3.9	10.95		90.1		
**Takahara-2020**	20	Standard treatments	54.8	9.7	50	7.6	0.52	26.13	5.03	11.48		90.18		
**Takahashi-2022**	27	SGLT2 inhibitors	56.7	13.8	62.5	6.6	0.8	29.8	3.9					
**Takahashi-2022**	28	Standard treatments	55.6	16.3	53.8	6.7	0.5	29.1	5.6					
**Takase 2007**	20	Thiazolidinediones	67.8	8.9	55	7	1.3	24.7	1.3					
**Takase 2007**	19	Alpha-glucosidase inhibitors	68.2	7.4	63.2	6.9	1.3	24.2	2.6					
**Takashima 2018**	20	SGLT2 inhibitors	64.7	9.8	55	7.5	0.9	24.9	3.1		20	57	139	mg/g
**Takashima 2018**	20	Standard treatments	65.4	10.4	60	7.3	0.7	25.5	3.2		15	55	159	mg/g
**Takeshita-2022**	20	SGLT2 inhibitors	55.6	17.4	35	7.9	0.8	31	6.7					
**Takeshita-2022**	20	Sulfonylureas	51.3	21.3	70	8.0	0.9	32	8.8					
**Tao 2018**	21	Metformin	28	3	100	7.3	0.2	26.4	4.150037					
**Tao 2018**	21	DPP-4 inhibitors	30	5	100	7.4	0.3	27.2	5.28399					
**Taskinen 2011**	523	DPP-4 inhibitors	56.5	10.1	53	8.1	0.9	29.9	4.8					

Study ID	N	Intervention	Age (mean)	Age (SD)	% of males	HbA1c (mean)	HbA1c (SD)	BMI (mean)	BMI (SD)	Duration of diabetes	% of CV disease	eGFR	ACR	ACR unit
**Taskinen 2011**	177	Standard treatments	56.6	10.9	57	8	0.9	30.1	5					
**TECOS**	7332	DPP-4 inhibitors	65.4	7.9	70.9	7.2	0.5	30.2	5.6	11.6	100	75	10.3	mg/g
**TECOS**	7339	Standard treatments	65.5	8	70.5	7.2	0.5	30.2	5.7	11.6	100	75	11.4	mg/g
**Teramoto 2007**	46	Sulfonylureas	56.4	11	76.1	8.4	1.3	25.2	4.8					
**Teramoto 2007**	46	Thiazolidinediones	57	10.7	71.7	8	1.3	24.7	3.4					
**Teupe 1991**	50	Metformin	51.5	10.1	40	9	1.3			8.1				
**Teupe 1991**	50	Standard treatments	56	7.6	40	8.7	0.8			6.4				
**Thrasher 2014**	106	DPP-4 inhibitors	53.7	10.1	56.6	8.7	1.1	32	6.1					
**Thrasher 2014**	120	Standard treatments	54.1	9.9	50.8	8.8	1.2	33.4	5.4					
**TIDE**	399	Thiazolidinediones	66.5	6.4	59.6	7.4	0.9	30.7	5.3	9.1	36			
**TIDE**	392	Thiazolidinediones	66.3	6.6	57.4	7.4	0.8	30.6	5.4	8.5	33			
**TIDE**	541	Standard treatments	66.4	6.8	59.3	7.3	0.9	30.4	5.2	8.7	35			
**Tinahones 2017_1**	126	DPP-4 inhibitors	56.6	9.5	56.6	8	1	31.3	5.4			92		
**Tinahones 2017_1**	128	Standard treatments	56.8	9.4	56	8	0.9	30.8	4.8			89.8		
**Tinahones 2017_2**	112	DPP-4 inhibitors	56.6	9.8	47.3	7.8	0.7	30.8	4.6			89		
**Tinahones 2017_2**	112	Standard treatments	56.1	10.6	57.3	7.9	0.9	32	5.3			91.1		
**Tofogliflozin 003**	55	SGLT2 inhibitors	57	9.1	67.2	8.4	0.8	25.8	4.1	6.7		86		
**Tofogliflozin 003**	55	SGLT2 inhibitors	56.6	10.2	67.2	8.3	0.8	25	4.6	6.4		86.78		
**Tofogliflozin 003**	55	SGLT2 inhibitors	58.6	9.8	66.7	8.5	0.8	25.1	3.5	6.3		84.9		
**Tofogliflozin 003**	55	Standard treatments	56.8	9.9	66.1	8.4	0.8	26	4.1	6		83.78		
**Tolman 2009**	1057	Sulfonylureas			55.5	9.5	2	32.5	6	6				
**Tolman 2009**	1063	Thiazolidinediones			57.2	9.5	2	32.5	6.1	6				
**ToPiND**	21	SGLT2 inhibitors	58.4	12.2	61.9	7.22	0.88	29.4	1			78.1		
**ToPiND**	19	Thiazolidinediones	58.8	8.1	42.1	7.06	0.64	30.8	1.1			71.2		

Study ID	N	Intervention	Age (mean)	Age (SD)	% of males	HbA1c (mean)	HbA1c (SD)	BMI (mean)	BMI (SD)	Duration of diabetes	% of CV disease	eGFR	ACR	ACR unit
**TOPSCORE**	38	DPP-4 inhibitors	70	9	79	7.2	1	24.8	4.1		100			
**TOPSCORE**	37	Standard treatments	72	10	83	7.3	1.2	25.1	4.1		100			
**TOSCA.IT**	1493	Sulfonylureas	62.2	6.5	58	7.7	0.5	30.4	4.5	8.5	10			
**TOSCA.IT**	1535	Thiazolidinediones	62.4	6.4	59	7.7	0.5	30.2	4.4	8.4	12			
**Trakarnvanich-2021**	100	DPP-4 inhibitors	62.9	8.7	58.5	8.37	1.95	28.5	5.9	13		46.83		
**Trakarnvanich-2021**	101	Standard treatments	62.7	10.5	59.1	8.12	1.7	27.8	4.7	11		44.27		
**Trial 843**	73	SGLT2 inhibitors	61	9.1	74	8.1	0.8	25.7	3.5	9.6		82		
**Trial 843**	70	Standard treatments	60	10.4	75.7	8	0.6	26	3.1	9		83.4		
**Tripathy 2013**	15	Thiazolidinediones	56	9.3	63.6	7	0.7	33.6	6.3					
**Tripathy 2013**	14	Standard treatments	57	6.6	77.8	8	1.5	33.4	5.7					
**TROICA**	108	DPP-4 inhibitors	61.4	9.2	37.4	8.2	0.8	25.1	3.1	13				
**TROICA**	110	Standard treatments	60.4	8.8	42.2	8.2	0.9	24.7	2.7	12.8				
**Truitt 2010**	91	Thiazolidinediones	56.6	10.1	58.2	8	0.8	32.9	5.7	6.6				
**Truitt 2010**	86	Thiazolidinediones	56.4	10.8	52.3	8.2	1	33	6.5	6.7				
**Truitt 2010**	85	Thiazolidinediones	55	10.8	56.5	8.1	0.9	33.1	6.4	5.2				
**Truitt 2010**	87	Thiazolidinediones	55.4	10.9	52.9	8	0.8	32.9	6	6.2				
**Truitt 2010**	92	Standard treatments	55.3	9.3	51.1	8.2	1	32.2	5.8	6.7				
**Türkmen Kemal 2007**	16	Metformin	56.8	8.8	25	6.3	1.2	34.5	7.9	1.5				
**Türkmen Kemal 2007**	13	Thiazolidinediones	55.92	8.26	23.1	6.2	0.7	30.8	6.9	2.75				
**Türkmen Kemal 2007**	17	Standard treatments	54.59	10.76	23.5	5.7	0.6	31.4	5.4	1.2				
**Tuttolomondo-2021**	60	GLP-1 receptor agonists	69.7	8.6	42.8	7.4	0.7	27.6	3.4	10.4		77.4		
**Tuttolomondo-2021**	64	Standard treatments	67.6	5.1	37.5	7.2	0.6	27.9	3.2	10.2		76.1		
**UKPDS 80**	342	Metformin	64	9	45.5	8.433333	1.862968	31.7	5.4				29.66667	

Study ID	N	Intervention	Age (mean)	Age (SD)	% of males	HbA1c (mean)	HbA1c (SD)	BMI (mean)	BMI (SD)	Duration of diabetes	% of CV disease	eGFR	ACR	ACR unit
**UKPDS 80**	411	Standard treatments	63	9	46	8.8	1.862022	32.2	5.7				36.93333	
**UKPDS 44**	973	Alpha-glucosidase inhibitors	60	9		8.9	3.266617	29.8	5.6	7.9				
**UKPDS 44**	973	Standard treatments	60	9		8.833333	3.118134	29.6	5.7	8				
**Umpierrez 2006**	96	Sulfonylureas	51.6	11.8	55.2	8.4	0.7	34.5	6.7	4.9				
**Umpierrez 2006**	107	Thiazolidinediones	55.7	9.7	52.3	8.3	0.8	33.8	6.6	5.9				
**UTOPIA**	169	SGLT2 inhibitors	61.3	9.3	58.6	7.4	0.7	27	5.8	12.1		80.7	20	mg/g
**UTOPIA**	171	Standard treatments	60.9	9.7	58.2	7.3	0.7	27	4.6	12.5		81.9	30.8	mg/g
**Vähätalo 2007**	26	Metformin				10.1								
**Vähätalo 2007**	15	Sulfonylureas				9.4								
**Van Eyk 2019**	22	GLP-1 receptor agonists	55	11	36	8.1	0.9	30.4	3.8	19				
**Van Eyk 2019**	25	Standard treatments	55	9	44	8.6	1.1	28.6	4	17				
**Van Gaal 2014**	161	DPP-4 inhibitors	43.4	4.7	45.3	8.1	1	36.8	6.3	4.4				
**Van Gaal 2014**	158	GLP-1 receptor agonists	42.7	5.2	34.8	8.2	0.9	36.8	7.3	4.4				
**Vanderheiden 2016**	35	GLP-1 receptor agonists	52.8	8.1	34	9	1.2	40.7	6.7	16				
**Vanderheiden 2016**	36	Standard treatments	55.5	6.6	39	8.9	1	41.6	10.4	18				
**Varghese 2009**	28	Thiazolidinediones	62.2	8.2	78			27.9	4.1					
**Varghese 2009**	29	Standard treatments	65.6	6.1	79			28.6	4.3					
**Veleba 2015_1**	18	Thiazolidinediones	62.33333	4.022289				32.33333	4.665855					
**Veleba 2015_1**	17	Standard treatments	61.66667	5.658659				30.7	4.688603					
**Veleba 2015_2**	17	Thiazolidinediones	60.43333	8.245474				33.03333	9.61972					

Study ID	N	Intervention	Age (mean)	Age (SD)	% of males	HbA1c (mean)	HbA1c (SD)	BMI (mean)	BMI (SD)	Duration of diabetes	% of CV disease	eGFR	ACR	ACR unit
**Veleba 2015_2**	17	Standard treatments	59.7	6.467039				33.56667	6.709553					
**VERIFY**	998	DPP-4 inhibitors	54.1	9.5	45.4	6.7	0.4	31.2	4.8	4.7				
**VERIFY**	1003	Standard treatments	54.6	9.2	48.7	6.7	0.5	31	4.7	4.9				
**VERTIS Asia**	169	SGLT2 inhibitors	56.3	9.3	58	8.1	0.9	25.7	3.2	7.5				
**VERTIS Asia**	170	SGLT2 inhibitors	56.1	9	55.9	8.1	0.9	26	2.8	7				
**VERTIS Asia**	167	Standard treatments	56.9	9	52.7	8.1	1	26.1	3.4	6.4		93		
**VERTIS CV**	2752	SGLT2 inhibitors	64.3	8.2	70.9	8.3	1	31.8	5.3	13.1	100	76	30.7	mg/g
**VERTIS CV**	2747	SGLT2 inhibitors	64.4	8	69.7	8.2	1	32	5.4	12.7	100	76.2	31.3	mg/g
**VERTIS CV**	2747	Standard treatments	64.4	8	69.3	8.2	0.9	32	5.5	13.1	100	75.7	30.7	mg/g
**VERTIS FACTORIAL**	247	DPP-4 inhibitors	54.8	10.7	62.3	8.6	1	31.5	5.8	7.3		93		
**VERTIS FACTORIAL**	248	SGLT2 inhibitors	55.3	9.5	54	8.6	1	31.5	5.8	7.3		92		
**VERTIS FACTORIAL**	250	SGLT2 inhibitors	55.1	10.1	50.8	8.6	1	31.8	6.2	7.1				
**VERTIS MET**	207	SGLT2 inhibitors	56.6	8.1	46.9	8.1	0.9	30.8	4.8	7.9		88.9		
**VERTIS MET**	205	SGLT2 inhibitors	56.9	9.4	45.4	8.1	0.9	31.1	4.5	8.1		91		
**VERTIS MET**	209	Standard treatments	56.5	8.7	46.9	8.2	0.9	30.7	4.7	8		91.6		
**VERTIS MONO**	152	SGLT2 inhibitors	56.2	10.8	59.2	8.4	1.1	32.5	5.7	5.2		88		
**VERTIS MONO**	156	SGLT2 inhibitors	56.8	11.4	57.1	8.2	0.9	33.2	7.4	5.1		89		
**VERTIS MONO**	153	Standard treatments	56.1	10.9	53.6	8.1	0.9	33.3	6.8	4.6		86		
**VERTIS RENAL**	155	SGLT2 inhibitors	67.5	8.5	48.4	8.2	0.9	31.7	5.3	14.5	49.7	47		
**VERTIS RENAL**	158	SGLT2 inhibitors	66.7	8.3	53.2	8.2	1	32.6	6.8	14.9	50	47		
**VERTIS RENAL**	154	Standard treatments	67.5	8.9	46.8	8.1	0.9	33.2	6.1	13.1	49.4	46		

Study ID	N	Intervention	Age (mean)	Age (SD)	% of males	HbA1c (mean)	HbA1c (SD)	BMI (mean)	BMI (SD)	Duration of diabetes	% of CV disease	eGFR	ACR	ACR unit
**VERTIS SITA2**	153	SGLT2 inhibitors	59.7	8.6	53.6	8	0.8	30.9	6.1	9.2		87		
**VERTIS SITA2**	156	SGLT2 inhibitors	59.2	9.3	51.9	8.1	0.9	31.2	5.5	9.9		87		
**VERTIS SITA2**	153	Standard treatments	58.3	9.2	65.4	8	0.9	30.3	6.4	9.4		90		
**VERTIS SU**	437	Sulfonylureas	57.8	9.2	51.3	7.8	0.6	31.2	6.4	7.5		87		
**VERTIS SU**	441	SGLT2 inhibitors	58	9.9	43.3	7.8	0.6	31.3	6.2	7.5		87		
**VERTIS SU**	448	SGLT2 inhibitors	58.8	9.7	50.7	7.8	0.6	31.7	5.5	7.4		88		
**Vianna 2017**	21	Sulfonylureas	61	5.3	0	7.3	0.6	33.8	6.7					
**Vianna 2017**	21	DPP-4 inhibitors	62.8	6.4	0	7.4	0.5	31.7	5.8					
**VICTORY**	98	Thiazolidinediones	64.2	7.3	92	6.9	1.3	30.2	4.2	7.8	100			
**VICTORY**	95	Standard treatments	65.1	6.9	92	6.9	0.8	29.5	4.6	8.4	100			
**VISION**	2501	DPP-4 inhibitors	56.5	10.6	54.4	7.2	0.9	25.1	3.2	4.3				
**VISION**	484	Standard treatments	56.2	10.8	49.6	7.2	0.8	25.1	3.2	4.1				
**VIVIDD**	128	DPP-4 inhibitors	62.9	8.5	77.3	7.8	1	29.6	4.6	9.5	64.1			
**VIVIDD**	126	Standard treatments	63.4	10.2	76.2	7.8	1.1	29.3	4.7	9.1	63.5			
**Vongthavaravat 2002**	175	Thiazolidinediones	54.6		45.7	9.1		27.1		5				
**Vongthavaravat 2002**	173	Standard treatments	57.3		42.4	8.9		27.1		6				
**Wada-2022**	154	SGLT2 inhibitors	62.5	10.5	74.7	7.75	1.1	26.7	4.4	15.43		56.3	855.7	mg/g
**Wada-2022**	154	Standard treatments	62.4	11.1	83.8	7.77	1.05	27.1	4.5	16.49		55.2	762.3	mg/g
**Wajcberg 2007**	16	Thiazolidinediones	51	8	43.8	8.1	1.6	32	4	7.1				
**Wajcberg 2007**	15	Standard treatments	49	11.6	46.7	7.9	1.5	31	3.9	6.9				
**Wang 2005**	35	Thiazolidinediones	60.1	8.5	83.3	7.3	0.2	26.1	2.5		100			
**Wang 2005**	35	Standard treatments	62.2	8.6	80	7.3	0.2	25.6	2.7		100			
**Wang 2013**	30	Metformin	54	10.3	62.1	8.1	0.8							
**Wang 2013**	30	Sulfonylureas	55.9	10.5	70	8.4	0.9							

Study ID	N	Intervention	Age (mean)	Age (SD)	% of males	HbA1c (mean)	HbA1c (SD)	BMI (mean)	BMI (SD)	Duration of diabetes	% of CV disease	eGFR	ACR	ACR unit
**Wang 2013**	30	Alpha-glucosidase inhibitors	54.7	8.9	66.7	8.1	0.8							
**Wang 2015**	45	DPP-4 inhibitors	64.3	2.6	43.9	8.3	0.5			12.3				
**Wang 2015**	45	Alpha-glucosidase inhibitors	65.1	3.1	47.5	8.3	0.4			14.2				
**Wang 2016**	205	DPP-4 inhibitors	55.1	10.7	49.8	8	0.8	25.5	3.9					
**Wang 2016**	101	Standard treatments	56.5	8.7	50	8	0.8	25.8	4					
**Wang 2016a**	18	SGLT2 inhibitors	60.7	10.1	61	8	0.3							
**Wang 2016a**	10	Standard treatments	59.3	9	20	9.5	0.4							
**Wang 2017**	191	DPP-4 inhibitors	56.5	8.9	50.8	8.1	0.8	25.9	4.6	7.4				
**Wang 2017**	190	Standard treatments	57.8	9.5	51.3	8.1	0.9	26	4.4	8.2				
**Wang 2019**	258	GLP-1 receptor agonists	55	9.6	53.4	8.5	1.2	26.6	3.7	7.9				
**Wang 2019**	257	GLP-1 receptor agonists	54.5	10	56.7	8.3	1.1	27	3.8	8.1				
**Wang 2019**	259	Basal insulin	55.4	9.2	55.6	8.3	1.1	26.7	3.5	8.4				
**Wang 2020**	30	DPP-4 inhibitors	67.2	7.1	56.7	8.6	3.3	25.8	3.8	8.2	100			
**Wang 2020**	30	GLP-1 receptor agonists	66.1	5.9	46.7	8.9	3.2	25.7	4.3	9	100			
**Wang 2020b**	49	GLP-1 receptor agonists	56.1	11.14	63.27	8.53	1.07	23.96	1.18	9				
**Wang 2020b**	46	Basal-Bolus insulin	60.37	10.83	50	8.47	0.92	23.5	1.24	10.66667				
**Wang-2020a**	46	GLP-1 receptor agonists	55.9	8.9	76.1	9.1	1.4	26.1	3.4	10.9	26.1	71.5	1644.7	mg/g

Study ID	N	Intervention	Age (mean)	Age (SD)	% of males	HbA1c (mean)	HbA1c (SD)	BMI (mean)	BMI (SD)	Duration of diabetes	% of CV disease	eGFR	ACR	ACR unit
**Wang-2020a**	46	Bolus insulin	56.2	8	63	9	1.3	23.5	3.1	11.4	28.3	69.2	1448	mg/g
**Wang-2022**	14	Standard treatments	53.4	5.2	57	8.08	1.13	24.06	2.85					
**Wang-2022**	17	DPP-4 inhibitors	54.4	9	58	7.93	0.91	25.41	3.45					
**Wang-2022**	17	Metformin	55.63	10.9	53	8.6	1.17	26.46	2.86					
**Watanabe 2005**	14	Sulfonylureas	65.1	8.1	85.7	7.2	0.5	24.7	3.7					
**Watanabe 2005**	13	Thiazolidinediones	62.9	10.3	84.6	6.9	0.2	24.4	4.4					
**Weng-2021**	166	SGLT2 inhibitors	54.3	9.5	63.6	8.5	0.8	25.5	2.9	5.53		125.4		
**Weng-2021**	164	SGLT2 inhibitors	54.7	10.7	63.1	8.4	0.9	25.6	3.2	6.39		128.9		
**Weng-2021**	165	Standard treatments	55.3	9.5	57.8	8.5	0.9	25.4	3.1	6.58		128		
**Wolffenbuttel 1999**	109	Sulfonylureas	61	9	68	7	1.2	28	3.4	6				
**Wolffenbuttel 1999**	211	Meglitinides	61	9	62	7.1	1.4	28.4	3.6	6				
**Wolffenbuttel 2000**	183	Thiazolidinediones	60.6	8.7	55.2	9.2	1.2	28.3	3.9	7				
**Wolffenbuttel 2000**	199	Thiazolidinediones	61	9.4	62.8	9.2	1.2	28	3.9	7				
**Wolffenbuttel 2000**	192	Standard treatments	61.9	9.1	57.3	9.2	1.3	28.1	4.1	8				
**Wong 2005**	26	Thiazolidinediones	62.9	7.3		7.3	1.3	24.5	6.2					
**Wong 2005**	26	Standard treatments	61.6	9.7		7.2	1.3	24.9	8.5					
**Wu 2014**	47	Metformin	60.1	9.6	50	7.2	2.4	24.9	5.8	73				
**Wu 2014**	46	Thiazolidinediones	60.3	9.7	51.5	7.1	2.5	24.6	5.6	7.2				
**Wu 2015**	34	DPP-4 inhibitors	52.5	11	65.7	8	1.5	24.4	2.1					
**Wu 2015**	23	Standard treatments	51.2	7.5	50	8	0.7	24.1	2.3					
**Xiao 2015**	40	Sulfonylureas	53.6	3.6	58.3	8.7	0.8	26.6	1.2					
**Xiao 2015**	40	Thiazolidinediones	54.2	4.9	58.8	8.7	0.8	26.5	1.3					
**Xiao 2016**	23	Sulfonylureas	69.1	6.5	55.6	7.3	1.2	27.9	3.9					
**Xiao 2016**	18	DPP-4 inhibitors	68.7	6.3	56.5	7.3	1	28.3	3.8					

Study ID	N	Intervention	Age (mean)	Age (SD)	% of males	HbA1c (mean)	HbA1c (SD)	BMI (mean)	BMI (SD)	Duration of diabetes	% of CV disease	eGFR	ACR	ACR unit
**Xu 2017**	549	Sulfonylureas	54	9.8	53.9	7.68	0.74	25.9	3.3	5.8				
**Xu 2017**	550	Sulfonylureas	53.3	9.9	55.1	7.8	0.8	25.8	3.5	5.5				
**Xu 2017**	551	Alpha-glucosidase inhibitors	52.6	10.2	56.6	7.7	0.7	25.7	3.4	5.9				
**Xu 2017**	545	Meglitinides	52.1	9.9	54.1	7.7	0.7	25.9	3.3	5.7				
**Xu-2022**	143	DPP-4 inhibitors	57.9	8.73	50	7.78	0.54	25.58	2.99					
**Xu-2022**	71	Standard treatments	57.3	9.14	50.7	7.88	0.58	25.38	3.51					
**Yagoglu-2020**	90	DPP-4 inhibitors	66.8	9.6	45.6	8.3	1.6					38.75		
**Yagoglu-2020**	86	Standard treatments	68.2	7.6	39.2	7.7	1.1					37.25		
**Yabiku2017**	92	Metformin												
**Yabiku2017**	91	Thiazolidinediones												
**Yabiku2017**	92	DPP-4 inhibitors												
**Yabiku2017**	91	Standard treatments												
**Yale 2013**	89	SGLT2 inhibitors	67.9	8.2	53.9	8	0.8	33.4	6.5	17	51.7	38.5	30.1	ug/mg
**Yale 2013**	90	SGLT2 inhibitors	69.5	8.2	64.4	7.9	0.9	32.4	5.5	15.6	55.6	39.7	23.7	ug/mg
**Yale 2013**	90	Standard treatments	68.2	8.4	63.3	8	0.9	33.1	6.5	16.4	56.7	40.1	31.3	ug/mg
**Yamakage 2019**	27	SGLT2 inhibitors	58.4	13	40.7	7.5	0.8	31.3	7.6			76.2		
**Yamakage 2019**	27	Standard treatments	60.7	11.9	51.9	7.4	0.9	30.7	6.2			73		
**Yamamoto 2018**	12	GLP-1 receptor agonists	60	7.8	66.7	7.3	1.2	27.8	3.1	10.8	0			
**Yamamoto 2018**	13	Bolus insulin	61.5	10.6	61.5	6.9	0.8	25.9	4.2	9.6	23			
**Yamanouchi 2005**	39	Metformin	54.7	9.8	51.3	9.9	0.7	26.2	3.8	3				
**Yamanouchi 2005**	37	Sulfonylureas	55.6	9.3	51.4	9.8	0.7	25.6	3.5	3.3				
**Yamanouchi 2005**	38	Thiazolidinediones	55.2	9.2	47.4	10.2	0.8	25.8	4.2	3.2				

Study ID	N	Intervention	Age (mean)	Age (SD)	% of males	HbA1c (mean)	HbA1c (SD)	BMI (mean)	BMI (SD)	Duration of diabetes	% of CV disease	eGFR	ACR	ACR unit
**Yamasaki 2005**	53	Alpha-glucosidase inhibitors	58.6	7.5	51	8.2	1.3	23	3.67	13.1				
**Yamasaki 2005**	50	Standard treatments	60.4	9.6	60	8.4	1.4	22.7	2.92	12.7				
**Yan 2019**	27	DPP-4 inhibitors	45.7	9.2	77.8	7.6	0.9	29.7	2.8	4.3				
**Yan 2019**	24	GLP-1 receptor agonists	43.1	9.7	70.8	7.8	1.4	30.1	3.3	3.3				
**Yan 2019**	24	Basal insulin	45.6	7.6	58.3	7.7	0.9	29.6	3.5	5.8				
**Yang 2002**	30	Thiazolidinediones	58.9	9.4	43.3	9.5	1.1	25.76	2.87					
**Yang 2002**	34	Standard treatments	57.8	8.9	38.2	9.7	1.4	25.84	3.5					
**Yang 2011**	283	DPP-4 inhibitors	53.8	10.4	48.1	7.9	0.8	26.3	3.6	5.1				
**Yang 2011**	287	Standard treatments	54.4	10.1	48.4	7.9	0.8	26.1	3.5	5.1				
**Yang 2012**	197	DPP-4 inhibitors	54.1	9	47	8.5	0.9	25.3	3.1	6.4				
**Yang 2012**	198	Standard treatments	55.1	9.8	55	8.5	0.9	25.3	3.6	7.3				
**Yang 2013**	90	DPP-4 inhibitors	54.25	2.245845	65.5	8.2	1	25.425	1.082089	3.2				
**Yang 2013**	92	Standard treatments	52.25	3.062515	50.6	8.3	1.1	26.525	1.122922	2.9				
**Yang 2015**	37	DPP-4 inhibitors	54.4	9.9	40.5	7.1	0.7	24.6	3	3.2				
**Yang 2015**	33	DPP-4 inhibitors	57.7	9.7	60	7.2	0.7	25	2.6	3.4				
**Yang 2015**	39	Standard treatments	56.7	9.7	63.2	7.1	0.6	25.4	3.2	4.1				
**Yang 2015a**	143	DPP-4 inhibitors	58.3	9.8	55.2	8.6	0.9	24.8	3	6.9				
**Yang 2015a**	136	Standard treatments	58.7	9.3	58.1	8.7	1	25	2.8	6.9				
**Yang 2016**	152	SGLT2 inhibitors	54.6	9.5	57.9	8.2	0.8	26.2	3.5	5.3				
**Yang 2016**	147	SGLT2 inhibitors	53.1	9.1	45.6	8.1	0.7	26.4	3.5	4.2				
**Yang 2016**	145	Standard treatments	53.5	9.2	59.3	8.1	0.9	25.7	2.9	5.3				
**Yang 2018**	139	SGLT2 inhibitors	56.5	8.4	47.5	8.5	0.8	26.4	3.8	12.7		88.24		

Study ID	N	Intervention	Age (mean)	Age (SD)	% of males	HbA1c (mean)	HbA1c (SD)	BMI (mean)	BMI (SD)	Duration of diabetes	% of CV disease	eGFR	ACR	ACR unit
**Yang 2018**	133	Standard treatments	58.6	8.9	48.1	8.6	0.8	26.7	3.3	12.2		88.32		
**Yang-2021**	104	DPP-4 inhibitors	60.1	9.5	50	8.49	0.74	25.79	3.81					
**Yang-2021**	102	Standard treatments	57.1	10.6	52.9	8.51	0.83	25.34	3.07					
**Yee 2010**	28	Thiazolidinediones	62.6	8.2	78.6	7.7	1.1	28	5.129518	7.5	46.4			
**Yee 2010**	29	Standard treatments	66.1	6	79.3	7.6	1.4	28.4	4.670806	10	48.3			
**Yki-Jarvinen 1999**	19	Metformin	57	8.7	58	9.8	1.7	28.9	4.8					
**Yki-Jarvinen 1999**	22	Sulfonylureas	61	9.4	59	9.8	1.4	29.7	4.7					
**Yki-Jarvinen 2013**	631	DPP-4 inhibitors	59.7	9.9	52.1	8.3	0.9	30.8	5.4					
**Yki-Jarvinen 2013**	630	Standard treatments	60.4	10	52.2	8.3	0.9	31.2	4.9					
**Yoon 2011**	261	DPP-4 inhibitors	50.2	10.2	52.5	9.5	1.2	29.7	5.1	2.6				
**Yoon 2011**	259	Standard treatments	51.7	11.2	56	9.5	1.2	29.6	5.2	2.1				
**Yoon 2011a**	114	Metformin	51.8	8.5	57.9	7.9	0.8	25.7	3.2					
**Yoon 2011a**	118	Sulfonylureas	50.8	8.9	55.9	7.8	0.8	25.5	3.1					
**Yoon 2011a**	117	Thiazolidinediones	50.1	8.2	52.1	7.8	0.8	25.8	3.3					
**Yuan 2012**	26	Metformin	56.8	7.6	46.2	8.1	1.9	29.3	2.6					
**Yuan 2012**	33	GLP-1 receptor agonists	58.5	10.6	51.5	8.3	1.6	30.6	2.8					
**Zang 2016**	184	DPP-4 inhibitors	51.4	11	63.6	8.1	0.8	27.2	4	5.2				
**Zang 2016**	184	GLP-1 receptor agonists	51.7	10.7	55.7	8.1	0.8	27.3	3.4	5.3				
**ZEUS II**	188	DPP-4 inhibitors	61.1	8.6	32.4	8.4	1	26.7	4.1	16.3				
**ZEUS II**	95	Standard treatments	59	10.3	30.5	8.4	1	26.8	4.2	16.5				
**Zhang 2020**	30	Thiazolidinediones	51.5	12.1	50	8.1	1.7	27.1	3.8					

Study ID	N	Intervention	Age (mean)	Age (SD)	% of males	HbA1c (mean)	HbA1c (SD)	BMI (mean)	BMI (SD)	Duration of diabetes	% of CV disease	eGFR	ACR	ACR unit
**Zhang 2020**	30	GLP-1 receptor agonists	50.2	11.5	43.3	8.1	2	27.6	5.2					
**Zhang 2020a**	33	GLP-1 receptor agonists	58.85	12.54	70	8.67	1.03	23.64	2.86	6.59				
**Zhang 2020a**	33	Basal-Bolus insulin	58.03	13.32	43.7	8.32	0.96	24.36	2.52	7.81				
**Zhou-2022**	16	Thiazolidinediones	47.69	5.32	52	8.33	0.92	27.69	1.45					
**Zhou-2022**	16	Standard treatments	47.72	5.26	54	8.34	0.95	27.61	1.49					
**Zhu 2003**	221	Thiazolidinediones	58.9	6.9	48	9.9	1.6	24.9	3.1	7.9				
**Zhu 2003**	221	Thiazolidinediones	59	7.5	41	9.8	1.5	24.8	3.4	7.2				
**Zhu 2003**	112	Standard treatments	58.8	7.7	46	9.8	1.3	25.1	2.8	7.6				
**Zib 2007**	16	Thiazolidinediones	51	9	43.8	11	2.8	32	8	5.6				
**Zib 2007**	16	Standard treatments	47	11	50	10.6	1.4	33	7	6.6				

Abbreviations: HbA1c, glycated hemoglobin (%); BMI, body mass index (kg/m^2); CV, cardiovascular; eGFR, estimated glomerular filtration rate ($mL/min/1.73m^2$); ACR, albumin-to-creatinine ratio; N, no. of patients.

Appendix 3 Risk of bias assessments

Notes: The study assessed the risk of bias for all included studies using the tool developed by the CLARITY Group at McMaster University. Each following question is answered by definitely yes, probably yes, probably no, and definitely no.

D1: Was the allocation sequence adequately generated?

Examples of low risk of bias: Referring to a random number table; Using a computer random number generator; Coin tossing; Shuffling cards or envelopes; Throwing dice; Drawing of lots; Minimization with or without a random element.

Examples of high risk of bias: Sequence generated by odd or even date of birth; Sequence generated by some rule based on date (or day) of admission; Sequence generated by some rule based on hospital or clinic record number; Allocation by judgement of the clinician; Allocation by preference of the participant; Allocation based on the results of a laboratory test or a series of tests; Allocation by availability of the intervention.

D2: Was allocation adequately concealed?

Examples of low risk of bias allocation concealment techniques: Central allocation (including telephone, web-based, and pharmacy-controlled, randomization);

Examples of possible low risk of bias: Sequentially numbered drug containers of identical appearance; Sequentially numbered, opaque, sealed envelopes.

Examples of high risk of bias allocation generation techniques: Using an open random allocation schedule (e.g. a list of random numbers); Assignment envelopes were used without appropriate safeguards (e.g. if envelopes were unsealed or non-opaque or not sequentially numbered); Alternation or rotation; Date of birth; Case record number; Any other explicitly unconcealed procedure.

D3: Blinding: Was knowledge of the allocated interventions adequately prevented?

Examples of low risk of bias: No blinding, but the review authors judge that the outcome and the outcome measurement are not likely to be influenced by lack of blinding; Blinding of participants and key study personnel ensured, and unlikely that the blinding could have been broken; Either participants or some key study personnel were not blinded, but outcome assessment was blinded and the nonblinding of others unlikely to introduce bias.

Examples of high risk of bias: No blinding or incomplete blinding, and the outcome or outcome measurement is likely to be influenced by lack of blinding; Blinding of key study participants and personnel attempted, but likely that the blinding could have been broken; Either participants or some key study personnel were not blinded, and the non-blinding of others likely to introduce bias.

3.a) Were patients blinded?

3.b) Were healthcare providers blinded?

3.c) Were data collectors blinded?

3.d) Were outcome assessors blinded?

3.e) Were data analysts blinded?

D4: Was loss to follow-up (missing outcome data) infrequent?

Examples of low risk of bias: No missing outcome data; Reasons for missing outcome data unlikely to be related to true outcome (for survival data, censoring unlikely to be introducing bias); Missing outcome data balanced in numbers across intervention groups, with similar reasons for missing data across groups; For dichotomous outcome data, the proportion of missing outcomes compared with observed event risk not enough to have a important impact on the intervention effect estimate; For continuous outcome data, plausible effect size (difference in means or standardized difference in means) among missing outcomes not enough to have an important impact on observed effect size; Missing data have been imputed using appropriate methods.

Examples of high risk of bias: Reason for missing outcome data likely to be related to true outcome, with either imbalance in numbers or reasons for missing data across intervention groups; For dichotomous outcome data, the proportion of missing outcomes compared with observed event risk enough to induce important bias in intervention effect estimate; For continuous outcome data, plausible effect size (difference in means or standardized difference in means)

among missing outcomes enough to induce clinically relevant bias in observed effect size; ‘As-treated’ analysis done with substantial departure of the intervention received from that assigned at randomization; Potentially inappropriate application of simple imputation.

D5: Are reports of the study free of suggestion of selective outcome reporting?

Examples of low risk of bias: The study protocol is available and all of the study’s pre-specified (primary and secondary) outcomes that are of interest in the review have been reported in the pre-specified way; The study protocol is not available, but it is clear that the published reports include all expected outcomes, including those that were pre-specified (convincing text of this nature may be uncommon).

Examples of high risk of bias: Not all of the study’s pre-specified primary outcomes have been reported; One or more primary outcomes is reported using measurements, analysis methods or subsets of the data (e.g. subscales) that were not pre-specified; One or more reported primary outcomes were not pre-specified (unless clear justification for their reporting is provided, such as an unexpected adverse effect); One or more outcomes of interest in the review are reported incompletely so that they cannot be entered in a meta-analysis; The study report fails to include results for a key outcome that would be expected to have been reported for such a study.

D6: Was the study apparently free of other problems that could put it at a risk of bias?

Examples of low risk of bias: The study appears to be free of other sources of bias.

Examples of high risk of bias: Had a potential source of bias related to the specific study design used; Stopped early due to some data-dependent process (including a formal-stopping rule); Had extreme baseline imbalance; Has been claimed to have been fraudulent; Had some other problem.

Study ID	Domain 1	Domain 2	Domain 3	Domain 4	Domain 5	Domain 6
**1860-LIRA-DPP-4**	**Definitely yes (low risk of bias)**	**Definitely yes (low risk of bias)**	**Definitely no (high risk of bias)**	**Definitely yes (low risk of bias)**	**Definitely yes (low risk of bias)**	**Probably yes**
**3D**	**Definitely yes (low risk of bias)**	**Definitely yes (low risk of bias)**	**Definitely no (high risk of bias)**	**Definitely yes (low risk of bias)**	**Definitely yes (low risk of bias)**	**Probably yes**
**4B**	**Definitely yes (low risk of bias)**	**Probably yes**	**Definitely no (high risk of bias)**	**Definitely yes (low risk of bias)**	**Definitely yes (low risk of bias)**	**Probably yes**
**4T**	**Definitely yes (low risk of bias)**	**Definitely yes (low risk of bias)**	**Definitely no (high risk of bias)**	**Definitely yes (low risk of bias)**	**Definitely yes (low risk of bias)**	**Probably yes**
**Abdullah-2021**	**Definitely no (high risk of bias)**	**Probably no**	**Definitely yes (low risk of bias)**	**Definitely yes (low risk of bias)**	**Probably no**	**Probably yes**
**Abe 2008**	**Probably yes**	**Probably yes**	**Definitely no (high risk of bias)**	**Probably yes**	**Definitely yes (low risk of bias)**	**Probably no**
**Abe 2016**	**Probably no**	**Definitely no (high risk of bias)**	**Definitely no (high risk of bias)**	**Definitely yes (low risk of bias)**	**Definitely yes (low risk of bias)**	**Definitely yes (low risk of bias)**
**ACTION-J**	**Probably no**	**Definitely no (high risk of bias)**	**Definitely no (high risk of bias)**	**Probably no**	**Definitely yes (low risk of bias)**	**Probably yes**
**Adel-2022**	**Definitely yes (low risk of bias)**	**Definitely yes (low risk of bias)**	**Definitely yes (low risk of bias)**	**Definitely yes (low risk of bias)**	**Probably no**	**Definitely yes (low risk of bias)**
**ADOPT**	**Probably yes**	**Definitely yes (low risk of bias)**	**Definitely yes (low risk of bias)**	**Probably yes**	**Definitely yes (low risk of bias)**	**Definitely yes (low risk of bias)**
**Ahmann 2015**	**Definitely yes (low risk of bias)**	**Probably yes**	**Probably yes**	**Probably no**	**Definitely yes (low risk of bias)**	**Probably yes**
**Ahrén 2004**	**Probably yes**	**Probably yes**	**Definitely yes (low risk of bias)**	**Probably yes**	**Definitely yes (low risk of bias)**	**Definitely yes (low risk of bias)**
**Allegretti 2019**	**Definitely yes (low risk of bias)**	**Definitely yes (low risk of bias)**	**Definitely yes (low risk of bias)**	**Definitely yes (low risk of bias)**	**Definitely yes (low risk of bias)**	**Definitely yes (low risk of bias)**

Study ID	Domain 1	Domain 2	Domain 3	Domain 4	Domain 5	Domain 6
**Alogliptin Study 007**	Probably yes	Probably yes	Definitely yes (low risk of bias)	Probably yes	Definitely yes (low risk of bias)	Definitely yes (low risk of bias)
**Alogliptin Study 009**	Probably yes	Probably yes	Definitely yes (low risk of bias)	Definitely yes (low risk of bias)	Definitely yes (low risk of bias)	Probably yes
**Alogliptin Study 010**	Probably yes	Probably yes	Probably yes	Definitely yes (low risk of bias)	Definitely yes (low risk of bias)	Definitely yes (low risk of bias)
**Alvarsson 2003**	Probably no	Definitely no (high risk of bias)	Definitely no (high risk of bias)	Probably yes	Definitely yes (low risk of bias)	Definitely no (high risk of bias)
**AMPLITUDE-M**	Definitely yes (low risk of bias)	Definitely yes (low risk of bias)	Definitely yes (low risk of bias)	Probably no	Definitely yes (low risk of bias)	Definitely yes (low risk of bias)
**AMPLITUDE-O**	Definitely yes (low risk of bias)	Definitely yes (low risk of bias)	Definitely yes (low risk of bias)	Definitely yes (low risk of bias)	Definitely yes (low risk of bias)	Definitely yes (low risk of bias)
**Ando-2021**	Definitely yes (low risk of bias)	Definitely yes (low risk of bias)	Probably no	Definitely yes (low risk of bias)	Definitely yes (low risk of bias)	Definitely yes (low risk of bias)
**APOLLO**	Definitely yes (low risk of bias)	Definitely yes (low risk of bias)	Definitely no (high risk of bias)	Definitely yes (low risk of bias)	Definitely yes (low risk of bias)	Definitely yes (low risk of bias)
**Apovian 2010**	Definitely yes (low risk of bias)	Definitely yes (low risk of bias)	Definitely yes (low risk of bias)	Definitely yes (low risk of bias)	Definitely yes (low risk of bias)	Probably yes
**APPROACH**	Probably yes	Probably yes	Definitely yes (low risk of bias)	Definitely yes (low risk of bias)	Definitely yes (low risk of bias)	Probably yes
**APRIME**	Probably yes	Definitely no (high risk of bias)	Definitely no (high risk of bias)	Probably no	Definitely yes (low risk of bias)	Probably yes
**Araki 2015**	Definitely yes (low risk of bias)	Probably no	Probably yes	Definitely yes (low risk of bias)	Definitely yes (low risk of bias)	Definitely yes (low risk of bias)
**Araki 2015a**	Definitely yes (low risk of bias)	Definitely no (high risk of bias)	Definitely no (high risk of bias)	Probably yes	Definitely yes (low risk of bias)	Probably yes

Study ID	Domain 1	Domain 2	Domain 3	Domain 4	Domain 5	Domain 6
Arechavaleta 2011	Definitely yes (low risk of bias)	Definitely yes (low risk of bias)	Definitely yes (low risk of bias)	Definitely yes (low risk of bias)	Definitely yes (low risk of bias)	Definitely yes (low risk of bias)
Arjona Ferreira 2013	Definitely yes (low risk of bias)	Probably yes	Definitely yes (low risk of bias)	Probably no	Definitely yes (low risk of bias)	Probably yes
Arjona Ferreira 2013a	Definitely yes (low risk of bias)	Probably yes	Definitely yes (low risk of bias)	Probably no	Definitely yes (low risk of bias)	Probably yes
Arturi 2017	Definitely yes (low risk of bias)	Definitely no (high risk of bias)	Definitely no (high risk of bias)	Probably yes	Definitely yes (low risk of bias)	Probably yes
Asian Acarbose Study	Probably yes	Probably yes	Definitely yes (low risk of bias)	Probably no	Definitely yes (low risk of bias)	Definitely yes (low risk of bias)
Aso 2019	Probably yes	Probably no	Definitely no (high risk of bias)	Definitely yes (low risk of bias)	Definitely yes (low risk of bias)	Probably yes
Avilés-Santa 1999	Probably no	Probably no	Probably yes	Definitely no (high risk of bias)	Definitely yes (low risk of bias)	Probably no
AWARD-1	Definitely yes (low risk of bias)	Definitely yes (low risk of bias)	Probably yes	Definitely yes (low risk of bias)	Definitely yes (low risk of bias)	Probably yes
AWARD-10	Definitely yes (low risk of bias)	Definitely yes (low risk of bias)	Definitely yes (low risk of bias)	Definitely yes (low risk of bias)	Definitely yes (low risk of bias)	Definitely yes (low risk of bias)
AWARD-2	Definitely yes (low risk of bias)	Definitely yes (low risk of bias)	Definitely no (high risk of bias)	Probably yes	Definitely yes (low risk of bias)	Probably yes
AWARD-3	Definitely yes (low risk of bias)	Definitely yes (low risk of bias)	Definitely yes (low risk of bias)	Definitely yes (low risk of bias)	Definitely yes (low risk of bias)	Definitely yes (low risk of bias)
AWARD-4	Definitely yes (low risk of bias)	Definitely yes (low risk of bias)	Probably no	Definitely yes (low risk of bias)	Definitely yes (low risk of bias)	Definitely yes (low risk of bias)
AWARD-5	Definitely yes (low risk of bias)	Probably yes	Definitely yes (low risk of bias)	Probably yes	Definitely yes (low risk of bias)	Probably yes

Study ID	Domain 1	Domain 2	Domain 3	Domain 4	Domain 5	Domain 6
**AWARD-7**	**Definitely yes (low risk of bias)**	**Definitely yes (low risk of bias)**	**Probably no**	**Definitely yes (low risk of bias)**	**Definitely yes (low risk of bias)**	**Probably yes**
**AWARD-8**	**Definitely yes (low risk of bias)**	**Probably yes**	**Probably no**	**Definitely yes (low risk of bias)**	**Definitely yes (low risk of bias)**	**Probably yes**
**AWARD-9**	**Definitely yes (low risk of bias)**	**Definitely yes (low risk of bias)**	**Probably no**	**Probably yes**	**Definitely yes (low risk of bias)**	**Probably yes**
**AWARD-CHN1**	**Definitely yes (low risk of bias)**	**Definitely yes (low risk of bias)**	**Definitely yes (low risk of bias)**	**Definitely yes (low risk of bias)**	**Definitely yes (low risk of bias)**	**Probably yes**
**Ba 2017**	**Definitely yes (low risk of bias)**	**Definitely yes (low risk of bias)**	**Definitely yes (low risk of bias)**	**Definitely yes (low risk of bias)**	**Definitely yes (low risk of bias)**	**Definitely yes (low risk of bias)**
**Babar-2021**	**Definitely yes (low risk of bias)**	**Probably no**	**Definitely no (high risk of bias)**	**Definitely yes (low risk of bias)**	**Probably yes**	**Definitely yes (low risk of bias)**
**Bachmann 2003**	**Probably yes**	**Probably yes**	**Probably no**	**Probably yes**	**Definitely yes (low risk of bias)**	**Probably yes**
**Bae-2021**	**Probably no**	**Definitely no (high risk of bias)**	**Definitely no (high risk of bias)**	**Probably yes**	**Probably yes**	**Definitely yes (low risk of bias)**
**Bailey 2010**	**Definitely yes (low risk of bias)**	**Definitely yes (low risk of bias)**	**Definitely yes (low risk of bias)**	**Definitely yes (low risk of bias)**	**Definitely yes (low risk of bias)**	**Probably yes**
**Bailey 2012**	**Definitely yes (low risk of bias)**	**Definitely yes (low risk of bias)**	**Definitely yes (low risk of bias)**	**Definitely yes (low risk of bias)**	**Definitely yes (low risk of bias)**	**Definitely yes (low risk of bias)**
**Bajaj 2014**	**Definitely yes (low risk of bias)**	**Definitely yes (low risk of bias)**	**Probably yes**	**Definitely yes (low risk of bias)**	**Definitely yes (low risk of bias)**	**Probably yes**
**Bakris 2006**	**Probably yes**	**Probably yes**	**Probably yes**	**Probably no**	**Definitely yes (low risk of bias)**	**Probably yes**
**BALANCE**	**Definitely yes (low risk of bias)**	**Probably no**	**Definitely yes (low risk of bias)**	**Probably no**	**Definitely yes (low risk of bias)**	**Probably yes**

Study ID	Domain 1	Domain 2	Domain 3	Domain 4	Domain 5	Domain 6
**Banerji 1995**	Definitely no (high risk of bias)	Definitely no (high risk of bias)	Probably yes	Definitely yes (low risk of bias)	Definitely yes (low risk of bias)	Probably no
**Barnett 2003**	Probably no	Probably no	Probably no	Probably yes	Definitely yes (low risk of bias)	Probably yes
**Barnett 2012**	Definitely yes (low risk of bias)	Definitely yes (low risk of bias)	Probably no	Probably yes	Definitely yes (low risk of bias)	Probably yes
**Barnett 2013**	Definitely yes (low risk of bias)	Definitely yes (low risk of bias)	Definitely yes (low risk of bias)	Definitely yes (low risk of bias)	Definitely yes (low risk of bias)	Definitely yes (low risk of bias)
**Barzilai 2011**	Definitely yes (low risk of bias)	Definitely yes (low risk of bias)	Definitely yes (low risk of bias)	Definitely yes (low risk of bias)	Definitely yes (low risk of bias)	Probably yes
**Bayrasheva 2020**	Definitely yes (low risk of bias)	Definitely no (high risk of bias)	Probably yes	Definitely yes (low risk of bias)	Definitely yes (low risk of bias)	Probably yes
**BEGIN: ADD TO GLP-1**	Probably yes	Probably yes	Definitely no (high risk of bias)	Definitely yes (low risk of bias)	Definitely yes (low risk of bias)	Probably yes
**BEGIN: VICTOZA ADD-ON**	Probably yes	Probably yes	Definitely no (high risk of bias)	Definitely yes (low risk of bias)	Definitely yes (low risk of bias)	Probably yes
**Berberoglu 2010**	Probably yes	Probably yes	Probably yes	Definitely yes (low risk of bias)	Definitely yes (low risk of bias)	Probably yes
**Bergenstal 2009**	Definitely yes (low risk of bias)	Definitely yes (low risk of bias)	Definitely no (high risk of bias)	Definitely yes (low risk of bias)	Definitely yes (low risk of bias)	Definitely yes (low risk of bias)
**Berndt-Zipfel 2013**	Probably yes	Probably yes	Definitely no (high risk of bias)	Definitely yes (low risk of bias)	Definitely yes (low risk of bias)	Probably yes
**BEST**	Probably no	Probably no	Definitely no (high risk of bias)	Probably yes	Definitely yes (low risk of bias)	Probably no
**BETA**	Probably no	Probably no	Definitely no (high risk of bias)	Probably no	Definitely yes (low risk of bias)	Probably no

Study ID	Domain 1	Domain 2	Domain 3	Domain 4	Domain 5	Domain 6
**BEYOND**	Definitely yes (low risk of bias)	Definitely no (high risk of bias)	Definitely no (high risk of bias)	Definitely no (high risk of bias)	Probably no	Definitely no (high risk of bias)
**Bi 2013**	Definitely yes (low risk of bias)	Definitely no (high risk of bias)	Definitely no (high risk of bias)	Probably yes	Definitely yes (low risk of bias)	Probably yes
**Bilezikian 2013**	Probably yes	Probably yes	Definitely yes (low risk of bias)	Definitely no (high risk of bias)	Definitely yes (low risk of bias)	Probably yes
**Birkeland 1994a**	Probably no	Probably no	Definitely no (high risk of bias)	Definitely yes (low risk of bias)	Definitely yes (low risk of bias)	Probably no
**Bode 2013**	Definitely yes (low risk of bias)	Definitely yes (low risk of bias)	Definitely yes (low risk of bias)	Definitely yes (low risk of bias)	Definitely yes (low risk of bias)	Definitely yes (low risk of bias)
**Bolinder 2012**	Probably yes	Probably yes	Definitely yes (low risk of bias)	Definitely yes (low risk of bias)	Definitely yes (low risk of bias)	Probably yes
**Bolli 2009**	Definitely yes (low risk of bias)	Definitely yes (low risk of bias)	Definitely yes (low risk of bias)	Definitely yes (low risk of bias)	Definitely yes (low risk of bias)	Probably yes
**Borges 2011**	Definitely yes (low risk of bias)	Probably yes	Definitely yes (low risk of bias)	Probably yes	Definitely yes (low risk of bias)	Definitely yes (low risk of bias)
**Bosi 2007**	Probably yes	Probably yes	Probably no	Definitely yes (low risk of bias)	Definitely yes (low risk of bias)	Probably yes
**Bosi 2009**	Probably yes	Probably yes	Probably yes	Probably yes	Definitely yes (low risk of bias)	Probably yes
**Bosi 2011**	Probably yes	Probably yes	Definitely yes (low risk of bias)	Definitely no (high risk of bias)	Definitely yes (low risk of bias)	Probably yes
**Bouchi 2017**	Definitely yes (low risk of bias)	Probably yes	Definitely no (high risk of bias)	Definitely yes (low risk of bias)	Definitely yes (low risk of bias)	Probably yes
**Braun 1996**	Probably no	Probably no	Definitely yes (low risk of bias)	Definitely yes (low risk of bias)	Definitely yes (low risk of bias)	Probably no

Study ID	Domain 1	Domain 2	Domain 3	Domain 4	Domain 5	Domain 6
**BRL-049653/334**	Definitely yes (low risk of bias)	Definitely yes (low risk of bias)	Definitely yes (low risk of bias)	Definitely yes (low risk of bias)	Definitely yes (low risk of bias)	Probably yes
**Bryson 2016**	Probably yes	Probably yes	Definitely yes (low risk of bias)	Definitely yes (low risk of bias)	Definitely yes (low risk of bias)	Probably yes
**Bunck 2009**	Definitely yes (low risk of bias)	Probably yes	Definitely no (high risk of bias)	Probably yes	Definitely yes (low risk of bias)	Probably no
**Buse 2011**	Definitely yes (low risk of bias)	Definitely yes (low risk of bias)	Definitely yes (low risk of bias)	Definitely yes (low risk of bias)	Definitely yes (low risk of bias)	Definitely yes (low risk of bias)
**Cai-2021**	Probably no	Probably no	Definitely no (high risk of bias)	Probably no	Definitely yes (low risk of bias)	Definitely yes (low risk of bias)
**Camerini-Davalos 1988**	Definitely yes (low risk of bias)	Definitely no (high risk of bias)	Definitely no (high risk of bias)	Definitely no (high risk of bias)	Definitely yes (low risk of bias)	Probably no
**Camerini-Davalos 1994**	Definitely yes (low risk of bias)	Probably yes	Definitely no (high risk of bias)	Probably yes	Definitely yes (low risk of bias)	Probably no
**Campbell 1994**	Probably no	Probably no	Definitely no (high risk of bias)	Definitely yes (low risk of bias)	Definitely yes (low risk of bias)	Probably no
**CANDLE**	Definitely yes (low risk of bias)	Definitely yes (low risk of bias)	Definitely no (high risk of bias)	Definitely yes (low risk of bias)	Definitely yes (low risk of bias)	Definitely yes (low risk of bias)
**CANTATA-D**	Definitely yes (low risk of bias)	Probably yes	Definitely yes (low risk of bias)	Definitely yes (low risk of bias)	Definitely yes (low risk of bias)	Probably yes
**CANTATA-D2**	Definitely yes (low risk of bias)	Probably yes	Definitely yes (low risk of bias)	Definitely yes (low risk of bias)	Definitely yes (low risk of bias)	Definitely yes (low risk of bias)
**CANTATA-M**	Probably yes	Probably yes	Definitely yes (low risk of bias)	Definitely yes (low risk of bias)	Definitely yes (low risk of bias)	Probably yes
**CANTATA-MSU**	Definitely yes (low risk of bias)	Definitely yes (low risk of bias)	Definitely yes (low risk of bias)	Definitely yes (low risk of bias)	Definitely yes (low risk of bias)	Definitely yes (low risk of bias)

Study ID	Domain 1	Domain 2	Domain 3	Domain 4	Domain 5	Domain 6
**CANTATA-SU**	Definitely yes (low risk of bias)	Definitely yes (low risk of bias)	Definitely yes (low risk of bias)	Definitely yes (low risk of bias)	Definitely yes (low risk of bias)	Probably yes
**CANVAS/CANVAS-R**	Definitely yes (low risk of bias)	Definitely yes (low risk of bias)	Probably no	Definitely yes (low risk of bias)	Definitely yes (low risk of bias)	Probably yes
**Carlson 2019**	Probably yes	Definitely no (high risk of bias)	Definitely no (high risk of bias)	Probably yes	Definitely yes (low risk of bias)	Probably yes
**CARMELINA**	Probably yes	Probably yes	Definitely yes (low risk of bias)	Definitely yes (low risk of bias)	Definitely yes (low risk of bias)	Definitely yes (low risk of bias)
**CAROLINA**	Definitely yes (low risk of bias)	Definitely yes (low risk of bias)	Definitely yes (low risk of bias)	Definitely yes (low risk of bias)	Definitely yes (low risk of bias)	Definitely yes (low risk of bias)
**Casner 1988**	Probably no	Probably no	Probably yes	Probably no	Definitely yes (low risk of bias)	Probably no
**Cefalu 2015**	Probably yes	Probably yes	Probably no	Definitely yes (low risk of bias)	Definitely yes (low risk of bias)	Probably yes
**Chacra 2017**	Probably yes	Probably yes	Definitely yes (low risk of bias)	Probably no	Definitely yes (low risk of bias)	Probably yes
**Chakraborty 2011**	Probably yes	Probably yes	Probably no	Probably no	Definitely yes (low risk of bias)	Probably yes
**Charbonnel 2013**	Definitely yes (low risk of bias)	Definitely yes (low risk of bias)	Definitely no (high risk of bias)	Definitely yes (low risk of bias)	Definitely yes (low risk of bias)	Probably yes
**Charpentier 2009**	Probably yes	Probably yes	Probably no	Definitely yes (low risk of bias)	Definitely yes (low risk of bias)	Probably yes
**Chehrehgosha-2021**	Definitely yes (low risk of bias)	Definitely yes (low risk of bias)	Definitely yes (low risk of bias)	Definitely yes (low risk of bias)	Definitely yes (low risk of bias)	Definitely yes (low risk of bias)
**Chen 2015**	Definitely yes (low risk of bias)	Definitely yes (low risk of bias)	Definitely yes (low risk of bias)	Definitely yes (low risk of bias)	Definitely yes (low risk of bias)	Definitely yes (low risk of bias)

Study ID	Domain 1	Domain 2	Domain 3	Domain 4	Domain 5	Domain 6
**Chen 2016**	**Definitely yes (low risk of bias)**	**Definitely no (high risk of bias)**	**Definitely no (high risk of bias)**	**Definitely yes (low risk of bias)**	**Definitely yes (low risk of bias)**	**Probably no**
**Cherney-2021**	**Probably no**	**Probably no**	**Definitely yes (low risk of bias)**	**Definitely yes (low risk of bias)**	**Definitely yes (low risk of bias)**	**Probably no**
**Chiasson 1994**	**Probably no**	**Probably no**	**Probably no**	**Probably yes**	**Definitely yes (low risk of bias)**	**Probably yes**
**Chiasson 2001**	**Probably no**	**Probably no**	**Probably no**	**Probably yes**	**Definitely yes (low risk of bias)**	**Probably no**
**CHICAGO**	**Probably yes**	**Probably yes**	**Definitely yes (low risk of bias)**	**Definitely yes (low risk of bias)**	**Definitely yes (low risk of bias)**	**Definitely yes (low risk of bias)**
**Chien 2011**	**Probably yes**	**Probably yes**	**Definitely no (high risk of bias)**	**Probably yes**	**Definitely yes (low risk of bias)**	**Probably yes**
**Cho 2019**	**Probably yes**	**Probably yes**	**Definitely no (high risk of bias)**	**Definitely yes (low risk of bias)**	**Definitely yes (low risk of bias)**	**Definitely yes (low risk of bias)**
**Choi 2004**	**Probably yes**	**Probably no**	**Probably no**	**Probably yes**	**Definitely yes (low risk of bias)**	**Definitely yes (low risk of bias)**
**Chou 2008**	**Definitely yes (low risk of bias)**	**Probably yes**	**Probably yes**	**Probably yes**	**Definitely yes (low risk of bias)**	**Definitely yes (low risk of bias)**
**Chou 2012**	**Probably yes**	**Probably yes**	**Definitely yes (low risk of bias)**	**Probably yes**	**Definitely yes (low risk of bias)**	**Definitely yes (low risk of bias)**
**Chuang-2020**	**Definitely yes (low risk of bias)**	**Definitely yes (low risk of bias)**	**Definitely no (high risk of bias)**	**Definitely yes (low risk of bias)**	**Probably yes**	**Probably yes**
**CIMT**	**Definitely yes (low risk of bias)**	**Definitely yes (low risk of bias)**	**Definitely yes (low risk of bias)**	**Probably no**	**Definitely yes (low risk of bias)**	**Definitely no (high risk of bias)**
**Civera 2008**	**Probably no**	**Probably no**	**Definitely no (high risk of bias)**	**Definitely yes (low risk of bias)**	**Definitely yes (low risk of bias)**	**Probably no**

Study ID	Domain 1	Domain 2	Domain 3	Domain 4	Domain 5	Domain 6
**COMPASS**	**Definitely yes (low risk of bias)**	**Definitely yes (low risk of bias)**	**Definitely no (high risk of bias)**	**Probably yes**	**Definitely yes (low risk of bias)**	**Definitely yes (low risk of bias)**
**CompoSIT-I**	**Definitely yes (low risk of bias)**	**Probably yes**	**Definitely yes (low risk of bias)**	**Definitely yes (low risk of bias)**	**Definitely yes (low risk of bias)**	**Definitely yes (low risk of bias)**
**CompoSIT-R**	**Definitely yes (low risk of bias)**	**Definitely yes (low risk of bias)**	**Definitely yes (low risk of bias)**	**Definitely yes (low risk of bias)**	**Definitely yes (low risk of bias)**	**Definitely yes (low risk of bias)**
**CONFIDENCE**	**Definitely yes (low risk of bias)**	**Definitely yes (low risk of bias)**	**Definitely no (high risk of bias)**	**Probably no**	**Definitely yes (low risk of bias)**	**Probably no**
**Coniff 1994**	**Probably yes**	**Probably no**	**Probably yes**	**Probably yes**	**Definitely yes (low risk of bias)**	**Probably no**
**Coniff 1995**	**Probably yes**	**Probably no**	**Probably yes**	**Probably no**	**Definitely yes (low risk of bias)**	**Probably no**
**Coniff 1995a**	**Probably yes**	**Probably yes**	**Probably yes**	**Definitely yes (low risk of bias)**	**Definitely yes (low risk of bias)**	**Probably yes**
**Costa 1997**	**Probably yes**	**Probably yes**	**Probably yes**	**Definitely no (high risk of bias)**	**Definitely yes (low risk of bias)**	**Definitely yes (low risk of bias)**
**CREDENCE**	**Definitely yes (low risk of bias)**	**Probably yes**	**Definitely yes (low risk of bias)**	**Definitely yes (low risk of bias)**	**Definitely yes (low risk of bias)**	**Definitely yes (low risk of bias)**
**Cusi 2019**	**Definitely yes (low risk of bias)**	**Probably yes**	**Definitely yes (low risk of bias)**	**Definitely yes (low risk of bias)**	**Definitely yes (low risk of bias)**	**Definitely yes (low risk of bias)**
**CV181-011**	**Probably yes**	**Probably yes**	**Definitely yes (low risk of bias)**	**Definitely no (high risk of bias)**	**Definitely yes (low risk of bias)**	**Definitely no (high risk of bias)**
**CV181-013**	**Definitely yes (low risk of bias)**	**Probably yes**	**Probably yes**	**Definitely no (high risk of bias)**	**Definitely yes (low risk of bias)**	**Definitely yes (low risk of bias)**
**CV181-039**	**Probably yes**	**Probably yes**	**Probably yes**	**Definitely no (high risk of bias)**	**Definitely yes (low risk of bias)**	**Definitely no (high risk of bias)**

Study ID	Domain 1	Domain 2	Domain 3	Domain 4	Domain 5	Domain 6
**da Silva 2016**	Probably yes	Probably no	Definitely no (high risk of bias)	Probably yes	Definitely yes (low risk of bias)	Definitely yes (low risk of bias)
**Dailey 2004**	Probably yes	Probably yes	Probably yes	Definitely no (high risk of bias)	Definitely yes (low risk of bias)	Definitely yes (low risk of bias)
**Dapagliflozin 006**	Definitely yes (low risk of bias)	Definitely yes (low risk of bias)	Definitely yes (low risk of bias)	Probably yes	Definitely yes (low risk of bias)	Probably yes
**DAPA-LVH**	Definitely yes (low risk of bias)	Definitely yes (low risk of bias)	Definitely yes (low risk of bias)	Probably no	Definitely yes (low risk of bias)	Probably no
**Dargie 2007**	Definitely yes (low risk of bias)	Probably yes	Probably yes	Definitely no (high risk of bias)	Definitely yes (low risk of bias)	Probably no
**Davidson 2007**	Definitely yes (low risk of bias)	Definitely yes (low risk of bias)	Probably yes	Definitely no (high risk of bias)	Definitely yes (low risk of bias)	Definitely yes (low risk of bias)
**Davies 2013**	Definitely yes (low risk of bias)	Definitely yes (low risk of bias)	Definitely no (high risk of bias)	Probably no	Definitely yes (low risk of bias)	Definitely yes (low risk of bias)
**Davies 2017**	Definitely yes (low risk of bias)	Definitely yes (low risk of bias)	Definitely yes (low risk of bias)	Definitely yes (low risk of bias)	Definitely yes (low risk of bias)	Probably yes
**DECLARE-TIMI 58**	Probably yes	Probably yes	Definitely yes (low risk of bias)	Probably no	Definitely yes (low risk of bias)	Probably yes
**DeFronzo 1995**	Probably yes	Probably yes	Probably yes	Definitely no (high risk of bias)	Definitely yes (low risk of bias)	Definitely yes (low risk of bias)
**DeFronzo 2005**	Probably yes	Probably yes	Probably yes	Definitely no (high risk of bias)	Definitely yes (low risk of bias)	Definitely yes (low risk of bias)
**DeFronzo 2012**	Probably yes	Probably yes	Probably yes	Definitely yes (low risk of bias)	Definitely yes (low risk of bias)	Definitely yes (low risk of bias)
**DeFronzo 2015**	Probably yes	Definitely yes (low risk of bias)	Probably yes	Probably yes	Definitely yes (low risk of bias)	Definitely yes (low risk of bias)

Study ID	Domain 1	Domain 2	Domain 3	Domain 4	Domain 5	Domain 6
Dei Cas 2017	Definitely yes (low risk of bias)	Definitely yes (low risk of bias)	Definitely no (high risk of bias)	Probably no	Definitely yes (low risk of bias)	Definitely yes (low risk of bias)
Dejager 2007	Probably yes	Probably yes	Probably yes	Definitely no (high risk of bias)	Definitely yes (low risk of bias)	Definitely yes (low risk of bias)
del Prato 2003	Probably yes	Probably yes	Probably yes	Definitely no (high risk of bias)	Definitely yes (low risk of bias)	Definitely yes (low risk of bias)
del Prato 2011	Definitely yes (low risk of bias)	Definitely yes (low risk of bias)	Probably yes	Definitely yes (low risk of bias)	Definitely yes (low risk of bias)	Definitely yes (low risk of bias)
Del Prato 2014	Probably yes	Probably yes	Definitely yes (low risk of bias)	Definitely no (high risk of bias)	Definitely yes (low risk of bias)	Definitely yes (low risk of bias)
DELIGHT	Definitely yes (low risk of bias)	Definitely yes (low risk of bias)	Definitely yes (low risk of bias)	Definitely yes (low risk of bias)	Definitely yes (low risk of bias)	Probably yes
Deng 2017	Definitely yes (low risk of bias)	Probably no	Probably no	Definitely yes (low risk of bias)	Definitely yes (low risk of bias)	Probably no
DERIVE	Definitely yes (low risk of bias)	Probably yes	Definitely yes (low risk of bias)	Definitely yes (low risk of bias)	Definitely yes (low risk of bias)	Definitely yes (low risk of bias)
Derosa 2003	Probably yes	Probably no	Probably no	Definitely yes (low risk of bias)	Definitely yes (low risk of bias)	Definitely yes (low risk of bias)
Derosa 2003a	Probably yes	Probably no	Probably no	Definitely yes (low risk of bias)	Definitely yes (low risk of bias)	Definitely yes (low risk of bias)
Derosa 2004	Probably yes	Probably no	Probably no	Definitely yes (low risk of bias)	Definitely yes (low risk of bias)	Definitely yes (low risk of bias)
Derosa 2005	Probably yes	Probably no	Probably yes	Definitely yes (low risk of bias)	Definitely yes (low risk of bias)	Probably no
Derosa 2007	Probably yes	Probably no	Probably yes	Definitely yes (low risk of bias)	Definitely yes (low risk of bias)	Definitely yes (low risk of bias)

Study ID	Domain 1	Domain 2	Domain 3	Domain 4	Domain 5	Domain 6
**Derosa 2009**	Probably yes	Probably no	Probably yes	Definitely no (high risk of bias)	Definitely yes (low risk of bias)	Definitely yes (low risk of bias)
**Derosa 2009a**	Probably yes	Probably no	Probably yes	Probably yes	Definitely yes (low risk of bias)	Probably yes
**Derosa 2010**	Probably yes	Probably no	Probably no	Definitely yes (low risk of bias)	Definitely yes (low risk of bias)	Definitely yes (low risk of bias)
**Derosa 2010a**	Probably yes	Probably no	Definitely no (high risk of bias)	Definitely yes (low risk of bias)	Definitely yes (low risk of bias)	Definitely yes (low risk of bias)
**Derosa 2011**	Probably yes	Probably no	Definitely no (high risk of bias)	Definitely yes (low risk of bias)	Definitely yes (low risk of bias)	Definitely yes (low risk of bias)
**Derosa 2012**	Probably yes	Probably no	Probably yes	Definitely yes (low risk of bias)	Definitely yes (low risk of bias)	Definitely yes (low risk of bias)
**Derosa 2012a**	Probably yes	Probably no	Probably yes	Definitely yes (low risk of bias)	Definitely yes (low risk of bias)	Probably yes
**Derosa 2013**	Probably yes	Probably no	Probably yes	Definitely yes (low risk of bias)	Definitely yes (low risk of bias)	Definitely yes (low risk of bias)
**Derosa 2014**	Probably yes	Probably no	Probably yes	Definitely yes (low risk of bias)	Definitely yes (low risk of bias)	Definitely yes (low risk of bias)
**Derosa 2014a**	Probably yes	Probably no	Probably yes	Definitely yes (low risk of bias)	Definitely yes (low risk of bias)	Probably yes
**DIA3004**	Probably yes	Probably yes	Definitely yes (low risk of bias)	Probably yes	Definitely yes (low risk of bias)	Definitely yes (low risk of bias)
**Distiller 2014**	Probably yes	Probably no	Probably no	Probably yes	Definitely yes (low risk of bias)	Probably no
**DIVERSITY-CVR**	Definitely yes (low risk of bias)	Definitely yes (low risk of bias)	Probably no	Definitely yes (low risk of bias)	Definitely yes (low risk of bias)	Definitely yes (low risk of bias)

Study ID	Domain 1	Domain 2	Domain 3	Domain 4	Domain 5	Domain 6
D-LIFT	Definitely yes (low risk of bias)	Probably yes	Probably no	Probably no	Definitely yes (low risk of bias)	Definitely yes (low risk of bias)
Dobs 2013	Definitely yes (low risk of bias)	Probably yes	Definitely yes (low risk of bias)	Probably no	Definitely yes (low risk of bias)	Probably no
Dorkhan 2009	Probably yes	Probably no	Probably no	Definitely yes (low risk of bias)	Definitely yes (low risk of bias)	Definitely yes (low risk of bias)
Douek 2005	Probably yes	Probably yes	Definitely yes (low risk of bias)	Probably no	Definitely yes (low risk of bias)	Definitely yes (low risk of bias)
Drent 2002	Definitely yes (low risk of bias)	Probably yes	Probably yes	Definitely no (high risk of bias)	Definitely yes (low risk of bias)	Probably yes
DUAL-I	Definitely yes (low risk of bias)	Definitely yes (low risk of bias)	Probably no	Definitely yes (low risk of bias)	Definitely yes (low risk of bias)	Definitely yes (low risk of bias)
DUAL-I JAPAN	Definitely yes (low risk of bias)	Definitely yes (low risk of bias)	Probably no	Definitely yes (low risk of bias)	Definitely yes (low risk of bias)	Definitely yes (low risk of bias)
DUAL-IX	Definitely yes (low risk of bias)	Definitely yes (low risk of bias)	Probably no	Definitely yes (low risk of bias)	Definitely yes (low risk of bias)	Definitely yes (low risk of bias)
DUAL-VIII	Definitely yes (low risk of bias)	Definitely yes (low risk of bias)	Probably no	Definitely yes (low risk of bias)	Definitely yes (low risk of bias)	Definitely yes (low risk of bias)
DURATION-2	Definitely yes (low risk of bias)	Definitely yes (low risk of bias)	Definitely yes (low risk of bias)	Definitely no (high risk of bias)	Definitely yes (low risk of bias)	Definitely yes (low risk of bias)
DURATION-3	Definitely yes (low risk of bias)	Definitely yes (low risk of bias)	Probably no	Definitely yes (low risk of bias)	Definitely yes (low risk of bias)	Definitely yes (low risk of bias)
DURATION-4	Definitely yes (low risk of bias)	Definitely yes (low risk of bias)	Definitely yes (low risk of bias)	Probably yes	Definitely yes (low risk of bias)	Definitely yes (low risk of bias)
DURATION-7	Probably yes	Definitely yes (low risk of bias)	Definitely yes (low risk of bias)	Definitely yes (low risk of bias)	Definitely yes (low risk of bias)	Definitely yes (low risk of bias)

Study ID	Domain 1	Domain 2	Domain 3	Domain 4	Domain 5	Domain 6
**DURATION-8**	**Definitely yes (low risk of bias)**	**Definitely yes (low risk of bias)**	**Definitely yes (low risk of bias)**	**Definitely yes (low risk of bias)**	**Definitely yes (low risk of bias)**	**Definitely yes (low risk of bias)**
**DURATION-NEO-2**	**Definitely yes (low risk of bias)**	**Definitely yes (low risk of bias)**	**Probably no**	**Probably yes**	**Definitely yes (low risk of bias)**	**Definitely yes (low risk of bias)**
**EAGLE**	**Probably yes**	**Definitely yes (low risk of bias)**	**Probably no**	**Probably no**	**Definitely yes (low risk of bias)**	**Definitely yes (low risk of bias)**
**EASIE**	**Definitely yes (low risk of bias)**	**Definitely yes (low risk of bias)**	**Probably no**	**Definitely yes (low risk of bias)**	**Definitely yes (low risk of bias)**	**Definitely yes (low risk of bias)**
**Ebato 2009**	**Probably yes**	**Probably no**	**Probably no**	**Probably yes**	**Definitely yes (low risk of bias)**	**Definitely yes (low risk of bias)**
**EDIT**	**Definitely yes (low risk of bias)**	**Probably no**	**Probably no**	**Definitely yes (low risk of bias)**	**Definitely yes (low risk of bias)**	**Probably yes**
**ELAID study**	**Definitely yes (low risk of bias)**	**Probably yes**	**Definitely yes (low risk of bias)**	**Definitely yes (low risk of bias)**	**Probably yes**	**Definitely yes (low risk of bias)**
**ELEGANT**	**Definitely yes (low risk of bias)**	**Probably no**	**Probably no**	**Definitely yes (low risk of bias)**	**Definitely yes (low risk of bias)**	**Definitely yes (low risk of bias)**
**ELITE**	**Definitely yes (low risk of bias)**	**Definitely yes (low risk of bias)**	**Definitely no (high risk of bias)**	**Definitely yes (low risk of bias)**	**Definitely yes (low risk of bias)**	**Definitely yes (low risk of bias)**
**ELIXA**	**Probably yes**	**Definitely yes (low risk of bias)**	**Definitely yes (low risk of bias)**	**Probably yes**	**Definitely yes (low risk of bias)**	**Definitely yes (low risk of bias)**
**ELLENA-IT**	**Definitely yes (low risk of bias)**	**Probably yes**	**Probably no**	**Definitely yes (low risk of bias)**	**Definitely yes (low risk of bias)**	**Definitely yes (low risk of bias)**
**EMBLEM**	**Definitely yes (low risk of bias)**	**Definitely yes (low risk of bias)**	**Definitely yes (low risk of bias)**	**Probably yes**	**Definitely yes (low risk of bias)**	**Definitely yes (low risk of bias)**
**EMBODY**	**Definitely yes (low risk of bias)**	**Probably no**	**Definitely yes (low risk of bias)**	**Definitely yes (low risk of bias)**	**Definitely yes (low risk of bias)**	**Definitely no (high risk of bias)**

Study ID	Domain 1	Domain 2	Domain 3	Domain 4	Domain 5	Domain 6
EMIT	Probably yes	Definitely yes (low risk of bias)	Definitely yes (low risk of bias)	Probably no	Definitely yes (low risk of bias)	Probably yes
EMLIFA001	Definitely yes (low risk of bias)	Definitely yes (low risk of bias)	Definitely yes (low risk of bias)	Definitely no (high risk of bias)	Definitely yes (low risk of bias)	Definitely yes (low risk of bias)
EMPA-HEART	Definitely yes (low risk of bias)	Definitely yes (low risk of bias)	Probably no	Definitely yes (low risk of bias)	Probably yes	Definitely yes (low risk of bias)
EMPA-HEART CardioLink-6	Definitely yes (low risk of bias)	Definitely yes (low risk of bias)	Definitely yes (low risk of bias)	Definitely yes (low risk of bias)	Definitely yes (low risk of bias)	Definitely yes (low risk of bias)
EMPA-REG BASAL	Probably yes	Definitely yes (low risk of bias)	Probably yes	Probably yes	Definitely yes (low risk of bias)	Probably yes
EMPA-REG H2H-SU	Definitely yes (low risk of bias)	Definitely yes (low risk of bias)	Definitely yes (low risk of bias)	Probably yes	Definitely yes (low risk of bias)	Definitely yes (low risk of bias)
EMPA-REG MDI	Probably yes	Definitely yes (low risk of bias)	Probably yes	Probably yes	Definitely yes (low risk of bias)	Definitely yes (low risk of bias)
EMPA-REG MET	Probably yes	Definitely yes (low risk of bias)	Probably yes	Definitely yes (low risk of bias)	Definitely yes (low risk of bias)	Definitely yes (low risk of bias)
EMPA-REG METSU	Probably yes	Definitely yes (low risk of bias)	Probably yes	Definitely yes (low risk of bias)	Definitely yes (low risk of bias)	Definitely yes (low risk of bias)
EMPA-REG MONO	Definitely yes (low risk of bias)	Definitely yes (low risk of bias)	Definitely yes (low risk of bias)	Definitely yes (low risk of bias)	Definitely yes (low risk of bias)	Definitely yes (low risk of bias)
EMPA-REG OUTCOME	Definitely yes (low risk of bias)	Definitely yes (low risk of bias)	Probably yes	Definitely yes (low risk of bias)	Definitely yes (low risk of bias)	Definitely yes (low risk of bias)
EMPA-REG PIO	Definitely yes (low risk of bias)	Definitely yes (low risk of bias)	Probably yes	Definitely yes (low risk of bias)	Definitely yes (low risk of bias)	Definitely yes (low risk of bias)
EMPA-REG RENAL	Definitely yes (low risk of bias)	Definitely yes (low risk of bias)	Definitely yes (low risk of bias)	Definitely yes (low risk of bias)	Definitely yes (low risk of bias)	Definitely yes (low risk of bias)

Study ID	Domain 1	Domain 2	Domain 3	Domain 4	Domain 5	Domain 6
**Engelbrechtsen 2016**	Probably yes	Probably no	Probably no	Probably yes	Definitely yes (low risk of bias)	Definitely yes (low risk of bias)
**Erem 2014**	Probably yes	Probably no	Probably no	Definitely yes (low risk of bias)	Definitely yes (low risk of bias)	Definitely yes (low risk of bias)
**ESAX-DN**	Definitely yes (low risk of bias)	Definitely yes (low risk of bias)	Definitely yes (low risk of bias)	Definitely yes (low risk of bias)	Definitely yes (low risk of bias)	Definitely yes (low risk of bias)
**ESPECIAL-ACS**	Probably yes	Probably no	Probably no	Definitely no (high risk of bias)	Definitely yes (low risk of bias)	Definitely yes (low risk of bias)
**Esposito 2004**	Definitely yes (low risk of bias)	Probably no	Probably no	Definitely yes (low risk of bias)	Definitely yes (low risk of bias)	Definitely yes (low risk of bias)
**Esposito 2011**	Definitely yes (low risk of bias)	Probably no	Definitely yes (low risk of bias)	Definitely yes (low risk of bias)	Definitely yes (low risk of bias)	Definitely yes (low risk of bias)
**Essen Study**	Definitely yes (low risk of bias)	Probably no	Probably no	Probably no	Definitely yes (low risk of bias)	Definitely yes (low risk of bias)
**EUREXA**	Definitely yes (low risk of bias)	Probably no	Probably no	Definitely no (high risk of bias)	Definitely yes (low risk of bias)	Definitely yes (low risk of bias)
**EUREXA extension**	Probably yes	Probably yes	Probably no	Probably no	Probably yes	Probably yes
**EXAMINE**	Probably yes	Probably yes	Definitely yes (low risk of bias)	Definitely yes (low risk of bias)	Definitely yes (low risk of bias)	Definitely yes (low risk of bias)
**Exenatide-113**	Probably yes	Probably yes	Probably no	Probably yes	Probably yes	Probably no
**EXENDA**	Definitely yes (low risk of bias)	Definitely yes (low risk of bias)	Definitely yes (low risk of bias)	Definitely yes (low risk of bias)	Definitely yes (low risk of bias)	Definitely yes (low risk of bias)
**EXEPUMP**	Definitely yes (low risk of bias)	Probably no	Definitely yes (low risk of bias)	Definitely no (high risk of bias)	Definitely no (high risk of bias)	Definitely no (high risk of bias)
**EXSCEL**	Definitely yes (low risk of bias)	Probably yes	Probably yes	Definitely yes (low risk of bias)	Definitely yes (low risk of bias)	Definitely yes (low risk of bias)

Study ID	Domain 1	Domain 2	Domain 3	Domain 4	Domain 5	Domain 6
**Feng 2017**	**Definitely yes (low risk of bias)**	**Definitely yes (low risk of bias)**	**Definitely no (high risk of bias)**	**Probably no**	**Probably yes**	**Probably no**
**Feng-2020**	**Probably no**	**Probably no**	**Definitely no (high risk of bias)**	**Definitely yes (low risk of bias)**	**Probably no**	**Probably no**
**Ferdinand 2019**	**Probably yes**	**Probably yes**	**Definitely no (high risk of bias)**	**Probably yes**	**Probably no**	**Probably yes**
**Fernandez 2008**	**Probably yes**	**Probably yes**	**Probably yes**	**Probably yes**	**Probably no**	**Probably no**
**Ferrannini 2010**	**Probably yes**	**Probably yes**	**Probably yes**	**Probably yes**	**Probably no**	**Probably yes**
**FIDELIO-DKD**	**Definitely yes (low risk of bias)**	**Definitely yes (low risk of bias)**	**Definitely yes (low risk of bias)**	**Definitely yes (low risk of bias)**	**Definitely yes (low risk of bias)**	**Definitely yes (low risk of bias)**
**FIGARO-DKD**	**Definitely yes (low risk of bias)**	**Definitely yes (low risk of bias)**	**Definitely yes (low risk of bias)**	**Definitely yes (low risk of bias)**	**Definitely yes (low risk of bias)**	**Definitely yes (low risk of bias)**
**Filozof 2010**	**Probably yes**	**Probably yes**	**Probably yes**	**Probably yes**	**Probably yes**	**Probably no**
**Finn 2009**	**Definitely yes (low risk of bias)**	**Probably yes**	**Probably yes**	**Probably yes**	**Probably yes**	**Probably yes**
**Fischer 1998**	**Probably yes**	**Probably yes**	**Probably yes**	**Probably yes**	**Probably no**	**Probably no**
**Foley 2009**	**Probably yes**	**Probably yes**	**Probably yes**	**Probably yes**	**Probably yes**	**Probably yes**
**Foley 2011**	**Probably yes**	**Probably yes**	**Probably yes**	**Probably yes**	**Probably yes**	**Probably yes**
**Fonseca 2000**	**Definitely yes (low risk of bias)**	**Definitely yes (low risk of bias)**	**Probably yes**	**Definitely yes (low risk of bias)**	**Probably yes**	**Probably yes**
**Fonseca 2003**	**Probably yes**	**Probably yes**	**Probably yes**	**Probably no**	**Probably yes**	**Probably yes**
**Fonseca 2007**	**Probably yes**	**Probably yes**	**Probably yes**	**Probably yes**	**Probably yes**	**Probably yes**
**Fonseca 2013**	**Probably yes**	**Probably yes**	**Probably yes**	**Probably yes**	**Probably yes**	**Probably yes**
**Forst 2003**	**Probably yes**	**Probably no**	**Definitely no (high risk of bias)**	**Probably no**	**Probably yes**	**Probably no**
**Forst 2014**	**Probably yes**	**Probably yes**	**Probably yes**	**Probably yes**	**Probably yes**	**Probably yes**

Study ID	Domain 1	Domain 2	Domain 3	Domain 4	Domain 5	Domain 6
Forst 2015	Probably yes	Probably yes	Definitely no (high risk of bias)	Probably no	Probably no	Probably no
Frederich 2012	Probably yes	Probably yes	Probably yes	Probably yes	Probably yes	Probably yes
FREEDOM CVO	Definitely yes (low risk of bias)	Probably no	Definitely yes (low risk of bias)	Probably yes	Definitely yes (low risk of bias)	Definitely yes (low risk of bias)
FREEDOM-1	Probably yes	Probably yes	Probably yes	Probably yes	Probably yes	Probably yes
Frias-2018	Definitely yes (low risk of bias)	Definitely yes (low risk of bias)	Definitely yes (low risk of bias)	Probably no	Definitely yes (low risk of bias)	Definitely yes (low risk of bias)
Gaal 2001	Probably yes	Probably yes	Probably yes	Probably yes	Probably yes	Probably yes
Gallwitz 2011	Probably yes	Probably yes	Definitely no (high risk of bias)	Probably no	Probably yes	Probably no
Gallwitz 2012	Definitely yes (low risk of bias)	Definitely yes (low risk of bias)	Probably yes	Probably yes	Probably yes	Probably yes
Gantz 2017	Definitely yes (low risk of bias)	Definitely yes (low risk of bias)	Probably yes	Probably yes	Probably yes	Probably no
Gantz 2017a	Definitely yes (low risk of bias)	Definitely yes (low risk of bias)	Probably yes	Probably yes	Probably yes	Definitely no (high risk of bias)
Gao-2020	Definitely yes (low risk of bias)	Definitely yes (low risk of bias)	Definitely yes (low risk of bias)	Definitely yes (low risk of bias)	Definitely yes (low risk of bias)	Definitely yes (low risk of bias)
Garber 2006	Probably yes	Probably yes	Probably yes	Probably yes	Probably yes	Probably yes
Garber 2007	Probably yes	Probably yes	Probably yes	Probably yes	Probably yes	Probably yes
Garber 2008	Probably yes	Probably yes	Probably yes	Probably yes	Probably yes	Probably yes
Gastaldelli 2014	Probably yes	Probably yes	Probably yes	Probably no	Probably yes	Probably no
GENERATION	Definitely yes (low risk of bias)	Definitely yes (low risk of bias)	Probably yes	Probably yes	Probably yes	Probably yes
Gentile 2001	Probably yes	Probably yes	Probably yes	Probably no	Probably yes	Probably no

Study ID	Domain 1	Domain 2	Domain 3	Domain 4	Domain 5	Domain 6
**GetGoal-Duo-1**	Probably yes	Probably yes	Probably yes	Probably yes	Probably yes	Probably yes
**GetGoal-Duo-2**	Probably yes	Probably yes	Definitely no (high risk of bias)	Definitely yes (low risk of bias)	Probably yes	Probably yes
**GetGoal-F1**	Probably yes	Probably yes	Probably yes	Definitely yes (low risk of bias)	Probably yes	Probably yes
**GetGoal-L**	Definitely yes (low risk of bias)	Definitely yes (low risk of bias)	Probably yes	Probably yes	Probably yes	Probably yes
**GetGoal-L Asia**	Probably yes	Probably yes	Probably yes	Probably yes	Probably yes	Probably yes
**GetGoal-L-C**	Definitely yes (low risk of bias)	Definitely yes (low risk of bias)	Probably yes	Definitely yes (low risk of bias)	Probably yes	Probably yes
**GetGoal-M**	Probably yes	Probably yes	Probably yes	Probably yes	Probably yes	Probably yes
**GetGoal-M-Asia**	Probably yes	Probably yes	Probably yes	Definitely yes (low risk of bias)	Probably yes	Probably yes
**GetGoal-O**	Definitely yes (low risk of bias)	Definitely yes (low risk of bias)	Probably yes	Probably yes	Probably yes	Probably yes
**GetGoal-P**	Definitely yes (low risk of bias)	Definitely yes (low risk of bias)	Probably yes	Probably yes	Probably yes	Probably yes
**GetGoal-S**	Probably yes	Probably yes	Probably yes	Probably yes	Probably yes	Probably yes
**Giles 2008**	Probably yes	Probably yes	Probably yes	Probably no	Probably yes	Probably no
**Giugliano 1993**	Probably yes	Probably yes	Probably yes	Probably yes	Probably yes	Probably no
**GLAC**	Probably yes	Probably yes	Probably no	Probably yes	Probably yes	Probably yes
**GLAD**	Definitely yes (low risk of bias)	Definitely yes (low risk of bias)	Probably yes	Probably yes	Probably yes	Probably yes
**GLAL**	Probably yes	Probably yes	Probably yes	Probably no	Probably yes	Probably yes
**Glimepiride Combination Group 1998**	Probably yes	Probably yes	Probably yes	Probably yes	Probably yes	Probably yes

Study ID	Domain 1	Domain 2	Domain 3	Domain 4	Domain 5	Domain 6
Göke 2002	Definitely yes (low risk of bias)	Definitely yes (low risk of bias)	Definitely no (high risk of bias)	Probably yes	Probably yes	Probably yes
Göke 2010	Definitely yes (low risk of bias)	Definitely yes (low risk of bias)	Probably yes	Probably yes	Probably yes	Probably yes
Gomez-Perez 2002	Probably yes	Probably yes	Probably yes	Probably yes	Probably yes	Probably yes
Gomis 2011	Definitely yes (low risk of bias)	Definitely yes (low risk of bias)	Probably yes	Probably yes	Probably yes	Probably yes
Goodman 2009	Probably yes	Probably yes	Probably yes	Probably yes	Probably yes	Probably yes
GRADE study	Definitely yes (low risk of bias)	Definitely yes (low risk of bias)	Definitely yes (low risk of bias)	Definitely yes (low risk of bias)	Definitely yes (low risk of bias)	Definitely yes (low risk of bias)
Grant 1996	Probably yes	Probably yes	Probably yes	Probably no	Probably yes	Probably no
GRAVITAS	Definitely yes (low risk of bias)	Definitely yes (low risk of bias)	Definitely yes (low risk of bias)	Probably yes	Probably yes	Probably yes
Grey 2014	Definitely yes (low risk of bias)	Definitely yes (low risk of bias)	Probably yes	Definitely yes (low risk of bias)	Probably yes	Probably yes
Gudipaty 2014	Probably yes	Probably yes	Definitely no (high risk of bias)	Probably yes	Probably yes	Probably no
Guo-2020	Definitely yes (low risk of bias)	Definitely no (high risk of bias)	Definitely no (high risk of bias)	Definitely yes (low risk of bias)	Definitely yes (low risk of bias)	Definitely yes (low risk of bias)
Gurkan 2014	Probably yes	Probably yes	Definitely no (high risk of bias)	Probably no	Probably yes	Probably yes
Gutniak 1987	Probably yes	Probably yes	Probably yes	Probably yes	Probably yes	Probably no
Guvener 1999	Probably yes	Probably yes	Probably yes	Probably yes	Probably yes	Probably no
Guzman 2017	Definitely yes (low risk of bias)	Definitely yes (low risk of bias)	Definitely yes (low risk of bias)	Probably no	Probably yes	Probably no
Haak 2012	Probably yes	Probably yes	Probably yes	Probably yes	Probably yes	Probably yes

Study ID	Domain 1	Domain 2	Domain 3	Domain 4	Domain 5	Domain 6
**Hadjadj 2016**	Definitely yes (low risk of bias)	Definitely yes (low risk of bias)	Probably yes	Probably yes	Probably yes	Probably yes
**Halimi 2000**	Probably yes	Probably yes	Probably yes	Probably yes	Probably yes	Probably no
**Hällsten 2002**	Probably yes	Probably yes	Probably yes	Probably yes	Probably yes	Probably no
**Halvorsen 2019**	Probably yes	Probably yes	Probably yes	Probably yes	Probably yes	Probably yes
**Halvorsen 2019a**	Probably yes	Probably yes	Probably yes	Definitely yes (low risk of bias)	Probably yes	Probably yes
**Han 2018**	Definitely yes (low risk of bias)	Definitely yes (low risk of bias)	Probably yes	Probably yes	Probably yes	Probably yes
**Han-2020**	Definitely yes (low risk of bias)	Definitely no (high risk of bias)	Definitely no (high risk of bias)	Definitely yes (low risk of bias)	Definitely yes (low risk of bias)	Definitely yes (low risk of bias)
**Han-2021**	Definitely yes (low risk of bias)	Probably no	Definitely yes (low risk of bias)	Definitely no (high risk of bias)	Definitely yes (low risk of bias)	Probably no
**Handelsman 2017**	Definitely yes (low risk of bias)	Definitely yes (low risk of bias)	Probably yes	Probably yes	Probably yes	Probably yes
**Handelsman 2019**	Definitely yes (low risk of bias)	Definitely yes (low risk of bias)	Probably yes	Definitely yes (low risk of bias)	Probably yes	Probably yes
**Haneda 2016**	Probably yes	Probably yes	Probably yes	Probably yes	Probably yes	Probably yes
**Hanefeld 1991**	Probably no	Probably no	Probably no	Probably yes	Probably yes	Probably no
**Hanefeld 2004**	Probably yes	Probably yes	Probably yes	Probably yes	Probably yes	Probably yes
**Hanefeld 2007**	Probably yes	Probably yes	Probably yes	Probably yes	Probably yes	Probably yes
**Hao-2022**	Definitely yes (low risk of bias)	Definitely no (high risk of bias)	Definitely no (high risk of bias)	Definitely no (high risk of bias)	Probably yes	Definitely yes (low risk of bias)
**HARMONY 1**	Definitely yes (low risk of bias)	Definitely yes (low risk of bias)	Probably yes	Definitely yes (low risk of bias)	Probably yes	Probably yes

Study ID	Domain 1	Domain 2	Domain 3	Domain 4	Domain 5	Domain 6
**HARMONY 2**	Definitely yes (low risk of bias)	Definitely yes (low risk of bias)	Probably yes	Probably yes	Probably yes	Probably yes
**HARMONY 3**	Probably yes	Probably yes	Probably yes	Probably yes	Probably yes	Probably yes
**HARMONY 4**	Probably yes	Probably yes	Definitely no (high risk of bias)	Probably yes	Probably yes	Probably no
**HARMONY 5**	Probably yes	Probably yes	Probably yes	Probably yes	Probably yes	Probably yes
**HARMONY 6**	Probably yes	Probably yes	Definitely no (high risk of bias)	Probably yes	Probably yes	Probably yes
**HARMONY OUTCOMES**	Definitely yes (low risk of bias)	Definitely yes (low risk of bias)	Probably yes	Probably yes	Probably yes	Probably yes
**Hartemann-Heurtier 2009**	Probably yes	Probably yes	Definitely no (high risk of bias)	Definitely yes (low risk of bias)	Probably yes	Probably no
**Hartley 2015**	Definitely yes (low risk of bias)	Definitely yes (low risk of bias)	Probably yes	Probably yes	Probably yes	Probably yes
**Hasche 1999**	Probably yes	Probably yes	Probably yes	Probably yes	Probably yes	Probably no
**Hattori 2017**	Probably yes	Probably yes	Definitely no (high risk of bias)	Probably yes	Probably yes	Probably no
**Hattori 2018**	Probably yes	Probably yes	Definitely no (high risk of bias)	Probably yes	Probably no	Probably no
**Hattori-2021**	Probably no	Definitely no (high risk of bias)	Definitely no (high risk of bias)	Probably no	Definitely yes (low risk of bias)	Probably no
**HEELA**	Probably yes	Probably yes	Definitely no (high risk of bias)	Probably yes	Probably yes	Probably yes
**Hegele 1995**	Probably no	Probably no	Probably yes	Probably yes	Probably yes	Probably no
**Heine 2005**	Probably yes	Probably yes	Definitely no (high risk of bias)	Probably yes	Probably yes	Probably yes

Study ID	Domain 1	Domain 2	Domain 3	Domain 4	Domain 5	Domain 6
**Heise-2022**	Definitely yes (low risk of bias)	Definitely yes (low risk of bias)	Definitely yes (low risk of bias)	Definitely yes (low risk of bias)	Definitely yes (low risk of bias)	Definitely yes (low risk of bias)
**Heliövaara 2007**	Probably yes	Probably yes	Probably yes	Probably yes	Probably no	Probably no
**Henry 2012**	Definitely yes (low risk of bias)	Definitely yes (low risk of bias)	Probably yes	Probably yes	Probably yes	Probably yes
**Henry 2014**	Probably yes	Probably yes	Probably yes	Probably yes	Probably yes	Probably no
**Hermann 1999**	Probably yes	Probably no	Probably yes	Probably yes	Probably yes	Probably no
**Hiramatsu 2018**	Probably yes	Probably yes	Probably no	Probably yes	Probably no	Probably no
**Hirano 2009**	Definitely yes (low risk of bias)	Definitely yes (low risk of bias)	Probably no	Definitely yes (low risk of bias)	Probably yes	Probably yes
**Hirano 2012**	Definitely yes (low risk of bias)	Definitely yes (low risk of bias)	Probably no	Definitely yes (low risk of bias)	Probably yes	Probably yes
**Hollander 2007**	Definitely yes (low risk of bias)	Probably yes	Definitely yes (low risk of bias)	Definitely no (high risk of bias)	Probably yes	Definitely yes (low risk of bias)
**HOME**	Definitely yes (low risk of bias)	Probably yes	Probably yes	Definitely no (high risk of bias)	Probably yes	Definitely yes (low risk of bias)
**Home 2018**	Definitely yes (low risk of bias)	Probably yes	Definitely yes (low risk of bias)	Probably yes	Probably yes	Definitely yes (low risk of bias)
**Hong 2015**	Definitely yes (low risk of bias)	Probably yes	Probably yes	Definitely yes (low risk of bias)	Probably yes	Definitely yes (low risk of bias)
**Hong 2016**	Definitely yes (low risk of bias)	Probably yes	Definitely yes (low risk of bias)	Probably no	Probably yes	Definitely yes (low risk of bias)
**Horton 2000**	Definitely yes (low risk of bias)	Definitely yes (low risk of bias)	Definitely yes (low risk of bias)	Definitely no (high risk of bias)	Probably yes	Definitely yes (low risk of bias)
**Hotta 1993**	Definitely yes (low risk of bias)	Probably yes	Definitely yes (low risk of bias)	Definitely yes (low risk of bias)	Probably yes	Definitely yes (low risk of bias)

Study ID	Domain 1	Domain 2	Domain 3	Domain 4	Domain 5	Domain 6
**Hsieh 2011**	Definitely yes (low risk of bias)	Probably yes	Definitely yes (low risk of bias)	Definitely yes (low risk of bias)	Definitely yes (low risk of bias)	Definitely yes (low risk of bias)
**Hwang 2008**	Definitely yes (low risk of bias)	Probably yes	Probably yes	Probably yes	Probably yes	Definitely yes (low risk of bias)
**Hygum 2020**	Definitely yes (low risk of bias)	Definitely yes (low risk of bias)	Definitely yes (low risk of bias)	Definitely yes (low risk of bias)	Definitely yes (low risk of bias)	Definitely yes (low risk of bias)
**Iacobellis 2017**	Definitely yes (low risk of bias)	Probably yes	Probably no	Probably yes	Definitely yes (low risk of bias)	Definitely yes (low risk of bias)
**Iacobellis-2020**	Definitely yes (low risk of bias)	Probably yes	Definitely yes (low risk of bias)	Probably no	Definitely yes (low risk of bias)	Definitely yes (low risk of bias)
**IDDIA**	Probably no	Probably no	Definitely yes (low risk of bias)	Probably yes	Definitely yes (low risk of bias)	Probably no
**Ikonomidis 2020**	Definitely yes (low risk of bias)	Probably yes	Definitely yes (low risk of bias)	Definitely yes (low risk of bias)	Definitely yes (low risk of bias)	Definitely yes (low risk of bias)
**ILLUMINATE**	Definitely yes (low risk of bias)	Probably yes	Definitely yes (low risk of bias)	Probably yes	Definitely yes (low risk of bias)	Definitely yes (low risk of bias)
**Inagaki 2012**	Definitely yes (low risk of bias)	Probably yes	Probably no	Definitely yes (low risk of bias)	Probably yes	Definitely yes (low risk of bias)
**Inagaki 2013**	Definitely yes (low risk of bias)	Probably yes	Probably no	Definitely yes (low risk of bias)	Definitely yes (low risk of bias)	Definitely yes (low risk of bias)
**Inagaki 2014**	Definitely yes (low risk of bias)	Definitely yes (low risk of bias)	Definitely yes (low risk of bias)	Probably yes	Definitely yes (low risk of bias)	Definitely yes (low risk of bias)
**Inagaki 2015**	Definitely yes (low risk of bias)	Definitely yes (low risk of bias)	Definitely yes (low risk of bias)	Definitely yes (low risk of bias)	Definitely yes (low risk of bias)	Definitely yes (low risk of bias)
**INICOM**	Definitely yes (low risk of bias)	Probably yes	Definitely yes (low risk of bias)	Definitely yes (low risk of bias)	Definitely yes (low risk of bias)	Definitely yes (low risk of bias)

Study ID	Domain 1	Domain 2	Domain 3	Domain 4	Domain 5	Domain 6
**Inoue 2019**	**Definitely yes (low risk of bias)**	**Definitely yes (low risk of bias)**	**Probably no**	**Definitely yes (low risk of bias)**	**Definitely yes (low risk of bias)**	**Definitely yes (low risk of bias)**
**Insulin Glargine 4014**	**Definitely yes (low risk of bias)**	**Probably yes**	**Probably no**	**Definitely yes (low risk of bias)**	**Probably yes**	**Definitely yes (low risk of bias)**
**INTERVAL**	**Definitely yes (low risk of bias)**	**Definitely yes (low risk of bias)**	**Definitely yes (low risk of bias)**	**Definitely yes (low risk of bias)**	**Definitely yes (low risk of bias)**	**Definitely yes (low risk of bias)**
**Ito 2011**	**Definitely yes (low risk of bias)**	**Probably yes**	**Probably no**	**Probably yes**	**Probably yes**	**Definitely yes (low risk of bias)**
**Ito 2017**	**Definitely yes (low risk of bias)**	**Probably yes**	**Probably no**	**Definitely yes (low risk of bias)**	**Definitely yes (low risk of bias)**	**Definitely yes (low risk of bias)**
**Jabbour 2014**	**Definitely yes (low risk of bias)**	**Probably yes**	**Definitely yes (low risk of bias)**	**Definitely yes (low risk of bias)**	**Definitely yes (low risk of bias)**	**Definitely yes (low risk of bias)**
**Jacob 2007**	**Definitely yes (low risk of bias)**	**Probably yes**	**Probably no**	**Probably yes**	**Probably yes**	**Definitely yes (low risk of bias)**
**Jain 2006**	**Definitely yes (low risk of bias)**	**Probably yes**	**Definitely yes (low risk of bias)**	**Definitely no (high risk of bias)**	**Probably yes**	**Definitely yes (low risk of bias)**
**J-BOND Study**	**Definitely yes (low risk of bias)**	**Probably no**	**Probably no**	**Definitely yes (low risk of bias)**	**Definitely yes (low risk of bias)**	**Definitely yes (low risk of bias)**
**JEDIS-1**	**Definitely yes (low risk of bias)**	**Probably yes**	**Probably no**	**Probably no**	**Probably yes**	**Definitely yes (low risk of bias)**
**Jeon 2011**	**Definitely yes (low risk of bias)**	**Probably yes**	**Probably no**	**Definitely yes (low risk of bias)**	**Probably yes**	**Definitely yes (low risk of bias)**
**Jeon 2018**	**Definitely no (high risk of bias)**	**Definitely no (high risk of bias)**	**Probably no**	**Definitely yes (low risk of bias)**	**Probably yes**	**Definitely yes (low risk of bias)**
**Jerums 1987**	**Definitely yes (low risk of bias)**	**Probably yes**	**Definitely yes (low risk of bias)**	**Probably yes**	**Probably yes**	**Definitely yes (low risk of bias)**

Study ID	Domain 1	Domain 2	Domain 3	Domain 4	Domain 5	Domain 6
**Ji 2014**	Definitely yes (low risk of bias)	Probably yes	Definitely yes (low risk of bias)	Probably yes	Definitely yes (low risk of bias)	Definitely yes (low risk of bias)
**Ji 2016**	Definitely yes (low risk of bias)	Definitely yes (low risk of bias)	Definitely yes (low risk of bias)	Definitely yes (low risk of bias)	Definitely yes (low risk of bias)	Definitely yes (low risk of bias)
**Ji 2017**	Definitely yes (low risk of bias)	Probably yes	Definitely yes (low risk of bias)	Probably no	Probably yes	Definitely yes (low risk of bias)
**Ji-2021**	Definitely yes (low risk of bias)	Definitely yes (low risk of bias)	Definitely yes (low risk of bias)	Probably no	Definitely yes (low risk of bias)	Definitely yes (low risk of bias)
**Ji-2021a**	Definitely yes (low risk of bias)	Definitely yes (low risk of bias)	Definitely yes (low risk of bias)	Probably no	Definitely yes (low risk of bias)	Definitely yes (low risk of bias)
**Jian 2018**	Definitely yes (low risk of bias)	Probably yes	Probably yes	Definitely yes (low risk of bias)	Probably yes	Definitely yes (low risk of bias)
**Jiang-2021**	Probably no	Probably no	Definitely yes (low risk of bias)	Definitely yes (low risk of bias)	Definitely yes (low risk of bias)	Probably yes
**Jibran 2006**	Definitely yes (low risk of bias)	Probably yes	Probably yes	Definitely yes (low risk of bias)	Probably yes	Definitely yes (low risk of bias)
**Johnston 1998**	Definitely yes (low risk of bias)	Probably yes	Definitely yes (low risk of bias)	Definitely yes (low risk of bias)	Probably yes	Definitely yes (low risk of bias)
**Johnston 1998a**	Definitely yes (low risk of bias)	Probably yes	Definitely yes (low risk of bias)	Definitely yes (low risk of bias)	Probably yes	Definitely yes (low risk of bias)
**Johnston 1998b**	Definitely yes (low risk of bias)	Probably yes	Definitely yes (low risk of bias)	Probably yes	Probably yes	Definitely yes (low risk of bias)
**Josse 2003**	Definitely yes (low risk of bias)	Probably yes	Definitely yes (low risk of bias)	Definitely yes (low risk of bias)	Probably yes	Definitely yes (low risk of bias)
**Jovanovic 2000**	Definitely yes (low risk of bias)	Probably yes	Definitely yes (low risk of bias)	Definitely no (high risk of bias)	Probably yes	Definitely yes (low risk of bias)

Study ID	Domain 1	Domain 2	Domain 3	Domain 4	Domain 5	Domain 6
Jovanovic 2004	Definitely yes (low risk of bias)	Probably yes	Probably no	Definitely no (high risk of bias)	Probably yes	Definitely yes (low risk of bias)
Jung 2005	Definitely yes (low risk of bias)	Probably yes	Probably yes	Definitely yes (low risk of bias)	Probably yes	Definitely yes (low risk of bias)
Juurinen 2009	Definitely yes (low risk of bias)	Definitely yes (low risk of bias)	Definitely yes (low risk of bias)	Definitely yes (low risk of bias)	Probably yes	Definitely yes (low risk of bias)
Kadoglou 2007	Definitely yes (low risk of bias)	Probably yes	Probably yes	Definitely yes (low risk of bias)	Definitely yes (low risk of bias)	Definitely yes (low risk of bias)
Kadoglou 2008	Definitely yes (low risk of bias)	Probably yes	Probably no	Definitely yes (low risk of bias)	Probably yes	Definitely yes (low risk of bias)
Kadoglou 2011	Definitely yes (low risk of bias)	Probably yes	Probably no	Definitely yes (low risk of bias)	Probably yes	Definitely yes (low risk of bias)
Kadowaki 2011	Definitely yes (low risk of bias)	Probably yes	Definitely yes (low risk of bias)	Probably yes	Definitely yes (low risk of bias)	Definitely yes (low risk of bias)
Kadowaki 2017	Definitely yes (low risk of bias)	Probably yes	Definitely yes (low risk of bias)	Definitely yes (low risk of bias)	Definitely yes (low risk of bias)	Definitely yes (low risk of bias)
Kadowaki 2018	Definitely yes (low risk of bias)	Probably yes	Definitely yes (low risk of bias)	Definitely yes (low risk of bias)	Definitely yes (low risk of bias)	Definitely yes (low risk of bias)
Kaku 2009	Definitely yes (low risk of bias)	Probably yes	Definitely yes (low risk of bias)	Definitely yes (low risk of bias)	Definitely yes (low risk of bias)	Definitely yes (low risk of bias)
Kaku 2009a	Definitely yes (low risk of bias)	Probably yes	Probably yes	Probably yes	Definitely yes (low risk of bias)	Definitely yes (low risk of bias)
Kaku 2011	Definitely yes (low risk of bias)	Probably yes	Probably yes	Probably yes	Probably yes	Definitely yes (low risk of bias)
Kaku 2014	Definitely yes (low risk of bias)	Probably yes	Definitely yes (low risk of bias)	Definitely yes (low risk of bias)	Probably yes	Definitely yes (low risk of bias)

Study ID	Domain 1	Domain 2	Domain 3	Domain 4	Domain 5	Domain 6
**Kaku 2017**	Definitely yes (low risk of bias)	Probably yes	Definitely yes (low risk of bias)	Definitely yes (low risk of bias)	Probably yes	Definitely yes (low risk of bias)
**Kaku 2019**	Definitely yes (low risk of bias)	Definitely yes (low risk of bias)	Definitely yes (low risk of bias)	Definitely yes (low risk of bias)	Definitely yes (low risk of bias)	Definitely yes (low risk of bias)
**Kanazawa 2010**	Definitely yes (low risk of bias)	Probably yes	Probably no	Probably yes	Definitely yes (low risk of bias)	Definitely yes (low risk of bias)
**Kanazawa 2011**	Definitely yes (low risk of bias)	Probably yes	Probably no	Definitely yes (low risk of bias)	Definitely yes (low risk of bias)	Definitely yes (low risk of bias)
**Kang-2021**	Definitely yes (low risk of bias)	Definitely yes (low risk of bias)	Definitely yes (low risk of bias)	Probably yes	Definitely yes (low risk of bias)	Probably yes
**Kashyap-2020**	Probably no	Probably no	Definitely yes (low risk of bias)	Definitely yes (low risk of bias)	Definitely no (high risk of bias)	Probably yes
**Kato 2015**	Probably yes	Probably yes	Probably no	Definitely yes (low risk of bias)	Definitely yes (low risk of bias)	Definitely yes (low risk of bias)
**Kawamori 2018**	Definitely yes (low risk of bias)	Definitely yes (low risk of bias)	Definitely yes (low risk of bias)	Probably yes	Definitely yes (low risk of bias)	Definitely yes (low risk of bias)
**Kayano-2020**	Definitely yes (low risk of bias)	Probably no	Definitely no (high risk of bias)	Probably yes	Definitely yes (low risk of bias)	Probably no
**Kelly 2007**	Definitely yes (low risk of bias)	Probably yes	Definitely yes (low risk of bias)	Probably yes	Definitely yes (low risk of bias)	Definitely yes (low risk of bias)
**Kendall 2005**	Definitely yes (low risk of bias)	Probably yes	Definitely yes (low risk of bias)	Probably no	Probably yes	Definitely yes (low risk of bias)
**Khaloo 2019**	Definitely yes (low risk of bias)	Probably yes	Definitely no (high risk of bias)	Probably yes	Definitely yes (low risk of bias)	Definitely yes (low risk of bias)
**Khan-2022**	Definitely yes (low risk of bias)	Probably yes	Definitely no (high risk of bias)	Definitely yes (low risk of bias)	Definitely yes (low risk of bias)	Definitely yes (low risk of bias)

Study ID	Domain 1	Domain 2	Domain 3	Domain 4	Domain 5	Domain 6
Khanolkar 2008	Definitely yes (low risk of bias)	Probably yes	Definitely yes (low risk of bias)	Definitely yes (low risk of bias)	Probably yes	Definitely yes (low risk of bias)
Kikuchi 2012	Definitely yes (low risk of bias)	Probably yes	Definitely yes (low risk of bias)	Probably yes	Definitely yes (low risk of bias)	Definitely yes (low risk of bias)
Kim 2014	Definitely yes (low risk of bias)	Definitely yes (low risk of bias)	Definitely yes (low risk of bias)	Probably yes	Definitely yes (low risk of bias)	Definitely yes (low risk of bias)
Kim 2019	Definitely yes (low risk of bias)	Probably yes	Probably no	Probably yes	Definitely yes (low risk of bias)	Definitely yes (low risk of bias)
Kim 2020	Definitely yes (low risk of bias)	Probably yes	Definitely yes (low risk of bias)	Definitely yes (low risk of bias)	Probably yes	Definitely yes (low risk of bias)
Kim-2022	Probably yes	Definitely no (high risk of bias)	Definitely no (high risk of bias)	Probably yes	Definitely yes (low risk of bias)	Definitely yes (low risk of bias)
KIND-LM	Definitely yes (low risk of bias)	Probably yes	Probably no	Definitely yes (low risk of bias)	Probably no	Definitely yes (low risk of bias)
Kinoshita 2020	Definitely yes (low risk of bias)	Definitely yes (low risk of bias)	Probably no	Definitely yes (low risk of bias)	Definitely yes (low risk of bias)	Definitely yes (low risk of bias)
Kiyici 2009	Definitely yes (low risk of bias)	Probably yes	Probably no	Definitely yes (low risk of bias)	Probably yes	Definitely yes (low risk of bias)
Ko 2006	Definitely yes (low risk of bias)	Probably yes	Probably yes	Definitely yes (low risk of bias)	Probably yes	Definitely yes (low risk of bias)
Koffert 2017	Definitely yes (low risk of bias)	Probably yes	Definitely yes (low risk of bias)	Probably yes	Definitely yes (low risk of bias)	Definitely yes (low risk of bias)
Kohan 2014	Definitely yes (low risk of bias)	Probably yes	Definitely yes (low risk of bias)	Definitely no (high risk of bias)	Probably yes	Definitely yes (low risk of bias)
Kondo 2016	Definitely yes (low risk of bias)	Probably yes	Probably no	Definitely yes (low risk of bias)	Definitely yes (low risk of bias)	Definitely yes (low risk of bias)

Study ID	Domain 1	Domain 2	Domain 3	Domain 4	Domain 5	Domain 6
**Kothny 2013**	Definitely yes (low risk of bias)	Probably yes	Definitely yes (low risk of bias)	Probably no	Probably yes	Definitely yes (low risk of bias)
**Krawczyk 2005**	Definitely yes (low risk of bias)	Probably yes	Probably yes	Probably yes	Probably yes	Probably yes
**Kudo-Fujimaki 2014**	Definitely yes (low risk of bias)	Probably yes	Probably yes	Probably yes	Definitely yes (low risk of bias)	Definitely yes (low risk of bias)
**Lam 1998**	Definitely yes (low risk of bias)	Probably yes	Definitely yes (low risk of bias)	Definitely yes (low risk of bias)	Probably yes	Definitely yes (low risk of bias)
**Lambadiari 2018**	Definitely yes (low risk of bias)	Probably yes	Probably yes	Definitely yes (low risk of bias)	Probably yes	Definitely yes (low risk of bias)
**LANTERN**	Definitely yes (low risk of bias)	Probably yes	Definitely yes (low risk of bias)	Definitely yes (low risk of bias)	Definitely yes (low risk of bias)	Definitely yes (low risk of bias)
**Lawrence 2004**	Definitely yes (low risk of bias)	Probably yes	Probably no	Definitely yes (low risk of bias)	Probably yes	Definitely yes (low risk of bias)
**LEAD-1**	Definitely yes (low risk of bias)	Probably yes	Definitely yes (low risk of bias)	Probably no	Probably yes	Definitely yes (low risk of bias)
**LEAD-2**	Definitely yes (low risk of bias)	Definitely yes (low risk of bias)	Definitely yes (low risk of bias)	Probably no	Probably yes	Definitely yes (low risk of bias)
**LEAD-3**	Definitely yes (low risk of bias)	Definitely yes (low risk of bias)	Definitely yes (low risk of bias)	Definitely no (high risk of bias)	Probably yes	Definitely yes (low risk of bias)
**LEAD-4**	Definitely yes (low risk of bias)	Definitely yes (low risk of bias)	Definitely yes (low risk of bias)	Definitely no (high risk of bias)	Definitely yes (low risk of bias)	Definitely yes (low risk of bias)
**LEAD-5**	Definitely yes (low risk of bias)	Definitely yes (low risk of bias)	Definitely yes (low risk of bias)	Probably yes	Definitely yes (low risk of bias)	Definitely yes (low risk of bias)
**LEADER**	Definitely yes (low risk of bias)	Probably yes	Definitely yes (low risk of bias)	Definitely yes (low risk of bias)	Definitely yes (low risk of bias)	Definitely yes (low risk of bias)

Study ID	Domain 1	Domain 2	Domain 3	Domain 4	Domain 5	Domain 6
Lebovitz 2001	Definitely yes (low risk of bias)	Probably yes	Definitely yes (low risk of bias)	Definitely no (high risk of bias)	Probably yes	Definitely yes (low risk of bias)
Ledesma 2019	Definitely yes (low risk of bias)	Definitely yes (low risk of bias)	Definitely yes (low risk of bias)	Definitely yes (low risk of bias)	Definitely yes (low risk of bias)	Definitely yes (low risk of bias)
Lee 2013	Definitely yes (low risk of bias)	Probably yes	Probably yes	Probably yes	Probably yes	Definitely yes (low risk of bias)
Lee 2017	Definitely yes (low risk of bias)	Definitely yes (low risk of bias)	Definitely yes (low risk of bias)	Definitely no (high risk of bias)	Definitely yes (low risk of bias)	Definitely yes (low risk of bias)
Lee-2013a	Probably no	Probably no	Definitely no (high risk of bias)	Definitely yes (low risk of bias)	Probably no	Probably no
Lee-2022	Definitely yes (low risk of bias)	Probably yes	Definitely no (high risk of bias)	Probably yes	Definitely yes (low risk of bias)	Definitely yes (low risk of bias)
Leiter 2014	Definitely yes (low risk of bias)	Definitely yes (low risk of bias)	Definitely yes (low risk of bias)	Definitely no (high risk of bias)	Probably yes	Definitely yes (low risk of bias)
Leiter 2014a	Definitely yes (low risk of bias)	Definitely yes (low risk of bias)	Definitely yes (low risk of bias)	Definitely yes (low risk of bias)	Definitely yes (low risk of bias)	Definitely yes (low risk of bias)
Leonhardt 1991	Definitely yes (low risk of bias)	Probably yes	Definitely yes (low risk of bias)	Definitely yes (low risk of bias)	Probably yes	Definitely yes (low risk of bias)
Lewin 2007	Definitely yes (low risk of bias)	Probably yes	Definitely yes (low risk of bias)	Definitely no (high risk of bias)	Probably yes	Definitely yes (low risk of bias)
Lewin 2015	Definitely yes (low risk of bias)	Definitely yes (low risk of bias)	Definitely yes (low risk of bias)	Probably no	Definitely yes (low risk of bias)	Definitely yes (low risk of bias)
Li 2014	Definitely yes (low risk of bias)	Probably yes	Probably no	Probably yes	Probably yes	Definitely yes (low risk of bias)
Li 2014a	Definitely yes (low risk of bias)	Probably yes	Probably yes	Definitely yes (low risk of bias)	Probably no	Definitely yes (low risk of bias)

Study ID	Domain 1	Domain 2	Domain 3	Domain 4	Domain 5	Domain 6
**Li 2017**	**Definitely yes (low risk of bias)**	**Probably yes**	**Definitely yes (low risk of bias)**	**Definitely yes (low risk of bias)**	**Probably no**	**Definitely yes (low risk of bias)**
**Li 2019**	**Definitely yes (low risk of bias)**	**Probably yes**	**Probably yes**	**Definitely yes (low risk of bias)**	**Probably yes**	**Probably yes**
**Li 2019a**	**Probably no**	**Probably no**	**Probably yes**	**Probably no**	**Probably no**	**Probably no**
**Li 2020**	**Definitely yes (low risk of bias)**	**Probably yes**	**Probably yes**	**Probably no**	**Probably no**	**Probably no**
**LIBRA**	**Probably no**	**Probably no**	**Definitely yes (low risk of bias)**	**Definitely yes (low risk of bias)**	**Probably yes**	**Probably no**
**Lim 2017**	**Probably yes**	**Probably no**	**Probably yes**	**Probably yes**	**Probably yes**	**Probably yes**
**Lin 2003**	**Definitely yes (low risk of bias)**	**Definitely yes (low risk of bias)**	**Definitely yes (low risk of bias)**	**Probably yes**	**Probably yes**	**Probably yes**
**Linagliptin-LTC Trial**	**Definitely yes (low risk of bias)**	**Definitely yes (low risk of bias)**	**Probably yes**	**Definitely yes (low risk of bias)**	**Probably no**	**Probably no**
**Lindstrom 2000**	**Probably yes**	**Probably no**	**Probably yes**	**Probably yes**	**Probably yes**	**Probably yes**
**Lingvay 2018**	**Definitely yes (low risk of bias)**	**Definitely yes (low risk of bias)**	**Definitely yes (low risk of bias)**	**Probably yes**	**Probably yes**	**Probably yes**
**LIPER2**	**Probably yes**	**Probably no**	**Probably yes**	**Probably yes**	**Probably no**	**Probably yes**
**LIRA-ADD2SGLT2i**	**Definitely yes (low risk of bias)**	**Definitely yes (low risk of bias)**	**Definitely yes (low risk of bias)**	**Probably yes**	**Probably yes**	**Probably yes**
**LIRAFLAME**	**Definitely yes (low risk of bias)**	**Definitely yes (low risk of bias)**	**Definitely yes (low risk of bias)**	**Definitely yes (low risk of bias)**	**Definitely yes (low risk of bias)**	**Probably no**
**Liraglutide-Detemir Study**	**Probably yes**	**Definitely yes (low risk of bias)**	**Probably yes**	**Probably no**	**Probably yes**	**Probably yes**
**LIRA-RENAL**	**Definitely yes (low risk of bias)**	**Definitely yes (low risk of bias)**	**Definitely yes (low risk of bias)**	**Probably no**	**Probably yes**	**Probably yes**

Study ID	Domain 1	Domain 2	Domain 3	Domain 4	Domain 5	Domain 6
LIRA-SWITCH	Definitely yes (low risk of bias)	Definitely yes (low risk of bias)	Probably yes	Probably yes	Probably yes	Probably yes
Liu 2013	Definitely yes (low risk of bias)	Definitely yes (low risk of bias)	Probably yes	Probably yes	Probably yes	Probably no
Liu 2014	Definitely yes (low risk of bias)	Probably yes	Definitely yes (low risk of bias)	Definitely yes (low risk of bias)	Probably yes	Probably yes
Liu 2020	Definitely yes (low risk of bias)	Definitely yes (low risk of bias)	Probably yes	Probably no	Probably yes	Probably yes
Liu-2021	Definitely yes (low risk of bias)	Definitely yes (low risk of bias)	Definitely no (high risk of bias)	Definitely yes (low risk of bias)	Definitely yes (low risk of bias)	Probably yes
Liutkus 2010	Definitely yes (low risk of bias)	Probably no	Probably yes	Probably no	Probably yes	Probably yes
Lou 2020	Definitely yes (low risk of bias)	Probably no	Probably yes	Probably no	Probably yes	Probably yes
Lu 2016	Definitely yes (low risk of bias)	Definitely yes (low risk of bias)	Definitely yes (low risk of bias)	Probably no	Definitely yes (low risk of bias)	Probably yes
Lu-2021	Definitely yes (low risk of bias)	Definitely yes (low risk of bias)	Definitely yes (low risk of bias)	Definitely yes (low risk of bias)	Definitely yes (low risk of bias)	Probably yes
Lukashevich 2011	Probably yes	Probably no	Probably yes	Probably no	Probably yes	Probably yes
Lund 2009	Definitely yes (low risk of bias)	Definitely yes (low risk of bias)	Definitely yes (low risk of bias)	Probably no	Probably yes	Probably yes
LYDIA	Definitely yes (low risk of bias)	Definitely yes (low risk of bias)	Probably yes	Definitely yes (low risk of bias)	Definitely yes (low risk of bias)	Probably no
Macauley 2015	Probably yes	Probably no	Probably yes	Probably no	Probably yes	Probably no
Madsbad 2001	Probably yes	Probably no	Definitely yes (low risk of bias)	Probably yes	Probably yes	Probably yes

Study ID	Domain 1	Domain 2	Domain 3	Domain 4	Domain 5	Domain 6
**Maffioli 2013**	**Definitely yes (low risk of bias)**	**Definitely yes (low risk of bias)**	**Definitely yes (low risk of bias)**	**Probably yes**	**Probably yes**	**Probably yes**
**MAGNA VICTORIA**	**Definitely yes (low risk of bias)**	**Definitely yes (low risk of bias)**	**Definitely yes (low risk of bias)**	**Probably yes**	**Probably yes**	**Definitely no (high risk of bias)**
**Marbury 1999**	**Probably yes**	**Probably no**	**Definitely yes (low risk of bias)**	**Probably no**	**Probably no**	**Probably no**
**MARCH**	**Definitely yes (low risk of bias)**	**Probably yes**	**Probably yes**	**Probably no**	**Probably yes**	**Probably no**
**Mari 2008**	**Probably yes**	**Probably no**	**Probably yes**	**Definitely no (high risk of bias)**	**Probably yes**	**Probably yes**
**MARLINA-T2D**	**Definitely yes (low risk of bias)**	**Definitely yes (low risk of bias)**	**Probably yes**	**Probably no**	**Probably yes**	**Probably yes**
**Marre 2002**	**Definitely yes (low risk of bias)**	**Definitely yes (low risk of bias)**	**Definitely yes (low risk of bias)**	**Probably no**	**Probably yes**	**Probably yes**
**MASTER**	**Definitely yes (low risk of bias)**	**Probably yes**	**Probably yes**	**Probably no**	**Probably yes**	**Probably yes**
**Mathieu 2015**	**Definitely yes (low risk of bias)**	**Probably no**	**Probably yes**	**Probably yes**	**Probably yes**	**Probably yes**
**Mathieu 2016**	**Probably yes**	**Probably no**	**Probably yes**	**Probably yes**	**Probably yes**	**Probably yes**
**Matthaei 2015**	**Definitely yes (low risk of bias)**	**Definitely yes (low risk of bias)**	**Probably yes**	**Probably yes**	**Probably yes**	**Probably yes**
**Matthaei 2015a**	**Definitely yes (low risk of bias)**	**Definitely yes (low risk of bias)**	**Definitely yes (low risk of bias)**	**Probably yes**	**Probably no**	**Probably no**
**Matthews 2005**	**Probably yes**	**Probably no**	**Probably yes**	**Probably yes**	**Probably yes**	**Probably yes**
**Matthews 2010**	**Probably yes**	**Probably no**	**Probably yes**	**Probably yes**	**Probably yes**	**Probably yes**

Study ID	Domain 1	Domain 2	Domain 3	Domain 4	Domain 5	Domain 6
**Mattoo 2005**	**Definitely yes (low risk of bias)**	**Definitely yes (low risk of bias)**	**Definitely yes (low risk of bias)**	**Probably no**	**Probably no**	**Probably yes**
**McCluskey 2004**	**Definitely yes (low risk of bias)**	**Probably no**	**Probably yes**	**Probably no**	**Probably no**	**Probably no**
**McGill 2013**	**Definitely yes (low risk of bias)**	**Probably no**	**Definitely yes (low risk of bias)**	**Probably no**	**Probably yes**	**Probably no**
**McGuire 2010**	**Definitely yes (low risk of bias)**	**Probably no**	**Probably yes**	**Probably no**	**Probably yes**	**Probably yes**
**MDI Liraglutide**	**Definitely yes (low risk of bias)**	**Probably no**	**Definitely yes (low risk of bias)**	**Probably yes**	**Definitely yes (low risk of bias)**	**Probably yes**
**Meneghini 2010**	**Definitely yes (low risk of bias)**	**Probably yes**	**Probably yes**	**Probably yes**	**Probably yes**	**Probably yes**
**Mita 2007**	**Definitely yes (low risk of bias)**	**Probably yes**	**Probably no**	**Probably no**	**Probably no**	**Probably yes**
**Mita 2019**	**Definitely yes (low risk of bias)**	**Probably yes**	**Probably no**	**Probably yes**	**Probably yes**	**Probably no**
**Mitrakou 1998**	**Probably yes**	**Probably no**	**Probably yes**	**Probably no**	**Probably yes**	**Probably yes**
**Miyagawa 2015**	**Definitely yes (low risk of bias)**	**Definitely yes (low risk of bias)**	**Definitely yes (low risk of bias)**	**Probably no**	**Probably yes**	**Probably yes**
**Miyazaki 2002**	**Probably yes**	**Probably no**	**Probably yes**	**Probably no**	**Probably yes**	**Probably no**
**MK-0431J-842**	**Definitely yes (low risk of bias)**	**Probably no**	**Definitely yes (low risk of bias)**	**Probably yes**	**Definitely yes (low risk of bias)**	**Definitely yes (low risk of bias)**
**Moeinzadeh-2021**	**Definitely yes (low risk of bias)**	**Definitely yes (low risk of bias)**	**Definitely yes (low risk of bias)**	**Definitely yes (low risk of bias)**	**Definitely yes (low risk of bias)**	**Probably no**
**Mokta 2018**	**Definitely yes (low risk of bias)**	**Definitely yes (low risk of bias)**	**Probably yes**	**Definitely no (high risk of bias)**	**Probably yes**	**Probably no**

Study ID	Domain 1	Domain 2	Domain 3	Domain 4	Domain 5	Domain 6
Moretto 2008	Definitely yes (low risk of bias)	Definitely yes (low risk of bias)	Probably yes	Probably no	Probably yes	Probably yes
Morino 2018	Definitely yes (low risk of bias)	Definitely yes (low risk of bias)	Probably no	Probably yes	Probably yes	Probably yes
Moriwaki 2018	Definitely yes (low risk of bias)	Probably no	Probably yes	Definitely yes (low risk of bias)	Definitely yes (low risk of bias)	Probably yes
Moses 2014	Definitely yes (low risk of bias)	Definitely yes (low risk of bias)	Definitely yes (low risk of bias)	Definitely yes (low risk of bias)	Probably yes	Probably yes
Moses 2016	Definitely yes (low risk of bias)	Probably no	Definitely yes (low risk of bias)	Probably no	Probably yes	Probably yes
Mu 2017	Probably yes	Probably yes	Probably yes	Probably no	Probably no	Probably yes
Müller-Wieland 2018	Definitely yes (low risk of bias)	Probably no	Definitely yes (low risk of bias)	Probably no	Probably yes	Probably yes
MUSCAT-HF	Definitely yes (low risk of bias)	Definitely yes (low risk of bias)	Definitely no (high risk of bias)	Definitely yes (low risk of bias)	Definitely yes (low risk of bias)	Probably no
Nahra-2021	Definitely yes (low risk of bias)	Definitely yes (low risk of bias)	Definitely yes (low risk of bias)	Definitely yes (low risk of bias)	Definitely yes (low risk of bias)	Definitely yes (low risk of bias)
Nakamura 2001	Probably yes	Probably no	Probably yes	Probably no	Probably yes	Probably yes
Nakamura 2004	Definitely yes (low risk of bias)	Probably yes	Probably no	Definitely yes (low risk of bias)	Probably yes	Probably yes
Nakamura 2006	Definitely yes (low risk of bias)	Probably yes	Probably no	Definitely yes (low risk of bias)	Probably yes	Probably yes
Nar 2009	Probably yes	Probably no	Probably yes	Probably no	Probably no	Probably yes
Nathan 1988	Definitely yes (low risk of bias)	Probably no	Definitely yes (low risk of bias)	Probably yes	Probably no	Probably yes

Study ID	Domain 1	Domain 2	Domain 3	Domain 4	Domain 5	Domain 6
Nauck 2007a	Definitely yes (low risk of bias)	Definitely yes (low risk of bias)	Probably yes	Probably yes	Probably yes	Probably yes
Nauck 2007b	Probably yes	Probably no	Probably yes	Probably yes	Probably yes	Probably yes
Nauck 2009	Definitely yes (low risk of bias)	Definitely yes (low risk of bias)	Probably yes	Probably no	Probably yes	Probably yes
Nauck 2011	Definitely yes (low risk of bias)	Definitely yes (low risk of bias)	Probably yes	Probably no	Probably yes	Probably yes
Nauck 2016	Definitely yes (low risk of bias)	Definitely yes (low risk of bias)	Probably yes	Probably no	Probably yes	Probably no
Negro 2005	Probably yes	Probably no	Probably yes	Definitely yes (low risk of bias)	Probably yes	Probably yes
Ning 2016	Definitely yes (low risk of bias)	Definitely yes (low risk of bias)	Probably yes	Probably yes	Definitely yes (low risk of bias)	Probably yes
Nino 2017	Definitely yes (low risk of bias)	Definitely yes (low risk of bias)	Probably yes	Probably no	Probably yes	Probably yes
Nishimura 2016	Definitely yes (low risk of bias)	Probably no	Probably yes	Definitely yes (low risk of bias)	Probably yes	Probably no
Nishio 2006	Probably yes	Probably no	Probably yes	Probably no	Probably yes	Probably yes
N-ISM	Definitely yes (low risk of bias)	Definitely no (high risk of bias)	Definitely no (high risk of bias)	Definitely no (high risk of bias)	Probably no	Probably no
Nogueira 2014	Probably yes	Probably no	Probably yes	Probably yes	Probably yes	Probably no
Nowicki 2011	Definitely yes (low risk of bias)	Definitely yes (low risk of bias)	Probably yes	Definitely no (high risk of bias)	Probably yes	Probably yes
Ogasawara 2009	Probably yes	Probably no	Probably yes	Probably no	Probably no	Probably yes
Oh 2019	Definitely yes (low risk of bias)	Probably no	Definitely yes (low risk of bias)	Probably yes	Definitely yes (low risk of bias)	Probably yes

Study ID	Domain 1	Domain 2	Domain 3	Domain 4	Domain 5	Domain 6
**Ohira 2014**	Definitely yes (low risk of bias)	Probably no	Probably yes	Probably no	Probably yes	Probably no
**Ohira 2014a**	Definitely yes (low risk of bias)	Probably yes	Probably yes	Probably no	Probably no	Probably yes
**Omarigliptin Protocol 020**	Definitely yes (low risk of bias)	Definitely yes (low risk of bias)	Definitely yes (low risk of bias)	Definitely yes (low risk of bias)	Probably yes	Probably yes
**Omariglitin Study 015**	Definitely yes (low risk of bias)	Definitely yes (low risk of bias)	Definitely yes (low risk of bias)	Definitely yes (low risk of bias)	Probably yes	Probably yes
**Onoue 2020**	Definitely yes (low risk of bias)	Definitely yes (low risk of bias)	Probably yes	Definitely yes (low risk of bias)	Definitely yes (low risk of bias)	Probably no
**Onuchin 2010**	Definitely yes (low risk of bias)	Probably no	Probably yes	Probably no	Probably yes	Probably yes
**Osman 2004**	Probably yes	Probably no	Probably yes	Probably no	Probably no	Probably yes
**Ovalle 2004**	Probably yes	Probably no	Probably no	Probably no	Probably no	Probably yes
**Owens 2011**	Definitely yes (low risk of bias)	Probably no	Probably yes	Probably no	Probably no	Probably yes
**Oyama 2008**	Definitely yes (low risk of bias)	Probably yes	Probably yes	Probably no	Probably yes	Probably yes
**Pagano 1995**	Probably yes	Probably no	Probably yes	Probably yes	Probably yes	Probably no
**Pan 2008**	Probably yes	Probably no	Probably yes	Probably yes	Probably yes	Probably yes
**Pan 2012**	Probably yes	Probably no	Probably yes	Probably no	Probably yes	Probably yes
**Pan 2012a**	Definitely yes (low risk of bias)	Definitely yes (low risk of bias)	Definitely yes (low risk of bias)	Probably no	Probably yes	Probably yes
**Park 2011**	Probably yes	Probably no	Probably no	Probably no	Probably yes	Probably yes
**Park 2014**	Definitely yes (low risk of bias)	Definitely yes (low risk of bias)	Probably yes	Probably no	Definitely yes (low risk of bias)	Probably yes

Study ID	Domain 1	Domain 2	Domain 3	Domain 4	Domain 5	Domain 6
Park 2017	Definitely yes (low risk of bias)	Probably no	Probably yes	Probably no	Probably no	Probably yes
Parmar Vinendra 2019	Probably yes	Probably no	Definitely no (high risk of bias)	Definitely yes (low risk of bias)	Probably yes	Definitely yes (low risk of bias)
Parthan 2018	Probably yes	Probably no	Definitely no (high risk of bias)	Probably yes	Definitely no (high risk of bias)	Definitely yes (low risk of bias)
Pasquel-2021	Definitely yes (low risk of bias)	Probably no	Definitely yes (low risk of bias)	Definitely no (high risk of bias)	Definitely yes (low risk of bias)	Definitely no (high risk of bias)
Patel 2013	Probably yes	Probably yes	Probably no	Probably no	Probably no	Definitely yes (low risk of bias)
Pavithra 2019	Probably yes	Probably no	Probably no	Probably yes	Probably no	Definitely yes (low risk of bias)
Pavo 2003	Probably yes	Probably yes	Probably no	Definitely yes (low risk of bias)	Probably no	Definitely yes (low risk of bias)
Perez 2009	Probably yes	Probably no	Probably no	Probably no	Definitely yes (low risk of bias)	Definitely yes (low risk of bias)
PERISCOPE	Definitely yes (low risk of bias)	Definitely yes (low risk of bias)	Definitely yes (low risk of bias)	Probably no	Definitely yes (low risk of bias)	Definitely yes (low risk of bias)
Perriello 2006	Probably yes	Probably yes	Probably no	Probably yes	Probably no	Definitely yes (low risk of bias)
Petrica 2009	Probably yes	Probably no	Probably yes	Probably yes	Probably no	Definitely yes (low risk of bias)
Petrica 2011	Probably yes	Probably no	Probably yes	Probably yes	Probably no	Definitely yes (low risk of bias)
Philis-Tsimikas 2013	Definitely yes (low risk of bias)	Probably yes	Definitely no (high risk of bias)	Probably no	Definitely yes (low risk of bias)	Definitely yes (low risk of bias)

Study ID	Domain 1	Domain 2	Domain 3	Domain 4	Domain 5	Domain 6
**Phillips 2001**	Probably yes	Probably no	Probably no	Probably no	Probably no	Definitely yes (low risk of bias)
**Phillips 2003**	Probably yes	Probably no	Probably no	Probably yes	Probably no	Definitely yes (low risk of bias)
**Phrommintikul 2019**	Definitely yes (low risk of bias)	Probably yes	Probably no	Probably yes	Definitely yes (low risk of bias)	Definitely yes (low risk of bias)
**PIOCOMB**	Probably yes	Probably no	Probably no	Definitely yes (low risk of bias)	Probably no	Definitely yes (low risk of bias)
**PIOfix**	Probably yes	Probably yes	Probably no	Probably no	Definitely no (high risk of bias)	Definitely yes (low risk of bias)
**Pioglitazone 001**	Probably yes	Probably no	Probably no	Probably no	Probably no	Definitely yes (low risk of bias)
**PIONEER**	Probably yes	Probably no	Probably yes	Probably no	Probably no	Definitely yes (low risk of bias)
**PIONEER 1**	Definitely yes (low risk of bias)	Definitely yes (low risk of bias)	Definitely yes (low risk of bias)	Definitely yes (low risk of bias)	Definitely yes (low risk of bias)	Definitely yes (low risk of bias)
**PIONEER 2**	Definitely yes (low risk of bias)	Probably yes	Definitely no (high risk of bias)	Probably yes	Definitely yes (low risk of bias)	Definitely yes (low risk of bias)
**PIONEER 3**	Definitely yes (low risk of bias)	Definitely yes (low risk of bias)	Definitely yes (low risk of bias)	Probably yes	Definitely yes (low risk of bias)	Definitely yes (low risk of bias)
**PIONEER 4**	Definitely yes (low risk of bias)	Definitely yes (low risk of bias)	Definitely yes (low risk of bias)	Probably yes	Definitely yes (low risk of bias)	Definitely yes (low risk of bias)
**PIONEER 5**	Definitely yes (low risk of bias)	Definitely yes (low risk of bias)	Definitely yes (low risk of bias)	Definitely yes (low risk of bias)	Definitely yes (low risk of bias)	Definitely yes (low risk of bias)
**PIONEER 6**	Definitely yes (low risk of bias)	Definitely yes (low risk of bias)	Definitely yes (low risk of bias)	Definitely yes (low risk of bias)	Definitely yes (low risk of bias)	Definitely yes (low risk of bias)

Study ID	Domain 1	Domain 2	Domain 3	Domain 4	Domain 5	Domain 6
PIONEER 7	Definitely yes (low risk of bias)	Probably yes	Definitely no (high risk of bias)	Probably no	Definitely yes (low risk of bias)	Definitely yes (low risk of bias)
PIONEER 8	Definitely yes (low risk of bias)	Definitely yes (low risk of bias)	Definitely yes (low risk of bias)	Definitely yes (low risk of bias)	Definitely yes (low risk of bias)	Definitely yes (low risk of bias)
PIONEER 9	Definitely yes (low risk of bias)	Definitely yes (low risk of bias)	Definitely yes (low risk of bias)	Definitely yes (low risk of bias)	Definitely yes (low risk of bias)	Definitely yes (low risk of bias)
PioRAGE	Definitely yes (low risk of bias)	Probably yes	Probably no	Probably yes	Definitely no (high risk of bias)	Definitely yes (low risk of bias)
PIRAMID	Definitely yes (low risk of bias)	Definitely yes (low risk of bias)	Probably no	Probably yes	Definitely no (high risk of bias)	Definitely yes (low risk of bias)
Pistrosch 2012	Probably yes	Probably no	Probably no	Probably yes	Definitely no (high risk of bias)	Probably yes
Pistrosch 2013	Probably yes	Probably no	Definitely no (high risk of bias)	Probably no	Definitely no (high risk of bias)	Definitely yes (low risk of bias)
Pi-Sunyer 2007	Probably yes	Probably no	Probably no	Probably yes	Probably no	Definitely yes (low risk of bias)
Pop-Busui 2009	Probably yes	Probably no	Probably no	Probably yes	Definitely no (high risk of bias)	Probably yes
POPPS	Probably yes	Probably no	Probably no	Probably yes	Probably no	Definitely yes (low risk of bias)
Pratley 2014	Probably yes	Probably no	Definitely yes (low risk of bias)	Probably no	Definitely yes (low risk of bias)	Definitely yes (low risk of bias)
PRESERVE-BETA	Probably yes	Probably no	Probably no	Probably no	Probably no	Definitely yes (low risk of bias)
PREVENT-J	Probably yes	Probably no	Probably yes	Probably no	Definitely no (high risk of bias)	Probably yes

Study ID	Domain 1	Domain 2	Domain 3	Domain 4	Domain 5	Domain 6
**PRIDE**	**Definitely yes (low risk of bias)**	**Probably no**	**Definitely no (high risk of bias)**	**Probably yes**	**Definitely yes (low risk of bias)**	**Definitely yes (low risk of bias)**
**PRIME-V**	**Probably yes**	**Probably no**	**Probably yes**	**Definitely yes (low risk of bias)**	**Definitely no (high risk of bias)**	**Definitely yes (low risk of bias)**
**PRISMA**	**Probably yes**	**Probably no**	**Probably no**	**Probably yes**	**Definitely yes (low risk of bias)**	**Definitely yes (low risk of bias)**
**PROActive**	**Definitely yes (low risk of bias)**	**Definitely yes (low risk of bias)**	**Definitely yes (low risk of bias)**	**Probably yes**	**Definitely yes (low risk of bias)**	**Definitely yes (low risk of bias)**
**PROLOGUE**	**Definitely yes (low risk of bias)**	**Probably yes**	**Probably no**	**Probably yes**	**Definitely yes (low risk of bias)**	**Definitely yes (low risk of bias)**
**QUARTER**	**Definitely yes (low risk of bias)**	**Definitely yes (low risk of bias)**	**Probably no**	**Probably yes**	**Probably no**	**Definitely yes (low risk of bias)**
**Quatraro 1986**	**Probably yes**	**Probably no**	**Probably no**	**Probably yes**	**Probably no**	**Definitely yes (low risk of bias)**
**Raghuveer 2020**	**Probably yes**	**Probably no**	**Definitely no (high risk of bias)**	**Definitely yes (low risk of bias)**	**Probably no**	**Probably yes**
**Rahman 2010**	**Probably yes**	**Probably yes**	**Probably no**	**Definitely yes (low risk of bias)**	**Definitely yes (low risk of bias)**	**Definitely yes (low risk of bias)**
**Rahman 2011**	**Probably yes**	**Probably no**	**Probably no**	**Probably yes**	**Probably no**	**Definitely yes (low risk of bias)**
**Raskin 2001**	**Definitely yes (low risk of bias)**	**Definitely yes (low risk of bias)**	**Probably no**	**Probably no**	**Probably no**	**Definitely yes (low risk of bias)**
**Raskin 2004**	**Probably yes**	**Probably no**	**Definitely no (high risk of bias)**	**Probably yes**	**Probably no**	**Definitely yes (low risk of bias)**
**Raskin 2009**	**Probably yes**	**Probably no**	**Definitely no (high risk of bias)**	**Definitely no (high risk of bias)**	**Probably yes**	**Definitely yes (low risk of bias)**

Study ID	Domain 1	Domain 2	Domain 3	Domain 4	Domain 5	Domain 6
Raz 2008	Definitely yes (low risk of bias)	Probably no	Probably no	Probably yes	Definitely yes (low risk of bias)	Definitely yes (low risk of bias)
RECORD	Definitely yes (low risk of bias)	Definitely yes (low risk of bias)	Probably yes	Definitely yes (low risk of bias)	Definitely yes (low risk of bias)	Definitely yes (low risk of bias)
REFORM	Definitely yes (low risk of bias)	Definitely yes (low risk of bias)	Probably no	Probably yes	Definitely yes (low risk of bias)	Definitely yes (low risk of bias)
REGO-F	Definitely yes (low risk of bias)	Definitely yes (low risk of bias)	Definitely no (high risk of bias)	Probably no	Definitely no (high risk of bias)	Definitely yes (low risk of bias)
RELEASE	Probably yes	Definitely yes (low risk of bias)	Probably no	Probably yes	Definitely no (high risk of bias)	Definitely yes (low risk of bias)
RESET-IT Main	Definitely yes (low risk of bias)	Probably yes	Probably no	Probably no	Probably no	Definitely yes (low risk of bias)
RESULT	Probably yes	Probably no	Probably no	Probably no	Probably yes	Definitely yes (low risk of bias)
REWIND	Definitely yes (low risk of bias)	Definitely yes (low risk of bias)	Definitely yes (low risk of bias)	Definitely yes (low risk of bias)	Definitely yes (low risk of bias)	Definitely yes (low risk of bias)
Reynolds 2002	Probably yes	Probably no	Probably no	Probably no	Probably no	Definitely yes (low risk of bias)
Reynolds 2007	Probably yes	Probably no	Probably no	Probably no	Probably no	Probably no
Ristic 2006	Definitely yes (low risk of bias)	Definitely yes (low risk of bias)	Probably no	Probably no	Probably yes	Definitely yes (low risk of bias)
Robbins 2007	Definitely yes (low risk of bias)	Definitely yes (low risk of bias)	Definitely no (high risk of bias)	Probably no	Definitely no (high risk of bias)	Definitely yes (low risk of bias)
Roberts 2005	Probably yes	Probably no	Probably no	Probably no	Probably yes	Definitely yes (low risk of bias)

Study ID	Domain 1	Domain 2	Domain 3	Domain 4	Domain 5	Domain 6
**Rodbard 2016**	**Definitely yes (low risk of bias)**	**Probably yes**	**Probably no**	**Probably no**	**Probably no**	**Definitely yes (low risk of bias)**
**Rosenstock 1998**	**Probably yes**	**Probably no**	**Probably no**	**Probably no**	**Probably no**	**Probably yes**
**Rosenstock 2006**	**Probably yes**	**Probably no**	**Probably no**	**Probably yes**	**Probably yes**	**Definitely yes (low risk of bias)**
**Rosenstock 2007**	**Probably yes**	**Probably no**	**Probably no**	**Probably yes**	**Definitely yes (low risk of bias)**	**Definitely yes (low risk of bias)**
**Rosenstock 2007a**	**Probably yes**	**Probably no**	**Probably no**	**Probably no**	**Definitely no (high risk of bias)**	**Definitely yes (low risk of bias)**
**Rosenstock 2009**	**Definitely yes (low risk of bias)**	**Probably no**	**Probably no**	**Probably no**	**Definitely yes (low risk of bias)**	**Definitely yes (low risk of bias)**
**Rosenstock 2010**	**Probably yes**	**Probably no**	**Probably no**	**Probably no**	**Definitely no (high risk of bias)**	**Probably yes**
**Rosenstock 2012**	**Probably yes**	**Probably no**	**Probably no**	**Probably no**	**Definitely yes (low risk of bias)**	**Definitely yes (low risk of bias)**
**Rosenstock 2013**	**Probably yes**	**Probably no**	**Probably no**	**Probably no**	**Definitely yes (low risk of bias)**	**Definitely yes (low risk of bias)**
**Rosenstock 2015**	**Definitely yes (low risk of bias)**	**Probably yes**	**Probably no**	**Probably no**	**Definitely yes (low risk of bias)**	**Definitely yes (low risk of bias)**
**Rosenstock 2016**	**Definitely yes (low risk of bias)**	**Probably no**	**Probably no**	**Probably yes**	**Definitely yes (low risk of bias)**	**Definitely yes (low risk of bias)**
**Rosenstock 2019**	**Definitely yes (low risk of bias)**	**Probably yes**	**Probably no**	**Probably yes**	**Definitely yes (low risk of bias)**	**Definitely yes (low risk of bias)**
**Rosenstock-2020**	**Definitely yes (low risk of bias)**	**Probably no**	**Probably yes**	**Probably no**	**Definitely yes (low risk of bias)**	**Definitely yes (low risk of bias)**

Study ID	Domain 1	Domain 2	Domain 3	Domain 4	Domain 5	Domain 6
Ross 2015	Definitely yes (low risk of bias)	Definitely yes (low risk of bias)	Probably no	Probably yes	Definitely yes (low risk of bias)	Definitely yes (low risk of bias)
SAIS1	Probably yes	Probably no	Probably yes	Probably yes	Definitely yes (low risk of bias)	Definitely yes (low risk of bias)
Saleem 2011	Probably yes	Probably no	Definitely no (high risk of bias)	Probably yes	Probably no	Definitely yes (low risk of bias)
Salman 2001	Probably yes	Probably no	Definitely no (high risk of bias)	Probably yes	Probably no	Probably yes
Saloranta 2002	Probably yes	Probably no	Probably no	Probably yes	Definitely yes (low risk of bias)	Definitely yes (low risk of bias)
Samson 2011	Definitely yes (low risk of bias)	Probably no	Definitely no (high risk of bias)	Probably yes	Probably no	Probably yes
Sato 2018	Probably yes	Probably yes	Probably no	Probably yes	Definitely no (high risk of bias)	Definitely yes (low risk of bias)
Sato 2019	Probably yes	Probably yes	Probably no	Probably yes	Definitely no (high risk of bias)	Definitely yes (low risk of bias)
SAVOR-TIMI 53	Definitely yes (low risk of bias)	Definitely yes (low risk of bias)	Definitely yes (low risk of bias)	Definitely yes (low risk of bias)	Definitely yes (low risk of bias)	Definitely yes (low risk of bias)
Saxagliptin 014	Definitely yes (low risk of bias)	Probably yes	Probably no	Definitely no (high risk of bias)	Definitely yes (low risk of bias)	Definitely yes (low risk of bias)
SCALE Diabetes	Definitely yes (low risk of bias)	Definitely yes (low risk of bias)	Probably no	Probably no	Definitely yes (low risk of bias)	Definitely yes (low risk of bias)
SCALE insulin	Definitely yes (low risk of bias)	Definitely yes (low risk of bias)	Definitely yes (low risk of bias)	Definitely yes (low risk of bias)	Definitely yes (low risk of bias)	Definitely yes (low risk of bias)
Scherbaum 2002	Probably yes	Probably no	Probably no	Probably no	Probably no	Definitely yes (low risk of bias)

Study ID	Domain 1	Domain 2	Domain 3	Domain 4	Domain 5	Domain 6
Scherbaum 2008	Probably yes	Probably no	Probably no	Definitely yes (low risk of bias)	Definitely yes (low risk of bias)	Definitely yes (low risk of bias)
Schiavon-2021	Probably no	Probably no	Definitely no (high risk of bias)	Probably yes	Definitely yes (low risk of bias)	Probably no
Schweizer 2007	Probably yes	Probably no	Probably no	Probably no	Definitely yes (low risk of bias)	Definitely yes (low risk of bias)
Schweizer 2009	Probably yes	Probably no	Probably no	Probably yes	Definitely yes (low risk of bias)	Definitely yes (low risk of bias)
SCORED	Definitely yes (low risk of bias)	Definitely yes (low risk of bias)	Definitely yes (low risk of bias)	Definitely yes (low risk of bias)	Definitely yes (low risk of bias)	Definitely yes (low risk of bias)
SEESAW	Definitely yes (low risk of bias)	Probably yes	Probably yes	Definitely yes (low risk of bias)	Definitely yes (low risk of bias)	Definitely yes (low risk of bias)
Segal 1997	Probably yes	Probably no	Probably no	Probably no	Probably no	Definitely yes (low risk of bias)
Segal 2005	Probably yes	Probably no	Probably no	Probably no	Definitely yes (low risk of bias)	Definitely yes (low risk of bias)
Seino 2011	Probably yes	Probably no	Definitely no (high risk of bias)	Probably no	Definitely yes (low risk of bias)	Definitely yes (low risk of bias)
Seino 2014	Probably yes	Probably no	Probably yes	Definitely yes (low risk of bias)	Definitely yes (low risk of bias)	Definitely yes (low risk of bias)
Seino 2015	Probably yes	Probably yes	Definitely no (high risk of bias)	Definitely yes (low risk of bias)	Probably no	Definitely yes (low risk of bias)
Seino 2016	Definitely yes (low risk of bias)	Probably yes	Probably no	Definitely yes (low risk of bias)	Definitely yes (low risk of bias)	Definitely yes (low risk of bias)
Seino 2018	Definitely yes (low risk of bias)	Probably yes	Probably yes	Probably yes	Definitely yes (low risk of bias)	Definitely yes (low risk of bias)

Study ID	Domain 1	Domain 2	Domain 3	Domain 4	Domain 5	Domain 6
**Shah 2011**	Probably yes	Probably no	Probably no	Probably yes	Probably no	Definitely yes (low risk of bias)
**Shankar 2017**	Probably yes	Probably no	Probably no	Probably yes	Definitely yes (low risk of bias)	Definitely yes (low risk of bias)
**Shankar 2017a**	Probably yes	Probably yes	Probably no	Probably yes	Definitely no (high risk of bias)	Definitely yes (low risk of bias)
**Shestakova 2018**	Definitely yes (low risk of bias)	Definitely yes (low risk of bias)	Probably no	Definitely yes (low risk of bias)	Definitely yes (low risk of bias)	Definitely yes (low risk of bias)
**Shibuya 2018**	Definitely yes (low risk of bias)	Probably yes	Definitely no (high risk of bias)	Definitely yes (low risk of bias)	Definitely yes (low risk of bias)	Definitely yes (low risk of bias)
**Shuai-2021**	Definitely yes (low risk of bias)	Probably yes	Definitely yes (low risk of bias)	Definitely no (high risk of bias)	Definitely yes (low risk of bias)	Probably no
**SIMPLE**	Definitely yes (low risk of bias)	Definitely yes (low risk of bias)	Definitely no (high risk of bias)	Definitely no (high risk of bias)	Definitely yes (low risk of bias)	Probably no
**Sit2Mix**	Definitely no (high risk of bias)	Definitely no (high risk of bias)	Definitely no (high risk of bias)	Definitely yes (low risk of bias)	Definitely yes (low risk of bias)	Probably no
**Sitagliptin Study 019**	Definitely no (high risk of bias)	Definitely no (high risk of bias)	Probably no	Definitely yes (low risk of bias)	Definitely yes (low risk of bias)	Probably no
**Sitagliptin Study 020**	Definitely no (high risk of bias)	Definitely no (high risk of bias)	Probably no	Definitely yes (low risk of bias)	Definitely yes (low risk of bias)	Probably no
**Sitagliptin Study 021**	Definitely no (high risk of bias)	Definitely no (high risk of bias)	Probably no	Definitely yes (low risk of bias)	Definitely yes (low risk of bias)	Probably no
**Sitagliptin Study 024**	Definitely yes (low risk of bias)	Probably yes	Probably no	Definitely yes (low risk of bias)	Definitely yes (low risk of bias)	Probably no
**Sitagliptin Study 035**	Definitely no (high risk of bias)	Definitely no (high risk of bias)	Probably no	Definitely yes (low risk of bias)	Definitely yes (low risk of bias)	Probably no

Study ID	Domain 1	Domain 2	Domain 3	Domain 4	Domain 5	Domain 6
Sitagliptin Study 036	Definitely no (high risk of bias)	Definitely no (high risk of bias)	Probably no	Definitely yes (low risk of bias)	Definitely yes (low risk of bias)	Probably no
Sitagliptin Study 049	Definitely yes (low risk of bias)	Probably yes	Probably no	Definitely yes (low risk of bias)	Definitely yes (low risk of bias)	Probably no
Sitagliptin Study 051	Definitely yes (low risk of bias)	Probably yes	Probably no	Definitely yes (low risk of bias)	Definitely yes (low risk of bias)	Probably no
SLIM	Probably no	Definitely no (high risk of bias)	Definitely no (high risk of bias)	Probably no	Definitely yes (low risk of bias)	Definitely yes (low risk of bias)
SMART	Definitely yes (low risk of bias)	Definitely yes (low risk of bias)	Probably no	Definitely yes (low risk of bias)	Definitely yes (low risk of bias)	Probably no
Smith 2005	Definitely no (high risk of bias)	Definitely no (high risk of bias)	Definitely yes (low risk of bias)	Definitely yes (low risk of bias)	Definitely yes (low risk of bias)	Probably no
Softeland 2017	Definitely yes (low risk of bias)	Definitely yes (low risk of bias)	Probably no	Definitely yes (low risk of bias)	Definitely yes (low risk of bias)	Probably no
Sohn 2008	Definitely yes (low risk of bias)	Definitely yes (low risk of bias)	Definitely no (high risk of bias)	Definitely no (high risk of bias)	Definitely yes (low risk of bias)	Probably yes
SOLOIST-WHF	Definitely yes (low risk of bias)	Definitely yes (low risk of bias)	Definitely yes (low risk of bias)	Definitely yes (low risk of bias)	Definitely yes (low risk of bias)	Probably no
Sone 2019	Definitely yes (low risk of bias)	Definitely no (high risk of bias)	Definitely no (high risk of bias)	Definitely yes (low risk of bias)	Definitely yes (low risk of bias)	Probably no
South Danish Diabetes Study	Definitely yes (low risk of bias)	Probably yes	Probably no	Definitely yes (low risk of bias)	Definitely yes (low risk of bias)	Definitely yes (low risk of bias)
SPEAD-A	Definitely yes (low risk of bias)	Definitely no (high risk of bias)	Probably no	Definitely yes (low risk of bias)	Definitely yes (low risk of bias)	Probably no
SPECIFY	Definitely yes (low risk of bias)	Definitely yes (low risk of bias)	Definitely yes (low risk of bias)	Probably no	Definitely yes (low risk of bias)	Probably no

Study ID	Domain 1	Domain 2	Domain 3	Domain 4	Domain 5	Domain 6
SPIKE	Probably yes	Definitely no (high risk of bias)	Probably no	Probably yes	Definitely yes (low risk of bias)	Probably no
SPOTLIGHT	Definitely yes (low risk of bias)	Definitely yes (low risk of bias)	Definitely yes (low risk of bias)	Probably yes	Definitely yes (low risk of bias)	Probably no
SPREAD-DIMCAD	Definitely yes (low risk of bias)	Definitely yes (low risk of bias)	Definitely yes (low risk of bias)	Definitely yes (low risk of bias)	Definitely yes (low risk of bias)	Definitely yes (low risk of bias)
Sridhar 2013	Probably yes	Probably yes	Definitely yes (low risk of bias)	Probably yes	Probably yes	Definitely yes (low risk of bias)
St. John Sutton 2002	Probably yes	Definitely no (high risk of bias)	Definitely no (high risk of bias)	Probably yes	Definitely yes (low risk of bias)	Probably yes
Standl 1999	Probably yes	Probably yes	Definitely yes (low risk of bias)	Probably yes	Definitely yes (low risk of bias)	Probably yes
Standl 2001	Probably yes	Probably yes	Definitely yes (low risk of bias)	Definitely yes (low risk of bias)	Definitely yes (low risk of bias)	Probably yes
START	Definitely yes (low risk of bias)	Probably yes	Definitely yes (low risk of bias)	Probably yes	Definitely yes (low risk of bias)	Probably yes
START-J	Definitely yes (low risk of bias)	Definitely yes (low risk of bias)	Definitely no (high risk of bias)	Probably yes	Definitely yes (low risk of bias)	Probably yes
STEP 2	Definitely yes (low risk of bias)	Definitely yes (low risk of bias)	Definitely yes (low risk of bias)	Definitely yes (low risk of bias)	Definitely yes (low risk of bias)	Definitely yes (low risk of bias)
Stewart 2006	Probably yes	Definitely yes (low risk of bias)	Definitely yes (low risk of bias)	Probably no	Definitely yes (low risk of bias)	Probably no
Stocker 2007	Definitely yes (low risk of bias)	Probably no	Definitely no (high risk of bias)	Probably no	Definitely yes (low risk of bias)	Definitely yes (low risk of bias)
STOP-OB	Definitely yes (low risk of bias)	Definitely yes (low risk of bias)	Definitely yes (low risk of bias)	Probably yes	Definitely yes (low risk of bias)	Probably no

Study ID	Domain 1	Domain 2	Domain 3	Domain 4	Domain 5	Domain 6
Strojek 2009	Definitely yes (low risk of bias)	Definitely yes (low risk of bias)	Definitely no (high risk of bias)	Probably yes	Definitely yes (low risk of bias)	Probably no
Strojek 2011	Definitely yes (low risk of bias)	Definitely yes (low risk of bias)	Definitely yes (low risk of bias)	Probably yes	Probably yes	Probably no
Strøm Halden 2019	Definitely yes (low risk of bias)	Definitely yes (low risk of bias)	Definitely yes (low risk of bias)	Probably yes	Definitely yes (low risk of bias)	Probably yes
Su 2014	Probably no	Probably no	Probably no	Probably yes	Definitely yes (low risk of bias)	Probably yes
SUCCESS	Definitely yes (low risk of bias)	Definitely no (high risk of bias)	Definitely no (high risk of bias)	Probably yes	Definitely yes (low risk of bias)	Probably yes
SUGAR-DM-HF	Definitely yes (low risk of bias)	Definitely yes (low risk of bias)	Probably yes	Definitely yes (low risk of bias)	Definitely yes (low risk of bias)	Probably no
Sun 2006	Probably no	Probably no	Probably no	Probably yes	Definitely yes (low risk of bias)	Probably yes
Sun 2016	Probably yes	Probably no	Probably no	Probably yes	Probably yes	Definitely yes (low risk of bias)
SUPER	Probably yes	Probably yes	Definitely yes (low risk of bias)	Probably yes	Definitely yes (low risk of bias)	Probably no
SURPASS J-mono	Definitely yes (low risk of bias)	Definitely yes (low risk of bias)	Definitely yes (low risk of bias)	Probably no	Definitely yes (low risk of bias)	Definitely yes (low risk of bias)
SURPASS-1	Definitely yes (low risk of bias)	Definitely yes (low risk of bias)	Definitely yes (low risk of bias)	Probably no	Definitely yes (low risk of bias)	Definitely yes (low risk of bias)
SURPASS-2	Probably no	Probably yes	Probably no	Definitely yes (low risk of bias)	Definitely yes (low risk of bias)	Definitely yes (low risk of bias)
SURPASS-3	Definitely yes (low risk of bias)	Definitely yes (low risk of bias)	Probably yes	Definitely yes (low risk of bias)	Definitely yes (low risk of bias)	Definitely yes (low risk of bias)

Study ID	Domain 1	Domain 2	Domain 3	Domain 4	Domain 5	Domain 6
**SURPASS-4**	**Definitely yes (low risk of bias)**	**Definitely yes (low risk of bias)**	**Probably yes**	**Definitely yes (low risk of bias)**	**Definitely yes (low risk of bias)**	**Definitely yes (low risk of bias)**
**SURPASS-5**	**Definitely yes (low risk of bias)**	**Definitely yes (low risk of bias)**	**Definitely yes (low risk of bias)**	**Probably yes**	**Definitely yes (low risk of bias)**	**Probably no**
**SUSTAIN 1**	**Definitely yes (low risk of bias)**	**Definitely yes (low risk of bias)**	**Definitely yes (low risk of bias)**	**Probably yes**	**Definitely yes (low risk of bias)**	**Probably no**
**SUSTAIN 2**	**Definitely yes (low risk of bias)**	**Definitely yes (low risk of bias)**	**Definitely yes (low risk of bias)**	**Probably yes**	**Definitely yes (low risk of bias)**	**Probably no**
**SUSTAIN 4**	**Definitely yes (low risk of bias)**	**Definitely yes (low risk of bias)**	**Definitely no (high risk of bias)**	**Probably yes**	**Definitely yes (low risk of bias)**	**Probably no**
**SUSTAIN 5**	**Definitely yes (low risk of bias)**	**Definitely yes (low risk of bias)**	**Definitely yes (low risk of bias)**	**Probably yes**	**Definitely yes (low risk of bias)**	**Probably no**
**SUSTAIN 6**	**Probably yes**	**Probably yes**	**Definitely yes (low risk of bias)**	**Definitely yes (low risk of bias)**	**Definitely yes (low risk of bias)**	**Probably no**
**SUSTAIN 8**	**Definitely yes (low risk of bias)**	**Definitely yes (low risk of bias)**	**Definitely yes (low risk of bias)**	**Probably yes**	**Definitely yes (low risk of bias)**	**Probably no**
**SUSTAIN 9**	**Definitely yes (low risk of bias)**	**Definitely yes (low risk of bias)**	**Definitely yes (low risk of bias)**	**Definitely yes (low risk of bias)**	**Definitely yes (low risk of bias)**	**Probably no**
**SUSTAIN 11**	**Probably yes**	**Probably yes**	**Definitely no (high risk of bias)**	**Definitely yes (low risk of bias)**	**Definitely yes (low risk of bias)**	**Definitely yes (low risk of bias)**
**SUSTAIN China**	**Definitely yes (low risk of bias)**	**Definitely yes (low risk of bias)**	**Definitely yes (low risk of bias)**	**Definitely yes (low risk of bias)**	**Definitely yes (low risk of bias)**	**Definitely yes (low risk of bias)**
**Suzuki 2014**	**Probably no**	**Definitely no (high risk of bias)**	**Definitely no (high risk of bias)**	**Probably no**	**Definitely yes (low risk of bias)**	**Definitely yes (low risk of bias)**
**SWIM**	**Definitely yes (low risk of bias)**	**Probably yes**	**Definitely no (high risk of bias)**	**Probably yes**	**Probably yes**	**Definitely yes (low risk of bias)**

Study ID	Domain 1	Domain 2	Domain 3	Domain 4	Domain 5	Domain 6
**Takagi 2003**	Probably no	Probably no	Probably no	**Definitely yes (low risk of bias)**	**Definitely yes (low risk of bias)**	Probably yes
**Takahara-2020**	**Definitely yes (low risk of bias)**	**Definitely no (high risk of bias)**	**Definitely yes (low risk of bias)**	**Definitely no (high risk of bias)**	**Definitely yes (low risk of bias)**	Probably no
**Takahashi-2022**	Probably no	**Definitely no (high risk of bias)**	**Definitely yes (low risk of bias)**	**Definitely yes (low risk of bias)**	**Definitely yes (low risk of bias)**	**Definitely no (high risk of bias)**
**Takase 2007**	**Definitely yes (low risk of bias)**	**Definitely yes (low risk of bias)**	Probably no	**Definitely yes (low risk of bias)**	**Definitely yes (low risk of bias)**	Probably yes
**Takashima 2018**	Probably no	**Definitely no (high risk of bias)**	**Definitely no (high risk of bias)**	Probably yes	**Definitely yes (low risk of bias)**	Probably yes
**Takeshita-2022**	**Definitely yes (low risk of bias)**	**Definitely yes (low risk of bias)**	**Definitely no (high risk of bias)**	**Definitely yes (low risk of bias)**	**Definitely yes (low risk of bias)**	**Definitely yes (low risk of bias)**
**Tao 2018**	**Definitely yes (low risk of bias)**	**Definitely no (high risk of bias)**	**Definitely no (high risk of bias)**	Probably yes	**Definitely yes (low risk of bias)**	**Definitely yes (low risk of bias)**
**Taskinen 2011**	Probably yes	Probably yes	**Definitely yes (low risk of bias)**	Probably yes	**Definitely yes (low risk of bias)**	Probably yes
**TECOS**	Probably yes	**Definitely yes (low risk of bias)**	**Definitely yes (low risk of bias)**	Probably yes	**Definitely yes (low risk of bias)**	Probably yes
**Teramoto 2007**	Probably no	**Definitely no (high risk of bias)**	**Definitely no (high risk of bias)**	**Definitely yes (low risk of bias)**	**Definitely yes (low risk of bias)**	Probably yes
**Teupe 1991**	Probably no	Probably no	Probably no	Probably no	Probably yes	Probably yes
**Thrasher 2014**	**Definitely yes (low risk of bias)**	Probably yes	**Definitely yes (low risk of bias)**	Probably no	**Definitely yes (low risk of bias)**	Probably yes
**TIDE**	**Definitely yes (low risk of bias)**	Probably yes	**Definitely yes (low risk of bias)**	**Definitely yes (low risk of bias)**	Probably yes	Probably no

Study ID	Domain 1	Domain 2	Domain 3	Domain 4	Domain 5	Domain 6
**Tinahones 2017**	Definitely yes (low risk of bias)	Probably yes	Definitely yes (low risk of bias)	Probably no	Definitely yes (low risk of bias)	Probably no
**Tofogliflozin 003**	Definitely yes (low risk of bias)	Definitely yes (low risk of bias)	Definitely yes (low risk of bias)	Definitely yes (low risk of bias)	Definitely yes (low risk of bias)	Probably no
**Tolman 2009**	Definitely yes (low risk of bias)	Definitely yes (low risk of bias)	Definitely yes (low risk of bias)	Definitely no (high risk of bias)	Definitely yes (low risk of bias)	Probably no
**ToPiND**	Definitely yes (low risk of bias)	Definitely yes (low risk of bias)	Probably yes	Definitely no (high risk of bias)	Definitely yes (low risk of bias)	Probably no
**TOPSCORE**	Probably no	Definitely no (high risk of bias)	Definitely no (high risk of bias)	Probably no	Definitely yes (low risk of bias)	Probably no
**TOSCA.IT**	Definitely yes (low risk of bias)	Definitely yes (low risk of bias)	Definitely no (high risk of bias)	Probably yes	Definitely yes (low risk of bias)	Probably no
**Trakarnvanich-2021**	Probably no	Probably no	Definitely no (high risk of bias)	Definitely no (high risk of bias)	Definitely yes (low risk of bias)	Definitely no (high risk of bias)
**Trial 843**	Definitely yes (low risk of bias)	Definitely yes (low risk of bias)	Definitely yes (low risk of bias)	Definitely yes (low risk of bias)	Definitely yes (low risk of bias)	Definitely yes (low risk of bias)
**Tripathy 2013**	Probably yes	Probably yes	Definitely yes (low risk of bias)	Probably no	Definitely yes (low risk of bias)	Definitely yes (low risk of bias)
**TROICA**	Probably yes	Probably yes	Definitely yes (low risk of bias)	Probably yes	Probably no	Probably yes
**Truitt 2010**	Probably yes	Probably yes	Definitely yes (low risk of bias)	Definitely no (high risk of bias)	Definitely yes (low risk of bias)	Probably no
**Türkmen Kemal 2007**	Probably no	Probably no	Probably no	Definitely yes (low risk of bias)	Definitely yes (low risk of bias)	Probably yes
**Tuttolomondo-2021**	Definitely yes (low risk of bias)	Definitely yes (low risk of bias)	Definitely yes (low risk of bias)	Probably no	Definitely yes (low risk of bias)	Definitely yes (low risk of bias)

Study ID	Domain 1	Domain 2	Domain 3	Domain 4	Domain 5	Domain 6
**UKPDS 44**	Definitely no (high risk of bias)	Probably yes	Definitely yes (low risk of bias)	Probably yes	Definitely yes (low risk of bias)	Probably yes
**UKPDS 80**	Probably no	Definitely yes (low risk of bias)	Probably no	Probably no	Definitely yes (low risk of bias)	Definitely yes (low risk of bias)
**Umpierrez 2006**	Probably no	Definitely no (high risk of bias)	Definitely no (high risk of bias)	Probably yes	Definitely yes (low risk of bias)	Probably no
**UTOPIA**	Definitely yes (low risk of bias)	Probably yes	Probably no	Definitely yes (low risk of bias)	Definitely yes (low risk of bias)	Probably no
**Vähätalo 2007**	Probably no	Probably no	Probably no	Definitely yes (low risk of bias)	Definitely yes (low risk of bias)	Probably yes
**van Eyk 2019**	Probably yes	Probably yes	Definitely yes (low risk of bias)	Probably yes	Probably yes	Definitely yes (low risk of bias)
**Van Gaal 2014**	Probably yes	Probably yes	Definitely yes (low risk of bias)	Probably yes	Definitely yes (low risk of bias)	Probably no
**Vanderheiden 2016**	Definitely yes (low risk of bias)	Definitely yes (low risk of bias)	Definitely yes (low risk of bias)	Probably yes	Probably yes	Probably no
**Varghese 2009**	Definitely yes (low risk of bias)	Probably yes	Definitely yes (low risk of bias)	Probably yes	Definitely yes (low risk of bias)	Probably yes
**Veleba 2015**	Definitely yes (low risk of bias)	Definitely yes (low risk of bias)	Probably no	Probably yes	Definitely yes (low risk of bias)	Definitely yes (low risk of bias)
**VERIFY**	Definitely yes (low risk of bias)	Definitely yes (low risk of bias)	Definitely yes (low risk of bias)	Definitely yes (low risk of bias)	Definitely yes (low risk of bias)	Definitely yes (low risk of bias)
**VERTIS Asia**	Definitely yes (low risk of bias)	Probably yes	Probably yes	Probably yes	Probably yes	Probably no
**VERTIS CV**	Definitely yes (low risk of bias)	Definitely yes (low risk of bias)	Definitely yes (low risk of bias)	Probably no	Definitely yes (low risk of bias)	Definitely yes (low risk of bias)

Study ID	Domain 1	Domain 2	Domain 3	Domain 4	Domain 5	Domain 6
**VERTIS FACTORIAL**	**Definitely yes (low risk of bias)**	**Definitely yes (low risk of bias)**	**Definitely yes (low risk of bias)**	**Probably yes**	**Definitely yes (low risk of bias)**	**Probably no**
**VERTIS MET**	**Definitely yes (low risk of bias)**	**Probably no**	**Definitely yes (low risk of bias)**	**Probably no**	**Definitely yes (low risk of bias)**	**Definitely yes (low risk of bias)**
**VERTIS MONO**	**Definitely yes (low risk of bias)**	**Probably yes**	**Definitely yes (low risk of bias)**	**Probably yes**	**Definitely yes (low risk of bias)**	**Probably no**
**VERTIS RENAL**	**Definitely yes (low risk of bias)**	**Definitely yes (low risk of bias)**	**Definitely yes (low risk of bias)**	**Probably yes**	**Definitely yes (low risk of bias)**	**Probably no**
**VERTIS SITA2**	**Definitely yes (low risk of bias)**	**Probably yes**	**Definitely yes (low risk of bias)**	**Definitely yes (low risk of bias)**	**Definitely yes (low risk of bias)**	**Probably no**
**VERTIS SU**	**Definitely yes (low risk of bias)**	**Definitely yes (low risk of bias)**	**Definitely yes (low risk of bias)**	**Probably no**	**Definitely yes (low risk of bias)**	**Probably no**
**Vianna 2017**	**Definitely yes (low risk of bias)**	**Definitely yes (low risk of bias)**	**Probably no**	**Probably yes**	**Probably no**	**Definitely yes (low risk of bias)**
**VICTORY**	**Probably yes**	**Definitely yes (low risk of bias)**	**Definitely yes (low risk of bias)**	**Probably yes**	**Definitely yes (low risk of bias)**	**Probably no**
**VISION**	**Probably no**	**Definitely no (high risk of bias)**	**Definitely no (high risk of bias)**	**Probably yes**	**Definitely yes (low risk of bias)**	**Probably no**
**VIVIDD**	**Definitely yes (low risk of bias)**	**Definitely yes (low risk of bias)**	**Definitely yes (low risk of bias)**	**Probably no**	**Definitely yes (low risk of bias)**	**Probably no**
**Vongthavaravat 2002**	**Definitely yes (low risk of bias)**	**Definitely yes (low risk of bias)**	**Definitely no (high risk of bias)**	**Probably no**	**Definitely yes (low risk of bias)**	**Probably yes**
**Wada-2022**	**Probably yes**	**Probably yes**	**Probably yes**	**Definitely no (high risk of bias)**	**Definitely yes (low risk of bias)**	**Definitely yes (low risk of bias)**
**Wajcberg 2007**	**Probably yes**	**Probably yes**	**Definitely yes (low risk of bias)**	**Definitely yes (low risk of bias)**	**Definitely yes (low risk of bias)**	**Probably no**

Study ID	Domain 1	Domain 2	Domain 3	Domain 4	Domain 5	Domain 6
**Wang 2005**	**Probably no**	**Probably no**	**Probably no**	**Definitely yes (low risk of bias)**	**Definitely yes (low risk of bias)**	**Probably yes**
**Wang 2013**	**Definitely yes (low risk of bias)**	**Probably no**	**Probably no**	**Probably yes**	**Definitely yes (low risk of bias)**	**Definitely yes (low risk of bias)**
**Wang 2015**	**Probably no**	**Probably no**	**Probably no**	**Probably yes**	**Definitely yes (low risk of bias)**	**Definitely yes (low risk of bias)**
**Wang 2016**	**Definitely yes (low risk of bias)**	**Probably yes**	**Definitely yes (low risk of bias)**	**Probably yes**	**Probably yes**	**Probably no**
**Wang 2016a**	**Probably no**	**Probably no**	**Probably no**	**Definitely yes (low risk of bias)**	**Definitely yes (low risk of bias)**	**Definitely yes (low risk of bias)**
**Wang 2017**	**Definitely yes (low risk of bias)**	**Probably yes**	**Definitely yes (low risk of bias)**	**Probably yes**	**Probably yes**	**Probably yes**
**Wang 2019**	**Definitely yes (low risk of bias)**	**Definitely yes (low risk of bias)**	**Definitely no (high risk of bias)**	**Probably yes**	**Definitely yes (low risk of bias)**	**Probably no**
**Wang 2020**	**Definitely yes (low risk of bias)**	**Probably no**	**Probably no**	**Definitely yes (low risk of bias)**	**Definitely yes (low risk of bias)**	**Definitely yes (low risk of bias)**
**Wang 2020b**	**Definitely yes (low risk of bias)**	**Probably no**	**Definitely no (high risk of bias)**	**Probably no**	**Definitely yes (low risk of bias)**	**Probably yes**
**Wang-2020a**	**Definitely yes (low risk of bias)**	**Probably no**	**Definitely no (high risk of bias)**	**Definitely no (high risk of bias)**	**Definitely no (high risk of bias)**	**Definitely yes (low risk of bias)**
**Wang-2022**	Probably yes	Definitely no (high risk of bias)	Definitely no (high risk of bias)	Probably yes	Definitely yes (low risk of bias)	Definitely yes (low risk of bias)
**Watanabe 2005**	**Definitely yes (low risk of bias)**	**Probably no**	**Probably no**	**Definitely yes (low risk of bias)**	**Definitely yes (low risk of bias)**	**Probably yes**
**Weng-2021**	**Definitely yes (low risk of bias)**	**Definitely yes (low risk of bias)**	**Definitely yes (low risk of bias)**	**Probably yes**	**Probably no**	**Definitely yes (low risk of bias)**

Study ID	Domain 1	Domain 2	Domain 3	Domain 4	Domain 5	Domain 6
**Wolffenbuttel 1999**	Probably yes	Probably yes	Definitely yes (low risk of bias)	Probably no	Definitely yes (low risk of bias)	Probably yes
**Wolffenbuttel 2000**	Probably yes	Probably yes	Definitely yes (low risk of bias)	Probably no	Definitely yes (low risk of bias)	Probably yes
**Wong 2005**	Definitely yes (low risk of bias)	Definitely no (high risk of bias)	Definitely no (high risk of bias)	Probably yes	Definitely yes (low risk of bias)	Probably yes
**Wu 2014**	Probably no	Probably no	Probably no	Definitely yes (low risk of bias)	Definitely yes (low risk of bias)	Probably yes
**Wu 2015**	Definitely yes (low risk of bias)	Definitely yes (low risk of bias)	Definitely yes (low risk of bias)	Probably yes	Definitely yes (low risk of bias)	Definitely yes (low risk of bias)
**Xiao 2015**	Definitely yes (low risk of bias)	Probably yes	Definitely no (high risk of bias)	Probably yes	Definitely yes (low risk of bias)	Definitely yes (low risk of bias)
**XIao 2016**	Probably no	Probably no	Probably no	Definitely yes (low risk of bias)	Definitely yes (low risk of bias)	Probably no
**Xu 2017**	Definitely yes (low risk of bias)	Definitely yes (low risk of bias)	Definitely no (high risk of bias)	Probably yes	Probably yes	Probably no
**Xu-2022**	Definitely yes (low risk of bias)	Definitely yes (low risk of bias)	Definitely yes (low risk of bias)	Definitely yes (low risk of bias)	Definitely yes (low risk of bias)	Definitely yes (low risk of bias)
**Yabiku 2017**	Probably no	Definitely no (high risk of bias)	Definitely no (high risk of bias)	Probably yes	Definitely yes (low risk of bias)	Probably no
**Yagoglu-2020**	Probably no	Probably no	Definitely no (high risk of bias)	Definitely yes (low risk of bias)	Probably no	Probably no
**Yale 2013**	Probably yes	Probably yes	Definitely yes (low risk of bias)	Probably no	Definitely yes (low risk of bias)	Definitely no (high risk of bias)
**Yamakage 2019**	Definitely yes (low risk of bias)	Probably yes	Definitely no (high risk of bias)	Probably yes	Definitely yes (low risk of bias)	Probably no

Study ID	Domain 1	Domain 2	Domain 3	Domain 4	Domain 5	Domain 6
**Yamamoto 2018**	Probably no	Definitely no (high risk of bias)	Definitely no (high risk of bias)	Probably no	Definitely yes (low risk of bias)	Definitely yes (low risk of bias)
**Yamanouchi 2005**	Definitely yes (low risk of bias)	Probably no	Probably no	Probably yes	Definitely yes (low risk of bias)	Definitely yes (low risk of bias)
**Yamasaki 2005**	Probably no	Definitely no (high risk of bias)	Definitely no (high risk of bias)	Definitely yes (low risk of bias)	Definitely yes (low risk of bias)	Probably yes
**Yan 2019**	Definitely yes (low risk of bias)	Definitely yes (low risk of bias)	Definitely no (high risk of bias)	Probably yes	Probably yes	Definitely yes (low risk of bias)
**Yang 2002**	Probably yes	Probably yes	Definitely yes (low risk of bias)	Definitely yes (low risk of bias)	Probably yes	Probably no
**Yang 2011**	Definitely yes (low risk of bias)	Probably yes	Definitely yes (low risk of bias)	Probably yes	Definitely yes (low risk of bias)	Probably yes
**Yang 2012**	Definitely yes (low risk of bias)	Probably yes	Definitely yes (low risk of bias)	Probably yes	Probably yes	Probably no
**Yang 2013**	Probably yes	Probably yes	Definitely yes (low risk of bias)	Probably yes	Definitely yes (low risk of bias)	Definitely yes (low risk of bias)
**Yang 2015**	Definitely no (high risk of bias)	Probably yes	Definitely yes (low risk of bias)	Probably no	Definitely yes (low risk of bias)	Definitely yes (low risk of bias)
**Yang 2015a**	Probably yes	Probably yes	Definitely yes (low risk of bias)	Probably yes	Definitely yes (low risk of bias)	Probably no
**Yang 2016**	Definitely yes (low risk of bias)	Definitely yes (low risk of bias)	Definitely yes (low risk of bias)	Probably yes	Probably yes	Probably no
**Yang 2018**	Definitely yes (low risk of bias)	Definitely yes (low risk of bias)	Definitely yes (low risk of bias)	Probably yes	Definitely yes (low risk of bias)	Probably no
**Yang-2021**	Probably no	Probably no	Definitely yes (low risk of bias)	Definitely yes (low risk of bias)	Definitely yes (low risk of bias)	Definitely yes (low risk of bias)

Study ID	Domain 1	Domain 2	Domain 3	Domain 4	Domain 5	Domain 6
**Yee 2010**	Probably yes	Probably yes	Definitely yes (low risk of bias)	Probably yes	Definitely yes (low risk of bias)	Definitely yes (low risk of bias)
**Yki-Jarvinen 1999**	Definitely yes (low risk of bias)	Probably no	Probably no	Probably yes	Definitely yes (low risk of bias)	Definitely yes (low risk of bias)
**Yki-Jarvinen 2013**	Probably yes	Probably yes	Definitely yes (low risk of bias)	Probably no	Definitely yes (low risk of bias)	Probably no
**Yoon 2011**	Probably yes	Definitely no (high risk of bias)	Definitely yes (low risk of bias)	Probably no	Definitely yes (low risk of bias)	Probably no
**Yoon 2011a**	Probably yes	Probably yes	Definitely yes (low risk of bias)	Definitely no (high risk of bias)	Definitely yes (low risk of bias)	Probably yes
**Yuan 2012**	Definitely yes (low risk of bias)	Probably no	Probably no	Definitely yes (low risk of bias)	Definitely yes (low risk of bias)	Definitely yes (low risk of bias)
**Zang 2016**	Definitely yes (low risk of bias)	Definitely yes (low risk of bias)	Definitely no (high risk of bias)	Probably yes	Probably yes	Probably no
**ZEUS II**	Definitely yes (low risk of bias)	Definitely yes (low risk of bias)	Definitely yes (low risk of bias)	Definitely yes (low risk of bias)	Definitely yes (low risk of bias)	Definitely yes (low risk of bias)
**Zhang 2020**	Definitely yes (low risk of bias)	Definitely no (high risk of bias)	Definitely no (high risk of bias)	Definitely yes (low risk of bias)	Definitely yes (low risk of bias)	Definitely yes (low risk of bias)
**Zhang 2020a**	Definitely yes (low risk of bias)	Definitely no (high risk of bias)	Definitely no (high risk of bias)	Definitely yes (low risk of bias)	Definitely yes (low risk of bias)	Definitely no (high risk of bias)
**Zhou-2022**	Definitely yes (low risk of bias)	Definitely no (high risk of bias)	Definitely no (high risk of bias)	Definitely yes (low risk of bias)	Probably no	Definitely yes (low risk of bias)
**Zhu 2003**	Definitely yes (low risk of bias)	Definitely yes (low risk of bias)	Definitely yes (low risk of bias)	Probably no	Definitely yes (low risk of bias)	Probably no
**Zib 2007**	Definitely no (high risk of bias)	Definitely no (high risk of bias)	Definitely no (high risk of bias)	Definitely yes (low risk of bias)	Definitely yes (low risk of bias)	Probably yes

Summary of overall risk of bias according to drug classes

Notes: The following table summarised the risk of bias across six domains stratified by the drug classes. Generally, arms with insulins and meglitinide show high proportion of high risk of bias across six domains.

Drug class	Total No. of arms with high risk of bias across six domains	Total No. of arms	% of high risk of bias
**Alpha-glucosidase inhibitors**	28	78	35.9
**Basal insulin**	29	55	52.7
**Basal-Bolus insulin**	18	13	138.5
**Bolus insulin**	23	15	153.3
**DPP-4 inhibitors**	50	324	15.4
**GLP-1 receptor agonists**	41	289	14.2
**Meglitinides**	17	37	45.9
**Metformin**	32	111	28.8
**Non-steroidal MRAs**	0	3	0
**SGLT2 inhibitors**	39	252	15.5
**Standard treatments**	55	522	10.5
**Sulfonylureas**	41	150	27.3
**Thiazolidinediones**	50	230	21.7
**Tirzepatide**	1	30	3.3

Appendix 4 Supplement to results

4.1 Network plots for the outcomes

4.1.1 All-cause death

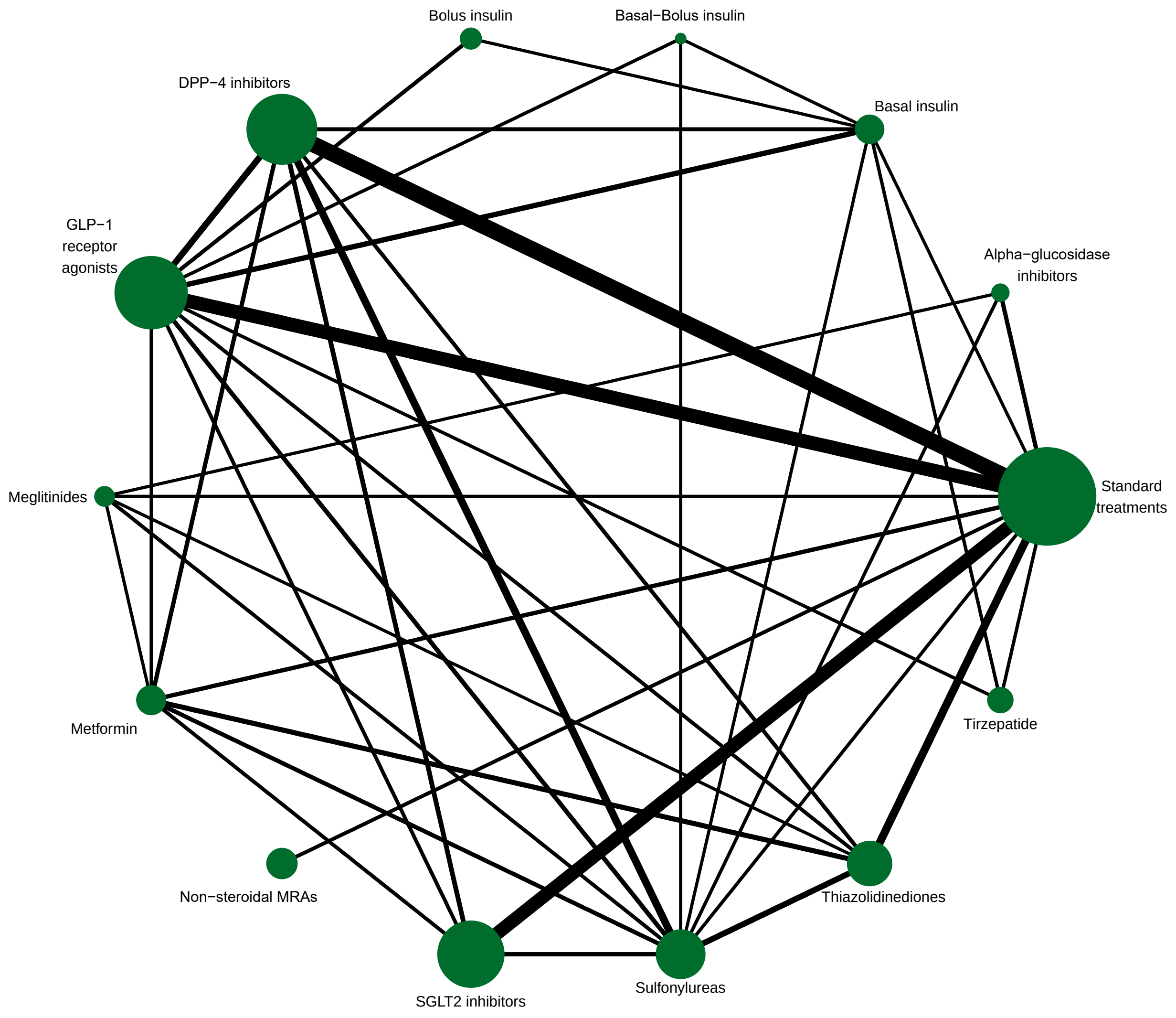

4.1.2 Cardiovascular death

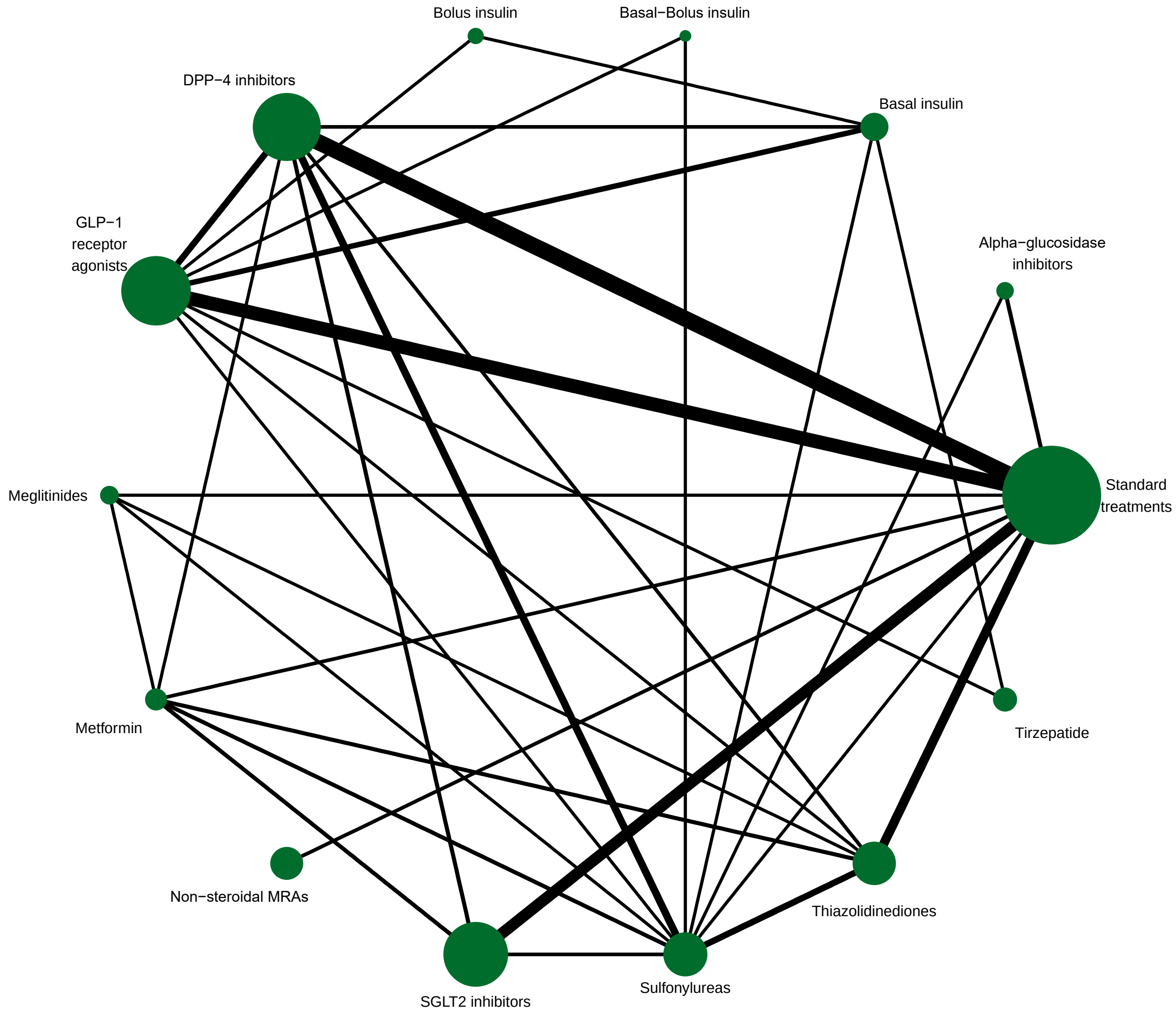

4.1.3 Non-fatal myocardial infarction

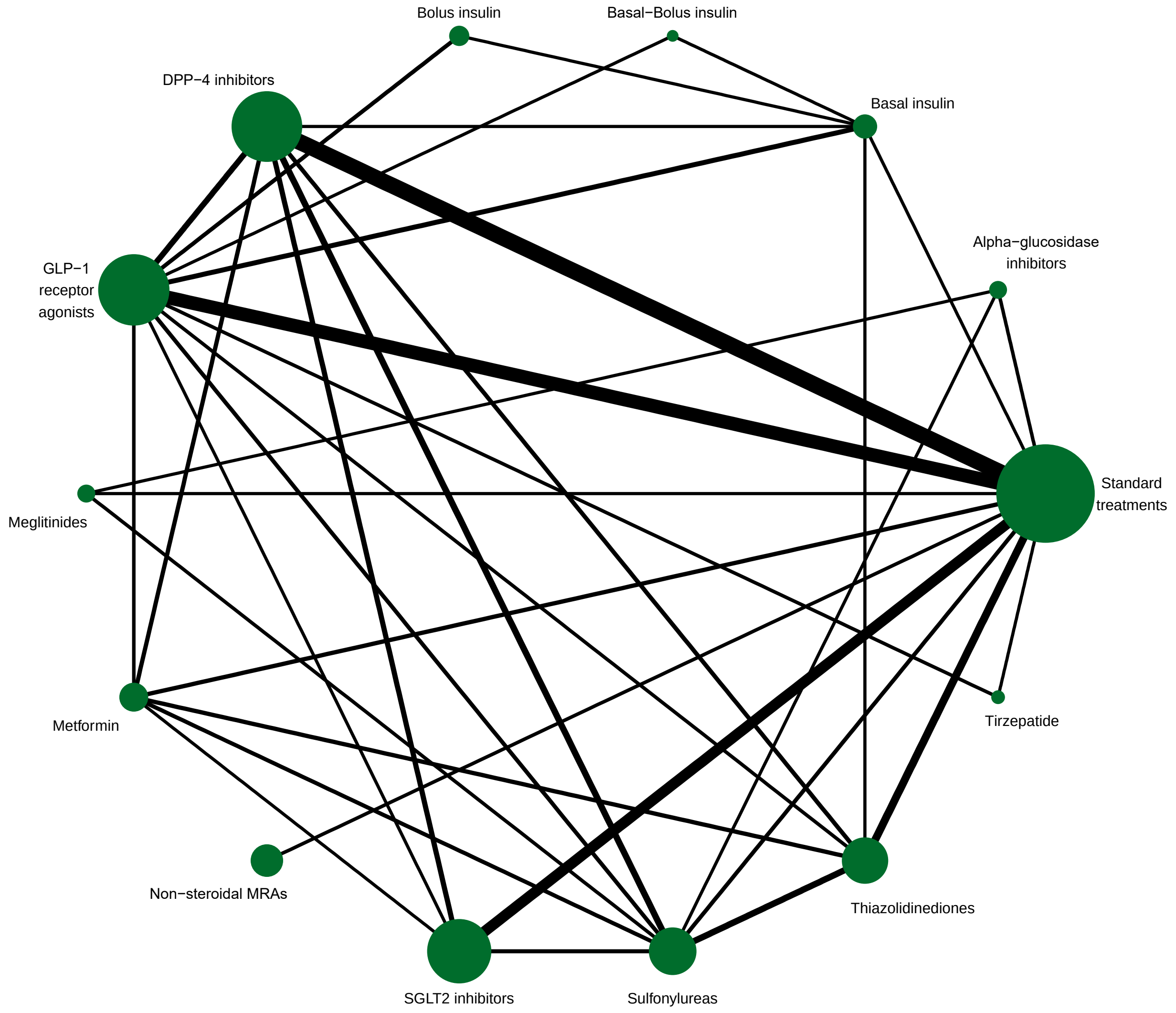

4.1.4 Non-fatal stroke

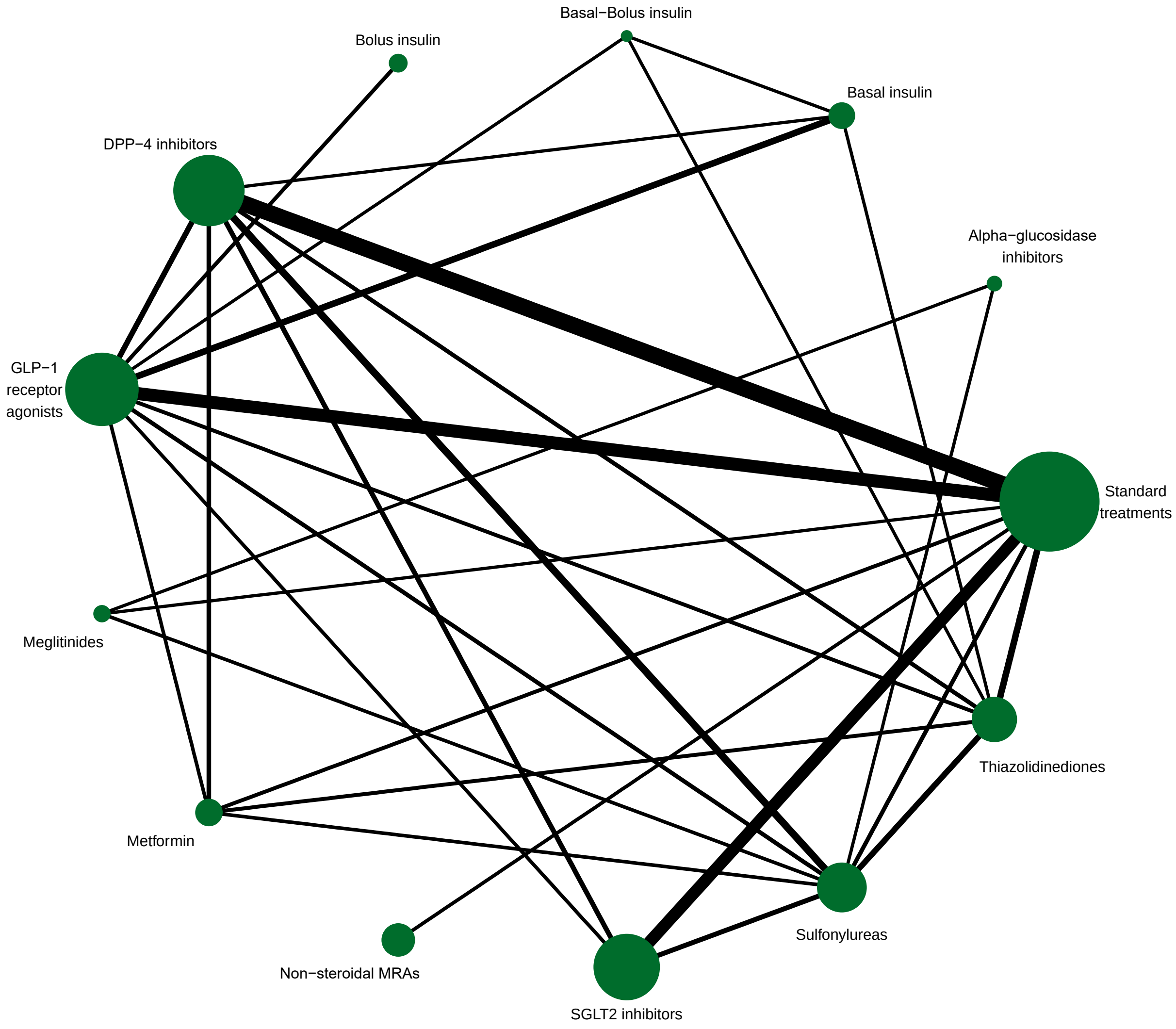

4.1.5 Hospitalization for heart failure

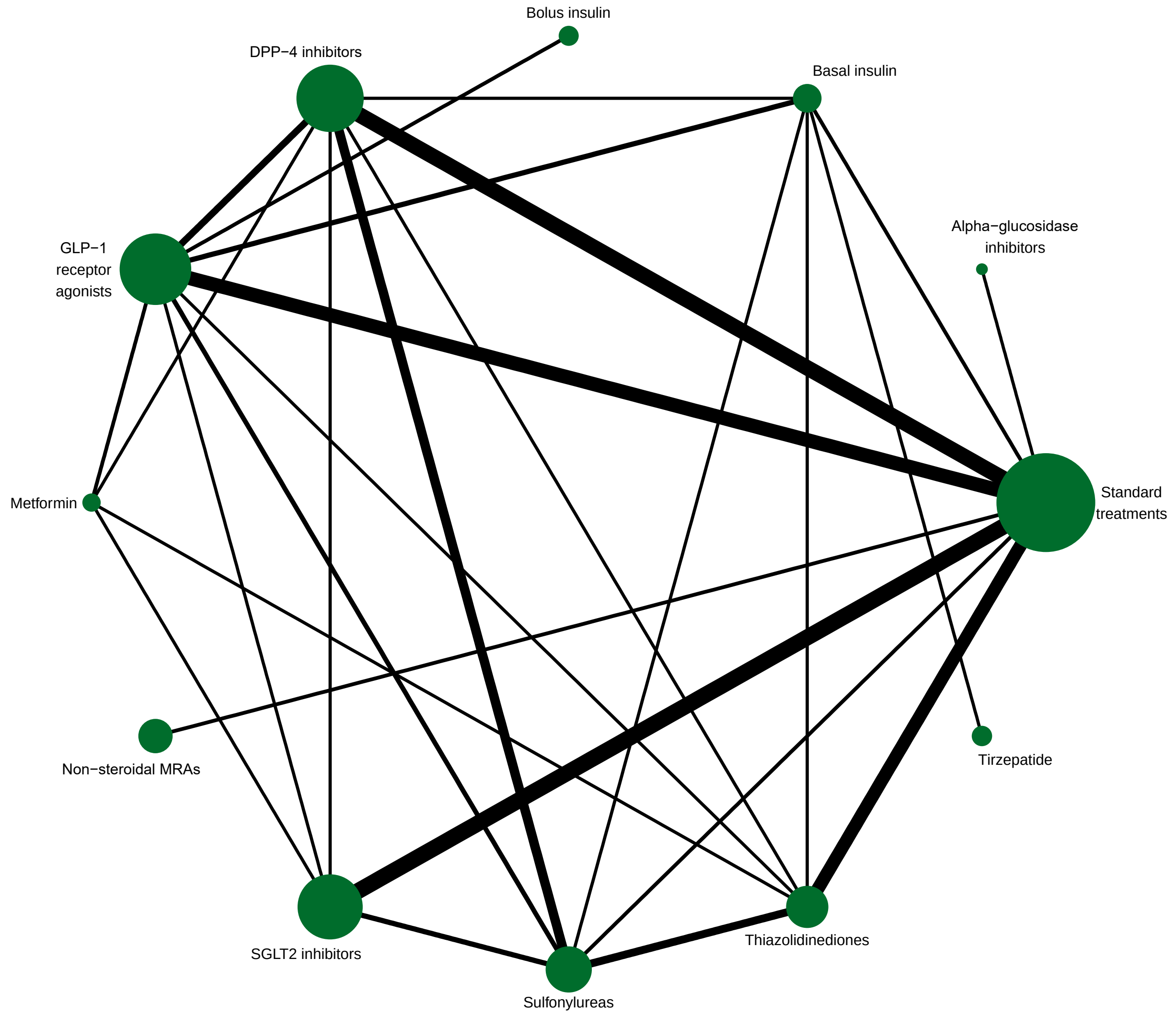

4.1.6 End-stage kidney disease

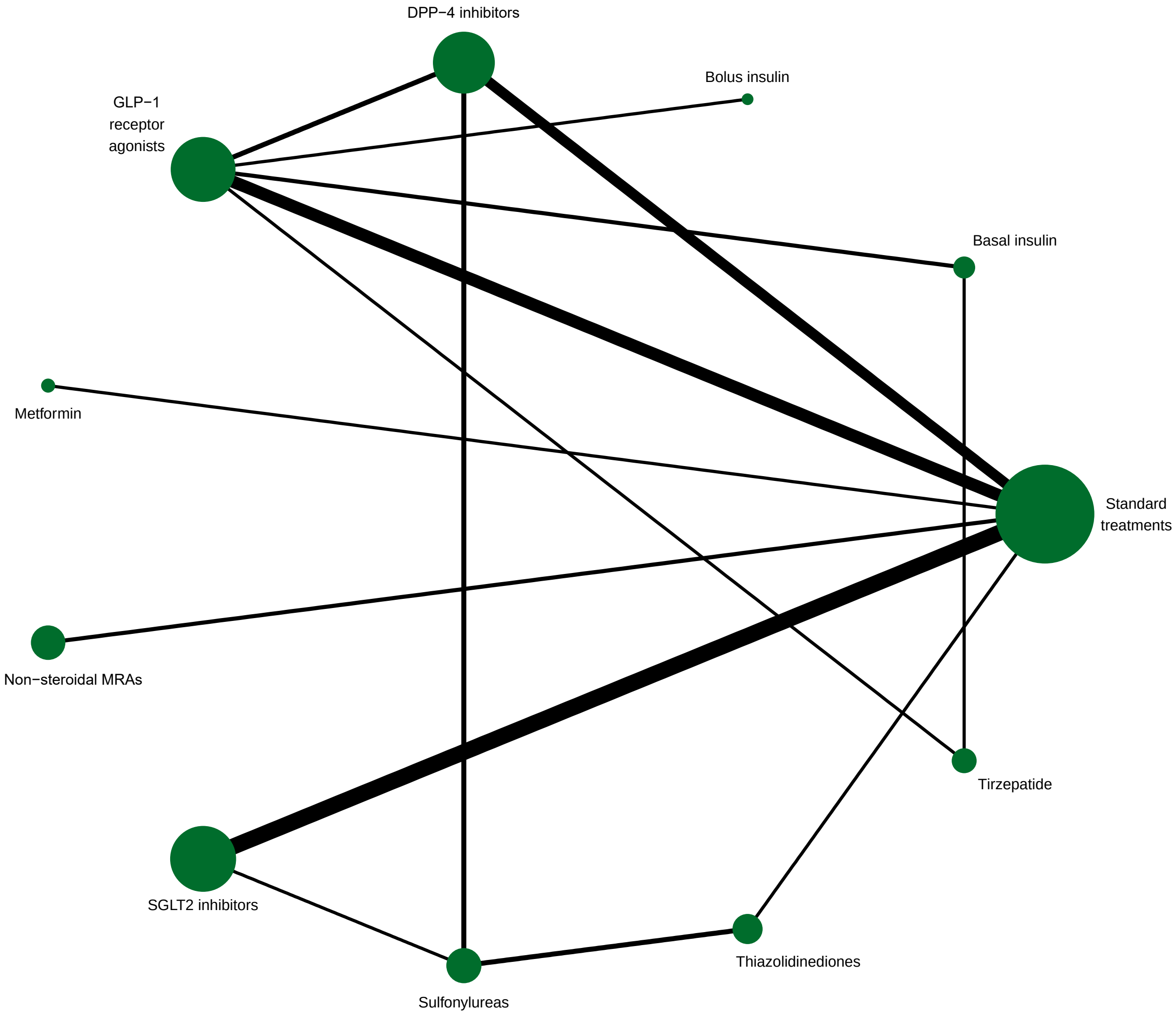

4.1.7 Severe Hypoglycaemia

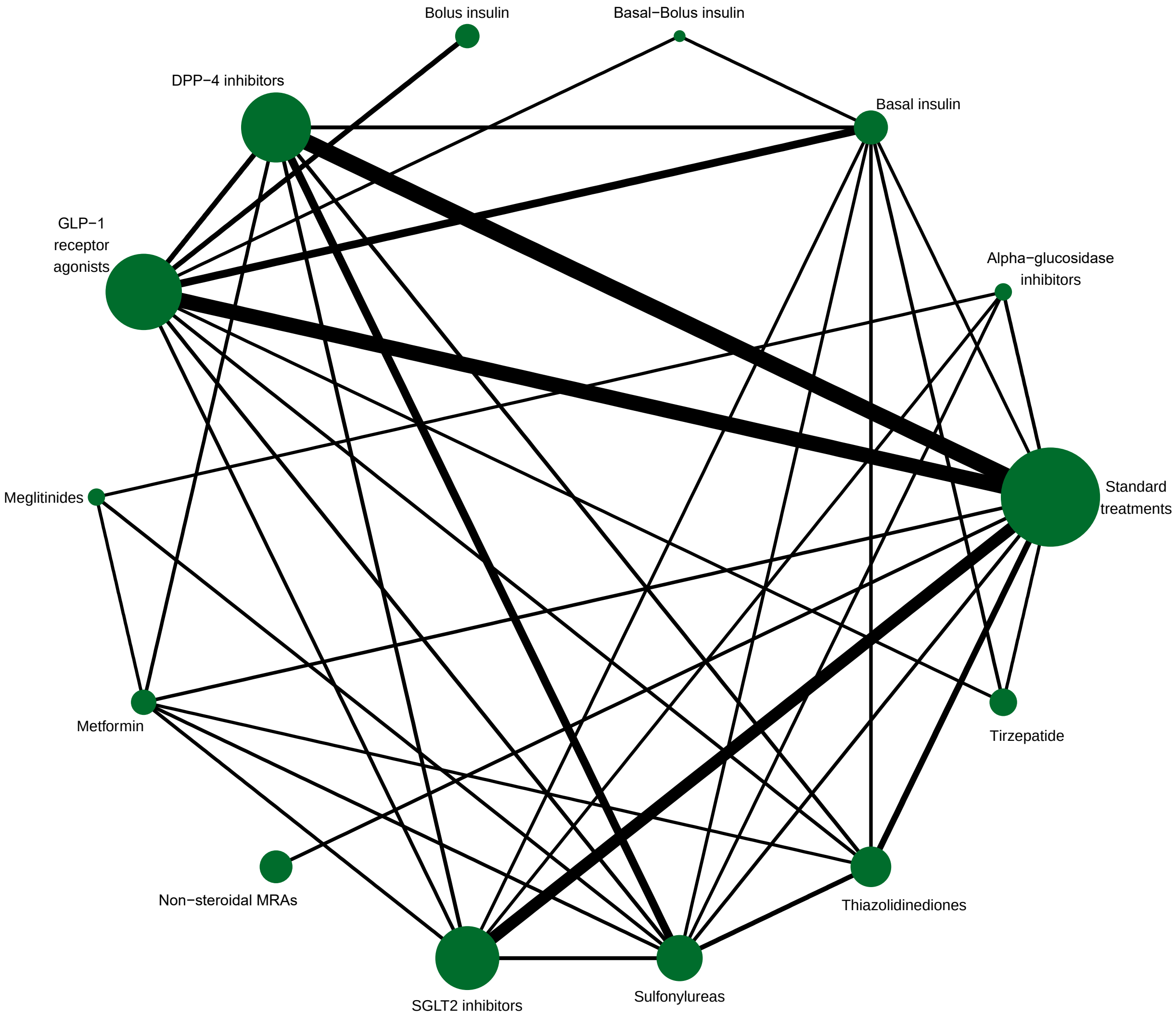

4.1.8 Severe gastrointestinal events

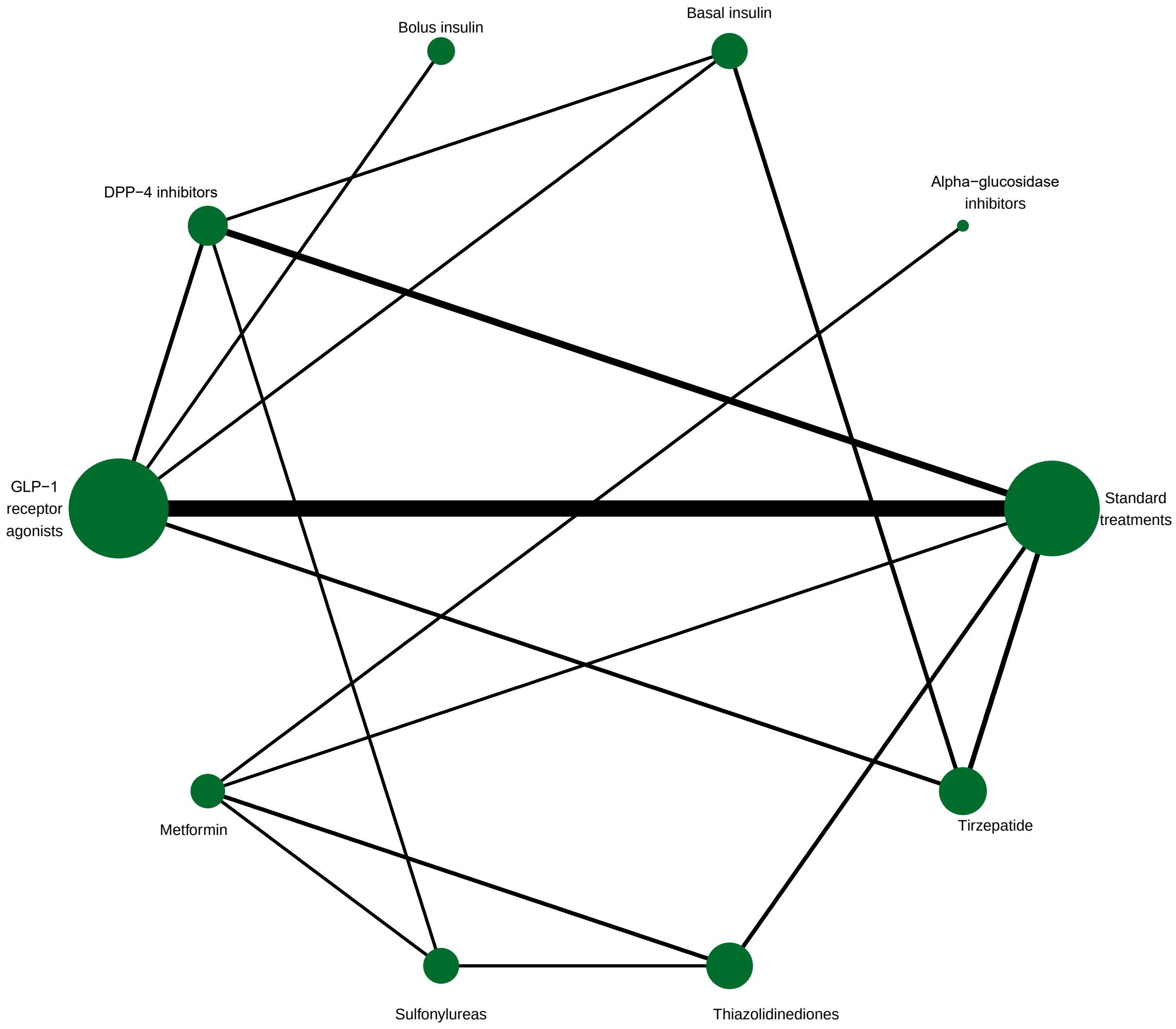

4.1.9 Genital infection

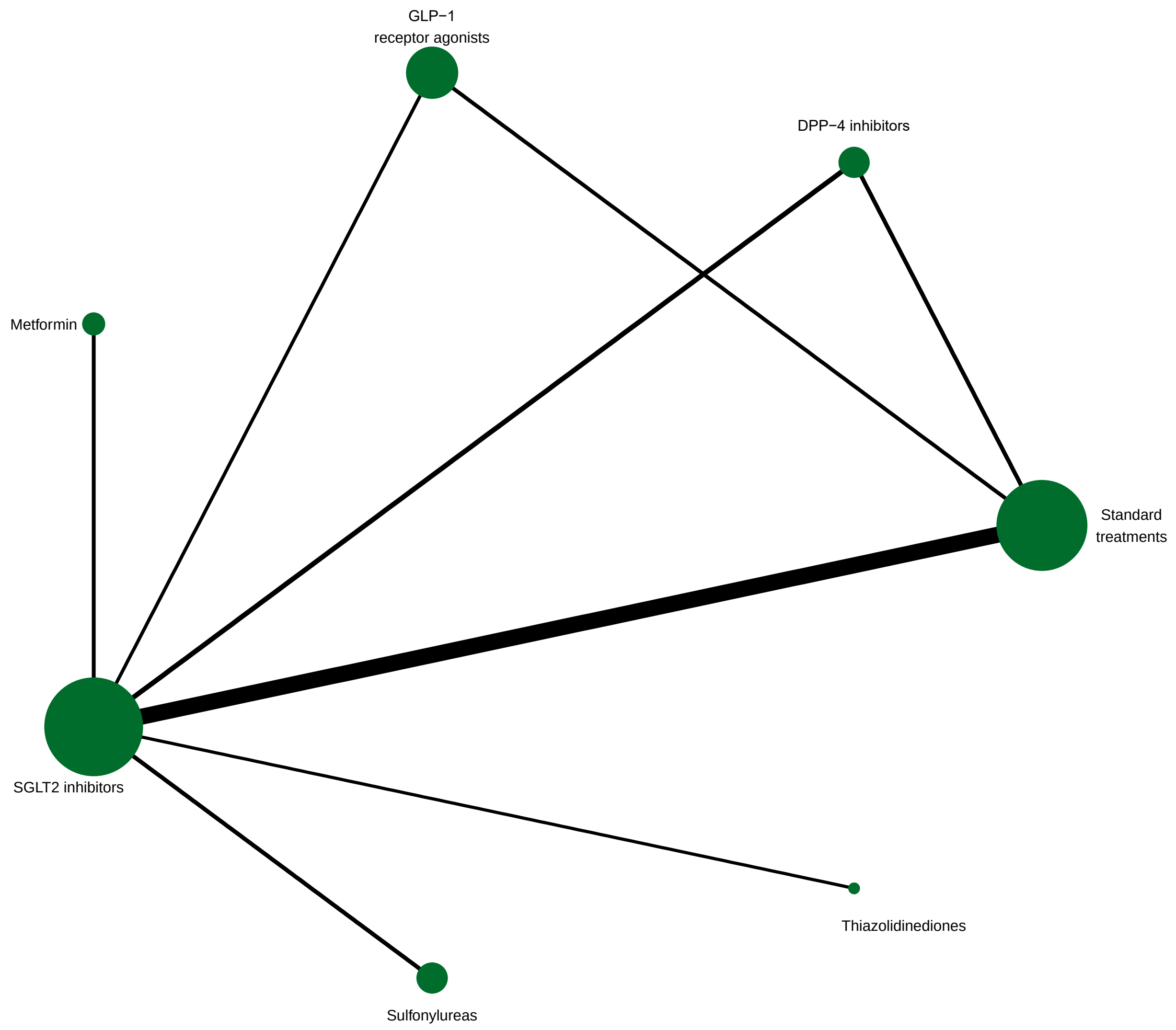

4.1.10 Amputation

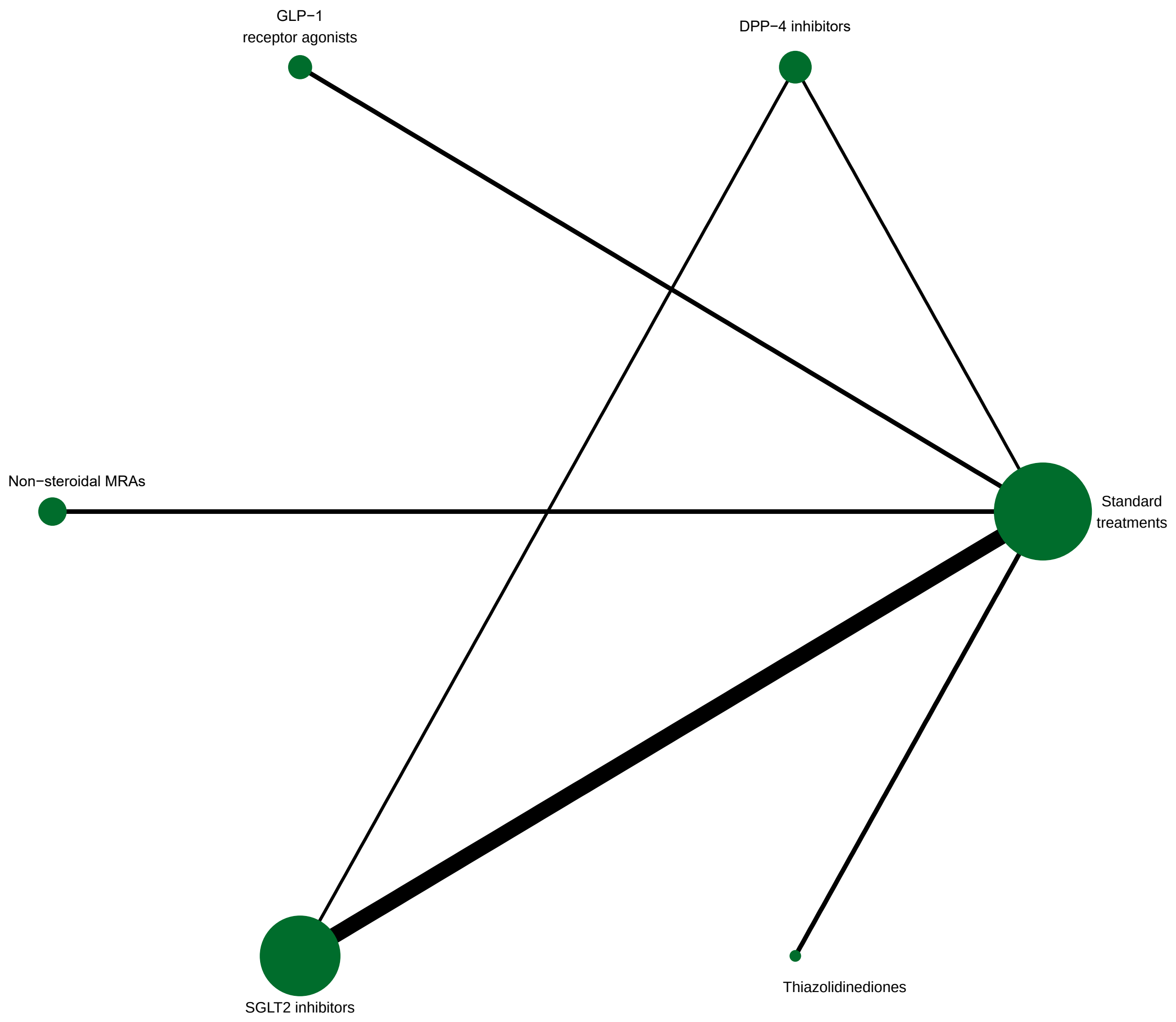

4.1.11 Ketoacidosis due to diabetes

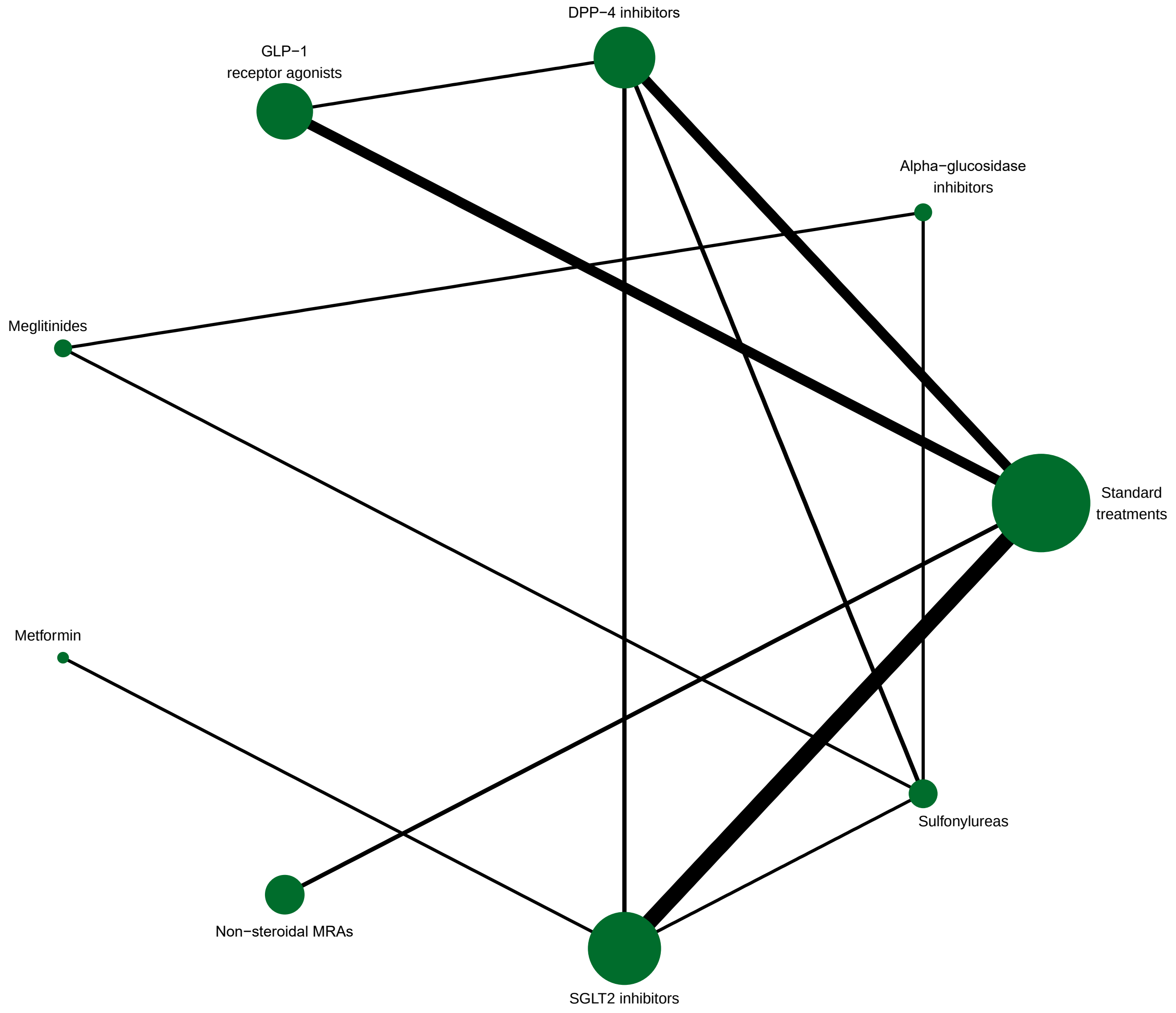

4.1.12 Health-related quality of life

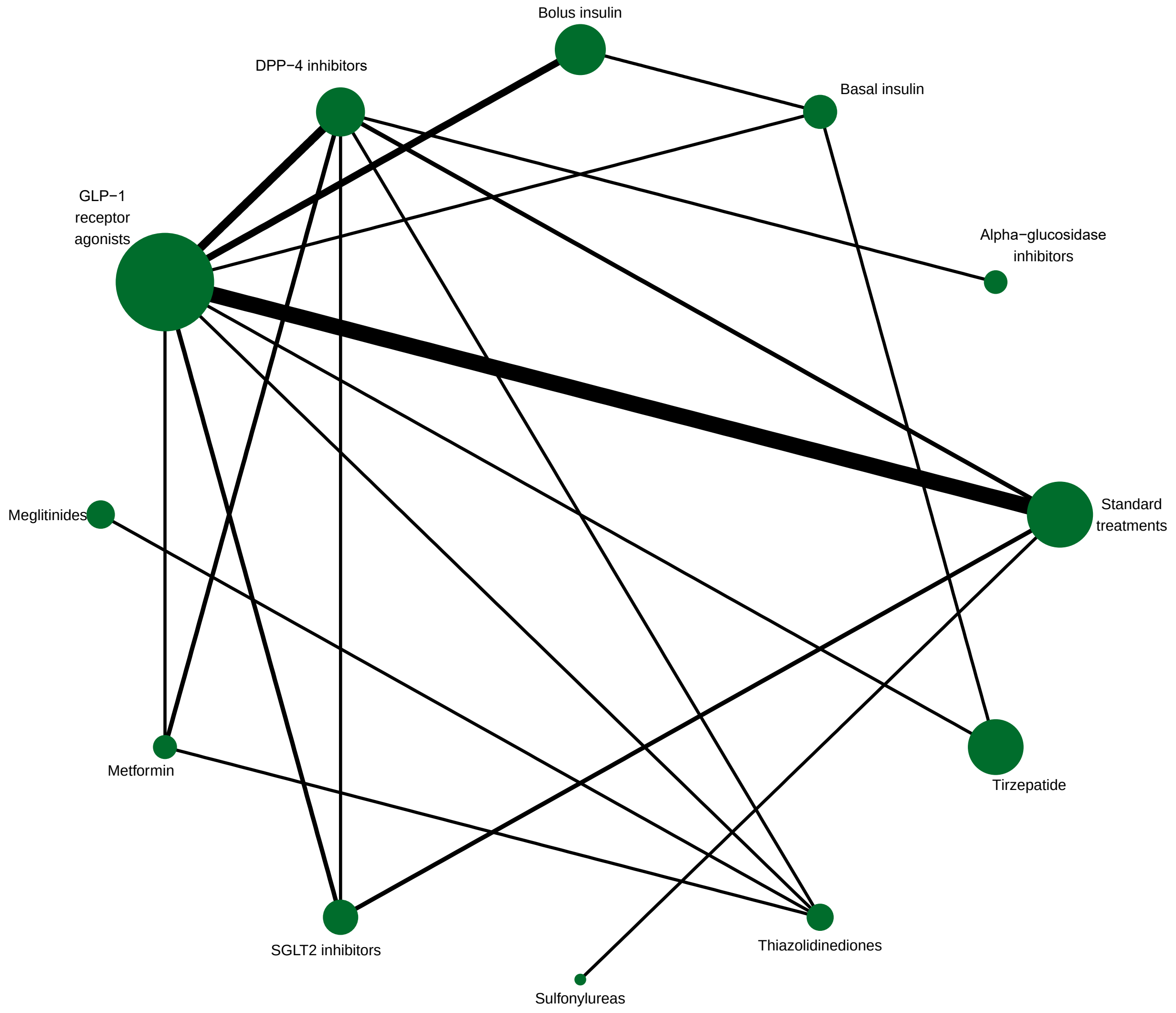

4.1.13 Bodyweight change

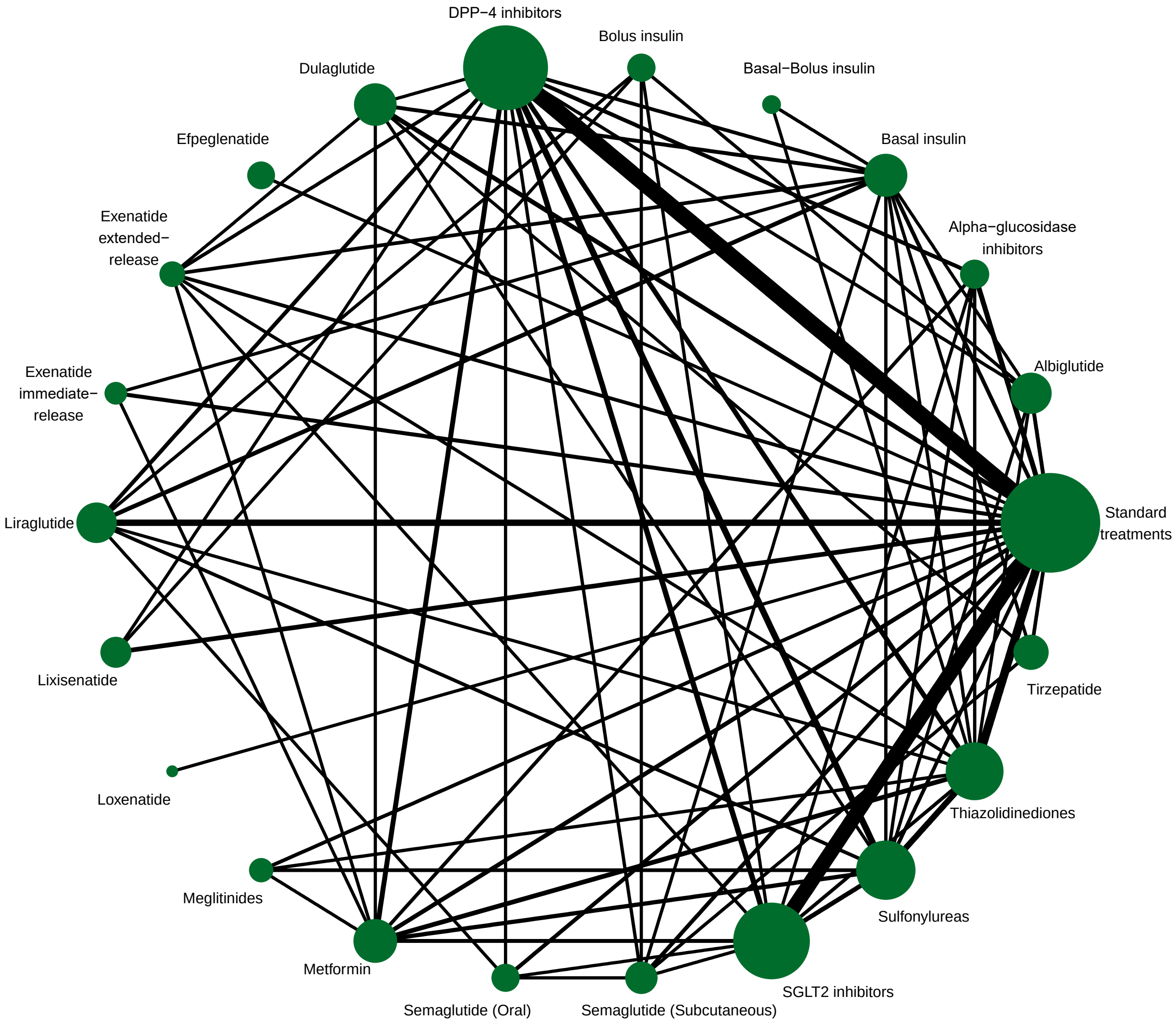

4.2 Forest plots

4.2.1 All-cause death

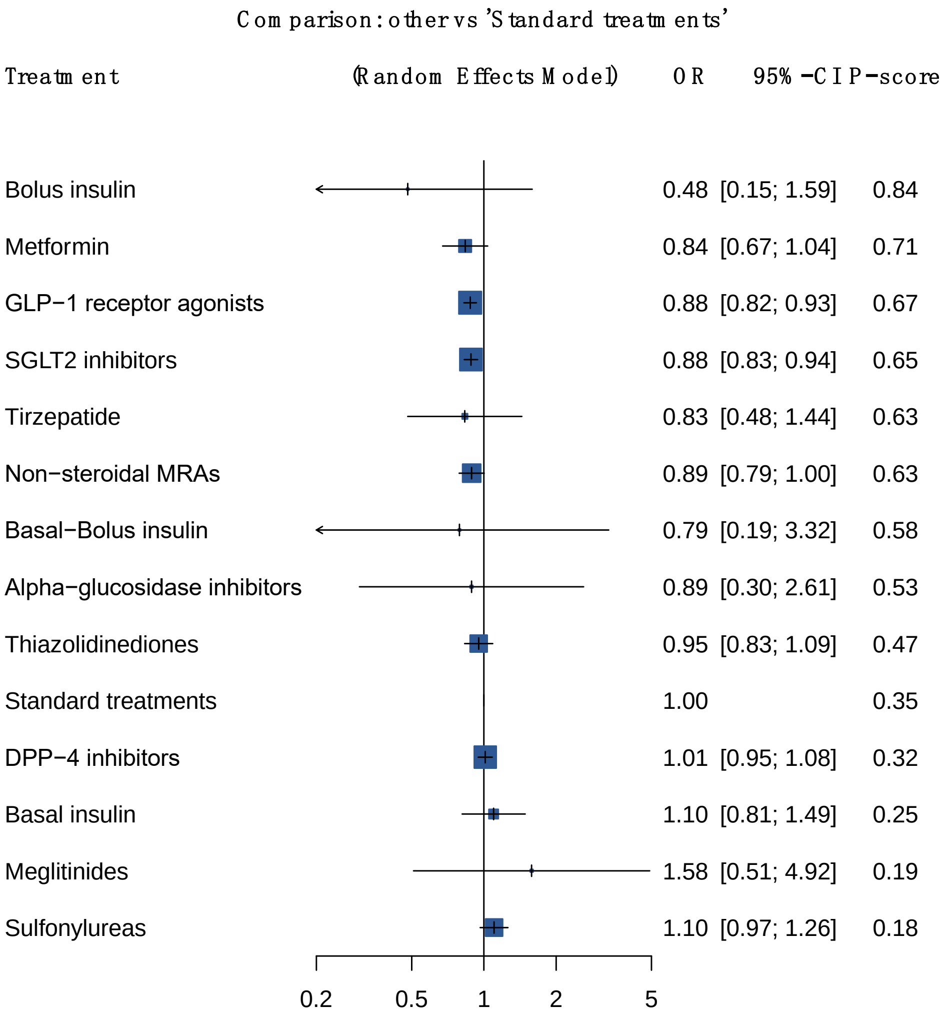

4.2.2 Cardiovascular death

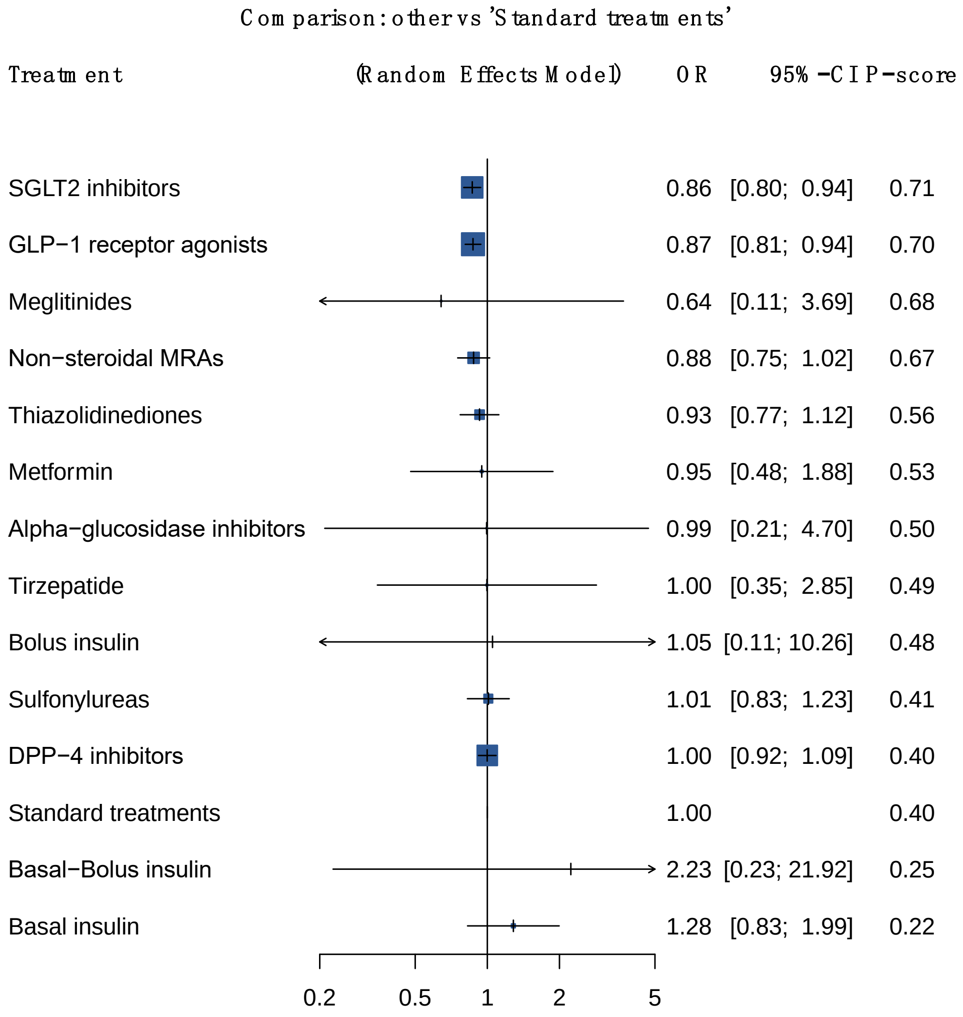

4.2.3 Non-fatal myocardial infarction

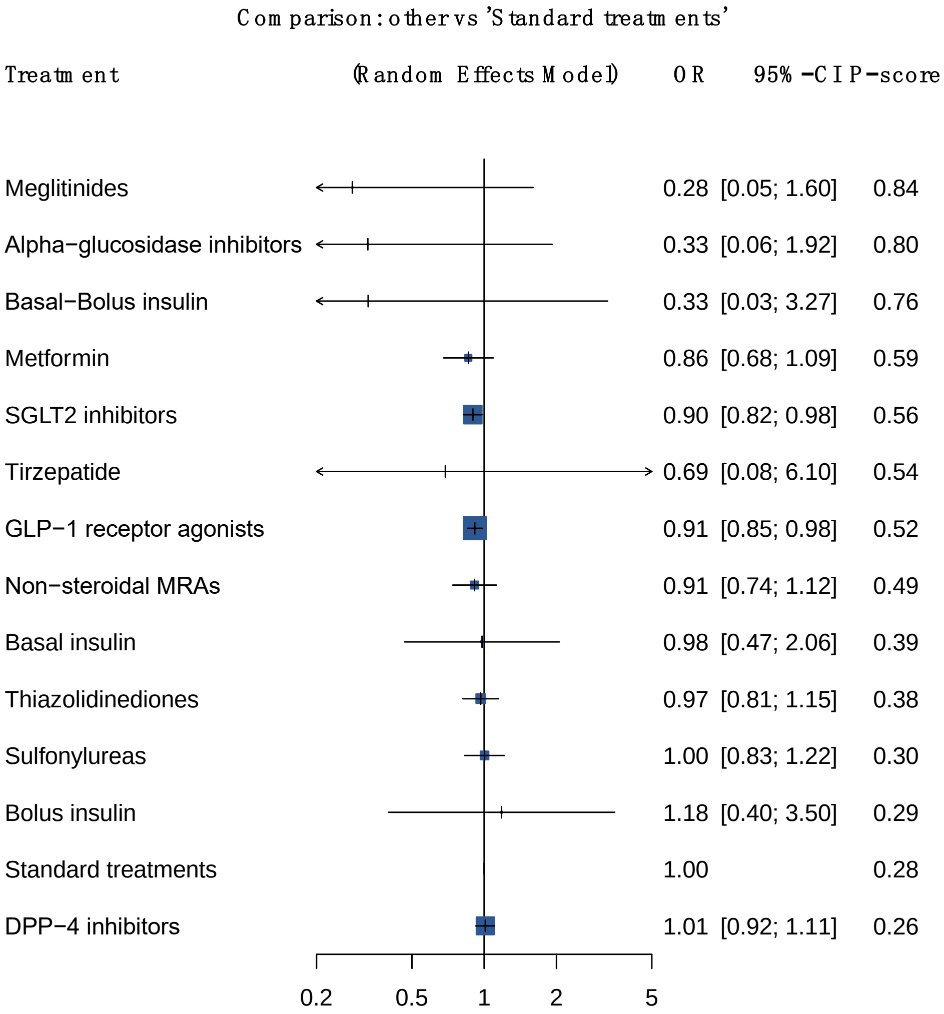

4.2.4 Non-fatal stroke

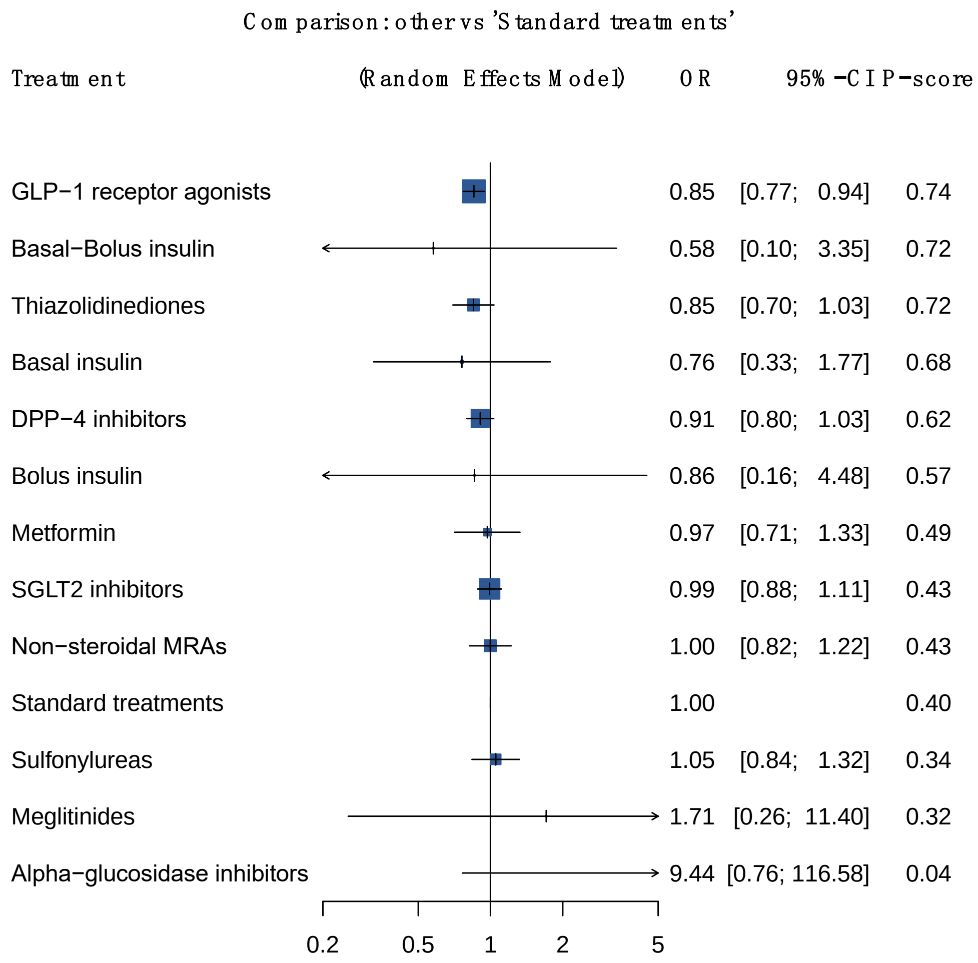

4.2.5 Hospitalization for heart failure

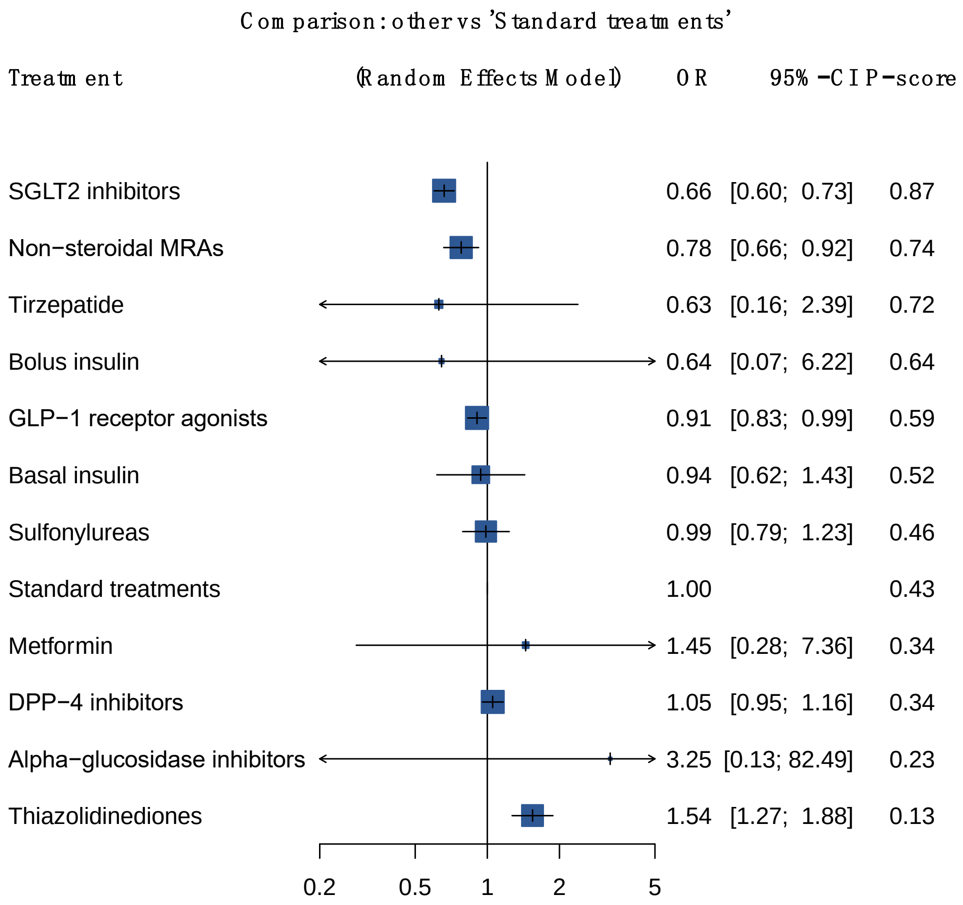

4.2.6 End-stage kidney disease

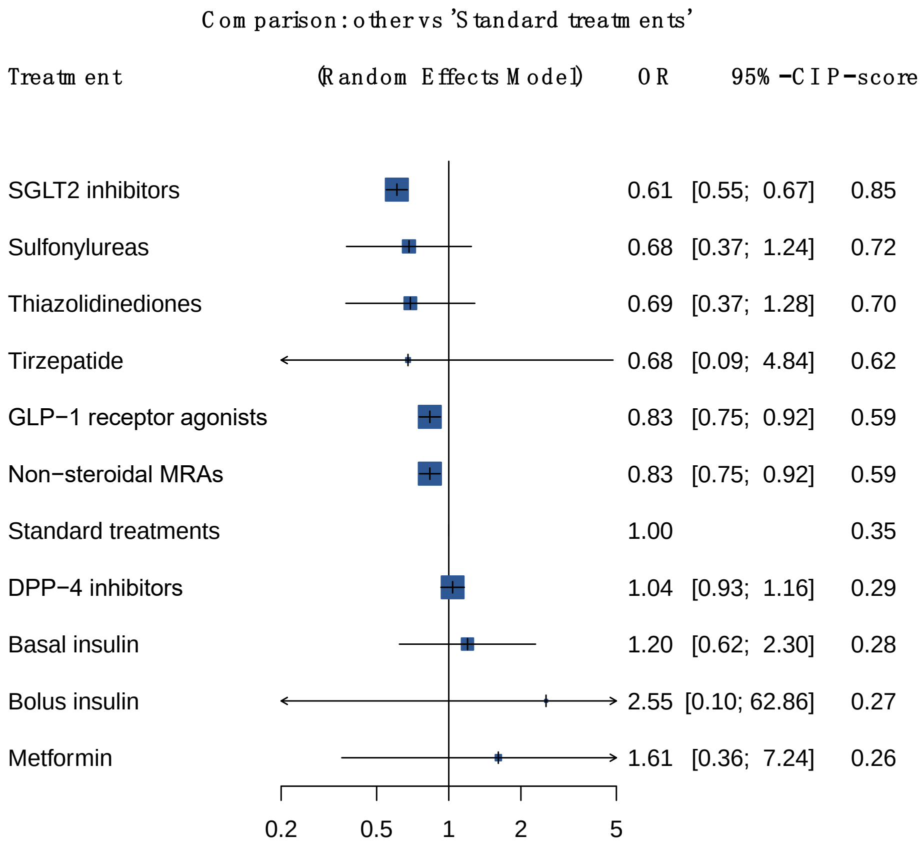

4.2.7 Severe hypoglycaemia

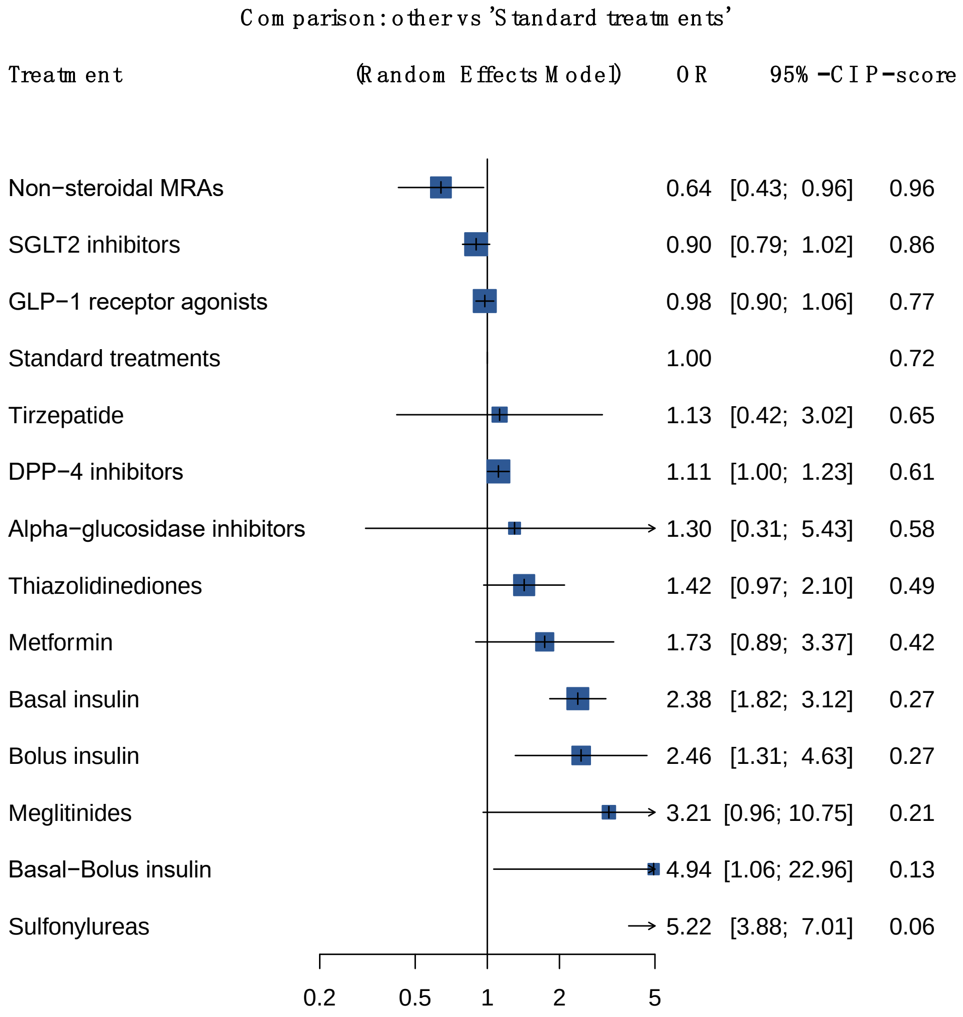

4.2.8 Severe gastrointestinal events

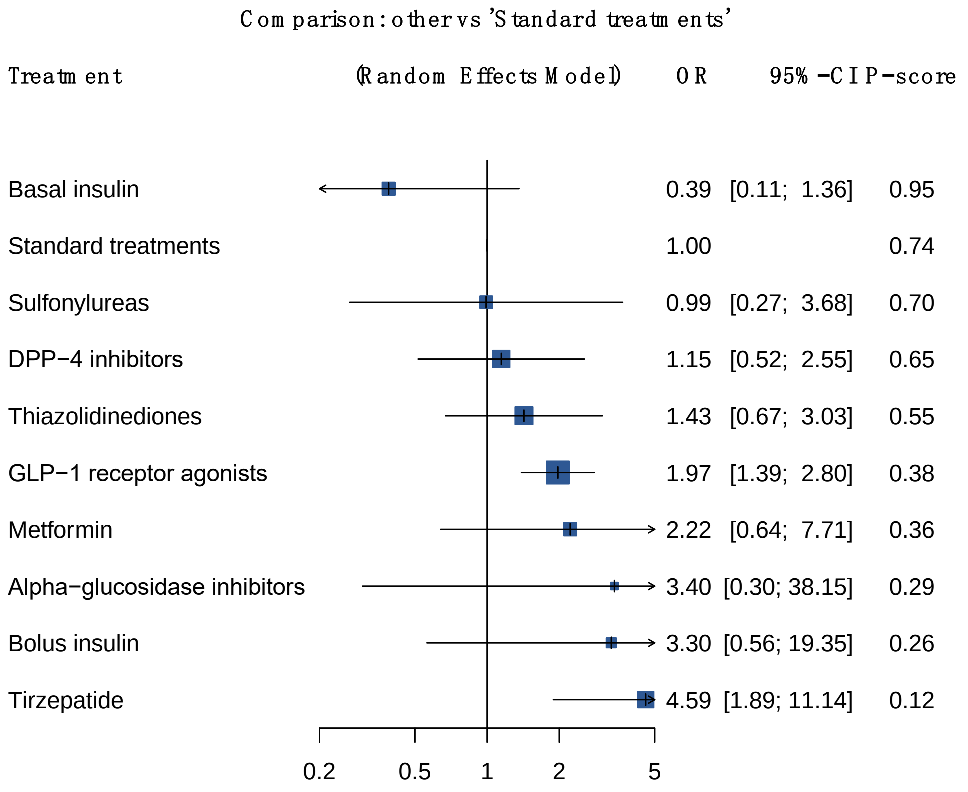

4.2.9 Genital infection

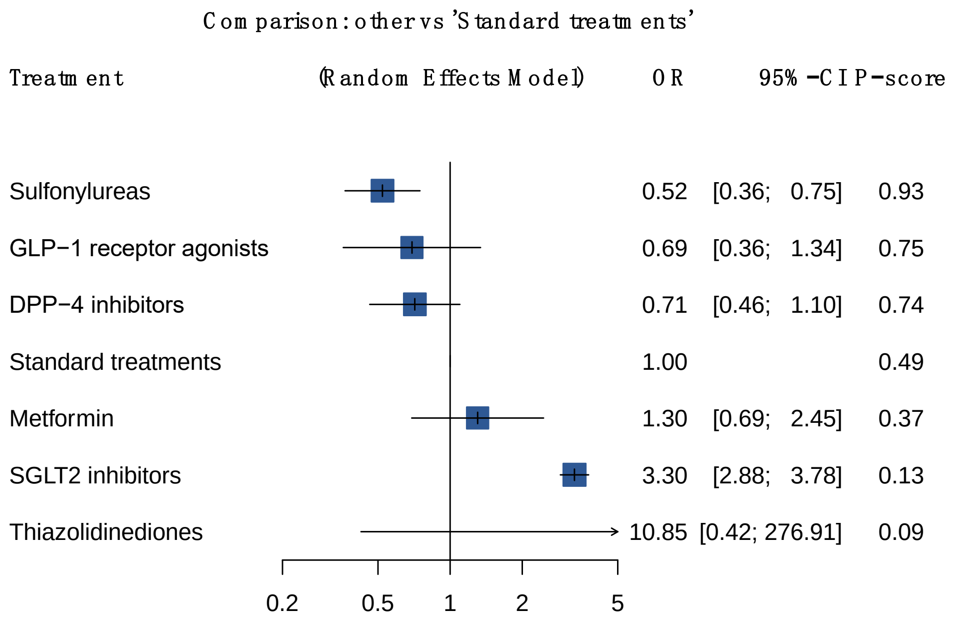

4.2.10 Amputation

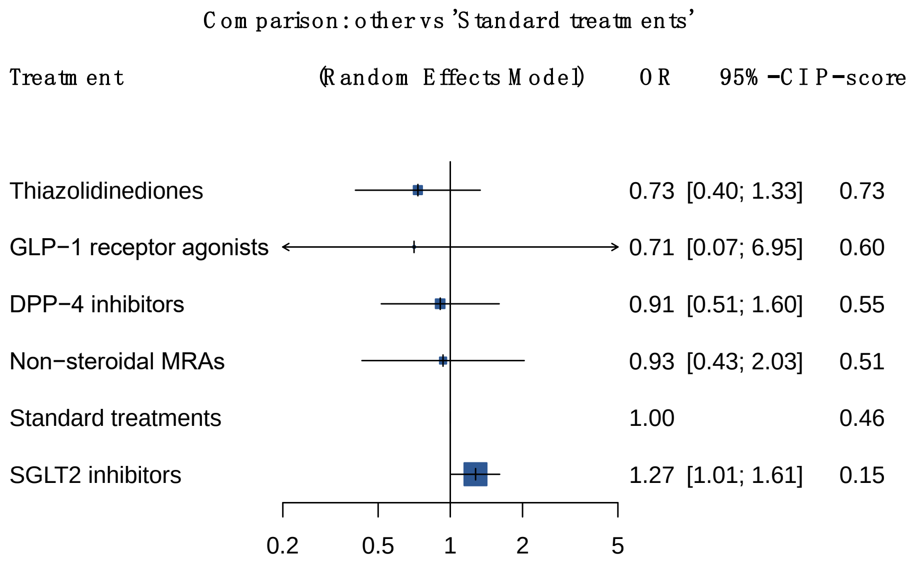

4.2.11 Ketoacidosis due to diabetes

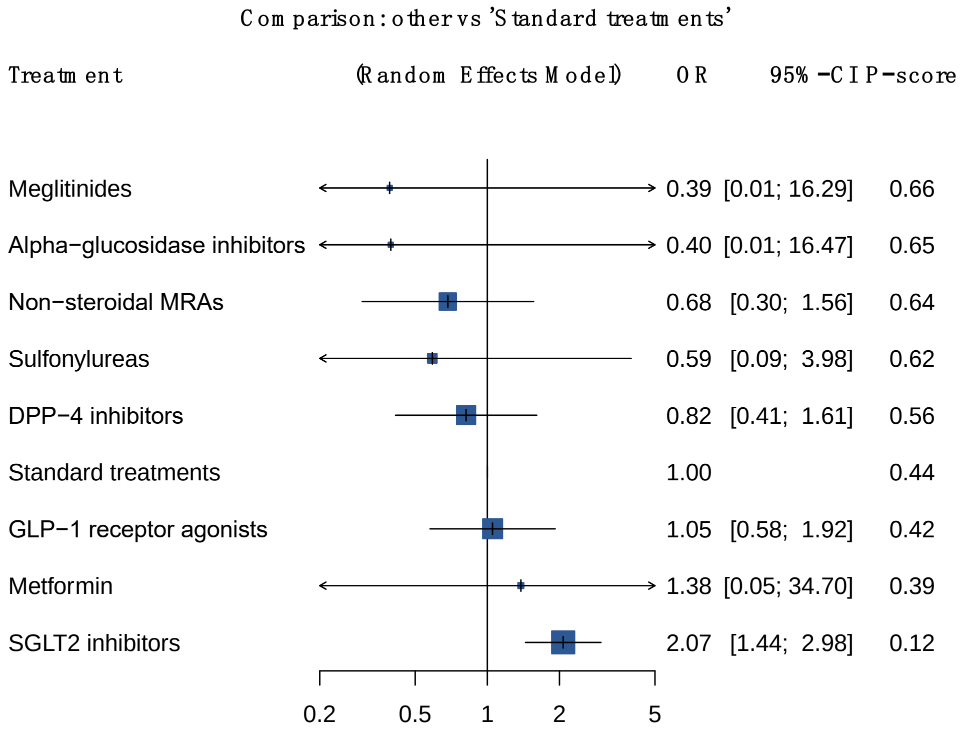

4.2.12 hyperkalaemia requiring intervention

Study	Experimental Events	Experimental Total	Control Events	Control Total	OR	95%-CI	Weight (common)	Weight (random)
FIDELIO−DKD	40	2827	8	2831	5.06	[2.37; 10.84]	79.8%	78.4%
FIGARO−DKD	21	3683	2	3658	10.48	[2.46; 44.74]	20.2%	21.6%
Common effect model		6510		6489	6.16	[3.15; 12.04]	100.0%	--
Random effects model					5.92	[3.02; 11.62]	--	100.0%

Heterogeneity: $I^2 = 0\%$, $\tau^2 = 0$, $p = 0.38$

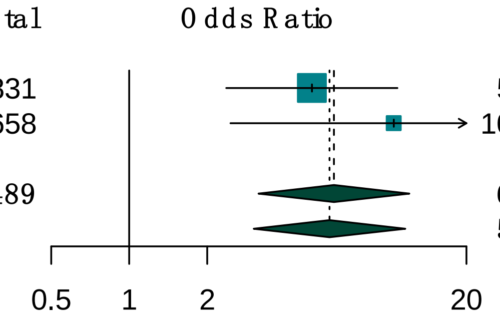

4.2.13 Health-related quality of life

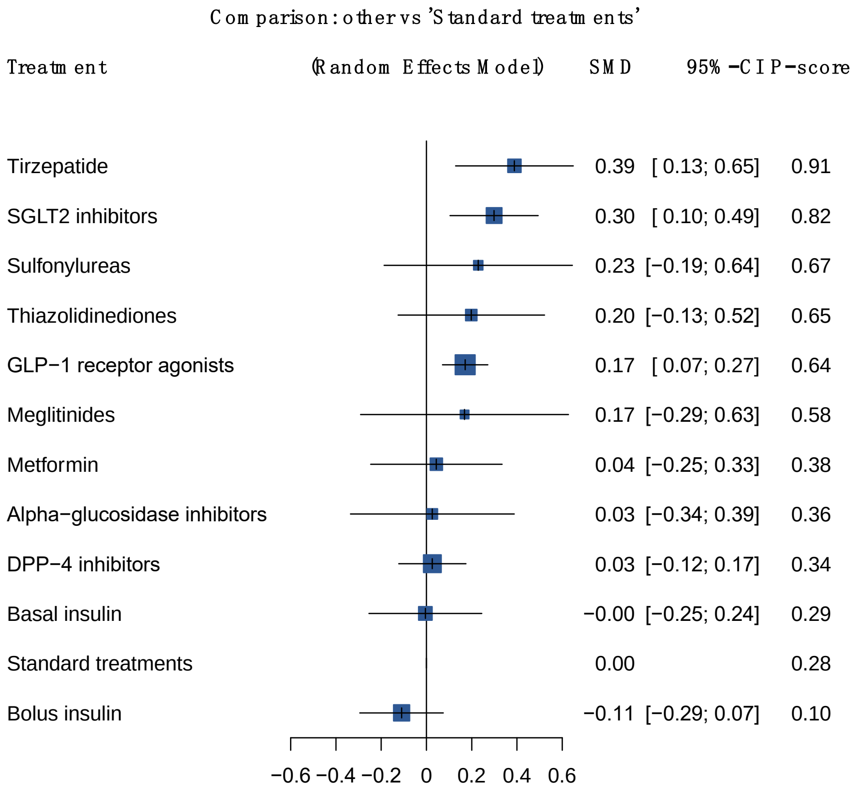

4.2.14 Bodyweight change

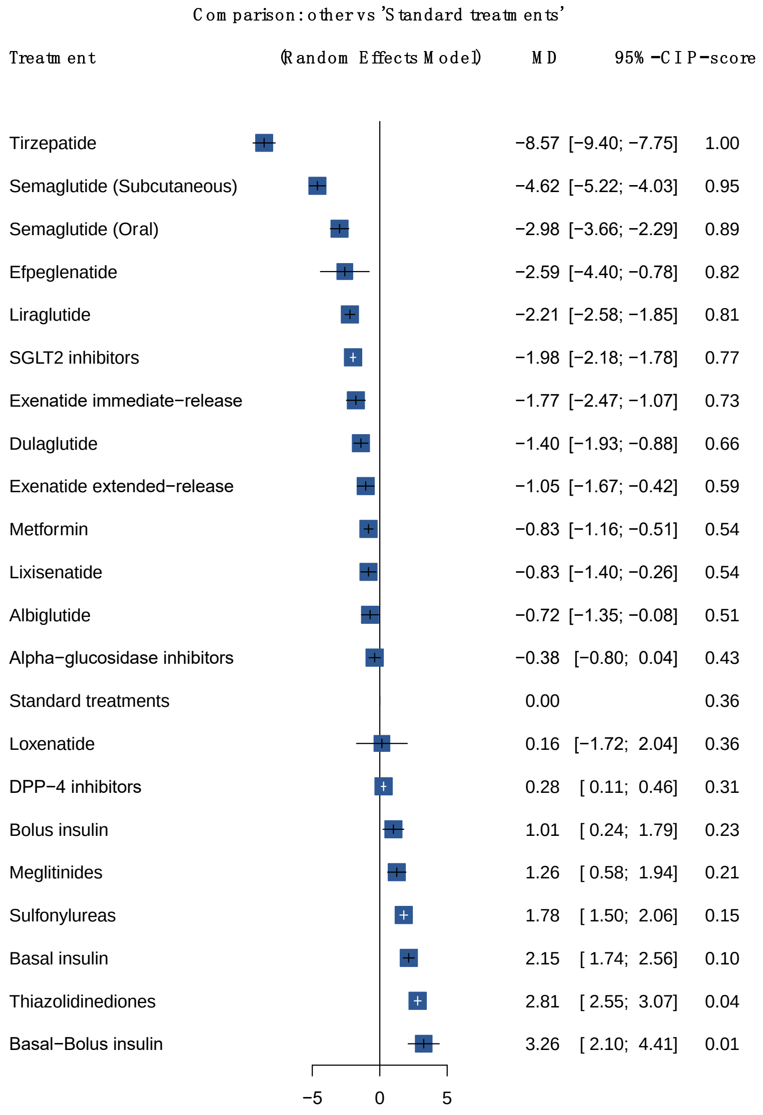

4.3 League tables

Notes: The figures show the relative effects of all diabetes drugs as well as standard treatments (the treatment of the column compared to the treatment of the row). The relative effects are measured using odds ratios for binary outcomes, a mean difference for bodyweight change, and a standardised mean difference for health-related quality of life score, along with the 95% confidence intervals. Bold indicates statistical significance. The colour of each cell indicates the certainty of evidence according to the Grading of Recommendations Assessment, Development, and Evaluation (GRADE) guideline. All tables list the treatments in alphabetical order.

Certainty of evidence

High **Moderate** **Low** **Very low**

4.3.1 Network estimates for all-cause death (lower left) and cardiovascular death (upper right)

**Alpha-glucosidase inhibitors**	1.30 (0.26, 6.67)	2.22 (0.14, 33.33)	1.06 (0.07, 16.67)	1.01 (0.21, 4.76)	0.88 (0.19, 4.17)	0.65 (0.06, 6.67)	0.95 (0.18, 5.26)	0.88 (0.19, 4.17)	0.87 (0.18, 4.17)	1.01 (0.21, 4.76)	1.02 (0.21, 4.76)	0.93 (0.20, 4.55)	1.00 (0.15, 6.67)
0.81 (0.26, 2.47)	**Basal insulin**	1.72 (0.17, 16.67)	0.82 (0.08, 7.69)	0.78 (0.50, 1.20)	0.68 (0.44, 1.05)	0.50 (0.08, 3.03)	0.74 (0.33, 1.64)	0.68 (0.43, 1.09)	0.68 (0.43, 1.05)	0.78 (0.50, 1.20)	0.79 (0.50, 1.25)	0.72 (0.45, 1.16)	0.78 (0.29, 2.08)
1.12 (0.19, 6.74)	1.39 (0.33, 5.92)	**Basal-Bolus insulin**	0.47 (0.02, 12.50)	0.45 (0.05, 4.35)	0.39 (0.04, 3.85)	0.29 (0.02, 5.00)	0.43 (0.04, 4.55)	0.39 (0.04, 3.85)	0.39 (0.04, 3.85)	0.45 (0.05, 4.35)	0.45 (0.05, 4.55)	0.42 (0.04, 4.17)	0.45 (0.04, 5.56)
1.85 (0.37, 9.23)	2.28 (0.67, 7.75)	1.64 (0.25, 10.61)	**Bolus insulin**	0.95 (0.10, 9.09)	0.83 (0.09, 8.33)	0.61 (0.03, 11.11)	0.90 (0.08, 10.00)	0.83 (0.09, 8.33)	0.82 (0.08, 8.33)	0.95 (0.10, 9.09)	0.96 (0.10, 9.09)	0.88 (0.09, 8.33)	0.94 (0.08, 11.11)
0.88 (0.30, 2.58)	1.08 (0.80, 1.47)	0.78 (0.19, 3.28)	0.47 (0.14, 1.57)	**DPP-4 inhibitors**	**0.87 (0.78, 0.98)**	0.64 (0.11, 3.70)	0.95 (0.48, 1.89)	0.88 (0.74, 1.04)	**0.86 (0.77, 0.97)**	1.00 (0.92, 1.09)	1.01 (0.84, 1.22)	0.93 (0.76, 1.14)	1.00 (0.35, 2.86)

1.01 (0.34, 2.98)	1.25 (0.93, 1.70)	0.90 (0.22, 3.78)	0.55 (0.17, 1.81)	**1.16 (1.06, 1.26)**	**GLP-1 receptor agonists**	0.74 (0.13, 4.17)	1.09 (0.55, 2.17)	1.01 (0.85, 1.19)	0.99 (0.88, 1.11)	**1.15 (1.06, 1.23)**	1.16 (0.93, 1.43)	1.06 (0.87, 1.30)	1.14 (0.40, 3.23)
0.56 (0.13, 2.48)	0.69 (0.22, 2.24)	0.50 (0.08, 3.11)	0.30 (0.06, 1.58)	0.64 (0.21, 1.99)	0.55 (0.18, 1.73)	**Meglitinides**	1.47 (0.24, 9.09)	1.37 (0.24, 7.69)	1.35 (0.23, 7.69)	1.56 (0.27, 9.09)	1.56 (0.27, 9.09)	1.45 (0.25, 8.33)	1.56 (0.20, 12.50)
1.06 (0.36, 3.18)	1.31 (0.91, 1.90)	0.95 (0.22, 4.04)	0.58 (0.17, 1.94)	1.21 (0.97, 1.52)	1.05 (0.84, 1.31)	1.89 (0.60, 5.97)	**Metformin**	0.93 (0.46, 1.85)	0.91 (0.46, 1.82)	1.05 (0.53, 2.08)	1.06 (0.54, 2.08)	0.98 (0.49, 1.96)	1.05 (0.30, 3.70)
1.00 (0.34, 2.95)	1.24 (0.89, 1.71)	0.89 (0.21, 3.75)	0.54 (0.16, 1.80)	1.14 (0.99, 1.31)	0.99 (0.86, 1.13)	1.78 (0.57, 5.57)	0.94 (0.73, 1.20)	**Non-steroidal MRAs**	0.99 (0.83, 1.18)	1.14 (0.98, 1.33)	1.15 (0.89, 1.49)	1.06 (0.83, 1.35)	1.14 (0.39, 3.33)
1.01 (0.34, 2.96)	1.24 (0.91, 1.70)	0.90 (0.21, 3.76)	0.54 (0.16, 1.81)	**1.15 (1.04, 1.26)**	0.99 (0.91, 1.08)	1.79 (0.58, 5.58)	0.95 (0.76, 1.19)	1.01 (0.88, 1.15)	**SGLT2 inhibitors**	**1.16 (1.06, 1.25)**	1.16 (0.94, 1.45)	1.08 (0.88, 1.32)	1.15 (0.40, 3.33)
0.89 (0.30, 2.61)	1.10 (0.81, 1.49)	0.79 (0.19, 3.32)	0.48 (0.15, 1.59)	1.01 (0.95, 1.08)	**0.88 (0.82, 0.93)**	1.58 (0.51, 4.92)	0.84 (0.67, 1.04)	**0.89 (0.79, 1.00)**	**0.88 (0.83, 0.94)**	**Standard treatments**	1.01 (0.83, 1.23)	0.93 (0.77, 1.12)	1.00 (0.35, 2.85)
0.81 (0.27, 2.37)	1.00 (0.73, 1.36)	0.72 (0.17, 3.02)	0.44 (0.13, 1.45)	0.92 (0.81, 1.04)	**0.79 (0.69, 0.92)**	1.43 (0.46, 4.45)	**0.76 (0.60, 0.96)**	**0.81 (0.67, 0.96)**	**0.80 (0.69, 0.93)**	0.91 (0.79, 1.03)	**Sulfonylureas**	0.92 (0.71, 1.19)	0.99 (0.34, 2.86)
0.93 (0.32, 2.76)	1.15 (0.83, 1.60)	0.83 (0.20, 3.51)	0.51 (0.15, 1.68)	1.06 (0.92, 1.23)	0.92 (0.80, 1.07)	1.66 (0.53, 5.19)	0.88 (0.69, 1.12)	0.93 (0.78, 1.12)	0.93 (0.80, 1.08)	1.05 (0.92, 1.20)	1.16 (0.98, 1.37)	**Thiazolidinediones**	1.08 (0.37, 3.12)
1.07 (0.32, 3.57)	1.32 (0.82, 2.13)	0.95 (0.21, 4.35)	0.58 (0.16, 2.13)	1.22 (0.70, 2.11)	1.05 (0.61, 1.82)	1.90 (0.54, 6.68)	1.00 (0.56, 1.81)	1.07 (0.61, 1.87)	1.06 (0.61, 1.84)	1.20 (0.69, 2.08)	1.32 (0.76, 2.31)	1.14 (0.65, 2.01)	**Tirzepatide**

4.3.2 Network estimates for non-fatal myocardial infarction (lower left) and non-fatal stroke (upper right)

**Alpha-glucosidase inhibitors**	0.08 (0.01, 1.14)	0.06 (0.00, 1.32)	0.09 (0.00, 1.85)	0.10 (0.01, 1.19)	0.09 (0.01, 1.11)	0.18 (0.01, 2.22)	0.10 (0.01, 1.28)	0.11 (0.01, 1.32)	0.11 (0.01, 1.30)	0.11 (0.01, 1.32)	0.11 (0.01, 1.37)	0.09 (0.01, 1.11)	—
0.33 (0.05, 2.28)	**Basal insulin**	0.76 (0.13, 4.35)	1.12 (0.18, 7.14)	1.19 (0.51, 2.78)	1.12 (0.48, 2.63)	2.22 (0.28, 16.67)	1.28 (0.52, 3.12)	1.32 (0.55, 3.12)	1.30 (0.56, 3.03)	1.32 (0.56, 3.03)	1.39 (0.58, 3.33)	1.11 (0.47, 2.63)	—
1.00 (0.05, 18.12)	2.98 (0.30, 29.67)	**Basal-Bolus insulin**	1.49 (0.13, 16.67)	1.56 (0.27, 9.09)	1.47 (0.26, 8.33)	2.94 (0.22, 33.33)	1.67 (0.28, 10.00)	1.72 (0.29, 10.00)	1.72 (0.29, 10.00)	1.72 (0.30, 10.00)	1.82 (0.31, 11.11)	1.47 (0.25, 8.33)	—
0.28 (0.03, 2.21)	0.83 (0.28, 2.50)	0.28 (0.02, 3.35)	**Bolus insulin**	1.05 (0.20, 5.56)	0.99 (0.19, 5.26)	2.00 (0.16, 25.00)	1.14 (0.21, 6.25)	1.16 (0.22, 6.25)	1.15 (0.22, 6.25)	1.16 (0.22, 6.25)	1.22 (0.23, 6.67)	0.99 (0.19, 5.26)	—
0.32 (0.06, 1.90)	0.97 (0.46, 2.05)	0.32 (0.03, 3.24)	1.17 (0.39, 3.48)	**DPP-4 inhibitors**	0.94 (0.80, 1.10)	1.89 (0.28, 12.50)	1.08 (0.78, 1.49)	1.10 (0.87, 1.39)	1.10 (0.93, 1.30)	1.10 (0.97, 1.25)	1.16 (0.93, 1.45)	0.93 (0.75, 1.18)	—
0.36 (0.06, 2.10)	1.07 (0.51, 2.25)	0.36 (0.04, 3.57)	1.29 (0.44, 3.82)	1.11 (0.99, 1.24)	**GLP-1 receptor agonists**	2.00 (0.30, 14.29)	1.14 (0.82, 1.59)	1.18 (0.93, 1.47)	**1.16 (1.00, 1.35)**	**1.18 (1.06, 1.30)**	1.23 (0.96, 1.59)	1.00 (0.80, 1.25)	—
1.16 (0.10, 12.83)	3.47 (0.53, 22.89)	1.16 (0.07, 20.69)	4.19 (0.54, 32.39)	3.58 (0.63, 20.28)	3.24 (0.57, 18.37)	**Meglitinides**	0.57 (0.08, 3.85)	0.58 (0.09, 4.00)	0.58 (0.09, 3.85)	0.58 (0.09, 3.85)	0.62 (0.09, 4.17)	0.50 (0.07, 3.33)	—
0.38 (0.06, 2.27)	1.14 (0.52, 2.49)	0.38 (0.04, 3.85)	1.37 (0.45, 4.18)	1.18 (0.91, 1.51)	1.06 (0.83, 1.36)	0.33 (0.06, 1.89)	**Metformin**	1.03 (0.71, 1.49)	1.02 (0.73, 1.43)	1.03 (0.75, 1.41)	1.09 (0.77, 1.52)	0.88 (0.62, 1.23)	—
0.36 (0.06, 2.13)	1.08 (0.50, 2.33)	0.36 (0.04, 3.62)	1.30 (0.43, 3.92)	1.11 (0.88, 1.39)	1.00 (0.80, 1.25)	0.31 (0.05, 1.78)	0.94 (0.69, 1.30)	**Non-steroidal MRAs**	0.99 (0.79, 1.25)	1.00 (0.82, 1.22)	1.05 (0.78, 1.43)	0.85 (0.64, 1.12)	—
0.36 (0.06, 2.14)	1.09 (0.52, 2.31)	0.37 (0.04, 3.65)	1.32 (0.44, 3.92)	1.13 (0.99, 1.28)	1.02 (0.91, 1.14)	0.31 (0.06, 1.79)	0.96 (0.74, 1.24)	1.02 (0.81, 1.27)	**SGLT2 inhibitors**	1.01 (0.90, 1.14)	1.06 (0.83, 1.37)	0.85 (0.68, 1.08)	—
0.33 (0.06, 1.92)	0.98 (0.47, 2.06)	0.33 (0.03, 3.27)	1.18 (0.40, 3.50)	1.01 (0.92, 1.11)	**0.91 (0.85, 0.98)**	0.28 (0.05, 1.60)	0.86 (0.68, 1.09)	0.91 (0.74, 1.12)	**0.90 (0.82, 0.98)**	**Standard treatments**	1.05 (0.84, 1.32)	0.85 (0.70, 1.03)	—

0.33 (0.06, 1.92)	0.98 (0.45, 2.10)	0.33 (0.03, 3.28)	1.18 (0.39, 3.55)	1.01 (0.84, 1.21)	0.91 (0.74, 1.12)	0.28 (0.05, 1.59)	0.86 (0.64, 1.14)	0.91 (0.68, 1.21)	0.89 (0.72, 1.10)	1.00 (0.82, 1.20)	**Sulfonylureas**	0.81 (0.62, 1.04)	—
0.34 (0.06, 2.00)	1.01 (0.47, 2.17)	0.34 (0.03, 3.40)	1.22 (0.41, 3.67)	1.04 (0.87, 1.26)	0.95 (0.79, 1.14)	0.29 (0.05, 1.66)	0.89 (0.67, 1.17)	0.94 (0.72, 1.24)	0.93 (0.76, 1.12)	1.03 (0.87, 1.23)	1.04 (0.83, 1.30)	**Thiazolidinediones**	—
0.48 (0.03, 7.88)	1.42 (0.14, 14.22)	0.48 (0.02, 11.31)	1.72 (0.15, 19.58)	1.47 (0.17, 13.01)	1.33 (0.15, 11.74)	0.41 (0.03, 6.64)	1.25 (0.14, 11.19)	1.32 (0.15, 11.83)	1.30 (0.15, 11.55)	1.45 (0.16, 12.50)	1.46 (0.16, 13.00)	1.40 (0.16, 12.51)	**Tirzepatide**

4.3.3 Network estimates for hospitalization for heart failure (lower left) and end-stage kidney disease (upper right)

**Alpha-glucosidase inhibitors**	—	—	—	—	—	—	—	—	—	—	—
3.47 (0.13, 90.34)	**Basal insulin**	2.13 (0.08, 50.00)	0.87 (0.45, 1.69)	0.69 (0.36, 1.33)	1.35 (0.26, 7.14)	0.69 (0.36, 1.35)	**0.51 (0.26, 0.98)**	0.83 (0.43, 1.61)	0.57 (0.23, 1.39)	0.58 (0.23, 1.43)	0.56 (0.08, 3.85)
5.05 (0.10, 262.01)	1.46 (0.15, 14.61)	**Bolus insulin**	0.41 (0.02, 10.00)	0.33 (0.01, 8.33)	0.63 (0.02, 20.00)	0.33 (0.01, 8.33)	0.24 (0.01, 5.88)	0.39 (0.02, 10.00)	0.27 (0.01, 7.14)	0.27 (0.01, 7.14)	0.27 (0.01, 11.11)
3.09 (0.12, 78.44)	0.89 (0.59, 1.36)	0.61 (0.06, 5.92)	**DPP-4 inhibitors**	**0.80 (0.69, 0.93)**	1.56 (0.34, 7.14)	**0.81 (0.69, 0.93)**	**0.58 (0.50, 0.68)**	0.96 (0.86, 1.08)	0.66 (0.36, 1.19)	0.67 (0.36, 1.23)	0.65 (0.09, 4.76)
3.59 (0.14, 91.17)	1.04 (0.68, 1.58)	0.71 (0.07, 6.85)	**1.16 (1.02, 1.33)**	**GLP-1 receptor agonists**	1.92 (0.43, 9.09)	1.00 (0.87, 1.15)	**0.73 (0.63, 0.84)**	**1.20 (1.09, 1.33)**	0.82 (0.45, 1.49)	0.83 (0.44, 1.56)	0.81 (0.11, 5.88)
2.25 (0.06, 84.00)	0.65 (0.12, 3.48)	0.45 (0.03, 7.25)	0.73 (0.14, 3.72)	0.63 (0.12, 3.19)	**Metformin**	0.52 (0.11, 2.33)	0.38 (0.08, 1.69)	0.62 (0.14, 2.78)	0.42 (0.08, 2.13)	0.43 (0.08, 2.17)	0.42 (0.04, 5.00)
4.18 (0.16, 106.46)	1.21 (0.77, 1.90)	0.83 (0.09, 8.04)	**1.35 (1.11, 1.65)**	1.16 (0.96, 1.41)	1.86 (0.36, 9.54)	**Non-steroidal MRAs**	**0.73 (0.63, 0.84)**	**1.20 (1.09, 1.33)**	0.82 (0.45, 1.49)	0.83 (0.44, 1.56)	0.81 (0.11, 5.88)
4.93 (0.19, 125.08)	1.42 (0.92, 2.19)	0.98 (0.10, 9.43)	**1.59 (1.39, 1.83)**	**1.37 (1.20, 1.56)**	2.19 (0.43, 11.16)	1.18 (0.97, 1.43)	**SGLT2 inhibitors**	**1.64 (1.49, 1.82)**	1.12 (0.61, 2.08)	1.14 (0.61, 2.13)	1.11 (0.16, 7.69)
3.25 (0.13, 82.49)	0.94 (0.62, 1.43)	0.64 (0.07, 6.22)	1.05 (0.95, 1.16)	**0.91 (0.83, 0.99)**	1.45 (0.28, 7.36)	**0.78 (0.66, 0.92)**	**0.66 (0.60, 0.73)**	**Standard treatments**	0.68 (0.37, 1.24)	0.69 (0.37, 1.28)	0.68 (0.09, 4.84)
3.30 (0.13, 84.25)	0.95 (0.62, 1.47)	0.65 (0.07, 6.37)	1.07 (0.86, 1.32)	0.92 (0.72, 1.16)	1.47 (0.28, 7.57)	0.79 (0.60, 1.04)	**0.67 (0.53, 0.85)**	1.01 (0.81, 1.27)	**Sulfonylureas**	1.01 (0.85, 1.22)	0.99 (0.13, 7.69)
2.11 (0.08, 53.77)	**0.61 (0.38, 0.96)**	0.42 (0.04, 4.06)	**0.68 (0.55, 0.85)**	**0.59 (0.47, 0.73)**	0.94 (0.18, 4.81)	**0.50 (0.39, 0.65)**	**0.43 (0.34, 0.53)**	**0.65 (0.53, 0.79)**	**0.64 (0.48, 0.85)**	**Thiazolidinediones**	0.98 (0.12, 7.69)
5.18 (0.16, 171.40)	1.50 (0.42, 5.32)	1.03 (0.07, 14.25)	1.68 (0.44, 6.38)	1.44 (0.38, 5.50)	2.30 (0.28, 18.91)	1.24 (0.32, 4.77)	1.05 (0.28, 4.02)	1.59 (0.42, 6.25)	1.57 (0.41, 6.01)	2.46 (0.64, 9.48)	**Tirzepatide**

4.3.4 Network estimates for health-related quality of life

**Alpha-glucosidase inhibitors**											
0.03 (-0.39, 0.45)	**Basal insulin**										
0.14 (-0.25, 0.52)	0.11 (-0.13, 0.34)	**Bolus insulin**									
0.00 (-0.33, 0.33)	-0.03 (-0.29, 0.23)	-0.14 (-0.34, 0.07)	**DPP-4 inhibitors**								
-0.14 (-0.50, 0.21)	-0.18 (-0.40, 0.05)	**-0.28 (-0.44, -0.13)**	**-0.14 (-0.28, -0.01)**	**GLP-1 receptor agonists**							
-0.14 (-0.70, 0.42)	-0.17 (-0.68, 0.33)	-0.28 (-0.76, 0.20)	-0.14 (-0.59, 0.31)	0.00 (-0.45, 0.45)	**Meglitinides**						
-0.02 (-0.45, 0.41)	-0.05 (-0.41, 0.31)	-0.15 (-0.47, 0.16)	-0.02 (-0.29, 0.26)	0.13 (-0.15, 0.40)	0.12 (-0.35, 0.59)	**Metformin**					
-0.27 (-0.66, 0.12)	**-0.30 (-0.60, -0.01)**	**-0.41 (-0.65, -0.16)**	**-0.27 (-0.48, -0.06)**	-0.13 (-0.32, 0.06)	-0.13 (-0.62, 0.35)	-0.26 (-0.58, 0.07)	**SGLT2 inhibitors**				
0.03 (-0.34, 0.39)	-0.00 (-0.25, 0.24)	-0.11 (-0.29, 0.07)	0.03 (-0.12, 0.17)	**0.17 (0.07, 0.27)**	0.17 (-0.29, 0.63)	0.04 (-0.25, 0.33)	**0.30 (0.10, 0.49)**	**Standard treatments**			
-0.20 (-0.75, 0.35)	-0.23 (-0.72, 0.25)	-0.34 (-0.79, 0.12)	-0.20 (-0.64, 0.24)	-0.06 (-0.49, 0.37)	-0.06 (-0.68, 0.56)	-0.19 (-0.69, 0.32)	0.07 (-0.39, 0.53)	-0.23 (-0.64, 0.19)	**Sulfonylureas**		
-0.17 (-0.63, 0.28)	-0.20 (-0.59, 0.18)	-0.31 (-0.66, 0.04)	-0.17 (-0.48, 0.14)	-0.03 (-0.34, 0.28)	-0.03 (-0.36, 0.30)	-0.15 (-0.49, 0.18)	0.10 (-0.26, 0.46)	-0.20 (-0.52, 0.13)	0.03 (-0.50, 0.56)	**Thiazolidinediones**	
-0.36 (-0.79, 0.07)	**-0.39 (-0.64, -0.15)**	**-0.50 (-0.77, -0.23)**	**-0.36 (-0.64, -0.09)**	-0.22 (-0.46, 0.02)	-0.22 (-0.73, 0.29)	-0.34 (-0.71, 0.02)	-0.09 (-0.40, 0.22)	**-0.39 (-0.65, -0.13)**	-0.16 (-0.65, 0.33)	-0.19 (-0.58, 0.20)	**Tirzepatide**

4.3.5 Network estimates for bodyweight change

**Albiglutide**							
-0.33 (-1.09, 0.42)	**Alpha-glucosidase inhibitors**						
**-2.87 (-3.59, -2.14)**	**-2.53 (-3.11, -1.95)**	**Basal insulin**					
**-3.97 (-5.27, -2.67)**	**-3.64 (-4.86, -2.42)**	-1.11 (-2.23, 0.01)	**Basal-Bolus insulin**				
**-1.73 (-2.59, -0.87)**	**-1.39 (-2.27, -0.52)**	**1.14 (0.29, 1.99)**	**2.25 (0.87, 3.62)**	**Bolus insulin**			
**-1.00 (-1.65, -0.36)**	**-0.67 (-1.10, -0.24)**	**1.87 (1.44, 2.29)**	**2.97 (1.82, 4.13)**	0.73 (-0.06, 1.51)	**DPP-4 inhibitors**		
0.69 (-0.12, 1.50)	**1.02 (0.36, 1.69)**	**3.55 (2.97, 4.13)**	**4.66 (3.43, 5.89)**	**2.41 (1.49, 3.34)**	**1.69 (1.15, 2.23)**	**Dulaglutide**	
1.87 (-0.04, 3.79)	**2.21 (0.35, 4.06)**	**4.74 (2.89, 6.59)**	**5.85 (3.71, 7.99)**	**3.60 (1.64, 5.57)**	**2.87 (1.06, 4.69)**	1.19 (-0.69, 3.07)	**Efpeglenatide**

0.33 (-0.55, 1.21)	0.66 (-0.08, 1.41)	**3.20 (2.53, 3.86)**	**4.30 (3.03, 5.58)**	**2.06 (1.07, 3.04)**	**1.33 (0.70, 1.96)**	-0.36 (-1.14, 0.42)	-1.54 (-3.46, 0.37)	**Exenatide extended-release**							
**1.05 (0.12, 1.99)**	**1.39 (0.58, 2.20)**	**3.92 (3.16, 4.68)**	**5.03 (3.70, 6.35)**	**2.78 (1.74, 3.82)**	**2.05 (1.34, 2.77)**	0.37 (-0.49, 1.22)	-0.82 (-2.76, 1.11)	0.72 (-0.20, 1.64)	**Exenatide immediate-release**						
**1.50 (0.78, 2.21)**	**1.83 (1.28, 2.38)**	**4.36 (3.88, 4.85)**	**5.47 (4.28, 6.66)**	**3.23 (2.42, 4.04)**	**2.50 (2.11, 2.88)**	**0.81 (0.19, 1.43)**	-0.38 (-2.22, 1.47)	**1.17 (0.46, 1.87)**	0.44 (-0.33, 1.22)	**Liraglutide**					
0.12 (-0.72, 0.95)	0.45 (-0.26, 1.16)	**2.98 (2.28, 3.68)**	**4.09 (2.81, 5.37)**	**1.84 (0.94, 2.75)**	**1.12 (0.53, 1.71)**	-0.57 (-1.34, 0.20)	-1.76 (-3.65, 0.13)	-0.21 (-1.06, 0.63)	**-0.94 (-1.84, -0.04)**	**-1.38 (-2.05, -0.71)**	**Lixisenatide**				
-0.88 (-2.86, 1.11)	-0.54 (-2.47, 1.39)	**1.99 (0.06, 3.92)**	**3.10 (0.89, 5.30)**	0.85 (-1.18, 2.89)	0.12 (-1.77, 2.01)	-1.56 (-3.52, 0.39)	**-2.75 (-5.36, -0.14)**	-1.21 (-3.19, 0.78)	-1.93 (-3.93, 0.08)	**-2.37 (-4.29, -0.46)**	-0.99 (-2.96, 0.97)	**Loxenatide**			
**-1.97 (-2.90, -1.05)**	**-1.64 (-2.43, -0.85)**	**0.89 (0.11, 1.68)**	**2.00 (0.67, 3.33)**	-0.25 (-1.27, 0.78)	**-0.97 (-1.66, -0.29)**	**-2.66 (-3.51, -1.81)**	**-3.85 (-5.78, -1.92)**	**-2.30 (-3.22, -1.39)**	**-3.03 (-4.00, -2.06)**	**-3.47 (-4.24, -2.71)**	**-2.09 (-2.98, -1.21)**	-1.10 (-3.10, 0.90)	**Meglitinides**		
0.12 (-0.59, 0.82)	0.45 (-0.05, 0.95)	**2.98 (2.48, 3.49)**	**4.09 (2.90, 5.28)**	**1.85 (1.01, 2.68)**	**1.12 (0.79, 1.45)**	-0.57 (-1.16, 0.02)	-1.76 (-3.59, 0.08)	-0.21 (-0.90, 0.47)	**-0.94 (-1.68, -0.19)**	**-1.38 (-1.86, -0.90)**	0.00 (-0.65, 0.66)	0.99 (-0.92, 2.90)	**2.09 (1.37, 2.82)**	**Metformin**	
**2.26 (1.33, 3.19)**	**2.60 (1.79, 3.40)**	**5.13 (4.34, 5.92)**	**6.24 (4.90, 7.57)**	**3.99 (2.96, 5.01)**	**3.26 (2.56, 3.96)**	**1.58 (0.72, 2.43)**	0.39 (-1.54, 2.32)	**1.93 (1.01, 2.86)**	**1.21 (0.23, 2.18)**	**0.76 (0.00, 1.52)**	**2.15 (1.26, 3.04)**	**3.14 (1.14, 5.14)**	**4.24 (3.27, 5.20)**	**2.14 (1.39, 2.90)**	**Semaglutide (Oral)**

**3.91 (3.06, 4.76)**	**4.24 (3.52, 4.97)**	**6.77 (6.09, 7.46)**	**7.88 (6.60, 9.16)**	**5.64 (4.73, 6.54)**	**4.91 (4.30, 5.51)**	**3.22 (2.44, 4.00)**	**2.03 (0.13, 3.93)**	**3.58 (2.73, 4.43)**	**2.85 (1.95, 3.76)**	**2.41 (1.73, 3.09)**	**3.79 (2.98, 4.61)**	**4.78 (2.81, 6.76)**	**5.88 (4.98, 6.78)**	**3.79 (3.12, 4.46)**	**1.65 (0.76, 2.53)**	**Semaglutide (Subcutaneous)**					
**1.26 (0.60, 1.92)**	**1.60 (1.14, 2.06)**	**4.13 (3.68, 4.58)**	**5.24 (4.07, 6.40)**	**2.99 (2.21, 3.78)**	**2.26 (2.02, 2.50)**	**0.58 (0.02, 1.13)**	-0.61 (-2.43, 1.21)	**0.93 (0.29, 1.58)**	0.21 (-0.51, 0.93)	-0.23 (-0.64, 0.18)	**1.15 (0.55, 1.75)**	**2.14 (0.25, 4.03)**	**3.24 (2.54, 3.94)**	**1.15 (0.79, 1.50)**	**-1.00 (-1.70, -0.29)**	**-2.64 (-3.26, -2.03)**	**SGLT2 inhibitors**				
**-0.72 (-1.35, -0.08)**	-0.38 (-0.80, 0.04)	**2.15 (1.74, 2.56)**	**3.26 (2.10, 4.41)**	**1.01 (0.24, 1.79)**	**0.28 (0.11, 0.46)**	**-1.40 (-1.93, -0.88)**	**-2.59 (-4.40, -0.78)**	**-1.05 (-1.67, -0.42)**	**-1.77 (-2.47, -1.07)**	**-2.21 (-2.58, -1.85)**	**-0.83 (-1.40, -0.26)**	0.16 (-1.72, 2.04)	**1.26 (0.58, 1.94)**	**-0.83 (-1.16, -0.51)**	**-2.98 (-3.66, -2.29)**	**-4.62 (-5.22, -4.03)**	**-1.98 (-2.18, -1.78)**	**Standard treatments**			
**-2.50 (-3.18, -1.82)**	**-2.16 (-2.65, -1.68)**	0.37 (-0.10, 0.84)	**1.48 (0.30, 2.65)**	-0.77 (-1.58, 0.04)	**-1.50 (-1.78, -1.22)**	**-3.18 (-3.76, -2.61)**	**-4.37 (-6.20, -2.54)**	**-2.83 (-3.50, -2.16)**	**-3.55 (-4.29, -2.80)**	**-3.99 (-4.43, -3.56)**	**-2.61 (-3.24, -1.98)**	-1.62 (-3.52, 0.28)	-0.52 (-1.20, 0.16)	**-2.61 (-2.99, -2.24)**	**-4.76 (-5.49, -4.02)**	**-6.40 (-7.05, -5.76)**	**-3.76 (-4.07, -3.45)**	**-1.78 (-2.06, -1.50)**	**Sulfonylureas**		
**-3.53 (-4.20, -2.86)**	**-3.19 (-3.66, -2.72)**	**-0.66 (-1.12, -0.20)**	0.45 (-0.71, 1.60)	**-1.80 (-2.60, -0.99)**	**-2.52 (-2.80, -2.25)**	**-4.21 (-4.78, -3.64)**	**-5.40 (-7.22, -3.58)**	**-3.86 (-4.51, -3.20)**	**-4.58 (-5.31, -3.84)**	**-5.02 (-5.45, -4.59)**	**-3.64 (-4.26, -3.02)**	**-2.65 (-4.55, -0.75)**	**-1.55 (-2.25, -0.85)**	**-3.64 (-4.00, -3.28)**	**-5.79 (-6.52, -5.06)**	**-7.43 (-8.07, -6.79)**	**-4.79 (-5.10, -4.48)**	**-2.81 (-3.07, -2.55)**	**-1.03 (-1.34, -0.72)**	**Thiazolidinediones**	
**7.85 (6.83, 8.88)**	**8.19 (7.26, 9.11)**	**10.72 (9.88, 11.57)**	**11.83 (10.45, 13.21)**	**9.58 (8.47, 10.69)**	**8.85 (8.02, 9.69)**	**7.17 (6.23, 8.10)**	**5.98 (4.00, 7.97)**	**7.52 (6.51, 8.54)**	**6.80 (5.74, 7.87)**	**6.36 (5.47, 7.24)**	**7.74 (6.74, 8.74)**	**8.73 (6.68, 10.78)**	**9.83 (8.76, 10.89)**	**7.74 (6.86, 8.62)**	**5.59 (4.53, 6.66)**	**3.95 (3.02, 4.87)**	**6.59 (5.75, 7.44)**	**8.57 (7.75, 9.40)**	**10.35 (9.49, 11.21)**	**11.38 (10.52, 12.24)**	**Tirzepatide**

4.3.6 Network estimates for severe hypoglycaemia

**Alpha-glucosidase inhibitors**									
0.54 (0.13, 2.33)	**Basal insulin**								
0.26 (0.03, 2.14)	0.48 (0.11, 2.22)	**Basal-Bolus insulin**							
0.53 (0.11, 2.52)	0.97 (0.49, 1.91)	2.01 (0.38, 10.53)	**Bolus insulin**						
1.17 (0.28, 4.90)	**2.14 (1.62, 2.85)**	4.44 (0.95, 20.70)	**2.21 (1.17, 4.20)**	**DPP-4 inhibitors**					
1.33 (0.32, 5.58)	**2.44 (1.88, 3.17)**	**5.06 (1.09, 23.49)**	**2.52 (1.35, 4.72)**	**1.14 (1.00, 1.30)**	**GLP-1 receptor agonists**				
0.40 (0.07, 2.52)	0.74 (0.22, 2.55)	1.54 (0.22, 10.84)	0.77 (0.20, 2.99)	0.35 (0.10, 1.16)	0.30 (0.09, 1.02)	**Meglitinides**			
0.75 (0.16, 3.58)	1.37 (0.67, 2.80)	2.85 (0.54, 15.16)	1.42 (0.57, 3.54)	0.64 (0.33, 1.25)	0.56 (0.29, 1.10)	1.85 (0.61, 5.63)	**Metformin**		
2.03 (0.46, 8.98)	**3.72 (2.28, 6.08)**	**7.71 (1.57, 37.81)**	**3.84 (1.81, 8.16)**	**1.74 (1.14, 2.65)**	**1.52 (1.00, 2.31)**	**5.01 (1.40, 17.96)**	**2.71 (1.24, 5.90)**	**Non-steroidal MRAs**	
1.45 (0.34, 6.07)	**2.66 (1.97, 3.58)**	**5.50 (1.18, 25.70)**	**2.74 (1.44, 5.22)**	**1.24 (1.05, 1.46)**	1.09 (0.93, 1.27)	**3.58 (1.06, 12.02)**	1.93 (0.99, 3.78)	0.71 (0.46, 1.10)	**SGLT2 inhibitors**

1.30 (0.31, 5.43)	**2.38 (1.82, 3.12)**	**4.94 (1.06, 22.96)**	**2.46 (1.31, 4.63)**	**1.11 (1.00, 1.23)**	0.98 (0.90, 1.06)	3.21 (0.96, 10.75)	1.73 (0.89, 3.37)	**0.64 (0.43, 0.96)**	0.90 (0.79, 1.02)	**Standard treatments**				
0.25 (0.06, 1.06)	**0.46 (0.32, 0.66)**	0.95 (0.20, 4.50)	**0.47 (0.24, 0.94)**	**0.21 (0.16, 0.29)**	**0.19 (0.14, 0.25)**	0.62 (0.18, 2.08)	**0.33 (0.17, 0.67)**	**0.12 (0.07, 0.20)**	**0.17 (0.13, 0.24)**	**0.19 (0.14, 0.26)**	**Sulfonylureas**			
0.91 (0.21, 4.01)	**1.67 (1.07, 2.63)**	3.47 (0.72, 16.84)	1.73 (0.82, 3.62)	0.78 (0.52, 1.16)	0.69 (0.46, 1.02)	2.26 (0.64, 7.99)	1.22 (0.57, 2.61)	**0.45 (0.26, 0.79)**	**0.63 (0.42, 0.95)**	0.70 (0.48, 1.03)	**3.66 (2.31, 5.80)**	**Thiazolidinediones**		
1.15 (0.20, 6.56)	2.12 (0.81, 5.56)	4.39 (0.72, 26.62)	2.19 (0.68, 7.03)	0.99 (0.37, 2.66)	0.87 (0.32, 2.32)	2.85 (0.60, 13.55)	1.54 (0.47, 5.06)	0.57 (0.20, 1.66)	0.80 (0.30, 2.16)	0.88 (0.33, 2.38)	**4.64 (1.67, 12.86)**	1.27 (0.44, 3.63)	**Tirzepatide**	

4.3.7 Network estimates for severe gastrointestinal events

**Alpha-glucosidase inhibitors**										
8.74 (0.58, 131.74)	**Basal insulin**									
1.03 (0.05, 20.56)	**0.12 (0.01, 0.98)**	**Bolus insulin**								
2.96 (0.24, 36.24)	0.34 (0.09, 1.34)	2.88 (0.43, 19.34)	**DPP-4 inhibitors**							
1.72 (0.15, 19.74)	**0.20 (0.06, 0.67)**	1.67 (0.29, 9.47)	0.58 (0.26, 1.28)	**GLP-1 receptor agonists**						
1.53 (0.19, 12.17)	0.18 (0.03, 1.01)	1.48 (0.17, 12.84)	0.52 (0.13, 2.10)	0.89 (0.25, 3.21)	**Metformin**					
3.40 (0.30, 38.15)	0.39 (0.11, 1.36)	3.30 (0.56, 19.35)	1.15 (0.52, 2.55)	**1.97 (1.39, 2.80)**	2.22 (0.64, 7.71)	**Standard treatments**				
3.43 (0.28, 42.63)	0.39 (0.07, 2.30)	3.33 (0.37, 29.63)	1.16 (0.31, 4.32)	1.99 (0.53, 7.54)	2.24 (0.53, 9.39)	1.01 (0.27, 3.70)	**Sulfonylureas**			
2.38 (0.22, 25.43)	0.27 (0.06, 1.16)	2.31 (0.34, 15.78)	0.80 (0.28, 2.29)	1.38 (0.61, 3.15)	1.56 (0.50, 4.88)	0.70 (0.33, 1.49)	0.70 (0.19, 2.53)	**Thiazolidinediones**		
0.74 (0.06, 9.69)	**0.08 (0.02, 0.30)**	0.72 (0.10, 4.97)	**0.25 (0.08, 0.78)**	0.43 (0.18, 1.01)	0.48 (0.11, 2.21)	**0.22 (0.09, 0.53)**	0.22 (0.05, 1.03)	**0.31 (0.10, 0.99)**	**Tirzepatide**	

4.3.8 Network estimates for Genital infection

**DPP-4 inhibitors**						
1.03 (0.47, 2.23)	**GLP-1 receptor agonists**					
0.55 (0.26, 1.16)	0.53 (0.22, 1.31)	**Metformin**				
**0.22 (0.14, 0.33)**	**0.21 (0.11, 0.40)**	**0.39 (0.21, 0.73)**	**SGLT2 inhibitors**			
0.71 (0.46, 1.10)	0.69 (0.36, 1.34)	1.30 (0.69, 2.45)	**3.30 (2.88, 3.78)**	**Standard treatments**		
1.36 (0.80, 2.33)	1.33 (0.64, 2.76)	**2.49 (1.24, 5.04)**	**6.32 (4.53, 8.82)**	**1.92 (1.33, 2.78)**	**Sulfonylureas**	
0.07 (0.00, 1.72)	0.06 (0.00, 1.73)	0.12 (0.00, 3.24)	0.30 (0.01, 7.75)	0.09 (0.00, 2.38)	0.05 (0.00, 1.25)	**Thiazolidinediones**

4.3.9 Network estimates for Amputation

**DPP-4 inhibitors**					
1.28 (0.12, 13.54)	**GLP-1 receptor agonists**				
0.97 (0.37, 2.55)	0.76 (0.07, 8.48)	**Non-steroidal MRAs**			
0.71 (0.39, 1.31)	0.55 (0.06, 5.52)	0.73 (0.32, 1.65)	**SGLT2 inhibitors**		
0.91 (0.51, 1.60)	0.71 (0.07, 6.95)	0.93 (0.43, 2.03)	**1.27 (1.01, 1.61)**	**Standard treatments**	
1.24 (0.54, 2.83)	0.96 (0.09, 10.26)	1.27 (0.48, 3.40)	1.74 (0.91, 3.31)	1.37 (0.75, 2.50)	**Thiazolidinediones**

4.3.10 Network estimates for Ketoacidosis due to diabetes

**Alpha-glucosidase inhibitors**								
0.49 (0.01, 19.75)	**DPP-4 inhibitors**							
0.38 (0.01, 16.39)	0.78 (0.32, 1.89)	**GLP-1 receptor agonists**						
1.01 (0.02, 51.04)	2.08 (0.05, 84.81)	2.69 (0.06, 116.94)	**Meglitinides**					
0.29 (0.00, 39.24)	0.59 (0.02, 15.88)	0.76 (0.03, 20.25)	0.28 (0.00, 38.81)	**Metformin**				
0.58 (0.01, 26.32)	1.19 (0.41, 3.46)	1.54 (0.55, 4.26)	0.57 (0.01, 26.04)	2.02 (0.07, 56.23)	**Non-steroidal MRAs**			
0.19 (0.00, 7.97)	**0.39 (0.19, 0.83)**	0.51 (0.25, 1.02)	0.19 (0.00, 7.89)	0.67 (0.03, 16.41)	**0.33 (0.13, 0.81)**	**SGLT2 inhibitors**		
0.40 (0.01, 16.47)	0.82 (0.41, 1.61)	1.05 (0.58, 1.92)	0.39 (0.01, 16.29)	1.38 (0.05, 34.70)	0.68 (0.30, 1.56)	**2.07 (1.44, 2.98)**	**Standard treatments**	
0.67 (0.03, 16.50)	1.38 (0.21, 8.94)	1.78 (0.24, 13.13)	0.66 (0.03, 16.32)	2.34 (0.06, 97.87)	1.16 (0.15, 9.29)	3.51 (0.52, 23.87)	1.69 (0.25, 11.11)	**Sulfonylureas**

4.4 Heterogeneity (Inconsistency) assessments

4.4.1 Heterogeneity (Inconsistency) assessed by pairwise meta-analyses

Notes: The study assess the between-study heterogeneity for all comparisons in each outcome by several pairwise random-effect meta-analyses. We first evaluated the potential heterogeneity if the point estimates vary greatly, or the confidence intervals are not overlapped, or the prediction intervals are contrary to the confidence intervals, or the P-value for Q-statistic is less than 0.1, or the I-squared is more than 50%. We then judged whether there is a clinical heterogeneity supervised by the clinical experts.

Abbreviations: k, no. of studies; Q, Q statistic; I, I statistic; tau, standard deviation of the random-effect distribution.

Outcome	Comparisons	Odds ratio	Mean difference	Prediction interval	k	Q	P-value	tau^2	I^2
**All-cause death**	Alpha-glucosidase inhibitors *vs* Meglitinides	0.33 (0.01 to 8.1)	NA	NA	1	0	NA	NA	NA
**All-cause death**	Alpha-glucosidase inhibitors *vs* Standard treatments	1.31 (0.33 to 5.18)	NA	(0.14 to 12.22)	5	1.782	0.776	0	0
**All-cause death**	Alpha-glucosidase inhibitors *vs* Sulfonylureas	0.64 (0.13 to 3.21)	NA	NA	2	0.389	0.533	0	0
**All-cause death**	Basal insulin *vs* Basal-Bolus insulin	7.03 (0.36 to 136.82)	NA	NA	1	0	NA	NA	NA
**All-cause death**	Basal insulin *vs* Bolus insulin	0.34 (0.01 to 8.36)	NA	NA	1	0	NA	NA	NA
**All-cause death**	Basal insulin *vs* DPP-4 inhibitors	0.99 (0.65 to 1.52)	NA	NA	4	2.123	0.547	0	0
**All-cause death**	Basal insulin *vs* GLP-1 receptor agonists	1.44 (0.98 to 2.12)	NA	(0.91 to 2.3)	9	4.25	0.834	0	0
**All-cause death**	Basal insulin *vs* Standard treatments	0.32 (0.01 to 7.99)	NA	NA	1	0	NA	NA	NA
**All-cause death**	Basal insulin *vs* Sulfonylureas	0.97 (0.63 to 1.49)	NA	NA	1	0	NA	NA	NA
**All-cause death**	Basal insulin *vs* Tirzepatide	1.36 (0.82 to 2.26)	NA	NA	2	0.303	0.582	0	0
**All-cause death**	Basal-Bolus insulin *vs* GLP-1 receptor agonists	0.75 (0.11 to 5.16)	NA	NA	2	0.283	0.595	0	0
**All-cause death**	Basal-Bolus insulin *vs* Sulfonylureas	6.63 (0.3 to 145.31)	NA	NA	1	0	NA	NA	NA
**All-cause death**	Bolus insulin *vs* GLP-1 receptor agonists	0.45 (0.1 to 2.07)	NA	NA	4	3.816	0.282	0.665	0.214
**All-cause death**	DPP-4 inhibitors *vs* GLP-1 receptor agonists	1.37 (0.93 to 2.02)	NA	(0.88 to 2.12)	13	5.153	0.953	0	0
**All-cause death**	DPP-4 inhibitors *vs* Metformin	1.1 (0.32 to 3.81)	NA	(0.15 to 8.28)	5	2.01	0.734	0	0
**All-cause death**	DPP-4 inhibitors *vs* SGLT2 inhibitors	0.5 (0.15 to 1.63)	NA	(0.11 to 2.35)	7	2.417	0.878	0	0
**All-cause death**	DPP-4 inhibitors *vs* Standard treatments	1.02 (0.95 to 1.1)	NA	(0.95 to 1.1)	49	28.473	0.989	0	0

Outcome	Comparisons	Odds ratio	Mean difference	Prediction interval	k	Q	P-value	tau^2	I^2
**All-cause death**	DPP-4 inhibitors *vs* Sulfonylureas	0.88 (0.76 to 1.01)	NA	(0.75 to 1.02)	17	8.251	0.941	0	0
**All-cause death**	DPP-4 inhibitors *vs* Thiazolidinediones	2.13 (0.43 to 10.59)	NA	NA	4	0.287	0.963	0	0
**All-cause death**	GLP-1 receptor agonists *vs* Metformin	0.33 (0.01 to 8.12)	NA	NA	1	0	NA	NA	NA
**All-cause death**	GLP-1 receptor agonists *vs* SGLT2 inhibitors	1.3 (0.25 to 6.78)	NA	NA	3	1.126	0.57	0	0
**All-cause death**	GLP-1 receptor agonists *vs* Standard treatments	0.88 (0.83 to 0.94)	NA	(0.83 to 0.94)	41	23.516	0.982	0	0
**All-cause death**	GLP-1 receptor agonists *vs* Sulfonylureas	0.68 (0.44 to 1.04)	NA	(0.37 to 1.24)	6	1.966	0.854	0	0
**All-cause death**	GLP-1 receptor agonists *vs* Thiazolidinediones	0.37 (0.05 to 2.78)	NA	NA	3	0.73	0.694	0	0
**All-cause death**	GLP-1 receptor agonists *vs* Tirzepatide	0.4 (0.07 to 2.24)	NA	NA	2	0.716	0.398	0	0
**All-cause death**	Meglitinides *vs* Metformin	0.33 (0.01 to 8.15)	NA	NA	1	0	NA	NA	NA
**All-cause death**	Meglitinides *vs* Standard treatments	1.72 (0.15 to 19.01)	NA	NA	2	0.134	0.714	0	0
**All-cause death**	Meglitinides *vs* Sulfonylureas	1.81 (0.39 to 8.46)	NA	NA	3	0.774	0.679	0	0
**All-cause death**	Meglitinides *vs* Thiazolidinediones	1.51 (0.06 to 37.15)	NA	NA	1	0	NA	NA	NA
**All-cause death**	Metformin *vs* SGLT2 inhibitors	1.86 (0.29 to 11.83)	NA	NA	3	1.722	0.423	0	0
**All-cause death**	Metformin *vs* Standard treatments	0.93 (0.52 to 1.65)	NA	(0.23 to 3.8)	5	3.986	0.408	0.11	0
**All-cause death**	Metformin *vs* Sulfonylureas	0.88 (0.49 to 1.6)	NA	(0.23 to 3.38)	5	4.106	0.392	0.085	0.026
**All-cause death**	Metformin *vs* Thiazolidinediones	0.94 (0.6 to 1.46)	NA	(0.54 to 1.64)	8	2.612	0.918	0	0
**All-cause death**	Non-steroidal MRAs *vs* Standard treatments	0.89 (0.79 to 1)	NA	NA	3	0.574	0.751	0	0
**All-cause death**	SGLT2 inhibitors *vs* Standard treatments	0.88 (0.8 to 0.96)	NA	(0.72 to 1.06)	39	28.28	0.875	0.007	0
**All-cause death**	SGLT2 inhibitors *vs* Sulfonylureas	0.78 (0.33 to 1.86)	NA	(0.19 to 3.2)	5	3.736	0.443	0	0
**All-cause death**	Sulfonylureas *vs* Standard treatments	1.78 (0.29 to 11)	NA	NA	2	0.718	0.397	0	0
**All-cause death**	Sulfonylureas *vs* Thiazolidinediones	0.97 (0.74 to 1.28)	NA	(0.71 to 1.33)	11	6.398	0.781	0	0
**All-cause death**	Thiazolidinediones *vs* Standard treatments	0.91 (0.78 to 1.06)	NA	(0.77 to 1.07)	19	9.64	0.943	0	0
**All-cause death**	Tirzepatide *vs* Standard treatments	0.28 (0.03 to 2.68)	NA	NA	2	0.704	0.401	0	0
**Cardiovascular death**	Alpha-glucosidase inhibitors *vs* Standard treatments	1.54 (0.24 to 9.79)	NA	NA	3	0.372	0.83	0	0
**Cardiovascular death**	Alpha-glucosidase inhibitors *vs* Sulfonylureas	0.51 (0.05 to 4.97)	NA	NA	1	0	NA	NA	NA
**Cardiovascular death**	Basal insulin *vs* Bolus insulin	0.34 (0.01 to 8.36)	NA	NA	1	0	NA	NA	NA

Outcome	Comparisons	Odds ratio	Mean difference	Prediction interval	k	Q	P-value	tau^2	I^2
**Cardiovascular death**	Basal insulin *vs* DPP-4 inhibitors	1.04 (0.57 to 1.9)	NA	NA	2	0.434	0.51	0	0
**Cardiovascular death**	Basal insulin *vs* GLP-1 receptor agonists	1.86 (1.04 to 3.31)	NA	(0.82 to 4.21)	6	4.226	0.517	0	0
**Cardiovascular death**	Basal insulin *vs* Sulfonylureas	1.31 (0.68 to 2.52)	NA	NA	1	0	NA	NA	NA
**Cardiovascular death**	Basal insulin *vs* Tirzepatide	1.5 (0.53 to 4.22)	NA	NA	1	0	NA	NA	NA
**Cardiovascular death**	Basal-Bolus insulin *vs* GLP-1 receptor agonists	1.51 (0.06 to 37.31)	NA	NA	1	0	NA	NA	NA
**Cardiovascular death**	Basal-Bolus insulin *vs* Sulfonylureas	3.8 (0.15 to 97.81)	NA	NA	1	0	NA	NA	NA
**Cardiovascular death**	Bolus insulin *vs* GLP-1 receptor agonists	0.34 (0.01 to 8.27)	NA	NA	1	0	NA	NA	NA
**Cardiovascular death**	DPP-4 inhibitors *vs* GLP-1 receptor agonists	1.94 (1.03 to 3.65)	NA	(0.88 to 4.28)	8	3.455	0.84	0	0
**Cardiovascular death**	DPP-4 inhibitors *vs* Metformin	2.98 (0.12 to 73.73)	NA	NA	1	0	NA	NA	NA
**Cardiovascular death**	DPP-4 inhibitors *vs* SGLT2 inhibitors	0.29 (0.05 to 1.72)	NA	NA	3	0.376	0.829	0	0
**Cardiovascular death**	DPP-4 inhibitors *vs* Standard treatments	0.99 (0.91 to 1.08)	NA	(0.91 to 1.09)	29	16.574	0.957	0	0
**Cardiovascular death**	DPP-4 inhibitors *vs* Sulfonylureas	1 (0.82 to 1.22)	NA	(0.79 to 1.27)	10	4.419	0.882	0	0
**Cardiovascular death**	DPP-4 inhibitors *vs* Thiazolidinediones	5.24 (0.54 to 50.59)	NA	NA	2	0.232	0.63	0	0
**Cardiovascular death**	GLP-1 receptor agonists *vs* Standard treatments	0.88 (0.81 to 0.95)	NA	(0.81 to 0.95)	24	13.484	0.941	0	0
**Cardiovascular death**	GLP-1 receptor agonists *vs* Sulfonylureas	0.56 (0.25 to 1.27)	NA	NA	1	0	NA	NA	NA
**Cardiovascular death**	GLP-1 receptor agonists *vs* Thiazolidinediones	1.03 (0.02 to 52.28)	NA	NA	1	0	NA	NA	NA
**Cardiovascular death**	GLP-1 receptor agonists *vs* Tirzepatide	0.27 (0.02 to 4.93)	NA	NA	1	0	NA	NA	NA
**Cardiovascular death**	Meglitinides *vs* Metformin	0.33 (0.01 to 8.15)	NA	NA	1	0	NA	NA	NA
**Cardiovascular death**	Meglitinides *vs* Standard treatments	0.96 (0.02 to 48.7)	NA	NA	1	0	NA	NA	NA
**Cardiovascular death**	Meglitinides *vs* Sulfonylureas	0.5 (0.03 to 8.08)	NA	NA	1	0	NA	NA	NA
**Cardiovascular death**	Meglitinides *vs* Thiazolidinediones	1.51 (0.06 to 37.15)	NA	NA	1	0	NA	NA	NA
**Cardiovascular death**	Metformin *vs* SGLT2 inhibitors	1.86 (0.29 to 11.83)	NA	NA	3	1.722	0.423	0	0
**Cardiovascular death**	Metformin *vs* Standard treatments	2.97 (0.47 to 18.97)	NA	NA	2	0	0.989	0	0
**Cardiovascular death**	Metformin *vs* Sulfonylureas	0.89 (0.27 to 2.89)	NA	NA	3	1.759	0.415	0.235	0
**Cardiovascular death**	Metformin *vs* Thiazolidinediones	0.7 (0.11 to 4.54)	NA	NA	3	1.215	0.545	0	0
**Cardiovascular death**	Non-steroidal MRAs *vs* Standard treatments	0.88 (0.75 to 1.02)	NA	NA	2	0.114	0.735	0	0

Outcome	Comparisons	Odds ratio	Mean difference	Prediction interval	k	Q	P-value	tau^2	I^2
**Cardiovascular death**	SGLT2 inhibitors *vs* Standard treatments	0.85 (0.75 to 0.96)	NA	(0.64 to 1.13)	19	22.124	0.227	0.014	0.186
**Cardiovascular death**	SGLT2 inhibitors *vs* Sulfonylureas	1.19 (0.15 to 9.68)	NA	NA	2	0.033	0.857	0	0
**Cardiovascular death**	Sulfonylureas *vs* Standard treatments	3.03 (0.12 to 75.42)	NA	NA	1	0	NA	NA	NA
**Cardiovascular death**	Sulfonylureas *vs* Thiazolidinediones	0.85 (0.45 to 1.59)	NA	(0.39 to 1.86)	8	5.666	0.579	0	0
**Cardiovascular death**	Thiazolidinediones *vs* Standard treatments	0.91 (0.75 to 1.11)	NA	(0.74 to 1.14)	14	6.329	0.933	0	0
**Non-fatal myocardial infarction**	Alpha-glucosidase inhibitors *vs* Meglitinides	0.99 (0.02 to 49.94)	NA	NA	1	0	NA	NA	NA
**Non-fatal myocardial infarction**	Alpha-glucosidase inhibitors *vs* Standard treatments	0.3 (0.03 to 2.7)	NA	NA	2	0.575	0.448	0	0
**Non-fatal myocardial infarction**	Alpha-glucosidase inhibitors *vs* Sulfonylureas	0.4 (0.02 to 8.3)	NA	NA	1	0	NA	NA	NA
**Non-fatal myocardial infarction**	Basal insulin *vs* Basal-Bolus insulin	2.92 (0.12 to 72.15)	NA	NA	1	0	NA	NA	NA
**Non-fatal myocardial infarction**	Basal insulin *vs* Bolus insulin	0.76 (0.17 to 3.45)	NA	NA	1	0	NA	NA	NA
**Non-fatal myocardial infarction**	Basal insulin *vs* DPP-4 inhibitors	0.37 (0.01 to 9.12)	NA	NA	1	0	NA	NA	NA
**Non-fatal myocardial infarction**	Basal insulin *vs* GLP-1 receptor agonists	1.19 (0.49 to 2.91)	NA	(0.37 to 3.85)	7	3.913	0.688	0	0
**Non-fatal myocardial infarction**	Basal insulin *vs* Standard treatments	3 (0.12 to 74.19)	NA	NA	1	0	NA	NA	NA
**Non-fatal myocardial infarction**	Basal insulin *vs* Thiazolidinediones	0.33 (0.01 to 8.21)	NA	NA	1	0	NA	NA	NA
**Non-fatal myocardial infarction**	Basal-Bolus insulin *vs* GLP-1 receptor agonists	0.35 (0.01 to 8.68)	NA	NA	1	0	NA	NA	NA
**Non-fatal myocardial infarction**	Bolus insulin *vs* GLP-1 receptor agonists	1.2 (0.29 to 4.9)	NA	NA	4	1.405	0.704	0	0
**Non-fatal myocardial infarction**	DPP-4 inhibitors *vs* GLP-1 receptor agonists	1.1 (0.45 to 2.68)	NA	(0.39 to 3.14)	10	2.835	0.97	0	0
**Non-fatal myocardial infarction**	DPP-4 inhibitors *vs* Metformin	0.48 (0.11 to 2.1)	NA	(0.04 to 5.24)	5	2.775	0.596	0	0
**Non-fatal myocardial infarction**	DPP-4 inhibitors *vs* SGLT2 inhibitors	1.09 (0.38 to 3.11)	NA	(0.3 to 4.03)	8	3.3	0.856	0	0
**Non-fatal myocardial infarction**	DPP-4 inhibitors *vs* Standard treatments	1.02 (0.93 to 1.12)	NA	(0.93 to 1.12)	45	25.17	0.99	0	0
**Non-fatal myocardial infarction**	DPP-4 inhibitors *vs* Sulfonylureas	0.89 (0.64 to 1.24)	NA	(0.54 to 1.49)	12	6.605	0.83	0.024	0
**Non-fatal myocardial infarction**	DPP-4 inhibitors *vs* Thiazolidinediones	1.01 (0.23 to 4.38)	NA	(0.05 to 22.43)	5	4.662	0.324	0.389	0.142
**Non-fatal myocardial infarction**	GLP-1 receptor agonists *vs* Metformin	1.27 (0.11 to 15.18)	NA	NA	2	0.025	0.874	0	0
**Non-fatal myocardial infarction**	GLP-1 receptor agonists *vs* SGLT2 inhibitors	5.11 (0.24 to 107.01)	NA	NA	1	0	NA	NA	NA
**Non-fatal myocardial infarction**	GLP-1 receptor agonists *vs* Standard treatments	0.91 (0.81 to 1.01)	NA	(0.71 to 1.15)	37	28.366	0.814	0.011	0
**Non-fatal myocardial infarction**	GLP-1 receptor agonists *vs* Sulfonylureas	1.05 (0.36 to 3.05)	NA	NA	4	1.168	0.761	0	0

Outcome	Comparisons	Odds ratio	Mean difference	Prediction interval	k	Q	P-value	tau^2	I^2
**Non-fatal myocardial infarction**	GLP-1 receptor agonists *vs* Thiazolidinediones	0.21 (0.02 to 1.91)	NA	NA	2	0.001	0.976	0	0
**Non-fatal myocardial infarction**	GLP-1 receptor agonists *vs* Tirzepatide	0.73 (0.07 to 8.1)	NA	NA	2	0.045	0.832	0	0
**Non-fatal myocardial infarction**	Meglitinides *vs* Standard treatments	1.33 (0.06 to 27.93)	NA	NA	1	0	NA	NA	NA
**Non-fatal myocardial infarction**	Meglitinides *vs* Sulfonylureas	0.14 (0.02 to 1.12)	NA	NA	2	0.954	0.329	0	0
**Non-fatal myocardial infarction**	Metformin *vs* SGLT2 inhibitors	1.43 (0.06 to 35.52)	NA	NA	1	0	NA	NA	NA
**Non-fatal myocardial infarction**	Metformin *vs* Standard treatments	0.88 (0.56 to 1.38)	NA	(0.3 to 2.58)	5	3.834	0.429	0.061	0
**Non-fatal myocardial infarction**	Metformin *vs* Sulfonylureas	1.24 (0.71 to 2.17)	NA	NA	4	0.982	0.806	0	0
**Non-fatal myocardial infarction**	Metformin *vs* Thiazolidinediones	0.79 (0.46 to 1.37)	NA	(0.32 to 1.93)	5	1.952	0.745	0	0
**Non-fatal myocardial infarction**	Non-steroidal MRAs *vs* Standard treatments	0.91 (0.73 to 1.13)	NA	NA	2	1.088	0.297	0.002	0.081
**Non-fatal myocardial infarction**	SGLT2 inhibitors *vs* Standard treatments	0.9 (0.82 to 0.98)	NA	(0.82 to 0.99)	27	19.64	0.808	0	0
**Non-fatal myocardial infarction**	SGLT2 inhibitors *vs* Sulfonylureas	0.67 (0.14 to 3.11)	NA	NA	4	4.343	0.227	0.754	0.309
**Non-fatal myocardial infarction**	Sulfonylureas *vs* Standard treatments	0.48 (0.12 to 1.81)	NA	NA	4	0.691	0.875	0	0
**Non-fatal myocardial infarction**	Sulfonylureas *vs* Thiazolidinediones	0.96 (0.66 to 1.39)	NA	(0.55 to 1.69)	12	7.529	0.755	0.028	0
**Non-fatal myocardial infarction**	Thiazolidinediones *vs* Standard treatments	0.97 (0.74 to 1.27)	NA	(0.63 to 1.49)	17	10.91	0.815	0.021	0
**Non-fatal myocardial infarction**	Tirzepatide *vs* Standard treatments	0.2 (0.01 to 5.12)	NA	NA	1	0	NA	NA	NA
**Non-fatal stroke**	Alpha-glucosidase inhibitors *vs* Meglitinides	4.96 (0.24 to 103.63)	NA	NA	1	0	NA	NA	NA
**Non-fatal stroke**	Alpha-glucosidase inhibitors *vs* Sulfonylureas	10 (0.48 to 208.75)	NA	NA	1	0	NA	NA	NA
**Non-fatal stroke**	Basal insulin *vs* Basal-Bolus insulin	1.95 (0.18 to 21.64)	NA	NA	1	0	NA	NA	NA
**Non-fatal stroke**	Basal insulin *vs* DPP-4 inhibitors	3.36 (0.14 to 82.76)	NA	NA	1	0	NA	NA	NA
**Non-fatal stroke**	Basal insulin *vs* GLP-1 receptor agonists	0.81 (0.32 to 2.08)	NA	(0.27 to 2.41)	11	5.464	0.858	0	0
**Non-fatal stroke**	Basal insulin *vs* Thiazolidinediones	0.32 (0.01 to 8.26)	NA	NA	1	0	NA	NA	NA
**Non-fatal stroke**	Basal-Bolus insulin *vs* GLP-1 receptor agonists	1.03 (0.06 to 16.62)	NA	NA	1	0	NA	NA	NA
**Non-fatal stroke**	Basal-Bolus insulin *vs* Thiazolidinediones	0.99 (0.06 to 15.92)	NA	NA	1	0	NA	NA	NA
**Non-fatal stroke**	Bolus insulin *vs* GLP-1 receptor agonists	1.01 (0.19 to 5.24)	NA	NA	3	0.914	0.633	0	0
**Non-fatal stroke**	DPP-4 inhibitors *vs* GLP-1 receptor agonists	0.98 (0.34 to 2.82)	NA	(0.26 to 3.67)	8	4.43	0.729	0	0
**Non-fatal stroke**	DPP-4 inhibitors *vs* Metformin	0.79 (0.2 to 3.13)	NA	(0.08 to 7.39)	5	2.609	0.625	0	0

Outcome	Comparisons	Odds ratio	Mean difference	Prediction interval	k	Q	P-value	tau^2	I^2
**Non-fatal stroke**	DPP-4 inhibitors *vs* SGLT2 inhibitors	0.93 (0.28 to 3.06)	NA	(0.17 to 5.02)	6	2.14	0.83	0	0
**Non-fatal stroke**	DPP-4 inhibitors *vs* Standard treatments	0.93 (0.82 to 1.07)	NA	(0.81 to 1.07)	40	22.756	0.982	0	0
**Non-fatal stroke**	DPP-4 inhibitors *vs* Sulfonylureas	0.66 (0.41 to 1.07)	NA	(0.28 to 1.56)	13	8.892	0.712	0.091	0
**Non-fatal stroke**	DPP-4 inhibitors *vs* Thiazolidinediones	0.7 (0.2 to 2.45)	NA	NA	4	1.472	0.689	0	0
**Non-fatal stroke**	GLP-1 receptor agonists *vs* Metformin	0.7 (0.07 to 6.77)	NA	NA	2	0.428	0.513	0	0
**Non-fatal stroke**	GLP-1 receptor agonists *vs* SGLT2 inhibitors	3.68 (0.6 to 22.52)	NA	NA	2	0.021	0.884	0	0
**Non-fatal stroke**	GLP-1 receptor agonists *vs* Standard treatments	0.85 (0.76 to 0.94)	NA	(0.76 to 0.94)	30	19.597	0.905	0	0
**Non-fatal stroke**	GLP-1 receptor agonists *vs* Sulfonylureas	0.87 (0.21 to 3.52)	NA	NA	4	3.288	0.349	0	0.088
**Non-fatal stroke**	GLP-1 receptor agonists *vs* Thiazolidinediones	0.62 (0.1 to 3.98)	NA	NA	3	0.227	0.893	0	0
**Non-fatal stroke**	Meglitinides *vs* Standard treatments	0.34 (0.01 to 8.71)	NA	NA	1	0	NA	NA	NA
**Non-fatal stroke**	Meglitinides *vs* Sulfonylureas	3.74 (0.36 to 38.67)	NA	NA	2	0.148	0.7	0	0
**Non-fatal stroke**	Metformin *vs* Standard treatments	0.97 (0.63 to 1.47)	NA	NA	3	0.121	0.941	0	0
**Non-fatal stroke**	Metformin *vs* Sulfonylureas	0.87 (0.49 to 1.55)	NA	NA	2	1.234	0.267	0.034	0.19
**Non-fatal stroke**	Metformin *vs* Thiazolidinediones	1.26 (0.67 to 2.4)	NA	NA	3	0.41	0.815	0	0
**Non-fatal stroke**	Non-steroidal MRAs *vs* Standard treatments	1 (0.82 to 1.22)	NA	NA	2	0.122	0.727	0	0
**Non-fatal stroke**	SGLT2 inhibitors *vs* Standard treatments	0.99 (0.87 to 1.12)	NA	(0.84 to 1.16)	27	12.546	0.988	0.002	0
**Non-fatal stroke**	SGLT2 inhibitors *vs* Sulfonylureas	2.33 (0.71 to 7.6)	NA	(0.44 to 12.44)	6	2.078	0.838	0	0
**Non-fatal stroke**	Sulfonylureas *vs* Standard treatments	0.86 (0.19 to 3.81)	NA	NA	4	1.743	0.627	0	0
**Non-fatal stroke**	Sulfonylureas *vs* Thiazolidinediones	1.1 (0.74 to 1.63)	NA	(0.68 to 1.77)	9	4.939	0.764	0	0
**Non-fatal stroke**	Thiazolidinediones *vs* Standard treatments	0.81 (0.65 to 1.02)	NA	(0.63 to 1.05)	11	2.308	0.993	0	0
**Hospitalisation for heart failure**	Alpha-glucosidase inhibitors *vs* Standard treatments	3.25 (0.13 to 82.49)	NA	NA	1	0	NA	NA	NA
**Hospitalisation for heart failure**	Basal insulin *vs* DPP-4 inhibitors	0.87 (0.51 to 1.48)	NA	NA	1	0	NA	NA	NA
**Hospitalisation for heart failure**	Basal insulin *vs* GLP-1 receptor agonists	1.6 (0.91 to 2.84)	NA	(0.63 to 4.05)	5	2.606	0.626	0	0
**Hospitalisation for heart failure**	Basal insulin *vs* Standard treatments	0.33 (0.03 to 3.16)	NA	NA	2	0	0.996	0	0
**Hospitalisation for heart failure**	Basal insulin *vs* Sulfonylureas	0.86 (0.5 to 1.46)	NA	NA	1	0	NA	NA	NA
**Hospitalisation for heart failure**	Basal insulin *vs* Thiazolidinediones	0.34 (0.01 to 8.53)	NA	NA	1	0	NA	NA	NA

Outcome	Comparisons	Odds ratio	Mean difference	Prediction interval	k	Q	P-value	tau^2	I^2
**Hospitalisation for heart failure**	Basal insulin *vs* Tirzepatide	1.5 (0.42 to 5.32)	NA	NA	1	0	NA	NA	NA
**Hospitalisation for heart failure**	Bolus insulin *vs* GLP-1 receptor agonists	0.71 (0.07 to 6.85)	NA	NA	2	0.422	0.516	0	0
**Hospitalisation for heart failure**	DPP-4 inhibitors *vs* GLP-1 receptor agonists	1.71 (1.04 to 2.82)	NA	(0.92 to 3.19)	8	3.247	0.861	0	0
**Hospitalisation for heart failure**	DPP-4 inhibitors *vs* Metformin	1.51 (0.03 to 76.36)	NA	NA	1	0	NA	NA	NA
**Hospitalisation for heart failure**	DPP-4 inhibitors *vs* SGLT2 inhibitors	0.4 (0.02 to 9.88)	NA	NA	1	0	NA	NA	NA
**Hospitalisation for heart failure**	DPP-4 inhibitors *vs* Standard treatments	0.98 (0.8 to 1.19)	NA	(0.64 to 1.48)	25	20.661	0.659	0.031	0
**Hospitalisation for heart failure**	DPP-4 inhibitors *vs* Sulfonylureas	1.12 (0.89 to 1.42)	NA	(0.87 to 1.46)	13	8.817	0.718	0	0
**Hospitalisation for heart failure**	DPP-4 inhibitors *vs* Thiazolidinediones	0.33 (0.01 to 8.19)	NA	NA	1	0	NA	NA	NA
**Hospitalisation for heart failure**	GLP-1 receptor agonists *vs* Metformin	0.36 (0.05 to 2.81)	NA	NA	2	0.35	0.554	0	0
**Hospitalisation for heart failure**	GLP-1 receptor agonists *vs* SGLT2 inhibitors	0.5 (0.05 to 5.54)	NA	NA	1	0	NA	NA	NA
**Hospitalisation for heart failure**	GLP-1 receptor agonists *vs* Standard treatments	0.92 (0.84 to 1.01)	NA	(0.84 to 1.02)	22	11.875	0.943	0	0
**Hospitalisation for heart failure**	GLP-1 receptor agonists *vs* Sulfonylureas	0.73 (0.28 to 1.9)	NA	NA	4	2.701	0.44	0.264	0
**Hospitalisation for heart failure**	GLP-1 receptor agonists *vs* Thiazolidinediones	0.22 (0.01 to 5.38)	NA	NA	1	0	NA	NA	NA
**Hospitalisation for heart failure**	Metformin *vs* SGLT2 inhibitors	1.43 (0.06 to 35.52)	NA	NA	1	0	NA	NA	NA
**Hospitalisation for heart failure**	Metformin *vs* Thiazolidinediones	0.22 (0.01 to 5.43)	NA	NA	1	0	NA	NA	NA
**Hospitalisation for heart failure**	Non-steroidal MRAs *vs* Standard treatments	0.78 (0.64 to 0.94)	NA	NA	2	1.25	0.264	0.004	0.2
**Hospitalisation for heart failure**	SGLT2 inhibitors *vs* Standard treatments	0.66 (0.6 to 0.72)	NA	(0.59 to 0.73)	26	12.758	0.979	0	0
**Hospitalisation for heart failure**	SGLT2 inhibitors *vs* Sulfonylureas	0.85 (0.23 to 3.17)	NA	(0.1 to 7.22)	5	1.95	0.745	0	0
**Hospitalisation for heart failure**	Sulfonylureas *vs* Standard treatments	0.76 (0.06 to 9.06)	NA	NA	2	0.07	0.791	0	0
**Hospitalisation for heart failure**	Sulfonylureas *vs* Thiazolidinediones	0.76 (0.42 to 1.39)	NA	(0.37 to 1.57)	9	3.151	0.925	0	0
**Hospitalisation for heart failure**	Thiazolidinediones *vs* Standard treatments	1.59 (1.25 to 2.01)	NA	(1.16 to 2.17)	20	9.692	0.96	0.008	0
**End-stage kidney disease**	Basal insulin *vs* GLP-1 receptor agonists	1.42 (0.74 to 2.74)	NA	NA	2	0.224	0.636	0	0
**End-stage kidney disease**	Basal insulin *vs* Tirzepatide	1.99 (0.18 to 22)	NA	NA	1	0	NA	NA	NA
**End-stage kidney disease**	Bolus insulin *vs* GLP-1 receptor agonists	3.05 (0.12 to 75.27)	NA	NA	1	0	NA	NA	NA
**End-stage kidney disease**	DPP-4 inhibitors *vs* GLP-1 receptor agonists	1.36 (0.41 to 4.47)	NA	NA	3	0.433	0.805	0	0
**End-stage kidney disease**	DPP-4 inhibitors *vs* Standard treatments	1.04 (0.93 to 1.16)	NA	(0.91 to 1.19)	9	4.132	0.845	0	0

Outcome	Comparisons	Odds ratio	Mean difference	Prediction interval	k	Q	P-value	tau^2	I^2
**End-stage kidney disease**	DPP-4 inhibitors *vs* Sulfonylureas	1.47 (0.76 to 2.83)	NA	NA	3	0.903	0.637	0	0
**End-stage kidney disease**	GLP-1 receptor agonists *vs* Standard treatments	0.84 (0.74 to 0.95)	NA	(0.68 to 1.03)	11	6.597	0.763	0.004	0
**End-stage kidney disease**	GLP-1 receptor agonists *vs* Tirzepatide	1 (0.04 to 24.59)	NA	NA	1	0	NA	NA	NA
**End-stage kidney disease**	Metformin *vs* Standard treatments	1.61 (0.36 to 7.24)	NA	NA	1	0	NA	NA	NA
**End-stage kidney disease**	Non-steroidal MRAs *vs* Standard treatments	0.83 (0.75 to 0.92)	NA	NA	2	0.496	0.481	0	0
**End-stage kidney disease**	SGLT2 inhibitors *vs* Standard treatments	0.63 (0.53 to 0.75)	NA	(0.4 to 0.99)	15	24.407	0.041	0.036	0.426
**End-stage kidney disease**	SGLT2 inhibitors *vs* Sulfonylureas	3.06 (0.12 to 75.3)	NA	NA	1	0	NA	NA	NA
**End-stage kidney disease**	Sulfonylureas *vs* Thiazolidinediones	0.99 (0.82 to 1.19)	NA	NA	3	0.91	0.634	0	0
**End-stage kidney disease**	Thiazolidinediones *vs* Standard treatments	0.75 (0.17 to 3.36)	NA	NA	1	0	NA	NA	NA
**Severe hypoglycaemia**	Alpha-glucosidase inhibitors *vs* Meglitinides	1.01 (0.02 to 51.04)	NA	NA	1	0	NA	NA	NA
**Severe hypoglycaemia**	Alpha-glucosidase inhibitors *vs* SGLT2 inhibitors	3.07 (0.12 to 76.55)	NA	NA	1	0	NA	NA	NA
**Severe hypoglycaemia**	Alpha-glucosidase inhibitors *vs* Standard treatments	0.74 (0.1 to 5.57)	NA	NA	2	1.14	0.286	0.284	0.123
**Severe hypoglycaemia**	Alpha-glucosidase inhibitors *vs* Sulfonylureas	0.67 (0.03 to 16.5)	NA	NA	1	0	NA	NA	NA
**Severe hypoglycaemia**	Basal insulin *vs* Basal-Bolus insulin	0.66 (0.11 to 3.99)	NA	NA	1	0	NA	NA	NA
**Severe hypoglycaemia**	Basal insulin *vs* DPP-4 inhibitors	1.99 (0.92 to 4.29)	NA	NA	3	0.575	0.75	0	0
**Severe hypoglycaemia**	Basal insulin *vs* GLP-1 receptor agonists	2.18 (1.17 to 4.09)	NA	(0.28 to 17.25)	15	35.841	0.001	0.813	0.609
**Severe hypoglycaemia**	Basal insulin *vs* SGLT2 inhibitors	3.31 (0.13 to 81.76)	NA	NA	1	0	NA	NA	NA
**Severe hypoglycaemia**	Basal insulin *vs* Standard treatments	0.49 (0.01 to 24.98)	NA	NA	1	0	NA	NA	NA
**Severe hypoglycaemia**	Basal insulin *vs* Sulfonylureas	0.56 (0.3 to 1.04)	NA	NA	1	0	NA	NA	NA
**Severe hypoglycaemia**	Basal insulin *vs* Thiazolidinediones	1.78 (0.13 to 24.43)	NA	NA	2	4.287	0.038	2.759	0.767
**Severe hypoglycaemia**	Basal insulin *vs* Tirzepatide	2.45 (0.83 to 7.23)	NA	NA	2	0.344	0.557	0	0
**Severe hypoglycaemia**	Basal-Bolus insulin *vs* GLP-1 receptor agonists	10.96 (0.64 to 188.62)	NA	NA	1	0	NA	NA	NA
**Severe hypoglycaemia**	Bolus insulin *vs* GLP-1 receptor agonists	2.52 (1.35 to 4.72)	NA	(1.11 to 5.74)	7	0.554	0.997	0	0
**Severe hypoglycaemia**	DPP-4 inhibitors *vs* GLP-1 receptor agonists	1.34 (0.91 to 1.99)	NA	(0.73 to 2.46)	7	5.196	0.519	0.015	0
**Severe hypoglycaemia**	DPP-4 inhibitors *vs* Metformin	1.02 (0.05 to 22.76)	NA	NA	2	2.086	0.149	2.616	0.521
**Severe hypoglycaemia**	DPP-4 inhibitors *vs* SGLT2 inhibitors	0.64 (0.26 to 1.58)	NA	NA	3	0.84	0.657	0	0

Outcome	Comparisons	Odds ratio	Mean difference	Prediction interval	k	Q	P-value	tau^2	I^2
**Severe hypoglycaemia**	DPP-4 inhibitors *vs* Standard treatments	1.1 (0.99 to 1.24)	NA	(0.98 to 1.24)	41	33.067	0.773	0	0
**Severe hypoglycaemia**	DPP-4 inhibitors *vs* Sulfonylureas	0.19 (0.13 to 0.27)	NA	(0.13 to 0.28)	16	8.896	0.883	0	0
**Severe hypoglycaemia**	DPP-4 inhibitors *vs* Thiazolidinediones	2.99 (0.54 to 16.61)	NA	NA	3	2.132	0.344	0.461	0.062
**Severe hypoglycaemia**	GLP-1 receptor agonists *vs* SGLT2 inhibitors	1.17 (0.54 to 2.55)	NA	NA	2	0.004	0.949	0	0
**Severe hypoglycaemia**	GLP-1 receptor agonists *vs* Standard treatments	0.99 (0.83 to 1.17)	NA	(0.59 to 1.64)	38	47.038	0.125	0.055	0.213
**Severe hypoglycaemia**	GLP-1 receptor agonists *vs* Sulfonylureas	0.5 (0.23 to 1.1)	NA	NA	3	1.605	0.448	0.066	0
**Severe hypoglycaemia**	GLP-1 receptor agonists *vs* Thiazolidinediones	0.54 (0.1 to 2.82)	NA	NA	2	0.193	0.661	0	0
**Severe hypoglycaemia**	GLP-1 receptor agonists *vs* Tirzepatide	0.6 (0.03 to 12.51)	NA	NA	1	0	NA	NA	NA
**Severe hypoglycaemia**	Meglitinides *vs* Metformin	2.34 (0.66 to 8.34)	NA	NA	1	0	NA	NA	NA
**Severe hypoglycaemia**	Meglitinides *vs* Sulfonylureas	0.34 (0.04 to 3.11)	NA	NA	2	0.311	0.577	0	0
**Severe hypoglycaemia**	Metformin *vs* SGLT2 inhibitors	3.66 (0.87 to 15.44)	NA	NA	2	0.117	0.732	0	0
**Severe hypoglycaemia**	Metformin *vs* Standard treatments	2.79 (0.27 to 28.45)	NA	NA	2	4.044	0.044	2.16	0.753
**Severe hypoglycaemia**	Metformin *vs* Sulfonylureas	0.46 (0.02 to 9.72)	NA	NA	2	2.583	0.108	3.036	0.613
**Severe hypoglycaemia**	Metformin *vs* Thiazolidinediones	1 (0.06 to 16.02)	NA	NA	1	0	NA	NA	NA
**Severe hypoglycaemia**	Non-steroidal MRAs *vs* Standard treatments	0.64 (0.43 to 0.96)	NA	NA	2	0.078	0.78	0	0
**Severe hypoglycaemia**	SGLT2 inhibitors *vs* Standard treatments	0.89 (0.78 to 1.02)	NA	(0.77 to 1.03)	28	22.858	0.693	0	0
**Severe hypoglycaemia**	SGLT2 inhibitors *vs* Sulfonylureas	0.17 (0.07 to 0.43)	NA	NA	3	0.201	0.905	0	0
**Severe hypoglycaemia**	Sulfonylureas *vs* Standard treatments	1.42 (0.12 to 17.07)	NA	NA	2	0.451	0.502	0	0
**Severe hypoglycaemia**	Sulfonylureas *vs* Thiazolidinediones	8.79 (3.06 to 25.28)	NA	(1.97 to 39.26)	6	1.9	0.863	0	0
**Severe hypoglycaemia**	Thiazolidinediones *vs* Standard treatments	1.77 (1.1 to 2.85)	NA	(1.01 to 3.09)	10	3.802	0.924	0	0
**Severe hypoglycaemia**	Tirzepatide *vs* Standard treatments	2.39 (0.12 to 46.66)	NA	NA	1	0	NA	NA	NA
**Severe gastrointestinal events**	Alpha-glucosidase inhibitors *vs* Metformin	1.53 (0.25 to 9.54)	NA	NA	1	0	NA	NA	NA
**Severe gastrointestinal events**	Basal insulin *vs* DPP-4 inhibitors	1.11 (0.07 to 17.92)	NA	NA	1	0	NA	NA	NA
**Severe gastrointestinal events**	Basal insulin *vs* GLP-1 receptor agonists	0.3 (0.07 to 1.36)	NA	NA	1	0	NA	NA	NA
**Severe gastrointestinal events**	Basal insulin *vs* Tirzepatide	0.04 (0.01 to 0.19)	NA	NA	2	0.848	0.357	0	0
**Severe gastrointestinal events**	Bolus insulin *vs* GLP-1 receptor agonists	1.67 (0.4 to 7.01)	NA	NA	1	0	NA	NA	NA

Outcome	Comparisons	Odds ratio	Mean difference	Prediction interval	k	Q	P-value	tau^2	I^2
**Severe gastrointestinal events**	DPP-4 inhibitors *vs* GLP-1 receptor agonists	0.63 (0.16 to 2.51)	NA	NA	2	2.758	0.097	0.632	0.637
**Severe gastrointestinal events**	DPP-4 inhibitors *vs* Standard treatments	1.75 (0.49 to 6.27)	NA	(0.22 to 13.89)	5	2.002	0.735	0	0
**Severe gastrointestinal events**	DPP-4 inhibitors *vs* Sulfonylureas	0.72 (0.16 to 3.24)	NA	NA	1	0	NA	NA	NA
**Severe gastrointestinal events**	GLP-1 receptor agonists *vs* Standard treatments	2.02 (1.32 to 3.1)	NA	(0.49 to 8.25)	15	63.649	0	0.377	0.78
**Severe gastrointestinal events**	GLP-1 receptor agonists *vs* Tirzepatide	0.71 (0.39 to 1.3)	NA	NA	2	0.218	0.64	0	0
**Severe gastrointestinal events**	Metformin *vs* Standard treatments	1.99 (0.18 to 22.14)	NA	NA	1	0	NA	NA	NA
**Severe gastrointestinal events**	Metformin *vs* Sulfonylureas	2.32 (0.6 to 8.98)	NA	NA	1	0	NA	NA	NA
**Severe gastrointestinal events**	Metformin *vs* Thiazolidinediones	2.5 (0.18 to 35.32)	NA	NA	2	3.152	0.076	2.657	0.683
**Severe gastrointestinal events**	Sulfonylureas *vs* Thiazolidinediones	0.38 (0.1 to 1.43)	NA	NA	1	0	NA	NA	NA
**Severe gastrointestinal events**	Thiazolidinediones *vs* Standard treatments	1.16 (0.91 to 1.48)	NA	NA	2	0.674	0.412	0	0
**Severe gastrointestinal events**	Tirzepatide *vs* Standard treatments	5.76 (1.33 to 24.97)	NA	NA	3	0.824	0.662	0	0
**Genital infection**	DPP-4 inhibitors *vs* SGLT2 inhibitors	0.26 (0.16 to 0.42)	NA	(0.15 to 0.45)	10	5.42	0.796	0	0
**Genital infection**	DPP-4 inhibitors *vs* Standard treatments	0.47 (0.2 to 1.1)	NA	(0.14 to 1.56)	6	4.096	0.536	0	0
**Genital infection**	GLP-1 receptor agonists *vs* SGLT2 inhibitors	0.18 (0.08 to 0.39)	NA	NA	2	1.124	0.289	0.034	0.11
**Genital infection**	GLP-1 receptor agonists *vs* Standard treatments	1.14 (0.22 to 5.83)	NA	NA	4	3.579	0.311	0.553	0.162
**Genital infection**	Metformin *vs* SGLT2 inhibitors	0.39 (0.21 to 0.73)	NA	NA	4	4.091	0.252	0	0.267
**Genital infection**	SGLT2 inhibitors *vs* Standard treatments	3.31 (2.84 to 3.85)	NA	(2.5 to 4.38)	63	58.967	0.586	0.014	0
**Genital infection**	SGLT2 inhibitors *vs* Sulfonylureas	6.32 (4.53 to 8.82)	NA	(3.94 to 10.14)	6	2.219	0.818	0	0
**Genital infection**	SGLT2 inhibitors *vs* Thiazolidinediones	0.3 (0.01 to 7.75)	NA	NA	1	0	NA	NA	NA
**Amputation**	DPP-4 inhibitors *vs* SGLT2 inhibitors	0.66 (0.03 to 16.38)	NA	NA	1	0	NA	NA	NA
**Amputation**	DPP-4 inhibitors *vs* Standard treatments	0.91 (0.64 to 1.29)	NA	NA	1	0	NA	NA	NA
**Amputation**	GLP-1 receptor agonists *vs* Standard treatments	0.71 (0.07 to 6.79)	NA	NA	2	0.423	0.516	0	0
**Amputation**	Non-steroidal MRAs *vs* Standard treatments	0.94 (0.46 to 1.9)	NA	NA	2	0.3	0.584	0	0
**Amputation**	SGLT2 inhibitors *vs* Standard treatments	1.28 (1 to 1.64)	NA	(0.66 to 2.48)	10	18.213	0.033	0.067	0.506
**Amputation**	Thiazolidinediones *vs* Standard treatments	0.63 (0.22 to 1.85)	NA	NA	2	3.573	0.059	0.444	0.72
**Ketoacidosis due to diabetes**	Alpha-glucosidase inhibitors *vs* Meglitinides	1.01 (0.02 to 51.04)	NA	NA	1	0	NA	NA	NA

Outcome	Comparisons	Odds ratio	Mean difference	Prediction interval	k	Q	P-value	tau^2	I^2
**Ketoacidosis due to diabetes**	Alpha-glucosidase inhibitors *vs* Sulfonylureas	0.67 (0.03 to 16.5)	NA	NA	1	0	NA	NA	NA
**Ketoacidosis due to diabetes**	DPP-4 inhibitors *vs* GLP-1 receptor agonists	0.33 (0.01 to 8.15)	NA	NA	1	0	NA	NA	NA
**Ketoacidosis due to diabetes**	DPP-4 inhibitors *vs* SGLT2 inhibitors	1.41 (0.08 to 24.61)	NA	NA	2	1.581	0.209	1.562	0.368
**Ketoacidosis due to diabetes**	DPP-4 inhibitors *vs* Standard treatments	0.71 (0.34 to 1.49)	NA	(0.27 to 1.88)	7	2.336	0.886	0	0
**Ketoacidosis due to diabetes**	DPP-4 inhibitors *vs* Sulfonylureas	2.13 (0.22 to 20.51)	NA	NA	2	0.095	0.757	0	0
**Ketoacidosis due to diabetes**	GLP-1 receptor agonists *vs* Standard treatments	1.02 (0.55 to 1.88)	NA	(0.46 to 2.27)	7	3.96	0.682	0	0
**Ketoacidosis due to diabetes**	Meglitinides *vs* Sulfonylureas	0.66 (0.03 to 16.32)	NA	NA	1	0	NA	NA	NA
**Ketoacidosis due to diabetes**	Metformin *vs* SGLT2 inhibitors	0.67 (0.03 to 16.41)	NA	NA	1	0	NA	NA	NA
**Ketoacidosis due to diabetes**	Non-steroidal MRAs *vs* Standard treatments	0.67 (0.26 to 1.73)	NA	NA	2	1.316	0.251	0.118	0.24
**Ketoacidosis due to diabetes**	SGLT2 inhibitors *vs* Standard treatments	2.17 (1.5 to 3.13)	NA	(1.43 to 3.29)	12	9.131	0.61	0	0
**Ketoacidosis due to diabetes**	SGLT2 inhibitors *vs* Sulfonylureas	1.49 (0.06 to 36.54)	NA	NA	1	0	NA	NA	NA
**Health-related quality of life**	Alpha-glucosidase inhibitors *vs* DPP-4 inhibitors	NA	0 (-0.18 to 0.18)	NA	1	0	NA	NA	NA
**Health-related quality of life**	Basal insulin *vs* Bolus insulin	NA	0.42 (0.21 to 0.62)	NA	1	0	NA	NA	NA
**Health-related quality of life**	Basal insulin *vs* GLP-1 receptor agonists	NA	-0.2 (-0.47 to 0.07)	NA	1	0	NA	NA	NA
**Health-related quality of life**	Basal insulin *vs* Tirzepatide	NA	-0.62 (-0.75 to -0.49)	NA	1	0	NA	NA	NA
**Health-related quality of life**	Bolus insulin *vs* GLP-1 receptor agonists	NA	-0.21 (-0.29 to -0.12)	NA	4	2.823	0.42	0.001	0
**Health-related quality of life**	DPP-4 inhibitors *vs* GLP-1 receptor agonists	NA	-0.16 (-0.28 to -0.03)	(-0.53 to 0.22)	5	8.28	0.082	0.01	0.517
**Health-related quality of life**	DPP-4 inhibitors *vs* Metformin	NA	0.01 (-0.19 to 0.21)	NA	2	0.029	0.864	0	0
**Health-related quality of life**	DPP-4 inhibitors *vs* SGLT2 inhibitors	NA	-0.26 (-0.53 to 0)	NA	1	0	NA	NA	NA
**Health-related quality of life**	DPP-4 inhibitors *vs* Standard treatments	NA	0.08 (-0.07 to 0.24)	NA	2	0.202	0.653	0	0
**Health-related quality of life**	DPP-4 inhibitors *vs* Thiazolidinediones	NA	-0.15 (-0.38 to 0.09)	NA	1	0	NA	NA	NA
**Health-related quality of life**	GLP-1 receptor agonists *vs* Metformin	NA	0.11 (-0.07 to 0.3)	NA	1	0	NA	NA	NA
**Health-related quality of life**	GLP-1 receptor agonists *vs* SGLT2 inhibitors	NA	-0.27 (-0.89 to 0.35)	NA	2	3.319	0.068	0.15	0.699
**Health-related quality of life**	GLP-1 receptor agonists *vs* Standard treatments	NA	0.15 (0.1 to 0.2)	(0.09 to 0.21)	11	14.87	0.137	0	0.327
**Health-related quality of life**	GLP-1 receptor agonists *vs* Thiazolidinediones	NA	-0.05 (-0.26 to 0.16)	NA	1	0	NA	NA	NA
**Health-related quality of life**	GLP-1 receptor agonists *vs* Tirzepatide	NA	0 (-0.11 to 0.11)	NA	1	0	NA	NA	NA

Outcome	Comparisons	Odds ratio	Mean difference	Prediction interval	k	Q	P-value	tau^2	I^2
**Health-related quality of life**	Meglitinides *vs* Thiazolidinediones	NA	-0.03 (-0.21 to 0.15)	NA	1	0	NA	NA	NA
**Health-related quality of life**	Metformin *vs* Thiazolidinediones	NA	-0.16 (-0.37 to 0.05)	NA	1	0	NA	NA	NA
**Health-related quality of life**	SGLT2 inhibitors *vs* Standard treatments	NA	0.23 (-0.63 to 1.09)	NA	2	12.101	0.001	0.353	0.917
**Health-related quality of life**	Sulfonylureas *vs* Standard treatments	NA	0.23 (-0.08 to 0.54)	NA	1	0	NA	NA	NA
**Body weight change**	Albiglutide *vs* Basal insulin	NA	-2.61 (-3.2 to -2.02)	NA	1	0	NA	NA	NA
**Body weight change**	Albiglutide *vs* Bolus insulin	NA	-2.97 (-5.77 to -0.17)	NA	2	52.544	0	4.012	0.981
**Body weight change**	Albiglutide *vs* DPP-4 inhibitors	NA	-0.5 (-0.92 to -0.09)	NA	2	0.333	0.564	0	0
**Body weight change**	Albiglutide *vs* Standard treatments	NA	-0.2 (-0.81 to 0.41)	(-2.23 to 1.82)	5	15.941	0.003	0.308	0.749
**Body weight change**	Albiglutide *vs* Sulfonylureas	NA	-2.38 (-3.04 to -1.72)	NA	1	0	NA	NA	NA
**Body weight change**	Albiglutide *vs* Thiazolidinediones	NA	-4.01 (-4.66 to -3.36)	NA	1	0	NA	NA	NA
**Body weight change**	Alpha-glucosidase inhibitors *vs* DPP-4 inhibitors	NA	-0.73 (-1.51 to 0.04)	(-3.39 to 1.92)	6	20.348	0.001	0.76	0.754
**Body weight change**	Alpha-glucosidase inhibitors *vs* Metformin	NA	0.57 (-0.69 to 1.84)	NA	3	13.358	0.001	1.034	0.85
**Body weight change**	Alpha-glucosidase inhibitors *vs* SGLT2 inhibitors	NA	0.27 (-1.94 to 2.48)	NA	1	0	NA	NA	NA
**Body weight change**	Alpha-glucosidase inhibitors *vs* Standard treatments	NA	-0.55 (-0.9 to -0.2)	(-1.47 to 0.37)	12	17.075	0.106	0.139	0.356
**Body weight change**	Alpha-glucosidase inhibitors *vs* Sulfonylureas	NA	-0.91 (-2.06 to 0.25)	NA	2	0.182	0.67	0	0
**Body weight change**	Alpha-glucosidase inhibitors *vs* Thiazolidinediones	NA	-2.62 (-4.01 to -1.22)	NA	2	3.063	0.08	0.679	0.674
**Body weight change**	Basal insulin *vs* Basal-Bolus insulin	NA	-0.87 (-2.47 to 0.72)	NA	2	13.63	0	1.231	0.927
**Body weight change**	Basal insulin *vs* DPP-4 inhibitors	NA	1.73 (0.84 to 2.63)	NA	4	10.911	0.012	0.597	0.725
**Body weight change**	Basal insulin *vs* Dulaglutide	NA	3.06 (2.49 to 3.63)	(1.31 to 4.8)	5	9.536	0.049	0.216	0.581
**Body weight change**	Basal insulin *vs* Exenatide extended-release	NA	3.21 (2.16 to 4.25)	NA	4	31.429	0	0.864	0.905
**Body weight change**	Basal insulin *vs* Exenatide immediate-release	NA	4.07 (3.54 to 4.59)	NA	2	0.148	0.7	0	0
**Body weight change**	Basal insulin *vs* Liraglutide	NA	4.16 (3.42 to 4.9)	(1.8 to 6.52)	8	67.722	0	0.786	0.897
**Body weight change**	Basal insulin *vs* Semaglutide (Subcutaneous)	NA	7.48 (6.68 to 8.28)	NA	1	0	NA	NA	NA
**Body weight change**	Basal insulin *vs* Standard treatments	NA	1.71 (0.43 to 2.98)	(-2.91 to 6.32)	5	24.999	0	1.682	0.84
**Body weight change**	Basal insulin *vs* Sulfonylureas	NA	0.5 (-0.82 to 1.83)	NA	2	1.834	0.176	0.568	0.455
**Body weight change**	Basal insulin *vs* Thiazolidinediones	NA	-1.3 (-2.28 to -0.32)	NA	2	0	1	0	0

Outcome	Comparisons	Odds ratio	Mean difference	Prediction interval	k	Q	P-value	tau^2	I^2
**Body weight change**	Basal insulin *vs* Tirzepatide	NA	11.96 (10.67 to 13.24)	NA	2	5.865	0.015	0.718	0.829
**Body weight change**	Basal-Bolus insulin *vs* Thiazolidinediones	NA	1 (0.03 to 1.97)	NA	1	0	NA	NA	NA
**Body weight change**	Bolus insulin *vs* Liraglutide	NA	2.28 (-0.03 to 4.6)	NA	2	5.236	0.022	2.33	0.809
**Body weight change**	Bolus insulin *vs* Lixisenatide	NA	1.8 (1.08 to 2.52)	NA	1	0	NA	NA	NA
**Body weight change**	Bolus insulin *vs* Semaglutide (Subcutaneous)	NA	7.1 (6.67 to 7.53)	NA	1	0	NA	NA	NA
**Body weight change**	Bolus insulin *vs* SGLT2 inhibitors	NA	0.9 (0.43 to 1.37)	NA	1	0	NA	NA	NA
**Body weight change**	DPP-4 inhibitors *vs* Dulaglutide	NA	1.72 (1.22 to 2.22)	NA	1	0	NA	NA	NA
**Body weight change**	DPP-4 inhibitors *vs* Exenatide extended-release	NA	0.87 (-0.06 to 1.81)	NA	3	8.063	0.018	0.513	0.752
**Body weight change**	DPP-4 inhibitors *vs* Liraglutide	NA	1.93 (1.45 to 2.42)	(0.61 to 3.26)	6	10.795	0.056	0.167	0.537
**Body weight change**	DPP-4 inhibitors *vs* Lixisenatide	NA	1.3 (0.48 to 2.12)	NA	1	0	NA	NA	NA
**Body weight change**	DPP-4 inhibitors *vs* Metformin	NA	1.18 (0.9 to 1.46)	(0.34 to 2.02)	11	21.883	0.016	0.118	0.543
**Body weight change**	DPP-4 inhibitors *vs* Semaglutide (Oral)	NA	1.9 (1.19 to 2.61)	NA	1	0	NA	NA	NA
**Body weight change**	DPP-4 inhibitors *vs* Semaglutide (Subcutaneous)	NA	4.09 (3.53 to 4.64)	NA	2	0.261	0.609	0	0
**Body weight change**	DPP-4 inhibitors *vs* SGLT2 inhibitors	NA	2.34 (2.16 to 2.52)	(2.14 to 2.54)	14	20.513	0.083	0	0.366
**Body weight change**	DPP-4 inhibitors *vs* Standard treatments	NA	0.26 (0.13 to 0.39)	(-0.69 to 1.21)	78	251.979	0	0.222	0.694
**Body weight change**	DPP-4 inhibitors *vs* Sulfonylureas	NA	-1.47 (-1.97 to -0.96)	(-3.81 to 0.87)	19	364.206	0	1.162	0.951
**Body weight change**	DPP-4 inhibitors *vs* Thiazolidinediones	NA	-1.86 (-2.8 to -0.92)	(-5.48 to 1.76)	11	187.681	0	2.332	0.947
**Body weight change**	Dulaglutide *vs* Exenatide extended-release	NA	1.66 (-0.12 to 3.44)	NA	1	0	NA	NA	NA
**Body weight change**	Dulaglutide *vs* Metformin	NA	-0.07 (-0.73 to 0.59)	NA	1	0	NA	NA	NA
**Body weight change**	Dulaglutide *vs* Standard treatments	NA	-1.67 (-2.26 to -1.08)	(-3.55 to 0.21)	9	25.695	0.001	0.538	0.689
**Body weight change**	Dulaglutide *vs* Sulfonylureas	NA	-2.35 (-2.87 to -1.83)	NA	1	0	NA	NA	NA
**Body weight change**	Dulaglutide *vs* Tirzepatide	NA	3.71 (1.91 to 5.52)	NA	1	0	NA	NA	NA
**Body weight change**	Efpeglenatide *vs* Standard treatments	NA	-2.59 (-2.91 to -2.27)	NA	1	0	NA	NA	NA
**Body weight change**	Exenatide extended-release *vs* Metformin	NA	0 (-0.56 to 0.56)	NA	1	0	NA	NA	NA
**Body weight change**	Exenatide extended-release *vs* SGLT2 inhibitors	NA	2.22 (0.89 to 3.55)	NA	1	0	NA	NA	NA
**Body weight change**	Exenatide extended-release *vs* Standard treatments	NA	-1.36 (-1.9 to -0.82)	NA	4	1.831	0.608	0	0

Outcome	Comparisons	Odds ratio	Mean difference	Prediction interval	k	Q	P-value	tau^2	I^2
**Body weight change**	Exenatide extended-release *vs* Thiazolidinediones	NA	-4.28 (-5.85 to -2.71)	NA	2	8.075	0.004	1.121	0.876
**Body weight change**	Exenatide immediate-release *vs* Metformin	NA	-1.99 (-2.77 to -1.21)	NA	1	0	NA	NA	NA
**Body weight change**	Exenatide immediate-release *vs* Standard treatments	NA	-1.56 (-2.47 to -0.65)	(-4.59 to 1.48)	6	22.457	0	0.978	0.777
**Body weight change**	Liraglutide *vs* Semaglutide (Oral)	NA	1.3 (0.46 to 2.14)	NA	1	0	NA	NA	NA
**Body weight change**	Liraglutide *vs* Standard treatments	NA	-2.61 (-3.21 to -2.02)	(-5.22 to -0.01)	20	107.627	0	1.447	0.823
**Body weight change**	Liraglutide *vs* Sulfonylureas	NA	-3.34 (-4.01 to -2.68)	NA	3	10.074	0.006	0.261	0.801
**Body weight change**	Liraglutide *vs* Thiazolidinediones	NA	-2.3 (-3.14 to -1.46)	NA	1	0	NA	NA	NA
**Body weight change**	Lixisenatide *vs* Standard treatments	NA	-0.88 (-1.13 to -0.63)	(-1.26 to -0.49)	10	9.12	0.426	0.012	0.013
**Body weight change**	Loxenatide *vs* Standard treatments	NA	0.16 (-0.46 to 0.78)	NA	1	0	NA	NA	NA
**Body weight change**	Meglitinides *vs* Metformin	NA	2.51 (0.97 to 4.05)	NA	1	0	NA	NA	NA
**Body weight change**	Meglitinides *vs* Standard treatments	NA	1.04 (0.15 to 1.93)	NA	4	7.55	0.056	0.455	0.603
**Body weight change**	Meglitinides *vs* Sulfonylureas	NA	-0.47 (-0.97 to 0.04)	NA	4	3.182	0.364	0	0.057
**Body weight change**	Meglitinides *vs* Thiazolidinediones	NA	-1.25 (-2.64 to 0.13)	NA	2	0.496	0.481	0	0
**Body weight change**	Metformin *vs* SGLT2 inhibitors	NA	1.69 (1.2 to 2.18)	(0.17 to 3.21)	5	8.854	0.065	0.166	0.548
**Body weight change**	Metformin *vs* Standard treatments	NA	-0.66 (-1.41 to 0.1)	(-3.26 to 1.95)	8	103.872	0	0.985	0.933
**Body weight change**	Metformin *vs* Sulfonylureas	NA	-2.44 (-3.68 to -1.2)	(-6.71 to 1.83)	7	53.911	0	2.355	0.889
**Body weight change**	Metformin *vs* Thiazolidinediones	NA	-4.11 (-5.09 to -3.14)	(-7.66 to -0.56)	10	174.852	0	2.122	0.949
**Body weight change**	Semaglutide (Oral) *vs* Semaglutide (Subcutaneous)	NA	1.6 (0.08 to 3.12)	NA	1	0	NA	NA	NA
**Body weight change**	Semaglutide (Oral) *vs* SGLT2 inhibitors	NA	-0.3 (-1 to 0.4)	NA	1	0	NA	NA	NA
**Body weight change**	Semaglutide (Oral) *vs* Standard treatments	NA	-3.36 (-4.01 to -2.71)	(-5.37 to -1.35)	6	14.921	0.011	0.415	0.665
**Body weight change**	Semaglutide (Subcutaneous) *vs* SGLT2 inhibitors	NA	-1.4 (-1.93 to -0.87)	NA	1	0	NA	NA	NA
**Body weight change**	Semaglutide (Subcutaneous) *vs* Standard treatments	NA	-4.9 (-5.83 to -3.96)	(-7.88 to -1.91)	6	18.49	0.002	0.932	0.73
**Body weight change**	Semaglutide (Subcutaneous) *vs* Tirzepatide	NA	3.99 (3.27 to 4.72)	NA	2	0.063	0.801	0	0
**Body weight change**	SGLT2 inhibitors *vs* Standard treatments	NA	-1.9 (-2.1 to -1.7)	(-3.35 to -0.45)	70	700.548	0	0.519	0.902
**Body weight change**	SGLT2 inhibitors *vs* Sulfonylureas	NA	-4.3 (-4.74 to -3.86)	(-5.72 to -2.88)	9	26.466	0.001	0.309	0.698
**Body weight change**	SGLT2 inhibitors *vs* Thiazolidinediones	NA	-4.29 (-5.6 to -2.99)	NA	3	6.772	0.034	0.891	0.705

Outcome	Comparisons	Odds ratio	Mean difference	Prediction interval	k	Q	P-value	tau^2	I^2
**Body weight change**	Sulfonylureas *vs* Standard treatments	NA	2.78 (2.21 to 3.35)	NA	4	11.287	0.01	0.214	0.734
**Body weight change**	Sulfonylureas *vs* Thiazolidinediones	NA	-1.19 (-1.93 to -0.45)	(-4.42 to 2.04)	17	347.399	0	2.154	0.954
**Body weight change**	Thiazolidinediones *vs* Standard treatments	NA	3.02 (2.54 to 3.5)	(0.47 to 5.57)	32	297.98	0	1.5	0.896
**Body weight change**	Tirzepatide *vs* Standard treatments	NA	-8.19 (-10.1 to -6.28)	NA	4	11.87	0.008	2.941	0.747

4.4.2 Heterogeneity assessed by design-based decomposition of Cochran's Q

Notes: We also considered the heterogeneity in the network meta-analysis. The analysis performed a design-based decomposition of generalised Cochran's Q statistic for assessing the heterogeneity in the whole network, the heterogeneity within designs, and the heterogeneity/inconsistency between designs.

Outcome	Overall design	Specific design	Q	df	P-value
**All-cause death**	Total	Total	161.949	261	1
**All-cause death**	Total	Within designs	133.778	212	1
**All-cause death**	Total	Between designs	28.171	49	0.993
**All-cause death**	Total	tau.within	0	NA	NA
**All-cause death**	Within	Basal insulin *vs* Basal-Bolus insulin	0	0	NA
**All-cause death**	Within	Basal insulin *vs* Bolus insulin	0	0	NA
**All-cause death**	Within	Basal insulin *vs* DPP-4 inhibitors	1.688	2	0.43
**All-cause death**	Within	Basal insulin *vs* GLP-1 receptor agonists	3.941	7	0.787
**All-cause death**	Within	Basal insulin *vs* Tirzepatide	0.303	1	0.582
**All-cause death**	Within	Basal-Bolus insulin *vs* GLP-1 receptor agonists	0.283	1	0.595
**All-cause death**	Within	Basal-Bolus insulin *vs* Sulfonylureas	0	0	NA
**All-cause death**	Within	Bolus insulin *vs* GLP-1 receptor agonists	3.816	3	0.282
**All-cause death**	Within	DPP-4 inhibitors *vs* GLP-1 receptor agonists	2.953	7	0.889
**All-cause death**	Within	DPP-4 inhibitors *vs* Metformin	1.737	3	0.629
**All-cause death**	Within	DPP-4 inhibitors *vs* SGLT2 inhibitors	1.889	5	0.864
**All-cause death**	Within	DPP-4 inhibitors *vs* Sulfonylureas	7.458	14	0.916
**All-cause death**	Within	DPP-4 inhibitors *vs* Thiazolidinediones	0.096	1	0.757
**All-cause death**	Within	GLP-1 receptor agonists *vs* SGLT2 inhibitors	1.126	2	0.57
**All-cause death**	Within	GLP-1 receptor agonists *vs* Sulfonylureas	1.285	3	0.733
**All-cause death**	Within	GLP-1 receptor agonists *vs* Tirzepatide	0	0	NA
**All-cause death**	Within	Meglitinides *vs* Sulfonylureas	0.064	1	0.8
**All-cause death**	Within	Meglitinides *vs* Thiazolidinediones	0	0	NA

Outcome	Overall design	Specific design	Q	df	P-value
**All-cause death**	Within	Metformin *vs* SGLT2 inhibitors	1.722	2	0.423
**All-cause death**	Within	Metformin *vs* Sulfonylureas	2.51	2	0.285
**All-cause death**	Within	Metformin *vs* Thiazolidinediones	1.128	3	0.77
**All-cause death**	Within	SGLT2 inhibitors *vs* Sulfonylureas	3.736	4	0.443
**All-cause death**	Within	Standard treatments *vs* Alpha-glucosidase inhibitors	1.539	3	0.673
**All-cause death**	Within	Standard treatments *vs* Basal insulin	0	0	NA
**All-cause death**	Within	Standard treatments *vs* DPP-4 inhibitors	27.307	45	0.983
**All-cause death**	Within	Standard treatments *vs* GLP-1 receptor agonists	22.244	36	0.965
**All-cause death**	Within	Standard treatments *vs* Meglitinides	0	0	NA
**All-cause death**	Within	Standard treatments *vs* Metformin	3.284	2	0.194
**All-cause death**	Within	Standard treatments *vs* Non-steroidal MRAs	0.574	2	0.751
**All-cause death**	Within	Standard treatments *vs* SGLT2 inhibitors	27.268	37	0.879
**All-cause death**	Within	Standard treatments *vs* Thiazolidinediones	8.666	16	0.927
**All-cause death**	Within	Standard treatments *vs* Tirzepatide	0	0	NA
**All-cause death**	Within	Sulfonylureas *vs* Thiazolidinediones	6.329	8	0.61
**All-cause death**	Within	Alpha-glucosidase inhibitors *vs* Meglitinides *vs* Sulfonylureas	0	0	NA
**All-cause death**	Within	Basal insulin *vs* DPP-4 inhibitors *vs* GLP-1 receptor agonists *vs* Sulfonylureas	0	0	NA
**All-cause death**	Within	DPP-4 inhibitors *vs* GLP-1 receptor agonists *vs* Metformin *vs* Thiazolidinediones	0	0	NA
**All-cause death**	Within	DPP-4 inhibitors *vs* GLP-1 receptor agonists *vs* Thiazolidinediones	0	0	NA
**All-cause death**	Within	Metformin *vs* Sulfonylureas *vs* Thiazolidinediones	0.833	2	0.659
**All-cause death**	Within	Standard treatments *vs* Alpha-glucosidase inhibitors *vs* Sulfonylureas	0	0	NA
**All-cause death**	Within	Standard treatments *vs* DPP-4 inhibitors *vs* GLP-1 receptor agonists	0	0	NA
**All-cause death**	Within	Standard treatments *vs* DPP-4 inhibitors *vs* GLP-1 receptor agonists *vs* Sulfonylureas	0	0	NA
**All-cause death**	Within	Standard treatments *vs* DPP-4 inhibitors *vs* SGLT2 inhibitors	0	0	NA
**All-cause death**	Within	Standard treatments *vs* GLP-1 receptor agonists *vs* Thiazolidinediones	0	0	NA

Outcome	Overall design	Specific design	Q	df	P-value
**All-cause death**	Within	Standard treatments *vs* GLP-1 receptor agonists *vs* Tirzepatide	0	0	NA
**All-cause death**	Within	Standard treatments *vs* Meglitinides *vs* Metformin	0	0	NA
**All-cause death**	Within	Standard treatments *vs* Metformin *vs* Thiazolidinediones	0	0	NA
**All-cause death**	Between	Basal insulin *vs* Basal-Bolus insulin	26.665	48	0.995
**All-cause death**	Between	Basal insulin *vs* Bolus insulin	26.58	48	0.995
**All-cause death**	Between	Basal insulin *vs* DPP-4 inhibitors	27.615	48	0.992
**All-cause death**	Between	Basal insulin *vs* GLP-1 receptor agonists	28.171	48	0.99
**All-cause death**	Between	Basal insulin *vs* Tirzepatide	28.041	48	0.991
**All-cause death**	Between	Basal-Bolus insulin *vs* GLP-1 receptor agonists	28.094	48	0.99
**All-cause death**	Between	Basal-Bolus insulin *vs* Sulfonylureas	25.628	48	0.997
**All-cause death**	Between	Bolus insulin *vs* GLP-1 receptor agonists	26.58	48	0.995
**All-cause death**	Between	DPP-4 inhibitors *vs* GLP-1 receptor agonists	28.127	48	0.99
**All-cause death**	Between	DPP-4 inhibitors *vs* Metformin	28.167	48	0.99
**All-cause death**	Between	DPP-4 inhibitors *vs* SGLT2 inhibitors	25.801	48	0.996
**All-cause death**	Between	DPP-4 inhibitors *vs* Sulfonylureas	26.84	48	0.994
**All-cause death**	Between	DPP-4 inhibitors *vs* Thiazolidinediones	27.659	48	0.992
**All-cause death**	Between	GLP-1 receptor agonists *vs* SGLT2 inhibitors	28.067	48	0.99
**All-cause death**	Between	GLP-1 receptor agonists *vs* Sulfonylureas	28.115	48	0.99
**All-cause death**	Between	GLP-1 receptor agonists *vs* Tirzepatide	26.105	48	0.996
**All-cause death**	Between	Meglitinides *vs* Sulfonylureas	28.135	48	0.99
**All-cause death**	Between	Meglitinides *vs* Thiazolidinediones	28.167	48	0.99
**All-cause death**	Between	Metformin *vs* SGLT2 inhibitors	27.652	48	0.992
**All-cause death**	Between	Metformin *vs* Sulfonylureas	27.867	48	0.991
**All-cause death**	Between	Metformin *vs* Thiazolidinediones	28.073	48	0.99
**All-cause death**	Between	SGLT2 inhibitors *vs* Sulfonylureas	28.168	48	0.99

Outcome	Overall design	Specific design	Q	df	P-value
**All-cause death**	Between	Standard treatments *vs* Alpha-glucosidase inhibitors	28.031	48	0.991
**All-cause death**	Between	Standard treatments *vs* Basal insulin	27.607	48	0.992
**All-cause death**	Between	Standard treatments *vs* DPP-4 inhibitors	27.461	48	0.992
**All-cause death**	Between	Standard treatments *vs* GLP-1 receptor agonists	27.175	48	0.993
**All-cause death**	Between	Standard treatments *vs* Meglitinides	28.082	48	0.99
**All-cause death**	Between	Standard treatments *vs* Metformin	27.183	48	0.993
**All-cause death**	Between	Standard treatments *vs* SGLT2 inhibitors	28.133	48	0.99
**All-cause death**	Between	Standard treatments *vs* Thiazolidinediones	27.085	48	0.994
**All-cause death**	Between	Standard treatments *vs* Tirzepatide	26.522	48	0.995
**All-cause death**	Between	Sulfonylureas *vs* Thiazolidinediones	27.065	48	0.994
**All-cause death**	Between	Alpha-glucosidase inhibitors *vs* Meglitinides *vs* Sulfonylureas	27.235	47	0.991
**All-cause death**	Between	Basal insulin *vs* DPP-4 inhibitors *vs* GLP-1 receptor agonists *vs* Sulfonylureas	26.606	46	0.99
**All-cause death**	Between	DPP-4 inhibitors *vs* GLP-1 receptor agonists *vs* Metformin *vs* Thiazolidinediones	27.451	46	0.986
**All-cause death**	Between	DPP-4 inhibitors *vs* GLP-1 receptor agonists *vs* Thiazolidinediones	27.594	47	0.989
**All-cause death**	Between	Metformin *vs* Sulfonylureas *vs* Thiazolidinediones	26.245	47	0.994
**All-cause death**	Between	Standard treatments *vs* Alpha-glucosidase inhibitors *vs* Sulfonylureas	27.105	47	0.991
**All-cause death**	Between	Standard treatments *vs* DPP-4 inhibitors *vs* GLP-1 receptor agonists	28.032	47	0.987
**All-cause death**	Between	Standard treatments *vs* DPP-4 inhibitors *vs* GLP-1 receptor agonists *vs* Sulfonylureas	26.977	46	0.989
**All-cause death**	Between	Standard treatments *vs* DPP-4 inhibitors *vs* SGLT2 inhibitors	26.975	47	0.992
**All-cause death**	Between	Standard treatments *vs* GLP-1 receptor agonists *vs* Thiazolidinediones	26.623	47	0.993
**All-cause death**	Between	Standard treatments *vs* GLP-1 receptor agonists *vs* Tirzepatide	28.153	47	0.987
**All-cause death**	Between	Standard treatments *vs* Meglitinides *vs* Metformin	26.632	47	0.993
**All-cause death**	Between	Standard treatments *vs* Metformin *vs* Thiazolidinediones	27.146	47	0.991
**Cardiovascular death**	Total	Total	93.94	138	0.998
**Cardiovascular death**	Total	Within designs	77.268	111	0.994

Outcome	Overall design	Specific design	Q	df	P-value
**Cardiovascular death**	Total	Between designs	16.672	27	0.939
**Cardiovascular death**	Total	tau.within	0	NA	NA
**Cardiovascular death**	Within	Basal insulin *vs* Bolus insulin	0	0	NA
**Cardiovascular death**	Within	Basal insulin *vs* DPP-4 inhibitors	0	0	NA
**Cardiovascular death**	Within	Basal insulin *vs* GLP-1 receptor agonists	3.488	4	0.48
**Cardiovascular death**	Within	Basal insulin *vs* Tirzepatide	0	0	NA
**Cardiovascular death**	Within	Basal-Bolus insulin *vs* GLP-1 receptor agonists	0	0	NA
**Cardiovascular death**	Within	Basal-Bolus insulin *vs* Sulfonylureas	0	0	NA
**Cardiovascular death**	Within	Bolus insulin *vs* GLP-1 receptor agonists	0	0	NA
**Cardiovascular death**	Within	DPP-4 inhibitors *vs* GLP-1 receptor agonists	2.42	4	0.659
**Cardiovascular death**	Within	DPP-4 inhibitors *vs* Metformin	0	0	NA
**Cardiovascular death**	Within	DPP-4 inhibitors *vs* SGLT2 inhibitors	0.376	2	0.829
**Cardiovascular death**	Within	DPP-4 inhibitors *vs* Sulfonylureas	3.736	8	0.88
**Cardiovascular death**	Within	DPP-4 inhibitors *vs* Thiazolidinediones	0	0	NA
**Cardiovascular death**	Within	GLP-1 receptor agonists *vs* Tirzepatide	0	0	NA
**Cardiovascular death**	Within	Meglitinides *vs* Sulfonylureas	0	0	NA
**Cardiovascular death**	Within	Meglitinides *vs* Thiazolidinediones	0	0	NA
**Cardiovascular death**	Within	Metformin *vs* SGLT2 inhibitors	1.722	2	0.423
**Cardiovascular death**	Within	Metformin *vs* Sulfonylureas	0.914	1	0.339
**Cardiovascular death**	Within	Metformin *vs* Thiazolidinediones	0	1	0.994
**Cardiovascular death**	Within	SGLT2 inhibitors *vs* Sulfonylureas	0.033	1	0.857
**Cardiovascular death**	Within	Standard treatments *vs* Alpha-glucosidase inhibitors	0.372	1	0.542
**Cardiovascular death**	Within	Standard treatments *vs* DPP-4 inhibitors	16.493	27	0.943
**Cardiovascular death**	Within	Standard treatments *vs* GLP-1 receptor agonists	13.484	22	0.919
**Cardiovascular death**	Within	Standard treatments *vs* Metformin	0	0	NA

Outcome	Overall design	Specific design	Q	df	P-value
**Cardiovascular death**	Within	Standard treatments *vs* Non-steroidal MRAs	0.114	1	0.735
**Cardiovascular death**	Within	Standard treatments *vs* SGLT2 inhibitors	22.124	18	0.227
**Cardiovascular death**	Within	Standard treatments *vs* Thiazolidinediones	6.329	13	0.933
**Cardiovascular death**	Within	Sulfonylureas *vs* Thiazolidinediones	5.662	6	0.462
**Cardiovascular death**	Within	Basal insulin *vs* DPP-4 inhibitors *vs* GLP-1 receptor agonists *vs* Sulfonylureas	0	0	NA
**Cardiovascular death**	Within	DPP-4 inhibitors *vs* GLP-1 receptor agonists *vs* Thiazolidinediones	0	0	NA
**Cardiovascular death**	Within	Metformin *vs* Sulfonylureas *vs* Thiazolidinediones	0	0	NA
**Cardiovascular death**	Within	Standard treatments *vs* Alpha-glucosidase inhibitors *vs* Sulfonylureas	0	0	NA
**Cardiovascular death**	Within	Standard treatments *vs* DPP-4 inhibitors *vs* GLP-1 receptor agonists	0	0	NA
**Cardiovascular death**	Within	Standard treatments *vs* Meglitinides *vs* Metformin	0	0	NA
**Cardiovascular death**	Between	Basal insulin *vs* Bolus insulin	15.433	26	0.949
**Cardiovascular death**	Between	Basal insulin *vs* DPP-4 inhibitors	16.395	26	0.926
**Cardiovascular death**	Between	Basal insulin *vs* GLP-1 receptor agonists	16.658	26	0.919
**Cardiovascular death**	Between	Basal insulin *vs* Tirzepatide	15.952	26	0.937
**Cardiovascular death**	Between	Basal-Bolus insulin *vs* GLP-1 receptor agonists	16.46	26	0.924
**Cardiovascular death**	Between	Basal-Bolus insulin *vs* Sulfonylureas	16.46	26	0.924
**Cardiovascular death**	Between	Bolus insulin *vs* GLP-1 receptor agonists	15.433	26	0.949
**Cardiovascular death**	Between	DPP-4 inhibitors *vs* GLP-1 receptor agonists	16.583	26	0.921
**Cardiovascular death**	Between	DPP-4 inhibitors *vs* Metformin	16.249	26	0.93
**Cardiovascular death**	Between	DPP-4 inhibitors *vs* SGLT2 inhibitors	14.337	26	0.968
**Cardiovascular death**	Between	DPP-4 inhibitors *vs* Sulfonylureas	16.54	26	0.922
**Cardiovascular death**	Between	DPP-4 inhibitors *vs* Thiazolidinediones	14.954	26	0.958
**Cardiovascular death**	Between	GLP-1 receptor agonists *vs* Tirzepatide	15.952	26	0.937
**Cardiovascular death**	Between	Meglitinides *vs* Sulfonylureas	16.626	26	0.92
**Cardiovascular death**	Between	Meglitinides *vs* Thiazolidinediones	16.348	26	0.927

Outcome	Overall design	Specific design	Q	df	P-value
**Cardiovascular death**	Between	Metformin *vs* SGLT2 inhibitors	16.309	26	0.928
**Cardiovascular death**	Between	Metformin *vs* Sulfonylureas	15.626	26	0.945
**Cardiovascular death**	Between	Metformin *vs* Thiazolidinediones	15.665	26	0.944
**Cardiovascular death**	Between	SGLT2 inhibitors *vs* Sulfonylureas	16.577	26	0.921
**Cardiovascular death**	Between	Standard treatments *vs* Alpha-glucosidase inhibitors	16.409	26	0.926
**Cardiovascular death**	Between	Standard treatments *vs* DPP-4 inhibitors	16.183	26	0.932
**Cardiovascular death**	Between	Standard treatments *vs* GLP-1 receptor agonists	15.168	26	0.954
**Cardiovascular death**	Between	Standard treatments *vs* Metformin	15.586	26	0.946
**Cardiovascular death**	Between	Standard treatments *vs* SGLT2 inhibitors	16.05	26	0.935
**Cardiovascular death**	Between	Standard treatments *vs* Thiazolidinediones	16.436	26	0.925
**Cardiovascular death**	Between	Sulfonylureas *vs* Thiazolidinediones	15.953	26	0.937
**Cardiovascular death**	Between	Basal insulin *vs* DPP-4 inhibitors *vs* GLP-1 receptor agonists *vs* Sulfonylureas	13.424	24	0.959
**Cardiovascular death**	Between	DPP-4 inhibitors *vs* GLP-1 receptor agonists *vs* Thiazolidinediones	16.098	25	0.912
**Cardiovascular death**	Between	Metformin *vs* Sulfonylureas *vs* Thiazolidinediones	15.794	25	0.921
**Cardiovascular death**	Between	Standard treatments *vs* Alpha-glucosidase inhibitors *vs* Sulfonylureas	15.955	25	0.916
**Cardiovascular death**	Between	Standard treatments *vs* DPP-4 inhibitors *vs* GLP-1 receptor agonists	16.537	25	0.898
**Cardiovascular death**	Between	Standard treatments *vs* Meglitinides *vs* Metformin	16.051	25	0.913
**Non-fatal myocardial infarction**	Total	Total	137.255	210	1
**Non-fatal myocardial infarction**	Total	Within designs	112.599	169	1
**Non-fatal myocardial infarction**	Total	Between designs	24.656	41	0.98
**Non-fatal myocardial infarction**	Total	tau.within	0	NA	NA
**Non-fatal myocardial infarction**	Within	Basal insulin *vs* Basal-Bolus insulin	0	0	NA
**Non-fatal myocardial infarction**	Within	Basal insulin *vs* Bolus insulin	0	0	NA
**Non-fatal myocardial infarction**	Within	Basal insulin *vs* DPP-4 inhibitors	0	0	NA
**Non-fatal myocardial infarction**	Within	Basal insulin *vs* GLP-1 receptor agonists	3.913	6	0.688

Outcome	Overall design	Specific design	Q	df	P-value
**Non-fatal myocardial infarction**	Within	Basal insulin *vs* Thiazolidinediones	0	0	NA
**Non-fatal myocardial infarction**	Within	Basal-Bolus insulin *vs* GLP-1 receptor agonists	0	0	NA
**Non-fatal myocardial infarction**	Within	Bolus insulin *vs* GLP-1 receptor agonists	1.405	3	0.704
**Non-fatal myocardial infarction**	Within	DPP-4 inhibitors *vs* GLP-1 receptor agonists	0.666	4	0.955
**Non-fatal myocardial infarction**	Within	DPP-4 inhibitors *vs* Metformin	0.402	2	0.818
**Non-fatal myocardial infarction**	Within	DPP-4 inhibitors *vs* SGLT2 inhibitors	2.951	6	0.815
**Non-fatal myocardial infarction**	Within	DPP-4 inhibitors *vs* Sulfonylureas	6.197	10	0.798
**Non-fatal myocardial infarction**	Within	DPP-4 inhibitors *vs* Thiazolidinediones	2.145	2	0.342
**Non-fatal myocardial infarction**	Within	GLP-1 receptor agonists *vs* Metformin	0	0	NA
**Non-fatal myocardial infarction**	Within	GLP-1 receptor agonists *vs* SGLT2 inhibitors	0	0	NA
**Non-fatal myocardial infarction**	Within	GLP-1 receptor agonists *vs* Sulfonylureas	0.156	1	0.693
**Non-fatal myocardial infarction**	Within	GLP-1 receptor agonists *vs* Tirzepatide	0	0	NA
**Non-fatal myocardial infarction**	Within	Meglitinides *vs* Sulfonylureas	0	0	NA
**Non-fatal myocardial infarction**	Within	Metformin *vs* SGLT2 inhibitors	0	0	NA
**Non-fatal myocardial infarction**	Within	Metformin *vs* Sulfonylureas	0.59	1	0.443
**Non-fatal myocardial infarction**	Within	Metformin *vs* Thiazolidinediones	0.003	1	0.957
**Non-fatal myocardial infarction**	Within	SGLT2 inhibitors *vs* Sulfonylureas	4.343	3	0.227
**Non-fatal myocardial infarction**	Within	Standard treatments *vs* Alpha-glucosidase inhibitors	0.575	1	0.448
**Non-fatal myocardial infarction**	Within	Standard treatments *vs* Basal insulin	0	0	NA
**Non-fatal myocardial infarction**	Within	Standard treatments *vs* DPP-4 inhibitors	21.487	39	0.99
**Non-fatal myocardial infarction**	Within	Standard treatments *vs* GLP-1 receptor agonists	25.839	31	0.729
**Non-fatal myocardial infarction**	Within	Standard treatments *vs* Meglitinides	0	0	NA
**Non-fatal myocardial infarction**	Within	Standard treatments *vs* Metformin	3.771	3	0.287
**Non-fatal myocardial infarction**	Within	Standard treatments *vs* Non-steroidal MRAs	1.088	1	0.297
**Non-fatal myocardial infarction**	Within	Standard treatments *vs* SGLT2 inhibitors	19.532	25	0.771

Outcome	Overall design	Specific design	Q	df	P-value
**Non-fatal myocardial infarction**	Within	Standard treatments *vs* Sulfonylureas	0.067	1	0.796
**Non-fatal myocardial infarction**	Within	Standard treatments *vs* Thiazolidinediones	10.91	16	0.815
**Non-fatal myocardial infarction**	Within	Sulfonylureas *vs* Thiazolidinediones	4.428	9	0.881
**Non-fatal myocardial infarction**	Within	Alpha-glucosidase inhibitors *vs* Meglitinides *vs* Sulfonylureas	0	0	NA
**Non-fatal myocardial infarction**	Within	DPP-4 inhibitors *vs* GLP-1 receptor agonists *vs* Metformin *vs* Thiazolidinediones	0	0	NA
**Non-fatal myocardial infarction**	Within	DPP-4 inhibitors *vs* GLP-1 receptor agonists *vs* Thiazolidinediones	0	0	NA
**Non-fatal myocardial infarction**	Within	Metformin *vs* Sulfonylureas *vs* Thiazolidinediones	0.917	2	0.632
**Non-fatal myocardial infarction**	Within	Standard treatments *vs* DPP-4 inhibitors *vs* GLP-1 receptor agonists	1.214	2	0.545
**Non-fatal myocardial infarction**	Within	Standard treatments *vs* DPP-4 inhibitors *vs* GLP-1 receptor agonists *vs* Sulfonylureas	0	0	NA
**Non-fatal myocardial infarction**	Within	Standard treatments *vs* DPP-4 inhibitors *vs* Metformin	0	0	NA
**Non-fatal myocardial infarction**	Within	Standard treatments *vs* DPP-4 inhibitors *vs* SGLT2 inhibitors	0	0	NA
**Non-fatal myocardial infarction**	Within	Standard treatments *vs* GLP-1 receptor agonists *vs* Sulfonylureas	0	0	NA
**Non-fatal myocardial infarction**	Within	Standard treatments *vs* GLP-1 receptor agonists *vs* Tirzepatide	0	0	NA
**Non-fatal myocardial infarction**	Between	Basal insulin *vs* Basal-Bolus insulin	24.656	40	0.973
**Non-fatal myocardial infarction**	Between	Basal insulin *vs* Bolus insulin	24.631	40	0.973
**Non-fatal myocardial infarction**	Between	Basal insulin *vs* DPP-4 inhibitors	24.289	40	0.976
**Non-fatal myocardial infarction**	Between	Basal insulin *vs* GLP-1 receptor agonists	24.485	40	0.975
**Non-fatal myocardial infarction**	Between	Basal insulin *vs* Thiazolidinediones	24.156	40	0.977
**Non-fatal myocardial infarction**	Between	Basal-Bolus insulin *vs* GLP-1 receptor agonists	24.656	40	0.973
**Non-fatal myocardial infarction**	Between	Bolus insulin *vs* GLP-1 receptor agonists	24.631	40	0.973
**Non-fatal myocardial infarction**	Between	DPP-4 inhibitors *vs* GLP-1 receptor agonists	24.613	40	0.973
**Non-fatal myocardial infarction**	Between	DPP-4 inhibitors *vs* Metformin	21.158	40	0.994
**Non-fatal myocardial infarction**	Between	DPP-4 inhibitors *vs* SGLT2 inhibitors	24.553	40	0.974
**Non-fatal myocardial infarction**	Between	DPP-4 inhibitors *vs* Sulfonylureas	24.108	40	0.978
**Non-fatal myocardial infarction**	Between	DPP-4 inhibitors *vs* Thiazolidinediones	23.777	40	0.98

Outcome	Overall design	Specific design	Q	df	P-value
**Non-fatal myocardial infarction**	Between	GLP-1 receptor agonists *vs* Metformin	24.612	40	0.973
**Non-fatal myocardial infarction**	Between	GLP-1 receptor agonists *vs* SGLT2 inhibitors	23.575	40	0.982
**Non-fatal myocardial infarction**	Between	GLP-1 receptor agonists *vs* Sulfonylureas	24.561	40	0.974
**Non-fatal myocardial infarction**	Between	GLP-1 receptor agonists *vs* Tirzepatide	24.109	40	0.978
**Non-fatal myocardial infarction**	Between	Meglitinides *vs* Sulfonylureas	22.568	40	0.988
**Non-fatal myocardial infarction**	Between	Metformin *vs* SGLT2 inhibitors	24.596	40	0.973
**Non-fatal myocardial infarction**	Between	Metformin *vs* Sulfonylureas	24.637	40	0.973
**Non-fatal myocardial infarction**	Between	Metformin *vs* Thiazolidinediones	23.871	40	0.98
**Non-fatal myocardial infarction**	Between	SGLT2 inhibitors *vs* Sulfonylureas	24.214	40	0.977
**Non-fatal myocardial infarction**	Between	Standard treatments *vs* Alpha-glucosidase inhibitors	24.643	40	0.973
**Non-fatal myocardial infarction**	Between	Standard treatments *vs* Basal insulin	24.163	40	0.977
**Non-fatal myocardial infarction**	Between	Standard treatments *vs* DPP-4 inhibitors	23.875	40	0.98
**Non-fatal myocardial infarction**	Between	Standard treatments *vs* GLP-1 receptor agonists	24.314	40	0.976
**Non-fatal myocardial infarction**	Between	Standard treatments *vs* Meglitinides	23.19	40	0.985
**Non-fatal myocardial infarction**	Between	Standard treatments *vs* Metformin	23.92	40	0.979
**Non-fatal myocardial infarction**	Between	Standard treatments *vs* SGLT2 inhibitors	24.289	40	0.976
**Non-fatal myocardial infarction**	Between	Standard treatments *vs* Sulfonylureas	23.816	40	0.98
**Non-fatal myocardial infarction**	Between	Standard treatments *vs* Thiazolidinediones	24.132	40	0.978
**Non-fatal myocardial infarction**	Between	Sulfonylureas *vs* Thiazolidinediones	24.414	40	0.975
**Non-fatal myocardial infarction**	Between	Alpha-glucosidase inhibitors *vs* Meglitinides *vs* Sulfonylureas	24.568	39	0.965
**Non-fatal myocardial infarction**	Between	DPP-4 inhibitors *vs* GLP-1 receptor agonists *vs* Metformin *vs* Thiazolidinediones	23.285	38	0.971
**Non-fatal myocardial infarction**	Between	DPP-4 inhibitors *vs* GLP-1 receptor agonists *vs* Thiazolidinediones	22.818	39	0.982
**Non-fatal myocardial infarction**	Between	Metformin *vs* Sulfonylureas *vs* Thiazolidinediones	21.51	39	0.99
**Non-fatal myocardial infarction**	Between	Standard treatments *vs* DPP-4 inhibitors *vs* GLP-1 receptor agonists	23.313	39	0.978
**Non-fatal myocardial infarction**	Between	Standard treatments *vs* DPP-4 inhibitors *vs* GLP-1 receptor agonists *vs* Sulfonylureas	21.832	38	0.983

Outcome	Overall design	Specific design	Q	df	P-value
**Non-fatal myocardial infarction**	Between	Standard treatments *vs* DPP-4 inhibitors *vs* Metformin	24.316	39	0.968
**Non-fatal myocardial infarction**	Between	Standard treatments *vs* DPP-4 inhibitors *vs* SGLT2 inhibitors	24.112	39	0.97
**Non-fatal myocardial infarction**	Between	Standard treatments *vs* GLP-1 receptor agonists *vs* Sulfonylureas	24.591	39	0.965
**Non-fatal myocardial infarction**	Between	Standard treatments *vs* GLP-1 receptor agonists *vs* Tirzepatide	23.294	39	0.978
**Non-fatal stroke**	Total	Total	103.694	180	1
**Non-fatal stroke**	Total	Within designs	86.021	142	1
**Non-fatal stroke**	Total	Between designs	17.673	38	0.998
**Non-fatal stroke**	Total	tau.within	0	NA	NA
**Non-fatal stroke**	Within	Basal insulin *vs* Basal-Bolus insulin	0	0	NA
**Non-fatal stroke**	Within	Basal insulin *vs* DPP-4 inhibitors	0	0	NA
**Non-fatal stroke**	Within	Basal insulin *vs* GLP-1 receptor agonists	5.464	10	0.858
**Non-fatal stroke**	Within	Basal insulin *vs* Thiazolidinediones	0	0	NA
**Non-fatal stroke**	Within	Bolus insulin *vs* GLP-1 receptor agonists	0.914	2	0.633
**Non-fatal stroke**	Within	DPP-4 inhibitors *vs* GLP-1 receptor agonists	3.198	2	0.202
**Non-fatal stroke**	Within	DPP-4 inhibitors *vs* Metformin	0.821	2	0.663
**Non-fatal stroke**	Within	DPP-4 inhibitors *vs* SGLT2 inhibitors	1.774	4	0.777
**Non-fatal stroke**	Within	DPP-4 inhibitors *vs* Sulfonylureas	8.618	11	0.657
**Non-fatal stroke**	Within	DPP-4 inhibitors *vs* Thiazolidinediones	1.266	1	0.26
**Non-fatal stroke**	Within	GLP-1 receptor agonists *vs* Metformin	0	0	NA
**Non-fatal stroke**	Within	GLP-1 receptor agonists *vs* SGLT2 inhibitors	0.021	1	0.884
**Non-fatal stroke**	Within	GLP-1 receptor agonists *vs* Sulfonylureas	3.221	1	0.073
**Non-fatal stroke**	Within	Meglitinides *vs* Sulfonylureas	0	0	NA
**Non-fatal stroke**	Within	Metformin *vs* Sulfonylureas	0	0	NA
**Non-fatal stroke**	Within	Metformin *vs* Thiazolidinediones	0	0	NA
**Non-fatal stroke**	Within	SGLT2 inhibitors *vs* Sulfonylureas	2.078	5	0.838

Outcome	Overall design	Specific design	Q	df	P-value
**Non-fatal stroke**	Within	Standard treatments *vs* DPP-4 inhibitors	20.509	34	0.967
**Non-fatal stroke**	Within	Standard treatments *vs* GLP-1 receptor agonists	18.838	25	0.805
**Non-fatal stroke**	Within	Standard treatments *vs* Meglitinides	0	0	NA
**Non-fatal stroke**	Within	Standard treatments *vs* Metformin	0.001	1	0.971
**Non-fatal stroke**	Within	Standard treatments *vs* Non-steroidal MRAs	0.122	1	0.727
**Non-fatal stroke**	Within	Standard treatments *vs* SGLT2 inhibitors	12.329	25	0.984
**Non-fatal stroke**	Within	Standard treatments *vs* Sulfonylureas	0	0	NA
**Non-fatal stroke**	Within	Standard treatments *vs* Thiazolidinediones	2.307	9	0.986
**Non-fatal stroke**	Within	Sulfonylureas *vs* Thiazolidinediones	4.524	6	0.606
**Non-fatal stroke**	Within	Alpha-glucosidase inhibitors *vs* Meglitinides *vs* Sulfonylureas	0	0	NA
**Non-fatal stroke**	Within	Basal-Bolus insulin *vs* GLP-1 receptor agonists *vs* Thiazolidinediones	0	0	NA
**Non-fatal stroke**	Within	DPP-4 inhibitors *vs* GLP-1 receptor agonists *vs* Metformin *vs* Thiazolidinediones	0	0	NA
**Non-fatal stroke**	Within	DPP-4 inhibitors *vs* GLP-1 receptor agonists *vs* Thiazolidinediones	0	0	NA
**Non-fatal stroke**	Within	Metformin *vs* Sulfonylureas *vs* Thiazolidinediones	0	0	NA
**Non-fatal stroke**	Within	Standard treatments *vs* DPP-4 inhibitors *vs* GLP-1 receptor agonists	0.014	2	0.993
**Non-fatal stroke**	Within	Standard treatments *vs* DPP-4 inhibitors *vs* GLP-1 receptor agonists *vs* Sulfonylureas	0	0	NA
**Non-fatal stroke**	Within	Standard treatments *vs* DPP-4 inhibitors *vs* Metformin	0	0	NA
**Non-fatal stroke**	Within	Standard treatments *vs* DPP-4 inhibitors *vs* SGLT2 inhibitors	0	0	NA
**Non-fatal stroke**	Within	Standard treatments *vs* GLP-1 receptor agonists *vs* Sulfonylureas	0	0	NA
**Non-fatal stroke**	Within	Standard treatments *vs* Sulfonylureas *vs* Thiazolidinediones	0	0	NA
**Non-fatal stroke**	Between	Basal insulin *vs* Basal-Bolus insulin	17.455	37	0.997
**Non-fatal stroke**	Between	Basal insulin *vs* DPP-4 inhibitors	16.899	37	0.998
**Non-fatal stroke**	Between	Basal insulin *vs* GLP-1 receptor agonists	17.482	37	0.997
**Non-fatal stroke**	Between	Basal insulin *vs* Thiazolidinediones	17.254	37	0.998
**Non-fatal stroke**	Between	DPP-4 inhibitors *vs* GLP-1 receptor agonists	17.655	37	0.997

Outcome	Overall design	Specific design	Q	df	P-value
**Non-fatal stroke**	Between	DPP-4 inhibitors *vs* Metformin	17.136	37	0.998
**Non-fatal stroke**	Between	DPP-4 inhibitors *vs* SGLT2 inhibitors	17.615	37	0.997
**Non-fatal stroke**	Between	DPP-4 inhibitors *vs* Sulfonylureas	16.619	37	0.998
**Non-fatal stroke**	Between	DPP-4 inhibitors *vs* Thiazolidinediones	17.023	37	0.998
**Non-fatal stroke**	Between	GLP-1 receptor agonists *vs* Metformin	17.566	37	0.997
**Non-fatal stroke**	Between	GLP-1 receptor agonists *vs* SGLT2 inhibitors	15.176	37	0.999
**Non-fatal stroke**	Between	GLP-1 receptor agonists *vs* Sulfonylureas	17.655	37	0.997
**Non-fatal stroke**	Between	Meglitinides *vs* Sulfonylureas	16.58	37	0.998
**Non-fatal stroke**	Between	Metformin *vs* Sulfonylureas	16.512	37	0.999
**Non-fatal stroke**	Between	Metformin *vs* Thiazolidinediones	17.296	37	0.998
**Non-fatal stroke**	Between	SGLT2 inhibitors *vs* Sulfonylureas	15.327	37	0.999
**Non-fatal stroke**	Between	Standard treatments *vs* DPP-4 inhibitors	15.909	37	0.999
**Non-fatal stroke**	Between	Standard treatments *vs* GLP-1 receptor agonists	17.469	37	0.997
**Non-fatal stroke**	Between	Standard treatments *vs* Meglitinides	16.231	37	0.999
**Non-fatal stroke**	Between	Standard treatments *vs* Metformin	17.672	37	0.997
**Non-fatal stroke**	Between	Standard treatments *vs* SGLT2 inhibitors	17.608	37	0.997
**Non-fatal stroke**	Between	Standard treatments *vs* Sulfonylureas	17.342	37	0.998
**Non-fatal stroke**	Between	Standard treatments *vs* Thiazolidinediones	17.012	37	0.998
**Non-fatal stroke**	Between	Sulfonylureas *vs* Thiazolidinediones	17.264	37	0.998
**Non-fatal stroke**	Between	Basal-Bolus insulin *vs* GLP-1 receptor agonists *vs* Thiazolidinediones	17.454	36	0.996
**Non-fatal stroke**	Between	DPP-4 inhibitors *vs* GLP-1 receptor agonists *vs* Metformin *vs* Thiazolidinediones	17.225	35	0.995
**Non-fatal stroke**	Between	DPP-4 inhibitors *vs* GLP-1 receptor agonists *vs* Thiazolidinediones	17.205	36	0.997
**Non-fatal stroke**	Between	Metformin *vs* Sulfonylureas *vs* Thiazolidinediones	17.251	36	0.997
**Non-fatal stroke**	Between	Standard treatments *vs* DPP-4 inhibitors *vs* GLP-1 receptor agonists	17.362	36	0.996
**Non-fatal stroke**	Between	Standard treatments *vs* DPP-4 inhibitors *vs* GLP-1 receptor agonists *vs* Sulfonylureas	15.655	35	0.998

Outcome	Overall design	Specific design	Q	df	P-value
**Non-fatal stroke**	Between	Standard treatments *vs* DPP-4 inhibitors *vs* Metformin	16.264	36	0.998
**Non-fatal stroke**	Between	Standard treatments *vs* DPP-4 inhibitors *vs* SGLT2 inhibitors	17.315	36	0.996
**Non-fatal stroke**	Between	Standard treatments *vs* GLP-1 receptor agonists *vs* Sulfonylureas	17.523	36	0.996
**Non-fatal stroke**	Between	Standard treatments *vs* Sulfonylureas *vs* Thiazolidinediones	17.145	36	0.997
**Hospitalisation for heart failure**	Total	Total	85.125	139	1
**Hospitalisation for heart failure**	Total	Within designs	73.851	119	1
**Hospitalisation for heart failure**	Total	Between designs	11.274	20	0.939
**Hospitalisation for heart failure**	Total	tau.within	0	NA	NA
**Hospitalisation for heart failure**	Within	Basal insulin *vs* GLP-1 receptor agonists	1.74	3	0.628
**Hospitalisation for heart failure**	Within	Basal insulin *vs* Thiazolidinediones	0	0	NA
**Hospitalisation for heart failure**	Within	Basal insulin *vs* Tirzepatide	0	0	NA
**Hospitalisation for heart failure**	Within	Bolus insulin *vs* GLP-1 receptor agonists	0.422	1	0.516
**Hospitalisation for heart failure**	Within	DPP-4 inhibitors *vs* GLP-1 receptor agonists	1.472	4	0.832
**Hospitalisation for heart failure**	Within	DPP-4 inhibitors *vs* SGLT2 inhibitors	0	0	NA
**Hospitalisation for heart failure**	Within	DPP-4 inhibitors *vs* Sulfonylureas	8.493	10	0.581
**Hospitalisation for heart failure**	Within	GLP-1 receptor agonists *vs* Metformin	0	0	NA
**Hospitalisation for heart failure**	Within	GLP-1 receptor agonists *vs* SGLT2 inhibitors	0	0	NA
**Hospitalisation for heart failure**	Within	GLP-1 receptor agonists *vs* Sulfonylureas	0	0	NA
**Hospitalisation for heart failure**	Within	Metformin *vs* SGLT2 inhibitors	0	0	NA
**Hospitalisation for heart failure**	Within	SGLT2 inhibitors *vs* Sulfonylureas	1.95	4	0.745
**Hospitalisation for heart failure**	Within	Standard treatments *vs* Alpha-glucosidase inhibitors	0	0	NA
**Hospitalisation for heart failure**	Within	Standard treatments *vs* Basal insulin	0	1	0.996
**Hospitalisation for heart failure**	Within	Standard treatments *vs* DPP-4 inhibitors	20.661	23	0.602
**Hospitalisation for heart failure**	Within	Standard treatments *vs* GLP-1 receptor agonists	11.59	19	0.902
**Hospitalisation for heart failure**	Within	Standard treatments *vs* Non-steroidal MRAs	1.25	1	0.264

Outcome	Overall design	Specific design	Q	df	P-value
**Hospitalisation for heart failure**	Within	Standard treatments *vs* SGLT2 inhibitors	12.758	25	0.979
**Hospitalisation for heart failure**	Within	Standard treatments *vs* Thiazolidinediones	9.692	19	0.96
**Hospitalisation for heart failure**	Within	Sulfonylureas *vs* Thiazolidinediones	3.824	9	0.923
**Hospitalisation for heart failure**	Within	Basal insulin *vs* DPP-4 inhibitors *vs* GLP-1 receptor agonists *vs* Sulfonylureas	0	0	NA
**Hospitalisation for heart failure**	Within	DPP-4 inhibitors *vs* GLP-1 receptor agonists *vs* Metformin *vs* Thiazolidinediones	0	0	NA
**Hospitalisation for heart failure**	Within	Standard treatments *vs* DPP-4 inhibitors *vs* GLP-1 receptor agonists *vs* Sulfonylureas	0	0	NA
**Hospitalisation for heart failure**	Within	Standard treatments *vs* GLP-1 receptor agonists *vs* Sulfonylureas	0	0	NA
**Hospitalisation for heart failure**	Between	Basal insulin *vs* GLP-1 receptor agonists	11.267	19	0.915
**Hospitalisation for heart failure**	Between	Basal insulin *vs* Thiazolidinediones	11.153	19	0.919
**Hospitalisation for heart failure**	Between	DPP-4 inhibitors *vs* GLP-1 receptor agonists	11.151	19	0.919
**Hospitalisation for heart failure**	Between	DPP-4 inhibitors *vs* SGLT2 inhibitors	10.559	19	0.938
**Hospitalisation for heart failure**	Between	DPP-4 inhibitors *vs* Sulfonylureas	10.23	19	0.947
**Hospitalisation for heart failure**	Between	GLP-1 receptor agonists *vs* Metformin	10.215	19	0.947
**Hospitalisation for heart failure**	Between	GLP-1 receptor agonists *vs* SGLT2 inhibitors	10.595	19	0.937
**Hospitalisation for heart failure**	Between	GLP-1 receptor agonists *vs* Sulfonylureas	10.754	19	0.932
**Hospitalisation for heart failure**	Between	Metformin *vs* SGLT2 inhibitors	11.183	19	0.918
**Hospitalisation for heart failure**	Between	SGLT2 inhibitors *vs* Sulfonylureas	11.154	19	0.919
**Hospitalisation for heart failure**	Between	Standard treatments *vs* Basal insulin	10.419	19	0.942
**Hospitalisation for heart failure**	Between	Standard treatments *vs* DPP-4 inhibitors	9.757	19	0.959
**Hospitalisation for heart failure**	Between	Standard treatments *vs* GLP-1 receptor agonists	7.504	19	0.991
**Hospitalisation for heart failure**	Between	Standard treatments *vs* SGLT2 inhibitors	10.273	19	0.946
**Hospitalisation for heart failure**	Between	Standard treatments *vs* Thiazolidinediones	11.246	19	0.915
**Hospitalisation for heart failure**	Between	Sulfonylureas *vs* Thiazolidinediones	11.013	19	0.923
**Hospitalisation for heart failure**	Between	Basal insulin *vs* DPP-4 inhibitors *vs* GLP-1 receptor agonists *vs* Sulfonylureas	5.496	17	0.996
**Hospitalisation for heart failure**	Between	DPP-4 inhibitors *vs* GLP-1 receptor agonists *vs* Metformin *vs* Thiazolidinediones	10.105	17	0.899

Outcome	Overall design	Specific design	Q	df	P-value
**Hospitalisation for heart failure**	Between	Standard treatments *vs* DPP-4 inhibitors *vs* GLP-1 receptor agonists *vs* Sulfonylureas	10.682	17	0.873
**Hospitalisation for heart failure**	Between	Standard treatments *vs* GLP-1 receptor agonists *vs* Sulfonylureas	11.125	18	0.889
**End-stage kidney disease**	Total	Total	38.746	44	0.696
**End-stage kidney disease**	Total	Within designs	38.103	40	0.556
**End-stage kidney disease**	Total	Between designs	0.644	4	0.958
**End-stage kidney disease**	Total	tau.within	0	NA	NA
**End-stage kidney disease**	Within	Basal insulin *vs* GLP-1 receptor agonists	0.224	1	0.636
**End-stage kidney disease**	Within	Basal insulin *vs* Tirzepatide	0	0	NA
**End-stage kidney disease**	Within	Bolus insulin *vs* GLP-1 receptor agonists	0	0	NA
**End-stage kidney disease**	Within	DPP-4 inhibitors *vs* GLP-1 receptor agonists	0.433	2	0.805
**End-stage kidney disease**	Within	DPP-4 inhibitors *vs* Sulfonylureas	0.903	2	0.637
**End-stage kidney disease**	Within	GLP-1 receptor agonists *vs* Tirzepatide	0	0	NA
**End-stage kidney disease**	Within	SGLT2 inhibitors *vs* Sulfonylureas	0	0	NA
**End-stage kidney disease**	Within	Standard treatments *vs* DPP-4 inhibitors	4.132	8	0.845
**End-stage kidney disease**	Within	Standard treatments *vs* GLP-1 receptor agonists	6.597	10	0.763
**End-stage kidney disease**	Within	Standard treatments *vs* Metformin	0	0	NA
**End-stage kidney disease**	Within	Standard treatments *vs* Non-steroidal MRAs	0.496	1	0.481
**End-stage kidney disease**	Within	Standard treatments *vs* SGLT2 inhibitors	24.407	14	0.041
**End-stage kidney disease**	Within	Standard treatments *vs* Thiazolidinediones	0	0	NA
**End-stage kidney disease**	Within	Sulfonylureas *vs* Thiazolidinediones	0.91	2	0.634
**End-stage kidney disease**	Between	Basal insulin *vs* GLP-1 receptor agonists	0.617	3	0.892
**End-stage kidney disease**	Between	Basal insulin *vs* Tirzepatide	0.617	3	0.892
**End-stage kidney disease**	Between	DPP-4 inhibitors *vs* GLP-1 receptor agonists	0.623	3	0.891
**End-stage kidney disease**	Between	DPP-4 inhibitors *vs* Sulfonylureas	0.582	3	0.9
**End-stage kidney disease**	Between	GLP-1 receptor agonists *vs* Tirzepatide	0.617	3	0.892

Outcome	Overall design	Specific design	Q	df	P-value
**End-stage kidney disease**	Between	SGLT2 inhibitors *vs* Sulfonylureas	0.05	3	0.997
**End-stage kidney disease**	Between	Standard treatments *vs* DPP-4 inhibitors	0.642	3	0.887
**End-stage kidney disease**	Between	Standard treatments *vs* GLP-1 receptor agonists	0.623	3	0.891
**End-stage kidney disease**	Between	Standard treatments *vs* SGLT2 inhibitors	0.05	3	0.997
**End-stage kidney disease**	Between	Standard treatments *vs* Thiazolidinediones	0.629	3	0.89
**End-stage kidney disease**	Between	Sulfonylureas *vs* Thiazolidinediones	0.629	3	0.89
**Severe hypoglycaemia**	Total	Total	195.973	197	0.507
**Severe hypoglycaemia**	Total	Within designs	161.108	165	0.571
**Severe hypoglycaemia**	Total	Between designs	34.866	32	0.333
**Severe hypoglycaemia**	Total	tau.within	0	NA	NA
**Severe hypoglycaemia**	Within	Alpha-glucosidase inhibitors *vs* SGLT2 inhibitors	0	0	NA
**Severe hypoglycaemia**	Within	Basal insulin *vs* Basal-Bolus insulin	0	0	NA
**Severe hypoglycaemia**	Within	Basal insulin *vs* DPP-4 inhibitors	0.074	1	0.785
**Severe hypoglycaemia**	Within	Basal insulin *vs* GLP-1 receptor agonists	28.584	12	0.005
**Severe hypoglycaemia**	Within	Basal insulin *vs* SGLT2 inhibitors	0	0	NA
**Severe hypoglycaemia**	Within	Basal insulin *vs* Thiazolidinediones	4.287	1	0.038
**Severe hypoglycaemia**	Within	Basal insulin *vs* Tirzepatide	0.344	1	0.557
**Severe hypoglycaemia**	Within	Basal-Bolus insulin *vs* GLP-1 receptor agonists	0	0	NA
**Severe hypoglycaemia**	Within	Bolus insulin *vs* GLP-1 receptor agonists	0.554	6	0.997
**Severe hypoglycaemia**	Within	DPP-4 inhibitors *vs* GLP-1 receptor agonists	3.025	5	0.696
**Severe hypoglycaemia**	Within	DPP-4 inhibitors *vs* Metformin	2.086	1	0.149
**Severe hypoglycaemia**	Within	DPP-4 inhibitors *vs* SGLT2 inhibitors	0.84	2	0.657
**Severe hypoglycaemia**	Within	DPP-4 inhibitors *vs* Sulfonylureas	6.687	14	0.946
**Severe hypoglycaemia**	Within	DPP-4 inhibitors *vs* Thiazolidinediones	2.132	2	0.344
**Severe hypoglycaemia**	Within	GLP-1 receptor agonists *vs* SGLT2 inhibitors	0.004	1	0.949

Outcome	Overall design	Specific design	Q	df	P-value
**Severe hypoglycaemia**	Within	GLP-1 receptor agonists *vs* Sulfonylureas	0	0	NA
**Severe hypoglycaemia**	Within	GLP-1 receptor agonists *vs* Tirzepatide	0	0	NA
**Severe hypoglycaemia**	Within	Meglitinides *vs* Metformin	0	0	NA
**Severe hypoglycaemia**	Within	Meglitinides *vs* Sulfonylureas	0	0	NA
**Severe hypoglycaemia**	Within	Metformin *vs* SGLT2 inhibitors	0.117	1	0.732
**Severe hypoglycaemia**	Within	Metformin *vs* Sulfonylureas	0	0	NA
**Severe hypoglycaemia**	Within	SGLT2 inhibitors *vs* Sulfonylureas	0.201	2	0.905
**Severe hypoglycaemia**	Within	Standard treatments *vs* Alpha-glucosidase inhibitors	1.14	1	0.286
**Severe hypoglycaemia**	Within	Standard treatments *vs* DPP-4 inhibitors	33.067	40	0.773
**Severe hypoglycaemia**	Within	Standard treatments *vs* GLP-1 receptor agonists	45.291	33	0.075
**Severe hypoglycaemia**	Within	Standard treatments *vs* Metformin	4.044	1	0.044
**Severe hypoglycaemia**	Within	Standard treatments *vs* Non-steroidal MRAs	0.078	1	0.78
**Severe hypoglycaemia**	Within	Standard treatments *vs* SGLT2 inhibitors	22.858	27	0.693
**Severe hypoglycaemia**	Within	Standard treatments *vs* Sulfonylureas	0	0	NA
**Severe hypoglycaemia**	Within	Standard treatments *vs* Thiazolidinediones	3.281	7	0.858
**Severe hypoglycaemia**	Within	Standard treatments *vs* Tirzepatide	0	0	NA
**Severe hypoglycaemia**	Within	Sulfonylureas *vs* Thiazolidinediones	1.893	4	0.755
**Severe hypoglycaemia**	Within	Alpha-glucosidase inhibitors *vs* Meglitinides *vs* Sulfonylureas	0	0	NA
**Severe hypoglycaemia**	Within	Basal insulin *vs* DPP-4 inhibitors *vs* GLP-1 receptor agonists *vs* Sulfonylureas	0	0	NA
**Severe hypoglycaemia**	Within	Metformin *vs* Sulfonylureas *vs* Thiazolidinediones	0	0	NA
**Severe hypoglycaemia**	Within	Standard treatments *vs* Basal insulin *vs* GLP-1 receptor agonists	0	0	NA
**Severe hypoglycaemia**	Within	Standard treatments *vs* GLP-1 receptor agonists *vs* Sulfonylureas	0	0	NA
**Severe hypoglycaemia**	Within	Standard treatments *vs* GLP-1 receptor agonists *vs* Thiazolidinediones	0.519	2	0.771
**Severe hypoglycaemia**	Between	Alpha-glucosidase inhibitors *vs* SGLT2 inhibitors	34.602	31	0.3
**Severe hypoglycaemia**	Between	Basal insulin *vs* Basal-Bolus insulin	34.467	31	0.305

Outcome	Overall design	Specific design	Q	df	P-value
**Severe hypoglycaemia**	Between	Basal insulin *vs* DPP-4 inhibitors	34.504	31	0.304
**Severe hypoglycaemia**	Between	Basal insulin *vs* GLP-1 receptor agonists	34.612	31	0.299
**Severe hypoglycaemia**	Between	Basal insulin *vs* SGLT2 inhibitors	34.848	31	0.29
**Severe hypoglycaemia**	Between	Basal insulin *vs* Thiazolidinediones	34.508	31	0.304
**Severe hypoglycaemia**	Between	Basal insulin *vs* Tirzepatide	34.518	31	0.303
**Severe hypoglycaemia**	Between	Basal-Bolus insulin *vs* GLP-1 receptor agonists	34.467	31	0.305
**Severe hypoglycaemia**	Between	DPP-4 inhibitors *vs* GLP-1 receptor agonists	32.377	31	0.399
**Severe hypoglycaemia**	Between	DPP-4 inhibitors *vs* Metformin	34.667	31	0.297
**Severe hypoglycaemia**	Between	DPP-4 inhibitors *vs* SGLT2 inhibitors	32.764	31	0.38
**Severe hypoglycaemia**	Between	DPP-4 inhibitors *vs* Sulfonylureas	31.843	31	0.424
**Severe hypoglycaemia**	Between	DPP-4 inhibitors *vs* Thiazolidinediones	32.09	31	0.412
**Severe hypoglycaemia**	Between	GLP-1 receptor agonists *vs* SGLT2 inhibitors	34.826	31	0.291
**Severe hypoglycaemia**	Between	GLP-1 receptor agonists *vs* Sulfonylureas	31.965	31	0.418
**Severe hypoglycaemia**	Between	GLP-1 receptor agonists *vs* Tirzepatide	34.802	31	0.292
**Severe hypoglycaemia**	Between	Meglitinides *vs* Metformin	34.306	31	0.312
**Severe hypoglycaemia**	Between	Meglitinides *vs* Sulfonylureas	34.177	31	0.318
**Severe hypoglycaemia**	Between	Metformin *vs* SGLT2 inhibitors	33.902	31	0.329
**Severe hypoglycaemia**	Between	Metformin *vs* Sulfonylureas	33.067	31	0.366
**Severe hypoglycaemia**	Between	SGLT2 inhibitors *vs* Sulfonylureas	34.865	31	0.289
**Severe hypoglycaemia**	Between	Standard treatments *vs* Alpha-glucosidase inhibitors	33.877	31	0.33
**Severe hypoglycaemia**	Between	Standard treatments *vs* DPP-4 inhibitors	34.779	31	0.293
**Severe hypoglycaemia**	Between	Standard treatments *vs* GLP-1 receptor agonists	34.865	31	0.289
**Severe hypoglycaemia**	Between	Standard treatments *vs* Metformin	34.843	31	0.29
**Severe hypoglycaemia**	Between	Standard treatments *vs* SGLT2 inhibitors	34.798	31	0.292
**Severe hypoglycaemia**	Between	Standard treatments *vs* Sulfonylureas	34.729	31	0.295

Outcome	Overall design	Specific design	Q	df	P-value
**Severe hypoglycaemia**	Between	Standard treatments *vs* Thiazolidinediones	32.57	31	0.39
**Severe hypoglycaemia**	Between	Standard treatments *vs* Tirzepatide	34.587	31	0.3
**Severe hypoglycaemia**	Between	Sulfonylureas *vs* Thiazolidinediones	32.442	31	0.396
**Severe hypoglycaemia**	Between	Alpha-glucosidase inhibitors *vs* Meglitinides *vs* Sulfonylureas	34.379	30	0.266
**Severe hypoglycaemia**	Between	Basal insulin *vs* DPP-4 inhibitors *vs* GLP-1 receptor agonists *vs* Sulfonylureas	27.584	29	0.54
**Severe hypoglycaemia**	Between	Metformin *vs* Sulfonylureas *vs* Thiazolidinediones	33.434	30	0.304
**Severe hypoglycaemia**	Between	Standard treatments *vs* Basal insulin *vs* GLP-1 receptor agonists	28.807	30	0.528
**Severe hypoglycaemia**	Between	Standard treatments *vs* GLP-1 receptor agonists *vs* Sulfonylureas	33.324	30	0.309
**Severe hypoglycaemia**	Between	Standard treatments *vs* GLP-1 receptor agonists *vs* Thiazolidinediones	34.71	30	0.253
**Severe gastrointestinal events**	Total	Total	80.718	30	0
**Severe gastrointestinal events**	Total	Within designs	70.058	21	0
**Severe gastrointestinal events**	Total	Between designs	10.66	9	0.3
**Severe gastrointestinal events**	Total	tau.within	0.505	NA	NA
**Severe gastrointestinal events**	Within	Alpha-glucosidase inhibitors *vs* Metformin	0	0	NA
**Severe gastrointestinal events**	Within	Basal insulin *vs* DPP-4 inhibitors	0	0	NA
**Severe gastrointestinal events**	Within	Basal insulin *vs* GLP-1 receptor agonists	0	0	NA
**Severe gastrointestinal events**	Within	Basal insulin *vs* Tirzepatide	0.848	1	0.357
**Severe gastrointestinal events**	Within	Bolus insulin *vs* GLP-1 receptor agonists	0	0	NA
**Severe gastrointestinal events**	Within	DPP-4 inhibitors *vs* GLP-1 receptor agonists	2.758	1	0.097
**Severe gastrointestinal events**	Within	DPP-4 inhibitors *vs* Sulfonylureas	0	0	NA
**Severe gastrointestinal events**	Within	GLP-1 receptor agonists *vs* Tirzepatide	0	0	NA
**Severe gastrointestinal events**	Within	Metformin *vs* Thiazolidinediones	0	0	NA
**Severe gastrointestinal events**	Within	Standard treatments *vs* DPP-4 inhibitors	2.002	4	0.735
**Severe gastrointestinal events**	Within	Standard treatments *vs* GLP-1 receptor agonists	63.51	13	0
**Severe gastrointestinal events**	Within	Standard treatments *vs* Metformin	0	0	NA

Outcome	Overall design	Specific design	Q	df	P-value
**Severe gastrointestinal events**	Within	Standard treatments *vs* Thiazolidinediones	0.674	1	0.412
**Severe gastrointestinal events**	Within	Standard treatments *vs* Tirzepatide	0.266	1	0.606
**Severe gastrointestinal events**	Within	Metformin *vs* Sulfonylureas *vs* Thiazolidinediones	0	0	NA
**Severe gastrointestinal events**	Within	Standard treatments *vs* GLP-1 receptor agonists *vs* Tirzepatide	0	0	NA
**Severe gastrointestinal events**	Between	Basal insulin *vs* DPP-4 inhibitors	9.644	8	0.291
**Severe gastrointestinal events**	Between	Basal insulin *vs* GLP-1 receptor agonists	9.879	8	0.274
**Severe gastrointestinal events**	Between	Basal insulin *vs* Tirzepatide	8.021	8	0.431
**Severe gastrointestinal events**	Between	DPP-4 inhibitors *vs* GLP-1 receptor agonists	10.622	8	0.224
**Severe gastrointestinal events**	Between	DPP-4 inhibitors *vs* Sulfonylureas	9.949	8	0.269
**Severe gastrointestinal events**	Between	GLP-1 receptor agonists *vs* Tirzepatide	6.401	8	0.602
**Severe gastrointestinal events**	Between	Metformin *vs* Thiazolidinediones	7.871	8	0.446
**Severe gastrointestinal events**	Between	Standard treatments *vs* DPP-4 inhibitors	8.987	8	0.343
**Severe gastrointestinal events**	Between	Standard treatments *vs* GLP-1 receptor agonists	9.17	8	0.328
**Severe gastrointestinal events**	Between	Standard treatments *vs* Metformin	10.62	8	0.224
**Severe gastrointestinal events**	Between	Standard treatments *vs* Thiazolidinediones	9.994	8	0.265
**Severe gastrointestinal events**	Between	Standard treatments *vs* Tirzepatide	8.729	8	0.366
**Severe gastrointestinal events**	Between	Metformin *vs* Sulfonylureas *vs* Thiazolidinediones	7.925	7	0.339
**Severe gastrointestinal events**	Between	Standard treatments *vs* GLP-1 receptor agonists *vs* Tirzepatide	10.51	7	0.161
**Genital infection**	Total	Total	80.123	89	0.738
**Genital infection**	Total	Within designs	75.537	85	0.759
**Genital infection**	Total	Between designs	4.586	4	0.332
**Genital infection**	Total	tau.within	0	NA	NA
**Genital infection**	Within	DPP-4 inhibitors *vs* SGLT2 inhibitors	5.374	8	0.717
**Genital infection**	Within	GLP-1 receptor agonists *vs* SGLT2 inhibitors	1.124	1	0.289
**Genital infection**	Within	Metformin *vs* SGLT2 inhibitors	4.091	3	0.252

Outcome	Overall design	Specific design	Q	df	P-value
**Genital infection**	Within	SGLT2 inhibitors *vs* Sulfonylureas	2.219	5	0.818
**Genital infection**	Within	SGLT2 inhibitors *vs* Thiazolidinediones	0	0	NA
**Genital infection**	Within	Standard treatments *vs* DPP-4 inhibitors	1.52	4	0.823
**Genital infection**	Within	Standard treatments *vs* GLP-1 receptor agonists	3.579	3	0.311
**Genital infection**	Within	Standard treatments *vs* SGLT2 inhibitors	57.63	61	0.599
**Genital infection**	Within	Standard treatments *vs* DPP-4 inhibitors *vs* SGLT2 inhibitors	0	0	NA
**Genital infection**	Between	DPP-4 inhibitors *vs* SGLT2 inhibitors	3.501	3	0.321
**Genital infection**	Between	GLP-1 receptor agonists *vs* SGLT2 inhibitors	3.846	3	0.279
**Genital infection**	Between	Standard treatments *vs* DPP-4 inhibitors	2.128	3	0.546
**Genital infection**	Between	Standard treatments *vs* GLP-1 receptor agonists	3.846	3	0.279
**Genital infection**	Between	Standard treatments *vs* SGLT2 inhibitors	4.337	3	0.227
**Genital infection**	Between	Standard treatments *vs* DPP-4 inhibitors *vs* SGLT2 inhibitors	2.918	2	0.232
**Amputation**	Total	Total	22.512	13	0.048
**Amputation**	Total	Within designs	22.509	12	0.032
**Amputation**	Total	Between designs	0.003	1	0.957
**Amputation**	Total	tau.within	0.245	NA	NA
**Amputation**	Within	DPP-4 inhibitors *vs* SGLT2 inhibitors	0	0	NA
**Amputation**	Within	Standard treatments *vs* DPP-4 inhibitors	0	0	NA
**Amputation**	Within	Standard treatments *vs* GLP-1 receptor agonists	0.423	1	0.516
**Amputation**	Within	Standard treatments *vs* Non-steroidal MRAs	0.3	1	0.584
**Amputation**	Within	Standard treatments *vs* SGLT2 inhibitors	18.213	9	0.033
**Amputation**	Within	Standard treatments *vs* Thiazolidinediones	3.573	1	0.059
**Amputation**	Between	DPP-4 inhibitors *vs* SGLT2 inhibitors	0	0	NA
**Amputation**	Between	Standard treatments *vs* DPP-4 inhibitors	0	0	NA
**Amputation**	Between	Standard treatments *vs* SGLT2 inhibitors	0	0	NA

Outcome	Overall design	Specific design	Q	df	P-value
**Ketoacidosis due to diabetes**	Total	Total	20.529	29	0.876
**Ketoacidosis due to diabetes**	Total	Within designs	18.419	26	0.86
**Ketoacidosis due to diabetes**	Total	Between designs	2.11	3	0.55
**Ketoacidosis due to diabetes**	Total	tau.within	0	NA	NA
**Ketoacidosis due to diabetes**	Within	DPP-4 inhibitors *vs* GLP-1 receptor agonists	0	0	NA
**Ketoacidosis due to diabetes**	Within	DPP-4 inhibitors *vs* SGLT2 inhibitors	1.581	1	0.209
**Ketoacidosis due to diabetes**	Within	DPP-4 inhibitors *vs* Sulfonylureas	0.095	1	0.757
**Ketoacidosis due to diabetes**	Within	Metformin *vs* SGLT2 inhibitors	0	0	NA
**Ketoacidosis due to diabetes**	Within	SGLT2 inhibitors *vs* Sulfonylureas	0	0	NA
**Ketoacidosis due to diabetes**	Within	Standard treatments *vs* DPP-4 inhibitors	2.336	6	0.886
**Ketoacidosis due to diabetes**	Within	Standard treatments *vs* GLP-1 receptor agonists	3.96	6	0.682
**Ketoacidosis due to diabetes**	Within	Standard treatments *vs* Non-steroidal MRAs	1.316	1	0.251
**Ketoacidosis due to diabetes**	Within	Standard treatments *vs* SGLT2 inhibitors	9.131	11	0.61
**Ketoacidosis due to diabetes**	Within	Alpha-glucosidase inhibitors *vs* Meglitinides *vs* Sulfonylureas	0	0	NA
**Ketoacidosis due to diabetes**	Between	DPP-4 inhibitors *vs* GLP-1 receptor agonists	1.814	2	0.404
**Ketoacidosis due to diabetes**	Between	DPP-4 inhibitors *vs* SGLT2 inhibitors	0.739	2	0.691
**Ketoacidosis due to diabetes**	Between	DPP-4 inhibitors *vs* Sulfonylureas	1.678	2	0.432
**Ketoacidosis due to diabetes**	Between	SGLT2 inhibitors *vs* Sulfonylureas	1.678	2	0.432
**Ketoacidosis due to diabetes**	Between	Standard treatments *vs* DPP-4 inhibitors	1.287	2	0.526
**Ketoacidosis due to diabetes**	Between	Standard treatments *vs* GLP-1 receptor agonists	1.814	2	0.404
**Ketoacidosis due to diabetes**	Between	Standard treatments *vs* SGLT2 inhibitors	0.193	2	0.908
**Health-related quality of life**	Total	Total	78.162	25	0
**Health-related quality of life**	Total	Within designs	31.934	16	0.01
**Health-related quality of life**	Total	Between designs	46.228	9	0
**Health-related quality of life**	Total	tau.within	0.091	NA	NA

Outcome	Overall design	Specific design	Q	df	P-value
**Health-related quality of life**	Within	Alpha-glucosidase inhibitors *vs* DPP-4 inhibitors	0	0	NA
**Health-related quality of life**	Within	Basal insulin *vs* Bolus insulin	0	0	NA
**Health-related quality of life**	Within	Basal insulin *vs* GLP-1 receptor agonists	0	0	NA
**Health-related quality of life**	Within	Basal insulin *vs* Tirzepatide	0	0	NA
**Health-related quality of life**	Within	Bolus insulin *vs* GLP-1 receptor agonists	2.823	3	0.42
**Health-related quality of life**	Within	DPP-4 inhibitors *vs* GLP-1 receptor agonists	1.108	2	0.575
**Health-related quality of life**	Within	DPP-4 inhibitors *vs* Metformin	0	0	NA
**Health-related quality of life**	Within	DPP-4 inhibitors *vs* SGLT2 inhibitors	0	0	NA
**Health-related quality of life**	Within	GLP-1 receptor agonists *vs* SGLT2 inhibitors	3.319	1	0.068
**Health-related quality of life**	Within	GLP-1 receptor agonists *vs* Tirzepatide	0	0	NA
**Health-related quality of life**	Within	Meglitinides *vs* Thiazolidinediones	0	0	NA
**Health-related quality of life**	Within	Standard treatments *vs* DPP-4 inhibitors	0	0	NA
**Health-related quality of life**	Within	Standard treatments *vs* GLP-1 receptor agonists	12.583	9	0.182
**Health-related quality of life**	Within	Standard treatments *vs* SGLT2 inhibitors	12.101	1	0.001
**Health-related quality of life**	Within	Standard treatments *vs* Sulfonylureas	0	0	NA
**Health-related quality of life**	Within	DPP-4 inhibitors *vs* GLP-1 receptor agonists *vs* Metformin *vs* Thiazolidinediones	0	0	NA
**Health-related quality of life**	Within	Standard treatments *vs* DPP-4 inhibitors *vs* GLP-1 receptor agonists	0	0	NA
**Health-related quality of life**	Between	Basal insulin *vs* Bolus insulin	17.318	8	0.027
**Health-related quality of life**	Between	Basal insulin *vs* GLP-1 receptor agonists	45.823	8	0
**Health-related quality of life**	Between	Basal insulin *vs* Tirzepatide	16.178	8	0.04
**Health-related quality of life**	Between	Bolus insulin *vs* GLP-1 receptor agonists	17.318	8	0.027
**Health-related quality of life**	Between	DPP-4 inhibitors *vs* GLP-1 receptor agonists	40.736	8	0
**Health-related quality of life**	Between	DPP-4 inhibitors *vs* Metformin	46.223	8	0
**Health-related quality of life**	Between	DPP-4 inhibitors *vs* SGLT2 inhibitors	46.219	8	0
**Health-related quality of life**	Between	GLP-1 receptor agonists *vs* SGLT2 inhibitors	45.374	8	0

Outcome	Overall design	Specific design	Q	df	P-value
**Health-related quality of life**	Between	GLP-1 receptor agonists *vs* Tirzepatide	16.178	8	0.04
**Health-related quality of life**	Between	Standard treatments *vs* DPP-4 inhibitors	44.788	8	0
**Health-related quality of life**	Between	Standard treatments *vs* GLP-1 receptor agonists	45.348	8	0
**Health-related quality of life**	Between	Standard treatments *vs* SGLT2 inhibitors	45.217	8	0
**Health-related quality of life**	Between	Standard treatments *vs* DPP-4 inhibitors *vs* GLP-1 receptor agonists	40.334	7	0
**Body weight change**	Total	Total	4140.403	438	0
**Body weight change**	Total	Within designs	2748.062	346	0
**Body weight change**	Total	Between designs	1392.341	92	0
**Body weight change**	Total	tau.within	0.864	NA	NA
**Body weight change**	Within	Albiglutide *vs* Basal insulin	0	0	NA
**Body weight change**	Within	Albiglutide *vs* Bolus insulin	52.544	1	0
**Body weight change**	Within	Albiglutide *vs* DPP-4 inhibitors	0	0	NA
**Body weight change**	Within	Alpha-glucosidase inhibitors *vs* DPP-4 inhibitors	20.348	5	0.001
**Body weight change**	Within	Alpha-glucosidase inhibitors *vs* Metformin	9.743	1	0.002
**Body weight change**	Within	Alpha-glucosidase inhibitors *vs* SGLT2 inhibitors	0	0	NA
**Body weight change**	Within	Alpha-glucosidase inhibitors *vs* Sulfonylureas	0.182	1	0.67
**Body weight change**	Within	Alpha-glucosidase inhibitors *vs* Thiazolidinediones	3.063	1	0.08
**Body weight change**	Within	Basal insulin *vs* Basal-Bolus insulin	13.63	1	0
**Body weight change**	Within	Basal insulin *vs* DPP-4 inhibitors	7.163	1	0.007
**Body weight change**	Within	Basal insulin *vs* Dulaglutide	8.203	3	0.042
**Body weight change**	Within	Basal insulin *vs* Exenatide extended-release	31.345	2	0
**Body weight change**	Within	Basal insulin *vs* Exenatide immediate-release	0.148	1	0.7
**Body weight change**	Within	Basal insulin *vs* Liraglutide	1.934	3	0.586
**Body weight change**	Within	Basal insulin *vs* Semaglutide (Subcutaneous)	0	0	NA
**Body weight change**	Within	Basal insulin *vs* Sulfonylureas	0	0	NA

Outcome	Overall design	Specific design	Q	df	P-value
**Body weight change**	Within	Basal insulin *vs* Thiazolidinediones	0	1	1
**Body weight change**	Within	Basal insulin *vs* Tirzepatide	5.865	1	0.015
**Body weight change**	Within	Basal-Bolus insulin *vs* Thiazolidinediones	0	0	NA
**Body weight change**	Within	Bolus insulin *vs* Liraglutide	5.236	1	0.022
**Body weight change**	Within	Bolus insulin *vs* Lixisenatide	0	0	NA
**Body weight change**	Within	Bolus insulin *vs* Semaglutide (Subcutaneous)	0	0	NA
**Body weight change**	Within	Bolus insulin *vs* SGLT2 inhibitors	0	0	NA
**Body weight change**	Within	DPP-4 inhibitors *vs* Liraglutide	7.61	3	0.055
**Body weight change**	Within	DPP-4 inhibitors *vs* Lixisenatide	0	0	NA
**Body weight change**	Within	DPP-4 inhibitors *vs* Metformin	21.304	7	0.003
**Body weight change**	Within	DPP-4 inhibitors *vs* Semaglutide (Oral)	0	0	NA
**Body weight change**	Within	DPP-4 inhibitors *vs* Semaglutide (Subcutaneous)	0.261	1	0.609
**Body weight change**	Within	DPP-4 inhibitors *vs* SGLT2 inhibitors	19.312	12	0.081
**Body weight change**	Within	DPP-4 inhibitors *vs* Sulfonylureas	359.358	16	0
**Body weight change**	Within	DPP-4 inhibitors *vs* Thiazolidinediones	157.595	9	0
**Body weight change**	Within	Dulaglutide *vs* Metformin	0	0	NA
**Body weight change**	Within	Dulaglutide *vs* Sulfonylureas	0	0	NA
**Body weight change**	Within	Exenatide extended-release *vs* SGLT2 inhibitors	0	0	NA
**Body weight change**	Within	Exenatide immediate-release *vs* Metformin	0	0	NA
**Body weight change**	Within	Liraglutide *vs* Sulfonylureas	0	0	NA
**Body weight change**	Within	Meglitinides *vs* Metformin	0	0	NA
**Body weight change**	Within	Meglitinides *vs* Sulfonylureas	3.182	3	0.364
**Body weight change**	Within	Meglitinides *vs* Thiazolidinediones	0.496	1	0.481
**Body weight change**	Within	Metformin *vs* SGLT2 inhibitors	8.854	4	0.065
**Body weight change**	Within	Metformin *vs* Sulfonylureas	37.544	4	0

Outcome	Overall design	Specific design	Q	df	P-value
**Body weight change**	Within	Metformin *vs* Thiazolidinediones	31.344	6	0
**Body weight change**	Within	Semaglutide (Oral) *vs* SGLT2 inhibitors	0	0	NA
**Body weight change**	Within	Semaglutide (Subcutaneous) *vs* SGLT2 inhibitors	0	0	NA
**Body weight change**	Within	Semaglutide (Subcutaneous) *vs* Tirzepatide	0	0	NA
**Body weight change**	Within	SGLT2 inhibitors *vs* Sulfonylureas	26.337	7	0
**Body weight change**	Within	SGLT2 inhibitors *vs* Thiazolidinediones	0.896	1	0.344
**Body weight change**	Within	Standard treatments *vs* Albiglutide	2.247	2	0.325
**Body weight change**	Within	Standard treatments *vs* Alpha-glucosidase inhibitors	12.119	10	0.277
**Body weight change**	Within	Standard treatments *vs* Basal insulin	16.299	1	0
**Body weight change**	Within	Standard treatments *vs* DPP-4 inhibitors	228.759	70	0
**Body weight change**	Within	Standard treatments *vs* Dulaglutide	16.033	5	0.007
**Body weight change**	Within	Standard treatments *vs* Efpeglenatide	0	0	NA
**Body weight change**	Within	Standard treatments *vs* Exenatide extended-release	0.41	1	0.522
**Body weight change**	Within	Standard treatments *vs* Exenatide immediate-release	22.457	5	0
**Body weight change**	Within	Standard treatments *vs* Liraglutide	62.09	14	0
**Body weight change**	Within	Standard treatments *vs* Lixisenatide	9.12	9	0.426
**Body weight change**	Within	Standard treatments *vs* Loxenatide	0	0	NA
**Body weight change**	Within	Standard treatments *vs* Meglitinides	7.55	3	0.056
**Body weight change**	Within	Standard treatments *vs* Metformin	77.067	4	0
**Body weight change**	Within	Standard treatments *vs* Semaglutide (Oral)	14.87	3	0.002
**Body weight change**	Within	Standard treatments *vs* Semaglutide (Subcutaneous)	15.937	3	0.001
**Body weight change**	Within	Standard treatments *vs* SGLT2 inhibitors	732.252	69	0
**Body weight change**	Within	Standard treatments *vs* Sulfonylureas	1.13	1	0.288
**Body weight change**	Within	Standard treatments *vs* Thiazolidinediones	281.963	29	0
**Body weight change**	Within	Standard treatments *vs* Tirzepatide	2.334	1	0.127

Outcome	Overall design	Specific design	Q	df	P-value
**Body weight change**	Within	Sulfonylureas *vs* Thiazolidinediones	315.308	13	0
**Body weight change**	Within	Basal insulin *vs* DPP-4 inhibitors *vs* Liraglutide	0	0	NA
**Body weight change**	Within	Basal insulin *vs* DPP-4 inhibitors *vs* Liraglutide *vs* Sulfonylureas	0	0	NA
**Body weight change**	Within	DPP-4 inhibitors *vs* Exenatide extended-release *vs* Metformin *vs* Thiazolidinediones	0	0	NA
**Body weight change**	Within	DPP-4 inhibitors *vs* Exenatide extended-release *vs* Thiazolidinediones	0	0	NA
**Body weight change**	Within	Metformin *vs* Sulfonylureas *vs* Thiazolidinediones	83.153	2	0
**Body weight change**	Within	SGLT2 inhibitors *vs* Sulfonylureas *vs* Thiazolidinediones	0	0	NA
**Body weight change**	Within	Standard treatments *vs* Albiglutide *vs* DPP-4 inhibitors *vs* Sulfonylureas	0	0	NA
**Body weight change**	Within	Standard treatments *vs* Albiglutide *vs* Thiazolidinediones	0	0	NA
**Body weight change**	Within	Standard treatments *vs* Alpha-glucosidase inhibitors *vs* Metformin	0	0	NA
**Body weight change**	Within	Standard treatments *vs* Basal insulin *vs* Dulaglutide *vs* Exenatide extended-release	0	0	NA
**Body weight change**	Within	Standard treatments *vs* Basal insulin *vs* Liraglutide	11.726	2	0.003
**Body weight change**	Within	Standard treatments *vs* DPP-4 inhibitors *vs* Dulaglutide	0	0	NA
**Body weight change**	Within	Standard treatments *vs* DPP-4 inhibitors *vs* Exenatide extended-release	0	0	NA
**Body weight change**	Within	Standard treatments *vs* DPP-4 inhibitors *vs* Metformin	1.741	2	0.419
**Body weight change**	Within	Standard treatments *vs* DPP-4 inhibitors *vs* SGLT2 inhibitors	0	0	NA
**Body weight change**	Within	Standard treatments *vs* Dulaglutide *vs* Tirzepatide	0	0	NA
**Body weight change**	Within	Standard treatments *vs* Liraglutide *vs* Semaglutide (Oral)	0	0	NA
**Body weight change**	Within	Standard treatments *vs* Liraglutide *vs* Sulfonylureas	0	0	NA
**Body weight change**	Within	Standard treatments *vs* Liraglutide *vs* Thiazolidinediones	0	0	NA
**Body weight change**	Within	Standard treatments *vs* Semaglutide (Oral) *vs* Semaglutide (Subcutaneous)	0	0	NA
**Body weight change**	Within	Standard treatments *vs* Semaglutide (Subcutaneous) *vs* Tirzepatide	0	0	NA
**Body weight change**	Between	Albiglutide *vs* Basal insulin	1389.252	91	0
**Body weight change**	Between	Albiglutide *vs* Bolus insulin	1321.747	91	0
**Body weight change**	Between	Albiglutide *vs* DPP-4 inhibitors	1385.545	91	0

Outcome	Overall design	Specific design	Q	df	P-value
**Body weight change**	Between	Alpha-glucosidase inhibitors *vs* DPP-4 inhibitors	1390.227	91	0
**Body weight change**	Between	Alpha-glucosidase inhibitors *vs* Metformin	1382.16	91	0
**Body weight change**	Between	Alpha-glucosidase inhibitors *vs* SGLT2 inhibitors	1391.267	91	0
**Body weight change**	Between	Alpha-glucosidase inhibitors *vs* Sulfonylureas	1385.198	91	0
**Body weight change**	Between	Alpha-glucosidase inhibitors *vs* Thiazolidinediones	1391.489	91	0
**Body weight change**	Between	Basal insulin *vs* Basal-Bolus insulin	1390.973	91	0
**Body weight change**	Between	Basal insulin *vs* DPP-4 inhibitors	1392.207	91	0
**Body weight change**	Between	Basal insulin *vs* Dulaglutide	1381.661	91	0
**Body weight change**	Between	Basal insulin *vs* Exenatide extended-release	1390.714	91	0
**Body weight change**	Between	Basal insulin *vs* Exenatide immediate-release	1391.374	91	0
**Body weight change**	Between	Basal insulin *vs* Liraglutide	1341.27	91	0
**Body weight change**	Between	Basal insulin *vs* Semaglutide (Subcutaneous)	1390.998	91	0
**Body weight change**	Between	Basal insulin *vs* Sulfonylureas	1390.536	91	0
**Body weight change**	Between	Basal insulin *vs* Thiazolidinediones	1388.72	91	0
**Body weight change**	Between	Basal insulin *vs* Tirzepatide	1378.792	91	0
**Body weight change**	Between	Basal-Bolus insulin *vs* Thiazolidinediones	1390.973	91	0
**Body weight change**	Between	Bolus insulin *vs* Liraglutide	1315.868	91	0
**Body weight change**	Between	Bolus insulin *vs* Lixisenatide	1392.056	91	0
**Body weight change**	Between	Bolus insulin *vs* Semaglutide (Subcutaneous)	1309.746	91	0
**Body weight change**	Between	Bolus insulin *vs* SGLT2 inhibitors	1332.491	91	0
**Body weight change**	Between	DPP-4 inhibitors *vs* Liraglutide	1391.983	91	0
**Body weight change**	Between	DPP-4 inhibitors *vs* Lixisenatide	1392.34	91	0
**Body weight change**	Between	DPP-4 inhibitors *vs* Metformin	1386.479	91	0
**Body weight change**	Between	DPP-4 inhibitors *vs* Semaglutide (Oral)	1374.337	91	0
**Body weight change**	Between	DPP-4 inhibitors *vs* Semaglutide (Subcutaneous)	1373.991	91	0

Outcome	Overall design	Specific design	Q	df	P-value
**Body weight change**	Between	DPP-4 inhibitors *vs* SGLT2 inhibitors	1391.925	91	0
**Body weight change**	Between	DPP-4 inhibitors *vs* Sulfonylureas	1293.365	91	0
**Body weight change**	Between	DPP-4 inhibitors *vs* Thiazolidinediones	1303.837	91	0
**Body weight change**	Between	Dulaglutide *vs* Metformin	1391.867	91	0
**Body weight change**	Between	Dulaglutide *vs* Sulfonylureas	1376.788	91	0
**Body weight change**	Between	Exenatide extended-release *vs* SGLT2 inhibitors	1388.747	91	0
**Body weight change**	Between	Exenatide immediate-release *vs* Metformin	1378.772	91	0
**Body weight change**	Between	Liraglutide *vs* Sulfonylureas	1392.016	91	0
**Body weight change**	Between	Meglitinides *vs* Metformin	1392.329	91	0
**Body weight change**	Between	Meglitinides *vs* Sulfonylureas	1391.602	91	0
**Body weight change**	Between	Meglitinides *vs* Thiazolidinediones	1392.32	91	0
**Body weight change**	Between	Metformin *vs* SGLT2 inhibitors	1364.702	91	0
**Body weight change**	Between	Metformin *vs* Sulfonylureas	1389.54	91	0
**Body weight change**	Between	Metformin *vs* Thiazolidinediones	1265.692	91	0
**Body weight change**	Between	Semaglutide (Oral) *vs* SGLT2 inhibitors	1386.852	91	0
**Body weight change**	Between	Semaglutide (Subcutaneous) *vs* SGLT2 inhibitors	1351.929	91	0
**Body weight change**	Between	Semaglutide (Subcutaneous) *vs* Tirzepatide	1392.171	91	0
**Body weight change**	Between	SGLT2 inhibitors *vs* Sulfonylureas	1361.132	91	0
**Body weight change**	Between	SGLT2 inhibitors *vs* Thiazolidinediones	1389.503	91	0
**Body weight change**	Between	Standard treatments *vs* Albiglutide	1390.045	91	0
**Body weight change**	Between	Standard treatments *vs* Alpha-glucosidase inhibitors	1387.755	91	0
**Body weight change**	Between	Standard treatments *vs* Basal insulin	1392.013	91	0
**Body weight change**	Between	Standard treatments *vs* DPP-4 inhibitors	1324.584	91	0
**Body weight change**	Between	Standard treatments *vs* Dulaglutide	1373.685	91	0
**Body weight change**	Between	Standard treatments *vs* Exenatide extended-release	1388.283	91	0

Outcome	Overall design	Specific design	Q	df	P-value
**Body weight change**	Between	Standard treatments *vs* Exenatide immediate-release	1380.611	91	0
**Body weight change**	Between	Standard treatments *vs* Liraglutide	1319.4	91	0
**Body weight change**	Between	Standard treatments *vs* Lixisenatide	1392.154	91	0
**Body weight change**	Between	Standard treatments *vs* Meglitinides	1391.638	91	0
**Body weight change**	Between	Standard treatments *vs* Metformin	1243.569	91	0
**Body weight change**	Between	Standard treatments *vs* Semaglutide (Oral)	1376.513	91	0
**Body weight change**	Between	Standard treatments *vs* Semaglutide (Subcutaneous)	1392.062	91	0
**Body weight change**	Between	Standard treatments *vs* SGLT2 inhibitors	1348.855	91	0
**Body weight change**	Between	Standard treatments *vs* Sulfonylureas	1105.679	91	0
**Body weight change**	Between	Standard treatments *vs* Thiazolidinediones	1245.582	91	0
**Body weight change**	Between	Standard treatments *vs* Tirzepatide	1388.925	91	0
**Body weight change**	Between	Sulfonylureas *vs* Thiazolidinediones	1355.322	91	0
**Body weight change**	Between	Basal insulin *vs* DPP-4 inhibitors *vs* Liraglutide	1390.334	90	0
**Body weight change**	Between	Basal insulin *vs* DPP-4 inhibitors *vs* Liraglutide *vs* Sulfonylureas	1348.418	89	0
**Body weight change**	Between	DPP-4 inhibitors *vs* Exenatide extended-release *vs* Metformin *vs* Thiazolidinediones	1392.004	89	0
**Body weight change**	Between	DPP-4 inhibitors *vs* Exenatide extended-release *vs* Thiazolidinediones	1374.954	90	0
**Body weight change**	Between	Metformin *vs* Sulfonylureas *vs* Thiazolidinediones	1344.548	90	0
**Body weight change**	Between	SGLT2 inhibitors *vs* Sulfonylureas *vs* Thiazolidinediones	1389.242	90	0
**Body weight change**	Between	Standard treatments *vs* Albiglutide *vs* DPP-4 inhibitors *vs* Sulfonylureas	1383.806	89	0
**Body weight change**	Between	Standard treatments *vs* Albiglutide *vs* Thiazolidinediones	1364.687	90	0
**Body weight change**	Between	Standard treatments *vs* Alpha-glucosidase inhibitors *vs* Metformin	1386.852	90	0
**Body weight change**	Between	Standard treatments *vs* Basal insulin *vs* Dulaglutide *vs* Exenatide extended-release	1384.363	89	0
**Body weight change**	Between	Standard treatments *vs* Basal insulin *vs* Liraglutide	1386.369	90	0
**Body weight change**	Between	Standard treatments *vs* DPP-4 inhibitors *vs* Dulaglutide	1390.162	90	0
**Body weight change**	Between	Standard treatments *vs* DPP-4 inhibitors *vs* Exenatide extended-release	1377.767	90	0

Outcome	Overall design	Specific design	Q	df	P-value
**Body weight change**	Between	Standard treatments *vs* DPP-4 inhibitors *vs* Metformin	1379.6	90	0
**Body weight change**	Between	Standard treatments *vs* DPP-4 inhibitors *vs* SGLT2 inhibitors	1389.828	90	0
**Body weight change**	Between	Standard treatments *vs* Dulaglutide *vs* Tirzepatide	1368.806	90	0
**Body weight change**	Between	Standard treatments *vs* Liraglutide *vs* Semaglutide (Oral)	1391.087	90	0
**Body weight change**	Between	Standard treatments *vs* Liraglutide *vs* Sulfonylureas	1390.308	90	0
**Body weight change**	Between	Standard treatments *vs* Liraglutide *vs* Thiazolidinediones	1367.309	90	0
**Body weight change**	Between	Standard treatments *vs* Semaglutide (Oral) *vs* Semaglutide (Subcutaneous)	1391.542	90	0
**Body weight change**	Between	Standard treatments *vs* Semaglutide (Subcutaneous) *vs* Tirzepatide	1389.347	90	0

4.5 Intransitivity assessments

Notes: The study assessed the intransitivity between direct comparisons by comparing their distribution of patients' characteristics. We chose the potential effect modifiers from the prognostic variables identified by the prognostic research and the systematic review of risk prediction models. The illustrated figures show pooled mean or proportion and corresponding confidence interval by the random-effect single-arm meta-analyses via a random-intercept logistic regression. The duration of baseline diabetes was shown using median and interquartile due to skew distribution.

4.5.1 All-cause death

4.5.1.1 Age

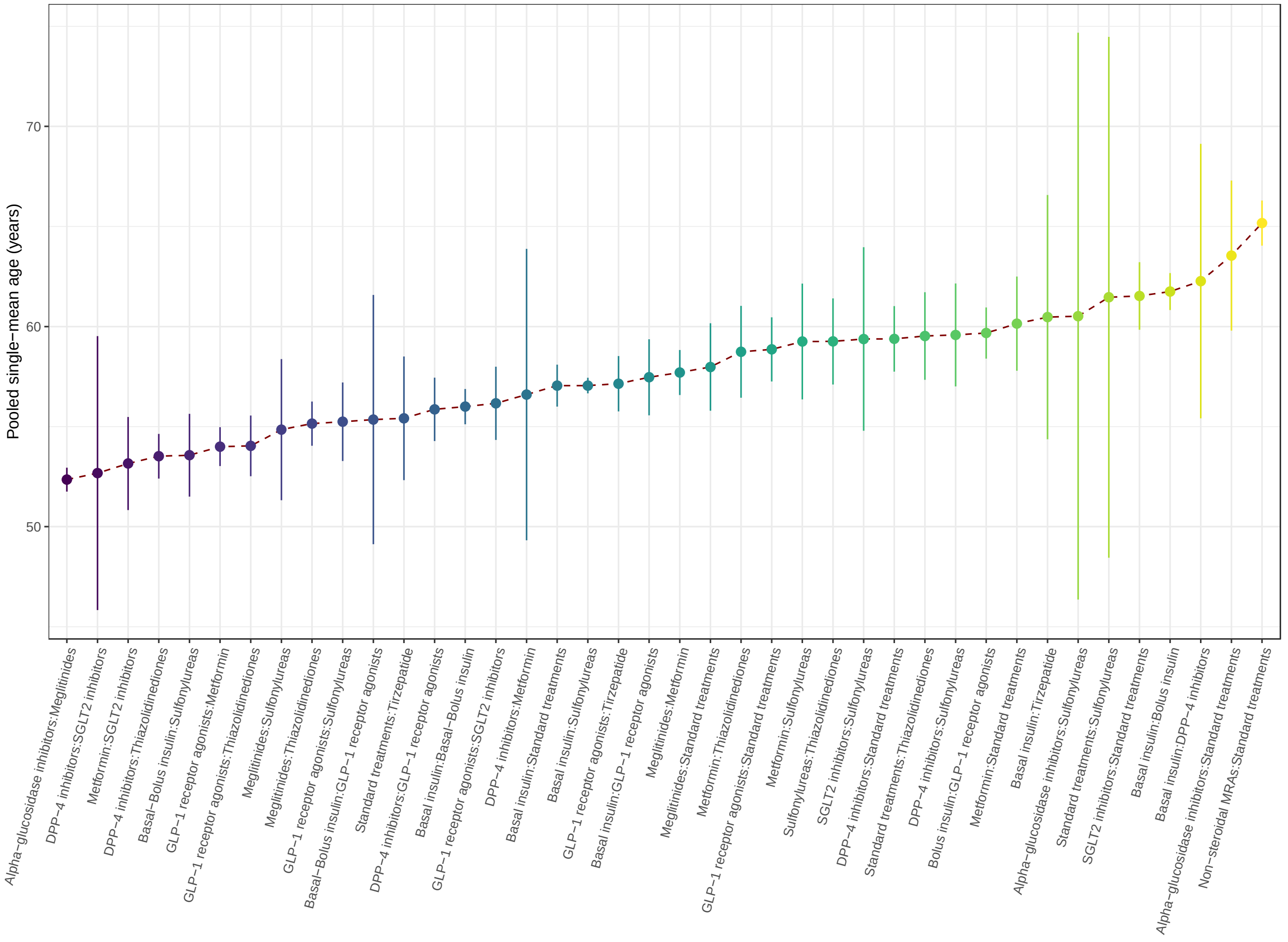

4.5.1.2 Body mass index

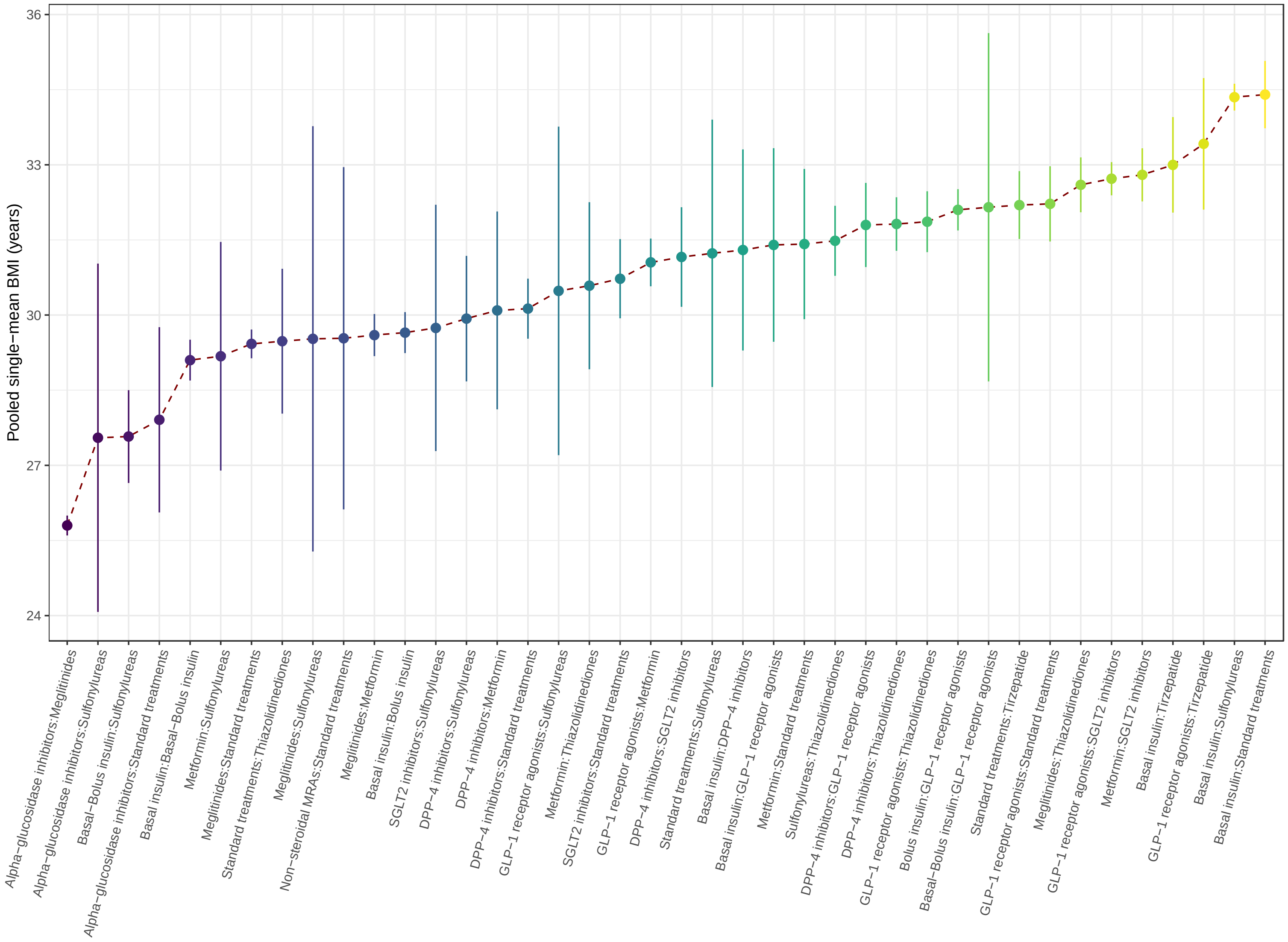

4.5.1.3 Proportion of cardiovascular disease

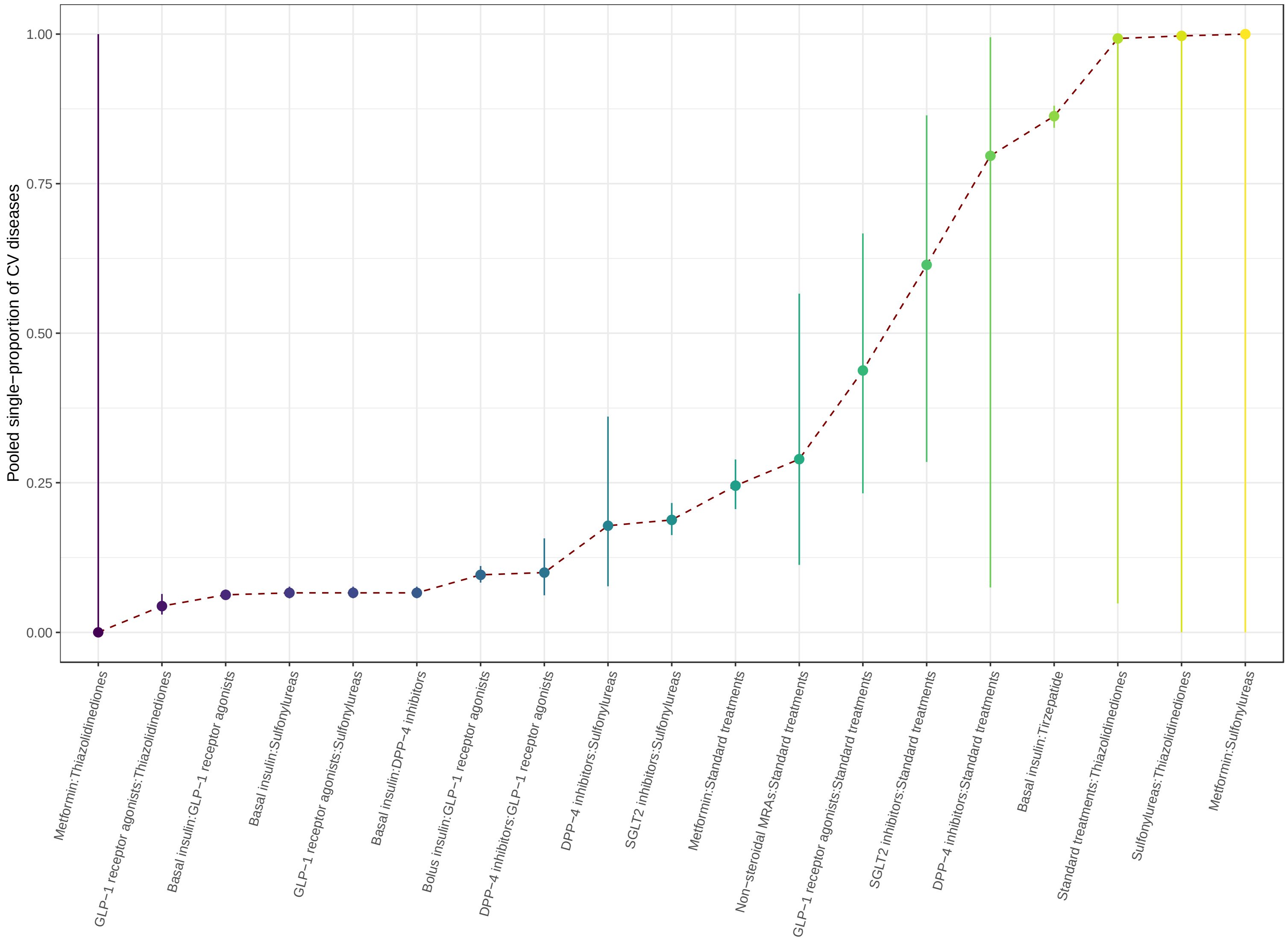

4.5.1.4 Duration of baseline diabetes

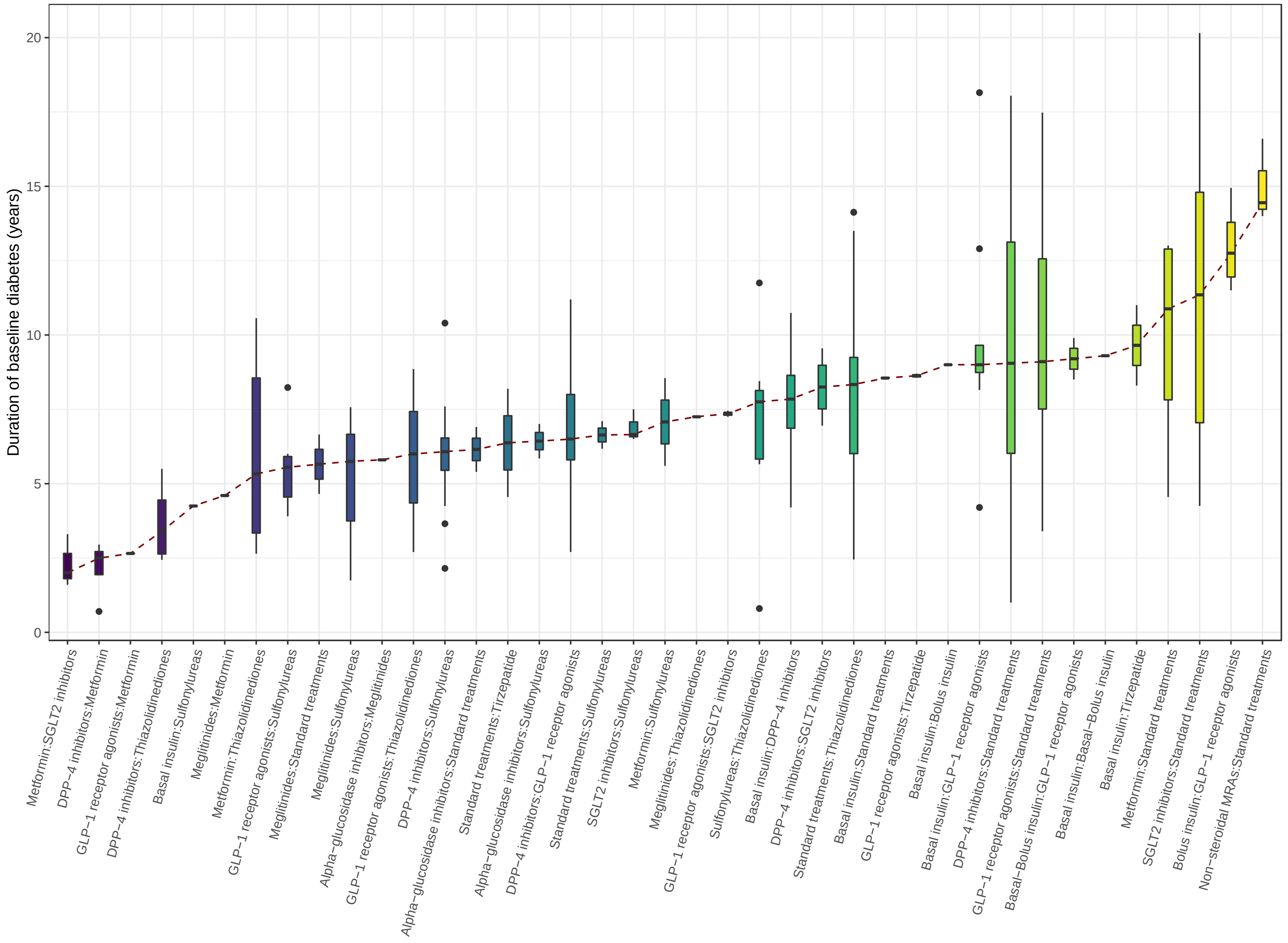

4.5.1.5 HbA1c

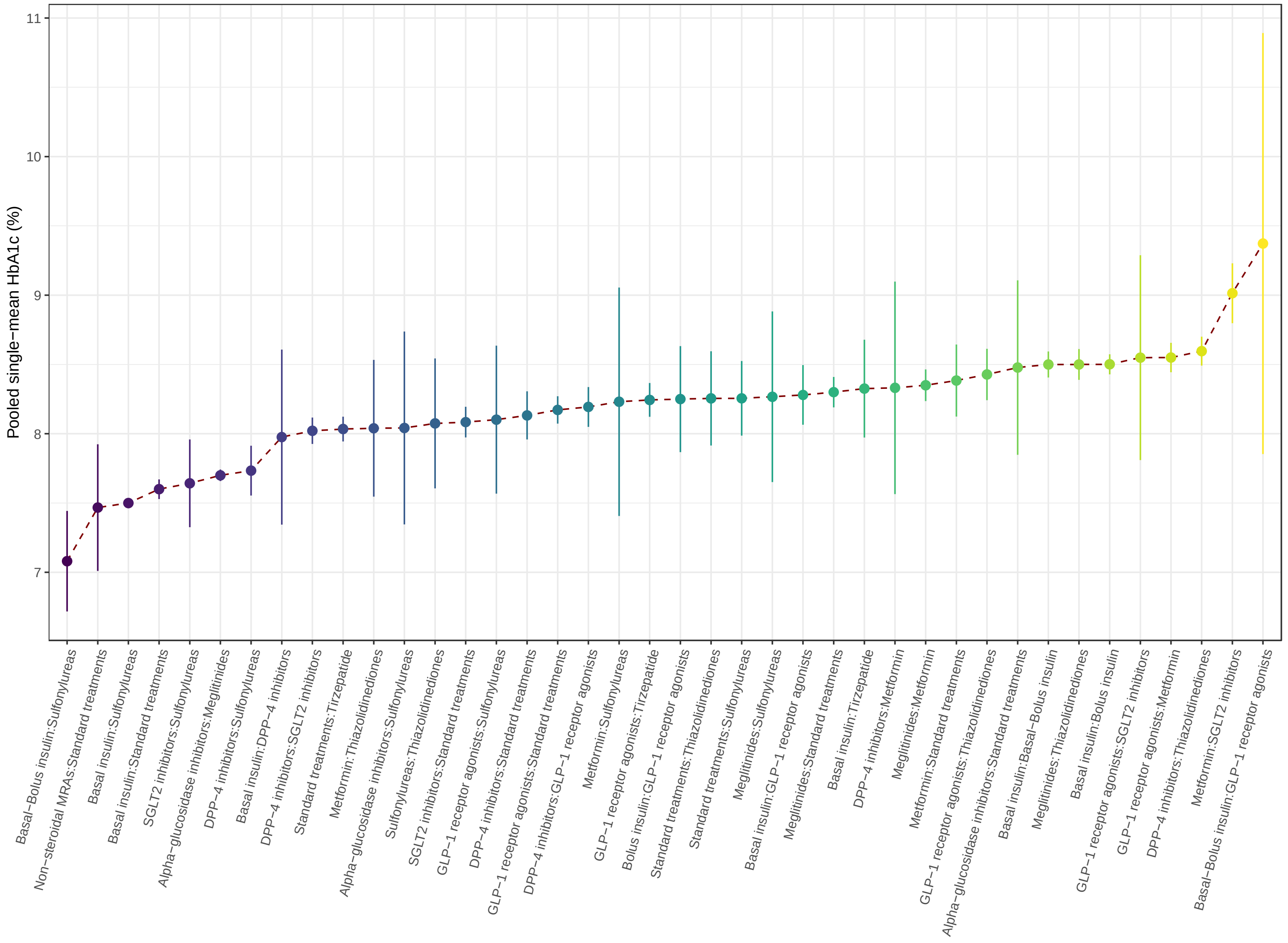
Pooled single−mean HbA1c (%)
11
10
9
8
7
Basal−Bolus insulin:Sulfonylureas
Non−steroidal MRAs:Standard treatments
Basal insulin:Sulfonylureas
Basal insulin:Standard treatments
SGLT2 inhibitors:Sulfonylureas
Alpha−glucosidase inhibitors:Meglitinides
DPP−4 inhibitors:Sulfonylureas
Basal insulin:DPP−4 inhibitors
DPP−4 inhibitors:SGLT2 inhibitors
Standard treatments:Tirzepatide
Metformin:Thiazolidinediones
Alpha−glucosidase inhibitors:Sulfonylureas
Sulfonylureas:Thiazolidinediones
SGLT2 inhibitors:Standard treatments
GLP−1 receptor agonists:Sulfonylureas
DPP−4 inhibitors:Standard treatments
GLP−1 receptor agonists:Standard treatments
DPP−4 inhibitors:GLP−1 receptor agonists
Metformin:Sulfonylureas
GLP−1 receptor agonists:Tirzepatide
Bolus insulin:GLP−1 receptor agonists
Standard treatments:Thiazolidinediones
Standard treatments:Sulfonylureas
Meglitinides:Sulfonylureas
Basal insulin:GLP−1 receptor agonists
Meglitinides:Standard treatments
Basal insulin:Tirzepatide
DPP−4 inhibitors:Metformin
Meglitinides:Metformin
Metformin:Standard treatments
GLP−1 receptor agonists:Thiazolidinediones
Alpha−glucosidase inhibitors:Standard treatments
Basal insulin:Basal−Bolus insulin
Meglitinides:Thiazolidinediones
Basal insulin:Bolus insulin
GLP−1 receptor agonists:SGLT2 inhibitors
GLP−1 receptor agonists:Metformin
DPP−4 inhibitors:Thiazolidinediones
Metformin:SGLT2 inhibitors
Basal−Bolus insulin:GLP−1 receptor agonists

4.5.1.6 Proportion of males

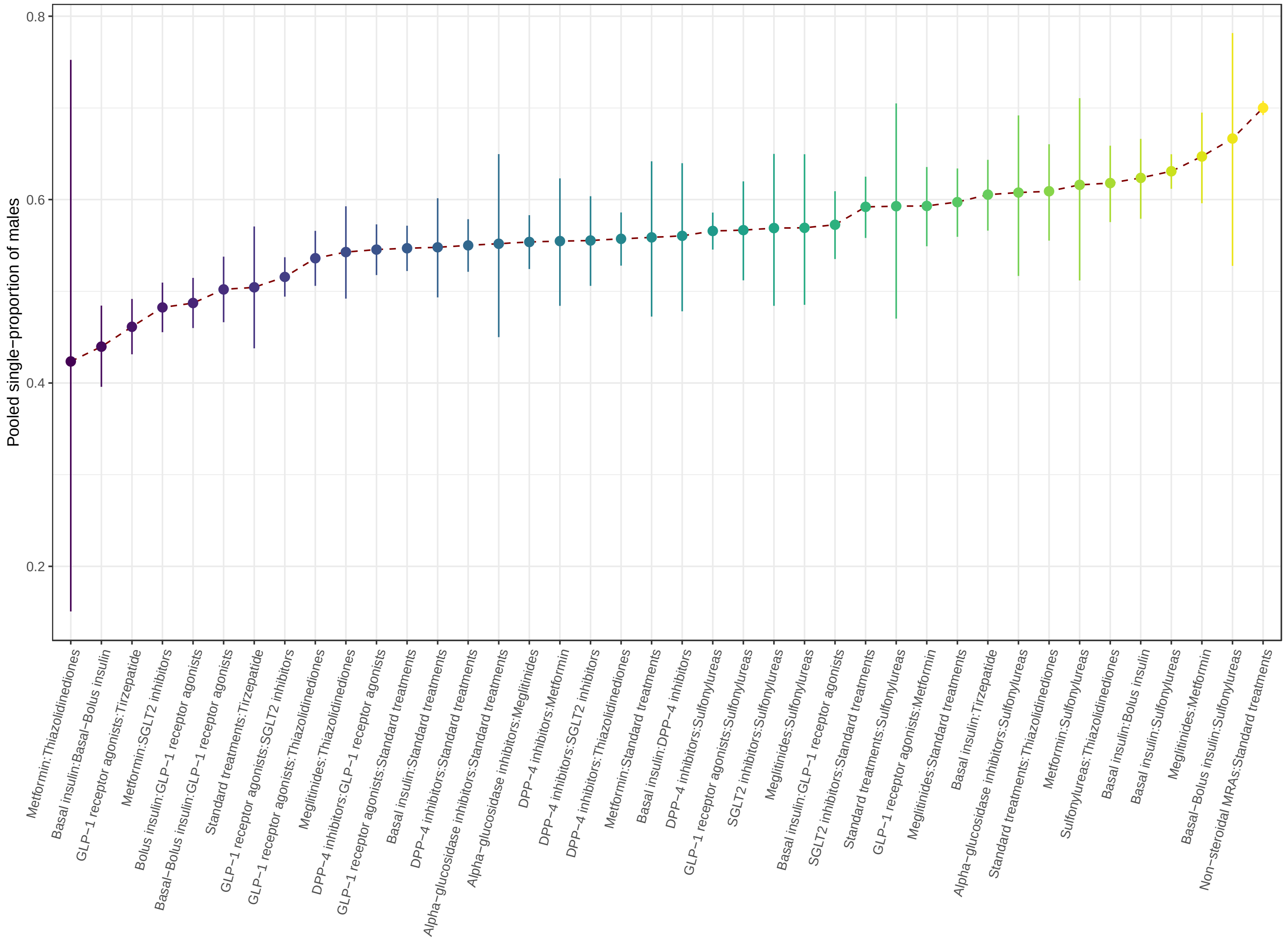

4.5.2 Cardiovascular death

4.5.2.1 Age

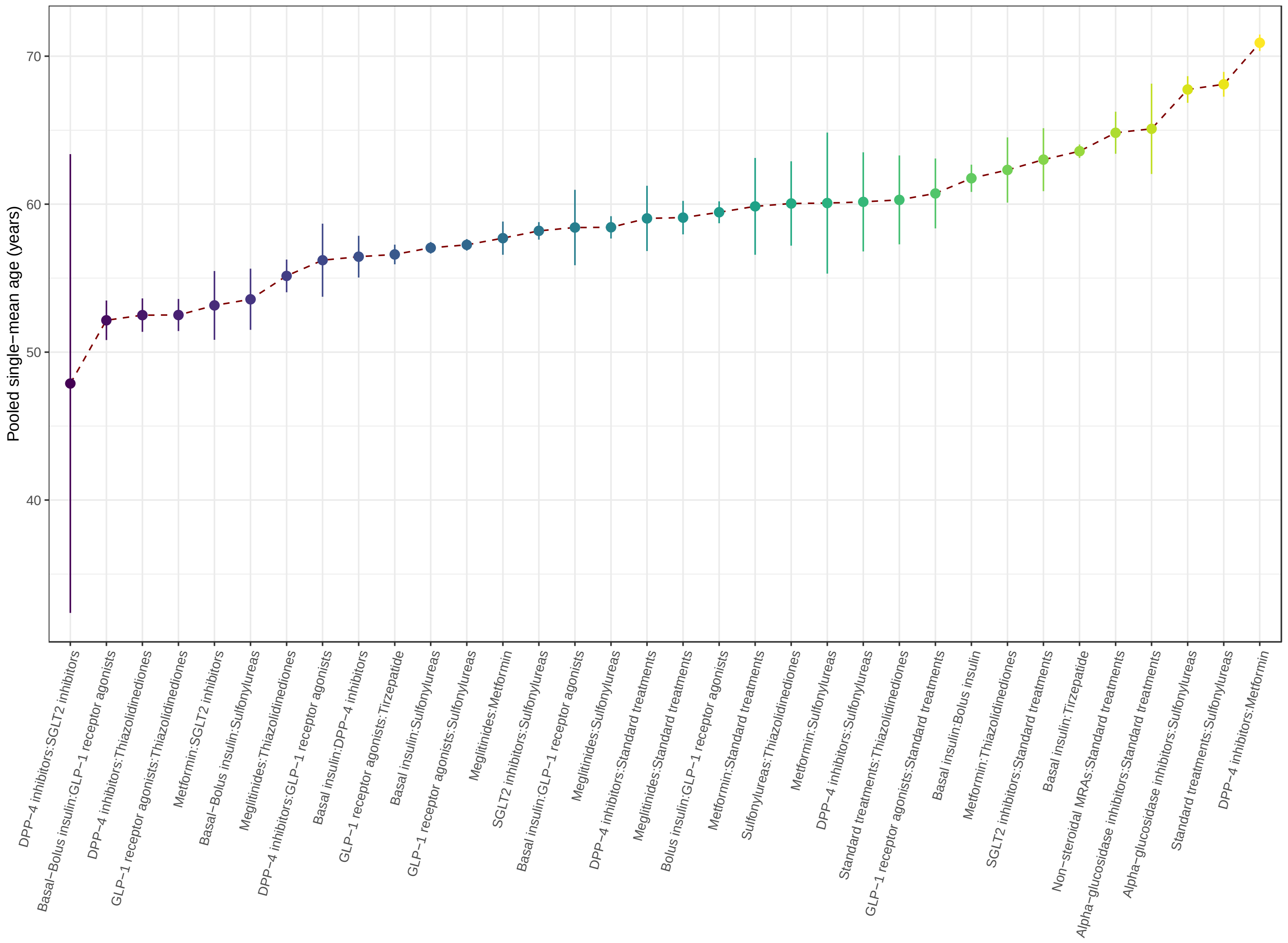

4.5.2.2 Body mass index

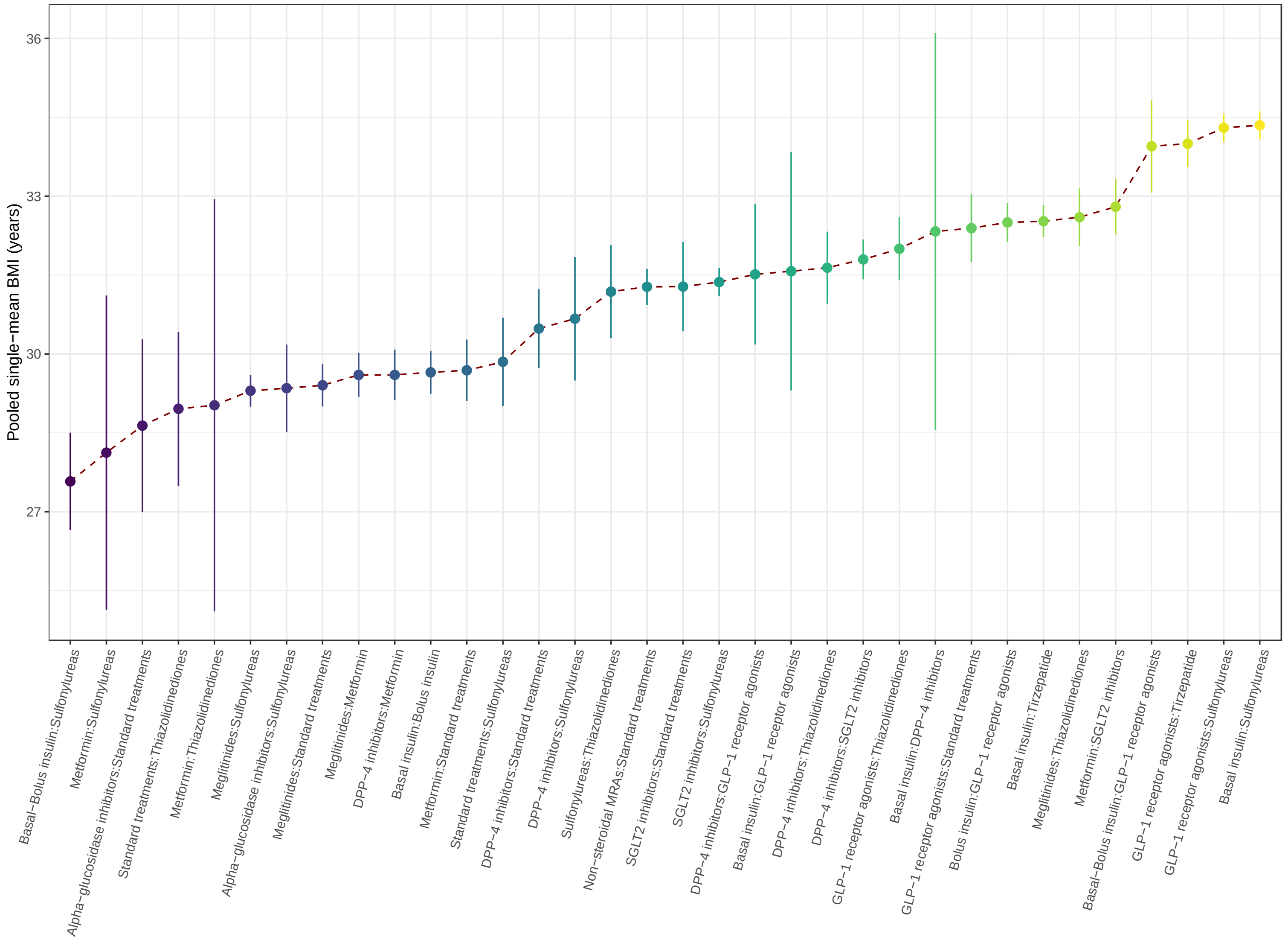

4.5.2.3 Proportion of cardiovascular disease

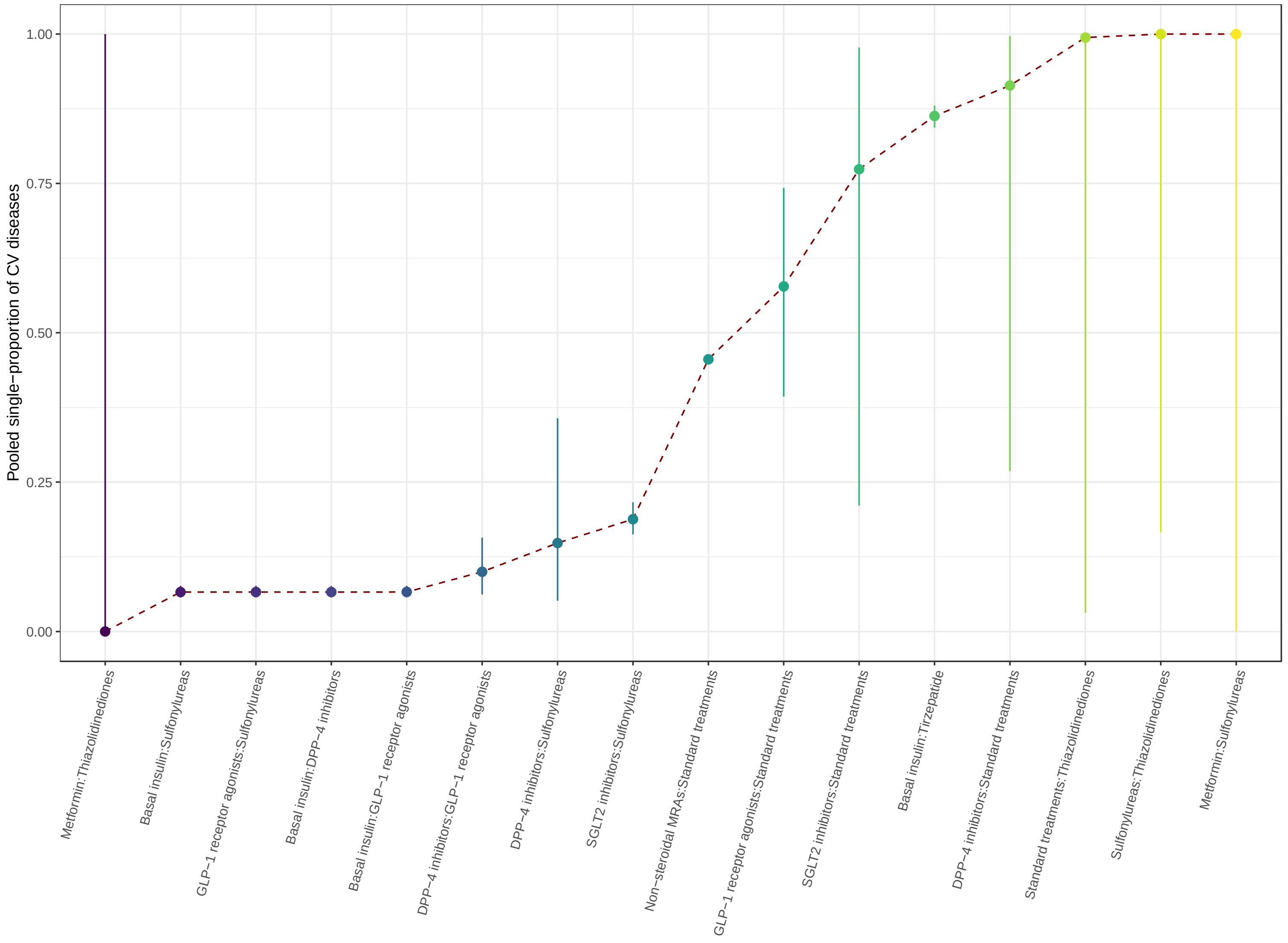

4.5.2.4 Duration of baseline diabetes

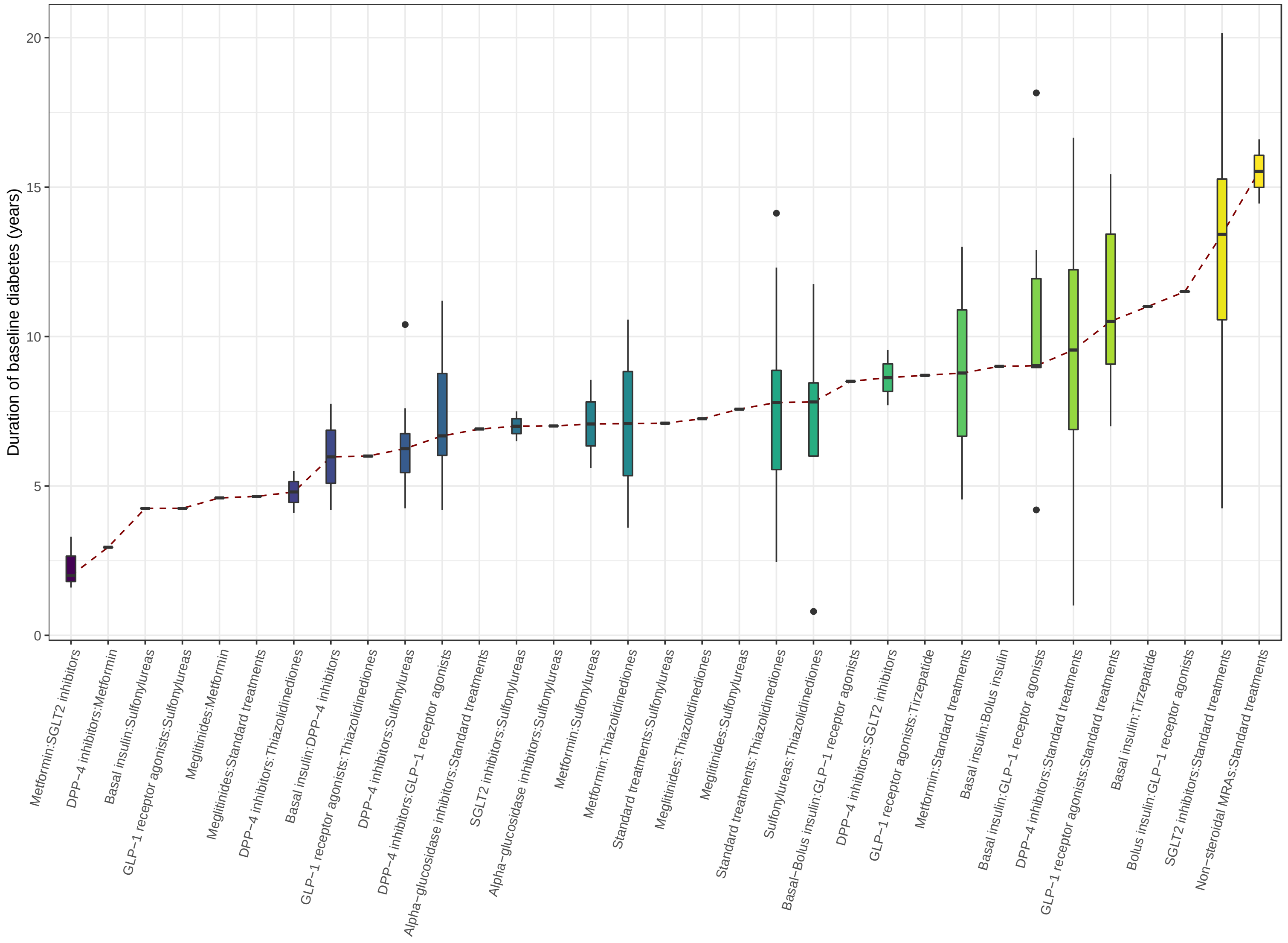

4.5.2.5 HbA1c

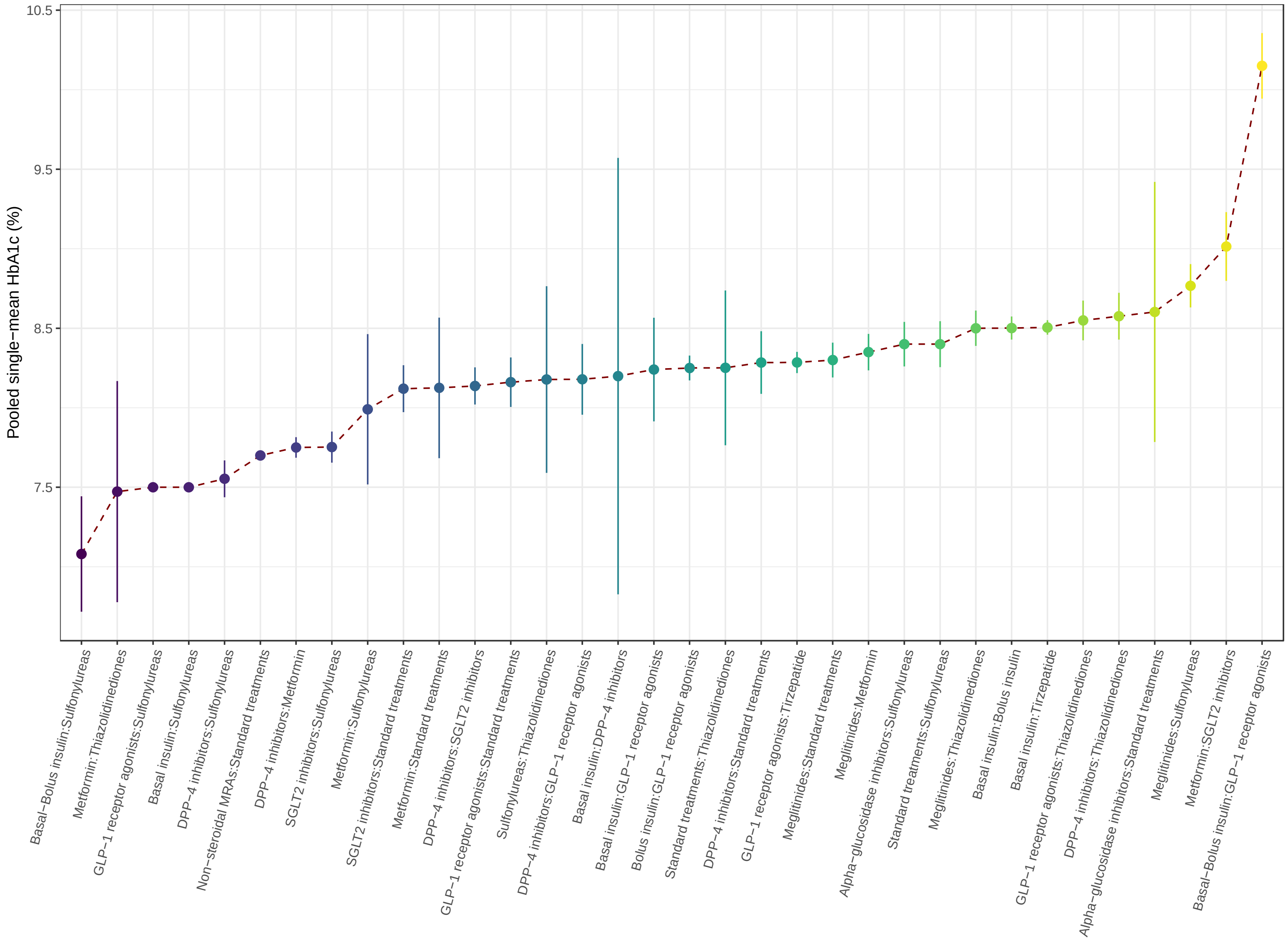

Pooled single−mean HbA1c (%)
10.5
9.5
8.5
7.5
Basal−Bolus insulin:Sulfonylureas
Metformin:Thiazolidinediones
GLP−1 receptor agonists:Sulfonylureas
Basal insulin:Sulfonylureas
DPP−4 inhibitors:Sulfonylureas
Non−steroidal MRAs:Standard treatments
DPP−4 inhibitors:Metformin
SGLT2 inhibitors:Sulfonylureas
Metformin:Sulfonylureas
SGLT2 inhibitors:Standard treatments
Metformin:Standard treatments
DPP−4 inhibitors:SGLT2 inhibitors
GLP−1 receptor agonists:Standard treatments
Sulfonylureas:Thiazolidinediones
DPP−4 inhibitors:GLP−1 receptor agonists
Basal insulin:DPP−4 inhibitors
Basal insulin:GLP−1 receptor agonists
Bolus insulin:GLP−1 receptor agonists
Standard treatments:Thiazolidinediones
DPP−4 inhibitors:Standard treatments
GLP−1 receptor agonists:Tirzepatide
Meglitinides:Standard treatments
Meglitinides:Metformin
Alpha−glucosidase inhibitors:Sulfonylureas
Standard treatments:Sulfonylureas
Meglitinides:Thiazolidinediones
Basal insulin:Bolus insulin
Basal insulin:Tirzepatide
GLP−1 receptor agonists:Thiazolidinediones
DPP−4 inhibitors:Thiazolidinediones
Alpha−glucosidase inhibitors:Standard treatments
Meglitinides:Sulfonylureas
Metformin:SGLT2 inhibitors
Basal−Bolus insulin:GLP−1 receptor agonists

4.5.2.6 Proportion of males

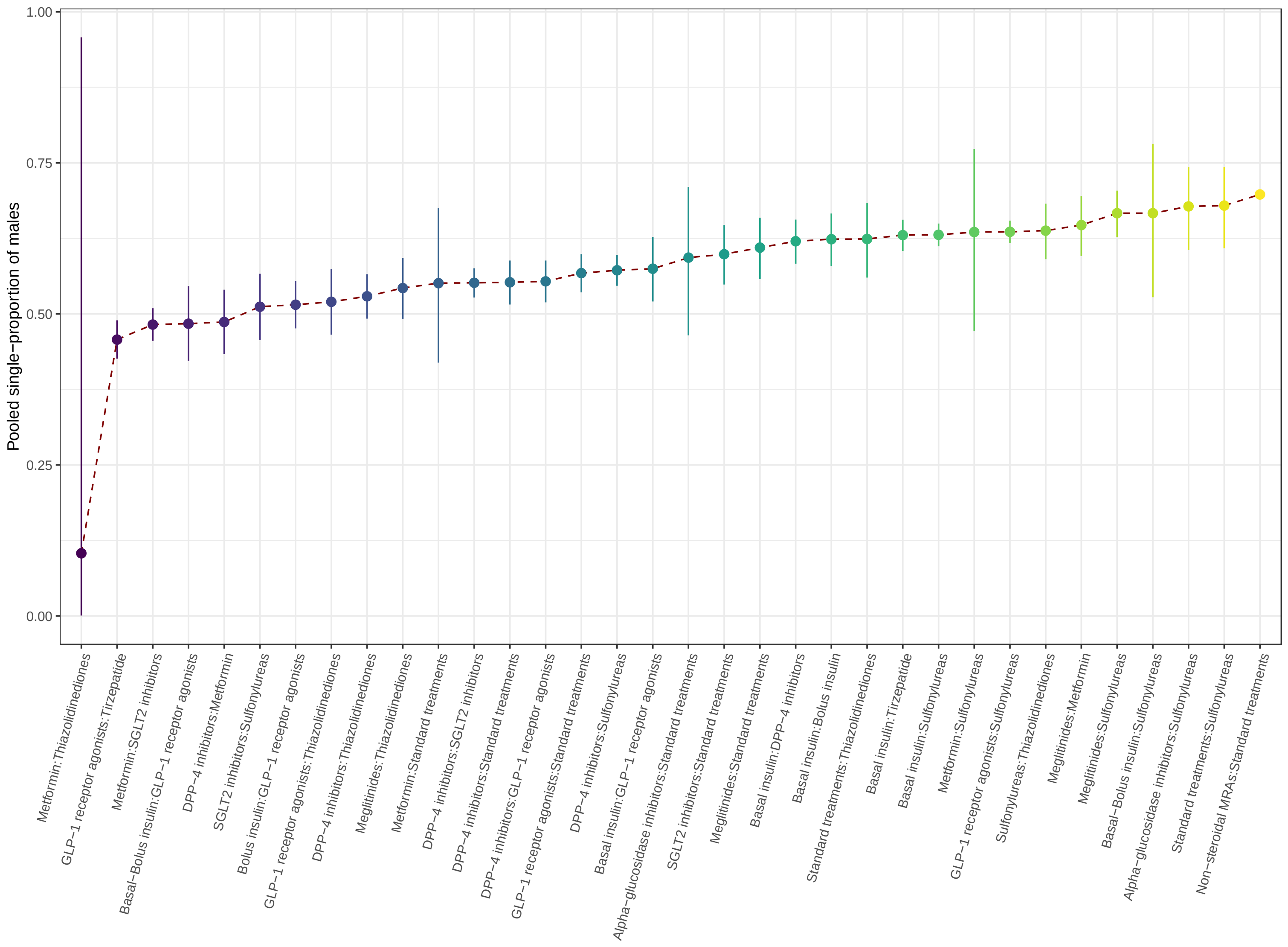

4.5.3 Non-fatal myocardial infarction

4.5.3.1 Age

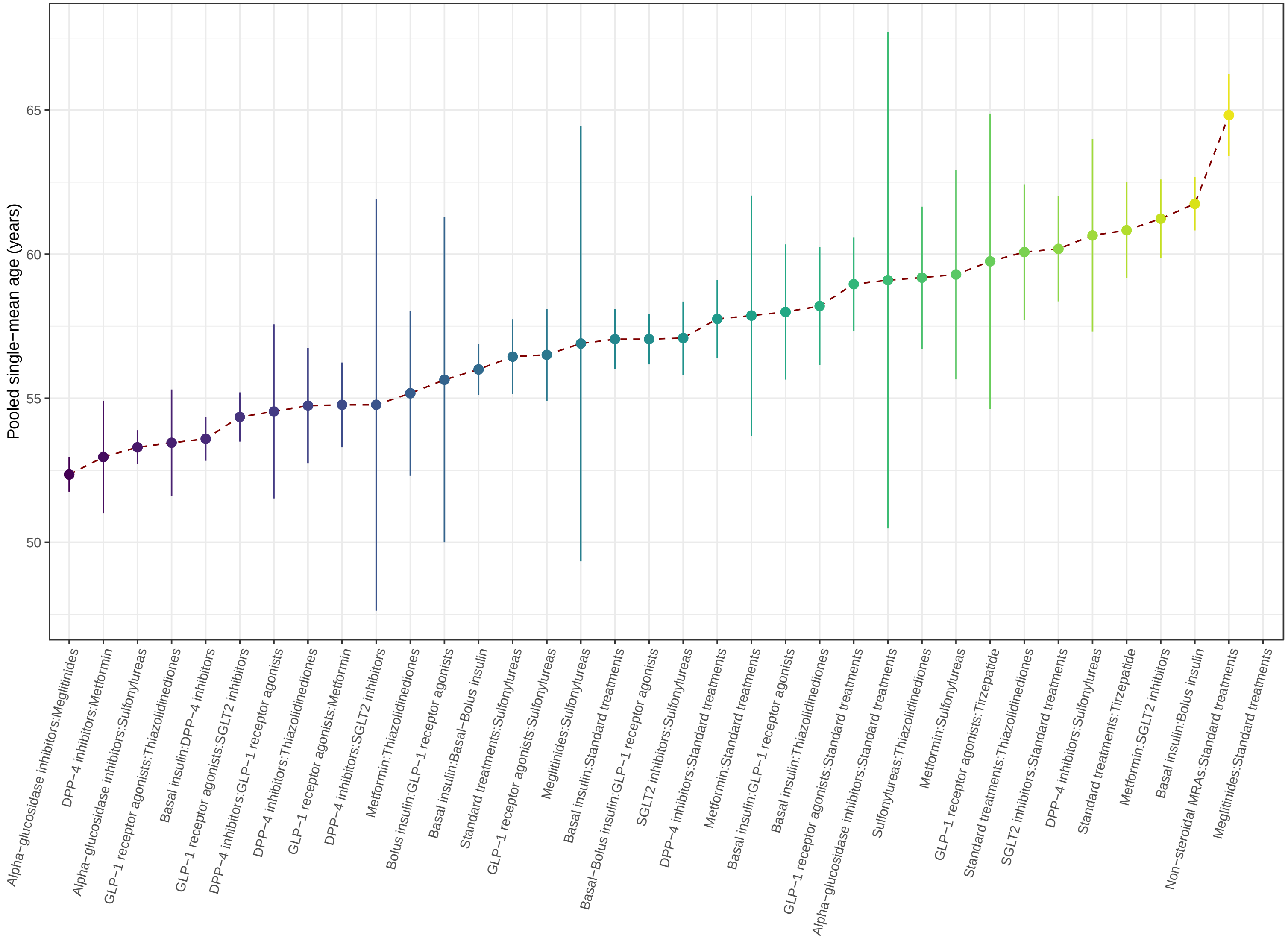

4.5.3.2 Body mass index

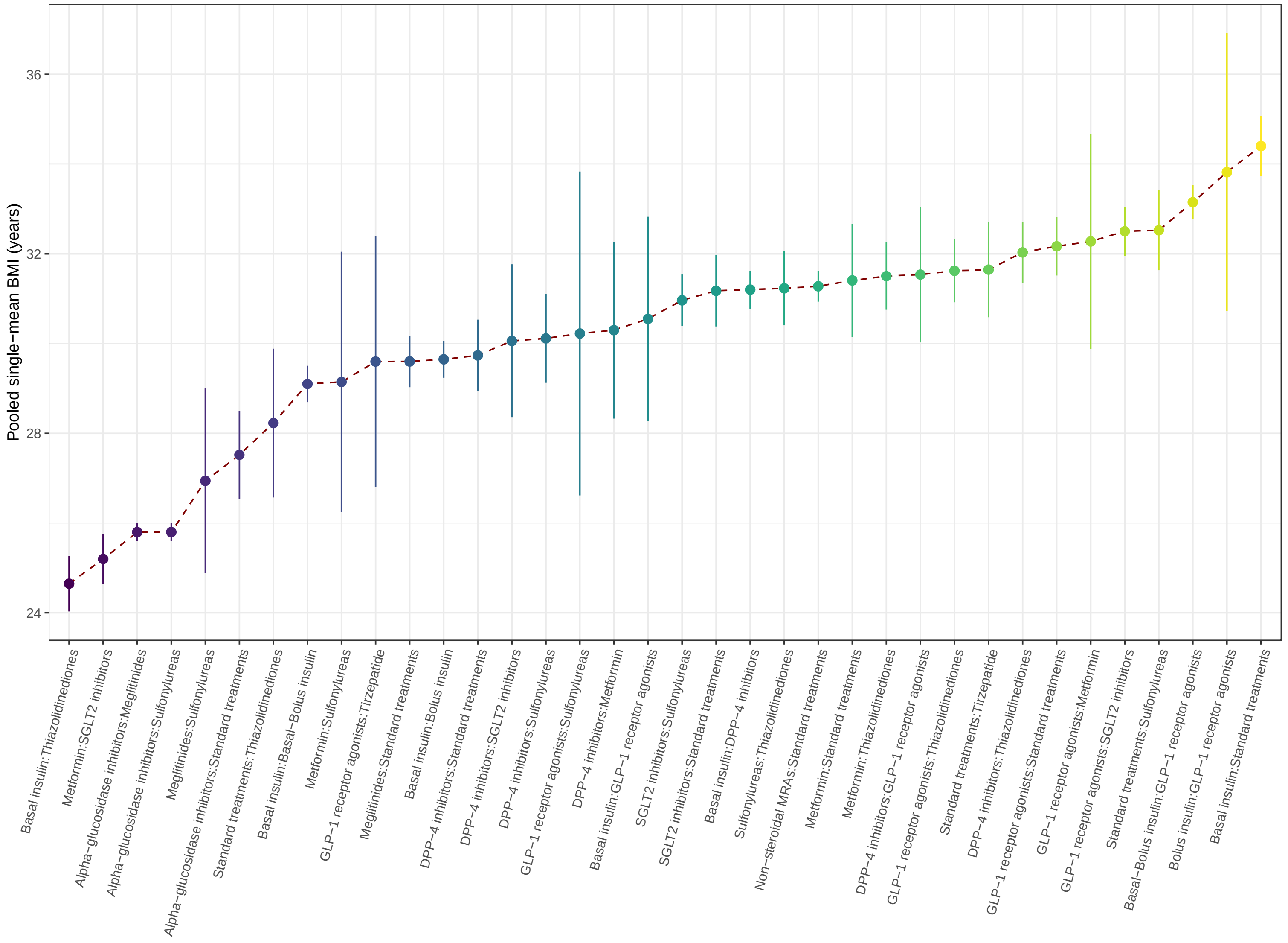

4.5.3.3 Proportion of cardiovascular disease

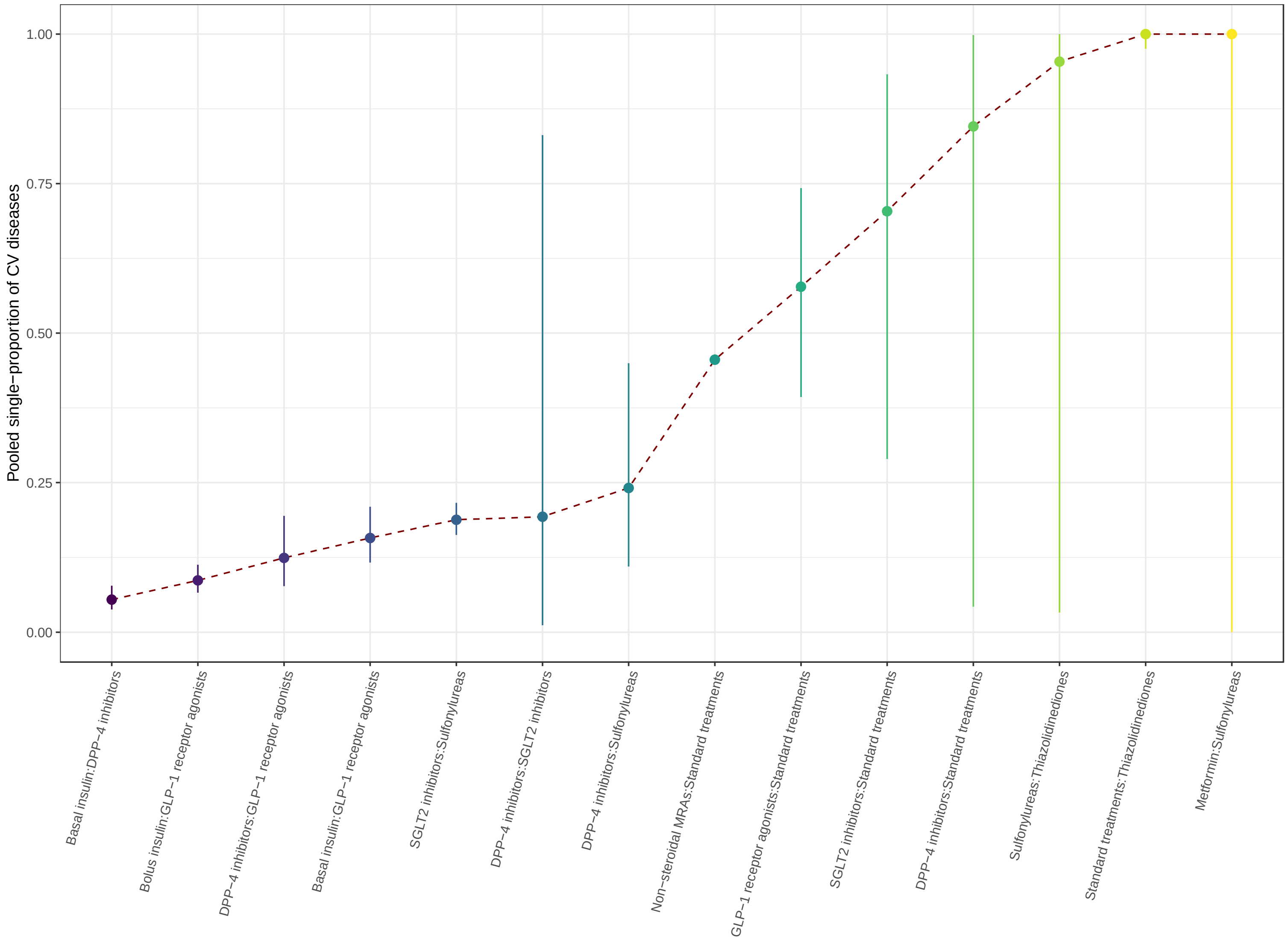

4.5.3.4 Duration of baseline diabetes

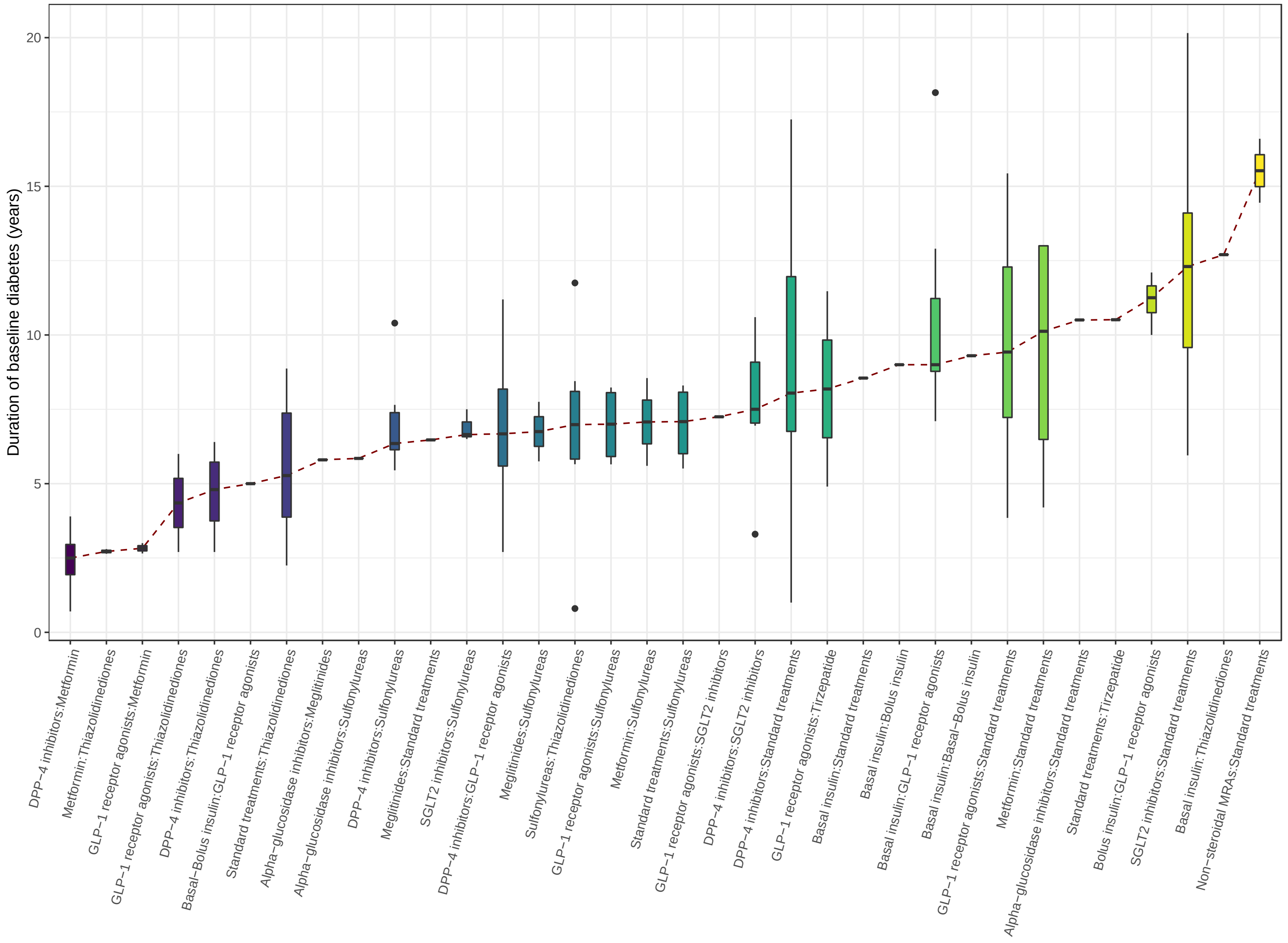

4.5.3.5 HbA1c

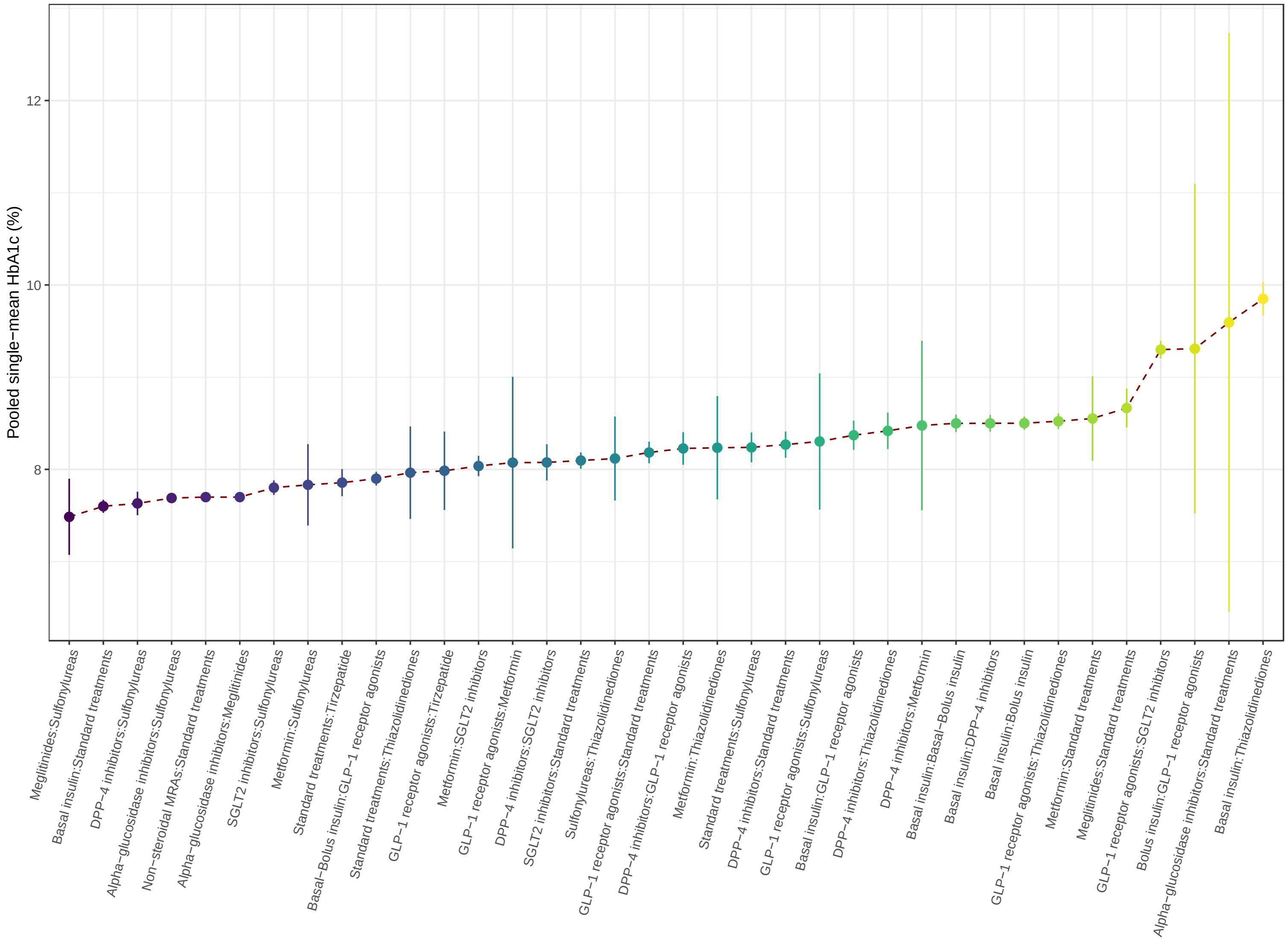

Pooled single−mean HbA1c (%)
12
10
8
Meglitinides:Sulfonylureas
Basal insulin:Standard treatments
DPP−4 inhibitors:Sulfonylureas
Alpha−glucosidase inhibitors:Sulfonylureas
Non−steroidal MRAs:Standard treatments
Alpha−glucosidase inhibitors:Meglitinides
SGLT2 inhibitors:Sulfonylureas
Metformin:Sulfonylureas
Standard treatments:Tirzepatide
Basal−Bolus insulin:GLP−1 receptor agonists
Standard treatments:Thiazolidinediones
GLP−1 receptor agonists:Tirzepatide
Metformin:SGLT2 inhibitors
GLP−1 receptor agonists:Metformin
DPP−4 inhibitors:SGLT2 inhibitors
SGLT2 inhibitors:Standard treatments
Sulfonylureas:Thiazolidinediones
GLP−1 receptor agonists:Standard treatments
DPP−4 inhibitors:GLP−1 receptor agonists
Metformin:Thiazolidinediones
Standard treatments:Sulfonylureas
DPP−4 inhibitors:Standard treatments
GLP−1 receptor agonists:Sulfonylureas
Basal insulin:GLP−1 receptor agonists
DPP−4 inhibitors:Thiazolidinediones
DPP−4 inhibitors:Metformin
Basal insulin:Basal−Bolus insulin
Basal insulin:DPP−4 inhibitors
Basal insulin:Bolus insulin
GLP−1 receptor agonists:Thiazolidinediones
Metformin:Standard treatments
Meglitinides:Standard treatments
GLP−1 receptor agonists:SGLT2 inhibitors
Bolus insulin:GLP−1 receptor agonists
Alpha−glucosidase inhibitors:Standard treatments
Basal insulin:Thiazolidinediones

4.5.3.6 Proportion of males

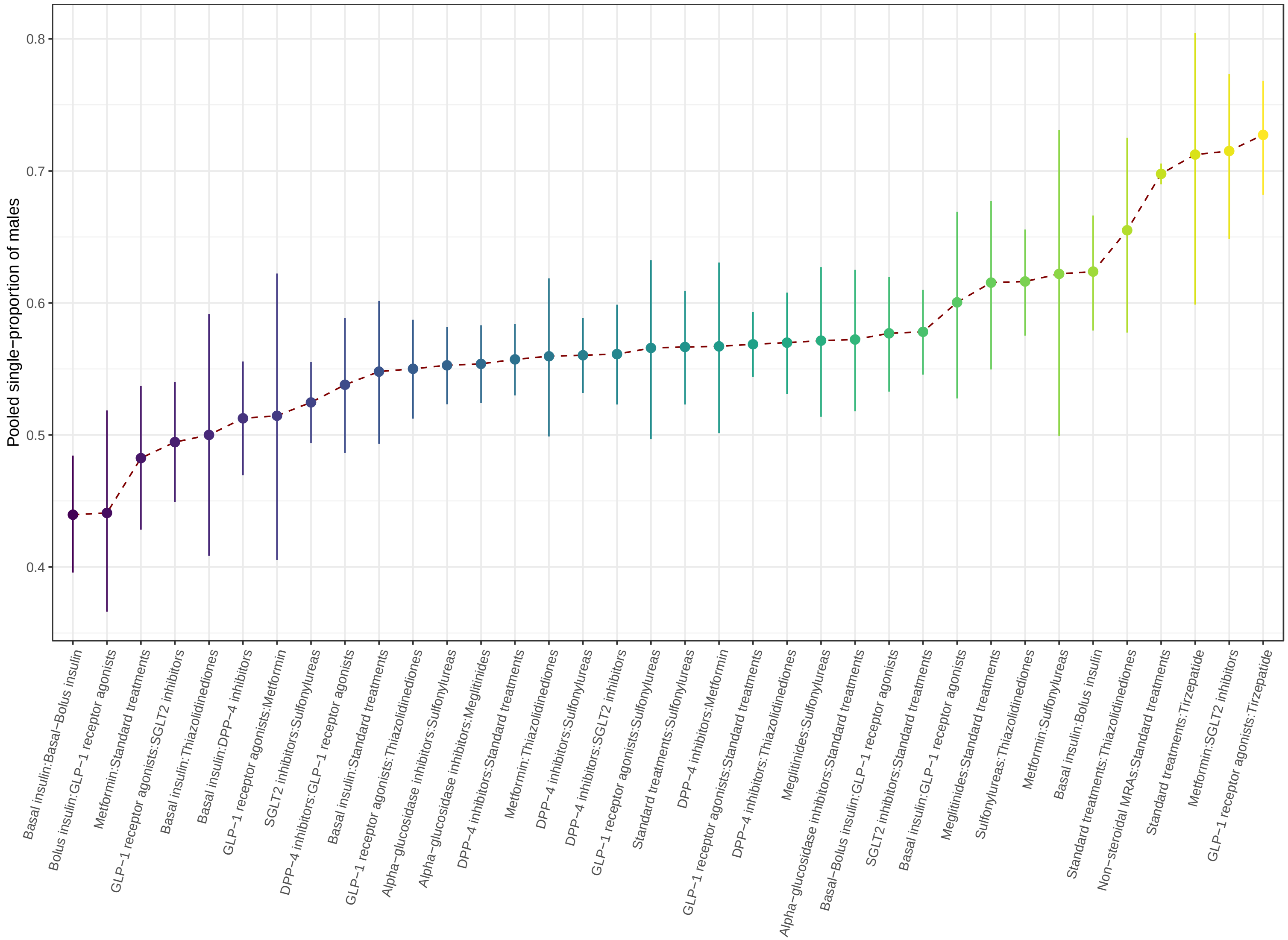

4.5.4 Non-fatal stroke

4.5.4.1 Age

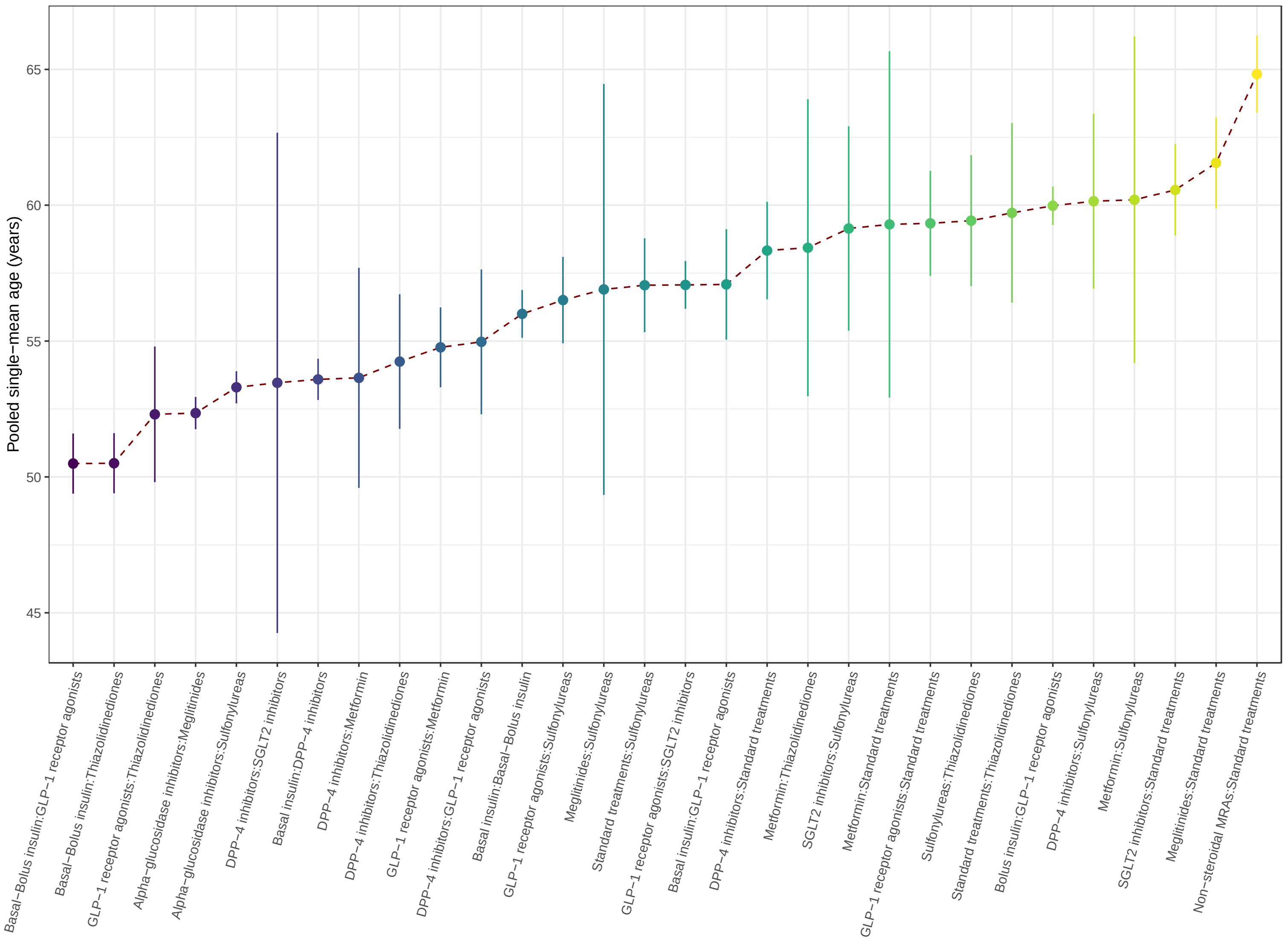

4.5.4.2 Body mass index

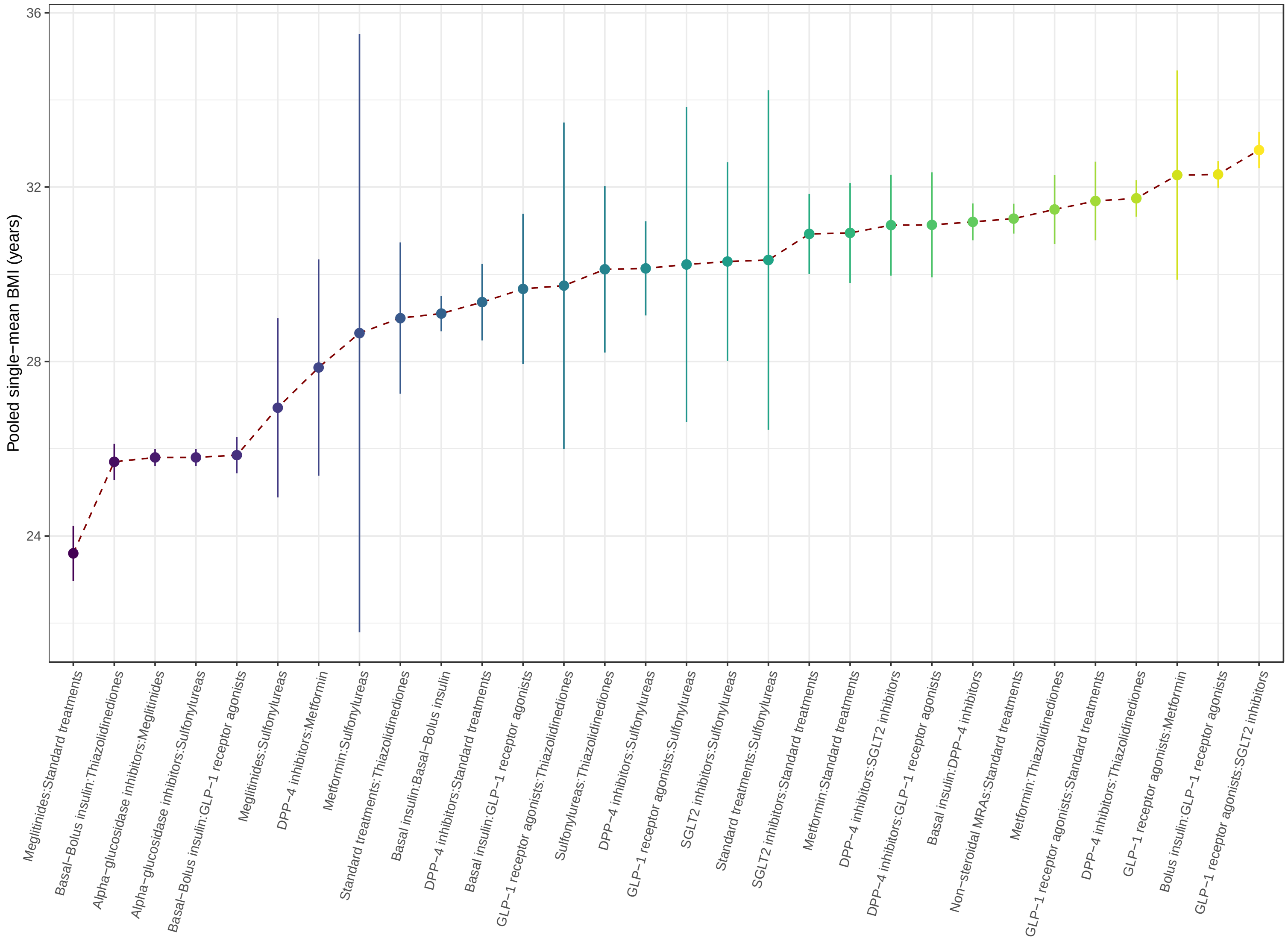

4.5.4.3 Proportion of cardiovascular disease

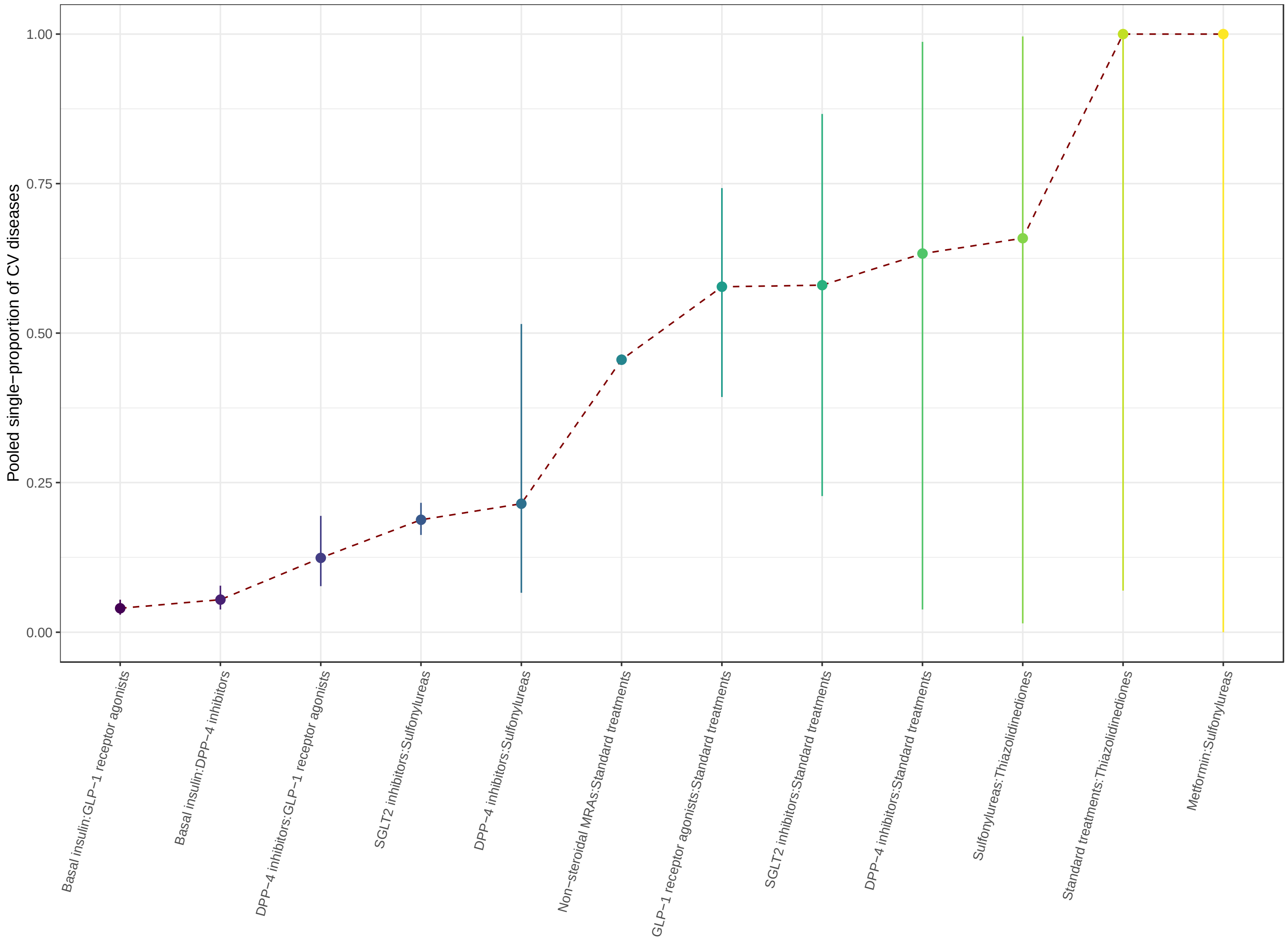

4.5.4.4 Duration of baseline diabetes

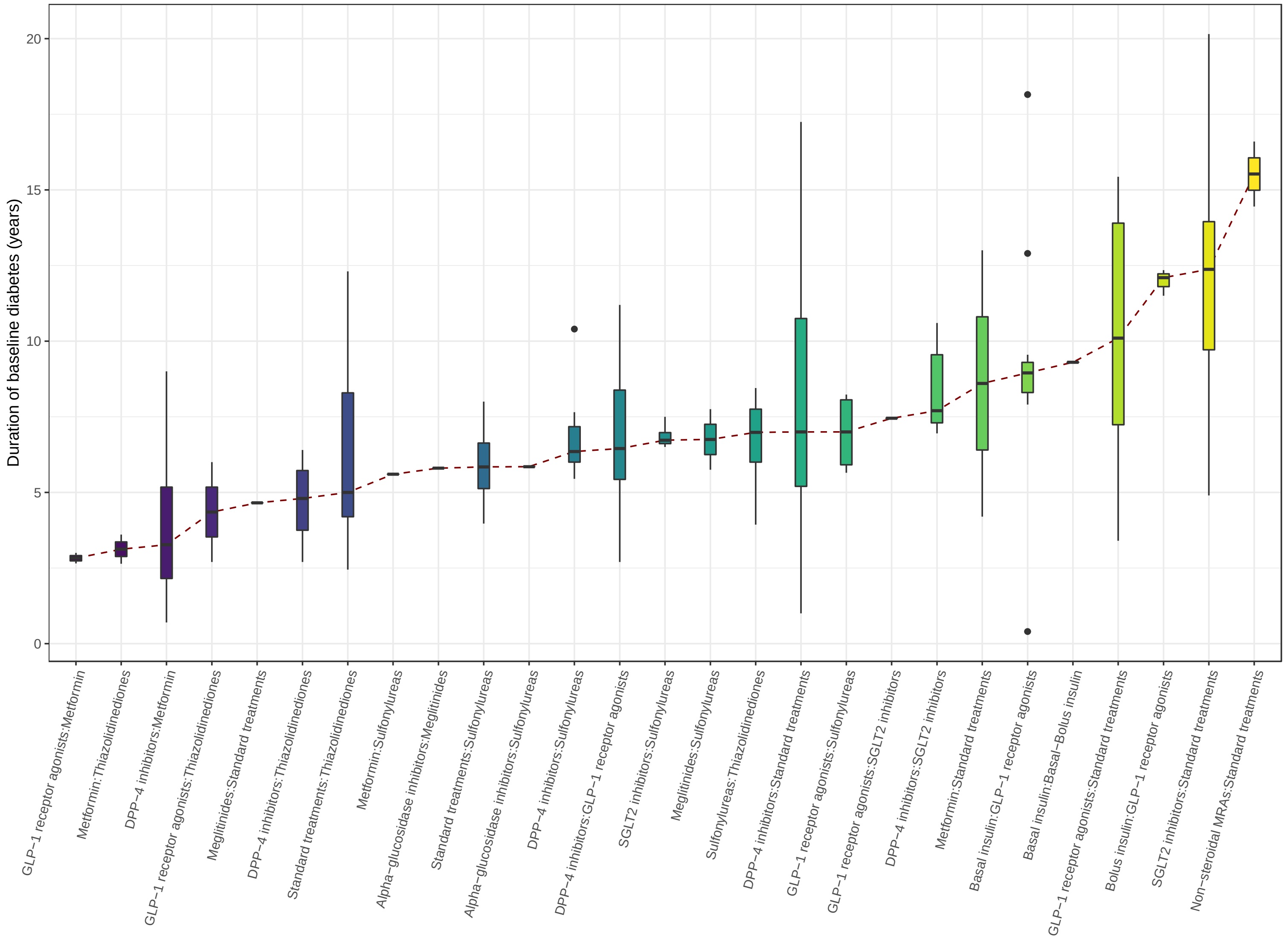

4.5.4.5 HbA1c

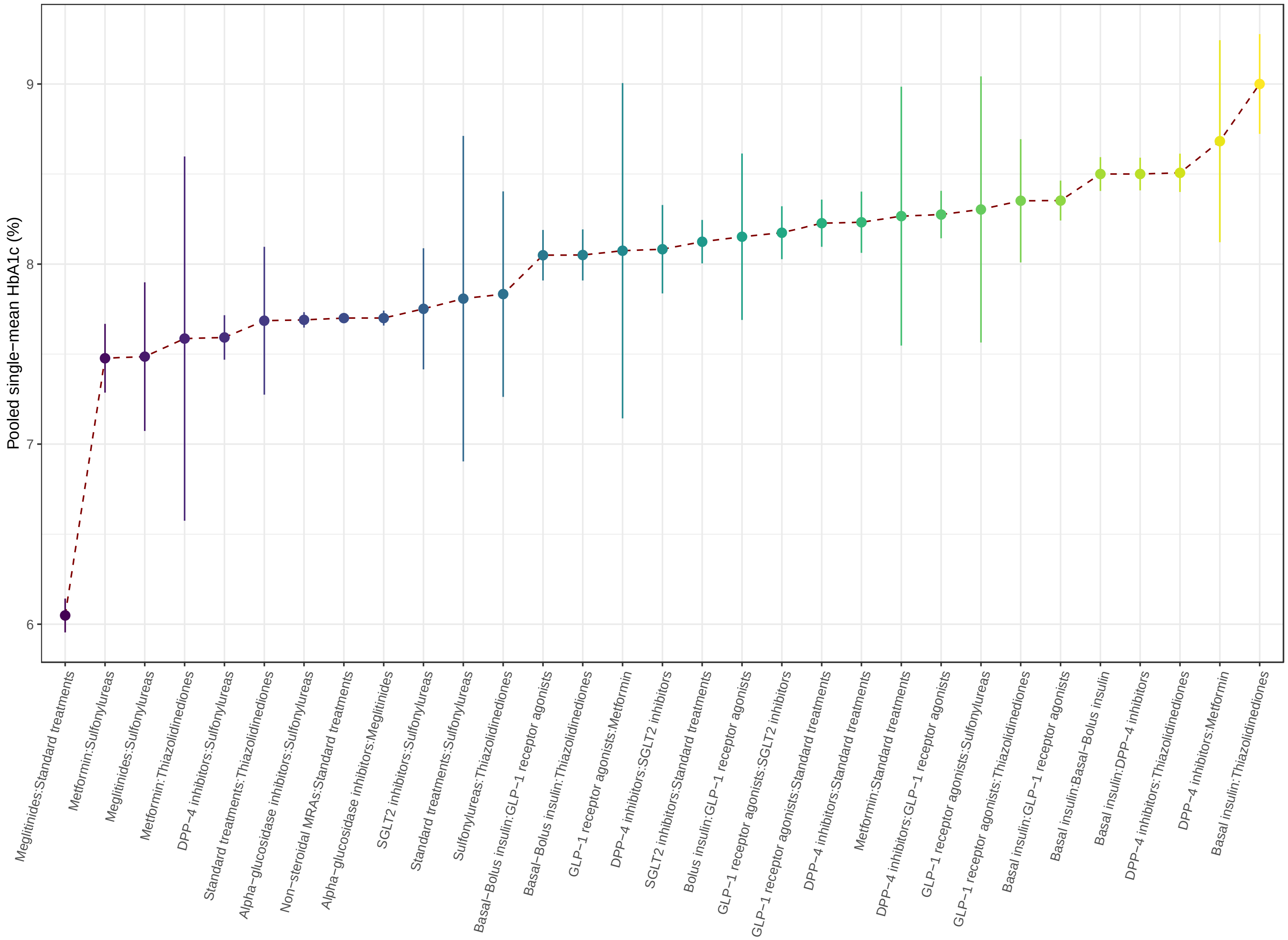
Pooled single−mean HbA1c (%)
9
8
7
6
Meglitinides:Standard treatments
Metformin:Sulfonylureas
Meglitinides:Sulfonylureas
Metformin:Thiazolidinediones
DPP−4 inhibitors:Sulfonylureas
Standard treatments:Thiazolidinediones
Alpha−glucosidase inhibitors:Sulfonylureas
Non−steroidal MRAs:Standard treatments
Alpha−glucosidase inhibitors:Meglitinides
SGLT2 inhibitors:Sulfonylureas
Standard treatments:Sulfonylureas
Sulfonylureas:Thiazolidinediones
Basal−Bolus insulin:GLP−1 receptor agonists
Basal−Bolus insulin:Thiazolidinediones
GLP−1 receptor agonists:Metformin
DPP−4 inhibitors:SGLT2 inhibitors
SGLT2 inhibitors:Standard treatments
Bolus insulin:GLP−1 receptor agonists
GLP−1 receptor agonists:SGLT2 inhibitors
GLP−1 receptor agonists:Standard treatments
DPP−4 inhibitors:Standard treatments
Metformin:Standard treatments
DPP−4 inhibitors:GLP−1 receptor agonists
GLP−1 receptor agonists:Sulfonylureas
GLP−1 receptor agonists:Thiazolidinediones
Basal insulin:GLP−1 receptor agonists
Basal insulin:Basal−Bolus insulin
Basal insulin:DPP−4 inhibitors
DPP−4 inhibitors:Thiazolidinediones
DPP−4 inhibitors:Metformin
Basal insulin:Thiazolidinediones

4.5.4.6 Proportion of males

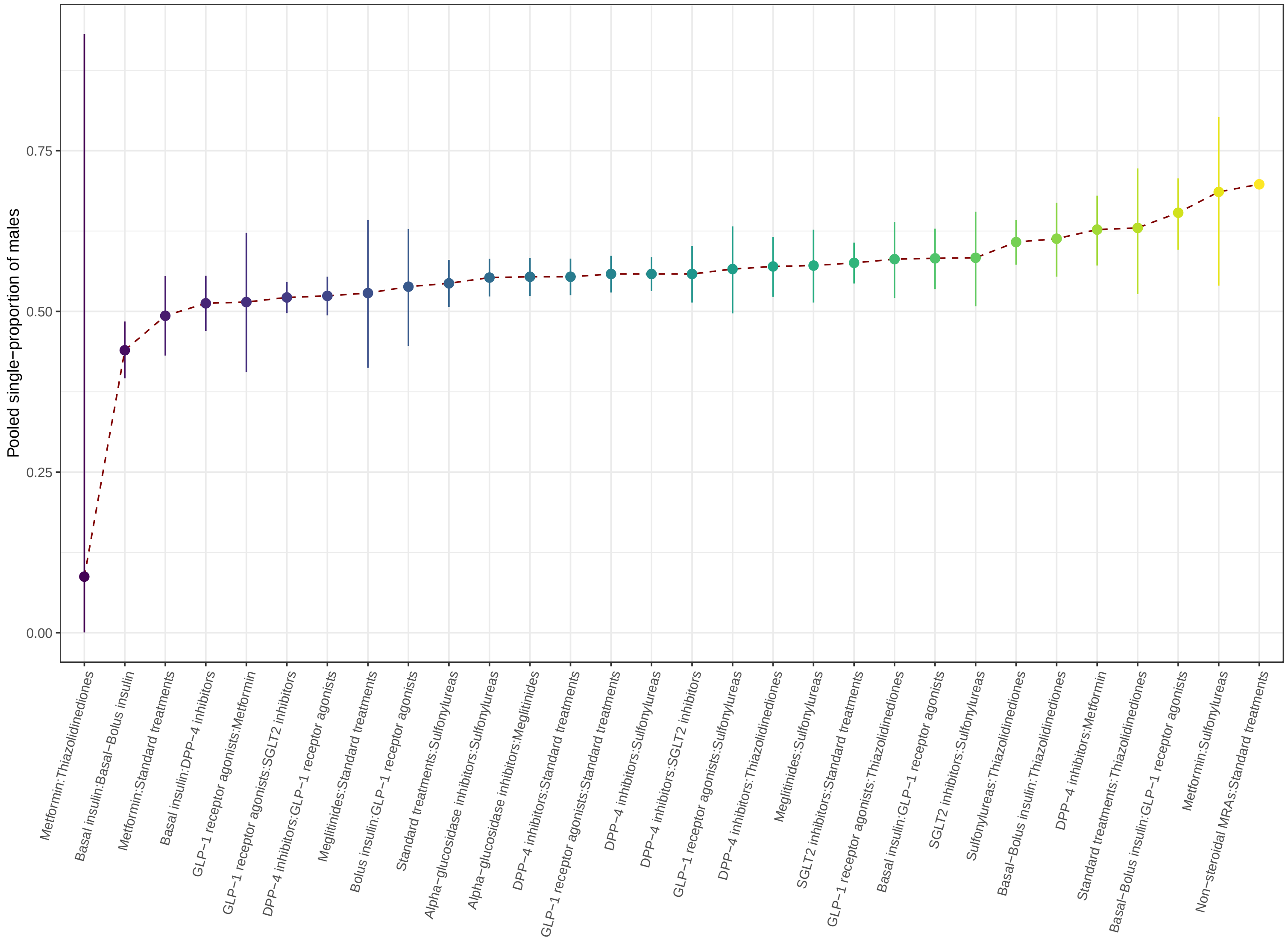

4.5.5 Hospitalization for heart failure

4.5.5.1 Age

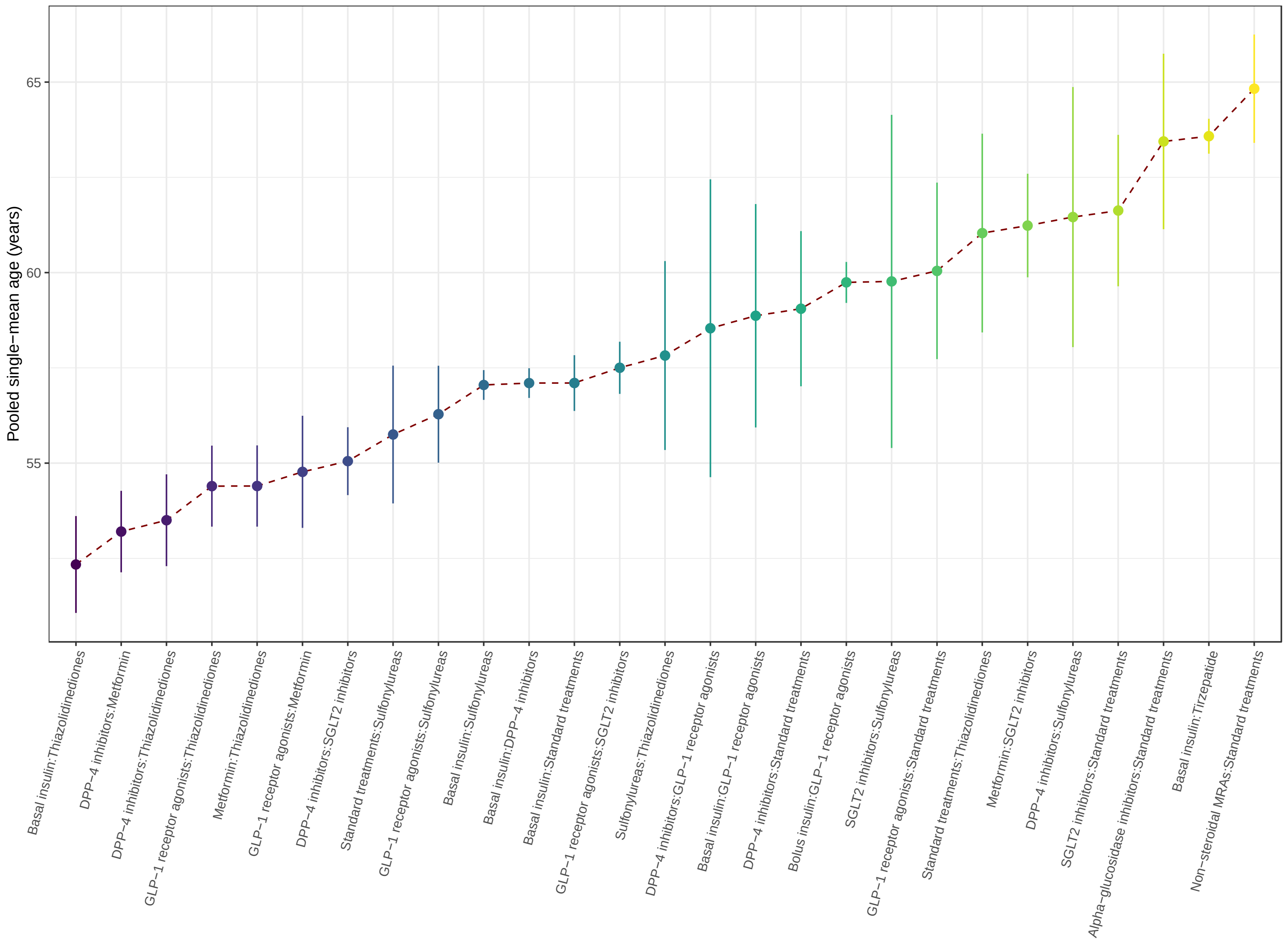

4.5.5.2 Body mass index

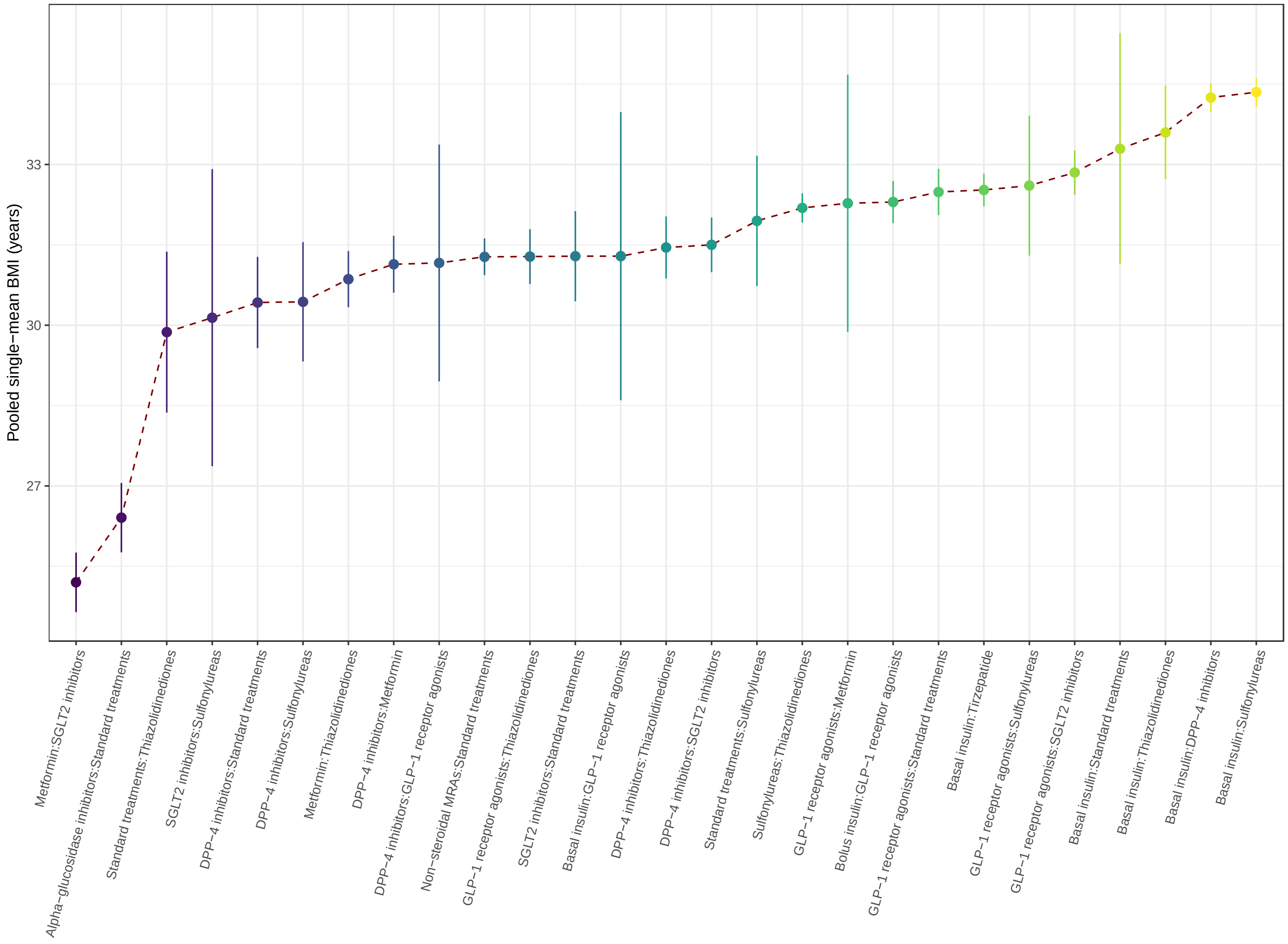

4.5.5.3 Proportion of cardiovascular disease

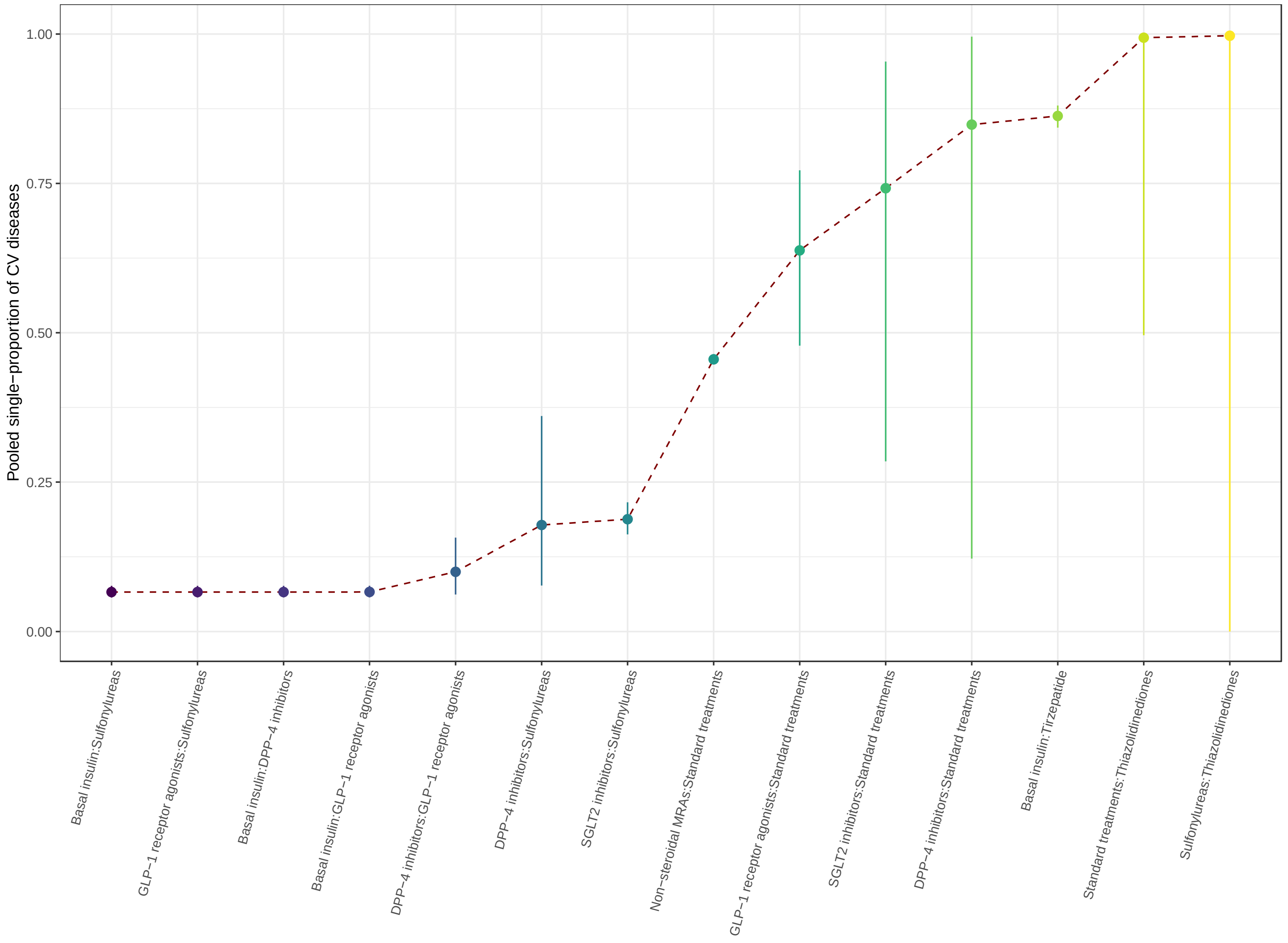

4.5.5.4 Duration of baseline diabetes

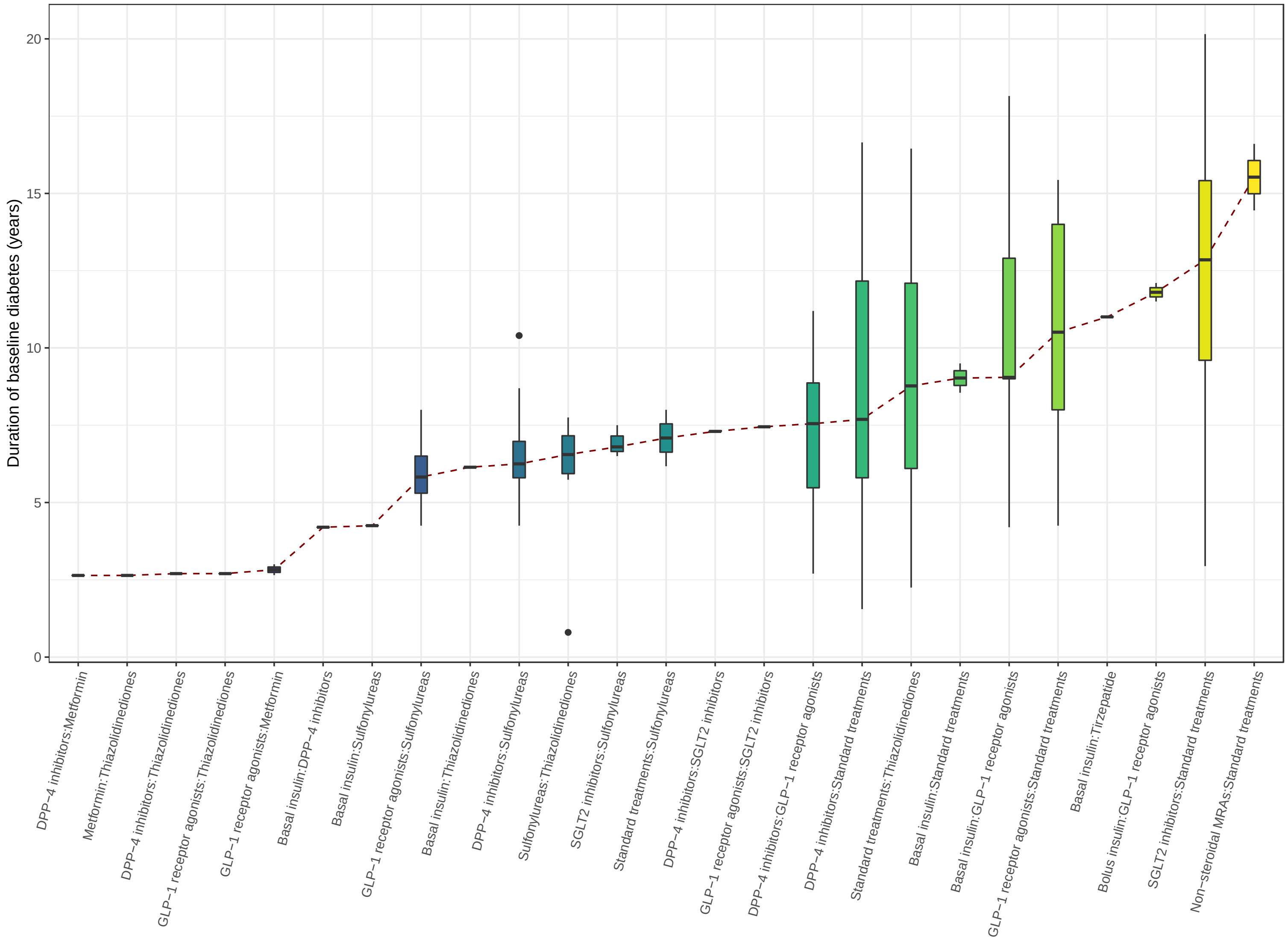

4.5.5.5 HbA1c

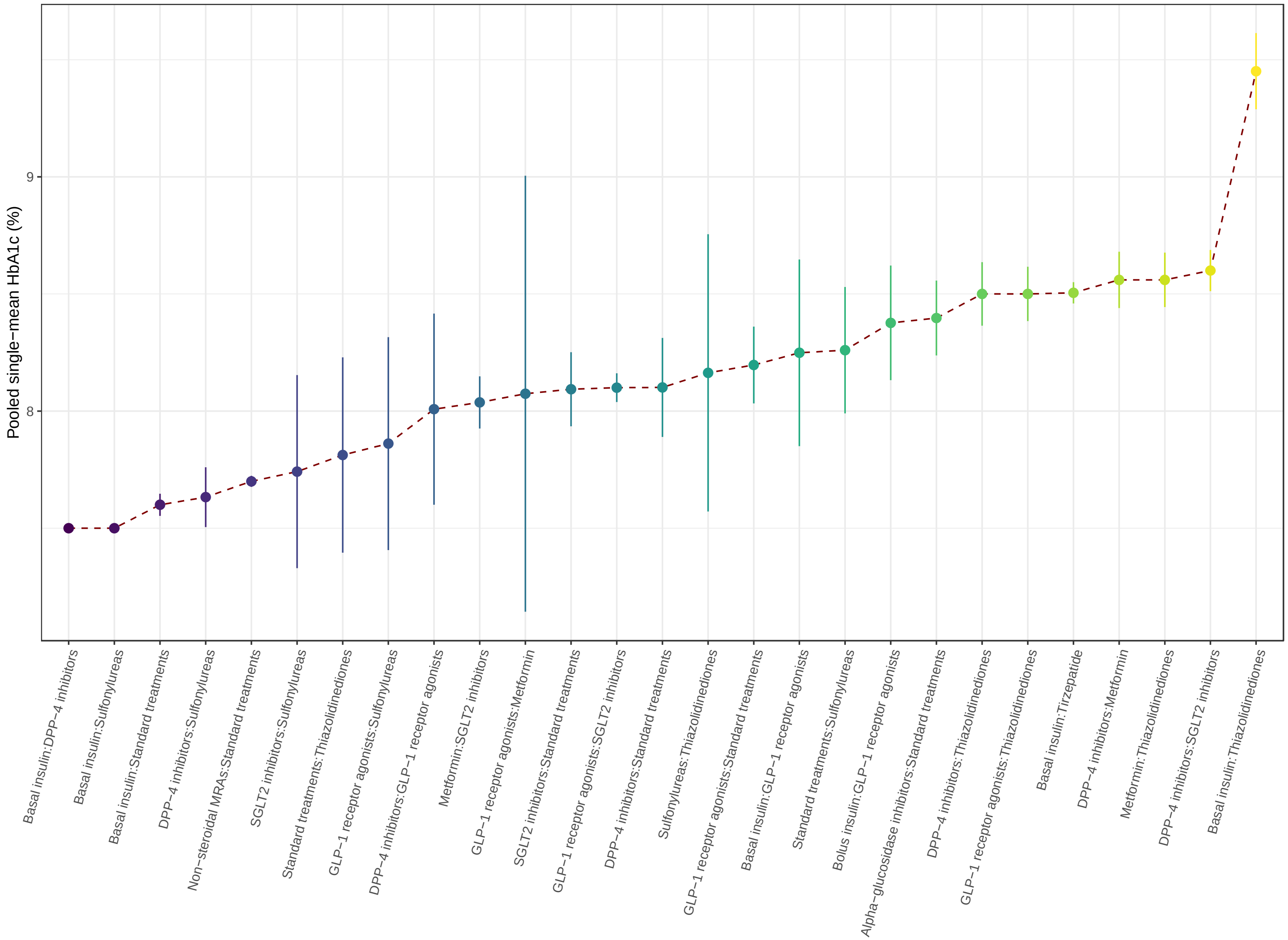

Pooled single−mean HbA1c (%)
9
8
Basal insulin:DPP−4 inhibitors
Basal insulin:Sulfonylureas
Basal insulin:Standard treatments
DPP−4 inhibitors:Sulfonylureas
Non−steroidal MRAs:Standard treatments
SGLT2 inhibitors:Sulfonylureas
Standard treatments:Thiazolidinediones
GLP−1 receptor agonists:Sulfonylureas
DPP−4 inhibitors:GLP−1 receptor agonists
Metformin:SGLT2 inhibitors
GLP−1 receptor agonists:Metformin
SGLT2 inhibitors:Standard treatments
GLP−1 receptor agonists:SGLT2 inhibitors
DPP−4 inhibitors:Standard treatments
Sulfonylureas:Thiazolidinediones
GLP−1 receptor agonists:Standard treatments
Basal insulin:GLP−1 receptor agonists
Standard treatments:Sulfonylureas
Bolus insulin:GLP−1 receptor agonists
Alpha−glucosidase inhibitors:Standard treatments
DPP−4 inhibitors:Thiazolidinediones
GLP−1 receptor agonists:Thiazolidinediones
Basal insulin:Tirzepatide
DPP−4 inhibitors:Metformin
Metformin:Thiazolidinediones
DPP−4 inhibitors:SGLT2 inhibitors
Basal insulin:Thiazolidinediones

4.5.5.6 Proportion of males

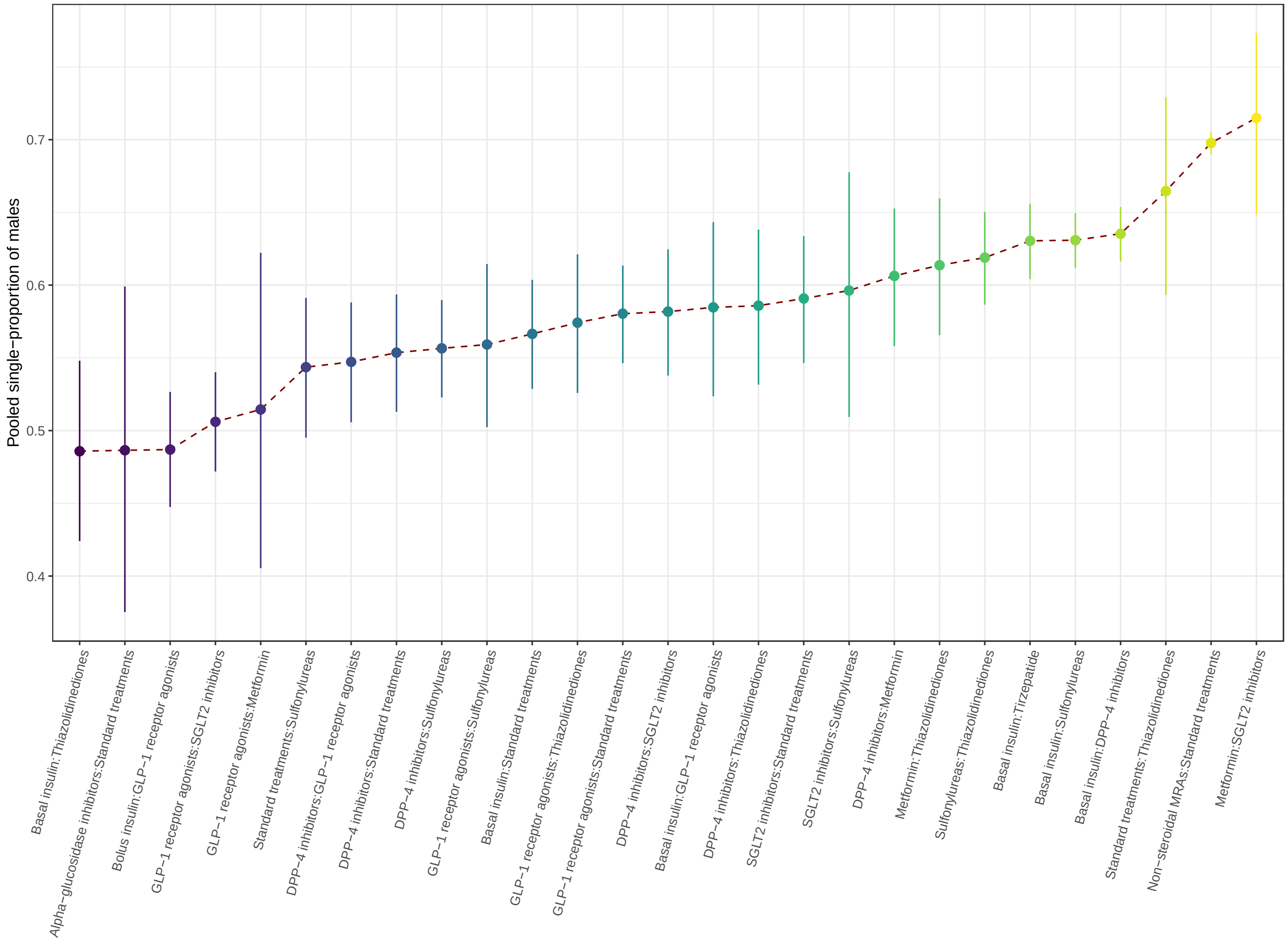

4.5.6 End-stage kidney disease

4.5.6.1 Age

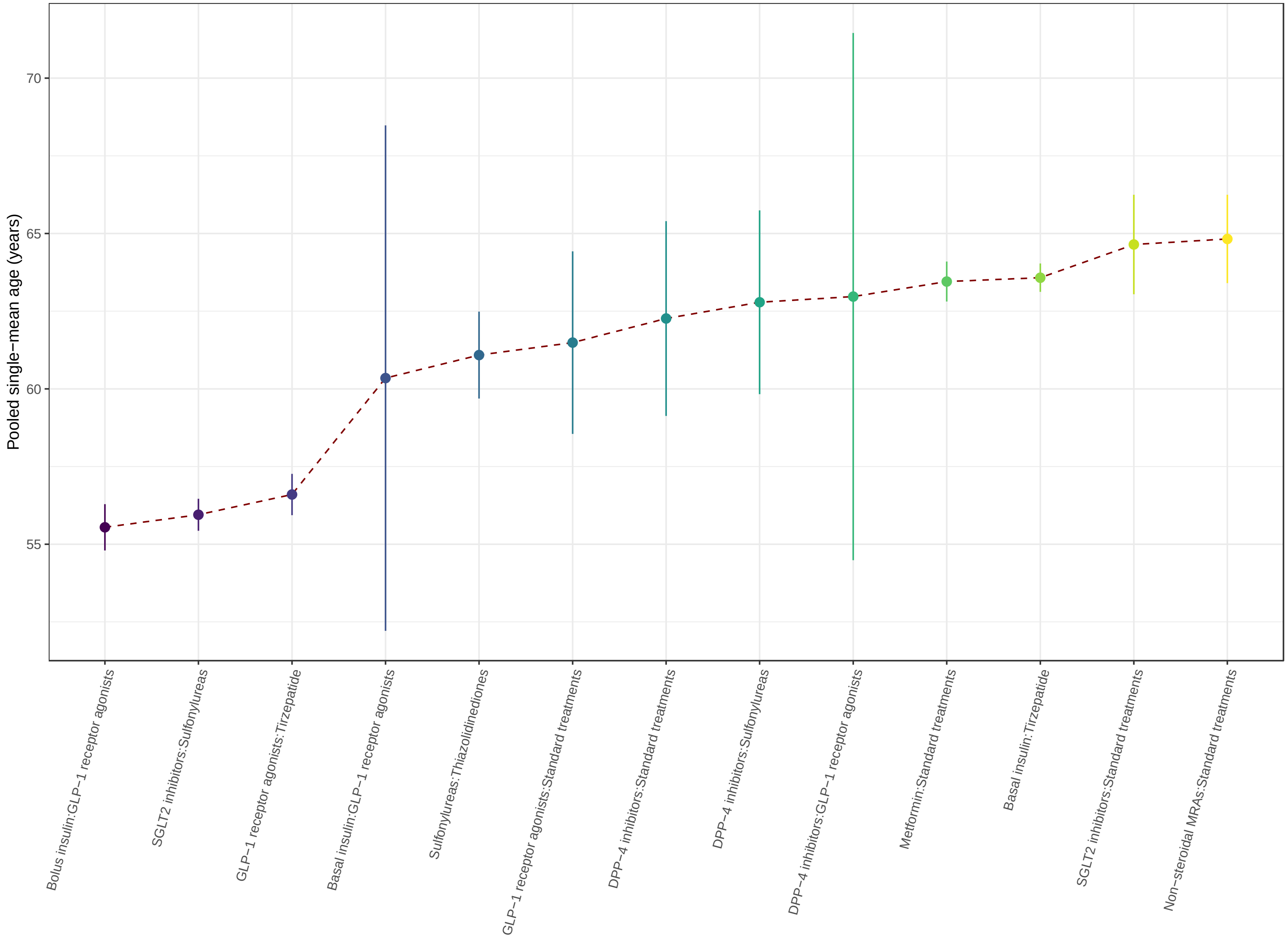

4.5.6.2 Body mass index

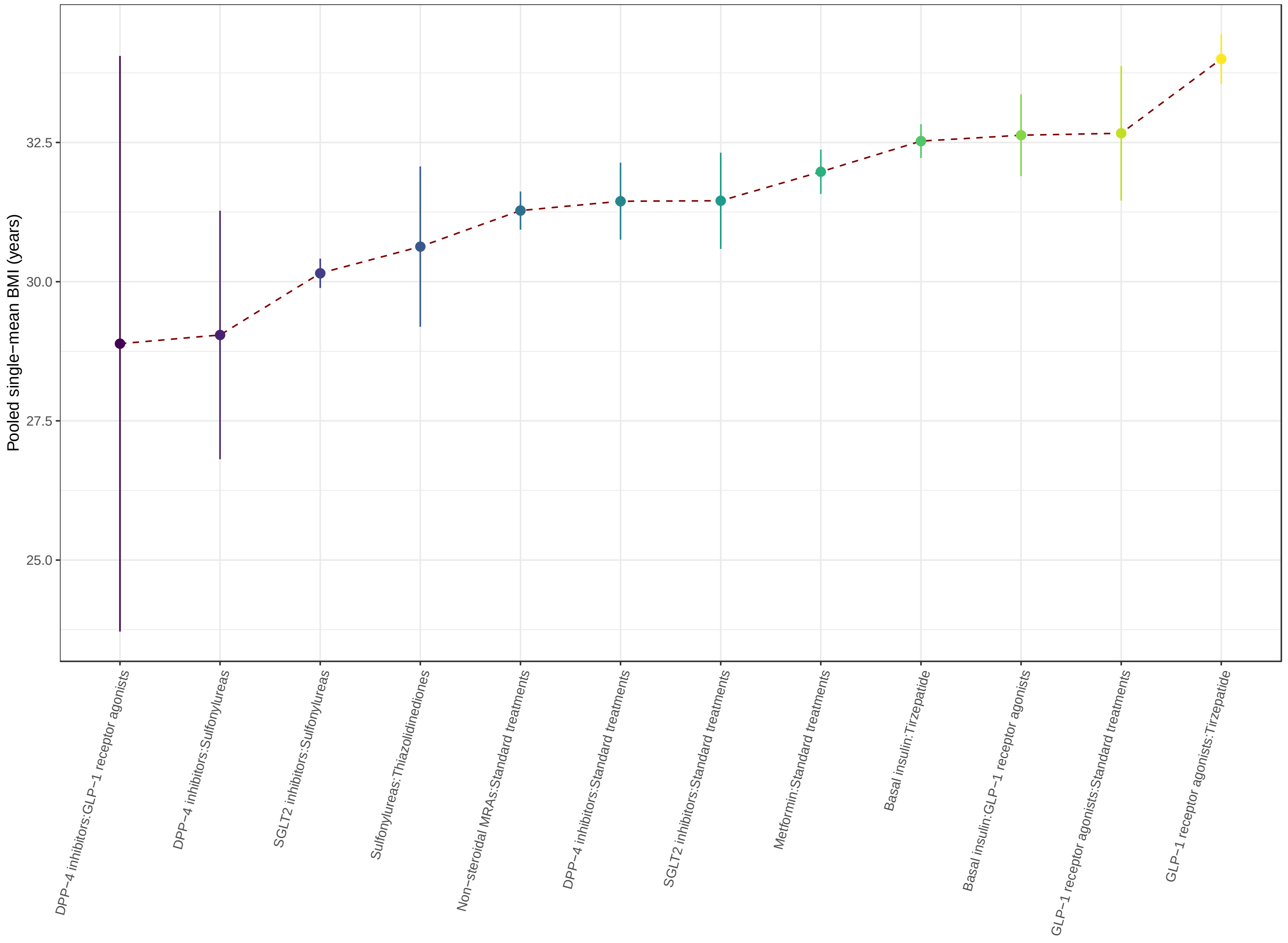

4.5.6.3 Proportion of cardiovascular disease

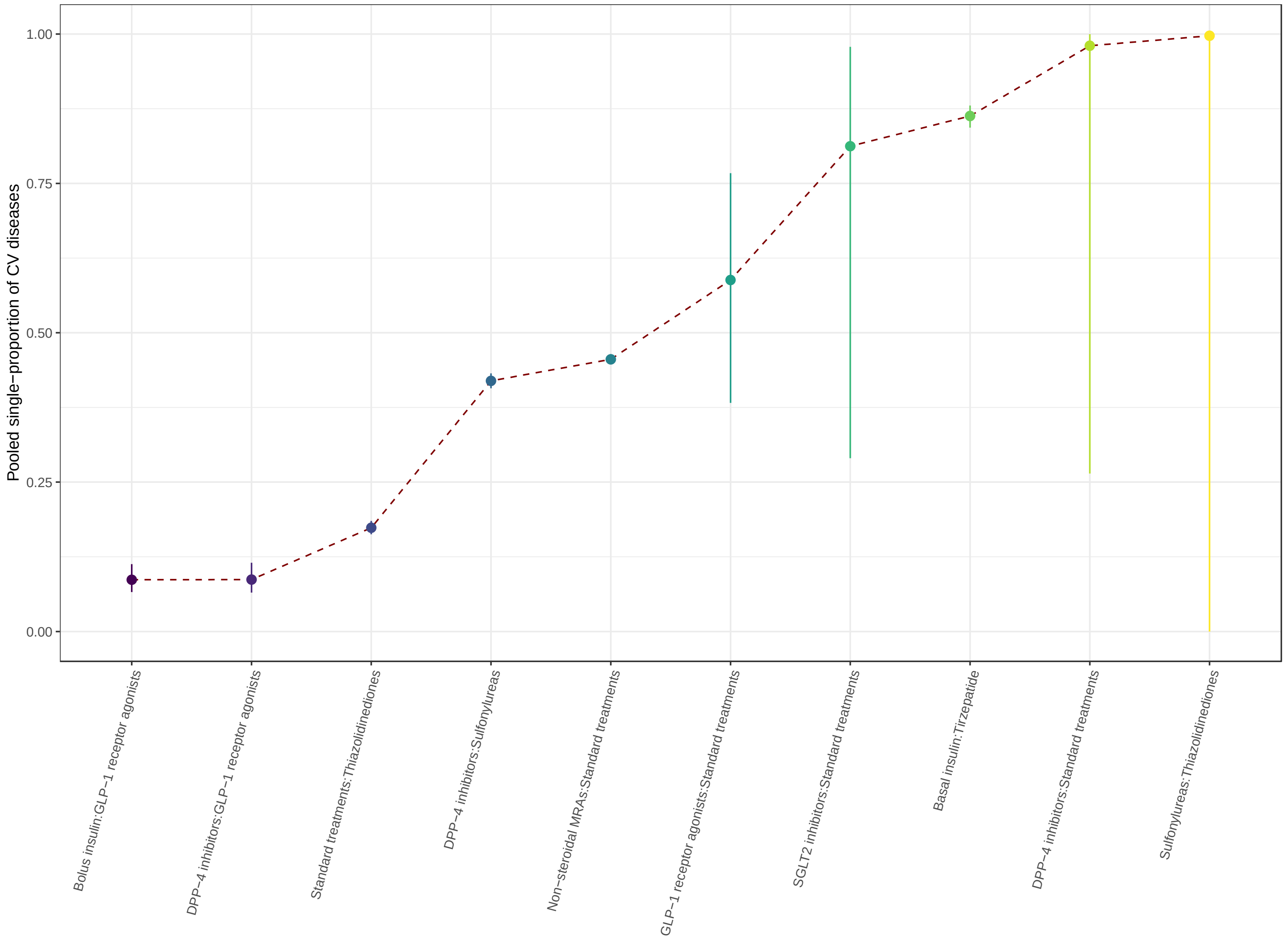

4.5.6.4 Duration of baseline diabetes

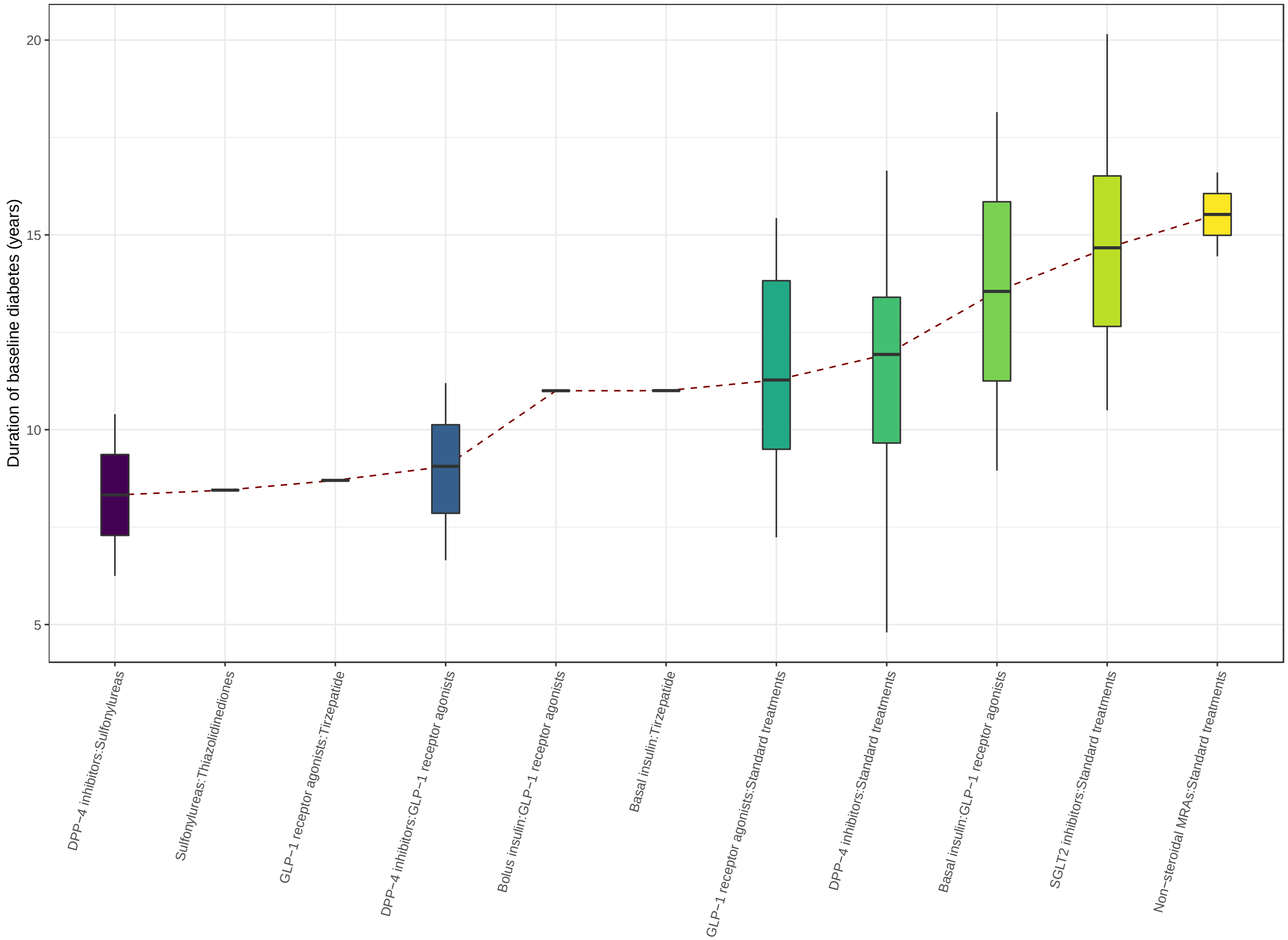

4.5.6.5 HbA1c

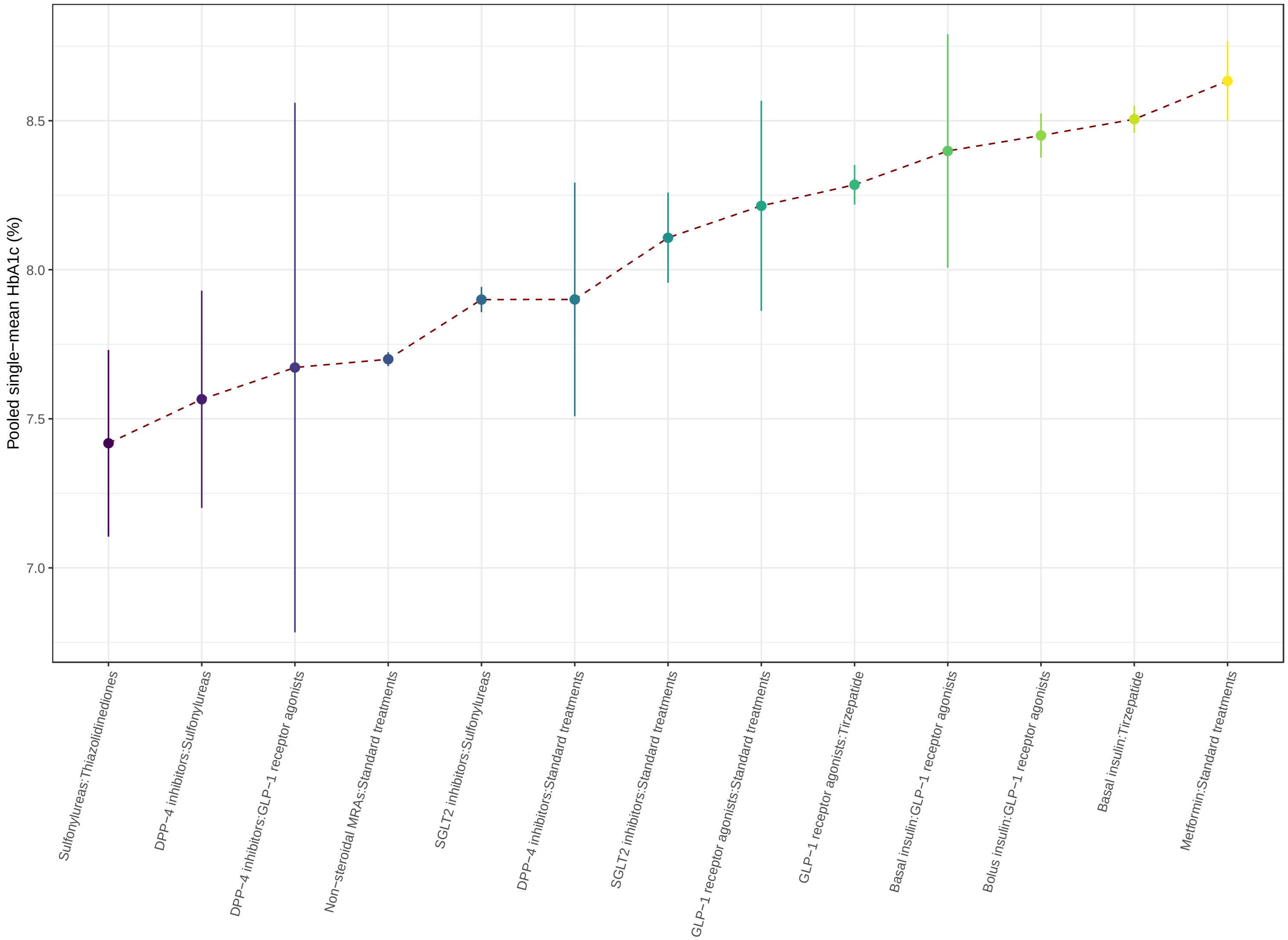

Pooled single−mean HbA1c (%)
8.5
8.0
7.5
7.0
Sulfonylureas:Thiazolidinediones
DPP−4 inhibitors:Sulfonylureas
DPP−4 inhibitors:GLP−1 receptor agonists
Non−steroidal MRAs:Standard treatments
SGLT2 inhibitors:Sulfonylureas
DPP−4 inhibitors:Standard treatments
SGLT2 inhibitors:Standard treatments
GLP−1 receptor agonists:Standard treatments
GLP−1 receptor agonists:Tirzepatide
Basal insulin:GLP−1 receptor agonists
Bolus insulin:GLP−1 receptor agonists
Basal insulin:Tirzepatide
Metformin:Standard treatments

4.5.6.6 Proportion of males

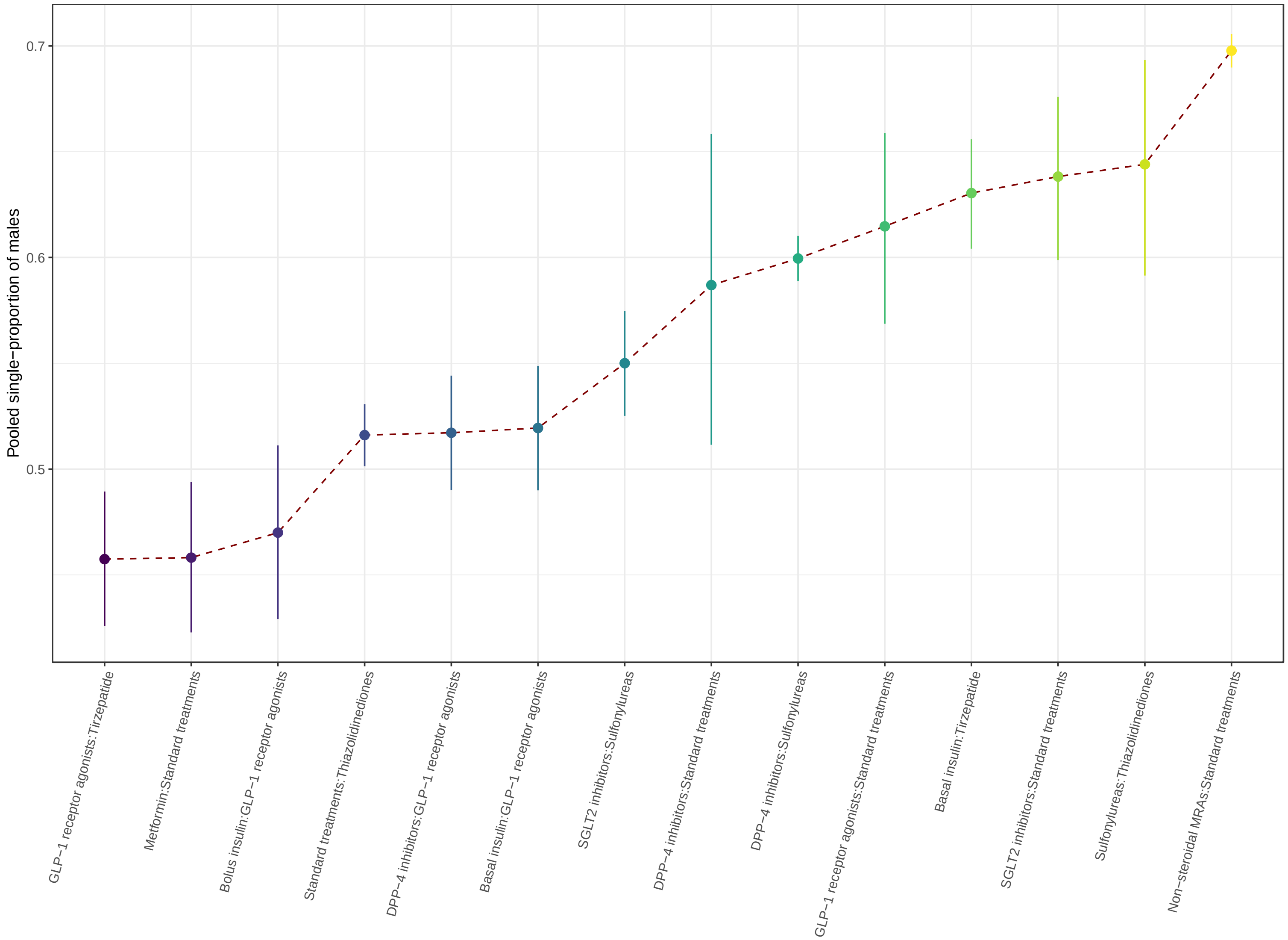

4.5.7 Severe hypoglycaemia

4.5.7.1 Age

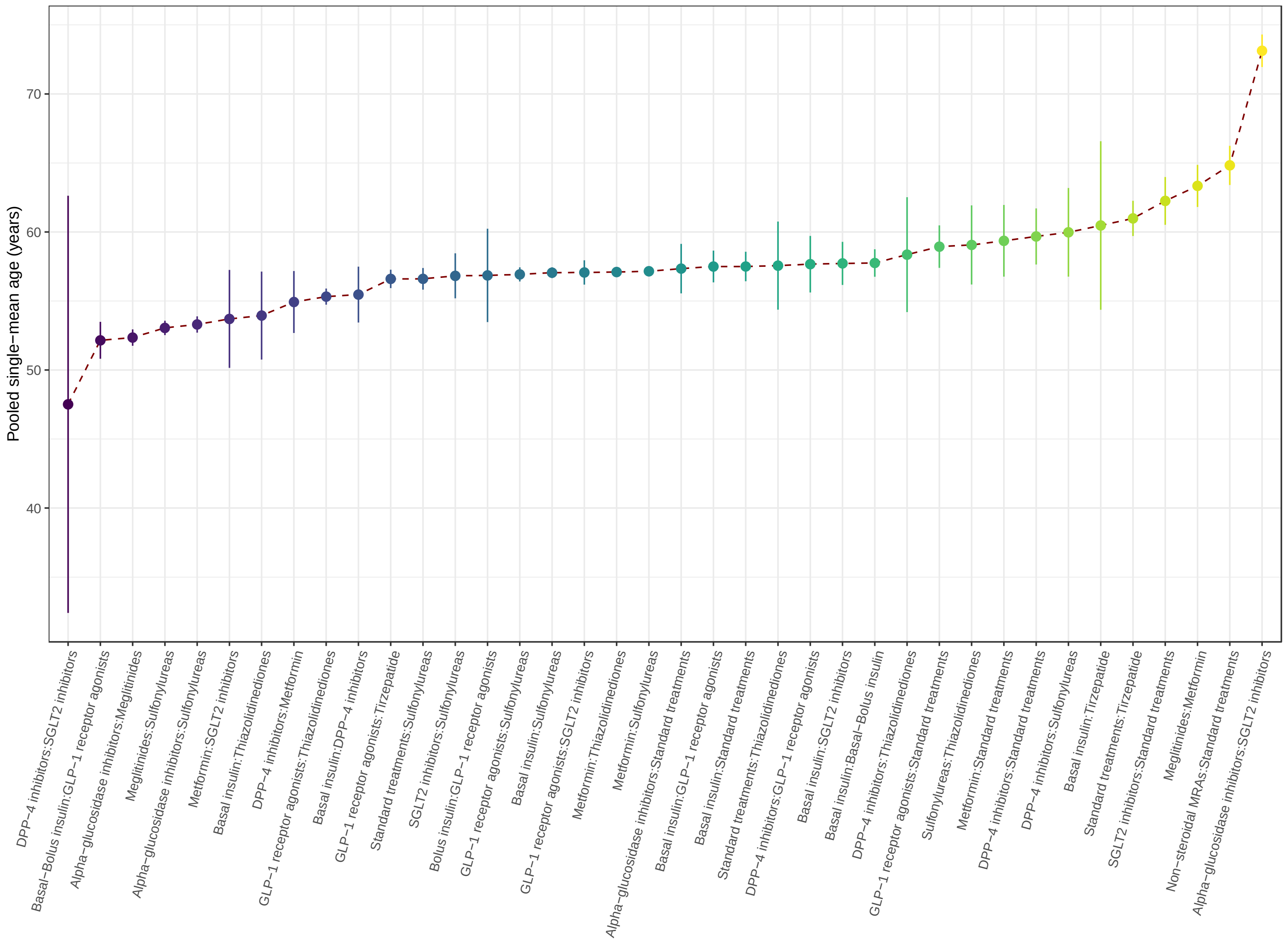

4.5.7.2 Body mass index

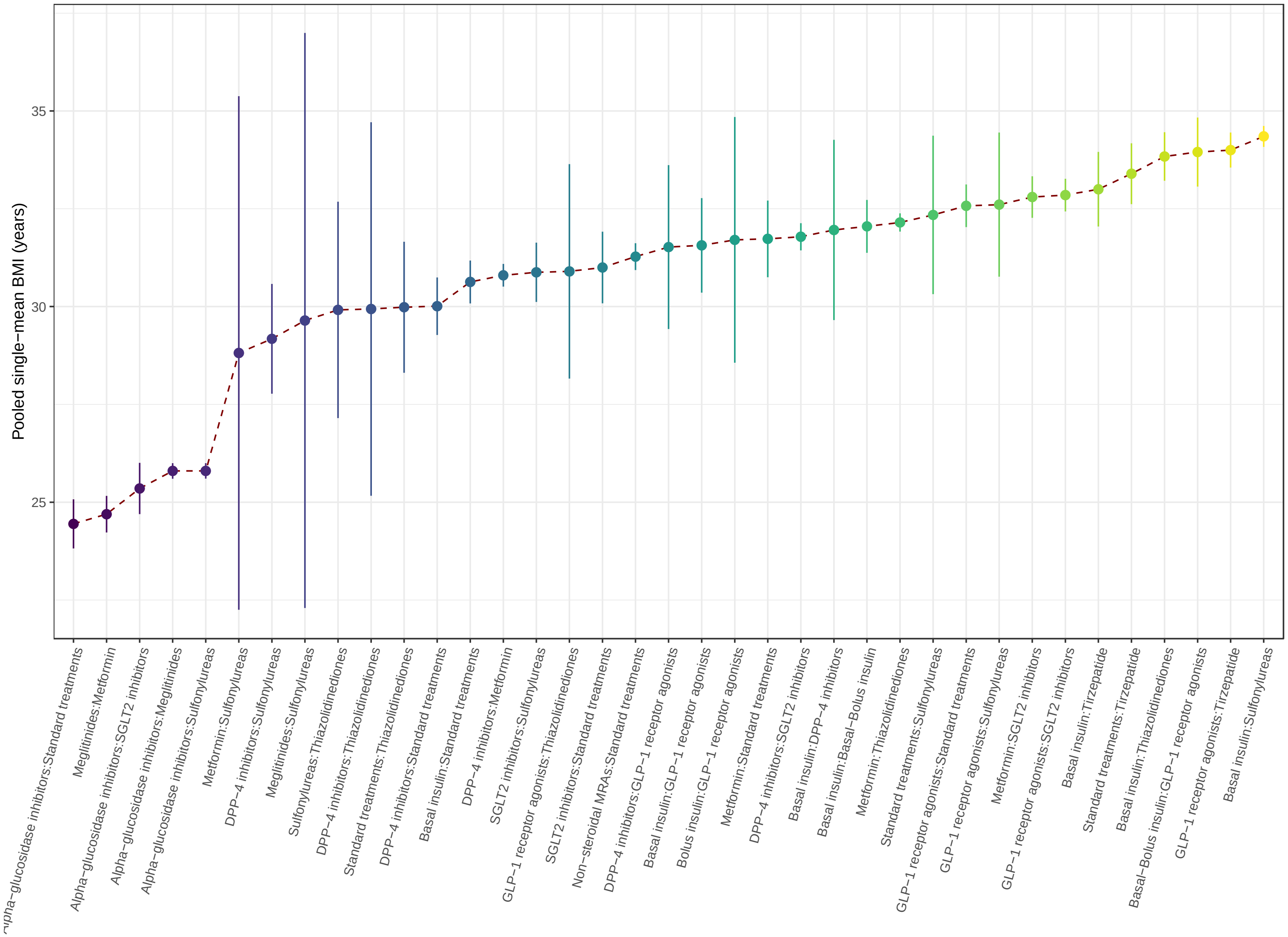

4.5.7.3 Proportion of cardiovascular disease

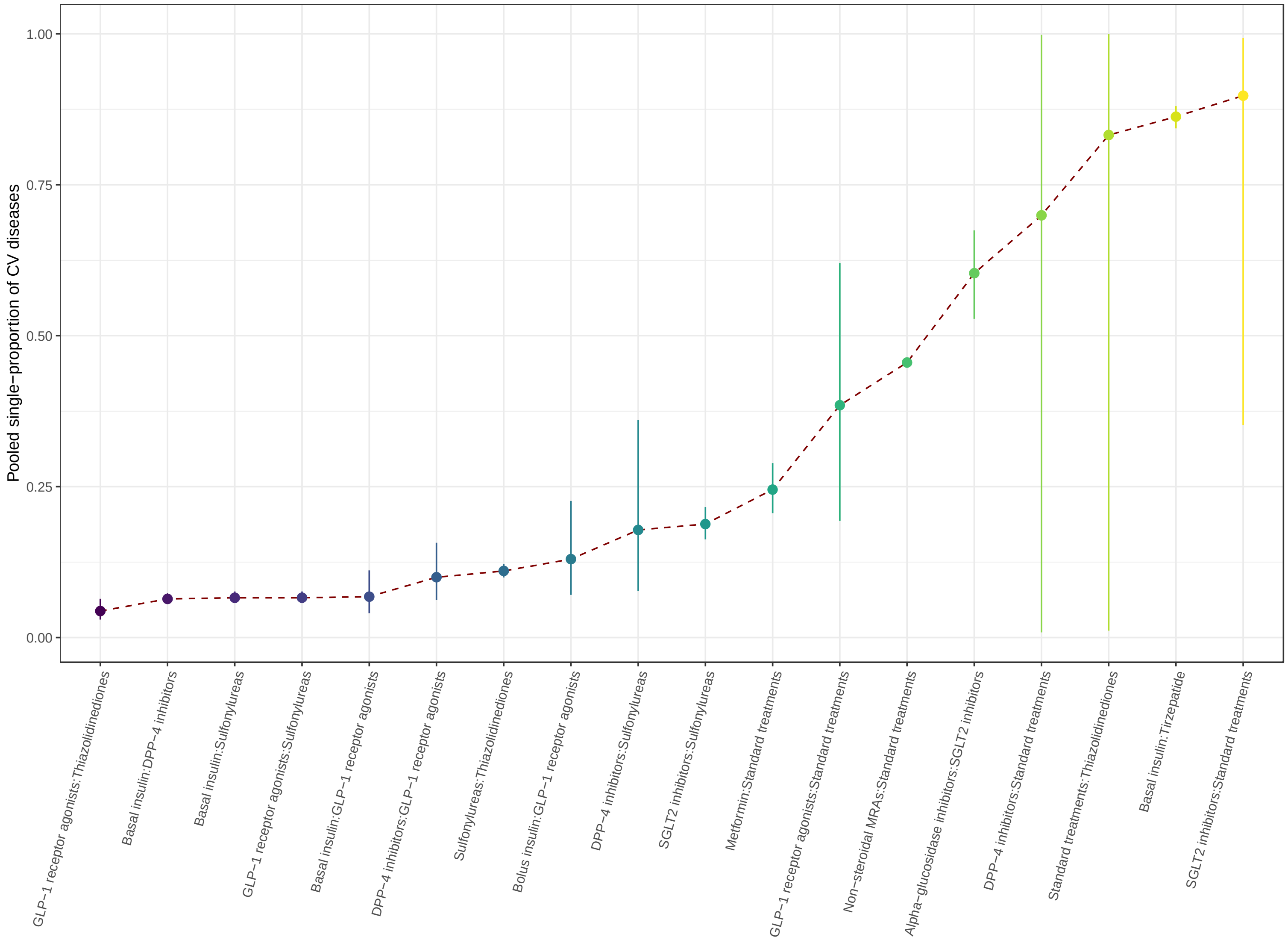

4.5.7.4 Duration of baseline diabetes

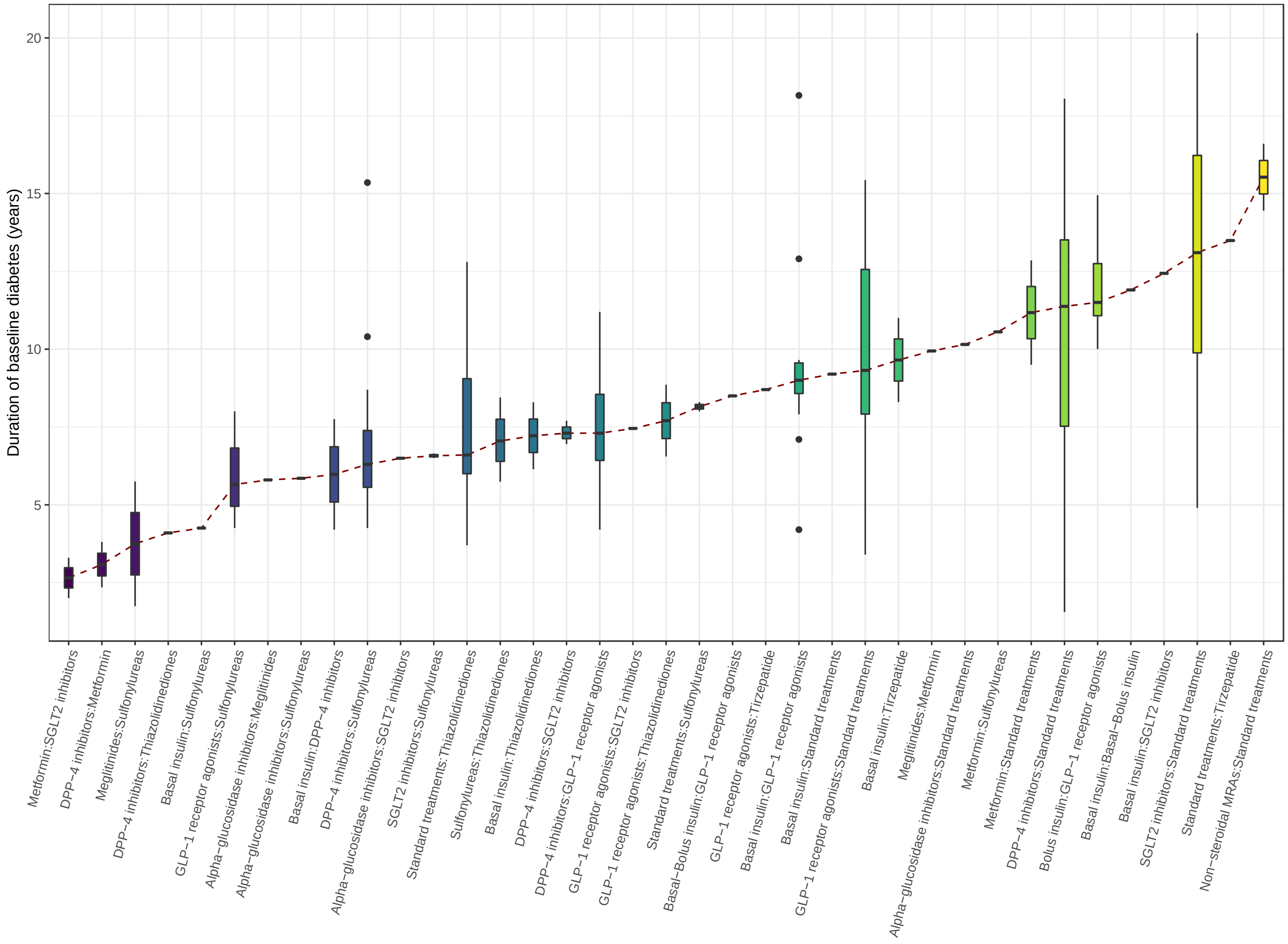

4.5.7.5 HbA1c

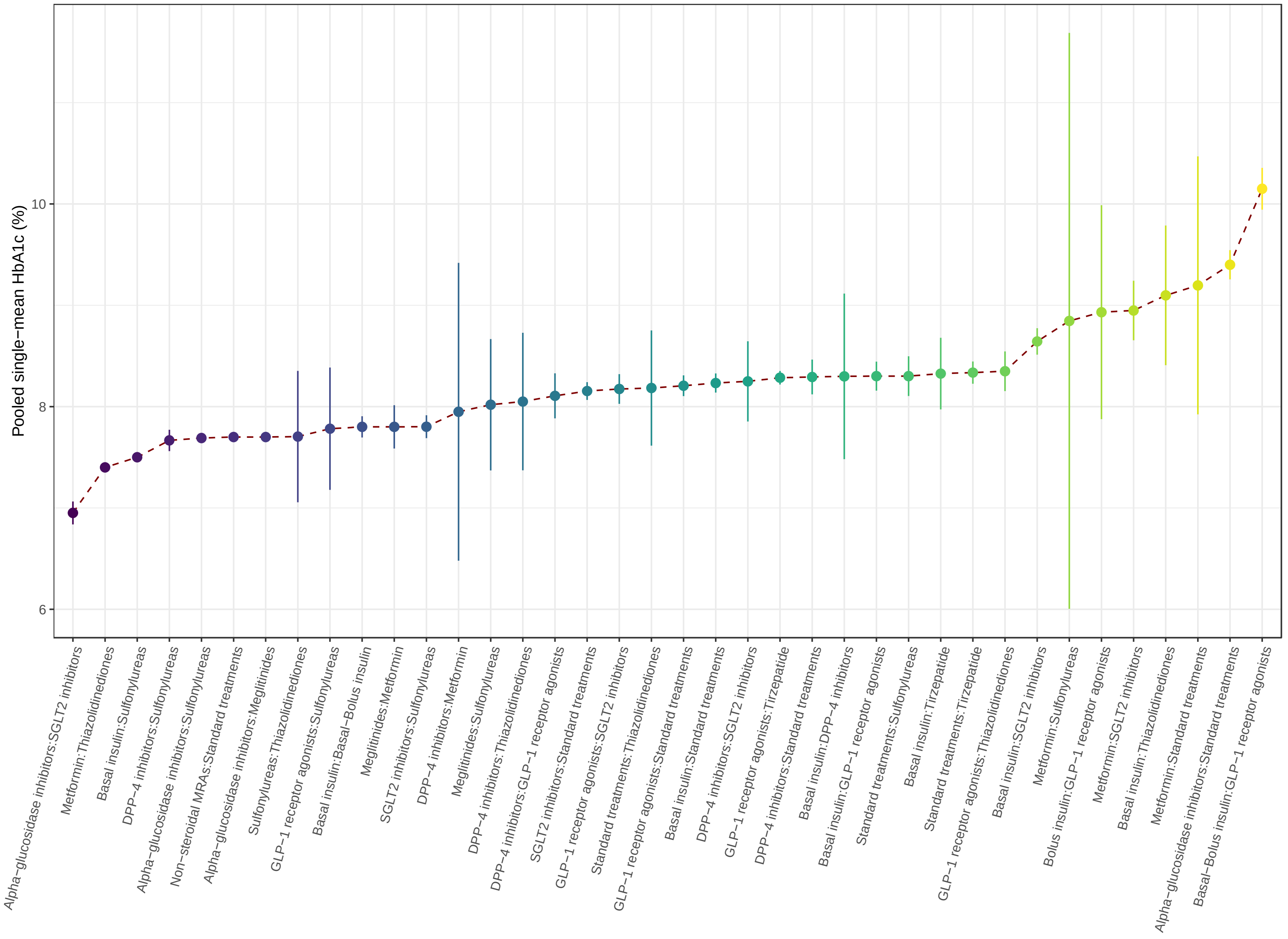

Pooled single−mean HbA1c (%)
10
8
6
Alpha−glucosidase inhibitors:SGLT2 inhibitors
Metformin:Thiazolidinediones
Basal insulin:Sulfonylureas
DPP−4 inhibitors:Sulfonylureas
Alpha−glucosidase inhibitors:Sulfonylureas
Non−steroidal MRAs:Standard treatments
Alpha−glucosidase inhibitors:Meglitinides
Sulfonylureas:Thiazolidinediones
GLP−1 receptor agonists:Sulfonylureas
Basal insulin:Basal−Bolus insulin
Meglitinides:Metformin
SGLT2 inhibitors:Sulfonylureas
DPP−4 inhibitors:Metformin
Meglitinides:Sulfonylureas
DPP−4 inhibitors:Thiazolidinediones
DPP−4 inhibitors:GLP−1 receptor agonists
SGLT2 inhibitors:Standard treatments
GLP−1 receptor agonists:SGLT2 inhibitors
Standard treatments:Thiazolidinediones
GLP−1 receptor agonists:Standard treatments
Basal insulin:Standard treatments
DPP−4 inhibitors:SGLT2 inhibitors
GLP−1 receptor agonists:Tirzepatide
DPP−4 inhibitors:Standard treatments
Basal insulin:DPP−4 inhibitors
Basal insulin:GLP−1 receptor agonists
Standard treatments:Sulfonylureas
Basal insulin:Tirzepatide
Standard treatments:Tirzepatide
GLP−1 receptor agonists:Thiazolidinediones
Basal insulin:SGLT2 inhibitors
Metformin:Sulfonylureas
Bolus insulin:GLP−1 receptor agonists
Metformin:SGLT2 inhibitors
Basal insulin:Thiazolidinediones
Metformin:Standard treatments
Alpha−glucosidase inhibitors:Standard treatments
Basal−Bolus insulin:GLP−1 receptor agonists

4.5.7.6 Proportion of males

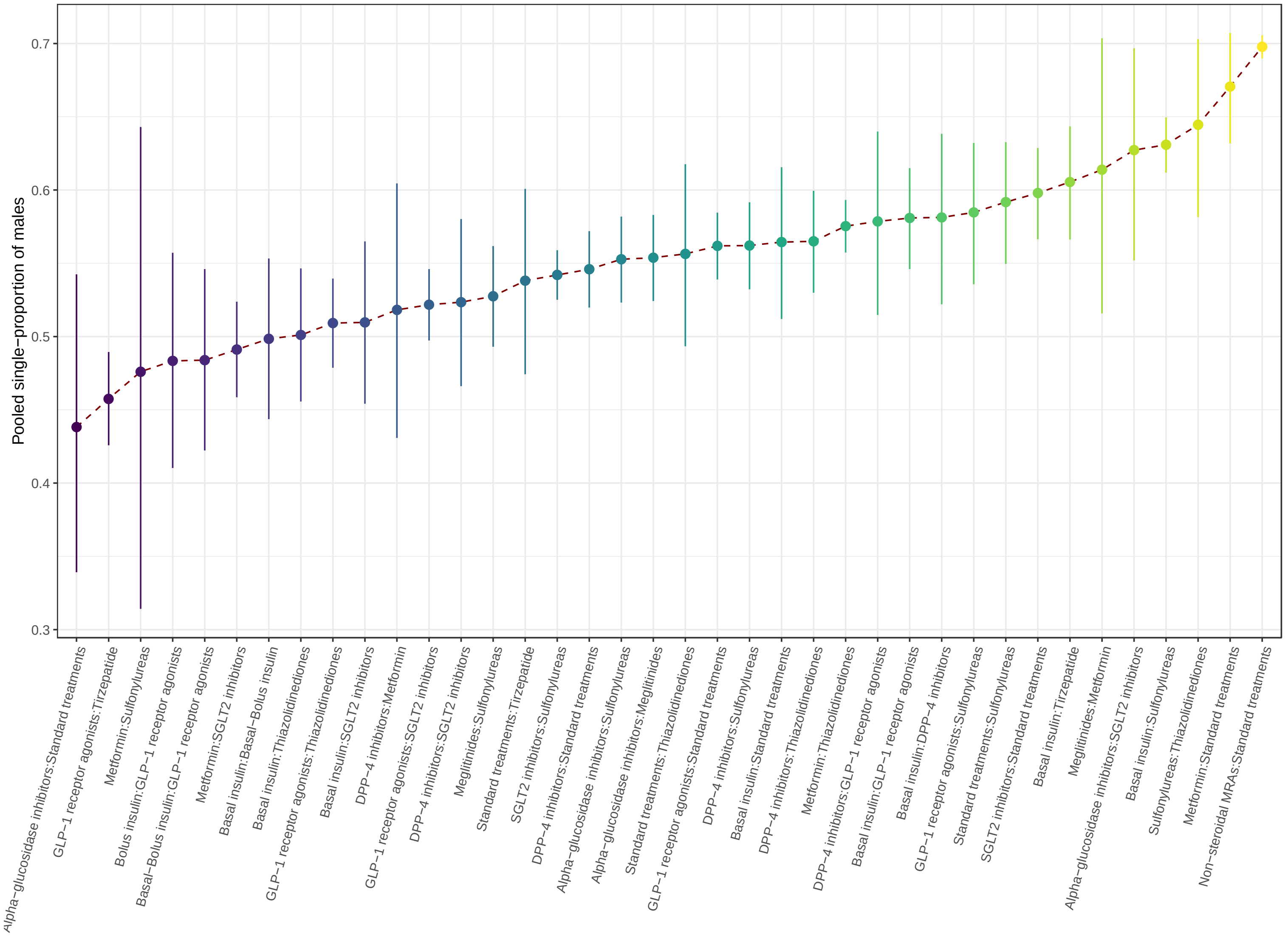

4.5.8 Severe gastrointestinal events

4.5.8.1 Age

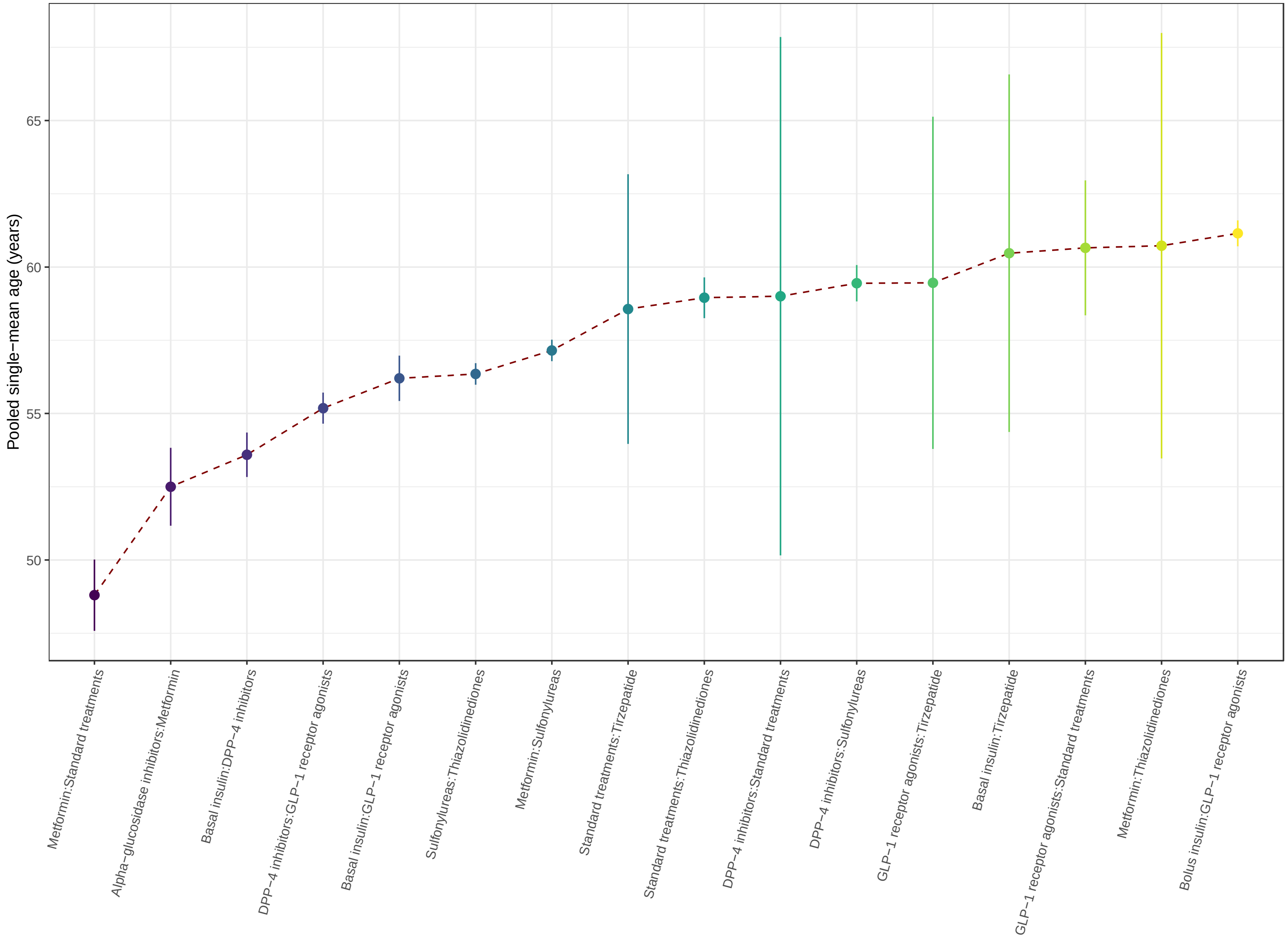

4.5.8.2 Body mass index

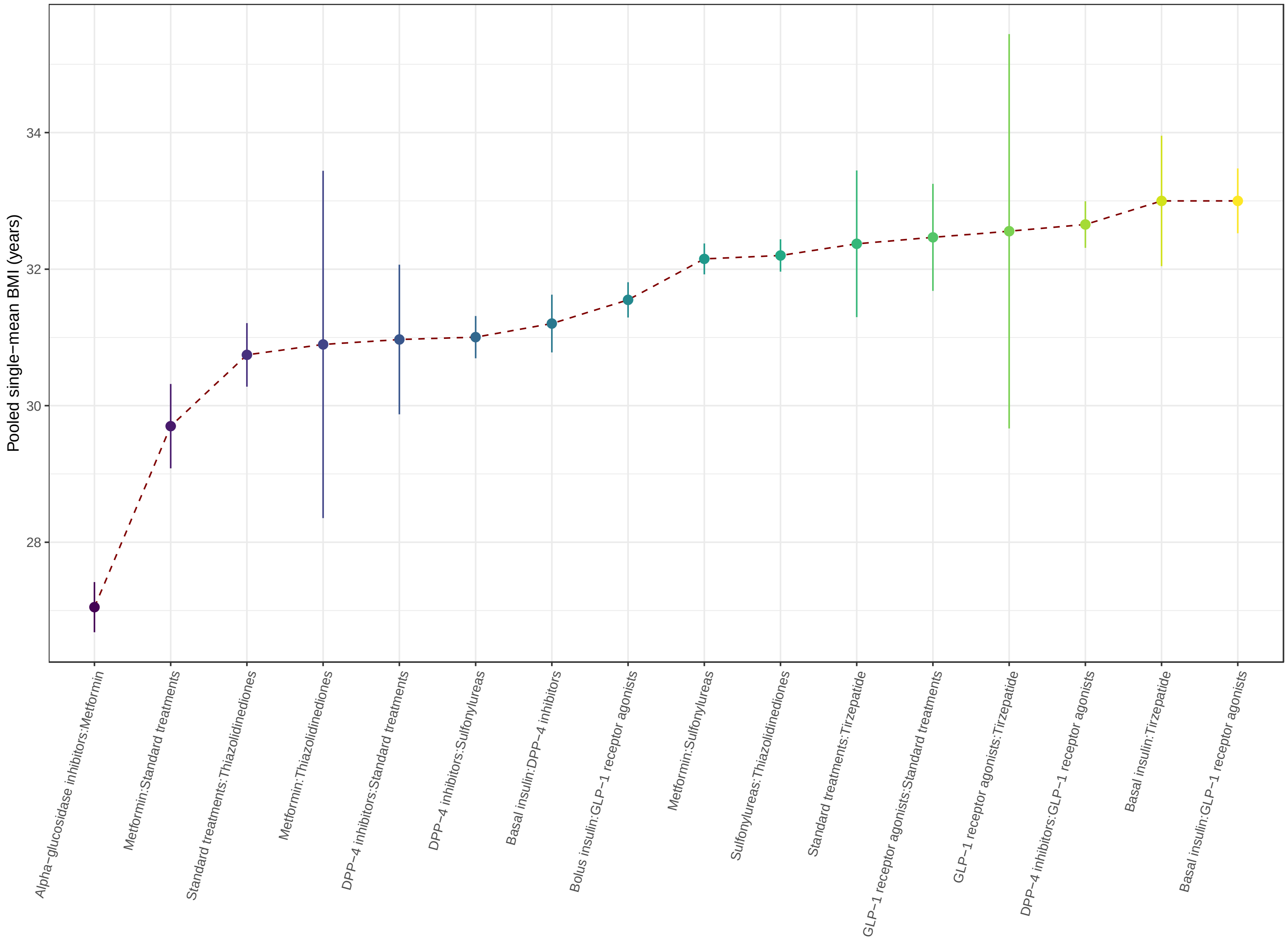

4.5.8.3 Proportion of cardiovascular disease

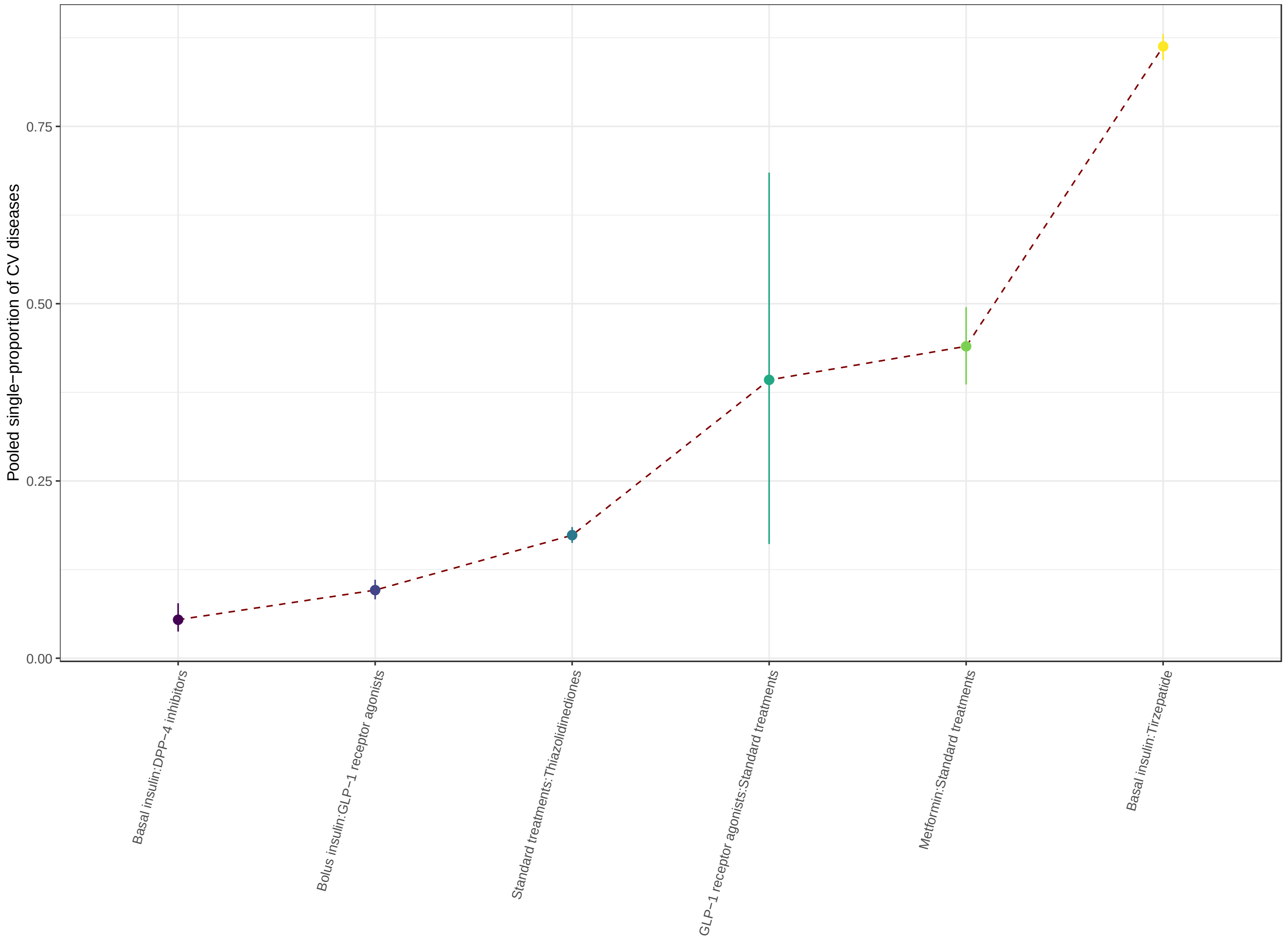

4.5.8.4 Duration of baseline diabetes

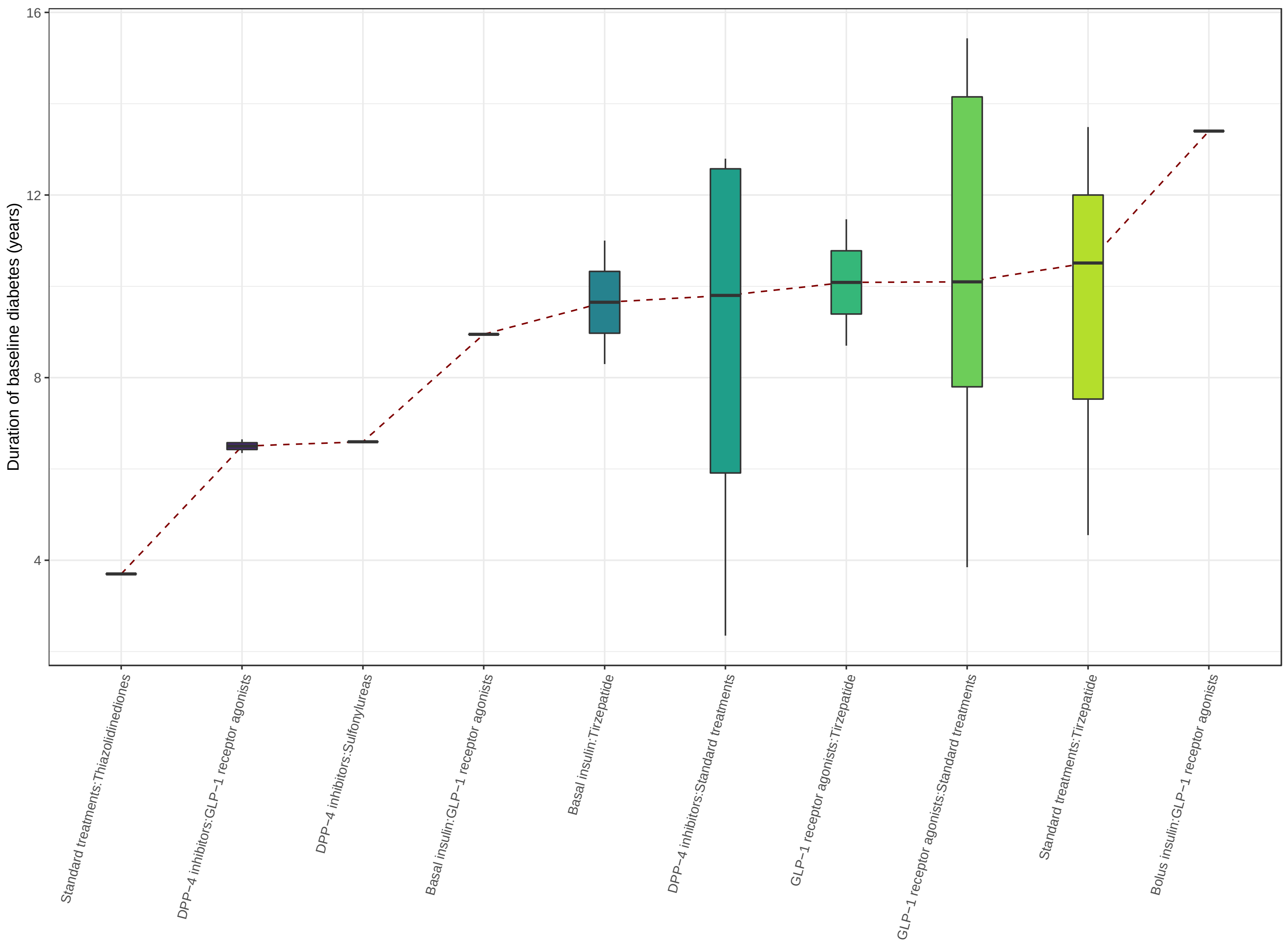

4.5.8.5 HbA1c

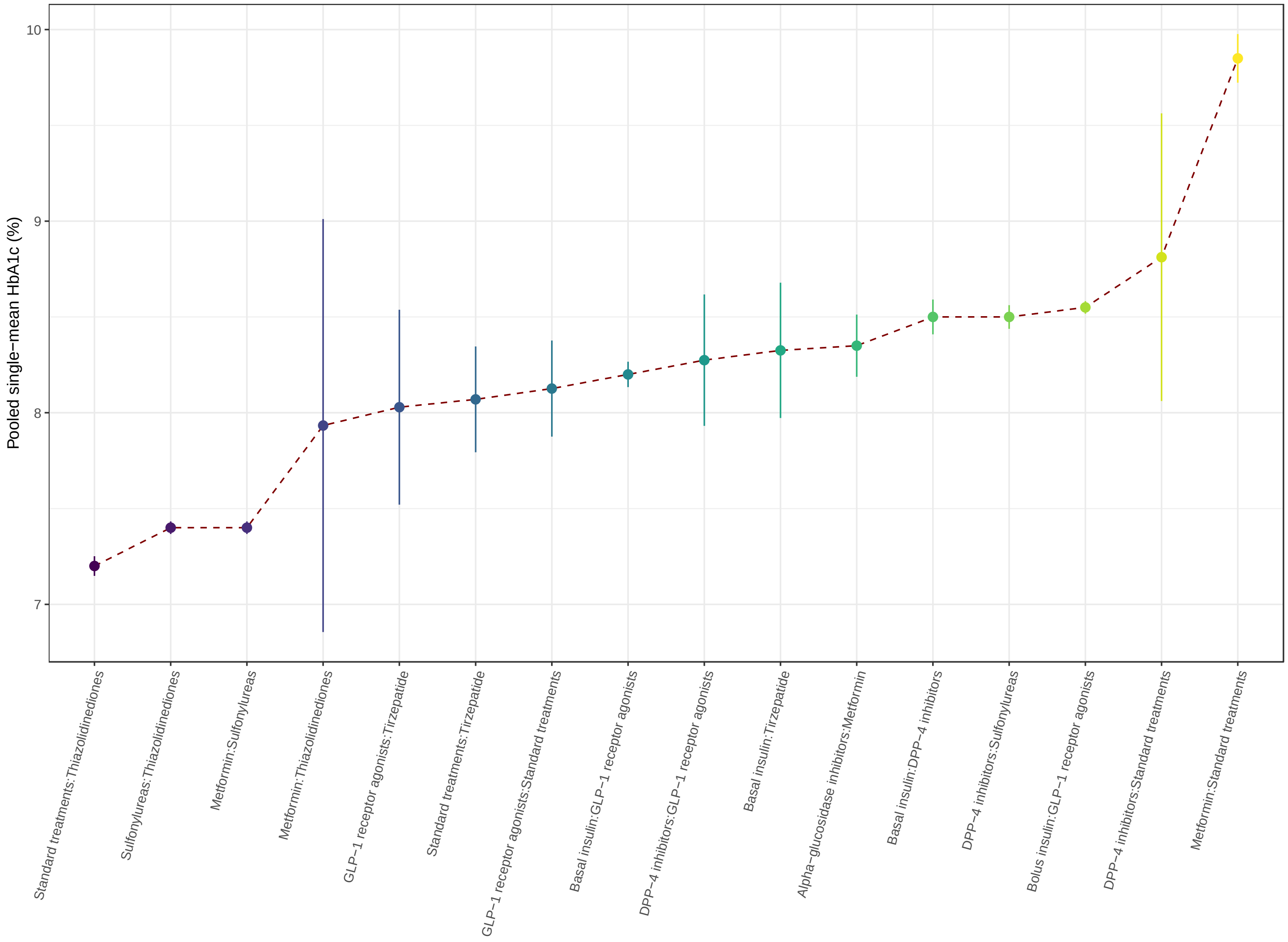

Pooled single−mean HbA1c (%)
10
9
8
7
Standard treatments:Thiazolidinediones
Sulfonylureas:Thiazolidinediones
Metformin:Sulfonylureas
Metformin:Thiazolidinediones
GLP−1 receptor agonists:Tirzepatide
Standard treatments:Tirzepatide
GLP−1 receptor agonists:Standard treatments
Basal insulin:GLP−1 receptor agonists
DPP−4 inhibitors:GLP−1 receptor agonists
Basal insulin:Tirzepatide
Alpha−glucosidase inhibitors:Metformin
Basal insulin:DPP−4 inhibitors
DPP−4 inhibitors:Sulfonylureas
Bolus insulin:GLP−1 receptor agonists
DPP−4 inhibitors:Standard treatments
Metformin:Standard treatments

4.5.8.6 Proportion of males

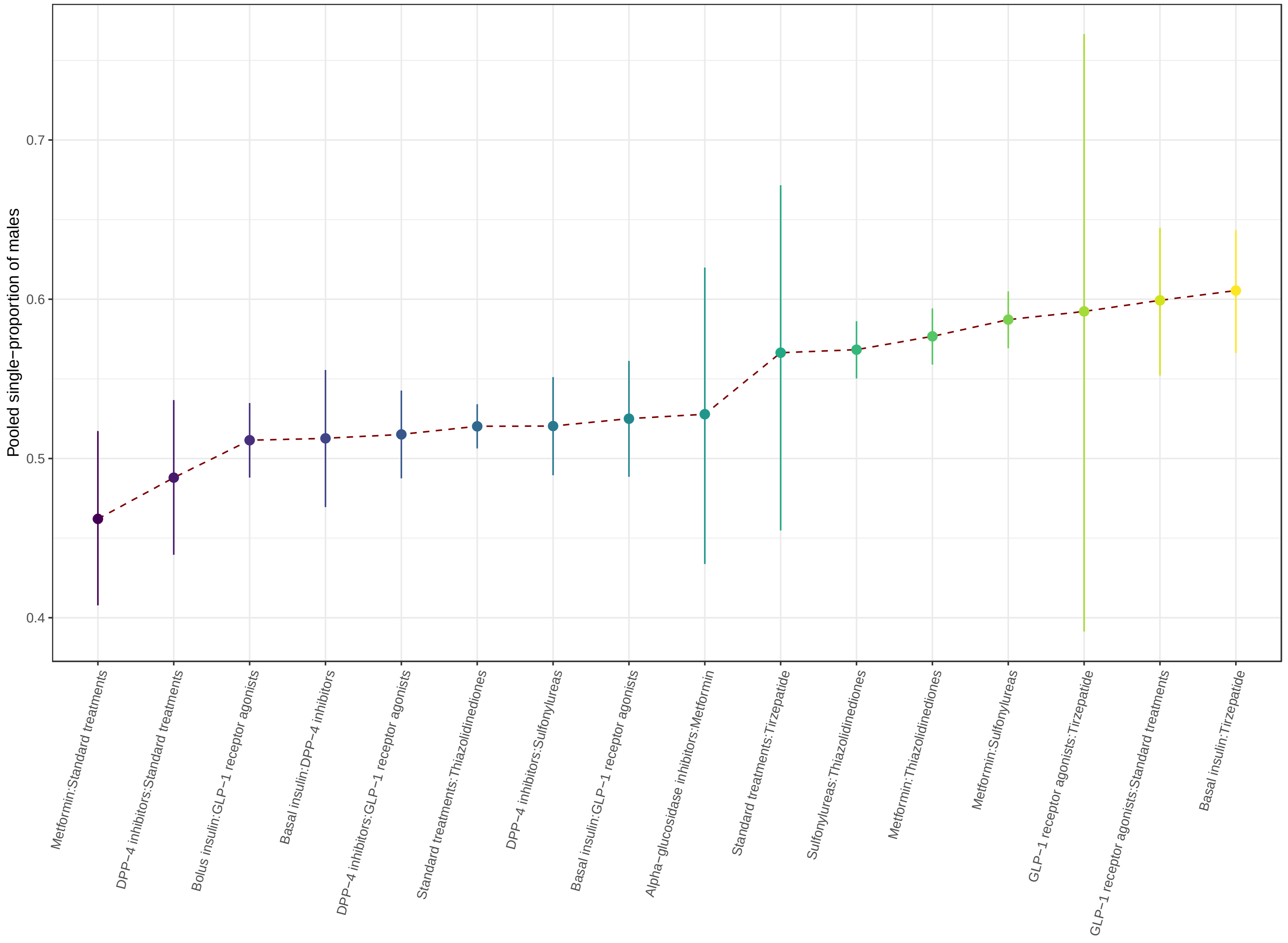

4.5.9 Genital infection

4.5.9.1 Age

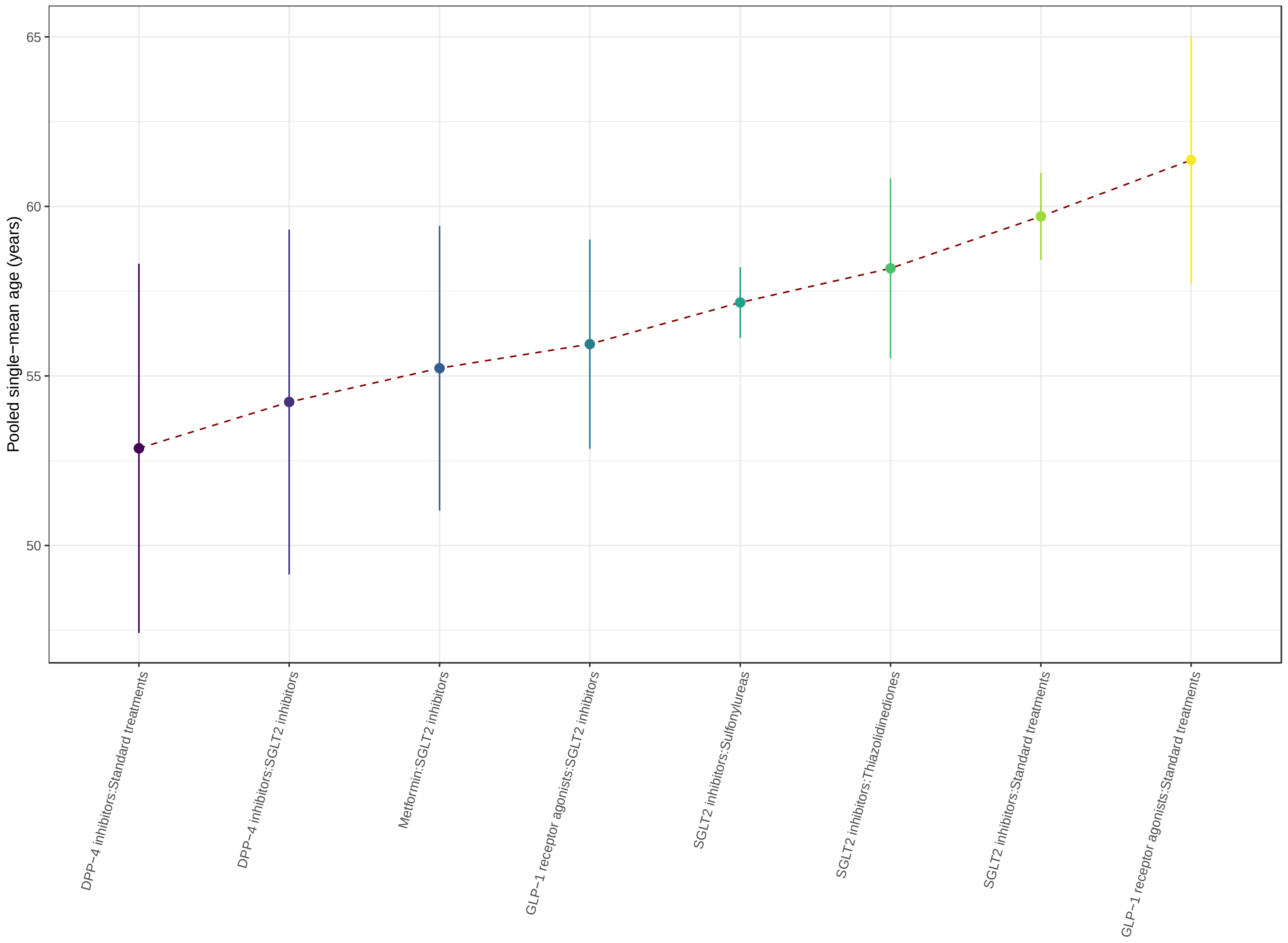

4.5.9.2 Body mass index

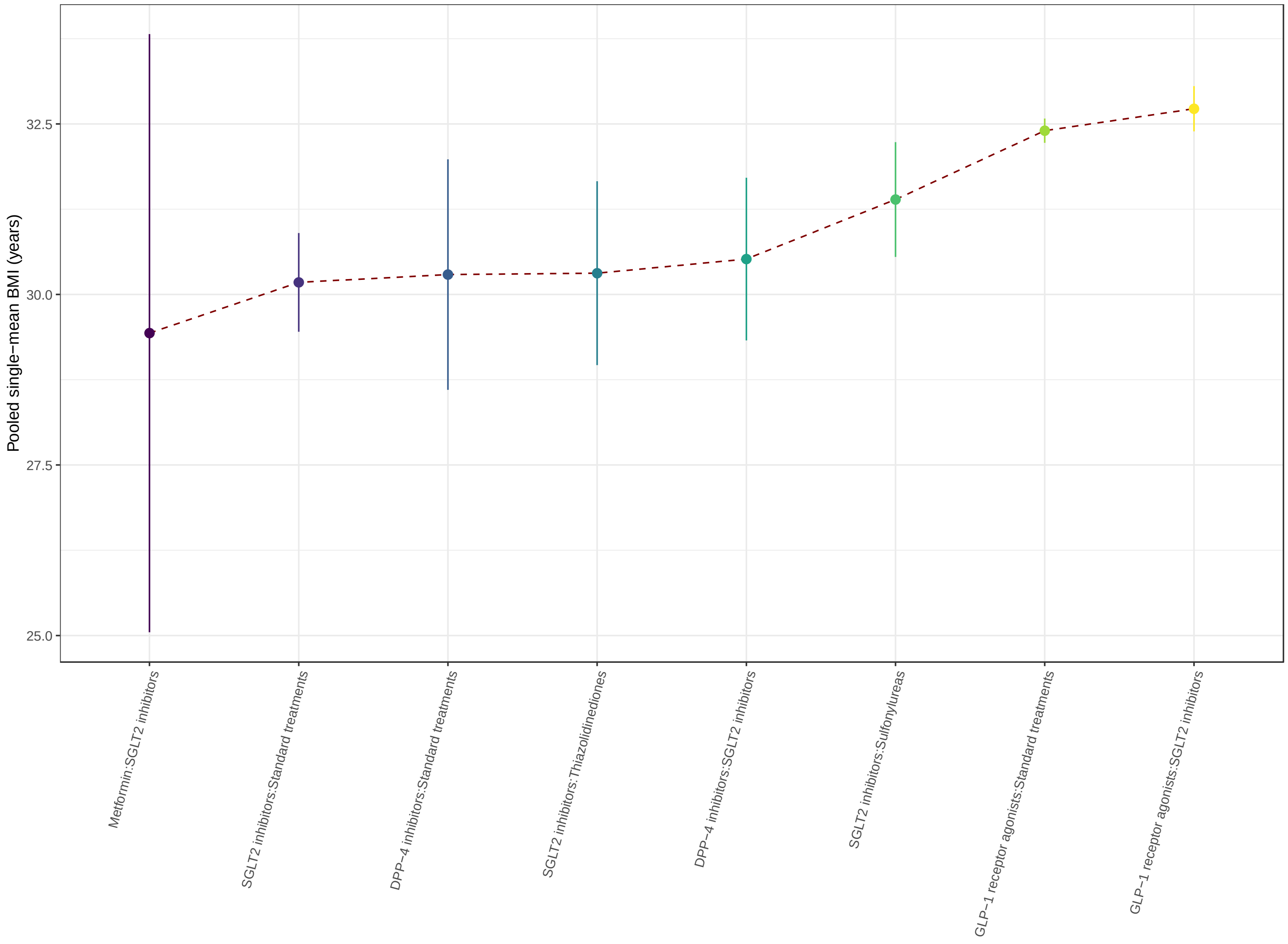

4.5.9.3 Proportion of cardiovascular disease

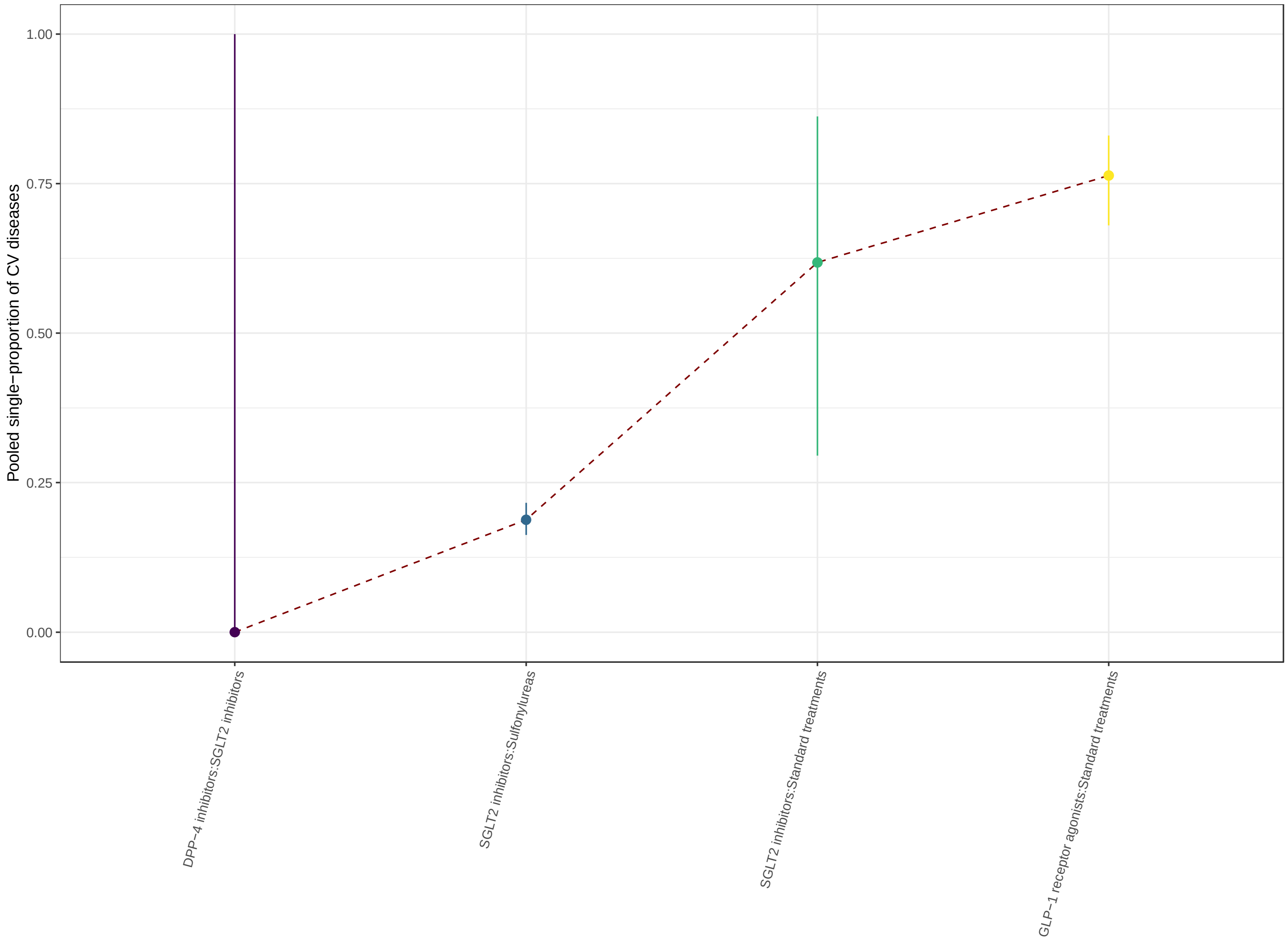

4.5.9.4 Duration of baseline diabetes

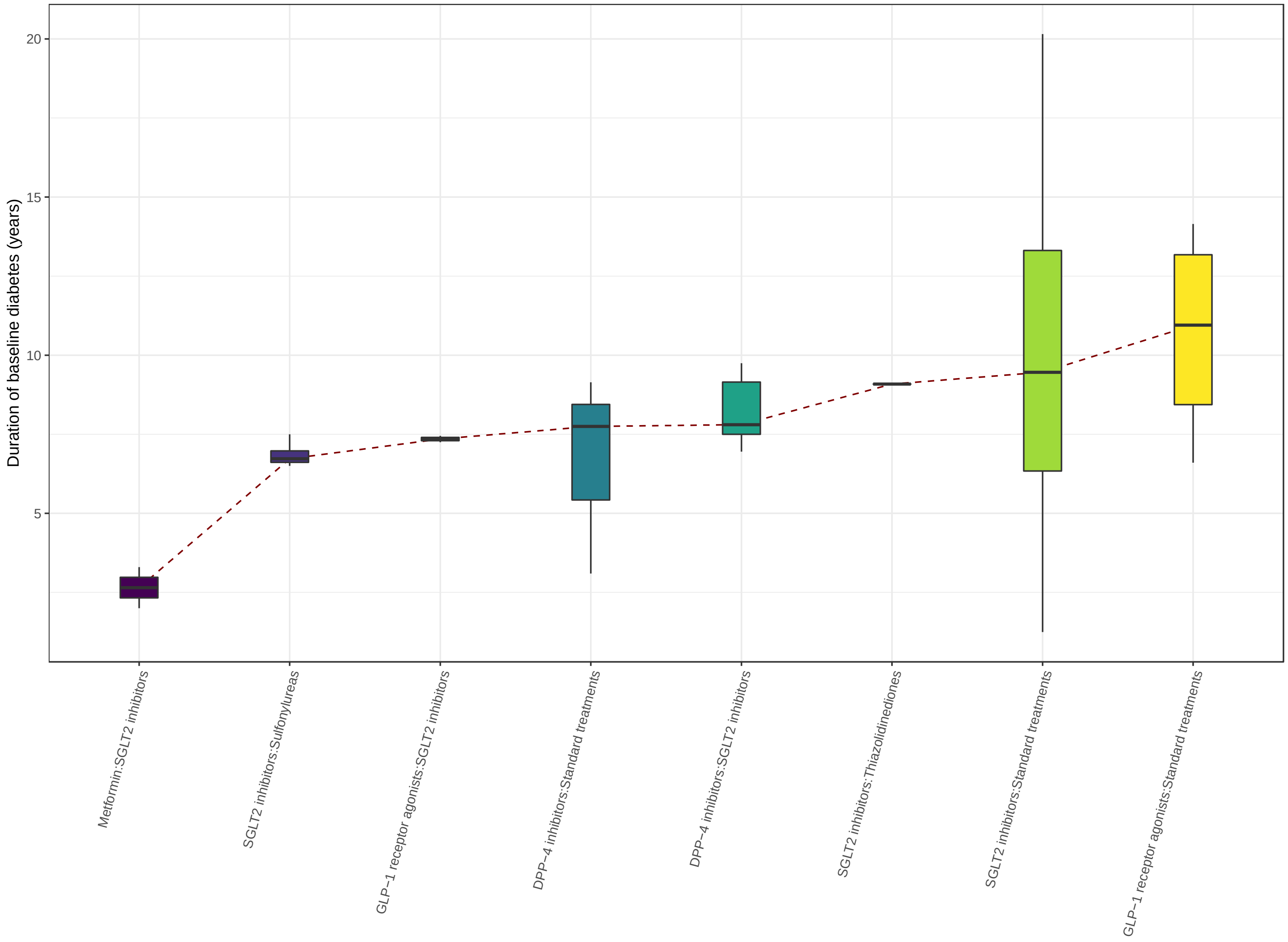

4.5.9.5 HbA1c

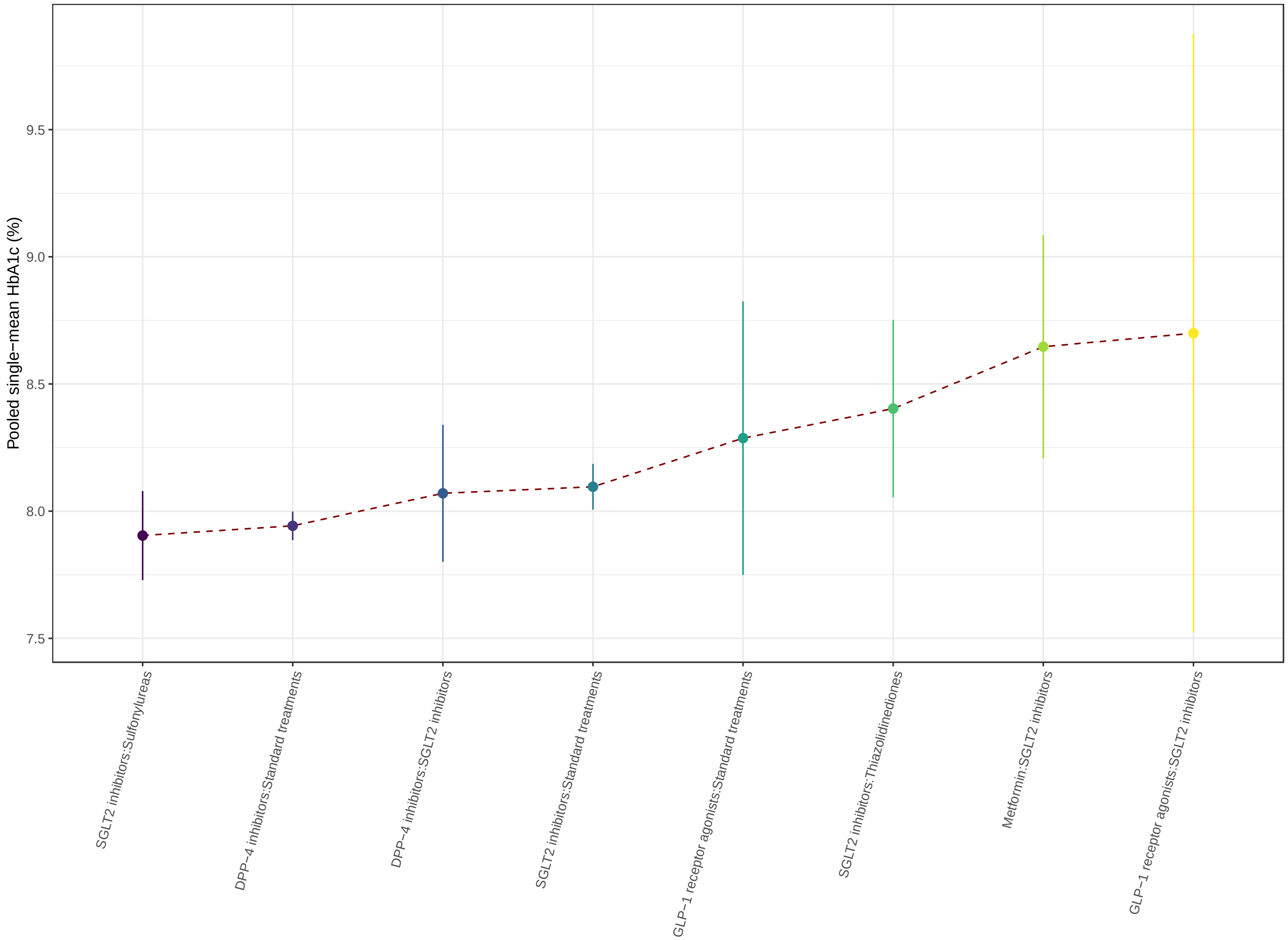

Pooled single−mean HbA1c (%)
9.5
9.0
8.5
8.0
7.5
SGLT2 inhibitors:Sulfonylureas
DPP−4 inhibitors:Standard treatments
DPP−4 inhibitors:SGLT2 inhibitors
SGLT2 inhibitors:Standard treatments
GLP−1 receptor agonists:Standard treatments
SGLT2 inhibitors:Thiazolidinediones
Metformin:SGLT2 inhibitors
GLP−1 receptor agonists:SGLT2 inhibitors

4.5.9.6 Proportion of males

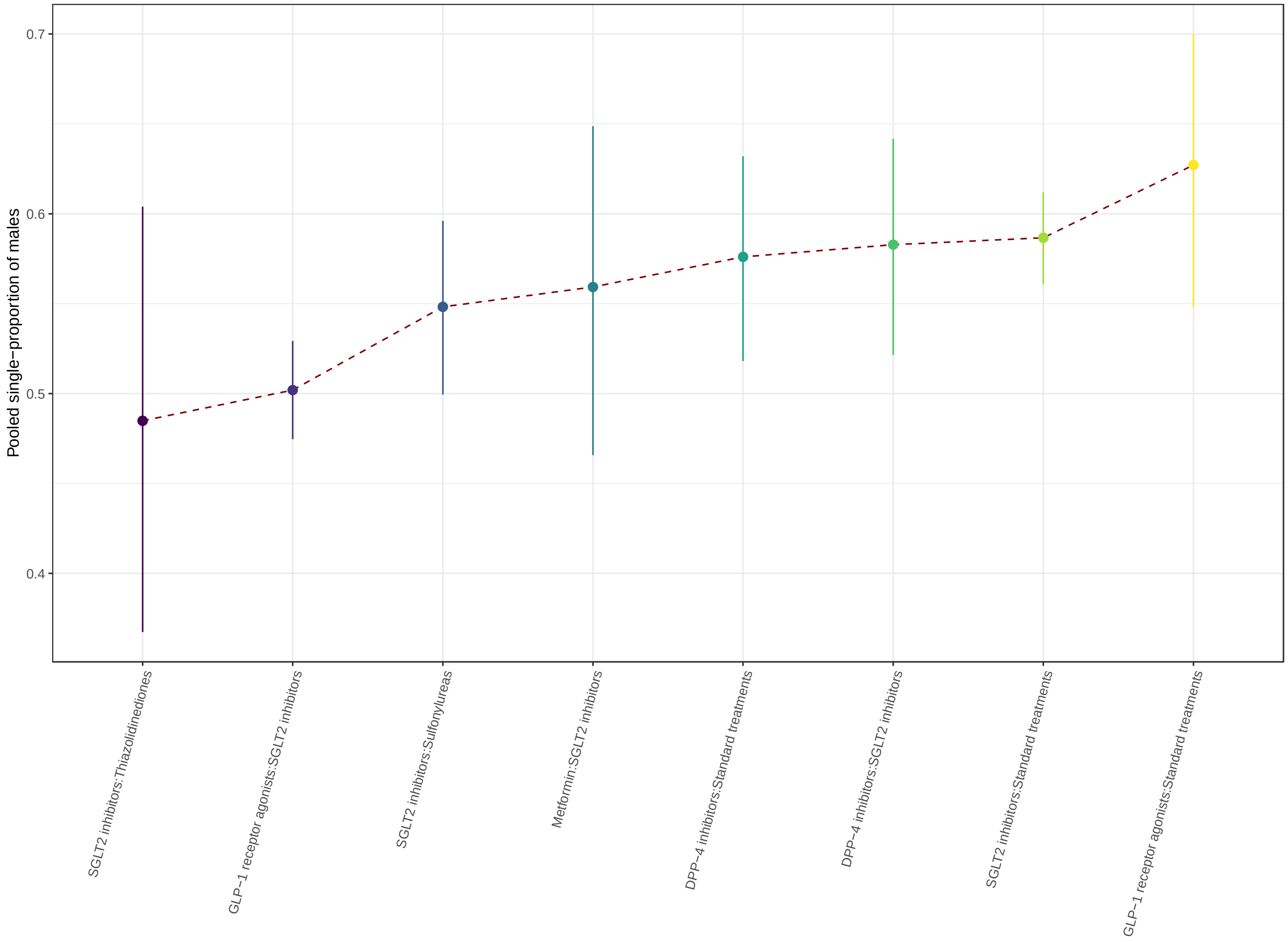

4.5.10 Amputation

4.5.10.1 Age

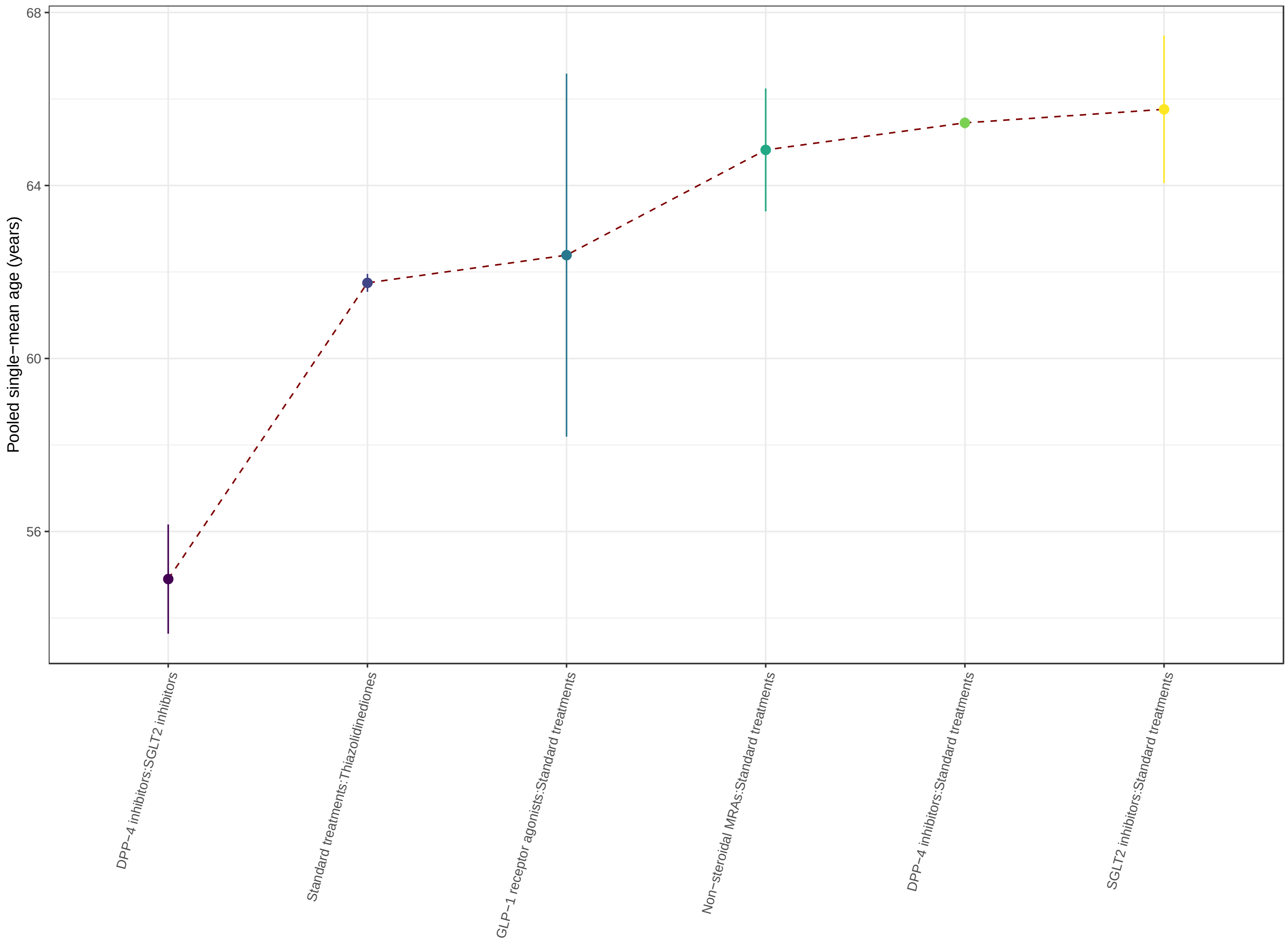

4.5.10.2 Body mass index

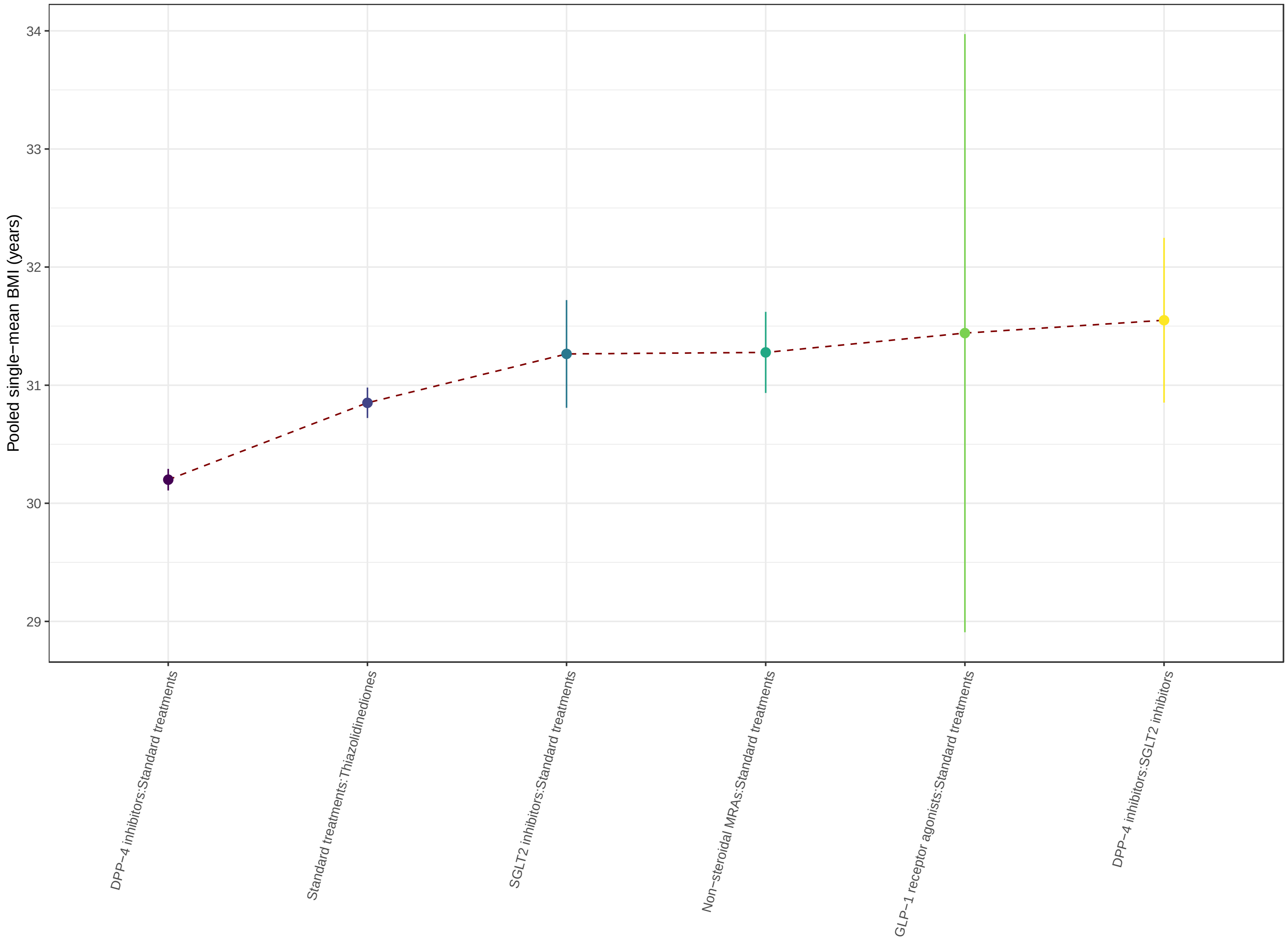

4.5.10.3 Proportion of cardiovascular disease

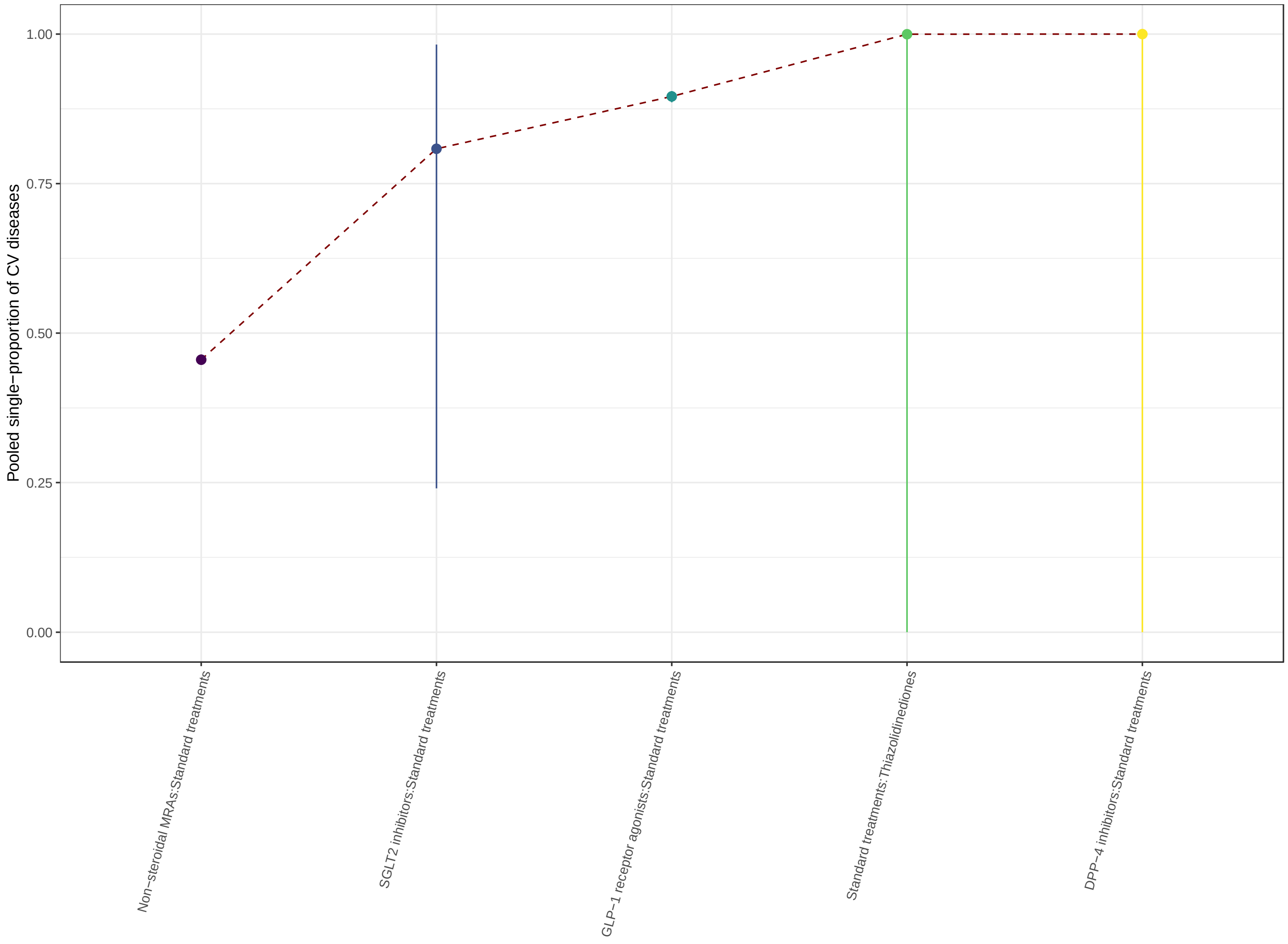

4.5.10.4 Duration of baseline diabetes

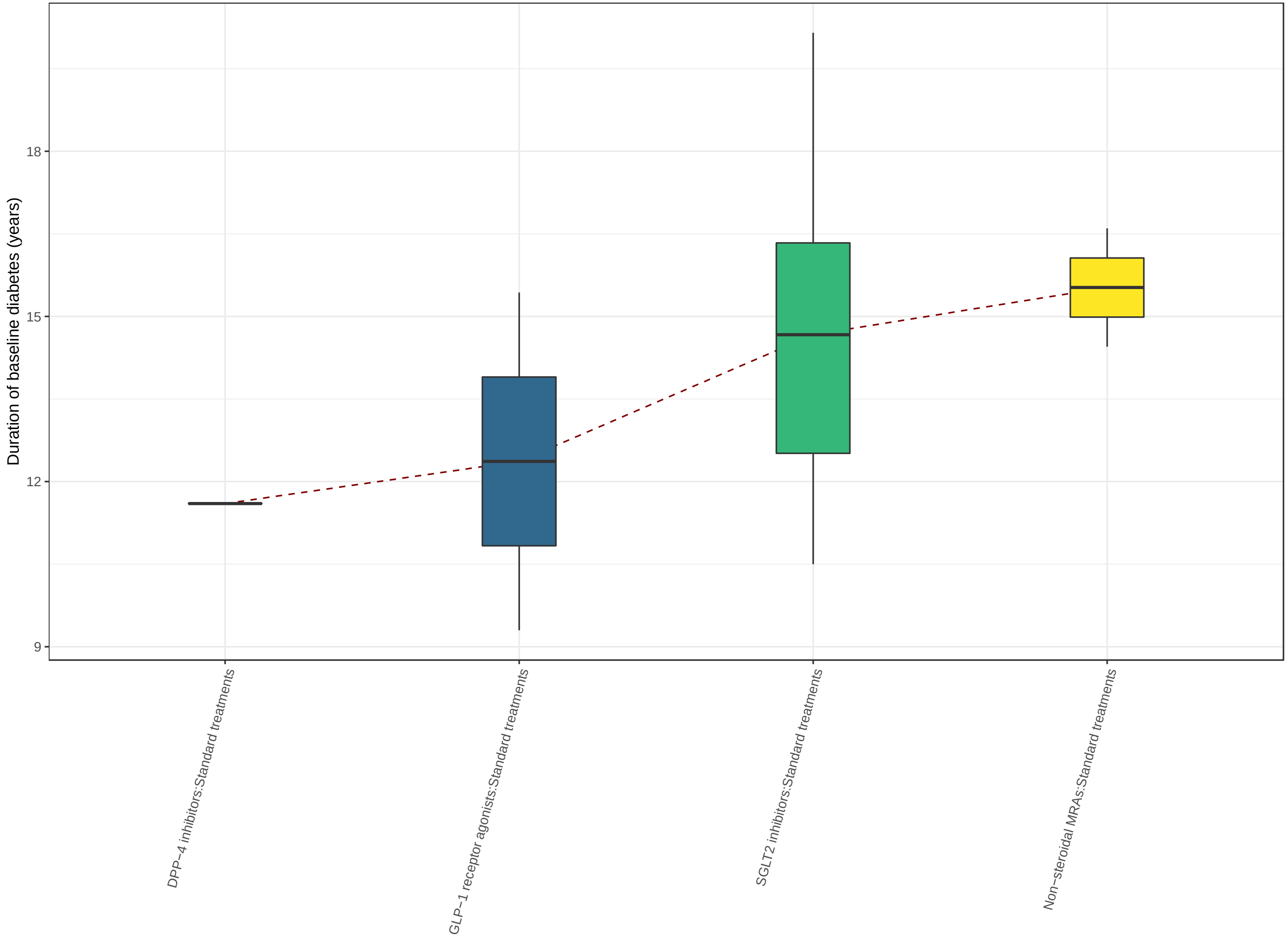

4.5.10.5 HbA1c

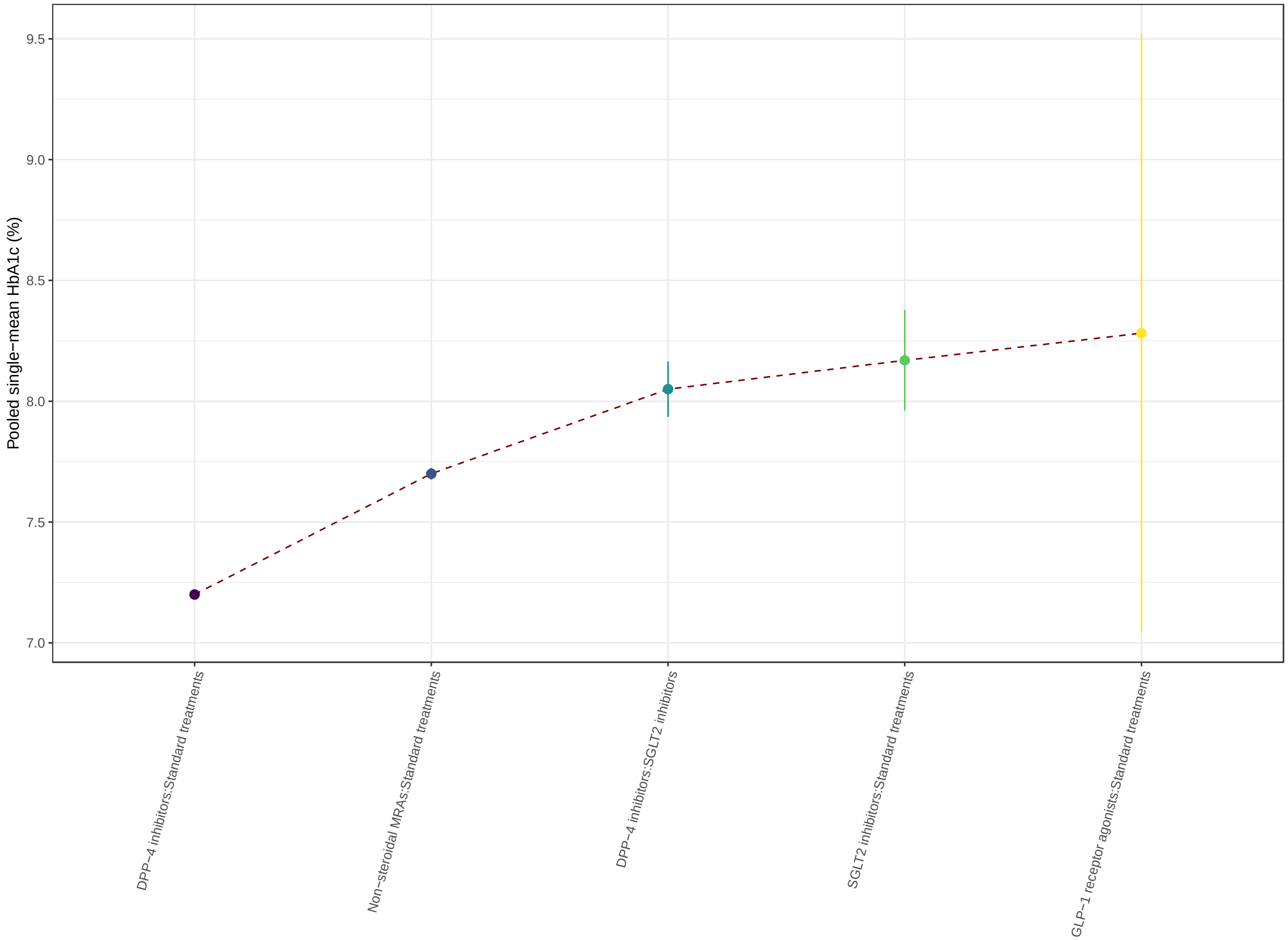

Pooled single−mean HbA1c (%)
9.5
9.0
8.5
8.0
7.5
7.0
DPP−4 inhibitors:Standard treatments
Non−steroidal MRAs:Standard treatments
DPP−4 inhibitors:SGLT2 inhibitors
SGLT2 inhibitors:Standard treatments
GLP−1 receptor agonists:Standard treatments

4.5.10.6 Proportion of males

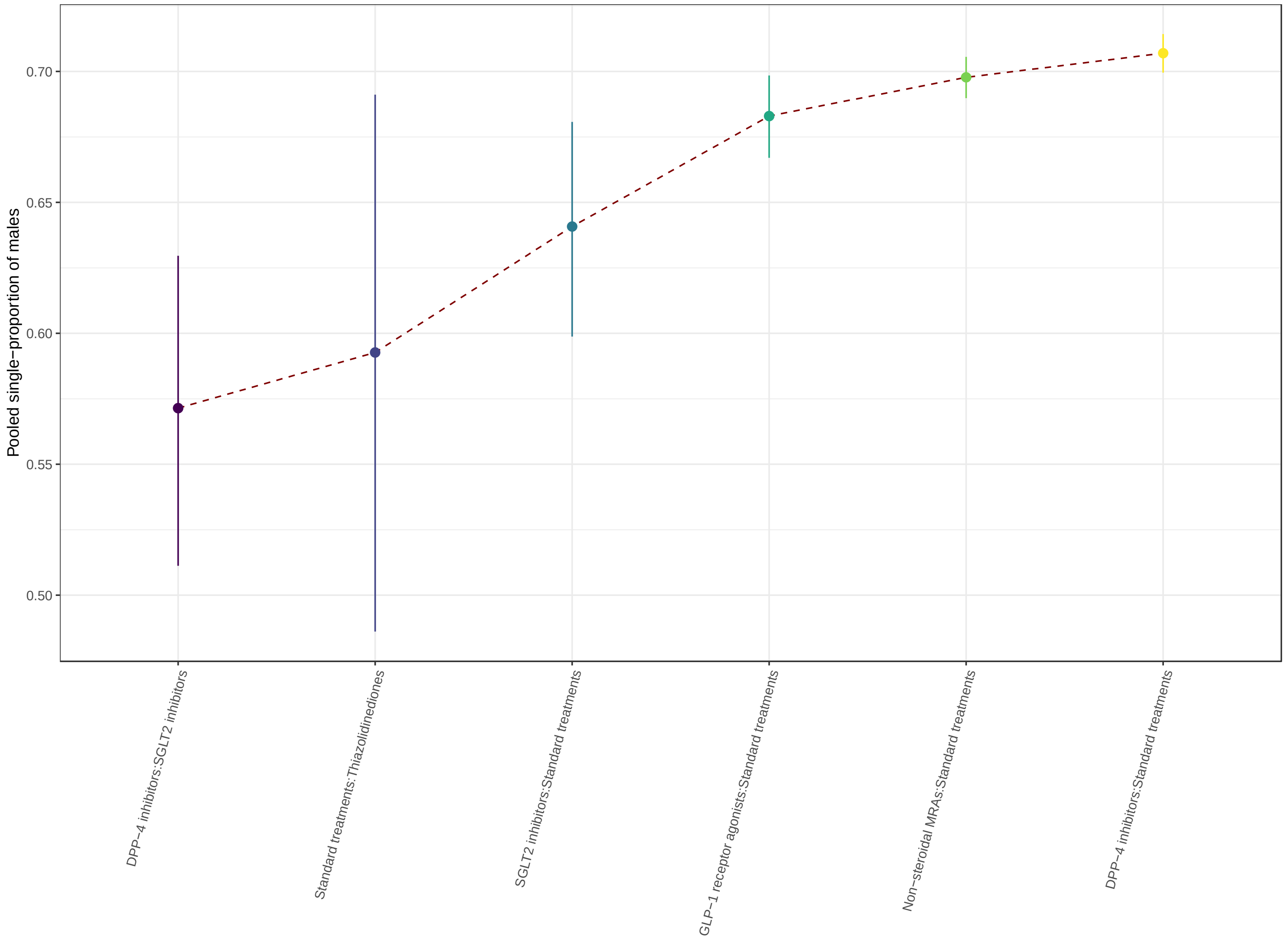

4.5.11 Ketoacidosis due to diabetes

4.5.11.1 Age

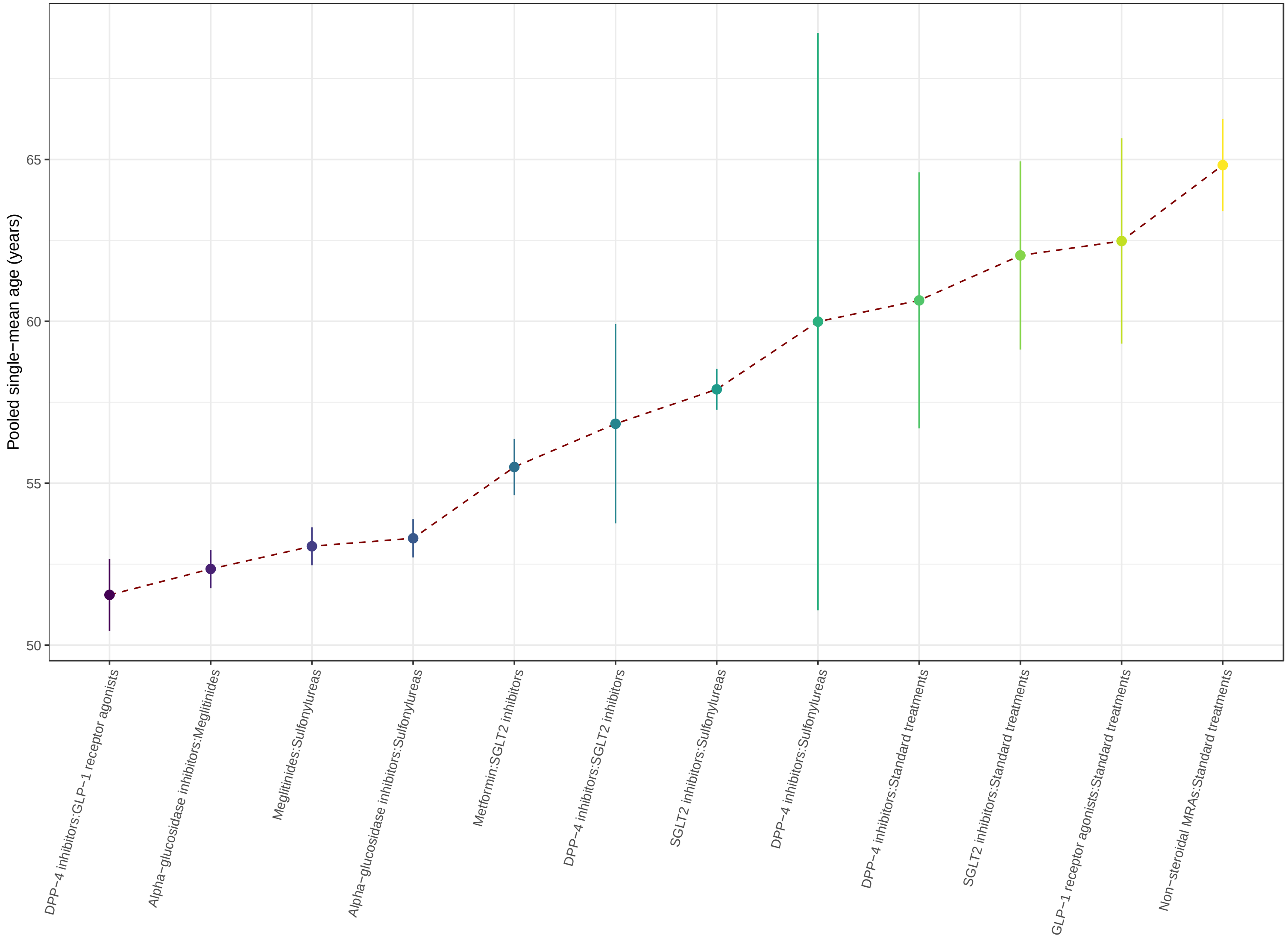

4.5.11.2 Body mass index

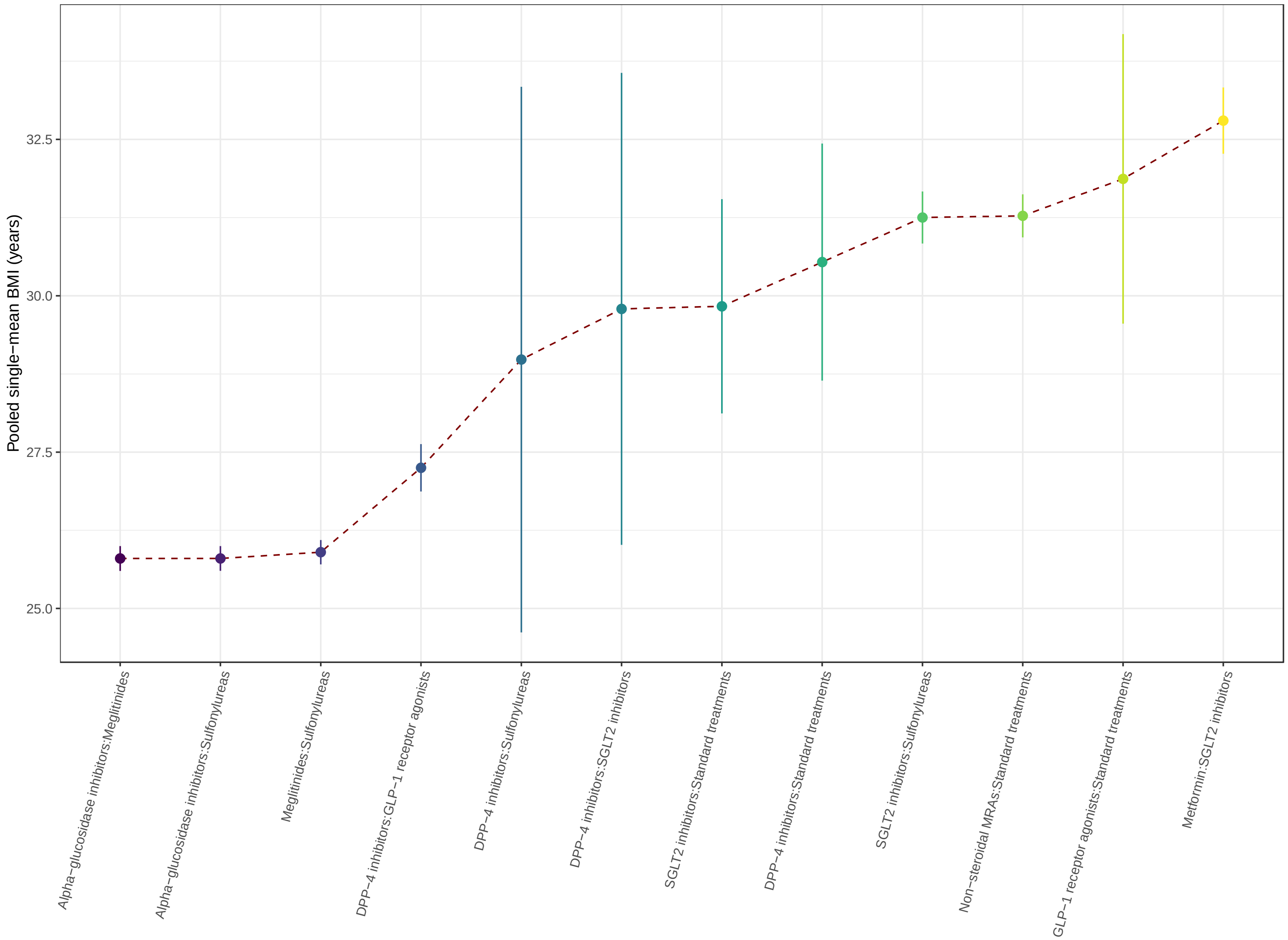

4.5.11.3 Proportion of cardiovascular disease

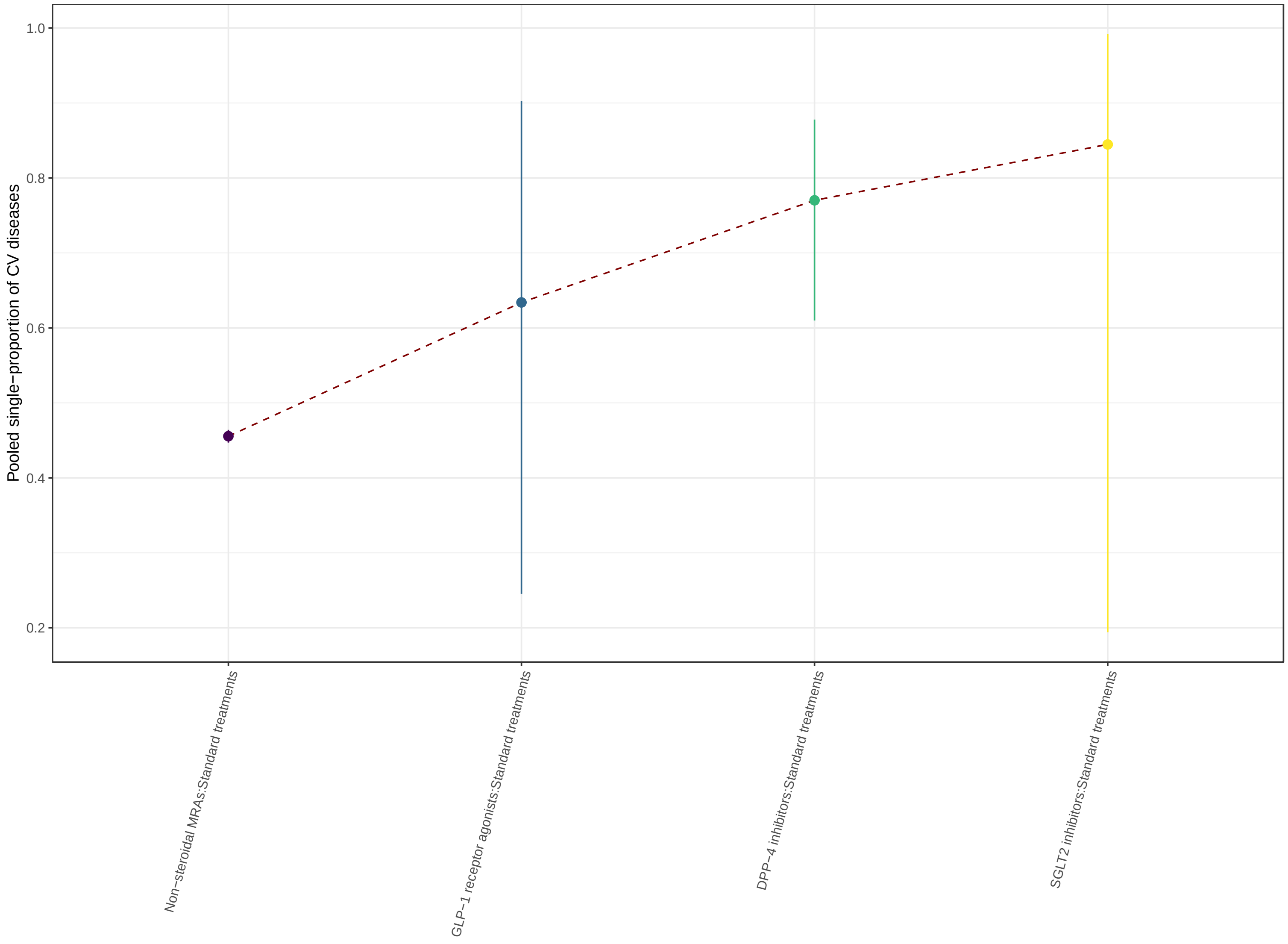

4.5.11.4 Duration of baseline diabetes

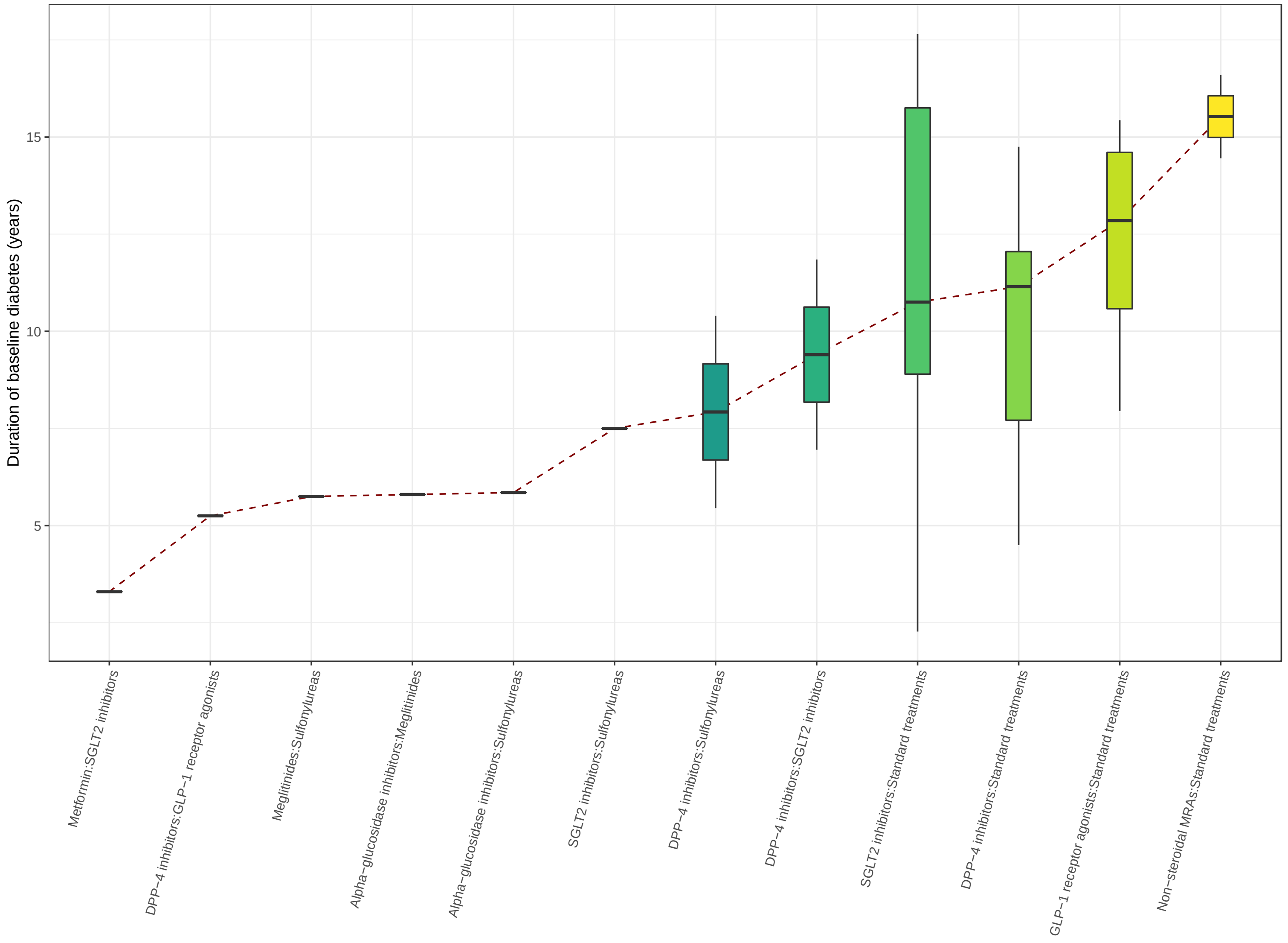

4.5.11.5 HbA1c

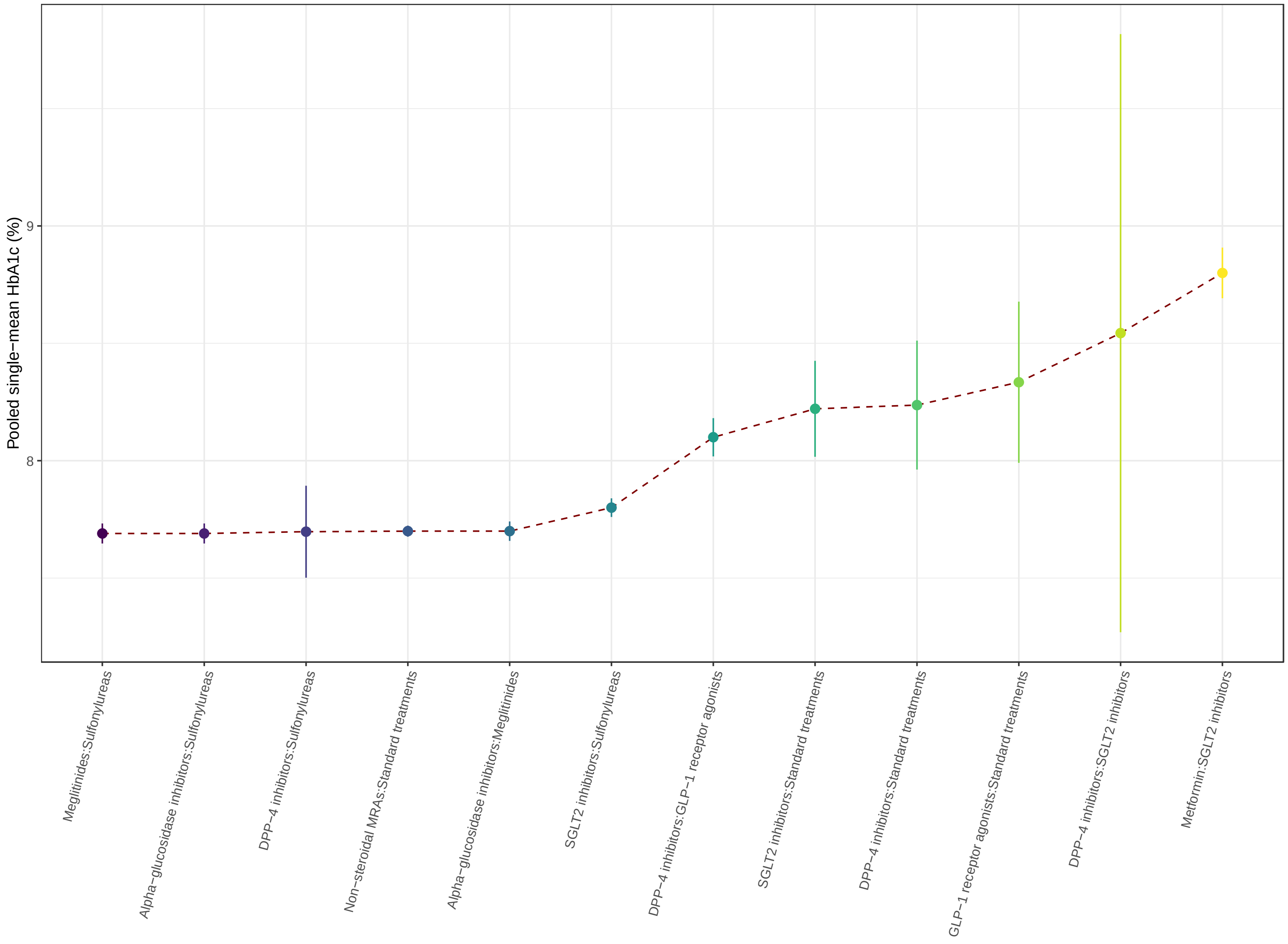

Pooled single−mean HbA1c (%)
9
8
Meglitinides:Sulfonylureas
Alpha−glucosidase inhibitors:Sulfonylureas
DPP−4 inhibitors:Sulfonylureas
Non−steroidal MRAs:Standard treatments
Alpha−glucosidase inhibitors:Meglitinides
SGLT2 inhibitors:Sulfonylureas
DPP−4 inhibitors:GLP−1 receptor agonists
SGLT2 inhibitors:Standard treatments
DPP−4 inhibitors:Standard treatments
GLP−1 receptor agonists:Standard treatments
DPP−4 inhibitors:SGLT2 inhibitors
Metformin:SGLT2 inhibitors

4.5.11.6 Proportion of males

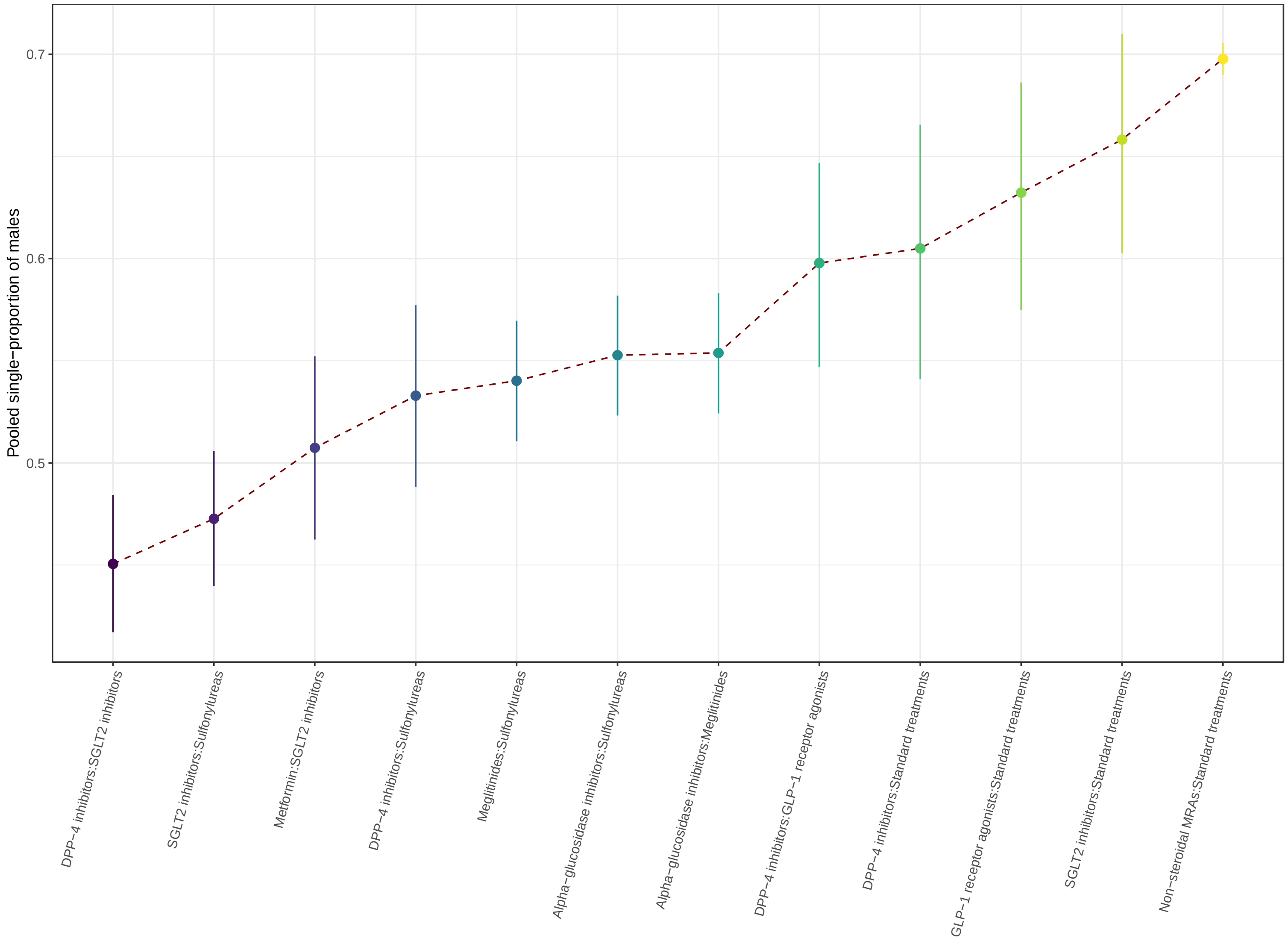

4.5.12 Health-related quality of life

4.5.12.1 Age

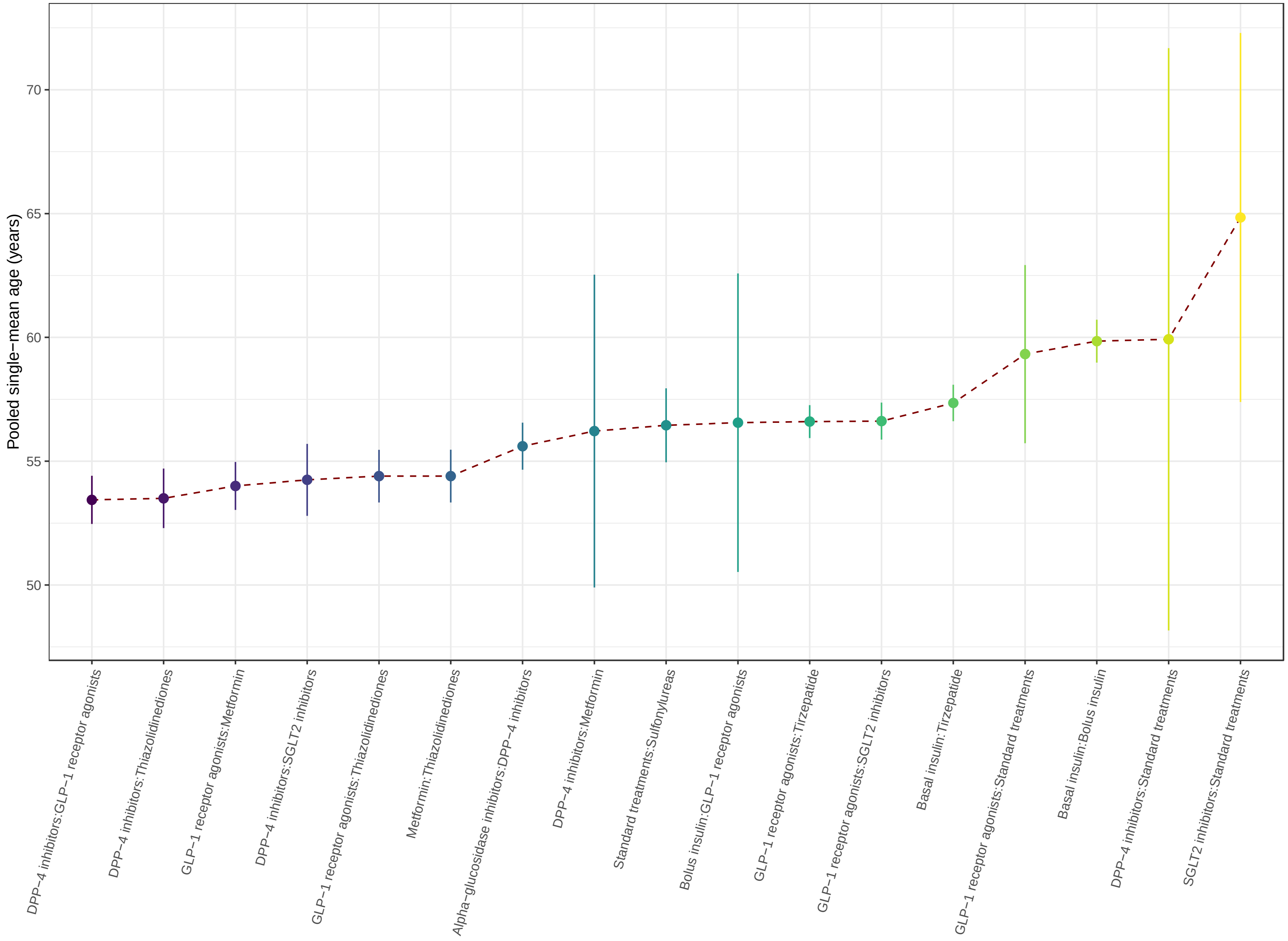

4.5.12.2 Body mass index

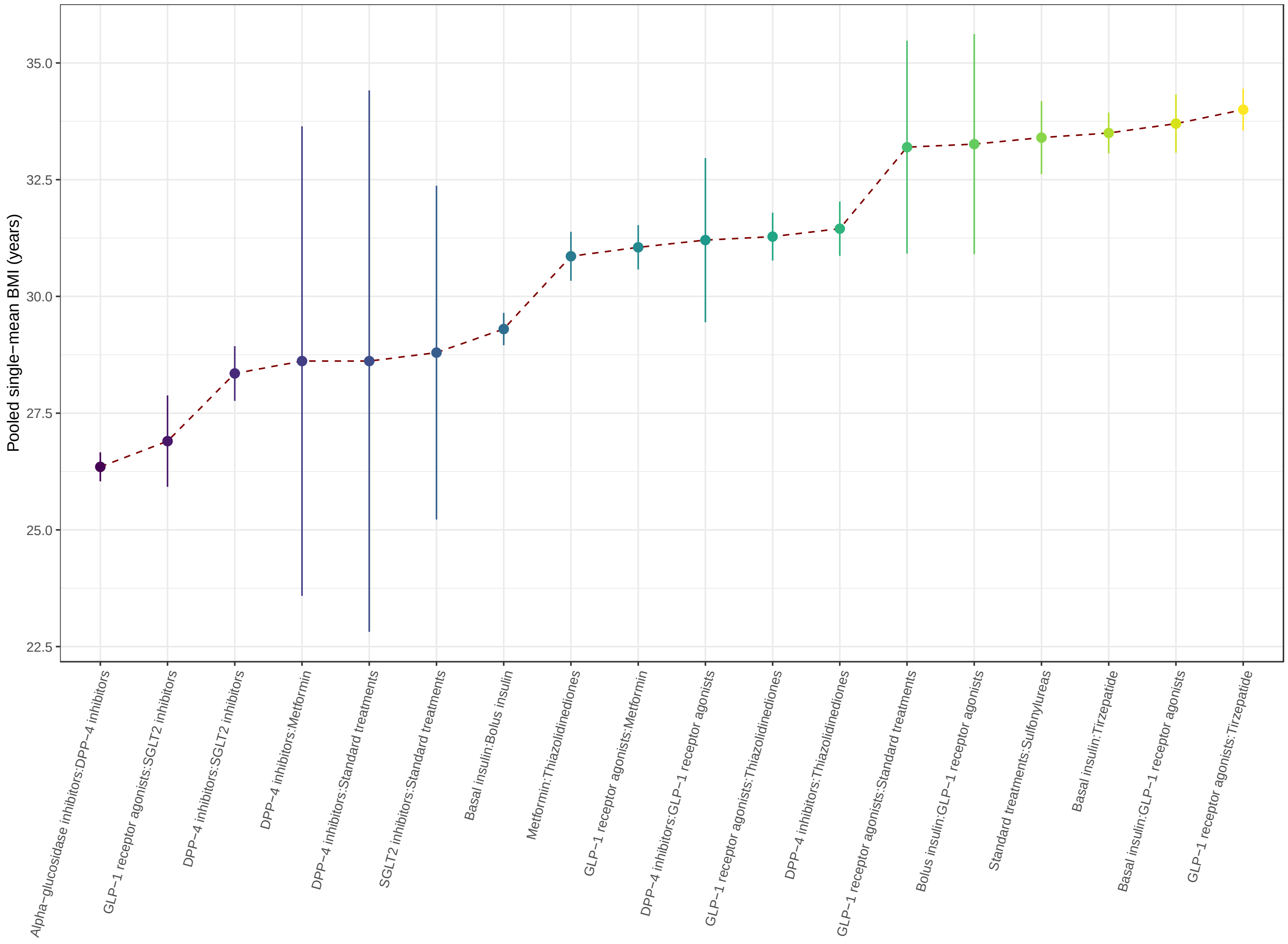

4.5.12.3 Proportion of cardiovascular disease

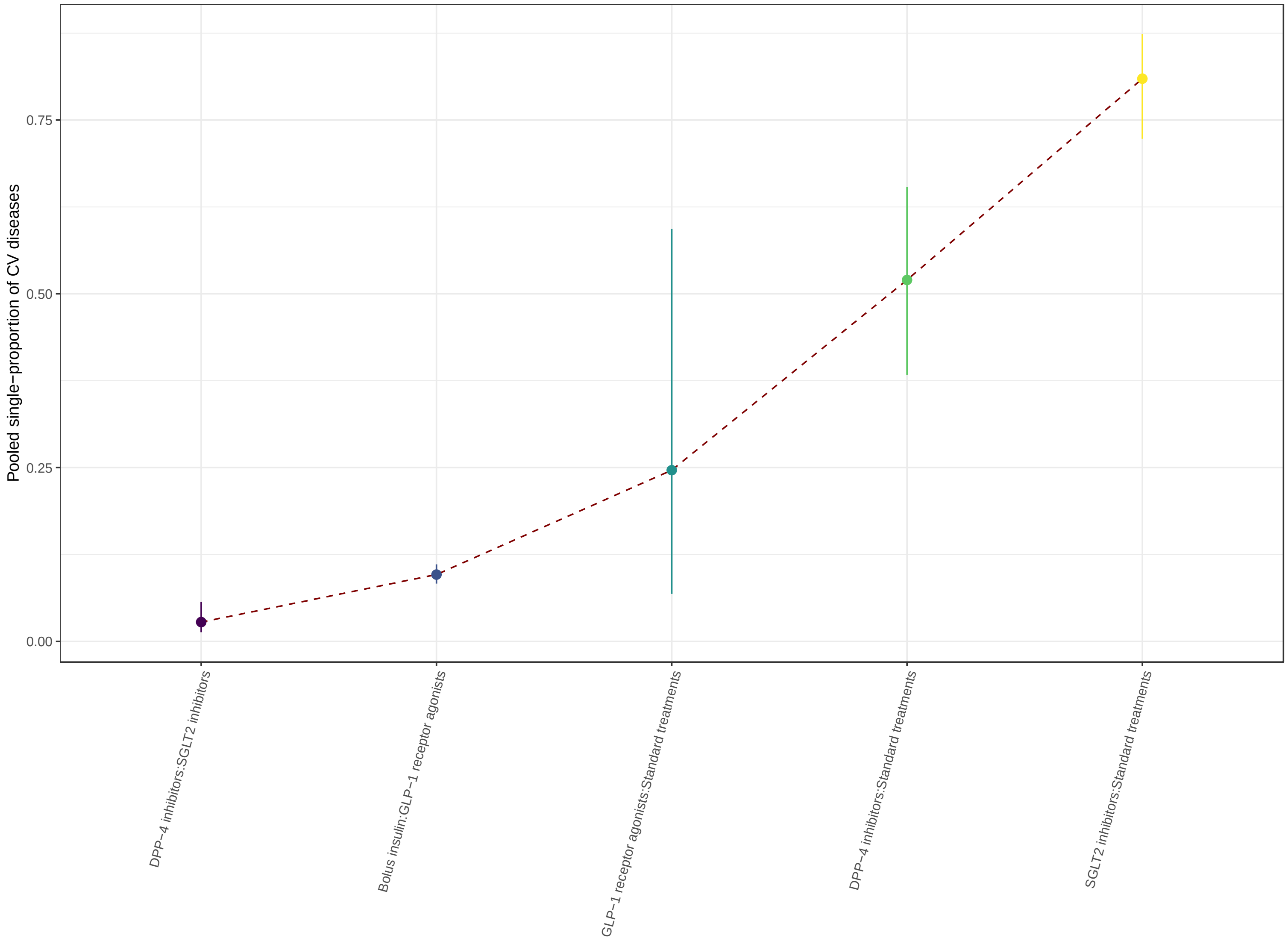

4.5.12.4 Duration of baseline diabetes

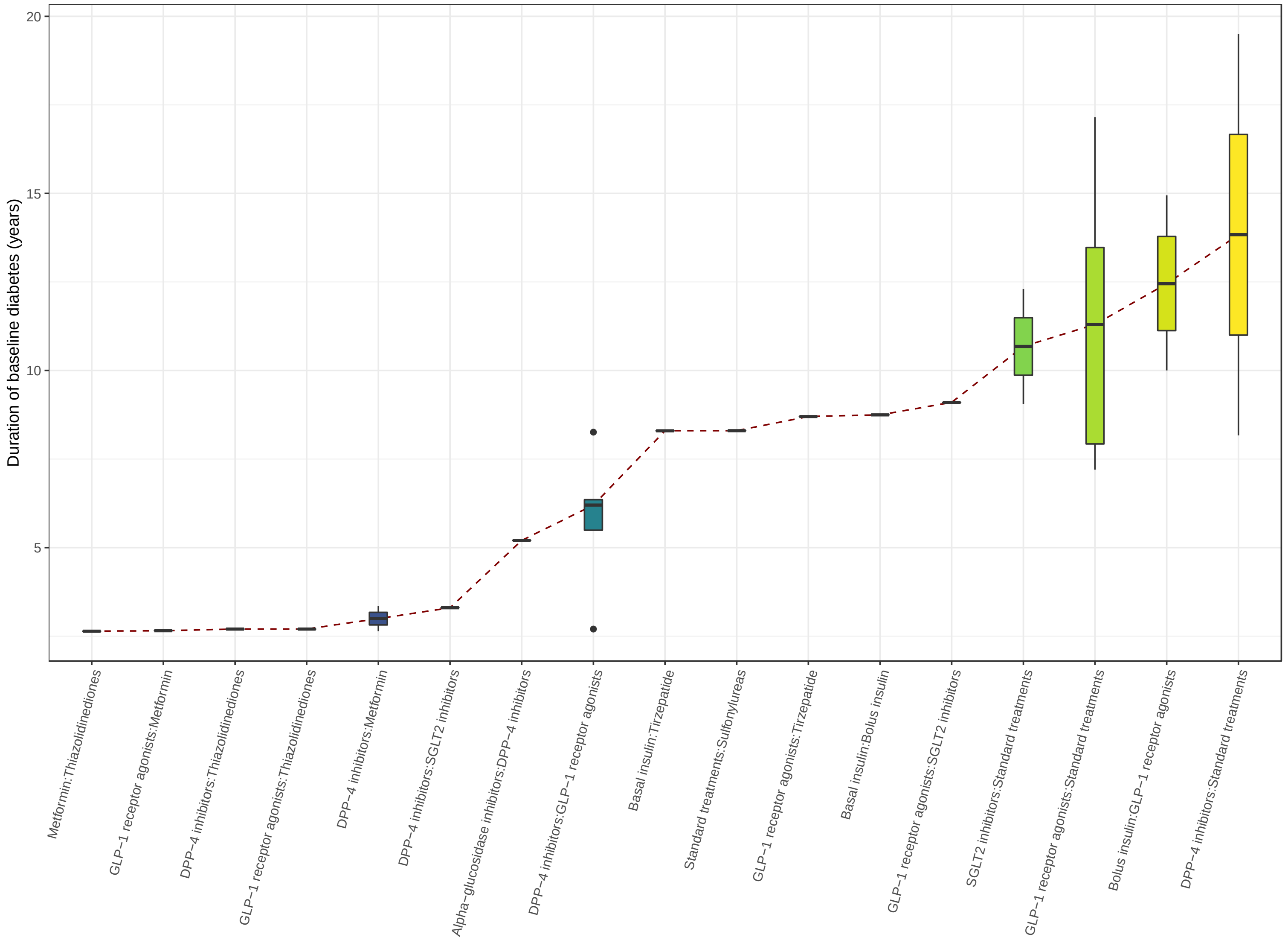

4.5.12.5 HbA1c

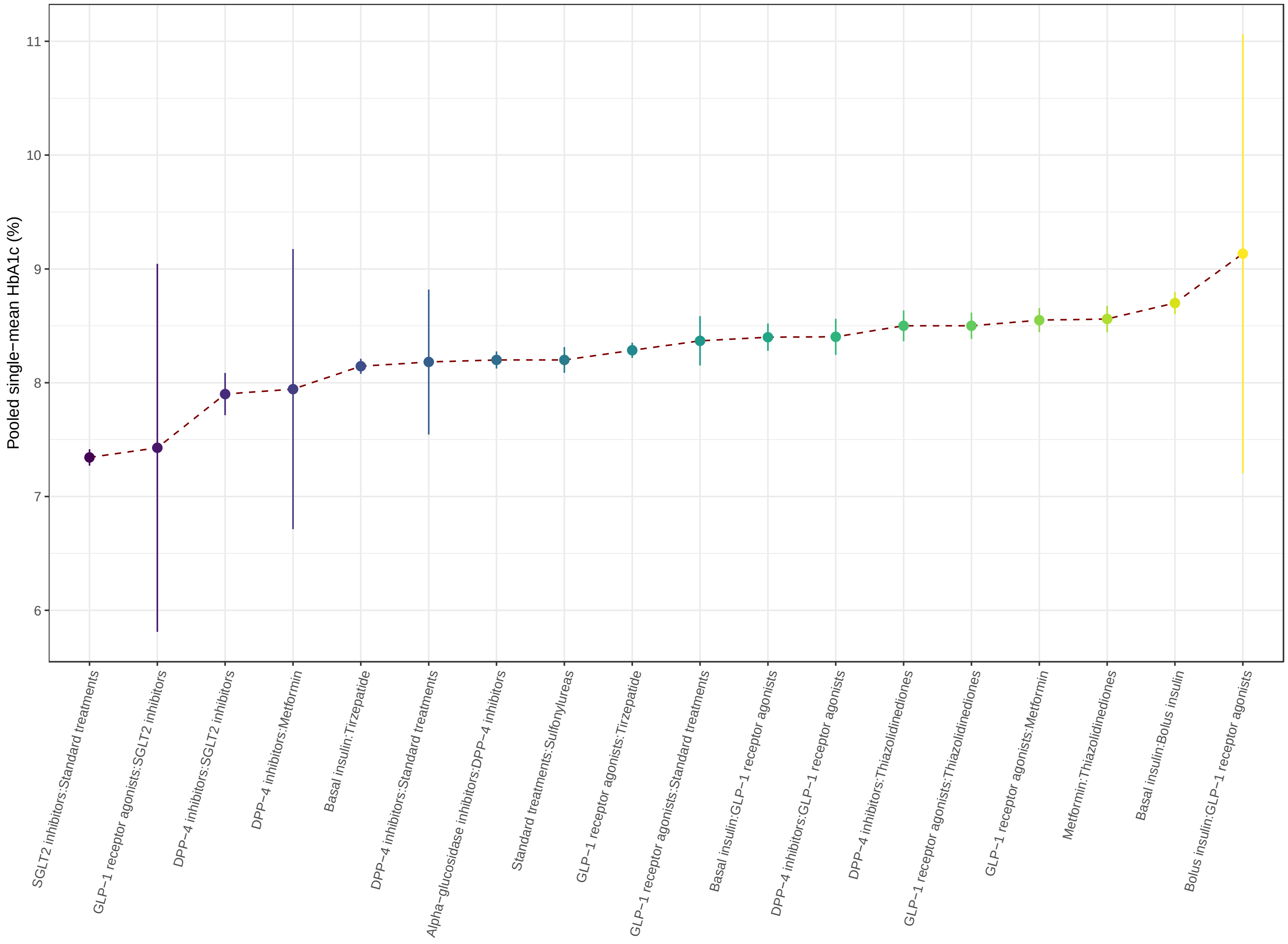

Pooled single−mean HbA1c (%)
11
10
9
8
7
6
SGLT2 inhibitors:Standard treatments
GLP−1 receptor agonists:SGLT2 inhibitors
DPP−4 inhibitors:SGLT2 inhibitors
DPP−4 inhibitors:Metformin
Basal insulin:Tirzepatide
DPP−4 inhibitors:Standard treatments
Alpha−glucosidase inhibitors:DPP−4 inhibitors
Standard treatments:Sulfonylureas
GLP−1 receptor agonists:Tirzepatide
GLP−1 receptor agonists:Standard treatments
Basal insulin:GLP−1 receptor agonists
DPP−4 inhibitors:GLP−1 receptor agonists
DPP−4 inhibitors:Thiazolidinediones
GLP−1 receptor agonists:Thiazolidinediones
GLP−1 receptor agonists:Metformin
Metformin:Thiazolidinediones
Basal insulin:Bolus insulin
Bolus insulin:GLP−1 receptor agonists

4.5.12.6 Proportion of males

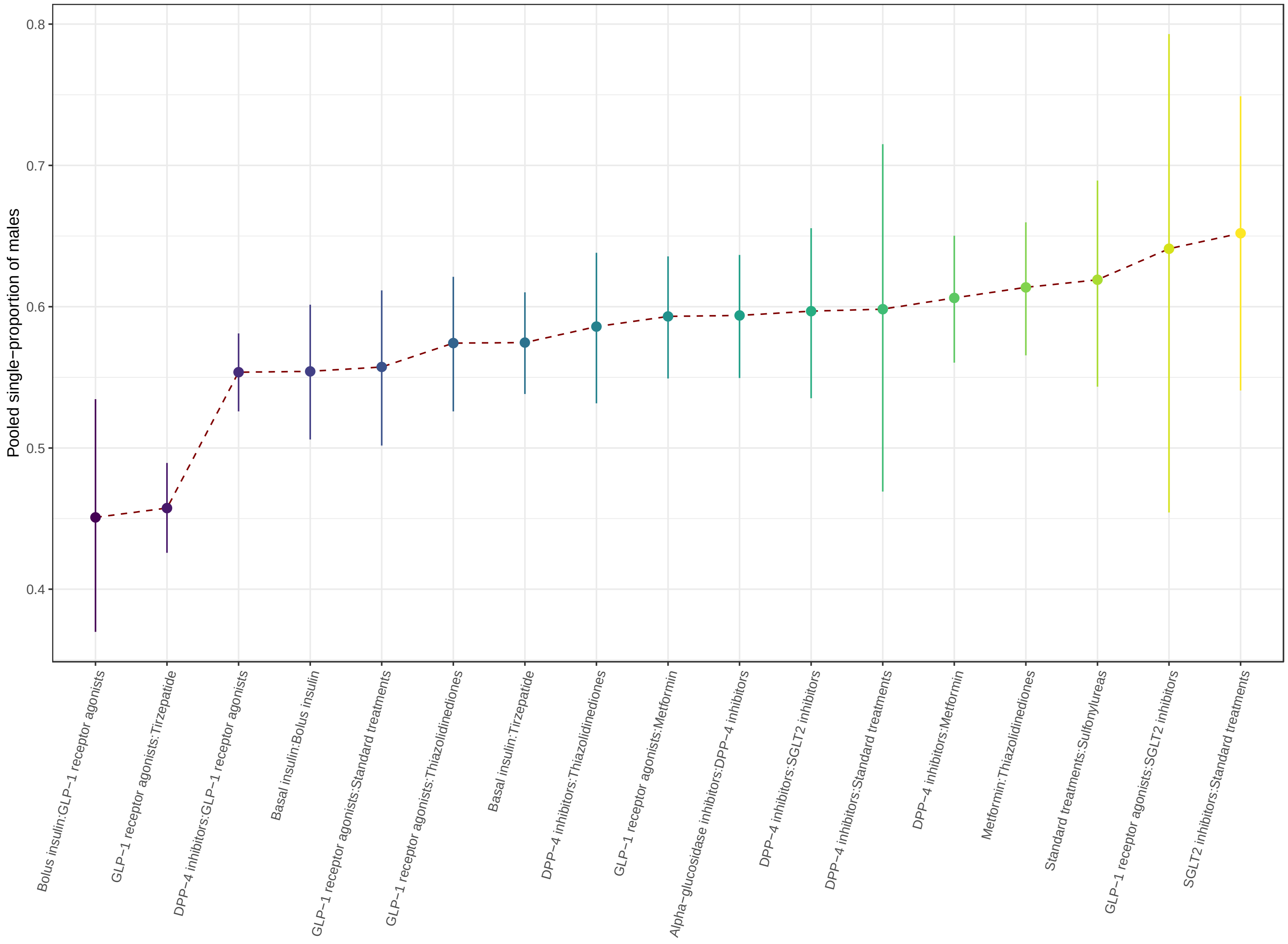

4.5.13 Bodyweight change

4.5.13.1 Age

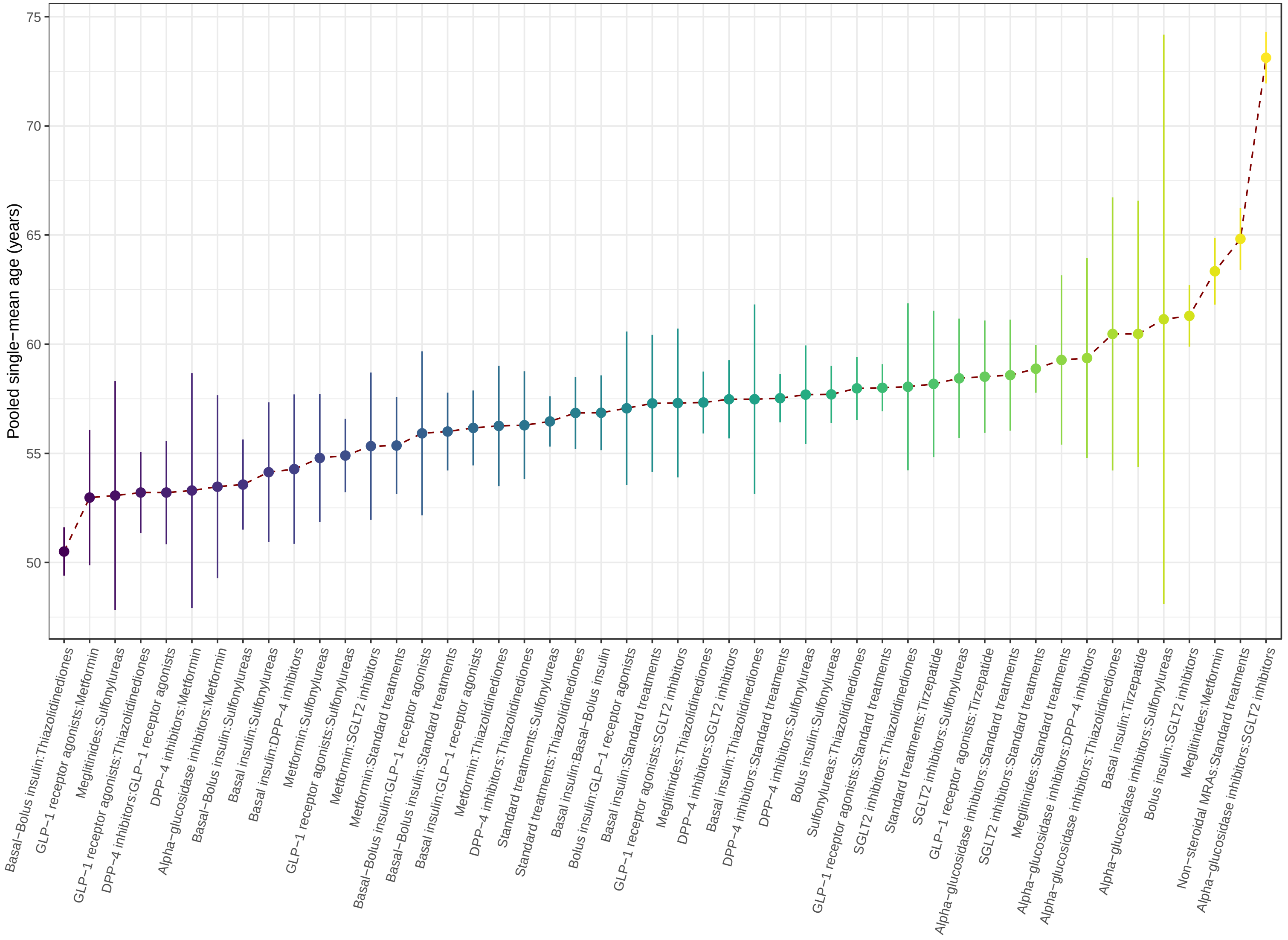

4.5.13.2 Body mass index

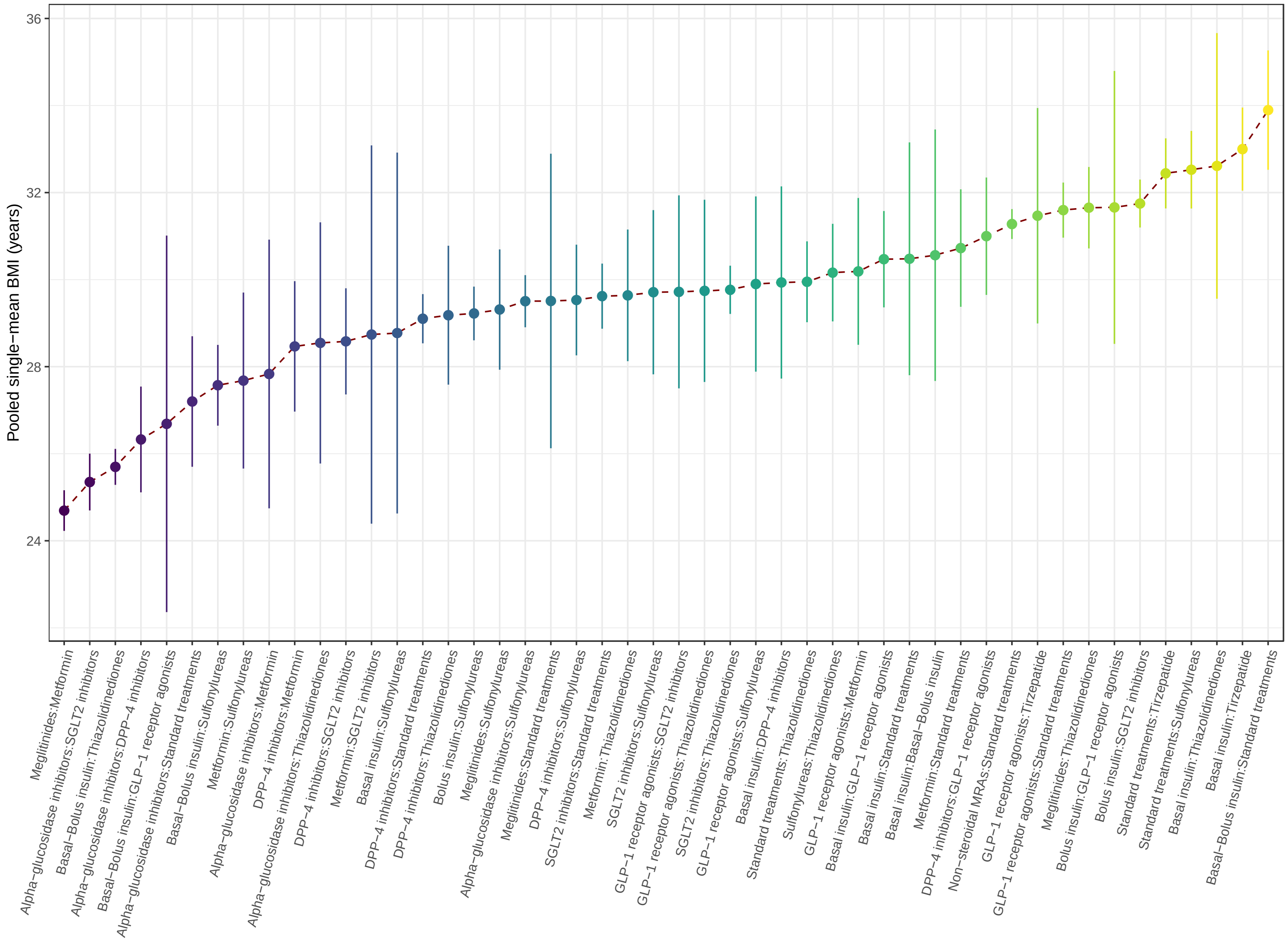

4.5.13.3 Proportion of cardiovascular disease

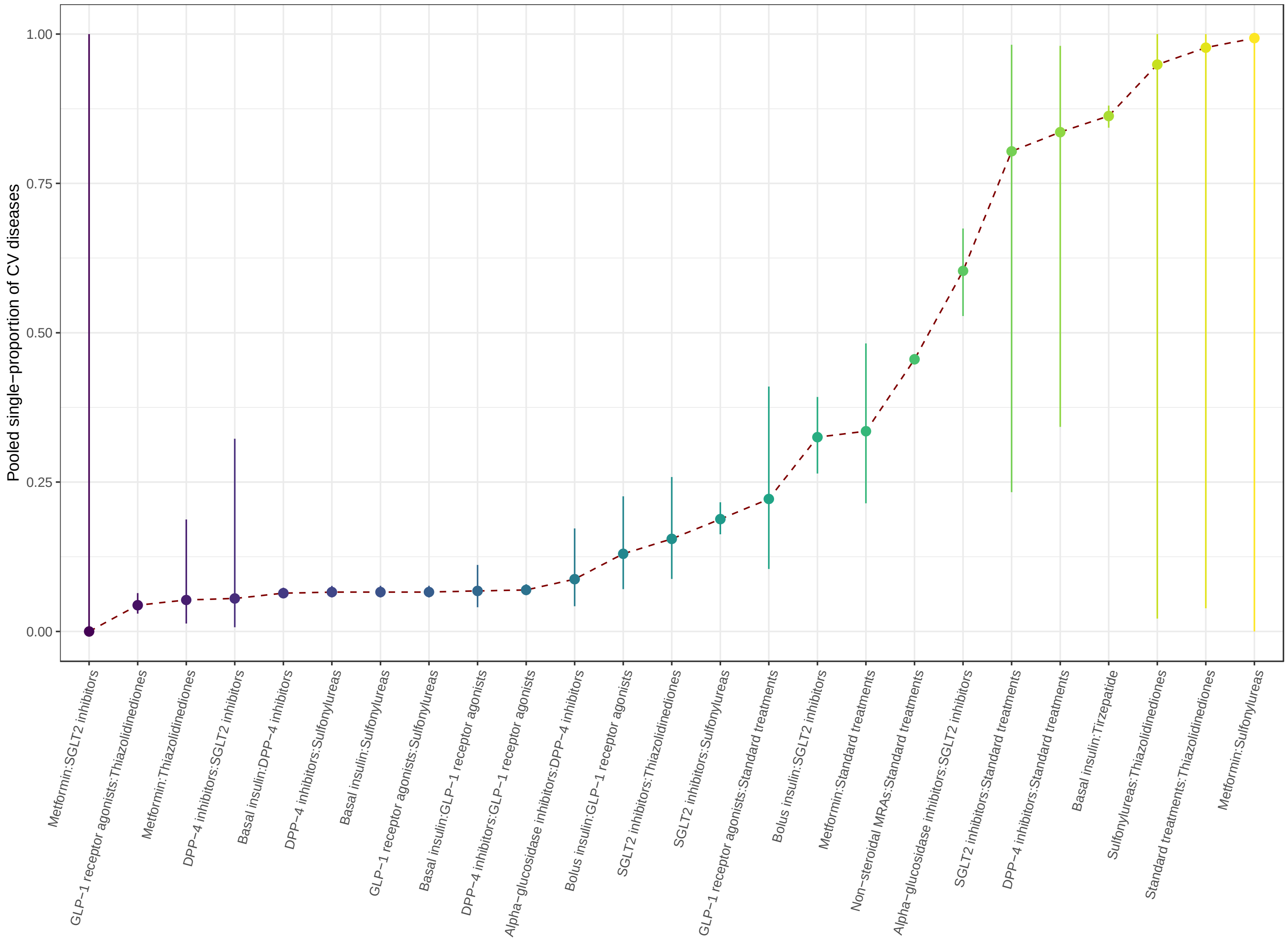

4.5.13.4 Duration of baseline diabetes

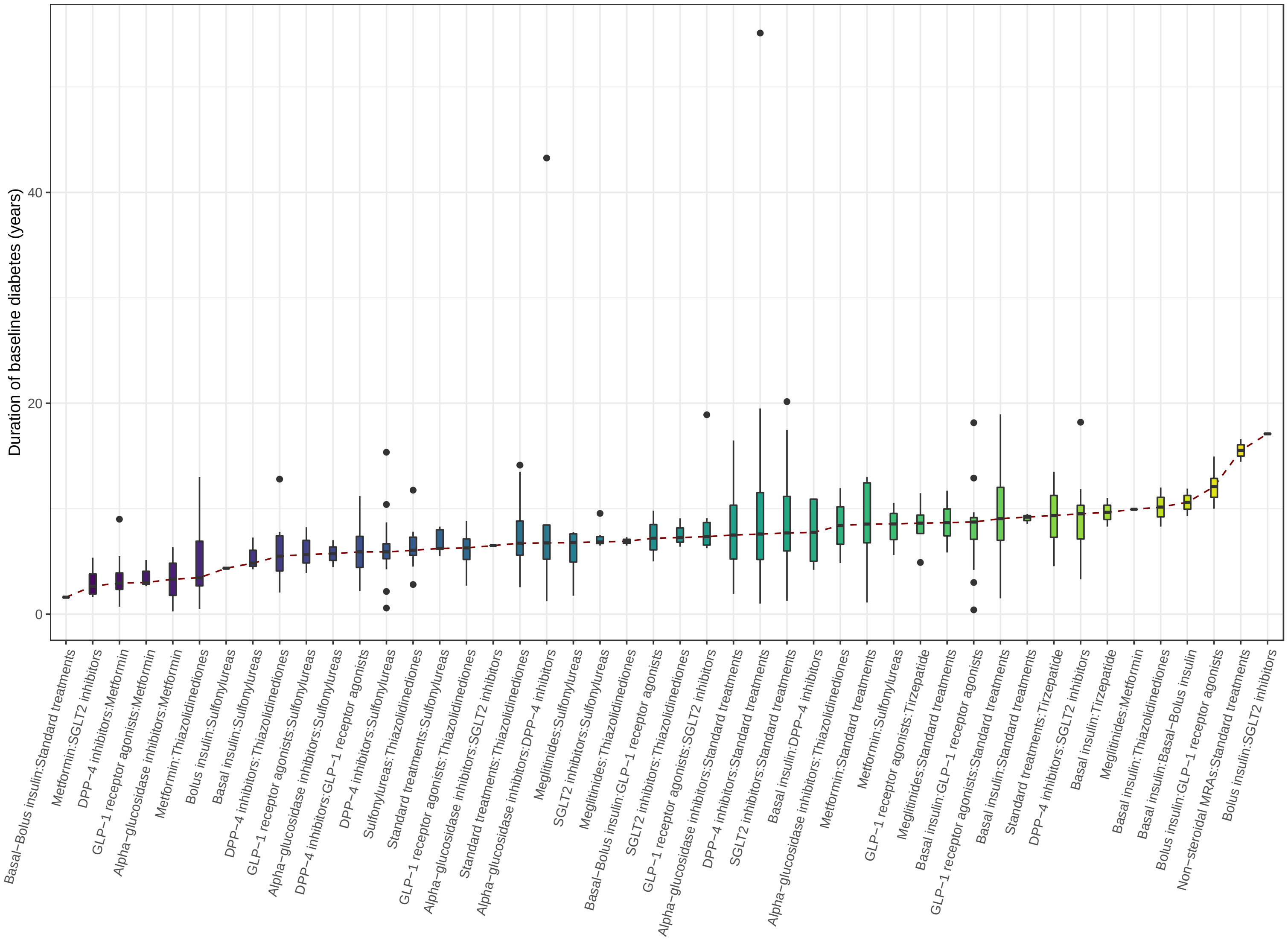

4.5.13.5 HbA1c

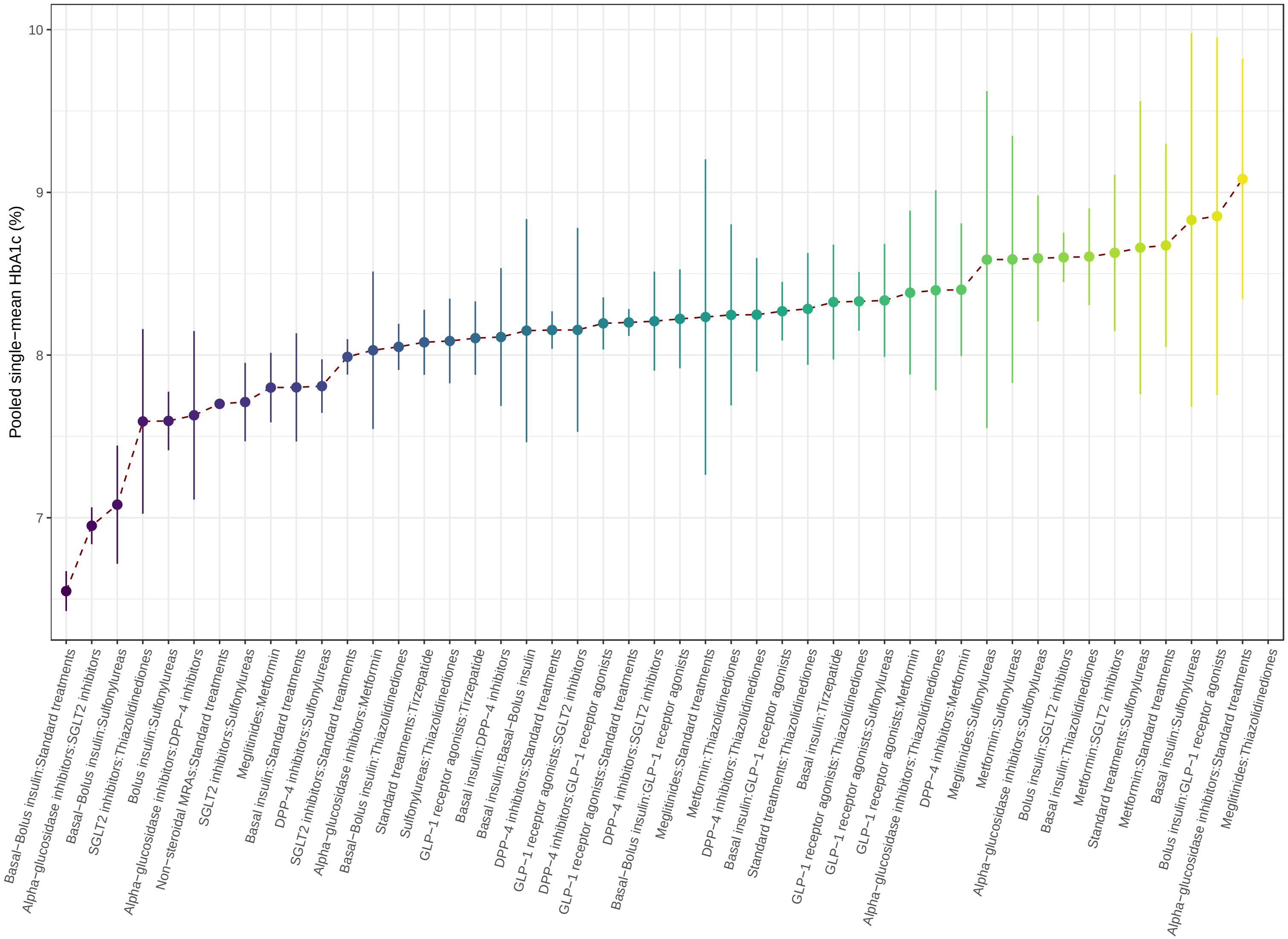

Pooled single−mean HbA1c (%)
10
9
8
7
Basal−Bolus insulin:Standard treatments
Alpha−glucosidase inhibitors:SGLT2 inhibitors
Basal−Bolus insulin:Sulfonylureas
SGLT2 inhibitors:Thiazolidinediones
Bolus insulin:Sulfonylureas
Alpha−glucosidase inhibitors:DPP−4 inhibitors
Non−steroidal MRAs:Standard treatments
SGLT2 inhibitors:Sulfonylureas
Meglitinides:Metformin
Basal insulin:Standard treatments
DPP−4 inhibitors:Sulfonylureas
SGLT2 inhibitors:Standard treatments
Alpha−glucosidase inhibitors:Metformin
Basal−Bolus insulin:Thiazolidinediones
Standard treatments:Tirzepatide
Sulfonylureas:Thiazolidinediones
GLP−1 receptor agonists:Tirzepatide
Basal insulin:DPP−4 inhibitors
Basal insulin:Basal−Bolus insulin
DPP−4 inhibitors:Standard treatments
GLP−1 receptor agonists:SGLT2 inhibitors
DPP−4 inhibitors:GLP−1 receptor agonists
GLP−1 receptor agonists:Standard treatments
DPP−4 inhibitors:SGLT2 inhibitors
Basal−Bolus insulin:GLP−1 receptor agonists
Meglitinides:Standard treatments
Metformin:Thiazolidinediones
DPP−4 inhibitors:Thiazolidinediones
Basal insulin:GLP−1 receptor agonists
Standard treatments:Thiazolidinediones
Basal insulin:Tirzepatide
GLP−1 receptor agonists:Thiazolidinediones
GLP−1 receptor agonists:Sulfonylureas
GLP−1 receptor agonists:Metformin
Alpha−glucosidase inhibitors:Thiazolidinediones
DPP−4 inhibitors:Metformin
Meglitinides:Sulfonylureas
Metformin:Sulfonylureas
Alpha−glucosidase inhibitors:Sulfonylureas
Bolus insulin:SGLT2 inhibitors
Basal insulin:Thiazolidinediones
Metformin:SGLT2 inhibitors
Standard treatments:Sulfonylureas
Metformin:Standard treatments
Basal insulin:Sulfonylureas
Bolus insulin:GLP−1 receptor agonists
Alpha−glucosidase inhibitors:Standard treatments
Meglitinides:Thiazolidinediones

4.5.13.6 Proportion of males

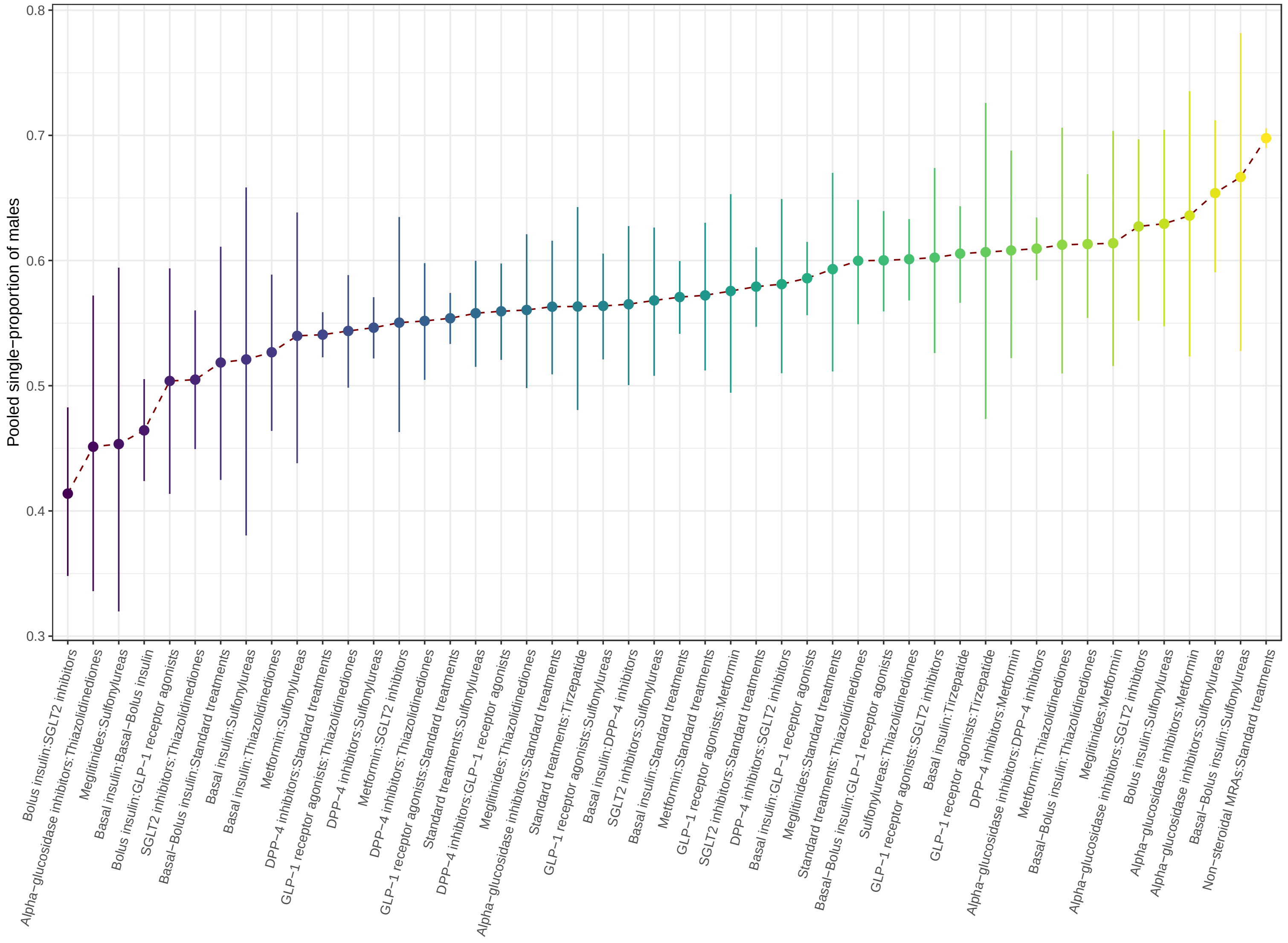

4.6 Incoherence assessments

Notes: The study assessed the incoherence (local inconsistency for each treatment loop) by the ratio of odds ratio (binary outcomes) or difference in mean difference (continuous outcomes) between direct estimates and indirect estimates, and the corresponding statistical tests for the ratio or difference.

Outcome	Comparisons	Ratio of odds ratio	Difference in mean difference	Z statistic	P-value
**All-cause death**	Alpha-glucosidase inhibitors *vs* Basal insulin	NA	NA	NA	NA
**All-cause death**	Alpha-glucosidase inhibitors *vs* Basal-Bolus insulin	NA	NA	NA	NA
**All-cause death**	Alpha-glucosidase inhibitors *vs* Bolus insulin	NA	NA	NA	NA
**All-cause death**	Alpha-glucosidase inhibitors *vs* DPP-4 inhibitors	NA	NA	NA	NA
**All-cause death**	Alpha-glucosidase inhibitors *vs* GLP-1 receptor agonists	NA	NA	NA	NA
**All-cause death**	Alpha-glucosidase inhibitors *vs* Meglitinides	0.51 (0.01 to 18.82)	NA	-0.369	0.712
**All-cause death**	Alpha-glucosidase inhibitors *vs* Metformin	NA	NA	NA	NA
**All-cause death**	Alpha-glucosidase inhibitors *vs* Non-steroidal MRAs	NA	NA	NA	NA
**All-cause death**	Alpha-glucosidase inhibitors *vs* SGLT2 inhibitors	NA	NA	NA	NA
**All-cause death**	Alpha-glucosidase inhibitors *vs* Standard treatments	2.71 (0.3 to 24.66)	NA	0.883	0.377
**All-cause death**	Alpha-glucosidase inhibitors *vs* Sulfonylureas	0.66 (0.08 to 5.77)	NA	-0.376	0.707
**All-cause death**	Alpha-glucosidase inhibitors *vs* Thiazolidinediones	NA	NA	NA	NA
**All-cause death**	Alpha-glucosidase inhibitors *vs* Tirzepatide	NA	NA	NA	NA
**All-cause death**	Basal insulin *vs* Basal-Bolus insulin	8.41 (0.28 to 252.51)	NA	1.227	0.22
**All-cause death**	Basal insulin *vs* Bolus insulin	0.11 (0 to 3.44)	NA	-1.262	0.207
**All-cause death**	Basal insulin *vs* DPP-4 inhibitors	0.84 (0.46 to 1.54)	NA	-0.573	0.567
**All-cause death**	Basal insulin *vs* GLP-1 receptor agonists	1.45 (0.78 to 2.71)	NA	1.173	0.241
**All-cause death**	Basal insulin *vs* Meglitinides	NA	NA	NA	NA
**All-cause death**	Basal insulin *vs* Metformin	NA	NA	NA	NA
**All-cause death**	Basal insulin *vs* Non-steroidal MRAs	NA	NA	NA	NA
**All-cause death**	Basal insulin *vs* SGLT2 inhibitors	NA	NA	NA	NA
**All-cause death**	Basal insulin *vs* Standard treatments	0.29 (0.01 to 7.3)	NA	-0.751	0.453

Outcome	Comparisons	Ratio of odds ratio	Difference in mean difference	Z statistic	P-value
**All-cause death**	Basal insulin *vs* Sulfonylureas	0.94 (0.5 to 1.77)	NA	-0.181	0.857
**All-cause death**	Basal insulin *vs* Thiazolidinediones	NA	NA	NA	NA
**All-cause death**	Basal insulin *vs* Tirzepatide	1.33 (0.28 to 6.23)	NA	0.361	0.718
**All-cause death**	Basal-Bolus insulin *vs* Bolus insulin	NA	NA	NA	NA
**All-cause death**	Basal-Bolus insulin *vs* DPP-4 inhibitors	NA	NA	NA	NA
**All-cause death**	Basal-Bolus insulin *vs* GLP-1 receptor agonists	0.66 (0.04 to 11.88)	NA	-0.278	0.781
**All-cause death**	Basal-Bolus insulin *vs* Meglitinides	NA	NA	NA	NA
**All-cause death**	Basal-Bolus insulin *vs* Metformin	NA	NA	NA	NA
**All-cause death**	Basal-Bolus insulin *vs* Non-steroidal MRAs	NA	NA	NA	NA
**All-cause death**	Basal-Bolus insulin *vs* SGLT2 inhibitors	NA	NA	NA	NA
**All-cause death**	Basal-Bolus insulin *vs* Standard treatments	NA	NA	NA	NA
**All-cause death**	Basal-Bolus insulin *vs* Sulfonylureas	17.08 (0.52 to 558.86)	NA	1.595	0.111
**All-cause death**	Basal-Bolus insulin *vs* Thiazolidinediones	NA	NA	NA	NA
**All-cause death**	Basal-Bolus insulin *vs* Tirzepatide	NA	NA	NA	NA
**All-cause death**	Bolus insulin *vs* DPP-4 inhibitors	NA	NA	NA	NA
**All-cause death**	Bolus insulin *vs* GLP-1 receptor agonists	0.11 (0 to 3.44)	NA	-1.262	0.207
**All-cause death**	Bolus insulin *vs* Meglitinides	NA	NA	NA	NA
**All-cause death**	Bolus insulin *vs* Metformin	NA	NA	NA	NA
**All-cause death**	Bolus insulin *vs* Non-steroidal MRAs	NA	NA	NA	NA
**All-cause death**	Bolus insulin *vs* SGLT2 inhibitors	NA	NA	NA	NA
**All-cause death**	Bolus insulin *vs* Standard treatments	NA	NA	NA	NA
**All-cause death**	Bolus insulin *vs* Sulfonylureas	NA	NA	NA	NA
**All-cause death**	Bolus insulin *vs* Thiazolidinediones	NA	NA	NA	NA
**All-cause death**	Bolus insulin *vs* Tirzepatide	NA	NA	NA	NA
**All-cause death**	DPP-4 inhibitors *vs* GLP-1 receptor agonists	1.2 (0.8 to 1.78)	NA	0.876	0.381

Outcome	Comparisons	Ratio of odds ratio	Difference in mean difference	Z statistic	P-value
**All-cause death**	DPP-4 inhibitors *vs* Meglitinides	NA	NA	NA	NA
**All-cause death**	DPP-4 inhibitors *vs* Metformin	0.91 (0.26 to 3.2)	NA	-0.154	0.877
**All-cause death**	DPP-4 inhibitors *vs* Non-steroidal MRAs	NA	NA	NA	NA
**All-cause death**	DPP-4 inhibitors *vs* SGLT2 inhibitors	0.43 (0.13 to 1.41)	NA	-1.385	0.166
**All-cause death**	DPP-4 inhibitors *vs* Standard treatments	1.09 (0.86 to 1.38)	NA	0.715	0.475
**All-cause death**	DPP-4 inhibitors *vs* Sulfonylureas	0.82 (0.62 to 1.1)	NA	-1.298	0.194
**All-cause death**	DPP-4 inhibitors *vs* Thiazolidinediones	2.01 (0.4 to 10.07)	NA	0.853	0.394
**All-cause death**	DPP-4 inhibitors *vs* Tirzepatide	NA	NA	NA	NA
**All-cause death**	GLP-1 receptor agonists *vs* Meglitinides	NA	NA	NA	NA
**All-cause death**	GLP-1 receptor agonists *vs* Metformin	0.31 (0.01 to 7.76)	NA	-0.71	0.477
**All-cause death**	GLP-1 receptor agonists *vs* Non-steroidal MRAs	NA	NA	NA	NA
**All-cause death**	GLP-1 receptor agonists *vs* SGLT2 inhibitors	1.31 (0.25 to 6.85)	NA	0.323	0.747
**All-cause death**	GLP-1 receptor agonists *vs* Standard treatments	1.17 (0.84 to 1.61)	NA	0.926	0.354
**All-cause death**	GLP-1 receptor agonists *vs* Sulfonylureas	0.84 (0.53 to 1.32)	NA	-0.764	0.445
**All-cause death**	GLP-1 receptor agonists *vs* Thiazolidinediones	0.39 (0.05 to 3.01)	NA	-0.896	0.37
**All-cause death**	GLP-1 receptor agonists *vs* Tirzepatide	0.34 (0.06 to 2.09)	NA	-1.163	0.245
**All-cause death**	Meglitinides *vs* Metformin	0.13 (0 to 4.18)	NA	-1.144	0.253
**All-cause death**	Meglitinides *vs* Non-steroidal MRAs	NA	NA	NA	NA
**All-cause death**	Meglitinides *vs* SGLT2 inhibitors	NA	NA	NA	NA
**All-cause death**	Meglitinides *vs* Standard treatments	1.11 (0.07 to 16.99)	NA	0.076	0.94
**All-cause death**	Meglitinides *vs* Sulfonylureas	1.66 (0.17 to 16.14)	NA	0.439	0.661
**All-cause death**	Meglitinides *vs* Thiazolidinediones	0.89 (0.03 to 27.53)	NA	-0.065	0.948
**All-cause death**	Meglitinides *vs* Tirzepatide	NA	NA	NA	NA
**All-cause death**	Metformin *vs* Non-steroidal MRAs	NA	NA	NA	NA
**All-cause death**	Metformin *vs* SGLT2 inhibitors	1.98 (0.31 to 12.81)	NA	0.72	0.471

Outcome	Comparisons	Ratio of odds ratio	Difference in mean difference	Z statistic	P-value
**All-cause death**	Metformin *vs* Standard treatments	0.8 (0.51 to 1.26)	NA	-0.954	0.34
**All-cause death**	Metformin *vs* Sulfonylureas	1.27 (0.76 to 2.13)	NA	0.9	0.368
**All-cause death**	Metformin *vs* Thiazolidinediones	1.1 (0.65 to 1.87)	NA	0.359	0.719
**All-cause death**	Metformin *vs* Tirzepatide	NA	NA	NA	NA
**All-cause death**	Non-steroidal MRAs *vs* SGLT2 inhibitors	NA	NA	NA	NA
**All-cause death**	Non-steroidal MRAs *vs* Standard treatments	NA	NA	NA	NA
**All-cause death**	Non-steroidal MRAs *vs* Sulfonylureas	NA	NA	NA	NA
**All-cause death**	Non-steroidal MRAs *vs* Thiazolidinediones	NA	NA	NA	NA
**All-cause death**	Non-steroidal MRAs *vs* Tirzepatide	NA	NA	NA	NA
**All-cause death**	SGLT2 inhibitors *vs* Standard treatments	0.88 (0.47 to 1.65)	NA	-0.394	0.694
**All-cause death**	SGLT2 inhibitors *vs* Sulfonylureas	0.98 (0.4 to 2.35)	NA	-0.055	0.956
**All-cause death**	SGLT2 inhibitors *vs* Thiazolidinediones	NA	NA	NA	NA
**All-cause death**	SGLT2 inhibitors *vs* Tirzepatide	NA	NA	NA	NA
**All-cause death**	Sulfonylureas *vs* Standard treatments	1.61 (0.26 to 10.05)	NA	0.514	0.608
**All-cause death**	Thiazolidinediones *vs* Standard treatments	0.83 (0.6 to 1.14)	NA	-1.154	0.248
**All-cause death**	Tirzepatide *vs* Standard treatments	0.31 (0.03 to 3.23)	NA	-0.979	0.328
**All-cause death**	Sulfonylureas *vs* Thiazolidinediones	0.75 (0.53 to 1.06)	NA	-1.606	0.108
**All-cause death**	Sulfonylureas *vs* Tirzepatide	NA	NA	NA	NA
**All-cause death**	Thiazolidinediones *vs* Tirzepatide	NA	NA	NA	NA
**Cardiovascular death**	Alpha-glucosidase inhibitors *vs* Basal insulin	NA	NA	NA	NA
**Cardiovascular death**	Alpha-glucosidase inhibitors *vs* Basal-Bolus insulin	NA	NA	NA	NA
**Cardiovascular death**	Alpha-glucosidase inhibitors *vs* Bolus insulin	NA	NA	NA	NA
**Cardiovascular death**	Alpha-glucosidase inhibitors *vs* DPP-4 inhibitors	NA	NA	NA	NA
**Cardiovascular death**	Alpha-glucosidase inhibitors *vs* GLP-1 receptor agonists	NA	NA	NA	NA
**Cardiovascular death**	Alpha-glucosidase inhibitors *vs* Meglitinides	NA	NA	NA	NA

Outcome	Comparisons	Ratio of odds ratio	Difference in mean difference	Z statistic	P-value
**Cardiovascular death**	Alpha-glucosidase inhibitors *vs* Metformin	NA	NA	NA	NA
**Cardiovascular death**	Alpha-glucosidase inhibitors *vs* Non-steroidal MRAs	NA	NA	NA	NA
**Cardiovascular death**	Alpha-glucosidase inhibitors *vs* SGLT2 inhibitors	NA	NA	NA	NA
**Cardiovascular death**	Alpha-glucosidase inhibitors *vs* Standard treatments	4.35 (0.14 to 130.77)	NA	0.846	0.397
**Cardiovascular death**	Alpha-glucosidase inhibitors *vs* Sulfonylureas	0.29 (0.01 to 6.59)	NA	-0.776	0.438
**Cardiovascular death**	Alpha-glucosidase inhibitors *vs* Thiazolidinediones	NA	NA	NA	NA
**Cardiovascular death**	Alpha-glucosidase inhibitors *vs* Tirzepatide	NA	NA	NA	NA
**Cardiovascular death**	Basal insulin *vs* Basal-Bolus insulin	NA	NA	NA	NA
**Cardiovascular death**	Basal insulin *vs* Bolus insulin	0.08 (0 to 7.16)	NA	-1.113	0.266
**Cardiovascular death**	Basal insulin *vs* DPP-4 inhibitors	0.64 (0.27 to 1.54)	NA	-0.992	0.321
**Cardiovascular death**	Basal insulin *vs* GLP-1 receptor agonists	1.74 (0.71 to 4.25)	NA	1.216	0.224
**Cardiovascular death**	Basal insulin *vs* Meglitinides	NA	NA	NA	NA
**Cardiovascular death**	Basal insulin *vs* Metformin	NA	NA	NA	NA
**Cardiovascular death**	Basal insulin *vs* Non-steroidal MRAs	NA	NA	NA	NA
**Cardiovascular death**	Basal insulin *vs* SGLT2 inhibitors	NA	NA	NA	NA
**Cardiovascular death**	Basal insulin *vs* Standard treatments	NA	NA	NA	NA
**Cardiovascular death**	Basal insulin *vs* Sulfonylureas	1.06 (0.42 to 2.64)	NA	0.115	0.908
**Cardiovascular death**	Basal insulin *vs* Thiazolidinediones	NA	NA	NA	NA
**Cardiovascular death**	Basal insulin *vs* Tirzepatide	3.84 (0.17 to 86.03)	NA	0.848	0.396
**Cardiovascular death**	Basal-Bolus insulin *vs* Bolus insulin	NA	NA	NA	NA
**Cardiovascular death**	Basal-Bolus insulin *vs* DPP-4 inhibitors	NA	NA	NA	NA
**Cardiovascular death**	Basal-Bolus insulin *vs* GLP-1 receptor agonists	0.34 (0 to 33.02)	NA	-0.46	0.645
**Cardiovascular death**	Basal-Bolus insulin *vs* Meglitinides	NA	NA	NA	NA
**Cardiovascular death**	Basal-Bolus insulin *vs* Metformin	NA	NA	NA	NA
**Cardiovascular death**	Basal-Bolus insulin *vs* Non-steroidal MRAs	NA	NA	NA	NA

Outcome	Comparisons	Ratio of odds ratio	Difference in mean difference	Z statistic	P-value
**Cardiovascular death**	Basal-Bolus insulin *vs* SGLT2 inhibitors	NA	NA	NA	NA
**Cardiovascular death**	Basal-Bolus insulin *vs* Standard treatments	NA	NA	NA	NA
**Cardiovascular death**	Basal-Bolus insulin *vs* Sulfonylureas	2.92 (0.03 to 282.27)	NA	0.46	0.645
**Cardiovascular death**	Basal-Bolus insulin *vs* Thiazolidinediones	NA	NA	NA	NA
**Cardiovascular death**	Basal-Bolus insulin *vs* Tirzepatide	NA	NA	NA	NA
**Cardiovascular death**	Bolus insulin *vs* DPP-4 inhibitors	NA	NA	NA	NA
**Cardiovascular death**	Bolus insulin *vs* GLP-1 receptor agonists	0.08 (0 to 7.16)	NA	-1.113	0.266
**Cardiovascular death**	Bolus insulin *vs* Meglitinides	NA	NA	NA	NA
**Cardiovascular death**	Bolus insulin *vs* Metformin	NA	NA	NA	NA
**Cardiovascular death**	Bolus insulin *vs* Non-steroidal MRAs	NA	NA	NA	NA
**Cardiovascular death**	Bolus insulin *vs* SGLT2 inhibitors	NA	NA	NA	NA
**Cardiovascular death**	Bolus insulin *vs* Standard treatments	NA	NA	NA	NA
**Cardiovascular death**	Bolus insulin *vs* Sulfonylureas	NA	NA	NA	NA
**Cardiovascular death**	Bolus insulin *vs* Thiazolidinediones	NA	NA	NA	NA
**Cardiovascular death**	Bolus insulin *vs* Tirzepatide	NA	NA	NA	NA
**Cardiovascular death**	DPP-4 inhibitors *vs* GLP-1 receptor agonists	1.72 (0.9 to 3.27)	NA	1.652	0.098
**Cardiovascular death**	DPP-4 inhibitors *vs* Meglitinides	NA	NA	NA	NA
**Cardiovascular death**	DPP-4 inhibitors *vs* Metformin	2.97 (0.11 to 79.22)	NA	0.65	0.516
**Cardiovascular death**	DPP-4 inhibitors *vs* Non-steroidal MRAs	NA	NA	NA	NA
**Cardiovascular death**	DPP-4 inhibitors *vs* SGLT2 inhibitors	0.25 (0.04 to 1.48)	NA	-1.528	0.127
**Cardiovascular death**	DPP-4 inhibitors *vs* Standard treatments	0.86 (0.57 to 1.29)	NA	-0.732	0.464
**Cardiovascular death**	DPP-4 inhibitors *vs* Sulfonylureas	1.08 (0.61 to 1.89)	NA	0.257	0.797
**Cardiovascular death**	DPP-4 inhibitors *vs* Thiazolidinediones	4.93 (0.51 to 47.99)	NA	1.373	0.17
**Cardiovascular death**	DPP-4 inhibitors *vs* Tirzepatide	NA	NA	NA	NA
**Cardiovascular death**	GLP-1 receptor agonists *vs* Meglitinides	NA	NA	NA	NA

Outcome	Comparisons	Ratio of odds ratio	Difference in mean difference	Z statistic	P-value
**Cardiovascular death**	GLP-1 receptor agonists *vs* Metformin	NA	NA	NA	NA
**Cardiovascular death**	GLP-1 receptor agonists *vs* Non-steroidal MRAs	NA	NA	NA	NA
**Cardiovascular death**	GLP-1 receptor agonists *vs* SGLT2 inhibitors	NA	NA	NA	NA
**Cardiovascular death**	GLP-1 receptor agonists *vs* Standard treatments	1.42 (0.82 to 2.47)	NA	1.245	0.213
**Cardiovascular death**	GLP-1 receptor agonists *vs* Sulfonylureas	0.63 (0.27 to 1.46)	NA	-1.082	0.279
**Cardiovascular death**	GLP-1 receptor agonists *vs* Thiazolidinediones	1.1 (0.02 to 55.96)	NA	0.047	0.963
**Cardiovascular death**	GLP-1 receptor agonists *vs* Tirzepatide	0.26 (0.01 to 5.83)	NA	-0.848	0.396
**Cardiovascular death**	Meglitinides *vs* Metformin	0.35 (0.01 to 16.97)	NA	-0.533	0.594
**Cardiovascular death**	Meglitinides *vs* Non-steroidal MRAs	NA	NA	NA	NA
**Cardiovascular death**	Meglitinides *vs* SGLT2 inhibitors	NA	NA	NA	NA
**Cardiovascular death**	Meglitinides *vs* Standard treatments	1.66 (0.02 to 132.98)	NA	0.225	0.822
**Cardiovascular death**	Meglitinides *vs* Sulfonylureas	0.68 (0.02 to 24.22)	NA	-0.213	0.831
**Cardiovascular death**	Meglitinides *vs* Thiazolidinediones	3.04 (0.07 to 139.68)	NA	0.569	0.57
**Cardiovascular death**	Meglitinides *vs* Tirzepatide	NA	NA	NA	NA
**Cardiovascular death**	Metformin *vs* Non-steroidal MRAs	NA	NA	NA	NA
**Cardiovascular death**	Metformin *vs* SGLT2 inhibitors	1.85 (0.25 to 13.54)	NA	0.602	0.547
**Cardiovascular death**	Metformin *vs* Standard treatments	3.75 (0.51 to 27.56)	NA	1.299	0.194
**Cardiovascular death**	Metformin *vs* Sulfonylureas	0.61 (0.15 to 2.39)	NA	-0.714	0.475
**Cardiovascular death**	Metformin *vs* Thiazolidinediones	0.65 (0.09 to 4.84)	NA	-0.425	0.671
**Cardiovascular death**	Metformin *vs* Tirzepatide	NA	NA	NA	NA
**Cardiovascular death**	Non-steroidal MRAs *vs* SGLT2 inhibitors	NA	NA	NA	NA
**Cardiovascular death**	Non-steroidal MRAs *vs* Standard treatments	NA	NA	NA	NA
**Cardiovascular death**	Non-steroidal MRAs *vs* Sulfonylureas	NA	NA	NA	NA
**Cardiovascular death**	Non-steroidal MRAs *vs* Thiazolidinediones	NA	NA	NA	NA
**Cardiovascular death**	Non-steroidal MRAs *vs* Tirzepatide	NA	NA	NA	NA

Outcome	Comparisons	Ratio of odds ratio	Difference in mean difference	Z statistic	P-value
**Cardiovascular death**	SGLT2 inhibitors *vs* Standard treatments	0.63 (0.2 to 1.97)	NA	-0.789	0.43
**Cardiovascular death**	SGLT2 inhibitors *vs* Sulfonylureas	1.39 (0.17 to 11.46)	NA	0.307	0.759
**Cardiovascular death**	SGLT2 inhibitors *vs* Thiazolidinediones	NA	NA	NA	NA
**Cardiovascular death**	SGLT2 inhibitors *vs* Tirzepatide	NA	NA	NA	NA
**Cardiovascular death**	Sulfonylureas *vs* Standard treatments	3.02 (0.12 to 75.5)	NA	0.672	0.501
**Cardiovascular death**	Thiazolidinediones *vs* Standard treatments	0.85 (0.45 to 1.62)	NA	-0.485	0.628
**Cardiovascular death**	Tirzepatide *vs* Standard treatments	NA	NA	NA	NA
**Cardiovascular death**	Sulfonylureas *vs* Thiazolidinediones	0.74 (0.37 to 1.48)	NA	-0.85	0.395
**Cardiovascular death**	Sulfonylureas *vs* Tirzepatide	NA	NA	NA	NA
**Cardiovascular death**	Thiazolidinediones *vs* Tirzepatide	NA	NA	NA	NA
**Non-fatal myocardial infarction**	Alpha-glucosidase inhibitors *vs* Basal insulin	NA	NA	NA	NA
**Non-fatal myocardial infarction**	Alpha-glucosidase inhibitors *vs* Basal-Bolus insulin	NA	NA	NA	NA
**Non-fatal myocardial infarction**	Alpha-glucosidase inhibitors *vs* Bolus insulin	NA	NA	NA	NA
**Non-fatal myocardial infarction**	Alpha-glucosidase inhibitors *vs* DPP-4 inhibitors	NA	NA	NA	NA
**Non-fatal myocardial infarction**	Alpha-glucosidase inhibitors *vs* GLP-1 receptor agonists	NA	NA	NA	NA
**Non-fatal myocardial infarction**	Alpha-glucosidase inhibitors *vs* Meglitinides	0.77 (0.01 to 110.71)	NA	-0.101	0.92
**Non-fatal myocardial infarction**	Alpha-glucosidase inhibitors *vs* Metformin	NA	NA	NA	NA
**Non-fatal myocardial infarction**	Alpha-glucosidase inhibitors *vs* Non-steroidal MRAs	NA	NA	NA	NA
**Non-fatal myocardial infarction**	Alpha-glucosidase inhibitors *vs* SGLT2 inhibitors	NA	NA	NA	NA
**Non-fatal myocardial infarction**	Alpha-glucosidase inhibitors *vs* Standard treatments	0.81 (0.02 to 33.41)	NA	-0.113	0.91
**Non-fatal myocardial infarction**	Alpha-glucosidase inhibitors *vs* Sulfonylureas	1.35 (0.03 to 56.9)	NA	0.158	0.875
**Non-fatal myocardial infarction**	Alpha-glucosidase inhibitors *vs* Thiazolidinediones	NA	NA	NA	NA
**Non-fatal myocardial infarction**	Alpha-glucosidase inhibitors *vs* Tirzepatide	NA	NA	NA	NA
**Non-fatal myocardial infarction**	Basal insulin *vs* Basal-Bolus insulin	0.96 (0.01 to 94.94)	NA	-0.018	0.986
**Non-fatal myocardial infarction**	Basal insulin *vs* Bolus insulin	0.84 (0.09 to 7.61)	NA	-0.159	0.874

Outcome	Comparisons	Ratio of odds ratio	Difference in mean difference	Z statistic	P-value
**Non-fatal myocardial infarction**	Basal insulin *vs* DPP-4 inhibitors	0.36 (0.01 to 9.75)	NA	-0.606	0.544
**Non-fatal myocardial infarction**	Basal insulin *vs* GLP-1 receptor agonists	1.4 (0.28 to 6.9)	NA	0.414	0.679
**Non-fatal myocardial infarction**	Basal insulin *vs* Meglitinides	NA	NA	NA	NA
**Non-fatal myocardial infarction**	Basal insulin *vs* Metformin	NA	NA	NA	NA
**Non-fatal myocardial infarction**	Basal insulin *vs* Non-steroidal MRAs	NA	NA	NA	NA
**Non-fatal myocardial infarction**	Basal insulin *vs* SGLT2 inhibitors	NA	NA	NA	NA
**Non-fatal myocardial infarction**	Basal insulin *vs* Standard treatments	3.26 (0.12 to 88.2)	NA	0.703	0.482
**Non-fatal myocardial infarction**	Basal insulin *vs* Sulfonylureas	NA	NA	NA	NA
**Non-fatal myocardial infarction**	Basal insulin *vs* Thiazolidinediones	0.3 (0.01 to 8.33)	NA	-0.707	0.48
**Non-fatal myocardial infarction**	Basal insulin *vs* Tirzepatide	NA	NA	NA	NA
**Non-fatal myocardial infarction**	Basal-Bolus insulin *vs* Bolus insulin	NA	NA	NA	NA
**Non-fatal myocardial infarction**	Basal-Bolus insulin *vs* DPP-4 inhibitors	NA	NA	NA	NA
**Non-fatal myocardial infarction**	Basal-Bolus insulin *vs* GLP-1 receptor agonists	0.96 (0.01 to 94.94)	NA	-0.018	0.986
**Non-fatal myocardial infarction**	Basal-Bolus insulin *vs* Meglitinides	NA	NA	NA	NA
**Non-fatal myocardial infarction**	Basal-Bolus insulin *vs* Metformin	NA	NA	NA	NA
**Non-fatal myocardial infarction**	Basal-Bolus insulin *vs* Non-steroidal MRAs	NA	NA	NA	NA
**Non-fatal myocardial infarction**	Basal-Bolus insulin *vs* SGLT2 inhibitors	NA	NA	NA	NA
**Non-fatal myocardial infarction**	Basal-Bolus insulin *vs* Standard treatments	NA	NA	NA	NA
**Non-fatal myocardial infarction**	Basal-Bolus insulin *vs* Sulfonylureas	NA	NA	NA	NA
**Non-fatal myocardial infarction**	Basal-Bolus insulin *vs* Thiazolidinediones	NA	NA	NA	NA
**Non-fatal myocardial infarction**	Basal-Bolus insulin *vs* Tirzepatide	NA	NA	NA	NA
**Non-fatal myocardial infarction**	Bolus insulin *vs* DPP-4 inhibitors	NA	NA	NA	NA
**Non-fatal myocardial infarction**	Bolus insulin *vs* GLP-1 receptor agonists	0.84 (0.09 to 7.61)	NA	-0.159	0.874
**Non-fatal myocardial infarction**	Bolus insulin *vs* Meglitinides	NA	NA	NA	NA
**Non-fatal myocardial infarction**	Bolus insulin *vs* Metformin	NA	NA	NA	NA

Outcome	Comparisons	Ratio of odds ratio	Difference in mean difference	Z statistic	P-value
**Non-fatal myocardial infarction**	Bolus insulin *vs* Non-steroidal MRAs	NA	NA	NA	NA
**Non-fatal myocardial infarction**	Bolus insulin *vs* SGLT2 inhibitors	NA	NA	NA	NA
**Non-fatal myocardial infarction**	Bolus insulin *vs* Standard treatments	NA	NA	NA	NA
**Non-fatal myocardial infarction**	Bolus insulin *vs* Sulfonylureas	NA	NA	NA	NA
**Non-fatal myocardial infarction**	Bolus insulin *vs* Thiazolidinediones	NA	NA	NA	NA
**Non-fatal myocardial infarction**	Bolus insulin *vs* Tirzepatide	NA	NA	NA	NA
**Non-fatal myocardial infarction**	DPP-4 inhibitors *vs* GLP-1 receptor agonists	1 (0.41 to 2.44)	NA	-0.004	0.997
**Non-fatal myocardial infarction**	DPP-4 inhibitors *vs* Meglitinides	NA	NA	NA	NA
**Non-fatal myocardial infarction**	DPP-4 inhibitors *vs* Metformin	0.4 (0.09 to 1.78)	NA	-1.203	0.229
**Non-fatal myocardial infarction**	DPP-4 inhibitors *vs* Non-steroidal MRAs	NA	NA	NA	NA
**Non-fatal myocardial infarction**	DPP-4 inhibitors *vs* SGLT2 inhibitors	0.97 (0.34 to 2.78)	NA	-0.055	0.956
**Non-fatal myocardial infarction**	DPP-4 inhibitors *vs* Standard treatments	1.15 (0.81 to 1.62)	NA	0.773	0.439
**Non-fatal myocardial infarction**	DPP-4 inhibitors *vs* Sulfonylureas	0.85 (0.57 to 1.26)	NA	-0.826	0.409
**Non-fatal myocardial infarction**	DPP-4 inhibitors *vs* Thiazolidinediones	0.97 (0.25 to 3.83)	NA	-0.045	0.964
**Non-fatal myocardial infarction**	DPP-4 inhibitors *vs* Tirzepatide	NA	NA	NA	NA
**Non-fatal myocardial infarction**	GLP-1 receptor agonists *vs* Meglitinides	NA	NA	NA	NA
**Non-fatal myocardial infarction**	GLP-1 receptor agonists *vs* Metformin	1.2 (0.1 to 14.48)	NA	0.14	0.888
**Non-fatal myocardial infarction**	GLP-1 receptor agonists *vs* Non-steroidal MRAs	NA	NA	NA	NA
**Non-fatal myocardial infarction**	GLP-1 receptor agonists *vs* SGLT2 inhibitors	5.03 (0.24 to 105.47)	NA	1.04	0.299
**Non-fatal myocardial infarction**	GLP-1 receptor agonists *vs* Standard treatments	1.07 (0.55 to 2.08)	NA	0.192	0.848
**Non-fatal myocardial infarction**	GLP-1 receptor agonists *vs* Sulfonylureas	1.16 (0.39 to 3.43)	NA	0.274	0.784
**Non-fatal myocardial infarction**	GLP-1 receptor agonists *vs* Thiazolidinediones	0.22 (0.02 to 2.02)	NA	-1.339	0.181
**Non-fatal myocardial infarction**	GLP-1 receptor agonists *vs* Tirzepatide	0.04 (0 to 10.48)	NA	-1.148	0.251
**Non-fatal myocardial infarction**	Meglitinides *vs* Metformin	NA	NA	NA	NA
**Non-fatal myocardial infarction**	Meglitinides *vs* Non-steroidal MRAs	NA	NA	NA	NA

Outcome	Comparisons	Ratio of odds ratio	Difference in mean difference	Z statistic	P-value
**Non-fatal myocardial infarction**	Meglitinides *vs* SGLT2 inhibitors	NA	NA	NA	NA
**Non-fatal myocardial infarction**	Meglitinides *vs* Standard treatments	9.87 (0.24 to 401.24)	NA	1.211	0.226
**Non-fatal myocardial infarction**	Meglitinides *vs* Sulfonylureas	0.11 (0 to 4.29)	NA	-1.187	0.235
**Non-fatal myocardial infarction**	Meglitinides *vs* Thiazolidinediones	NA	NA	NA	NA
**Non-fatal myocardial infarction**	Meglitinides *vs* Tirzepatide	NA	NA	NA	NA
**Non-fatal myocardial infarction**	Metformin *vs* Non-steroidal MRAs	NA	NA	NA	NA
**Non-fatal myocardial infarction**	Metformin *vs* SGLT2 inhibitors	1.5 (0.06 to 37.55)	NA	0.245	0.806
**Non-fatal myocardial infarction**	Metformin *vs* Standard treatments	0.78 (0.46 to 1.33)	NA	-0.9	0.368
**Non-fatal myocardial infarction**	Metformin *vs* Sulfonylureas	1.65 (0.86 to 3.17)	NA	1.517	0.129
**Non-fatal myocardial infarction**	Metformin *vs* Thiazolidinediones	0.86 (0.45 to 1.62)	NA	-0.477	0.633
**Non-fatal myocardial infarction**	Metformin *vs* Tirzepatide	NA	NA	NA	NA
**Non-fatal myocardial infarction**	Non-steroidal MRAs *vs* SGLT2 inhibitors	NA	NA	NA	NA
**Non-fatal myocardial infarction**	Non-steroidal MRAs *vs* Standard treatments	NA	NA	NA	NA
**Non-fatal myocardial infarction**	Non-steroidal MRAs *vs* Sulfonylureas	NA	NA	NA	NA
**Non-fatal myocardial infarction**	Non-steroidal MRAs *vs* Thiazolidinediones	NA	NA	NA	NA
**Non-fatal myocardial infarction**	Non-steroidal MRAs *vs* Tirzepatide	NA	NA	NA	NA
**Non-fatal myocardial infarction**	SGLT2 inhibitors *vs* Standard treatments	1.32 (0.61 to 2.89)	NA	0.704	0.481
**Non-fatal myocardial infarction**	SGLT2 inhibitors *vs* Sulfonylureas	0.65 (0.18 to 2.32)	NA	-0.665	0.506
**Non-fatal myocardial infarction**	SGLT2 inhibitors *vs* Thiazolidinediones	NA	NA	NA	NA
**Non-fatal myocardial infarction**	SGLT2 inhibitors *vs* Tirzepatide	NA	NA	NA	NA
**Non-fatal myocardial infarction**	Sulfonylureas *vs* Standard treatments	0.47 (0.12 to 1.8)	NA	-1.106	0.269
**Non-fatal myocardial infarction**	Thiazolidinediones *vs* Standard treatments	0.86 (0.58 to 1.29)	NA	-0.724	0.469
**Non-fatal myocardial infarction**	Tirzepatide *vs* Standard treatments	0.11 (0 to 8.39)	NA	-1.008	0.313
**Non-fatal myocardial infarction**	Sulfonylureas *vs* Thiazolidinediones	0.87 (0.55 to 1.36)	NA	-0.609	0.543
**Non-fatal myocardial infarction**	Sulfonylureas *vs* Tirzepatide	NA	NA	NA	NA

Outcome	Comparisons	Ratio of odds ratio	Difference in mean difference	Z statistic	P-value
**Non-fatal myocardial infarction**	Thiazolidinediones *vs* Tirzepatide	NA	NA	NA	NA
**Non-fatal stroke**	Alpha-glucosidase inhibitors *vs* Basal insulin	NA	NA	NA	NA
**Non-fatal stroke**	Alpha-glucosidase inhibitors *vs* Basal-Bolus insulin	NA	NA	NA	NA
**Non-fatal stroke**	Alpha-glucosidase inhibitors *vs* Bolus insulin	NA	NA	NA	NA
**Non-fatal stroke**	Alpha-glucosidase inhibitors *vs* DPP-4 inhibitors	NA	NA	NA	NA
**Non-fatal stroke**	Alpha-glucosidase inhibitors *vs* GLP-1 receptor agonists	NA	NA	NA	NA
**Non-fatal stroke**	Alpha-glucosidase inhibitors *vs* Meglitinides	0.71 (0 to 154.12)	NA	-0.124	0.901
**Non-fatal stroke**	Alpha-glucosidase inhibitors *vs* Metformin	NA	NA	NA	NA
**Non-fatal stroke**	Alpha-glucosidase inhibitors *vs* Non-steroidal MRAs	NA	NA	NA	NA
**Non-fatal stroke**	Alpha-glucosidase inhibitors *vs* SGLT2 inhibitors	NA	NA	NA	NA
**Non-fatal stroke**	Alpha-glucosidase inhibitors *vs* Standard treatments	NA	NA	NA	NA
**Non-fatal stroke**	Alpha-glucosidase inhibitors *vs* Sulfonylureas	1.41 (0.01 to 304.57)	NA	0.124	0.901
**Non-fatal stroke**	Alpha-glucosidase inhibitors *vs* Thiazolidinediones	NA	NA	NA	NA
**Non-fatal stroke**	Basal insulin *vs* Basal-Bolus insulin	2.31 (0.07 to 77.87)	NA	0.467	0.64
**Non-fatal stroke**	Basal insulin *vs* Bolus insulin	NA	NA	NA	NA
**Non-fatal stroke**	Basal insulin *vs* DPP-4 inhibitors	4.45 (0.16 to 123.88)	NA	0.88	0.379
**Non-fatal stroke**	Basal insulin *vs* GLP-1 receptor agonists	0.62 (0.07 to 5.2)	NA	-0.437	0.662
**Non-fatal stroke**	Basal insulin *vs* Meglitinides	NA	NA	NA	NA
**Non-fatal stroke**	Basal insulin *vs* Metformin	NA	NA	NA	NA
**Non-fatal stroke**	Basal insulin *vs* Non-steroidal MRAs	NA	NA	NA	NA
**Non-fatal stroke**	Basal insulin *vs* SGLT2 inhibitors	NA	NA	NA	NA
**Non-fatal stroke**	Basal insulin *vs* Standard treatments	NA	NA	NA	NA
**Non-fatal stroke**	Basal insulin *vs* Sulfonylureas	NA	NA	NA	NA
**Non-fatal stroke**	Basal insulin *vs* Thiazolidinediones	0.33 (0.01 to 9.63)	NA	-0.647	0.517
**Non-fatal stroke**	Basal-Bolus insulin *vs* Bolus insulin	NA	NA	NA	NA

Outcome	Comparisons	Ratio of odds ratio	Difference in mean difference	Z statistic	P-value
**Non-fatal stroke**	Basal-Bolus insulin *vs* DPP-4 inhibitors	NA	NA	NA	NA
**Non-fatal stroke**	Basal-Bolus insulin *vs* GLP-1 receptor agonists	2 (0.06 to 72.22)	NA	0.379	0.705
**Non-fatal stroke**	Basal-Bolus insulin *vs* Meglitinides	NA	NA	NA	NA
**Non-fatal stroke**	Basal-Bolus insulin *vs* Metformin	NA	NA	NA	NA
**Non-fatal stroke**	Basal-Bolus insulin *vs* Non-steroidal MRAs	NA	NA	NA	NA
**Non-fatal stroke**	Basal-Bolus insulin *vs* SGLT2 inhibitors	NA	NA	NA	NA
**Non-fatal stroke**	Basal-Bolus insulin *vs* Standard treatments	NA	NA	NA	NA
**Non-fatal stroke**	Basal-Bolus insulin *vs* Sulfonylureas	NA	NA	NA	NA
**Non-fatal stroke**	Basal-Bolus insulin *vs* Thiazolidinediones	1.85 (0.05 to 67.43)	NA	0.337	0.736
**Non-fatal stroke**	Bolus insulin *vs* DPP-4 inhibitors	NA	NA	NA	NA
**Non-fatal stroke**	Bolus insulin *vs* GLP-1 receptor agonists	NA	NA	NA	NA
**Non-fatal stroke**	Bolus insulin *vs* Meglitinides	NA	NA	NA	NA
**Non-fatal stroke**	Bolus insulin *vs* Metformin	NA	NA	NA	NA
**Non-fatal stroke**	Bolus insulin *vs* Non-steroidal MRAs	NA	NA	NA	NA
**Non-fatal stroke**	Bolus insulin *vs* SGLT2 inhibitors	NA	NA	NA	NA
**Non-fatal stroke**	Bolus insulin *vs* Standard treatments	NA	NA	NA	NA
**Non-fatal stroke**	Bolus insulin *vs* Sulfonylureas	NA	NA	NA	NA
**Non-fatal stroke**	Bolus insulin *vs* Thiazolidinediones	NA	NA	NA	NA
**Non-fatal stroke**	DPP-4 inhibitors *vs* GLP-1 receptor agonists	0.92 (0.32 to 2.68)	NA	-0.151	0.88
**Non-fatal stroke**	DPP-4 inhibitors *vs* Meglitinides	NA	NA	NA	NA
**Non-fatal stroke**	DPP-4 inhibitors *vs* Metformin	0.84 (0.2 to 3.46)	NA	-0.243	0.808
**Non-fatal stroke**	DPP-4 inhibitors *vs* Non-steroidal MRAs	NA	NA	NA	NA
**Non-fatal stroke**	DPP-4 inhibitors *vs* SGLT2 inhibitors	1.01 (0.3 to 3.38)	NA	0.023	0.982
**Non-fatal stroke**	DPP-4 inhibitors *vs* Standard treatments	1.29 (0.86 to 1.93)	NA	1.248	0.212
**Non-fatal stroke**	DPP-4 inhibitors *vs* Sulfonylureas	0.77 (0.48 to 1.22)	NA	-1.113	0.266

Outcome	Comparisons	Ratio of odds ratio	Difference in mean difference	Z statistic	P-value
**Non-fatal stroke**	DPP-4 inhibitors *vs* Thiazolidinediones	0.65 (0.18 to 2.31)	NA	-0.671	0.502
**Non-fatal stroke**	GLP-1 receptor agonists *vs* Meglitinides	NA	NA	NA	NA
**Non-fatal stroke**	GLP-1 receptor agonists *vs* Metformin	0.8 (0.08 to 7.86)	NA	-0.196	0.845
**Non-fatal stroke**	GLP-1 receptor agonists *vs* Non-steroidal MRAs	NA	NA	NA	NA
**Non-fatal stroke**	GLP-1 receptor agonists *vs* SGLT2 inhibitors	4.33 (0.7 to 26.65)	NA	1.58	0.114
**Non-fatal stroke**	GLP-1 receptor agonists *vs* Standard treatments	0.73 (0.34 to 1.55)	NA	-0.819	0.413
**Non-fatal stroke**	GLP-1 receptor agonists *vs* Sulfonylureas	1.07 (0.26 to 4.46)	NA	0.094	0.925
**Non-fatal stroke**	GLP-1 receptor agonists *vs* Thiazolidinediones	0.62 (0.1 to 3.99)	NA	-0.505	0.613
**Non-fatal stroke**	Meglitinides *vs* Metformin	NA	NA	NA	NA
**Non-fatal stroke**	Meglitinides *vs* Non-steroidal MRAs	NA	NA	NA	NA
**Non-fatal stroke**	Meglitinides *vs* SGLT2 inhibitors	NA	NA	NA	NA
**Non-fatal stroke**	Meglitinides *vs* Standard treatments	0.09 (0 to 4.7)	NA	-1.201	0.23
**Non-fatal stroke**	Meglitinides *vs* Sulfonylureas	11.57 (0.21 to 629.04)	NA	1.201	0.23
**Non-fatal stroke**	Meglitinides *vs* Thiazolidinediones	NA	NA	NA	NA
**Non-fatal stroke**	Metformin *vs* Non-steroidal MRAs	NA	NA	NA	NA
**Non-fatal stroke**	Metformin *vs* SGLT2 inhibitors	NA	NA	NA	NA
**Non-fatal stroke**	Metformin *vs* Standard treatments	0.99 (0.53 to 1.86)	NA	-0.031	0.975
**Non-fatal stroke**	Metformin *vs* Sulfonylureas	0.92 (0.46 to 1.83)	NA	-0.239	0.811
**Non-fatal stroke**	Metformin *vs* Thiazolidinediones	1.15 (0.54 to 2.46)	NA	0.364	0.716
**Non-fatal stroke**	Non-steroidal MRAs *vs* SGLT2 inhibitors	NA	NA	NA	NA
**Non-fatal stroke**	Non-steroidal MRAs *vs* Standard treatments	NA	NA	NA	NA
**Non-fatal stroke**	Non-steroidal MRAs *vs* Sulfonylureas	NA	NA	NA	NA
**Non-fatal stroke**	Non-steroidal MRAs *vs* Thiazolidinediones	NA	NA	NA	NA
**Non-fatal stroke**	SGLT2 inhibitors *vs* Standard treatments	0.85 (0.38 to 1.89)	NA	-0.4	0.689
**Non-fatal stroke**	SGLT2 inhibitors *vs* Sulfonylureas	2.58 (0.77 to 8.65)	NA	1.532	0.126

Outcome	Comparisons	Ratio of odds ratio	Difference in mean difference	Z statistic	P-value
**Non-fatal stroke**	SGLT2 inhibitors *vs* Thiazolidinediones	NA	NA	NA	NA
**Non-fatal stroke**	Sulfonylureas *vs* Standard treatments	0.81 (0.18 to 3.67)	NA	-0.266	0.79
**Non-fatal stroke**	Thiazolidinediones *vs* Standard treatments	0.82 (0.52 to 1.31)	NA	-0.814	0.415
**Non-fatal stroke**	Sulfonylureas *vs* Thiazolidinediones	0.81 (0.48 to 1.37)	NA	-0.798	0.425
**Hospitalisation for heart failure**	Alpha-glucosidase inhibitors *vs* Basal insulin	NA	NA	NA	NA
**Hospitalisation for heart failure**	Alpha-glucosidase inhibitors *vs* Bolus insulin	NA	NA	NA	NA
**Hospitalisation for heart failure**	Alpha-glucosidase inhibitors *vs* DPP-4 inhibitors	NA	NA	NA	NA
**Hospitalisation for heart failure**	Alpha-glucosidase inhibitors *vs* GLP-1 receptor agonists	NA	NA	NA	NA
**Hospitalisation for heart failure**	Alpha-glucosidase inhibitors *vs* Metformin	NA	NA	NA	NA
**Hospitalisation for heart failure**	Alpha-glucosidase inhibitors *vs* Non-steroidal MRAs	NA	NA	NA	NA
**Hospitalisation for heart failure**	Alpha-glucosidase inhibitors *vs* SGLT2 inhibitors	NA	NA	NA	NA
**Hospitalisation for heart failure**	Alpha-glucosidase inhibitors *vs* Standard treatments	NA	NA	NA	NA
**Hospitalisation for heart failure**	Alpha-glucosidase inhibitors *vs* Sulfonylureas	NA	NA	NA	NA
**Hospitalisation for heart failure**	Alpha-glucosidase inhibitors *vs* Thiazolidinediones	NA	NA	NA	NA
**Hospitalisation for heart failure**	Alpha-glucosidase inhibitors *vs* Tirzepatide	NA	NA	NA	NA
**Hospitalisation for heart failure**	Basal insulin *vs* Bolus insulin	NA	NA	NA	NA
**Hospitalisation for heart failure**	Basal insulin *vs* DPP-4 inhibitors	0.93 (0.39 to 2.22)	NA	-0.152	0.879
**Hospitalisation for heart failure**	Basal insulin *vs* GLP-1 receptor agonists	2.67 (1.13 to 6.27)	NA	2.246	0.025
**Hospitalisation for heart failure**	Basal insulin *vs* Metformin	NA	NA	NA	NA
**Hospitalisation for heart failure**	Basal insulin *vs* Non-steroidal MRAs	NA	NA	NA	NA
**Hospitalisation for heart failure**	Basal insulin *vs* SGLT2 inhibitors	NA	NA	NA	NA
**Hospitalisation for heart failure**	Basal insulin *vs* Standard treatments	0.34 (0.03 to 3.38)	NA	-0.926	0.354
**Hospitalisation for heart failure**	Basal insulin *vs* Sulfonylureas	0.73 (0.29 to 1.84)	NA	-0.67	0.503
**Hospitalisation for heart failure**	Basal insulin *vs* Thiazolidinediones	0.56 (0.02 to 14.34)	NA	-0.351	0.726
**Hospitalisation for heart failure**	Basal insulin *vs* Tirzepatide	NA	NA	NA	NA

Outcome	Comparisons	Ratio of odds ratio	Difference in mean difference	Z statistic	P-value
**Hospitalisation for heart failure**	Bolus insulin *vs* DPP-4 inhibitors	NA	NA	NA	NA
**Hospitalisation for heart failure**	Bolus insulin *vs* GLP-1 receptor agonists	NA	NA	NA	NA
**Hospitalisation for heart failure**	Bolus insulin *vs* Metformin	NA	NA	NA	NA
**Hospitalisation for heart failure**	Bolus insulin *vs* Non-steroidal MRAs	NA	NA	NA	NA
**Hospitalisation for heart failure**	Bolus insulin *vs* SGLT2 inhibitors	NA	NA	NA	NA
**Hospitalisation for heart failure**	Bolus insulin *vs* Standard treatments	NA	NA	NA	NA
**Hospitalisation for heart failure**	Bolus insulin *vs* Sulfonylureas	NA	NA	NA	NA
**Hospitalisation for heart failure**	Bolus insulin *vs* Thiazolidinediones	NA	NA	NA	NA
**Hospitalisation for heart failure**	Bolus insulin *vs* Tirzepatide	NA	NA	NA	NA
**Hospitalisation for heart failure**	DPP-4 inhibitors *vs* GLP-1 receptor agonists	1.52 (0.9 to 2.55)	NA	1.578	0.115
**Hospitalisation for heart failure**	DPP-4 inhibitors *vs* Metformin	2.41 (0.03 to 180.1)	NA	0.399	0.69
**Hospitalisation for heart failure**	DPP-4 inhibitors *vs* Non-steroidal MRAs	NA	NA	NA	NA
**Hospitalisation for heart failure**	DPP-4 inhibitors *vs* SGLT2 inhibitors	0.25 (0.01 to 6.2)	NA	-0.846	0.398
**Hospitalisation for heart failure**	DPP-4 inhibitors *vs* Standard treatments	0.77 (0.52 to 1.14)	NA	-1.305	0.192
**Hospitalisation for heart failure**	DPP-4 inhibitors *vs* Sulfonylureas	1.34 (0.77 to 2.33)	NA	1.022	0.307
**Hospitalisation for heart failure**	DPP-4 inhibitors *vs* Thiazolidinediones	0.48 (0.02 to 12.05)	NA	-0.443	0.658
**Hospitalisation for heart failure**	DPP-4 inhibitors *vs* Tirzepatide	NA	NA	NA	NA
**Hospitalisation for heart failure**	GLP-1 receptor agonists *vs* Metformin	0.23 (0.01 to 6.57)	NA	-0.866	0.387
**Hospitalisation for heart failure**	GLP-1 receptor agonists *vs* Non-steroidal MRAs	NA	NA	NA	NA
**Hospitalisation for heart failure**	GLP-1 receptor agonists *vs* SGLT2 inhibitors	0.36 (0.03 to 4.04)	NA	-0.824	0.41
**Hospitalisation for heart failure**	GLP-1 receptor agonists *vs* Standard treatments	1.59 (1 to 2.5)	NA	1.978	0.048
**Hospitalisation for heart failure**	GLP-1 receptor agonists *vs* Sulfonylureas	0.56 (0.29 to 1.06)	NA	-1.793	0.073
**Hospitalisation for heart failure**	GLP-1 receptor agonists *vs* Thiazolidinediones	0.37 (0.01 to 9.2)	NA	-0.607	0.544
**Hospitalisation for heart failure**	GLP-1 receptor agonists *vs* Tirzepatide	NA	NA	NA	NA
**Hospitalisation for heart failure**	Metformin *vs* Non-steroidal MRAs	NA	NA	NA	NA

Outcome	Comparisons	Ratio of odds ratio	Difference in mean difference	Z statistic	P-value
**Hospitalisation for heart failure**	Metformin *vs* SGLT2 inhibitors	0.56 (0.01 to 23.44)	NA	-0.301	0.763
**Hospitalisation for heart failure**	Metformin *vs* Standard treatments	NA	NA	NA	NA
**Hospitalisation for heart failure**	Metformin *vs* Sulfonylureas	NA	NA	NA	NA
**Hospitalisation for heart failure**	Metformin *vs* Thiazolidinediones	0.14 (0 to 5.86)	NA	-1.03	0.303
**Hospitalisation for heart failure**	Metformin *vs* Tirzepatide	NA	NA	NA	NA
**Hospitalisation for heart failure**	Non-steroidal MRAs *vs* SGLT2 inhibitors	NA	NA	NA	NA
**Hospitalisation for heart failure**	Non-steroidal MRAs *vs* Standard treatments	NA	NA	NA	NA
**Hospitalisation for heart failure**	Non-steroidal MRAs *vs* Sulfonylureas	NA	NA	NA	NA
**Hospitalisation for heart failure**	Non-steroidal MRAs *vs* Thiazolidinediones	NA	NA	NA	NA
**Hospitalisation for heart failure**	Non-steroidal MRAs *vs* Tirzepatide	NA	NA	NA	NA
**Hospitalisation for heart failure**	SGLT2 inhibitors *vs* Standard treatments	0.58 (0.2 to 1.67)	NA	-1.007	0.314
**Hospitalisation for heart failure**	SGLT2 inhibitors *vs* Sulfonylureas	1.27 (0.33 to 4.88)	NA	0.354	0.723
**Hospitalisation for heart failure**	SGLT2 inhibitors *vs* Thiazolidinediones	NA	NA	NA	NA
**Hospitalisation for heart failure**	SGLT2 inhibitors *vs* Tirzepatide	NA	NA	NA	NA
**Hospitalisation for heart failure**	Sulfonylureas *vs* Standard treatments	0.76 (0.06 to 9.25)	NA	-0.212	0.832
**Hospitalisation for heart failure**	Thiazolidinediones *vs* Standard treatments	1.1 (0.58 to 2.1)	NA	0.285	0.776
**Hospitalisation for heart failure**	Tirzepatide *vs* Standard treatments	NA	NA	NA	NA
**Hospitalisation for heart failure**	Sulfonylureas *vs* Thiazolidinediones	1.25 (0.63 to 2.46)	NA	0.643	0.52
**Hospitalisation for heart failure**	Sulfonylureas *vs* Tirzepatide	NA	NA	NA	NA
**Hospitalisation for heart failure**	Thiazolidinediones *vs* Tirzepatide	NA	NA	NA	NA
**End-stage kidney disease**	Basal insulin *vs* Bolus insulin	NA	NA	NA	NA
**End-stage kidney disease**	Basal insulin *vs* DPP-4 inhibitors	NA	NA	NA	NA
**End-stage kidney disease**	Basal insulin *vs* GLP-1 receptor agonists	0.71 (0.01 to 41.26)	NA	-0.162	0.871
**End-stage kidney disease**	Basal insulin *vs* Metformin	NA	NA	NA	NA
**End-stage kidney disease**	Basal insulin *vs* Non-steroidal MRAs	NA	NA	NA	NA

Outcome	Comparisons	Ratio of odds ratio	Difference in mean difference	Z statistic	P-value
**End-stage kidney disease**	Basal insulin *vs* SGLT2 inhibitors	NA	NA	NA	NA
**End-stage kidney disease**	Basal insulin *vs* Standard treatments	NA	NA	NA	NA
**End-stage kidney disease**	Basal insulin *vs* Sulfonylureas	NA	NA	NA	NA
**End-stage kidney disease**	Basal insulin *vs* Thiazolidinediones	NA	NA	NA	NA
**End-stage kidney disease**	Basal insulin *vs* Tirzepatide	1.4 (0.02 to 80.84)	NA	0.162	0.871
**End-stage kidney disease**	Bolus insulin *vs* DPP-4 inhibitors	NA	NA	NA	NA
**End-stage kidney disease**	Bolus insulin *vs* GLP-1 receptor agonists	NA	NA	NA	NA
**End-stage kidney disease**	Bolus insulin *vs* Metformin	NA	NA	NA	NA
**End-stage kidney disease**	Bolus insulin *vs* Non-steroidal MRAs	NA	NA	NA	NA
**End-stage kidney disease**	Bolus insulin *vs* SGLT2 inhibitors	NA	NA	NA	NA
**End-stage kidney disease**	Bolus insulin *vs* Standard treatments	NA	NA	NA	NA
**End-stage kidney disease**	Bolus insulin *vs* Sulfonylureas	NA	NA	NA	NA
**End-stage kidney disease**	Bolus insulin *vs* Thiazolidinediones	NA	NA	NA	NA
**End-stage kidney disease**	Bolus insulin *vs* Tirzepatide	NA	NA	NA	NA
**End-stage kidney disease**	DPP-4 inhibitors *vs* GLP-1 receptor agonists	1.09 (0.33 to 3.63)	NA	0.145	0.885
**End-stage kidney disease**	DPP-4 inhibitors *vs* Metformin	NA	NA	NA	NA
**End-stage kidney disease**	DPP-4 inhibitors *vs* Non-steroidal MRAs	NA	NA	NA	NA
**End-stage kidney disease**	DPP-4 inhibitors *vs* SGLT2 inhibitors	NA	NA	NA	NA
**End-stage kidney disease**	DPP-4 inhibitors *vs* Standard treatments	1.02 (0.4 to 2.62)	NA	0.04	0.968
**End-stage kidney disease**	DPP-4 inhibitors *vs* Sulfonylureas	0.83 (0.18 to 3.77)	NA	-0.247	0.805
**End-stage kidney disease**	DPP-4 inhibitors *vs* Thiazolidinediones	NA	NA	NA	NA
**End-stage kidney disease**	DPP-4 inhibitors *vs* Tirzepatide	NA	NA	NA	NA
**End-stage kidney disease**	GLP-1 receptor agonists *vs* Metformin	NA	NA	NA	NA
**End-stage kidney disease**	GLP-1 receptor agonists *vs* Non-steroidal MRAs	NA	NA	NA	NA
**End-stage kidney disease**	GLP-1 receptor agonists *vs* SGLT2 inhibitors	NA	NA	NA	NA

Outcome	Comparisons	Ratio of odds ratio	Difference in mean difference	Z statistic	P-value
**End-stage kidney disease**	GLP-1 receptor agonists *vs* Standard treatments	1.09 (0.33 to 3.63)	NA	0.145	0.885
**End-stage kidney disease**	GLP-1 receptor agonists *vs* Sulfonylureas	NA	NA	NA	NA
**End-stage kidney disease**	GLP-1 receptor agonists *vs* Thiazolidinediones	NA	NA	NA	NA
**End-stage kidney disease**	GLP-1 receptor agonists *vs* Tirzepatide	0.71 (0.01 to 41.26)	NA	-0.162	0.871
**End-stage kidney disease**	Metformin *vs* Non-steroidal MRAs	NA	NA	NA	NA
**End-stage kidney disease**	Metformin *vs* SGLT2 inhibitors	NA	NA	NA	NA
**End-stage kidney disease**	Metformin *vs* Standard treatments	NA	NA	NA	NA
**End-stage kidney disease**	Metformin *vs* Sulfonylureas	NA	NA	NA	NA
**End-stage kidney disease**	Metformin *vs* Thiazolidinediones	NA	NA	NA	NA
**End-stage kidney disease**	Metformin *vs* Tirzepatide	NA	NA	NA	NA
**End-stage kidney disease**	Non-steroidal MRAs *vs* SGLT2 inhibitors	NA	NA	NA	NA
**End-stage kidney disease**	Non-steroidal MRAs *vs* Standard treatments	NA	NA	NA	NA
**End-stage kidney disease**	Non-steroidal MRAs *vs* Sulfonylureas	NA	NA	NA	NA
**End-stage kidney disease**	Non-steroidal MRAs *vs* Thiazolidinediones	NA	NA	NA	NA
**End-stage kidney disease**	Non-steroidal MRAs *vs* Tirzepatide	NA	NA	NA	NA
**End-stage kidney disease**	SGLT2 inhibitors *vs* Standard treatments	0.28 (0.01 to 7.24)	NA	-0.77	0.441
**End-stage kidney disease**	SGLT2 inhibitors *vs* Sulfonylureas	3.6 (0.14 to 94)	NA	0.77	0.441
**End-stage kidney disease**	SGLT2 inhibitors *vs* Thiazolidinediones	NA	NA	NA	NA
**End-stage kidney disease**	SGLT2 inhibitors *vs* Tirzepatide	NA	NA	NA	NA
**End-stage kidney disease**	Sulfonylureas *vs* Standard treatments	NA	NA	NA	NA
**End-stage kidney disease**	Thiazolidinediones *vs* Standard treatments	1.11 (0.21 to 5.73)	NA	0.121	0.904
**End-stage kidney disease**	Tirzepatide *vs* Standard treatments	NA	NA	NA	NA
**End-stage kidney disease**	Sulfonylureas *vs* Thiazolidinediones	1.11 (0.21 to 5.73)	NA	0.121	0.904
**End-stage kidney disease**	Sulfonylureas *vs* Tirzepatide	NA	NA	NA	NA
**End-stage kidney disease**	Thiazolidinediones *vs* Tirzepatide	NA	NA	NA	NA

Outcome	Comparisons	Ratio of odds ratio	Difference in mean difference	Z statistic	P-value
**Severe hypoglycaemia**	Alpha-glucosidase inhibitors *vs* Basal insulin	NA	NA	NA	NA
**Severe hypoglycaemia**	Alpha-glucosidase inhibitors *vs* Basal-Bolus insulin	NA	NA	NA	NA
**Severe hypoglycaemia**	Alpha-glucosidase inhibitors *vs* Bolus insulin	NA	NA	NA	NA
**Severe hypoglycaemia**	Alpha-glucosidase inhibitors *vs* DPP-4 inhibitors	NA	NA	NA	NA
**Severe hypoglycaemia**	Alpha-glucosidase inhibitors *vs* GLP-1 receptor agonists	NA	NA	NA	NA
**Severe hypoglycaemia**	Alpha-glucosidase inhibitors *vs* Meglitinides	3.22 (0.04 to 271.34)	NA	0.518	0.605
**Severe hypoglycaemia**	Alpha-glucosidase inhibitors *vs* Metformin	NA	NA	NA	NA
**Severe hypoglycaemia**	Alpha-glucosidase inhibitors *vs* Non-steroidal MRAs	NA	NA	NA	NA
**Severe hypoglycaemia**	Alpha-glucosidase inhibitors *vs* SGLT2 inhibitors	2.56 (0.07 to 93.09)	NA	0.514	0.608
**Severe hypoglycaemia**	Alpha-glucosidase inhibitors *vs* Standard treatments	0.23 (0.01 to 4.2)	NA	-0.994	0.32
**Severe hypoglycaemia**	Alpha-glucosidase inhibitors *vs* Sulfonylureas	3.48 (0.1 to 126.39)	NA	0.681	0.496
**Severe hypoglycaemia**	Alpha-glucosidase inhibitors *vs* Thiazolidinediones	NA	NA	NA	NA
**Severe hypoglycaemia**	Alpha-glucosidase inhibitors *vs* Tirzepatide	NA	NA	NA	NA
**Severe hypoglycaemia**	Basal insulin *vs* Basal-Bolus insulin	2.97 (0.1 to 87.15)	NA	0.632	0.528
**Severe hypoglycaemia**	Basal insulin *vs* Bolus insulin	NA	NA	NA	NA
**Severe hypoglycaemia**	Basal insulin *vs* DPP-4 inhibitors	0.92 (0.4 to 2.1)	NA	-0.205	0.837
**Severe hypoglycaemia**	Basal insulin *vs* GLP-1 receptor agonists	0.58 (0.28 to 1.2)	NA	-1.468	0.142
**Severe hypoglycaemia**	Basal insulin *vs* Meglitinides	NA	NA	NA	NA
**Severe hypoglycaemia**	Basal insulin *vs* Metformin	NA	NA	NA	NA
**Severe hypoglycaemia**	Basal insulin *vs* Non-steroidal MRAs	NA	NA	NA	NA
**Severe hypoglycaemia**	Basal insulin *vs* SGLT2 inhibitors	1.25 (0.05 to 31.28)	NA	0.134	0.893
**Severe hypoglycaemia**	Basal insulin *vs* Standard treatments	0.21 (0 to 10.5)	NA	-0.789	0.43
**Severe hypoglycaemia**	Basal insulin *vs* Sulfonylureas	1.37 (0.64 to 2.96)	NA	0.81	0.418
**Severe hypoglycaemia**	Basal insulin *vs* Thiazolidinediones	0.68 (0.19 to 2.42)	NA	-0.598	0.55
**Severe hypoglycaemia**	Basal insulin *vs* Tirzepatide	2.06 (0.19 to 22.63)	NA	0.59	0.555

Outcome	Comparisons	Ratio of odds ratio	Difference in mean difference	Z statistic	P-value
**Severe hypoglycaemia**	Basal-Bolus insulin *vs* Bolus insulin	NA	NA	NA	NA
**Severe hypoglycaemia**	Basal-Bolus insulin *vs* DPP-4 inhibitors	NA	NA	NA	NA
**Severe hypoglycaemia**	Basal-Bolus insulin *vs* GLP-1 receptor agonists	2.97 (0.1 to 87.15)	NA	0.632	0.528
**Severe hypoglycaemia**	Basal-Bolus insulin *vs* Meglitinides	NA	NA	NA	NA
**Severe hypoglycaemia**	Basal-Bolus insulin *vs* Metformin	NA	NA	NA	NA
**Severe hypoglycaemia**	Basal-Bolus insulin *vs* Non-steroidal MRAs	NA	NA	NA	NA
**Severe hypoglycaemia**	Basal-Bolus insulin *vs* SGLT2 inhibitors	NA	NA	NA	NA
**Severe hypoglycaemia**	Basal-Bolus insulin *vs* Standard treatments	NA	NA	NA	NA
**Severe hypoglycaemia**	Basal-Bolus insulin *vs* Sulfonylureas	NA	NA	NA	NA
**Severe hypoglycaemia**	Basal-Bolus insulin *vs* Thiazolidinediones	NA	NA	NA	NA
**Severe hypoglycaemia**	Basal-Bolus insulin *vs* Tirzepatide	NA	NA	NA	NA
**Severe hypoglycaemia**	Bolus insulin *vs* DPP-4 inhibitors	NA	NA	NA	NA
**Severe hypoglycaemia**	Bolus insulin *vs* GLP-1 receptor agonists	NA	NA	NA	NA
**Severe hypoglycaemia**	Bolus insulin *vs* Meglitinides	NA	NA	NA	NA
**Severe hypoglycaemia**	Bolus insulin *vs* Metformin	NA	NA	NA	NA
**Severe hypoglycaemia**	Bolus insulin *vs* Non-steroidal MRAs	NA	NA	NA	NA
**Severe hypoglycaemia**	Bolus insulin *vs* SGLT2 inhibitors	NA	NA	NA	NA
**Severe hypoglycaemia**	Bolus insulin *vs* Standard treatments	NA	NA	NA	NA
**Severe hypoglycaemia**	Bolus insulin *vs* Sulfonylureas	NA	NA	NA	NA
**Severe hypoglycaemia**	Bolus insulin *vs* Thiazolidinediones	NA	NA	NA	NA
**Severe hypoglycaemia**	Bolus insulin *vs* Tirzepatide	NA	NA	NA	NA
**Severe hypoglycaemia**	DPP-4 inhibitors *vs* GLP-1 receptor agonists	1.22 (0.84 to 1.78)	NA	1.051	0.293
**Severe hypoglycaemia**	DPP-4 inhibitors *vs* Meglitinides	NA	NA	NA	NA
**Severe hypoglycaemia**	DPP-4 inhibitors *vs* Metformin	1.67 (0.17 to 16.08)	NA	0.446	0.656
**Severe hypoglycaemia**	DPP-4 inhibitors *vs* Non-steroidal MRAs	NA	NA	NA	NA

Outcome	Comparisons	Ratio of odds ratio	Difference in mean difference	Z statistic	P-value
**Severe hypoglycaemia**	DPP-4 inhibitors *vs* SGLT2 inhibitors	0.51 (0.2 to 1.27)	NA	-1.45	0.147
**Severe hypoglycaemia**	DPP-4 inhibitors *vs* Standard treatments	0.95 (0.7 to 1.3)	NA	-0.295	0.768
**Severe hypoglycaemia**	DPP-4 inhibitors *vs* Sulfonylureas	0.74 (0.4 to 1.35)	NA	-0.996	0.319
**Severe hypoglycaemia**	DPP-4 inhibitors *vs* Thiazolidinediones	3.74 (0.79 to 17.65)	NA	1.666	0.096
**Severe hypoglycaemia**	DPP-4 inhibitors *vs* Tirzepatide	NA	NA	NA	NA
**Severe hypoglycaemia**	GLP-1 receptor agonists *vs* Meglitinides	NA	NA	NA	NA
**Severe hypoglycaemia**	GLP-1 receptor agonists *vs* Metformin	NA	NA	NA	NA
**Severe hypoglycaemia**	GLP-1 receptor agonists *vs* Non-steroidal MRAs	NA	NA	NA	NA
**Severe hypoglycaemia**	GLP-1 receptor agonists *vs* SGLT2 inhibitors	1.08 (0.49 to 2.4)	NA	0.199	0.842
**Severe hypoglycaemia**	GLP-1 receptor agonists *vs* Standard treatments	1.01 (0.74 to 1.37)	NA	0.033	0.974
**Severe hypoglycaemia**	GLP-1 receptor agonists *vs* Sulfonylureas	3.24 (1.56 to 6.76)	NA	3.14	0.002
**Severe hypoglycaemia**	GLP-1 receptor agonists *vs* Thiazolidinediones	0.78 (0.14 to 4.26)	NA	-0.286	0.775
**Severe hypoglycaemia**	GLP-1 receptor agonists *vs* Tirzepatide	0.66 (0.03 to 16.43)	NA	-0.252	0.801
**Severe hypoglycaemia**	Meglitinides *vs* Metformin	2.72 (0.2 to 37.46)	NA	0.748	0.454
**Severe hypoglycaemia**	Meglitinides *vs* Non-steroidal MRAs	NA	NA	NA	NA
**Severe hypoglycaemia**	Meglitinides *vs* SGLT2 inhibitors	NA	NA	NA	NA
**Severe hypoglycaemia**	Meglitinides *vs* Standard treatments	NA	NA	NA	NA
**Severe hypoglycaemia**	Meglitinides *vs* Sulfonylureas	0.43 (0.03 to 6.07)	NA	-0.623	0.533
**Severe hypoglycaemia**	Meglitinides *vs* Thiazolidinediones	NA	NA	NA	NA
**Severe hypoglycaemia**	Meglitinides *vs* Tirzepatide	NA	NA	NA	NA
**Severe hypoglycaemia**	Metformin *vs* Non-steroidal MRAs	NA	NA	NA	NA
**Severe hypoglycaemia**	Metformin *vs* SGLT2 inhibitors	2.26 (0.44 to 11.49)	NA	0.981	0.326
**Severe hypoglycaemia**	Metformin *vs* Standard treatments	0.9 (0.24 to 3.41)	NA	-0.149	0.881
**Severe hypoglycaemia**	Metformin *vs* Sulfonylureas	0.92 (0.14 to 6.21)	NA	-0.084	0.933
**Severe hypoglycaemia**	Metformin *vs* Thiazolidinediones	0.81 (0.05 to 14.46)	NA	-0.144	0.885

Outcome	Comparisons	Ratio of odds ratio	Difference in mean difference	Z statistic	P-value
**Severe hypoglycaemia**	Metformin *vs* Tirzepatide	NA	NA	NA	NA
**Severe hypoglycaemia**	Non-steroidal MRAs *vs* SGLT2 inhibitors	NA	NA	NA	NA
**Severe hypoglycaemia**	Non-steroidal MRAs *vs* Standard treatments	NA	NA	NA	NA
**Severe hypoglycaemia**	Non-steroidal MRAs *vs* Sulfonylureas	NA	NA	NA	NA
**Severe hypoglycaemia**	Non-steroidal MRAs *vs* Thiazolidinediones	NA	NA	NA	NA
**Severe hypoglycaemia**	Non-steroidal MRAs *vs* Tirzepatide	NA	NA	NA	NA
**Severe hypoglycaemia**	SGLT2 inhibitors *vs* Standard treatments	0.94 (0.57 to 1.54)	NA	-0.259	0.795
**Severe hypoglycaemia**	SGLT2 inhibitors *vs* Sulfonylureas	0.99 (0.37 to 2.64)	NA	-0.023	0.982
**Severe hypoglycaemia**	SGLT2 inhibitors *vs* Thiazolidinediones	NA	NA	NA	NA
**Severe hypoglycaemia**	SGLT2 inhibitors *vs* Tirzepatide	NA	NA	NA	NA
**Severe hypoglycaemia**	Sulfonylureas *vs* Standard treatments	0.27 (0.02 to 3.27)	NA	-1.033	0.302
**Severe hypoglycaemia**	Thiazolidinediones *vs* Standard treatments	1.93 (0.85 to 4.41)	NA	1.565	0.118
**Severe hypoglycaemia**	Tirzepatide *vs* Standard treatments	2.34 (0.1 to 54.48)	NA	0.528	0.597
**Severe hypoglycaemia**	Sulfonylureas *vs* Thiazolidinediones	2.94 (0.91 to 9.52)	NA	1.804	0.071
**Severe hypoglycaemia**	Sulfonylureas *vs* Tirzepatide	NA	NA	NA	NA
**Severe hypoglycaemia**	Thiazolidinediones *vs* Tirzepatide	NA	NA	NA	NA
**Severe gastrointestinal events**	Alpha-glucosidase inhibitors *vs* Basal insulin	NA	NA	NA	NA
**Severe gastrointestinal events**	Alpha-glucosidase inhibitors *vs* Bolus insulin	NA	NA	NA	NA
**Severe gastrointestinal events**	Alpha-glucosidase inhibitors *vs* DPP-4 inhibitors	NA	NA	NA	NA
**Severe gastrointestinal events**	Alpha-glucosidase inhibitors *vs* GLP-1 receptor agonists	NA	NA	NA	NA
**Severe gastrointestinal events**	Alpha-glucosidase inhibitors *vs* Metformin	NA	NA	NA	NA
**Severe gastrointestinal events**	Alpha-glucosidase inhibitors *vs* Standard treatments	NA	NA	NA	NA
**Severe gastrointestinal events**	Alpha-glucosidase inhibitors *vs* Sulfonylureas	NA	NA	NA	NA
**Severe gastrointestinal events**	Alpha-glucosidase inhibitors *vs* Thiazolidinediones	NA	NA	NA	NA
**Severe gastrointestinal events**	Alpha-glucosidase inhibitors *vs* Tirzepatide	NA	NA	NA	NA

Outcome	Comparisons	Ratio of odds ratio	Difference in mean difference	Z statistic	P-value
**Severe gastrointestinal events**	Basal insulin *vs* Bolus insulin	NA	NA	NA	NA
**Severe gastrointestinal events**	Basal insulin *vs* DPP-4 inhibitors	4.58 (0.16 to 127.69)	NA	0.895	0.371
**Severe gastrointestinal events**	Basal insulin *vs* GLP-1 receptor agonists	2.27 (0.2 to 26.18)	NA	0.656	0.512
**Severe gastrointestinal events**	Basal insulin *vs* Metformin	NA	NA	NA	NA
**Severe gastrointestinal events**	Basal insulin *vs* Standard treatments	NA	NA	NA	NA
**Severe gastrointestinal events**	Basal insulin *vs* Sulfonylureas	NA	NA	NA	NA
**Severe gastrointestinal events**	Basal insulin *vs* Thiazolidinediones	NA	NA	NA	NA
**Severe gastrointestinal events**	Basal insulin *vs* Tirzepatide	0.18 (0.01 to 2.18)	NA	-1.354	0.176
**Severe gastrointestinal events**	Bolus insulin *vs* DPP-4 inhibitors	NA	NA	NA	NA
**Severe gastrointestinal events**	Bolus insulin *vs* GLP-1 receptor agonists	NA	NA	NA	NA
**Severe gastrointestinal events**	Bolus insulin *vs* Metformin	NA	NA	NA	NA
**Severe gastrointestinal events**	Bolus insulin *vs* Standard treatments	NA	NA	NA	NA
**Severe gastrointestinal events**	Bolus insulin *vs* Sulfonylureas	NA	NA	NA	NA
**Severe gastrointestinal events**	Bolus insulin *vs* Thiazolidinediones	NA	NA	NA	NA
**Severe gastrointestinal events**	Bolus insulin *vs* Tirzepatide	NA	NA	NA	NA
**Severe gastrointestinal events**	DPP-4 inhibitors *vs* GLP-1 receptor agonists	1.14 (0.23 to 5.53)	NA	0.16	0.873
**Severe gastrointestinal events**	DPP-4 inhibitors *vs* Metformin	NA	NA	NA	NA
**Severe gastrointestinal events**	DPP-4 inhibitors *vs* Standard treatments	1.92 (0.36 to 10.28)	NA	0.766	0.444
**Severe gastrointestinal events**	DPP-4 inhibitors *vs* Sulfonylureas	0.36 (0.03 to 5.02)	NA	-0.764	0.445
**Severe gastrointestinal events**	DPP-4 inhibitors *vs* Thiazolidinediones	NA	NA	NA	NA
**Severe gastrointestinal events**	DPP-4 inhibitors *vs* Tirzepatide	NA	NA	NA	NA
**Severe gastrointestinal events**	GLP-1 receptor agonists *vs* Metformin	NA	NA	NA	NA
**Severe gastrointestinal events**	GLP-1 receptor agonists *vs* Standard treatments	0.68 (0.18 to 2.49)	NA	-0.587	0.557
**Severe gastrointestinal events**	GLP-1 receptor agonists *vs* Sulfonylureas	NA	NA	NA	NA
**Severe gastrointestinal events**	GLP-1 receptor agonists *vs* Thiazolidinediones	NA	NA	NA	NA

Outcome	Comparisons	Ratio of odds ratio	Difference in mean difference	Z statistic	P-value
**Severe gastrointestinal events**	GLP-1 receptor agonists *vs* Tirzepatide	3.2 (0.55 to 18.66)	NA	1.293	0.196
**Severe gastrointestinal events**	Metformin *vs* Standard treatments	0.87 (0.04 to 16.74)	NA	-0.095	0.924
**Severe gastrointestinal events**	Metformin *vs* Sulfonylureas	1.14 (0.04 to 29.44)	NA	0.078	0.937
**Severe gastrointestinal events**	Metformin *vs* Thiazolidinediones	0.65 (0.04 to 10.9)	NA	-0.303	0.762
**Severe gastrointestinal events**	Metformin *vs* Tirzepatide	NA	NA	NA	NA
**Severe gastrointestinal events**	Sulfonylureas *vs* Standard treatments	NA	NA	NA	NA
**Severe gastrointestinal events**	Thiazolidinediones *vs* Standard treatments	0.54 (0.06 to 4.74)	NA	-0.559	0.576
**Severe gastrointestinal events**	Tirzepatide *vs* Standard treatments	1.38 (0.2 to 9.37)	NA	0.332	0.74
**Severe gastrointestinal events**	Sulfonylureas *vs* Thiazolidinediones	0.21 (0.01 to 2.92)	NA	-1.167	0.243
**Severe gastrointestinal events**	Sulfonylureas *vs* Tirzepatide	NA	NA	NA	NA
**Severe gastrointestinal events**	Thiazolidinediones *vs* Tirzepatide	NA	NA	NA	NA
**Genital infection**	DPP-4 inhibitors *vs* GLP-1 receptor agonists	NA	NA	NA	NA
**Genital infection**	DPP-4 inhibitors *vs* Metformin	NA	NA	NA	NA
**Genital infection**	DPP-4 inhibitors *vs* SGLT2 inhibitors	2.17 (0.79 to 5.94)	NA	1.51	0.131
**Genital infection**	DPP-4 inhibitors *vs* Standard treatments	0.57 (0.21 to 1.53)	NA	-1.115	0.265
**Genital infection**	DPP-4 inhibitors *vs* Sulfonylureas	NA	NA	NA	NA
**Genital infection**	DPP-4 inhibitors *vs* Thiazolidinediones	NA	NA	NA	NA
**Genital infection**	GLP-1 receptor agonists *vs* Metformin	NA	NA	NA	NA
**Genital infection**	GLP-1 receptor agonists *vs* SGLT2 inhibitors	0.49 (0.1 to 2.49)	NA	-0.86	0.39
**Genital infection**	GLP-1 receptor agonists *vs* Standard treatments	2.04 (0.4 to 10.38)	NA	0.86	0.39
**Genital infection**	GLP-1 receptor agonists *vs* Sulfonylureas	NA	NA	NA	NA
**Genital infection**	GLP-1 receptor agonists *vs* Thiazolidinediones	NA	NA	NA	NA
**Genital infection**	Metformin *vs* SGLT2 inhibitors	NA	NA	NA	NA
**Genital infection**	Metformin *vs* Standard treatments	NA	NA	NA	NA
**Genital infection**	Metformin *vs* Sulfonylureas	NA	NA	NA	NA

Outcome	Comparisons	Ratio of odds ratio	Difference in mean difference	Z statistic	P-value
**Genital infection**	Metformin *vs* Thiazolidinediones	NA	NA	NA	NA
**Genital infection**	SGLT2 inhibitors *vs* Standard treatments	1.47 (0.62 to 3.49)	NA	0.877	0.381
**Genital infection**	SGLT2 inhibitors *vs* Sulfonylureas	NA	NA	NA	NA
**Genital infection**	SGLT2 inhibitors *vs* Thiazolidinediones	NA	NA	NA	NA
**Genital infection**	Sulfonylureas *vs* Standard treatments	NA	NA	NA	NA
**Genital infection**	Thiazolidinediones *vs* Standard treatments	NA	NA	NA	NA
**Genital infection**	Sulfonylureas *vs* Thiazolidinediones	NA	NA	NA	NA
**Amputation**	DPP-4 inhibitors *vs* GLP-1 receptor agonists	NA	NA	NA	NA
**Amputation**	DPP-4 inhibitors *vs* Non-steroidal MRAs	NA	NA	NA	NA
**Amputation**	DPP-4 inhibitors *vs* SGLT2 inhibitors	0.93 (0.03 to 25.12)	NA	-0.044	0.965
**Amputation**	DPP-4 inhibitors *vs* Standard treatments	1.08 (0.04 to 29.17)	NA	0.044	0.965
**Amputation**	DPP-4 inhibitors *vs* Thiazolidinediones	NA	NA	NA	NA
**Amputation**	GLP-1 receptor agonists *vs* Non-steroidal MRAs	NA	NA	NA	NA
**Amputation**	GLP-1 receptor agonists *vs* SGLT2 inhibitors	NA	NA	NA	NA
**Amputation**	GLP-1 receptor agonists *vs* Standard treatments	NA	NA	NA	NA
**Amputation**	GLP-1 receptor agonists *vs* Thiazolidinediones	NA	NA	NA	NA
**Amputation**	Non-steroidal MRAs *vs* SGLT2 inhibitors	NA	NA	NA	NA
**Amputation**	Non-steroidal MRAs *vs* Standard treatments	NA	NA	NA	NA
**Amputation**	Non-steroidal MRAs *vs* Thiazolidinediones	NA	NA	NA	NA
**Amputation**	SGLT2 inhibitors *vs* Standard treatments	0.93 (0.03 to 25.12)	NA	-0.044	0.965
**Amputation**	SGLT2 inhibitors *vs* Thiazolidinediones	NA	NA	NA	NA
**Amputation**	Thiazolidinediones *vs* Standard treatments	NA	NA	NA	NA
**Ketoacidosis due to diabetes**	Alpha-glucosidase inhibitors *vs* DPP-4 inhibitors	NA	NA	NA	NA
**Ketoacidosis due to diabetes**	Alpha-glucosidase inhibitors *vs* GLP-1 receptor agonists	NA	NA	NA	NA
**Ketoacidosis due to diabetes**	Alpha-glucosidase inhibitors *vs* Meglitinides	NA	NA	NA	NA

Outcome	Comparisons	Ratio of odds ratio	Difference in mean difference	Z statistic	P-value
**Ketoacidosis due to diabetes**	Alpha-glucosidase inhibitors *vs* Metformin	NA	NA	NA	NA
**Ketoacidosis due to diabetes**	Alpha-glucosidase inhibitors *vs* Non-steroidal MRAs	NA	NA	NA	NA
**Ketoacidosis due to diabetes**	Alpha-glucosidase inhibitors *vs* SGLT2 inhibitors	NA	NA	NA	NA
**Ketoacidosis due to diabetes**	Alpha-glucosidase inhibitors *vs* Standard treatments	NA	NA	NA	NA
**Ketoacidosis due to diabetes**	Alpha-glucosidase inhibitors *vs* Sulfonylureas	NA	NA	NA	NA
**Ketoacidosis due to diabetes**	DPP-4 inhibitors *vs* GLP-1 receptor agonists	0.4 (0.01 to 11.15)	NA	-0.544	0.586
**Ketoacidosis due to diabetes**	DPP-4 inhibitors *vs* Meglitinides	NA	NA	NA	NA
**Ketoacidosis due to diabetes**	DPP-4 inhibitors *vs* Metformin	NA	NA	NA	NA
**Ketoacidosis due to diabetes**	DPP-4 inhibitors *vs* Non-steroidal MRAs	NA	NA	NA	NA
**Ketoacidosis due to diabetes**	DPP-4 inhibitors *vs* SGLT2 inhibitors	4.21 (0.38 to 46.71)	NA	1.171	0.242
**Ketoacidosis due to diabetes**	DPP-4 inhibitors *vs* Standard treatments	0.42 (0.07 to 2.71)	NA	-0.907	0.364
**Ketoacidosis due to diabetes**	DPP-4 inhibitors *vs* Sulfonylureas	3.82 (0.07 to 207.92)	NA	0.658	0.511
**Ketoacidosis due to diabetes**	GLP-1 receptor agonists *vs* Meglitinides	NA	NA	NA	NA
**Ketoacidosis due to diabetes**	GLP-1 receptor agonists *vs* Metformin	NA	NA	NA	NA
**Ketoacidosis due to diabetes**	GLP-1 receptor agonists *vs* Non-steroidal MRAs	NA	NA	NA	NA
**Ketoacidosis due to diabetes**	GLP-1 receptor agonists *vs* SGLT2 inhibitors	NA	NA	NA	NA
**Ketoacidosis due to diabetes**	GLP-1 receptor agonists *vs* Standard treatments	0.4 (0.01 to 11.15)	NA	-0.544	0.586
**Ketoacidosis due to diabetes**	GLP-1 receptor agonists *vs* Sulfonylureas	NA	NA	NA	NA
**Ketoacidosis due to diabetes**	Meglitinides *vs* Metformin	NA	NA	NA	NA
**Ketoacidosis due to diabetes**	Meglitinides *vs* Non-steroidal MRAs	NA	NA	NA	NA
**Ketoacidosis due to diabetes**	Meglitinides *vs* SGLT2 inhibitors	NA	NA	NA	NA
**Ketoacidosis due to diabetes**	Meglitinides *vs* Standard treatments	NA	NA	NA	NA
**Ketoacidosis due to diabetes**	Meglitinides *vs* Sulfonylureas	NA	NA	NA	NA
**Ketoacidosis due to diabetes**	Metformin *vs* Non-steroidal MRAs	NA	NA	NA	NA
**Ketoacidosis due to diabetes**	Metformin *vs* SGLT2 inhibitors	NA	NA	NA	NA

Outcome	Comparisons	Ratio of odds ratio	Difference in mean difference	Z statistic	P-value
**Ketoacidosis due to diabetes**	Metformin *vs* Standard treatments	NA	NA	NA	NA
**Ketoacidosis due to diabetes**	Metformin *vs* Sulfonylureas	NA	NA	NA	NA
**Ketoacidosis due to diabetes**	Non-steroidal MRAs *vs* SGLT2 inhibitors	NA	NA	NA	NA
**Ketoacidosis due to diabetes**	Non-steroidal MRAs *vs* Standard treatments	NA	NA	NA	NA
**Ketoacidosis due to diabetes**	Non-steroidal MRAs *vs* Sulfonylureas	NA	NA	NA	NA
**Ketoacidosis due to diabetes**	SGLT2 inhibitors *vs* Standard treatments	4.49 (0.54 to 37.64)	NA	1.385	0.166
**Ketoacidosis due to diabetes**	SGLT2 inhibitors *vs* Sulfonylureas	0.26 (0 to 14.23)	NA	-0.658	0.511
**Ketoacidosis due to diabetes**	Sulfonylureas *vs* Standard treatments	NA	NA	NA	NA
**Health-related quality of life**	Alpha-glucosidase inhibitors *vs* Basal insulin	NA	NA	NA	NA
**Health-related quality of life**	Alpha-glucosidase inhibitors *vs* Bolus insulin	NA	NA	NA	NA
**Health-related quality of life**	Alpha-glucosidase inhibitors *vs* DPP-4 inhibitors	NA	NA	NA	NA
**Health-related quality of life**	Alpha-glucosidase inhibitors *vs* GLP-1 receptor agonists	NA	NA	NA	NA
**Health-related quality of life**	Alpha-glucosidase inhibitors *vs* Meglitinides	NA	NA	NA	NA
**Health-related quality of life**	Alpha-glucosidase inhibitors *vs* Metformin	NA	NA	NA	NA
**Health-related quality of life**	Alpha-glucosidase inhibitors *vs* SGLT2 inhibitors	NA	NA	NA	NA
**Health-related quality of life**	Alpha-glucosidase inhibitors *vs* Standard treatments	NA	NA	NA	NA
**Health-related quality of life**	Alpha-glucosidase inhibitors *vs* Sulfonylureas	NA	NA	NA	NA
**Health-related quality of life**	Alpha-glucosidase inhibitors *vs* Thiazolidinediones	NA	NA	NA	NA
**Health-related quality of life**	Alpha-glucosidase inhibitors *vs* Tirzepatide	NA	NA	NA	NA
**Health-related quality of life**	Basal insulin *vs* Bolus insulin	NA	0.6 (0.12 to 1.07)	2.468	0.014
**Health-related quality of life**	Basal insulin *vs* DPP-4 inhibitors	NA	NA	NA	NA
**Health-related quality of life**	Basal insulin *vs* GLP-1 receptor agonists	NA	-0.04 (-0.52 to 0.44)	-0.162	0.871
**Health-related quality of life**	Basal insulin *vs* Meglitinides	NA	NA	NA	NA
**Health-related quality of life**	Basal insulin *vs* Metformin	NA	NA	NA	NA
**Health-related quality of life**	Basal insulin *vs* SGLT2 inhibitors	NA	NA	NA	NA

Outcome	Comparisons	Ratio of odds ratio	Difference in mean difference	Z statistic	P-value
**Health-related quality of life**	Basal insulin *vs* Standard treatments	NA	NA	NA	NA
**Health-related quality of life**	Basal insulin *vs* Sulfonylureas	NA	NA	NA	NA
**Health-related quality of life**	Basal insulin *vs* Thiazolidinediones	NA	NA	NA	NA
**Health-related quality of life**	Basal insulin *vs* Tirzepatide	NA	-0.63 (-1.13 to -0.12)	-2.445	0.014
**Health-related quality of life**	Bolus insulin *vs* DPP-4 inhibitors	NA	NA	NA	NA
**Health-related quality of life**	Bolus insulin *vs* GLP-1 receptor agonists	NA	0.6 (0.12 to 1.07)	2.468	0.014
**Health-related quality of life**	Bolus insulin *vs* Meglitinides	NA	NA	NA	NA
**Health-related quality of life**	Bolus insulin *vs* Metformin	NA	NA	NA	NA
**Health-related quality of life**	Bolus insulin *vs* SGLT2 inhibitors	NA	NA	NA	NA
**Health-related quality of life**	Bolus insulin *vs* Standard treatments	NA	NA	NA	NA
**Health-related quality of life**	Bolus insulin *vs* Sulfonylureas	NA	NA	NA	NA
**Health-related quality of life**	Bolus insulin *vs* Thiazolidinediones	NA	NA	NA	NA
**Health-related quality of life**	Bolus insulin *vs* Tirzepatide	NA	NA	NA	NA
**Health-related quality of life**	DPP-4 inhibitors *vs* GLP-1 receptor agonists	NA	-0.03 (-0.33 to 0.28)	-0.175	0.861
**Health-related quality of life**	DPP-4 inhibitors *vs* Meglitinides	NA	NA	NA	NA
**Health-related quality of life**	DPP-4 inhibitors *vs* Metformin	NA	0.08 (-0.59 to 0.76)	0.244	0.808
**Health-related quality of life**	DPP-4 inhibitors *vs* SGLT2 inhibitors	NA	0.01 (-0.44 to 0.47)	0.062	0.951
**Health-related quality of life**	DPP-4 inhibitors *vs* Standard treatments	NA	0.07 (-0.25 to 0.38)	0.428	0.668
**Health-related quality of life**	DPP-4 inhibitors *vs* Sulfonylureas	NA	NA	NA	NA
**Health-related quality of life**	DPP-4 inhibitors *vs* Thiazolidinediones	NA	0.1 (-0.62 to 0.83)	0.285	0.776
**Health-related quality of life**	DPP-4 inhibitors *vs* Tirzepatide	NA	NA	NA	NA
**Health-related quality of life**	GLP-1 receptor agonists *vs* Meglitinides	NA	NA	NA	NA
**Health-related quality of life**	GLP-1 receptor agonists *vs* Metformin	NA	-0.05 (-0.66 to 0.55)	-0.166	0.868
**Health-related quality of life**	GLP-1 receptor agonists *vs* SGLT2 inhibitors	NA	0 (-0.38 to 0.38)	0.008	0.994
**Health-related quality of life**	GLP-1 receptor agonists *vs* Standard treatments	NA	-0.11 (-0.4 to 0.18)	-0.753	0.452

Outcome	Comparisons	Ratio of odds ratio	Difference in mean difference	Z statistic	P-value
**Health-related quality of life**	GLP-1 receptor agonists *vs* Sulfonylureas	NA	NA	NA	NA
**Health-related quality of life**	GLP-1 receptor agonists *vs* Thiazolidinediones	NA	-0.1 (-0.89 to 0.69)	-0.244	0.808
**Health-related quality of life**	GLP-1 receptor agonists *vs* Tirzepatide	NA	0.63 (0.12 to 1.13)	2.445	0.014
**Health-related quality of life**	Meglitinides *vs* Metformin	NA	NA	NA	NA
**Health-related quality of life**	Meglitinides *vs* SGLT2 inhibitors	NA	NA	NA	NA
**Health-related quality of life**	Meglitinides *vs* Standard treatments	NA	NA	NA	NA
**Health-related quality of life**	Meglitinides *vs* Sulfonylureas	NA	NA	NA	NA
**Health-related quality of life**	Meglitinides *vs* Thiazolidinediones	NA	NA	NA	NA
**Health-related quality of life**	Meglitinides *vs* Tirzepatide	NA	NA	NA	NA
**Health-related quality of life**	Metformin *vs* SGLT2 inhibitors	NA	NA	NA	NA
**Health-related quality of life**	Metformin *vs* Standard treatments	NA	NA	NA	NA
**Health-related quality of life**	Metformin *vs* Sulfonylureas	NA	NA	NA	NA
**Health-related quality of life**	Metformin *vs* Thiazolidinediones	NA	-0.07 (-1.71 to 1.56)	-0.089	0.929
**Health-related quality of life**	Metformin *vs* Tirzepatide	NA	NA	NA	NA
**Health-related quality of life**	SGLT2 inhibitors *vs* Standard treatments	NA	0.01 (-0.38 to 0.41)	0.062	0.95
**Health-related quality of life**	SGLT2 inhibitors *vs* Sulfonylureas	NA	NA	NA	NA
**Health-related quality of life**	SGLT2 inhibitors *vs* Thiazolidinediones	NA	NA	NA	NA
**Health-related quality of life**	SGLT2 inhibitors *vs* Tirzepatide	NA	NA	NA	NA
**Health-related quality of life**	Sulfonylureas *vs* Standard treatments	NA	NA	NA	NA
**Health-related quality of life**	Thiazolidinediones *vs* Standard treatments	NA	NA	NA	NA
**Health-related quality of life**	Tirzepatide *vs* Standard treatments	NA	NA	NA	NA
**Health-related quality of life**	Sulfonylureas *vs* Thiazolidinediones	NA	NA	NA	NA
**Health-related quality of life**	Sulfonylureas *vs* Tirzepatide	NA	NA	NA	NA
**Health-related quality of life**	Thiazolidinediones *vs* Tirzepatide	NA	NA	NA	NA
**Body weight change**	Albiglutide *vs* Alpha-glucosidase inhibitors	NA	NA	NA	NA

Outcome	Comparisons	Ratio of odds ratio	Difference in mean difference	Z statistic	P-value
**Body weight change**	Albiglutide *vs* Basal insulin	NA	0.3 (-1.74 to 2.34)	0.289	0.772
**Body weight change**	Albiglutide *vs* Basal-Bolus insulin	NA	NA	NA	NA
**Body weight change**	Albiglutide *vs* Bolus insulin	NA	-2.15 (-3.9 to -0.41)	-2.421	0.015
**Body weight change**	Albiglutide *vs* DPP-4 inhibitors	NA	0.69 (-0.84 to 2.21)	0.881	0.378
**Body weight change**	Albiglutide *vs* Dulaglutide	NA	NA	NA	NA
**Body weight change**	Albiglutide *vs* Efpeglenatide	NA	NA	NA	NA
**Body weight change**	Albiglutide *vs* Exenatide extended-release	NA	NA	NA	NA
**Body weight change**	Albiglutide *vs* Exenatide immediate-release	NA	NA	NA	NA
**Body weight change**	Albiglutide *vs* Liraglutide	NA	NA	NA	NA
**Body weight change**	Albiglutide *vs* Lixisenatide	NA	NA	NA	NA
**Body weight change**	Albiglutide *vs* Loxenatide	NA	NA	NA	NA
**Body weight change**	Albiglutide *vs* Meglitinides	NA	NA	NA	NA
**Body weight change**	Albiglutide *vs* Metformin	NA	NA	NA	NA
**Body weight change**	Albiglutide *vs* Semaglutide (Oral)	NA	NA	NA	NA
**Body weight change**	Albiglutide *vs* Semaglutide (Subcutaneous)	NA	NA	NA	NA
**Body weight change**	Albiglutide *vs* SGLT2 inhibitors	NA	NA	NA	NA
**Body weight change**	Albiglutide *vs* Standard treatments	NA	1.15 (-0.13 to 2.43)	1.768	0.077
**Body weight change**	Albiglutide *vs* Sulfonylureas	NA	0.14 (-1.9 to 2.17)	0.132	0.895
**Body weight change**	Albiglutide *vs* Thiazolidinediones	NA	-0.56 (-2.59 to 1.48)	-0.536	0.592
**Body weight change**	Albiglutide *vs* Tirzepatide	NA	NA	NA	NA
**Body weight change**	Alpha-glucosidase inhibitors *vs* Basal insulin	NA	NA	NA	NA
**Body weight change**	Alpha-glucosidase inhibitors *vs* Basal-Bolus insulin	NA	NA	NA	NA
**Body weight change**	Alpha-glucosidase inhibitors *vs* Bolus insulin	NA	NA	NA	NA
**Body weight change**	Alpha-glucosidase inhibitors *vs* DPP-4 inhibitors	NA	-0.09 (-1.04 to 0.86)	-0.181	0.856
**Body weight change**	Alpha-glucosidase inhibitors *vs* Dulaglutide	NA	NA	NA	NA

Outcome	Comparisons	Ratio of odds ratio	Difference in mean difference	Z statistic	P-value
**Body weight change**	Alpha-glucosidase inhibitors *vs* Efpeglenatide	NA	NA	NA	NA
**Body weight change**	Alpha-glucosidase inhibitors *vs* Exenatide extended-release	NA	NA	NA	NA
**Body weight change**	Alpha-glucosidase inhibitors *vs* Exenatide immediate-release	NA	NA	NA	NA
**Body weight change**	Alpha-glucosidase inhibitors *vs* Liraglutide	NA	NA	NA	NA
**Body weight change**	Alpha-glucosidase inhibitors *vs* Lixisenatide	NA	NA	NA	NA
**Body weight change**	Alpha-glucosidase inhibitors *vs* Loxenatide	NA	NA	NA	NA
**Body weight change**	Alpha-glucosidase inhibitors *vs* Meglitinides	NA	NA	NA	NA
**Body weight change**	Alpha-glucosidase inhibitors *vs* Metformin	NA	0.18 (-1.1 to 1.46)	0.275	0.783
**Body weight change**	Alpha-glucosidase inhibitors *vs* Semaglutide (Oral)	NA	NA	NA	NA
**Body weight change**	Alpha-glucosidase inhibitors *vs* Semaglutide (Subcutaneous)	NA	NA	NA	NA
**Body weight change**	Alpha-glucosidase inhibitors *vs* SGLT2 inhibitors	NA	-1.34 (-4.22 to 1.54)	-0.912	0.362
**Body weight change**	Alpha-glucosidase inhibitors *vs* Standard treatments	NA	-0.29 (-1.14 to 0.56)	-0.672	0.502
**Body weight change**	Alpha-glucosidase inhibitors *vs* Sulfonylureas	NA	1.25 (-0.66 to 3.17)	1.284	0.199
**Body weight change**	Alpha-glucosidase inhibitors *vs* Thiazolidinediones	NA	0.64 (-0.93 to 2.21)	0.797	0.426
**Body weight change**	Alpha-glucosidase inhibitors *vs* Tirzepatide	NA	NA	NA	NA
**Body weight change**	Basal insulin *vs* Basal-Bolus insulin	NA	0.82 (-1.66 to 3.29)	0.647	0.518
**Body weight change**	Basal insulin *vs* Bolus insulin	NA	NA	NA	NA
**Body weight change**	Basal insulin *vs* DPP-4 inhibitors	NA	-0.17 (-1.29 to 0.95)	-0.297	0.766
**Body weight change**	Basal insulin *vs* Dulaglutide	NA	-0.86 (-2.05 to 0.32)	-1.425	0.154
**Body weight change**	Basal insulin *vs* Efpeglenatide	NA	NA	NA	NA
**Body weight change**	Basal insulin *vs* Exenatide extended-release	NA	0.02 (-1.33 to 1.37)	0.023	0.982
**Body weight change**	Basal insulin *vs* Exenatide immediate-release	NA	0.07 (-1.66 to 1.8)	0.08	0.936
**Body weight change**	Basal insulin *vs* Liraglutide	NA	-0.35 (-1.34 to 0.64)	-0.699	0.484
**Body weight change**	Basal insulin *vs* Lixisenatide	NA	NA	NA	NA
**Body weight change**	Basal insulin *vs* Loxenatide	NA	NA	NA	NA

Outcome	Comparisons	Ratio of odds ratio	Difference in mean difference	Z statistic	P-value
**Body weight change**	Basal insulin *vs* Meglitinides	NA	NA	NA	NA
**Body weight change**	Basal insulin *vs* Metformin	NA	NA	NA	NA
**Body weight change**	Basal insulin *vs* Semaglutide (Oral)	NA	NA	NA	NA
**Body weight change**	Basal insulin *vs* Semaglutide (Subcutaneous)	NA	0.81 (-1.28 to 2.9)	0.759	0.448
**Body weight change**	Basal insulin *vs* SGLT2 inhibitors	NA	NA	NA	NA
**Body weight change**	Basal insulin *vs* Standard treatments	NA	-0.53 (-1.6 to 0.55)	-0.963	0.335
**Body weight change**	Basal insulin *vs* Sulfonylureas	NA	0.23 (-1.39 to 1.85)	0.278	0.781
**Body weight change**	Basal insulin *vs* Thiazolidinediones	NA	-0.7 (-2.42 to 1.03)	-0.788	0.431
**Body weight change**	Basal insulin *vs* Tirzepatide	NA	2.02 (0.28 to 3.76)	2.272	0.023
**Body weight change**	Basal-Bolus insulin *vs* Bolus insulin	NA	NA	NA	NA
**Body weight change**	Basal-Bolus insulin *vs* DPP-4 inhibitors	NA	NA	NA	NA
**Body weight change**	Basal-Bolus insulin *vs* Dulaglutide	NA	NA	NA	NA
**Body weight change**	Basal-Bolus insulin *vs* Efpeglenatide	NA	NA	NA	NA
**Body weight change**	Basal-Bolus insulin *vs* Exenatide extended-release	NA	NA	NA	NA
**Body weight change**	Basal-Bolus insulin *vs* Exenatide immediate-release	NA	NA	NA	NA
**Body weight change**	Basal-Bolus insulin *vs* Liraglutide	NA	NA	NA	NA
**Body weight change**	Basal-Bolus insulin *vs* Lixisenatide	NA	NA	NA	NA
**Body weight change**	Basal-Bolus insulin *vs* Loxenatide	NA	NA	NA	NA
**Body weight change**	Basal-Bolus insulin *vs* Meglitinides	NA	NA	NA	NA
**Body weight change**	Basal-Bolus insulin *vs* Metformin	NA	NA	NA	NA
**Body weight change**	Basal-Bolus insulin *vs* Semaglutide (Oral)	NA	NA	NA	NA
**Body weight change**	Basal-Bolus insulin *vs* Semaglutide (Subcutaneous)	NA	NA	NA	NA
**Body weight change**	Basal-Bolus insulin *vs* SGLT2 inhibitors	NA	NA	NA	NA
**Body weight change**	Basal-Bolus insulin *vs* Standard treatments	NA	NA	NA	NA
**Body weight change**	Basal-Bolus insulin *vs* Sulfonylureas	NA	NA	NA	NA

Outcome	Comparisons	Ratio of odds ratio	Difference in mean difference	Z statistic	P-value
**Body weight change**	Basal-Bolus insulin *vs* Thiazolidinediones	NA	0.82 (-1.66 to 3.29)	0.647	0.518
**Body weight change**	Basal-Bolus insulin *vs* Tirzepatide	NA	NA	NA	NA
**Body weight change**	Bolus insulin *vs* DPP-4 inhibitors	NA	NA	NA	NA
**Body weight change**	Bolus insulin *vs* Dulaglutide	NA	NA	NA	NA
**Body weight change**	Bolus insulin *vs* Efpeglenatide	NA	NA	NA	NA
**Body weight change**	Bolus insulin *vs* Exenatide extended-release	NA	NA	NA	NA
**Body weight change**	Bolus insulin *vs* Exenatide immediate-release	NA	NA	NA	NA
**Body weight change**	Bolus insulin *vs* Liraglutide	NA	-1.67 (-3.46 to 0.12)	-1.83	0.067
**Body weight change**	Bolus insulin *vs* Lixisenatide	NA	-0.06 (-2.24 to 2.12)	-0.056	0.955
**Body weight change**	Bolus insulin *vs* Loxenatide	NA	NA	NA	NA
**Body weight change**	Bolus insulin *vs* Meglitinides	NA	NA	NA	NA
**Body weight change**	Bolus insulin *vs* Metformin	NA	NA	NA	NA
**Body weight change**	Bolus insulin *vs* Semaglutide (Oral)	NA	NA	NA	NA
**Body weight change**	Bolus insulin *vs* Semaglutide (Subcutaneous)	NA	1.94 (-0.17 to 4.05)	1.798	0.072
**Body weight change**	Bolus insulin *vs* SGLT2 inhibitors	NA	-2.54 (-4.58 to -0.49)	-2.434	0.015
**Body weight change**	Bolus insulin *vs* Standard treatments	NA	NA	NA	NA
**Body weight change**	Bolus insulin *vs* Sulfonylureas	NA	NA	NA	NA
**Body weight change**	Bolus insulin *vs* Thiazolidinediones	NA	NA	NA	NA
**Body weight change**	Bolus insulin *vs* Tirzepatide	NA	NA	NA	NA
**Body weight change**	DPP-4 inhibitors *vs* Dulaglutide	NA	0.04 (-1.9 to 1.97)	0.035	0.972
**Body weight change**	DPP-4 inhibitors *vs* Efpeglenatide	NA	NA	NA	NA
**Body weight change**	DPP-4 inhibitors *vs* Exenatide extended-release	NA	-0.67 (-2.03 to 0.7)	-0.957	0.339
**Body weight change**	DPP-4 inhibitors *vs* Exenatide immediate-release	NA	NA	NA	NA
**Body weight change**	DPP-4 inhibitors *vs* Liraglutide	NA	-0.53 (-1.49 to 0.43)	-1.085	0.278
**Body weight change**	DPP-4 inhibitors *vs* Lixisenatide	NA	0.2 (-1.86 to 2.26)	0.191	0.849

Outcome	Comparisons	Ratio of odds ratio	Difference in mean difference	Z statistic	P-value
**Body weight change**	DPP-4 inhibitors *vs* Loxenatide	NA	NA	NA	NA
**Body weight change**	DPP-4 inhibitors *vs* Meglitinides	NA	NA	NA	NA
**Body weight change**	DPP-4 inhibitors *vs* Metformin	NA	0.12 (-0.58 to 0.83)	0.347	0.729
**Body weight change**	DPP-4 inhibitors *vs* Semaglutide (Oral)	NA	-1.57 (-3.63 to 0.49)	-1.493	0.136
**Body weight change**	DPP-4 inhibitors *vs* Semaglutide (Subcutaneous)	NA	-1.05 (-2.59 to 0.49)	-1.338	0.181
**Body weight change**	DPP-4 inhibitors *vs* SGLT2 inhibitors	NA	-0.07 (-0.67 to 0.52)	-0.238	0.812
**Body weight change**	DPP-4 inhibitors *vs* Standard treatments	NA	-0.1 (-0.47 to 0.26)	-0.562	0.574
**Body weight change**	DPP-4 inhibitors *vs* Sulfonylureas	NA	0.06 (-0.51 to 0.63)	0.215	0.83
**Body weight change**	DPP-4 inhibitors *vs* Thiazolidinediones	NA	0.89 (0.23 to 1.55)	2.652	0.008
**Body weight change**	DPP-4 inhibitors *vs* Tirzepatide	NA	NA	NA	NA
**Body weight change**	Dulaglutide *vs* Efpeglenatide	NA	NA	NA	NA
**Body weight change**	Dulaglutide *vs* Exenatide extended-release	NA	2.23 (-0.42 to 4.89)	1.649	0.099
**Body weight change**	Dulaglutide *vs* Exenatide immediate-release	NA	NA	NA	NA
**Body weight change**	Dulaglutide *vs* Liraglutide	NA	NA	NA	NA
**Body weight change**	Dulaglutide *vs* Lixisenatide	NA	NA	NA	NA
**Body weight change**	Dulaglutide *vs* Loxenatide	NA	NA	NA	NA
**Body weight change**	Dulaglutide *vs* Meglitinides	NA	NA	NA	NA
**Body weight change**	Dulaglutide *vs* Metformin	NA	0.56 (-1.45 to 2.56)	0.546	0.585
**Body weight change**	Dulaglutide *vs* Semaglutide (Oral)	NA	NA	NA	NA
**Body weight change**	Dulaglutide *vs* Semaglutide (Subcutaneous)	NA	NA	NA	NA
**Body weight change**	Dulaglutide *vs* SGLT2 inhibitors	NA	NA	NA	NA
**Body weight change**	Dulaglutide *vs* Standard treatments	NA	-0.59 (-1.65 to 0.47)	-1.095	0.273
**Body weight change**	Dulaglutide *vs* Sulfonylureas	NA	0.93 (-1.03 to 2.88)	0.928	0.354
**Body weight change**	Dulaglutide *vs* Thiazolidinediones	NA	NA	NA	NA
**Body weight change**	Dulaglutide *vs* Tirzepatide	NA	-4 (-6.73 to -1.27)	-2.872	0.004

Outcome	Comparisons	Ratio of odds ratio	Difference in mean difference	Z statistic	P-value
**Body weight change**	Efpeglenatide *vs* Exenatide extended-release	NA	NA	NA	NA
**Body weight change**	Efpeglenatide *vs* Exenatide immediate-release	NA	NA	NA	NA
**Body weight change**	Efpeglenatide *vs* Liraglutide	NA	NA	NA	NA
**Body weight change**	Efpeglenatide *vs* Lixisenatide	NA	NA	NA	NA
**Body weight change**	Efpeglenatide *vs* Loxenatide	NA	NA	NA	NA
**Body weight change**	Efpeglenatide *vs* Meglitinides	NA	NA	NA	NA
**Body weight change**	Efpeglenatide *vs* Metformin	NA	NA	NA	NA
**Body weight change**	Efpeglenatide *vs* Semaglutide (Oral)	NA	NA	NA	NA
**Body weight change**	Efpeglenatide *vs* Semaglutide (Subcutaneous)	NA	NA	NA	NA
**Body weight change**	Efpeglenatide *vs* SGLT2 inhibitors	NA	NA	NA	NA
**Body weight change**	Efpeglenatide *vs* Standard treatments	NA	NA	NA	NA
**Body weight change**	Efpeglenatide *vs* Sulfonylureas	NA	NA	NA	NA
**Body weight change**	Efpeglenatide *vs* Thiazolidinediones	NA	NA	NA	NA
**Body weight change**	Efpeglenatide *vs* Tirzepatide	NA	NA	NA	NA
**Body weight change**	Exenatide extended-release *vs* Exenatide immediate-release	NA	NA	NA	NA
**Body weight change**	Exenatide extended-release *vs* Liraglutide	NA	NA	NA	NA
**Body weight change**	Exenatide extended-release *vs* Lixisenatide	NA	NA	NA	NA
**Body weight change**	Exenatide extended-release *vs* Loxenatide	NA	NA	NA	NA
**Body weight change**	Exenatide extended-release *vs* Meglitinides	NA	NA	NA	NA
**Body weight change**	Exenatide extended-release *vs* Metformin	NA	0.25 (-1.76 to 2.26)	0.242	0.809
**Body weight change**	Exenatide extended-release *vs* Semaglutide (Oral)	NA	NA	NA	NA
**Body weight change**	Exenatide extended-release *vs* Semaglutide (Subcutaneous)	NA	NA	NA	NA
**Body weight change**	Exenatide extended-release *vs* SGLT2 inhibitors	NA	1.43 (-0.9 to 3.76)	1.206	0.228
**Body weight change**	Exenatide extended-release *vs* Standard treatments	NA	-0.37 (-1.75 to 1.01)	-0.523	0.601
**Body weight change**	Exenatide extended-release *vs* Sulfonylureas	NA	NA	NA	NA

Outcome	Comparisons	Ratio of odds ratio	Difference in mean difference	Z statistic	P-value
**Body weight change**	Exenatide extended-release *vs* Thiazolidinediones	NA	-0.55 (-2.12 to 1.02)	-0.687	0.492
**Body weight change**	Exenatide extended-release *vs* Tirzepatide	NA	NA	NA	NA
**Body weight change**	Exenatide immediate-release *vs* Liraglutide	NA	NA	NA	NA
**Body weight change**	Exenatide immediate-release *vs* Lixisenatide	NA	NA	NA	NA
**Body weight change**	Exenatide immediate-release *vs* Loxenatide	NA	NA	NA	NA
**Body weight change**	Exenatide immediate-release *vs* Meglitinides	NA	NA	NA	NA
**Body weight change**	Exenatide immediate-release *vs* Metformin	NA	-1.23 (-3.34 to 0.87)	-1.147	0.251
**Body weight change**	Exenatide immediate-release *vs* Semaglutide (Oral)	NA	NA	NA	NA
**Body weight change**	Exenatide immediate-release *vs* Semaglutide (Subcutaneous)	NA	NA	NA	NA
**Body weight change**	Exenatide immediate-release *vs* SGLT2 inhibitors	NA	NA	NA	NA
**Body weight change**	Exenatide immediate-release *vs* Standard treatments	NA	0.67 (-0.82 to 2.16)	0.878	0.38
**Body weight change**	Exenatide immediate-release *vs* Sulfonylureas	NA	NA	NA	NA
**Body weight change**	Exenatide immediate-release *vs* Thiazolidinediones	NA	NA	NA	NA
**Body weight change**	Exenatide immediate-release *vs* Tirzepatide	NA	NA	NA	NA
**Body weight change**	Liraglutide *vs* Lixisenatide	NA	NA	NA	NA
**Body weight change**	Liraglutide *vs* Loxenatide	NA	NA	NA	NA
**Body weight change**	Liraglutide *vs* Meglitinides	NA	NA	NA	NA
**Body weight change**	Liraglutide *vs* Metformin	NA	NA	NA	NA
**Body weight change**	Liraglutide *vs* Semaglutide (Oral)	NA	0.63 (-1.5 to 2.77)	0.582	0.561
**Body weight change**	Liraglutide *vs* Semaglutide (Subcutaneous)	NA	NA	NA	NA
**Body weight change**	Liraglutide *vs* SGLT2 inhibitors	NA	NA	NA	NA
**Body weight change**	Liraglutide *vs* Standard treatments	NA	-0.9 (-1.65 to -0.15)	-2.34	0.019
**Body weight change**	Liraglutide *vs* Sulfonylureas	NA	0.76 (-0.43 to 1.94)	1.251	0.211
**Body weight change**	Liraglutide *vs* Thiazolidinediones	NA	2.86 (0.84 to 4.88)	2.776	0.006
**Body weight change**	Liraglutide *vs* Tirzepatide	NA	NA	NA	NA

Outcome	Comparisons	Ratio of odds ratio	Difference in mean difference	Z statistic	P-value
**Body weight change**	Lixisenatide *vs* Loxenatide	NA	NA	NA	NA
**Body weight change**	Lixisenatide *vs* Meglitinides	NA	NA	NA	NA
**Body weight change**	Lixisenatide *vs* Metformin	NA	NA	NA	NA
**Body weight change**	Lixisenatide *vs* Semaglutide (Oral)	NA	NA	NA	NA
**Body weight change**	Lixisenatide *vs* Semaglutide (Subcutaneous)	NA	NA	NA	NA
**Body weight change**	Lixisenatide *vs* SGLT2 inhibitors	NA	NA	NA	NA
**Body weight change**	Lixisenatide *vs* Standard treatments	NA	0.08 (-1.48 to 1.65)	0.105	0.917
**Body weight change**	Lixisenatide *vs* Sulfonylureas	NA	NA	NA	NA
**Body weight change**	Lixisenatide *vs* Thiazolidinediones	NA	NA	NA	NA
**Body weight change**	Lixisenatide *vs* Tirzepatide	NA	NA	NA	NA
**Body weight change**	Loxenatide *vs* Meglitinides	NA	NA	NA	NA
**Body weight change**	Loxenatide *vs* Metformin	NA	NA	NA	NA
**Body weight change**	Loxenatide *vs* Semaglutide (Oral)	NA	NA	NA	NA
**Body weight change**	Loxenatide *vs* Semaglutide (Subcutaneous)	NA	NA	NA	NA
**Body weight change**	Loxenatide *vs* SGLT2 inhibitors	NA	NA	NA	NA
**Body weight change**	Loxenatide *vs* Standard treatments	NA	NA	NA	NA
**Body weight change**	Loxenatide *vs* Sulfonylureas	NA	NA	NA	NA
**Body weight change**	Loxenatide *vs* Thiazolidinediones	NA	NA	NA	NA
**Body weight change**	Loxenatide *vs* Tirzepatide	NA	NA	NA	NA
**Body weight change**	Meglitinides *vs* Metformin	NA	0.47 (-2.01 to 2.94)	0.368	0.713
**Body weight change**	Meglitinides *vs* Semaglutide (Oral)	NA	NA	NA	NA
**Body weight change**	Meglitinides *vs* Semaglutide (Subcutaneous)	NA	NA	NA	NA
**Body weight change**	Meglitinides *vs* SGLT2 inhibitors	NA	NA	NA	NA
**Body weight change**	Meglitinides *vs* Standard treatments	NA	-0.41 (-1.81 to 0.98)	-0.581	0.561
**Body weight change**	Meglitinides *vs* Sulfonylureas	NA	0.09 (-1.29 to 1.47)	0.124	0.902

Outcome	Comparisons	Ratio of odds ratio	Difference in mean difference	Z statistic	P-value
**Body weight change**	Meglitinides *vs* Thiazolidinediones	NA	0.37 (-1.65 to 2.39)	0.36	0.719
**Body weight change**	Meglitinides *vs* Tirzepatide	NA	NA	NA	NA
**Body weight change**	Metformin *vs* Semaglutide (Oral)	NA	NA	NA	NA
**Body weight change**	Metformin *vs* Semaglutide (Subcutaneous)	NA	NA	NA	NA
**Body weight change**	Metformin *vs* SGLT2 inhibitors	NA	0.71 (-0.25 to 1.66)	1.449	0.147
**Body weight change**	Metformin *vs* Standard treatments	NA	0.23 (-0.56 to 1.02)	0.566	0.571
**Body weight change**	Metformin *vs* Sulfonylureas	NA	0.1 (-0.82 to 1.02)	0.214	0.831
**Body weight change**	Metformin *vs* Thiazolidinediones	NA	-0.7 (-1.49 to 0.08)	-1.759	0.079
**Body weight change**	Metformin *vs* Tirzepatide	NA	NA	NA	NA
**Body weight change**	Semaglutide (Oral) *vs* Semaglutide (Subcutaneous)	NA	-0.05 (-2.58 to 2.48)	-0.041	0.967
**Body weight change**	Semaglutide (Oral) *vs* SGLT2 inhibitors	NA	0.83 (-1.23 to 2.9)	0.792	0.428
**Body weight change**	Semaglutide (Oral) *vs* Standard treatments	NA	-1.18 (-2.65 to 0.3)	-1.563	0.118
**Body weight change**	Semaglutide (Oral) *vs* Sulfonylureas	NA	NA	NA	NA
**Body weight change**	Semaglutide (Oral) *vs* Thiazolidinediones	NA	NA	NA	NA
**Body weight change**	Semaglutide (Oral) *vs* Tirzepatide	NA	NA	NA	NA
**Body weight change**	Semaglutide (Subcutaneous) *vs* SGLT2 inhibitors	NA	1.42 (-0.55 to 3.4)	1.412	0.158
**Body weight change**	Semaglutide (Subcutaneous) *vs* Standard treatments	NA	-0.48 (-1.68 to 0.72)	-0.79	0.429
**Body weight change**	Semaglutide (Subcutaneous) *vs* Sulfonylureas	NA	NA	NA	NA
**Body weight change**	Semaglutide (Subcutaneous) *vs* Thiazolidinediones	NA	NA	NA	NA
**Body weight change**	Semaglutide (Subcutaneous) *vs* Tirzepatide	NA	0.16 (-1.82 to 2.15)	0.162	0.871
**Body weight change**	SGLT2 inhibitors *vs* Standard treatments	NA	0.3 (-0.15 to 0.75)	1.315	0.188
**Body weight change**	SGLT2 inhibitors *vs* Sulfonylureas	NA	-0.71 (-1.46 to 0.04)	-1.864	0.062
**Body weight change**	SGLT2 inhibitors *vs* Thiazolidinediones	NA	0.5 (-0.82 to 1.81)	0.742	0.458
**Body weight change**	SGLT2 inhibitors *vs* Tirzepatide	NA	NA	NA	NA
**Body weight change**	Sulfonylureas *vs* Standard treatments	NA	1 (-0.01 to 2.02)	1.938	0.053

Outcome	Comparisons	Ratio of odds ratio	Difference in mean difference	Z statistic	P-value
**Body weight change**	Thiazolidinediones *vs* Standard treatments	NA	0.45 (-0.07 to 0.97)	1.698	0.089
**Body weight change**	Tirzepatide *vs* Standard treatments	NA	0.91 (-0.76 to 2.57)	1.07	0.285
**Body weight change**	Sulfonylureas *vs* Thiazolidinediones	NA	-0.24 (-0.88 to 0.39)	-0.75	0.453
**Body weight change**	Sulfonylureas *vs* Tirzepatide	NA	NA	NA	NA
**Body weight change**	Thiazolidinediones *vs* Tirzepatide	NA	NA	NA	NA

4.7 Publication bias assessments

4.7.1 Small-study effect assessed by Harbord's test or Egger's test in pairwise meta-analyses

Notes: The study evaluated the small-study effect (a source of publication bias) using Harbord's score regression method in the pairwise meta-analyses for binary outcomes (Egger's method for continuous outcomes). It demonstrated that Harbord's method performs well in binary outcome settings than Egger's method. We only performed the tests for the meta-analyses with at least 5 trials. We also performed the trim and fill analysis for the meta-analyses with at least 10 trials using both L-type estimator and R-type estimator to assess the robustness.

Abbreviations: k, no. of studies; df, degree of freedom.

Outcome	Comparisons	k	T-statistic	df	P-value	Bias estimate	tau	Odds ratio	Trim and fill odds ratio (L estimator)	Trim and fill odds ratio (R estimator)	Mean difference	Trim and fill mean difference (L estimator)	Trim and fill mean difference (R estimator)
**All-cause death**	Alpha-glucosidase inhibitors *vs* Meglitinides	1	NA	NA	NA	NA	NA	0.33 (0.01 to 8.1)	NA	NA	NA	NA	NA
**All-cause death**	Alpha-glucosidase inhibitors *vs* Standard treatments	5	-0.132	3	0.903	-0.35	1.015	1.31 (0.33 to 5.18)	NA	NA	NA	NA	NA
**All-cause death**	Alpha-glucosidase inhibitors *vs* Sulfonylureas	1	NA	NA	NA	NA	NA	0.64 (0.13 to 3.21)	NA	NA	NA	NA	NA
**All-cause death**	Basal insulin *vs* Basal-Bolus insulin	1	NA	NA	NA	NA	NA	7.03 (0.36 to 136.82)	NA	NA	NA	NA	NA
**All-cause death**	Basal insulin *vs* Bolus insulin	1	NA	NA	NA	NA	NA	0.34 (0.01 to 8.36)	NA	NA	NA	NA	NA
**All-cause death**	Basal insulin *vs* DPP-4 inhibitors	4	NA	NA	NA	NA	NA	0.99 (0.65 to 1.52)	NA	NA	NA	NA	NA

Outcome	Comparisons	k	T-statistic	df	P-value	Bias estimate	tau	Odds ratio	Trim and fill odds ratio (L estimator)	Trim and fill odds ratio (R estimator)	Mean difference	Trim and fill mean difference (L estimator)	Trim and fill mean difference (R estimator)
**All-cause death**	Basal insulin *vs* GLP-1 receptor agonists	9	-0.996	7	0.352	-0.445	0.822	1.44 (0.98 to 2.12)	NA	NA	NA	NA	NA
**All-cause death**	Basal insulin *vs* Standard treatments	1	NA	NA	NA	NA	NA	0.32 (0.01 to 7.99)	NA	NA	NA	NA	NA
**All-cause death**	Basal insulin *vs* Sulfonylureas	1	NA	NA	NA	NA	NA	0.97 (0.63 to 1.49)	NA	NA	NA	NA	NA
**All-cause death**	Basal insulin *vs* Tirzepatide	2	NA	NA	NA	NA	NA	1.36 (0.82 to 2.26)	NA	NA	NA	NA	NA
**All-cause death**	Basal-Bolus insulin *vs* GLP-1 receptor agonists	2	NA	NA	NA	NA	NA	0.75 (0.11 to 5.16)	NA	NA	NA	NA	NA
**All-cause death**	Basal-Bolus insulin *vs* Sulfonylureas	1	NA	NA	NA	NA	NA	6.63 (0.3 to 145.31)	NA	NA	NA	NA	NA
**All-cause death**	Bolus insulin *vs* GLP-1 receptor agonists	4	NA	NA	NA	NA	NA	0.45 (0.1 to 2.07)	NA	NA	NA	NA	NA
**All-cause death**	DPP-4 inhibitors *vs* GLP-1 receptor agonists	12	-0.558	10	0.589	-0.227	0.927	1.37 (0.93 to 2.02)	1.45 (1 to 2.11)	1.45 (1 to 2.11)	NA	NA	NA
**All-cause death**	DPP-4 inhibitors *vs* Metformin	5	1.41	3	0.253	1.783	0.896	1.1 (0.32 to 3.81)	NA	NA	NA	NA	NA
**All-cause death**	DPP-4 inhibitors *vs* SGLT2 inhibitors	6	-0.254	4	0.812	-0.43	0.795	0.5 (0.15 to 1.63)	NA	NA	NA	NA	NA
**All-cause death**	DPP-4 inhibitors *vs* Standard treatments	48	-0.893	46	0.377	-0.139	0.956	1.02 (0.95 to 1.1)	1.03 (0.96 to 1.1)	1.02 (0.95 to 1.09)	NA	NA	NA

Outcome	Comparisons	k	T-statistic	df	P-value	Bias estimate	tau	Odds ratio	Trim and fill odds ratio (L estimator)	Trim and fill odds ratio (R estimator)	Mean difference	Trim and fill mean difference (L estimator)	Trim and fill mean difference (R estimator)
**All-cause death**	DPP-4 inhibitors *vs* Sulfonylureas	17	-1.943	15	0.071	-0.446	0.775	0.88 (0.76 to 1.01)	0.9 (0.79 to 1.03)	0.89 (0.78 to 1.01)	NA	NA	NA
**All-cause death**	DPP-4 inhibitors *vs* Thiazolidinediones	3	NA	NA	NA	NA	NA	2.13 (0.43 to 10.59)	NA	NA	NA	NA	NA
**All-cause death**	GLP-1 receptor agonists *vs* Metformin	1	NA	NA	NA	NA	NA	0.33 (0.01 to 8.12)	NA	NA	NA	NA	NA
**All-cause death**	GLP-1 receptor agonists *vs* SGLT2 inhibitors	3	NA	NA	NA	NA	NA	1.3 (0.25 to 6.78)	NA	NA	NA	NA	NA
**All-cause death**	GLP-1 receptor agonists *vs* Standard treatments	40	-0.263	38	0.794	-0.047	0.969	0.88 (0.83 to 0.94)	0.89 (0.84 to 0.94)	0.89 (0.84 to 0.94)	NA	NA	NA
**All-cause death**	GLP-1 receptor agonists *vs* Sulfonylureas	6	0.81	4	0.464	0.42	0.865	0.68 (0.44 to 1.04)	NA	NA	NA	NA	NA
**All-cause death**	GLP-1 receptor agonists *vs* Thiazolidinediones	1	NA	NA	NA	NA	NA	0.37 (0.05 to 2.78)	NA	NA	NA	NA	NA
**All-cause death**	GLP-1 receptor agonists *vs* Tirzepatide	2	NA	NA	NA	NA	NA	0.4 (0.07 to 2.24)	NA	NA	NA	NA	NA
**All-cause death**	Meglitinides *vs* Metformin	1	NA	NA	NA	NA	NA	0.33 (0.01 to 8.15)	NA	NA	NA	NA	NA
**All-cause death**	Meglitinides *vs* Standard treatments	1	NA	NA	NA	NA	NA	1.72 (0.15 to 19.01)	NA	NA	NA	NA	NA
**All-cause death**	Meglitinides *vs* Sulfonylureas	3	NA	NA	NA	NA	NA	1.81 (0.39 to 8.46)	NA	NA	NA	NA	NA

Outcome	Comparisons	k	T-statistic	df	P-value	Bias estimate	tau	Odds ratio	Trim and fill odds ratio (L estimator)	Trim and fill odds ratio (R estimator)	Mean difference	Trim and fill mean difference (L estimator)	Trim and fill mean difference (R estimator)
**All-cause death**	Meglitinides *vs* Thiazolidinediones	1	NA	NA	NA	NA	NA	1.51 (0.06 to 37.15)	NA	NA	NA	NA	NA
**All-cause death**	Metformin *vs* SGLT2 inhibitors	3	NA	NA	NA	NA	NA	1.86 (0.29 to 11.83)	NA	NA	NA	NA	NA
**All-cause death**	Metformin *vs* Standard treatments	5	1.952	3	0.146	0.895	0.84	0.93 (0.52 to 1.65)	NA	NA	NA	NA	NA
**All-cause death**	Metformin *vs* Sulfonylureas	5	1.441	3	0.245	1.04	1.074	0.88 (0.49 to 1.6)	NA	NA	NA	NA	NA
**All-cause death**	Metformin *vs* Thiazolidinediones	8	0.569	6	0.59	0.251	0.915	0.94 (0.6 to 1.46)	NA	NA	NA	NA	NA
**All-cause death**	Non-steroidal MRAs *vs* Standard treatments	3	NA	NA	NA	NA	NA	0.89 (0.79 to 1)	NA	NA	NA	NA	NA
**All-cause death**	SGLT2 inhibitors *vs* Standard treatments	39	1.38	37	0.176	0.257	0.986	0.88 (0.8 to 0.96)	0.87 (0.8 to 0.95)	0.88 (0.81 to 0.96)	NA	NA	NA
**All-cause death**	SGLT2 inhibitors *vs* Sulfonylureas	5	-1.423	3	0.25	-2.721	1.089	0.78 (0.33 to 1.86)	NA	NA	NA	NA	NA
**All-cause death**	Sulfonylureas *vs* Standard treatments	2	NA	NA	NA	NA	NA	1.78 (0.29 to 11)	NA	NA	NA	NA	NA
**All-cause death**	Sulfonylureas *vs* Thiazolidinediones	10	0.779	8	0.459	0.412	1.089	0.97 (0.74 to 1.28)	0.94 (0.72 to 1.23)	0.93 (0.71 to 1.21)	NA	NA	NA
**All-cause death**	Thiazolidinediones *vs* Standard treatments	19	0.474	17	0.642	0.121	0.943	0.91 (0.78 to 1.06)	1.08 (0.94 to 1.25)	1.09 (0.94 to 1.25)	NA	NA	NA

Outcome	Comparisons	k	T-statistic	df	P-value	Bias estimate	tau	Odds ratio	Trim and fill odds ratio (L estimator)	Trim and fill odds ratio (R estimator)	Mean difference	Trim and fill mean difference (L estimator)	Trim and fill mean difference (R estimator)
**All-cause death**	Tirzepatide *vs* Standard treatments	2	NA	NA	NA	NA	NA	0.28 (0.03 to 2.68)	NA	NA	NA	NA	NA
**Cardiovascular death**	Alpha-glucosidase inhibitors *vs* Standard treatments	3	NA	NA	NA	NA	NA	1.54 (0.24 to 9.79)	NA	NA	NA	NA	NA
**Cardiovascular death**	Alpha-glucosidase inhibitors *vs* Sulfonylureas	1	NA	NA	NA	NA	NA	0.51 (0.05 to 4.97)	NA	NA	NA	NA	NA
**Cardiovascular death**	Basal insulin *vs* Bolus insulin	1	NA	NA	NA	NA	NA	0.34 (0.01 to 8.36)	NA	NA	NA	NA	NA
**Cardiovascular death**	Basal insulin *vs* DPP-4 inhibitors	2	NA	NA	NA	NA	NA	1.04 (0.57 to 1.9)	NA	NA	NA	NA	NA
**Cardiovascular death**	Basal insulin *vs* GLP-1 receptor agonists	6	-0.107	4	0.92	-0.103	1.205	1.86 (1.04 to 3.31)	NA	NA	NA	NA	NA
**Cardiovascular death**	Basal insulin *vs* Sulfonylureas	1	NA	NA	NA	NA	NA	1.31 (0.68 to 2.52)	NA	NA	NA	NA	NA
**Cardiovascular death**	Basal insulin *vs* Tirzepatide	1	NA	NA	NA	NA	NA	1.5 (0.53 to 4.22)	NA	NA	NA	NA	NA
**Cardiovascular death**	Basal-Bolus insulin *vs* GLP-1 receptor agonists	1	NA	NA	NA	NA	NA	1.51 (0.06 to 37.31)	NA	NA	NA	NA	NA
**Cardiovascular death**	Basal-Bolus insulin *vs* Sulfonylureas	1	NA	NA	NA	NA	NA	3.8 (0.15 to 97.81)	NA	NA	NA	NA	NA

Outcome	Comparisons	k	T-statistic	df	P-value	Bias estimate	tau	Odds ratio	Trim and fill odds ratio (L estimator)	Trim and fill odds ratio (R estimator)	Mean difference	Trim and fill mean difference (L estimator)	Trim and fill mean difference (R estimator)
**Cardiovascular death**	Bolus insulin *vs* GLP-1 receptor agonists	1	NA	NA	NA	NA	NA	0.34 (0.01 to 8.27)	NA	NA	NA	NA	NA
**Cardiovascular death**	DPP-4 inhibitors *vs* GLP-1 receptor agonists	8	-0.461	6	0.661	-0.28	1.06	1.94 (1.03 to 3.65)	NA	NA	NA	NA	NA
**Cardiovascular death**	DPP-4 inhibitors *vs* Metformin	1	NA	NA	NA	NA	NA	2.98 (0.12 to 73.73)	NA	NA	NA	NA	NA
**Cardiovascular death**	DPP-4 inhibitors *vs* SGLT2 inhibitors	3	NA	NA	NA	NA	NA	0.29 (0.05 to 1.72)	NA	NA	NA	NA	NA
**Cardiovascular death**	DPP-4 inhibitors *vs* Standard treatments	28	-0.3	26	0.766	-0.065	0.986	0.99 (0.91 to 1.08)	1 (0.92 to 1.08)	0.99 (0.92 to 1.08)	NA	NA	NA
**Cardiovascular death**	DPP-4 inhibitors *vs* Sulfonylureas	10	-0.908	8	0.39	-0.306	0.851	1 (0.82 to 1.22)	1.01 (0.83 to 1.22)	1 (0.83 to 1.21)	NA	NA	NA
**Cardiovascular death**	DPP-4 inhibitors *vs* Thiazolidinediones	2	NA	NA	NA	NA	NA	5.24 (0.54 to 50.59)	NA	NA	NA	NA	NA
**Cardiovascular death**	GLP-1 receptor agonists *vs* Standard treatments	24	-0.608	22	0.55	-0.148	0.936	0.88 (0.81 to 0.95)	0.89 (0.82 to 0.95)	0.89 (0.82 to 0.95)	NA	NA	NA
**Cardiovascular death**	GLP-1 receptor agonists *vs* Sulfonylureas	1	NA	NA	NA	NA	NA	0.56 (0.25 to 1.27)	NA	NA	NA	NA	NA
**Cardiovascular death**	GLP-1 receptor agonists *vs* Thiazolidinediones	0	NA	NA	NA	NA	NA	1.03 (0.02 to 52.28)	NA	NA	NA	NA	NA
**Cardiovascular death**	GLP-1 receptor agonists *vs* Tirzepatide	1	NA	NA	NA	NA	NA	0.27 (0.02 to 4.93)	NA	NA	NA	NA	NA

Outcome	Comparisons	k	T-statistic	df	P-value	Bias estimate	tau	Odds ratio	Trim and fill odds ratio (L estimator)	Trim and fill odds ratio (R estimator)	Mean difference	Trim and fill mean difference (L estimator)	Trim and fill mean difference (R estimator)
**Cardiovascular death**	Meglitinides *vs* Metformin	1	NA	NA	NA	NA	NA	0.33 (0.01 to 8.15)	NA	NA	NA	NA	NA
**Cardiovascular death**	Meglitinides *vs* Standard treatments	0	NA	NA	NA	NA	NA	0.96 (0.02 to 48.7)	NA	NA	NA	NA	NA
**Cardiovascular death**	Meglitinides *vs* Sulfonylureas	1	NA	NA	NA	NA	NA	0.5 (0.03 to 8.08)	NA	NA	NA	NA	NA
**Cardiovascular death**	Meglitinides *vs* Thiazolidinediones	1	NA	NA	NA	NA	NA	1.51 (0.06 to 37.15)	NA	NA	NA	NA	NA
**Cardiovascular death**	Metformin *vs* SGLT2 inhibitors	3	NA	NA	NA	NA	NA	1.86 (0.29 to 11.83)	NA	NA	NA	NA	NA
**Cardiovascular death**	Metformin *vs* Standard treatments	2	NA	NA	NA	NA	NA	2.97 (0.47 to 18.97)	NA	NA	NA	NA	NA
**Cardiovascular death**	Metformin *vs* Sulfonylureas	3	NA	NA	NA	NA	NA	0.89 (0.27 to 2.89)	NA	NA	NA	NA	NA
**Cardiovascular death**	Metformin *vs* Thiazolidinediones	3	NA	NA	NA	NA	NA	0.7 (0.11 to 4.54)	NA	NA	NA	NA	NA
**Cardiovascular death**	Non-steroidal MRAs *vs* Standard treatments	2	NA	NA	NA	NA	NA	0.88 (0.75 to 1.02)	NA	NA	NA	NA	NA
**Cardiovascular death**	SGLT2 inhibitors *vs* Standard treatments	19	-0.195	17	0.848	-0.075	1.238	0.85 (0.75 to 0.96)	0.86 (0.76 to 0.96)	0.86 (0.77 to 0.97)	NA	NA	NA
**Cardiovascular death**	SGLT2 inhibitors *vs* Sulfonylureas	2	NA	NA	NA	NA	NA	1.19 (0.15 to 9.68)	NA	NA	NA	NA	NA

Outcome	Comparisons	k	T-statistic	df	P-value	Bias estimate	tau	Odds ratio	Trim and fill odds ratio (L estimator)	Trim and fill odds ratio (R estimator)	Mean difference	Trim and fill mean difference (L estimator)	Trim and fill mean difference (R estimator)
**Cardiovascular death**	Sulfonylureas *vs* Standard treatments	1	NA	NA	NA	NA	NA	3.03 (0.12 to 75.42)	NA	NA	NA	NA	NA
**Cardiovascular death**	Sulfonylureas *vs* Thiazolidinediones	7	0.229	5	0.828	0.274	1.221	0.85 (0.45 to 1.59)	NA	NA	NA	NA	NA
**Cardiovascular death**	Thiazolidinediones *vs* Standard treatments	14	-0.492	12	0.631	-0.142	0.883	0.91 (0.75 to 1.11)	1.08 (0.9 to 1.3)	1.08 (0.89 to 1.3)	NA	NA	NA
**Non-fatal myocardial infarction**	Alpha-glucosidase inhibitors *vs* Meglitinides	0	NA	NA	NA	NA	NA	0.99 (0.02 to 49.94)	NA	NA	NA	NA	NA
**Non-fatal myocardial infarction**	Alpha-glucosidase inhibitors *vs* Standard treatments	2	NA	NA	NA	NA	NA	0.3 (0.03 to 2.7)	NA	NA	NA	NA	NA
**Non-fatal myocardial infarction**	Alpha-glucosidase inhibitors *vs* Sulfonylureas	1	NA	NA	NA	NA	NA	0.4 (0.02 to 8.3)	NA	NA	NA	NA	NA
**Non-fatal myocardial infarction**	Basal insulin *vs* Basal-Bolus insulin	1	NA	NA	NA	NA	NA	2.92 (0.12 to 72.15)	NA	NA	NA	NA	NA
**Non-fatal myocardial infarction**	Basal insulin *vs* Bolus insulin	1	NA	NA	NA	NA	NA	0.76 (0.17 to 3.45)	NA	NA	NA	NA	NA

Outcome	Comparisons	k	T-statistic	df	P-value	Bias estimate	tau	Odds ratio	Trim and fill odds ratio (L estimator)	Trim and fill odds ratio (R estimator)	Mean difference	Trim and fill mean difference (L estimator)	Trim and fill mean difference (R estimator)
**Non-fatal myocardial infarction**	Basal insulin *vs* DPP-4 inhibitors	1	NA	NA	NA	NA	NA	0.37 (0.01 to 9.12)	NA	NA	NA	NA	NA
**Non-fatal myocardial infarction**	Basal insulin *vs* GLP-1 receptor agonists	7	-0.379	5	0.72	-0.396	1.2	1.19 (0.49 to 2.91)	NA	NA	NA	NA	NA
**Non-fatal myocardial infarction**	Basal insulin *vs* Standard treatments	1	NA	NA	NA	NA	NA	3 (0.12 to 74.19)	NA	NA	NA	NA	NA
**Non-fatal myocardial infarction**	Basal insulin *vs* Thiazolidinediones	1	NA	NA	NA	NA	NA	0.33 (0.01 to 8.21)	NA	NA	NA	NA	NA
**Non-fatal myocardial infarction**	Basal-Bolus insulin *vs* GLP-1 receptor agonists	1	NA	NA	NA	NA	NA	0.35 (0.01 to 8.68)	NA	NA	NA	NA	NA
**Non-fatal myocardial infarction**	Bolus insulin *vs* GLP-1 receptor agonists	4	NA	NA	NA	NA	NA	1.2 (0.29 to 4.9)	NA	NA	NA	NA	NA
**Non-fatal myocardial infarction**	DPP-4 inhibitors *vs* GLP-1 receptor agonists	7	-0.167	5	0.874	-0.196	1.008	1.1 (0.45 to 2.68)	1.03 (0.38 to 2.79)	1.03 (0.38 to 2.79)	NA	NA	NA

Outcome	Comparisons	k	T-statistic	df	P-value	Bias estimate	tau	Odds ratio	Trim and fill odds ratio (L estimator)	Trim and fill odds ratio (R estimator)	Mean difference	Trim and fill mean difference (L estimator)	Trim and fill mean difference (R estimator)
**Non-fatal myocardial infarction**	DPP-4 inhibitors *vs* Metformin	4	NA	NA	NA	NA	NA	0.48 (0.11 to 2.1)	NA	NA	NA	NA	NA
**Non-fatal myocardial infarction**	DPP-4 inhibitors *vs* SGLT2 inhibitors	8	-2.047	6	0.087	-3.437	0.809	1.09 (0.38 to 3.11)	NA	NA	NA	NA	NA
**Non-fatal myocardial infarction**	DPP-4 inhibitors *vs* Standard treatments	45	-0.302	43	0.764	-0.051	0.991	1.02 (0.93 to 1.12)	1.03 (0.94 to 1.12)	1.03 (0.94 to 1.13)	NA	NA	NA
**Non-fatal myocardial infarction**	DPP-4 inhibitors *vs* Sulfonylureas	12	-2.566	10	0.028	-0.721	0.802	0.89 (0.64 to 1.24)	0.99 (0.8 to 1.22)	0.96 (0.75 to 1.21)	NA	NA	NA
**Non-fatal myocardial infarction**	DPP-4 inhibitors *vs* Thiazolidinediones	5	-0.746	3	0.51	-3.07	1.48	1.01 (0.23 to 4.38)	NA	NA	NA	NA	NA
**Non-fatal myocardial infarction**	GLP-1 receptor agonists *vs* Metformin	1	NA	NA	NA	NA	NA	1.27 (0.11 to 15.18)	NA	NA	NA	NA	NA
**Non-fatal myocardial infarction**	GLP-1 receptor agonists *vs* SGLT2 inhibitors	1	NA	NA	NA	NA	NA	5.11 (0.24 to 107.01)	NA	NA	NA	NA	NA

Outcome	Comparisons	k	T-statistic	df	P-value	Bias estimate	tau	Odds ratio	Trim and fill odds ratio (L estimator)	Trim and fill odds ratio (R estimator)	Mean difference	Trim and fill mean difference (L estimator)	Trim and fill mean difference (R estimator)
**Non-fatal myocardial infarction**	GLP-1 receptor agonists *vs* Standard treatments	37	-0.145	35	0.886	-0.029	1.03	0.91 (0.81 to 1.01)	0.91 (0.83 to 1.01)	0.91 (0.82 to 1.01)	NA	NA	NA
**Non-fatal myocardial infarction**	GLP-1 receptor agonists *vs* Sulfonylureas	4	NA	NA	NA	NA	NA	1.05 (0.36 to 3.05)	NA	NA	NA	NA	NA
**Non-fatal myocardial infarction**	GLP-1 receptor agonists *vs* Thiazolidinediones	2	NA	NA	NA	NA	NA	0.21 (0.02 to 1.91)	NA	NA	NA	NA	NA
**Non-fatal myocardial infarction**	GLP-1 receptor agonists *vs* Tirzepatide	1	NA	NA	NA	NA	NA	0.73 (0.07 to 8.1)	NA	NA	NA	NA	NA
**Non-fatal myocardial infarction**	Meglitinides *vs* Standard treatments	1	NA	NA	NA	NA	NA	1.33 (0.06 to 27.93)	NA	NA	NA	NA	NA
**Non-fatal myocardial infarction**	Meglitinides *vs* Sulfonylureas	2	NA	NA	NA	NA	NA	0.14 (0.02 to 1.12)	NA	NA	NA	NA	NA
**Non-fatal myocardial infarction**	Metformin *vs* SGLT2 inhibitors	1	NA	NA	NA	NA	NA	1.43 (0.06 to 35.52)	NA	NA	NA	NA	NA

Outcome	Comparisons	k	T-statistic	df	P-value	Bias estimate	tau	Odds ratio	Trim and fill odds ratio (L estimator)	Trim and fill odds ratio (R estimator)	Mean difference	Trim and fill mean difference (L estimator)	Trim and fill mean difference (R estimator)
**Non-fatal myocardial infarction**	Metformin *vs* Standard treatments	4	NA	NA	NA	NA	NA	0.88 (0.56 to 1.38)	NA	NA	NA	NA	NA
**Non-fatal myocardial infarction**	Metformin *vs* Sulfonylureas	4	NA	NA	NA	NA	NA	1.24 (0.71 to 2.17)	NA	NA	NA	NA	NA
**Non-fatal myocardial infarction**	Metformin *vs* Thiazolidinediones	5	-0.861	3	0.452	-0.552	1.05	0.79 (0.46 to 1.37)	NA	NA	NA	NA	NA
**Non-fatal myocardial infarction**	Non-steroidal MRAs *vs* Standard treatments	2	NA	NA	NA	NA	NA	0.91 (0.73 to 1.13)	NA	NA	NA	NA	NA
**Non-fatal myocardial infarction**	SGLT2 inhibitors *vs* Standard treatments	27	0.12	25	0.905	0.03	1.086	0.9 (0.82 to 0.98)	0.91 (0.83 to 0.99)	0.91 (0.83 to 0.99)	NA	NA	NA
**Non-fatal myocardial infarction**	SGLT2 inhibitors *vs* Sulfonylureas	4	NA	NA	NA	NA	NA	0.67 (0.14 to 3.11)	NA	NA	NA	NA	NA
**Non-fatal myocardial infarction**	Sulfonylureas *vs* Standard treatments	4	NA	NA	NA	NA	NA	0.48 (0.12 to 1.81)	NA	NA	NA	NA	NA

Outcome	Comparisons	k	T-statistic	df	P-value	Bias estimate	tau	Odds ratio	Trim and fill odds ratio (L estimator)	Trim and fill odds ratio (R estimator)	Mean difference	Trim and fill mean difference (L estimator)	Trim and fill mean difference (R estimator)
**Non-fatal myocardial infarction**	Sulfonylureas *vs* Thiazolidinediones	12	-0.185	10	0.857	-0.092	1.017	0.96 (0.66 to 1.39)	0.96 (0.67 to 1.39)	0.96 (0.67 to 1.39)	NA	NA	NA
**Non-fatal myocardial infarction**	Thiazolidinediones *vs* Standard treatments	17	-1.299	15	0.214	-0.391	1.032	0.97 (0.74 to 1.27)	1.07 (0.83 to 1.38)	1.09 (0.85 to 1.39)	NA	NA	NA
**Non-fatal myocardial infarction**	Tirzepatide *vs* Standard treatments	1	NA	NA	NA	NA	NA	0.2 (0.01 to 5.12)	NA	NA	NA	NA	NA
**Non-fatal stroke**	Alpha-glucosidase inhibitors *vs* Meglitinides	1	NA	NA	NA	NA	NA	4.96 (0.24 to 103.63)	NA	NA	NA	NA	NA
**Non-fatal stroke**	Alpha-glucosidase inhibitors *vs* Sulfonylureas	1	NA	NA	NA	NA	NA	10 (0.48 to 208.75)	NA	NA	NA	NA	NA
**Non-fatal stroke**	Basal insulin *vs* Basal-Bolus insulin	1	NA	NA	NA	NA	NA	1.95 (0.18 to 21.64)	NA	NA	NA	NA	NA
**Non-fatal stroke**	Basal insulin *vs* DPP-4 inhibitors	1	NA	NA	NA	NA	NA	3.36 (0.14 to 82.76)	NA	NA	NA	NA	NA
**Non-fatal stroke**	Basal insulin *vs* GLP-1 receptor agonists	11	0.762	9	0.466	1.12	1.023	0.81 (0.32 to 2.08)	0.81 (0.32 to 2.08)	0.81 (0.32 to 2.08)	NA	NA	NA
**Non-fatal stroke**	Basal insulin *vs* Thiazolidinediones	1	NA	NA	NA	NA	NA	0.32 (0.01 to 8.26)	NA	NA	NA	NA	NA

Outcome	Comparisons	k	T-statistic	df	P-value	Bias estimate	tau	Odds ratio	Trim and fill odds ratio (L estimator)	Trim and fill odds ratio (R estimator)	Mean difference	Trim and fill mean difference (L estimator)	Trim and fill mean difference (R estimator)
**Non-fatal stroke**	Basal-Bolus insulin *vs* GLP-1 receptor agonists	1	NA	NA	NA	NA	NA	1.03 (0.06 to 16.62)	NA	NA	NA	NA	NA
**Non-fatal stroke**	Basal-Bolus insulin *vs* Thiazolidinediones	1	NA	NA	NA	NA	NA	0.99 (0.06 to 15.92)	NA	NA	NA	NA	NA
**Non-fatal stroke**	Bolus insulin *vs* GLP-1 receptor agonists	3	NA	NA	NA	NA	NA	1.01 (0.19 to 5.24)	NA	NA	NA	NA	NA
**Non-fatal stroke**	DPP-4 inhibitors *vs* GLP-1 receptor agonists	7	0.356	5	0.736	0.357	1.252	0.98 (0.34 to 2.82)	NA	NA	NA	NA	NA
**Non-fatal stroke**	DPP-4 inhibitors *vs* Metformin	5	-2.86	3	0.065	-4.942	0.664	0.79 (0.2 to 3.13)	NA	NA	NA	NA	NA
**Non-fatal stroke**	DPP-4 inhibitors *vs* SGLT2 inhibitors	6	1.494	4	0.209	4.25	0.796	0.93 (0.28 to 3.06)	NA	NA	NA	NA	NA
**Non-fatal stroke**	DPP-4 inhibitors *vs* Standard treatments	38	-2.061	36	0.047	-0.378	0.939	0.93 (0.82 to 1.07)	0.96 (0.84 to 1.09)	0.96 (0.84 to 1.09)	NA	NA	NA
**Non-fatal stroke**	DPP-4 inhibitors *vs* Sulfonylureas	13	-1.956	11	0.076	-0.687	1.014	0.66 (0.41 to 1.07)	0.86 (0.67 to 1.11)	0.66 (0.41 to 1.07)	NA	NA	NA
**Non-fatal stroke**	DPP-4 inhibitors *vs* Thiazolidinediones	3	NA	NA	NA	NA	NA	0.7 (0.2 to 2.45)	NA	NA	NA	NA	NA
**Non-fatal stroke**	GLP-1 receptor agonists *vs* Metformin	2	NA	NA	NA	NA	NA	0.7 (0.07 to 6.77)	NA	NA	NA	NA	NA
**Non-fatal stroke**	GLP-1 receptor agonists *vs* SGLT2 inhibitors	2	NA	NA	NA	NA	NA	3.68 (0.6 to 22.52)	NA	NA	NA	NA	NA

Outcome	Comparisons	k	T-statistic	df	P-value	Bias estimate	tau	Odds ratio	Trim and fill odds ratio (L estimator)	Trim and fill odds ratio (R estimator)	Mean difference	Trim and fill mean difference (L estimator)	Trim and fill mean difference (R estimator)
**Non-fatal stroke**	GLP-1 receptor agonists *vs* Standard treatments	30	0.11	28	0.913	0.025	0.975	0.85 (0.76 to 0.94)	0.86 (0.77 to 0.95)	0.86 (0.78 to 0.95)	NA	NA	NA
**Non-fatal stroke**	GLP-1 receptor agonists *vs* Sulfonylureas	4	NA	NA	NA	NA	NA	0.87 (0.21 to 3.52)	NA	NA	NA	NA	NA
**Non-fatal stroke**	GLP-1 receptor agonists *vs* Thiazolidinediones	2	NA	NA	NA	NA	NA	0.62 (0.1 to 3.98)	NA	NA	NA	NA	NA
**Non-fatal stroke**	Meglitinides *vs* Standard treatments	1	NA	NA	NA	NA	NA	0.34 (0.01 to 8.71)	NA	NA	NA	NA	NA
**Non-fatal stroke**	Meglitinides *vs* Sulfonylureas	1	NA	NA	NA	NA	NA	3.74 (0.36 to 38.67)	NA	NA	NA	NA	NA
**Non-fatal stroke**	Metformin *vs* Standard treatments	2	NA	NA	NA	NA	NA	0.97 (0.63 to 1.47)	NA	NA	NA	NA	NA
**Non-fatal stroke**	Metformin *vs* Sulfonylureas	2	NA	NA	NA	NA	NA	0.87 (0.49 to 1.55)	NA	NA	NA	NA	NA
**Non-fatal stroke**	Metformin *vs* Thiazolidinediones	3	NA	NA	NA	NA	NA	1.26 (0.67 to 2.4)	NA	NA	NA	NA	NA
**Non-fatal stroke**	Non-steroidal MRAs *vs* Standard treatments	2	NA	NA	NA	NA	NA	1 (0.82 to 1.22)	NA	NA	NA	NA	NA
**Non-fatal stroke**	SGLT2 inhibitors *vs* Standard treatments	27	-0.477	25	0.638	-0.1	0.925	0.99 (0.87 to 1.12)	0.99 (0.88 to 1.12)	1 (0.89 to 1.13)	NA	NA	NA
**Non-fatal stroke**	SGLT2 inhibitors *vs* Sulfonylureas	6	-0.459	4	0.67	-0.418	0.838	2.33 (0.71 to 7.6)	NA	NA	NA	NA	NA

Outcome	Comparisons	k	T-statistic	df	P-value	Bias estimate	tau	Odds ratio	Trim and fill odds ratio (L estimator)	Trim and fill odds ratio (R estimator)	Mean difference	Trim and fill mean difference (L estimator)	Trim and fill mean difference (R estimator)
**Non-fatal stroke**	Sulfonylureas *vs* Standard treatments	3	NA	NA	NA	NA	NA	0.86 (0.19 to 3.81)	NA	NA	NA	NA	NA
**Non-fatal stroke**	Sulfonylureas *vs* Thiazolidinediones	9	0.627	7	0.551	0.36	1.058	1.1 (0.74 to 1.63)	NA	NA	NA	NA	NA
**Non-fatal stroke**	Thiazolidinediones *vs* Standard treatments	10	-2.749	8	0.025	-0.553	0.472	0.81 (0.65 to 1.02)	1.26 (1.01 to 1.56)	1.25 (1.01 to 1.56)	NA	NA	NA
**Hospitalisation for heart failure**	Alpha-glucosidase inhibitors *vs* Standard treatments	1	NA	NA	NA	NA	NA	3.25 (0.13 to 82.49)	NA	NA	NA	NA	NA
**Hospitalisation for heart failure**	Basal insulin *vs* DPP-4 inhibitors	1	NA	NA	NA	NA	NA	0.87 (0.51 to 1.48)	NA	NA	NA	NA	NA
**Hospitalisation for heart failure**	Basal insulin *vs* GLP-1 receptor agonists	5	-0.662	3	0.555	-0.51	1.053	1.6 (0.91 to 2.84)	NA	NA	NA	NA	NA
**Hospitalisation for heart failure**	Basal insulin *vs* Standard treatments	2	NA	NA	NA	NA	NA	0.33 (0.03 to 3.16)	NA	NA	NA	NA	NA

Outcome	Comparisons	k	T-statistic	df	P-value	Bias estimate	tau	Odds ratio	Trim and fill odds ratio (L estimator)	Trim and fill odds ratio (R estimator)	Mean difference	Trim and fill mean difference (L estimator)	Trim and fill mean difference (R estimator)
**Hospitalisation for heart failure**	Basal insulin *vs* Sulfonylureas	1	NA	NA	NA	NA	NA	0.86 (0.5 to 1.46)	NA	NA	NA	NA	NA
**Hospitalisation for heart failure**	Basal insulin *vs* Thiazolidinediones	1	NA	NA	NA	NA	NA	0.34 (0.01 to 8.53)	NA	NA	NA	NA	NA
**Hospitalisation for heart failure**	Basal insulin *vs* Tirzepatide	1	NA	NA	NA	NA	NA	1.5 (0.42 to 5.32)	NA	NA	NA	NA	NA
**Hospitalisation for heart failure**	Bolus insulin *vs* GLP-1 receptor agonists	2	NA	NA	NA	NA	NA	0.71 (0.07 to 6.85)	NA	NA	NA	NA	NA
**Hospitalisation for heart failure**	DPP-4 inhibitors *vs* GLP-1 receptor agonists	7	-0.622	5	0.561	-0.386	0.97	1.71 (1.04 to 2.82)	NA	NA	NA	NA	NA
**Hospitalisation for**	DPP-4 inhibitors *vs* Metformin	0	NA	NA	NA	NA	NA	1.51 (0.03 to 76.36)	NA	NA	NA	NA	NA

Outcome	Comparisons	k	T-statistic	df	P-value	Bias estimate	tau	Odds ratio	Trim and fill odds ratio (L estimator)	Trim and fill odds ratio (R estimator)	Mean difference	Trim and fill mean difference (L estimator)	Trim and fill mean difference (R estimator)
**heart failure**													
**Hospitalisation for heart failure**	DPP-4 inhibitors *vs* SGLT2 inhibitors	1	NA	NA	NA	NA	NA	0.4 (0.02 to 9.88)	NA	NA	NA	NA	NA
**Hospitalisation for heart failure**	DPP-4 inhibitors *vs* Standard treatments	25	-0.631	23	0.534	-0.16	1.066	0.98 (0.8 to 1.19)	1.01 (0.84 to 1.2)	1.01 (0.84 to 1.2)	NA	NA	NA
**Hospitalisation for heart failure**	DPP-4 inhibitors *vs* Sulfonylureas	13	-1.056	11	0.313	-0.404	1.03	1.12 (0.89 to 1.42)	1.13 (0.91 to 1.42)	1.13 (0.91 to 1.42)	NA	NA	NA
**Hospitalisation for heart failure**	DPP-4 inhibitors *vs* Thiazolidinediones	1	NA	NA	NA	NA	NA	0.33 (0.01 to 8.19)	NA	NA	NA	NA	NA
**Hospitalisation for heart failure**	GLP-1 receptor agonists *vs* Metformin	1	NA	NA	NA	NA	NA	0.36 (0.05 to 2.81)	NA	NA	NA	NA	NA

Outcome	Comparisons	k	T-statistic	df	P-value	Bias estimate	tau	Odds ratio	Trim and fill odds ratio (L estimator)	Trim and fill odds ratio (R estimator)	Mean difference	Trim and fill mean difference (L estimator)	Trim and fill mean difference (R estimator)
**Hospitalisation for heart failure**	GLP-1 receptor agonists *vs* SGLT2 inhibitors	1	NA	NA	NA	NA	NA	0.5 (0.05 to 5.54)	NA	NA	NA	NA	NA
**Hospitalisation for heart failure**	GLP-1 receptor agonists *vs* Standard treatments	22	-0.123	20	0.903	-0.03	0.893	0.92 (0.84 to 1.01)	0.93 (0.85 to 1.02)	0.93 (0.85 to 1.01)	NA	NA	NA
**Hospitalisation for heart failure**	GLP-1 receptor agonists *vs* Sulfonylureas	4	NA	NA	NA	NA	NA	0.73 (0.28 to 1.9)	NA	NA	NA	NA	NA
**Hospitalisation for heart failure**	GLP-1 receptor agonists *vs* Thiazolidinediones	1	NA	NA	NA	NA	NA	0.22 (0.01 to 5.38)	NA	NA	NA	NA	NA
**Hospitalisation for heart failure**	Metformin *vs* SGLT2 inhibitors	1	NA	NA	NA	NA	NA	1.43 (0.06 to 35.52)	NA	NA	NA	NA	NA
**Hospitalisation for**	Metformin *vs* Thiazolidinediones	1	NA	NA	NA	NA	NA	0.22 (0.01 to 5.43)	NA	NA	NA	NA	NA

Outcome	Comparisons	k	T-statistic	df	P-value	Bias estimate	tau	Odds ratio	Trim and fill odds ratio (L estimator)	Trim and fill odds ratio (R estimator)	Mean difference	Trim and fill mean difference (L estimator)	Trim and fill mean difference (R estimator)
**heart failure**													
**Hospitalisation for heart failure**	Non-steroidal MRAs *vs* Standard treatments	2	NA	NA	NA	NA	NA	0.78 (0.64 to 0.94)	NA	NA	NA	NA	NA
**Hospitalisation for heart failure**	SGLT2 inhibitors *vs* Standard treatments	26	0.557	24	0.583	0.115	0.862	0.66 (0.6 to 0.72)	0.68 (0.62 to 0.74)	0.68 (0.63 to 0.74)	NA	NA	NA
**Hospitalisation for heart failure**	SGLT2 inhibitors *vs* Sulfonylureas	5	-3.624	3	0.036	-3.486	0.507	0.85 (0.23 to 3.17)	NA	NA	NA	NA	NA
**Hospitalisation for heart failure**	Sulfonylureas *vs* Standard treatments	1	NA	NA	NA	NA	NA	0.76 (0.06 to 9.06)	NA	NA	NA	NA	NA
**Hospitalisation for heart failure**	Sulfonylureas *vs* Thiazolidinediones	9	-4.062	7	0.005	-1.252	0.523	0.76 (0.42 to 1.39)	NA	NA	NA	NA	NA

Outcome	Comparisons	k	T-statistic	df	P-value	Bias estimate	tau	Odds ratio	Trim and fill odds ratio (L estimator)	Trim and fill odds ratio (R estimator)	Mean difference	Trim and fill mean difference (L estimator)	Trim and fill mean difference (R estimator)
**Hospitalisation for heart failure**	Thiazolidinediones *vs* Standard treatments	20	-0.952	18	0.354	-0.249	0.926	1.59 (1.25 to 2.01)	0.66 (0.53 to 0.82)	0.64 (0.5 to 0.81)	NA	NA	NA
**End-stage kidney disease**	Basal insulin *vs* GLP-1 receptor agonists	2	NA	NA	NA	NA	NA	1.42 (0.74 to 2.74)	NA	NA	NA	NA	NA
**End-stage kidney disease**	Basal insulin *vs* Tirzepatide	1	NA	NA	NA	NA	NA	1.99 (0.18 to 22)	NA	NA	NA	NA	NA
**End-stage kidney disease**	Bolus insulin *vs* GLP-1 receptor agonists	1	NA	NA	NA	NA	NA	3.05 (0.12 to 75.27)	NA	NA	NA	NA	NA
**End-stage kidney disease**	DPP-4 inhibitors *vs* GLP-1 receptor agonists	3	NA	NA	NA	NA	NA	1.36 (0.41 to 4.47)	NA	NA	NA	NA	NA
**End-stage kidney disease**	DPP-4 inhibitors *vs* Standard treatments	9	-1.738	7	0.126	-0.649	0.834	1.04 (0.93 to 1.16)	NA	NA	NA	NA	NA
**End-stage kidney disease**	DPP-4 inhibitors *vs* Sulfonylureas	3	NA	NA	NA	NA	NA	1.47 (0.76 to 2.83)	NA	NA	NA	NA	NA

Outcome	Comparisons	k	T-statistic	df	P-value	Bias estimate	tau	Odds ratio	Trim and fill odds ratio (L estimator)	Trim and fill odds ratio (R estimator)	Mean difference	Trim and fill mean difference (L estimator)	Trim and fill mean difference (R estimator)
**End-stage kidney disease**	GLP-1 receptor agonists *vs* Standard treatments	11	1.812	9	0.103	0.641	0.782	0.84 (0.74 to 0.95)	0.84 (0.75 to 0.94)	0.84 (0.75 to 0.95)	NA	NA	NA
**End-stage kidney disease**	GLP-1 receptor agonists *vs* Tirzepatide	1	NA	NA	NA	NA	NA	1 (0.04 to 24.59)	NA	NA	NA	NA	NA
**End-stage kidney disease**	Metformin *vs* Standard treatments	1	NA	NA	NA	NA	NA	1.61 (0.36 to 7.24)	NA	NA	NA	NA	NA
**End-stage kidney disease**	Non-steroidal MRAs *vs* Standard treatments	2	NA	NA	NA	NA	NA	0.83 (0.75 to 0.92)	NA	NA	NA	NA	NA
**End-stage kidney disease**	SGLT2 inhibitors *vs* Standard treatments	15	1.087	13	0.297	0.603	1.433	0.63 (0.53 to 0.75)	0.64 (0.54 to 0.76)	0.64 (0.54 to 0.77)	NA	NA	NA
**End-stage kidney disease**	SGLT2 inhibitors *vs* Sulfonylureas	1	NA	NA	NA	NA	NA	3.06 (0.12 to 75.3)	NA	NA	NA	NA	NA
**End-stage kidney disease**	Sulfonylureas *vs* Thiazolidinediones	3	NA	NA	NA	NA	NA	0.99 (0.82 to 1.19)	NA	NA	NA	NA	NA

Outcome	Comparisons	k	T-statistic	df	P-value	Bias estimate	tau	Odds ratio	Trim and fill odds ratio (L estimator)	Trim and fill odds ratio (R estimator)	Mean difference	Trim and fill mean difference (L estimator)	Trim and fill mean difference (R estimator)
**End-stage kidney disease**	Thiazolidinediones *vs* Standard treatments	1	NA	NA	NA	NA	NA	0.75 (0.17 to 3.36)	NA	NA	NA	NA	NA
**Severe hypoglycaemia**	Alpha-glucosidase inhibitors *vs* Meglitinides	0	NA	NA	NA	NA	NA	1.01 (0.02 to 51.04)	NA	NA	NA	NA	NA
**Severe hypoglycaemia**	Alpha-glucosidase inhibitors *vs* SGLT2 inhibitors	1	NA	NA	NA	NA	NA	3.07 (0.12 to 76.55)	NA	NA	NA	NA	NA
**Severe hypoglycaemia**	Alpha-glucosidase inhibitors *vs* Standard treatments	2	NA	NA	NA	NA	NA	0.74 (0.1 to 5.57)	NA	NA	NA	NA	NA
**Severe hypoglycaemia**	Alpha-glucosidase inhibitors *vs* Sulfonylureas	1	NA	NA	NA	NA	NA	0.67 (0.03 to 16.5)	NA	NA	NA	NA	NA
**Severe hypoglycaemia**	Basal insulin *vs* Basal-Bolus insulin	1	NA	NA	NA	NA	NA	0.66 (0.11 to 3.99)	NA	NA	NA	NA	NA
**Severe hypoglycaemia**	Basal insulin *vs* DPP-4 inhibitors	3	NA	NA	NA	NA	NA	1.99 (0.92 to 4.29)	NA	NA	NA	NA	NA

Outcome	Comparisons	k	T-statistic	df	P-value	Bias estimate	tau	Odds ratio	Trim and fill odds ratio (L estimator)	Trim and fill odds ratio (R estimator)	Mean difference	Trim and fill mean difference (L estimator)	Trim and fill mean difference (R estimator)
**Severe hypoglycaemia**	Basal insulin *vs* GLP-1 receptor agonists	15	-1.693	13	0.114	-1.25	1.709	2.18 (1.17 to 4.09)	2.32 (1.29 to 4.2)	2.11 (1.19 to 3.75)	NA	NA	NA
**Severe hypoglycaemia**	Basal insulin *vs* SGLT2 inhibitors	1	NA	NA	NA	NA	NA	3.31 (0.13 to 81.76)	NA	NA	NA	NA	NA
**Severe hypoglycaemia**	Basal insulin *vs* Standard treatments	0	NA	NA	NA	NA	NA	0.49 (0.01 to 24.98)	NA	NA	NA	NA	NA
**Severe hypoglycaemia**	Basal insulin *vs* Sulfonylureas	1	NA	NA	NA	NA	NA	0.56 (0.3 to 1.04)	NA	NA	NA	NA	NA
**Severe hypoglycaemia**	Basal insulin *vs* Thiazolidinediones	2	NA	NA	NA	NA	NA	1.78 (0.13 to 24.43)	NA	NA	NA	NA	NA
**Severe hypoglycaemia**	Basal insulin *vs* Tirzepatide	2	NA	NA	NA	NA	NA	2.45 (0.83 to 7.23)	NA	NA	NA	NA	NA
**Severe hypoglycaemia**	Basal-Bolus insulin *vs* GLP-1 receptor agonists	1	NA	NA	NA	NA	NA	10.96 (0.64 to 188.62)	NA	NA	NA	NA	NA

Outcome	Comparisons	k	T-statistic	df	P-value	Bias estimate	tau	Odds ratio	Trim and fill odds ratio (L estimator)	Trim and fill odds ratio (R estimator)	Mean difference	Trim and fill mean difference (L estimator)	Trim and fill mean difference (R estimator)
**Severe hypoglycaemia**	Bolus insulin *vs* GLP-1 receptor agonists	7	2.287	5	0.071	0.646	0.425	2.52 (1.35 to 4.72)	NA	NA	NA	NA	NA
**Severe hypoglycaemia**	DPP-4 inhibitors *vs* GLP-1 receptor agonists	7	0.405	5	0.702	0.253	1.221	1.34 (0.91 to 1.99)	NA	NA	NA	NA	NA
**Severe hypoglycaemia**	DPP-4 inhibitors *vs* Metformin	2	NA	NA	NA	NA	NA	1.02 (0.05 to 22.76)	NA	NA	NA	NA	NA
**Severe hypoglycaemia**	DPP-4 inhibitors *vs* SGLT2 inhibitors	3	NA	NA	NA	NA	NA	0.64 (0.26 to 1.58)	NA	NA	NA	NA	NA
**Severe hypoglycaemia**	DPP-4 inhibitors *vs* Standard treatments	41	-1.122	39	0.269	-0.235	1.032	1.1 (0.99 to 1.24)	1.12 (1 to 1.24)	1.1 (0.99 to 1.23)	NA	NA	NA
**Severe hypoglycaemia**	DPP-4 inhibitors *vs* Sulfonylureas	16	-0.761	14	0.459	-0.246	0.68	0.19 (0.13 to 0.27)	0.2 (0.14 to 0.29)	0.19 (0.13 to 0.28)	NA	NA	NA
**Severe hypoglycaemia**	DPP-4 inhibitors *vs* Thiazolidinediones	3	NA	NA	NA	NA	NA	2.99 (0.54 to 16.61)	NA	NA	NA	NA	NA

Outcome	Comparisons	k	T-statistic	df	P-value	Bias estimate	tau	Odds ratio	Trim and fill odds ratio (L estimator)	Trim and fill odds ratio (R estimator)	Mean difference	Trim and fill mean difference (L estimator)	Trim and fill mean difference (R estimator)
**Severe hypoglycaemia**	GLP-1 receptor agonists *vs* SGLT2 inhibitors	2	NA	NA	NA	NA	NA	1.17 (0.54 to 2.55)	NA	NA	NA	NA	NA
**Severe hypoglycaemia**	GLP-1 receptor agonists *vs* Standard treatments	38	1.364	36	0.181	0.35	1.252	0.99 (0.83 to 1.17)	0.97 (0.82 to 1.13)	0.99 (0.84 to 1.16)	NA	NA	NA
**Severe hypoglycaemia**	GLP-1 receptor agonists *vs* Sulfonylureas	3	NA	NA	NA	NA	NA	0.5 (0.23 to 1.1)	NA	NA	NA	NA	NA
**Severe hypoglycaemia**	GLP-1 receptor agonists *vs* Thiazolidinediones	2	NA	NA	NA	NA	NA	0.54 (0.1 to 2.82)	NA	NA	NA	NA	NA
**Severe hypoglycaemia**	GLP-1 receptor agonists *vs* Tirzepatide	1	NA	NA	NA	NA	NA	0.6 (0.03 to 12.51)	NA	NA	NA	NA	NA
**Severe hypoglycaemia**	Meglitinides *vs* Metformin	1	NA	NA	NA	NA	NA	2.34 (0.66 to 8.34)	NA	NA	NA	NA	NA
**Severe hypoglycaemia**	Meglitinides *vs* Sulfonylureas	2	NA	NA	NA	NA	NA	0.34 (0.04 to 3.11)	NA	NA	NA	NA	NA

Outcome	Comparisons	k	T-statistic	df	P-value	Bias estimate	tau	Odds ratio	Trim and fill odds ratio (L estimator)	Trim and fill odds ratio (R estimator)	Mean difference	Trim and fill mean difference (L estimator)	Trim and fill mean difference (R estimator)
**Severe hypoglycaemia**	Metformin *vs* SGLT2 inhibitors	2	NA	NA	NA	NA	NA	3.66 (0.87 to 15.44)	NA	NA	NA	NA	NA
**Severe hypoglycaemia**	Metformin *vs* Standard treatments	2	NA	NA	NA	NA	NA	2.79 (0.27 to 28.45)	NA	NA	NA	NA	NA
**Severe hypoglycaemia**	Metformin *vs* Sulfonylureas	2	NA	NA	NA	NA	NA	0.46 (0.02 to 9.72)	NA	NA	NA	NA	NA
**Severe hypoglycaemia**	Metformin *vs* Thiazolidinediones	1	NA	NA	NA	NA	NA	1 (0.06 to 16.02)	NA	NA	NA	NA	NA
**Severe hypoglycaemia**	Non-steroidal MRAs *vs* Standard treatments	2	NA	NA	NA	NA	NA	0.64 (0.43 to 0.96)	NA	NA	NA	NA	NA
**Severe hypoglycaemia**	SGLT2 inhibitors *vs* Standard treatments	28	2.174	26	0.039	0.531	0.993	0.89 (0.78 to 1.02)	0.86 (0.76 to 0.99)	0.9 (0.79 to 1.02)	NA	NA	NA
**Severe hypoglycaemia**	SGLT2 inhibitors *vs* Sulfonylureas	3	NA	NA	NA	NA	NA	0.17 (0.07 to 0.43)	NA	NA	NA	NA	NA

Outcome	Comparisons	k	T-statistic	df	P-value	Bias estimate	tau	Odds ratio	Trim and fill odds ratio (L estimator)	Trim and fill odds ratio (R estimator)	Mean difference	Trim and fill mean difference (L estimator)	Trim and fill mean difference (R estimator)
**Severe hypoglycaemia**	Sulfonylureas *vs* Standard treatments	1	NA	NA	NA	NA	NA	1.42 (0.12 to 17.07)	NA	NA	NA	NA	NA
**Severe hypoglycaemia**	Sulfonylureas *vs* Thiazolidinediones	6	0.567	4	0.601	0.102	0.232	8.79 (3.06 to 25.28)	NA	NA	NA	NA	NA
**Severe hypoglycaemia**	Thiazolidinediones *vs* Standard treatments	9	-0.922	7	0.387	-0.462	0.865	1.77 (1.1 to 2.85)	0.58 (0.37 to 0.92)	0.56 (0.35 to 0.9)	NA	NA	NA
**Severe hypoglycaemia**	Tirzepatide *vs* Standard treatments	1	NA	NA	NA	NA	NA	2.39 (0.12 to 46.66)	NA	NA	NA	NA	NA
**Severe gastrointestinal events**	Alpha-glucosidase inhibitors *vs* Metformin	1	NA	NA	NA	NA	NA	1.53 (0.25 to 9.54)	NA	NA	NA	NA	NA
**Severe gastrointestinal events**	Basal insulin *vs* DPP-4 inhibitors	1	NA	NA	NA	NA	NA	1.11 (0.07 to 17.92)	NA	NA	NA	NA	NA
**Severe gastrointestinal events**	Basal insulin *vs* GLP-1 receptor agonists	1	NA	NA	NA	NA	NA	0.3 (0.07 to 1.36)	NA	NA	NA	NA	NA

Outcome	Comparisons	k	T-statistic	df	P-value	Bias estimate	tau	Odds ratio	Trim and fill odds ratio (L estimator)	Trim and fill odds ratio (R estimator)	Mean difference	Trim and fill mean difference (L estimator)	Trim and fill mean difference (R estimator)
**Severe gastrointestinal events**	Basal insulin *vs* Tirzepatide	2	NA	NA	NA	NA	NA	0.04 (0.01 to 0.19)	NA	NA	NA	NA	NA
**Severe gastrointestinal events**	Bolus insulin *vs* GLP-1 receptor agonists	1	NA	NA	NA	NA	NA	1.67 (0.4 to 7.01)	NA	NA	NA	NA	NA
**Severe gastrointestinal events**	DPP-4 inhibitors *vs* GLP-1 receptor agonists	2	NA	NA	NA	NA	NA	0.63 (0.16 to 2.51)	NA	NA	NA	NA	NA
**Severe gastrointestinal events**	DPP-4 inhibitors *vs* Standard treatments	5	0.089	3	0.934	0.22	0.908	1.75 (0.49 to 6.27)	NA	NA	NA	NA	NA
**Severe gastrointestinal events**	DPP-4 inhibitors *vs* Sulfonylureas	1	NA	NA	NA	NA	NA	0.72 (0.16 to 3.24)	NA	NA	NA	NA	NA
**Severe gastrointestinal events**	GLP-1 receptor agonists *vs* Standard treatments	14	1.727	12	0.11	1.757	2.01	2.02 (1.32 to 3.1)	1.24 (0.75 to 2.04)	1.14 (0.7 to 1.84)	NA	NA	NA
**Severe gastrointestinal events**	GLP-1 receptor agonists *vs* Tirzepatide	2	NA	NA	NA	NA	NA	0.71 (0.39 to 1.3)	NA	NA	NA	NA	NA

Outcome	Comparisons	k	T-statistic	df	P-value	Bias estimate	tau	Odds ratio	Trim and fill odds ratio (L estimator)	Trim and fill odds ratio (R estimator)	Mean difference	Trim and fill mean difference (L estimator)	Trim and fill mean difference (R estimator)
**Severe gastrointestinal events**	Metformin *vs* Standard treatments	1	NA	NA	NA	NA	NA	1.99 (0.18 to 22.14)	NA	NA	NA	NA	NA
**Severe gastrointestinal events**	Metformin *vs* Sulfonylureas	1	NA	NA	NA	NA	NA	2.32 (0.6 to 8.98)	NA	NA	NA	NA	NA
**Severe gastrointestinal events**	Metformin *vs* Thiazolidinediones	2	NA	NA	NA	NA	NA	2.5 (0.18 to 35.32)	NA	NA	NA	NA	NA
**Severe gastrointestinal events**	Sulfonylureas *vs* Thiazolidinediones	1	NA	NA	NA	NA	NA	0.38 (0.1 to 1.43)	NA	NA	NA	NA	NA
**Severe gastrointestinal events**	Thiazolidinediones *vs* Standard treatments	2	NA	NA	NA	NA	NA	1.16 (0.91 to 1.48)	NA	NA	NA	NA	NA
**Severe gastrointestinal events**	Tirzepatide *vs* Standard treatments	3	NA	NA	NA	NA	NA	5.76 (1.33 to 24.97)	NA	NA	NA	NA	NA
**Genital infection**	DPP-4 inhibitors *vs* SGLT2 inhibitors	10	-2.429	8	0.041	-1.185	0.772	0.26 (0.16 to 0.42)	0.32 (0.2 to 0.49)	0.3 (0.19 to 0.47)	NA	NA	NA
**Genital infection**	DPP-4 inhibitors *vs* Standard treatments	6	0.017	4	0.987	0.021	1.293	0.47 (0.2 to 1.1)	NA	NA	NA	NA	NA

Outcome	Comparisons	k	T-statistic	df	P-value	Bias estimate	tau	Odds ratio	Trim and fill odds ratio (L estimator)	Trim and fill odds ratio (R estimator)	Mean difference	Trim and fill mean difference (L estimator)	Trim and fill mean difference (R estimator)
**Genital infection**	GLP-1 receptor agonists *vs* SGLT2 inhibitors	2	NA	NA	NA	NA	NA	0.18 (0.08 to 0.39)	NA	NA	NA	NA	NA
**Genital infection**	GLP-1 receptor agonists *vs* Standard treatments	4	NA	NA	NA	NA	NA	1.14 (0.22 to 5.83)	NA	NA	NA	NA	NA
**Genital infection**	Metformin *vs* SGLT2 inhibitors	4	NA	NA	NA	NA	NA	0.39 (0.21 to 0.73)	NA	NA	NA	NA	NA
**Genital infection**	SGLT2 inhibitors *vs* Standard treatments	63	0.414	61	0.681	0.08	1.016	3.31 (2.84 to 3.85)	3.16 (2.71 to 3.68)	3.16 (2.71 to 3.68)	NA	NA	NA
**Genital infection**	SGLT2 inhibitors *vs* Sulfonylureas	6	0.949	4	0.396	0.818	0.883	6.32 (4.53 to 8.82)	NA	NA	NA	NA	NA
**Genital infection**	SGLT2 inhibitors *vs* Thiazolidinediones	1	NA	NA	NA	NA	NA	0.3 (0.01 to 7.75)	NA	NA	NA	NA	NA
**Amputation**	DPP-4 inhibitors *vs* SGLT2 inhibitors	1	NA	NA	NA	NA	NA	0.66 (0.03 to 16.38)	NA	NA	NA	NA	NA
**Amputation**	DPP-4 inhibitors *vs* Standard treatments	1	NA	NA	NA	NA	NA	0.91 (0.64 to 1.29)	NA	NA	NA	NA	NA
**Amputation**	GLP-1 receptor agonists *vs* Standard treatments	2	NA	NA	NA	NA	NA	0.71 (0.07 to 6.79)	NA	NA	NA	NA	NA
**Amputation**	Non-steroidal MRAs *vs* Standard treatments	2	NA	NA	NA	NA	NA	0.94 (0.46 to 1.9)	NA	NA	NA	NA	NA
**Amputation**	SGLT2 inhibitors *vs* Standard treatments	10	1.059	8	0.32	0.848	1.452	1.28 (1 to 1.64)	1.24 (0.97 to 1.57)	1.23 (0.97 to 1.56)	NA	NA	NA

Outcome	Comparisons	k	T-statistic	df	P-value	Bias estimate	tau	Odds ratio	Trim and fill odds ratio (L estimator)	Trim and fill odds ratio (R estimator)	Mean difference	Trim and fill mean difference (L estimator)	Trim and fill mean difference (R estimator)
**Amputation**	Thiazolidinediones *vs* Standard treatments	2	NA	NA	NA	NA	NA	0.63 (0.22 to 1.85)	NA	NA	NA	NA	NA
**Ketoacidosis due to diabetes**	Alpha-glucosidase inhibitors *vs* Meglitinides	0	NA	NA	NA	NA	NA	1.01 (0.02 to 51.04)	NA	NA	NA	NA	NA
**Ketoacidosis due to diabetes**	Alpha-glucosidase inhibitors *vs* Sulfonylureas	1	NA	NA	NA	NA	NA	0.67 (0.03 to 16.5)	NA	NA	NA	NA	NA
**Ketoacidosis due to diabetes**	DPP-4 inhibitors *vs* GLP-1 receptor agonists	1	NA	NA	NA	NA	NA	0.33 (0.01 to 8.15)	NA	NA	NA	NA	NA
**Ketoacidosis due to diabetes**	DPP-4 inhibitors *vs* SGLT2 inhibitors	2	NA	NA	NA	NA	NA	1.41 (0.08 to 24.61)	NA	NA	NA	NA	NA
**Ketoacidosis due to diabetes**	DPP-4 inhibitors *vs* Standard treatments	7	-0.641	5	0.55	-0.452	0.903	0.71 (0.34 to 1.49)	NA	NA	NA	NA	NA
**Ketoacidosis due to diabetes**	DPP-4 inhibitors *vs* Sulfonylureas	2	NA	NA	NA	NA	NA	2.13 (0.22 to 20.51)	NA	NA	NA	NA	NA
**Ketoacidosis due to diabetes**	GLP-1 receptor agonists *vs* Standard treatments	7	0.192	5	0.856	0.175	1.164	1.02 (0.55 to 1.88)	NA	NA	NA	NA	NA

Outcome	Comparisons	k	T-statistic	df	P-value	Bias estimate	tau	Odds ratio	Trim and fill odds ratio (L estimator)	Trim and fill odds ratio (R estimator)	Mean difference	Trim and fill mean difference (L estimator)	Trim and fill mean difference (R estimator)
**Ketoacidosis due to diabetes**	Meglitinides *vs* Sulfonylureas	1	NA	NA	NA	NA	NA	0.66 (0.03 to 16.32)	NA	NA	NA	NA	NA
**Ketoacidosis due to diabetes**	Metformin *vs* SGLT2 inhibitors	1	NA	NA	NA	NA	NA	0.67 (0.03 to 16.41)	NA	NA	NA	NA	NA
**Ketoacidosis due to diabetes**	Non-steroidal MRAs *vs* Standard treatments	2	NA	NA	NA	NA	NA	0.67 (0.26 to 1.73)	NA	NA	NA	NA	NA
**Ketoacidosis due to diabetes**	SGLT2 inhibitors *vs* Standard treatments	12	-0.36	10	0.727	-0.199	0.93	2.17 (1.5 to 3.13)	2.15 (1.49 to 3.11)	2.04 (1.42 to 2.93)	NA	NA	NA
**Ketoacidosis due to diabetes**	SGLT2 inhibitors *vs* Sulfonylureas	1	NA	NA	NA	NA	NA	1.49 (0.06 to 36.54)	NA	NA	NA	NA	NA
**Health-related quality of life**	Alpha-glucosidase inhibitors *vs* DPP-4 inhibitors	1	NA	NA	NA	NA	NA	NA	NA	NA	0 (-0.18 to 0.18)	NA	NA
**Health-related quality of life**	Basal insulin *vs* Bolus insulin	1	NA	NA	NA	NA	NA	NA	NA	NA	0.42 (0.21 to 0.62)	NA	NA

Outcome	Comparisons	k	T-statistic	df	P-value	Bias estimate	tau	Odds ratio	Trim and fill odds ratio (L estimator)	Trim and fill odds ratio (R estimator)	Mean difference	Trim and fill mean difference (L estimator)	Trim and fill mean difference (R estimator)
**Health-related quality of life**	Basal insulin *vs* GLP-1 receptor agonists	1	NA	NA	NA	NA	NA	NA	NA	NA	-0.2 (-0.47 to 0.07)	NA	NA
**Health-related quality of life**	Basal insulin *vs* Tirzepatide	1	NA	NA	NA	NA	NA	NA	NA	NA	-0.62 (-0.75 to -0.49)	NA	NA
**Health-related quality of life**	Bolus insulin *vs* GLP-1 receptor agonists	4	NA	NA	NA	NA	NA	NA	NA	NA	-0.21 (-0.29 to -0.12)	NA	NA
**Health-related quality of life**	DPP-4 inhibitors *vs* GLP-1 receptor agonists	5	1.569	3	0.215	5.069	1.231	NA	NA	NA	-0.16 (-0.28 to -0.03)	NA	NA
**Health-related quality of life**	DPP-4 inhibitors *vs* Metformin	2	NA	NA	NA	NA	NA	NA	NA	NA	0.01 (-0.19 to 0.21)	NA	NA
**Health-related**	DPP-4 inhibitors *vs* SGLT2 inhibitors	1	NA	NA	NA	NA	NA	NA	NA	NA	-0.26 (-0.53 to 0)	NA	NA

Outcome	Comparisons	k	T-statistic	df	P-value	Bias estimate	tau	Odds ratio	Trim and fill odds ratio (L estimator)	Trim and fill odds ratio (R estimator)	Mean difference	Trim and fill mean difference (L estimator)	Trim and fill mean difference (R estimator)
**quality of life**													
**Health-related quality of life**	DPP-4 inhibitors *vs* Standard treatments	2	NA	NA	NA	NA	NA	NA	NA	NA	0.08 (-0.07 to 0.24)	NA	NA
**Health-related quality of life**	DPP-4 inhibitors *vs* Thiazolidinediones	1	NA	NA	NA	NA	NA	NA	NA	NA	-0.15 (-0.38 to 0.09)	NA	NA
**Health-related quality of life**	GLP-1 receptor agonists *vs* Metformin	1	NA	NA	NA	NA	NA	NA	NA	NA	0.11 (-0.07 to 0.3)	NA	NA
**Health-related quality of life**	GLP-1 receptor agonists *vs* SGLT2 inhibitors	2	NA	NA	NA	NA	NA	NA	NA	NA	-0.27 (-0.89 to 0.35)	NA	NA
**Health-related quality of life**	GLP-1 receptor agonists *vs* Standard treatments	11	0.389	9	0.706	0.308	1.275	NA	NA	NA	0.15 (0.1 to 0.2)	0.15 (0.1 to 0.2)	0.15 (0.1 to 0.2)

Outcome	Comparisons	k	T-statistic	df	P-value	Bias estimate	tau	Odds ratio	Trim and fill odds ratio (L estimator)	Trim and fill odds ratio (R estimator)	Mean difference	Trim and fill mean difference (L estimator)	Trim and fill mean difference (R estimator)
**Health-related quality of life**	GLP-1 receptor agonists *vs* Thiazolidinediones	1	NA	NA	NA	NA	NA	NA	NA	NA	-0.05 (-0.26 to 0.16)	NA	NA
**Health-related quality of life**	GLP-1 receptor agonists *vs* Tirzepatide	1	NA	NA	NA	NA	NA	NA	NA	NA	0 (-0.11 to 0.11)	NA	NA
**Health-related quality of life**	Meglitinides *vs* Thiazolidinediones	1	NA	NA	NA	NA	NA	NA	NA	NA	-0.03 (-0.21 to 0.15)	NA	NA
**Health-related quality of life**	Metformin *vs* Thiazolidinediones	1	NA	NA	NA	NA	NA	NA	NA	NA	-0.16 (-0.37 to 0.05)	NA	NA
**Health-related quality of life**	SGLT2 inhibitors *vs* Standard treatments	2	NA	NA	NA	NA	NA	NA	NA	NA	0.23 (-0.63 to 1.09)	NA	NA
**Health-related**	Sulfonylureas *vs* Standard treatments	1	NA	NA	NA	NA	NA	NA	NA	NA	0.23 (-0.08 to 0.54)	NA	NA

Outcome	Comparisons	k	T-statistic	df	P-value	Bias estimate	tau	Odds ratio	Trim and fill odds ratio (L estimator)	Trim and fill odds ratio (R estimator)	Mean difference	Trim and fill mean difference (L estimator)	Trim and fill mean difference (R estimator)
**quality of life**													
**Body weight change**	Albiglutide *vs* Basal insulin	1	NA	NA	NA	NA	NA	NA	NA	NA	-2.61 (-3.2 to -2.02)	NA	NA
**Body weight change**	Albiglutide *vs* Bolus insulin	2	NA	NA	NA	NA	NA	NA	NA	NA	-2.97 (-5.77 to -0.17)	NA	NA
**Body weight change**	Albiglutide *vs* DPP-4 inhibitors	2	NA	NA	NA	NA	NA	NA	NA	NA	-0.5 (-0.92 to -0.09)	NA	NA
**Body weight change**	Albiglutide *vs* Standard treatments	5	2.311	3	0.104	2.322	1.382	NA	NA	NA	-0.2 (-0.81 to 0.41)	NA	NA
**Body weight change**	Albiglutide *vs* Sulfonylureas	1	NA	NA	NA	NA	NA	NA	NA	NA	-2.38 (-3.04 to -1.72)	NA	NA
**Body weight change**	Albiglutide *vs* Thiazolidinediones	1	NA	NA	NA	NA	NA	NA	NA	NA	-4.01 (-4.66 to -3.36)	NA	NA
**Body weight change**	Alpha-glucosidase inhibitors *vs* DPP-4 inhibitors	6	1.097	4	0.334	2.473	1.978	NA	NA	NA	-0.73 (-1.51 to 0.04)	NA	NA

Outcome	Comparisons	k	T-statistic	df	P-value	Bias estimate	tau	Odds ratio	Trim and fill odds ratio (L estimator)	Trim and fill odds ratio (R estimator)	Mean difference	Trim and fill mean difference (L estimator)	Trim and fill mean difference (R estimator)
**Body weight change**	Alpha-glucosidase inhibitors *vs* Metformin	3	NA	NA	NA	NA	NA	NA	NA	NA	0.57 (-0.69 to 1.84)	NA	NA
**Body weight change**	Alpha-glucosidase inhibitors *vs* SGLT2 inhibitors	1	NA	NA	NA	NA	NA	NA	NA	NA	0.27 (-1.94 to 2.48)	NA	NA
**Body weight change**	Alpha-glucosidase inhibitors *vs* Standard treatments	12	0.371	10	0.719	0.49	1.298	NA	NA	NA	-0.55 (-0.9 to -0.2)	-0.71 (-1.07 to -0.35)	-0.55 (-0.9 to -0.2)
**Body weight change**	Alpha-glucosidase inhibitors *vs* Sulfonylureas	2	NA	NA	NA	NA	NA	NA	NA	NA	-0.91 (-2.06 to 0.25)	NA	NA
**Body weight change**	Alpha-glucosidase inhibitors *vs* Thiazolidinediones	2	NA	NA	NA	NA	NA	NA	NA	NA	-2.62 (-4.01 to -1.22)	NA	NA
**Body weight change**	Basal insulin *vs* Basal-Bolus insulin	2	NA	NA	NA	NA	NA	NA	NA	NA	-0.87 (-2.47 to 0.72)	NA	NA
**Body weight change**	Basal insulin *vs* DPP-4 inhibitors	4	NA	NA	NA	NA	NA	NA	NA	NA	1.73 (0.84 to 2.63)	NA	NA

Outcome	Comparisons	k	T-statistic	df	P-value	Bias estimate	tau	Odds ratio	Trim and fill odds ratio (L estimator)	Trim and fill odds ratio (R estimator)	Mean difference	Trim and fill mean difference (L estimator)	Trim and fill mean difference (R estimator)
**Body weight change**	Basal insulin *vs* Dulaglutide	5	0.151	3	0.89	0.332	1.776	NA	NA	NA	3.06 (2.49 to 3.63)	NA	NA
**Body weight change**	Basal insulin *vs* Exenatide extended-release	4	NA	NA	NA	NA	NA	NA	NA	NA	3.21 (2.16 to 4.25)	NA	NA
**Body weight change**	Basal insulin *vs* Exenatide immediate-release	2	NA	NA	NA	NA	NA	NA	NA	NA	4.07 (3.54 to 4.59)	NA	NA
**Body weight change**	Basal insulin *vs* Liraglutide	8	0.294	6	0.779	0.665	3.336	NA	NA	NA	4.16 (3.42 to 4.9)	NA	NA
**Body weight change**	Basal insulin *vs* Semaglutide (Subcutaneous)	1	NA	NA	NA	NA	NA	NA	NA	NA	7.48 (6.68 to 8.28)	NA	NA
**Body weight change**	Basal insulin *vs* Standard treatments	5	-0.334	3	0.76	-1.396	2.834	NA	NA	NA	1.71 (0.43 to 2.98)	NA	NA
**Body weight change**	Basal insulin *vs* Sulfonylureas	2	NA	NA	NA	NA	NA	NA	NA	NA	0.5 (-0.82 to 1.83)	NA	NA

Outcome	Comparisons	k	T-statistic	df	P-value	Bias estimate	tau	Odds ratio	Trim and fill odds ratio (L estimator)	Trim and fill odds ratio (R estimator)	Mean difference	Trim and fill mean difference (L estimator)	Trim and fill mean difference (R estimator)
**Body weight change**	Basal insulin *vs* Thiazolidinediones	2	NA	NA	NA	NA	NA	NA	NA	NA	-1.3 (-2.28 to -0.32)	NA	NA
**Body weight change**	Basal insulin *vs* Tirzepatide	2	NA	NA	NA	NA	NA	NA	NA	NA	11.96 (10.67 to 13.24)	NA	NA
**Body weight change**	Basal-Bolus insulin *vs* Thiazolidinediones	1	NA	NA	NA	NA	NA	NA	NA	NA	1 (0.03 to 1.97)	NA	NA
**Body weight change**	Bolus insulin *vs* Liraglutide	2	NA	NA	NA	NA	NA	NA	NA	NA	2.28 (-0.03 to 4.6)	NA	NA
**Body weight change**	Bolus insulin *vs* Lixisenatide	1	NA	NA	NA	NA	NA	NA	NA	NA	1.8 (1.08 to 2.52)	NA	NA
**Body weight change**	Bolus insulin *vs* Semaglutide (Subcutaneous)	1	NA	NA	NA	NA	NA	NA	NA	NA	7.1 (6.67 to 7.53)	NA	NA
**Body weight change**	Bolus insulin *vs* SGLT2 inhibitors	1	NA	NA	NA	NA	NA	NA	NA	NA	0.9 (0.43 to 1.37)	NA	NA

Outcome	Comparisons	k	T-statistic	df	P-value	Bias estimate	tau	Odds ratio	Trim and fill odds ratio (L estimator)	Trim and fill odds ratio (R estimator)	Mean difference	Trim and fill mean difference (L estimator)	Trim and fill mean difference (R estimator)
**Body weight change**	DPP-4 inhibitors *vs* Dulaglutide	1	NA	NA	NA	NA	NA	NA	NA	NA	1.72 (1.22 to 2.22)	NA	NA
**Body weight change**	DPP-4 inhibitors *vs* Exenatide extended-release	3	NA	NA	NA	NA	NA	NA	NA	NA	0.87 (-0.06 to 1.81)	NA	NA
**Body weight change**	DPP-4 inhibitors *vs* Liraglutide	6	2.125	4	0.101	2.019	1.126	NA	NA	NA	1.93 (1.45 to 2.42)	NA	NA
**Body weight change**	DPP-4 inhibitors *vs* Lixisenatide	1	NA	NA	NA	NA	NA	NA	NA	NA	1.3 (0.48 to 2.12)	NA	NA
**Body weight change**	DPP-4 inhibitors *vs* Metformin	11	0.339	9	0.743	0.581	1.549	NA	NA	NA	1.18 (0.9 to 1.46)	1.18 (0.9 to 1.46)	1.09 (0.77 to 1.41)
**Body weight change**	DPP-4 inhibitors *vs* Semaglutide (Oral)	1	NA	NA	NA	NA	NA	NA	NA	NA	1.9 (1.19 to 2.61)	NA	NA
**Body weight change**	DPP-4 inhibitors *vs* Semaglutide (Subcutaneous)	2	NA	NA	NA	NA	NA	NA	NA	NA	4.09 (3.53 to 4.64)	NA	NA

Outcome	Comparisons	k	T-statistic	df	P-value	Bias estimate	tau	Odds ratio	Trim and fill odds ratio (L estimator)	Trim and fill odds ratio (R estimator)	Mean difference	Trim and fill mean difference (L estimator)	Trim and fill mean difference (R estimator)
**Body weight change**	DPP-4 inhibitors *vs* SGLT2 inhibitors	14	-2.227	12	0.046	-1.838	1.1	NA	NA	NA	2.34 (2.16 to 2.52)	2.45 (2.16 to 2.74)	2.43 (2.14 to 2.72)
**Body weight change**	DPP-4 inhibitors *vs* Standard treatments	78	1.539	76	0.128	0.848	1.793	NA	NA	NA	0.26 (0.13 to 0.39)	0.06 (-0.08 to 0.21)	0.26 (0.13 to 0.39)
**Body weight change**	DPP-4 inhibitors *vs* Sulfonylureas	19	-0.813	17	0.428	-2.203	4.541	NA	NA	NA	-1.47 (-1.97 to -0.96)	-0.98 (-1.47 to -0.48)	-1.47 (-1.97 to -0.96)
**Body weight change**	DPP-4 inhibitors *vs* Thiazolidinediones	11	-1.163	9	0.275	-3.826	4.258	NA	NA	NA	-1.86 (-2.8 to -0.92)	-1.12 (-2.09 to -0.16)	-1.86 (-2.8 to -0.92)
**Body weight change**	Dulaglutide *vs* Exenatide extended-release	1	NA	NA	NA	NA	NA	NA	NA	NA	1.66 (-0.12 to 3.44)	NA	NA
**Body weight change**	Dulaglutide *vs* Metformin	1	NA	NA	NA	NA	NA	NA	NA	NA	-0.07 (-0.73 to 0.59)	NA	NA
**Body weight change**	Dulaglutide *vs* Standard treatments	9	-0.461	7	0.659	-0.449	1.887	NA	NA	NA	-1.67 (-2.26 to -1.08)	NA	NA

Outcome	Comparisons	k	T-statistic	df	P-value	Bias estimate	tau	Odds ratio	Trim and fill odds ratio (L estimator)	Trim and fill odds ratio (R estimator)	Mean difference	Trim and fill mean difference (L estimator)	Trim and fill mean difference (R estimator)
**Body weight change**	Dulaglutide *vs* Sulfonylureas	1	NA	NA	NA	NA	NA	NA	NA	NA	-2.35 (-2.87 to -1.83)	NA	NA
**Body weight change**	Dulaglutide *vs* Tirzepatide	1	NA	NA	NA	NA	NA	NA	NA	NA	3.71 (1.91 to 5.52)	NA	NA
**Body weight change**	Efpeglenatide *vs* Standard treatments	1	NA	NA	NA	NA	NA	NA	NA	NA	-2.59 (-2.91 to -2.27)	NA	NA
**Body weight change**	Exenatide extended-release *vs* Metformin	1	NA	NA	NA	NA	NA	NA	NA	NA	0 (-0.56 to 0.56)	NA	NA
**Body weight change**	Exenatide extended-release *vs* SGLT2 inhibitors	1	NA	NA	NA	NA	NA	NA	NA	NA	2.22 (0.89 to 3.55)	NA	NA
**Body weight change**	Exenatide extended-release *vs* Standard treatments	4	NA	NA	NA	NA	NA	NA	NA	NA	-1.36 (-1.9 to -0.82)	NA	NA
**Body weight change**	Exenatide extended-release *vs* Thiazolidinediones	2	NA	NA	NA	NA	NA	NA	NA	NA	-4.28 (-5.85 to -2.71)	NA	NA

Outcome	Comparisons	k	T-statistic	df	P-value	Bias estimate	tau	Odds ratio	Trim and fill odds ratio (L estimator)	Trim and fill odds ratio (R estimator)	Mean difference	Trim and fill mean difference (L estimator)	Trim and fill mean difference (R estimator)
**Body weight change**	Exenatide immediate-release *vs* Metformin	1	NA	NA	NA	NA	NA	NA	NA	NA	-1.99 (-2.77 to -1.21)	NA	NA
**Body weight change**	Exenatide immediate-release *vs* Standard treatments	6	-1.393	4	0.236	-3.077	1.944	NA	NA	NA	-1.56 (-2.47 to -0.65)	NA	NA
**Body weight change**	Liraglutide *vs* Semaglutide (Oral)	1	NA	NA	NA	NA	NA	NA	NA	NA	1.3 (0.46 to 2.14)	NA	NA
**Body weight change**	Liraglutide *vs* Standard treatments	20	-1.572	18	0.133	-2.451	2.293	NA	NA	NA	-2.61 (-3.21 to -2.02)	-2.05 (-2.73 to -1.37)	-2.61 (-3.21 to -2.02)
**Body weight change**	Liraglutide *vs* Sulfonylureas	3	NA	NA	NA	NA	NA	NA	NA	NA	-3.34 (-4.01 to -2.68)	NA	NA
**Body weight change**	Liraglutide *vs* Thiazolidinediones	1	NA	NA	NA	NA	NA	NA	NA	NA	-2.3 (-3.14 to -1.46)	NA	NA
**Body weight change**	Lixisenatide *vs* Standard treatments	10	1.387	8	0.203	1.81	0.959	NA	NA	NA	-0.88 (-1.13 to -0.63)	-1.04 (-1.32 to -0.76)	-1.04 (-1.32 to -0.76)

Outcome	Comparisons	k	T-statistic	df	P-value	Bias estimate	tau	Odds ratio	Trim and fill odds ratio (L estimator)	Trim and fill odds ratio (R estimator)	Mean difference	Trim and fill mean difference (L estimator)	Trim and fill mean difference (R estimator)
**Body weight change**	Loxenatide *vs* Standard treatments	1	NA	NA	NA	NA	NA	NA	NA	NA	0.16 (-0.46 to 0.78)	NA	NA
**Body weight change**	Meglitinides *vs* Metformin	1	NA	NA	NA	NA	NA	NA	NA	NA	2.51 (0.97 to 4.05)	NA	NA
**Body weight change**	Meglitinides *vs* Standard treatments	4	NA	NA	NA	NA	NA	NA	NA	NA	1.04 (0.15 to 1.93)	NA	NA
**Body weight change**	Meglitinides *vs* Sulfonylureas	4	NA	NA	NA	NA	NA	NA	NA	NA	-0.47 (-0.97 to 0.04)	NA	NA
**Body weight change**	Meglitinides *vs* Thiazolidinediones	2	NA	NA	NA	NA	NA	NA	NA	NA	-1.25 (-2.64 to 0.13)	NA	NA
**Body weight change**	Metformin *vs* SGLT2 inhibitors	5	0.354	3	0.747	1.448	1.683	NA	NA	NA	1.69 (1.2 to 2.18)	NA	NA
**Body weight change**	Metformin *vs* Standard treatments	8	1.547	6	0.173	2.357	3.518	NA	NA	NA	-0.66 (-1.41 to 0.1)	NA	NA

Outcome	Comparisons	k	T-statistic	df	P-value	Bias estimate	tau	Odds ratio	Trim and fill odds ratio (L estimator)	Trim and fill odds ratio (R estimator)	Mean difference	Trim and fill mean difference (L estimator)	Trim and fill mean difference (R estimator)
**Body weight change**	Metformin *vs* Sulfonylureas	7	1.316	5	0.245	3.448	2.83	NA	NA	NA	-2.44 (-3.68 to -1.2)	NA	NA
**Body weight change**	Metformin *vs* Thiazolidinediones	10	-1.759	8	0.117	-2.846	3.97	NA	NA	NA	-4.11 (-5.09 to -3.14)	-3.27 (-4.48 to -2.06)	-3.56 (-4.91 to -2.21)
**Body weight change**	Semaglutide (Oral) *vs* Semaglutide (Subcutaneous)	1	NA	NA	NA	NA	NA	NA	NA	NA	1.6 (0.08 to 3.12)	NA	NA
**Body weight change**	Semaglutide (Oral) *vs* SGLT2 inhibitors	1	NA	NA	NA	NA	NA	NA	NA	NA	-0.3 (-1 to 0.4)	NA	NA
**Body weight change**	Semaglutide (Oral) *vs* Standard treatments	6	0.759	4	0.49	1.835	1.806	NA	NA	NA	-3.36 (-4.01 to -2.71)	NA	NA
**Body weight change**	Semaglutide (Subcutaneous) *vs* SGLT2 inhibitors	1	NA	NA	NA	NA	NA	NA	NA	NA	-1.4 (-1.93 to -0.87)	NA	NA
**Body weight change**	Semaglutide (Subcutaneous) *vs* Standard treatments	6	-0.367	4	0.732	-0.739	2.115	NA	NA	NA	-4.9 (-5.83 to -3.96)	NA	NA

Outcome	Comparisons	k	T-statistic	df	P-value	Bias estimate	tau	Odds ratio	Trim and fill odds ratio (L estimator)	Trim and fill odds ratio (R estimator)	Mean difference	Trim and fill mean difference (L estimator)	Trim and fill mean difference (R estimator)
**Body weight change**	Semaglutide (Subcutaneous) *vs* Tirzepatide	2	NA	NA	NA	NA	NA	NA	NA	NA	3.99 (3.27 to 4.72)	NA	NA
**Body weight change**	SGLT2 inhibitors *vs* Standard treatments	70	-1.671	68	0.099	-1.035	3.146	NA	NA	NA	-1.9 (-2.1 to -1.7)	-1.4 (-1.62 to -1.17)	-1.9 (-2.1 to -1.7)
**Body weight change**	SGLT2 inhibitors *vs* Sulfonylureas	9	0.521	7	0.618	0.827	1.908	NA	NA	NA	-4.3 (-4.74 to -3.86)	NA	NA
**Body weight change**	SGLT2 inhibitors *vs* Thiazolidinediones	3	NA	NA	NA	NA	NA	NA	NA	NA	-4.29 (-5.6 to -2.99)	NA	NA
**Body weight change**	Sulfonylureas *vs* Standard treatments	4	NA	NA	NA	NA	NA	NA	NA	NA	2.78 (2.21 to 3.35)	NA	NA
**Body weight change**	Sulfonylureas *vs* Thiazolidinediones	17	0.253	15	0.804	0.956	4.802	NA	NA	NA	-1.19 (-1.93 to -0.45)	-1.19 (-1.93 to -0.45)	-1.19 (-1.93 to -0.45)
**Body weight change**	Thiazolidinediones *vs* Standard treatments	32	2.625	30	0.013	3.088	2.842	NA	NA	NA	3.02 (2.54 to 3.5)	-3.89 (-4.47 to -3.31)	-3.29 (-3.83 to -2.75)

Outcome	Comparisons	k	T-statistic	df	P-value	Bias estimate	tau	Odds ratio	Trim and fill odds ratio (L estimator)	Trim and fill odds ratio (R estimator)	Mean difference	Trim and fill mean difference (L estimator)	Trim and fill mean difference (R estimator)
**Body weight change**	Tirzepatide *vs* Standard treatments	4	NA	NA	NA	NA	NA	NA	NA	NA	-8.19 (-10.1 to -6.28)	NA	NA

4.7.2 Small-study effect assessed by comparison-adjusted funnel plots

Notes: We evaluated the global small-study effect in the network meta-analysis as well using the comparison-adjusted funnel plot. The treatment effects are adjusted by applying network estimates minus direct estimates.

4.7.2.1 All-cause death

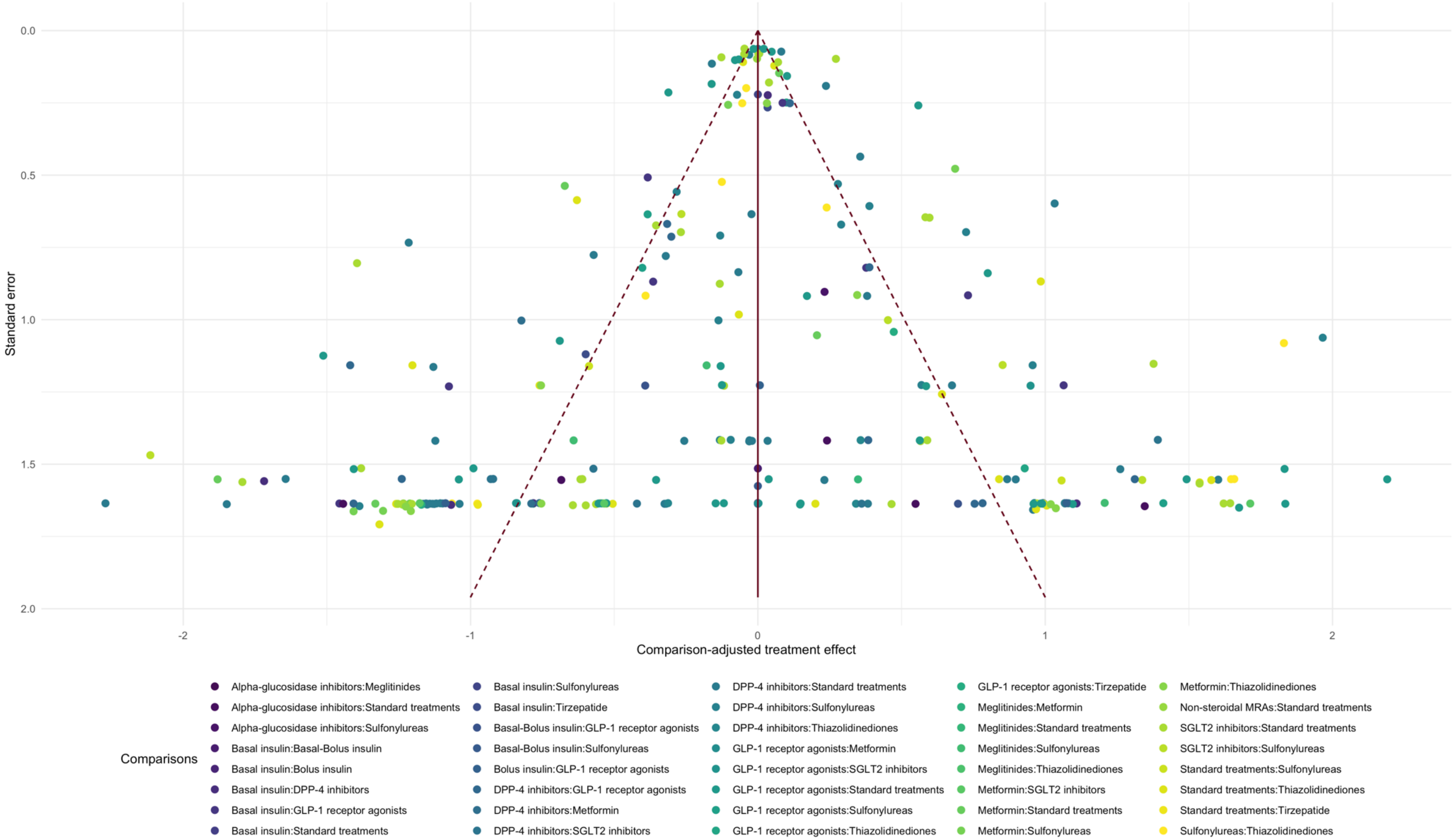

4.7.2.2 Cardiovascular death

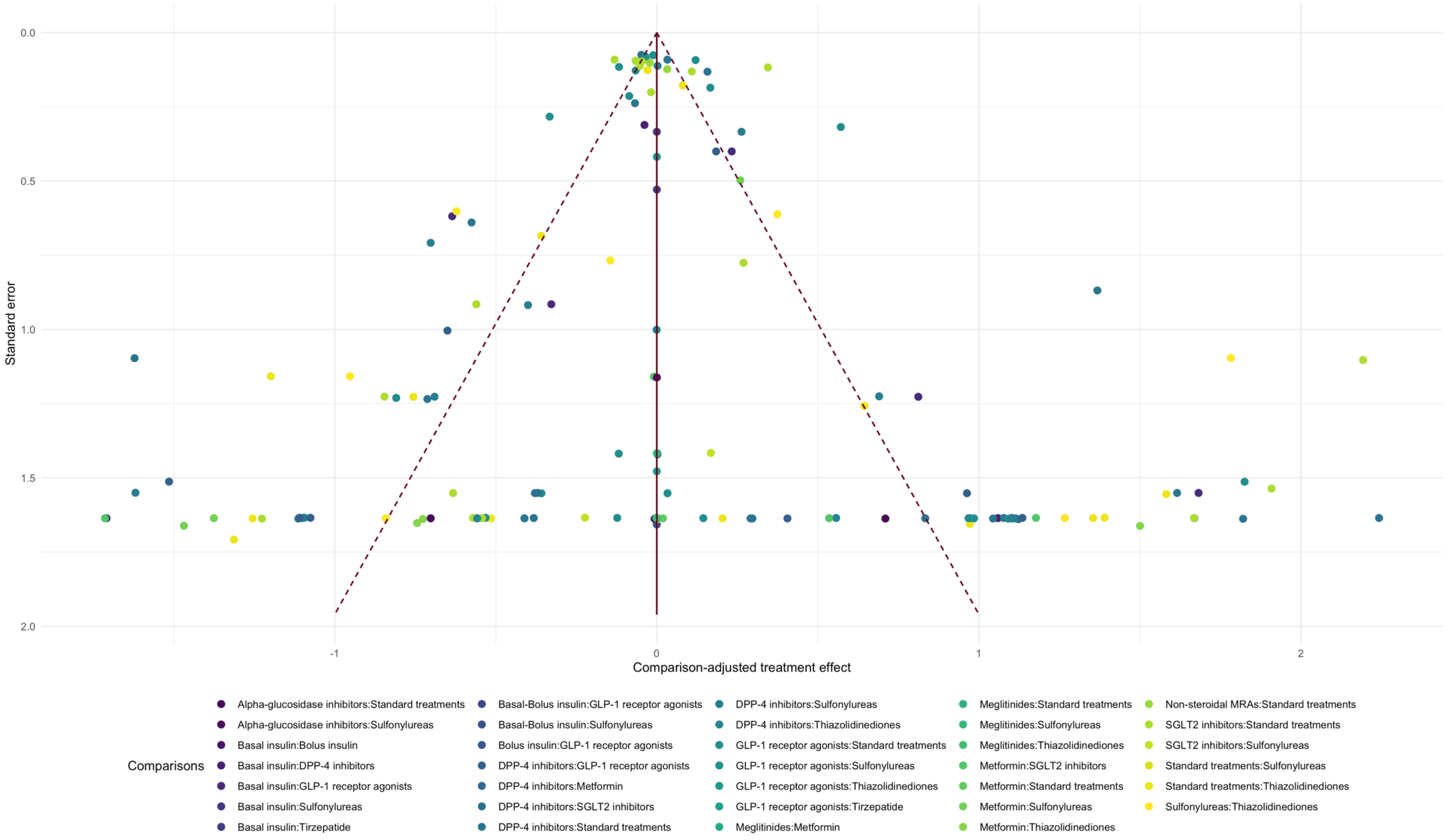

4.7.2.3 Non-fatal myocardial infarction

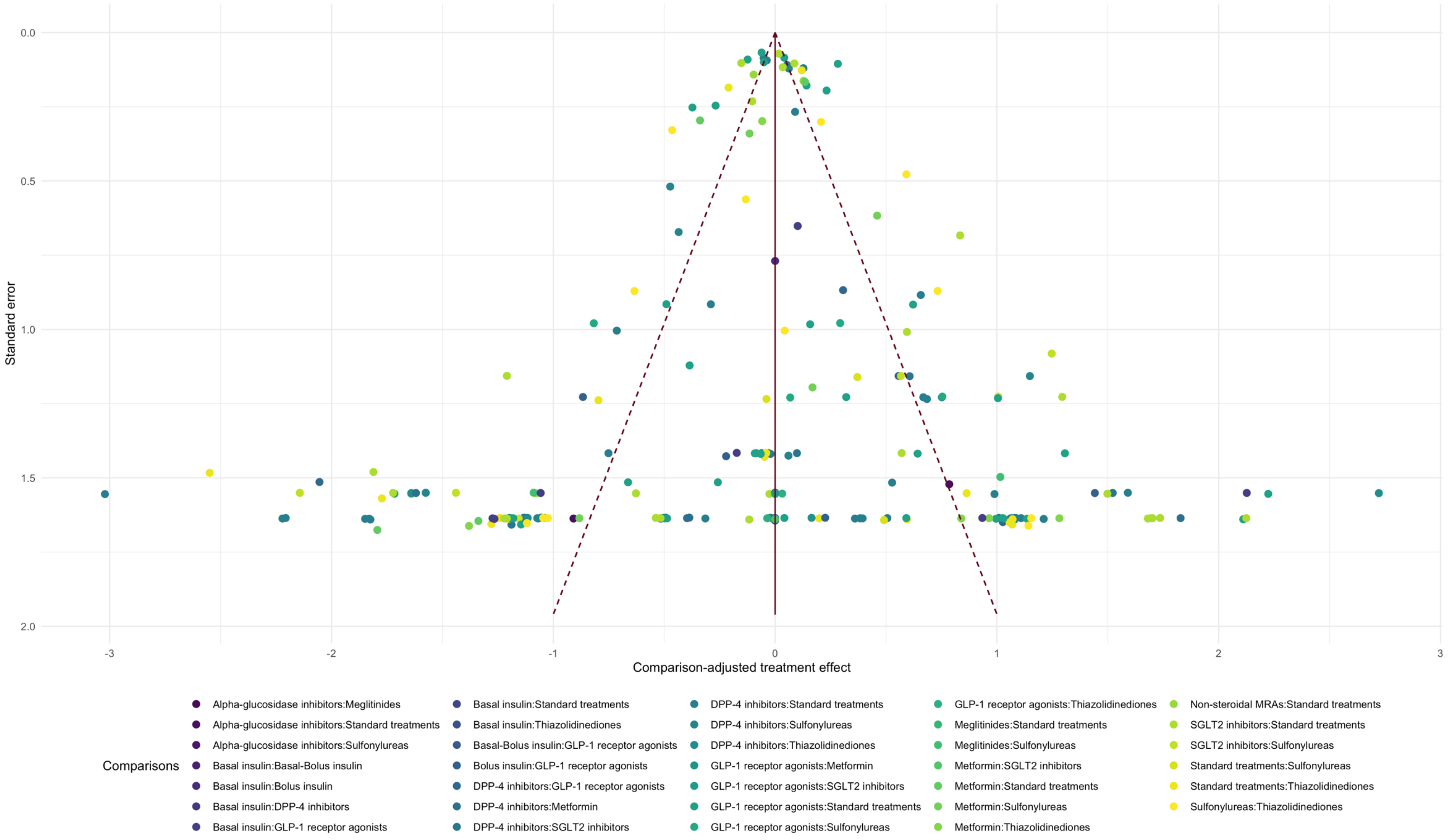

4.7.2.4 Non-fatal stroke

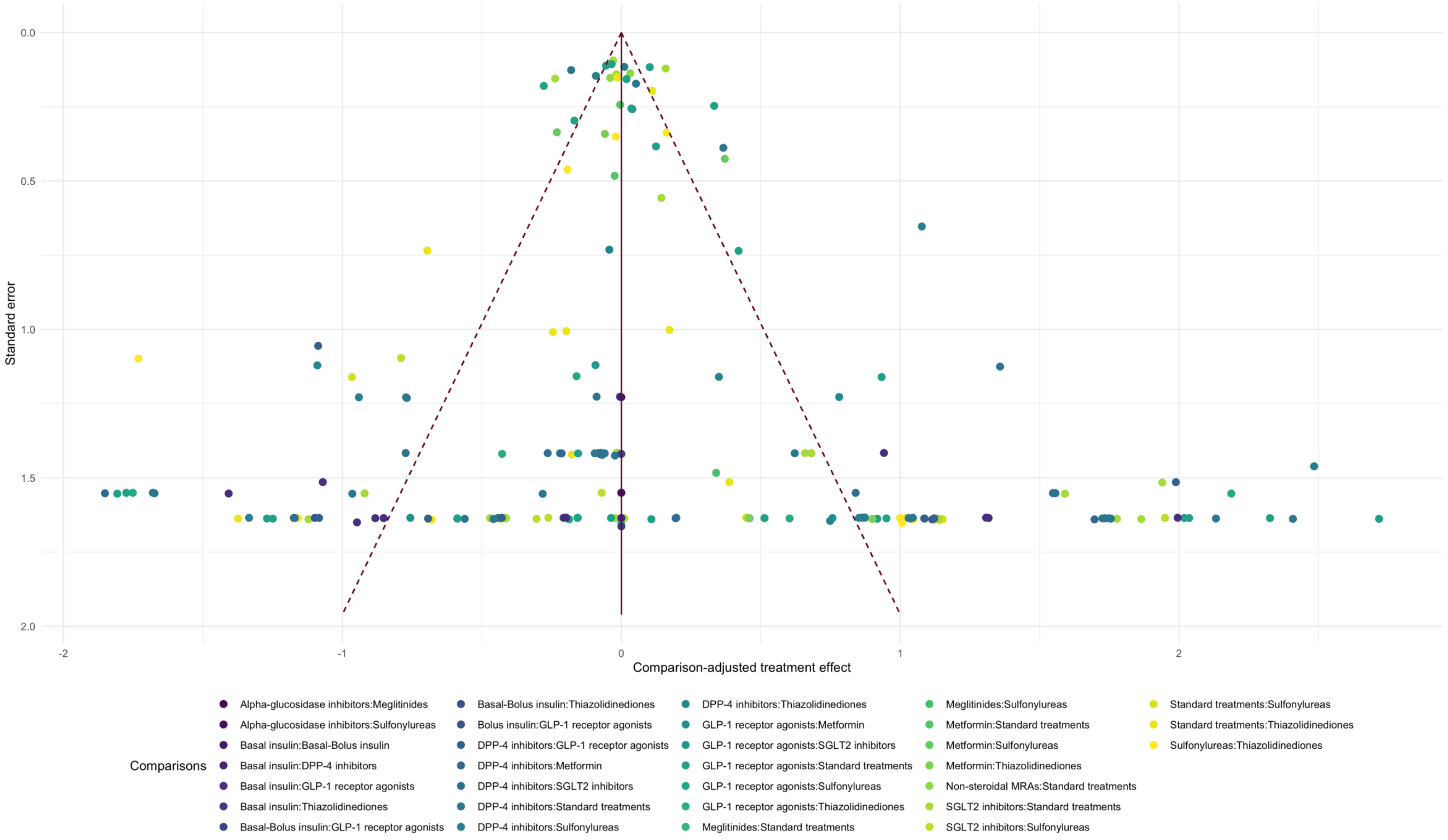

4.7.2.5 Hospitalization for heart failure

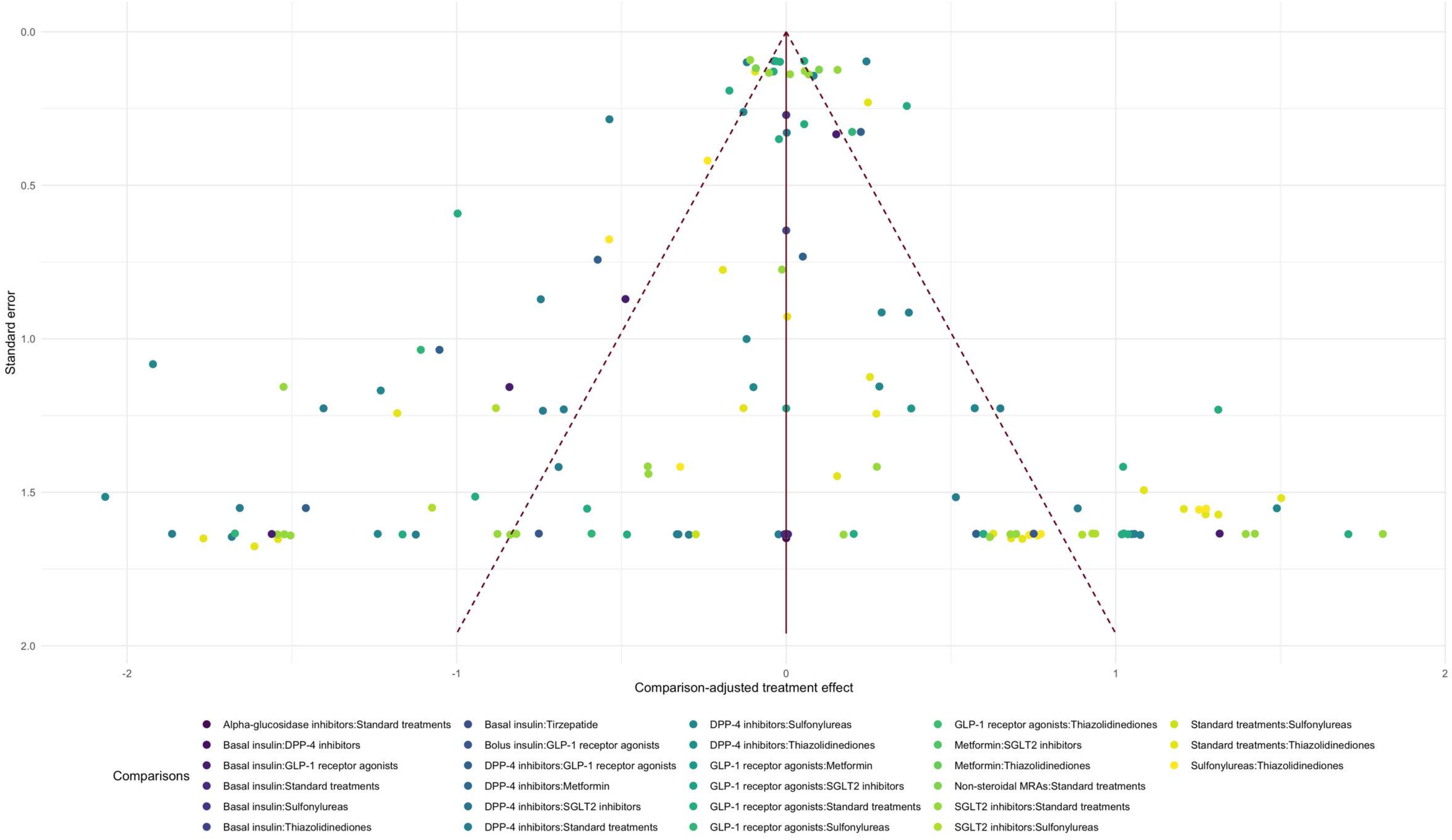

4.7.2.6 End-stage kidney disease

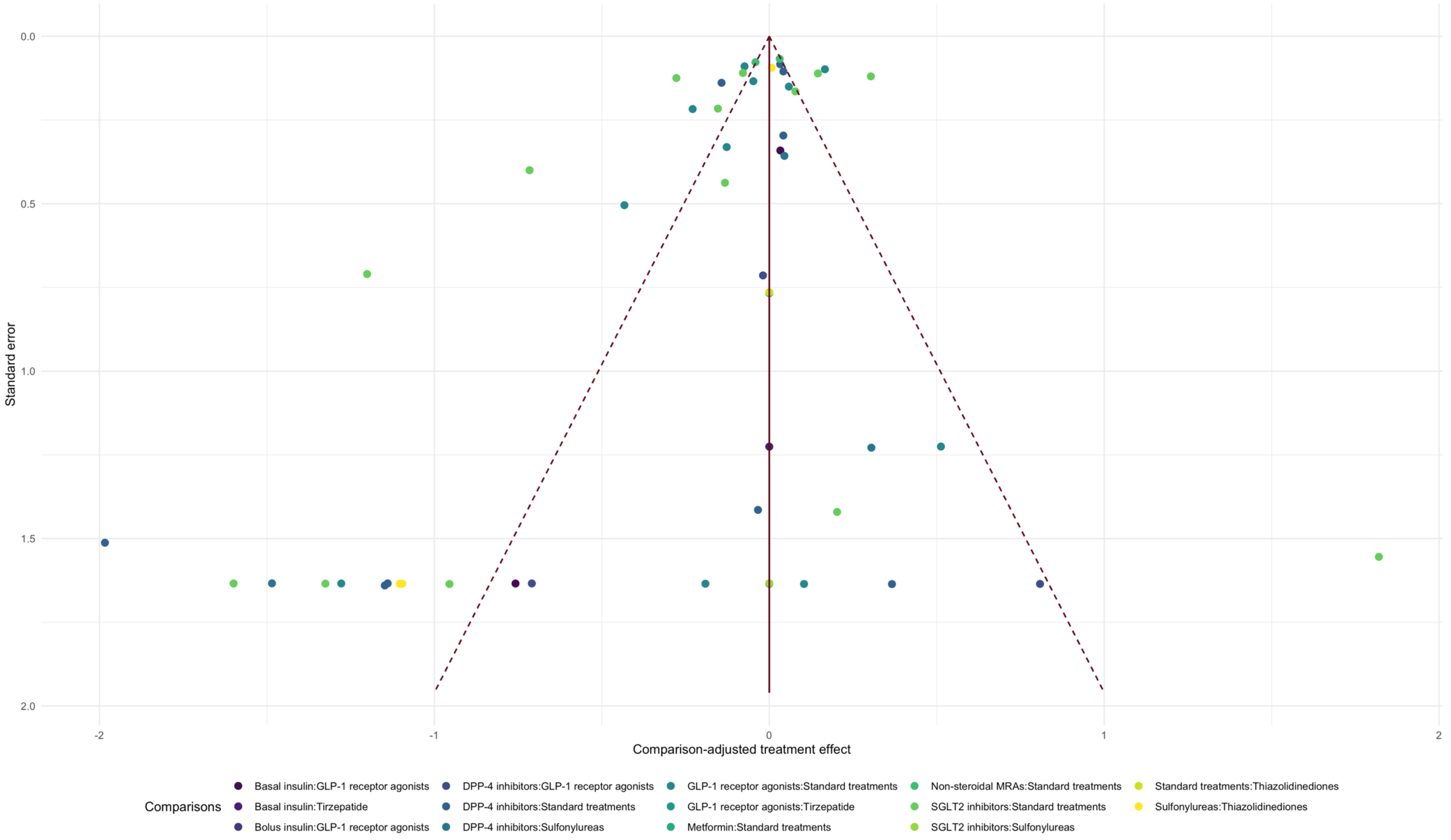

4.7.2.7 Severe hypoglycaemia

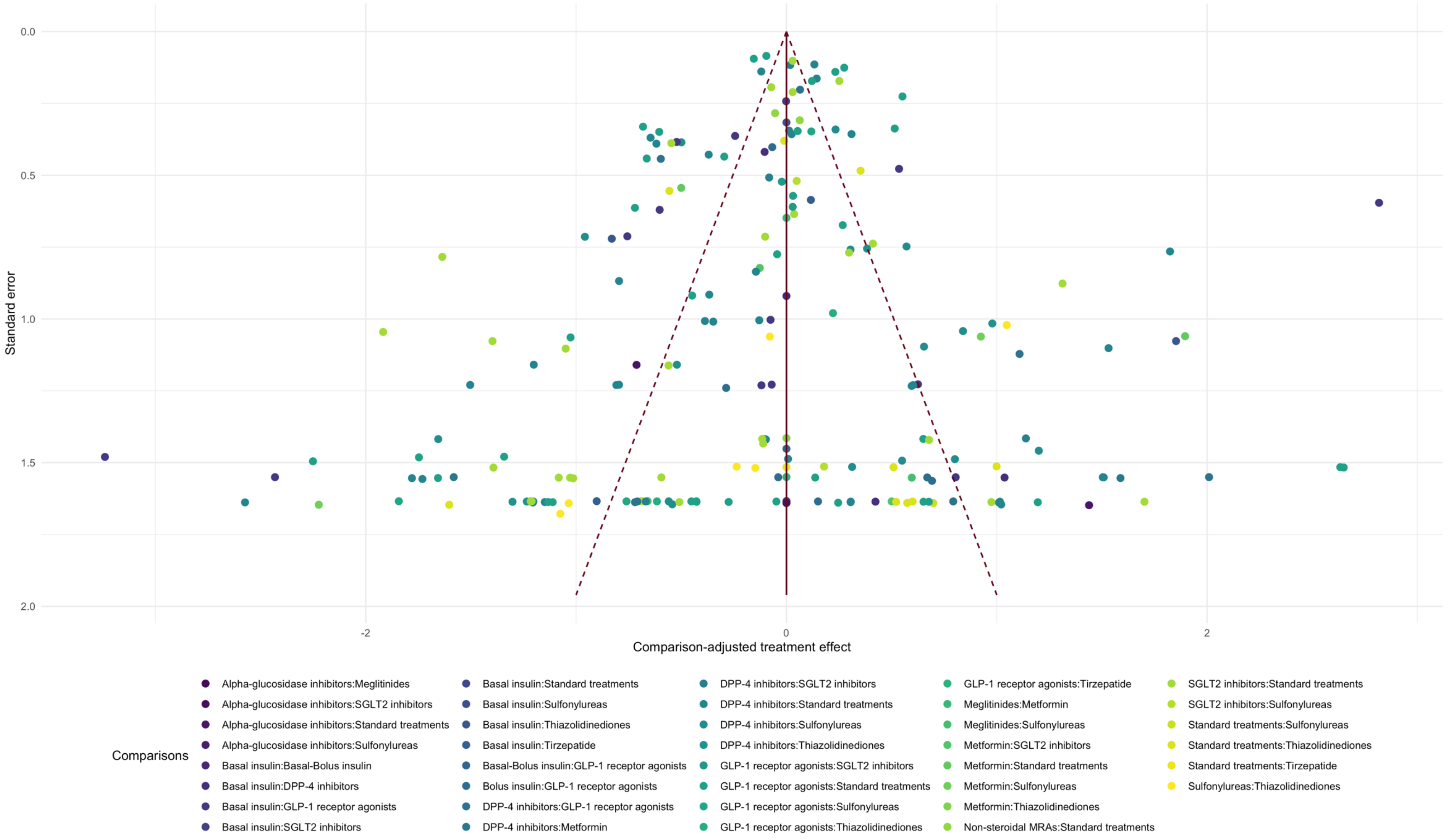

4.7.2.8 Severe gastrointestinal events

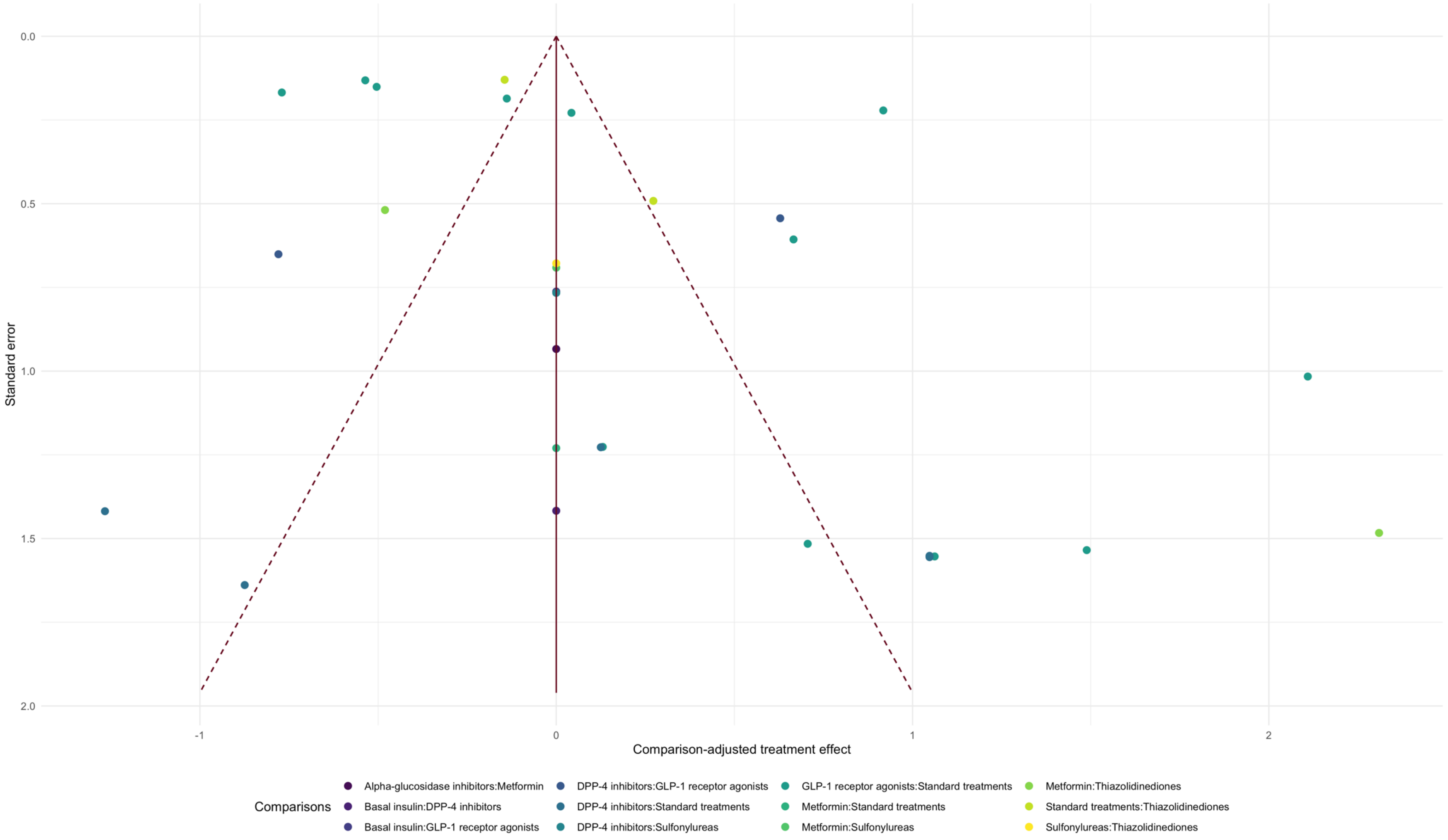

4.7.2.9 Genital infection

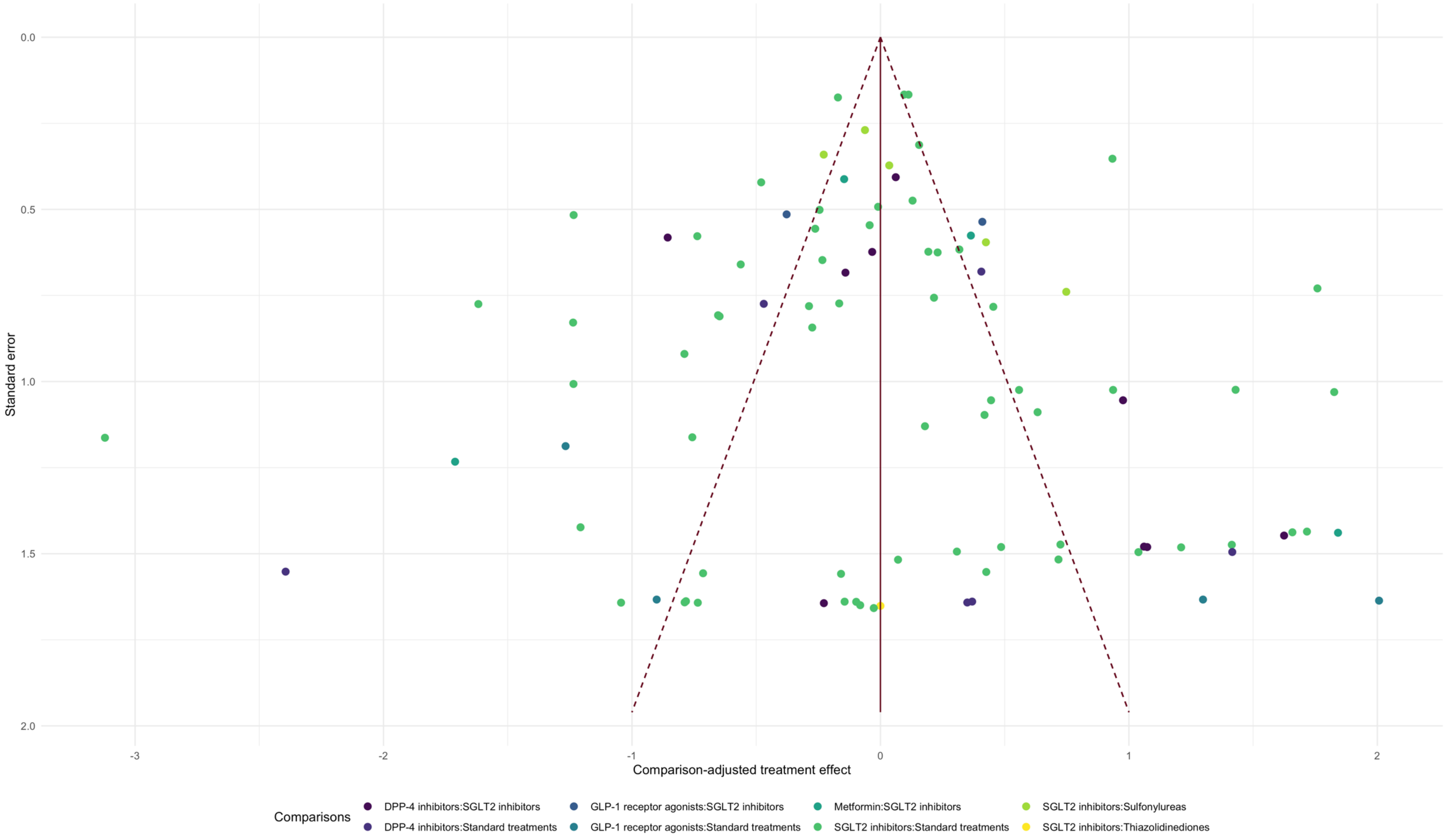

4.7.2.10 Amputation

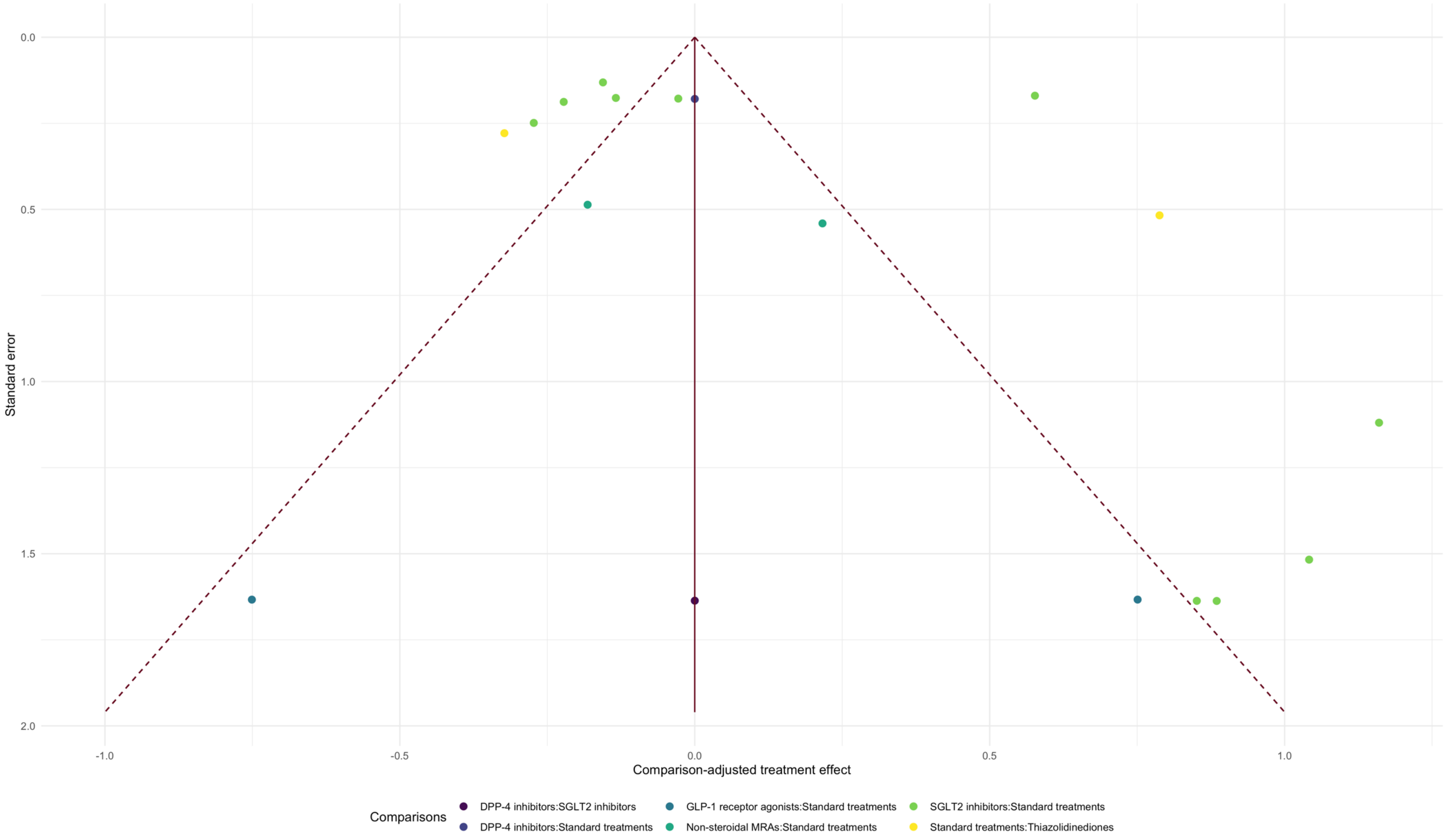

4.7.2.11 Ketoacidosis due to diabetes

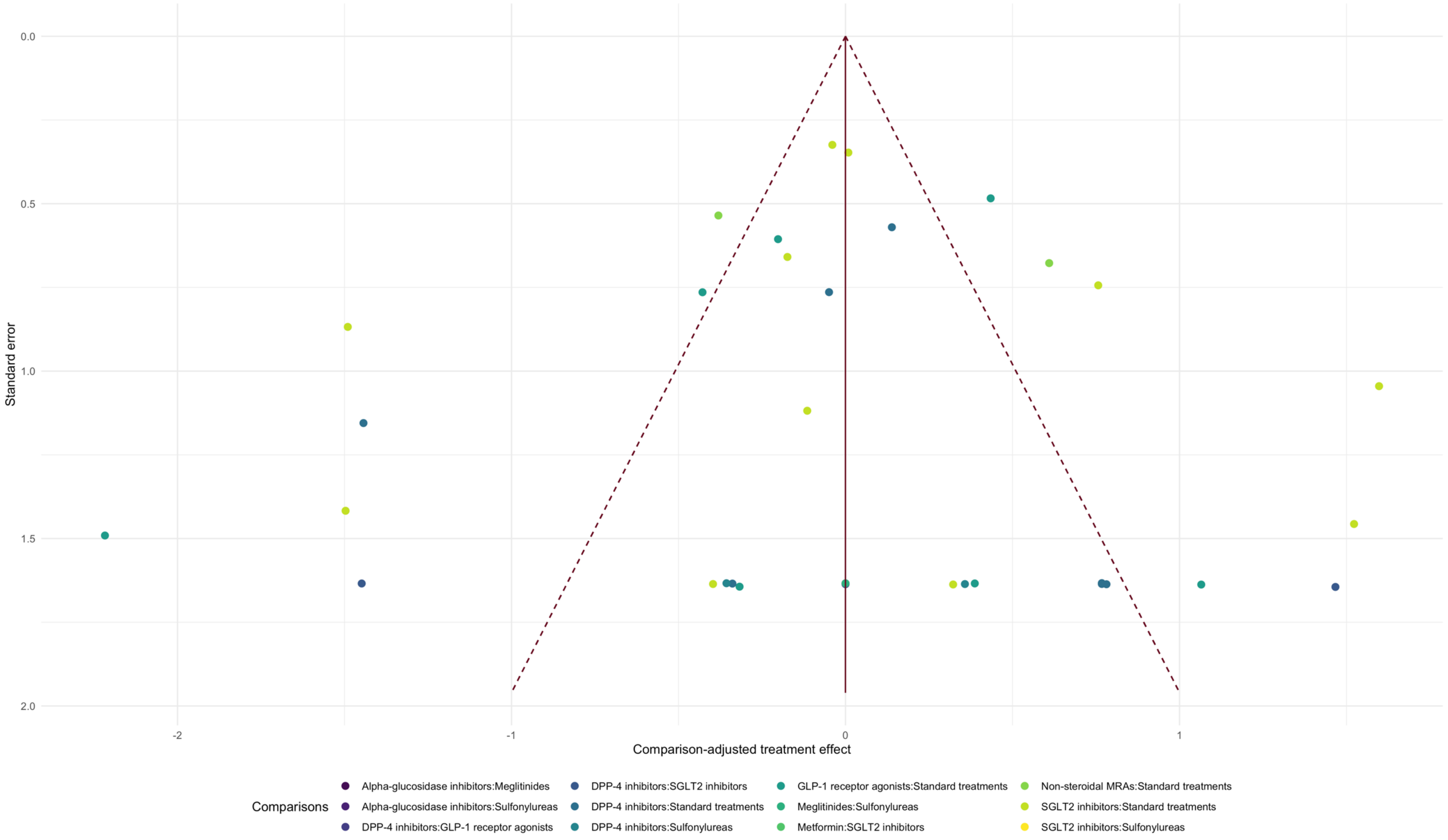

4.7.2.12 Health-related quality of life

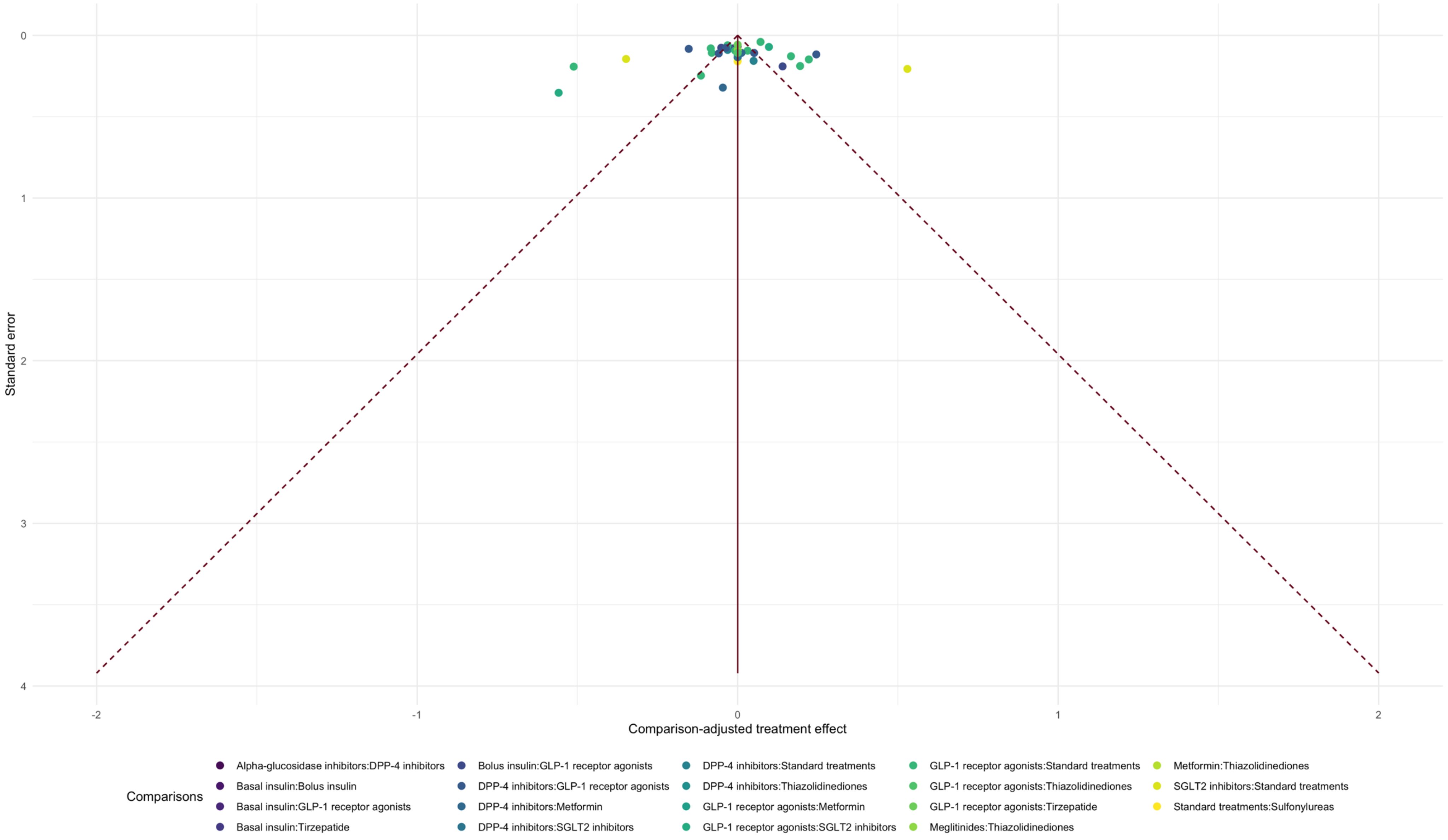

4.7.2.13 Bodyweight change

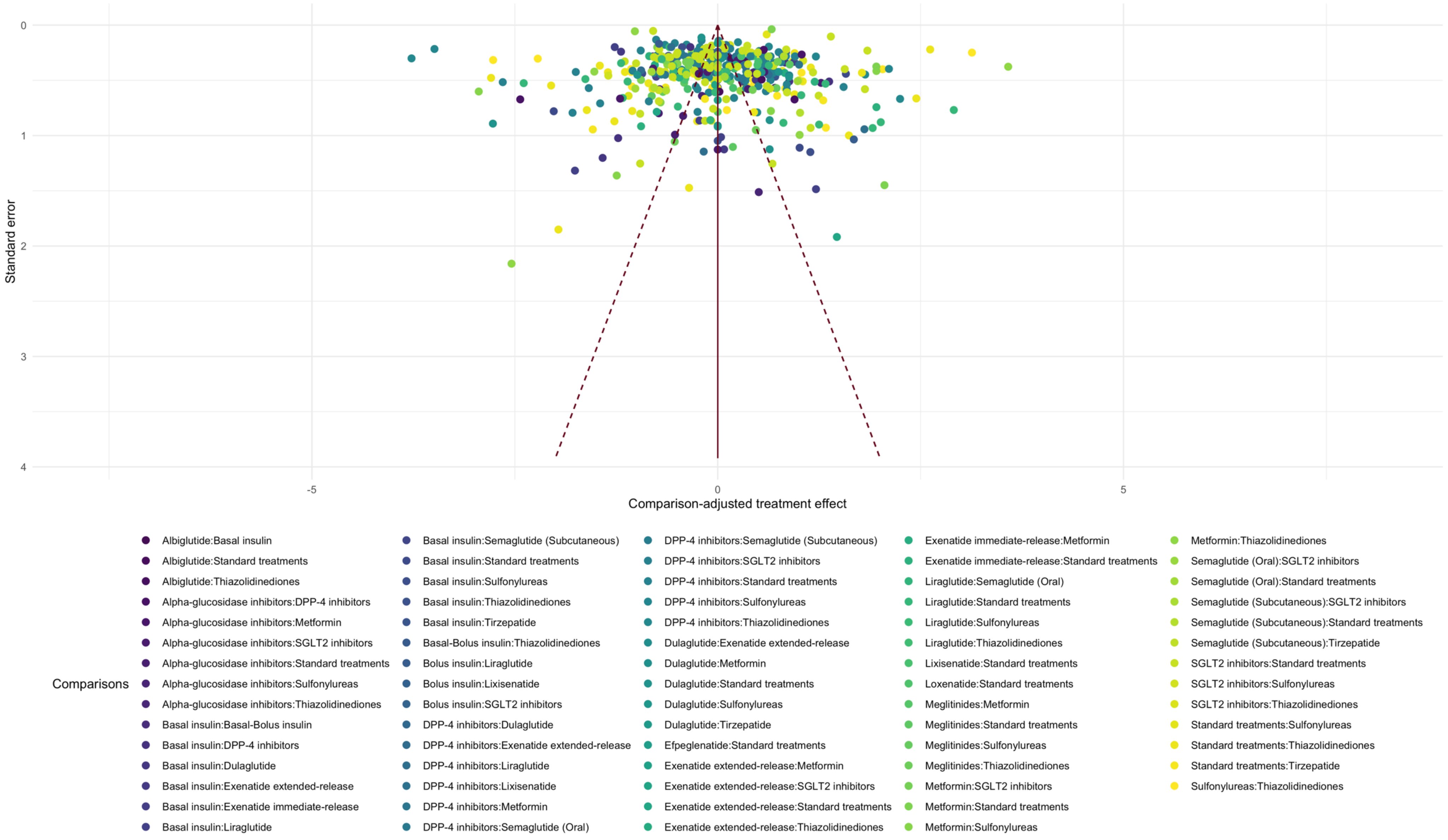

4.8 Between-study variance

Outcome	Between-study variance (tau^2)
All-cause death	0
Cardiovascular death	0
Non-fatal myocardial infarction	0
Non-fatal stroke	0
Hospitalisation for heart failure	0
End-stage kidney disease	0
Health-related quality of life	0.0197
Bodyweight change	0.8217
Severe hypoglycaemia	0
Severe gastrointestinal events	0.2479
Genital infection	0
Amputation	0.0541
Ketoacidosis due to diabetes	0
Hyperkalaemia leading to hospitalisation	0

Appendix 5 GRADE certainty of evidence assessments

5.1 GRADE assessment table

Notes: The study followed GRADE guidance to assess the certainty of evidence. The assessment starts from high certainty for direct evidence and then potentially is rated down for risk of bias (D1), indirectness (D2), heterogeneity (D3) and local publication bias (D4). The evidence from indirect comparisons starts as a certainty that consists of the certainty from direct evidence that contributes to the indirect evidence, and then is rated down for intransitivity (D5) if necessary. The evidence from network comparisons starts as a certainty that consists of the certainty of direct evidence and indirect evidence weighted by the proportion of contribution (Prop), and then is rated down for incoherence (D6) and imprecision (D7). We used the random walk approach to calculate the percentage contribution matrix of network comparisons and indirect comparisons linked to direct comparisons via the network structure (see results in 5.2). An online tool facilitates the quick inquiry (https://qingys.shinyapps.io/data_visualization).

Abbreviations: D1, risk of bias; D2, indirectness; D3, heterogeneity (inconsistency); D4, publication bias; D5, intransitivity; D6, incoherence; D7, imprecision; k, no. of studies; NMA, network meta-analysis; Prop, proportion of contribution of direct estimates to network estimates; MI, myocardial infarction; HHF, hospitalization for heart failure; ESKD, end-stage kidney disease; DKA, Ketoacidosis due to diabetes; HRQoL, health-related quality of life.

Outcome	Comparisons	k	Direct estimates	D1	D2	D3	D4	Direct Certainty	Indirect estimates	Initial Indirect Certainty	D5	Indirect Certainty	NMA estimates	Prop	Initial NMA Certainty	D6	D7	NMA Certainty
**All-cause death**	Alpha-glucosidase inhibitors *vs* Basal insulin	0	NA	NA	NA	NA	NA	NA	0.81 (0.26 to 2.47)	Moderate	0	Moderate	0.81 (0.26 to 2.47)	0	Moderate	NA	-2	Very low
**All-cause death**	Alpha-glucosidase inhibitors *vs* Basal-Bolus insulin	0	NA	NA	NA	NA	NA	NA	1.12 (0.19 to 6.74)	Moderate	0	Moderate	1.12 (0.19 to 6.74)	0	Moderate	NA	-2	Very low
**All-cause death**	Alpha-glucosidase inhibitors *vs* Bolus insulin	0	NA	NA	NA	NA	NA	NA	1.85 (0.37 to 9.23)	Low	0	Low	1.85 (0.37 to 9.23)	0	Low	NA	-2	Very low
**All-cause death**	Alpha-glucosidase inhibitors *vs* DPP-4 inhibitors	0	NA	NA	NA	NA	NA	NA	0.88 (0.30 to 2.58)	Low	0	Low	0.88 (0.30 to 2.58)	0	Low	NA	-2	Very low

Outcome	Comparisons	k	Direct estimates	D1	D2	D3	D4	Direct Certainty	Indirect estimates	Initial Indirect Certainty	D5	Indirect Certainty	NMA estimates	Prop	Initial NMA Certainty	D6	D7	NMA Certainty
**All-cause death**	Alpha-glucosidase inhibitors *vs* GLP-1 receptor agonists	0	NA	NA	NA	NA	NA	NA	1.01 (0.34 to 2.98)	Moderate	0	Moderate	1.01 (0.34 to 2.98)	0	Moderate	NA	-2	Very low
**All-cause death**	Alpha-glucosidase inhibitors *vs* Meglitinides	1	0.33 (0.01 to 8.10)	-2	0	0	0	Low	0.65 (0.12 to 3.48)	Low	0	Low	0.56 (0.13 to 2.48)	0.22	Low	0	-2	Very low
**All-cause death**	Alpha-glucosidase inhibitors *vs* Metformin	0	NA	NA	NA	NA	NA	NA	1.06 (0.36 to 3.18)	Moderate	0	Moderate	1.06 (0.36 to 3.18)	0	Moderate	NA	-2	Very low
**All-cause death**	Alpha-glucosidase inhibitors *vs* Non-steroidal MRAs	0	NA	NA	NA	NA	NA	NA	1.00 (0.34 to 2.95)	Low	0	Low	1.00 (0.34 to 2.95)	0	Low	NA	-2	Very low
**All-cause death**	Alpha-glucosidase inhibitors *vs* SGLT2 inhibitors	0	NA	NA	NA	NA	NA	NA	1.01 (0.34 to 2.96)	Moderate	0	Moderate	1.01 (0.34 to 2.96)	0	Moderate	NA	-2	Very low
**All-cause death**	Alpha-glucosidase inhibitors *vs* Standard treatments	5	1.31 (0.33 to 5.18)	-1	0	0	0	Moderate	0.48 (0.09 to 2.72)	Low	0	Low	0.89 (0.30 to 2.61)	0.61	Moderate	0	-2	Very low
**All-cause death**	Alpha-glucosidase inhibitors *vs* Sulfonylureas	2	0.64 (0.13 to 3.21)	-1	0	0	0	Moderate	0.97 (0.23 to 4.13)	Low	0	Low	0.81 (0.27 to 2.37)	0.45	Low	0	-2	Very low
**All-cause death**	Alpha-glucosidase inhibitors *vs* Thiazolidinediones	0	NA	NA	NA	NA	NA	NA	0.93 (0.32 to 2.76)	Low	0	Low	0.93 (0.32 to 2.76)	0	Low	NA	-2	Very low
**All-cause death**	Alpha-glucosidase inhibitors *vs* Tirzepatide	0	NA	NA	NA	NA	NA	NA	1.07 (0.32 to 3.57)	Moderate	0	Moderate	1.07 (0.32 to 3.57)	0	Moderate	NA	-2	Very low
**All-cause death**	Basal insulin *vs* Basal-Bolus insulin	1	7.03 (0.36 to 136.82)	-2	0	0	0	Low	0.84 (0.16 to 4.40)	Moderate	0	Moderate	1.39 (0.33 to 5.92)	0.24	Moderate	0	-2	Very low
**All-cause death**	Basal insulin *vs* Bolus insulin	1	0.34 (0.01 to 8.36)	-2	0	0	0	Low	3.16 (0.84 to 11.85)	Low	0	Low	2.28 (0.67 to 7.75)	0.15	Low	0	-2	Very low
**All-cause death**	Basal insulin *vs* DPP-4 inhibitors	4	0.99 (0.65 to 1.52)	-1	0	0	0	Moderate	1.19 (0.77 to 1.83)	Moderate	0	Moderate	1.08 (0.80 to 1.47)	0.51	Moderate	0	-2	Very low

Outcome	Comparisons	k	Direct estimates	D1	D2	D3	D4	Direct Certainty	Indirect estimates	Initial Indirect Certainty	D5	Indirect Certainty	NMA estimates	Prop	Initial NMA Certainty	D6	D7	NMA Certainty
**All-cause death**	Basal insulin *vs* GLP-1 receptor agonists	9	1.44 (0.98 to 2.12)	-1	0	0	0	Moderate	0.99 (0.61 to 1.63)	Moderate	0	Moderate	1.25 (0.93 to 1.70)	0.62	Moderate	0	-2	Very low
**All-cause death**	Basal insulin *vs* Meglitinides	0	NA	NA	NA	NA	NA	NA	0.69 (0.22 to 2.24)	Low	0	Low	0.69 (0.22 to 2.24)	0	Low	NA	-2	Very low
**All-cause death**	Basal insulin *vs* Metformin	0	NA	NA	NA	NA	NA	NA	1.31 (0.91 to 1.90)	Moderate	0	Moderate	1.31 (0.91 to 1.90)	0	Moderate	NA	-2	Very low
**All-cause death**	Basal insulin *vs* Non-steroidal MRAs	0	NA	NA	NA	NA	NA	NA	1.24 (0.89 to 1.71)	Moderate	0	Moderate	1.24 (0.89 to 1.71)	0	Moderate	NA	-2	Very low
**All-cause death**	Basal insulin *vs* SGLT2 inhibitors	0	NA	NA	NA	NA	NA	NA	1.24 (0.91 to 1.70)	Moderate	0	Moderate	1.24 (0.91 to 1.70)	0	Moderate	NA	-2	Very low
**All-cause death**	Basal insulin *vs* Standard treatments	1	0.32 (0.01 to 7.99)	-1	0	0	0	Moderate	1.11 (0.82 to 1.50)	Moderate	0	Moderate	1.10 (0.81 to 1.49)	0.0089	Moderate	0	-2	Very low
**All-cause death**	Basal insulin *vs* Sulfonylureas	1	0.97 (0.63 to 1.49)	0	0	0	0	High	1.03 (0.65 to 1.61)	Moderate	0	Moderate	1.00 (0.73 to 1.36)	0.52	High	0	-2	Low
**All-cause death**	Basal insulin *vs* Thiazolidinediones	0	NA	NA	NA	NA	NA	NA	1.15 (0.83 to 1.60)	Moderate	0	Moderate	1.15 (0.83 to 1.60)	0	Moderate	NA	-2	Very low
**All-cause death**	Basal insulin *vs* Tirzepatide	2	1.36 (0.82 to 2.26)	0	0	0	0	High	1.02 (0.24 to 4.40)	Moderate	0	Moderate	1.32 (0.82 to 2.13)	0.89	High	0	-2	Low
**All-cause death**	Basal-Bolus insulin *vs* Bolus insulin	0	NA	NA	NA	NA	NA	NA	1.64 (0.25 to 10.61)	Low	0	Low	1.64 (0.25 to 10.61)	0	Low	NA	-2	Very low
**All-cause death**	Basal-Bolus insulin *vs* DPP-4 inhibitors	0	NA	NA	NA	NA	NA	NA	0.78 (0.19 to 3.28)	Moderate	0	Moderate	0.78 (0.19 to 3.28)	0	Moderate	NA	-2	Very low
**All-cause death**	Basal-Bolus insulin *vs* GLP-1 receptor agonists	2	0.75 (0.11 to 5.16)	-1	0	0	0	Moderate	1.13 (0.13 to 9.70)	Low	0	Low	0.90 (0.22 to 3.78)	0.55	Moderate	0	-2	Very low

Outcome	Comparisons	k	Direct estimates	D1	D2	D3	D4	Direct Certainty	Indirect estimates	Initial Indirect Certainty	D5	Indirect Certainty	NMA estimates	Prop	Initial NMA Certainty	D6	D7	NMA Certainty
**All-cause death**	Basal-Bolus insulin *vs* Meglitinides	0	NA	NA	NA	NA	NA	NA	0.50 (0.08 to 3.11)	Low	0	Low	0.50 (0.08 to 3.11)	0	Low	NA	-2	Very low
**All-cause death**	Basal-Bolus insulin *vs* Metformin	0	NA	NA	NA	NA	NA	NA	0.95 (0.22 to 4.04)	Moderate	0	Moderate	0.95 (0.22 to 4.04)	0	Moderate	NA	-2	Very low
**All-cause death**	Basal-Bolus insulin *vs* Non-steroidal MRAs	0	NA	NA	NA	NA	NA	NA	0.89 (0.21 to 3.75)	Moderate	0	Moderate	0.89 (0.21 to 3.75)	0	Moderate	NA	-2	Very low
**All-cause death**	Basal-Bolus insulin *vs* SGLT2 inhibitors	0	NA	NA	NA	NA	NA	NA	0.90 (0.21 to 3.76)	Moderate	0	Moderate	0.90 (0.21 to 3.76)	0	Moderate	NA	-2	Very low
**All-cause death**	Basal-Bolus insulin *vs* Standard treatments	0	NA	NA	NA	NA	NA	NA	0.79 (0.19 to 3.32)	Moderate	0	Moderate	0.79 (0.19 to 3.32)	0	Moderate	NA	-2	Very low
**All-cause death**	Basal-Bolus insulin *vs* Sulfonylureas	1	6.63 (0.30 to 145.31)	-2	0	0	0	Low	0.39 (0.08 to 1.97)	Moderate	0	Moderate	0.72 (0.17 to 3.02)	0.22	Moderate	0	-2	Very low
**All-cause death**	Basal-Bolus insulin *vs* Thiazolidinediones	0	NA	NA	NA	NA	NA	NA	0.83 (0.20 to 3.51)	Moderate	0	Moderate	0.83 (0.20 to 3.51)	0	Moderate	NA	-2	Very low
**All-cause death**	Basal-Bolus insulin *vs* Tirzepatide	0	NA	NA	NA	NA	NA	NA	0.95 (0.21 to 4.35)	Moderate	0	Moderate	0.95 (0.21 to 4.35)	0	Moderate	NA	-2	Very low
**All-cause death**	Bolus insulin *vs* DPP-4 inhibitors	0	NA	NA	NA	NA	NA	NA	0.47 (0.14 to 1.57)	Low	0	Low	0.47 (0.14 to 1.57)	0	Low	NA	-2	Very low
**All-cause death**	Bolus insulin *vs* GLP-1 receptor agonists	4	0.40 (0.11 to 1.46)	-2	0	0	0	Low	3.76 (0.15 to 94.11)	Low	0	Low	0.55 (0.17 to 1.81)	0.86	Low	0	-2	Very low
**All-cause death**	Bolus insulin *vs* Meglitinides	0	NA	NA	NA	NA	NA	NA	0.30 (0.06 to 1.58)	Low	0	Low	0.30 (0.06 to 1.58)	0	Low	NA	-2	Very low
**All-cause death**	Bolus insulin *vs* Metformin	0	NA	NA	NA	NA	NA	NA	0.58 (0.17 to 1.94)	Moderate	0	Moderate	0.58 (0.17 to 1.94)	0	Moderate	NA	-2	Very low

**Outcome**	**Comparisons**	**k**	**Direct estimates**	**D1**	**D2**	**D3**	**D4**	**Direct Certainty**	**Indirect estimates**	**Initial Indirect Certainty**	**D5**	**Indirect Certainty**	**NMA estimates**	**Prop**	**Initial NMA Certainty**	**D6**	**D7**	**NMA Certainty**
**All-cause death**	Bolus insulin *vs* Non-steroidal MRAs	0	NA	NA	NA	NA	NA	NA	0.54 (0.16 to 1.80)	Low	0	Low	0.54 (0.16 to 1.80)	0	Low	NA	-2	Very low
**All-cause death**	Bolus insulin *vs* SGLT2 inhibitors	0	NA	NA	NA	NA	NA	NA	0.54 (0.16 to 1.81)	Moderate	0	Moderate	0.54 (0.16 to 1.81)	0	Moderate	NA	-2	Very low
**All-cause death**	Bolus insulin *vs* Standard treatments	0	NA	NA	NA	NA	NA	NA	0.48 (0.15 to 1.59)	Low	0	Low	0.48 (0.15 to 1.59)	0	Low	NA	-2	Very low
**All-cause death**	Bolus insulin *vs* Sulfonylureas	0	NA	NA	NA	NA	NA	NA	0.44 (0.13 to 1.45)	Low	0	Low	0.44 (0.13 to 1.45)	0	Low	NA	-2	Very low
**All-cause death**	Bolus insulin *vs* Thiazolidinediones	0	NA	NA	NA	NA	NA	NA	0.51 (0.15 to 1.68)	Low	0	Low	0.51 (0.15 to 1.68)	0	Low	NA	-2	Very low
**All-cause death**	Bolus insulin *vs* Tirzepatide	0	NA	NA	NA	NA	NA	NA	0.58 (0.16 to 2.13)	Moderate	0	Moderate	0.58 (0.16 to 2.13)	0	Moderate	NA	-2	Very low
**All-cause death**	DPP-4 inhibitors *vs* GLP-1 receptor agonists	13	1.37 (0.93 to 2.02)	-1	0	0	0	Moderate	1.14 (1.04 to 1.26)	Moderate	0	Moderate	1.16 (1.06 to 1.26)	0.053	Moderate	0	0	Moderate
**All-cause death**	DPP-4 inhibitors *vs* Meglitinides	0	NA	NA	NA	NA	NA	NA	0.64 (0.21 to 1.99)	Low	0	Low	0.64 (0.21 to 1.99)	0	Low	NA	-2	Very low
**All-cause death**	DPP-4 inhibitors *vs* Metformin	5	1.10 (0.32 to 3.81)	0	0	0	0	High	1.22 (0.97 to 1.53)	Moderate	0	Moderate	1.21 (0.97 to 1.52)	0.032	Moderate	0	-1	Low
**All-cause death**	DPP-4 inhibitors *vs* Non-steroidal MRAs	0	NA	NA	NA	NA	NA	NA	1.14 (0.99 to 1.31)	Moderate	0	Moderate	1.14 (0.99 to 1.31)	0	Moderate	NA	-1	Low
**All-cause death**	DPP-4 inhibitors *vs* SGLT2 inhibitors	7	0.50 (0.15 to 1.63)	0	0	0	0	High	1.15 (1.05 to 1.27)	Moderate	0	Moderate	1.15 (1.04 to 1.26)	0.006 3	Moderate	0	0	Moderate
**All-cause death**	DPP-4 inhibitors *vs* Standard treatments	49	1.02 (0.95 to 1.10)	-1	0	0	0	Moderate	0.94 (0.75 to 1.17)	Moderate	0	Moderate	1.01 (0.95 to 1.08)	0.91	Moderate	0	-1	Low

Outcome	Comparisons	k	Direct estimates	D1	D2	D3	D4	Direct Certainty	Indirect estimates	Initial Indirect Certainty	D5	Indirect Certainty	NMA estimates	Prop	Initial NMA Certainty	D6	D7	NMA Certainty
**All-cause death**	DPP-4 inhibitors *vs* Sulfonylureas	17	0.88 (0.76 to 1.01)	-1	0	0	0	Moderate	1.06 (0.82 to 1.37)	Moderate	0	Moderate	0.92 (0.81 to 1.04)	0.76	Moderate	0	-1	Low
**All-cause death**	DPP-4 inhibitors *vs* Thiazolidinediones	4	2.13 (0.43 to 10.59)	-1	0	0	0	Moderate	1.06 (0.91 to 1.23)	Moderate	0	Moderate	1.06 (0.92 to 1.23)	0.0082	Moderate	0	-2	Very low
**All-cause death**	DPP-4 inhibitors *vs* Tirzepatide	0	NA	NA	NA	NA	NA	NA	1.22 (0.70 to 2.11)	Moderate	0	Moderate	1.22 (0.70 to 2.11)	0	Moderate	NA	-2	Very low
**All-cause death**	GLP-1 receptor agonists *vs* Meglitinides	0	NA	NA	NA	NA	NA	NA	0.55 (0.18 to 1.73)	Moderate	0	Moderate	0.55 (0.18 to 1.73)	0	Moderate	NA	-2	Very low
**All-cause death**	GLP-1 receptor agonists *vs* Metformin	1	0.33 (0.01 to 8.12)	0	0	0	0	High	1.06 (0.84 to 1.32)	Moderate	0	Moderate	1.05 (0.84 to 1.31)	0.0049	Moderate	0	-2	Very low
**All-cause death**	GLP-1 receptor agonists *vs* Non-steroidal MRAs	0	NA	NA	NA	NA	NA	NA	0.99 (0.86 to 1.13)	Moderate	0	Moderate	0.99 (0.86 to 1.13)	0	Moderate	NA	-2	Very low
**All-cause death**	GLP-1 receptor agonists *vs* SGLT2 inhibitors	3	1.30 (0.25 to 6.78)	0	0	0	0	High	0.99 (0.91 to 1.08)	Moderate	0	Moderate	0.99 (0.91 to 1.08)	0.0029	Moderate	0	-2	Very low
**All-cause death**	GLP-1 receptor agonists *vs* Standard treatments	41	0.88 (0.83 to 0.94)	0	0	0	0	High	0.76 (0.55 to 1.04)	Moderate	0	Moderate	0.88 (0.82 to 0.93)	0.96	High	0	0	High
**All-cause death**	GLP-1 receptor agonists *vs* Sulfonylureas	6	0.68 (0.44 to 1.04)	-1	0	0	0	Moderate	0.81 (0.70 to 0.94)	Moderate	0	Moderate	0.79 (0.69 to 0.92)	0.11	Moderate	0	0	Moderate
**All-cause death**	GLP-1 receptor agonists *vs* Thiazolidinediones	3	0.37 (0.05 to 2.78)	-1	0	0	0	Moderate	0.93 (0.80 to 1.07)	Moderate	0	Moderate	0.92 (0.80 to 1.07)	0.0052	Moderate	0	-1	Low
**All-cause death**	GLP-1 receptor agonists *vs* Tirzepatide	2	0.40 (0.07 to 2.24)	0	0	0	0	High	1.17 (0.66 to 2.09)	Moderate	0	Moderate	1.05 (0.61 to 1.82)	0.1	Moderate	0	-2	Very low
**All-cause death**	Meglitinides *vs* Metformin	1	0.33 (0.01 to 8.15)	-2	0	0	0	Low	2.45 (0.72 to 8.38)	Low	0	Low	1.89 (0.60 to 5.97)	0.13	Low	0	-2	Very low

Outcome	Comparisons	k	Direct estimates	D1	D2	D3	D4	Direct Certainty	Indirect estimates	Initial Indirect Certainty	D5	Indirect Certainty	NMA estimates	Prop	Initial NMA Certainty	D6	D7	NMA Certainty
**All-cause death**	Meglitinides *vs* Non-steroidal MRAs	0	NA	NA	NA	NA	NA	NA	1.78 (0.57 to 5.57)	Low	0	Low	1.78 (0.57 to 5.57)	0	Low	NA	-2	Very low
**All-cause death**	Meglitinides *vs* SGLT2 inhibitors	0	NA	NA	NA	NA	NA	NA	1.79 (0.58 to 5.58)	Moderate	0	Moderate	1.79 (0.58 to 5.58)	0	Moderate	NA	-2	Very low
**All-cause death**	Meglitinides *vs* Standard treatments	2	1.72 (0.15 to 19.01)	-1	0	0	0	Moderate	1.55 (0.43 to 5.59)	Low	0	Low	1.58 (0.51 to 4.92)	0.22	Low	0	-2	Very low
**All-cause death**	Meglitinides *vs* Sulfonylureas	3	1.81 (0.39 to 8.46)	-2	0	0	0	Low	1.09 (0.21 to 5.79)	Low	0	Low	1.43 (0.46 to 4.45)	0.54	Low	0	-2	Very low
**All-cause death**	Meglitinides *vs* Thiazolidinediones	1	1.51 (0.06 to 37.15)	-2	0	0	0	Low	1.69 (0.50 to 5.70)	Low	0	Low	1.66 (0.53 to 5.19)	0.13	Low	0	-2	Very low
**All-cause death**	Meglitinides *vs* Tirzepatide	0	NA	NA	NA	NA	NA	NA	1.90 (0.54 to 6.68)	Moderate	0	Moderate	1.90 (0.54 to 6.68)	0	Moderate	NA	-2	Very low
**All-cause death**	Metformin *vs* Non-steroidal MRAs	0	NA	NA	NA	NA	NA	NA	0.94 (0.73 to 1.20)	Moderate	0	Moderate	0.94 (0.73 to 1.20)	0	Moderate	NA	-2	Very low
**All-cause death**	Metformin *vs* SGLT2 inhibitors	3	1.86 (0.29 to 11.83)	0	0	0	0	High	0.94 (0.75 to 1.18)	Moderate	0	Moderate	0.95 (0.76 to 1.19)	0.015	Moderate	0	-2	Very low
**All-cause death**	Metformin *vs* Standard treatments	5	0.77 (0.59 to 1.01)	-1	0	0	0	Moderate	0.96 (0.67 to 1.36)	Moderate	0	Moderate	0.84 (0.67 to 1.04)	0.63	Moderate	0	-1	Low
**All-cause death**	Metformin *vs* Sulfonylureas	5	0.89 (0.58 to 1.38)	0	0	0	0	High	0.70 (0.53 to 0.94)	Moderate	0	Moderate	0.76 (0.60 to 0.96)	0.3	Moderate	0	0	Moderate
**All-cause death**	Metformin *vs* Thiazolidinediones	8	0.94 (0.60 to 1.46)	-1	0	0	0	Moderate	0.85 (0.64 to 1.14)	Moderate	0	Moderate	0.88 (0.69 to 1.12)	0.29	Moderate	0	-2	Very low
**All-cause death**	Metformin *vs* Tirzepatide	0	NA	NA	NA	NA	NA	NA	1.00 (0.56 to 1.81)	Moderate	0	Moderate	1.00 (0.56 to 1.81)	0	Moderate	NA	-2	Very low

Outcome	Comparisons	k	Direct estimates	D1	D2	D3	D4	Direct Certainty	Indirect estimates	Initial Indirect Certainty	D5	Indirect Certainty	NMA estimates	Prop	Initial NMA Certainty	D6	D7	NMA Certainty
**All-cause death**	Non-steroidal MRAs *vs* SGLT2 inhibitors	0	NA	NA	NA	NA	NA	NA	1.01 (0.88 to 1.15)	Moderate	0	Moderate	1.01 (0.88 to 1.15)	0	Moderate	NA	-2	Very low
**All-cause death**	Non-steroidal MRAs *vs* Standard treatments	3	0.89 (0.79 to 1.00)	0	-1	0	0	Moderate	NA	NA	0	NA	0.89 (0.79 to 1.00)	1	Moderate	NA	0	Moderate
**All-cause death**	Non-steroidal MRAs *vs* Sulfonylureas	0	NA	NA	NA	NA	NA	NA	0.81 (0.67 to 0.96)	Moderate	0	Moderate	0.81 (0.67 to 0.96)	0	Moderate	NA	0	Moderate
**All-cause death**	Non-steroidal MRAs *vs* Thiazolidinediones	0	NA	NA	NA	NA	NA	NA	0.93 (0.78 to 1.12)	Moderate	0	Moderate	0.93 (0.78 to 1.12)	0	Moderate	NA	-2	Very low
**All-cause death**	Non-steroidal MRAs *vs* Tirzepatide	0	NA	NA	NA	NA	NA	NA	1.07 (0.61 to 1.87)	Moderate	0	Moderate	1.07 (0.61 to 1.87)	0	Moderate	NA	-2	Very low
**All-cause death**	SGLT2 inhibitors *vs* Standard treatments	39	0.88 (0.83 to 0.94)	0	0	0	0	High	1.00 (0.54 to 1.86)	Moderate	0	Moderate	0.88 (0.83 to 0.94)	0.99	High	0	0	High
**All-cause death**	SGLT2 inhibitors *vs* Sulfonylureas	5	0.78 (0.33 to 1.86)	0	0	0	0	High	0.80 (0.69 to 0.93)	Moderate	0	Moderate	0.80 (0.69 to 0.93)	0.029	Moderate	0	0	Moderate
**All-cause death**	SGLT2 inhibitors *vs* Thiazolidinediones	0	NA	NA	NA	NA	NA	NA	0.93 (0.80 to 1.08)	Moderate	0	Moderate	0.93 (0.80 to 1.08)	0	Moderate	NA	-2	Very low
**All-cause death**	SGLT2 inhibitors *vs* Tirzepatide	0	NA	NA	NA	NA	NA	NA	1.06 (0.61 to 1.84)	Moderate	0	Moderate	1.06 (0.61 to 1.84)	0	Moderate	NA	-2	Very low
**All-cause death**	Sulfonylureas *vs* Standard treatments	2	1.78 (0.29 to 11.00)	0	0	0	0	High	1.10 (0.96 to 1.26)	Moderate	0	Moderate	1.10 (0.97 to 1.26)	0.005 3	Moderate	0	-1	Low
**All-cause death**	Thiazolidinediones *vs* Standard treatments	19	0.91 (0.78 to 1.06)	-1	0	0	0	Moderate	1.10 (0.83 to 1.45)	Moderate	0	Moderate	0.95 (0.83 to 1.09)	0.77	Moderate	0	-2	Very low
**All-cause death**	Tirzepatide *vs* Standard treatments	2	0.28 (0.03 to 2.68)	0	0	0	0	High	0.89 (0.51 to 1.57)	Moderate	0	Moderate	0.83 (0.48 to 1.44)	0.058	Moderate	0	-2	Very low

Outcome	Comparisons	k	Direct estimates	D1	D2	D3	D4	Direct Certainty	Indirect estimates	Initial Indirect Certainty	D5	Indirect Certainty	NMA estimates	Prop	Initial NMA Certainty	D6	D7	NMA Certainty
**All-cause death**	Sulfonylureas *vs* Thiazolidinediones	11	0.97 (0.74 to 1.28)	-1	0	0	0	Moderate	1.29 (1.04 to 1.59)	Moderate	0	Moderate	1.16 (0.98 to 1.37)	0.37	Moderate	0	-1	Low
**All-cause death**	Sulfonylureas *vs* Tirzepatide	0	NA	NA	NA	NA	NA	NA	1.32 (0.76 to 2.31)	Moderate	0	Moderate	1.32 (0.76 to 2.31)	0	Moderate	NA	-2	Very low
**All-cause death**	Thiazolidinediones *vs* Tirzepatide	0	NA	NA	NA	NA	NA	NA	1.14 (0.65 to 2.01)	Moderate	0	Moderate	1.14 (0.65 to 2.01)	0	Moderate	NA	-2	Very low
**Cardiovascular death**	Alpha-glucosidase inhibitors *vs* Basal insulin	0	NA	NA	NA	NA	NA	NA	0.77 (0.15 to 3.88)	Moderate	0	Moderate	0.77 (0.15 to 3.88)	0	Moderate	NA	-2	Very low
**Cardiovascular death**	Alpha-glucosidase inhibitors *vs* Basal-Bolus insulin	0	NA	NA	NA	NA	NA	NA	0.45 (0.03 to 7.04)	Moderate	0	Moderate	0.45 (0.03 to 7.04)	0	Moderate	NA	-2	Very low
**Cardiovascular death**	Alpha-glucosidase inhibitors *vs* Bolus insulin	0	NA	NA	NA	NA	NA	NA	0.94 (0.06 to 14.87)	Moderate	0	Moderate	0.94 (0.06 to 14.87)	0	Moderate	NA	-2	Very low
**Cardiovascular death**	Alpha-glucosidase inhibitors *vs* DPP-4 inhibitors	0	NA	NA	NA	NA	NA	NA	0.99 (0.21 to 4.71)	Moderate	0	Moderate	0.99 (0.21 to 4.71)	0	Moderate	NA	-2	Very low
**Cardiovascular death**	Alpha-glucosidase inhibitors *vs* GLP-1 receptor agonists	0	NA	NA	NA	NA	NA	NA	1.14 (0.24 to 5.40)	Moderate	0	Moderate	1.14 (0.24 to 5.40)	0	Moderate	NA	-2	Very low
**Cardiovascular death**	Alpha-glucosidase inhibitors *vs* Meglitinides	0	NA	NA	NA	NA	NA	NA	1.55 (0.15 to 16.03)	Moderate	0	Moderate	1.55 (0.15 to 16.03)	0	Moderate	NA	-2	Very low
**Cardiovascular death**	Alpha-glucosidase inhibitors *vs* Metformin	0	NA	NA	NA	NA	NA	NA	1.05 (0.19 to 5.69)	Moderate	0	Moderate	1.05 (0.19 to 5.69)	0	Moderate	NA	-2	Very low
**Cardiovascular death**	Alpha-glucosidase inhibitors *vs* Non-steroidal MRAs	0	NA	NA	NA	NA	NA	NA	1.13 (0.24 to 5.40)	Moderate	0	Moderate	1.13 (0.24 to 5.40)	0	Moderate	NA	-2	Very low
**Cardiovascular death**	Alpha-glucosidase inhibitors *vs* SGLT2 inhibitors	0	NA	NA	NA	NA	NA	NA	1.15 (0.24 to 5.44)	Moderate	0	Moderate	1.15 (0.24 to 5.44)	0	Moderate	NA	-2	Very low

Outcome	Comparisons	k	Direct estimates	D1	D2	D3	D4	Direct Certainty	Indirect estimates	Initial Indirect Certainty	D5	Indirect Certainty	NMA estimates	Prop	Initial NMA Certainty	D6	D7	NMA Certainty
**Cardiovascular death**	Alpha-glucosidase inhibitors *vs* Standard treatments	3	1.54 (0.24 to 9.79)	-1	0	0	0	Moderate	0.35 (0.02 to 6.13)	Moderate	0	Moderate	0.99 (0.21 to 4.70)	0.7	Moderate	0	-2	Very low
**Cardiovascular death**	Alpha-glucosidase inhibitors *vs* Sulfonylureas	1	0.51 (0.05 to 4.97)	0	0	0	0	High	1.75 (0.21 to 14.79)	Moderate	0	Moderate	0.98 (0.21 to 4.67)	0.47	Moderate	0	-2	Very low
**Cardiovascular death**	Alpha-glucosidase inhibitors *vs* Thiazolidinediones	0	NA	NA	NA	NA	NA	NA	1.07 (0.22 to 5.12)	Moderate	0	Moderate	1.07 (0.22 to 5.12)	0	Moderate	NA	-2	Very low
**Cardiovascular death**	Alpha-glucosidase inhibitors *vs* Tirzepatide	0	NA	NA	NA	NA	NA	NA	1.00 (0.15 to 6.50)	Moderate	0	Moderate	1.00 (0.15 to 6.50)	0	Moderate	NA	-2	Very low
**Cardiovascular death**	Basal insulin *vs* Basal-Bolus insulin	0	NA	NA	NA	NA	NA	NA	0.58 (0.06 to 5.88)	Moderate	0	Moderate	0.58 (0.06 to 5.88)	0	Moderate	NA	-2	Very low
**Cardiovascular death**	Basal insulin *vs* Bolus insulin	1	0.34 (0.01 to 8.36)	-2	0	0	0	Low	4.50 (0.18 to 114.30)	Low	0	Low	1.22 (0.13 to 11.90)	0.5	Low	0	-2	Very low
**Cardiovascular death**	Basal insulin *vs* DPP-4 inhibitors	2	1.04 (0.57 to 1.90)	-1	0	0	0	Moderate	1.63 (0.86 to 3.11)	Moderate	0	Moderate	1.28 (0.83 to 1.99)	0.54	Moderate	0	-2	Very low
**Cardiovascular death**	Basal insulin *vs* GLP-1 receptor agonists	6	1.86 (1.04 to 3.31)	-1	0	0	0	Moderate	1.07 (0.54 to 2.11)	Moderate	0	Moderate	1.47 (0.95 to 2.29)	0.58	Moderate	0	-1	Low
**Cardiovascular death**	Basal insulin *vs* Meglitinides	0	NA	NA	NA	NA	NA	NA	2.00 (0.33 to 12.12)	Moderate	0	Moderate	2.00 (0.33 to 12.12)	0	Moderate	NA	-2	Very low
**Cardiovascular death**	Basal insulin *vs* Metformin	0	NA	NA	NA	NA	NA	NA	1.35 (0.61 to 3.03)	Moderate	0	Moderate	1.35 (0.61 to 3.03)	0	Moderate	NA	-2	Very low
**Cardiovascular death**	Basal insulin *vs* Non-steroidal MRAs	0	NA	NA	NA	NA	NA	NA	1.46 (0.92 to 2.33)	Moderate	0	Moderate	1.46 (0.92 to 2.33)	0	Moderate	NA	-1	Low
**Cardiovascular death**	Basal insulin *vs* SGLT2 inhibitors	0	NA	NA	NA	NA	NA	NA	1.48 (0.95 to 2.32)	Moderate	0	Moderate	1.48 (0.95 to 2.32)	0	Moderate	NA	-1	Low

Outcome	Comparisons	k	Direct estimates	D1	D2	D3	D4	Direct Certainty	Indirect estimates	Initial Indirect Certainty	D5	Indirect Certainty	NMA estimates	Prop	Initial NMA Certainty	D6	D7	NMA Certainty
**Cardiovascular death**	Basal insulin *vs* Standard treatments	0	NA	NA	NA	NA	NA	NA	1.28 (0.83 to 1.99)	Moderate	0	Moderate	1.28 (0.83 to 1.99)	0	Moderate	NA	-2	Very low
**Cardiovascular death**	Basal insulin *vs* Sulfonylureas	1	1.31 (0.68 to 2.52)	0	0	0	0	High	1.24 (0.65 to 2.35)	Moderate	0	Moderate	1.27 (0.80 to 2.01)	0.49	Moderate	0	-2	Very low
**Cardiovascular death**	Basal insulin *vs* Thiazolidinediones	0	NA	NA	NA	NA	NA	NA	1.38 (0.86 to 2.22)	Moderate	0	Moderate	1.38 (0.86 to 2.22)	0	Moderate	NA	-2	Very low
**Cardiovascular death**	Basal insulin *vs* Tirzepatide	1	1.50 (0.53 to 4.22)	0	0	0	0	High	0.39 (0.02 to 7.30)	Moderate	0	Moderate	1.29 (0.48 to 3.43)	0.89	High	0	-2	Low
**Cardiovascular death**	Basal-Bolus insulin *vs* Bolus insulin	0	NA	NA	NA	NA	NA	NA	2.12 (0.08 to 53.34)	Low	0	Low	2.12 (0.08 to 53.34)	0	Low	NA	-2	Very low
**Cardiovascular death**	Basal-Bolus insulin *vs* DPP-4 inhibitors	0	NA	NA	NA	NA	NA	NA	2.23 (0.23 to 21.94)	Moderate	0	Moderate	2.23 (0.23 to 21.94)	0	Moderate	NA	-2	Very low
**Cardiovascular death**	Basal-Bolus insulin *vs* GLP-1 receptor agonists	1	1.51 (0.06 to 37.31)	-1	0	0	0	Moderate	4.41 (0.17 to 114.35)	Moderate	0	Moderate	2.56 (0.26 to 25.15)	0.51	Moderate	0	-2	Very low
**Cardiovascular death**	Basal-Bolus insulin *vs* Meglitinides	0	NA	NA	NA	NA	NA	NA	3.48 (0.20 to 61.62)	Low	0	Low	3.48 (0.20 to 61.62)	0	Low	NA	-2	Very low
**Cardiovascular death**	Basal-Bolus insulin *vs* Metformin	0	NA	NA	NA	NA	NA	NA	2.35 (0.22 to 25.43)	Low	0	Low	2.35 (0.22 to 25.43)	0	Low	NA	-2	Very low
**Cardiovascular death**	Basal-Bolus insulin *vs* Non-steroidal MRAs	0	NA	NA	NA	NA	NA	NA	2.55 (0.26 to 25.14)	Moderate	0	Moderate	2.55 (0.26 to 25.14)	0	Moderate	NA	-2	Very low
**Cardiovascular death**	Basal-Bolus insulin *vs* SGLT2 inhibitors	0	NA	NA	NA	NA	NA	NA	2.58 (0.26 to 25.38)	Moderate	0	Moderate	2.58 (0.26 to 25.38)	0	Moderate	NA	-2	Very low
**Cardiovascular death**	Basal-Bolus insulin *vs* Standard treatments	0	NA	NA	NA	NA	NA	NA	2.23 (0.23 to 21.92)	Moderate	0	Moderate	2.23 (0.23 to 21.92)	0	Moderate	NA	-2	Very low

Outcome	Comparisons	k	Direct estimates	D1	D2	D3	D4	Direct Certainty	Indirect estimates	Initial Indirect Certainty	D5	Indirect Certainty	NMA estimates	Prop	Initial NMA Certainty	D6	D7	NMA Certainty
**Cardiovascular death**	Basal-Bolus insulin *vs* Sulfonylureas	1	3.80 (0.15 to 97.81)	-2	0	0	0	Low	1.30 (0.05 to 32.37)	Moderate	0	Moderate	2.21 (0.22 to 21.71)	0.49	Moderate	0	-2	Very low
**Cardiovascular death**	Basal-Bolus insulin *vs* Thiazolidinediones	0	NA	NA	NA	NA	NA	NA	2.40 (0.24 to 23.79)	Moderate	0	Moderate	2.40 (0.24 to 23.79)	0	Moderate	NA	-2	Very low
**Cardiovascular death**	Basal-Bolus insulin *vs* Tirzepatide	0	NA	NA	NA	NA	NA	NA	2.24 (0.18 to 27.63)	Moderate	0	Moderate	2.24 (0.18 to 27.63)	0	Moderate	NA	-2	Very low
**Cardiovascular death**	Bolus insulin *vs* DPP-4 inhibitors	0	NA	NA	NA	NA	NA	NA	1.05 (0.11 to 10.27)	Low	0	Low	1.05 (0.11 to 10.27)	0	Low	NA	-2	Very low
**Cardiovascular death**	Bolus insulin *vs* GLP-1 receptor agonists	1	0.34 (0.01 to 8.27)	-2	0	0	0	Low	4.45 (0.18 to 113.24)	Low	0	Low	1.21 (0.12 to 11.76)	0.5	Low	0	-2	Very low
**Cardiovascular death**	Bolus insulin *vs* Meglitinides	0	NA	NA	NA	NA	NA	NA	1.64 (0.09 to 28.95)	Low	0	Low	1.64 (0.09 to 28.95)	0	Low	NA	-2	Very low
**Cardiovascular death**	Bolus insulin *vs* Metformin	0	NA	NA	NA	NA	NA	NA	1.11 (0.10 to 11.94)	Moderate	0	Moderate	1.11 (0.10 to 11.94)	0	Moderate	NA	-2	Very low
**Cardiovascular death**	Bolus insulin *vs* Non-steroidal MRAs	0	NA	NA	NA	NA	NA	NA	1.20 (0.12 to 11.76)	Low	0	Low	1.20 (0.12 to 11.76)	0	Low	NA	-2	Very low
**Cardiovascular death**	Bolus insulin *vs* SGLT2 inhibitors	0	NA	NA	NA	NA	NA	NA	1.22 (0.12 to 11.88)	Moderate	0	Moderate	1.22 (0.12 to 11.88)	0	Moderate	NA	-2	Very low
**Cardiovascular death**	Bolus insulin *vs* Standard treatments	0	NA	NA	NA	NA	NA	NA	1.05 (0.11 to 10.26)	Low	0	Low	1.05 (0.11 to 10.26)	0	Low	NA	-2	Very low
**Cardiovascular death**	Bolus insulin *vs* Sulfonylureas	0	NA	NA	NA	NA	NA	NA	1.04 (0.11 to 10.22)	Moderate	0	Moderate	1.04 (0.11 to 10.22)	0	Moderate	NA	-2	Very low
**Cardiovascular death**	Bolus insulin *vs* Thiazolidinediones	0	NA	NA	NA	NA	NA	NA	1.13 (0.12 to 11.14)	Low	0	Low	1.13 (0.12 to 11.14)	0	Low	NA	-2	Very low

Outcome	Comparisons	k	Direct estimates	D1	D2	D3	D4	Direct Certainty	Indirect estimates	Initial Indirect Certainty	D5	Indirect Certainty	NMA estimates	Prop	Initial NMA Certainty	D6	D7	NMA Certainty
**Cardiovascular death**	Bolus insulin *vs* Tirzepatide	0	NA	NA	NA	NA	NA	NA	1.06 (0.09 to 12.53)	Moderate	0	Moderate	1.06 (0.09 to 12.53)	0	Moderate	NA	-2	Very low
**Cardiovascular death**	DPP-4 inhibitors *vs* GLP-1 receptor agonists	8	1.94 (1.03 to 3.65)	-1	0	0	0	Moderate	1.13 (1.01 to 1.26)	Moderate	0	Moderate	1.15 (1.02 to 1.28)	0.031	Moderate	0	0	Moderate
**Cardiovascular death**	DPP-4 inhibitors *vs* Meglitinides	0	NA	NA	NA	NA	NA	NA	1.56 (0.27 to 8.97)	Moderate	0	Moderate	1.56 (0.27 to 8.97)	0	Moderate	NA	-2	Very low
**Cardiovascular death**	DPP-4 inhibitors *vs* Metformin	1	2.98 (0.12 to 73.73)	-1	0	0	0	Moderate	1.00 (0.50 to 2.02)	Moderate	0	Moderate	1.05 (0.53 to 2.09)	0.045	Moderate	0	-2	Very low
**Cardiovascular death**	DPP-4 inhibitors *vs* Non-steroidal MRAs	0	NA	NA	NA	NA	NA	NA	1.14 (0.96 to 1.36)	Moderate	0	Moderate	1.14 (0.96 to 1.36)	0	Moderate	NA	-1	Low
**Cardiovascular death**	DPP-4 inhibitors *vs* SGLT2 inhibitors	3	0.29 (0.05 to 1.72)	0	0	0	0	High	1.16 (1.03 to 1.31)	Moderate	0	Moderate	1.16 (1.03 to 1.30)	0.0043	Moderate	0	0	Moderate
**Cardiovascular death**	DPP-4 inhibitors *vs* Standard treatments	29	0.99 (0.91 to 1.08)	-1	0	0	0	Moderate	1.16 (0.78 to 1.72)	Moderate	0	Moderate	1.00 (0.92 to 1.09)	0.95	Moderate	0	0	Moderate
**Cardiovascular death**	DPP-4 inhibitors *vs* Sulfonylureas	10	1.00 (0.82 to 1.22)	0	0	0	0	High	0.93 (0.55 to 1.57)	Moderate	0	Moderate	0.99 (0.82 to 1.19)	0.87	High	0	-2	Low
**Cardiovascular death**	DPP-4 inhibitors *vs* Thiazolidinediones	2	5.24 (0.54 to 50.59)	-1	0	0	0	Moderate	1.06 (0.87 to 1.30)	Moderate	0	Moderate	1.08 (0.88 to 1.32)	0.0079	Moderate	0	-2	Very low
**Cardiovascular death**	DPP-4 inhibitors *vs* Tirzepatide	0	NA	NA	NA	NA	NA	NA	1.00 (0.35 to 2.87)	Moderate	0	Moderate	1.00 (0.35 to 2.87)	0	Moderate	NA	-2	Very low
**Cardiovascular death**	GLP-1 receptor agonists *vs* Meglitinides	0	NA	NA	NA	NA	NA	NA	1.36 (0.24 to 7.83)	Moderate	0	Moderate	1.36 (0.24 to 7.83)	0	Moderate	NA	-2	Very low
**Cardiovascular death**	GLP-1 receptor agonists *vs* Metformin	0	NA	NA	NA	NA	NA	NA	0.92 (0.46 to 1.83)	Moderate	0	Moderate	0.92 (0.46 to 1.83)	0	Moderate	NA	-2	Very low

Outcome	Comparisons	k	Direct estimates	D1	D2	D3	D4	Direct Certainty	Indirect estimates	Initial Indirect Certainty	D5	Indirect Certainty	NMA estimates	Prop	Initial NMA Certainty	D6	D7	NMA Certainty
**Cardiovascular death**	GLP-1 receptor agonists *vs* Non-steroidal MRAs	0	NA	NA	NA	NA	NA	NA	0.99 (0.84 to 1.18)	Moderate	0	Moderate	0.99 (0.84 to 1.18)	0	Moderate	NA	-2	Very low
**Cardiovascular death**	GLP-1 receptor agonists *vs* SGLT2 inhibitors	0	NA	NA	NA	NA	NA	NA	1.01 (0.90 to 1.13)	Moderate	0	Moderate	1.01 (0.90 to 1.13)	0	Moderate	NA	-1	Low
**Cardiovascular death**	GLP-1 receptor agonists *vs* Standard treatments	24	0.88 (0.81 to 0.95)	0	0	0	0	High	0.62 (0.36 to 1.07)	Moderate	0	Moderate	0.87 (0.81 to 0.94)	0.98	High	0	0	High
**Cardiovascular death**	GLP-1 receptor agonists *vs* Sulfonylureas	1	0.56 (0.25 to 1.27)	0	0	0	0	High	0.89 (0.71 to 1.11)	Moderate	0	Moderate	0.86 (0.70 to 1.07)	0.067	Moderate	0	-1	Low
**Cardiovascular death**	GLP-1 receptor agonists *vs* Thiazolidinediones	1	1.03 (0.02 to 52.28)	-1	0	0	0	Moderate	0.94 (0.77 to 1.15)	Moderate	0	Moderate	0.94 (0.77 to 1.15)	0.0026	Moderate	0	-2	Very low
**Cardiovascular death**	GLP-1 receptor agonists *vs* Tirzepatide	1	0.27 (0.02 to 4.93)	0	0	0	0	High	1.04 (0.34 to 3.23)	Moderate	0	Moderate	0.88 (0.31 to 2.50)	0.13	Moderate	0	-2	Very low
**Cardiovascular death**	Meglitinides *vs* Metformin	1	0.33 (0.01 to 8.15)	-2	0	0	0	Low	0.95 (0.11 to 8.56)	Low	0	Low	0.68 (0.11 to 4.15)	0.32	Low	0	-2	Very low
**Cardiovascular death**	Meglitinides *vs* Non-steroidal MRAs	0	NA	NA	NA	NA	NA	NA	0.73 (0.13 to 4.24)	Low	0	Low	0.73 (0.13 to 4.24)	0	Low	NA	-2	Very low
**Cardiovascular death**	Meglitinides *vs* SGLT2 inhibitors	0	NA	NA	NA	NA	NA	NA	0.74 (0.13 to 4.28)	Moderate	0	Moderate	0.74 (0.13 to 4.28)	0	Moderate	NA	-2	Very low
**Cardiovascular death**	Meglitinides *vs* Standard treatments	1	0.96 (0.02 to 48.70)	-2	0	0	0	Low	0.58 (0.08 to 4.10)	Low	0	Low	0.64 (0.11 to 3.69)	0.2	Low	0	-2	Very low
**Cardiovascular death**	Meglitinides *vs* Sulfonylureas	1	0.50 (0.03 to 8.08)	-1	0	0	0	Moderate	0.74 (0.08 to 7.05)	Low	0	Low	0.64 (0.11 to 3.65)	0.4	Low	0	-2	Very low
**Cardiovascular death**	Meglitinides *vs* Thiazolidinediones	1	1.51 (0.06 to 37.15)	-2	0	0	0	Low	0.50 (0.06 to 4.03)	Low	0	Low	0.69 (0.12 to 3.99)	0.3	Low	0	-2	Very low

Outcome	Comparisons	k	Direct estimates	D1	D2	D3	D4	Direct Certainty	Indirect estimates	Initial Indirect Certainty	D5	Indirect Certainty	NMA estimates	Prop	Initial NMA Certainty	D6	D7	NMA Certainty
**Cardiovascular death**	Meglitinides *vs* Tirzepatide	0	NA	NA	NA	NA	NA	NA	0.64 (0.08 to 4.95)	Moderate	0	Moderate	0.64 (0.08 to 4.95)	0	Moderate	NA	-2	Very low
**Cardiovascular death**	Metformin *vs* Non-steroidal MRAs	0	NA	NA	NA	NA	NA	NA	1.08 (0.54 to 2.18)	Moderate	0	Moderate	1.08 (0.54 to 2.18)	0	Moderate	NA	-2	Very low
**Cardiovascular death**	Metformin *vs* SGLT2 inhibitors	3	1.86 (0.29 to 11.83)	0	0	0	0	High	1.01 (0.48 to 2.11)	Moderate	0	Moderate	1.10 (0.55 to 2.18)	0.14	Moderate	0	-2	Very low
**Cardiovascular death**	Metformin *vs* Standard treatments	2	2.97 (0.47 to 18.97)	-2	0	0	0	Low	0.79 (0.38 to 1.65)	Moderate	0	Moderate	0.95 (0.48 to 1.88)	0.14	Moderate	0	-2	Very low
**Cardiovascular death**	Metformin *vs* Sulfonylureas	3	0.76 (0.31 to 1.86)	-1	0	0	0	Moderate	1.25 (0.44 to 3.52)	Moderate	0	Moderate	0.94 (0.48 to 1.85)	0.57	Moderate	0	-2	Very low
**Cardiovascular death**	Metformin *vs* Thiazolidinediones	3	0.70 (0.11 to 4.54)	-2	0	0	0	Low	1.09 (0.51 to 2.31)	Moderate	0	Moderate	1.02 (0.51 to 2.05)	0.14	Moderate	0	-2	Very low
**Cardiovascular death**	Metformin *vs* Tirzepatide	0	NA	NA	NA	NA	NA	NA	0.95 (0.27 to 3.32)	Moderate	0	Moderate	0.95 (0.27 to 3.32)	0	Moderate	NA	-2	Very low
**Cardiovascular death**	Non-steroidal MRAs *vs* SGLT2 inhibitors	0	NA	NA	NA	NA	NA	NA	1.01 (0.85 to 1.21)	Moderate	0	Moderate	1.01 (0.85 to 1.21)	0	Moderate	NA	-2	Very low
**Cardiovascular death**	Non-steroidal MRAs *vs* Standard treatments	2	0.88 (0.75 to 1.02)	0	-1	0	0	Moderate	NA	NA	0	NA	0.88 (0.75 to 1.02)	1	Moderate	NA	-1	Low
**Cardiovascular death**	Non-steroidal MRAs *vs* Sulfonylureas	0	NA	NA	NA	NA	NA	NA	0.87 (0.67 to 1.12)	Moderate	0	Moderate	0.87 (0.67 to 1.12)	0	Moderate	NA	-2	Very low
**Cardiovascular death**	Non-steroidal MRAs *vs* Thiazolidinediones	0	NA	NA	NA	NA	NA	NA	0.94 (0.74 to 1.20)	Moderate	0	Moderate	0.94 (0.74 to 1.20)	0	Moderate	NA	-2	Very low
**Cardiovascular death**	Non-steroidal MRAs *vs* Tirzepatide	0	NA	NA	NA	NA	NA	NA	0.88 (0.30 to 2.55)	Moderate	0	Moderate	0.88 (0.30 to 2.55)	0	Moderate	NA	-2	Very low

Outcome	Comparisons	k	Direct estimates	D1	D2	D3	D4	Direct Certainty	Indirect estimates	Initial Indirect Certainty	D5	Indirect Certainty	NMA estimates	Prop	Initial NMA Certainty	D6	D7	NMA Certainty
**Cardiovascular death**	SGLT2 inhibitors *vs* Standard treatments	19	0.86 (0.80 to 0.94)	0	0	0	0	High	1.36 (0.44 to 4.20)	Moderate	0	Moderate	0.86 (0.80 to 0.94)	0.99	High	0	0	High
**Cardiovascular death**	SGLT2 inhibitors *vs* Sulfonylureas	2	1.19 (0.15 to 9.68)	0	0	0	0	High	0.85 (0.69 to 1.06)	Moderate	0	Moderate	0.86 (0.69 to 1.06)	0.011	Moderate	0	-1	Low
**Cardiovascular death**	SGLT2 inhibitors *vs* Thiazolidinediones	0	NA	NA	NA	NA	NA	NA	0.93 (0.76 to 1.14)	Moderate	0	Moderate	0.93 (0.76 to 1.14)	0	Moderate	NA	-2	Very low
**Cardiovascular death**	SGLT2 inhibitors *vs* Tirzepatide	0	NA	NA	NA	NA	NA	NA	0.87 (0.30 to 2.49)	Moderate	0	Moderate	0.87 (0.30 to 2.49)	0	Moderate	NA	-2	Very low
**Cardiovascular death**	Sulfonylureas *vs* Standard treatments	1	3.03 (0.12 to 75.42)	0	0	0	0	High	1.01 (0.82 to 1.23)	Moderate	0	Moderate	1.01 (0.83 to 1.23)	0.0039	Moderate	0	-2	Very low
**Cardiovascular death**	Thiazolidinediones *vs* Standard treatments	14	0.91 (0.75 to 1.11)	-1	0	0	0	Moderate	1.07 (0.58 to 1.97)	Moderate	0	Moderate	0.93 (0.77 to 1.12)	0.91	Moderate	0	-2	Very low
**Cardiovascular death**	Tirzepatide *vs* Standard treatments	0	NA	NA	NA	NA	NA	NA	1.00 (0.35 to 2.85)	Moderate	0	Moderate	1.00 (0.35 to 2.85)	0	Moderate	NA	-2	Very low
**Cardiovascular death**	Sulfonylureas *vs* Thiazolidinediones	8	0.85 (0.45 to 1.59)	-1	0	0	0	Moderate	1.14 (0.86 to 1.52)	Moderate	0	Moderate	1.09 (0.84 to 1.41)	0.17	Moderate	0	-2	Very low
**Cardiovascular death**	Sulfonylureas *vs* Tirzepatide	0	NA	NA	NA	NA	NA	NA	1.01 (0.35 to 2.93)	Moderate	0	Moderate	1.01 (0.35 to 2.93)	0	Moderate	NA	-2	Very low
**Cardiovascular death**	Thiazolidinediones *vs* Tirzepatide	0	NA	NA	NA	NA	NA	NA	0.93 (0.32 to 2.71)	Moderate	0	Moderate	0.93 (0.32 to 2.71)	0	Moderate	NA	-2	Very low
**Non-fatal MI**	Alpha-glucosidase inhibitors *vs* Basal insulin	0	NA	NA	NA	NA	NA	NA	0.33 (0.05 to 2.28)	Moderate	0	Moderate	0.33 (0.05 to 2.28)	0	Moderate	NA	-2	Very low
**Non-fatal MI**	Alpha-glucosidase inhibitors *vs* Basal-Bolus insulin	0	NA	NA	NA	NA	NA	NA	1.00 (0.05 to 18.12)	Low	0	Low	1.00 (0.05 to 18.12)	0	Low	NA	-2	Very low

Outcome	Comparisons	k	Direct estimates	D1	D2	D3	D4	Direct Certainty	Indirect estimates	Initial Indirect Certainty	D5	Indirect Certainty	NMA estimates	Prop	Initial NMA Certainty	D6	D7	NMA Certainty
**Non-fatal MI**	Alpha-glucosidase inhibitors *vs* Bolus insulin	0	NA	NA	NA	NA	NA	NA	0.28 (0.03 to 2.21)	Low	0	Low	0.28 (0.03 to 2.21)	0	Low	NA	-2	Very low
**Non-fatal MI**	Alpha-glucosidase inhibitors *vs* DPP-4 inhibitors	0	NA	NA	NA	NA	NA	NA	0.32 (0.06 to 1.90)	Low	0	Low	0.32 (0.06 to 1.90)	0	Low	NA	-2	Very low
**Non-fatal MI**	Alpha-glucosidase inhibitors *vs* GLP-1 receptor agonists	0	NA	NA	NA	NA	NA	NA	0.36 (0.06 to 2.10)	Moderate	0	Moderate	0.36 (0.06 to 2.10)	0	Moderate	NA	-2	Very low
**Non-fatal MI**	Alpha-glucosidase inhibitors *vs* Meglitinides	1	0.99 (0.02 to 49.94)	-2	0	0	0	Low	1.28 (0.06 to 26.70)	Low	0	Low	1.16 (0.10 to 12.83)	0.38	Low	0	-2	Very low
**Non-fatal MI**	Alpha-glucosidase inhibitors *vs* Metformin	0	NA	NA	NA	NA	NA	NA	0.38 (0.06 to 2.27)	Moderate	0	Moderate	0.38 (0.06 to 2.27)	0	Moderate	NA	-2	Very low
**Non-fatal MI**	Alpha-glucosidase inhibitors *vs* Non-steroidal MRAs	0	NA	NA	NA	NA	NA	NA	0.36 (0.06 to 2.13)	Low	0	Low	0.36 (0.06 to 2.13)	0	Low	NA	-2	Very low
**Non-fatal MI**	Alpha-glucosidase inhibitors *vs* SGLT2 inhibitors	0	NA	NA	NA	NA	NA	NA	0.36 (0.06 to 2.14)	Moderate	0	Moderate	0.36 (0.06 to 2.14)	0	Moderate	NA	-2	Very low
**Non-fatal MI**	Alpha-glucosidase inhibitors *vs* Standard treatments	2	0.30 (0.03 to 2.70)	-1	0	0	0	Moderate	0.38 (0.02 to 7.68)	Low	0	Low	0.33 (0.06 to 1.92)	0.66	Moderate	0	-2	Very low
**Non-fatal MI**	Alpha-glucosidase inhibitors *vs* Sulfonylureas	1	0.40 (0.02 to 8.30)	-2	0	0	0	Low	0.29 (0.03 to 2.61)	Moderate	0	Moderate	0.33 (0.06 to 1.92)	0.34	Moderate	0	-2	Very low
**Non-fatal MI**	Alpha-glucosidase inhibitors *vs* Thiazolidinediones	0	NA	NA	NA	NA	NA	NA	0.34 (0.06 to 2.00)	Low	0	Low	0.34 (0.06 to 2.00)	0	Low	NA	-2	Very low
**Non-fatal MI**	Alpha-glucosidase inhibitors *vs* Tirzepatide	0	NA	NA	NA	NA	NA	NA	0.48 (0.03 to 7.88)	Moderate	0	Moderate	0.48 (0.03 to 7.88)	0	Moderate	NA	-2	Very low
**Non-fatal MI**	Basal insulin *vs* Basal-Bolus insulin	1	2.92 (0.12 to 72.15)	-2	0	0	0	Low	3.05 (0.11 to 82.04)	Low	0	Low	2.98 (0.30 to 29.67)	0.51	Low	0	-2	Very low

Outcome	Comparisons	k	Direct estimates	D1	D2	D3	D4	Direct Certainty	Indirect estimates	Initial Indirect Certainty	D5	Indirect Certainty	NMA estimates	Prop	Initial NMA Certainty	D6	D7	NMA Certainty
**Non-fatal MI**	Basal insulin *vs* Bolus insulin	1	0.76 (0.17 to 3.45)	-2	0	0	0	Low	0.91 (0.18 to 4.58)	Low	0	Low	0.83 (0.28 to 2.50)	0.53	Low	0	-2	Very low
**Non-fatal MI**	Basal insulin *vs* DPP-4 inhibitors	1	0.37 (0.01 to 9.12)	0	0	0	0	High	1.02 (0.48 to 2.21)	Moderate	0	Moderate	0.97 (0.46 to 2.05)	0.054	Moderate	0	-2	Very low
**Non-fatal MI**	Basal insulin *vs* GLP-1 receptor agonists	7	1.19 (0.49 to 2.91)	-1	0	0	0	Moderate	0.85 (0.23 to 3.18)	Low	0	Low	1.07 (0.51 to 2.25)	0.69	Moderate	0	-2	Very low
**Non-fatal MI**	Basal insulin *vs* Meglitinides	0	NA	NA	NA	NA	NA	NA	3.47 (0.53 to 22.89)	Moderate	0	Moderate	3.47 (0.53 to 22.89)	0	Moderate	NA	-2	Very low
**Non-fatal MI**	Basal insulin *vs* Metformin	0	NA	NA	NA	NA	NA	NA	1.14 (0.52 to 2.49)	Moderate	0	Moderate	1.14 (0.52 to 2.49)	0	Moderate	NA	-2	Very low
**Non-fatal MI**	Basal insulin *vs* Non-steroidal MRAs	0	NA	NA	NA	NA	NA	NA	1.08 (0.50 to 2.33)	Moderate	0	Moderate	1.08 (0.50 to 2.33)	0	Moderate	NA	-2	Very low
**Non-fatal MI**	Basal insulin *vs* SGLT2 inhibitors	0	NA	NA	NA	NA	NA	NA	1.09 (0.52 to 2.31)	Moderate	0	Moderate	1.09 (0.52 to 2.31)	0	Moderate	NA	-2	Very low
**Non-fatal MI**	Basal insulin *vs* Standard treatments	1	3.00 (0.12 to 74.19)	-1	0	0	0	Moderate	0.92 (0.43 to 1.97)	Moderate	0	Moderate	0.98 (0.47 to 2.06)	0.054	Moderate	0	-2	Very low
**Non-fatal MI**	Basal insulin *vs* Sulfonylureas	0	NA	NA	NA	NA	NA	NA	0.98 (0.45 to 2.10)	Moderate	0	Moderate	0.98 (0.45 to 2.10)	0	Moderate	NA	-2	Very low
**Non-fatal MI**	Basal insulin *vs* Thiazolidinediones	1	0.33 (0.01 to 8.21)	0	0	0	0	High	1.08 (0.50 to 2.37)	Moderate	0	Moderate	1.01 (0.47 to 2.17)	0.056	Moderate	0	-2	Very low
**Non-fatal MI**	Basal insulin *vs* Tirzepatide	0	NA	NA	NA	NA	NA	NA	1.42 (0.14 to 14.22)	Moderate	0	Moderate	1.42 (0.14 to 14.22)	0	Moderate	NA	-2	Very low
**Non-fatal MI**	Basal-Bolus insulin *vs* Bolus insulin	0	NA	NA	NA	NA	NA	NA	0.28 (0.02 to 3.35)	Low	0	Low	0.28 (0.02 to 3.35)	0	Low	NA	-2	Very low

Outcome	Comparisons	k	Direct estimates	D1	D2	D3	D4	Direct Certainty	Indirect estimates	Initial Indirect Certainty	D5	Indirect Certainty	NMA estimates	Prop	Initial NMA Certainty	D6	D7	NMA Certainty
**Non-fatal MI**	Basal-Bolus insulin *vs* DPP-4 inhibitors	0	NA	NA	NA	NA	NA	NA	0.32 (0.03 to 3.24)	Low	0	Low	0.32 (0.03 to 3.24)	0	Low	NA	-2	Very low
**Non-fatal MI**	Basal-Bolus insulin *vs* GLP-1 receptor agonists	1	0.35 (0.01 to 8.68)	-2	0	0	0	Low	0.37 (0.01 to 9.87)	Low	0	Low	0.36 (0.04 to 3.57)	0.51	Low	0	-2	Very low
**Non-fatal MI**	Basal-Bolus insulin *vs* Meglitinides	0	NA	NA	NA	NA	NA	NA	1.16 (0.07 to 20.69)	Low	0	Low	1.16 (0.07 to 20.69)	0	Low	NA	-2	Very low
**Non-fatal MI**	Basal-Bolus insulin *vs* Metformin	0	NA	NA	NA	NA	NA	NA	0.38 (0.04 to 3.85)	Moderate	0	Moderate	0.38 (0.04 to 3.85)	0	Moderate	NA	-2	Very low
**Non-fatal MI**	Basal-Bolus insulin *vs* Non-steroidal MRAs	0	NA	NA	NA	NA	NA	NA	0.36 (0.04 to 3.62)	Low	0	Low	0.36 (0.04 to 3.62)	0	Low	NA	-2	Very low
**Non-fatal MI**	Basal-Bolus insulin *vs* SGLT2 inhibitors	0	NA	NA	NA	NA	NA	NA	0.37 (0.04 to 3.65)	Moderate	0	Moderate	0.37 (0.04 to 3.65)	0	Moderate	NA	-2	Very low
**Non-fatal MI**	Basal-Bolus insulin *vs* Standard treatments	0	NA	NA	NA	NA	NA	NA	0.33 (0.03 to 3.27)	Low	0	Low	0.33 (0.03 to 3.27)	0	Low	NA	-2	Very low
**Non-fatal MI**	Basal-Bolus insulin *vs* Sulfonylureas	0	NA	NA	NA	NA	NA	NA	0.33 (0.03 to 3.28)	Moderate	0	Moderate	0.33 (0.03 to 3.28)	0	Moderate	NA	-2	Very low
**Non-fatal MI**	Basal-Bolus insulin *vs* Thiazolidinediones	0	NA	NA	NA	NA	NA	NA	0.34 (0.03 to 3.40)	Moderate	0	Moderate	0.34 (0.03 to 3.40)	0	Moderate	NA	-2	Very low
**Non-fatal MI**	Basal-Bolus insulin *vs* Tirzepatide	0	NA	NA	NA	NA	NA	NA	0.48 (0.02 to 11.31)	Moderate	0	Moderate	0.48 (0.02 to 11.31)	0	Moderate	NA	-2	Very low
**Non-fatal MI**	Bolus insulin *vs* DPP-4 inhibitors	0	NA	NA	NA	NA	NA	NA	1.17 (0.39 to 3.48)	Low	0	Low	1.17 (0.39 to 3.48)	0	Low	NA	-2	Very low
**Non-fatal MI**	Bolus insulin *vs* GLP-1 receptor agonists	4	1.20 (0.29 to 4.90)	-2	0	0	0	Low	1.44 (0.26 to 7.90)	Low	0	Low	1.29 (0.44 to 3.82)	0.6	Low	0	-2	Very low

Outcome	Comparisons	k	Direct estimates	D1	D2	D3	D4	Direct Certainty	Indirect estimates	Initial Indirect Certainty	D5	Indirect Certainty	NMA estimates	Prop	Initial NMA Certainty	D6	D7	NMA Certainty
**Non-fatal MI**	Bolus insulin *vs* Meglitinides	0	NA	NA	NA	NA	NA	NA	4.19 (0.54 to 32.39)	Low	0	Low	4.19 (0.54 to 32.39)	0	Low	NA	-2	Very low
**Non-fatal MI**	Bolus insulin *vs* Metformin	0	NA	NA	NA	NA	NA	NA	1.37 (0.45 to 4.18)	Moderate	0	Moderate	1.37 (0.45 to 4.18)	0	Moderate	NA	-2	Very low
**Non-fatal MI**	Bolus insulin *vs* Non-steroidal MRAs	0	NA	NA	NA	NA	NA	NA	1.30 (0.43 to 3.92)	Low	0	Low	1.30 (0.43 to 3.92)	0	Low	NA	-2	Very low
**Non-fatal MI**	Bolus insulin *vs* SGLT2 inhibitors	0	NA	NA	NA	NA	NA	NA	1.32 (0.44 to 3.92)	Moderate	0	Moderate	1.32 (0.44 to 3.92)	0	Moderate	NA	-2	Very low
**Non-fatal MI**	Bolus insulin *vs* Standard treatments	0	NA	NA	NA	NA	NA	NA	1.18 (0.40 to 3.50)	Low	0	Low	1.18 (0.40 to 3.50)	0	Low	NA	-2	Very low
**Non-fatal MI**	Bolus insulin *vs* Sulfonylureas	0	NA	NA	NA	NA	NA	NA	1.18 (0.39 to 3.55)	Moderate	0	Moderate	1.18 (0.39 to 3.55)	0	Moderate	NA	-2	Very low
**Non-fatal MI**	Bolus insulin *vs* Thiazolidinediones	0	NA	NA	NA	NA	NA	NA	1.22 (0.41 to 3.67)	Moderate	0	Moderate	1.22 (0.41 to 3.67)	0	Moderate	NA	-2	Very low
**Non-fatal MI**	Bolus insulin *vs* Tirzepatide	0	NA	NA	NA	NA	NA	NA	1.72 (0.15 to 19.58)	Moderate	0	Moderate	1.72 (0.15 to 19.58)	0	Moderate	NA	-2	Very low
**Non-fatal MI**	DPP-4 inhibitors *vs* GLP-1 receptor agonists	10	1.10 (0.45 to 2.68)	-1	0	0	0	Moderate	1.11 (0.99 to 1.24)	Moderate	0	Moderate	1.11 (0.99 to 1.24)	0.016	Moderate	0	-1	Low
**Non-fatal MI**	DPP-4 inhibitors *vs* Meglitinides	0	NA	NA	NA	NA	NA	NA	3.58 (0.63 to 20.28)	Moderate	0	Moderate	3.58 (0.63 to 20.28)	0	Moderate	NA	-2	Very low
**Non-fatal MI**	DPP-4 inhibitors *vs* Metformin	5	0.48 (0.11 to 2.10)	0	0	0	0	High	1.21 (0.94 to 1.56)	Moderate	0	Moderate	1.18 (0.91 to 1.51)	0.029	Moderate	0	-1	Low
**Non-fatal MI**	DPP-4 inhibitors *vs* Non-steroidal MRAs	0	NA	NA	NA	NA	NA	NA	1.11 (0.88 to 1.39)	Moderate	0	Moderate	1.11 (0.88 to 1.39)	0	Moderate	NA	-2	Very low

Outcome	Comparisons	k	Direct estimates	D1	D2	D3	D4	Direct Certainty	Indirect estimates	Initial Indirect Certainty	D5	Indirect Certainty	NMA estimates	Prop	Initial NMA Certainty	D6	D7	NMA Certainty
**Non-fatal MI**	DPP-4 inhibitors *vs* SGLT2 inhibitors	8	1.09 (0.38 to 3.11)	0	0	0	0	High	1.13 (0.99 to 1.28)	Moderate	0	Moderate	1.13 (0.99 to 1.28)	0.014	Moderate	0	-1	Low
**Non-fatal MI**	DPP-4 inhibitors *vs* Standard treatments	45	1.02 (0.93 to 1.12)	-1	0	0	0	Moderate	0.89 (0.64 to 1.24)	Moderate	0	Moderate	1.01 (0.92 to 1.11)	0.93	Moderate	0	-1	Low
**Non-fatal MI**	DPP-4 inhibitors *vs* Sulfonylureas	12	0.96 (0.77 to 1.19)	0	0	0	0	High	1.13 (0.81 to 1.58)	Moderate	0	Moderate	1.01 (0.84 to 1.21)	0.69	High	0	-2	Low
**Non-fatal MI**	DPP-4 inhibitors *vs* Thiazolidinediones	5	1.01 (0.26 to 3.95)	-1	0	0	0	Moderate	1.05 (0.87 to 1.26)	Moderate	0	Moderate	1.04 (0.87 to 1.26)	0.019	Moderate	0	-2	Very low
**Non-fatal MI**	DPP-4 inhibitors *vs* Tirzepatide	0	NA	NA	NA	NA	NA	NA	1.47 (0.17 to 13.01)	Moderate	0	Moderate	1.47 (0.17 to 13.01)	0	Moderate	NA	-2	Very low
**Non-fatal MI**	GLP-1 receptor agonists *vs* Meglitinides	0	NA	NA	NA	NA	NA	NA	3.24 (0.57 to 18.37)	Moderate	0	Moderate	3.24 (0.57 to 18.37)	0	Moderate	NA	-2	Very low
**Non-fatal MI**	GLP-1 receptor agonists *vs* Metformin	2	1.27 (0.11 to 15.18)	0	0	0	0	High	1.06 (0.83 to 1.36)	Moderate	0	Moderate	1.06 (0.83 to 1.36)	0.01	Moderate	0	-2	Very low
**Non-fatal MI**	GLP-1 receptor agonists *vs* Non-steroidal MRAs	0	NA	NA	NA	NA	NA	NA	1.00 (0.80 to 1.25)	Moderate	0	Moderate	1.00 (0.80 to 1.25)	0	Moderate	NA	-2	Very low
**Non-fatal MI**	GLP-1 receptor agonists *vs* SGLT2 inhibitors	1	5.11 (0.24 to 107.01)	0	0	0	0	High	1.02 (0.91 to 1.14)	Moderate	0	Moderate	1.02 (0.91 to 1.14)	0.0014	Moderate	0	-1	Low
**Non-fatal MI**	GLP-1 receptor agonists *vs* Standard treatments	37	0.92 (0.85 to 0.98)	0	0	0	0	High	0.86 (0.44 to 1.66)	Moderate	0	Moderate	0.91 (0.85 to 0.98)	0.99	High	0	-1	Moderate
**Non-fatal MI**	GLP-1 receptor agonists *vs* Sulfonylureas	4	1.05 (0.36 to 3.05)	-1	0	0	0	Moderate	0.91 (0.74 to 1.11)	Moderate	0	Moderate	0.91 (0.74 to 1.12)	0.037	Moderate	0	-2	Very low
**Non-fatal MI**	GLP-1 receptor agonists *vs* Thiazolidinediones	2	0.21 (0.02 to 1.91)	-1	0	0	0	Moderate	0.96 (0.79 to 1.15)	Moderate	0	Moderate	0.95 (0.79 to 1.14)	0.0071	Moderate	0	-2	Very low

Outcome	Comparisons	k	Direct estimates	D1	D2	D3	D4	Direct Certainty	Indirect estimates	Initial Indirect Certainty	D5	Indirect Certainty	NMA estimates	Prop	Initial NMA Certainty	D6	D7	NMA Certainty
**Non-fatal MI**	GLP-1 receptor agonists *vs* Tirzepatide	2	0.73 (0.07 to 8.10)	0	0	0	0	High	20.18 (0.12 to 3422.85)	Moderate	0	Moderate	1.33 (0.15 to 11.74)	0.82	High	0	-2	Low
**Non-fatal MI**	Meglitinides *vs* Metformin	0	NA	NA	NA	NA	NA	NA	0.33 (0.06 to 1.89)	Moderate	0	Moderate	0.33 (0.06 to 1.89)	0	Moderate	NA	-2	Very low
**Non-fatal MI**	Meglitinides *vs* Non-steroidal MRAs	0	NA	NA	NA	NA	NA	NA	0.31 (0.05 to 1.78)	Low	0	Low	0.31 (0.05 to 1.78)	0	Low	NA	-2	Very low
**Non-fatal MI**	Meglitinides *vs* SGLT2 inhibitors	0	NA	NA	NA	NA	NA	NA	0.31 (0.06 to 1.79)	Moderate	0	Moderate	0.31 (0.06 to 1.79)	0	Moderate	NA	-2	Very low
**Non-fatal MI**	Meglitinides *vs* Standard treatments	1	1.33 (0.06 to 27.93)	-2	0	0	0	Low	0.13 (0.02 to 1.11)	Moderate	0	Moderate	0.28 (0.05 to 1.60)	0.32	Moderate	0	-2	Very low
**Non-fatal MI**	Meglitinides *vs* Sulfonylureas	2	0.14 (0.02 to 1.12)	-1	0	0	0	Moderate	1.26 (0.06 to 26.07)	Low	0	Low	0.28 (0.05 to 1.59)	0.67	Moderate	0	-2	Very low
**Non-fatal MI**	Meglitinides *vs* Thiazolidinediones	0	NA	NA	NA	NA	NA	NA	0.29 (0.05 to 1.66)	Low	0	Low	0.29 (0.05 to 1.66)	0	Low	NA	-2	Very low
**Non-fatal MI**	Meglitinides *vs* Tirzepatide	0	NA	NA	NA	NA	NA	NA	0.41 (0.03 to 6.64)	Moderate	0	Moderate	0.41 (0.03 to 6.64)	0	Moderate	NA	-2	Very low
**Non-fatal MI**	Metformin *vs* Non-steroidal MRAs	0	NA	NA	NA	NA	NA	NA	0.94 (0.69 to 1.30)	Moderate	0	Moderate	0.94 (0.69 to 1.30)	0	Moderate	NA	-2	Very low
**Non-fatal MI**	Metformin *vs* SGLT2 inhibitors	1	1.43 (0.06 to 35.52)	0	0	0	0	High	0.96 (0.74 to 1.23)	Moderate	0	Moderate	0.96 (0.74 to 1.24)	0.0063	Moderate	0	-2	Very low
**Non-fatal MI**	Metformin *vs* Standard treatments	5	0.80 (0.61 to 1.06)	-1	0	0	0	Moderate	1.03 (0.65 to 1.61)	Moderate	0	Moderate	0.86 (0.68 to 1.09)	0.72	Moderate	0	-1	Low
**Non-fatal MI**	Metformin *vs* Sulfonylureas	4	1.24 (0.71 to 2.17)	0	0	0	0	High	0.75 (0.54 to 1.05)	Moderate	0	Moderate	0.86 (0.64 to 1.14)	0.26	Moderate	0	-2	Very low

**Outcome**	**Comparisons**	**k**	**Direct estimates**	**D1**	**D2**	**D3**	**D4**	**Direct Certainty**	**Indirect estimates**	**Initial Indirect Certainty**	**D5**	**Indirect Certainty**	**NMA estimates**	**Prop**	**Initial NMA Certainty**	**D6**	**D7**	**NMA Certainty**
**Non-fatal MI**	Metformin *vs* Thiazolidinediones	5	0.79 (0.46 to 1.37)	0	0	0	0	High	0.92 (0.67 to 1.27)	Moderate	0	Moderate	0.89 (0.67 to 1.17)	0.25	Moderate	0	-2	Very low
**Non-fatal MI**	Metformin *vs* Tirzepatide	0	NA	NA	NA	NA	NA	NA	1.25 (0.14 to 11.19)	Moderate	0	Moderate	1.25 (0.14 to 11.19)	0	Moderate	NA	-2	Very low
**Non-fatal MI**	Non-steroidal MRAs *vs* SGLT2 inhibitors	0	NA	NA	NA	NA	NA	NA	1.02 (0.81 to 1.27)	Moderate	0	Moderate	1.02 (0.81 to 1.27)	0	Moderate	NA	-2	Very low
**Non-fatal MI**	Non-steroidal MRAs *vs* Standard treatments	2	0.91 (0.74 to 1.12)	0	-1	0	0	Moderate	NA	NA	0	NA	0.91 (0.74 to 1.12)	1	Moderate	NA	-2	Very low
**Non-fatal MI**	Non-steroidal MRAs *vs* Sulfonylureas	0	NA	NA	NA	NA	NA	NA	0.91 (0.68 to 1.21)	Moderate	0	Moderate	0.91 (0.68 to 1.21)	0	Moderate	NA	-2	Very low
**Non-fatal MI**	Non-steroidal MRAs *vs* Thiazolidinediones	0	NA	NA	NA	NA	NA	NA	0.94 (0.72 to 1.24)	Moderate	0	Moderate	0.94 (0.72 to 1.24)	0	Moderate	NA	-2	Very low
**Non-fatal MI**	Non-steroidal MRAs *vs* Tirzepatide	0	NA	NA	NA	NA	NA	NA	1.32 (0.15 to 11.83)	Moderate	0	Moderate	1.32 (0.15 to 11.83)	0	Moderate	NA	-2	Very low
**Non-fatal MI**	SGLT2 inhibitors *vs* Standard treatments	27	0.90 (0.82 to 0.98)	0	0	0	0	High	0.68 (0.31 to 1.48)	Moderate	0	Moderate	0.90 (0.82 to 0.98)	0.99	High	0	0	High
**Non-fatal MI**	SGLT2 inhibitors *vs* Sulfonylureas	4	0.59 (0.17 to 2.06)	0	0	0	0	High	0.91 (0.73 to 1.12)	Moderate	0	Moderate	0.89 (0.72 to 1.10)	0.028	Moderate	0	-2	Very low
**Non-fatal MI**	SGLT2 inhibitors *vs* Thiazolidinediones	0	NA	NA	NA	NA	NA	NA	0.93 (0.76 to 1.12)	Moderate	0	Moderate	0.93 (0.76 to 1.12)	0	Moderate	NA	-2	Very low
**Non-fatal MI**	SGLT2 inhibitors *vs* Tirzepatide	0	NA	NA	NA	NA	NA	NA	1.30 (0.15 to 11.55)	Moderate	0	Moderate	1.30 (0.15 to 11.55)	0	Moderate	NA	-2	Very low
**Non-fatal MI**	Sulfonylureas *vs* Standard treatments	4	0.48 (0.12 to 1.81)	-1	0	0	0	Moderate	1.02 (0.84 to 1.24)	Moderate	0	Moderate	1.00 (0.83 to 1.22)	0.021	Moderate	0	-2	Very low

Outcome	Comparisons	k	Direct estimates	D1	D2	D3	D4	Direct Certainty	Indirect estimates	Initial Indirect Certainty	D5	Indirect Certainty	NMA estimates	Prop	Initial NMA Certainty	D6	D7	NMA Certainty
**Non-fatal MI**	Thiazolidinediones *vs* Standard treatments	17	0.93 (0.76 to 1.14)	-1	0	0	0	Moderate	1.08 (0.77 to 1.53)	Moderate	0	Moderate	0.97 (0.81 to 1.15)	0.75	Moderate	0	-2	Very low
**Non-fatal MI**	Tirzepatide *vs* Standard treatments	1	0.20 (0.01 to 5.12)	0	0	0	0	High	1.92 (0.10 to 36.66)	Moderate	0	Moderate	0.69 (0.08 to 6.10)	0.45	Moderate	0	-2	Very low
**Non-fatal MI**	Sulfonylureas *vs* Thiazolidinediones	12	0.96 (0.68 to 1.34)	-1	0	0	0	Moderate	1.10 (0.82 to 1.48)	Moderate	0	Moderate	1.04 (0.83 to 1.30)	0.43	Moderate	0	-2	Very low
**Non-fatal MI**	Sulfonylureas *vs* Tirzepatide	0	NA	NA	NA	NA	NA	NA	1.46 (0.16 to 13.00)	Moderate	0	Moderate	1.46 (0.16 to 13.00)	0	Moderate	NA	-2	Very low
**Non-fatal MI**	Thiazolidinediones *vs* Tirzepatide	0	NA	NA	NA	NA	NA	NA	1.40 (0.16 to 12.51)	Moderate	0	Moderate	1.40 (0.16 to 12.51)	0	Moderate	NA	-2	Very low
**Non-fatal stroke**	Alpha-glucosidase inhibitors *vs* Basal insulin	0	NA	NA	NA	NA	NA	NA	12.42 (0.88 to 176.12)	Moderate	0	Moderate	12.42 (0.88 to 176.12)	0	Moderate	NA	-2	Very low
**Non-fatal stroke**	Alpha-glucosidase inhibitors *vs* Basal-Bolus insulin	0	NA	NA	NA	NA	NA	NA	16.34 (0.76 to 350.38)	Low	0	Low	16.34 (0.76 to 350.38)	0	Low	NA	-2	Very low
**Non-fatal stroke**	Alpha-glucosidase inhibitors *vs* Bolus insulin	0	NA	NA	NA	NA	NA	NA	11.00 (0.54 to 222.94)	Low	0	Low	11.00 (0.54 to 222.94)	0	Low	NA	-2	Very low
**Non-fatal stroke**	Alpha-glucosidase inhibitors *vs* DPP-4 inhibitors	0	NA	NA	NA	NA	NA	NA	10.41 (0.84 to 128.53)	Low	0	Low	10.41 (0.84 to 128.53)	0	Low	NA	-2	Very low
**Non-fatal stroke**	Alpha-glucosidase inhibitors *vs* GLP-1 receptor agonists	0	NA	NA	NA	NA	NA	NA	11.08 (0.90 to 137.09)	Moderate	0	Moderate	11.08 (0.90 to 137.09)	0	Moderate	NA	-2	Very low
**Non-fatal stroke**	Alpha-glucosidase inhibitors *vs* Meglitinides	1	4.96 (0.24 to 103.63)	-2	0	0	0	Low	6.98 (0.08 to 589.86)	Low	0	Low	5.53 (0.45 to 67.89)	0.68	Low	0	-2	Very low
**Non-fatal stroke**	Alpha-glucosidase inhibitors *vs* Metformin	0	NA	NA	NA	NA	NA	NA	9.73 (0.78 to 121.84)	Low	0	Low	9.73 (0.78 to 121.84)	0	Low	NA	-2	Very low

Outcome	Comparisons	k	Direct estimates	D1	D2	D3	D4	Direct Certainty	Indirect estimates	Initial Indirect Certainty	D5	Indirect Certainty	NMA estimates	Prop	Initial NMA Certainty	D6	D7	NMA Certainty
**Non-fatal stroke**	Alpha-glucosidase inhibitors *vs* Non-steroidal MRAs	0	NA	NA	NA	NA	NA	NA	9.46 (0.76 to 117.72)	Low	0	Low	9.46 (0.76 to 117.72)	0	Low	NA	-2	Very low
**Non-fatal stroke**	Alpha-glucosidase inhibitors *vs* SGLT2 inhibitors	0	NA	NA	NA	NA	NA	NA	9.52 (0.77 to 117.89)	Moderate	0	Moderate	9.52 (0.77 to 117.89)	0	Moderate	NA	-2	Very low
**Non-fatal stroke**	Alpha-glucosidase inhibitors *vs* Standard treatments	0	NA	NA	NA	NA	NA	NA	9.44 (0.76 to 116.58)	Low	0	Low	9.44 (0.76 to 116.58)	0	Low	NA	-2	Very low
**Non-fatal stroke**	Alpha-glucosidase inhibitors *vs* Sulfonylureas	1	10.00 (0.48 to 208.75)	-2	0	0	0	Low	7.12 (0.08 to 602.42)	Low	0	Low	8.97 (0.73 to 110.10)	0.68	Low	0	-2	Very low
**Non-fatal stroke**	Alpha-glucosidase inhibitors *vs* Thiazolidinediones	0	NA	NA	NA	NA	NA	NA	11.12 (0.90 to 137.86)	Low	0	Low	11.12 (0.90 to 137.86)	0	Low	NA	-2	Very low
**Non-fatal stroke**	Basal insulin *vs* Basal-Bolus insulin	1	1.95 (0.18 to 21.64)	-2	0	0	0	Low	0.84 (0.06 to 10.94)	Low	0	Low	1.32 (0.23 to 7.61)	0.53	Low	0	-2	Very low
**Non-fatal stroke**	Basal insulin *vs* Bolus insulin	0	NA	NA	NA	NA	NA	NA	0.89 (0.14 to 5.66)	Low	0	Low	0.89 (0.14 to 5.66)	0	Low	NA	-2	Very low
**Non-fatal stroke**	Basal insulin *vs* DPP-4 inhibitors	1	3.36 (0.14 to 82.76)	0	0	0	0	High	0.75 (0.31 to 1.83)	Moderate	0	Moderate	0.84 (0.36 to 1.97)	0.071	Moderate	0	-2	Very low
**Non-fatal stroke**	Basal insulin *vs* GLP-1 receptor agonists	11	0.81 (0.32 to 2.08)	-1	0	0	0	Moderate	1.31 (0.19 to 8.76)	Low	0	Low	0.89 (0.38 to 2.07)	0.8	Moderate	0	-2	Very low
**Non-fatal stroke**	Basal insulin *vs* Meglitinides	0	NA	NA	NA	NA	NA	NA	0.45 (0.06 to 3.56)	Moderate	0	Moderate	0.45 (0.06 to 3.56)	0	Moderate	NA	-2	Very low
**Non-fatal stroke**	Basal insulin *vs* Metformin	0	NA	NA	NA	NA	NA	NA	0.78 (0.32 to 1.93)	Moderate	0	Moderate	0.78 (0.32 to 1.93)	0	Moderate	NA	-2	Very low
**Non-fatal stroke**	Basal insulin *vs* Non-steroidal MRAs	0	NA	NA	NA	NA	NA	NA	0.76 (0.32 to 1.82)	Moderate	0	Moderate	0.76 (0.32 to 1.82)	0	Moderate	NA	-2	Very low

Outcome	Comparisons	k	Direct estimates	D1	D2	D3	D4	Direct Certainty	Indirect estimates	Initial Indirect Certainty	D5	Indirect Certainty	NMA estimates	Prop	Initial NMA Certainty	D6	D7	NMA Certainty
**Non-fatal stroke**	Basal insulin *vs* SGLT2 inhibitors	0	NA	NA	NA	NA	NA	NA	0.77 (0.33 to 1.80)	Moderate	0	Moderate	0.77 (0.33 to 1.80)	0	Moderate	NA	-2	Very low
**Non-fatal stroke**	Basal insulin *vs* Standard treatments	0	NA	NA	NA	NA	NA	NA	0.76 (0.33 to 1.77)	Moderate	0	Moderate	0.76 (0.33 to 1.77)	0	Moderate	NA	-2	Very low
**Non-fatal stroke**	Basal insulin *vs* Sulfonylureas	0	NA	NA	NA	NA	NA	NA	0.72 (0.30 to 1.73)	Moderate	0	Moderate	0.72 (0.30 to 1.73)	0	Moderate	NA	-2	Very low
**Non-fatal stroke**	Basal insulin *vs* Thiazolidinediones	1	0.32 (0.01 to 8.26)	-2	0	0	0	Low	0.97 (0.39 to 2.38)	Moderate	0	Moderate	0.90 (0.38 to 2.13)	0.071	Moderate	0	-2	Very low
**Non-fatal stroke**	Basal-Bolus insulin *vs* Bolus insulin	0	NA	NA	NA	NA	NA	NA	0.67 (0.06 to 7.50)	Low	0	Low	0.67 (0.06 to 7.50)	0	Low	NA	-2	Very low
**Non-fatal stroke**	Basal-Bolus insulin *vs* DPP-4 inhibitors	0	NA	NA	NA	NA	NA	NA	0.64 (0.11 to 3.71)	Low	0	Low	0.64 (0.11 to 3.71)	0	Low	NA	-2	Very low
**Non-fatal stroke**	Basal-Bolus insulin *vs* GLP-1 receptor agonists	1	1.03 (0.06 to 16.62)	-2	0	0	0	Low	0.51 (0.05 to 4.95)	Low	0	Low	0.68 (0.12 to 3.92)	0.4	Low	0	-2	Very low
**Non-fatal stroke**	Basal-Bolus insulin *vs* Meglitinides	0	NA	NA	NA	NA	NA	NA	0.34 (0.03 to 4.50)	Low	0	Low	0.34 (0.03 to 4.50)	0	Low	NA	-2	Very low
**Non-fatal stroke**	Basal-Bolus insulin *vs* Metformin	0	NA	NA	NA	NA	NA	NA	0.60 (0.10 to 3.54)	Low	0	Low	0.60 (0.10 to 3.54)	0	Low	NA	-2	Very low
**Non-fatal stroke**	Basal-Bolus insulin *vs* Non-steroidal MRAs	0	NA	NA	NA	NA	NA	NA	0.58 (0.10 to 3.39)	Low	0	Low	0.58 (0.10 to 3.39)	0	Low	NA	-2	Very low
**Non-fatal stroke**	Basal-Bolus insulin *vs* SGLT2 inhibitors	0	NA	NA	NA	NA	NA	NA	0.58 (0.10 to 3.39)	Moderate	0	Moderate	0.58 (0.10 to 3.39)	0	Moderate	NA	-2	Very low
**Non-fatal stroke**	Basal-Bolus insulin *vs* Standard treatments	0	NA	NA	NA	NA	NA	NA	0.58 (0.10 to 3.35)	Low	0	Low	0.58 (0.10 to 3.35)	0	Low	NA	-2	Very low

Outcome	Comparisons	k	Direct estimates	D1	D2	D3	D4	Direct Certainty	Indirect estimates	Initial Indirect Certainty	D5	Indirect Certainty	NMA estimates	Prop	Initial NMA Certainty	D6	D7	NMA Certainty
**Non-fatal stroke**	Basal-Bolus insulin *vs* Sulfonylureas	0	NA	NA	NA	NA	NA	NA	0.55 (0.09 to 3.22)	Moderate	0	Moderate	0.55 (0.09 to 3.22)	0	Moderate	NA	-2	Very low
**Non-fatal stroke**	Basal-Bolus insulin *vs* Thiazolidinediones	1	0.99 (0.06 to 15.92)	-2	0	0	0	Low	0.53 (0.05 to 5.17)	Low	0	Low	0.68 (0.12 to 3.96)	0.4	Low	0	-2	Very low
**Non-fatal stroke**	Bolus insulin *vs* DPP-4 inhibitors	0	NA	NA	NA	NA	NA	NA	0.95 (0.18 to 4.97)	Moderate	0	Moderate	0.95 (0.18 to 4.97)	0	Moderate	NA	-2	Very low
**Non-fatal stroke**	Bolus insulin *vs* GLP-1 receptor agonists	3	1.01 (0.19 to 5.24)	-2	0	0	0	Low	NA	NA	0	NA	1.01 (0.19 to 5.24)	1	Low	NA	-2	Very low
**Non-fatal stroke**	Bolus insulin *vs* Meglitinides	0	NA	NA	NA	NA	NA	NA	0.50 (0.04 to 6.24)	Moderate	0	Moderate	0.50 (0.04 to 6.24)	0	Moderate	NA	-2	Very low
**Non-fatal stroke**	Bolus insulin *vs* Metformin	0	NA	NA	NA	NA	NA	NA	0.88 (0.16 to 4.76)	Moderate	0	Moderate	0.88 (0.16 to 4.76)	0	Moderate	NA	-2	Very low
**Non-fatal stroke**	Bolus insulin *vs* Non-steroidal MRAs	0	NA	NA	NA	NA	NA	NA	0.86 (0.16 to 4.54)	Low	0	Low	0.86 (0.16 to 4.54)	0	Low	NA	-2	Very low
**Non-fatal stroke**	Bolus insulin *vs* SGLT2 inhibitors	0	NA	NA	NA	NA	NA	NA	0.87 (0.16 to 4.54)	Moderate	0	Moderate	0.87 (0.16 to 4.54)	0	Moderate	NA	-2	Very low
**Non-fatal stroke**	Bolus insulin *vs* Standard treatments	0	NA	NA	NA	NA	NA	NA	0.86 (0.16 to 4.48)	Low	0	Low	0.86 (0.16 to 4.48)	0	Low	NA	-2	Very low
**Non-fatal stroke**	Bolus insulin *vs* Sulfonylureas	0	NA	NA	NA	NA	NA	NA	0.82 (0.15 to 4.33)	Moderate	0	Moderate	0.82 (0.15 to 4.33)	0	Moderate	NA	-2	Very low
**Non-fatal stroke**	Bolus insulin *vs* Thiazolidinediones	0	NA	NA	NA	NA	NA	NA	1.01 (0.19 to 5.34)	Low	0	Low	1.01 (0.19 to 5.34)	0	Low	NA	-2	Very low
**Non-fatal stroke**	DPP-4 inhibitors *vs* GLP-1 receptor agonists	8	0.98 (0.34 to 2.82)	-1	0	0	0	Moderate	1.07 (0.91 to 1.26)	Moderate	0	Moderate	1.06 (0.91 to 1.25)	0.024	Moderate	0	-2	Very low

Outcome	Comparisons	k	Direct estimates	D1	D2	D3	D4	Direct Certainty	Indirect estimates	Initial Indirect Certainty	D5	Indirect Certainty	NMA estimates	Prop	Initial NMA Certainty	D6	D7	NMA Certainty
**Non-fatal stroke**	DPP-4 inhibitors *vs* Meglitinides	0	NA	NA	NA	NA	NA	NA	0.53 (0.08 to 3.55)	Moderate	0	Moderate	0.53 (0.08 to 3.55)	0	Moderate	NA	-2	Very low
**Non-fatal stroke**	DPP-4 inhibitors *vs* Metformin	5	0.79 (0.20 to 3.13)	0	0	0	0	High	0.94 (0.67 to 1.32)	Moderate	0	Moderate	0.93 (0.67 to 1.29)	0.056	Moderate	0	-2	Very low
**Non-fatal stroke**	DPP-4 inhibitors *vs* Non-steroidal MRAs	0	NA	NA	NA	NA	NA	NA	0.91 (0.72 to 1.15)	Moderate	0	Moderate	0.91 (0.72 to 1.15)	0	Moderate	NA	-2	Very low
**Non-fatal stroke**	DPP-4 inhibitors *vs* SGLT2 inhibitors	6	0.93 (0.28 to 3.06)	0	0	0	0	High	0.91 (0.77 to 1.09)	Moderate	0	Moderate	0.91 (0.77 to 1.08)	0.02	Moderate	0	-1	Low
**Non-fatal stroke**	DPP-4 inhibitors *vs* Standard treatments	40	0.93 (0.82 to 1.07)	-1	0	0	0	Moderate	0.72 (0.50 to 1.06)	Moderate	0	Moderate	0.91 (0.80 to 1.03)	0.89	Moderate	0	-1	Low
**Non-fatal stroke**	DPP-4 inhibitors *vs* Sulfonylureas	13	0.79 (0.61 to 1.03)	0	0	0	0	High	1.03 (0.70 to 1.50)	Moderate	0	Moderate	0.86 (0.69 to 1.07)	0.68	High	0	-1	Moderate
**Non-fatal stroke**	DPP-4 inhibitors *vs* Thiazolidinediones	4	0.70 (0.20 to 2.45)	0	0	0	0	High	1.08 (0.86 to 1.36)	Low	0	Low	1.07 (0.85 to 1.33)	0.032	Low	0	-2	Very low
**Non-fatal stroke**	GLP-1 receptor agonists *vs* Meglitinides	0	NA	NA	NA	NA	NA	NA	0.50 (0.07 to 3.35)	Moderate	0	Moderate	0.50 (0.07 to 3.35)	0	Moderate	NA	-2	Very low
**Non-fatal stroke**	GLP-1 receptor agonists *vs* Metformin	2	0.70 (0.07 to 6.77)	0	0	0	0	High	0.88 (0.63 to 1.23)	Moderate	0	Moderate	0.88 (0.63 to 1.22)	0.021	Moderate	0	-2	Very low
**Non-fatal stroke**	GLP-1 receptor agonists *vs* Non-steroidal MRAs	0	NA	NA	NA	NA	NA	NA	0.85 (0.68 to 1.07)	Moderate	0	Moderate	0.85 (0.68 to 1.07)	0	Moderate	NA	-1	Low
**Non-fatal stroke**	GLP-1 receptor agonists *vs* SGLT2 inhibitors	2	3.68 (0.60 to 22.52)	-1	0	0	0	Moderate	0.85 (0.73 to 0.99)	Moderate	0	Moderate	0.86 (0.74 to 1.00)	0.0071	Moderate	0	-1	Low
**Non-fatal stroke**	GLP-1 receptor agonists *vs* Standard treatments	30	0.85 (0.76 to 0.94)	0	0	0	0	High	1.16 (0.55 to 2.45)	Moderate	0	Moderate	0.85 (0.77 to 0.94)	0.98	High	0	0	High

Outcome	Comparisons	k	Direct estimates	D1	D2	D3	D4	Direct Certainty	Indirect estimates	Initial Indirect Certainty	D5	Indirect Certainty	NMA estimates	Prop	Initial NMA Certainty	D6	D7	NMA Certainty
**Non-fatal stroke**	GLP-1 receptor agonists *vs* Sulfonylureas	4	0.87 (0.21 to 3.52)	-1	0	0	0	Moderate	0.81 (0.63 to 1.04)	Moderate	0	Moderate	0.81 (0.63 to 1.04)	0.031	Moderate	0	-1	Low
**Non-fatal stroke**	GLP-1 receptor agonists *vs* Thiazolidinediones	3	0.62 (0.10 to 3.98)	-1	0	0	0	Moderate	1.01 (0.81 to 1.26)	Moderate	0	Moderate	1.00 (0.80 to 1.25)	0.014	Moderate	0	-2	Very low
**Non-fatal stroke**	Meglitinides *vs* Metformin	0	NA	NA	NA	NA	NA	NA	1.76 (0.26 to 11.98)	Moderate	0	Moderate	1.76 (0.26 to 11.98)	0	Moderate	NA	-2	Very low
**Non-fatal stroke**	Meglitinides *vs* Non-steroidal MRAs	0	NA	NA	NA	NA	NA	NA	1.71 (0.25 to 11.54)	Moderate	0	Moderate	1.71 (0.25 to 11.54)	0	Moderate	NA	-2	Very low
**Non-fatal stroke**	Meglitinides *vs* SGLT2 inhibitors	0	NA	NA	NA	NA	NA	NA	1.72 (0.26 to 11.53)	Moderate	0	Moderate	1.72 (0.26 to 11.53)	0	Moderate	NA	-2	Very low
**Non-fatal stroke**	Meglitinides *vs* Standard treatments	1	0.34 (0.01 to 8.71)	-1	0	0	0	Moderate	3.97 (0.38 to 41.45)	Moderate	0	Moderate	1.71 (0.26 to 11.40)	0.34	Moderate	0	-2	Very low
**Non-fatal stroke**	Meglitinides *vs* Sulfonylureas	2	3.74 (0.36 to 38.67)	-1	0	0	0	Moderate	0.32 (0.01 to 8.28)	Low	0	Low	1.62 (0.24 to 10.79)	0.66	Moderate	0	-2	Very low
**Non-fatal stroke**	Meglitinides *vs* Thiazolidinediones	0	NA	NA	NA	NA	NA	NA	2.01 (0.30 to 13.51)	Low	0	Low	2.01 (0.30 to 13.51)	0	Low	NA	-2	Very low
**Non-fatal stroke**	Metformin *vs* Non-steroidal MRAs	0	NA	NA	NA	NA	NA	NA	0.97 (0.67 to 1.41)	Moderate	0	Moderate	0.97 (0.67 to 1.41)	0	Moderate	NA	-2	Very low
**Non-fatal stroke**	Metformin *vs* SGLT2 inhibitors	0	NA	NA	NA	NA	NA	NA	0.98 (0.70 to 1.37)	Moderate	0	Moderate	0.98 (0.70 to 1.37)	0	Moderate	NA	-2	Very low
**Non-fatal stroke**	Metformin *vs* Standard treatments	3	0.97 (0.63 to 1.47)	-1	0	0	0	Moderate	0.98 (0.61 to 1.56)	Moderate	0	Moderate	0.97 (0.71 to 1.33)	0.55	Moderate	0	-2	Very low
**Non-fatal stroke**	Metformin *vs* Sulfonylureas	2	0.88 (0.52 to 1.48)	0	0	0	0	High	0.96 (0.61 to 1.51)	Moderate	0	Moderate	0.92 (0.66 to 1.30)	0.44	Moderate	0	-2	Very low

Outcome	Comparisons	k	Direct estimates	D1	D2	D3	D4	Direct Certainty	Indirect estimates	Initial Indirect Certainty	D5	Indirect Certainty	NMA estimates	Prop	Initial NMA Certainty	D6	D7	NMA Certainty
**Non-fatal stroke**	Metformin *vs* Thiazolidinediones	3	1.26 (0.67 to 2.40)	-1	0	0	0	Moderate	1.10 (0.73 to 1.65)	Low	0	Low	1.14 (0.81 to 1.61)	0.29	Low	0	-2	Very low
**Non-fatal stroke**	Non-steroidal MRAs *vs* SGLT2 inhibitors	0	NA	NA	NA	NA	NA	NA	1.01 (0.80 to 1.27)	Moderate	0	Moderate	1.01 (0.80 to 1.27)	0	Moderate	NA	-2	Very low
**Non-fatal stroke**	Non-steroidal MRAs *vs* Standard treatments	2	1.00 (0.82 to 1.22)	0	-1	0	0	Moderate	NA	NA	0	NA	1.00 (0.82 to 1.22)	1	Moderate	NA	-2	Very low
**Non-fatal stroke**	Non-steroidal MRAs *vs* Sulfonylureas	0	NA	NA	NA	NA	NA	NA	0.95 (0.70 to 1.29)	Moderate	0	Moderate	0.95 (0.70 to 1.29)	0	Moderate	NA	-2	Very low
**Non-fatal stroke**	Non-steroidal MRAs *vs* Thiazolidinediones	0	NA	NA	NA	NA	NA	NA	1.18 (0.89 to 1.56)	Low	0	Low	1.18 (0.89 to 1.56)	0	Low	NA	-2	Very low
**Non-fatal stroke**	SGLT2 inhibitors *vs* Standard treatments	27	0.99 (0.88 to 1.11)	0	0	0	0	High	1.16 (0.53 to 2.57)	Moderate	0	Moderate	0.99 (0.88 to 1.11)	0.98	High	0	-2	Low
**Non-fatal stroke**	SGLT2 inhibitors *vs* Sulfonylureas	6	2.33 (0.71 to 7.60)	0	0	0	0	High	0.90 (0.70 to 1.17)	Moderate	0	Moderate	0.94 (0.73 to 1.21)	0.046	Moderate	0	-2	Very low
**Non-fatal stroke**	SGLT2 inhibitors *vs* Thiazolidinediones	0	NA	NA	NA	NA	NA	NA	1.17 (0.93 to 1.47)	Moderate	0	Moderate	1.17 (0.93 to 1.47)	0	Moderate	NA	-1	Low
**Non-fatal stroke**	Sulfonylureas *vs* Standard treatments	4	0.86 (0.19 to 3.81)	-1	0	0	0	Moderate	1.06 (0.84 to 1.33)	Moderate	0	Moderate	1.05 (0.84 to 1.32)	0.023	Moderate	0	-2	Very low
**Non-fatal stroke**	Thiazolidinediones *vs* Standard treatments	11	0.81 (0.65 to 1.02)	-1	0	0	-1	Low	0.99 (0.65 to 1.49)	Moderate	0	Moderate	0.85 (0.70 to 1.03)	0.77	Low	0	-1	Very low
**Non-fatal stroke**	Sulfonylureas *vs* Thiazolidinediones	9	1.10 (0.74 to 1.63)	-1	0	0	0	Moderate	1.36 (0.96 to 1.91)	Moderate	0	Moderate	1.24 (0.96 to 1.61)	0.43	Moderate	0	-1	Low
**HHF**	Alpha-glucosidase inhibitors *vs* Basal insulin	0	NA	NA	NA	NA	NA	NA	3.47 (0.13 to 90.34)	Moderate	0	Moderate	3.47 (0.13 to 90.34)	0	Moderate	NA	-2	Very low

Outcome	Comparisons	k	Direct estimates	D1	D2	D3	D4	Direct Certainty	Indirect estimates	Initial Indirect Certainty	D5	Indirect Certainty	NMA estimates	Prop	Initial NMA Certainty	D6	D7	NMA Certainty
**HHF**	Alpha-glucosidase inhibitors *vs* Bolus insulin	0	NA	NA	NA	NA	NA	NA	5.05 (0.10 to 262.01)	Low	0	Low	5.05 (0.10 to 262.01)	0	Low	NA	-2	Very low
**HHF**	Alpha-glucosidase inhibitors *vs* DPP-4 inhibitors	0	NA	NA	NA	NA	NA	NA	3.09 (0.12 to 78.44)	Moderate	0	Moderate	3.09 (0.12 to 78.44)	0	Moderate	NA	-2	Very low
**HHF**	Alpha-glucosidase inhibitors *vs* GLP-1 receptor agonists	0	NA	NA	NA	NA	NA	NA	3.59 (0.14 to 91.17)	Moderate	0	Moderate	3.59 (0.14 to 91.17)	0	Moderate	NA	-2	Very low
**HHF**	Alpha-glucosidase inhibitors *vs* Metformin	0	NA	NA	NA	NA	NA	NA	2.25 (0.06 to 84.00)	Moderate	0	Moderate	2.25 (0.06 to 84.00)	0	Moderate	NA	-2	Very low
**HHF**	Alpha-glucosidase inhibitors *vs* Non-steroidal MRAs	0	NA	NA	NA	NA	NA	NA	4.18 (0.16 to 106.46)	Moderate	0	Moderate	4.18 (0.16 to 106.46)	0	Moderate	NA	-2	Very low
**HHF**	Alpha-glucosidase inhibitors *vs* SGLT2 inhibitors	0	NA	NA	NA	NA	NA	NA	4.93 (0.19 to 125.08)	Moderate	0	Moderate	4.93 (0.19 to 125.08)	0	Moderate	NA	-2	Very low
**HHF**	Alpha-glucosidase inhibitors *vs* Standard treatments	1	3.25 (0.13 to 82.49)	-1	0	0	0	Moderate	NA	NA	0	NA	3.25 (0.13 to 82.49)	1	Moderate	NA	-2	Very low
**HHF**	Alpha-glucosidase inhibitors *vs* Sulfonylureas	0	NA	NA	NA	NA	NA	NA	3.30 (0.13 to 84.25)	Moderate	0	Moderate	3.30 (0.13 to 84.25)	0	Moderate	NA	-2	Very low
**HHF**	Alpha-glucosidase inhibitors *vs* Thiazolidinediones	0	NA	NA	NA	NA	NA	NA	2.11 (0.08 to 53.77)	Moderate	0	Moderate	2.11 (0.08 to 53.77)	0	Moderate	NA	-2	Very low
**HHF**	Alpha-glucosidase inhibitors *vs* Tirzepatide	0	NA	NA	NA	NA	NA	NA	5.18 (0.16 to 171.40)	Moderate	0	Moderate	5.18 (0.16 to 171.40)	0	Moderate	NA	-2	Very low
**HHF**	Basal insulin *vs* Bolus insulin	0	NA	NA	NA	NA	NA	NA	1.46 (0.15 to 14.61)	Moderate	0	Moderate	1.46 (0.15 to 14.61)	0	Moderate	NA	-2	Very low
**HHF**	Basal insulin *vs* DPP-4 inhibitors	1	0.87 (0.51 to 1.48)	0	0	0	0	High	0.93 (0.47 to 1.84)	Moderate	0	Moderate	0.89 (0.59 to 1.36)	0.62	High	0	-2	Low

Outcome	Comparisons	k	Direct estimates	D1	D2	D3	D4	Direct Certainty	Indirect estimates	Initial Indirect Certainty	D5	Indirect Certainty	NMA estimates	Prop	Initial NMA Certainty	D6	D7	NMA Certainty
**HHF**	Basal insulin *vs* GLP-1 receptor agonists	5	1.60 (0.91 to 2.84)	0	0	0	0	High	0.60 (0.32 to 1.14)	Moderate	0	Moderate	1.04 (0.68 to 1.58)	0.55	High	-1	-2	Very low
**HHF**	Basal insulin *vs* Metformin	0	NA	NA	NA	NA	NA	NA	0.65 (0.12 to 3.48)	Moderate	0	Moderate	0.65 (0.12 to 3.48)	0	Moderate	NA	-2	Very low
**HHF**	Basal insulin *vs* Non-steroidal MRAs	0	NA	NA	NA	NA	NA	NA	1.21 (0.77 to 1.90)	Moderate	0	Moderate	1.21 (0.77 to 1.90)	0	Moderate	NA	-2	Very low
**HHF**	Basal insulin *vs* SGLT2 inhibitors	0	NA	NA	NA	NA	NA	NA	1.42 (0.92 to 2.19)	Moderate	0	Moderate	1.42 (0.92 to 2.19)	0	Moderate	NA	-1	Low
**HHF**	Basal insulin *vs* Standard treatments	2	0.33 (0.03 to 3.16)	-1	0	0	0	Moderate	0.97 (0.63 to 1.50)	Moderate	0	Moderate	0.94 (0.62 to 1.43)	0.035	Moderate	0	-2	Very low
**HHF**	Basal insulin *vs* Sulfonylureas	1	0.86 (0.50 to 1.46)	0	0	0	0	High	1.18 (0.55 to 2.50)	Moderate	0	Moderate	0.95 (0.62 to 1.47)	0.67	High	0	-2	Low
**HHF**	Basal insulin *vs* Thiazolidinediones	1	0.34 (0.01 to 8.53)	0	0	0	0	High	0.62 (0.39 to 0.98)	Moderate	0	Moderate	0.61 (0.38 to 0.96)	0.021	Moderate	0	0	Moderate
**HHF**	Basal insulin *vs* Tirzepatide	1	1.50 (0.42 to 5.32)	0	0	0	0	High	NA	NA	0	NA	1.50 (0.42 to 5.32)	1	High	NA	-2	Low
**HHF**	Bolus insulin *vs* DPP-4 inhibitors	0	NA	NA	NA	NA	NA	NA	0.61 (0.06 to 5.92)	Low	0	Low	0.61 (0.06 to 5.92)	0	Low	NA	-2	Very low
**HHF**	Bolus insulin *vs* GLP-1 receptor agonists	2	0.71 (0.07 to 6.85)	-2	0	0	0	Low	NA	NA	0	NA	0.71 (0.07 to 6.85)	1	Low	NA	-2	Very low
**HHF**	Bolus insulin *vs* Metformin	0	NA	NA	NA	NA	NA	NA	0.45 (0.03 to 7.25)	Moderate	0	Moderate	0.45 (0.03 to 7.25)	0	Moderate	NA	-2	Very low
**HHF**	Bolus insulin *vs* Non-steroidal MRAs	0	NA	NA	NA	NA	NA	NA	0.83 (0.09 to 8.04)	Low	0	Low	0.83 (0.09 to 8.04)	0	Low	NA	-2	Very low

Outcome	Comparisons	k	Direct estimates	D1	D2	D3	D4	Direct Certainty	Indirect estimates	Initial Indirect Certainty	D5	Indirect Certainty	NMA estimates	Prop	Initial NMA Certainty	D6	D7	NMA Certainty
**HHF**	Bolus insulin *vs* SGLT2 inhibitors	0	NA	NA	NA	NA	NA	NA	0.98 (0.10 to 9.43)	Moderate	0	Moderate	0.98 (0.10 to 9.43)	0	Moderate	NA	-2	Very low
**HHF**	Bolus insulin *vs* Standard treatments	0	NA	NA	NA	NA	NA	NA	0.64 (0.07 to 6.22)	Low	0	Low	0.64 (0.07 to 6.22)	0	Low	NA	-2	Very low
**HHF**	Bolus insulin *vs* Sulfonylureas	0	NA	NA	NA	NA	NA	NA	0.65 (0.07 to 6.37)	Moderate	0	Moderate	0.65 (0.07 to 6.37)	0	Moderate	NA	-2	Very low
**HHF**	Bolus insulin *vs* Thiazolidinediones	0	NA	NA	NA	NA	NA	NA	0.42 (0.04 to 4.06)	Moderate	0	Moderate	0.42 (0.04 to 4.06)	0	Moderate	NA	-2	Very low
**HHF**	Bolus insulin *vs* Tirzepatide	0	NA	NA	NA	NA	NA	NA	1.03 (0.07 to 14.25)	Moderate	0	Moderate	1.03 (0.07 to 14.25)	0	Moderate	NA	-2	Very low
**HHF**	DPP-4 inhibitors *vs* GLP-1 receptor agonists	8	1.71 (1.04 to 2.82)	-1	0	0	0	Moderate	1.13 (0.98 to 1.29)	Moderate	0	Moderate	1.16 (1.02 to 1.33)	0.07	Moderate	0	0	Moderate
**HHF**	DPP-4 inhibitors *vs* Metformin	1	1.51 (0.03 to 76.36)	0	0	0	0	High	0.63 (0.10 to 3.76)	Moderate	0	Moderate	0.73 (0.14 to 3.72)	0.17	Moderate	0	-2	Very low
**HHF**	DPP-4 inhibitors *vs* Non-steroidal MRAs	0	NA	NA	NA	NA	NA	NA	1.35 (1.11 to 1.65)	Moderate	0	Moderate	1.35 (1.11 to 1.65)	0	Moderate	NA	0	Moderate
**HHF**	DPP-4 inhibitors *vs* SGLT2 inhibitors	1	0.40 (0.02 to 9.88)	0	0	0	0	High	1.60 (1.39 to 1.84)	Moderate	0	Moderate	1.59 (1.39 to 1.83)	0.0019	Moderate	0	0	Moderate
**HHF**	DPP-4 inhibitors *vs* Standard treatments	25	1.03 (0.93 to 1.15)	-1	0	0	0	Moderate	1.34 (0.92 to 1.95)	Moderate	0	Moderate	1.05 (0.95 to 1.16)	0.93	Moderate	0	-1	Low
**HHF**	DPP-4 inhibitors *vs* Sulfonylureas	13	1.12 (0.89 to 1.42)	0	0	0	0	High	0.84 (0.51 to 1.39)	Moderate	0	Moderate	1.07 (0.86 to 1.32)	0.83	High	0	-2	Low
**HHF**	DPP-4 inhibitors *vs* Thiazolidinediones	1	0.33 (0.01 to 8.19)	0	0	0	0	High	0.68 (0.55 to 0.85)	Moderate	0	Moderate	0.68 (0.55 to 0.85)	0.0046	Moderate	0	0	Moderate

Outcome	Comparisons	k	Direct estimates	D1	D2	D3	D4	Direct Certainty	Indirect estimates	Initial Indirect Certainty	D5	Indirect Certainty	NMA estimates	Prop	Initial NMA Certainty	D6	D7	NMA Certainty
**HHF**	DPP-4 inhibitors *vs* Tirzepatide	0	NA	NA	NA	NA	NA	NA	1.68 (0.44 to 6.38)	Moderate	0	Moderate	1.68 (0.44 to 6.38)	0	Moderate	NA	-2	Very low
**HHF**	GLP-1 receptor agonists *vs* Metformin	2	0.36 (0.05 to 2.81)	0	0	0	0	High	1.60 (0.11 to 23.28)	Moderate	0	Moderate	0.63 (0.12 to 3.19)	0.63	High	0	-2	Low
**HHF**	GLP-1 receptor agonists *vs* Non-steroidal MRAs	0	NA	NA	NA	NA	NA	NA	1.16 (0.96 to 1.41)	Moderate	0	Moderate	1.16 (0.96 to 1.41)	0	Moderate	NA	-1	Low
**HHF**	GLP-1 receptor agonists *vs* SGLT2 inhibitors	1	0.50 (0.05 to 5.54)	-2	0	0	0	Low	1.38 (1.21 to 1.57)	Moderate	0	Moderate	1.37 (1.20 to 1.56)	0.003	Moderate	0	0	Moderate
**HHF**	GLP-1 receptor agonists *vs* Standard treatments	22	0.92 (0.84 to 1.01)	0	0	0	0	High	0.58 (0.37 to 0.91)	Moderate	0	Moderate	0.91 (0.83 to 0.99)	0.96	High	-1	0	Moderate
**HHF**	GLP-1 receptor agonists *vs* Sulfonylureas	4	0.56 (0.31 to 1.01)	-1	0	0	0	Moderate	1.01 (0.78 to 1.31)	Moderate	0	Moderate	0.92 (0.72 to 1.16)	0.16	Moderate	0	-2	Very low
**HHF**	GLP-1 receptor agonists *vs* Thiazolidinediones	1	0.22 (0.01 to 5.38)	0	0	0	0	High	0.59 (0.47 to 0.73)	Moderate	0	Moderate	0.59 (0.47 to 0.73)	0.0045	Moderate	0	0	Moderate
**HHF**	GLP-1 receptor agonists *vs* Tirzepatide	0	NA	NA	NA	NA	NA	NA	1.44 (0.38 to 5.50)	Moderate	0	Moderate	1.44 (0.38 to 5.50)	0	Moderate	NA	-2	Very low
**HHF**	Metformin *vs* Non-steroidal MRAs	0	NA	NA	NA	NA	NA	NA	1.86 (0.36 to 9.54)	Moderate	0	Moderate	1.86 (0.36 to 9.54)	0	Moderate	NA	-2	Very low
**HHF**	Metformin *vs* SGLT2 inhibitors	1	1.43 (0.06 to 35.52)	0	0	0	0	High	2.54 (0.38 to 16.79)	Moderate	0	Moderate	2.19 (0.43 to 11.16)	0.26	Moderate	0	-2	Very low
**HHF**	Metformin *vs* Standard treatments	0	NA	NA	NA	NA	NA	NA	1.45 (0.28 to 7.36)	Moderate	0	Moderate	1.45 (0.28 to 7.36)	0	Moderate	NA	-2	Very low
**HHF**	Metformin *vs* Sulfonylureas	0	NA	NA	NA	NA	NA	NA	1.47 (0.28 to 7.57)	Moderate	0	Moderate	1.47 (0.28 to 7.57)	0	Moderate	NA	-2	Very low

Outcome	Comparisons	k	Direct estimates	D1	D2	D3	D4	Direct Certainty	Indirect estimates	Initial Indirect Certainty	D5	Indirect Certainty	NMA estimates	Prop	Initial NMA Certainty	D6	D7	NMA Certainty
**HHF**	Metformin *vs* Thiazolidinediones	1	0.22 (0.01 to 5.43)	0	0	0	0	High	1.56 (0.23 to 10.44)	Moderate	0	Moderate	0.94 (0.18 to 4.81)	0.26	Moderate	0	-2	Very low
**HHF**	Metformin *vs* Tirzepatide	0	NA	NA	NA	NA	NA	NA	2.30 (0.28 to 18.91)	Moderate	0	Moderate	2.30 (0.28 to 18.91)	0	Moderate	NA	-2	Very low
**HHF**	Non-steroidal MRAs *vs* SGLT2 inhibitors	0	NA	NA	NA	NA	NA	NA	1.18 (0.97 to 1.43)	Moderate	0	Moderate	1.18 (0.97 to 1.43)	0	Moderate	NA	-1	Low
**HHF**	Non-steroidal MRAs *vs* Standard treatments	2	0.78 (0.66 to 0.92)	0	-1	0	0	Moderate	NA	NA	0	NA	0.78 (0.66 to 0.92)	1	Moderate	NA	0	Moderate
**HHF**	Non-steroidal MRAs *vs* Sulfonylureas	0	NA	NA	NA	NA	NA	NA	0.79 (0.60 to 1.04)	Moderate	0	Moderate	0.79 (0.60 to 1.04)	0	Moderate	NA	-1	Low
**HHF**	Non-steroidal MRAs *vs* Thiazolidinediones	0	NA	NA	NA	NA	NA	NA	0.50 (0.39 to 0.65)	Moderate	0	Moderate	0.50 (0.39 to 0.65)	0	Moderate	NA	0	Moderate
**HHF**	Non-steroidal MRAs *vs* Tirzepatide	0	NA	NA	NA	NA	NA	NA	1.24 (0.32 to 4.77)	Moderate	0	Moderate	1.24 (0.32 to 4.77)	0	Moderate	NA	-2	Very low
**HHF**	SGLT2 inhibitors *vs* Standard treatments	26	0.66 (0.60 to 0.72)	0	0	0	0	High	1.13 (0.39 to 3.26)	Moderate	0	Moderate	0.66 (0.60 to 0.73)	0.99	High	0	0	High
**HHF**	SGLT2 inhibitors *vs* Sulfonylureas	5	0.85 (0.23 to 3.17)	-1	0	0	-1	Low	0.66 (0.52 to 0.85)	Moderate	0	Moderate	0.67 (0.53 to 0.85)	0.034	Moderate	0	0	Moderate
**HHF**	SGLT2 inhibitors *vs* Thiazolidinediones	0	NA	NA	NA	NA	NA	NA	0.43 (0.34 to 0.53)	Moderate	0	Moderate	0.43 (0.34 to 0.53)	0	Moderate	NA	0	Moderate
**HHF**	SGLT2 inhibitors *vs* Tirzepatide	0	NA	NA	NA	NA	NA	NA	1.05 (0.28 to 4.02)	Moderate	0	Moderate	1.05 (0.28 to 4.02)	0	Moderate	NA	-2	Very low
**HHF**	Sulfonylureas *vs* Standard treatments	2	0.76 (0.06 to 9.06)	0	0	0	0	High	0.99 (0.79 to 1.24)	Moderate	0	Moderate	0.99 (0.79 to 1.23)	0.0081	Moderate	0	-2	Very low

Outcome	Comparisons	k	Direct estimates	D1	D2	D3	D4	Direct Certainty	Indirect estimates	Initial Indirect Certainty	D5	Indirect Certainty	NMA estimates	Prop	Initial NMA Certainty	D6	D7	NMA Certainty
**HHF**	Thiazolidinediones *vs* Standard treatments	20	1.56 (1.27 to 1.92)	-1	0	0	0	Moderate	1.42 (0.77 to 2.62)	Moderate	0	Moderate	1.54 (1.27 to 1.88)	0.9	Moderate	0	0	Moderate
**HHF**	Tirzepatide *vs* Standard treatments	0	NA	NA	NA	NA	NA	NA	0.63 (0.16 to 2.39)	Moderate	0	Moderate	0.63 (0.16 to 2.39)	0	Moderate	NA	-2	Very low
**HHF**	Sulfonylureas *vs* Thiazolidinediones	9	0.76 (0.42 to 1.39)	-1	0	0	0	Moderate	0.61 (0.44 to 0.84)	Moderate	0	Moderate	0.64 (0.48 to 0.85)	0.22	Moderate	0	0	Moderate
**HHF**	Sulfonylureas *vs* Tirzepatide	0	NA	NA	NA	NA	NA	NA	1.57 (0.41 to 6.01)	Moderate	0	Moderate	1.57 (0.41 to 6.01)	0	Moderate	NA	-2	Very low
**HHF**	Thiazolidinediones *vs* Tirzepatide	0	NA	NA	NA	NA	NA	NA	2.46 (0.64 to 9.48)	Moderate	0	Moderate	2.46 (0.64 to 9.48)	0	Moderate	NA	-2	Very low
**ESKD**	Basal insulin *vs* Bolus insulin	0	NA	NA	NA	NA	NA	NA	0.47 (0.02 to 12.36)	Very low	0	Very low	0.47 (0.02 to 12.36)	0	Very low	NA	-2	Very low
**ESKD**	Basal insulin *vs* DPP-4 inhibitors	0	NA	NA	NA	NA	NA	NA	1.15 (0.59 to 2.24)	Low	0	Low	1.15 (0.59 to 2.24)	0	Low	NA	-2	Very low
**ESKD**	Basal insulin *vs* GLP-1 receptor agonists	2	1.42 (0.74 to 2.74)	-1	-1	0	0	Low	1.99 (0.04 to 109.09)	Moderate	0	Moderate	1.44 (0.75 to 2.74)	0.97	Low	0	-2	Very low
**ESKD**	Basal insulin *vs* Metformin	0	NA	NA	NA	NA	NA	NA	0.74 (0.14 to 3.83)	Low	0	Low	0.74 (0.14 to 3.83)	0	Low	NA	-2	Very low
**ESKD**	Basal insulin *vs* Non-steroidal MRAs	0	NA	NA	NA	NA	NA	NA	1.44 (0.74 to 2.78)	Low	0	Low	1.44 (0.74 to 2.78)	0	Low	NA	-2	Very low
**ESKD**	Basal insulin *vs* SGLT2 inhibitors	0	NA	NA	NA	NA	NA	NA	1.97 (1.02 to 3.82)	Low	0	Low	1.97 (1.02 to 3.82)	0	Low	NA	0	Low
**ESKD**	Basal insulin *vs* Standard treatments	0	NA	NA	NA	NA	NA	NA	1.20 (0.62 to 2.30)	Low	0	Low	1.20 (0.62 to 2.30)	0	Low	NA	-2	Very low

Outcome	Comparisons	k	Direct estimates	D1	D2	D3	D4	Direct Certainty	Indirect estimates	Initial Indirect Certainty	D5	Indirect Certainty	NMA estimates	Prop	Initial NMA Certainty	D6	D7	NMA Certainty
**ESKD**	Basal insulin *vs* Sulfonylureas	0	NA	NA	NA	NA	NA	NA	1.75 (0.72 to 4.26)	Low	0	Low	1.75 (0.72 to 4.26)	0	Low	NA	-2	Very low
**ESKD**	Basal insulin *vs* Thiazolidinediones	0	NA	NA	NA	NA	NA	NA	1.73 (0.70 to 4.26)	Low	0	Low	1.73 (0.70 to 4.26)	0	Low	NA	-2	Very low
**ESKD**	Basal insulin *vs* Tirzepatide	1	1.99 (0.18 to 22.00)	0	-1	0	0	Moderate	1.42 (0.05 to 37.39)	Low	0	Low	1.77 (0.26 to 12.27)	0.65	Moderate	0	-2	Very low
**ESKD**	Bolus insulin *vs* DPP-4 inhibitors	0	NA	NA	NA	NA	NA	NA	2.45 (0.10 to 60.68)	Low	0	Low	2.45 (0.10 to 60.68)	0	Low	NA	-2	Very low
**ESKD**	Bolus insulin *vs* GLP-1 receptor agonists	1	3.05 (0.12 to 75.27)	-2	-1	0	0	Very low	NA	NA	0	NA	3.05 (0.12 to 75.27)	1	Very low	NA	-2	Very low
**ESKD**	Bolus insulin *vs* Metformin	0	NA	NA	NA	NA	NA	NA	1.58 (0.05 to 54.60)	Low	0	Low	1.58 (0.05 to 54.60)	0	Low	NA	-2	Very low
**ESKD**	Bolus insulin *vs* Non-steroidal MRAs	0	NA	NA	NA	NA	NA	NA	3.05 (0.12 to 75.50)	Low	0	Low	3.05 (0.12 to 75.50)	0	Low	NA	-2	Very low
**ESKD**	Bolus insulin *vs* SGLT2 inhibitors	0	NA	NA	NA	NA	NA	NA	4.19 (0.17 to 103.67)	Low	0	Low	4.19 (0.17 to 103.67)	0	Low	NA	-2	Very low
**ESKD**	Bolus insulin *vs* Standard treatments	0	NA	NA	NA	NA	NA	NA	2.55 (0.10 to 62.86)	Low	0	Low	2.55 (0.10 to 62.86)	0	Low	NA	-2	Very low
**ESKD**	Bolus insulin *vs* Sulfonylureas	0	NA	NA	NA	NA	NA	NA	3.73 (0.14 to 97.39)	Low	0	Low	3.73 (0.14 to 97.39)	0	Low	NA	-2	Very low
**ESKD**	Bolus insulin *vs* Thiazolidinediones	0	NA	NA	NA	NA	NA	NA	3.68 (0.14 to 96.44)	Low	0	Low	3.68 (0.14 to 96.44)	0	Low	NA	-2	Very low
**ESKD**	Bolus insulin *vs* Tirzepatide	0	NA	NA	NA	NA	NA	NA	3.77 (0.09 to 161.65)	Low	0	Low	3.77 (0.09 to 161.65)	0	Low	NA	-2	Very low

Outcome	Comparisons	k	Direct estimates	D1	D2	D3	D4	Direct Certainty	Indirect estimates	Initial Indirect Certainty	D5	Indirect Certainty	NMA estimates	Prop	Initial NMA Certainty	D6	D7	NMA Certainty
**ESKD**	DPP-4 inhibitors *vs* GLP-1 receptor agonists	3	1.36 (0.41 to 4.47)	-1	-1	0	0	Low	1.24 (1.07 to 1.45)	Low	0	Low	1.25 (1.07 to 1.45)	0.016	Low	0	0	Low
**ESKD**	DPP-4 inhibitors *vs* Metformin	0	NA	NA	NA	NA	NA	NA	0.64 (0.14 to 2.91)	Low	0	Low	0.64 (0.14 to 2.91)	0	Low	NA	-2	Very low
**ESKD**	DPP-4 inhibitors *vs* Non-steroidal MRAs	0	NA	NA	NA	NA	NA	NA	1.24 (1.07 to 1.45)	Low	0	Low	1.24 (1.07 to 1.45)	0	Low	NA	0	Low
**ESKD**	DPP-4 inhibitors *vs* SGLT2 inhibitors	0	NA	NA	NA	NA	NA	NA	1.71 (1.47 to 1.99)	Low	0	Low	1.71 (1.47 to 1.99)	0	Low	NA	0	Low
**ESKD**	DPP-4 inhibitors *vs* Standard treatments	9	1.04 (0.93 to 1.16)	0	-1	0	0	Moderate	1.02 (0.40 to 2.60)	Low	0	Low	1.04 (0.93 to 1.16)	0.99	Moderate	0	-1	Low
**ESKD**	DPP-4 inhibitors *vs* Sulfonylureas	3	1.47 (0.76 to 2.83)	0	-1	0	0	Moderate	1.78 (0.45 to 7.00)	Low	0	Low	1.52 (0.84 to 2.75)	0.81	Moderate	0	-2	Very low
**ESKD**	DPP-4 inhibitors *vs* Thiazolidinediones	0	NA	NA	NA	NA	NA	NA	1.50 (0.81 to 2.77)	Low	0	Low	1.50 (0.81 to 2.77)	0	Low	NA	-2	Very low
**ESKD**	DPP-4 inhibitors *vs* Tirzepatide	0	NA	NA	NA	NA	NA	NA	1.54 (0.21 to 11.02)	Low	0	Low	1.54 (0.21 to 11.02)	0	Low	NA	-2	Very low
**ESKD**	GLP-1 receptor agonists *vs* Metformin	0	NA	NA	NA	NA	NA	NA	0.52 (0.11 to 2.34)	Low	0	Low	0.52 (0.11 to 2.34)	0	Low	NA	-2	Very low
**ESKD**	GLP-1 receptor agonists *vs* Non-steroidal MRAs	0	NA	NA	NA	NA	NA	NA	1.00 (0.87 to 1.15)	Low	0	Low	1.00 (0.87 to 1.15)	0	Low	NA	-2	Very low
**ESKD**	GLP-1 receptor agonists *vs* SGLT2 inhibitors	0	NA	NA	NA	NA	NA	NA	1.37 (1.19 to 1.59)	Low	0	Low	1.37 (1.19 to 1.59)	0	Low	NA	0	Low
**ESKD**	GLP-1 receptor agonists *vs* Standard treatments	11	0.83 (0.75 to 0.92)	0	-1	0	0	Moderate	0.76 (0.23 to 2.52)	Low	0	Low	0.83 (0.75 to 0.92)	0.99	Moderate	0	0	Moderate

Outcome	Comparisons	k	Direct estimates	D1	D2	D3	D4	Direct Certainty	Indirect estimates	Initial Indirect Certainty	D5	Indirect Certainty	NMA estimates	Prop	Initial NMA Certainty	D6	D7	NMA Certainty
**ESKD**	GLP-1 receptor agonists *vs* Sulfonylureas	0	NA	NA	NA	NA	NA	NA	1.22 (0.67 to 2.24)	Low	0	Low	1.22 (0.67 to 2.24)	0	Low	NA	-2	Very low
**ESKD**	GLP-1 receptor agonists *vs* Thiazolidinediones	0	NA	NA	NA	NA	NA	NA	1.21 (0.64 to 2.26)	Low	0	Low	1.21 (0.64 to 2.26)	0	Low	NA	-2	Very low
**ESKD**	GLP-1 receptor agonists *vs* Tirzepatide	1	1.00 (0.04 to 24.59)	0	-1	0	0	Moderate	1.40 (0.12 to 16.88)	Low	0	Low	1.23 (0.17 to 8.80)	0.38	Low	0	-2	Very low
**ESKD**	Metformin *vs* Non-steroidal MRAs	0	NA	NA	NA	NA	NA	NA	1.93 (0.43 to 8.71)	Low	0	Low	1.93 (0.43 to 8.71)	0	Low	NA	-2	Very low
**ESKD**	Metformin *vs* SGLT2 inhibitors	0	NA	NA	NA	NA	NA	NA	2.65 (0.59 to 11.96)	Low	0	Low	2.65 (0.59 to 11.96)	0	Low	NA	-2	Very low
**ESKD**	Metformin *vs* Standard treatments	1	1.61 (0.36 to 7.24)	-1	-1	0	0	Low	NA	NA	0	NA	1.61 (0.36 to 7.24)	1	Low	NA	-2	Very low
**ESKD**	Metformin *vs* Sulfonylureas	0	NA	NA	NA	NA	NA	NA	2.36 (0.47 to 11.91)	Low	0	Low	2.36 (0.47 to 11.91)	0	Low	NA	-2	Very low
**ESKD**	Metformin *vs* Thiazolidinediones	0	NA	NA	NA	NA	NA	NA	2.33 (0.46 to 11.83)	Low	0	Low	2.33 (0.46 to 11.83)	0	Low	NA	-2	Very low
**ESKD**	Metformin *vs* Tirzepatide	0	NA	NA	NA	NA	NA	NA	2.38 (0.20 to 28.34)	Low	0	Low	2.38 (0.20 to 28.34)	0	Low	NA	-2	Very low
**ESKD**	Non-steroidal MRAs *vs* SGLT2 inhibitors	0	NA	NA	NA	NA	NA	NA	1.37 (1.19 to 1.58)	Low	0	Low	1.37 (1.19 to 1.58)	0	Low	NA	0	Low
**ESKD**	Non-steroidal MRAs *vs* Standard treatments	2	0.83 (0.75 to 0.92)	0	-1	0	0	Moderate	NA	NA	0	NA	0.83 (0.75 to 0.92)	1	Moderate	NA	0	Moderate
**ESKD**	Non-steroidal MRAs *vs* Sulfonylureas	0	NA	NA	NA	NA	NA	NA	1.22 (0.67 to 2.24)	Low	0	Low	1.22 (0.67 to 2.24)	0	Low	NA	-2	Very low

Outcome	Comparisons	k	Direct estimates	D1	D2	D3	D4	Direct Certainty	Indirect estimates	Initial Indirect Certainty	D5	Indirect Certainty	NMA estimates	Prop	Initial NMA Certainty	D6	D7	NMA Certainty
**ESKD**	Non-steroidal MRAs *vs* Thiazolidinediones	0	NA	NA	NA	NA	NA	NA	1.21 (0.64 to 2.26)	Low	0	Low	1.21 (0.64 to 2.26)	0	Low	NA	-2	Very low
**ESKD**	Non-steroidal MRAs *vs* Tirzepatide	0	NA	NA	NA	NA	NA	NA	1.23 (0.17 to 8.85)	Low	0	Low	1.23 (0.17 to 8.85)	0	Low	NA	-2	Very low
**ESKD**	SGLT2 inhibitors *vs* Standard treatments	15	0.61 (0.55 to 0.67)	0	-1	0	0	Moderate	2.19 (0.08 to 56.93)	Low	0	Low	0.61 (0.55 to 0.67)	1	Moderate	0	0	Moderate
**ESKD**	SGLT2 inhibitors *vs* Sulfonylureas	1	3.06 (0.12 to 75.30)	0	-1	0	0	Moderate	0.85 (0.46 to 1.58)	Low	0	Low	0.89 (0.48 to 1.63)	0.036	Low	0	-2	Very low
**ESKD**	SGLT2 inhibitors *vs* Thiazolidinediones	0	NA	NA	NA	NA	NA	NA	0.88 (0.47 to 1.64)	Low	0	Low	0.88 (0.47 to 1.64)	0	Low	NA	-2	Very low
**ESKD**	SGLT2 inhibitors *vs* Tirzepatide	0	NA	NA	NA	NA	NA	NA	0.90 (0.13 to 6.45)	Low	0	Low	0.90 (0.13 to 6.45)	0	Low	NA	-2	Very low
**ESKD**	Sulfonylureas *vs* Standard treatments	0	NA	NA	NA	NA	NA	NA	0.68 (0.37 to 1.24)	Low	0	Low	0.68 (0.37 to 1.24)	0	Low	NA	-2	Very low
**ESKD**	Thiazolidinediones *vs* Standard treatments	1	0.75 (0.17 to 3.36)	0	-1	0	0	Moderate	0.68 (0.34 to 1.34)	Low	0	Low	0.69 (0.37 to 1.28)	0.17	Low	0	-2	Very low
**ESKD**	Tirzepatide *vs* Standard treatments	0	NA	NA	NA	NA	NA	NA	0.68 (0.09 to 4.84)	Low	0	Low	0.68 (0.09 to 4.84)	0	Low	NA	-2	Very low
**ESKD**	Sulfonylureas *vs* Thiazolidinediones	3	0.99 (0.82 to 1.19)	-1	-1	0	0	Low	0.89 (0.17 to 4.58)	Low	0	Low	0.99 (0.82 to 1.18)	0.99	Low	0	-2	Very low
**ESKD**	Sulfonylureas *vs* Tirzepatide	0	NA	NA	NA	NA	NA	NA	1.01 (0.13 to 7.90)	Low	0	Low	1.01 (0.13 to 7.90)	0	Low	NA	-2	Very low
**ESKD**	Thiazolidinediones *vs* Tirzepatide	0	NA	NA	NA	NA	NA	NA	1.02 (0.13 to 8.05)	Low	0	Low	1.02 (0.13 to 8.05)	0	Low	NA	-2	Very low

Outcome	Comparisons	k	Direct estimates	D1	D2	D3	D4	Direct Certainty	Indirect estimates	Initial Indirect Certainty	D5	Indirect Certainty	NMA estimates	Prop	Initial NMA Certainty	D6	D7	NMA Certainty
**Severe hypoglycaemia**	Alpha-glucosidase inhibitors *vs* Basal insulin	0	NA	NA	NA	NA	NA	NA	0.54 (0.13 to 2.33)	Moderate	0	Moderate	0.54 (0.13 to 2.33)	0	Moderate	NA	-2	Very low
**Severe hypoglycaemia**	Alpha-glucosidase inhibitors *vs* Basal-Bolus insulin	0	NA	NA	NA	NA	NA	NA	0.26 (0.03 to 2.14)	Moderate	0	Moderate	0.26 (0.03 to 2.14)	0	Moderate	NA	-2	Very low
**Severe hypoglycaemia**	Alpha-glucosidase inhibitors *vs* Bolus insulin	0	NA	NA	NA	NA	NA	NA	0.53 (0.11 to 2.52)	Moderate	0	Moderate	0.53 (0.11 to 2.52)	0	Moderate	NA	-2	Very low
**Severe hypoglycaemia**	Alpha-glucosidase inhibitors *vs* DPP-4 inhibitors	0	NA	NA	NA	NA	NA	NA	1.17 (0.28 to 4.90)	Moderate	0	Moderate	1.17 (0.28 to 4.90)	0	Moderate	NA	-2	Very low
**Severe hypoglycaemia**	Alpha-glucosidase inhibitors *vs* GLP-1 receptor agonists	0	NA	NA	NA	NA	NA	NA	1.33 (0.32 to 5.58)	Moderate	0	Moderate	1.33 (0.32 to 5.58)	0	Moderate	NA	-2	Very low
**Severe hypoglycaemia**	Alpha-glucosidase inhibitors *vs* Meglitinides	1	1.01 (0.02 to 51.04)	-2	0	0	0	Low	0.31 (0.04 to 2.47)	Moderate	0	Moderate	0.40 (0.07 to 2.52)	0.22	Moderate	0	-2	Very low
**Severe hypoglycaemia**	Alpha-glucosidase inhibitors *vs* Metformin	0	NA	NA	NA	NA	NA	NA	0.75 (0.16 to 3.58)	Low	0	Low	0.75 (0.16 to 3.58)	0	Low	NA	-2	Very low
**Severe hypoglycaemia**	Alpha-glucosidase inhibitors *vs* Non-steroidal MRAs	0	NA	NA	NA	NA	NA	NA	2.03 (0.46 to 8.98)	Moderate	0	Moderate	2.03 (0.46 to 8.98)	0	Moderate	NA	-2	Very low

Outcome	Comparisons	k	Direct estimates	D1	D2	D3	D4	Direct Certainty	Indirect estimates	Initial Indirect Certainty	D5	Indirect Certainty	NMA estimates	Prop	Initial NMA Certainty	D6	D7	NMA Certainty
**Severe hypoglycaemia**	Alpha-glucosidase inhibitors *vs* SGLT2 inhibitors	1	3.07 (0.12 to 76.55)	-2	0	0	0	Low	1.20 (0.24 to 5.95)	Moderate	0	Moderate	1.45 (0.34 to 6.07)	0.2	Moderate	0	-2	Very low
**Severe hypoglycaemia**	Alpha-glucosidase inhibitors *vs* Standard treatments	2	0.71 (0.11 to 4.56)	0	0	0	0	High	3.12 (0.33 to 29.34)	Moderate	0	Moderate	1.30 (0.31 to 5.43)	0.59	High	0	-2	Low
**Severe hypoglycaemia**	Alpha-glucosidase inhibitors *vs* Sulfonylureas	1	0.67 (0.03 to 16.50)	-2	0	0	0	Low	0.19 (0.04 to 0.98)	Moderate	0	Moderate	0.25 (0.06 to 1.06)	0.21	Moderate	0	-1	Low
**Severe hypoglycaemia**	Alpha-glucosidase inhibitors *vs* Thiazolidinediones	0	NA	NA	NA	NA	NA	NA	0.91 (0.21 to 4.01)	Moderate	0	Moderate	0.91 (0.21 to 4.01)	0	Moderate	NA	-2	Very low
**Severe hypoglycaemia**	Alpha-glucosidase inhibitors *vs* Tirzepatide	0	NA	NA	NA	NA	NA	NA	1.15 (0.20 to 6.56)	Moderate	0	Moderate	1.15 (0.20 to 6.56)	0	Moderate	NA	-2	Very low
**Severe hypoglycaemia**	Basal insulin *vs* Basal-Bolus insulin	1	0.66 (0.11 to 3.99)	-2	0	0	0	Low	0.22 (0.01 to 3.86)	Moderate	0	Moderate	0.48 (0.11 to 2.22)	0.72	Low	0	-2	Very low
**Severe hypoglycaemia**	Basal insulin *vs* Bolus insulin	0	NA	NA	NA	NA	NA	NA	0.97 (0.49 to 1.91)	Low	0	Low	0.97 (0.49 to 1.91)	0	Low	NA	-2	Very low
**Severe hypoglycaemia**	Basal insulin *vs* DPP-4 inhibitors	3	1.99 (0.92 to 4.29)	0	0	0	0	High	2.17 (1.60 to 2.94)	Moderate	0	Moderate	2.14 (1.62 to 2.85)	0.14	Moderate	0	0	Moderate

Outcome	Comparisons	k	Direct estimates	D1	D2	D3	D4	Direct Certainty	Indirect estimates	Initial Indirect Certainty	D5	Indirect Certainty	NMA estimates	Prop	Initial NMA Certainty	D6	D7	NMA Certainty
**Severe hypoglycaemia**	Basal insulin *vs* GLP-1 receptor agonists	15	2.25 (1.70 to 2.99)	-1	0	0	0	Moderate	3.90 (1.98 to 7.68)	Moderate	0	Moderate	2.44 (1.88 to 3.17)	0.85	Moderate	0	0	Moderate
**Severe hypoglycaemia**	Basal insulin *vs* Meglitinides	0	NA	NA	NA	NA	NA	NA	0.74 (0.22 to 2.55)	Moderate	0	Moderate	0.74 (0.22 to 2.55)	0	Moderate	NA	-2	Very low
**Severe hypoglycaemia**	Basal insulin *vs* Metformin	0	NA	NA	NA	NA	NA	NA	1.37 (0.67 to 2.80)	Moderate	0	Moderate	1.37 (0.67 to 2.80)	0	Moderate	NA	-1	Low
**Severe hypoglycaemia**	Basal insulin *vs* Non-steroidal MRAs	0	NA	NA	NA	NA	NA	NA	3.72 (2.28 to 6.08)	Moderate	0	Moderate	3.72 (2.28 to 6.08)	0	Moderate	NA	0	Moderate
**Severe hypoglycaemia**	Basal insulin *vs* SGLT2 inhibitors	1	3.31 (0.13 to 81.76)	-2	0	0	0	Low	2.65 (1.96 to 3.58)	Moderate	0	Moderate	2.66 (1.97 to 3.58)	0.0086	Moderate	0	0	Moderate
**Severe hypoglycaemia**	Basal insulin *vs* Standard treatments	1	0.49 (0.01 to 24.98)	0	0	0	0	High	2.40 (1.83 to 3.15)	Moderate	0	Moderate	2.38 (1.82 to 3.12)	0.0047	Moderate	0	0	Moderate
**Severe hypoglycaemia**	Basal insulin *vs* Sulfonylureas	1	0.56 (0.30 to 1.04)	0	0	0	0	High	0.41 (0.26 to 0.64)	Moderate	0	Moderate	0.46 (0.32 to 0.66)	0.35	Moderate	0	0	Moderate
**Severe hypoglycaemia**	Basal insulin *vs* Thiazolidinediones	2	1.20 (0.37 to 3.89)	0	0	-1	0	Moderate	1.77 (1.09 to 2.89)	Moderate	0	Moderate	1.67 (1.07 to 2.63)	0.15	Moderate	0	0	Moderate

Outcome	Comparisons	k	Direct estimates	D1	D2	D3	D4	Direct Certainty	Indirect estimates	Initial Indirect Certainty	D5	Indirect Certainty	NMA estimates	Prop	Initial NMA Certainty	D6	D7	NMA Certainty
**Severe hypoglycaemia**	Basal insulin *vs* Tirzepatide	2	2.45 (0.83 to 7.23)	0	0	0	0	High	1.19 (0.14 to 10.14)	Moderate	0	Moderate	2.12 (0.81 to 5.56)	0.8	High	0	-1	Moderate
**Severe hypoglycaemia**	Basal-Bolus insulin *vs* Bolus insulin	0	NA	NA	NA	NA	NA	NA	2.01 (0.38 to 10.53)	Low	0	Low	2.01 (0.38 to 10.53)	0	Low	NA	-2	Very low
**Severe hypoglycaemia**	Basal-Bolus insulin *vs* DPP-4 inhibitors	0	NA	NA	NA	NA	NA	NA	4.44 (0.95 to 20.70)	Moderate	0	Moderate	4.44 (0.95 to 20.70)	0	Moderate	NA	-1	Low
**Severe hypoglycaemia**	Basal-Bolus insulin *vs* GLP-1 receptor agonists	1	10.96 (0.64 to 188.62)	-1	0	0	0	Moderate	3.69 (0.60 to 22.81)	Low	0	Low	5.06 (1.09 to 23.49)	0.29	Low	0	0	Low
**Severe hypoglycaemia**	Basal-Bolus insulin *vs* Meglitinides	0	NA	NA	NA	NA	NA	NA	1.54 (0.22 to 10.84)	Moderate	0	Moderate	1.54 (0.22 to 10.84)	0	Moderate	NA	-2	Very low
**Severe hypoglycaemia**	Basal-Bolus insulin *vs* Metformin	0	NA	NA	NA	NA	NA	NA	2.85 (0.54 to 15.16)	Moderate	0	Moderate	2.85 (0.54 to 15.16)	0	Moderate	NA	-2	Very low
**Severe hypoglycaemia**	Basal-Bolus insulin *vs* Non-steroidal MRAs	0	NA	NA	NA	NA	NA	NA	7.71 (1.57 to 37.81)	Moderate	0	Moderate	7.71 (1.57 to 37.81)	0	Moderate	NA	0	Moderate
**Severe hypoglycaemia**	Basal-Bolus insulin *vs* SGLT2 inhibitors	0	NA	NA	NA	NA	NA	NA	5.50 (1.18 to 25.70)	Moderate	0	Moderate	5.50 (1.18 to 25.70)	0	Moderate	NA	0	Moderate

Outcome	Comparisons	k	Direct estimates	D1	D2	D3	D4	Direct Certainty	Indirect estimates	Initial Indirect Certainty	D5	Indirect Certainty	NMA estimates	Prop	Initial NMA Certainty	D6	D7	NMA Certainty
**Severe hypoglycaemia**	Basal-Bolus insulin *vs* Standard treatments	0	NA	NA	NA	NA	NA	NA	4.94 (1.06 to 22.96)	Moderate	0	Moderate	4.94 (1.06 to 22.96)	0	Moderate	NA	0	Moderate
**Severe hypoglycaemia**	Basal-Bolus insulin *vs* Sulfonylureas	0	NA	NA	NA	NA	NA	NA	0.95 (0.20 to 4.50)	Moderate	0	Moderate	0.95 (0.20 to 4.50)	0	Moderate	NA	-2	Very low
**Severe hypoglycaemia**	Basal-Bolus insulin *vs* Thiazolidinediones	0	NA	NA	NA	NA	NA	NA	3.47 (0.72 to 16.84)	Moderate	0	Moderate	3.47 (0.72 to 16.84)	0	Moderate	NA	-1	Low
**Severe hypoglycaemia**	Basal-Bolus insulin *vs* Tirzepatide	0	NA	NA	NA	NA	NA	NA	4.39 (0.72 to 26.62)	Moderate	0	Moderate	4.39 (0.72 to 26.62)	0	Moderate	NA	-1	Low
**Severe hypoglycaemia**	Bolus insulin *vs* DPP-4 inhibitors	0	NA	NA	NA	NA	NA	NA	2.21 (1.17 to 4.20)	Low	0	Low	2.21 (1.17 to 4.20)	0	Low	NA	0	Low
**Severe hypoglycaemia**	Bolus insulin *vs* GLP-1 receptor agonists	7	2.52 (1.35 to 4.72)	-2	0	0	0	Low	NA	NA	0	NA	2.52 (1.35 to 4.72)	1	Low	NA	0	Low
**Severe hypoglycaemia**	Bolus insulin *vs* Meglitinides	0	NA	NA	NA	NA	NA	NA	0.77 (0.20 to 2.99)	Moderate	0	Moderate	0.77 (0.20 to 2.99)	0	Moderate	NA	-2	Very low
**Severe hypoglycaemia**	Bolus insulin *vs* Metformin	0	NA	NA	NA	NA	NA	NA	1.42 (0.57 to 3.54)	Low	0	Low	1.42 (0.57 to 3.54)	0	Low	NA	-2	Very low

Outcome	Comparisons	k	Direct estimates	D1	D2	D3	D4	Direct Certainty	Indirect estimates	Initial Indirect Certainty	D5	Indirect Certainty	NMA estimates	Prop	Initial NMA Certainty	D6	D7	NMA Certainty
**Severe hypoglycaemia**	Bolus insulin *vs* Non-steroidal MRAs	0	NA	NA	NA	NA	NA	NA	3.84 (1.81 to 8.16)	Low	0	Low	3.84 (1.81 to 8.16)	0	Low	NA	0	Low
**Severe hypoglycaemia**	Bolus insulin *vs* SGLT2 inhibitors	0	NA	NA	NA	NA	NA	NA	2.74 (1.44 to 5.22)	Moderate	0	Moderate	2.74 (1.44 to 5.22)	0	Moderate	NA	0	Moderate
**Severe hypoglycaemia**	Bolus insulin *vs* Standard treatments	0	NA	NA	NA	NA	NA	NA	2.46 (1.31 to 4.63)	Moderate	0	Moderate	2.46 (1.31 to 4.63)	0	Moderate	NA	0	Moderate
**Severe hypoglycaemia**	Bolus insulin *vs* Sulfonylureas	0	NA	NA	NA	NA	NA	NA	0.47 (0.24 to 0.94)	Moderate	0	Moderate	0.47 (0.24 to 0.94)	0	Moderate	NA	0	Moderate
**Severe hypoglycaemia**	Bolus insulin *vs* Thiazolidinediones	0	NA	NA	NA	NA	NA	NA	1.73 (0.82 to 3.62)	Low	0	Low	1.73 (0.82 to 3.62)	0	Low	NA	-1	Very low
**Severe hypoglycaemia**	Bolus insulin *vs* Tirzepatide	0	NA	NA	NA	NA	NA	NA	2.19 (0.68 to 7.03)	Moderate	0	Moderate	2.19 (0.68 to 7.03)	0	Moderate	NA	-1	Low
**Severe hypoglycaemia**	DPP-4 inhibitors *vs* GLP-1 receptor agonists	7	1.35 (0.96 to 1.92)	-1	0	0	0	Moderate	1.11 (0.96 to 1.27)	Moderate	0	Moderate	1.14 (1.00 to 1.30)	0.14	Moderate	0	0	Moderate
**Severe hypoglycaemia**	DPP-4 inhibitors *vs* Meglitinides	0	NA	NA	NA	NA	NA	NA	0.35 (0.10 to 1.16)	Moderate	0	Moderate	0.35 (0.10 to 1.16)	0	Moderate	NA	-1	Low

Outcome	Comparisons	k	Direct estimates	D1	D2	D3	D4	Direct Certainty	Indirect estimates	Initial Indirect Certainty	D5	Indirect Certainty	NMA estimates	Prop	Initial NMA Certainty	D6	D7	NMA Certainty
**Severe hypoglycaemia**	DPP-4 inhibitors *vs* Metformin	2	1.02 (0.12 to 8.77)	0	0	-1	0	Moderate	0.61 (0.30 to 1.23)	Low	0	Low	0.64 (0.33 to 1.25)	0.096	Low	0	-1	Very low
**Severe hypoglycaemia**	DPP-4 inhibitors *vs* Non-steroidal MRAs	0	NA	NA	NA	NA	NA	NA	1.74 (1.14 to 2.65)	Moderate	0	Moderate	1.74 (1.14 to 2.65)	0	Moderate	NA	0	Moderate
**Severe hypoglycaemia**	DPP-4 inhibitors *vs* SGLT2 inhibitors	3	0.64 (0.26 to 1.58)	0	0	0	0	High	1.27 (1.07 to 1.50)	Moderate	0	Moderate	1.24 (1.05 to 1.46)	0.034	Moderate	0	-1	Low
**Severe hypoglycaemia**	DPP-4 inhibitors *vs* Standard treatments	41	1.10 (0.99 to 1.24)	-1	0	0	0	Moderate	1.16 (0.87 to 1.54)	Moderate	0	Moderate	1.11 (1.00 to 1.23)	0.87	Moderate	0	0	Moderate
**Severe hypoglycaemia**	DPP-4 inhibitors *vs* Sulfonylureas	16	0.19 (0.13 to 0.27)	0	0	0	0	High	0.26 (0.16 to 0.42)	Moderate	0	Moderate	0.21 (0.16 to 0.29)	0.63	High	0	0	High
**Severe hypoglycaemia**	DPP-4 inhibitors *vs* Thiazolidinediones	3	2.66 (0.60 to 11.87)	-1	0	0	0	Moderate	0.71 (0.47 to 1.07)	Moderate	0	Moderate	0.78 (0.52 to 1.16)	0.071	Moderate	0	-1	Low
**Severe hypoglycaemia**	DPP-4 inhibitors *vs* Tirzepatide	0	NA	NA	NA	NA	NA	NA	0.99 (0.37 to 2.66)	Moderate	0	Moderate	0.99 (0.37 to 2.66)	0	Moderate	NA	-2	Very low
**Severe hypoglycaemia**	GLP-1 receptor agonists *vs* Meglitinides	0	NA	NA	NA	NA	NA	NA	0.30 (0.09 to 1.02)	Moderate	0	Moderate	0.30 (0.09 to 1.02)	0	Moderate	NA	-1	Low

Outcome	Comparisons	k	Direct estimates	D1	D2	D3	D4	Direct Certainty	Indirect estimates	Initial Indirect Certainty	D5	Indirect Certainty	NMA estimates	Prop	Initial NMA Certainty	D6	D7	NMA Certainty
**Severe hypoglycaemia**	GLP-1 receptor agonists *vs* Metformin	0	NA	NA	NA	NA	NA	NA	0.56 (0.29 to 1.10)	Moderate	0	Moderate	0.56 (0.29 to 1.10)	0	Moderate	NA	-1	Low
**Severe hypoglycaemia**	GLP-1 receptor agonists *vs* Non-steroidal MRAs	0	NA	NA	NA	NA	NA	NA	1.52 (1.00 to 2.31)	Moderate	0	Moderate	1.52 (1.00 to 2.31)	0	Moderate	NA	0	Moderate
**Severe hypoglycaemia**	GLP-1 receptor agonists *vs* SGLT2 inhibitors	2	1.17 (0.54 to 2.55)	-1	0	0	0	Moderate	1.08 (0.93 to 1.27)	Moderate	0	Moderate	1.09 (0.93 to 1.27)	0.039	Moderate	0	0	Moderate
**Severe hypoglycaemia**	GLP-1 receptor agonists *vs* Standard treatments	38	0.98 (0.89 to 1.07)	0	0	0	0	High	0.97 (0.72 to 1.30)	Moderate	0	Moderate	0.98 (0.90 to 1.06)	0.92	High	0	0	High
**Severe hypoglycaemia**	GLP-1 receptor agonists *vs* Sulfonylureas	3	0.47 (0.25 to 0.91)	-1	0	0	0	Moderate	0.15 (0.10 to 0.20)	Moderate	0	Moderate	0.19 (0.14 to 0.25)	0.21	Moderate	-2	0	Very low
**Severe hypoglycaemia**	GLP-1 receptor agonists *vs* Thiazolidinediones	2	0.54 (0.10 to 2.82)	-1	0	0	0	Moderate	0.70 (0.46 to 1.04)	Moderate	0	Moderate	0.69 (0.46 to 1.02)	0.057	Moderate	0	-1	Low
**Severe hypoglycaemia**	GLP-1 receptor agonists *vs* Tirzepatide	1	0.60 (0.03 to 12.51)	0	0	0	0	High	0.91 (0.32 to 2.57)	Moderate	0	Moderate	0.87 (0.32 to 2.32)	0.11	Moderate	0	-2	Very low
**Severe hypoglycaemia**	Meglitinides *vs* Metformin	1	2.34 (0.66 to 8.34)	0	0	0	0	High	0.86 (0.09 to 8.54)	Low	0	Low	1.85 (0.61 to 5.63)	0.77	High	0	-2	Low

Outcome	Comparisons	k	Direct estimates	D1	D2	D3	D4	Direct Certainty	Indirect estimates	Initial Indirect Certainty	D5	Indirect Certainty	NMA estimates	Prop	Initial NMA Certainty	D6	D7	NMA Certainty
**Severe hypoglycaemia**	Meglitinides *vs* Non-steroidal MRAs	0	NA	NA	NA	NA	NA	NA	5.01 (1.40 to 17.96)	Moderate	0	Moderate	5.01 (1.40 to 17.96)	0	Moderate	NA	0	Moderate
**Severe hypoglycaemia**	Meglitinides *vs* SGLT2 inhibitors	0	NA	NA	NA	NA	NA	NA	3.58 (1.06 to 12.02)	Moderate	0	Moderate	3.58 (1.06 to 12.02)	0	Moderate	NA	0	Moderate
**Severe hypoglycaemia**	Meglitinides *vs* Standard treatments	0	NA	NA	NA	NA	NA	NA	3.21 (0.96 to 10.75)	Moderate	0	Moderate	3.21 (0.96 to 10.75)	0	Moderate	NA	-1	Low
**Severe hypoglycaemia**	Meglitinides *vs* Sulfonylureas	2	0.34 (0.04 to 3.11)	-2	0	0	0	Low	0.80 (0.18 to 3.42)	Moderate	0	Moderate	0.62 (0.18 to 2.08)	0.3	Moderate	0	-2	Very low
**Severe hypoglycaemia**	Meglitinides *vs* Thiazolidinediones	0	NA	NA	NA	NA	NA	NA	2.26 (0.64 to 7.99)	Moderate	0	Moderate	2.26 (0.64 to 7.99)	0	Moderate	NA	-2	Very low
**Severe hypoglycaemia**	Meglitinides *vs* Tirzepatide	0	NA	NA	NA	NA	NA	NA	2.85 (0.60 to 13.55)	Moderate	0	Moderate	2.85 (0.60 to 13.55)	0	Moderate	NA	-2	Very low
**Severe hypoglycaemia**	Metformin *vs* Non-steroidal MRAs	0	NA	NA	NA	NA	NA	NA	2.71 (1.24 to 5.90)	Low	0	Low	2.71 (1.24 to 5.90)	0	Low	NA	0	Low
**Severe hypoglycaemia**	Metformin *vs* SGLT2 inhibitors	2	3.66 (0.87 to 15.44)	0	0	0	0	High	1.62 (0.76 to 3.45)	Moderate	0	Moderate	1.93 (0.99 to 3.78)	0.22	Moderate	0	-1	Low

Outcome	Comparisons	k	Direct estimates	D1	D2	D3	D4	Direct Certainty	Indirect estimates	Initial Indirect Certainty	D5	Indirect Certainty	NMA estimates	Prop	Initial NMA Certainty	D6	D7	NMA Certainty
**Severe hypoglycaemia**	Metformin *vs* Standard treatments	2	1.65 (0.64 to 4.25)	-1	0	-1	0	Low	1.82 (0.72 to 4.61)	Moderate	0	Moderate	1.73 (0.89 to 3.37)	0.49	Moderate	0	-1	Low
**Severe hypoglycaemia**	Metformin *vs* Sulfonylureas	2	0.31 (0.05 to 1.78)	-1	0	0	0	Moderate	0.34 (0.16 to 0.72)	Moderate	0	Moderate	0.33 (0.17 to 0.67)	0.16	Moderate	0	0	Moderate
**Severe hypoglycaemia**	Metformin *vs* Thiazolidinediones	1	1.00 (0.06 to 16.02)	0	0	0	0	High	1.24 (0.56 to 2.74)	Low	0	Low	1.22 (0.57 to 2.61)	0.076	Low	0	-2	Very low
**Severe hypoglycaemia**	Metformin *vs* Tirzepatide	0	NA	NA	NA	NA	NA	NA	1.54 (0.47 to 5.06)	Moderate	0	Moderate	1.54 (0.47 to 5.06)	0	Moderate	NA	-2	Very low
**Severe hypoglycaemia**	Non-steroidal MRAs *vs* SGLT2 inhibitors	0	NA	NA	NA	NA	NA	NA	0.71 (0.46 to 1.10)	Moderate	0	Moderate	0.71 (0.46 to 1.10)	0	Moderate	NA	-1	Low
**Severe hypoglycaemia**	Non-steroidal MRAs *vs* Standard treatments	2	0.64 (0.43 to 0.96)	0	-1	0	0	Moderate	NA	NA	0	NA	0.64 (0.43 to 0.96)	1	Moderate	NA	0	Moderate
**Severe hypoglycaemia**	Non-steroidal MRAs *vs* Sulfonylureas	0	NA	NA	NA	NA	NA	NA	0.12 (0.07 to 0.20)	Moderate	0	Moderate	0.12 (0.07 to 0.20)	0	Moderate	NA	0	Moderate
**Severe hypoglycaemia**	Non-steroidal MRAs *vs* Thiazolidinediones	0	NA	NA	NA	NA	NA	NA	0.45 (0.26 to 0.79)	Moderate	0	Moderate	0.45 (0.26 to 0.79)	0	Moderate	NA	0	Moderate

Outcome	Comparisons	k	Direct estimates	D1	D2	D3	D4	Direct Certainty	Indirect estimates	Initial Indirect Certainty	D5	Indirect Certainty	NMA estimates	Prop	Initial NMA Certainty	D6	D7	NMA Certainty
**Severe hypoglycaemia**	Non-steroidal MRAs *vs* Tirzepatide	0	NA	NA	NA	NA	NA	NA	0.57 (0.20 to 1.66)	Moderate	0	Moderate	0.57 (0.20 to 1.66)	0	Moderate	NA	-2	Very low
**Severe hypoglycaemia**	SGLT2 inhibitors *vs* Standard treatments	28	0.89 (0.78 to 1.02)	0	0	0	0	High	0.95 (0.59 to 1.54)	Moderate	0	Moderate	0.90 (0.79 to 1.02)	0.92	High	0	0	High
**Severe hypoglycaemia**	SGLT2 inhibitors *vs* Sulfonylureas	3	0.17 (0.07 to 0.43)	0	0	0	0	High	0.17 (0.12 to 0.24)	Moderate	0	Moderate	0.17 (0.13 to 0.24)	0.12	Moderate	0	0	Moderate
**Severe hypoglycaemia**	SGLT2 inhibitors *vs* Thiazolidinediones	0	NA	NA	NA	NA	NA	NA	0.63 (0.42 to 0.95)	Moderate	0	Moderate	0.63 (0.42 to 0.95)	0	Moderate	NA	0	Moderate
**Severe hypoglycaemia**	SGLT2 inhibitors *vs* Tirzepatide	0	NA	NA	NA	NA	NA	NA	0.80 (0.30 to 2.16)	Moderate	0	Moderate	0.80 (0.30 to 2.16)	0	Moderate	NA	-2	Very low
**Severe hypoglycaemia**	Sulfonylureas *vs* Standard treatments	2	1.42 (0.12 to 17.07)	-1	0	0	0	Moderate	5.31 (3.95 to 7.15)	Moderate	0	Moderate	5.22 (3.88 to 7.01)	0.014	Moderate	0	0	Moderate
**Severe hypoglycaemia**	Thiazolidinediones *vs* Standard treatments	10	1.77 (1.10 to 2.85)	-1	0	0	0	Moderate	0.92 (0.47 to 1.80)	Moderate	0	Moderate	1.42 (0.97 to 2.10)	0.67	Moderate	0	-1	Low
**Severe hypoglycaemia**	Tirzepatide *vs* Standard treatments	1	2.39 (0.12 to 46.66)	0	0	0	0	High	1.02 (0.36 to 2.92)	Moderate	0	Moderate	1.13 (0.42 to 3.02)	0.11	Moderate	0	-2	Very low

Outcome	Comparisons	k	Direct estimates	D1	D2	D3	D4	Direct Certainty	Indirect estimates	Initial Indirect Certainty	D5	Indirect Certainty	NMA estimates	Prop	Initial NMA Certainty	D6	D7	NMA Certainty
**Severe hypoglycaemia**	Sulfonylureas *vs* Thiazolidinediones	6	8.79 (3.06 to 25.28)	-1	0	0	0	Moderate	2.99 (1.79 to 4.98)	Moderate	0	Moderate	3.66 (2.31 to 5.80)	0.19	Moderate	0	0	Moderate
**Severe hypoglycaemia**	Sulfonylureas *vs* Tirzepatide	0	NA	NA	NA	NA	NA	NA	4.64 (1.67 to 12.86)	Moderate	0	Moderate	4.64 (1.67 to 12.86)	0	Moderate	NA	0	Moderate
**Severe hypoglycaemia**	Thiazolidinediones *vs* Tirzepatide	0	NA	NA	NA	NA	NA	NA	1.27 (0.44 to 3.63)	Moderate	0	Moderate	1.27 (0.44 to 3.63)	0	Moderate	NA	-2	Very low
**Severe gastrointestinal events**	Alpha-glucosidase inhibitors *vs* Basal insulin	0	NA	NA	NA	NA	NA	NA	8.74 (0.58 to 131.74)	Moderate	0	Moderate	8.74 (0.58 to 131.74)	0	Moderate	NA	-2	Very low
**Severe gastrointestinal events**	Alpha-glucosidase inhibitors *vs* Bolus insulin	0	NA	NA	NA	NA	NA	NA	1.03 (0.05 to 20.56)	Low	0	Low	1.03 (0.05 to 20.56)	0	Low	NA	-2	Very low
**Severe gastrointestinal events**	Alpha-glucosidase inhibitors *vs* DPP-4 inhibitors	0	NA	NA	NA	NA	NA	NA	2.96 (0.24 to 36.24)	Moderate	0	Moderate	2.96 (0.24 to 36.24)	0	Moderate	NA	-2	Very low
**Severe gastrointestinal events**	Alpha-glucosidase inhibitors *vs* GLP-1 receptor agonists	0	NA	NA	NA	NA	NA	NA	1.72 (0.15 to 19.74)	Moderate	0	Moderate	1.72 (0.15 to 19.74)	0	Moderate	NA	-2	Very low
**Severe gastrointestinal events**	Alpha-glucosidase inhibitors *vs* Metformin	1	1.53 (0.19 to 12.17)	-1	0	0	0	Moderate	NA	NA	0	NA	1.53 (0.19 to 12.17)	1	Moderate	NA	-2	Very low

Outcome	Comparisons	k	Direct estimates	D1	D2	D3	D4	Direct Certainty	Indirect estimates	Initial Indirect Certainty	D5	Indirect Certainty	NMA estimates	Prop	Initial NMA Certainty	D6	D7	NMA Certainty
**Severe gastrointestinal events**	Alpha-glucosidase inhibitors *vs* Standard treatments	0	NA	NA	NA	NA	NA	NA	3.40 (0.30 to 38.15)	Moderate	0	Moderate	3.40 (0.30 to 38.15)	0	Moderate	NA	-2	Very low
**Severe gastrointestinal events**	Alpha-glucosidase inhibitors *vs* Sulfonylureas	0	NA	NA	NA	NA	NA	NA	3.43 (0.28 to 42.63)	Moderate	0	Moderate	3.43 (0.28 to 42.63)	0	Moderate	NA	-2	Very low
**Severe gastrointestinal events**	Alpha-glucosidase inhibitors *vs* Thiazolidinediones	0	NA	NA	NA	NA	NA	NA	2.38 (0.22 to 25.43)	Low	0	Low	2.38 (0.22 to 25.43)	0	Low	NA	-2	Very low
**Severe gastrointestinal events**	Alpha-glucosidase inhibitors *vs* Tirzepatide	0	NA	NA	NA	NA	NA	NA	0.74 (0.06 to 9.69)	Moderate	0	Moderate	0.74 (0.06 to 9.69)	0	Moderate	NA	-2	Very low
**Severe gastrointestinal events**	Basal insulin *vs* Bolus insulin	0	NA	NA	NA	NA	NA	NA	0.12 (0.01 to 0.98)	Low	0	Low	0.12 (0.01 to 0.98)	0	Low	NA	0	Low
**Severe gastrointestinal events**	Basal insulin *vs* DPP-4 inhibitors	1	1.11 (0.06 to 21.16)	0	0	0	0	High	0.24 (0.05 to 1.15)	Low	0	Low	0.34 (0.09 to 1.34)	0.22	Low	0	-2	Very low
**Severe gastrointestinal events**	Basal insulin *vs* GLP-1 receptor agonists	1	0.30 (0.05 to 1.81)	-2	0	0	0	Low	0.13 (0.03 to 0.72)	Moderate	0	Moderate	0.20 (0.06 to 0.67)	0.47	Moderate	0	0	Moderate
**Severe gastrointestinal events**	Basal insulin *vs* Metformin	0	NA	NA	NA	NA	NA	NA	0.18 (0.03 to 1.01)	Moderate	0	Moderate	0.18 (0.03 to 1.01)	0	Moderate	NA	-1	Low

Outcome	Comparisons	k	Direct estimates	D1	D2	D3	D4	Direct Certainty	Indirect estimates	Initial Indirect Certainty	D5	Indirect Certainty	NMA estimates	Prop	Initial NMA Certainty	D6	D7	NMA Certainty
**Severe gastrointestinal events**	Basal insulin *vs* Standard treatments	0	NA	NA	NA	NA	NA	NA	0.39 (0.11 to 1.36)	Moderate	0	Moderate	0.39 (0.11 to 1.36)	0	Moderate	NA	-2	Very low
**Severe gastrointestinal events**	Basal insulin *vs* Sulfonylureas	0	NA	NA	NA	NA	NA	NA	0.39 (0.07 to 2.30)	Moderate	0	Moderate	0.39 (0.07 to 2.30)	0	Moderate	NA	-2	Very low
**Severe gastrointestinal events**	Basal insulin *vs* Thiazolidinediones	0	NA	NA	NA	NA	NA	NA	0.27 (0.06 to 1.16)	Moderate	0	Moderate	0.27 (0.06 to 1.16)	0	Moderate	NA	-1	Low
**Severe gastrointestinal events**	Basal insulin *vs* Tirzepatide	2	0.04 (0.01 to 0.21)	0	0	0	0	High	0.20 (0.03 to 1.23)	Moderate	0	Moderate	0.08 (0.02 to 0.30)	0.51	High	0	0	High
**Severe gastrointestinal events**	Bolus insulin *vs* DPP-4 inhibitors	0	NA	NA	NA	NA	NA	NA	2.88 (0.43 to 19.34)	Low	0	Low	2.88 (0.43 to 19.34)	0	Low	NA	-2	Very low
**Severe gastrointestinal events**	Bolus insulin *vs* GLP-1 receptor agonists	1	1.67 (0.29 to 9.47)	-2	0	0	0	Low	NA	NA	0	NA	1.67 (0.29 to 9.47)	1	Low	NA	-2	Very low
**Severe gastrointestinal events**	Bolus insulin *vs* Metformin	0	NA	NA	NA	NA	NA	NA	1.48 (0.17 to 12.84)	Low	0	Low	1.48 (0.17 to 12.84)	0	Low	NA	-2	Very low
**Severe gastrointestinal events**	Bolus insulin *vs* Standard treatments	0	NA	NA	NA	NA	NA	NA	3.30 (0.56 to 19.35)	Low	0	Low	3.30 (0.56 to 19.35)	0	Low	NA	-2	Very low

Outcome	Comparisons	k	Direct estimates	D1	D2	D3	D4	Direct Certainty	Indirect estimates	Initial Indirect Certainty	D5	Indirect Certainty	NMA estimates	Prop	Initial NMA Certainty	D6	D7	NMA Certainty
**Severe gastrointestinal events**	Bolus insulin *vs* Sulfonylureas	0	NA	NA	NA	NA	NA	NA	3.33 (0.37 to 29.63)	Low	0	Low	3.33 (0.37 to 29.63)	0	Low	NA	-2	Very low
**Severe gastrointestinal events**	Bolus insulin *vs* Thiazolidinediones	0	NA	NA	NA	NA	NA	NA	2.31 (0.34 to 15.78)	Low	0	Low	2.31 (0.34 to 15.78)	0	Low	NA	-2	Very low
**Severe gastrointestinal events**	Bolus insulin *vs* Tirzepatide	0	NA	NA	NA	NA	NA	NA	0.72 (0.10 to 4.97)	Low	0	Low	0.72 (0.10 to 4.97)	0	Low	NA	-2	Very low
**Severe gastrointestinal events**	DPP-4 inhibitors *vs* GLP-1 receptor agonists	2	0.62 (0.21 to 1.80)	-1	0	-1	0	Low	0.54 (0.17 to 1.73)	Moderate	0	Moderate	0.58 (0.26 to 1.28)	0.54	Low	0	-2	Very low
**Severe gastrointestinal events**	DPP-4 inhibitors *vs* Metformin	0	NA	NA	NA	NA	NA	NA	0.52 (0.13 to 2.10)	Moderate	0	Moderate	0.52 (0.13 to 2.10)	0	Moderate	NA	-2	Very low
**Severe gastrointestinal events**	DPP-4 inhibitors *vs* Standard treatments	5	1.75 (0.45 to 6.77)	-1	0	0	0	Moderate	0.91 (0.34 to 2.46)	Low	0	Low	1.15 (0.52 to 2.55)	0.35	Low	0	-2	Very low
**Severe gastrointestinal events**	DPP-4 inhibitors *vs* Sulfonylureas	1	0.72 (0.12 to 4.32)	-1	0	0	0	Moderate	2.02 (0.29 to 14.08)	Moderate	0	Moderate	1.16 (0.31 to 4.32)	0.54	Moderate	0	-2	Very low
**Severe gastrointestinal events**	DPP-4 inhibitors *vs* Thiazolidinediones	0	NA	NA	NA	NA	NA	NA	0.80 (0.28 to 2.29)	Moderate	0	Moderate	0.80 (0.28 to 2.29)	0	Moderate	NA	-2	Very low

Outcome	Comparisons	k	Direct estimates	D1	D2	D3	D4	Direct Certainty	Indirect estimates	Initial Indirect Certainty	D5	Indirect Certainty	NMA estimates	Prop	Initial NMA Certainty	D6	D7	NMA Certainty
**Severe gastrointestinal events**	DPP-4 inhibitors *vs* Tirzepatide	0	NA	NA	NA	NA	NA	NA	0.25 (0.08 to 0.78)	Moderate	0	Moderate	0.25 (0.08 to 0.78)	0	Moderate	NA	0	Moderate
**Severe gastrointestinal events**	GLP-1 receptor agonists *vs* Metformin	0	NA	NA	NA	NA	NA	NA	0.89 (0.25 to 3.21)	Moderate	0	Moderate	0.89 (0.25 to 3.21)	0	Moderate	NA	-2	Very low
**Severe gastrointestinal events**	GLP-1 receptor agonists *vs* Standard treatments	15	1.91 (1.33 to 2.76)	0	0	-1	0	Moderate	2.83 (0.81 to 9.91)	Moderate	0	Moderate	1.97 (1.39 to 2.80)	0.92	Moderate	0	0	Moderate
**Severe gastrointestinal events**	GLP-1 receptor agonists *vs* Sulfonylureas	0	NA	NA	NA	NA	NA	NA	1.99 (0.53 to 7.54)	Moderate	0	Moderate	1.99 (0.53 to 7.54)	0	Moderate	NA	-2	Very low
**Severe gastrointestinal events**	GLP-1 receptor agonists *vs* Thiazolidinediones	0	NA	NA	NA	NA	NA	NA	1.38 (0.61 to 3.15)	Moderate	0	Moderate	1.38 (0.61 to 3.15)	0	Moderate	NA	-2	Very low
**Severe gastrointestinal events**	GLP-1 receptor agonists *vs* Tirzepatide	2	0.67 (0.23 to 2.00)	0	0	0	0	High	0.21 (0.05 to 0.84)	Moderate	0	Moderate	0.43 (0.18 to 1.01)	0.62	High	0	-1	Moderate
**Severe gastrointestinal events**	Metformin *vs* Standard treatments	1	1.99 (0.15 to 26.77)	0	0	0	0	High	2.29 (0.56 to 9.47)	Moderate	0	Moderate	2.22 (0.64 to 7.71)	0.23	Moderate	0	-2	Very low
**Severe gastrointestinal events**	Metformin *vs* Sulfonylureas	1	2.32 (0.44 to 12.31)	0	0	0	0	High	2.04 (0.12 to 33.17)	Moderate	0	Moderate	2.24 (0.53 to 9.39)	0.74	High	0	-2	Low

Outcome	Comparisons	k	Direct estimates	D1	D2	D3	D4	Direct Certainty	Indirect estimates	Initial Indirect Certainty	D5	Indirect Certainty	NMA estimates	Prop	Initial NMA Certainty	D6	D7	NMA Certainty
**Severe gastrointestinal events**	Metformin *vs* Thiazolidinediones	2	1.42 (0.40 to 5.13)	-1	0	-1	0	Low	2.21 (0.18 to 27.38)	Moderate	0	Moderate	1.56 (0.50 to 4.88)	0.79	Low	0	-2	Very low
**Severe gastrointestinal events**	Metformin *vs* Tirzepatide	0	NA	NA	NA	NA	NA	NA	0.48 (0.11 to 2.21)	Moderate	0	Moderate	0.48 (0.11 to 2.21)	0	Moderate	NA	-2	Very low
**Severe gastrointestinal events**	Sulfonylureas *vs* Standard treatments	0	NA	NA	NA	NA	NA	NA	0.99 (0.27 to 3.68)	Moderate	0	Moderate	0.99 (0.27 to 3.68)	0	Moderate	NA	-2	Very low
**Severe gastrointestinal events**	Thiazolidinediones *vs* Standard treatments	2	1.31 (0.58 to 2.95)	0	0	0	0	High	2.43 (0.32 to 18.28)	Moderate	0	Moderate	1.43 (0.67 to 3.03)	0.86	High	0	-2	Low
**Severe gastrointestinal events**	Tirzepatide *vs* Standard treatments	3	5.73 (1.17 to 27.99)	0	0	0	0	High	4.14 (1.42 to 12.08)	Moderate	0	Moderate	4.59 (1.89 to 11.14)	0.31	Moderate	0	0	Moderate
**Severe gastrointestinal events**	Sulfonylureas *vs* Thiazolidinediones	1	0.38 (0.07 to 1.96)	0	0	0	0	High	1.83 (0.23 to 14.55)	Moderate	0	Moderate	0.70 (0.19 to 2.53)	0.61	High	0	-2	Low
**Severe gastrointestinal events**	Sulfonylureas *vs* Tirzepatide	0	NA	NA	NA	NA	NA	NA	0.22 (0.05 to 1.03)	Moderate	0	Moderate	0.22 (0.05 to 1.03)	0	Moderate	NA	-1	Low
**Severe gastrointestinal events**	Thiazolidinediones *vs* Tirzepatide	0	NA	NA	NA	NA	NA	NA	0.31 (0.10 to 0.99)	Moderate	0	Moderate	0.31 (0.10 to 0.99)	0	Moderate	NA	0	Moderate

Outcome	Comparisons	k	Direct estimates	D1	D2	D3	D4	Direct Certainty	Indirect estimates	Initial Indirect Certainty	D5	Indirect Certainty	NMA estimates	Prop	Initial NMA Certainty	D6	D7	NMA Certainty
**Genital infection**	DPP-4 inhibitors *vs* GLP-1 receptor agonists	0	NA	NA	NA	NA	NA	NA	1.03 (0.47 to 2.23)	Moderate	0	Moderate	1.03 (0.47 to 2.23)	0	Moderate	NA	-2	Very low
**Genital infection**	DPP-4 inhibitors *vs* Metformin	0	NA	NA	NA	NA	NA	NA	0.55 (0.26 to 1.16)	Moderate	0	Moderate	0.55 (0.26 to 1.16)	0	Moderate	NA	-2	Very low
**Genital infection**	DPP-4 inhibitors *vs* SGLT2 inhibitors	10	0.26 (0.16 to 0.42)	-1	0	0	0	Moderate	0.12 (0.05 to 0.29)	Moderate	0	Moderate	0.22 (0.14 to 0.33)	0.77	Moderate	0	0	Moderate
**Genital infection**	DPP-4 inhibitors *vs* Standard treatments	6	0.47 (0.20 to 1.10)	0	0	0	0	High	0.83 (0.50 to 1.37)	Moderate	0	Moderate	0.71 (0.46 to 1.10)	0.26	Moderate	0	-1	Low
**Genital infection**	DPP-4 inhibitors *vs* Sulfonylureas	0	NA	NA	NA	NA	NA	NA	1.36 (0.80 to 2.33)	Moderate	0	Moderate	1.36 (0.80 to 2.33)	0	Moderate	NA	-2	Very low
**Genital infection**	DPP-4 inhibitors *vs* Thiazolidinediones	0	NA	NA	NA	NA	NA	NA	0.07 (0.00 to 1.72)	Moderate	0	Moderate	0.07 (0.00 to 1.72)	0	Moderate	NA	-2	Very low
**Genital infection**	GLP-1 receptor agonists *vs* Metformin	0	NA	NA	NA	NA	NA	NA	0.53 (0.22 to 1.31)	Moderate	0	Moderate	0.53 (0.22 to 1.31)	0	Moderate	NA	-2	Very low
**Genital infection**	GLP-1 receptor agonists *vs* SGLT2 inhibitors	2	0.18 (0.09 to 0.38)	-1	0	0	0	Moderate	0.37 (0.09 to 1.59)	Moderate	0	Moderate	0.21 (0.11 to 0.40)	0.8	Moderate	0	0	Moderate
**Genital infection**	GLP-1 receptor agonists *vs* Standard treatments	4	1.22 (0.29 to 5.19)	0	0	0	0	High	0.60 (0.29 to 1.25)	Moderate	0	Moderate	0.69 (0.36 to 1.34)	0.21	Moderate	0	-2	Very low
**Genital infection**	GLP-1 receptor agonists *vs* Sulfonylureas	0	NA	NA	NA	NA	NA	NA	1.33 (0.64 to 2.76)	Moderate	0	Moderate	1.33 (0.64 to 2.76)	0	Moderate	NA	-2	Very low
**Genital infection**	GLP-1 receptor agonists *vs* Thiazolidinediones	0	NA	NA	NA	NA	NA	NA	0.06 (0.00 to 1.73)	Moderate	0	Moderate	0.06 (0.00 to 1.73)	0	Moderate	NA	-2	Very low
**Genital infection**	Metformin *vs* SGLT2 inhibitors	4	0.39 (0.21 to 0.73)	0	0	0	0	High	NA	NA	0	NA	0.39 (0.21 to 0.73)	1	High	NA	0	High

Outcome	Comparisons	k	Direct estimates	D1	D2	D3	D4	Direct Certainty	Indirect estimates	Initial Indirect Certainty	D5	Indirect Certainty	NMA estimates	Prop	Initial NMA Certainty	D6	D7	NMA Certainty
**Genital infection**	Metformin *vs* Standard treatments	0	NA	NA	NA	NA	NA	NA	1.30 (0.69 to 2.45)	Moderate	0	Moderate	1.30 (0.69 to 2.45)	0	Moderate	NA	-2	Very low
**Genital infection**	Metformin *vs* Sulfonylureas	0	NA	NA	NA	NA	NA	NA	2.49 (1.24 to 5.04)	High	0	High	2.49 (1.24 to 5.04)	0	High	NA	0	High
**Genital infection**	Metformin *vs* Thiazolidinediones	0	NA	NA	NA	NA	NA	NA	0.12 (0.00 to 3.24)	High	0	High	0.12 (0.00 to 3.24)	0	High	NA	-2	Low
**Genital infection**	SGLT2 inhibitors *vs* Standard treatments	63	3.33 (2.91 to 3.82)	0	0	0	0	High	2.27 (0.97 to 5.31)	Moderate	0	Moderate	3.30 (2.88 to 3.78)	0.97	High	0	0	High
**Genital infection**	SGLT2 inhibitors *vs* Sulfonylureas	6	6.32 (4.53 to 8.82)	0	0	0	0	High	NA	NA	0	NA	6.32 (4.53 to 8.82)	1	High	NA	0	High
**Genital infection**	SGLT2 inhibitors *vs* Thiazolidinediones	1	0.30 (0.01 to 7.75)	0	0	0	0	High	NA	NA	0	NA	0.30 (0.01 to 7.75)	1	High	NA	-2	Low
**Genital infection**	Sulfonylureas *vs* Standard treatments	0	NA	NA	NA	NA	NA	NA	0.52 (0.36 to 0.75)	Moderate	-1	Low	0.52 (0.36 to 0.75)	0	Low	NA	0	Low
**Genital infection**	Thiazolidinediones *vs* Standard treatments	0	NA	NA	NA	NA	NA	NA	10.85 (0.42 to 276.91)	Moderate	0	Moderate	10.85 (0.42 to 276.91)	0	Moderate	NA	-2	Very low
**Genital infection**	Sulfonylureas *vs* Thiazolidinediones	0	NA	NA	NA	NA	NA	NA	0.05 (0.00 to 1.25)	High	0	High	0.05 (0.00 to 1.25)	0	High	NA	-2	Low
**Amputation**	DPP-4 inhibitors *vs* GLP-1 receptor agonists	0	NA	NA	NA	NA	NA	NA	1.28 (0.12 to 13.54)	Moderate	0	Moderate	1.28 (0.12 to 13.54)	0	Moderate	NA	-1	Low
**Amputation**	DPP-4 inhibitors *vs* Non-steroidal MRAs	0	NA	NA	NA	NA	NA	NA	0.97 (0.37 to 2.55)	Moderate	0	Moderate	0.97 (0.37 to 2.55)	0	Moderate	NA	-1	Low
**Amputation**	DPP-4 inhibitors *vs* SGLT2 inhibitors	1	0.66 (0.03 to 16.92)	0	0	0	0	High	0.71 (0.38 to 1.33)	Moderate	0	Moderate	0.71 (0.39 to 1.31)	0.035	Moderate	0	0	Moderate

Outcome	Comparisons	k	Direct estimates	D1	D2	D3	D4	Direct Certainty	Indirect estimates	Initial Indirect Certainty	D5	Indirect Certainty	NMA estimates	Prop	Initial NMA Certainty	D6	D7	NMA Certainty
**Amputation**	DPP-4 inhibitors *vs* Standard treatments	1	0.91 (0.51 to 1.62)	0	0	0	0	High	0.84 (0.03 to 21.71)	Moderate	0	Moderate	0.91 (0.51 to 1.60)	0.97	High	0	0	High
**Amputation**	DPP-4 inhibitors *vs* Thiazolidinediones	0	NA	NA	NA	NA	NA	NA	1.24 (0.54 to 2.83)	Moderate	0	Moderate	1.24 (0.54 to 2.83)	0	Moderate	NA	-1	Low
**Amputation**	GLP-1 receptor agonists *vs* Non-steroidal MRAs	0	NA	NA	NA	NA	NA	NA	0.76 (0.07 to 8.48)	Moderate	0	Moderate	0.76 (0.07 to 8.48)	0	Moderate	NA	-1	Low
**Amputation**	GLP-1 receptor agonists *vs* SGLT2 inhibitors	0	NA	NA	NA	NA	NA	NA	0.55 (0.06 to 5.52)	Moderate	0	Moderate	0.55 (0.06 to 5.52)	0	Moderate	NA	-1	Low
**Amputation**	GLP-1 receptor agonists *vs* Standard treatments	2	0.71 (0.07 to 6.95)	0	0	0	0	High	NA	NA	0	NA	0.71 (0.07 to 6.95)	1	High	NA	-1	Moderate
**Amputation**	GLP-1 receptor agonists *vs* Thiazolidinediones	0	NA	NA	NA	NA	NA	NA	0.96 (0.09 to 10.26)	Moderate	0	Moderate	0.96 (0.09 to 10.26)	0	Moderate	NA	-1	Low
**Amputation**	Non-steroidal MRAs *vs* SGLT2 inhibitors	0	NA	NA	NA	NA	NA	NA	0.73 (0.32 to 1.65)	Moderate	0	Moderate	0.73 (0.32 to 1.65)	0	Moderate	NA	0	Moderate
**Amputation**	Non-steroidal MRAs *vs* Standard treatments	2	0.93 (0.43 to 2.03)	0	-1	0	0	Moderate	NA	NA	0	NA	0.93 (0.43 to 2.03)	1	Moderate	NA	-1	Low
**Amputation**	Non-steroidal MRAs *vs* Thiazolidinediones	0	NA	NA	NA	NA	NA	NA	1.27 (0.48 to 3.40)	Moderate	0	Moderate	1.27 (0.48 to 3.40)	0	Moderate	NA	-1	Low
**Amputation**	SGLT2 inhibitors *vs* Standard treatments	10	1.27 (1.01 to 1.61)	0	0	-1	0	Moderate	1.37 (0.05 to 36.82)	High	0	High	1.27 (1.01 to 1.61)	1	Moderate	0	0	Moderate
**Amputation**	SGLT2 inhibitors *vs* Thiazolidinediones	0	NA	NA	NA	NA	NA	NA	1.74 (0.91 to 3.31)	Moderate	0	Moderate	1.74 (0.91 to 3.31)	0	Moderate	NA	-1	Low
**Amputation**	Thiazolidinediones *vs* Standard treatments	2	0.73 (0.40 to 1.33)	0	0	-1	0	Moderate	NA	NA	0	NA	0.73 (0.40 to 1.33)	1	Moderate	NA	0	Moderate

Outcome	Comparisons	k	Direct estimates	D1	D2	D3	D4	Direct Certainty	Indirect estimates	Initial Indirect Certainty	D5	Indirect Certainty	NMA estimates	Prop	Initial NMA Certainty	D6	D7	NMA Certainty
**DKA**	Alpha-glucosidase inhibitors *vs* DPP-4 inhibitors	0	NA	NA	NA	NA	NA	NA	0.49 (0.01 to 19.75)	Low	0	Low	0.49 (0.01 to 19.75)	0	Low	NA	-1	Very low
**DKA**	Alpha-glucosidase inhibitors *vs* GLP-1 receptor agonists	0	NA	NA	NA	NA	NA	NA	0.38 (0.01 to 16.39)	Moderate	0	Moderate	0.38 (0.01 to 16.39)	0	Moderate	NA	-1	Low
**DKA**	Alpha-glucosidase inhibitors *vs* Meglitinides	1	1.01 (0.02 to 51.04)	-2	0	0	0	Low	NA	Low	0	Low	1.01 (0.02 to 51.04)	1	Low	NA	-1	Very low
**DKA**	Alpha-glucosidase inhibitors *vs* Metformin	0	NA	NA	NA	NA	NA	NA	0.29 (0.00 to 39.24)	Moderate	0	Moderate	0.29 (0.00 to 39.24)	0	Moderate	NA	-1	Low
**DKA**	Alpha-glucosidase inhibitors *vs* Non-steroidal MRAs	0	NA	NA	NA	NA	NA	NA	0.58 (0.01 to 26.32)	Low	0	Low	0.58 (0.01 to 26.32)	0	Low	NA	-1	Very low
**DKA**	Alpha-glucosidase inhibitors *vs* SGLT2 inhibitors	0	NA	NA	NA	NA	NA	NA	0.19 (0.00 to 7.97)	Low	0	Low	0.19 (0.00 to 7.97)	0	Low	NA	-1	Very low
**DKA**	Alpha-glucosidase inhibitors *vs* Standard treatments	0	NA	NA	NA	NA	NA	NA	0.40 (0.01 to 16.47)	Low	0	Low	0.40 (0.01 to 16.47)	0	Low	NA	-1	Very low
**DKA**	Alpha-glucosidase inhibitors *vs* Sulfonylureas	1	0.67 (0.03 to 16.50)	-2	0	0	0	Low	NA	Low	0	Low	0.67 (0.03 to 16.50)	1	Low	NA	-1	Very low
**DKA**	DPP-4 inhibitors *vs* GLP-1 receptor agonists	1	0.33 (0.01 to 8.15)	-2	0	0	0	Low	0.83 (0.33 to 2.10)	Moderate	0	Moderate	0.78 (0.32 to 1.89)	0.077	Moderate	0	0	Moderate
**DKA**	DPP-4 inhibitors *vs* Meglitinides	0	NA	NA	NA	NA	NA	NA	2.08 (0.05 to 84.81)	Low	0	Low	2.08 (0.05 to 84.81)	0	Low	NA	-1	Very low
**DKA**	DPP-4 inhibitors *vs* Metformin	0	NA	NA	NA	NA	NA	NA	0.59 (0.02 to 15.88)	Moderate	0	Moderate	0.59 (0.02 to 15.88)	0	Moderate	NA	-1	Low
**DKA**	DPP-4 inhibitors *vs* Non-steroidal MRAs	0	NA	NA	NA	NA	NA	NA	1.19 (0.41 to 3.46)	Moderate	0	Moderate	1.19 (0.41 to 3.46)	0	Moderate	NA	0	Moderate

Outcome	Comparisons	k	Direct estimates	D1	D2	D3	D4	Direct Certainty	Indirect estimates	Initial Indirect Certainty	D5	Indirect Certainty	NMA estimates	Prop	Initial NMA Certainty	D6	D7	NMA Certainty
**DKA**	DPP-4 inhibitors *vs* SGLT2 inhibitors	2	1.42 (0.15 to 13.75)	-1	0	0	0	Moderate	0.34 (0.15 to 0.74)	Moderate	0	Moderate	0.39 (0.19 to 0.83)	0.11	Moderate	0	0	Moderate
**DKA**	DPP-4 inhibitors *vs* Standard treatments	7	0.71 (0.34 to 1.49)	-1	0	0	0	Moderate	1.68 (0.31 to 9.27)	Moderate	0	Moderate	0.82 (0.41 to 1.61)	0.84	Moderate	0	0	Moderate
**DKA**	DPP-4 inhibitors *vs* Sulfonylureas	2	2.13 (0.22 to 20.51)	-1	0	0	0	Moderate	0.56 (0.02 to 14.98)	Moderate	0	Moderate	1.38 (0.21 to 8.94)	0.68	Moderate	0	-1	Low
**DKA**	GLP-1 receptor agonists *vs* Meglitinides	0	NA	NA	NA	NA	NA	NA	2.69 (0.06 to 116.94)	Moderate	0	Moderate	2.69 (0.06 to 116.94)	0	Moderate	NA	-1	Low
**DKA**	GLP-1 receptor agonists *vs* Metformin	0	NA	NA	NA	NA	NA	NA	0.76 (0.03 to 20.25)	Moderate	0	Moderate	0.76 (0.03 to 20.25)	0	Moderate	NA	-1	Low
**DKA**	GLP-1 receptor agonists *vs* Non-steroidal MRAs	0	NA	NA	NA	NA	NA	NA	1.54 (0.55 to 4.26)	Moderate	0	Moderate	1.54 (0.55 to 4.26)	0	Moderate	NA	0	Moderate
**DKA**	GLP-1 receptor agonists *vs* SGLT2 inhibitors	0	NA	NA	NA	NA	NA	NA	0.51 (0.25 to 1.02)	Moderate	0	Moderate	0.51 (0.25 to 1.02)	0	Moderate	NA	0	Moderate
**DKA**	GLP-1 receptor agonists *vs* Standard treatments	7	1.02 (0.55 to 1.88)	0	0	0	0	High	2.57 (0.10 to 68.50)	Low	0	Low	1.05 (0.58 to 1.92)	0.97	High	0	0	High
**DKA**	GLP-1 receptor agonists *vs* Sulfonylureas	0	NA	NA	NA	NA	NA	NA	1.78 (0.24 to 13.13)	Moderate	0	Moderate	1.78 (0.24 to 13.13)	0	Moderate	NA	-1	Low
**DKA**	Meglitinides *vs* Metformin	0	NA	NA	NA	NA	NA	NA	0.28 (0.00 to 38.81)	Moderate	0	Moderate	0.28 (0.00 to 38.81)	0	Moderate	NA	-1	Low
**DKA**	Meglitinides *vs* Non-steroidal MRAs	0	NA	NA	NA	NA	NA	NA	0.57 (0.01 to 26.04)	Low	0	Low	0.57 (0.01 to 26.04)	0	Low	NA	-1	Very low
**DKA**	Meglitinides *vs* SGLT2 inhibitors	0	NA	NA	NA	NA	NA	NA	0.19 (0.00 to 7.89)	Low	0	Low	0.19 (0.00 to 7.89)	0	Low	NA	-1	Very low

Outcome	Comparisons	k	Direct estimates	D1	D2	D3	D4	Direct Certainty	Indirect estimates	Initial Indirect Certainty	D5	Indirect Certainty	NMA estimates	Prop	Initial NMA Certainty	D6	D7	NMA Certainty
**DKA**	Meglitinides *vs* Standard treatments	0	NA	NA	NA	NA	NA	NA	0.39 (0.01 to 16.29)	Low	0	Low	0.39 (0.01 to 16.29)	0	Low	NA	-1	Very low
**DKA**	Meglitinides *vs* Sulfonylureas	1	0.66 (0.03 to 16.32)	-2	0	0	0	Low	NA	Low	0	Low	0.66 (0.03 to 16.32)	1	Low	NA	-1	Very low
**DKA**	Metformin *vs* Non-steroidal MRAs	0	NA	NA	NA	NA	NA	NA	2.02 (0.07 to 56.23)	Moderate	0	Moderate	2.02 (0.07 to 56.23)	0	Moderate	NA	-1	Low
**DKA**	Metformin *vs* SGLT2 inhibitors	1	0.67 (0.03 to 16.41)	0	0	0	0	High	NA	NA	0	NA	0.67 (0.03 to 16.41)	1	High	NA	-1	Moderate
**DKA**	Metformin *vs* Standard treatments	0	NA	NA	NA	NA	NA	NA	1.38 (0.05 to 34.70)	Moderate	0	Moderate	1.38 (0.05 to 34.70)	0	Moderate	NA	-1	Low
**DKA**	Metformin *vs* Sulfonylureas	0	NA	NA	NA	NA	NA	NA	2.34 (0.06 to 97.87)	Moderate	0	Moderate	2.34 (0.06 to 97.87)	0	Moderate	NA	-1	Low
**DKA**	Non-steroidal MRAs *vs* SGLT2 inhibitors	0	NA	NA	NA	NA	NA	NA	0.33 (0.13 to 0.81)	Moderate	0	Moderate	0.33 (0.13 to 0.81)	0	Moderate	NA	0	Moderate
**DKA**	Non-steroidal MRAs *vs* Standard treatments	2	0.68 (0.30 to 1.56)	0	-1	0	0	Moderate	NA	NA	0	NA	0.68 (0.30 to 1.56)	1	Moderate	NA	0	Moderate
**DKA**	Non-steroidal MRAs *vs* Sulfonylureas	0	NA	NA	NA	NA	NA	NA	1.16 (0.15 to 9.29)	Moderate	0	Moderate	1.16 (0.15 to 9.29)	0	Moderate	NA	-1	Low
**DKA**	SGLT2 inhibitors *vs* Standard treatments	12	2.17 (1.50 to 3.13)	0	0	0	0	High	0.48 (0.06 to 3.92)	Moderate	0	Moderate	2.07 (1.44 to 2.98)	0.97	High	0	0	High
**DKA**	SGLT2 inhibitors *vs* Sulfonylureas	1	1.49 (0.06 to 36.54)	0	0	0	0	High	5.68 (0.52 to 61.99)	Moderate	0	Moderate	3.51 (0.52 to 23.87)	0.36	Moderate	0	-1	Low
**DKA**	Sulfonylureas *vs* Standard treatments	0	NA	NA	NA	NA	NA	NA	0.59 (0.09 to 3.98)	Moderate	0	Moderate	0.59 (0.09 to 3.98)	0	Moderate	NA	0	Moderate

Outcome	Comparisons	k	Direct estimates	D1	D2	D3	D4	Direct Certainty	Indirect estimates	Initial Indirect Certainty	D5	Indirect Certainty	NMA estimates	Prop	Initial NMA Certainty	D6	D7	NMA Certainty
**HRQoL**	Alpha-glucosidase inhibitors *vs* Basal insulin	0	NA	NA	NA	NA	NA	NA	0.03 (-0.39 to 0.45)	Moderate	0	Moderate	0.03 (-0.39 to 0.45)	0	Moderate	NA	-2	Very low
**HRQoL**	Alpha-glucosidase inhibitors *vs* Bolus insulin	0	NA	NA	NA	NA	NA	NA	0.14 (-0.25 to 0.52)	Moderate	0	Moderate	0.14 (-0.25 to 0.52)	0	Moderate	NA	-2	Very low
**HRQoL**	Alpha-glucosidase inhibitors *vs* DPP-4 inhibitors	1	0.00 (-0.33 to 0.33)	0	0	0	0	High	NA	NA	0	NA	0.00 (-0.33 to 0.33)	1	High	NA	-2	Low
**HRQoL**	Alpha-glucosidase inhibitors *vs* GLP-1 receptor agonists	0	NA	NA	NA	NA	NA	NA	-0.14 (-0.50 to 0.21)	Moderate	0	Moderate	-0.14 (-0.50 to 0.21)	0	Moderate	NA	-2	Very low
**HRQoL**	Alpha-glucosidase inhibitors *vs* Meglitinides	0	NA	NA	NA	NA	NA	NA	-0.14 (-0.70 to 0.42)	Moderate	0	Moderate	-0.14 (-0.70 to 0.42)	0	Moderate	NA	-2	Very low
**HRQoL**	Alpha-glucosidase inhibitors *vs* Metformin	0	NA	NA	NA	NA	NA	NA	-0.02 (-0.45 to 0.41)	Moderate	0	Moderate	-0.02 (-0.45 to 0.41)	0	Moderate	NA	-2	Very low
**HRQoL**	Alpha-glucosidase inhibitors *vs* SGLT2 inhibitors	0	NA	NA	NA	NA	NA	NA	-0.27 (-0.66 to 0.12)	Moderate	0	Moderate	-0.27 (-0.66 to 0.12)	0	Moderate	NA	-1	Low
**HRQoL**	Alpha-glucosidase inhibitors *vs* Standard treatments	0	NA	NA	NA	NA	NA	NA	0.03 (-0.34 to 0.39)	Moderate	0	Moderate	0.03 (-0.34 to 0.39)	0	Moderate	NA	-2	Very low
**HRQoL**	Alpha-glucosidase inhibitors *vs* Sulfonylureas	0	NA	NA	NA	NA	NA	NA	-0.20 (-0.75 to 0.35)	Moderate	0	Moderate	-0.20 (-0.75 to 0.35)	0	Moderate	NA	-2	Very low
**HRQoL**	Alpha-glucosidase inhibitors *vs* Thiazolidinediones	0	NA	NA	NA	NA	NA	NA	-0.17 (-0.63 to 0.28)	Moderate	0	Moderate	-0.17 (-0.63 to 0.28)	0	Moderate	NA	-2	Very low
**HRQoL**	Alpha-glucosidase inhibitors *vs* Tirzepatide	0	NA	NA	NA	NA	NA	NA	-0.36 (-0.79 to 0.07)	Moderate	0	Moderate	-0.36 (-0.79 to 0.07)	0	Moderate	NA	-1	Low
**HRQoL**	Basal insulin *vs* Bolus insulin	1	0.42 (0.07 to 0.76)	-1	0	0	0	Moderate	-0.18 (-0.51 to 0.15)	Low	0	Low	0.11 (-0.13 to 0.34)	0.48	Low	-1	-1	Very low

Outcome	Comparisons	k	Direct estimates	D1	D2	D3	D4	Direct Certainty	Indirect estimates	Initial Indirect Certainty	D5	Indirect Certainty	NMA estimates	Prop	Initial NMA Certainty	D6	D7	NMA Certainty
**HRQoL**	Basal insulin *vs* DPP-4 inhibitors	0	NA	NA	NA	NA	NA	NA	-0.03 (-0.29 to 0.23)	Moderate	0	Moderate	-0.03 (-0.29 to 0.23)	0	Moderate	NA	-2	Very low
**HRQoL**	Basal insulin *vs* GLP-1 receptor agonists	1	-0.20 (-0.58 to 0.18)	-2	0	0	0	Low	-0.16 (-0.45 to 0.12)	Moderate	0	Moderate	-0.18 (-0.40 to 0.05)	0.35	Moderate	0	-1	Low
**HRQoL**	Basal insulin *vs* Meglitinides	0	NA	NA	NA	NA	NA	NA	-0.17 (-0.68 to 0.33)	Moderate	0	Moderate	-0.17 (-0.68 to 0.33)	0	Moderate	NA	-2	Very low
**HRQoL**	Basal insulin *vs* Metformin	0	NA	NA	NA	NA	NA	NA	-0.05 (-0.41 to 0.31)	Moderate	0	Moderate	-0.05 (-0.41 to 0.31)	0	Moderate	NA	-2	Very low
**HRQoL**	Basal insulin *vs* SGLT2 inhibitors	0	NA	NA	NA	NA	NA	NA	-0.30 (-0.60 to -0.01)	Moderate	0	Moderate	-0.30 (-0.60 to -0.01)	0	Moderate	NA	0	Moderate
**HRQoL**	Basal insulin *vs* Standard treatments	0	NA	NA	NA	NA	NA	NA	-0.00 (-0.25 to 0.24)	Moderate	0	Moderate	-0.00 (-0.25 to 0.24)	0	Moderate	NA	-2	Very low
**HRQoL**	Basal insulin *vs* Sulfonylureas	0	NA	NA	NA	NA	NA	NA	-0.23 (-0.72 to 0.25)	Moderate	0	Moderate	-0.23 (-0.72 to 0.25)	0	Moderate	NA	-2	Very low
**HRQoL**	Basal insulin *vs* Thiazolidinediones	0	NA	NA	NA	NA	NA	NA	-0.20 (-0.59 to 0.18)	Moderate	0	Moderate	-0.20 (-0.59 to 0.18)	0	Moderate	NA	-1	Low
**HRQoL**	Basal insulin *vs* Tirzepatide	1	-0.62 (-0.93 to -0.32)	0	0	0	0	High	0.01 (-0.40 to 0.41)	Moderate	0	Moderate	-0.39 (-0.64 to -0.15)	0.63	High	-1	0	Moderate
**HRQoL**	Bolus insulin *vs* DPP-4 inhibitors	0	NA	NA	NA	NA	NA	NA	-0.14 (-0.34 to 0.07)	Low	0	Low	-0.14 (-0.34 to 0.07)	0	Low	NA	-1	Very low
**HRQoL**	Bolus insulin *vs* GLP-1 receptor agonists	4	-0.21 (-0.37 to -0.04)	-2	0	0	0	Low	-0.81 (-1.25 to -0.36)	Moderate	0	Moderate	-0.28 (-0.44 to -0.13)	0.88	Low	-1	0	Very low
**HRQoL**	Bolus insulin *vs* Meglitinides	0	NA	NA	NA	NA	NA	NA	-0.28 (-0.76 to 0.20)	Low	0	Low	-0.28 (-0.76 to 0.20)	0	Low	NA	-1	Very low

Outcome	Comparisons	k	Direct estimates	D1	D2	D3	D4	Direct Certainty	Indirect estimates	Initial Indirect Certainty	D5	Indirect Certainty	NMA estimates	Prop	Initial NMA Certainty	D6	D7	NMA Certainty
**HRQoL**	Bolus insulin *vs* Metformin	0	NA	NA	NA	NA	NA	NA	-0.15 (-0.47 to 0.16)	Moderate	0	Moderate	-0.15 (-0.47 to 0.16)	0	Moderate	NA	-1	Low
**HRQoL**	Bolus insulin *vs* SGLT2 inhibitors	0	NA	NA	NA	NA	NA	NA	-0.41 (-0.65 to -0.16)	Moderate	0	Moderate	-0.41 (-0.65 to -0.16)	0	Moderate	NA	0	Moderate
**HRQoL**	Bolus insulin *vs* Standard treatments	0	NA	NA	NA	NA	NA	NA	-0.11 (-0.29 to 0.07)	Moderate	0	Moderate	-0.11 (-0.29 to 0.07)	0	Moderate	NA	-1	Low
**HRQoL**	Bolus insulin *vs* Sulfonylureas	0	NA	NA	NA	NA	NA	NA	-0.34 (-0.79 to 0.12)	Moderate	0	Moderate	-0.34 (-0.79 to 0.12)	0	Moderate	NA	-1	Low
**HRQoL**	Bolus insulin *vs* Thiazolidinediones	0	NA	NA	NA	NA	NA	NA	-0.31 (-0.66 to 0.04)	Moderate	0	Moderate	-0.31 (-0.66 to 0.04)	0	Moderate	NA	-1	Low
**HRQoL**	Bolus insulin *vs* Tirzepatide	0	NA	NA	NA	NA	NA	NA	-0.50 (-0.77 to -0.23)	Moderate	0	Moderate	-0.50 (-0.77 to -0.23)	0	Moderate	NA	0	Moderate
**HRQoL**	DPP-4 inhibitors *vs* GLP-1 receptor agonists	5	-0.15 (-0.30 to -0.00)	-1	0	0	0	Moderate	-0.12 (-0.39 to 0.14)	Moderate	0	Moderate	-0.14 (-0.28 to -0.01)	0.75	Moderate	0	-1	Low
**HRQoL**	DPP-4 inhibitors *vs* Meglitinides	0	NA	NA	NA	NA	NA	NA	-0.14 (-0.59 to 0.31)	Moderate	0	Moderate	-0.14 (-0.59 to 0.31)	0	Moderate	NA	-2	Very low
**HRQoL**	DPP-4 inhibitors *vs* Metformin	2	0.00 (-0.31 to 0.31)	0	0	0	0	High	-0.08 (-0.68 to 0.51)	Moderate	0	Moderate	-0.02 (-0.29 to 0.26)	0.79	High	0	-2	Low
**HRQoL**	DPP-4 inhibitors *vs* SGLT2 inhibitors	1	-0.26 (-0.64 to 0.12)	0	0	0	0	High	-0.28 (-0.53 to -0.03)	Moderate	0	Moderate	-0.27 (-0.48 to -0.06)	0.3	Moderate	0	0	Moderate
**HRQoL**	DPP-4 inhibitors *vs* Standard treatments	2	0.07 (-0.18 to 0.33)	0	0	0	0	High	0.00 (-0.18 to 0.19)	Moderate	0	Moderate	0.03 (-0.12 to 0.17)	0.34	Moderate	0	0	Moderate
**HRQoL**	DPP-4 inhibitors *vs* Sulfonylureas	0	NA	NA	NA	NA	NA	NA	-0.20 (-0.64 to 0.24)	Moderate	0	Moderate	-0.20 (-0.64 to 0.24)	0	Moderate	NA	-2	Very low

Outcome	Comparisons	k	Direct estimates	D1	D2	D3	D4	Direct Certainty	Indirect estimates	Initial Indirect Certainty	D5	Indirect Certainty	NMA estimates	Prop	Initial NMA Certainty	D6	D7	NMA Certainty
**HRQoL**	DPP-4 inhibitors *vs* Thiazolidinediones	1	-0.15 (-0.51 to 0.21)	0	0	0	0	High	-0.25 (-0.87 to 0.37)	Moderate	0	Moderate	-0.17 (-0.48 to 0.14)	0.75	High	0	-1	Moderate
**HRQoL**	DPP-4 inhibitors *vs* Tirzepatide	0	NA	NA	NA	NA	NA	NA	-0.36 (-0.64 to -0.09)	Moderate	0	Moderate	-0.36 (-0.64 to -0.09)	0	Moderate	NA	0	Moderate
**HRQoL**	GLP-1 receptor agonists *vs* Meglitinides	0	NA	NA	NA	NA	NA	NA	0.00 (-0.45 to 0.45)	Moderate	0	Moderate	0.00 (-0.45 to 0.45)	0	Moderate	NA	-2	Very low
**HRQoL**	GLP-1 receptor agonists *vs* Metformin	1	0.11 (-0.22 to 0.44)	0	0	0	0	High	0.16 (-0.34 to 0.67)	Moderate	0	Moderate	0.13 (-0.15 to 0.40)	0.7	High	0	-1	Moderate
**HRQoL**	GLP-1 receptor agonists *vs* SGLT2 inhibitors	2	-0.13 (-0.41 to 0.16)	0	0	0	0	High	-0.13 (-0.38 to 0.13)	Moderate	0	Moderate	-0.13 (-0.32 to 0.06)	0.45	Moderate	0	-1	Low
**HRQoL**	GLP-1 receptor agonists *vs* Standard treatments	11	0.16 (0.05 to 0.26)	0	0	0	0	High	0.27 (-0.00 to 0.54)	Moderate	0	Moderate	0.17 (0.07 to 0.27)	0.86	High	0	-1	Moderate
**HRQoL**	GLP-1 receptor agonists *vs* Sulfonylureas	0	NA	NA	NA	NA	NA	NA	-0.06 (-0.49 to 0.37)	Moderate	0	Moderate	-0.06 (-0.49 to 0.37)	0	Moderate	NA	-2	Very low
**HRQoL**	GLP-1 receptor agonists *vs* Thiazolidinediones	1	-0.05 (-0.39 to 0.30)	0	0	0	0	High	0.05 (-0.66 to 0.77)	Moderate	0	Moderate	-0.03 (-0.34 to 0.28)	0.81	High	0	-2	Low
**HRQoL**	GLP-1 receptor agonists *vs* Tirzepatide	1	0.00 (-0.30 to 0.30)	0	0	0	0	High	-0.63 (-1.04 to -0.22)	Moderate	0	Moderate	-0.22 (-0.46 to 0.02)	0.65	High	-1	-1	Low
**HRQoL**	Meglitinides *vs* Metformin	0	NA	NA	NA	NA	NA	NA	0.12 (-0.35 to 0.59)	Moderate	0	Moderate	0.12 (-0.35 to 0.59)	0	Moderate	NA	-2	Very low
**HRQoL**	Meglitinides *vs* SGLT2 inhibitors	0	NA	NA	NA	NA	NA	NA	-0.13 (-0.62 to 0.35)	Moderate	0	Moderate	-0.13 (-0.62 to 0.35)	0	Moderate	NA	-2	Very low
**HRQoL**	Meglitinides *vs* Standard treatments	0	NA	NA	NA	NA	NA	NA	0.17 (-0.29 to 0.63)	Moderate	0	Moderate	0.17 (-0.29 to 0.63)	0	Moderate	NA	-2	Very low

Outcome	Comparisons	k	Direct estimates	D1	D2	D3	D4	Direct Certainty	Indirect estimates	Initial Indirect Certainty	D5	Indirect Certainty	NMA estimates	Prop	Initial NMA Certainty	D6	D7	NMA Certainty
**HRQoL**	Meglitinides *vs* Sulfonylureas	0	NA	NA	NA	NA	NA	NA	-0.06 (-0.68 to 0.56)	Moderate	0	Moderate	-0.06 (-0.68 to 0.56)	0	Moderate	NA	-2	Very low
**HRQoL**	Meglitinides *vs* Thiazolidinediones	1	-0.03 (-0.36 to 0.30)	-2	0	0	0	Low	NA	NA	0	NA	-0.03 (-0.36 to 0.30)	1	Low	NA	-2	Very low
**HRQoL**	Meglitinides *vs* Tirzepatide	0	NA	NA	NA	NA	NA	NA	-0.22 (-0.73 to 0.29)	Moderate	0	Moderate	-0.22 (-0.73 to 0.29)	0	Moderate	NA	-2	Very low
**HRQoL**	Metformin *vs* SGLT2 inhibitors	0	NA	NA	NA	NA	NA	NA	-0.26 (-0.58 to 0.07)	Moderate	0	Moderate	-0.26 (-0.58 to 0.07)	0	Moderate	NA	-1	Low
**HRQoL**	Metformin *vs* Standard treatments	0	NA	NA	NA	NA	NA	NA	0.04 (-0.25 to 0.33)	Moderate	0	Moderate	0.04 (-0.25 to 0.33)	0	Moderate	NA	-2	Very low
**HRQoL**	Metformin *vs* Sulfonylureas	0	NA	NA	NA	NA	NA	NA	-0.19 (-0.69 to 0.32)	Moderate	0	Moderate	-0.19 (-0.69 to 0.32)	0	Moderate	NA	-2	Very low
**HRQoL**	Metformin *vs* Thiazolidinediones	1	-0.16 (-0.50 to 0.19)	0	0	0	0	High	-0.08 (-1.68 to 1.51)	Moderate	0	Moderate	-0.15 (-0.49 to 0.18)	0.96	High	0	-1	Moderate
**HRQoL**	Metformin *vs* Tirzepatide	0	NA	NA	NA	NA	NA	NA	-0.34 (-0.71 to 0.02)	Moderate	0	Moderate	-0.34 (-0.71 to 0.02)	0	Moderate	NA	-1	Low
**HRQoL**	SGLT2 inhibitors *vs* Standard treatments	2	0.31 (-0.00 to 0.61)	0	0	-1	0	Moderate	0.29 (0.04 to 0.55)	Moderate	0	Moderate	0.30 (0.10 to 0.49)	0.4	Moderate	0	0	Moderate
**HRQoL**	SGLT2 inhibitors *vs* Sulfonylureas	0	NA	NA	NA	NA	NA	NA	0.07 (-0.39 to 0.53)	Moderate	0	Moderate	0.07 (-0.39 to 0.53)	0	Moderate	NA	-2	Very low
**HRQoL**	SGLT2 inhibitors *vs* Thiazolidinediones	0	NA	NA	NA	NA	NA	NA	0.10 (-0.26 to 0.46)	Moderate	0	Moderate	0.10 (-0.26 to 0.46)	0	Moderate	NA	-2	Very low
**HRQoL**	SGLT2 inhibitors *vs* Tirzepatide	0	NA	NA	NA	NA	NA	NA	-0.09 (-0.40 to 0.22)	Moderate	0	Moderate	-0.09 (-0.40 to 0.22)	0	Moderate	NA	-2	Very low

Outcome	Comparisons	k	Direct estimates	D1	D2	D3	D4	Direct Certainty	Indirect estimates	Initial Indirect Certainty	D5	Indirect Certainty	NMA estimates	Prop	Initial NMA Certainty	D6	D7	NMA Certainty
**HRQoL**	Sulfonylureas *vs* Standard treatments	1	0.23 (-0.19 to 0.64)	-1	0	0	0	Moderate	NA	NA	0	NA	0.23 (-0.19 to 0.64)	1	Moderate	NA	-1	Low
**HRQoL**	Thiazolidinediones *vs* Standard treatments	0	NA	NA	NA	NA	NA	NA	0.20 (-0.13 to 0.52)	Moderate	0	Moderate	0.20 (-0.13 to 0.52)	0	Moderate	NA	-1	Low
**HRQoL**	Tirzepatide *vs* Standard treatments	0	NA	NA	NA	NA	NA	NA	0.39 (0.13 to 0.65)	Moderate	0	Moderate	0.39 (0.13 to 0.65)	0	Moderate	NA	0	Moderate
**HRQoL**	Sulfonylureas *vs* Thiazolidinediones	0	NA	NA	NA	NA	NA	NA	0.03 (-0.50 to 0.56)	Moderate	0	Moderate	0.03 (-0.50 to 0.56)	0	Moderate	NA	-2	Very low
**HRQoL**	Sulfonylureas *vs* Tirzepatide	0	NA	NA	NA	NA	NA	NA	-0.16 (-0.65 to 0.33)	Moderate	0	Moderate	-0.16 (-0.65 to 0.33)	0	Moderate	NA	-2	Very low
**HRQoL**	Thiazolidinediones *vs* Tirzepatide	0	NA	NA	NA	NA	NA	NA	-0.19 (-0.58 to 0.20)	Moderate	0	Moderate	-0.19 (-0.58 to 0.20)	0	Moderate	NA	-2	Very low
**Body weight change**	Albiglutide *vs* Alpha-glucosidase inhibitors	0	NA	NA	NA	NA	NA	NA	-0.33 (-1.09 to 0.42)	Moderate	0	Moderate	-0.33 (-1.09 to 0.42)	0	Moderate	NA	0	Moderate
**Body weight change**	Albiglutide *vs* Basal insulin	1	-2.61 (-4.48 to -0.74)	-2	0	0	0	Low	-2.91 (-3.69 to -2.13)	Moderate	0	Moderate	-2.87 (-3.59 to -2.14)	0.15	Moderate	0	0	Moderate
**Body weight change**	Albiglutide *vs* Basal-Bolus insulin	0	NA	NA	NA	NA	NA	NA	-3.97 (-5.27 to -2.67)	Low	0	Low	-3.97 (-5.27 to -2.67)	0	Low	NA	-1	Very low
**Body weight change**	Albiglutide *vs* Bolus insulin	2	-2.97 (-4.28 to -1.65)	-1	0	0	0	Moderate	-0.81 (-1.94 to 0.33)	Moderate	0	Moderate	-1.73 (-2.59 to -0.87)	0.43	Moderate	-1	0	Low
**Body weight change**	Albiglutide *vs* DPP-4 inhibitors	2	-0.48 (-1.80 to 0.85)	-1	0	0	0	Moderate	-1.16 (-1.90 to -0.42)	Moderate	0	Moderate	-1.00 (-1.65 to -0.36)	0.24	Moderate	0	0	Moderate
**Body weight change**	Albiglutide *vs* Dulaglutide	0	NA	NA	NA	NA	NA	NA	0.69 (-0.12 to 1.50)	Moderate	0	Moderate	0.69 (-0.12 to 1.50)	0	Moderate	NA	0	Moderate

Outcome	Comparisons	k	Direct estimates	D1	D2	D3	D4	Direct Certainty	Indirect estimates	Initial Indirect Certainty	D5	Indirect Certainty	NMA estimates	Prop	Initial NMA Certainty	D6	D7	NMA Certainty
**Body weight change**	Albiglutide *vs* Efpeglenatide	0	NA	NA	NA	NA	NA	NA	1.87 (-0.04 to 3.79)	Moderate	0	Moderate	1.87 (-0.04 to 3.79)	0	Moderate	NA	0	Moderate
**Body weight change**	Albiglutide *vs* Exenatide extended-release	0	NA	NA	NA	NA	NA	NA	0.33 (-0.55 to 1.21)	Moderate	0	Moderate	0.33 (-0.55 to 1.21)	0	Moderate	NA	0	Moderate
**Body weight change**	Albiglutide *vs* Exenatide immediate-release	0	NA	NA	NA	NA	NA	NA	1.05 (0.12 to 1.99)	Moderate	0	Moderate	1.05 (0.12 to 1.99)	0	Moderate	NA	0	Moderate
**Body weight change**	Albiglutide *vs* Liraglutide	0	NA	NA	NA	NA	NA	NA	1.50 (0.78 to 2.21)	Moderate	0	Moderate	1.50 (0.78 to 2.21)	0	Moderate	NA	0	Moderate
**Body weight change**	Albiglutide *vs* Lixisenatide	0	NA	NA	NA	NA	NA	NA	0.12 (-0.72 to 0.95)	Moderate	0	Moderate	0.12 (-0.72 to 0.95)	0	Moderate	NA	0	Moderate
**Body weight change**	Albiglutide *vs* Loxenatide	0	NA	NA	NA	NA	NA	NA	-0.88 (-2.86 to 1.11)	Low	0	Low	-0.88 (-2.86 to 1.11)	0	Low	NA	0	Low
**Body weight change**	Albiglutide *vs* Meglitinides	0	NA	NA	NA	NA	NA	NA	-1.97 (-2.90 to -1.05)	Moderate	0	Moderate	-1.97 (-2.90 to -1.05)	0	Moderate	NA	0	Moderate
**Body weight change**	Albiglutide *vs* Metformin	0	NA	NA	NA	NA	NA	NA	0.12 (-0.59 to 0.82)	Moderate	0	Moderate	0.12 (-0.59 to 0.82)	0	Moderate	NA	0	Moderate
**Body weight change**	Albiglutide *vs* Semaglutide (Oral)	0	NA	NA	NA	NA	NA	NA	2.26 (1.33 to 3.19)	Moderate	0	Moderate	2.26 (1.33 to 3.19)	0	Moderate	NA	0	Moderate
**Body weight change**	Albiglutide *vs* Semaglutide (Subcutaneous)	0	NA	NA	NA	NA	NA	NA	3.91 (3.06 to 4.76)	Moderate	0	Moderate	3.91 (3.06 to 4.76)	0	Moderate	NA	0	Moderate
**Body weight change**	Albiglutide *vs* SGLT2 inhibitors	0	NA	NA	NA	NA	NA	NA	1.26 (0.60 to 1.92)	Moderate	0	Moderate	1.26 (0.60 to 1.92)	0	Moderate	NA	0	Moderate
**Body weight change**	Albiglutide *vs* Standard treatments	5	-0.16 (-1.04 to 0.72)	0	0	0	0	High	-1.31 (-2.23 to -0.40)	Low	0	Low	-0.72 (-1.35 to -0.08)	0.52	Moderate	0	0	Moderate

Outcome	Comparisons	k	Direct estimates	D1	D2	D3	D4	Direct Certainty	Indirect estimates	Initial Indirect Certainty	D5	Indirect Certainty	NMA estimates	Prop	Initial NMA Certainty	D6	D7	NMA Certainty
**Body weight change**	Albiglutide *vs* Sulfonylureas	1	-2.38 (-4.27 to -0.49)	-1	0	0	0	Moderate	-2.51 (-3.24 to -1.79)	Moderate	0	Moderate	-2.50 (-3.18 to -1.82)	0.13	Moderate	0	0	Moderate
**Body weight change**	Albiglutide *vs* Thiazolidinediones	1	-4.01 (-5.90 to -2.12)	-1	0	0	0	Moderate	-3.46 (-4.17 to -2.74)	Low	0	Low	-3.53 (-4.20 to -2.86)	0.13	Low	0	0	Low
**Body weight change**	Albiglutide *vs* Tirzepatide	0	NA	NA	NA	NA	NA	NA	7.85 (6.83 to 8.88)	Moderate	0	Moderate	7.85 (6.83 to 8.88)	0	Moderate	NA	0	Moderate
**Body weight change**	Alpha-glucosidase inhibitors *vs* Basal insulin	0	NA	NA	NA	NA	NA	NA	-2.53 (-3.11 to -1.95)	Low	0	Low	-2.53 (-3.11 to -1.95)	0	Low	NA	0	Low
**Body weight change**	Alpha-glucosidase inhibitors *vs* Basal-Bolus insulin	0	NA	NA	NA	NA	NA	NA	-3.64 (-4.86 to -2.42)	Low	0	Low	-3.64 (-4.86 to -2.42)	0	Low	NA	0	Low
**Body weight change**	Alpha-glucosidase inhibitors *vs* Bolus insulin	0	NA	NA	NA	NA	NA	NA	-1.39 (-2.27 to -0.52)	Low	0	Low	-1.39 (-2.27 to -0.52)	0	Low	NA	0	Low
**Body weight change**	Alpha-glucosidase inhibitors *vs* DPP-4 inhibitors	6	-0.73 (-1.53 to 0.07)	-2	0	0	0	Low	-0.64 (-1.15 to -0.13)	Low	0	Low	-0.67 (-1.10 to -0.24)	0.29	Low	0	0	Low
**Body weight change**	Alpha-glucosidase inhibitors *vs* Dulaglutide	0	NA	NA	NA	NA	NA	NA	1.02 (0.36 to 1.69)	Moderate	0	Moderate	1.02 (0.36 to 1.69)	0	Moderate	NA	0	Moderate
**Body weight change**	Alpha-glucosidase inhibitors *vs* Efpeglenatide	0	NA	NA	NA	NA	NA	NA	2.21 (0.35 to 4.06)	Moderate	0	Moderate	2.21 (0.35 to 4.06)	0	Moderate	NA	0	Moderate
**Body weight change**	Alpha-glucosidase inhibitors *vs* Exenatide extended-release	0	NA	NA	NA	NA	NA	NA	0.66 (-0.08 to 1.41)	Moderate	0	Moderate	0.66 (-0.08 to 1.41)	0	Moderate	NA	0	Moderate
**Body weight change**	Alpha-glucosidase inhibitors *vs* Exenatide immediate-release	0	NA	NA	NA	NA	NA	NA	1.39 (0.58 to 2.20)	Low	0	Low	1.39 (0.58 to 2.20)	0	Low	NA	0	Low

Outcome	Comparisons	k	Direct estimates	D1	D2	D3	D4	Direct Certainty	Indirect estimates	Initial Indirect Certainty	D5	Indirect Certainty	NMA estimates	Prop	Initial NMA Certainty	D6	D7	NMA Certainty
**Body weight change**	Alpha-glucosidase inhibitors *vs* Liraglutide	0	NA	NA	NA	NA	NA	NA	1.83 (1.28 to 2.38)	Moderate	0	Moderate	1.83 (1.28 to 2.38)	0	Moderate	NA	0	Moderate
**Body weight change**	Alpha-glucosidase inhibitors *vs* Lixisenatide	0	NA	NA	NA	NA	NA	NA	0.45 (-0.26 to 1.16)	Moderate	0	Moderate	0.45 (-0.26 to 1.16)	0	Moderate	NA	0	Moderate
**Body weight change**	Alpha-glucosidase inhibitors *vs* Loxenatide	0	NA	NA	NA	NA	NA	NA	-0.54 (-2.47 to 1.39)	Low	0	Low	-0.54 (-2.47 to 1.39)	0	Low	NA	0	Low
**Body weight change**	Alpha-glucosidase inhibitors *vs* Meglitinides	0	NA	NA	NA	NA	NA	NA	-1.64 (-2.43 to -0.85)	Low	0	Low	-1.64 (-2.43 to -0.85)	0	Low	NA	0	Low
**Body weight change**	Alpha-glucosidase inhibitors *vs* Metformin	3	0.60 (-0.56 to 1.75)	-1	0	0	0	Moderate	0.42 (-0.13 to 0.97)	Low	0	Low	0.45 (-0.05 to 0.95)	0.19	Low	0	0	Low
**Body weight change**	Alpha-glucosidase inhibitors *vs* Semaglutide (Oral)	0	NA	NA	NA	NA	NA	NA	2.60 (1.79 to 3.40)	Moderate	0	Moderate	2.60 (1.79 to 3.40)	0	Moderate	NA	0	Moderate
**Body weight change**	Alpha-glucosidase inhibitors *vs* Semaglutide (Subcutaneous)	0	NA	NA	NA	NA	NA	NA	4.24 (3.52 to 4.97)	Moderate	0	Moderate	4.24 (3.52 to 4.97)	0	Moderate	NA	0	Moderate
**Body weight change**	Alpha-glucosidase inhibitors *vs* SGLT2 inhibitors	1	0.27 (-2.56 to 3.10)	-2	0	0	0	Low	1.63 (1.17 to 2.10)	Moderate	0	Moderate	1.60 (1.14 to 2.06)	0.026	Moderate	0	0	Moderate
**Body weight change**	Alpha-glucosidase inhibitors *vs* Standard treatments	12	-0.53 (-1.12 to 0.07)	-1	0	0	0	Moderate	-0.24 (-0.84 to 0.36)	Low	0	Low	-0.38 (-0.80 to 0.04)	0.5	Low	0	0	Low
**Body weight change**	Alpha-glucosidase inhibitors *vs* Sulfonylureas	2	-1.00 (-2.84 to 0.84)	-1	0	0	0	Moderate	-2.25 (-2.75 to -1.75)	Low	0	Low	-2.16 (-2.65 to -1.68)	0.069	Low	0	0	Low
**Body weight change**	Alpha-glucosidase inhibitors *vs* Thiazolidinediones	2	-2.61 (-4.10 to -1.13)	-2	0	0	0	Low	-3.26 (-3.75 to -2.76)	Low	0	Low	-3.19 (-3.66 to -2.72)	0.1	Low	0	0	Low

Outcome	Comparisons	k	Direct estimates	D1	D2	D3	D4	Direct Certainty	Indirect estimates	Initial Indirect Certainty	D5	Indirect Certainty	NMA estimates	Prop	Initial NMA Certainty	D6	D7	NMA Certainty
**Body weight change**	Alpha-glucosidase inhibitors *vs* Tirzepatide	0	NA	NA	NA	NA	NA	NA	8.19 (7.26 to 9.11)	Moderate	0	Moderate	8.19 (7.26 to 9.11)	0	Moderate	NA	0	Moderate
**Body weight change**	Basal insulin *vs* Basal-Bolus insulin	2	-0.87 (-2.20 to 0.46)	-2	0	0	0	Low	-1.69 (-3.77 to 0.39)	Low	0	Low	-1.11 (-2.23 to 0.01)	0.71	Low	0	0	Low
**Body weight change**	Basal insulin *vs* Bolus insulin	0	NA	NA	NA	NA	NA	NA	1.14 (0.29 to 1.99)	Low	0	Low	1.14 (0.29 to 1.99)	0	Low	NA	0	Low
**Body weight change**	Basal insulin *vs* DPP-4 inhibitors	4	1.72 (0.71 to 2.74)	-1	0	0	0	Moderate	1.90 (1.43 to 2.36)	Moderate	0	Moderate	1.87 (1.44 to 2.29)	0.17	Moderate	0	0	Moderate
**Body weight change**	Basal insulin *vs* Dulaglutide	5	3.03 (2.12 to 3.95)	-1	0	0	0	Moderate	3.90 (3.15 to 4.64)	Moderate	0	Moderate	3.55 (2.97 to 4.13)	0.4	Moderate	0	0	Moderate
**Body weight change**	Basal insulin *vs* Efpeglenatide	0	NA	NA	NA	NA	NA	NA	4.74 (2.89 to 6.59)	Moderate	0	Moderate	4.74 (2.89 to 6.59)	0	Moderate	NA	-1	Low
**Body weight change**	Basal insulin *vs* Exenatide extended-release	4	3.20 (2.18 to 4.23)	-1	0	0	0	Moderate	3.19 (2.32 to 4.06)	Moderate	0	Moderate	3.20 (2.53 to 3.86)	0.42	Moderate	0	0	Moderate
**Body weight change**	Basal insulin *vs* Exenatide immediate-release	2	3.97 (2.49 to 5.45)	-1	0	0	0	Moderate	3.90 (3.01 to 4.79)	Moderate	0	Moderate	3.92 (3.16 to 4.68)	0.26	Moderate	0	0	Moderate
**Body weight change**	Basal insulin *vs* Liraglutide	8	4.16 (3.40 to 4.91)	-1	0	0	0	Moderate	4.51 (3.87 to 5.14)	Moderate	0	Moderate	4.36 (3.88 to 4.85)	0.41	Moderate	0	0	Moderate
**Body weight change**	Basal insulin *vs* Lixisenatide	0	NA	NA	NA	NA	NA	NA	2.98 (2.28 to 3.68)	Moderate	0	Moderate	2.98 (2.28 to 3.68)	0	Moderate	NA	0	Moderate
**Body weight change**	Basal insulin *vs* Loxenatide	0	NA	NA	NA	NA	NA	NA	1.99 (0.06 to 3.92)	Low	0	Low	1.99 (0.06 to 3.92)	0	Low	NA	0	Low
**Body weight change**	Basal insulin *vs* Meglitinides	0	NA	NA	NA	NA	NA	NA	0.89 (0.11 to 1.68)	Moderate	0	Moderate	0.89 (0.11 to 1.68)	0	Moderate	NA	0	Moderate

Outcome	Comparisons	k	Direct estimates	D1	D2	D3	D4	Direct Certainty	Indirect estimates	Initial Indirect Certainty	D5	Indirect Certainty	NMA estimates	Prop	Initial NMA Certainty	D6	D7	NMA Certainty
**Body weight change**	Basal insulin *vs* Metformin	0	NA	NA	NA	NA	NA	NA	2.98 (2.48 to 3.49)	Moderate	0	Moderate	2.98 (2.48 to 3.49)	0	Moderate	NA	0	Moderate
**Body weight change**	Basal insulin *vs* Semaglutide (Oral)	0	NA	NA	NA	NA	NA	NA	5.13 (4.34 to 5.92)	Moderate	0	Moderate	5.13 (4.34 to 5.92)	0	Moderate	NA	0	Moderate
**Body weight change**	Basal insulin *vs* Semaglutide (Subcutaneous)	1	7.48 (5.53 to 9.43)	-2	0	0	0	Low	6.67 (5.94 to 7.41)	Moderate	0	Moderate	6.77 (6.09 to 7.46)	0.12	Moderate	0	0	Moderate
**Body weight change**	Basal insulin *vs* SGLT2 inhibitors	0	NA	NA	NA	NA	NA	NA	4.13 (3.68 to 4.58)	Moderate	0	Moderate	4.13 (3.68 to 4.58)	0	Moderate	NA	0	Moderate
**Body weight change**	Basal insulin *vs* Standard treatments	5	1.72 (0.75 to 2.69)	-2	0	0	0	Low	2.24 (1.79 to 2.70)	Moderate	0	Moderate	2.15 (1.74 to 2.56)	0.18	Moderate	0	0	Moderate
**Body weight change**	Basal insulin *vs* Sulfonylureas	2	0.57 (-0.96 to 2.11)	-1	0	0	0	Moderate	0.35 (-0.14 to 0.84)	Moderate	0	Moderate	0.37 (-0.10 to 0.84)	0.094	Moderate	0	0	Moderate
**Body weight change**	Basal insulin *vs* Thiazolidinediones	2	-1.30 (-2.96 to 0.36)	-1	0	0	0	Moderate	-0.61 (-1.08 to -0.13)	Low	0	Low	-0.66 (-1.12 to -0.20)	0.076	Low	0	0	Low
**Body weight change**	Basal insulin *vs* Tirzepatide	2	11.96 (10.60 to 13.32)	0	0	0	0	High	9.94 (8.87 to 11.02)	Moderate	0	Moderate	10.72 (9.88 to 11.57)	0.39	Moderate	-1	0	Low
**Body weight change**	Basal-Bolus insulin *vs* Bolus insulin	0	NA	NA	NA	NA	NA	NA	2.25 (0.87 to 3.62)	Low	0	Low	2.25 (0.87 to 3.62)	0	Low	NA	0	Low
**Body weight change**	Basal-Bolus insulin *vs* DPP-4 inhibitors	0	NA	NA	NA	NA	NA	NA	2.97 (1.82 to 4.13)	Low	0	Low	2.97 (1.82 to 4.13)	0	Low	NA	0	Low
**Body weight change**	Basal-Bolus insulin *vs* Dulaglutide	0	NA	NA	NA	NA	NA	NA	4.66 (3.43 to 5.89)	Low	0	Low	4.66 (3.43 to 5.89)	0	Low	NA	-1	Very low

Outcome	Comparisons	k	Direct estimates	D1	D2	D3	D4	Direct Certainty	Indirect estimates	Initial Indirect Certainty	D5	Indirect Certainty	NMA estimates	Prop	Initial NMA Certainty	D6	D7	NMA Certainty
**Body weight change**	Basal-Bolus insulin *vs* Efpeglenatide	0	NA	NA	NA	NA	NA	NA	5.85 (3.71 to 7.99)	Moderate	0	Moderate	5.85 (3.71 to 7.99)	0	Moderate	NA	0	Moderate
**Body weight change**	Basal-Bolus insulin *vs* Exenatide extended-release	0	NA	NA	NA	NA	NA	NA	4.30 (3.03 to 5.58)	Low	0	Low	4.30 (3.03 to 5.58)	0	Low	NA	-1	Very low
**Body weight change**	Basal-Bolus insulin *vs* Exenatide immediate-release	0	NA	NA	NA	NA	NA	NA	5.03 (3.70 to 6.35)	Low	0	Low	5.03 (3.70 to 6.35)	0	Low	NA	0	Low
**Body weight change**	Basal-Bolus insulin *vs* Liraglutide	0	NA	NA	NA	NA	NA	NA	5.47 (4.28 to 6.66)	Low	0	Low	5.47 (4.28 to 6.66)	0	Low	NA	0	Low
**Body weight change**	Basal-Bolus insulin *vs* Lixisenatide	0	NA	NA	NA	NA	NA	NA	4.09 (2.81 to 5.37)	Low	0	Low	4.09 (2.81 to 5.37)	0	Low	NA	-1	Very low
**Body weight change**	Basal-Bolus insulin *vs* Loxenatide	0	NA	NA	NA	NA	NA	NA	3.10 (0.89 to 5.30)	Low	0	Low	3.10 (0.89 to 5.30)	0	Low	NA	-1	Very low
**Body weight change**	Basal-Bolus insulin *vs* Meglitinides	0	NA	NA	NA	NA	NA	NA	2.00 (0.67 to 3.33)	Low	0	Low	2.00 (0.67 to 3.33)	0	Low	NA	0	Low
**Body weight change**	Basal-Bolus insulin *vs* Metformin	0	NA	NA	NA	NA	NA	NA	4.09 (2.90 to 5.28)	Low	0	Low	4.09 (2.90 to 5.28)	0	Low	NA	-1	Very low
**Body weight change**	Basal-Bolus insulin *vs* Semaglutide (Oral)	0	NA	NA	NA	NA	NA	NA	6.24 (4.90 to 7.57)	Low	0	Low	6.24 (4.90 to 7.57)	0	Low	NA	0	Low
**Body weight change**	Basal-Bolus insulin *vs* Semaglutide (Subcutaneous)	0	NA	NA	NA	NA	NA	NA	7.88 (6.60 to 9.16)	Low	0	Low	7.88 (6.60 to 9.16)	0	Low	NA	0	Low
**Body weight change**	Basal-Bolus insulin *vs* SGLT2 inhibitors	0	NA	NA	NA	NA	NA	NA	5.24 (4.07 to 6.40)	Low	0	Low	5.24 (4.07 to 6.40)	0	Low	NA	0	Low
**Body weight change**	Basal-Bolus insulin *vs* Standard treatments	0	NA	NA	NA	NA	NA	NA	3.26 (2.10 to 4.41)	Low	0	Low	3.26 (2.10 to 4.41)	0	Low	NA	0	Low

Outcome	Comparisons	k	Direct estimates	D1	D2	D3	D4	Direct Certainty	Indirect estimates	Initial Indirect Certainty	D5	Indirect Certainty	NMA estimates	Prop	Initial NMA Certainty	D6	D7	NMA Certainty
**Body weight change**	Basal-Bolus insulin *vs* Sulfonylureas	0	NA	NA	NA	NA	NA	NA	1.48 (0.30 to 2.65)	Low	0	Low	1.48 (0.30 to 2.65)	0	Low	NA	0	Low
**Body weight change**	Basal-Bolus insulin *vs* Thiazolidinediones	1	1.00 (-1.03 to 3.03)	-2	0	0	0	Low	0.18 (-1.23 to 1.59)	Low	0	Low	0.45 (-0.71 to 1.60)	0.33	Low	0	0	Low
**Body weight change**	Basal-Bolus insulin *vs* Tirzepatide	0	NA	NA	NA	NA	NA	NA	11.83 (10.45 to 13.21)	Low	0	Low	11.83 (10.45 to 13.21)	0	Low	NA	0	Low
**Body weight change**	Bolus insulin *vs* DPP-4 inhibitors	0	NA	NA	NA	NA	NA	NA	0.73 (-0.06 to 1.51)	Low	0	Low	0.73 (-0.06 to 1.51)	0	Low	NA	0	Low
**Body weight change**	Bolus insulin *vs* Dulaglutide	0	NA	NA	NA	NA	NA	NA	2.41 (1.49 to 3.34)	Moderate	0	Moderate	2.41 (1.49 to 3.34)	0	Moderate	NA	0	Moderate
**Body weight change**	Bolus insulin *vs* Efpeglenatide	0	NA	NA	NA	NA	NA	NA	3.60 (1.64 to 5.57)	Moderate	0	Moderate	3.60 (1.64 to 5.57)	0	Moderate	NA	-1	Low
**Body weight change**	Bolus insulin *vs* Exenatide extended-release	0	NA	NA	NA	NA	NA	NA	2.06 (1.07 to 3.04)	Moderate	0	Moderate	2.06 (1.07 to 3.04)	0	Moderate	NA	0	Moderate
**Body weight change**	Bolus insulin *vs* Exenatide immediate-release	0	NA	NA	NA	NA	NA	NA	2.78 (1.74 to 3.82)	Moderate	0	Moderate	2.78 (1.74 to 3.82)	0	Moderate	NA	0	Moderate
**Body weight change**	Bolus insulin *vs* Liraglutide	2	2.04 (0.54 to 3.55)	-2	0	0	0	Low	3.71 (2.75 to 4.67)	Moderate	0	Moderate	3.23 (2.42 to 4.04)	0.29	Moderate	0	0	Moderate
**Body weight change**	Bolus insulin *vs* Lixisenatide	1	1.80 (-0.12 to 3.72)	-2	0	0	0	Low	1.86 (0.83 to 2.88)	Moderate	0	Moderate	1.84 (0.94 to 2.75)	0.22	Moderate	0	0	Moderate
**Body weight change**	Bolus insulin *vs* Loxenatide	0	NA	NA	NA	NA	NA	NA	0.85 (-1.18 to 2.89)	Low	0	Low	0.85 (-1.18 to 2.89)	0	Low	NA	0	Low

Outcome	Comparisons	k	Direct estimates	D1	D2	D3	D4	Direct Certainty	Indirect estimates	Initial Indirect Certainty	D5	Indirect Certainty	NMA estimates	Prop	Initial NMA Certainty	D6	D7	NMA Certainty
**Body weight change**	Bolus insulin *vs* Meglitinides	0	NA	NA	NA	NA	NA	NA	-0.25 (-1.27 to 0.78)	Low	0	Low	-0.25 (-1.27 to 0.78)	0	Low	NA	0	Low
**Body weight change**	Bolus insulin *vs* Metformin	0	NA	NA	NA	NA	NA	NA	1.85 (1.01 to 2.68)	Low	0	Low	1.85 (1.01 to 2.68)	0	Low	NA	0	Low
**Body weight change**	Bolus insulin *vs* Semaglutide (Oral)	0	NA	NA	NA	NA	NA	NA	3.99 (2.96 to 5.01)	Moderate	0	Moderate	3.99 (2.96 to 5.01)	0	Moderate	NA	-1	Low
**Body weight change**	Bolus insulin *vs* Semaglutide (Subcutaneous)	1	7.10 (5.27 to 8.93)	-2	0	0	0	Low	5.16 (4.12 to 6.20)	Moderate	0	Moderate	5.64 (4.73 to 6.54)	0.25	Moderate	0	0	Moderate
**Body weight change**	Bolus insulin *vs* SGLT2 inhibitors	1	0.90 (-0.94 to 2.74)	-2	0	0	0	Low	3.46 (2.59 to 4.33)	Moderate	0	Moderate	2.99 (2.21 to 3.78)	0.18	Moderate	-1	0	Low
**Body weight change**	Bolus insulin *vs* Standard treatments	0	NA	NA	NA	NA	NA	NA	1.01 (0.24 to 1.79)	Moderate	0	Moderate	1.01 (0.24 to 1.79)	0	Moderate	NA	0	Moderate
**Body weight change**	Bolus insulin *vs* Sulfonylureas	0	NA	NA	NA	NA	NA	NA	-0.77 (-1.58 to 0.04)	Low	0	Low	-0.77 (-1.58 to 0.04)	0	Low	NA	0	Low
**Body weight change**	Bolus insulin *vs* Thiazolidinediones	0	NA	NA	NA	NA	NA	NA	-1.80 (-2.60 to -0.99)	Low	0	Low	-1.80 (-2.60 to -0.99)	0	Low	NA	0	Low
**Body weight change**	Bolus insulin *vs* Tirzepatide	0	NA	NA	NA	NA	NA	NA	9.58 (8.47 to 10.69)	Moderate	0	Moderate	9.58 (8.47 to 10.69)	0	Moderate	NA	0	Moderate
**Body weight change**	DPP-4 inhibitors *vs* Dulaglutide	1	1.72 (-0.13 to 3.57)	0	0	0	0	High	1.68 (1.12 to 2.25)	Moderate	0	Moderate	1.69 (1.15 to 2.23)	0.085	Moderate	0	0	Moderate
**Body weight change**	DPP-4 inhibitors *vs* Efpeglenatide	0	NA	NA	NA	NA	NA	NA	2.87 (1.06 to 4.69)	Moderate	0	Moderate	2.87 (1.06 to 4.69)	0	Moderate	NA	0	Moderate
**Body weight change**	DPP-4 inhibitors *vs* Exenatide extended-release	3	0.87 (-0.25 to 2.00)	0	0	0	0	High	1.54 (0.78 to 2.31)	Moderate	0	Moderate	1.33 (0.70 to 1.96)	0.32	Moderate	0	0	Moderate

Outcome	Comparisons	k	Direct estimates	D1	D2	D3	D4	Direct Certainty	Indirect estimates	Initial Indirect Certainty	D5	Indirect Certainty	NMA estimates	Prop	Initial NMA Certainty	D6	D7	NMA Certainty
**Body weight change**	DPP-4 inhibitors *vs* Exenatide immediate-release	0	NA	NA	NA	NA	NA	NA	2.05 (1.34 to 2.77)	Moderate	0	Moderate	2.05 (1.34 to 2.77)	0	Moderate	NA	0	Moderate
**Body weight change**	DPP-4 inhibitors *vs* Liraglutide	6	2.08 (1.23 to 2.93)	-1	0	0	0	Moderate	2.61 (2.17 to 3.04)	Moderate	0	Moderate	2.50 (2.11 to 2.88)	0.21	Moderate	0	0	Moderate
**Body weight change**	DPP-4 inhibitors *vs* Lixisenatide	1	1.30 (-0.66 to 3.26)	0	0	0	0	High	1.10 (0.48 to 1.72)	Moderate	0	Moderate	1.12 (0.53 to 1.71)	0.091	Moderate	0	0	Moderate
**Body weight change**	DPP-4 inhibitors *vs* Loxenatide	0	NA	NA	NA	NA	NA	NA	0.12 (-1.77 to 2.01)	Low	0	Low	0.12 (-1.77 to 2.01)	0	Low	NA	0	Low
**Body weight change**	DPP-4 inhibitors *vs* Meglitinides	0	NA	NA	NA	NA	NA	NA	-0.97 (-1.66 to -0.29)	Low	0	Low	-0.97 (-1.66 to -0.29)	0	Low	NA	0	Low
**Body weight change**	DPP-4 inhibitors *vs* Metformin	11	1.20 (0.63 to 1.77)	-1	0	0	0	Moderate	1.08 (0.67 to 1.48)	Low	0	Low	1.12 (0.79 to 1.45)	0.33	Low	0	0	Low
**Body weight change**	DPP-4 inhibitors *vs* Semaglutide (Oral)	1	1.90 (-0.01 to 3.81)	-2	0	0	0	Low	3.47 (2.72 to 4.22)	Moderate	0	Moderate	3.26 (2.56 to 3.96)	0.13	Moderate	0	0	Moderate
**Body weight change**	DPP-4 inhibitors *vs* Semaglutide (Subcutaneous)	2	4.06 (2.68 to 5.44)	0	0	0	0	High	5.11 (4.44 to 5.79)	Moderate	0	Moderate	4.91 (4.30 to 5.51)	0.19	Moderate	0	-1	Low
**Body weight change**	DPP-4 inhibitors *vs* SGLT2 inhibitors	14	2.18 (1.66 to 2.71)	-1	0	0	0	Moderate	2.29 (2.02 to 2.56)	Moderate	0	Moderate	2.26 (2.02 to 2.50)	0.21	Moderate	0	0	Moderate
**Body weight change**	DPP-4 inhibitors *vs* Standard treatments	78	0.25 (0.03 to 0.47)	-1	0	0	0	Moderate	0.34 (0.05 to 0.63)	Moderate	0	Moderate	0.28 (0.11 to 0.46)	0.64	Moderate	0	0	Moderate
**Body weight change**	DPP-4 inhibitors *vs* Sulfonylureas	19	-1.46 (-1.89 to -1.03)	-1	0	0	0	Moderate	-1.52 (-1.89 to -1.15)	Moderate	0	Moderate	-1.50 (-1.78 to -1.22)	0.43	Moderate	0	0	Moderate
**Body weight change**	DPP-4 inhibitors *vs* Thiazolidinediones	11	-1.80 (-2.40 to -1.20)	-1	0	0	0	Moderate	-2.72 (-3.03 to -2.41)	Low	0	Low	-2.52 (-2.80 to -2.25)	0.21	Low	-2	0	Very low

Outcome	Comparisons	k	Direct estimates	D1	D2	D3	D4	Direct Certainty	Indirect estimates	Initial Indirect Certainty	D5	Indirect Certainty	NMA estimates	Prop	Initial NMA Certainty	D6	D7	NMA Certainty
**Body weight change**	DPP-4 inhibitors *vs* Tirzepatide	0	NA	NA	NA	NA	NA	NA	8.85 (8.02 to 9.69)	Moderate	0	Moderate	8.85 (8.02 to 9.69)	0	Moderate	NA	0	Moderate
**Body weight change**	Dulaglutide *vs* Efpeglenatide	0	NA	NA	NA	NA	NA	NA	1.19 (-0.69 to 3.07)	Moderate	0	Moderate	1.19 (-0.69 to 3.07)	0	Moderate	NA	0	Moderate
**Body weight change**	Dulaglutide *vs* Exenatide extended-release	1	1.66 (-0.86 to 4.18)	-2	0	0	0	Low	-0.57 (-1.39 to 0.25)	Moderate	0	Moderate	-0.36 (-1.14 to 0.42)	0.096	Moderate	0	0	Moderate
**Body weight change**	Dulaglutide *vs* Exenatide immediate-release	0	NA	NA	NA	NA	NA	NA	0.37 (-0.49 to 1.22)	Moderate	0	Moderate	0.37 (-0.49 to 1.22)	0	Moderate	NA	0	Moderate
**Body weight change**	Dulaglutide *vs* Liraglutide	0	NA	NA	NA	NA	NA	NA	0.81 (0.19 to 1.43)	Moderate	0	Moderate	0.81 (0.19 to 1.43)	0	Moderate	NA	0	Moderate
**Body weight change**	Dulaglutide *vs* Lixisenatide	0	NA	NA	NA	NA	NA	NA	-0.57 (-1.34 to 0.20)	Moderate	0	Moderate	-0.57 (-1.34 to 0.20)	0	Moderate	NA	0	Moderate
**Body weight change**	Dulaglutide *vs* Loxenatide	0	NA	NA	NA	NA	NA	NA	-1.56 (-3.52 to 0.39)	Low	0	Low	-1.56 (-3.52 to 0.39)	0	Low	NA	0	Low
**Body weight change**	Dulaglutide *vs* Meglitinides	0	NA	NA	NA	NA	NA	NA	-2.66 (-3.51 to -1.81)	Moderate	0	Moderate	-2.66 (-3.51 to -1.81)	0	Moderate	NA	0	Moderate
**Body weight change**	Dulaglutide *vs* Metformin	1	-0.07 (-1.97 to 1.83)	0	0	0	0	High	-0.62 (-1.25 to 0.00)	Moderate	0	Moderate	-0.57 (-1.16 to 0.02)	0.098	Moderate	0	0	Moderate
**Body weight change**	Dulaglutide *vs* Semaglutide (Oral)	0	NA	NA	NA	NA	NA	NA	1.58 (0.72 to 2.43)	Moderate	0	Moderate	1.58 (0.72 to 2.43)	0	Moderate	NA	0	Moderate
**Body weight change**	Dulaglutide *vs* Semaglutide (Subcutaneous)	0	NA	NA	NA	NA	NA	NA	3.22 (2.44 to 4.00)	Moderate	0	Moderate	3.22 (2.44 to 4.00)	0	Moderate	NA	0	Moderate
**Body weight change**	Dulaglutide *vs* SGLT2 inhibitors	0	NA	NA	NA	NA	NA	NA	0.58 (0.02 to 1.13)	Moderate	0	Moderate	0.58 (0.02 to 1.13)	0	Moderate	NA	0	Moderate

Outcome	Comparisons	k	Direct estimates	D1	D2	D3	D4	Direct Certainty	Indirect estimates	Initial Indirect Certainty	D5	Indirect Certainty	NMA estimates	Prop	Initial NMA Certainty	D6	D7	NMA Certainty
**Body weight change**	Dulaglutide *vs* Standard treatments	9	-1.66 (-2.35 to -0.96)	0	0	0	0	High	-1.07 (-1.86 to -0.27)	Moderate	0	Moderate	-1.40 (-1.93 to -0.88)	0.57	High	0	0	High
**Body weight change**	Dulaglutide *vs* Sulfonylureas	1	-2.35 (-4.20 to -0.50)	0	0	0	0	High	-3.27 (-3.87 to -2.67)	Moderate	0	Moderate	-3.18 (-3.76 to -2.61)	0.096	Moderate	0	0	Moderate
**Body weight change**	Dulaglutide *vs* Thiazolidinediones	0	NA	NA	NA	NA	NA	NA	-4.21 (-4.78 to -3.64)	Moderate	0	Moderate	-4.21 (-4.78 to -3.64)	0	Moderate	NA	0	Moderate
**Body weight change**	Dulaglutide *vs* Tirzepatide	1	3.71 (1.18 to 6.24)	0	0	0	0	High	7.71 (6.71 to 8.72)	Moderate	0	Moderate	7.17 (6.23 to 8.10)	0.14	Moderate	-2	0	Very low
**Body weight change**	Efpeglenatide *vs* Exenatide extended-release	0	NA	NA	NA	NA	NA	NA	-1.54 (-3.46 to 0.37)	Moderate	0	Moderate	-1.54 (-3.46 to 0.37)	0	Moderate	NA	0	Moderate
**Body weight change**	Efpeglenatide *vs* Exenatide immediate-release	0	NA	NA	NA	NA	NA	NA	-0.82 (-2.76 to 1.11)	Moderate	0	Moderate	-0.82 (-2.76 to 1.11)	0	Moderate	NA	0	Moderate
**Body weight change**	Efpeglenatide *vs* Liraglutide	0	NA	NA	NA	NA	NA	NA	-0.38 (-2.22 to 1.47)	Moderate	0	Moderate	-0.38 (-2.22 to 1.47)	0	Moderate	NA	0	Moderate
**Body weight change**	Efpeglenatide *vs* Lixisenatide	0	NA	NA	NA	NA	NA	NA	-1.76 (-3.65 to 0.13)	Moderate	0	Moderate	-1.76 (-3.65 to 0.13)	0	Moderate	NA	0	Moderate
**Body weight change**	Efpeglenatide *vs* Loxenatide	0	NA	NA	NA	NA	NA	NA	-2.75 (-5.36 to -0.14)	Moderate	0	Moderate	-2.75 (-5.36 to -0.14)	0	Moderate	NA	-1	Low
**Body weight change**	Efpeglenatide *vs* Meglitinides	0	NA	NA	NA	NA	NA	NA	-3.85 (-5.78 to -1.92)	Moderate	0	Moderate	-3.85 (-5.78 to -1.92)	0	Moderate	NA	-1	Low
**Body weight change**	Efpeglenatide *vs* Metformin	0	NA	NA	NA	NA	NA	NA	-1.76 (-3.59 to 0.08)	Moderate	0	Moderate	-1.76 (-3.59 to 0.08)	0	Moderate	NA	0	Moderate
**Body weight change**	Efpeglenatide *vs* Semaglutide (Oral)	0	NA	NA	NA	NA	NA	NA	0.39 (-1.54 to 2.32)	Moderate	0	Moderate	0.39 (-1.54 to 2.32)	0	Moderate	NA	0	Moderate

Outcome	Comparisons	k	Direct estimates	D1	D2	D3	D4	Direct Certainty	Indirect estimates	Initial Indirect Certainty	D5	Indirect Certainty	NMA estimates	Prop	Initial NMA Certainty	D6	D7	NMA Certainty
**Body weight change**	Efpeglenatide *vs* Semaglutide (Subcutaneous)	0	NA	NA	NA	NA	NA	NA	2.03 (0.13 to 3.93)	Moderate	0	Moderate	2.03 (0.13 to 3.93)	0	Moderate	NA	0	Moderate
**Body weight change**	Efpeglenatide *vs* SGLT2 inhibitors	0	NA	NA	NA	NA	NA	NA	-0.61 (-2.43 to 1.21)	Moderate	0	Moderate	-0.61 (-2.43 to 1.21)	0	Moderate	NA	0	Moderate
**Body weight change**	Efpeglenatide *vs* Standard treatments	1	-2.59 (-4.40 to -0.78)	0	0	0	0	High	NA	NA	0	NA	-2.59 (-4.40 to -0.78)	1	High	NA	0	High
**Body weight change**	Efpeglenatide *vs* Sulfonylureas	0	NA	NA	NA	NA	NA	NA	-4.37 (-6.20 to -2.54)	Moderate	0	Moderate	-4.37 (-6.20 to -2.54)	0	Moderate	NA	-1	Low
**Body weight change**	Efpeglenatide *vs* Thiazolidinediones	0	NA	NA	NA	NA	NA	NA	-5.40 (-7.22 to -3.58)	Moderate	0	Moderate	-5.40 (-7.22 to -3.58)	0	Moderate	NA	0	Moderate
**Body weight change**	Efpeglenatide *vs* Tirzepatide	0	NA	NA	NA	NA	NA	NA	5.98 (4.00 to 7.97)	Moderate	0	Moderate	5.98 (4.00 to 7.97)	0	Moderate	NA	0	Moderate
**Body weight change**	Exenatide extended-release *vs* Exenatide immediate-release	0	NA	NA	NA	NA	NA	NA	0.72 (-0.20 to 1.64)	Moderate	0	Moderate	0.72 (-0.20 to 1.64)	0	Moderate	NA	0	Moderate
**Body weight change**	Exenatide extended-release *vs* Liraglutide	0	NA	NA	NA	NA	NA	NA	1.17 (0.46 to 1.87)	Moderate	0	Moderate	1.17 (0.46 to 1.87)	0	Moderate	NA	0	Moderate
**Body weight change**	Exenatide extended-release *vs* Lixisenatide	0	NA	NA	NA	NA	NA	NA	-0.21 (-1.06 to 0.63)	Moderate	0	Moderate	-0.21 (-1.06 to 0.63)	0	Moderate	NA	0	Moderate
**Body weight change**	Exenatide extended-release *vs* Loxenatide	0	NA	NA	NA	NA	NA	NA	-1.21 (-3.19 to 0.78)	Low	0	Low	-1.21 (-3.19 to 0.78)	0	Low	NA	0	Low
**Body weight change**	Exenatide extended-release *vs* Meglitinides	0	NA	NA	NA	NA	NA	NA	-2.30 (-3.22 to -1.39)	Moderate	0	Moderate	-2.30 (-3.22 to -1.39)	0	Moderate	NA	0	Moderate

Outcome	Comparisons	k	Direct estimates	D1	D2	D3	D4	Direct Certainty	Indirect estimates	Initial Indirect Certainty	D5	Indirect Certainty	NMA estimates	Prop	Initial NMA Certainty	D6	D7	NMA Certainty
**Body weight change**	Exenatide extended-release *vs* Metformin	1	0.00 (-1.86 to 1.86)	0	0	0	0	High	-0.25 (-0.98 to 0.49)	Moderate	0	Moderate	-0.21 (-0.90 to 0.47)	0.14	Moderate	0	0	Moderate
**Body weight change**	Exenatide extended-release *vs* Semaglutide (Oral)	0	NA	NA	NA	NA	NA	NA	1.93 (1.01 to 2.86)	Moderate	0	Moderate	1.93 (1.01 to 2.86)	0	Moderate	NA	0	Moderate
**Body weight change**	Exenatide extended-release *vs* Semaglutide (Subcutaneous)	0	NA	NA	NA	NA	NA	NA	3.58 (2.73 to 4.43)	Moderate	0	Moderate	3.58 (2.73 to 4.43)	0	Moderate	NA	0	Moderate
**Body weight change**	Exenatide extended-release *vs* SGLT2 inhibitors	1	2.22 (0.00 to 4.44)	0	0	0	0	High	0.81 (0.14 to 1.49)	Moderate	0	Moderate	0.93 (0.29 to 1.58)	0.085	Moderate	0	0	Moderate
**Body weight change**	Exenatide extended-release *vs* Standard treatments	4	-1.30 (-2.46 to -0.15)	0	0	0	0	High	-0.94 (-1.69 to -0.19)	Moderate	0	Moderate	-1.05 (-1.67 to -0.42)	0.29	Moderate	0	0	Moderate
**Body weight change**	Exenatide extended-release *vs* Sulfonylureas	0	NA	NA	NA	NA	NA	NA	-2.83 (-3.50 to -2.16)	Moderate	0	Moderate	-2.83 (-3.50 to -2.16)	0	Moderate	NA	0	Moderate
**Body weight change**	Exenatide extended-release *vs* Thiazolidinediones	2	-4.28 (-5.65 to -2.91)	-1	0	0	0	Moderate	-3.73 (-4.48 to -2.99)	Moderate	0	Moderate	-3.86 (-4.51 to -3.20)	0.23	Moderate	0	0	Moderate
**Body weight change**	Exenatide extended-release *vs* Tirzepatide	0	NA	NA	NA	NA	NA	NA	7.52 (6.51 to 8.54)	Moderate	0	Moderate	7.52 (6.51 to 8.54)	0	Moderate	NA	0	Moderate
**Body weight change**	Exenatide immediate-release *vs* Liraglutide	0	NA	NA	NA	NA	NA	NA	0.44 (-0.33 to 1.22)	Moderate	0	Moderate	0.44 (-0.33 to 1.22)	0	Moderate	NA	0	Moderate
**Body weight change**	Exenatide immediate-release *vs* Lixisenatide	0	NA	NA	NA	NA	NA	NA	-0.94 (-1.84 to -0.04)	Moderate	0	Moderate	-0.94 (-1.84 to -0.04)	0	Moderate	NA	0	Moderate
**Body weight change**	Exenatide immediate-release *vs* Loxenatide	0	NA	NA	NA	NA	NA	NA	-1.93 (-3.93 to 0.08)	Low	0	Low	-1.93 (-3.93 to 0.08)	0	Low	NA	0	Low

Outcome	Comparisons	k	Direct estimates	D1	D2	D3	D4	Direct Certainty	Indirect estimates	Initial Indirect Certainty	D5	Indirect Certainty	NMA estimates	Prop	Initial NMA Certainty	D6	D7	NMA Certainty
**Body weight change**	Exenatide immediate-release *vs* Meglitinides	0	NA	NA	NA	NA	NA	NA	-3.03 (-4.00 to -2.06)	Moderate	0	Moderate	-3.03 (-4.00 to -2.06)	0	Moderate	NA	0	Moderate
**Body weight change**	Exenatide immediate-release *vs* Metformin	1	-1.99 (-3.93 to -0.05)	0	0	0	0	High	-0.75 (-1.56 to 0.06)	Moderate	0	Moderate	-0.94 (-1.68 to -0.19)	0.15	Moderate	0	0	Moderate
**Body weight change**	Exenatide immediate-release *vs* Semaglutide (Oral)	0	NA	NA	NA	NA	NA	NA	1.21 (0.23 to 2.18)	Moderate	0	Moderate	1.21 (0.23 to 2.18)	0	Moderate	NA	0	Moderate
**Body weight change**	Exenatide immediate-release *vs* Semaglutide (Subcutaneous)	0	NA	NA	NA	NA	NA	NA	2.85 (1.95 to 3.76)	Moderate	0	Moderate	2.85 (1.95 to 3.76)	0	Moderate	NA	0	Moderate
**Body weight change**	Exenatide immediate-release *vs* SGLT2 inhibitors	0	NA	NA	NA	NA	NA	NA	0.21 (-0.51 to 0.93)	Moderate	0	Moderate	0.21 (-0.51 to 0.93)	0	Moderate	NA	0	Moderate
**Body weight change**	Exenatide immediate-release *vs* Standard treatments	6	-1.55 (-2.40 to -0.70)	-1	0	0	0	Moderate	-2.22 (-3.43 to -1.00)	Moderate	0	Moderate	-1.77 (-2.47 to -1.07)	0.67	Moderate	0	0	Moderate
**Body weight change**	Exenatide immediate-release *vs* Sulfonylureas	0	NA	NA	NA	NA	NA	NA	-3.55 (-4.29 to -2.80)	Moderate	0	Moderate	-3.55 (-4.29 to -2.80)	0	Moderate	NA	0	Moderate
**Body weight change**	Exenatide immediate-release *vs* Thiazolidinediones	0	NA	NA	NA	NA	NA	NA	-4.58 (-5.31 to -3.84)	Low	0	Low	-4.58 (-5.31 to -3.84)	0	Low	NA	-1	Very low
**Body weight change**	Exenatide immediate-release *vs* Tirzepatide	0	NA	NA	NA	NA	NA	NA	6.80 (5.74 to 7.87)	Moderate	0	Moderate	6.80 (5.74 to 7.87)	0	Moderate	NA	0	Moderate
**Body weight change**	Liraglutide *vs* Lixisenatide	0	NA	NA	NA	NA	NA	NA	-1.38 (-2.05 to -0.71)	Moderate	0	Moderate	-1.38 (-2.05 to -0.71)	0	Moderate	NA	0	Moderate
**Body weight change**	Liraglutide *vs* Loxenatide	0	NA	NA	NA	NA	NA	NA	-2.37 (-4.29 to -0.46)	Low	0	Low	-2.37 (-4.29 to -0.46)	0	Low	NA	0	Low

Outcome	Comparisons	k	Direct estimates	D1	D2	D3	D4	Direct Certainty	Indirect estimates	Initial Indirect Certainty	D5	Indirect Certainty	NMA estimates	Prop	Initial NMA Certainty	D6	D7	NMA Certainty
**Body weight change**	Liraglutide *vs* Meglitinides	0	NA	NA	NA	NA	NA	NA	-3.47 (-4.24 to -2.71)	Moderate	0	Moderate	-3.47 (-4.24 to -2.71)	0	Moderate	NA	0	Moderate
**Body weight change**	Liraglutide *vs* Metformin	0	NA	NA	NA	NA	NA	NA	-1.38 (-1.86 to -0.90)	Moderate	0	Moderate	-1.38 (-1.86 to -0.90)	0	Moderate	NA	0	Moderate
**Body weight change**	Liraglutide *vs* Semaglutide (Oral)	1	1.30 (-0.66 to 3.26)	0	0	0	0	High	0.67 (-0.16 to 1.49)	Moderate	0	Moderate	0.76 (0.00 to 1.52)	0.15	Moderate	0	0	Moderate
**Body weight change**	Liraglutide *vs* Semaglutide (Subcutaneous)	0	NA	NA	NA	NA	NA	NA	2.41 (1.73 to 3.09)	Moderate	0	Moderate	2.41 (1.73 to 3.09)	0	Moderate	NA	0	Moderate
**Body weight change**	Liraglutide *vs* SGLT2 inhibitors	0	NA	NA	NA	NA	NA	NA	-0.23 (-0.64 to 0.18)	Moderate	0	Moderate	-0.23 (-0.64 to 0.18)	0	Moderate	NA	0	Moderate
**Body weight change**	Liraglutide *vs* Standard treatments	20	-2.58 (-3.05 to -2.10)	0	0	0	0	High	-1.68 (-2.26 to -1.10)	Moderate	0	Moderate	-2.21 (-2.58 to -1.85)	0.59	High	-1	0	Moderate
**Body weight change**	Liraglutide *vs* Sulfonylureas	3	-3.37 (-4.45 to -2.29)	0	0	0	0	High	-4.12 (-4.60 to -3.64)	Moderate	0	Moderate	-3.99 (-4.43 to -3.56)	0.16	Moderate	0	0	Moderate
**Body weight change**	Liraglutide *vs* Thiazolidinediones	1	-2.30 (-4.26 to -0.34)	0	0	0	0	High	-5.16 (-5.60 to -4.72)	Moderate	0	Moderate	-5.02 (-5.45 to -4.59)	0.048	Moderate	-2	0	Very low
**Body weight change**	Liraglutide *vs* Tirzepatide	0	NA	NA	NA	NA	NA	NA	6.36 (5.47 to 7.24)	Moderate	0	Moderate	6.36 (5.47 to 7.24)	0	Moderate	NA	0	Moderate
**Body weight change**	Lixisenatide *vs* Loxenatide	0	NA	NA	NA	NA	NA	NA	-0.99 (-2.96 to 0.97)	Low	0	Low	-0.99 (-2.96 to 0.97)	0	Low	NA	0	Low
**Body weight change**	Lixisenatide *vs* Meglitinides	0	NA	NA	NA	NA	NA	NA	-2.09 (-2.98 to -1.21)	Moderate	0	Moderate	-2.09 (-2.98 to -1.21)	0	Moderate	NA	0	Moderate
**Body weight change**	Lixisenatide *vs* Metformin	0	NA	NA	NA	NA	NA	NA	0.00 (-0.65 to 0.66)	Moderate	0	Moderate	0.00 (-0.65 to 0.66)	0	Moderate	NA	0	Moderate

Outcome	Comparisons	k	Direct estimates	D1	D2	D3	D4	Direct Certainty	Indirect estimates	Initial Indirect Certainty	D5	Indirect Certainty	NMA estimates	Prop	Initial NMA Certainty	D6	D7	NMA Certainty
**Body weight change**	Lixisenatide *vs* Semaglutide (Oral)	0	NA	NA	NA	NA	NA	NA	2.15 (1.26 to 3.04)	Moderate	0	Moderate	2.15 (1.26 to 3.04)	0	Moderate	NA	0	Moderate
**Body weight change**	Lixisenatide *vs* Semaglutide (Subcutaneous)	0	NA	NA	NA	NA	NA	NA	3.79 (2.98 to 4.61)	Moderate	0	Moderate	3.79 (2.98 to 4.61)	0	Moderate	NA	0	Moderate
**Body weight change**	Lixisenatide *vs* SGLT2 inhibitors	0	NA	NA	NA	NA	NA	NA	1.15 (0.55 to 1.75)	Moderate	0	Moderate	1.15 (0.55 to 1.75)	0	Moderate	NA	0	Moderate
**Body weight change**	Lixisenatide *vs* Standard treatments	10	-0.82 (-1.44 to -0.20)	0	0	0	0	High	-0.91 (-2.34 to 0.53)	Moderate	0	Moderate	-0.83 (-1.40 to -0.26)	0.84	High	0	0	High
**Body weight change**	Lixisenatide *vs* Sulfonylureas	0	NA	NA	NA	NA	NA	NA	-2.61 (-3.24 to -1.98)	Moderate	0	Moderate	-2.61 (-3.24 to -1.98)	0	Moderate	NA	0	Moderate
**Body weight change**	Lixisenatide *vs* Thiazolidinediones	0	NA	NA	NA	NA	NA	NA	-3.64 (-4.26 to -3.02)	Moderate	0	Moderate	-3.64 (-4.26 to -3.02)	0	Moderate	NA	0	Moderate
**Body weight change**	Lixisenatide *vs* Tirzepatide	0	NA	NA	NA	NA	NA	NA	7.74 (6.74 to 8.74)	Moderate	0	Moderate	7.74 (6.74 to 8.74)	0	Moderate	NA	0	Moderate
**Body weight change**	Loxenatide *vs* Meglitinides	0	NA	NA	NA	NA	NA	NA	-1.10 (-3.10 to 0.90)	Low	0	Low	-1.10 (-3.10 to 0.90)	0	Low	NA	0	Low
**Body weight change**	Loxenatide *vs* Metformin	0	NA	NA	NA	NA	NA	NA	0.99 (-0.92 to 2.90)	Low	0	Low	0.99 (-0.92 to 2.90)	0	Low	NA	0	Low
**Body weight change**	Loxenatide *vs* Semaglutide (Oral)	0	NA	NA	NA	NA	NA	NA	3.14 (1.14 to 5.14)	Low	0	Low	3.14 (1.14 to 5.14)	0	Low	NA	-1	Very low
**Body weight change**	Loxenatide *vs* Semaglutide (Subcutaneous)	0	NA	NA	NA	NA	NA	NA	4.78 (2.81 to 6.76)	Low	0	Low	4.78 (2.81 to 6.76)	0	Low	NA	-1	Very low
**Body weight change**	Loxenatide *vs* SGLT2 inhibitors	0	NA	NA	NA	NA	NA	NA	2.14 (0.25 to 4.03)	Low	0	Low	2.14 (0.25 to 4.03)	0	Low	NA	0	Low

Outcome	Comparisons	k	Direct estimates	D1	D2	D3	D4	Direct Certainty	Indirect estimates	Initial Indirect Certainty	D5	Indirect Certainty	NMA estimates	Prop	Initial NMA Certainty	D6	D7	NMA Certainty
**Body weight change**	Loxenatide *vs* Standard treatments	1	0.16 (-1.72 to 2.04)	-2	0	0	0	Low	NA	NA	0	NA	0.16 (-1.72 to 2.04)	1	Low	NA	0	Low
**Body weight change**	Loxenatide *vs* Sulfonylureas	0	NA	NA	NA	NA	NA	NA	-1.62 (-3.52 to 0.28)	Low	0	Low	-1.62 (-3.52 to 0.28)	0	Low	NA	0	Low
**Body weight change**	Loxenatide *vs* Thiazolidinediones	0	NA	NA	NA	NA	NA	NA	-2.65 (-4.55 to -0.75)	Low	0	Low	-2.65 (-4.55 to -0.75)	0	Low	NA	0	Low
**Body weight change**	Loxenatide *vs* Tirzepatide	0	NA	NA	NA	NA	NA	NA	8.73 (6.68 to 10.78)	Moderate	0	Moderate	8.73 (6.68 to 10.78)	0	Moderate	NA	0	Moderate
**Body weight change**	Meglitinides *vs* Metformin	1	2.51 (0.16 to 4.86)	0	0	0	0	High	2.05 (1.28 to 2.81)	Low	0	Low	2.09 (1.37 to 2.82)	0.095	Low	0	0	Low
**Body weight change**	Meglitinides *vs* Semaglutide (Oral)	0	NA	NA	NA	NA	NA	NA	4.24 (3.27 to 5.20)	Moderate	0	Moderate	4.24 (3.27 to 5.20)	0	Moderate	NA	-1	Low
**Body weight change**	Meglitinides *vs* Semaglutide (Subcutaneous)	0	NA	NA	NA	NA	NA	NA	5.88 (4.98 to 6.78)	Moderate	0	Moderate	5.88 (4.98 to 6.78)	0	Moderate	NA	0	Moderate
**Body weight change**	Meglitinides *vs* SGLT2 inhibitors	0	NA	NA	NA	NA	NA	NA	3.24 (2.54 to 3.94)	Moderate	0	Moderate	3.24 (2.54 to 3.94)	0	Moderate	NA	0	Moderate
**Body weight change**	Meglitinides *vs* Standard treatments	4	1.01 (-0.07 to 2.09)	-1	0	0	0	Moderate	1.42 (0.55 to 2.29)	Low	0	Low	1.26 (0.58 to 1.94)	0.39	Low	0	0	Low
**Body weight change**	Meglitinides *vs* Sulfonylureas	4	-0.48 (-1.50 to 0.55)	-1	0	0	0	Moderate	-0.56 (-1.47 to 0.35)	Moderate	0	Moderate	-0.52 (-1.20 to 0.16)	0.44	Moderate	0	0	Moderate
**Body weight change**	Meglitinides *vs* Thiazolidinediones	2	-1.23 (-3.10 to 0.64)	-2	0	0	0	Low	-1.60 (-2.36 to -0.85)	Low	0	Low	-1.55 (-2.25 to -0.85)	0.14	Low	0	0	Low
**Body weight change**	Meglitinides *vs* Tirzepatide	0	NA	NA	NA	NA	NA	NA	9.83 (8.76 to 10.89)	Moderate	0	Moderate	9.83 (8.76 to 10.89)	0	Moderate	NA	0	Moderate

Outcome	Comparisons	k	Direct estimates	D1	D2	D3	D4	Direct Certainty	Indirect estimates	Initial Indirect Certainty	D5	Indirect Certainty	NMA estimates	Prop	Initial NMA Certainty	D6	D7	NMA Certainty
**Body weight change**	Metformin *vs* Semaglutide (Oral)	0	NA	NA	NA	NA	NA	NA	2.14 (1.39 to 2.90)	Moderate	0	Moderate	2.14 (1.39 to 2.90)	0	Moderate	NA	0	Moderate
**Body weight change**	Metformin *vs* Semaglutide (Subcutaneous)	0	NA	NA	NA	NA	NA	NA	3.79 (3.12 to 4.46)	Moderate	0	Moderate	3.79 (3.12 to 4.46)	0	Moderate	NA	0	Moderate
**Body weight change**	Metformin *vs* SGLT2 inhibitors	5	1.71 (0.84 to 2.58)	-1	0	0	0	Moderate	1.03 (0.64 to 1.42)	Moderate	0	Moderate	1.15 (0.79 to 1.50)	0.17	Moderate	0	0	Moderate
**Body weight change**	Metformin *vs* Standard treatments	8	-0.65 (-1.35 to 0.05)	-1	0	0	0	Moderate	-0.88 (-1.25 to -0.52)	Moderate	0	Moderate	-0.83 (-1.16 to -0.51)	0.22	Moderate	0	0	Moderate
**Body weight change**	Metformin *vs* Sulfonylureas	7	-2.54 (-3.35 to -1.72)	-2	0	0	0	Low	-2.64 (-3.06 to -2.21)	Moderate	0	Moderate	-2.61 (-2.99 to -2.24)	0.21	Moderate	0	0	Moderate
**Body weight change**	Metformin *vs* Thiazolidinediones	10	-4.12 (-4.77 to -3.47)	-1	0	0	0	Moderate	-3.42 (-3.86 to -2.99)	Low	0	Low	-3.64 (-4.00 to -3.28)	0.31	Low	0	0	Low
**Body weight change**	Metformin *vs* Tirzepatide	0	NA	NA	NA	NA	NA	NA	7.74 (6.86 to 8.62)	Moderate	0	Moderate	7.74 (6.86 to 8.62)	0	Moderate	NA	0	Moderate
**Body weight change**	Semaglutide (Oral) *vs* Semaglutide (Subcutaneous)	1	1.60 (-0.74 to 3.94)	0	0	0	0	High	1.65 (0.70 to 2.61)	Moderate	0	Moderate	1.65 (0.76 to 2.53)	0.14	Moderate	0	0	Moderate
**Body weight change**	Semaglutide (Oral) *vs* SGLT2 inhibitors	1	-0.30 (-2.21 to 1.61)	-2	0	0	0	Low	-1.11 (-1.87 to -0.35)	Moderate	0	Moderate	-1.00 (-1.70 to -0.29)	0.14	Moderate	0	0	Moderate
**Body weight change**	Semaglutide (Oral) *vs* Standard treatments	6	-3.35 (-4.18 to -2.52)	0	0	0	0	High	-2.18 (-3.40 to -0.97)	Moderate	0	Moderate	-2.98 (-3.66 to -2.29)	0.68	High	0	0	High
**Body weight change**	Semaglutide (Oral) *vs* Sulfonylureas	0	NA	NA	NA	NA	NA	NA	-4.76 (-5.49 to -4.02)	Moderate	0	Moderate	-4.76 (-5.49 to -4.02)	0	Moderate	NA	-1	Low
**Body weight change**	Semaglutide (Oral) *vs* Thiazolidinediones	0	NA	NA	NA	NA	NA	NA	-5.79 (-6.52 to -5.06)	Moderate	0	Moderate	-5.79 (-6.52 to -5.06)	0	Moderate	NA	0	Moderate

Outcome	Comparisons	k	Direct estimates	D1	D2	D3	D4	Direct Certainty	Indirect estimates	Initial Indirect Certainty	D5	Indirect Certainty	NMA estimates	Prop	Initial NMA Certainty	D6	D7	NMA Certainty
**Body weight change**	Semaglutide (Oral) *vs* Tirzepatide	0	NA	NA	NA	NA	NA	NA	5.59 (4.53 to 6.66)	Moderate	0	Moderate	5.59 (4.53 to 6.66)	0	Moderate	NA	0	Moderate
**Body weight change**	Semaglutide (Subcutaneous) *vs* SGLT2 inhibitors	1	-1.40 (-3.26 to 0.46)	0	0	0	0	High	-2.80 (-3.45 to -2.14)	Moderate	0	Moderate	-2.64 (-3.26 to -2.03)	0.11	Moderate	0	0	Moderate
**Body weight change**	Semaglutide (Subcutaneous) *vs* Standard treatments	6	-4.89 (-5.78 to -4.00)	0	0	0	0	High	-4.41 (-5.21 to -3.61)	Moderate	0	Moderate	-4.62 (-5.22 to -4.03)	0.45	Moderate	0	0	Moderate
**Body weight change**	Semaglutide (Subcutaneous) *vs* Sulfonylureas	0	NA	NA	NA	NA	NA	NA	-6.40 (-7.05 to -5.76)	Moderate	0	Moderate	-6.40 (-7.05 to -5.76)	0	Moderate	NA	0	Moderate
**Body weight change**	Semaglutide (Subcutaneous) *vs* Thiazolidinediones	0	NA	NA	NA	NA	NA	NA	-7.43 (-8.07 to -6.79)	Moderate	0	Moderate	-7.43 (-8.07 to -6.79)	0	Moderate	NA	0	Moderate
**Body weight change**	Semaglutide (Subcutaneous) *vs* Tirzepatide	2	4.06 (2.43 to 5.69)	0	0	0	0	High	3.89 (2.77 to 5.02)	Moderate	0	Moderate	3.95 (3.02 to 4.87)	0.32	Moderate	0	0	Moderate
**Body weight change**	SGLT2 inhibitors *vs* Standard treatments	70	-1.91 (-2.14 to -1.67)	0	0	0	0	High	-2.17 (-2.55 to -1.79)	Moderate	0	Moderate	-1.98 (-2.18 to -1.78)	0.72	High	0	0	High
**Body weight change**	SGLT2 inhibitors *vs* Sulfonylureas	9	-4.28 (-4.94 to -3.63)	0	0	0	0	High	-3.60 (-3.96 to -3.24)	Moderate	0	Moderate	-3.76 (-4.07 to -3.45)	0.23	Moderate	0	0	Moderate
**Body weight change**	SGLT2 inhibitors *vs* Thiazolidinediones	3	-4.30 (-5.57 to -3.03)	-1	0	0	0	Moderate	-4.82 (-5.14 to -4.50)	Moderate	0	Moderate	-4.79 (-5.10 to -4.48)	0.059	Moderate	0	-1	Low
**Body weight change**	SGLT2 inhibitors *vs* Tirzepatide	0	NA	NA	NA	NA	NA	NA	6.59 (5.75 to 7.44)	Moderate	0	Moderate	6.59 (5.75 to 7.44)	0	Moderate	NA	0	Moderate
**Body weight change**	Sulfonylureas *vs* Standard treatments	4	2.70 (1.74 to 3.67)	-1	0	0	0	Moderate	1.69 (1.40 to 1.99)	Moderate	0	Moderate	1.78 (1.50 to 2.06)	0.086	Moderate	0	0	Moderate
**Body weight change**	Thiazolidinediones *vs* Standard treatments	32	3.05 (2.67 to 3.42)	-1	0	0	-1	Low	2.60 (2.24 to 2.95)	Moderate	0	Moderate	2.81 (2.55 to 3.07)	0.47	Moderate	0	0	Moderate

Outcome	Comparisons	k	Direct estimates	D1	D2	D3	D4	Direct Certainty	Indirect estimates	Initial Indirect Certainty	D5	Indirect Certainty	NMA estimates	Prop	Initial NMA Certainty	D6	D7	NMA Certainty
**Body weight change**	Tirzepatide *vs* Standard treatments	4	-8.06 (-9.30 to -6.82)	0	0	0	0	High	-8.98 (-10.08 to -7.87)	Moderate	0	Moderate	-8.57 (-9.40 to -7.75)	0.44	Moderate	0	0	Moderate
**Body weight change**	Sulfonylureas *vs* Thiazolidinediones	17	-1.17 (-1.66 to -0.68)	-1	0	0	0	Moderate	-0.93 (-1.34 to -0.53)	Low	0	Low	-1.03 (-1.34 to -0.72)	0.41	Low	0	0	Low
**Body weight change**	Sulfonylureas *vs* Tirzepatide	0	NA	NA	NA	NA	NA	NA	10.35 (9.49 to 11.21)	Moderate	0	Moderate	10.35 (9.49 to 11.21)	0	Moderate	NA	0	Moderate
**Body weight change**	Thiazolidinediones *vs* Tirzepatide	0	NA	NA	NA	NA	NA	NA	11.38 (10.52 to 12.24)	Moderate	0	Moderate	11.38 (10.52 to 12.24)	0	Moderate	NA	0	Moderate

5.2 Percentage contribution matrix

Notes: The study calculated the percentage contribution matrix of each direct comparison to each network comparison using a random walk approach. A random walk on a graph is a stochastic process that describes a succession of random walk between vertices which are connected by an edge. The weight of an edge relates to the probability that the walker moves along that edge. The approach uses the graph representation of the network meta-analysis to construct the transition matrix for a random walk on the network structure. The approach shows that the net number of times a walker crosses each edge of the network is related to the evidence flow network. See details in the paper by Davies and colleagues.

Reference:

Davies, A. L., Papakonstantinou, T., Nikolakopoulou, A., Rücker, G., & Galla, T. (2022). Network meta-analysis and random walks. Statistics in Medicine, 41(12), 2091-2114.

5.2.1 All-cause death

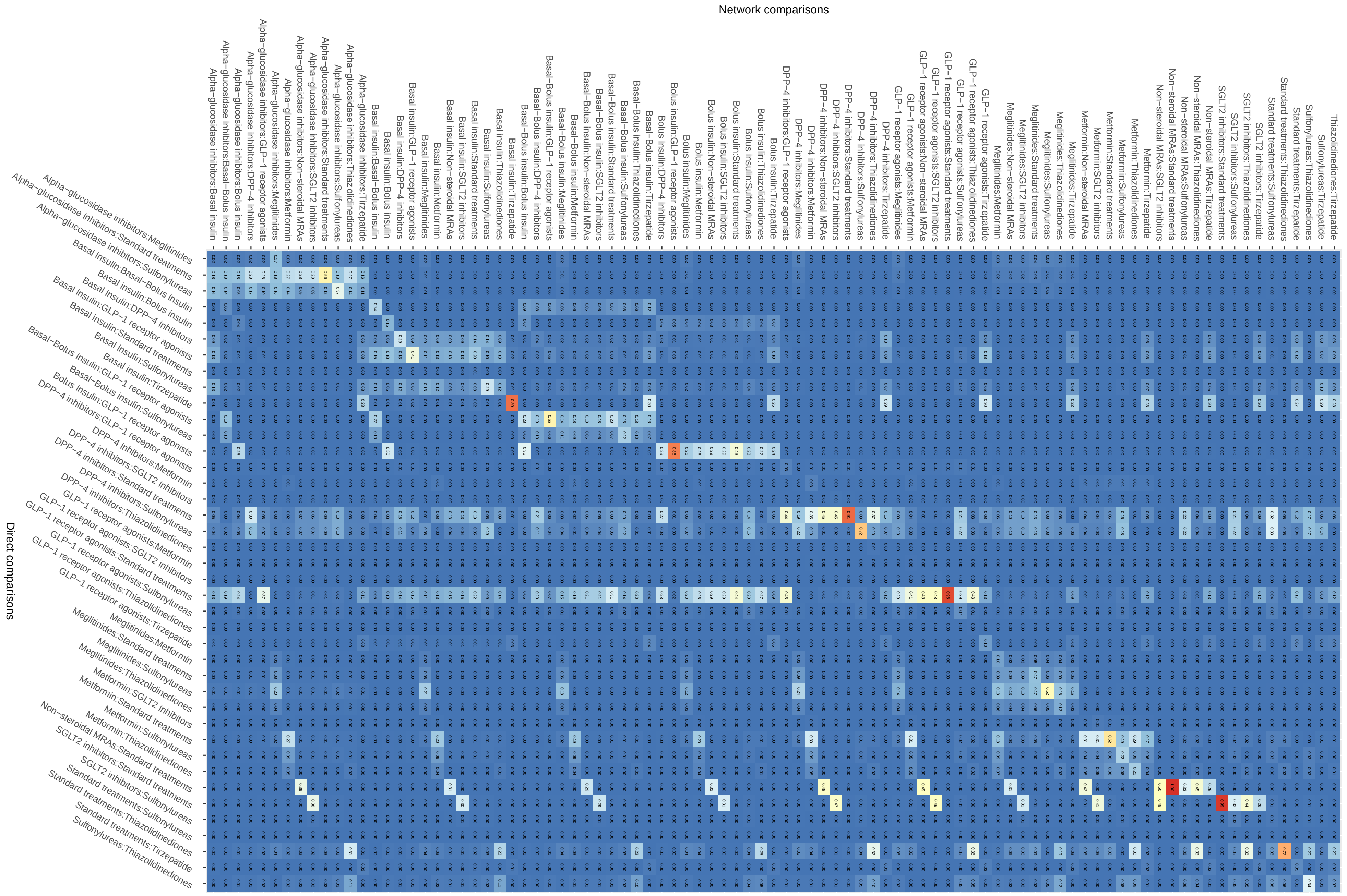

5.2.2 Cardiovascular death

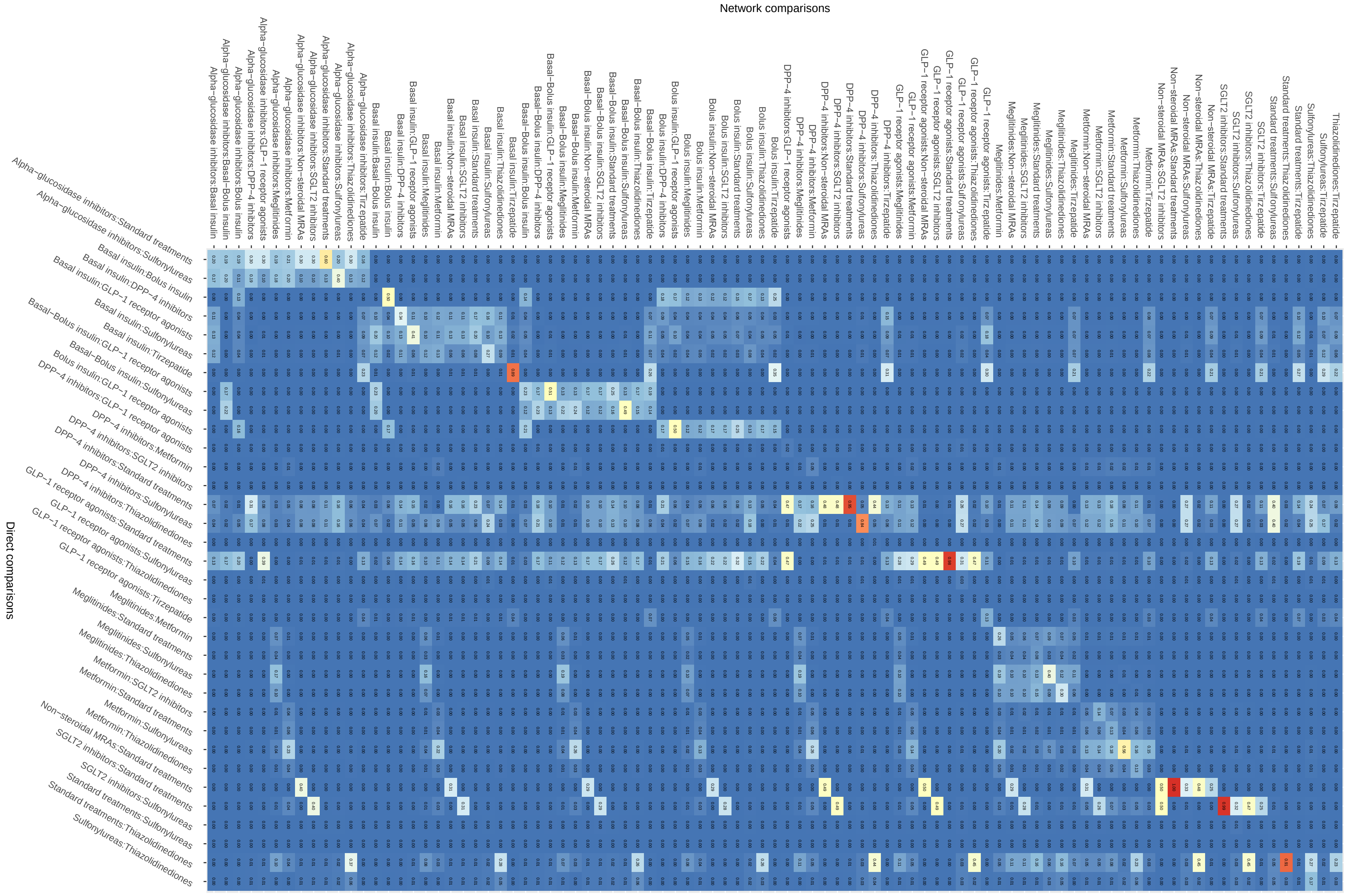

5.2.3 Non-fatal myocardial infarction

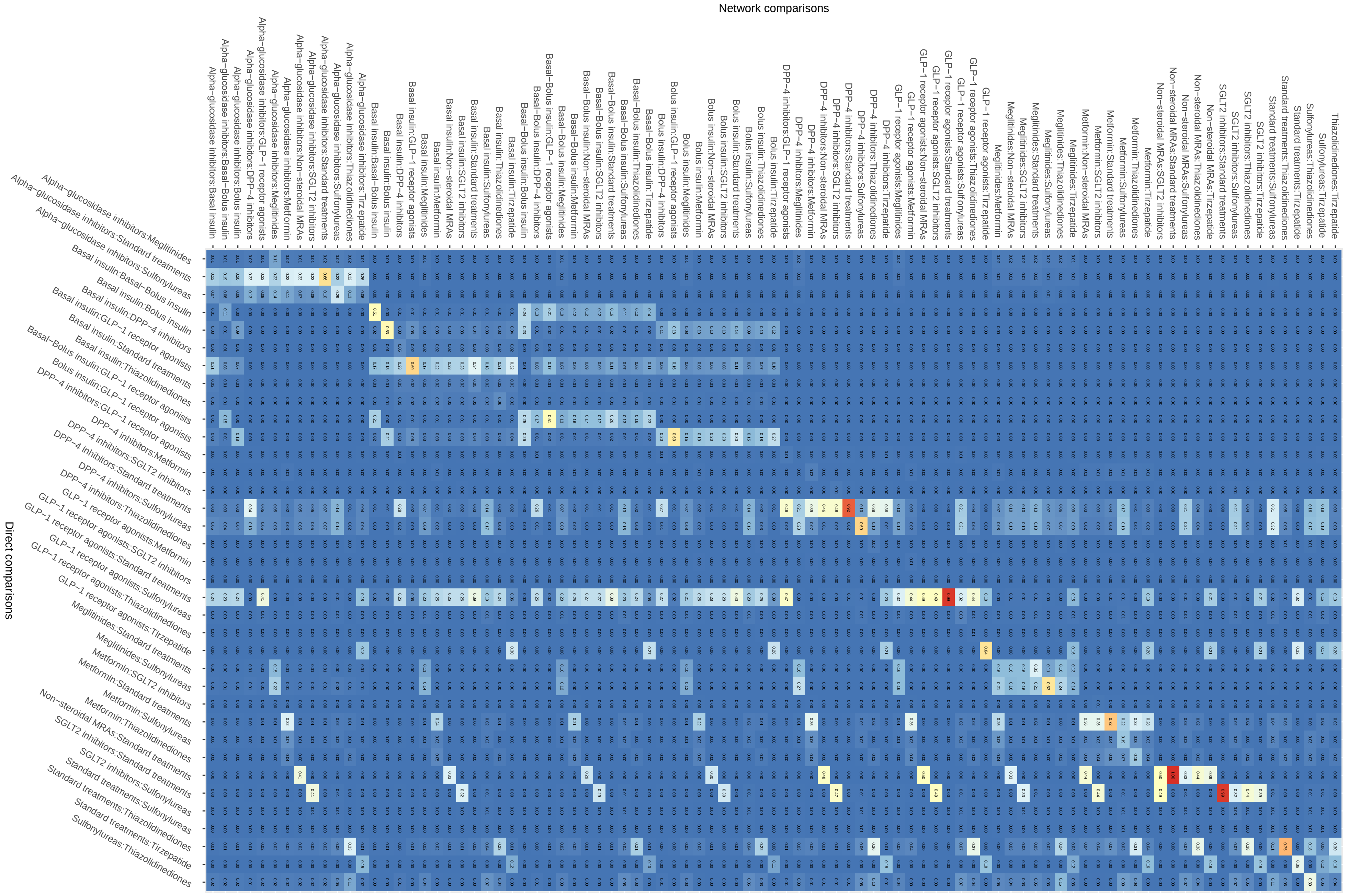

5.2.4 Non-fatal stroke

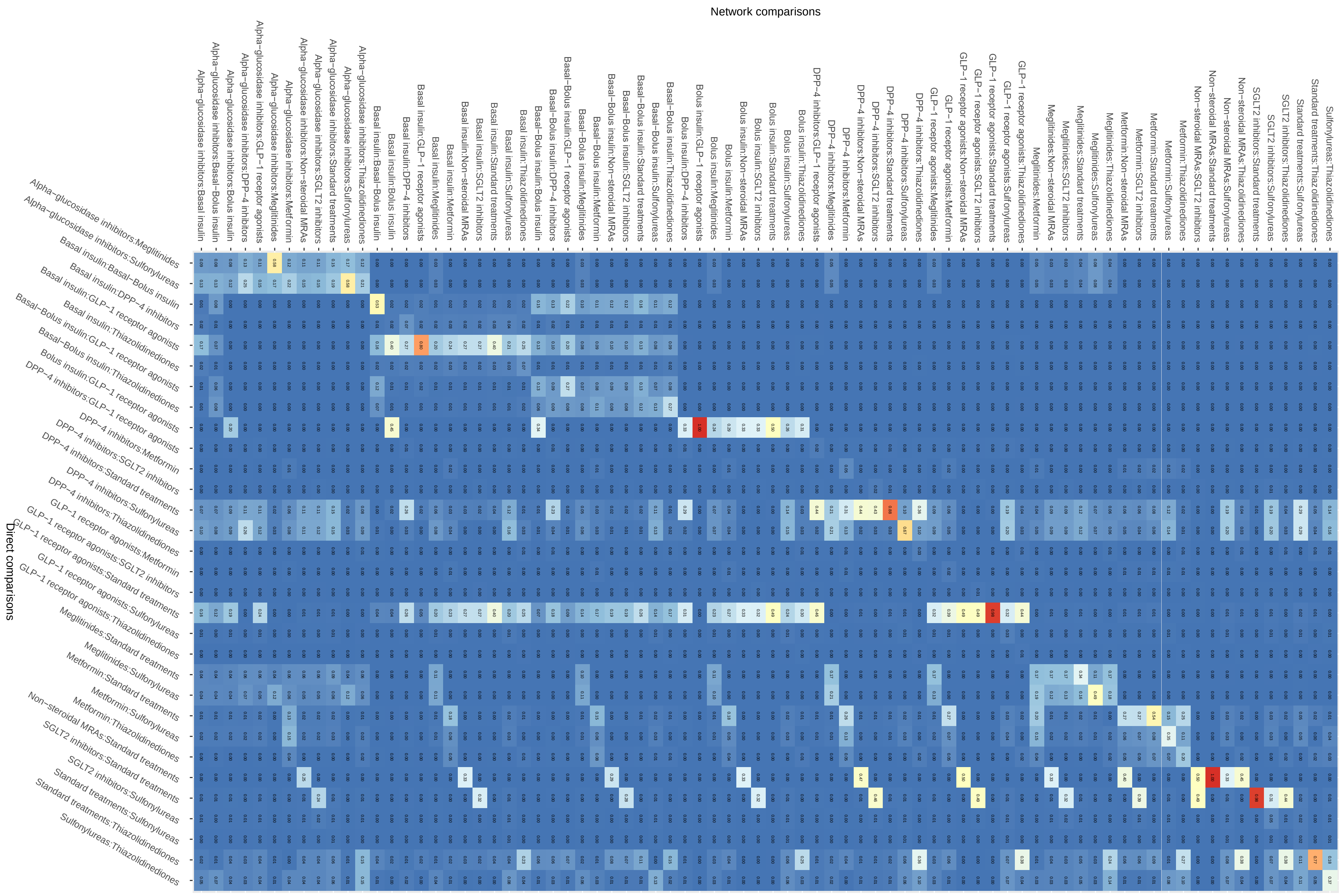

5.2.5 Hospitalization for heart failure

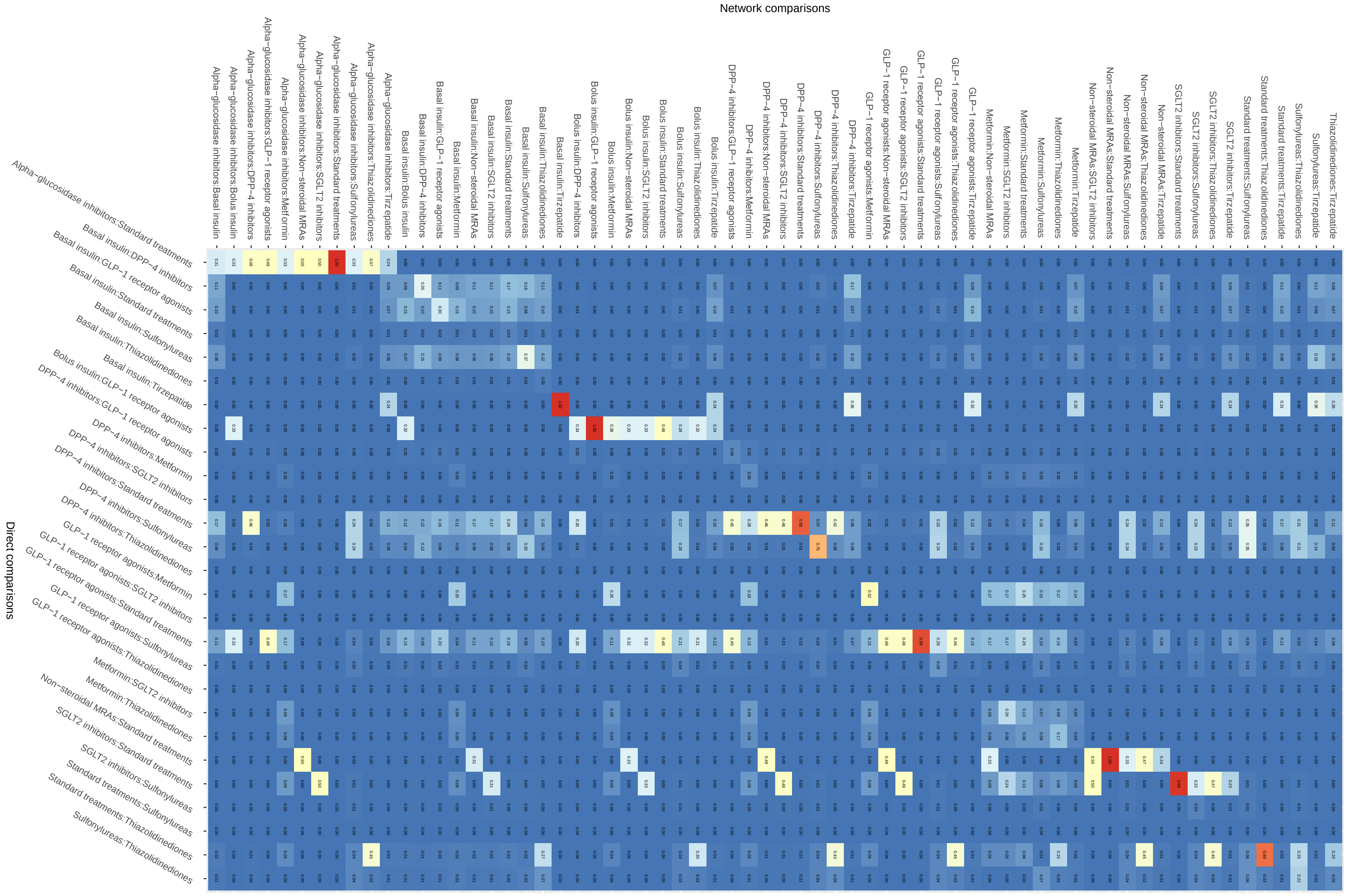

5.2.6 End-stage kidney disease

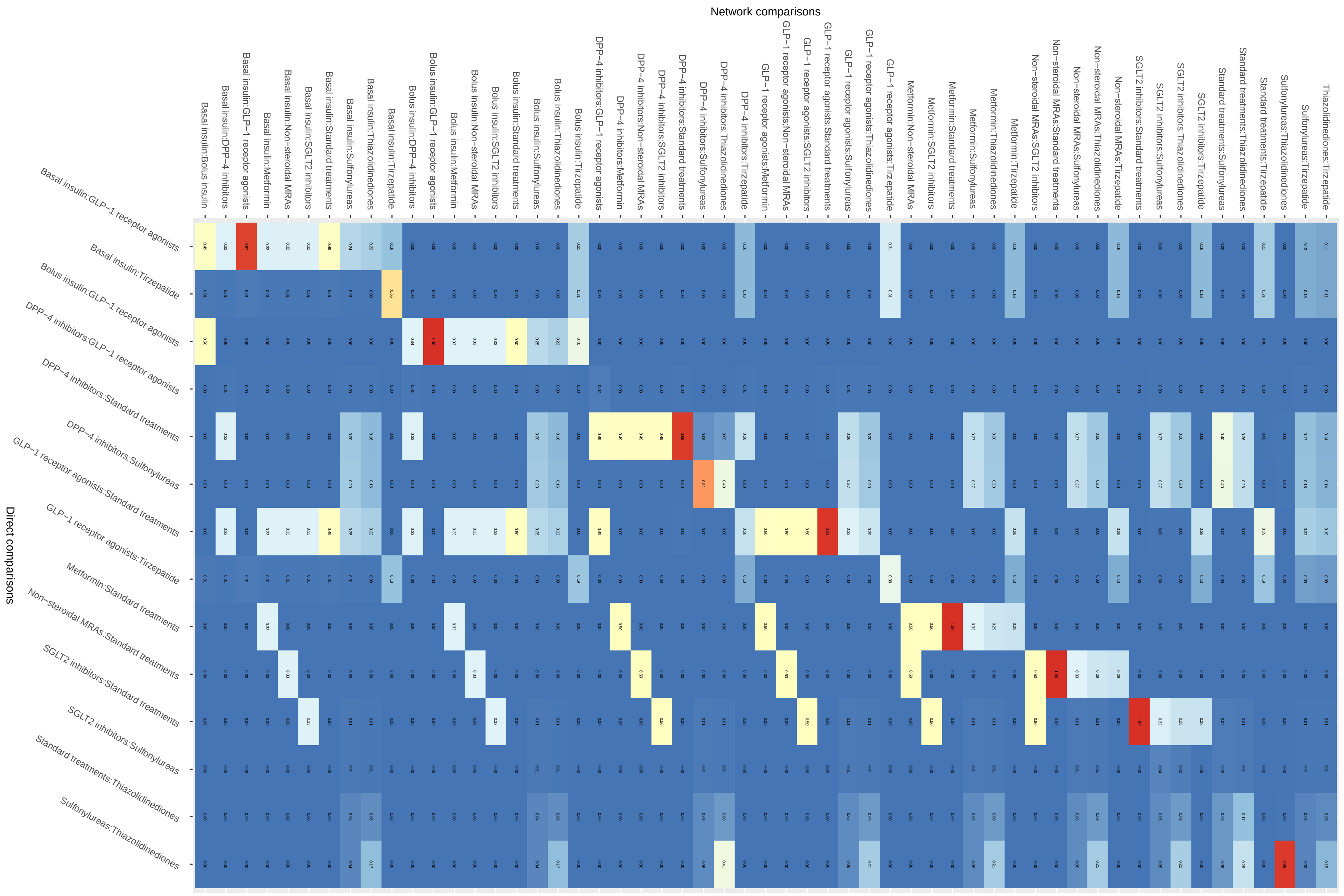

5.2.7 Severe hypoglycaemia

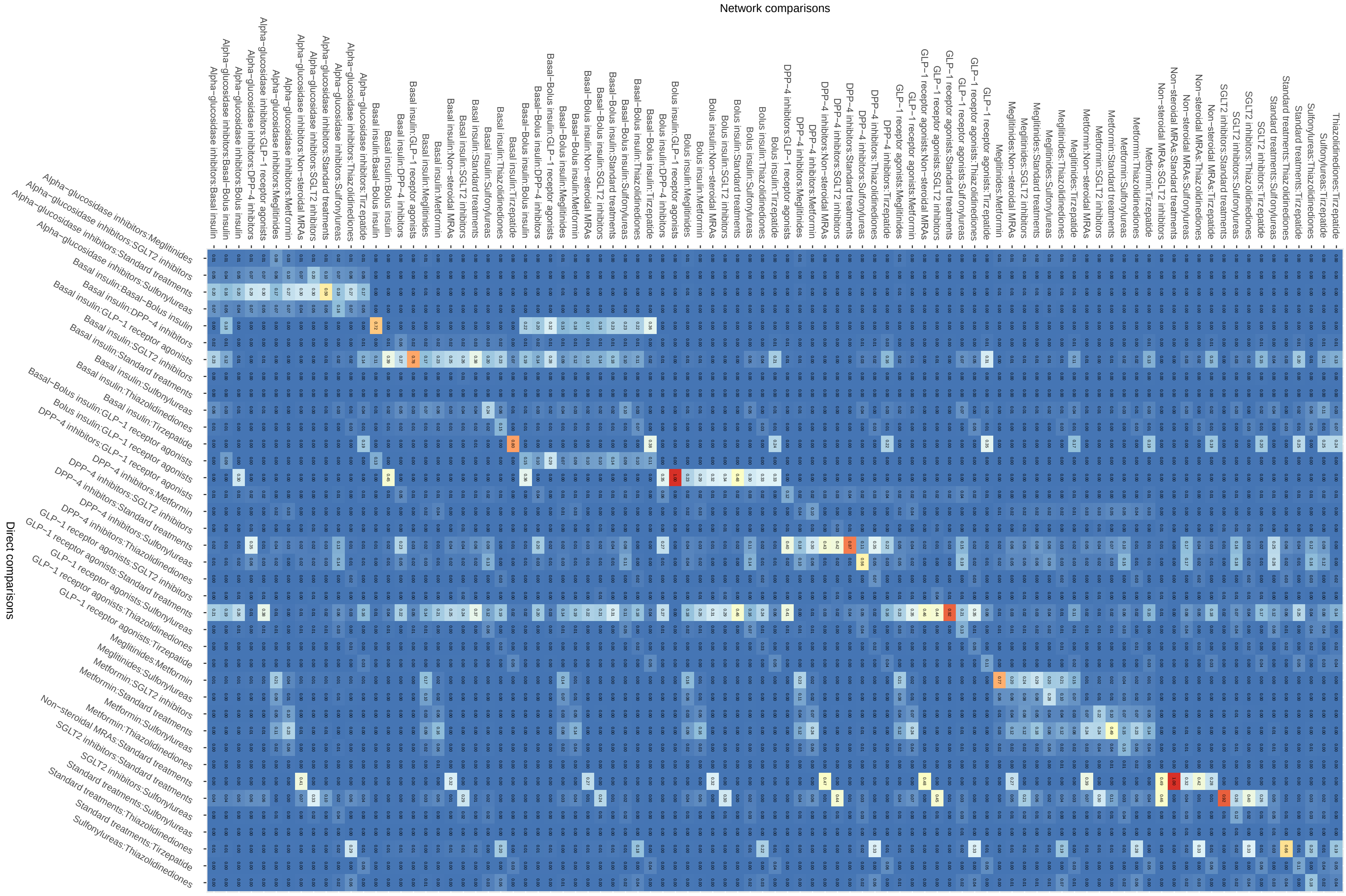

5.2.8 Severe gastrointestinal events

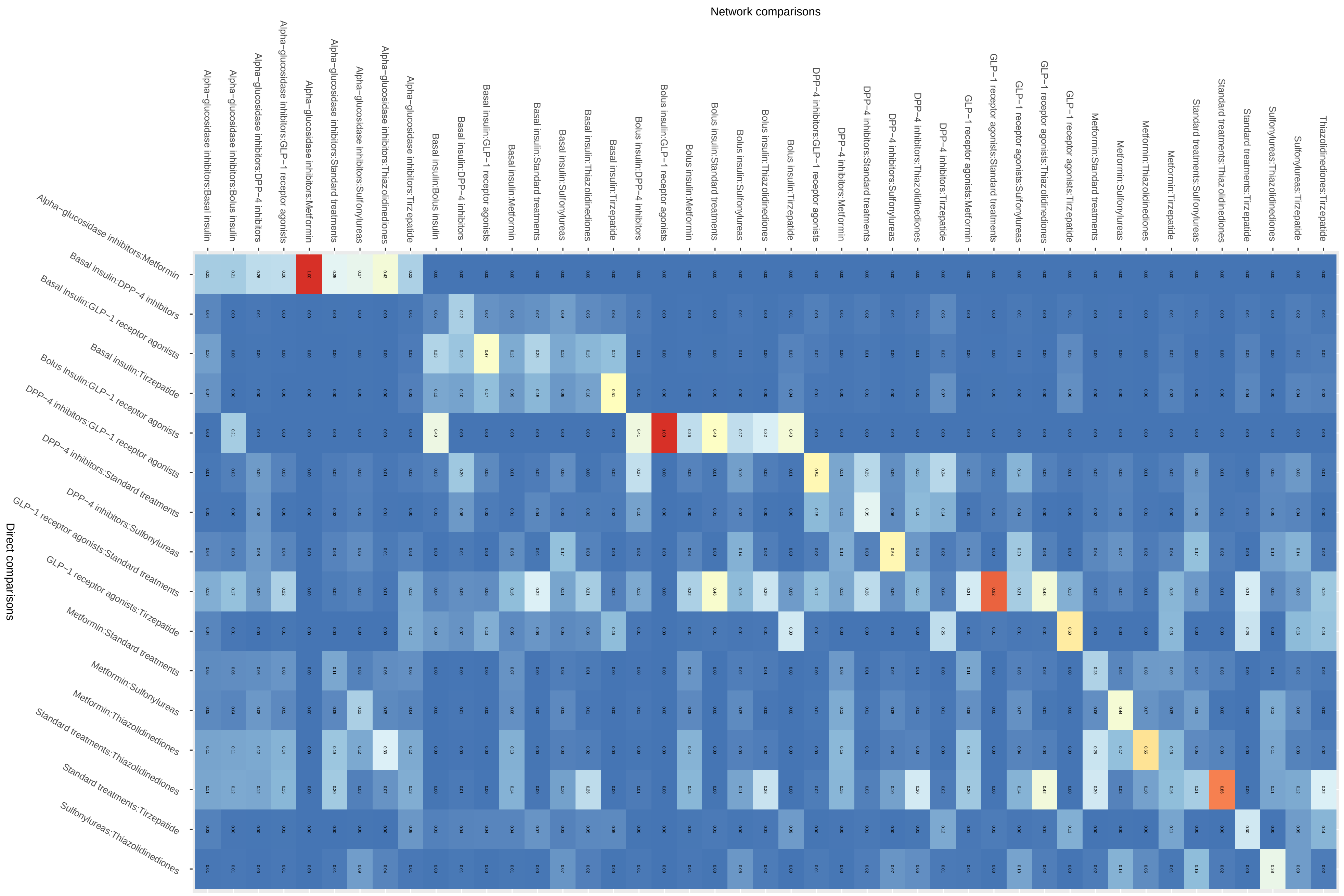

5.2.9 Genital infection

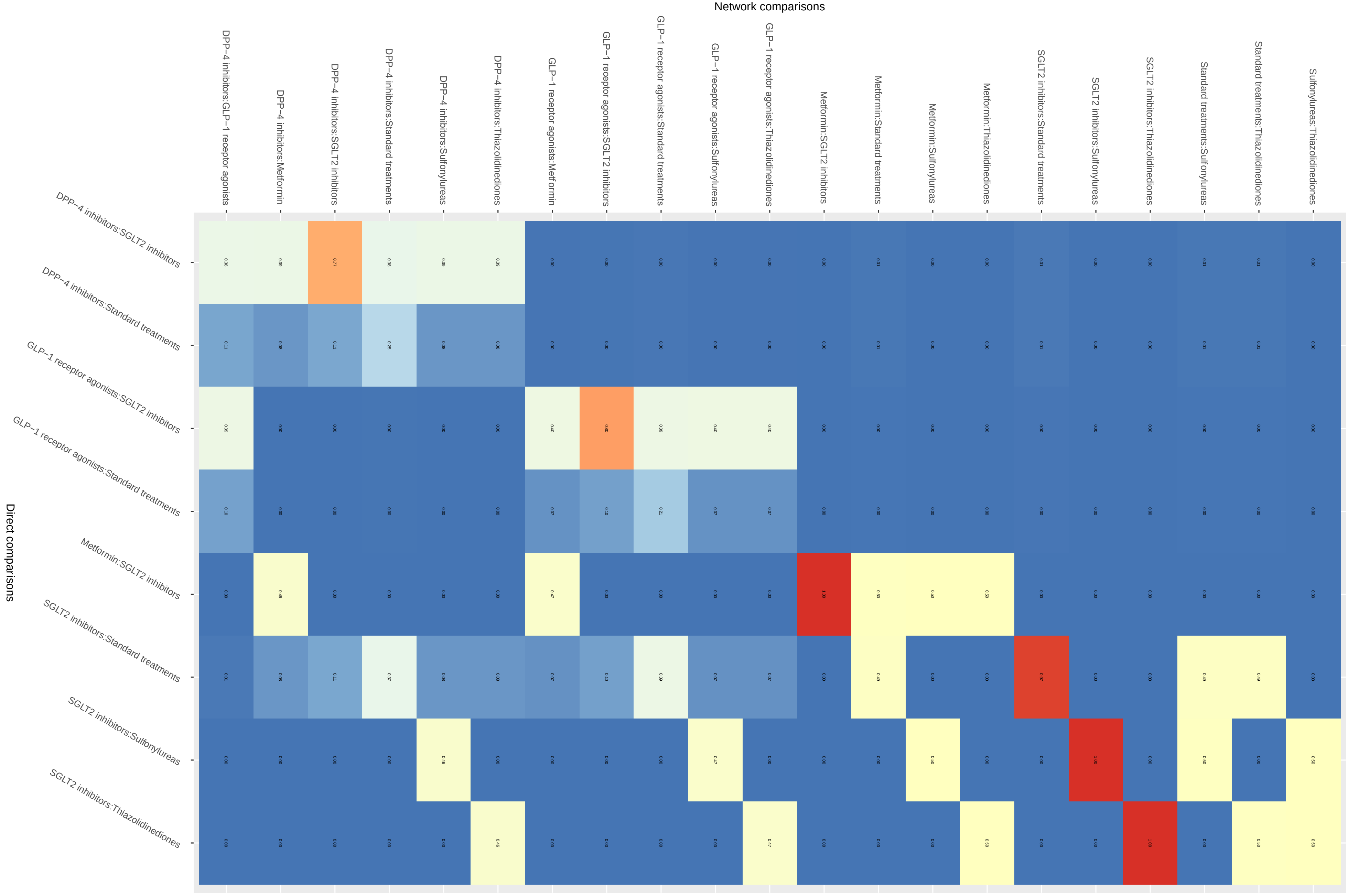

5.2.10 Amputation

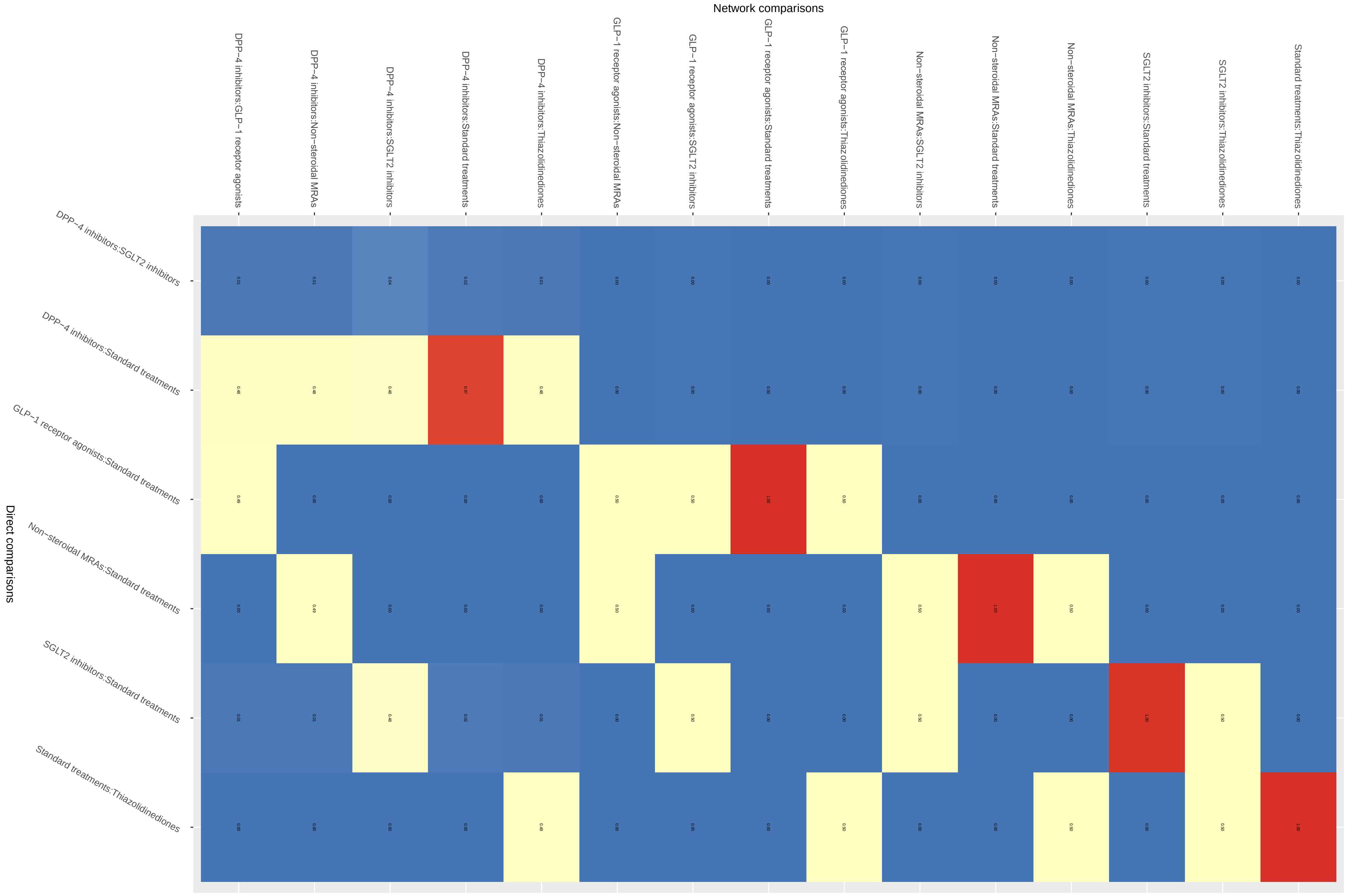

5.2.11 Ketoacidosis due to diabetes

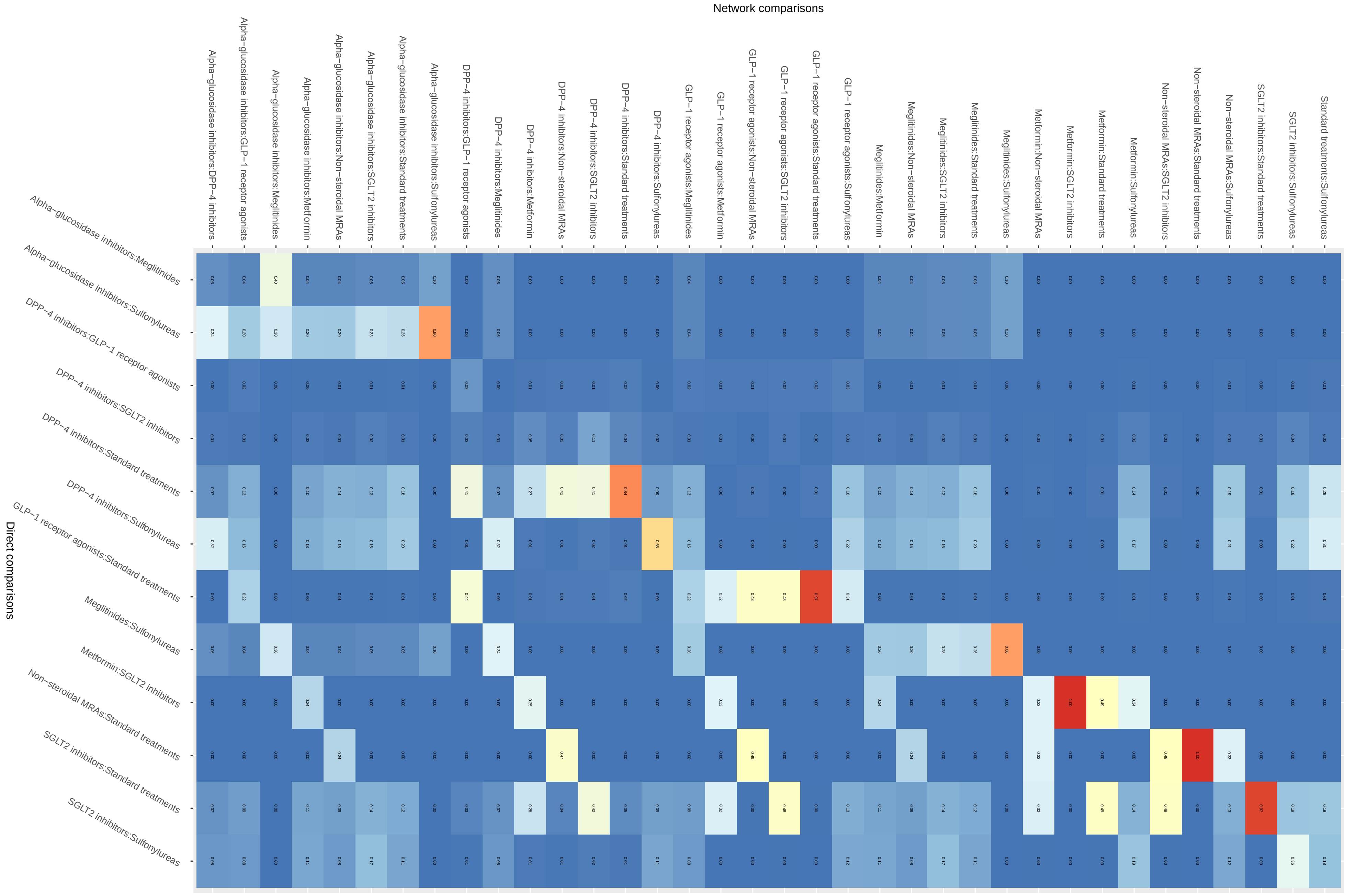

5.2.12 Health-related quality of life

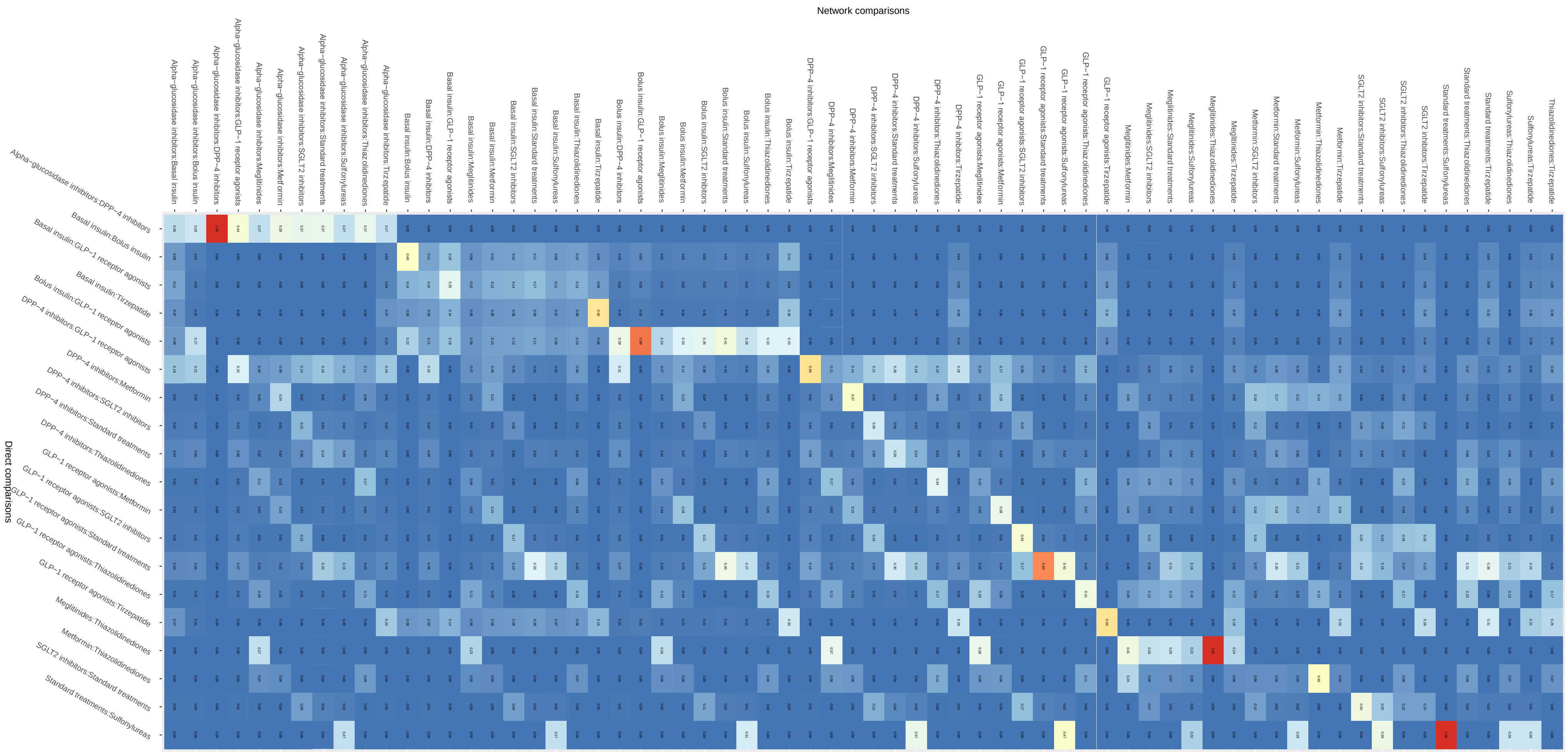

5.2.13 Bodyweight change

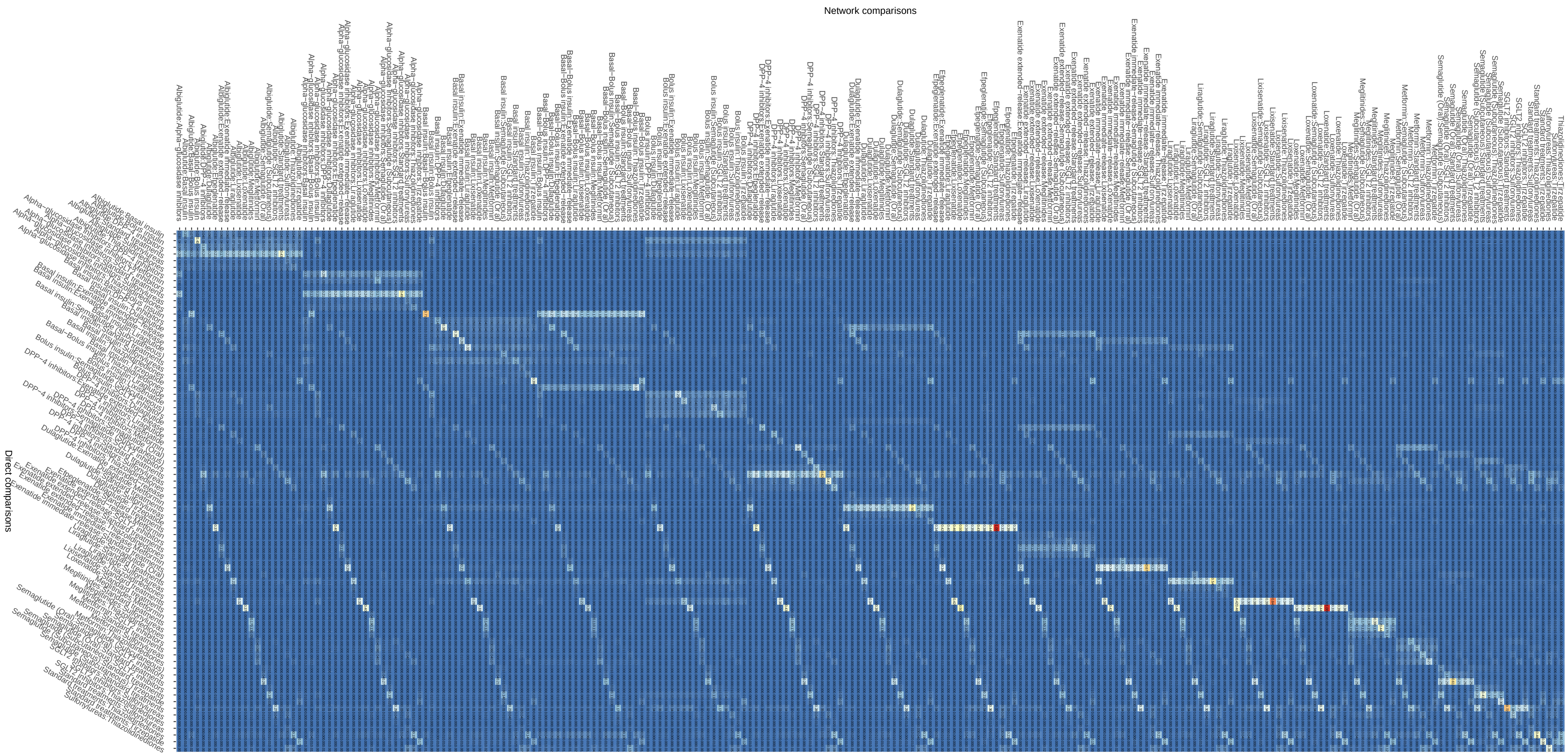

5.3 Baseline risk estimation

Notes: The study estimated the baseline risks from standard treatments arms in the trials that were followed up with at least 1 year and with at least 6000 person-months (i.e., 100 persons per 5 years) for severe hypoglycaemia, severe gastrointestinal events, amputation, ketoacidosis due to diabetes, and hyperkalaemia leading to hospitalisation. The analysis used a single-arm random-effect meta-analysis to pool the incidence rates of the control arms. The study adopted the baseline risks of other outcomes from the previous guideline [Li et al, 2021] (see details in Appendix 1.4).

5.3.1 Severe hypoglycaemia

Study	Events	Time	Rate	95%-CI
AMPLITUDE−O	13	2459.7900	0.0053	[0.0031; 0.0091]
CARMELINA	108	7667.0000	0.0141	[0.0117; 0.0170]
CREDENCE	11	5748.8167	0.0019	[0.0011; 0.0035]
DECLARE−TIMI 58	83	35989.8000	0.0023	[0.0019; 0.0029]
DUAL−VIII	156	1008.0000	0.1548	[0.1323; 0.1811]
ELIXA	24	6316.6667	0.0038	[0.0025; 0.0057]
EMPA−REG OUTCOME	36	6765.7000	0.0053	[0.0038; 0.0074]
EXAMINE	16	4018.5000	0.0040	[0.0024; 0.0065]
EXSCEL	219	23590.4000	0.0093	[0.0081; 0.0106]
FIDELIO−DKD	31	7360.6000	0.0042	[0.0030; 0.0060]
FIGARO−DKD	28	12437.2000	0.0023	[0.0016; 0.0033]
FREEDOM CVO	4	2760.0000	0.0014	[0.0005; 0.0039]
Gantz 2017	70	3902.5000	0.0179	[0.0142; 0.0227]
HARMONY OUTCOMES	55	7544.0000	0.0073	[0.0056; 0.0095]
LEADER	153	16352.0000	0.0094	[0.0080; 0.0110]
PIONEER 6	13	2109.4000	0.0062	[0.0036; 0.0106]
PROactive	11	7569.8750	0.0015	[0.0008; 0.0026]
RECORD	6	12248.5000	0.0005	[0.0002; 0.0011]
REWIND	74	26692.2000	0.0028	[0.0022; 0.0035]
SAVOR−TIMI 53	140	17245.2000	0.0081	[0.0069; 0.0096]
SUSTAIN 6	350	3298.0000	0.1061	[0.0956; 0.1178]
TECOS	143	22017.0000	0.0065	[0.0055; 0.0077]
VERTIS CV	162	8235.0000	0.0197	[0.0169; 0.0229]
Yki−Jarvinen 2013	4	630.0000	0.0063	[0.0024; 0.0169]
Common effect model			0.0078	[0.0075; 0.0082]
Random effects model			0.0060	[0.0036; 0.0100]

Heterogeneity: $I^2 = 99\%$, $\tau^2 = 1.5424$, $p = 0$

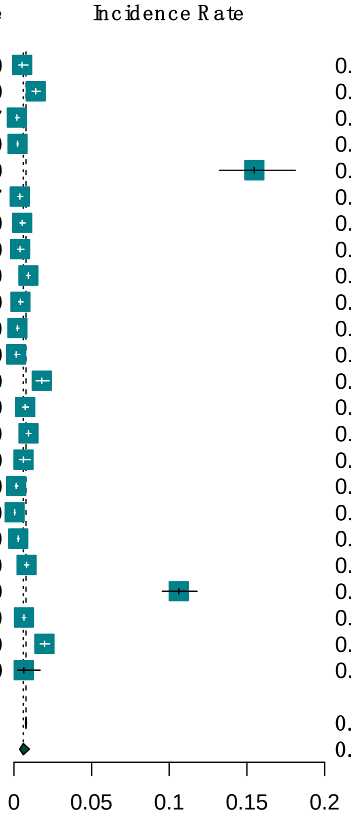

5.3.2 Severe gastrointestinal events

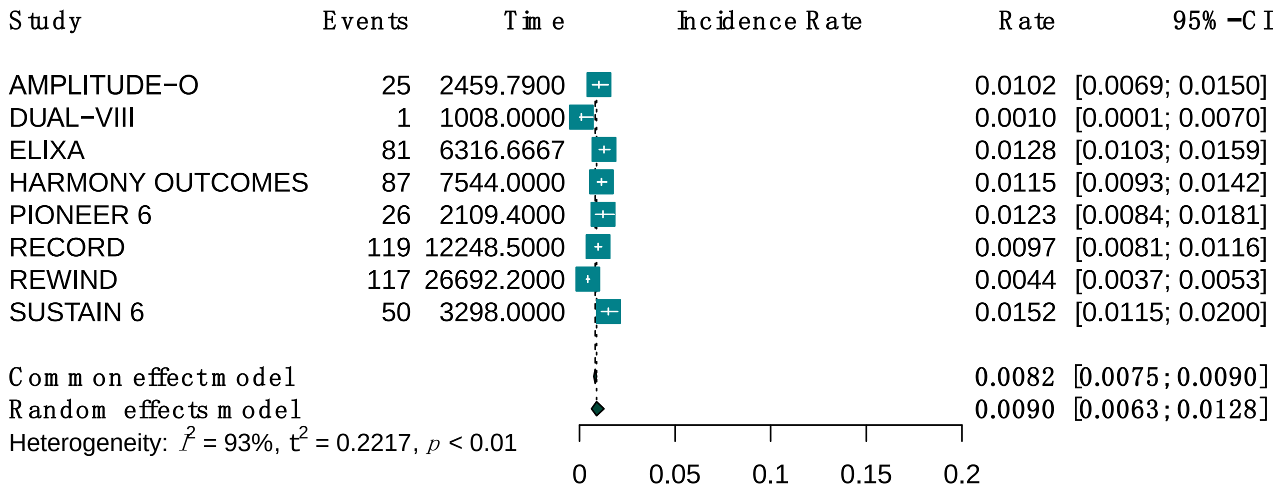

5.3.3 Amputation

Study	Events	Time	Rate	95%−CI
AMPLITUDE−O	0	2452.5500	0.0002	[0.0000; 0.0033]
CANVAS/CANVAS−R	47	15566.0000	0.0030	[0.0023; 0.0040]
CREDENCE	63	5748.8167	0.0110	[0.0086; 0.0140]
DECLARE−TIMI 58	113	35989.8000	0.0031	[0.0026; 0.0038]
ELIXA	1	6316.6667	0.0002	[0.0000; 0.0011]
EMPA−REG OUTCOME	43	6765.7000	0.0064	[0.0047; 0.0086]
FIDELIO−DKD	8	7360.6000	0.0011	[0.0005; 0.0022]
FIGARO−DKD	8	12437.2000	0.0006	[0.0003; 0.0013]
PROactive	26	7569.8750	0.0034	[0.0023; 0.0050]
RECORD	15	12248.5000	0.0012	[0.0007; 0.0020]
TECOS	66	22017.0000	0.0030	[0.0024; 0.0038]
VERTIS CV	45	8235.0000	0.0055	[0.0041; 0.0073]
Common effect model			0.0030	[0.0028; 0.0033]
Random effects model			0.0020	[0.0010; 0.0039]

Heterogeneity: $I^2 = 93\%$, $\tau^2 = 1.2001$, $p < 0.01$

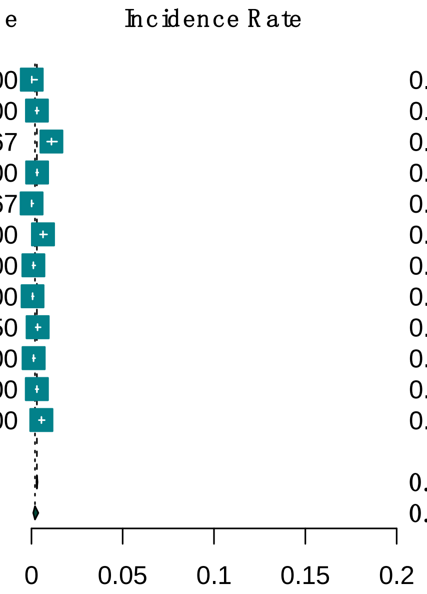

5.3.4 Ketoacidosis due to diabetes

Study	Events	Time	Rate	95%-CI
AMPLITUDE−O	3	2452.5500	0.0012	[0.0004; 0.0038]
CARMELINA	4	7667.0000	0.0005	[0.0002; 0.0014]
CREDENCE	1	5748.8167	0.0002	[0.0000; 0.0012]
DECLARE−TIMI 58	12	36027.6000	0.0003	[0.0002; 0.0006]
ELIXA	4	6316.6667	0.0006	[0.0002; 0.0017]
EMPA−REG OUTCOME	1	6765.7000	0.0001	[0.0000; 0.0010]
EXAMINE	1	4018.5000	0.0002	[0.0000; 0.0018]
FIDELIO−DKD	7	7360.6000	0.0010	[0.0005; 0.0020]
FIGARO−DKD	8	12437.2000	0.0006	[0.0003; 0.0013]
Gantz 2017	1	3902.5000	0.0003	[0.0000; 0.0018]
HARMONY OUTCOMES	6	7544.0000	0.0008	[0.0004; 0.0018]
LEADER	7	16352.0000	0.0004	[0.0002; 0.0009]
SAVOR−TIMI 53	8	17245.2000	0.0005	[0.0002; 0.0009]
VERTIS CV	2	8235.0000	0.0002	[0.0001; 0.0010]
Common effect model			0.0005	[0.0004; 0.0006]
Random effects model			0.0005	[0.0004; 0.0006]

Heterogeneity: $I^2 = 8\%$, $\tau^2 = 0$, $p = 0.37$

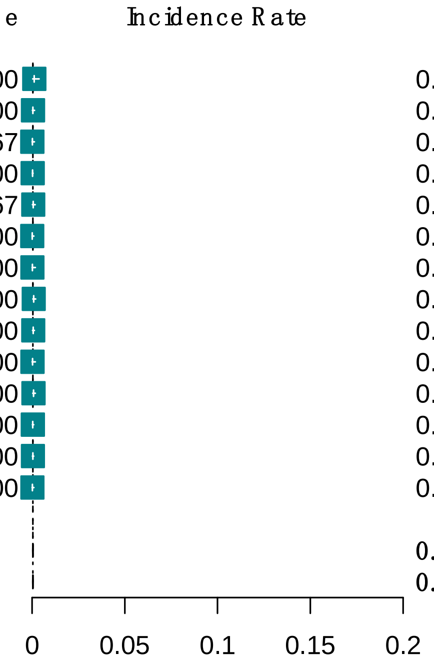

5.3.5 Hyperkalaemia leading to hospitalization

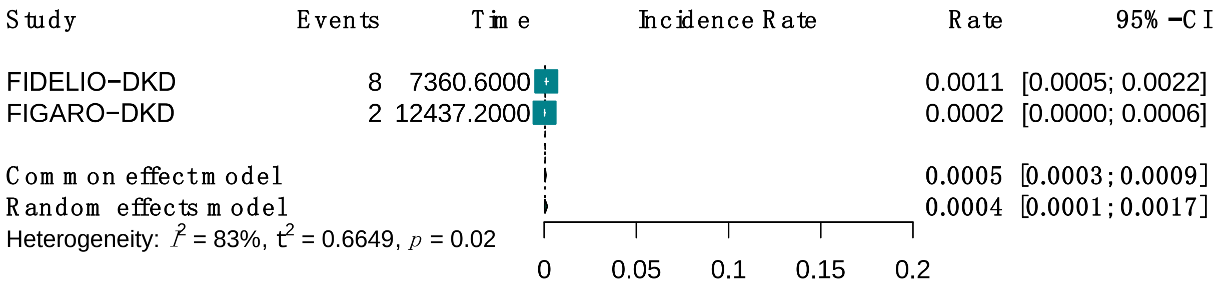

Appendix 6 Anticipated absolute effects

Notes: This study anticipated the absolute effects by applying the relative effects to the baseline risks in five tiers of cardiovascular and kidney diseases, which are adults with type 2 diabetes and 1) three or fewer cardiovascular risk factors, 2) more than three cardiovascular risk factors, 3) established cardiovascular disease but not chronic kidney disease, 4) established chronic kidney disease but not cardiovascular disease, 5) established cardiovascular disease and chronic kidney disease. We adopted the baseline risks from previous guideline (see details in Appendix 1.4). An online tool facilitates the quick inquire for the absolute effects (https://qingys.shinyapps.io/data_visualization).

6.1 In patients with 3 or fewer cardiovascular risk factors

Outcome	Intervention	Comparator	Odds ratio	Baseline risk	Risk if treated	Risk difference	Certainty
**All-cause death**	Alpha-glucosidase inhibitors	Basal insulin	0.81 (0.26 to 2.47)	22 per 1000 persons	18 per 1000 persons (6 to 53 per 1000 persons)	4 fewer (16 fewer to 31 more)	Very low
**All-cause death**	Alpha-glucosidase inhibitors	Basal-Bolus insulin	1.12 (0.19 to 6.74)	16 per 1000 persons	18 per 1000 persons (3 to 99 per 1000 persons)	2 more (13 fewer to 83 more)	Very low
**All-cause death**	Alpha-glucosidase inhibitors	Bolus insulin	1.85 (0.37 to 9.23)	10 per 1000 persons	18 per 1000 persons (4 to 85 per 1000 persons)	8 more (6 fewer to 75 more)	Very low
**All-cause death**	Alpha-glucosidase inhibitors	DPP-4 inhibitors	0.88 (0.30 to 2.58)	20 per 1000 persons	18 per 1000 persons (6 to 50 per 1000 persons)	2 fewer (14 fewer to 30 more)	Very low
**All-cause death**	Alpha-glucosidase inhibitors	GLP-1 receptor agonists	1.01 (0.34 to 2.98)	18 per 1000 persons	18 per 1000 persons (6 to 52 per 1000 persons)	0 (12 fewer to 34 more)	Very low
**All-cause death**	Alpha-glucosidase inhibitors	Meglitinides	0.56 (0.13 to 2.48)	31 per 1000 persons	18 per 1000 persons (4 to 74 per 1000 persons)	13 fewer (27 fewer to 43 more)	Very low
**All-cause death**	Alpha-glucosidase inhibitors	Metformin	1.06 (0.36 to 3.18)	17 per 1000 persons	18 per 1000 persons (6 to 52 per 1000 persons)	1 more (11 fewer to 35 more)	Very low
**All-cause death**	Alpha-glucosidase inhibitors	Non-steroidal MRAs	1.00 (0.34 to 2.95)	18 per 1000 persons	18 per 1000 persons (6 to 51 per 1000 persons)	0 (12 fewer to 33 more)	Very low
**All-cause death**	Alpha-glucosidase inhibitors	SGLT2 inhibitors	1.01 (0.34 to 2.96)	18 per 1000 persons	18 per 1000 persons (6 to 51 per 1000 persons)	0 (12 fewer to 33 more)	Very low

Outcome	Intervention	Comparator	Odds ratio	Baseline risk	Risk if treated	Risk difference	Certainty
**All-cause death**	Alpha-glucosidase inhibitors	Standard treatments	0.89 (0.30 to 2.61)	20 per 1000 persons	18 per 1000 persons (6 to 51 per 1000 persons)	2 fewer (14 fewer to 31 more)	Very low
**All-cause death**	Alpha-glucosidase inhibitors	Sulfonylureas	0.81 (0.27 to 2.37)	22 per 1000 persons	18 per 1000 persons (6 to 51 per 1000 persons)	4 fewer (16 fewer to 29 more)	Very low
**All-cause death**	Alpha-glucosidase inhibitors	Thiazolidinediones	0.93 (0.32 to 2.76)	19 per 1000 persons	18 per 1000 persons (6 to 51 per 1000 persons)	1 fewer (13 fewer to 32 more)	Very low
**All-cause death**	Alpha-glucosidase inhibitors	Tirzepatide	1.07 (0.32 to 3.57)	17 per 1000 persons	18 per 1000 persons (6 to 58 per 1000 persons)	1 more (11 fewer to 41 more)	Very low
**All-cause death**	Basal insulin	Basal-Bolus insulin	1.39 (0.33 to 5.92)	16 per 1000 persons	22 per 1000 persons (5 to 88 per 1000 persons)	6 more (11 fewer to 72 more)	Very low
**All-cause death**	Basal insulin	Bolus insulin	2.28 (0.67 to 7.75)	10 per 1000 persons	23 per 1000 persons (7 to 73 per 1000 persons)	13 more (3 fewer to 63 more)	Very low
**All-cause death**	Basal insulin	DPP-4 inhibitors	1.08 (0.80 to 1.47)	20 per 1000 persons	22 per 1000 persons (16 to 29 per 1000 persons)	2 more (4 fewer to 9 more)	Moderate
**All-cause death**	Basal insulin	GLP-1 receptor agonists	1.25 (0.93 to 1.70)	18 per 1000 persons	22 per 1000 persons (17 to 30 per 1000 persons)	4 more (1 fewer to 12 more)	Low
**All-cause death**	Basal insulin	Meglitinides	0.69 (0.22 to 2.24)	31 per 1000 persons	22 per 1000 persons (7 to 67 per 1000 persons)	9 fewer (24 fewer to 36 more)	Very low
**All-cause death**	Basal insulin	Metformin	1.31 (0.91 to 1.90)	17 per 1000 persons	22 per 1000 persons (15 to 32 per 1000 persons)	5 more (2 fewer to 15 more)	Low
**All-cause death**	Basal insulin	Non-steroidal MRAs	1.24 (0.89 to 1.71)	18 per 1000 persons	22 per 1000 persons (16 to 30 per 1000 persons)	4 more (2 fewer to 12 more)	Low
**All-cause death**	Basal insulin	SGLT2 inhibitors	1.24 (0.91 to 1.70)	18 per 1000 persons	22 per 1000 persons (16 to 30 per 1000 persons)	4 more (2 fewer to 12 more)	Low
**All-cause death**	Basal insulin	Standard treatments	1.10 (0.81 to 1.49)	20 per 1000 persons	22 per 1000 persons (16 to 30 per 1000 persons)	2 more (4 fewer to 10 more)	Moderate
**All-cause death**	Basal insulin	Sulfonylureas	1.00 (0.73 to 1.36)	22 per 1000 persons	22 per 1000 persons (16 to 30 per 1000 persons)	0 (6 fewer to 8 more)	High
**All-cause death**	Basal insulin	Thiazolidinediones	1.15 (0.83 to 1.60)	19 per 1000 persons	22 per 1000 persons (16 to 30 per 1000 persons)	3 more (3 fewer to 11 more)	Low

Outcome	Intervention	Comparator	Odds ratio	Baseline risk	Risk if treated	Risk difference	Certainty
**All-cause death**	Basal insulin	Tirzepatide	1.32 (0.82 to 2.13)	17 per 1000 persons	22 per 1000 persons (14 to 36 per 1000 persons)	5 more (3 fewer to 19 more)	Moderate
**All-cause death**	Basal-Bolus insulin	Bolus insulin	1.64 (0.25 to 10.61)	10 per 1000 persons	16 per 1000 persons (3 to 97 per 1000 persons)	6 more (7 fewer to 87 more)	Very low
**All-cause death**	Basal-Bolus insulin	DPP-4 inhibitors	0.78 (0.19 to 3.28)	20 per 1000 persons	16 per 1000 persons (4 to 63 per 1000 persons)	4 fewer (16 fewer to 43 more)	Very low
**All-cause death**	Basal-Bolus insulin	GLP-1 receptor agonists	0.90 (0.22 to 3.78)	18 per 1000 persons	16 per 1000 persons (4 to 65 per 1000 persons)	2 fewer (14 fewer to 47 more)	Very low
**All-cause death**	Basal-Bolus insulin	Meglitinides	0.50 (0.08 to 3.11)	31 per 1000 persons	16 per 1000 persons (3 to 90 per 1000 persons)	15 fewer (28 fewer to 59 more)	Very low
**All-cause death**	Basal-Bolus insulin	Metformin	0.95 (0.22 to 4.04)	17 per 1000 persons	16 per 1000 persons (4 to 65 per 1000 persons)	1 fewer (13 fewer to 48 more)	Very low
**All-cause death**	Basal-Bolus insulin	Non-steroidal MRAs	0.89 (0.21 to 3.75)	18 per 1000 persons	16 per 1000 persons (4 to 64 per 1000 persons)	2 fewer (14 fewer to 46 more)	Very low
**All-cause death**	Basal-Bolus insulin	SGLT2 inhibitors	0.90 (0.21 to 3.76)	18 per 1000 persons	16 per 1000 persons (4 to 64 per 1000 persons)	2 fewer (14 fewer to 46 more)	Very low
**All-cause death**	Basal-Bolus insulin	Standard treatments	0.79 (0.19 to 3.32)	20 per 1000 persons	16 per 1000 persons (4 to 63 per 1000 persons)	4 fewer (16 fewer to 43 more)	Very low
**All-cause death**	Basal-Bolus insulin	Sulfonylureas	0.72 (0.17 to 3.02)	22 per 1000 persons	16 per 1000 persons (4 to 64 per 1000 persons)	6 fewer (18 fewer to 42 more)	Very low
**All-cause death**	Basal-Bolus insulin	Thiazolidinediones	0.83 (0.20 to 3.51)	19 per 1000 persons	16 per 1000 persons (4 to 64 per 1000 persons)	3 fewer (15 fewer to 45 more)	Very low
**All-cause death**	Basal-Bolus insulin	Tirzepatide	0.95 (0.21 to 4.35)	17 per 1000 persons	16 per 1000 persons (4 to 70 per 1000 persons)	1 fewer (13 fewer to 53 more)	Very low
**All-cause death**	Bolus insulin	DPP-4 inhibitors	0.47 (0.14 to 1.57)	20 per 1000 persons	10 per 1000 persons (3 to 31 per 1000 persons)	10 fewer (17 fewer to 11 more)	Very low
**All-cause death**	Bolus insulin	GLP-1 receptor agonists	0.55 (0.17 to 1.81)	18 per 1000 persons	10 per 1000 persons (3 to 32 per 1000 persons)	8 fewer (15 fewer to 14 more)	Very low
**All-cause death**	Bolus insulin	Meglitinides	0.30 (0.06 to 1.58)	31 per 1000 persons	10 per 1000 persons (2 to 48 per 1000 persons)	21 fewer (29 fewer to 17 more)	Very low

Outcome	Intervention	Comparator	Odds ratio	Baseline risk	Risk if treated	Risk difference	Certainty
**All-cause death**	Bolus insulin	Metformin	0.58 (0.17 to 1.94)	17 per 1000 persons	10 per 1000 persons (3 to 32 per 1000 persons)	7 fewer (14 fewer to 15 more)	Very low
**All-cause death**	Bolus insulin	Non-steroidal MRAs	0.54 (0.16 to 1.80)	18 per 1000 persons	10 per 1000 persons (3 to 32 per 1000 persons)	8 fewer (15 fewer to 14 more)	Very low
**All-cause death**	Bolus insulin	SGLT2 inhibitors	0.54 (0.16 to 1.81)	18 per 1000 persons	10 per 1000 persons (3 to 32 per 1000 persons)	8 fewer (15 fewer to 14 more)	Very low
**All-cause death**	Bolus insulin	Standard treatments	0.48 (0.15 to 1.59)	20 per 1000 persons	10 per 1000 persons (3 to 31 per 1000 persons)	10 fewer (17 fewer to 11 more)	Very low
**All-cause death**	Bolus insulin	Sulfonylureas	0.44 (0.13 to 1.45)	22 per 1000 persons	10 per 1000 persons (3 to 32 per 1000 persons)	12 fewer (19 fewer to 10 more)	Very low
**All-cause death**	Bolus insulin	Thiazolidinediones	0.51 (0.15 to 1.68)	19 per 1000 persons	10 per 1000 persons (3 to 32 per 1000 persons)	9 fewer (16 fewer to 13 more)	Very low
**All-cause death**	Bolus insulin	Tirzepatide	0.58 (0.16 to 2.13)	17 per 1000 persons	10 per 1000 persons (3 to 36 per 1000 persons)	7 fewer (14 fewer to 19 more)	Very low
**All-cause death**	DPP-4 inhibitors	GLP-1 receptor agonists	1.16 (1.06 to 1.26)	18 per 1000 persons	21 per 1000 persons (19 to 23 per 1000 persons)	3 more (1 more to 5 more)	Moderate
**All-cause death**	DPP-4 inhibitors	Meglitinides	0.64 (0.21 to 1.99)	31 per 1000 persons	20 per 1000 persons (7 to 60 per 1000 persons)	11 fewer (24 fewer to 29 more)	Very low
**All-cause death**	DPP-4 inhibitors	Metformin	1.21 (0.97 to 1.52)	17 per 1000 persons	20 per 1000 persons (16 to 26 per 1000 persons)	3 more (1 fewer to 9 more)	Moderate
**All-cause death**	DPP-4 inhibitors	Non-steroidal MRAs	1.14 (0.99 to 1.31)	18 per 1000 persons	20 per 1000 persons (18 to 23 per 1000 persons)	2 more (0 to 5 more)	Moderate
**All-cause death**	DPP-4 inhibitors	SGLT2 inhibitors	1.15 (1.04 to 1.26)	18 per 1000 persons	21 per 1000 persons (19 to 23 per 1000 persons)	3 more (1 more to 5 more)	Moderate
**All-cause death**	DPP-4 inhibitors	Standard treatments	1.01 (0.95 to 1.08)	20 per 1000 persons	20 per 1000 persons (19 to 22 per 1000 persons)	0 (1 fewer to 2 more)	Moderate
**All-cause death**	DPP-4 inhibitors	Sulfonylureas	0.92 (0.81 to 1.04)	22 per 1000 persons	20 per 1000 persons (18 to 23 per 1000 persons)	2 fewer (4 fewer to 1 more)	Moderate
**All-cause death**	DPP-4 inhibitors	Thiazolidinediones	1.06 (0.92 to 1.23)	19 per 1000 persons	20 per 1000 persons (18 to 23 per 1000 persons)	1 more (1 fewer to 4 more)	Moderate

Outcome	Intervention	Comparator	Odds ratio	Baseline risk	Risk if treated	Risk difference	Certainty
**All-cause death**	DPP-4 inhibitors	Tirzepatide	1.22 (0.70 to 2.11)	17 per 1000 persons	21 per 1000 persons (12 to 35 per 1000 persons)	4 more (5 fewer to 18 more)	Low
**All-cause death**	GLP-1 receptor agonists	Meglitinides	0.55 (0.18 to 1.73)	31 per 1000 persons	17 per 1000 persons (6 to 52 per 1000 persons)	14 fewer (25 fewer to 21 more)	Very low
**All-cause death**	GLP-1 receptor agonists	Metformin	1.05 (0.84 to 1.31)	17 per 1000 persons	18 per 1000 persons (14 to 22 per 1000 persons)	1 more (3 fewer to 5 more)	Moderate
**All-cause death**	GLP-1 receptor agonists	Non-steroidal MRAs	0.99 (0.86 to 1.13)	18 per 1000 persons	18 per 1000 persons (16 to 20 per 1000 persons)	0 (2 fewer to 2 more)	Moderate
**All-cause death**	GLP-1 receptor agonists	SGLT2 inhibitors	0.99 (0.91 to 1.08)	18 per 1000 persons	18 per 1000 persons (16 to 19 per 1000 persons)	0 (2 fewer to 1 more)	Moderate
**All-cause death**	GLP-1 receptor agonists	Standard treatments	0.88 (0.82 to 0.93)	20 per 1000 persons	18 per 1000 persons (16 to 19 per 1000 persons)	2 fewer (4 fewer to 1 fewer)	High
**All-cause death**	GLP-1 receptor agonists	Sulfonylureas	0.79 (0.69 to 0.92)	22 per 1000 persons	17 per 1000 persons (15 to 20 per 1000 persons)	5 fewer (7 fewer to 2 fewer)	Moderate
**All-cause death**	GLP-1 receptor agonists	Thiazolidinediones	0.92 (0.80 to 1.07)	19 per 1000 persons	18 per 1000 persons (15 to 20 per 1000 persons)	1 fewer (4 fewer to 1 more)	Moderate
**All-cause death**	GLP-1 receptor agonists	Tirzepatide	1.05 (0.61 to 1.82)	17 per 1000 persons	18 per 1000 persons (10 to 31 per 1000 persons)	1 more (7 fewer to 14 more)	Low
**All-cause death**	Meglitinides	Metformin	1.89 (0.60 to 5.97)	17 per 1000 persons	32 per 1000 persons (10 to 94 per 1000 persons)	15 more (7 fewer to 77 more)	Very low
**All-cause death**	Meglitinides	Non-steroidal MRAs	1.78 (0.57 to 5.57)	18 per 1000 persons	32 per 1000 persons (10 to 93 per 1000 persons)	14 more (8 fewer to 75 more)	Very low
**All-cause death**	Meglitinides	SGLT2 inhibitors	1.79 (0.58 to 5.58)	18 per 1000 persons	32 per 1000 persons (11 to 93 per 1000 persons)	14 more (7 fewer to 75 more)	Low
**All-cause death**	Meglitinides	Standard treatments	1.58 (0.51 to 4.92)	20 per 1000 persons	31 per 1000 persons (10 to 91 per 1000 persons)	11 more (10 fewer to 71 more)	Very low
**All-cause death**	Meglitinides	Sulfonylureas	1.43 (0.46 to 4.45)	22 per 1000 persons	31 per 1000 persons (10 to 91 per 1000 persons)	9 more (12 fewer to 69 more)	Very low
**All-cause death**	Meglitinides	Thiazolidinediones	1.66 (0.53 to 5.19)	19 per 1000 persons	31 per 1000 persons (10 to 91 per 1000 persons)	12 more (9 fewer to 72 more)	Very low

Outcome	Intervention	Comparator	Odds ratio	Baseline risk	Risk if treated	Risk difference	Certainty
**All-cause death**	Meglitinides	Tirzepatide	1.90 (0.54 to 6.68)	17 per 1000 persons	32 per 1000 persons (9 to 104 per 1000 persons)	15 more (8 fewer to 87 more)	Low
**All-cause death**	Metformin	Non-steroidal MRAs	0.94 (0.73 to 1.20)	18 per 1000 persons	17 per 1000 persons (13 to 22 per 1000 persons)	1 fewer (5 fewer to 4 more)	Moderate
**All-cause death**	Metformin	SGLT2 inhibitors	0.95 (0.76 to 1.19)	18 per 1000 persons	17 per 1000 persons (14 to 21 per 1000 persons)	1 fewer (4 fewer to 3 more)	Moderate
**All-cause death**	Metformin	Standard treatments	0.84 (0.67 to 1.04)	20 per 1000 persons	17 per 1000 persons (13 to 21 per 1000 persons)	3 fewer (7 fewer to 1 more)	Moderate
**All-cause death**	Metformin	Sulfonylureas	0.76 (0.60 to 0.96)	22 per 1000 persons	17 per 1000 persons (13 to 21 per 1000 persons)	5 fewer (9 fewer to 1 fewer)	Moderate
**All-cause death**	Metformin	Thiazolidinediones	0.88 (0.69 to 1.12)	19 per 1000 persons	17 per 1000 persons (13 to 21 per 1000 persons)	2 fewer (6 fewer to 2 more)	Moderate
**All-cause death**	Metformin	Tirzepatide	1.00 (0.56 to 1.81)	17 per 1000 persons	17 per 1000 persons (10 to 30 per 1000 persons)	0 (7 fewer to 13 more)	Low
**All-cause death**	Non-steroidal MRAs	SGLT2 inhibitors	1.01 (0.88 to 1.15)	18 per 1000 persons	18 per 1000 persons (16 to 21 per 1000 persons)	0 (2 fewer to 3 more)	Moderate
**All-cause death**	Non-steroidal MRAs	Standard treatments	0.89 (0.79 to 1.00)	20 per 1000 persons	18 per 1000 persons (16 to 20 per 1000 persons)	2 fewer (4 fewer to 0)	Moderate
**All-cause death**	Non-steroidal MRAs	Sulfonylureas	0.81 (0.67 to 0.96)	22 per 1000 persons	18 per 1000 persons (15 to 21 per 1000 persons)	4 fewer (7 fewer to 1 fewer)	Moderate
**All-cause death**	Non-steroidal MRAs	Thiazolidinediones	0.93 (0.78 to 1.12)	19 per 1000 persons	18 per 1000 persons (15 to 21 per 1000 persons)	1 fewer (4 fewer to 2 more)	Moderate
**All-cause death**	Non-steroidal MRAs	Tirzepatide	1.07 (0.61 to 1.87)	17 per 1000 persons	18 per 1000 persons (10 to 31 per 1000 persons)	1 more (7 fewer to 14 more)	Low
**All-cause death**	SGLT2 inhibitors	Standard treatments	0.88 (0.83 to 0.94)	20 per 1000 persons	18 per 1000 persons (17 to 19 per 1000 persons)	2 fewer (3 fewer to 1 fewer)	High
**All-cause death**	SGLT2 inhibitors	Sulfonylureas	0.80 (0.69 to 0.93)	22 per 1000 persons	18 per 1000 persons (15 to 20 per 1000 persons)	4 fewer (7 fewer to 2 fewer)	Moderate
**All-cause death**	SGLT2 inhibitors	Thiazolidinediones	0.93 (0.80 to 1.08)	19 per 1000 persons	18 per 1000 persons (15 to 20 per 1000 persons)	1 fewer (4 fewer to 1 more)	Moderate

Outcome	Intervention	Comparator	Odds ratio	Baseline risk	Risk if treated	Risk difference	Certainty
**All-cause death**	SGLT2 inhibitors	Tirzepatide	1.06 (0.61 to 1.84)	17 per 1000 persons	18 per 1000 persons (10 to 31 per 1000 persons)	1 more (7 fewer to 14 more)	Low
**All-cause death**	Sulfonylureas	Standard treatments	1.10 (0.97 to 1.26)	20 per 1000 persons	22 per 1000 persons (19 to 25 per 1000 persons)	2 more (1 fewer to 5 more)	Moderate
**All-cause death**	Sulfonylureas	Thiazolidinediones	1.16 (0.98 to 1.37)	19 per 1000 persons	22 per 1000 persons (19 to 26 per 1000 persons)	3 more (0 to 7 more)	Moderate
**All-cause death**	Sulfonylureas	Tirzepatide	1.32 (0.76 to 2.31)	17 per 1000 persons	22 per 1000 persons (13 to 38 per 1000 persons)	5 more (4 fewer to 21 more)	Low
**All-cause death**	Thiazolidinediones	Standard treatments	0.95 (0.83 to 1.09)	20 per 1000 persons	19 per 1000 persons (17 to 22 per 1000 persons)	1 fewer (3 fewer to 2 more)	Moderate
**All-cause death**	Thiazolidinediones	Tirzepatide	1.14 (0.65 to 2.01)	17 per 1000 persons	19 per 1000 persons (11 to 34 per 1000 persons)	2 more (6 fewer to 17 more)	Low
**All-cause death**	Tirzepatide	Standard treatments	0.83 (0.48 to 1.44)	20 per 1000 persons	17 per 1000 persons (10 to 29 per 1000 persons)	3 fewer (10 fewer to 9 more)	Moderate
**All-cause death**	Basal insulin	Alpha-glucosidase inhibitors	1.23 (0.40 to 3.85)	18 per 1000 persons	22 per 1000 persons (7 to 66 per 1000 persons)	4 more (11 fewer to 48 more)	Very low
**All-cause death**	Basal-Bolus insulin	Alpha-glucosidase inhibitors	0.89 (0.15 to 5.26)	18 per 1000 persons	16 per 1000 persons (3 to 88 per 1000 persons)	2 fewer (15 fewer to 70 more)	Very low
**All-cause death**	Bolus insulin	Alpha-glucosidase inhibitors	0.54 (0.11 to 2.70)	18 per 1000 persons	10 per 1000 persons (2 to 47 per 1000 persons)	8 fewer (16 fewer to 29 more)	Very low
**All-cause death**	DPP-4 inhibitors	Alpha-glucosidase inhibitors	1.14 (0.39 to 3.33)	18 per 1000 persons	20 per 1000 persons (7 to 58 per 1000 persons)	2 more (11 fewer to 40 more)	Very low
**All-cause death**	GLP-1 receptor agonists	Alpha-glucosidase inhibitors	0.99 (0.34 to 2.94)	18 per 1000 persons	18 per 1000 persons (6 to 51 per 1000 persons)	0 (12 fewer to 33 more)	Very low
**All-cause death**	Meglitinides	Alpha-glucosidase inhibitors	1.79 (0.40 to 7.69)	18 per 1000 persons	32 per 1000 persons (7 to 124 per 1000 persons)	14 more (11 fewer to 106 more)	Very low
**All-cause death**	Metformin	Alpha-glucosidase inhibitors	0.94 (0.31 to 2.78)	18 per 1000 persons	17 per 1000 persons (6 to 48 per 1000 persons)	1 fewer (12 fewer to 30 more)	Very low
**All-cause death**	Non-steroidal MRAs	Alpha-glucosidase inhibitors	1.00 (0.34 to 2.94)	18 per 1000 persons	18 per 1000 persons (6 to 51 per 1000 persons)	0 (12 fewer to 33 more)	Very low

Outcome	Intervention	Comparator	Odds ratio	Baseline risk	Risk if treated	Risk difference	Certainty
**All-cause death**	SGLT2 inhibitors	Alpha-glucosidase inhibitors	0.99 (0.34 to 2.94)	18 per 1000 persons	18 per 1000 persons (6 to 51 per 1000 persons)	0 (12 fewer to 33 more)	Very low
**All-cause death**	Standard treatments	Alpha-glucosidase inhibitors	1.12 (0.38 to 3.33)	18 per 1000 persons	20 per 1000 persons (7 to 58 per 1000 persons)	2 more (11 fewer to 40 more)	Very low
**All-cause death**	Sulfonylureas	Alpha-glucosidase inhibitors	1.23 (0.42 to 3.70)	18 per 1000 persons	22 per 1000 persons (8 to 64 per 1000 persons)	4 more (10 fewer to 46 more)	Very low
**All-cause death**	Thiazolidinediones	Alpha-glucosidase inhibitors	1.08 (0.36 to 3.12)	18 per 1000 persons	19 per 1000 persons (7 to 54 per 1000 persons)	1 more (11 fewer to 36 more)	Very low
**All-cause death**	Tirzepatide	Alpha-glucosidase inhibitors	0.93 (0.28 to 3.12)	18 per 1000 persons	17 per 1000 persons (5 to 54 per 1000 persons)	1 fewer (13 fewer to 36 more)	Very low
**All-cause death**	Basal-Bolus insulin	Basal insulin	0.72 (0.17 to 3.03)	22 per 1000 persons	16 per 1000 persons (4 to 64 per 1000 persons)	6 fewer (18 fewer to 42 more)	Very low
**All-cause death**	Bolus insulin	Basal insulin	0.44 (0.13 to 1.49)	22 per 1000 persons	10 per 1000 persons (3 to 32 per 1000 persons)	12 fewer (19 fewer to 10 more)	Very low
**All-cause death**	DPP-4 inhibitors	Basal insulin	0.93 (0.68 to 1.25)	22 per 1000 persons	20 per 1000 persons (15 to 27 per 1000 persons)	2 fewer (7 fewer to 5 more)	Moderate
**All-cause death**	GLP-1 receptor agonists	Basal insulin	0.80 (0.59 to 1.08)	22 per 1000 persons	18 per 1000 persons (13 to 24 per 1000 persons)	4 fewer (9 fewer to 2 more)	Moderate
**All-cause death**	Meglitinides	Basal insulin	1.45 (0.45 to 4.55)	22 per 1000 persons	32 per 1000 persons (10 to 93 per 1000 persons)	10 more (12 fewer to 71 more)	Very low
**All-cause death**	Metformin	Basal insulin	0.76 (0.53 to 1.10)	22 per 1000 persons	17 per 1000 persons (12 to 24 per 1000 persons)	5 fewer (10 fewer to 2 more)	Moderate
**All-cause death**	Non-steroidal MRAs	Basal insulin	0.81 (0.58 to 1.12)	22 per 1000 persons	18 per 1000 persons (13 to 25 per 1000 persons)	4 fewer (9 fewer to 3 more)	Moderate
**All-cause death**	SGLT2 inhibitors	Basal insulin	0.81 (0.59 to 1.10)	22 per 1000 persons	18 per 1000 persons (13 to 24 per 1000 persons)	4 fewer (9 fewer to 2 more)	Moderate
**All-cause death**	Standard treatments	Basal insulin	0.91 (0.67 to 1.23)	22 per 1000 persons	20 per 1000 persons (15 to 27 per 1000 persons)	2 fewer (7 fewer to 5 more)	Moderate
**All-cause death**	Sulfonylureas	Basal insulin	1.00 (0.74 to 1.37)	22 per 1000 persons	22 per 1000 persons (16 to 30 per 1000 persons)	0 (6 fewer to 8 more)	High

Outcome	Intervention	Comparator	Odds ratio	Baseline risk	Risk if treated	Risk difference	Certainty
**All-cause death**	Thiazolidinediones	Basal insulin	0.87 (0.62 to 1.20)	22 per 1000 persons	19 per 1000 persons (14 to 26 per 1000 persons)	3 fewer (8 fewer to 4 more)	Moderate
**All-cause death**	Tirzepatide	Basal insulin	0.76 (0.47 to 1.22)	22 per 1000 persons	17 per 1000 persons (10 to 27 per 1000 persons)	5 fewer (12 fewer to 5 more)	Moderate
**All-cause death**	Bolus insulin	Basal-Bolus insulin	0.61 (0.09 to 4.00)	16 per 1000 persons	10 per 1000 persons (2 to 61 per 1000 persons)	6 fewer (14 fewer to 45 more)	Very low
**All-cause death**	DPP-4 inhibitors	Basal-Bolus insulin	1.28 (0.30 to 5.26)	16 per 1000 persons	20 per 1000 persons (5 to 79 per 1000 persons)	4 more (11 fewer to 63 more)	Very low
**All-cause death**	GLP-1 receptor agonists	Basal-Bolus insulin	1.11 (0.26 to 4.55)	16 per 1000 persons	18 per 1000 persons (4 to 69 per 1000 persons)	2 more (12 fewer to 53 more)	Very low
**All-cause death**	Meglitinides	Basal-Bolus insulin	2.00 (0.32 to 12.50)	16 per 1000 persons	31 per 1000 persons (5 to 169 per 1000 persons)	15 more (11 fewer to 153 more)	Very low
**All-cause death**	Metformin	Basal-Bolus insulin	1.05 (0.25 to 4.55)	16 per 1000 persons	17 per 1000 persons (4 to 69 per 1000 persons)	1 more (12 fewer to 53 more)	Very low
**All-cause death**	Non-steroidal MRAs	Basal-Bolus insulin	1.12 (0.27 to 4.76)	16 per 1000 persons	18 per 1000 persons (4 to 72 per 1000 persons)	2 more (12 fewer to 56 more)	Very low
**All-cause death**	SGLT2 inhibitors	Basal-Bolus insulin	1.11 (0.27 to 4.76)	16 per 1000 persons	18 per 1000 persons (4 to 72 per 1000 persons)	2 more (12 fewer to 56 more)	Very low
**All-cause death**	Standard treatments	Basal-Bolus insulin	1.27 (0.30 to 5.26)	16 per 1000 persons	20 per 1000 persons (5 to 79 per 1000 persons)	4 more (11 fewer to 63 more)	Very low
**All-cause death**	Sulfonylureas	Basal-Bolus insulin	1.39 (0.33 to 5.88)	16 per 1000 persons	22 per 1000 persons (5 to 87 per 1000 persons)	6 more (11 fewer to 71 more)	Very low
**All-cause death**	Thiazolidinediones	Basal-Bolus insulin	1.20 (0.28 to 5.00)	16 per 1000 persons	19 per 1000 persons (5 to 75 per 1000 persons)	3 more (11 fewer to 59 more)	Very low
**All-cause death**	Tirzepatide	Basal-Bolus insulin	1.05 (0.23 to 4.76)	16 per 1000 persons	17 per 1000 persons (4 to 72 per 1000 persons)	1 more (12 fewer to 56 more)	Very low
**All-cause death**	DPP-4 inhibitors	Bolus insulin	2.13 (0.64 to 7.14)	10 per 1000 persons	21 per 1000 persons (6 to 67 per 1000 persons)	11 more (4 fewer to 57 more)	Very low
**All-cause death**	GLP-1 receptor agonists	Bolus insulin	1.82 (0.55 to 5.88)	10 per 1000 persons	18 per 1000 persons (6 to 56 per 1000 persons)	8 more (4 fewer to 46 more)	Very low

Outcome	Intervention	Comparator	Odds ratio	Baseline risk	Risk if treated	Risk difference	Certainty
**All-cause death**	Meglitinides	Bolus insulin	3.33 (0.63 to 16.67)	10 per 1000 persons	33 per 1000 persons (6 to 144 per 1000 persons)	23 more (4 fewer to 134 more)	Very low
**All-cause death**	Metformin	Bolus insulin	1.72 (0.52 to 5.88)	10 per 1000 persons	17 per 1000 persons (5 to 56 per 1000 persons)	7 more (5 fewer to 46 more)	Low
**All-cause death**	Non-steroidal MRAs	Bolus insulin	1.85 (0.56 to 6.25)	10 per 1000 persons	18 per 1000 persons (6 to 59 per 1000 persons)	8 more (4 fewer to 49 more)	Very low
**All-cause death**	SGLT2 inhibitors	Bolus insulin	1.85 (0.55 to 6.25)	10 per 1000 persons	18 per 1000 persons (6 to 59 per 1000 persons)	8 more (4 fewer to 49 more)	Low
**All-cause death**	Standard treatments	Bolus insulin	2.08 (0.63 to 6.67)	10 per 1000 persons	21 per 1000 persons (6 to 63 per 1000 persons)	11 more (4 fewer to 53 more)	Very low
**All-cause death**	Sulfonylureas	Bolus insulin	2.27 (0.69 to 7.69)	10 per 1000 persons	22 per 1000 persons (7 to 72 per 1000 persons)	12 more (3 fewer to 62 more)	Very low
**All-cause death**	Thiazolidinediones	Bolus insulin	1.96 (0.60 to 6.67)	10 per 1000 persons	19 per 1000 persons (6 to 63 per 1000 persons)	9 more (4 fewer to 53 more)	Very low
**All-cause death**	Tirzepatide	Bolus insulin	1.72 (0.47 to 6.25)	10 per 1000 persons	17 per 1000 persons (5 to 59 per 1000 persons)	7 more (5 fewer to 49 more)	Low
**All-cause death**	GLP-1 receptor agonists	DPP-4 inhibitors	0.86 (0.79 to 0.94)	20 per 1000 persons	17 per 1000 persons (16 to 19 per 1000 persons)	3 fewer (4 fewer to 1 fewer)	Moderate
**All-cause death**	Meglitinides	DPP-4 inhibitors	1.56 (0.50 to 4.76)	20 per 1000 persons	31 per 1000 persons (10 to 89 per 1000 persons)	11 more (10 fewer to 69 more)	Very low
**All-cause death**	Metformin	DPP-4 inhibitors	0.83 (0.66 to 1.03)	20 per 1000 persons	17 per 1000 persons (13 to 21 per 1000 persons)	3 fewer (7 fewer to 1 more)	Moderate
**All-cause death**	Non-steroidal MRAs	DPP-4 inhibitors	0.88 (0.76 to 1.01)	20 per 1000 persons	18 per 1000 persons (15 to 20 per 1000 persons)	2 fewer (5 fewer to 0)	Moderate
**All-cause death**	SGLT2 inhibitors	DPP-4 inhibitors	0.87 (0.79 to 0.96)	20 per 1000 persons	17 per 1000 persons (16 to 19 per 1000 persons)	3 fewer (4 fewer to 1 fewer)	Moderate
**All-cause death**	Standard treatments	DPP-4 inhibitors	0.99 (0.93 to 1.05)	20 per 1000 persons	20 per 1000 persons (19 to 21 per 1000 persons)	0 (1 fewer to 1 more)	Moderate
**All-cause death**	Sulfonylureas	DPP-4 inhibitors	1.09 (0.96 to 1.23)	20 per 1000 persons	22 per 1000 persons (19 to 25 per 1000 persons)	2 more (1 fewer to 5 more)	Moderate

Outcome	Intervention	Comparator	Odds ratio	Baseline risk	Risk if treated	Risk difference	Certainty
**All-cause death**	Thiazolidinediones	DPP-4 inhibitors	0.94 (0.81 to 1.09)	20 per 1000 persons	19 per 1000 persons (16 to 22 per 1000 persons)	1 fewer (4 fewer to 2 more)	Moderate
**All-cause death**	Tirzepatide	DPP-4 inhibitors	0.82 (0.47 to 1.43)	20 per 1000 persons	16 per 1000 persons (10 to 28 per 1000 persons)	4 fewer (10 fewer to 8 more)	Moderate
**All-cause death**	Meglitinides	GLP-1 receptor agonists	1.82 (0.58 to 5.56)	18 per 1000 persons	32 per 1000 persons (10 to 92 per 1000 persons)	14 more (8 fewer to 74 more)	Low
**All-cause death**	Metformin	GLP-1 receptor agonists	0.95 (0.76 to 1.19)	18 per 1000 persons	17 per 1000 persons (14 to 21 per 1000 persons)	1 fewer (4 fewer to 3 more)	Moderate
**All-cause death**	Non-steroidal MRAs	GLP-1 receptor agonists	1.01 (0.88 to 1.16)	18 per 1000 persons	18 per 1000 persons (16 to 21 per 1000 persons)	0 (2 fewer to 3 more)	Moderate
**All-cause death**	SGLT2 inhibitors	GLP-1 receptor agonists	1.01 (0.93 to 1.10)	18 per 1000 persons	18 per 1000 persons (17 to 20 per 1000 persons)	0 (1 fewer to 2 more)	Moderate
**All-cause death**	Standard treatments	GLP-1 receptor agonists	1.14 (1.08 to 1.22)	18 per 1000 persons	20 per 1000 persons (19 to 22 per 1000 persons)	2 more (1 more to 4 more)	High
**All-cause death**	Sulfonylureas	GLP-1 receptor agonists	1.27 (1.09 to 1.45)	18 per 1000 persons	23 per 1000 persons (20 to 26 per 1000 persons)	5 more (2 more to 8 more)	Moderate
**All-cause death**	Thiazolidinediones	GLP-1 receptor agonists	1.09 (0.93 to 1.25)	18 per 1000 persons	20 per 1000 persons (17 to 22 per 1000 persons)	2 more (1 fewer to 4 more)	Moderate
**All-cause death**	Tirzepatide	GLP-1 receptor agonists	0.95 (0.55 to 1.64)	18 per 1000 persons	17 per 1000 persons (10 to 29 per 1000 persons)	1 fewer (8 fewer to 11 more)	Low
**All-cause death**	Metformin	Meglitinides	0.53 (0.17 to 1.67)	31 per 1000 persons	17 per 1000 persons (5 to 51 per 1000 persons)	14 fewer (26 fewer to 20 more)	Very low
**All-cause death**	Non-steroidal MRAs	Meglitinides	0.56 (0.18 to 1.75)	31 per 1000 persons	18 per 1000 persons (6 to 53 per 1000 persons)	13 fewer (25 fewer to 22 more)	Very low
**All-cause death**	SGLT2 inhibitors	Meglitinides	0.56 (0.18 to 1.72)	31 per 1000 persons	18 per 1000 persons (6 to 52 per 1000 persons)	13 fewer (25 fewer to 21 more)	Very low
**All-cause death**	Standard treatments	Meglitinides	0.63 (0.20 to 1.96)	31 per 1000 persons	20 per 1000 persons (6 to 59 per 1000 persons)	11 fewer (25 fewer to 28 more)	Very low
**All-cause death**	Sulfonylureas	Meglitinides	0.70 (0.22 to 2.17)	31 per 1000 persons	22 per 1000 persons (7 to 65 per 1000 persons)	9 fewer (24 fewer to 34 more)	Very low

Outcome	Intervention	Comparator	Odds ratio	Baseline risk	Risk if treated	Risk difference	Certainty
**All-cause death**	Thiazolidinediones	Meglitinides	0.60 (0.19 to 1.89)	31 per 1000 persons	19 per 1000 persons (6 to 57 per 1000 persons)	12 fewer (25 fewer to 26 more)	Very low
**All-cause death**	Tirzepatide	Meglitinides	0.53 (0.15 to 1.85)	31 per 1000 persons	17 per 1000 persons (5 to 56 per 1000 persons)	14 fewer (26 fewer to 25 more)	Very low
**All-cause death**	Non-steroidal MRAs	Metformin	1.06 (0.83 to 1.37)	17 per 1000 persons	18 per 1000 persons (14 to 23 per 1000 persons)	1 more (3 fewer to 6 more)	Moderate
**All-cause death**	SGLT2 inhibitors	Metformin	1.05 (0.84 to 1.32)	17 per 1000 persons	18 per 1000 persons (14 to 22 per 1000 persons)	1 more (3 fewer to 5 more)	Moderate
**All-cause death**	Standard treatments	Metformin	1.19 (0.96 to 1.49)	17 per 1000 persons	20 per 1000 persons (16 to 25 per 1000 persons)	3 more (1 fewer to 8 more)	Moderate
**All-cause death**	Sulfonylureas	Metformin	1.32 (1.04 to 1.67)	17 per 1000 persons	22 per 1000 persons (18 to 28 per 1000 persons)	5 more (1 more to 11 more)	Low
**All-cause death**	Thiazolidinediones	Metformin	1.14 (0.89 to 1.45)	17 per 1000 persons	19 per 1000 persons (15 to 24 per 1000 persons)	2 more (2 fewer to 7 more)	Moderate
**All-cause death**	Tirzepatide	Metformin	1.00 (0.55 to 1.79)	17 per 1000 persons	17 per 1000 persons (9 to 30 per 1000 persons)	0 (8 fewer to 13 more)	Low
**All-cause death**	SGLT2 inhibitors	Non-steroidal MRAs	0.99 (0.87 to 1.14)	18 per 1000 persons	18 per 1000 persons (16 to 20 per 1000 persons)	0 (2 fewer to 2 more)	Moderate
**All-cause death**	Standard treatments	Non-steroidal MRAs	1.12 (1.00 to 1.27)	18 per 1000 persons	20 per 1000 persons (18 to 23 per 1000 persons)	2 more (0 to 5 more)	Moderate
**All-cause death**	Sulfonylureas	Non-steroidal MRAs	1.23 (1.04 to 1.49)	18 per 1000 persons	22 per 1000 persons (19 to 27 per 1000 persons)	4 more (1 more to 9 more)	Moderate
**All-cause death**	Thiazolidinediones	Non-steroidal MRAs	1.08 (0.89 to 1.28)	18 per 1000 persons	19 per 1000 persons (16 to 23 per 1000 persons)	1 more (2 fewer to 5 more)	Moderate
**All-cause death**	Tirzepatide	Non-steroidal MRAs	0.93 (0.53 to 1.64)	18 per 1000 persons	17 per 1000 persons (10 to 29 per 1000 persons)	1 fewer (8 fewer to 11 more)	Low
**All-cause death**	Standard treatments	SGLT2 inhibitors	1.14 (1.06 to 1.20)	18 per 1000 persons	20 per 1000 persons (19 to 22 per 1000 persons)	2 more (1 more to 4 more)	High
**All-cause death**	Sulfonylureas	SGLT2 inhibitors	1.25 (1.08 to 1.45)	18 per 1000 persons	22 per 1000 persons (19 to 26 per 1000 persons)	4 more (1 more to 8 more)	Moderate

Outcome	Intervention	Comparator	Odds ratio	Baseline risk	Risk if treated	Risk difference	Certainty
**All-cause death**	Thiazolidinediones	SGLT2 inhibitors	1.08 (0.93 to 1.25)	18 per 1000 persons	19 per 1000 persons (17 to 22 per 1000 persons)	1 more (1 fewer to 4 more)	Moderate
**All-cause death**	Tirzepatide	SGLT2 inhibitors	0.94 (0.54 to 1.64)	18 per 1000 persons	17 per 1000 persons (10 to 29 per 1000 persons)	1 fewer (8 fewer to 11 more)	Low
**All-cause death**	Standard treatments	Sulfonylureas	0.91 (0.79 to 1.03)	22 per 1000 persons	20 per 1000 persons (18 to 23 per 1000 persons)	2 fewer (4 fewer to 1 more)	Moderate
**All-cause death**	Thiazolidinediones	Sulfonylureas	0.86 (0.73 to 1.02)	22 per 1000 persons	19 per 1000 persons (16 to 22 per 1000 persons)	3 fewer (6 fewer to 0)	Moderate
**All-cause death**	Tirzepatide	Sulfonylureas	0.76 (0.43 to 1.32)	22 per 1000 persons	17 per 1000 persons (10 to 29 per 1000 persons)	5 fewer (12 fewer to 7 more)	Low
**All-cause death**	Standard treatments	Thiazolidinediones	1.05 (0.92 to 1.20)	19 per 1000 persons	20 per 1000 persons (17 to 23 per 1000 persons)	1 more (2 fewer to 4 more)	Moderate
**All-cause death**	Tirzepatide	Thiazolidinediones	0.88 (0.50 to 1.54)	19 per 1000 persons	17 per 1000 persons (10 to 29 per 1000 persons)	2 fewer (9 fewer to 10 more)	Moderate
**All-cause death**	Standard treatments	Tirzepatide	1.20 (0.69 to 2.08)	17 per 1000 persons	20 per 1000 persons (12 to 35 per 1000 persons)	3 more (5 fewer to 18 more)	Low
**Cardiovascular death**	Alpha-glucosidase inhibitors	Basal insulin	0.77 (0.15 to 3.88)	17 per 1000 persons	13 per 1000 persons (3 to 63 per 1000 persons)	4 fewer (14 fewer to 46 more)	Very low
**Cardiovascular death**	Alpha-glucosidase inhibitors	Basal-Bolus insulin	0.45 (0.03 to 7.04)	29 per 1000 persons	13 per 1000 persons (1 to 174 per 1000 persons)	16 fewer (28 fewer to 145 more)	Very low
**Cardiovascular death**	Alpha-glucosidase inhibitors	Bolus insulin	0.94 (0.06 to 14.87)	14 per 1000 persons	13 per 1000 persons (1 to 174 per 1000 persons)	1 fewer (13 fewer to 160 more)	Very low
**Cardiovascular death**	Alpha-glucosidase inhibitors	DPP-4 inhibitors	0.99 (0.21 to 4.71)	13 per 1000 persons	13 per 1000 persons (3 to 58 per 1000 persons)	0 (10 fewer to 45 more)	Low
**Cardiovascular death**	Alpha-glucosidase inhibitors	GLP-1 receptor agonists	1.14 (0.24 to 5.40)	11 per 1000 persons	13 per 1000 persons (3 to 57 per 1000 persons)	2 more (8 fewer to 46 more)	Low
**Cardiovascular death**	Alpha-glucosidase inhibitors	Meglitinides	1.55 (0.15 to 16.03)	8 per 1000 persons	12 per 1000 persons (1 to 114 per 1000 persons)	4 more (7 fewer to 106 more)	Low
**Cardiovascular death**	Alpha-glucosidase inhibitors	Metformin	1.05 (0.19 to 5.69)	12 per 1000 persons	13 per 1000 persons (2 to 65 per 1000 persons)	1 more (10 fewer to 53 more)	Low

Outcome	Intervention	Comparator	Odds ratio	Baseline risk	Risk if treated	Risk difference	Certainty
**Cardiovascular death**	Alpha-glucosidase inhibitors	Non-steroidal MRAs	1.13 (0.24 to 5.40)	11 per 1000 persons	12 per 1000 persons (3 to 57 per 1000 persons)	1 more (8 fewer to 46 more)	Low
**Cardiovascular death**	Alpha-glucosidase inhibitors	SGLT2 inhibitors	1.15 (0.24 to 5.44)	11 per 1000 persons	13 per 1000 persons (3 to 57 per 1000 persons)	2 more (8 fewer to 46 more)	Low
**Cardiovascular death**	Alpha-glucosidase inhibitors	Standard treatments	0.99 (0.21 to 4.70)	13 per 1000 persons	13 per 1000 persons (3 to 58 per 1000 persons)	0 (10 fewer to 45 more)	Low
**Cardiovascular death**	Alpha-glucosidase inhibitors	Sulfonylureas	0.98 (0.21 to 4.67)	13 per 1000 persons	13 per 1000 persons (3 to 58 per 1000 persons)	0 (10 fewer to 45 more)	Low
**Cardiovascular death**	Alpha-glucosidase inhibitors	Thiazolidinediones	1.07 (0.22 to 5.12)	12 per 1000 persons	13 per 1000 persons (3 to 59 per 1000 persons)	1 more (9 fewer to 47 more)	Low
**Cardiovascular death**	Alpha-glucosidase inhibitors	Tirzepatide	1.00 (0.15 to 6.50)	13 per 1000 persons	13 per 1000 persons (2 to 79 per 1000 persons)	0 (11 fewer to 66 more)	Very low
**Cardiovascular death**	Basal insulin	Basal-Bolus insulin	0.58 (0.06 to 5.88)	29 per 1000 persons	17 per 1000 persons (2 to 149 per 1000 persons)	12 fewer (27 fewer to 120 more)	Very low
**Cardiovascular death**	Basal insulin	Bolus insulin	1.22 (0.13 to 11.90)	14 per 1000 persons	17 per 1000 persons (2 to 145 per 1000 persons)	3 more (12 fewer to 131 more)	Very low
**Cardiovascular death**	Basal insulin	DPP-4 inhibitors	1.28 (0.83 to 1.99)	13 per 1000 persons	17 per 1000 persons (11 to 26 per 1000 persons)	4 more (2 fewer to 13 more)	Low
**Cardiovascular death**	Basal insulin	GLP-1 receptor agonists	1.47 (0.95 to 2.29)	11 per 1000 persons	16 per 1000 persons (10 to 25 per 1000 persons)	5 more (1 fewer to 14 more)	Low
**Cardiovascular death**	Basal insulin	Meglitinides	2.00 (0.33 to 12.12)	8 per 1000 persons	16 per 1000 persons (3 to 89 per 1000 persons)	8 more (5 fewer to 81 more)	Low
**Cardiovascular death**	Basal insulin	Metformin	1.35 (0.61 to 3.03)	12 per 1000 persons	16 per 1000 persons (7 to 35 per 1000 persons)	4 more (5 fewer to 23 more)	Low
**Cardiovascular death**	Basal insulin	Non-steroidal MRAs	1.46 (0.92 to 2.33)	11 per 1000 persons	16 per 1000 persons (10 to 25 per 1000 persons)	5 more (1 fewer to 14 more)	Low
**Cardiovascular death**	Basal insulin	SGLT2 inhibitors	1.48 (0.95 to 2.32)	11 per 1000 persons	16 per 1000 persons (10 to 25 per 1000 persons)	5 more (1 fewer to 14 more)	Low
**Cardiovascular death**	Basal insulin	Standard treatments	1.28 (0.83 to 1.99)	13 per 1000 persons	17 per 1000 persons (11 to 26 per 1000 persons)	4 more (2 fewer to 13 more)	Low

Outcome	Intervention	Comparator	Odds ratio	Baseline risk	Risk if treated	Risk difference	Certainty
**Cardiovascular death**	Basal insulin	Sulfonylureas	1.27 (0.80 to 2.01)	13 per 1000 persons	16 per 1000 persons (10 to 26 per 1000 persons)	3 more (3 fewer to 13 more)	Low
**Cardiovascular death**	Basal insulin	Thiazolidinediones	1.38 (0.86 to 2.22)	12 per 1000 persons	16 per 1000 persons (10 to 26 per 1000 persons)	4 more (2 fewer to 14 more)	Low
**Cardiovascular death**	Basal insulin	Tirzepatide	1.29 (0.48 to 3.43)	13 per 1000 persons	17 per 1000 persons (6 to 43 per 1000 persons)	4 more (7 fewer to 30 more)	Moderate
**Cardiovascular death**	Basal-Bolus insulin	Bolus insulin	2.12 (0.08 to 53.34)	14 per 1000 persons	29 per 1000 persons (1 to 431 per 1000 persons)	15 more (13 fewer to 417 more)	Very low
**Cardiovascular death**	Basal-Bolus insulin	DPP-4 inhibitors	2.23 (0.23 to 21.94)	13 per 1000 persons	29 per 1000 persons (3 to 224 per 1000 persons)	16 more (10 fewer to 211 more)	Low
**Cardiovascular death**	Basal-Bolus insulin	GLP-1 receptor agonists	2.56 (0.26 to 25.15)	11 per 1000 persons	28 per 1000 persons (3 to 219 per 1000 persons)	17 more (8 fewer to 208 more)	Low
**Cardiovascular death**	Basal-Bolus insulin	Meglitinides	3.48 (0.20 to 61.62)	8 per 1000 persons	27 per 1000 persons (2 to 332 per 1000 persons)	19 more (6 fewer to 324 more)	Very low
**Cardiovascular death**	Basal-Bolus insulin	Metformin	2.35 (0.22 to 25.43)	12 per 1000 persons	28 per 1000 persons (3 to 236 per 1000 persons)	16 more (9 fewer to 224 more)	Very low
**Cardiovascular death**	Basal-Bolus insulin	Non-steroidal MRAs	2.55 (0.26 to 25.14)	11 per 1000 persons	28 per 1000 persons (3 to 219 per 1000 persons)	17 more (8 fewer to 208 more)	Low
**Cardiovascular death**	Basal-Bolus insulin	SGLT2 inhibitors	2.58 (0.26 to 25.38)	11 per 1000 persons	28 per 1000 persons (3 to 220 per 1000 persons)	17 more (8 fewer to 209 more)	Low
**Cardiovascular death**	Basal-Bolus insulin	Standard treatments	2.23 (0.23 to 21.92)	13 per 1000 persons	29 per 1000 persons (3 to 224 per 1000 persons)	16 more (10 fewer to 211 more)	Low
**Cardiovascular death**	Basal-Bolus insulin	Sulfonylureas	2.21 (0.22 to 21.71)	13 per 1000 persons	28 per 1000 persons (3 to 222 per 1000 persons)	15 more (10 fewer to 209 more)	Low
**Cardiovascular death**	Basal-Bolus insulin	Thiazolidinediones	2.40 (0.24 to 23.79)	12 per 1000 persons	28 per 1000 persons (3 to 224 per 1000 persons)	16 more (9 fewer to 212 more)	Low
**Cardiovascular death**	Basal-Bolus insulin	Tirzepatide	2.24 (0.18 to 27.63)	13 per 1000 persons	29 per 1000 persons (2 to 267 per 1000 persons)	16 more (11 fewer to 254 more)	Very low
**Cardiovascular death**	Bolus insulin	DPP-4 inhibitors	1.05 (0.11 to 10.27)	13 per 1000 persons	14 per 1000 persons (1 to 119 per 1000 persons)	1 more (12 fewer to 106 more)	Very low

Outcome	Intervention	Comparator	Odds ratio	Baseline risk	Risk if treated	Risk difference	Certainty
**Cardiovascular death**	Bolus insulin	GLP-1 receptor agonists	1.21 (0.12 to 11.76)	11 per 1000 persons	13 per 1000 persons (1 to 116 per 1000 persons)	2 more (10 fewer to 105 more)	Very low
**Cardiovascular death**	Bolus insulin	Meglitinides	1.64 (0.09 to 28.95)	8 per 1000 persons	13 per 1000 persons (1 to 189 per 1000 persons)	5 more (7 fewer to 181 more)	Very low
**Cardiovascular death**	Bolus insulin	Metformin	1.11 (0.10 to 11.94)	12 per 1000 persons	13 per 1000 persons (1 to 127 per 1000 persons)	1 more (11 fewer to 115 more)	Very low
**Cardiovascular death**	Bolus insulin	Non-steroidal MRAs	1.20 (0.12 to 11.76)	11 per 1000 persons	13 per 1000 persons (1 to 116 per 1000 persons)	2 more (10 fewer to 105 more)	Very low
**Cardiovascular death**	Bolus insulin	SGLT2 inhibitors	1.22 (0.12 to 11.88)	11 per 1000 persons	13 per 1000 persons (1 to 117 per 1000 persons)	2 more (10 fewer to 106 more)	Low
**Cardiovascular death**	Bolus insulin	Standard treatments	1.05 (0.11 to 10.26)	13 per 1000 persons	14 per 1000 persons (1 to 119 per 1000 persons)	1 more (12 fewer to 106 more)	Very low
**Cardiovascular death**	Bolus insulin	Sulfonylureas	1.04 (0.11 to 10.22)	13 per 1000 persons	14 per 1000 persons (1 to 119 per 1000 persons)	1 more (12 fewer to 106 more)	Very low
**Cardiovascular death**	Bolus insulin	Thiazolidinediones	1.13 (0.12 to 11.14)	12 per 1000 persons	14 per 1000 persons (1 to 119 per 1000 persons)	2 more (11 fewer to 107 more)	Very low
**Cardiovascular death**	Bolus insulin	Tirzepatide	1.06 (0.09 to 12.53)	13 per 1000 persons	14 per 1000 persons (1 to 142 per 1000 persons)	1 more (12 fewer to 129 more)	Very low
**Cardiovascular death**	DPP-4 inhibitors	GLP-1 receptor agonists	1.15 (1.02 to 1.28)	11 per 1000 persons	13 per 1000 persons (11 to 14 per 1000 persons)	2 more (0 to 3 more)	Moderate
**Cardiovascular death**	DPP-4 inhibitors	Meglitinides	1.56 (0.27 to 8.97)	8 per 1000 persons	12 per 1000 persons (2 to 67 per 1000 persons)	4 more (6 fewer to 59 more)	Low
**Cardiovascular death**	DPP-4 inhibitors	Metformin	1.05 (0.53 to 2.09)	12 per 1000 persons	13 per 1000 persons (6 to 25 per 1000 persons)	1 more (6 fewer to 13 more)	Low
**Cardiovascular death**	DPP-4 inhibitors	Non-steroidal MRAs	1.14 (0.96 to 1.36)	11 per 1000 persons	13 per 1000 persons (11 to 15 per 1000 persons)	2 more (0 to 4 more)	Moderate
**Cardiovascular death**	DPP-4 inhibitors	SGLT2 inhibitors	1.16 (1.03 to 1.30)	11 per 1000 persons	13 per 1000 persons (11 to 14 per 1000 persons)	2 more (0 to 3 more)	Moderate
**Cardiovascular death**	DPP-4 inhibitors	Standard treatments	1.00 (0.92 to 1.09)	13 per 1000 persons	13 per 1000 persons (12 to 14 per 1000 persons)	0 (1 fewer to 1 more)	Moderate

Outcome	Intervention	Comparator	Odds ratio	Baseline risk	Risk if treated	Risk difference	Certainty
**Cardiovascular death**	DPP-4 inhibitors	Sulfonylureas	0.99 (0.82 to 1.19)	13 per 1000 persons	13 per 1000 persons (11 to 15 per 1000 persons)	0 (2 fewer to 2 more)	High
**Cardiovascular death**	DPP-4 inhibitors	Thiazolidinediones	1.08 (0.88 to 1.32)	12 per 1000 persons	13 per 1000 persons (11 to 16 per 1000 persons)	1 more (1 fewer to 4 more)	Moderate
**Cardiovascular death**	DPP-4 inhibitors	Tirzepatide	1.00 (0.35 to 2.87)	13 per 1000 persons	13 per 1000 persons (5 to 36 per 1000 persons)	0 (8 fewer to 23 more)	Low
**Cardiovascular death**	GLP-1 receptor agonists	Meglitinides	1.36 (0.24 to 7.83)	8 per 1000 persons	11 per 1000 persons (2 to 59 per 1000 persons)	3 more (6 fewer to 51 more)	Low
**Cardiovascular death**	GLP-1 receptor agonists	Metformin	0.92 (0.46 to 1.83)	12 per 1000 persons	11 per 1000 persons (6 to 22 per 1000 persons)	1 fewer (6 fewer to 10 more)	Moderate
**Cardiovascular death**	GLP-1 receptor agonists	Non-steroidal MRAs	0.99 (0.84 to 1.18)	11 per 1000 persons	11 per 1000 persons (9 to 13 per 1000 persons)	0 (2 fewer to 2 more)	Moderate
**Cardiovascular death**	GLP-1 receptor agonists	SGLT2 inhibitors	1.01 (0.90 to 1.13)	11 per 1000 persons	11 per 1000 persons (10 to 12 per 1000 persons)	0 (1 fewer to 1 more)	Moderate
**Cardiovascular death**	GLP-1 receptor agonists	Standard treatments	0.87 (0.81 to 0.94)	13 per 1000 persons	11 per 1000 persons (11 to 12 per 1000 persons)	2 fewer (2 fewer to 1 fewer)	High
**Cardiovascular death**	GLP-1 receptor agonists	Sulfonylureas	0.86 (0.70 to 1.07)	13 per 1000 persons	11 per 1000 persons (9 to 14 per 1000 persons)	2 fewer (4 fewer to 1 more)	Moderate
**Cardiovascular death**	GLP-1 receptor agonists	Thiazolidinediones	0.94 (0.77 to 1.15)	12 per 1000 persons	11 per 1000 persons (9 to 14 per 1000 persons)	1 fewer (3 fewer to 2 more)	Moderate
**Cardiovascular death**	GLP-1 receptor agonists	Tirzepatide	0.88 (0.31 to 2.50)	13 per 1000 persons	11 per 1000 persons (4 to 32 per 1000 persons)	2 fewer (9 fewer to 19 more)	Low
**Cardiovascular death**	Meglitinides	Metformin	0.68 (0.11 to 4.15)	12 per 1000 persons	8 per 1000 persons (1 to 48 per 1000 persons)	4 fewer (11 fewer to 36 more)	Very low
**Cardiovascular death**	Meglitinides	Non-steroidal MRAs	0.73 (0.13 to 4.24)	11 per 1000 persons	8 per 1000 persons (1 to 45 per 1000 persons)	3 fewer (10 fewer to 34 more)	Very low
**Cardiovascular death**	Meglitinides	SGLT2 inhibitors	0.74 (0.13 to 4.28)	11 per 1000 persons	8 per 1000 persons (1 to 45 per 1000 persons)	3 fewer (10 fewer to 34 more)	Low
**Cardiovascular death**	Meglitinides	Standard treatments	0.64 (0.11 to 3.69)	13 per 1000 persons	8 per 1000 persons (1 to 46 per 1000 persons)	5 fewer (12 fewer to 33 more)	Very low

Outcome	Intervention	Comparator	Odds ratio	Baseline risk	Risk if treated	Risk difference	Certainty
**Cardiovascular death**	Meglitinides	Sulfonylureas	0.64 (0.11 to 3.65)	13 per 1000 persons	8 per 1000 persons (1 to 46 per 1000 persons)	5 fewer (12 fewer to 33 more)	Very low
**Cardiovascular death**	Meglitinides	Thiazolidinediones	0.69 (0.12 to 3.99)	12 per 1000 persons	8 per 1000 persons (1 to 46 per 1000 persons)	4 fewer (11 fewer to 34 more)	Very low
**Cardiovascular death**	Meglitinides	Tirzepatide	0.64 (0.08 to 4.95)	13 per 1000 persons	8 per 1000 persons (1 to 61 per 1000 persons)	5 fewer (12 fewer to 48 more)	Very low
**Cardiovascular death**	Metformin	Non-steroidal MRAs	1.08 (0.54 to 2.18)	11 per 1000 persons	12 per 1000 persons (6 to 24 per 1000 persons)	1 more (5 fewer to 13 more)	Low
**Cardiovascular death**	Metformin	SGLT2 inhibitors	1.10 (0.55 to 2.18)	11 per 1000 persons	12 per 1000 persons (6 to 24 per 1000 persons)	1 more (5 fewer to 13 more)	Low
**Cardiovascular death**	Metformin	Standard treatments	0.95 (0.48 to 1.88)	13 per 1000 persons	12 per 1000 persons (6 to 24 per 1000 persons)	1 fewer (7 fewer to 11 more)	Low
**Cardiovascular death**	Metformin	Sulfonylureas	0.94 (0.48 to 1.85)	13 per 1000 persons	12 per 1000 persons (6 to 24 per 1000 persons)	1 fewer (7 fewer to 11 more)	Low
**Cardiovascular death**	Metformin	Thiazolidinediones	1.02 (0.51 to 2.05)	12 per 1000 persons	12 per 1000 persons (6 to 24 per 1000 persons)	0 (6 fewer to 12 more)	Low
**Cardiovascular death**	Metformin	Tirzepatide	0.95 (0.27 to 3.32)	13 per 1000 persons	12 per 1000 persons (4 to 42 per 1000 persons)	1 fewer (9 fewer to 29 more)	Low
**Cardiovascular death**	Non-steroidal MRAs	SGLT2 inhibitors	1.01 (0.85 to 1.21)	11 per 1000 persons	11 per 1000 persons (9 to 13 per 1000 persons)	0 (2 fewer to 2 more)	Moderate
**Cardiovascular death**	Non-steroidal MRAs	Standard treatments	0.88 (0.75 to 1.02)	13 per 1000 persons	11 per 1000 persons (10 to 13 per 1000 persons)	2 fewer (3 fewer to 0)	Moderate
**Cardiovascular death**	Non-steroidal MRAs	Sulfonylureas	0.87 (0.67 to 1.12)	13 per 1000 persons	11 per 1000 persons (9 to 15 per 1000 persons)	2 fewer (4 fewer to 2 more)	Moderate
**Cardiovascular death**	Non-steroidal MRAs	Thiazolidinediones	0.94 (0.74 to 1.20)	12 per 1000 persons	11 per 1000 persons (9 to 14 per 1000 persons)	1 fewer (3 fewer to 2 more)	Moderate
**Cardiovascular death**	Non-steroidal MRAs	Tirzepatide	0.88 (0.30 to 2.55)	13 per 1000 persons	11 per 1000 persons (4 to 32 per 1000 persons)	2 fewer (9 fewer to 19 more)	Low
**Cardiovascular death**	SGLT2 inhibitors	Standard treatments	0.86 (0.80 to 0.94)	13 per 1000 persons	11 per 1000 persons (10 to 12 per 1000 persons)	2 fewer (3 fewer to 1 fewer)	High

Outcome	Intervention	Comparator	Odds ratio	Baseline risk	Risk if treated	Risk difference	Certainty
**Cardiovascular death**	SGLT2 inhibitors	Sulfonylureas	0.86 (0.69 to 1.06)	13 per 1000 persons	11 per 1000 persons (9 to 14 per 1000 persons)	2 fewer (4 fewer to 1 more)	Moderate
**Cardiovascular death**	SGLT2 inhibitors	Thiazolidinediones	0.93 (0.76 to 1.14)	12 per 1000 persons	11 per 1000 persons (9 to 14 per 1000 persons)	1 fewer (3 fewer to 2 more)	Moderate
**Cardiovascular death**	SGLT2 inhibitors	Tirzepatide	0.87 (0.30 to 2.49)	13 per 1000 persons	11 per 1000 persons (4 to 32 per 1000 persons)	2 fewer (9 fewer to 19 more)	Low
**Cardiovascular death**	Sulfonylureas	Standard treatments	1.01 (0.83 to 1.23)	13 per 1000 persons	13 per 1000 persons (11 to 16 per 1000 persons)	0 (2 fewer to 3 more)	Moderate
**Cardiovascular death**	Sulfonylureas	Thiazolidinediones	1.09 (0.84 to 1.41)	12 per 1000 persons	13 per 1000 persons (10 to 17 per 1000 persons)	1 more (2 fewer to 5 more)	Moderate
**Cardiovascular death**	Sulfonylureas	Tirzepatide	1.01 (0.35 to 2.93)	13 per 1000 persons	13 per 1000 persons (5 to 37 per 1000 persons)	0 (8 fewer to 24 more)	Low
**Cardiovascular death**	Thiazolidinediones	Standard treatments	0.93 (0.77 to 1.12)	13 per 1000 persons	12 per 1000 persons (10 to 15 per 1000 persons)	1 fewer (3 fewer to 2 more)	Moderate
**Cardiovascular death**	Thiazolidinediones	Tirzepatide	0.93 (0.32 to 2.71)	13 per 1000 persons	12 per 1000 persons (4 to 34 per 1000 persons)	1 fewer (9 fewer to 21 more)	Low
**Cardiovascular death**	Tirzepatide	Standard treatments	1.00 (0.35 to 2.85)	13 per 1000 persons	13 per 1000 persons (5 to 36 per 1000 persons)	0 (8 fewer to 23 more)	Low
**Cardiovascular death**	Basal insulin	Alpha-glucosidase inhibitors	1.30 (0.26 to 6.67)	13 per 1000 persons	17 per 1000 persons (3 to 81 per 1000 persons)	4 more (10 fewer to 68 more)	Low
**Cardiovascular death**	Basal-Bolus insulin	Alpha-glucosidase inhibitors	2.22 (0.14 to 33.33)	13 per 1000 persons	28 per 1000 persons (2 to 305 per 1000 persons)	15 more (11 fewer to 292 more)	Very low
**Cardiovascular death**	Bolus insulin	Alpha-glucosidase inhibitors	1.06 (0.07 to 16.67)	13 per 1000 persons	14 per 1000 persons (1 to 180 per 1000 persons)	1 more (12 fewer to 167 more)	Very low
**Cardiovascular death**	DPP-4 inhibitors	Alpha-glucosidase inhibitors	1.01 (0.21 to 4.76)	13 per 1000 persons	13 per 1000 persons (3 to 59 per 1000 persons)	0 (10 fewer to 46 more)	Low
**Cardiovascular death**	GLP-1 receptor agonists	Alpha-glucosidase inhibitors	0.88 (0.19 to 4.17)	13 per 1000 persons	11 per 1000 persons (2 to 52 per 1000 persons)	2 fewer (11 fewer to 39 more)	Very low
**Cardiovascular death**	Meglitinides	Alpha-glucosidase inhibitors	0.65 (0.06 to 6.67)	13 per 1000 persons	8 per 1000 persons (1 to 81 per 1000 persons)	5 fewer (12 fewer to 68 more)	Very low

Outcome	Intervention	Comparator	Odds ratio	Baseline risk	Risk if treated	Risk difference	Certainty
**Cardiovascular death**	Metformin	Alpha-glucosidase inhibitors	0.95 (0.18 to 5.26)	13 per 1000 persons	12 per 1000 persons (2 to 65 per 1000 persons)	1 fewer (11 fewer to 52 more)	Very low
**Cardiovascular death**	Non-steroidal MRAs	Alpha-glucosidase inhibitors	0.88 (0.19 to 4.17)	13 per 1000 persons	12 per 1000 persons (2 to 52 per 1000 persons)	1 fewer (11 fewer to 39 more)	Very low
**Cardiovascular death**	SGLT2 inhibitors	Alpha-glucosidase inhibitors	0.87 (0.18 to 4.17)	13 per 1000 persons	11 per 1000 persons (2 to 52 per 1000 persons)	2 fewer (11 fewer to 39 more)	Very low
**Cardiovascular death**	Standard treatments	Alpha-glucosidase inhibitors	1.01 (0.21 to 4.76)	13 per 1000 persons	13 per 1000 persons (3 to 59 per 1000 persons)	0 (10 fewer to 46 more)	Low
**Cardiovascular death**	Sulfonylureas	Alpha-glucosidase inhibitors	1.02 (0.21 to 4.76)	13 per 1000 persons	13 per 1000 persons (3 to 59 per 1000 persons)	0 (10 fewer to 46 more)	Low
**Cardiovascular death**	Thiazolidinediones	Alpha-glucosidase inhibitors	0.93 (0.20 to 4.55)	13 per 1000 persons	12 per 1000 persons (3 to 56 per 1000 persons)	1 fewer (10 fewer to 43 more)	Low
**Cardiovascular death**	Tirzepatide	Alpha-glucosidase inhibitors	1.00 (0.15 to 6.67)	13 per 1000 persons	13 per 1000 persons (2 to 81 per 1000 persons)	0 (11 fewer to 68 more)	Very low
**Cardiovascular death**	Basal-Bolus insulin	Basal insulin	1.72 (0.17 to 16.67)	17 per 1000 persons	29 per 1000 persons (3 to 224 per 1000 persons)	12 more (14 fewer to 207 more)	Very low
**Cardiovascular death**	Bolus insulin	Basal insulin	0.82 (0.08 to 7.69)	17 per 1000 persons	14 per 1000 persons (1 to 117 per 1000 persons)	3 fewer (16 fewer to 100 more)	Very low
**Cardiovascular death**	DPP-4 inhibitors	Basal insulin	0.78 (0.50 to 1.20)	17 per 1000 persons	13 per 1000 persons (9 to 20 per 1000 persons)	4 fewer (8 fewer to 3 more)	Moderate
**Cardiovascular death**	GLP-1 receptor agonists	Basal insulin	0.68 (0.44 to 1.05)	17 per 1000 persons	12 per 1000 persons (7 to 18 per 1000 persons)	5 fewer (10 fewer to 1 more)	Moderate
**Cardiovascular death**	Meglitinides	Basal insulin	0.50 (0.08 to 3.03)	17 per 1000 persons	9 per 1000 persons (1 to 50 per 1000 persons)	8 fewer (16 fewer to 33 more)	Very low
**Cardiovascular death**	Metformin	Basal insulin	0.74 (0.33 to 1.64)	17 per 1000 persons	13 per 1000 persons (6 to 28 per 1000 persons)	4 fewer (11 fewer to 11 more)	Very low
**Cardiovascular death**	Non-steroidal MRAs	Basal insulin	0.68 (0.43 to 1.09)	17 per 1000 persons	12 per 1000 persons (7 to 18 per 1000 persons)	5 fewer (10 fewer to 1 more)	Moderate
**Cardiovascular death**	SGLT2 inhibitors	Basal insulin	0.68 (0.43 to 1.05)	17 per 1000 persons	12 per 1000 persons (7 to 18 per 1000 persons)	5 fewer (10 fewer to 1 more)	Moderate

Outcome	Intervention	Comparator	Odds ratio	Baseline risk	Risk if treated	Risk difference	Certainty
**Cardiovascular death**	Standard treatments	Basal insulin	0.78 (0.50 to 1.20)	17 per 1000 persons	13 per 1000 persons (9 to 20 per 1000 persons)	4 fewer (8 fewer to 3 more)	Moderate
**Cardiovascular death**	Sulfonylureas	Basal insulin	0.79 (0.50 to 1.25)	17 per 1000 persons	13 per 1000 persons (9 to 21 per 1000 persons)	4 fewer (8 fewer to 4 more)	Moderate
**Cardiovascular death**	Thiazolidinediones	Basal insulin	0.72 (0.45 to 1.16)	17 per 1000 persons	12 per 1000 persons (8 to 20 per 1000 persons)	5 fewer (9 fewer to 3 more)	Moderate
**Cardiovascular death**	Tirzepatide	Basal insulin	0.78 (0.29 to 2.08)	17 per 1000 persons	13 per 1000 persons (5 to 35 per 1000 persons)	4 fewer (12 fewer to 18 more)	Low
**Cardiovascular death**	Bolus insulin	Basal-Bolus insulin	0.47 (0.02 to 12.50)	29 per 1000 persons	14 per 1000 persons (1 to 272 per 1000 persons)	15 fewer (28 fewer to 243 more)	Very low
**Cardiovascular death**	DPP-4 inhibitors	Basal-Bolus insulin	0.45 (0.05 to 4.35)	29 per 1000 persons	13 per 1000 persons (1 to 115 per 1000 persons)	16 fewer (28 fewer to 86 more)	Very low
**Cardiovascular death**	GLP-1 receptor agonists	Basal-Bolus insulin	0.39 (0.04 to 3.85)	29 per 1000 persons	12 per 1000 persons (1 to 103 per 1000 persons)	17 fewer (28 fewer to 74 more)	Very low
**Cardiovascular death**	Meglitinides	Basal-Bolus insulin	0.29 (0.02 to 5.00)	29 per 1000 persons	9 per 1000 persons (0 to 130 per 1000 persons)	20 fewer (29 fewer to 101 more)	Very low
**Cardiovascular death**	Metformin	Basal-Bolus insulin	0.43 (0.04 to 4.55)	29 per 1000 persons	13 per 1000 persons (1 to 120 per 1000 persons)	16 fewer (28 fewer to 91 more)	Very low
**Cardiovascular death**	Non-steroidal MRAs	Basal-Bolus insulin	0.39 (0.04 to 3.85)	29 per 1000 persons	12 per 1000 persons (1 to 103 per 1000 persons)	17 fewer (28 fewer to 74 more)	Very low
**Cardiovascular death**	SGLT2 inhibitors	Basal-Bolus insulin	0.39 (0.04 to 3.85)	29 per 1000 persons	11 per 1000 persons (1 to 103 per 1000 persons)	18 fewer (28 fewer to 74 more)	Very low
**Cardiovascular death**	Standard treatments	Basal-Bolus insulin	0.45 (0.05 to 4.35)	29 per 1000 persons	13 per 1000 persons (1 to 115 per 1000 persons)	16 fewer (28 fewer to 86 more)	Very low
**Cardiovascular death**	Sulfonylureas	Basal-Bolus insulin	0.45 (0.05 to 4.55)	29 per 1000 persons	13 per 1000 persons (1 to 120 per 1000 persons)	16 fewer (28 fewer to 91 more)	Very low
**Cardiovascular death**	Thiazolidinediones	Basal-Bolus insulin	0.42 (0.04 to 4.17)	29 per 1000 persons	12 per 1000 persons (1 to 111 per 1000 persons)	17 fewer (28 fewer to 82 more)	Very low
**Cardiovascular death**	Tirzepatide	Basal-Bolus insulin	0.45 (0.04 to 5.56)	29 per 1000 persons	13 per 1000 persons (1 to 142 per 1000 persons)	16 fewer (28 fewer to 113 more)	Very low

Outcome	Intervention	Comparator	Odds ratio	Baseline risk	Risk if treated	Risk difference	Certainty
**Cardiovascular death**	DPP-4 inhibitors	Bolus insulin	0.95 (0.10 to 9.09)	14 per 1000 persons	13 per 1000 persons (1 to 114 per 1000 persons)	1 fewer (13 fewer to 100 more)	Very low
**Cardiovascular death**	GLP-1 receptor agonists	Bolus insulin	0.83 (0.09 to 8.33)	14 per 1000 persons	12 per 1000 persons (1 to 106 per 1000 persons)	2 fewer (13 fewer to 92 more)	Very low
**Cardiovascular death**	Meglitinides	Bolus insulin	0.61 (0.03 to 11.11)	14 per 1000 persons	9 per 1000 persons (0 to 136 per 1000 persons)	5 fewer (14 fewer to 122 more)	Very low
**Cardiovascular death**	Metformin	Bolus insulin	0.90 (0.08 to 10.00)	14 per 1000 persons	13 per 1000 persons (1 to 124 per 1000 persons)	1 fewer (13 fewer to 110 more)	Very low
**Cardiovascular death**	Non-steroidal MRAs	Bolus insulin	0.83 (0.09 to 8.33)	14 per 1000 persons	12 per 1000 persons (1 to 106 per 1000 persons)	2 fewer (13 fewer to 92 more)	Very low
**Cardiovascular death**	SGLT2 inhibitors	Bolus insulin	0.82 (0.08 to 8.33)	14 per 1000 persons	12 per 1000 persons (1 to 106 per 1000 persons)	2 fewer (13 fewer to 92 more)	Very low
**Cardiovascular death**	Standard treatments	Bolus insulin	0.95 (0.10 to 9.09)	14 per 1000 persons	13 per 1000 persons (1 to 114 per 1000 persons)	1 fewer (13 fewer to 100 more)	Very low
**Cardiovascular death**	Sulfonylureas	Bolus insulin	0.96 (0.10 to 9.09)	14 per 1000 persons	13 per 1000 persons (1 to 114 per 1000 persons)	1 fewer (13 fewer to 100 more)	Very low
**Cardiovascular death**	Thiazolidinediones	Bolus insulin	0.88 (0.09 to 8.33)	14 per 1000 persons	12 per 1000 persons (1 to 106 per 1000 persons)	2 fewer (13 fewer to 92 more)	Very low
**Cardiovascular death**	Tirzepatide	Bolus insulin	0.94 (0.08 to 11.11)	14 per 1000 persons	13 per 1000 persons (1 to 136 per 1000 persons)	1 fewer (13 fewer to 122 more)	Very low
**Cardiovascular death**	GLP-1 receptor agonists	DPP-4 inhibitors	0.87 (0.78 to 0.98)	13 per 1000 persons	11 per 1000 persons (10 to 13 per 1000 persons)	2 fewer (3 fewer to 0)	Moderate
**Cardiovascular death**	Meglitinides	DPP-4 inhibitors	0.64 (0.11 to 3.70)	13 per 1000 persons	8 per 1000 persons (1 to 47 per 1000 persons)	5 fewer (12 fewer to 34 more)	Very low
**Cardiovascular death**	Metformin	DPP-4 inhibitors	0.95 (0.48 to 1.89)	13 per 1000 persons	12 per 1000 persons (6 to 24 per 1000 persons)	1 fewer (7 fewer to 11 more)	Low
**Cardiovascular death**	Non-steroidal MRAs	DPP-4 inhibitors	0.88 (0.74 to 1.04)	13 per 1000 persons	11 per 1000 persons (10 to 14 per 1000 persons)	2 fewer (3 fewer to 1 more)	Moderate
**Cardiovascular death**	SGLT2 inhibitors	DPP-4 inhibitors	0.86 (0.77 to 0.97)	13 per 1000 persons	11 per 1000 persons (10 to 13 per 1000 persons)	2 fewer (3 fewer to 0)	Moderate

Outcome	Intervention	Comparator	Odds ratio	Baseline risk	Risk if treated	Risk difference	Certainty
**Cardiovascular death**	Standard treatments	DPP-4 inhibitors	1.00 (0.92 to 1.09)	13 per 1000 persons	13 per 1000 persons (12 to 14 per 1000 persons)	0 (1 fewer to 1 more)	Moderate
**Cardiovascular death**	Sulfonylureas	DPP-4 inhibitors	1.01 (0.84 to 1.22)	13 per 1000 persons	13 per 1000 persons (11 to 16 per 1000 persons)	0 (2 fewer to 3 more)	High
**Cardiovascular death**	Thiazolidinediones	DPP-4 inhibitors	0.93 (0.76 to 1.14)	13 per 1000 persons	12 per 1000 persons (10 to 15 per 1000 persons)	1 fewer (3 fewer to 2 more)	Moderate
**Cardiovascular death**	Tirzepatide	DPP-4 inhibitors	1.00 (0.35 to 2.86)	13 per 1000 persons	13 per 1000 persons (5 to 36 per 1000 persons)	0 (8 fewer to 23 more)	Low
**Cardiovascular death**	Meglitinides	GLP-1 receptor agonists	0.74 (0.13 to 4.17)	11 per 1000 persons	8 per 1000 persons (1 to 44 per 1000 persons)	3 fewer (10 fewer to 33 more)	Low
**Cardiovascular death**	Metformin	GLP-1 receptor agonists	1.09 (0.55 to 2.17)	11 per 1000 persons	12 per 1000 persons (6 to 24 per 1000 persons)	1 more (5 fewer to 13 more)	Low
**Cardiovascular death**	Non-steroidal MRAs	GLP-1 receptor agonists	1.01 (0.85 to 1.19)	11 per 1000 persons	11 per 1000 persons (9 to 13 per 1000 persons)	0 (2 fewer to 2 more)	Moderate
**Cardiovascular death**	SGLT2 inhibitors	GLP-1 receptor agonists	0.99 (0.88 to 1.11)	11 per 1000 persons	11 per 1000 persons (10 to 12 per 1000 persons)	0 (1 fewer to 1 more)	Moderate
**Cardiovascular death**	Standard treatments	GLP-1 receptor agonists	1.15 (1.06 to 1.23)	11 per 1000 persons	13 per 1000 persons (12 to 14 per 1000 persons)	2 more (1 more to 3 more)	High
**Cardiovascular death**	Sulfonylureas	GLP-1 receptor agonists	1.16 (0.93 to 1.43)	11 per 1000 persons	13 per 1000 persons (10 to 16 per 1000 persons)	2 more (1 fewer to 5 more)	Moderate
**Cardiovascular death**	Thiazolidinediones	GLP-1 receptor agonists	1.06 (0.87 to 1.30)	11 per 1000 persons	12 per 1000 persons (10 to 14 per 1000 persons)	1 more (1 fewer to 3 more)	Moderate
**Cardiovascular death**	Tirzepatide	GLP-1 receptor agonists	1.14 (0.40 to 3.23)	11 per 1000 persons	12 per 1000 persons (4 to 35 per 1000 persons)	1 more (7 fewer to 24 more)	Low
**Cardiovascular death**	Metformin	Meglitinides	1.47 (0.24 to 9.09)	8 per 1000 persons	12 per 1000 persons (2 to 68 per 1000 persons)	4 more (6 fewer to 60 more)	Very low
**Cardiovascular death**	Non-steroidal MRAs	Meglitinides	1.37 (0.24 to 7.69)	8 per 1000 persons	11 per 1000 persons (2 to 58 per 1000 persons)	3 more (6 fewer to 50 more)	Very low
**Cardiovascular death**	SGLT2 inhibitors	Meglitinides	1.35 (0.23 to 7.69)	8 per 1000 persons	11 per 1000 persons (2 to 58 per 1000 persons)	3 more (6 fewer to 50 more)	Low

Outcome	Intervention	Comparator	Odds ratio	Baseline risk	Risk if treated	Risk difference	Certainty
**Cardiovascular death**	Standard treatments	Meglitinides	1.56 (0.27 to 9.09)	8 per 1000 persons	12 per 1000 persons (2 to 68 per 1000 persons)	4 more (6 fewer to 60 more)	Very low
**Cardiovascular death**	Sulfonylureas	Meglitinides	1.56 (0.27 to 9.09)	8 per 1000 persons	12 per 1000 persons (2 to 68 per 1000 persons)	4 more (6 fewer to 60 more)	Very low
**Cardiovascular death**	Thiazolidinediones	Meglitinides	1.45 (0.25 to 8.33)	8 per 1000 persons	12 per 1000 persons (2 to 63 per 1000 persons)	4 more (6 fewer to 55 more)	Very low
**Cardiovascular death**	Tirzepatide	Meglitinides	1.56 (0.20 to 12.50)	8 per 1000 persons	12 per 1000 persons (2 to 92 per 1000 persons)	4 more (6 fewer to 84 more)	Low
**Cardiovascular death**	Non-steroidal MRAs	Metformin	0.93 (0.46 to 1.85)	12 per 1000 persons	11 per 1000 persons (6 to 22 per 1000 persons)	1 fewer (6 fewer to 10 more)	Moderate
**Cardiovascular death**	SGLT2 inhibitors	Metformin	0.91 (0.46 to 1.82)	12 per 1000 persons	11 per 1000 persons (6 to 22 per 1000 persons)	1 fewer (6 fewer to 10 more)	Moderate
**Cardiovascular death**	Standard treatments	Metformin	1.05 (0.53 to 2.08)	12 per 1000 persons	13 per 1000 persons (6 to 25 per 1000 persons)	1 more (6 fewer to 13 more)	Low
**Cardiovascular death**	Sulfonylureas	Metformin	1.06 (0.54 to 2.08)	12 per 1000 persons	13 per 1000 persons (7 to 25 per 1000 persons)	1 more (5 fewer to 13 more)	Low
**Cardiovascular death**	Thiazolidinediones	Metformin	0.98 (0.49 to 1.96)	12 per 1000 persons	12 per 1000 persons (6 to 23 per 1000 persons)	0 (6 fewer to 11 more)	Low
**Cardiovascular death**	Tirzepatide	Metformin	1.05 (0.30 to 3.70)	12 per 1000 persons	13 per 1000 persons (4 to 43 per 1000 persons)	1 more (8 fewer to 31 more)	Low
**Cardiovascular death**	SGLT2 inhibitors	Non-steroidal MRAs	0.99 (0.83 to 1.18)	11 per 1000 persons	11 per 1000 persons (9 to 13 per 1000 persons)	0 (2 fewer to 2 more)	Moderate
**Cardiovascular death**	Standard treatments	Non-steroidal MRAs	1.14 (0.98 to 1.33)	11 per 1000 persons	12 per 1000 persons (11 to 15 per 1000 persons)	1 more (0 to 4 more)	Moderate
**Cardiovascular death**	Sulfonylureas	Non-steroidal MRAs	1.15 (0.89 to 1.49)	11 per 1000 persons	13 per 1000 persons (10 to 16 per 1000 persons)	2 more (1 fewer to 5 more)	Moderate
**Cardiovascular death**	Thiazolidinediones	Non-steroidal MRAs	1.06 (0.83 to 1.35)	11 per 1000 persons	12 per 1000 persons (9 to 15 per 1000 persons)	1 more (2 fewer to 4 more)	Moderate
**Cardiovascular death**	Tirzepatide	Non-steroidal MRAs	1.14 (0.39 to 3.33)	11 per 1000 persons	12 per 1000 persons (4 to 36 per 1000 persons)	1 more (7 fewer to 25 more)	Low

Outcome	Intervention	Comparator	Odds ratio	Baseline risk	Risk if treated	Risk difference	Certainty
**Cardiovascular death**	Standard treatments	SGLT2 inhibitors	1.16 (1.06 to 1.25)	11 per 1000 persons	13 per 1000 persons (12 to 14 per 1000 persons)	2 more (1 more to 3 more)	High
**Cardiovascular death**	Sulfonylureas	SGLT2 inhibitors	1.16 (0.94 to 1.45)	11 per 1000 persons	13 per 1000 persons (10 to 16 per 1000 persons)	2 more (1 fewer to 5 more)	Moderate
**Cardiovascular death**	Thiazolidinediones	SGLT2 inhibitors	1.08 (0.88 to 1.32)	11 per 1000 persons	12 per 1000 persons (10 to 14 per 1000 persons)	1 more (1 fewer to 3 more)	Moderate
**Cardiovascular death**	Tirzepatide	SGLT2 inhibitors	1.15 (0.40 to 3.33)	11 per 1000 persons	13 per 1000 persons (4 to 36 per 1000 persons)	2 more (7 fewer to 25 more)	Low
**Cardiovascular death**	Standard treatments	Sulfonylureas	0.99 (0.81 to 1.20)	13 per 1000 persons	13 per 1000 persons (11 to 16 per 1000 persons)	0 (2 fewer to 3 more)	Moderate
**Cardiovascular death**	Thiazolidinediones	Sulfonylureas	0.92 (0.71 to 1.19)	13 per 1000 persons	12 per 1000 persons (9 to 15 per 1000 persons)	1 fewer (4 fewer to 2 more)	Moderate
**Cardiovascular death**	Tirzepatide	Sulfonylureas	0.99 (0.34 to 2.86)	13 per 1000 persons	13 per 1000 persons (4 to 36 per 1000 persons)	0 (9 fewer to 23 more)	Low
**Cardiovascular death**	Standard treatments	Thiazolidinediones	1.08 (0.89 to 1.30)	12 per 1000 persons	13 per 1000 persons (11 to 16 per 1000 persons)	1 more (1 fewer to 4 more)	Moderate
**Cardiovascular death**	Tirzepatide	Thiazolidinediones	1.08 (0.37 to 3.12)	12 per 1000 persons	13 per 1000 persons (4 to 37 per 1000 persons)	1 more (8 fewer to 25 more)	Low
**Cardiovascular death**	Standard treatments	Tirzepatide	1.00 (0.35 to 2.86)	13 per 1000 persons	13 per 1000 persons (5 to 36 per 1000 persons)	0 (8 fewer to 23 more)	Low
**Non-fatal myocardial infarction**	Alpha-glucosidase inhibitors	Basal insulin	0.33 (0.05 to 2.28)	29 per 1000 persons	10 per 1000 persons (1 to 64 per 1000 persons)	19 fewer (28 fewer to 35 more)	Very low
**Non-fatal myocardial infarction**	Alpha-glucosidase inhibitors	Basal-Bolus insulin	1.00 (0.05 to 18.12)	10 per 1000 persons	10 per 1000 persons (1 to 155 per 1000 persons)	0 (9 fewer to 145 more)	Very low
**Non-fatal myocardial infarction**	Alpha-glucosidase inhibitors	Bolus insulin	0.28 (0.03 to 2.21)	35 per 1000 persons	10 per 1000 persons (1 to 74 per 1000 persons)	25 fewer (34 fewer to 39 more)	Very low
**Non-fatal myocardial infarction**	Alpha-glucosidase inhibitors	DPP-4 inhibitors	0.32 (0.06 to 1.90)	30 per 1000 persons	10 per 1000 persons (2 to 56 per 1000 persons)	20 fewer (28 fewer to 26 more)	Very low
**Non-fatal myocardial infarction**	Alpha-glucosidase inhibitors	GLP-1 receptor agonists	0.36 (0.06 to 2.10)	27 per 1000 persons	10 per 1000 persons (2 to 55 per 1000 persons)	17 fewer (25 fewer to 28 more)	Very low

Outcome	Intervention	Comparator	Odds ratio	Baseline risk	Risk if treated	Risk difference	Certainty
**Non-fatal myocardial infarction**	Alpha-glucosidase inhibitors	Meglitinides	1.16 (0.10 to 12.83)	9 per 1000 persons	10 per 1000 persons (1 to 104 per 1000 persons)	1 more (8 fewer to 95 more)	Very low
**Non-fatal myocardial infarction**	Alpha-glucosidase inhibitors	Metformin	0.38 (0.06 to 2.27)	26 per 1000 persons	10 per 1000 persons (2 to 57 per 1000 persons)	16 fewer (24 fewer to 31 more)	Very low
**Non-fatal myocardial infarction**	Alpha-glucosidase inhibitors	Non-steroidal MRAs	0.36 (0.06 to 2.13)	27 per 1000 persons	10 per 1000 persons (2 to 56 per 1000 persons)	17 fewer (25 fewer to 29 more)	Very low
**Non-fatal myocardial infarction**	Alpha-glucosidase inhibitors	SGLT2 inhibitors	0.36 (0.06 to 2.14)	27 per 1000 persons	10 per 1000 persons (2 to 56 per 1000 persons)	17 fewer (25 fewer to 29 more)	Very low
**Non-fatal myocardial infarction**	Alpha-glucosidase inhibitors	Standard treatments	0.33 (0.06 to 1.92)	30 per 1000 persons	10 per 1000 persons (2 to 56 per 1000 persons)	20 fewer (28 fewer to 26 more)	Very low
**Non-fatal myocardial infarction**	Alpha-glucosidase inhibitors	Sulfonylureas	0.33 (0.06 to 1.92)	30 per 1000 persons	10 per 1000 persons (2 to 56 per 1000 persons)	20 fewer (28 fewer to 26 more)	Very low
**Non-fatal myocardial infarction**	Alpha-glucosidase inhibitors	Thiazolidinediones	0.34 (0.06 to 2.00)	29 per 1000 persons	10 per 1000 persons (2 to 56 per 1000 persons)	19 fewer (27 fewer to 27 more)	Very low
**Non-fatal myocardial infarction**	Alpha-glucosidase inhibitors	Tirzepatide	0.48 (0.03 to 7.88)	21 per 1000 persons	10 per 1000 persons (1 to 145 per 1000 persons)	11 fewer (20 fewer to 124 more)	Very low
**Non-fatal myocardial infarction**	Basal insulin	Basal-Bolus insulin	2.98 (0.30 to 29.67)	10 per 1000 persons	29 per 1000 persons (3 to 231 per 1000 persons)	19 more (7 fewer to 221 more)	Very low
**Non-fatal myocardial infarction**	Basal insulin	Bolus insulin	0.83 (0.28 to 2.50)	35 per 1000 persons	29 per 1000 persons (10 to 83 per 1000 persons)	6 fewer (25 fewer to 48 more)	Very low
**Non-fatal myocardial infarction**	Basal insulin	DPP-4 inhibitors	0.97 (0.46 to 2.05)	30 per 1000 persons	29 per 1000 persons (14 to 60 per 1000 persons)	1 fewer (16 fewer to 30 more)	Very low
**Non-fatal myocardial infarction**	Basal insulin	GLP-1 receptor agonists	1.07 (0.51 to 2.25)	27 per 1000 persons	29 per 1000 persons (14 to 59 per 1000 persons)	2 more (13 fewer to 32 more)	Very low
**Non-fatal myocardial infarction**	Basal insulin	Meglitinides	3.47 (0.53 to 22.89)	9 per 1000 persons	31 per 1000 persons (5 to 172 per 1000 persons)	22 more (4 fewer to 163 more)	Low
**Non-fatal myocardial infarction**	Basal insulin	Metformin	1.14 (0.52 to 2.49)	26 per 1000 persons	30 per 1000 persons (14 to 62 per 1000 persons)	4 more (12 fewer to 36 more)	Very low
**Non-fatal myocardial infarction**	Basal insulin	Non-steroidal MRAs	1.08 (0.50 to 2.33)	27 per 1000 persons	29 per 1000 persons (14 to 61 per 1000 persons)	2 more (13 fewer to 34 more)	Very low

Outcome	Intervention	Comparator	Odds ratio	Baseline risk	Risk if treated	Risk difference	Certainty
**Non-fatal myocardial infarction**	Basal insulin	SGLT2 inhibitors	1.09 (0.52 to 2.31)	27 per 1000 persons	29 per 1000 persons (14 to 60 per 1000 persons)	2 more (13 fewer to 33 more)	Very low
**Non-fatal myocardial infarction**	Basal insulin	Standard treatments	0.98 (0.47 to 2.06)	30 per 1000 persons	29 per 1000 persons (14 to 60 per 1000 persons)	1 fewer (16 fewer to 30 more)	Very low
**Non-fatal myocardial infarction**	Basal insulin	Sulfonylureas	0.98 (0.45 to 2.10)	30 per 1000 persons	29 per 1000 persons (14 to 61 per 1000 persons)	1 fewer (16 fewer to 31 more)	Very low
**Non-fatal myocardial infarction**	Basal insulin	Thiazolidinediones	1.01 (0.47 to 2.17)	29 per 1000 persons	29 per 1000 persons (14 to 61 per 1000 persons)	0 (15 fewer to 32 more)	Very low
**Non-fatal myocardial infarction**	Basal insulin	Tirzepatide	1.42 (0.14 to 14.22)	21 per 1000 persons	30 per 1000 persons (3 to 234 per 1000 persons)	9 more (18 fewer to 213 more)	Very low
**Non-fatal myocardial infarction**	Basal-Bolus insulin	Bolus insulin	0.28 (0.02 to 3.35)	35 per 1000 persons	10 per 1000 persons (1 to 108 per 1000 persons)	25 fewer (34 fewer to 73 more)	Very low
**Non-fatal myocardial infarction**	Basal-Bolus insulin	DPP-4 inhibitors	0.32 (0.03 to 3.24)	30 per 1000 persons	10 per 1000 persons (1 to 91 per 1000 persons)	20 fewer (29 fewer to 61 more)	Very low
**Non-fatal myocardial infarction**	Basal-Bolus insulin	GLP-1 receptor agonists	0.36 (0.04 to 3.57)	27 per 1000 persons	10 per 1000 persons (1 to 90 per 1000 persons)	17 fewer (26 fewer to 63 more)	Very low
**Non-fatal myocardial infarction**	Basal-Bolus insulin	Meglitinides	1.16 (0.07 to 20.69)	9 per 1000 persons	10 per 1000 persons (1 to 158 per 1000 persons)	1 more (8 fewer to 149 more)	Very low
**Non-fatal myocardial infarction**	Basal-Bolus insulin	Metformin	0.38 (0.04 to 3.85)	26 per 1000 persons	10 per 1000 persons (1 to 93 per 1000 persons)	16 fewer (25 fewer to 67 more)	Very low
**Non-fatal myocardial infarction**	Basal-Bolus insulin	Non-steroidal MRAs	0.36 (0.04 to 3.62)	27 per 1000 persons	10 per 1000 persons (1 to 91 per 1000 persons)	17 fewer (26 fewer to 64 more)	Very low
**Non-fatal myocardial infarction**	Basal-Bolus insulin	SGLT2 inhibitors	0.37 (0.04 to 3.65)	27 per 1000 persons	10 per 1000 persons (1 to 92 per 1000 persons)	17 fewer (26 fewer to 65 more)	Very low
**Non-fatal myocardial infarction**	Basal-Bolus insulin	Standard treatments	0.33 (0.03 to 3.27)	30 per 1000 persons	10 per 1000 persons (1 to 92 per 1000 persons)	20 fewer (29 fewer to 62 more)	Very low
**Non-fatal myocardial infarction**	Basal-Bolus insulin	Sulfonylureas	0.33 (0.03 to 3.28)	30 per 1000 persons	10 per 1000 persons (1 to 92 per 1000 persons)	20 fewer (29 fewer to 62 more)	Very low
**Non-fatal myocardial infarction**	Basal-Bolus insulin	Thiazolidinediones	0.34 (0.03 to 3.40)	29 per 1000 persons	10 per 1000 persons (1 to 92 per 1000 persons)	19 fewer (28 fewer to 63 more)	Very low

Outcome	Intervention	Comparator	Odds ratio	Baseline risk	Risk if treated	Risk difference	Certainty
**Non-fatal myocardial infarction**	Basal-Bolus insulin	Tirzepatide	0.48 (0.02 to 11.31)	21 per 1000 persons	10 per 1000 persons (0 to 195 per 1000 persons)	11 fewer (21 fewer to 174 more)	Very low
**Non-fatal myocardial infarction**	Bolus insulin	DPP-4 inhibitors	1.17 (0.39 to 3.48)	30 per 1000 persons	35 per 1000 persons (12 to 97 per 1000 persons)	5 more (18 fewer to 67 more)	Very low
**Non-fatal myocardial infarction**	Bolus insulin	GLP-1 receptor agonists	1.29 (0.44 to 3.82)	27 per 1000 persons	35 per 1000 persons (12 to 96 per 1000 persons)	8 more (15 fewer to 69 more)	Very low
**Non-fatal myocardial infarction**	Bolus insulin	Meglitinides	4.19 (0.54 to 32.39)	9 per 1000 persons	37 per 1000 persons (5 to 227 per 1000 persons)	28 more (4 fewer to 218 more)	Very low
**Non-fatal myocardial infarction**	Bolus insulin	Metformin	1.37 (0.45 to 4.18)	26 per 1000 persons	35 per 1000 persons (12 to 100 per 1000 persons)	9 more (14 fewer to 74 more)	Very low
**Non-fatal myocardial infarction**	Bolus insulin	Non-steroidal MRAs	1.30 (0.43 to 3.92)	27 per 1000 persons	35 per 1000 persons (12 to 98 per 1000 persons)	8 more (15 fewer to 71 more)	Very low
**Non-fatal myocardial infarction**	Bolus insulin	SGLT2 inhibitors	1.32 (0.44 to 3.92)	27 per 1000 persons	35 per 1000 persons (12 to 98 per 1000 persons)	8 more (15 fewer to 71 more)	Very low
**Non-fatal myocardial infarction**	Bolus insulin	Standard treatments	1.18 (0.40 to 3.50)	30 per 1000 persons	35 per 1000 persons (12 to 98 per 1000 persons)	5 more (18 fewer to 68 more)	Very low
**Non-fatal myocardial infarction**	Bolus insulin	Sulfonylureas	1.18 (0.39 to 3.55)	30 per 1000 persons	35 per 1000 persons (12 to 99 per 1000 persons)	5 more (18 fewer to 69 more)	Very low
**Non-fatal myocardial infarction**	Bolus insulin	Thiazolidinediones	1.22 (0.41 to 3.67)	29 per 1000 persons	35 per 1000 persons (12 to 99 per 1000 persons)	6 more (17 fewer to 70 more)	Very low
**Non-fatal myocardial infarction**	Bolus insulin	Tirzepatide	1.72 (0.15 to 19.58)	21 per 1000 persons	36 per 1000 persons (3 to 296 per 1000 persons)	15 more (18 fewer to 275 more)	Very low
**Non-fatal myocardial infarction**	DPP-4 inhibitors	GLP-1 receptor agonists	1.11 (0.99 to 1.24)	27 per 1000 persons	30 per 1000 persons (27 to 33 per 1000 persons)	3 more (0 to 6 more)	Moderate
**Non-fatal myocardial infarction**	DPP-4 inhibitors	Meglitinides	3.58 (0.63 to 20.28)	9 per 1000 persons	31 per 1000 persons (6 to 156 per 1000 persons)	22 more (3 fewer to 147 more)	Low
**Non-fatal myocardial infarction**	DPP-4 inhibitors	Metformin	1.18 (0.91 to 1.51)	26 per 1000 persons	31 per 1000 persons (24 to 39 per 1000 persons)	5 more (2 fewer to 13 more)	Low
**Non-fatal myocardial infarction**	DPP-4 inhibitors	Non-steroidal MRAs	1.11 (0.88 to 1.39)	27 per 1000 persons	30 per 1000 persons (24 to 37 per 1000 persons)	3 more (3 fewer to 10 more)	Moderate

Outcome	Intervention	Comparator	Odds ratio	Baseline risk	Risk if treated	Risk difference	Certainty
**Non-fatal myocardial infarction**	DPP-4 inhibitors	SGLT2 inhibitors	1.13 (0.99 to 1.28)	27 per 1000 persons	30 per 1000 persons (27 to 34 per 1000 persons)	3 more (0 to 7 more)	Moderate
**Non-fatal myocardial infarction**	DPP-4 inhibitors	Standard treatments	1.01 (0.92 to 1.11)	30 per 1000 persons	30 per 1000 persons (28 to 33 per 1000 persons)	0 (2 fewer to 3 more)	Moderate
**Non-fatal myocardial infarction**	DPP-4 inhibitors	Sulfonylureas	1.01 (0.84 to 1.21)	30 per 1000 persons	30 per 1000 persons (25 to 36 per 1000 persons)	0 (5 fewer to 6 more)	High
**Non-fatal myocardial infarction**	DPP-4 inhibitors	Thiazolidinediones	1.04 (0.87 to 1.26)	29 per 1000 persons	30 per 1000 persons (25 to 36 per 1000 persons)	1 more (4 fewer to 7 more)	Moderate
**Non-fatal myocardial infarction**	DPP-4 inhibitors	Tirzepatide	1.47 (0.17 to 13.01)	21 per 1000 persons	31 per 1000 persons (4 to 218 per 1000 persons)	10 more (17 fewer to 197 more)	Very low
**Non-fatal myocardial infarction**	GLP-1 receptor agonists	Meglitinides	3.24 (0.57 to 18.37)	9 per 1000 persons	29 per 1000 persons (5 to 143 per 1000 persons)	20 more (4 fewer to 134 more)	Low
**Non-fatal myocardial infarction**	GLP-1 receptor agonists	Metformin	1.06 (0.83 to 1.36)	26 per 1000 persons	28 per 1000 persons (22 to 35 per 1000 persons)	2 more (4 fewer to 9 more)	Moderate
**Non-fatal myocardial infarction**	GLP-1 receptor agonists	Non-steroidal MRAs	1.00 (0.80 to 1.25)	27 per 1000 persons	27 per 1000 persons (22 to 34 per 1000 persons)	0 (5 fewer to 7 more)	Moderate
**Non-fatal myocardial infarction**	GLP-1 receptor agonists	SGLT2 inhibitors	1.02 (0.91 to 1.14)	27 per 1000 persons	28 per 1000 persons (25 to 31 per 1000 persons)	1 more (2 fewer to 4 more)	Moderate
**Non-fatal myocardial infarction**	GLP-1 receptor agonists	Standard treatments	0.91 (0.85 to 0.98)	30 per 1000 persons	27 per 1000 persons (26 to 29 per 1000 persons)	3 fewer (4 fewer to 1 fewer)	High
**Non-fatal myocardial infarction**	GLP-1 receptor agonists	Sulfonylureas	0.91 (0.74 to 1.12)	30 per 1000 persons	27 per 1000 persons (22 to 33 per 1000 persons)	3 fewer (8 fewer to 3 more)	Moderate
**Non-fatal myocardial infarction**	GLP-1 receptor agonists	Thiazolidinediones	0.95 (0.79 to 1.14)	29 per 1000 persons	28 per 1000 persons (23 to 33 per 1000 persons)	1 fewer (6 fewer to 4 more)	Moderate
**Non-fatal myocardial infarction**	GLP-1 receptor agonists	Tirzepatide	1.33 (0.15 to 11.74)	21 per 1000 persons	28 per 1000 persons (3 to 201 per 1000 persons)	7 more (18 fewer to 180 more)	Low
**Non-fatal myocardial infarction**	Meglitinides	Metformin	0.33 (0.06 to 1.89)	26 per 1000 persons	9 per 1000 persons (2 to 48 per 1000 persons)	17 fewer (24 fewer to 22 more)	Very low
**Non-fatal myocardial infarction**	Meglitinides	Non-steroidal MRAs	0.31 (0.05 to 1.78)	27 per 1000 persons	9 per 1000 persons (1 to 47 per 1000 persons)	18 fewer (26 fewer to 20 more)	Very low

Outcome	Intervention	Comparator	Odds ratio	Baseline risk	Risk if treated	Risk difference	Certainty
**Non-fatal myocardial infarction**	Meglitinides	SGLT2 inhibitors	0.31 (0.06 to 1.79)	27 per 1000 persons	9 per 1000 persons (2 to 47 per 1000 persons)	18 fewer (25 fewer to 20 more)	Very low
**Non-fatal myocardial infarction**	Meglitinides	Standard treatments	0.28 (0.05 to 1.60)	30 per 1000 persons	9 per 1000 persons (2 to 47 per 1000 persons)	21 fewer (28 fewer to 17 more)	Very low
**Non-fatal myocardial infarction**	Meglitinides	Sulfonylureas	0.28 (0.05 to 1.59)	30 per 1000 persons	9 per 1000 persons (2 to 47 per 1000 persons)	21 fewer (28 fewer to 17 more)	Very low
**Non-fatal myocardial infarction**	Meglitinides	Thiazolidinediones	0.29 (0.05 to 1.66)	29 per 1000 persons	9 per 1000 persons (1 to 47 per 1000 persons)	20 fewer (28 fewer to 18 more)	Very low
**Non-fatal myocardial infarction**	Meglitinides	Tirzepatide	0.41 (0.03 to 6.64)	21 per 1000 persons	9 per 1000 persons (1 to 125 per 1000 persons)	12 fewer (20 fewer to 104 more)	Very low
**Non-fatal myocardial infarction**	Metformin	Non-steroidal MRAs	0.94 (0.69 to 1.30)	27 per 1000 persons	25 per 1000 persons (19 to 35 per 1000 persons)	2 fewer (8 fewer to 8 more)	Moderate
**Non-fatal myocardial infarction**	Metformin	SGLT2 inhibitors	0.96 (0.74 to 1.24)	27 per 1000 persons	26 per 1000 persons (20 to 33 per 1000 persons)	1 fewer (7 fewer to 6 more)	Moderate
**Non-fatal myocardial infarction**	Metformin	Standard treatments	0.86 (0.68 to 1.09)	30 per 1000 persons	26 per 1000 persons (21 to 33 per 1000 persons)	4 fewer (9 fewer to 3 more)	Moderate
**Non-fatal myocardial infarction**	Metformin	Sulfonylureas	0.86 (0.64 to 1.14)	30 per 1000 persons	26 per 1000 persons (19 to 34 per 1000 persons)	4 fewer (11 fewer to 4 more)	Low
**Non-fatal myocardial infarction**	Metformin	Thiazolidinediones	0.89 (0.67 to 1.17)	29 per 1000 persons	26 per 1000 persons (20 to 34 per 1000 persons)	3 fewer (9 fewer to 5 more)	Moderate
**Non-fatal myocardial infarction**	Metformin	Tirzepatide	1.25 (0.14 to 11.19)	21 per 1000 persons	26 per 1000 persons (3 to 194 per 1000 persons)	5 more (18 fewer to 173 more)	Very low
**Non-fatal myocardial infarction**	Non-steroidal MRAs	SGLT2 inhibitors	1.02 (0.81 to 1.27)	27 per 1000 persons	28 per 1000 persons (22 to 34 per 1000 persons)	1 more (5 fewer to 7 more)	Moderate
**Non-fatal myocardial infarction**	Non-steroidal MRAs	Standard treatments	0.91 (0.74 to 1.12)	30 per 1000 persons	27 per 1000 persons (22 to 33 per 1000 persons)	3 fewer (8 fewer to 3 more)	Moderate
**Non-fatal myocardial infarction**	Non-steroidal MRAs	Sulfonylureas	0.91 (0.68 to 1.21)	30 per 1000 persons	27 per 1000 persons (21 to 36 per 1000 persons)	3 fewer (9 fewer to 6 more)	Moderate
**Non-fatal myocardial infarction**	Non-steroidal MRAs	Thiazolidinediones	0.94 (0.72 to 1.24)	29 per 1000 persons	27 per 1000 persons (21 to 36 per 1000 persons)	2 fewer (8 fewer to 7 more)	Moderate

Outcome	Intervention	Comparator	Odds ratio	Baseline risk	Risk if treated	Risk difference	Certainty
**Non-fatal myocardial infarction**	Non-steroidal MRAs	Tirzepatide	1.32 (0.15 to 11.83)	21 per 1000 persons	28 per 1000 persons (3 to 202 per 1000 persons)	7 more (18 fewer to 181 more)	Very low
**Non-fatal myocardial infarction**	SGLT2 inhibitors	Standard treatments	0.90 (0.82 to 0.98)	30 per 1000 persons	27 per 1000 persons (25 to 29 per 1000 persons)	3 fewer (5 fewer to 1 fewer)	High
**Non-fatal myocardial infarction**	SGLT2 inhibitors	Sulfonylureas	0.89 (0.72 to 1.10)	30 per 1000 persons	27 per 1000 persons (22 to 33 per 1000 persons)	3 fewer (8 fewer to 3 more)	Moderate
**Non-fatal myocardial infarction**	SGLT2 inhibitors	Thiazolidinediones	0.93 (0.76 to 1.12)	29 per 1000 persons	27 per 1000 persons (22 to 32 per 1000 persons)	2 fewer (7 fewer to 3 more)	Moderate
**Non-fatal myocardial infarction**	SGLT2 inhibitors	Tirzepatide	1.30 (0.15 to 11.55)	21 per 1000 persons	27 per 1000 persons (3 to 199 per 1000 persons)	6 more (18 fewer to 178 more)	Very low
**Non-fatal myocardial infarction**	Sulfonylureas	Standard treatments	1.00 (0.83 to 1.22)	30 per 1000 persons	30 per 1000 persons (25 to 36 per 1000 persons)	0 (5 fewer to 6 more)	Moderate
**Non-fatal myocardial infarction**	Sulfonylureas	Thiazolidinediones	1.04 (0.83 to 1.30)	29 per 1000 persons	30 per 1000 persons (24 to 37 per 1000 persons)	1 more (5 fewer to 8 more)	Moderate
**Non-fatal myocardial infarction**	Sulfonylureas	Tirzepatide	1.46 (0.16 to 13.00)	21 per 1000 persons	30 per 1000 persons (3 to 218 per 1000 persons)	9 more (18 fewer to 197 more)	Very low
**Non-fatal myocardial infarction**	Thiazolidinediones	Standard treatments	0.97 (0.81 to 1.15)	30 per 1000 persons	29 per 1000 persons (24 to 34 per 1000 persons)	1 fewer (6 fewer to 4 more)	Moderate
**Non-fatal myocardial infarction**	Thiazolidinediones	Tirzepatide	1.40 (0.16 to 12.51)	21 per 1000 persons	29 per 1000 persons (3 to 212 per 1000 persons)	8 more (18 fewer to 191 more)	Very low
**Non-fatal myocardial infarction**	Tirzepatide	Standard treatments	0.69 (0.08 to 6.10)	30 per 1000 persons	21 per 1000 persons (2 to 159 per 1000 persons)	9 fewer (28 fewer to 129 more)	Very low
**Non-fatal myocardial infarction**	Basal insulin	Alpha-glucosidase inhibitors	3.03 (0.44 to 20.00)	10 per 1000 persons	30 per 1000 persons (4 to 168 per 1000 persons)	20 more (6 fewer to 158 more)	Low
**Non-fatal myocardial infarction**	Basal-Bolus insulin	Alpha-glucosidase inhibitors	1.00 (0.06 to 20.00)	10 per 1000 persons	10 per 1000 persons (1 to 168 per 1000 persons)	0 (9 fewer to 158 more)	Very low
**Non-fatal myocardial infarction**	Bolus insulin	Alpha-glucosidase inhibitors	3.57 (0.45 to 33.33)	10 per 1000 persons	35 per 1000 persons (5 to 252 per 1000 persons)	25 more (5 fewer to 242 more)	Very low
**Non-fatal myocardial infarction**	DPP-4 inhibitors	Alpha-glucosidase inhibitors	3.12 (0.53 to 16.67)	10 per 1000 persons	31 per 1000 persons (5 to 144 per 1000 persons)	21 more (5 fewer to 134 more)	Very low

Outcome	Intervention	Comparator	Odds ratio	Baseline risk	Risk if treated	Risk difference	Certainty
**Non-fatal myocardial infarction**	GLP-1 receptor agonists	Alpha-glucosidase inhibitors	2.78 (0.48 to 16.67)	10 per 1000 persons	27 per 1000 persons (5 to 144 per 1000 persons)	17 more (5 fewer to 134 more)	Low
**Non-fatal myocardial infarction**	Meglitinides	Alpha-glucosidase inhibitors	0.86 (0.08 to 10.00)	10 per 1000 persons	9 per 1000 persons (1 to 92 per 1000 persons)	1 fewer (9 fewer to 82 more)	Very low
**Non-fatal myocardial infarction**	Metformin	Alpha-glucosidase inhibitors	2.63 (0.44 to 16.67)	10 per 1000 persons	26 per 1000 persons (4 to 144 per 1000 persons)	16 more (6 fewer to 134 more)	Low
**Non-fatal myocardial infarction**	Non-steroidal MRAs	Alpha-glucosidase inhibitors	2.78 (0.47 to 16.67)	10 per 1000 persons	27 per 1000 persons (5 to 144 per 1000 persons)	17 more (5 fewer to 134 more)	Very low
**Non-fatal myocardial infarction**	SGLT2 inhibitors	Alpha-glucosidase inhibitors	2.78 (0.47 to 16.67)	10 per 1000 persons	27 per 1000 persons (5 to 144 per 1000 persons)	17 more (5 fewer to 134 more)	Low
**Non-fatal myocardial infarction**	Standard treatments	Alpha-glucosidase inhibitors	3.03 (0.52 to 16.67)	10 per 1000 persons	30 per 1000 persons (5 to 144 per 1000 persons)	20 more (5 fewer to 134 more)	Low
**Non-fatal myocardial infarction**	Sulfonylureas	Alpha-glucosidase inhibitors	3.03 (0.52 to 16.67)	10 per 1000 persons	30 per 1000 persons (5 to 144 per 1000 persons)	20 more (5 fewer to 134 more)	Low
**Non-fatal myocardial infarction**	Thiazolidinediones	Alpha-glucosidase inhibitors	2.94 (0.50 to 16.67)	10 per 1000 persons	29 per 1000 persons (5 to 144 per 1000 persons)	19 more (5 fewer to 134 more)	Very low
**Non-fatal myocardial infarction**	Tirzepatide	Alpha-glucosidase inhibitors	2.08 (0.13 to 33.33)	10 per 1000 persons	21 per 1000 persons (1 to 252 per 1000 persons)	11 more (9 fewer to 242 more)	Low
**Non-fatal myocardial infarction**	Basal-Bolus insulin	Basal insulin	0.34 (0.03 to 3.33)	29 per 1000 persons	10 per 1000 persons (1 to 91 per 1000 persons)	19 fewer (28 fewer to 62 more)	Very low
**Non-fatal myocardial infarction**	Bolus insulin	Basal insulin	1.20 (0.40 to 3.57)	29 per 1000 persons	35 per 1000 persons (12 to 96 per 1000 persons)	6 more (17 fewer to 67 more)	Very low
**Non-fatal myocardial infarction**	DPP-4 inhibitors	Basal insulin	1.03 (0.49 to 2.17)	29 per 1000 persons	30 per 1000 persons (14 to 61 per 1000 persons)	1 more (15 fewer to 32 more)	Very low
**Non-fatal myocardial infarction**	GLP-1 receptor agonists	Basal insulin	0.93 (0.44 to 1.96)	29 per 1000 persons	27 per 1000 persons (13 to 55 per 1000 persons)	2 fewer (16 fewer to 26 more)	Very low
**Non-fatal myocardial infarction**	Meglitinides	Basal insulin	0.29 (0.04 to 1.89)	29 per 1000 persons	9 per 1000 persons (1 to 53 per 1000 persons)	20 fewer (28 fewer to 24 more)	Very low
**Non-fatal myocardial infarction**	Metformin	Basal insulin	0.88 (0.40 to 1.92)	29 per 1000 persons	26 per 1000 persons (12 to 54 per 1000 persons)	3 fewer (17 fewer to 25 more)	Very low

Outcome	Intervention	Comparator	Odds ratio	Baseline risk	Risk if treated	Risk difference	Certainty
**Non-fatal myocardial infarction**	Non-steroidal MRAs	Basal insulin	0.93 (0.43 to 2.00)	29 per 1000 persons	27 per 1000 persons (13 to 56 per 1000 persons)	2 fewer (16 fewer to 27 more)	Very low
**Non-fatal myocardial infarction**	SGLT2 inhibitors	Basal insulin	0.92 (0.43 to 1.92)	29 per 1000 persons	27 per 1000 persons (13 to 54 per 1000 persons)	2 fewer (16 fewer to 25 more)	Very low
**Non-fatal myocardial infarction**	Standard treatments	Basal insulin	1.02 (0.49 to 2.13)	29 per 1000 persons	30 per 1000 persons (14 to 60 per 1000 persons)	1 more (15 fewer to 31 more)	Very low
**Non-fatal myocardial infarction**	Sulfonylureas	Basal insulin	1.02 (0.48 to 2.22)	29 per 1000 persons	30 per 1000 persons (14 to 62 per 1000 persons)	1 more (15 fewer to 33 more)	Very low
**Non-fatal myocardial infarction**	Thiazolidinediones	Basal insulin	0.99 (0.46 to 2.13)	29 per 1000 persons	29 per 1000 persons (14 to 60 per 1000 persons)	0 (15 fewer to 31 more)	Very low
**Non-fatal myocardial infarction**	Tirzepatide	Basal insulin	0.70 (0.07 to 7.14)	29 per 1000 persons	21 per 1000 persons (2 to 176 per 1000 persons)	8 fewer (27 fewer to 147 more)	Very low
**Non-fatal myocardial infarction**	Bolus insulin	Basal-Bolus insulin	3.57 (0.30 to 50.00)	10 per 1000 persons	35 per 1000 persons (3 to 336 per 1000 persons)	25 more (7 fewer to 326 more)	Very low
**Non-fatal myocardial infarction**	DPP-4 inhibitors	Basal-Bolus insulin	3.12 (0.31 to 33.33)	10 per 1000 persons	31 per 1000 persons (3 to 252 per 1000 persons)	21 more (7 fewer to 242 more)	Very low
**Non-fatal myocardial infarction**	GLP-1 receptor agonists	Basal-Bolus insulin	2.78 (0.28 to 25.00)	10 per 1000 persons	27 per 1000 persons (3 to 202 per 1000 persons)	17 more (7 fewer to 192 more)	Very low
**Non-fatal myocardial infarction**	Meglitinides	Basal-Bolus insulin	0.86 (0.05 to 14.29)	10 per 1000 persons	9 per 1000 persons (0 to 126 per 1000 persons)	1 fewer (10 fewer to 116 more)	Very low
**Non-fatal myocardial infarction**	Metformin	Basal-Bolus insulin	2.63 (0.26 to 25.00)	10 per 1000 persons	26 per 1000 persons (3 to 202 per 1000 persons)	16 more (7 fewer to 192 more)	Low
**Non-fatal myocardial infarction**	Non-steroidal MRAs	Basal-Bolus insulin	2.78 (0.28 to 25.00)	10 per 1000 persons	27 per 1000 persons (3 to 202 per 1000 persons)	17 more (7 fewer to 192 more)	Very low
**Non-fatal myocardial infarction**	SGLT2 inhibitors	Basal-Bolus insulin	2.70 (0.27 to 25.00)	10 per 1000 persons	27 per 1000 persons (3 to 202 per 1000 persons)	17 more (7 fewer to 192 more)	Low
**Non-fatal myocardial infarction**	Standard treatments	Basal-Bolus insulin	3.03 (0.31 to 33.33)	10 per 1000 persons	30 per 1000 persons (3 to 252 per 1000 persons)	20 more (7 fewer to 242 more)	Very low
**Non-fatal myocardial infarction**	Sulfonylureas	Basal-Bolus insulin	3.03 (0.30 to 33.33)	10 per 1000 persons	30 per 1000 persons (3 to 252 per 1000 persons)	20 more (7 fewer to 242 more)	Low

Outcome	Intervention	Comparator	Odds ratio	Baseline risk	Risk if treated	Risk difference	Certainty
**Non-fatal myocardial infarction**	Thiazolidinediones	Basal-Bolus insulin	2.94 (0.29 to 33.33)	10 per 1000 persons	29 per 1000 persons (3 to 252 per 1000 persons)	19 more (7 fewer to 242 more)	Low
**Non-fatal myocardial infarction**	Tirzepatide	Basal-Bolus insulin	2.08 (0.09 to 50.00)	10 per 1000 persons	21 per 1000 persons (1 to 336 per 1000 persons)	11 more (9 fewer to 326 more)	Low
**Non-fatal myocardial infarction**	DPP-4 inhibitors	Bolus insulin	0.85 (0.29 to 2.56)	35 per 1000 persons	30 per 1000 persons (10 to 85 per 1000 persons)	5 fewer (25 fewer to 50 more)	Very low
**Non-fatal myocardial infarction**	GLP-1 receptor agonists	Bolus insulin	0.78 (0.26 to 2.27)	35 per 1000 persons	27 per 1000 persons (9 to 76 per 1000 persons)	8 fewer (26 fewer to 41 more)	Very low
**Non-fatal myocardial infarction**	Meglitinides	Bolus insulin	0.24 (0.03 to 1.85)	35 per 1000 persons	9 per 1000 persons (1 to 63 per 1000 persons)	26 fewer (34 fewer to 28 more)	Very low
**Non-fatal myocardial infarction**	Metformin	Bolus insulin	0.73 (0.24 to 2.22)	35 per 1000 persons	26 per 1000 persons (9 to 75 per 1000 persons)	9 fewer (26 fewer to 40 more)	Very low
**Non-fatal myocardial infarction**	Non-steroidal MRAs	Bolus insulin	0.77 (0.26 to 2.33)	35 per 1000 persons	27 per 1000 persons (9 to 78 per 1000 persons)	8 fewer (26 fewer to 43 more)	Very low
**Non-fatal myocardial infarction**	SGLT2 inhibitors	Bolus insulin	0.76 (0.26 to 2.27)	35 per 1000 persons	27 per 1000 persons (9 to 76 per 1000 persons)	8 fewer (26 fewer to 41 more)	Very low
**Non-fatal myocardial infarction**	Standard treatments	Bolus insulin	0.85 (0.29 to 2.50)	35 per 1000 persons	30 per 1000 persons (10 to 83 per 1000 persons)	5 fewer (25 fewer to 48 more)	Very low
**Non-fatal myocardial infarction**	Sulfonylureas	Bolus insulin	0.85 (0.28 to 2.56)	35 per 1000 persons	30 per 1000 persons (10 to 85 per 1000 persons)	5 fewer (25 fewer to 50 more)	Very low
**Non-fatal myocardial infarction**	Thiazolidinediones	Bolus insulin	0.82 (0.27 to 2.44)	35 per 1000 persons	29 per 1000 persons (10 to 81 per 1000 persons)	6 fewer (25 fewer to 46 more)	Very low
**Non-fatal myocardial infarction**	Tirzepatide	Bolus insulin	0.58 (0.05 to 6.67)	35 per 1000 persons	21 per 1000 persons (2 to 195 per 1000 persons)	14 fewer (33 fewer to 160 more)	Very low
**Non-fatal myocardial infarction**	GLP-1 receptor agonists	DPP-4 inhibitors	0.90 (0.81 to 1.01)	30 per 1000 persons	27 per 1000 persons (24 to 30 per 1000 persons)	3 fewer (6 fewer to 0)	Moderate
**Non-fatal myocardial infarction**	Meglitinides	DPP-4 inhibitors	0.28 (0.05 to 1.59)	30 per 1000 persons	9 per 1000 persons (2 to 47 per 1000 persons)	21 fewer (28 fewer to 17 more)	Very low
**Non-fatal myocardial infarction**	Metformin	DPP-4 inhibitors	0.85 (0.66 to 1.10)	30 per 1000 persons	26 per 1000 persons (20 to 33 per 1000 persons)	4 fewer (10 fewer to 3 more)	Moderate

Outcome	Intervention	Comparator	Odds ratio	Baseline risk	Risk if treated	Risk difference	Certainty
**Non-fatal myocardial infarction**	Non-steroidal MRAs	DPP-4 inhibitors	0.90 (0.72 to 1.14)	30 per 1000 persons	27 per 1000 persons (22 to 34 per 1000 persons)	3 fewer (8 fewer to 4 more)	Moderate
**Non-fatal myocardial infarction**	SGLT2 inhibitors	DPP-4 inhibitors	0.88 (0.78 to 1.01)	30 per 1000 persons	27 per 1000 persons (24 to 30 per 1000 persons)	3 fewer (6 fewer to 0)	Moderate
**Non-fatal myocardial infarction**	Standard treatments	DPP-4 inhibitors	0.99 (0.90 to 1.09)	30 per 1000 persons	30 per 1000 persons (27 to 33 per 1000 persons)	0 (3 fewer to 3 more)	Moderate
**Non-fatal myocardial infarction**	Sulfonylureas	DPP-4 inhibitors	0.99 (0.83 to 1.19)	30 per 1000 persons	30 per 1000 persons (25 to 36 per 1000 persons)	0 (5 fewer to 6 more)	High
**Non-fatal myocardial infarction**	Thiazolidinediones	DPP-4 inhibitors	0.96 (0.79 to 1.15)	30 per 1000 persons	29 per 1000 persons (24 to 34 per 1000 persons)	1 fewer (6 fewer to 4 more)	Moderate
**Non-fatal myocardial infarction**	Tirzepatide	DPP-4 inhibitors	0.68 (0.08 to 5.88)	30 per 1000 persons	21 per 1000 persons (2 to 154 per 1000 persons)	9 fewer (28 fewer to 124 more)	Very low
**Non-fatal myocardial infarction**	Meglitinides	GLP-1 receptor agonists	0.31 (0.05 to 1.75)	27 per 1000 persons	8 per 1000 persons (2 to 46 per 1000 persons)	19 fewer (25 fewer to 19 more)	Very low
**Non-fatal myocardial infarction**	Metformin	GLP-1 receptor agonists	0.94 (0.74 to 1.20)	27 per 1000 persons	26 per 1000 persons (20 to 32 per 1000 persons)	1 fewer (7 fewer to 5 more)	Moderate
**Non-fatal myocardial infarction**	Non-steroidal MRAs	GLP-1 receptor agonists	1.00 (0.80 to 1.25)	27 per 1000 persons	27 per 1000 persons (22 to 34 per 1000 persons)	0 (5 fewer to 7 more)	Moderate
**Non-fatal myocardial infarction**	SGLT2 inhibitors	GLP-1 receptor agonists	0.98 (0.88 to 1.10)	27 per 1000 persons	26 per 1000 persons (24 to 30 per 1000 persons)	1 fewer (3 fewer to 3 more)	Moderate
**Non-fatal myocardial infarction**	Standard treatments	GLP-1 receptor agonists	1.10 (1.02 to 1.18)	27 per 1000 persons	30 per 1000 persons (28 to 32 per 1000 persons)	3 more (1 more to 5 more)	High
**Non-fatal myocardial infarction**	Sulfonylureas	GLP-1 receptor agonists	1.10 (0.89 to 1.35)	27 per 1000 persons	30 per 1000 persons (24 to 36 per 1000 persons)	3 more (3 fewer to 9 more)	Moderate
**Non-fatal myocardial infarction**	Thiazolidinediones	GLP-1 receptor agonists	1.05 (0.88 to 1.27)	27 per 1000 persons	28 per 1000 persons (24 to 34 per 1000 persons)	1 more (3 fewer to 7 more)	Moderate
**Non-fatal myocardial infarction**	Tirzepatide	GLP-1 receptor agonists	0.75 (0.09 to 6.67)	27 per 1000 persons	20 per 1000 persons (2 to 156 per 1000 persons)	7 fewer (25 fewer to 129 more)	Low
**Non-fatal myocardial infarction**	Metformin	Meglitinides	3.03 (0.53 to 16.67)	9 per 1000 persons	27 per 1000 persons (5 to 131 per 1000 persons)	18 more (4 fewer to 122 more)	Low

Outcome	Intervention	Comparator	Odds ratio	Baseline risk	Risk if treated	Risk difference	Certainty
**Non-fatal myocardial infarction**	Non-steroidal MRAs	Meglitinides	3.23 (0.56 to 20.00)	9 per 1000 persons	28 per 1000 persons (5 to 154 per 1000 persons)	19 more (4 fewer to 145 more)	Very low
**Non-fatal myocardial infarction**	SGLT2 inhibitors	Meglitinides	3.23 (0.56 to 16.67)	9 per 1000 persons	28 per 1000 persons (5 to 131 per 1000 persons)	19 more (4 fewer to 122 more)	Low
**Non-fatal myocardial infarction**	Standard treatments	Meglitinides	3.57 (0.62 to 20.00)	9 per 1000 persons	31 per 1000 persons (6 to 154 per 1000 persons)	22 more (3 fewer to 145 more)	Low
**Non-fatal myocardial infarction**	Sulfonylureas	Meglitinides	3.57 (0.63 to 20.00)	9 per 1000 persons	31 per 1000 persons (6 to 154 per 1000 persons)	22 more (3 fewer to 145 more)	Low
**Non-fatal myocardial infarction**	Thiazolidinediones	Meglitinides	3.45 (0.60 to 20.00)	9 per 1000 persons	30 per 1000 persons (5 to 154 per 1000 persons)	21 more (4 fewer to 145 more)	Very low
**Non-fatal myocardial infarction**	Tirzepatide	Meglitinides	2.44 (0.15 to 33.33)	9 per 1000 persons	22 per 1000 persons (1 to 232 per 1000 persons)	13 more (8 fewer to 223 more)	Low
**Non-fatal myocardial infarction**	Non-steroidal MRAs	Metformin	1.06 (0.77 to 1.45)	26 per 1000 persons	28 per 1000 persons (20 to 37 per 1000 persons)	2 more (6 fewer to 11 more)	Low
**Non-fatal myocardial infarction**	SGLT2 inhibitors	Metformin	1.04 (0.81 to 1.35)	26 per 1000 persons	27 per 1000 persons (21 to 35 per 1000 persons)	1 more (5 fewer to 9 more)	Moderate
**Non-fatal myocardial infarction**	Standard treatments	Metformin	1.16 (0.92 to 1.47)	26 per 1000 persons	30 per 1000 persons (24 to 38 per 1000 persons)	4 more (2 fewer to 12 more)	Low
**Non-fatal myocardial infarction**	Sulfonylureas	Metformin	1.16 (0.88 to 1.56)	26 per 1000 persons	30 per 1000 persons (23 to 40 per 1000 persons)	4 more (3 fewer to 14 more)	Low
**Non-fatal myocardial infarction**	Thiazolidinediones	Metformin	1.12 (0.85 to 1.49)	26 per 1000 persons	29 per 1000 persons (22 to 38 per 1000 persons)	3 more (4 fewer to 12 more)	Low
**Non-fatal myocardial infarction**	Tirzepatide	Metformin	0.80 (0.09 to 7.14)	26 per 1000 persons	21 per 1000 persons (2 to 160 per 1000 persons)	5 fewer (24 fewer to 134 more)	Very low
**Non-fatal myocardial infarction**	SGLT2 inhibitors	Non-steroidal MRAs	0.98 (0.79 to 1.23)	27 per 1000 persons	26 per 1000 persons (21 to 33 per 1000 persons)	1 fewer (6 fewer to 6 more)	Moderate
**Non-fatal myocardial infarction**	Standard treatments	Non-steroidal MRAs	1.10 (0.89 to 1.35)	27 per 1000 persons	30 per 1000 persons (24 to 36 per 1000 persons)	3 more (3 fewer to 9 more)	Moderate
**Non-fatal myocardial infarction**	Sulfonylureas	Non-steroidal MRAs	1.10 (0.83 to 1.47)	27 per 1000 persons	30 per 1000 persons (22 to 39 per 1000 persons)	3 more (5 fewer to 12 more)	Low

Outcome	Intervention	Comparator	Odds ratio	Baseline risk	Risk if treated	Risk difference	Certainty
**Non-fatal myocardial infarction**	Thiazolidinediones	Non-steroidal MRAs	1.06 (0.81 to 1.39)	27 per 1000 persons	29 per 1000 persons (22 to 37 per 1000 persons)	2 more (5 fewer to 10 more)	Moderate
**Non-fatal myocardial infarction**	Tirzepatide	Non-steroidal MRAs	0.76 (0.08 to 6.67)	27 per 1000 persons	21 per 1000 persons (2 to 156 per 1000 persons)	6 fewer (25 fewer to 129 more)	Very low
**Non-fatal myocardial infarction**	Standard treatments	SGLT2 inhibitors	1.11 (1.02 to 1.22)	27 per 1000 persons	30 per 1000 persons (28 to 33 per 1000 persons)	3 more (1 more to 6 more)	High
**Non-fatal myocardial infarction**	Sulfonylureas	SGLT2 inhibitors	1.12 (0.91 to 1.39)	27 per 1000 persons	30 per 1000 persons (25 to 37 per 1000 persons)	3 more (2 fewer to 10 more)	Moderate
**Non-fatal myocardial infarction**	Thiazolidinediones	SGLT2 inhibitors	1.08 (0.89 to 1.32)	27 per 1000 persons	29 per 1000 persons (24 to 35 per 1000 persons)	2 more (3 fewer to 8 more)	Moderate
**Non-fatal myocardial infarction**	Tirzepatide	SGLT2 inhibitors	0.77 (0.09 to 6.67)	27 per 1000 persons	21 per 1000 persons (2 to 156 per 1000 persons)	6 fewer (25 fewer to 129 more)	Very low
**Non-fatal myocardial infarction**	Standard treatments	Sulfonylureas	1.00 (0.82 to 1.20)	30 per 1000 persons	30 per 1000 persons (25 to 36 per 1000 persons)	0 (5 fewer to 6 more)	Moderate
**Non-fatal myocardial infarction**	Thiazolidinediones	Sulfonylureas	0.96 (0.77 to 1.20)	30 per 1000 persons	29 per 1000 persons (23 to 36 per 1000 persons)	1 fewer (7 fewer to 6 more)	Moderate
**Non-fatal myocardial infarction**	Tirzepatide	Sulfonylureas	0.68 (0.08 to 6.25)	30 per 1000 persons	21 per 1000 persons (2 to 162 per 1000 persons)	9 fewer (28 fewer to 132 more)	Very low
**Non-fatal myocardial infarction**	Standard treatments	Thiazolidinediones	1.03 (0.87 to 1.23)	29 per 1000 persons	30 per 1000 persons (25 to 36 per 1000 persons)	1 more (4 fewer to 7 more)	Moderate
**Non-fatal myocardial infarction**	Tirzepatide	Thiazolidinediones	0.71 (0.08 to 6.25)	29 per 1000 persons	21 per 1000 persons (2 to 157 per 1000 persons)	8 fewer (27 fewer to 128 more)	Very low
**Non-fatal myocardial infarction**	Standard treatments	Tirzepatide	1.45 (0.16 to 12.50)	21 per 1000 persons	30 per 1000 persons (4 to 211 per 1000 persons)	9 more (17 fewer to 190 more)	Very low
**Non-fatal stroke**	Alpha-glucosidase inhibitors	Basal insulin	12.42 (0.88 to 176.12)	23 per 1000 persons	226 per 1000 persons (20 to 806 per 1000 persons)	203 more (3 fewer to 783 more)	Low
**Non-fatal stroke**	Alpha-glucosidase inhibitors	Basal-Bolus insulin	16.34 (0.76 to 350.38)	18 per 1000 persons	230 per 1000 persons (14 to 865 per 1000 persons)	212 more (4 fewer to 847 more)	Very low
**Non-fatal stroke**	Alpha-glucosidase inhibitors	Bolus insulin	11.00 (0.54 to 222.94)	26 per 1000 persons	227 per 1000 persons (14 to 856 per 1000 persons)	201 more (12 fewer to 830 more)	Very low

Outcome	Intervention	Comparator	Odds ratio	Baseline risk	Risk if treated	Risk difference	Certainty
**Non-fatal stroke**	Alpha-glucosidase inhibitors	DPP-4 inhibitors	10.41 (0.84 to 128.53)	27 per 1000 persons	224 per 1000 persons (23 to 781 per 1000 persons)	197 more (4 fewer to 754 more)	Very low
**Non-fatal stroke**	Alpha-glucosidase inhibitors	GLP-1 receptor agonists	11.08 (0.90 to 137.09)	26 per 1000 persons	228 per 1000 persons (23 to 785 per 1000 persons)	202 more (3 fewer to 759 more)	Low
**Non-fatal stroke**	Alpha-glucosidase inhibitors	Meglitinides	5.53 (0.45 to 67.89)	50 per 1000 persons	225 per 1000 persons (23 to 781 per 1000 persons)	175 more (27 fewer to 731 more)	Very low
**Non-fatal stroke**	Alpha-glucosidase inhibitors	Metformin	9.73 (0.78 to 121.84)	29 per 1000 persons	225 per 1000 persons (23 to 784 per 1000 persons)	196 more (6 fewer to 755 more)	Very low
**Non-fatal stroke**	Alpha-glucosidase inhibitors	Non-steroidal MRAs	9.46 (0.76 to 117.72)	30 per 1000 persons	226 per 1000 persons (23 to 785 per 1000 persons)	196 more (7 fewer to 755 more)	Very low
**Non-fatal stroke**	Alpha-glucosidase inhibitors	SGLT2 inhibitors	9.52 (0.77 to 117.89)	30 per 1000 persons	227 per 1000 persons (23 to 785 per 1000 persons)	197 more (7 fewer to 755 more)	Low
**Non-fatal stroke**	Alpha-glucosidase inhibitors	Standard treatments	9.44 (0.76 to 116.58)	30 per 1000 persons	226 per 1000 persons (23 to 783 per 1000 persons)	196 more (7 fewer to 753 more)	Very low
**Non-fatal stroke**	Alpha-glucosidase inhibitors	Sulfonylureas	8.97 (0.73 to 110.10)	31 per 1000 persons	223 per 1000 persons (23 to 779 per 1000 persons)	192 more (8 fewer to 748 more)	Very low
**Non-fatal stroke**	Alpha-glucosidase inhibitors	Thiazolidinediones	11.12 (0.90 to 137.86)	26 per 1000 persons	229 per 1000 persons (23 to 786 per 1000 persons)	203 more (3 fewer to 760 more)	Very low
**Non-fatal stroke**	Basal insulin	Basal-Bolus insulin	1.32 (0.23 to 7.61)	18 per 1000 persons	24 per 1000 persons (4 to 122 per 1000 persons)	6 more (14 fewer to 104 more)	Very low
**Non-fatal stroke**	Basal insulin	Bolus insulin	0.89 (0.14 to 5.66)	26 per 1000 persons	23 per 1000 persons (4 to 131 per 1000 persons)	3 fewer (22 fewer to 105 more)	Very low
**Non-fatal stroke**	Basal insulin	DPP-4 inhibitors	0.84 (0.36 to 1.97)	27 per 1000 persons	23 per 1000 persons (10 to 52 per 1000 persons)	4 fewer (17 fewer to 25 more)	Very low
**Non-fatal stroke**	Basal insulin	GLP-1 receptor agonists	0.89 (0.38 to 2.07)	26 per 1000 persons	23 per 1000 persons (10 to 52 per 1000 persons)	3 fewer (16 fewer to 26 more)	Very low
**Non-fatal stroke**	Basal insulin	Meglitinides	0.45 (0.06 to 3.56)	50 per 1000 persons	23 per 1000 persons (3 to 158 per 1000 persons)	27 fewer (47 fewer to 108 more)	Very low
**Non-fatal stroke**	Basal insulin	Metformin	0.78 (0.32 to 1.93)	29 per 1000 persons	23 per 1000 persons (9 to 55 per 1000 persons)	6 fewer (20 fewer to 26 more)	Very low

Outcome	Intervention	Comparator	Odds ratio	Baseline risk	Risk if treated	Risk difference	Certainty
**Non-fatal stroke**	Basal insulin	Non-steroidal MRAs	0.76 (0.32 to 1.82)	30 per 1000 persons	23 per 1000 persons (10 to 53 per 1000 persons)	7 fewer (20 fewer to 23 more)	Very low
**Non-fatal stroke**	Basal insulin	SGLT2 inhibitors	0.77 (0.33 to 1.80)	30 per 1000 persons	23 per 1000 persons (10 to 53 per 1000 persons)	7 fewer (20 fewer to 23 more)	Very low
**Non-fatal stroke**	Basal insulin	Standard treatments	0.76 (0.33 to 1.77)	30 per 1000 persons	23 per 1000 persons (10 to 52 per 1000 persons)	7 fewer (20 fewer to 22 more)	Very low
**Non-fatal stroke**	Basal insulin	Sulfonylureas	0.72 (0.30 to 1.73)	31 per 1000 persons	23 per 1000 persons (10 to 52 per 1000 persons)	8 fewer (21 fewer to 21 more)	Very low
**Non-fatal stroke**	Basal insulin	Thiazolidinediones	0.90 (0.38 to 2.13)	26 per 1000 persons	23 per 1000 persons (10 to 54 per 1000 persons)	3 fewer (16 fewer to 28 more)	Very low
**Non-fatal stroke**	Basal-Bolus insulin	Bolus insulin	0.67 (0.06 to 7.50)	26 per 1000 persons	18 per 1000 persons (2 to 167 per 1000 persons)	8 fewer (24 fewer to 141 more)	Very low
**Non-fatal stroke**	Basal-Bolus insulin	DPP-4 inhibitors	0.64 (0.11 to 3.71)	27 per 1000 persons	17 per 1000 persons (3 to 93 per 1000 persons)	10 fewer (24 fewer to 66 more)	Very low
**Non-fatal stroke**	Basal-Bolus insulin	GLP-1 receptor agonists	0.68 (0.12 to 3.92)	26 per 1000 persons	18 per 1000 persons (3 to 95 per 1000 persons)	8 fewer (23 fewer to 69 more)	Very low
**Non-fatal stroke**	Basal-Bolus insulin	Meglitinides	0.34 (0.03 to 4.50)	50 per 1000 persons	18 per 1000 persons (2 to 191 per 1000 persons)	32 fewer (48 fewer to 141 more)	Very low
**Non-fatal stroke**	Basal-Bolus insulin	Metformin	0.60 (0.10 to 3.54)	29 per 1000 persons	18 per 1000 persons (3 to 96 per 1000 persons)	11 fewer (26 fewer to 67 more)	Very low
**Non-fatal stroke**	Basal-Bolus insulin	Non-steroidal MRAs	0.58 (0.10 to 3.39)	30 per 1000 persons	18 per 1000 persons (3 to 95 per 1000 persons)	12 fewer (27 fewer to 65 more)	Very low
**Non-fatal stroke**	Basal-Bolus insulin	SGLT2 inhibitors	0.58 (0.10 to 3.39)	30 per 1000 persons	18 per 1000 persons (3 to 95 per 1000 persons)	12 fewer (27 fewer to 65 more)	Very low
**Non-fatal stroke**	Basal-Bolus insulin	Standard treatments	0.58 (0.10 to 3.35)	30 per 1000 persons	18 per 1000 persons (3 to 94 per 1000 persons)	12 fewer (27 fewer to 64 more)	Very low
**Non-fatal stroke**	Basal-Bolus insulin	Sulfonylureas	0.55 (0.09 to 3.22)	31 per 1000 persons	17 per 1000 persons (3 to 93 per 1000 persons)	14 fewer (28 fewer to 62 more)	Very low
**Non-fatal stroke**	Basal-Bolus insulin	Thiazolidinediones	0.68 (0.12 to 3.96)	26 per 1000 persons	18 per 1000 persons (3 to 96 per 1000 persons)	8 fewer (23 fewer to 70 more)	Very low

Outcome	Intervention	Comparator	Odds ratio	Baseline risk	Risk if treated	Risk difference	Certainty
**Non-fatal stroke**	Bolus insulin	DPP-4 inhibitors	0.95 (0.18 to 4.97)	27 per 1000 persons	26 per 1000 persons (5 to 121 per 1000 persons)	1 fewer (22 fewer to 94 more)	Very low
**Non-fatal stroke**	Bolus insulin	GLP-1 receptor agonists	1.01 (0.19 to 5.24)	26 per 1000 persons	26 per 1000 persons (5 to 123 per 1000 persons)	0 (21 fewer to 97 more)	Very low
**Non-fatal stroke**	Bolus insulin	Meglitinides	0.50 (0.04 to 6.24)	50 per 1000 persons	26 per 1000 persons (2 to 247 per 1000 persons)	24 fewer (48 fewer to 197 more)	Very low
**Non-fatal stroke**	Bolus insulin	Metformin	0.88 (0.16 to 4.76)	29 per 1000 persons	26 per 1000 persons (5 to 124 per 1000 persons)	3 fewer (24 fewer to 95 more)	Very low
**Non-fatal stroke**	Bolus insulin	Non-steroidal MRAs	0.86 (0.16 to 4.54)	30 per 1000 persons	26 per 1000 persons (5 to 123 per 1000 persons)	4 fewer (25 fewer to 93 more)	Very low
**Non-fatal stroke**	Bolus insulin	SGLT2 inhibitors	0.87 (0.16 to 4.54)	30 per 1000 persons	26 per 1000 persons (5 to 123 per 1000 persons)	4 fewer (25 fewer to 93 more)	Very low
**Non-fatal stroke**	Bolus insulin	Standard treatments	0.86 (0.16 to 4.48)	30 per 1000 persons	26 per 1000 persons (5 to 122 per 1000 persons)	4 fewer (25 fewer to 92 more)	Very low
**Non-fatal stroke**	Bolus insulin	Sulfonylureas	0.82 (0.15 to 4.33)	31 per 1000 persons	26 per 1000 persons (5 to 122 per 1000 persons)	5 fewer (26 fewer to 91 more)	Very low
**Non-fatal stroke**	Bolus insulin	Thiazolidinediones	1.01 (0.19 to 5.34)	26 per 1000 persons	26 per 1000 persons (5 to 125 per 1000 persons)	0 (21 fewer to 99 more)	Very low
**Non-fatal stroke**	DPP-4 inhibitors	GLP-1 receptor agonists	1.06 (0.91 to 1.25)	26 per 1000 persons	28 per 1000 persons (24 to 32 per 1000 persons)	2 more (2 fewer to 6 more)	Moderate
**Non-fatal stroke**	DPP-4 inhibitors	Meglitinides	0.53 (0.08 to 3.55)	50 per 1000 persons	27 per 1000 persons (4 to 157 per 1000 persons)	23 fewer (46 fewer to 107 more)	Very low
**Non-fatal stroke**	DPP-4 inhibitors	Metformin	0.93 (0.67 to 1.29)	29 per 1000 persons	27 per 1000 persons (20 to 37 per 1000 persons)	2 fewer (9 fewer to 8 more)	Moderate
**Non-fatal stroke**	DPP-4 inhibitors	Non-steroidal MRAs	0.91 (0.72 to 1.15)	30 per 1000 persons	27 per 1000 persons (22 to 34 per 1000 persons)	3 fewer (8 fewer to 4 more)	Moderate
**Non-fatal stroke**	DPP-4 inhibitors	SGLT2 inhibitors	0.91 (0.77 to 1.08)	30 per 1000 persons	27 per 1000 persons (23 to 32 per 1000 persons)	3 fewer (7 fewer to 2 more)	Moderate
**Non-fatal stroke**	DPP-4 inhibitors	Standard treatments	0.91 (0.80 to 1.03)	30 per 1000 persons	27 per 1000 persons (24 to 31 per 1000 persons)	3 fewer (6 fewer to 1 more)	Moderate

Outcome	Intervention	Comparator	Odds ratio	Baseline risk	Risk if treated	Risk difference	Certainty
**Non-fatal stroke**	DPP-4 inhibitors	Sulfonylureas	0.86 (0.69 to 1.07)	31 per 1000 persons	27 per 1000 persons (22 to 33 per 1000 persons)	4 fewer (9 fewer to 2 more)	High
**Non-fatal stroke**	DPP-4 inhibitors	Thiazolidinediones	1.07 (0.85 to 1.33)	26 per 1000 persons	28 per 1000 persons (22 to 34 per 1000 persons)	2 more (4 fewer to 8 more)	Low
**Non-fatal stroke**	GLP-1 receptor agonists	Meglitinides	0.50 (0.07 to 3.35)	50 per 1000 persons	26 per 1000 persons (4 to 150 per 1000 persons)	24 fewer (46 fewer to 100 more)	Very low
**Non-fatal stroke**	GLP-1 receptor agonists	Metformin	0.88 (0.63 to 1.22)	29 per 1000 persons	26 per 1000 persons (18 to 35 per 1000 persons)	3 fewer (11 fewer to 6 more)	Low
**Non-fatal stroke**	GLP-1 receptor agonists	Non-steroidal MRAs	0.85 (0.68 to 1.07)	30 per 1000 persons	26 per 1000 persons (21 to 32 per 1000 persons)	4 fewer (9 fewer to 2 more)	Moderate
**Non-fatal stroke**	GLP-1 receptor agonists	SGLT2 inhibitors	0.86 (0.74 to 1.00)	30 per 1000 persons	26 per 1000 persons (22 to 30 per 1000 persons)	4 fewer (8 fewer to 0)	Moderate
**Non-fatal stroke**	GLP-1 receptor agonists	Standard treatments	0.85 (0.77 to 0.94)	30 per 1000 persons	26 per 1000 persons (23 to 28 per 1000 persons)	4 fewer (7 fewer to 2 fewer)	High
**Non-fatal stroke**	GLP-1 receptor agonists	Sulfonylureas	0.81 (0.63 to 1.04)	31 per 1000 persons	25 per 1000 persons (20 to 32 per 1000 persons)	6 fewer (11 fewer to 1 more)	Low
**Non-fatal stroke**	GLP-1 receptor agonists	Thiazolidinediones	1.00 (0.80 to 1.25)	26 per 1000 persons	26 per 1000 persons (21 to 32 per 1000 persons)	0 (5 fewer to 6 more)	Moderate
**Non-fatal stroke**	Meglitinides	Metformin	1.76 (0.26 to 11.98)	29 per 1000 persons	50 per 1000 persons (8 to 264 per 1000 persons)	21 more (21 fewer to 235 more)	Very low
**Non-fatal stroke**	Meglitinides	Non-steroidal MRAs	1.71 (0.25 to 11.54)	30 per 1000 persons	50 per 1000 persons (8 to 263 per 1000 persons)	20 more (22 fewer to 233 more)	Very low
**Non-fatal stroke**	Meglitinides	SGLT2 inhibitors	1.72 (0.26 to 11.53)	30 per 1000 persons	51 per 1000 persons (8 to 263 per 1000 persons)	21 more (22 fewer to 233 more)	Very low
**Non-fatal stroke**	Meglitinides	Standard treatments	1.71 (0.26 to 11.40)	30 per 1000 persons	50 per 1000 persons (8 to 261 per 1000 persons)	20 more (22 fewer to 231 more)	Very low
**Non-fatal stroke**	Meglitinides	Sulfonylureas	1.62 (0.24 to 10.79)	31 per 1000 persons	49 per 1000 persons (8 to 257 per 1000 persons)	18 more (23 fewer to 226 more)	Very low
**Non-fatal stroke**	Meglitinides	Thiazolidinediones	2.01 (0.30 to 13.51)	26 per 1000 persons	51 per 1000 persons (8 to 265 per 1000 persons)	25 more (18 fewer to 239 more)	Very low

Outcome	Intervention	Comparator	Odds ratio	Baseline risk	Risk if treated	Risk difference	Certainty
**Non-fatal stroke**	Metformin	Non-steroidal MRAs	0.97 (0.67 to 1.41)	30 per 1000 persons	29 per 1000 persons (20 to 42 per 1000 persons)	1 fewer (10 fewer to 12 more)	Low
**Non-fatal stroke**	Metformin	SGLT2 inhibitors	0.98 (0.70 to 1.37)	30 per 1000 persons	29 per 1000 persons (21 to 41 per 1000 persons)	1 fewer (9 fewer to 11 more)	Low
**Non-fatal stroke**	Metformin	Standard treatments	0.97 (0.71 to 1.33)	30 per 1000 persons	29 per 1000 persons (21 to 40 per 1000 persons)	1 fewer (9 fewer to 10 more)	Moderate
**Non-fatal stroke**	Metformin	Sulfonylureas	0.92 (0.66 to 1.30)	31 per 1000 persons	29 per 1000 persons (21 to 40 per 1000 persons)	2 fewer (10 fewer to 9 more)	Moderate
**Non-fatal stroke**	Metformin	Thiazolidinediones	1.14 (0.81 to 1.61)	26 per 1000 persons	30 per 1000 persons (21 to 41 per 1000 persons)	4 more (5 fewer to 15 more)	Very low
**Non-fatal stroke**	Non-steroidal MRAs	SGLT2 inhibitors	1.01 (0.80 to 1.27)	30 per 1000 persons	30 per 1000 persons (24 to 38 per 1000 persons)	0 (6 fewer to 8 more)	Moderate
**Non-fatal stroke**	Non-steroidal MRAs	Standard treatments	1.00 (0.82 to 1.22)	30 per 1000 persons	30 per 1000 persons (25 to 36 per 1000 persons)	0 (5 fewer to 6 more)	Moderate
**Non-fatal stroke**	Non-steroidal MRAs	Sulfonylureas	0.95 (0.70 to 1.29)	31 per 1000 persons	29 per 1000 persons (22 to 40 per 1000 persons)	2 fewer (9 fewer to 9 more)	Moderate
**Non-fatal stroke**	Non-steroidal MRAs	Thiazolidinediones	1.18 (0.89 to 1.56)	26 per 1000 persons	31 per 1000 persons (23 to 40 per 1000 persons)	5 more (3 fewer to 14 more)	Very low
**Non-fatal stroke**	SGLT2 inhibitors	Standard treatments	0.99 (0.88 to 1.11)	30 per 1000 persons	30 per 1000 persons (26 to 33 per 1000 persons)	0 (4 fewer to 3 more)	High
**Non-fatal stroke**	SGLT2 inhibitors	Sulfonylureas	0.94 (0.73 to 1.21)	31 per 1000 persons	29 per 1000 persons (23 to 37 per 1000 persons)	2 fewer (8 fewer to 6 more)	Moderate
**Non-fatal stroke**	SGLT2 inhibitors	Thiazolidinediones	1.17 (0.93 to 1.47)	26 per 1000 persons	30 per 1000 persons (24 to 38 per 1000 persons)	4 more (2 fewer to 12 more)	Low
**Non-fatal stroke**	Sulfonylureas	Standard treatments	1.05 (0.84 to 1.32)	30 per 1000 persons	31 per 1000 persons (25 to 39 per 1000 persons)	1 more (5 fewer to 9 more)	Moderate
**Non-fatal stroke**	Sulfonylureas	Thiazolidinediones	1.24 (0.96 to 1.61)	26 per 1000 persons	32 per 1000 persons (25 to 41 per 1000 persons)	6 more (1 fewer to 15 more)	Low
**Non-fatal stroke**	Thiazolidinediones	Standard treatments	0.85 (0.70 to 1.03)	30 per 1000 persons	26 per 1000 persons (21 to 31 per 1000 persons)	4 fewer (9 fewer to 1 more)	Low

Outcome	Intervention	Comparator	Odds ratio	Baseline risk	Risk if treated	Risk difference	Certainty
**Non-fatal stroke**	Basal insulin	Alpha-glucosidase inhibitors	0.08 (0.01 to 1.14)	226 per 1000 persons	23 per 1000 persons (2 to 249 per 1000 persons)	203 fewer (224 fewer to 23 more)	Very low
**Non-fatal stroke**	Basal-Bolus insulin	Alpha-glucosidase inhibitors	0.06 (0.00 to 1.32)	226 per 1000 persons	18 per 1000 persons (1 to 278 per 1000 persons)	208 fewer (225 fewer to 52 more)	Very low
**Non-fatal stroke**	Bolus insulin	Alpha-glucosidase inhibitors	0.09 (0.00 to 1.85)	226 per 1000 persons	26 per 1000 persons (1 to 351 per 1000 persons)	200 fewer (225 fewer to 125 more)	Very low
**Non-fatal stroke**	DPP-4 inhibitors	Alpha-glucosidase inhibitors	0.10 (0.01 to 1.19)	226 per 1000 persons	27 per 1000 persons (2 to 258 per 1000 persons)	199 fewer (224 fewer to 32 more)	Very low
**Non-fatal stroke**	GLP-1 receptor agonists	Alpha-glucosidase inhibitors	0.09 (0.01 to 1.11)	226 per 1000 persons	26 per 1000 persons (2 to 245 per 1000 persons)	200 fewer (224 fewer to 19 more)	Very low
**Non-fatal stroke**	Meglitinides	Alpha-glucosidase inhibitors	0.18 (0.01 to 2.22)	226 per 1000 persons	50 per 1000 persons (4 to 394 per 1000 persons)	176 fewer (222 fewer to 168 more)	Very low
**Non-fatal stroke**	Metformin	Alpha-glucosidase inhibitors	0.10 (0.01 to 1.28)	226 per 1000 persons	29 per 1000 persons (2 to 272 per 1000 persons)	197 fewer (224 fewer to 46 more)	Very low
**Non-fatal stroke**	Non-steroidal MRAs	Alpha-glucosidase inhibitors	0.11 (0.01 to 1.32)	226 per 1000 persons	30 per 1000 persons (2 to 278 per 1000 persons)	196 fewer (224 fewer to 52 more)	Very low
**Non-fatal stroke**	SGLT2 inhibitors	Alpha-glucosidase inhibitors	0.11 (0.01 to 1.30)	226 per 1000 persons	30 per 1000 persons (2 to 275 per 1000 persons)	196 fewer (224 fewer to 49 more)	Very low
**Non-fatal stroke**	Standard treatments	Alpha-glucosidase inhibitors	0.11 (0.01 to 1.32)	226 per 1000 persons	30 per 1000 persons (2 to 278 per 1000 persons)	196 fewer (224 fewer to 52 more)	Very low
**Non-fatal stroke**	Sulfonylureas	Alpha-glucosidase inhibitors	0.11 (0.01 to 1.37)	226 per 1000 persons	32 per 1000 persons (3 to 286 per 1000 persons)	194 fewer (223 fewer to 60 more)	Very low
**Non-fatal stroke**	Thiazolidinediones	Alpha-glucosidase inhibitors	0.09 (0.01 to 1.11)	226 per 1000 persons	26 per 1000 persons (2 to 245 per 1000 persons)	200 fewer (224 fewer to 19 more)	Very low
**Non-fatal stroke**	Basal-Bolus insulin	Basal insulin	0.76 (0.13 to 4.35)	23 per 1000 persons	18 per 1000 persons (3 to 93 per 1000 persons)	5 fewer (20 fewer to 70 more)	Very low
**Non-fatal stroke**	Bolus insulin	Basal insulin	1.12 (0.18 to 7.14)	23 per 1000 persons	26 per 1000 persons (4 to 144 per 1000 persons)	3 more (19 fewer to 121 more)	Very low
**Non-fatal stroke**	DPP-4 inhibitors	Basal insulin	1.19 (0.51 to 2.78)	23 per 1000 persons	27 per 1000 persons (12 to 61 per 1000 persons)	4 more (11 fewer to 38 more)	Very low

Outcome	Intervention	Comparator	Odds ratio	Baseline risk	Risk if treated	Risk difference	Certainty
**Non-fatal stroke**	GLP-1 receptor agonists	Basal insulin	1.12 (0.48 to 2.63)	23 per 1000 persons	26 per 1000 persons (11 to 58 per 1000 persons)	3 more (12 fewer to 35 more)	Very low
**Non-fatal stroke**	Meglitinides	Basal insulin	2.22 (0.28 to 16.67)	23 per 1000 persons	50 per 1000 persons (7 to 282 per 1000 persons)	27 more (16 fewer to 259 more)	Very low
**Non-fatal stroke**	Metformin	Basal insulin	1.28 (0.52 to 3.12)	23 per 1000 persons	29 per 1000 persons (12 to 69 per 1000 persons)	6 more (11 fewer to 46 more)	Very low
**Non-fatal stroke**	Non-steroidal MRAs	Basal insulin	1.32 (0.55 to 3.12)	23 per 1000 persons	30 per 1000 persons (13 to 69 per 1000 persons)	7 more (10 fewer to 46 more)	Low
**Non-fatal stroke**	SGLT2 inhibitors	Basal insulin	1.30 (0.56 to 3.03)	23 per 1000 persons	30 per 1000 persons (13 to 67 per 1000 persons)	7 more (10 fewer to 44 more)	Low
**Non-fatal stroke**	Standard treatments	Basal insulin	1.32 (0.56 to 3.03)	23 per 1000 persons	30 per 1000 persons (13 to 67 per 1000 persons)	7 more (10 fewer to 44 more)	Low
**Non-fatal stroke**	Sulfonylureas	Basal insulin	1.39 (0.58 to 3.33)	23 per 1000 persons	32 per 1000 persons (13 to 73 per 1000 persons)	9 more (10 fewer to 50 more)	Low
**Non-fatal stroke**	Thiazolidinediones	Basal insulin	1.11 (0.47 to 2.63)	23 per 1000 persons	25 per 1000 persons (11 to 58 per 1000 persons)	2 more (12 fewer to 35 more)	Very low
**Non-fatal stroke**	Bolus insulin	Basal-Bolus insulin	1.49 (0.13 to 16.67)	18 per 1000 persons	27 per 1000 persons (2 to 234 per 1000 persons)	9 more (16 fewer to 216 more)	Very low
**Non-fatal stroke**	DPP-4 inhibitors	Basal-Bolus insulin	1.56 (0.27 to 9.09)	18 per 1000 persons	28 per 1000 persons (5 to 143 per 1000 persons)	10 more (13 fewer to 125 more)	Very low
**Non-fatal stroke**	GLP-1 receptor agonists	Basal-Bolus insulin	1.47 (0.26 to 8.33)	18 per 1000 persons	26 per 1000 persons (5 to 133 per 1000 persons)	8 more (13 fewer to 115 more)	Very low
**Non-fatal stroke**	Meglitinides	Basal-Bolus insulin	2.94 (0.22 to 33.33)	18 per 1000 persons	51 per 1000 persons (4 to 379 per 1000 persons)	33 more (14 fewer to 361 more)	Very low
**Non-fatal stroke**	Metformin	Basal-Bolus insulin	1.67 (0.28 to 10.00)	18 per 1000 persons	30 per 1000 persons (5 to 155 per 1000 persons)	12 more (13 fewer to 137 more)	Very low
**Non-fatal stroke**	Non-steroidal MRAs	Basal-Bolus insulin	1.72 (0.29 to 10.00)	18 per 1000 persons	31 per 1000 persons (5 to 155 per 1000 persons)	13 more (13 fewer to 137 more)	Very low
**Non-fatal stroke**	SGLT2 inhibitors	Basal-Bolus insulin	1.72 (0.29 to 10.00)	18 per 1000 persons	31 per 1000 persons (5 to 155 per 1000 persons)	13 more (13 fewer to 137 more)	Very low

Outcome	Intervention	Comparator	Odds ratio	Baseline risk	Risk if treated	Risk difference	Certainty
**Non-fatal stroke**	Standard treatments	Basal-Bolus insulin	1.72 (0.30 to 10.00)	18 per 1000 persons	31 per 1000 persons (5 to 155 per 1000 persons)	13 more (13 fewer to 137 more)	Very low
**Non-fatal stroke**	Sulfonylureas	Basal-Bolus insulin	1.82 (0.31 to 11.11)	18 per 1000 persons	32 per 1000 persons (6 to 169 per 1000 persons)	14 more (12 fewer to 151 more)	Very low
**Non-fatal stroke**	Thiazolidinediones	Basal-Bolus insulin	1.47 (0.25 to 8.33)	18 per 1000 persons	26 per 1000 persons (5 to 133 per 1000 persons)	8 more (13 fewer to 115 more)	Very low
**Non-fatal stroke**	DPP-4 inhibitors	Bolus insulin	1.05 (0.20 to 5.56)	26 per 1000 persons	27 per 1000 persons (5 to 129 per 1000 persons)	1 more (21 fewer to 103 more)	Very low
**Non-fatal stroke**	GLP-1 receptor agonists	Bolus insulin	0.99 (0.19 to 5.26)	26 per 1000 persons	26 per 1000 persons (5 to 123 per 1000 persons)	0 (21 fewer to 97 more)	Very low
**Non-fatal stroke**	Meglitinides	Bolus insulin	2.00 (0.16 to 25.00)	26 per 1000 persons	51 per 1000 persons (4 to 400 per 1000 persons)	25 more (22 fewer to 374 more)	Very low
**Non-fatal stroke**	Metformin	Bolus insulin	1.14 (0.21 to 6.25)	26 per 1000 persons	29 per 1000 persons (6 to 143 per 1000 persons)	3 more (20 fewer to 117 more)	Very low
**Non-fatal stroke**	Non-steroidal MRAs	Bolus insulin	1.16 (0.22 to 6.25)	26 per 1000 persons	30 per 1000 persons (6 to 143 per 1000 persons)	4 more (20 fewer to 117 more)	Very low
**Non-fatal stroke**	SGLT2 inhibitors	Bolus insulin	1.15 (0.22 to 6.25)	26 per 1000 persons	30 per 1000 persons (6 to 143 per 1000 persons)	4 more (20 fewer to 117 more)	Very low
**Non-fatal stroke**	Standard treatments	Bolus insulin	1.16 (0.22 to 6.25)	26 per 1000 persons	30 per 1000 persons (6 to 143 per 1000 persons)	4 more (20 fewer to 117 more)	Very low
**Non-fatal stroke**	Sulfonylureas	Bolus insulin	1.22 (0.23 to 6.67)	26 per 1000 persons	32 per 1000 persons (6 to 151 per 1000 persons)	6 more (20 fewer to 125 more)	Very low
**Non-fatal stroke**	Thiazolidinediones	Bolus insulin	0.99 (0.19 to 5.26)	26 per 1000 persons	26 per 1000 persons (5 to 123 per 1000 persons)	0 (21 fewer to 97 more)	Very low
**Non-fatal stroke**	GLP-1 receptor agonists	DPP-4 inhibitors	0.94 (0.80 to 1.10)	27 per 1000 persons	26 per 1000 persons (22 to 30 per 1000 persons)	1 fewer (5 fewer to 3 more)	Moderate
**Non-fatal stroke**	Meglitinides	DPP-4 inhibitors	1.89 (0.28 to 12.50)	27 per 1000 persons	50 per 1000 persons (8 to 258 per 1000 persons)	23 more (19 fewer to 231 more)	Very low
**Non-fatal stroke**	Metformin	DPP-4 inhibitors	1.08 (0.78 to 1.49)	27 per 1000 persons	29 per 1000 persons (21 to 40 per 1000 persons)	2 more (6 fewer to 13 more)	Low

Outcome	Intervention	Comparator	Odds ratio	Baseline risk	Risk if treated	Risk difference	Certainty
**Non-fatal stroke**	Non-steroidal MRAs	DPP-4 inhibitors	1.10 (0.87 to 1.39)	27 per 1000 persons	30 per 1000 persons (24 to 37 per 1000 persons)	3 more (3 fewer to 10 more)	Moderate
**Non-fatal stroke**	SGLT2 inhibitors	DPP-4 inhibitors	1.10 (0.93 to 1.30)	27 per 1000 persons	30 per 1000 persons (25 to 35 per 1000 persons)	3 more (2 fewer to 8 more)	Moderate
**Non-fatal stroke**	Standard treatments	DPP-4 inhibitors	1.10 (0.97 to 1.25)	27 per 1000 persons	30 per 1000 persons (26 to 34 per 1000 persons)	3 more (1 fewer to 7 more)	Moderate
**Non-fatal stroke**	Sulfonylureas	DPP-4 inhibitors	1.16 (0.93 to 1.45)	27 per 1000 persons	31 per 1000 persons (25 to 39 per 1000 persons)	4 more (2 fewer to 12 more)	Moderate
**Non-fatal stroke**	Thiazolidinediones	DPP-4 inhibitors	0.93 (0.75 to 1.18)	27 per 1000 persons	25 per 1000 persons (20 to 32 per 1000 persons)	2 fewer (7 fewer to 5 more)	Low
**Non-fatal stroke**	Meglitinides	GLP-1 receptor agonists	2.00 (0.30 to 14.29)	26 per 1000 persons	51 per 1000 persons (8 to 276 per 1000 persons)	25 more (18 fewer to 250 more)	Very low
**Non-fatal stroke**	Metformin	GLP-1 receptor agonists	1.14 (0.82 to 1.59)	26 per 1000 persons	29 per 1000 persons (21 to 41 per 1000 persons)	3 more (5 fewer to 15 more)	Low
**Non-fatal stroke**	Non-steroidal MRAs	GLP-1 receptor agonists	1.18 (0.93 to 1.47)	26 per 1000 persons	30 per 1000 persons (24 to 38 per 1000 persons)	4 more (2 fewer to 12 more)	Low
**Non-fatal stroke**	SGLT2 inhibitors	GLP-1 receptor agonists	1.16 (1.00 to 1.35)	26 per 1000 persons	30 per 1000 persons (26 to 35 per 1000 persons)	4 more (0 to 9 more)	Moderate
**Non-fatal stroke**	Standard treatments	GLP-1 receptor agonists	1.18 (1.06 to 1.30)	26 per 1000 persons	30 per 1000 persons (28 to 34 per 1000 persons)	4 more (2 more to 8 more)	High
**Non-fatal stroke**	Sulfonylureas	GLP-1 receptor agonists	1.23 (0.96 to 1.59)	26 per 1000 persons	32 per 1000 persons (25 to 41 per 1000 persons)	6 more (1 fewer to 15 more)	Low
**Non-fatal stroke**	Thiazolidinediones	GLP-1 receptor agonists	1.00 (0.80 to 1.25)	26 per 1000 persons	26 per 1000 persons (21 to 32 per 1000 persons)	0 (5 fewer to 6 more)	Moderate
**Non-fatal stroke**	Metformin	Meglitinides	0.57 (0.08 to 3.85)	50 per 1000 persons	29 per 1000 persons (4 to 168 per 1000 persons)	21 fewer (46 fewer to 118 more)	Very low
**Non-fatal stroke**	Non-steroidal MRAs	Meglitinides	0.58 (0.09 to 4.00)	50 per 1000 persons	30 per 1000 persons (5 to 174 per 1000 persons)	20 fewer (45 fewer to 124 more)	Very low
**Non-fatal stroke**	SGLT2 inhibitors	Meglitinides	0.58 (0.09 to 3.85)	50 per 1000 persons	30 per 1000 persons (5 to 168 per 1000 persons)	20 fewer (45 fewer to 118 more)	Very low

Outcome	Intervention	Comparator	Odds ratio	Baseline risk	Risk if treated	Risk difference	Certainty
**Non-fatal stroke**	Standard treatments	Meglitinides	0.58 (0.09 to 3.85)	50 per 1000 persons	30 per 1000 persons (5 to 168 per 1000 persons)	20 fewer (45 fewer to 118 more)	Very low
**Non-fatal stroke**	Sulfonylureas	Meglitinides	0.62 (0.09 to 4.17)	50 per 1000 persons	31 per 1000 persons (5 to 180 per 1000 persons)	19 fewer (45 fewer to 130 more)	Very low
**Non-fatal stroke**	Thiazolidinediones	Meglitinides	0.50 (0.07 to 3.33)	50 per 1000 persons	26 per 1000 persons (4 to 149 per 1000 persons)	24 fewer (46 fewer to 99 more)	Very low
**Non-fatal stroke**	Non-steroidal MRAs	Metformin	1.03 (0.71 to 1.49)	29 per 1000 persons	30 per 1000 persons (21 to 43 per 1000 persons)	1 more (8 fewer to 14 more)	Low
**Non-fatal stroke**	SGLT2 inhibitors	Metformin	1.02 (0.73 to 1.43)	29 per 1000 persons	30 per 1000 persons (21 to 41 per 1000 persons)	1 more (8 fewer to 12 more)	Low
**Non-fatal stroke**	Standard treatments	Metformin	1.03 (0.75 to 1.41)	29 per 1000 persons	30 per 1000 persons (22 to 40 per 1000 persons)	1 more (7 fewer to 11 more)	Low
**Non-fatal stroke**	Sulfonylureas	Metformin	1.09 (0.77 to 1.52)	29 per 1000 persons	31 per 1000 persons (22 to 43 per 1000 persons)	2 more (7 fewer to 14 more)	Low
**Non-fatal stroke**	Thiazolidinediones	Metformin	0.88 (0.62 to 1.23)	29 per 1000 persons	26 per 1000 persons (18 to 36 per 1000 persons)	3 fewer (11 fewer to 7 more)	Very low
**Non-fatal stroke**	SGLT2 inhibitors	Non-steroidal MRAs	0.99 (0.79 to 1.25)	30 per 1000 persons	30 per 1000 persons (24 to 37 per 1000 persons)	0 (6 fewer to 7 more)	Moderate
**Non-fatal stroke**	Standard treatments	Non-steroidal MRAs	1.00 (0.82 to 1.22)	30 per 1000 persons	30 per 1000 persons (25 to 36 per 1000 persons)	0 (5 fewer to 6 more)	Moderate
**Non-fatal stroke**	Sulfonylureas	Non-steroidal MRAs	1.05 (0.78 to 1.43)	30 per 1000 persons	32 per 1000 persons (23 to 42 per 1000 persons)	2 more (7 fewer to 12 more)	Low
**Non-fatal stroke**	Thiazolidinediones	Non-steroidal MRAs	0.85 (0.64 to 1.12)	30 per 1000 persons	26 per 1000 persons (19 to 34 per 1000 persons)	4 fewer (11 fewer to 4 more)	Very low
**Non-fatal stroke**	Standard treatments	SGLT2 inhibitors	1.01 (0.90 to 1.14)	30 per 1000 persons	30 per 1000 persons (27 to 34 per 1000 persons)	0 (3 fewer to 4 more)	High
**Non-fatal stroke**	Sulfonylureas	SGLT2 inhibitors	1.06 (0.83 to 1.37)	30 per 1000 persons	32 per 1000 persons (25 to 41 per 1000 persons)	2 more (5 fewer to 11 more)	Low
**Non-fatal stroke**	Thiazolidinediones	SGLT2 inhibitors	0.85 (0.68 to 1.08)	30 per 1000 persons	26 per 1000 persons (21 to 32 per 1000 persons)	4 fewer (9 fewer to 2 more)	Moderate

Outcome	Intervention	Comparator	Odds ratio	Baseline risk	Risk if treated	Risk difference	Certainty
**Non-fatal stroke**	Standard treatments	Sulfonylureas	0.95 (0.76 to 1.19)	31 per 1000 persons	30 per 1000 persons (24 to 37 per 1000 persons)	1 fewer (7 fewer to 6 more)	Moderate
**Non-fatal stroke**	Thiazolidinediones	Sulfonylureas	0.81 (0.62 to 1.04)	31 per 1000 persons	25 per 1000 persons (19 to 32 per 1000 persons)	6 fewer (12 fewer to 1 more)	Low
**Non-fatal stroke**	Standard treatments	Thiazolidinediones	1.18 (0.97 to 1.43)	26 per 1000 persons	30 per 1000 persons (25 to 37 per 1000 persons)	4 more (1 fewer to 11 more)	Very low
**Hospitalisation for heart failure**	Alpha-glucosidase inhibitors	Basal insulin	3.47 (0.13 to 90.34)	5 per 1000 persons	17 per 1000 persons (1 to 312 per 1000 persons)	12 more (4 fewer to 307 more)	Low
**Hospitalisation for heart failure**	Alpha-glucosidase inhibitors	Bolus insulin	5.05 (0.10 to 262.01)	3 per 1000 persons	15 per 1000 persons (0 to 441 per 1000 persons)	12 more (3 fewer to 438 more)	Very low
**Hospitalisation for heart failure**	Alpha-glucosidase inhibitors	DPP-4 inhibitors	3.09 (0.12 to 78.44)	5 per 1000 persons	15 per 1000 persons (1 to 283 per 1000 persons)	10 more (4 fewer to 278 more)	Low
**Hospitalisation for heart failure**	Alpha-glucosidase inhibitors	GLP-1 receptor agonists	3.59 (0.14 to 91.17)	5 per 1000 persons	18 per 1000 persons (1 to 314 per 1000 persons)	13 more (4 fewer to 309 more)	Low
**Hospitalisation for heart failure**	Alpha-glucosidase inhibitors	Metformin	2.25 (0.06 to 84.00)	7 per 1000 persons	16 per 1000 persons (0 to 372 per 1000 persons)	9 more (7 fewer to 365 more)	Low
**Hospitalisation for heart failure**	Alpha-glucosidase inhibitors	Non-steroidal MRAs	4.18 (0.16 to 106.46)	4 per 1000 persons	17 per 1000 persons (1 to 299 per 1000 persons)	13 more (3 fewer to 295 more)	Low
**Hospitalisation for heart failure**	Alpha-glucosidase inhibitors	SGLT2 inhibitors	4.93 (0.19 to 125.08)	3 per 1000 persons	15 per 1000 persons (1 to 273 per 1000 persons)	12 more (2 fewer to 270 more)	Low
**Hospitalisation for heart failure**	Alpha-glucosidase inhibitors	Standard treatments	3.25 (0.13 to 82.49)	5 per 1000 persons	16 per 1000 persons (1 to 293 per 1000 persons)	11 more (4 fewer to 288 more)	Low
**Hospitalisation for heart failure**	Alpha-glucosidase inhibitors	Sulfonylureas	3.30 (0.13 to 84.25)	5 per 1000 persons	16 per 1000 persons (1 to 297 per 1000 persons)	11 more (4 fewer to 292 more)	Low
**Hospitalisation for heart failure**	Alpha-glucosidase inhibitors	Thiazolidinediones	2.11 (0.08 to 53.77)	8 per 1000 persons	17 per 1000 persons (1 to 302 per 1000 persons)	9 more (7 fewer to 294 more)	Low
**Hospitalisation for heart failure**	Alpha-glucosidase inhibitors	Tirzepatide	5.18 (0.16 to 171.40)	3 per 1000 persons	15 per 1000 persons (0 to 340 per 1000 persons)	12 more (3 fewer to 337 more)	Low
**Hospitalisation for heart failure**	Basal insulin	Bolus insulin	1.46 (0.15 to 14.61)	3 per 1000 persons	4 per 1000 persons (0 to 42 per 1000 persons)	1 more (3 fewer to 39 more)	Low

Outcome	Intervention	Comparator	Odds ratio	Baseline risk	Risk if treated	Risk difference	Certainty
**Hospitalisation for heart failure**	Basal insulin	DPP-4 inhibitors	0.89 (0.59 to 1.36)	5 per 1000 persons	4 per 1000 persons (3 to 7 per 1000 persons)	1 fewer (2 fewer to 2 more)	High
**Hospitalisation for heart failure**	Basal insulin	GLP-1 receptor agonists	1.04 (0.68 to 1.58)	5 per 1000 persons	5 per 1000 persons (3 to 8 per 1000 persons)	0 (2 fewer to 3 more)	Moderate
**Hospitalisation for heart failure**	Basal insulin	Metformin	0.65 (0.12 to 3.48)	7 per 1000 persons	5 per 1000 persons (1 to 24 per 1000 persons)	2 fewer (6 fewer to 17 more)	Low
**Hospitalisation for heart failure**	Basal insulin	Non-steroidal MRAs	1.21 (0.77 to 1.90)	4 per 1000 persons	5 per 1000 persons (3 to 8 per 1000 persons)	1 more (1 fewer to 4 more)	Moderate
**Hospitalisation for heart failure**	Basal insulin	SGLT2 inhibitors	1.42 (0.92 to 2.19)	3 per 1000 persons	4 per 1000 persons (3 to 7 per 1000 persons)	1 more (0 to 4 more)	Moderate
**Hospitalisation for heart failure**	Basal insulin	Standard treatments	0.94 (0.62 to 1.43)	5 per 1000 persons	5 per 1000 persons (3 to 7 per 1000 persons)	0 (2 fewer to 2 more)	Moderate
**Hospitalisation for heart failure**	Basal insulin	Sulfonylureas	0.95 (0.62 to 1.47)	5 per 1000 persons	5 per 1000 persons (3 to 7 per 1000 persons)	0 (2 fewer to 2 more)	High
**Hospitalisation for heart failure**	Basal insulin	Thiazolidinediones	0.61 (0.38 to 0.96)	8 per 1000 persons	5 per 1000 persons (3 to 8 per 1000 persons)	3 fewer (5 fewer to 0)	Moderate
**Hospitalisation for heart failure**	Basal insulin	Tirzepatide	1.50 (0.42 to 5.32)	3 per 1000 persons	4 per 1000 persons (1 to 16 per 1000 persons)	1 more (2 fewer to 13 more)	Moderate
**Hospitalisation for heart failure**	Bolus insulin	DPP-4 inhibitors	0.61 (0.06 to 5.92)	5 per 1000 persons	3 per 1000 persons (0 to 29 per 1000 persons)	2 fewer (5 fewer to 24 more)	Very low
**Hospitalisation for heart failure**	Bolus insulin	GLP-1 receptor agonists	0.71 (0.07 to 6.85)	5 per 1000 persons	4 per 1000 persons (0 to 33 per 1000 persons)	1 fewer (5 fewer to 28 more)	Very low
**Hospitalisation for heart failure**	Bolus insulin	Metformin	0.45 (0.03 to 7.25)	7 per 1000 persons	3 per 1000 persons (0 to 49 per 1000 persons)	4 fewer (7 fewer to 42 more)	Low
**Hospitalisation for heart failure**	Bolus insulin	Non-steroidal MRAs	0.83 (0.09 to 8.04)	4 per 1000 persons	3 per 1000 persons (0 to 31 per 1000 persons)	1 fewer (4 fewer to 27 more)	Very low
**Hospitalisation for heart failure**	Bolus insulin	SGLT2 inhibitors	0.98 (0.10 to 9.43)	3 per 1000 persons	3 per 1000 persons (0 to 28 per 1000 persons)	0 (3 fewer to 25 more)	Low
**Hospitalisation for heart failure**	Bolus insulin	Standard treatments	0.64 (0.07 to 6.22)	5 per 1000 persons	3 per 1000 persons (0 to 30 per 1000 persons)	2 fewer (5 fewer to 25 more)	Very low

Outcome	Intervention	Comparator	Odds ratio	Baseline risk	Risk if treated	Risk difference	Certainty
**Hospitalisation for heart failure**	Bolus insulin	Sulfonylureas	0.65 (0.07 to 6.37)	5 per 1000 persons	3 per 1000 persons (0 to 31 per 1000 persons)	2 fewer (5 fewer to 26 more)	Low
**Hospitalisation for heart failure**	Bolus insulin	Thiazolidinediones	0.42 (0.04 to 4.06)	8 per 1000 persons	3 per 1000 persons (0 to 32 per 1000 persons)	5 fewer (8 fewer to 24 more)	Low
**Hospitalisation for heart failure**	Bolus insulin	Tirzepatide	1.03 (0.07 to 14.25)	3 per 1000 persons	3 per 1000 persons (0 to 41 per 1000 persons)	0 (3 fewer to 38 more)	Low
**Hospitalisation for heart failure**	DPP-4 inhibitors	GLP-1 receptor agonists	1.16 (1.02 to 1.33)	5 per 1000 persons	6 per 1000 persons (5 to 7 per 1000 persons)	1 more (0 to 2 more)	Moderate
**Hospitalisation for heart failure**	DPP-4 inhibitors	Metformin	0.73 (0.14 to 3.72)	7 per 1000 persons	5 per 1000 persons (1 to 26 per 1000 persons)	2 fewer (6 fewer to 19 more)	Low
**Hospitalisation for heart failure**	DPP-4 inhibitors	Non-steroidal MRAs	1.35 (1.11 to 1.65)	4 per 1000 persons	5 per 1000 persons (4 to 7 per 1000 persons)	1 more (0 to 3 more)	Moderate
**Hospitalisation for heart failure**	DPP-4 inhibitors	SGLT2 inhibitors	1.59 (1.39 to 1.83)	3 per 1000 persons	5 per 1000 persons (4 to 5 per 1000 persons)	2 more (1 more to 2 more)	Moderate
**Hospitalisation for heart failure**	DPP-4 inhibitors	Standard treatments	1.05 (0.95 to 1.16)	5 per 1000 persons	5 per 1000 persons (5 to 6 per 1000 persons)	0 (0 to 1 more)	Moderate
**Hospitalisation for heart failure**	DPP-4 inhibitors	Sulfonylureas	1.07 (0.86 to 1.32)	5 per 1000 persons	5 per 1000 persons (4 to 7 per 1000 persons)	0 (1 fewer to 2 more)	High
**Hospitalisation for heart failure**	DPP-4 inhibitors	Thiazolidinediones	0.68 (0.55 to 0.85)	8 per 1000 persons	5 per 1000 persons (4 to 7 per 1000 persons)	3 fewer (4 fewer to 1 fewer)	Moderate
**Hospitalisation for heart failure**	DPP-4 inhibitors	Tirzepatide	1.68 (0.44 to 6.38)	3 per 1000 persons	5 per 1000 persons (1 to 19 per 1000 persons)	2 more (2 fewer to 16 more)	Low
**Hospitalisation for heart failure**	GLP-1 receptor agonists	Metformin	0.63 (0.12 to 3.19)	7 per 1000 persons	4 per 1000 persons (1 to 22 per 1000 persons)	3 fewer (6 fewer to 15 more)	Moderate
**Hospitalisation for heart failure**	GLP-1 receptor agonists	Non-steroidal MRAs	1.16 (0.96 to 1.41)	4 per 1000 persons	5 per 1000 persons (4 to 6 per 1000 persons)	1 more (0 to 2 more)	Moderate
**Hospitalisation for heart failure**	GLP-1 receptor agonists	SGLT2 inhibitors	1.37 (1.20 to 1.56)	3 per 1000 persons	4 per 1000 persons (4 to 5 per 1000 persons)	1 more (1 more to 2 more)	Moderate
**Hospitalisation for heart failure**	GLP-1 receptor agonists	Standard treatments	0.91 (0.83 to 0.99)	5 per 1000 persons	5 per 1000 persons (4 to 5 per 1000 persons)	0 (1 fewer to 0)	Moderate

Outcome	Intervention	Comparator	Odds ratio	Baseline risk	Risk if treated	Risk difference	Certainty
**Hospitalisation for heart failure**	GLP-1 receptor agonists	Sulfonylureas	0.92 (0.72 to 1.16)	5 per 1000 persons	5 per 1000 persons (4 to 6 per 1000 persons)	0 (1 fewer to 1 more)	Moderate
**Hospitalisation for heart failure**	GLP-1 receptor agonists	Thiazolidinediones	0.59 (0.47 to 0.73)	8 per 1000 persons	5 per 1000 persons (4 to 6 per 1000 persons)	3 fewer (4 fewer to 2 fewer)	Moderate
**Hospitalisation for heart failure**	GLP-1 receptor agonists	Tirzepatide	1.44 (0.38 to 5.50)	3 per 1000 persons	4 per 1000 persons (1 to 16 per 1000 persons)	1 more (2 fewer to 13 more)	Low
**Hospitalisation for heart failure**	Metformin	Non-steroidal MRAs	1.86 (0.36 to 9.54)	4 per 1000 persons	7 per 1000 persons (1 to 37 per 1000 persons)	3 more (3 fewer to 33 more)	Low
**Hospitalisation for heart failure**	Metformin	SGLT2 inhibitors	2.19 (0.43 to 11.16)	3 per 1000 persons	7 per 1000 persons (1 to 32 per 1000 persons)	4 more (2 fewer to 29 more)	Low
**Hospitalisation for heart failure**	Metformin	Standard treatments	1.45 (0.28 to 7.36)	5 per 1000 persons	7 per 1000 persons (1 to 36 per 1000 persons)	2 more (4 fewer to 31 more)	Low
**Hospitalisation for heart failure**	Metformin	Sulfonylureas	1.47 (0.28 to 7.57)	5 per 1000 persons	7 per 1000 persons (1 to 37 per 1000 persons)	2 more (4 fewer to 32 more)	Low
**Hospitalisation for heart failure**	Metformin	Thiazolidinediones	0.94 (0.18 to 4.81)	8 per 1000 persons	8 per 1000 persons (1 to 37 per 1000 persons)	0 (7 fewer to 29 more)	Low
**Hospitalisation for heart failure**	Metformin	Tirzepatide	2.30 (0.28 to 18.91)	3 per 1000 persons	7 per 1000 persons (1 to 54 per 1000 persons)	4 more (2 fewer to 51 more)	Low
**Hospitalisation for heart failure**	Non-steroidal MRAs	SGLT2 inhibitors	1.18 (0.97 to 1.43)	3 per 1000 persons	4 per 1000 persons (3 to 4 per 1000 persons)	1 more (0 to 1 more)	Moderate
**Hospitalisation for heart failure**	Non-steroidal MRAs	Standard treatments	0.78 (0.66 to 0.92)	5 per 1000 persons	4 per 1000 persons (3 to 5 per 1000 persons)	1 fewer (2 fewer to 0)	Moderate
**Hospitalisation for heart failure**	Non-steroidal MRAs	Sulfonylureas	0.79 (0.60 to 1.04)	5 per 1000 persons	4 per 1000 persons (3 to 5 per 1000 persons)	1 fewer (2 fewer to 0)	Moderate
**Hospitalisation for heart failure**	Non-steroidal MRAs	Thiazolidinediones	0.50 (0.39 to 0.65)	8 per 1000 persons	4 per 1000 persons (3 to 5 per 1000 persons)	4 fewer (5 fewer to 3 fewer)	Moderate
**Hospitalisation for heart failure**	Non-steroidal MRAs	Tirzepatide	1.24 (0.32 to 4.77)	3 per 1000 persons	4 per 1000 persons (1 to 14 per 1000 persons)	1 more (2 fewer to 11 more)	Low
**Hospitalisation for heart failure**	SGLT2 inhibitors	Standard treatments	0.66 (0.60 to 0.73)	5 per 1000 persons	3 per 1000 persons (3 to 4 per 1000 persons)	2 fewer (2 fewer to 1 fewer)	High

Outcome	Intervention	Comparator	Odds ratio	Baseline risk	Risk if treated	Risk difference	Certainty
**Hospitalisation for heart failure**	SGLT2 inhibitors	Sulfonylureas	0.67 (0.53 to 0.85)	5 per 1000 persons	3 per 1000 persons (3 to 4 per 1000 persons)	2 fewer (2 fewer to 1 fewer)	Moderate
**Hospitalisation for heart failure**	SGLT2 inhibitors	Thiazolidinediones	0.43 (0.34 to 0.53)	8 per 1000 persons	3 per 1000 persons (3 to 4 per 1000 persons)	5 fewer (5 fewer to 4 fewer)	Moderate
**Hospitalisation for heart failure**	SGLT2 inhibitors	Tirzepatide	1.05 (0.28 to 4.02)	3 per 1000 persons	3 per 1000 persons (1 to 12 per 1000 persons)	0 (2 fewer to 9 more)	Moderate
**Hospitalisation for heart failure**	Sulfonylureas	Standard treatments	0.99 (0.79 to 1.23)	5 per 1000 persons	5 per 1000 persons (4 to 6 per 1000 persons)	0 (1 fewer to 1 more)	Moderate
**Hospitalisation for heart failure**	Sulfonylureas	Thiazolidinediones	0.64 (0.48 to 0.85)	8 per 1000 persons	5 per 1000 persons (4 to 7 per 1000 persons)	3 fewer (4 fewer to 1 fewer)	Moderate
**Hospitalisation for heart failure**	Sulfonylureas	Tirzepatide	1.57 (0.41 to 6.01)	3 per 1000 persons	5 per 1000 persons (1 to 18 per 1000 persons)	2 more (2 fewer to 15 more)	Low
**Hospitalisation for heart failure**	Thiazolidinediones	Standard treatments	1.54 (1.27 to 1.88)	5 per 1000 persons	8 per 1000 persons (6 to 9 per 1000 persons)	3 more (1 more to 4 more)	Moderate
**Hospitalisation for heart failure**	Thiazolidinediones	Tirzepatide	2.46 (0.64 to 9.48)	3 per 1000 persons	7 per 1000 persons (2 to 28 per 1000 persons)	4 more (1 fewer to 25 more)	Low
**Hospitalisation for heart failure**	Tirzepatide	Standard treatments	0.63 (0.16 to 2.39)	5 per 1000 persons	3 per 1000 persons (1 to 12 per 1000 persons)	2 fewer (4 fewer to 7 more)	Moderate
**Hospitalisation for heart failure**	Basal insulin	Alpha-glucosidase inhibitors	0.29 (0.01 to 7.69)	16 per 1000 persons	5 per 1000 persons (0 to 111 per 1000 persons)	11 fewer (16 fewer to 95 more)	Very low
**Hospitalisation for heart failure**	Bolus insulin	Alpha-glucosidase inhibitors	0.20 (0.00 to 10.00)	16 per 1000 persons	3 per 1000 persons (0 to 140 per 1000 persons)	13 fewer (16 fewer to 124 more)	Very low
**Hospitalisation for heart failure**	DPP-4 inhibitors	Alpha-glucosidase inhibitors	0.32 (0.01 to 8.33)	16 per 1000 persons	5 per 1000 persons (0 to 119 per 1000 persons)	11 fewer (16 fewer to 103 more)	Very low
**Hospitalisation for heart failure**	GLP-1 receptor agonists	Alpha-glucosidase inhibitors	0.28 (0.01 to 7.14)	16 per 1000 persons	5 per 1000 persons (0 to 104 per 1000 persons)	11 fewer (16 fewer to 88 more)	Very low
**Hospitalisation for heart failure**	Metformin	Alpha-glucosidase inhibitors	0.44 (0.01 to 16.67)	16 per 1000 persons	7 per 1000 persons (0 to 213 per 1000 persons)	9 fewer (16 fewer to 197 more)	Very low
**Hospitalisation for heart failure**	Non-steroidal MRAs	Alpha-glucosidase inhibitors	0.24 (0.01 to 6.25)	16 per 1000 persons	4 per 1000 persons (0 to 92 per 1000 persons)	12 fewer (16 fewer to 76 more)	Very low

Outcome	Intervention	Comparator	Odds ratio	Baseline risk	Risk if treated	Risk difference	Certainty
**Hospitalisation for heart failure**	SGLT2 inhibitors	Alpha-glucosidase inhibitors	0.20 (0.01 to 5.26)	16 per 1000 persons	3 per 1000 persons (0 to 79 per 1000 persons)	13 fewer (16 fewer to 63 more)	Very low
**Hospitalisation for heart failure**	Standard treatments	Alpha-glucosidase inhibitors	0.31 (0.01 to 7.69)	16 per 1000 persons	5 per 1000 persons (0 to 111 per 1000 persons)	11 fewer (16 fewer to 95 more)	Very low
**Hospitalisation for heart failure**	Sulfonylureas	Alpha-glucosidase inhibitors	0.30 (0.01 to 7.69)	16 per 1000 persons	5 per 1000 persons (0 to 111 per 1000 persons)	11 fewer (16 fewer to 95 more)	Very low
**Hospitalisation for heart failure**	Thiazolidinediones	Alpha-glucosidase inhibitors	0.47 (0.02 to 12.50)	16 per 1000 persons	8 per 1000 persons (0 to 169 per 1000 persons)	8 fewer (16 fewer to 153 more)	Very low
**Hospitalisation for heart failure**	Tirzepatide	Alpha-glucosidase inhibitors	0.19 (0.01 to 6.25)	16 per 1000 persons	3 per 1000 persons (0 to 92 per 1000 persons)	13 fewer (16 fewer to 76 more)	Very low
**Hospitalisation for heart failure**	Bolus insulin	Basal insulin	0.68 (0.07 to 6.67)	5 per 1000 persons	3 per 1000 persons (0 to 32 per 1000 persons)	2 fewer (5 fewer to 27 more)	Low
**Hospitalisation for heart failure**	DPP-4 inhibitors	Basal insulin	1.12 (0.74 to 1.69)	5 per 1000 persons	6 per 1000 persons (4 to 8 per 1000 persons)	1 more (1 fewer to 3 more)	High
**Hospitalisation for heart failure**	GLP-1 receptor agonists	Basal insulin	0.96 (0.63 to 1.47)	5 per 1000 persons	5 per 1000 persons (3 to 7 per 1000 persons)	0 (2 fewer to 2 more)	Moderate
**Hospitalisation for heart failure**	Metformin	Basal insulin	1.54 (0.29 to 8.33)	5 per 1000 persons	8 per 1000 persons (1 to 40 per 1000 persons)	3 more (4 fewer to 35 more)	Low
**Hospitalisation for heart failure**	Non-steroidal MRAs	Basal insulin	0.83 (0.53 to 1.30)	5 per 1000 persons	4 per 1000 persons (3 to 6 per 1000 persons)	1 fewer (2 fewer to 1 more)	Moderate
**Hospitalisation for heart failure**	SGLT2 inhibitors	Basal insulin	0.70 (0.46 to 1.09)	5 per 1000 persons	4 per 1000 persons (2 to 5 per 1000 persons)	1 fewer (3 fewer to 0)	Moderate
**Hospitalisation for heart failure**	Standard treatments	Basal insulin	1.06 (0.70 to 1.61)	5 per 1000 persons	5 per 1000 persons (4 to 8 per 1000 persons)	0 (1 fewer to 3 more)	Moderate
**Hospitalisation for heart failure**	Sulfonylureas	Basal insulin	1.05 (0.68 to 1.61)	5 per 1000 persons	5 per 1000 persons (3 to 8 per 1000 persons)	0 (2 fewer to 3 more)	High
**Hospitalisation for heart failure**	Thiazolidinediones	Basal insulin	1.64 (1.04 to 2.63)	5 per 1000 persons	8 per 1000 persons (5 to 13 per 1000 persons)	3 more (0 to 8 more)	Moderate
**Hospitalisation for heart failure**	Tirzepatide	Basal insulin	0.67 (0.19 to 2.38)	5 per 1000 persons	3 per 1000 persons (1 to 12 per 1000 persons)	2 fewer (4 fewer to 7 more)	High

Outcome	Intervention	Comparator	Odds ratio	Baseline risk	Risk if treated	Risk difference	Certainty
**Hospitalisation for heart failure**	DPP-4 inhibitors	Bolus insulin	1.64 (0.17 to 16.67)	3 per 1000 persons	5 per 1000 persons (1 to 48 per 1000 persons)	2 more (2 fewer to 45 more)	Very low
**Hospitalisation for heart failure**	GLP-1 receptor agonists	Bolus insulin	1.41 (0.15 to 14.29)	3 per 1000 persons	4 per 1000 persons (0 to 41 per 1000 persons)	1 more (3 fewer to 38 more)	Very low
**Hospitalisation for heart failure**	Metformin	Bolus insulin	2.22 (0.14 to 33.33)	3 per 1000 persons	7 per 1000 persons (0 to 91 per 1000 persons)	4 more (3 fewer to 88 more)	Low
**Hospitalisation for heart failure**	Non-steroidal MRAs	Bolus insulin	1.20 (0.12 to 11.11)	3 per 1000 persons	4 per 1000 persons (0 to 32 per 1000 persons)	1 more (3 fewer to 29 more)	Very low
**Hospitalisation for heart failure**	SGLT2 inhibitors	Bolus insulin	1.02 (0.11 to 10.00)	3 per 1000 persons	3 per 1000 persons (0 to 29 per 1000 persons)	0 (3 fewer to 26 more)	Low
**Hospitalisation for heart failure**	Standard treatments	Bolus insulin	1.56 (0.16 to 14.29)	3 per 1000 persons	5 per 1000 persons (0 to 41 per 1000 persons)	2 more (3 fewer to 38 more)	Very low
**Hospitalisation for heart failure**	Sulfonylureas	Bolus insulin	1.54 (0.16 to 14.29)	3 per 1000 persons	5 per 1000 persons (0 to 41 per 1000 persons)	2 more (3 fewer to 38 more)	Low
**Hospitalisation for heart failure**	Thiazolidinediones	Bolus insulin	2.38 (0.25 to 25.00)	3 per 1000 persons	7 per 1000 persons (1 to 70 per 1000 persons)	4 more (2 fewer to 67 more)	Low
**Hospitalisation for heart failure**	Tirzepatide	Bolus insulin	0.97 (0.07 to 14.29)	3 per 1000 persons	3 per 1000 persons (0 to 41 per 1000 persons)	0 (3 fewer to 38 more)	Low
**Hospitalisation for heart failure**	GLP-1 receptor agonists	DPP-4 inhibitors	0.86 (0.75 to 0.98)	5 per 1000 persons	4 per 1000 persons (4 to 5 per 1000 persons)	1 fewer (1 fewer to 0)	Moderate
**Hospitalisation for heart failure**	Metformin	DPP-4 inhibitors	1.37 (0.27 to 7.14)	5 per 1000 persons	7 per 1000 persons (1 to 35 per 1000 persons)	2 more (4 fewer to 30 more)	Low
**Hospitalisation for heart failure**	Non-steroidal MRAs	DPP-4 inhibitors	0.74 (0.61 to 0.90)	5 per 1000 persons	4 per 1000 persons (3 to 5 per 1000 persons)	1 fewer (2 fewer to 0)	Moderate
**Hospitalisation for heart failure**	SGLT2 inhibitors	DPP-4 inhibitors	0.63 (0.55 to 0.72)	5 per 1000 persons	3 per 1000 persons (3 to 4 per 1000 persons)	2 fewer (2 fewer to 1 fewer)	Moderate
**Hospitalisation for heart failure**	Standard treatments	DPP-4 inhibitors	0.95 (0.86 to 1.05)	5 per 1000 persons	5 per 1000 persons (4 to 5 per 1000 persons)	0 (1 fewer to 0)	Moderate
**Hospitalisation for heart failure**	Sulfonylureas	DPP-4 inhibitors	0.93 (0.76 to 1.16)	5 per 1000 persons	5 per 1000 persons (4 to 6 per 1000 persons)	0 (1 fewer to 1 more)	High

Outcome	Intervention	Comparator	Odds ratio	Baseline risk	Risk if treated	Risk difference	Certainty
**Hospitalisation for heart failure**	Thiazolidinediones	DPP-4 inhibitors	1.47 (1.18 to 1.82)	5 per 1000 persons	7 per 1000 persons (6 to 9 per 1000 persons)	2 more (1 more to 4 more)	Moderate
**Hospitalisation for heart failure**	Tirzepatide	DPP-4 inhibitors	0.60 (0.16 to 2.27)	5 per 1000 persons	3 per 1000 persons (1 to 11 per 1000 persons)	2 fewer (4 fewer to 6 more)	Moderate
**Hospitalisation for heart failure**	Metformin	GLP-1 receptor agonists	1.59 (0.31 to 8.33)	5 per 1000 persons	8 per 1000 persons (2 to 40 per 1000 persons)	3 more (3 fewer to 35 more)	Moderate
**Hospitalisation for heart failure**	Non-steroidal MRAs	GLP-1 receptor agonists	0.86 (0.71 to 1.04)	5 per 1000 persons	4 per 1000 persons (4 to 5 per 1000 persons)	1 fewer (1 fewer to 0)	Moderate
**Hospitalisation for heart failure**	SGLT2 inhibitors	GLP-1 receptor agonists	0.73 (0.64 to 0.83)	5 per 1000 persons	4 per 1000 persons (3 to 4 per 1000 persons)	1 fewer (2 fewer to 1 fewer)	Moderate
**Hospitalisation for heart failure**	Standard treatments	GLP-1 receptor agonists	1.10 (1.01 to 1.20)	5 per 1000 persons	5 per 1000 persons (5 to 6 per 1000 persons)	0 (0 to 1 more)	Moderate
**Hospitalisation for heart failure**	Sulfonylureas	GLP-1 receptor agonists	1.09 (0.86 to 1.39)	5 per 1000 persons	5 per 1000 persons (4 to 7 per 1000 persons)	0 (1 fewer to 2 more)	Moderate
**Hospitalisation for heart failure**	Thiazolidinediones	GLP-1 receptor agonists	1.69 (1.37 to 2.13)	5 per 1000 persons	8 per 1000 persons (7 to 11 per 1000 persons)	3 more (2 more to 6 more)	Moderate
**Hospitalisation for heart failure**	Tirzepatide	GLP-1 receptor agonists	0.69 (0.18 to 2.63)	5 per 1000 persons	3 per 1000 persons (1 to 13 per 1000 persons)	2 fewer (4 fewer to 8 more)	Moderate
**Hospitalisation for heart failure**	Non-steroidal MRAs	Metformin	0.54 (0.10 to 2.78)	7 per 1000 persons	4 per 1000 persons (1 to 19 per 1000 persons)	3 fewer (6 fewer to 12 more)	Low
**Hospitalisation for heart failure**	SGLT2 inhibitors	Metformin	0.46 (0.09 to 2.33)	7 per 1000 persons	3 per 1000 persons (1 to 16 per 1000 persons)	4 fewer (6 fewer to 9 more)	Moderate
**Hospitalisation for heart failure**	Standard treatments	Metformin	0.69 (0.14 to 3.57)	7 per 1000 persons	5 per 1000 persons (1 to 25 per 1000 persons)	2 fewer (6 fewer to 18 more)	Low
**Hospitalisation for heart failure**	Sulfonylureas	Metformin	0.68 (0.13 to 3.57)	7 per 1000 persons	5 per 1000 persons (1 to 25 per 1000 persons)	2 fewer (6 fewer to 18 more)	Low
**Hospitalisation for heart failure**	Thiazolidinediones	Metformin	1.06 (0.21 to 5.56)	7 per 1000 persons	7 per 1000 persons (1 to 38 per 1000 persons)	0 (6 fewer to 31 more)	Low
**Hospitalisation for heart failure**	Tirzepatide	Metformin	0.43 (0.05 to 3.57)	7 per 1000 persons	3 per 1000 persons (0 to 25 per 1000 persons)	4 fewer (7 fewer to 18 more)	Low

Outcome	Intervention	Comparator	Odds ratio	Baseline risk	Risk if treated	Risk difference	Certainty
**Hospitalisation for heart failure**	SGLT2 inhibitors	Non-steroidal MRAs	0.85 (0.70 to 1.03)	4 per 1000 persons	3 per 1000 persons (3 to 4 per 1000 persons)	1 fewer (1 fewer to 0)	Moderate
**Hospitalisation for heart failure**	Standard treatments	Non-steroidal MRAs	1.28 (1.09 to 1.52)	4 per 1000 persons	5 per 1000 persons (4 to 6 per 1000 persons)	1 more (0 to 2 more)	Moderate
**Hospitalisation for heart failure**	Sulfonylureas	Non-steroidal MRAs	1.27 (0.96 to 1.67)	4 per 1000 persons	5 per 1000 persons (4 to 7 per 1000 persons)	1 more (0 to 3 more)	Moderate
**Hospitalisation for heart failure**	Thiazolidinediones	Non-steroidal MRAs	2.00 (1.54 to 2.56)	4 per 1000 persons	8 per 1000 persons (6 to 10 per 1000 persons)	4 more (2 more to 6 more)	Moderate
**Hospitalisation for heart failure**	Tirzepatide	Non-steroidal MRAs	0.81 (0.21 to 3.12)	4 per 1000 persons	3 per 1000 persons (1 to 12 per 1000 persons)	1 fewer (3 fewer to 8 more)	Moderate
**Hospitalisation for heart failure**	Standard treatments	SGLT2 inhibitors	1.52 (1.37 to 1.67)	3 per 1000 persons	5 per 1000 persons (4 to 5 per 1000 persons)	2 more (1 more to 2 more)	High
**Hospitalisation for heart failure**	Sulfonylureas	SGLT2 inhibitors	1.49 (1.18 to 1.89)	3 per 1000 persons	4 per 1000 persons (4 to 6 per 1000 persons)	1 more (1 more to 3 more)	Moderate
**Hospitalisation for heart failure**	Thiazolidinediones	SGLT2 inhibitors	2.33 (1.89 to 2.94)	3 per 1000 persons	7 per 1000 persons (6 to 9 per 1000 persons)	4 more (3 more to 6 more)	Moderate
**Hospitalisation for heart failure**	Tirzepatide	SGLT2 inhibitors	0.95 (0.25 to 3.57)	3 per 1000 persons	3 per 1000 persons (1 to 11 per 1000 persons)	0 (2 fewer to 8 more)	Moderate
**Hospitalisation for heart failure**	Standard treatments	Sulfonylureas	1.01 (0.81 to 1.27)	5 per 1000 persons	5 per 1000 persons (4 to 6 per 1000 persons)	0 (1 fewer to 1 more)	Moderate
**Hospitalisation for heart failure**	Thiazolidinediones	Sulfonylureas	1.56 (1.18 to 2.08)	5 per 1000 persons	8 per 1000 persons (6 to 10 per 1000 persons)	3 more (1 more to 5 more)	Moderate
**Hospitalisation for heart failure**	Tirzepatide	Sulfonylureas	0.64 (0.17 to 2.44)	5 per 1000 persons	3 per 1000 persons (1 to 12 per 1000 persons)	2 fewer (4 fewer to 7 more)	Moderate
**Hospitalisation for heart failure**	Standard treatments	Thiazolidinediones	0.65 (0.53 to 0.79)	8 per 1000 persons	5 per 1000 persons (4 to 6 per 1000 persons)	3 fewer (4 fewer to 2 fewer)	Moderate
**Hospitalisation for heart failure**	Tirzepatide	Thiazolidinediones	0.41 (0.11 to 1.56)	8 per 1000 persons	3 per 1000 persons (1 to 12 per 1000 persons)	5 fewer (7 fewer to 4 more)	Moderate
**Hospitalisation for heart failure**	Standard treatments	Tirzepatide	1.59 (0.42 to 6.25)	3 per 1000 persons	5 per 1000 persons (1 to 18 per 1000 persons)	2 more (2 fewer to 15 more)	Low

Outcome	Intervention	Comparator	Odds ratio	Baseline risk	Risk if treated	Risk difference	Certainty
**End-stage kidney disease**	Basal insulin	Bolus insulin	0.47 (0.02 to 12.36)	5 per 1000 persons	2 per 1000 persons (0 to 58 per 1000 persons)	3 fewer (5 fewer to 53 more)	Very low
**End-stage kidney disease**	Basal insulin	DPP-4 inhibitors	1.15 (0.59 to 2.24)	2 per 1000 persons	2 per 1000 persons (1 to 4 per 1000 persons)	0 (1 fewer to 2 more)	Low
**End-stage kidney disease**	Basal insulin	GLP-1 receptor agonists	1.44 (0.75 to 2.74)	2 per 1000 persons	3 per 1000 persons (2 to 5 per 1000 persons)	1 more (0 to 3 more)	Low
**End-stage kidney disease**	Basal insulin	Metformin	0.74 (0.14 to 3.83)	3 per 1000 persons	2 per 1000 persons (0 to 11 per 1000 persons)	1 fewer (3 fewer to 8 more)	Low
**End-stage kidney disease**	Basal insulin	Non-steroidal MRAs	1.44 (0.74 to 2.78)	2 per 1000 persons	3 per 1000 persons (1 to 6 per 1000 persons)	1 more (1 fewer to 4 more)	Low
**End-stage kidney disease**	Basal insulin	SGLT2 inhibitors	1.97 (1.02 to 3.82)	1 per 1000 persons	2 per 1000 persons (1 to 4 per 1000 persons)	1 more (0 to 3 more)	Low
**End-stage kidney disease**	Basal insulin	Standard treatments	1.20 (0.62 to 2.30)	2 per 1000 persons	2 per 1000 persons (1 to 5 per 1000 persons)	0 (1 fewer to 3 more)	Low
**End-stage kidney disease**	Basal insulin	Sulfonylureas	1.75 (0.72 to 4.26)	1 per 1000 persons	2 per 1000 persons (1 to 4 per 1000 persons)	1 more (0 to 3 more)	Low
**End-stage kidney disease**	Basal insulin	Thiazolidinediones	1.73 (0.70 to 4.26)	1 per 1000 persons	2 per 1000 persons (1 to 4 per 1000 persons)	1 more (0 to 3 more)	Low
**End-stage kidney disease**	Basal insulin	Tirzepatide	1.77 (0.26 to 12.27)	1 per 1000 persons	2 per 1000 persons (0 to 12 per 1000 persons)	1 more (1 fewer to 11 more)	Low
**End-stage kidney disease**	Bolus insulin	DPP-4 inhibitors	2.45 (0.10 to 60.68)	2 per 1000 persons	5 per 1000 persons (0 to 108 per 1000 persons)	3 more (2 fewer to 106 more)	Very low
**End-stage kidney disease**	Bolus insulin	GLP-1 receptor agonists	3.05 (0.12 to 75.27)	2 per 1000 persons	6 per 1000 persons (0 to 131 per 1000 persons)	4 more (2 fewer to 129 more)	Very low
**End-stage kidney disease**	Bolus insulin	Metformin	1.58 (0.05 to 54.60)	3 per 1000 persons	5 per 1000 persons (0 to 141 per 1000 persons)	2 more (3 fewer to 138 more)	Very low
**End-stage kidney disease**	Bolus insulin	Non-steroidal MRAs	3.05 (0.12 to 75.50)	2 per 1000 persons	6 per 1000 persons (0 to 131 per 1000 persons)	4 more (2 fewer to 129 more)	Very low
**End-stage kidney disease**	Bolus insulin	SGLT2 inhibitors	4.19 (0.17 to 103.67)	1 per 1000 persons	4 per 1000 persons (0 to 94 per 1000 persons)	3 more (1 fewer to 93 more)	Very low

Outcome	Intervention	Comparator	Odds ratio	Baseline risk	Risk if treated	Risk difference	Certainty
**End-stage kidney disease**	Bolus insulin	Standard treatments	2.55 (0.10 to 62.86)	2 per 1000 persons	5 per 1000 persons (0 to 112 per 1000 persons)	3 more (2 fewer to 110 more)	Very low
**End-stage kidney disease**	Bolus insulin	Sulfonylureas	3.73 (0.14 to 97.39)	1 per 1000 persons	4 per 1000 persons (0 to 89 per 1000 persons)	3 more (1 fewer to 88 more)	Very low
**End-stage kidney disease**	Bolus insulin	Thiazolidinediones	3.68 (0.14 to 96.44)	1 per 1000 persons	4 per 1000 persons (0 to 88 per 1000 persons)	3 more (1 fewer to 87 more)	Very low
**End-stage kidney disease**	Bolus insulin	Tirzepatide	3.77 (0.09 to 161.65)	1 per 1000 persons	4 per 1000 persons (0 to 139 per 1000 persons)	3 more (1 fewer to 138 more)	Very low
**End-stage kidney disease**	DPP-4 inhibitors	GLP-1 receptor agonists	1.25 (1.07 to 1.45)	2 per 1000 persons	2 per 1000 persons (2 to 3 per 1000 persons)	0 (0 to 1 more)	Low
**End-stage kidney disease**	DPP-4 inhibitors	Metformin	0.64 (0.14 to 2.91)	3 per 1000 persons	2 per 1000 persons (0 to 9 per 1000 persons)	1 fewer (3 fewer to 6 more)	Low
**End-stage kidney disease**	DPP-4 inhibitors	Non-steroidal MRAs	1.24 (1.07 to 1.45)	2 per 1000 persons	2 per 1000 persons (2 to 3 per 1000 persons)	0 (0 to 1 more)	Low
**End-stage kidney disease**	DPP-4 inhibitors	SGLT2 inhibitors	1.71 (1.47 to 1.99)	1 per 1000 persons	2 per 1000 persons (1 to 2 per 1000 persons)	1 more (0 to 1 more)	Low
**End-stage kidney disease**	DPP-4 inhibitors	Standard treatments	1.04 (0.93 to 1.16)	2 per 1000 persons	2 per 1000 persons (2 to 2 per 1000 persons)	0 (0 to 0)	Moderate
**End-stage kidney disease**	DPP-4 inhibitors	Sulfonylureas	1.52 (0.84 to 2.75)	1 per 1000 persons	2 per 1000 persons (1 to 3 per 1000 persons)	1 more (0 to 2 more)	Moderate
**End-stage kidney disease**	DPP-4 inhibitors	Thiazolidinediones	1.50 (0.81 to 2.77)	1 per 1000 persons	1 per 1000 persons (1 to 3 per 1000 persons)	0 (0 to 2 more)	Low
**End-stage kidney disease**	DPP-4 inhibitors	Tirzepatide	1.54 (0.21 to 11.02)	1 per 1000 persons	2 per 1000 persons (0 to 11 per 1000 persons)	1 more (1 fewer to 10 more)	Low
**End-stage kidney disease**	GLP-1 receptor agonists	Metformin	0.52 (0.11 to 2.34)	3 per 1000 persons	2 per 1000 persons (0 to 7 per 1000 persons)	1 fewer (3 fewer to 4 more)	Low
**End-stage kidney disease**	GLP-1 receptor agonists	Non-steroidal MRAs	1.00 (0.87 to 1.15)	2 per 1000 persons	2 per 1000 persons (2 to 2 per 1000 persons)	0 (0 to 0)	Low
**End-stage kidney disease**	GLP-1 receptor agonists	SGLT2 inhibitors	1.37 (1.19 to 1.59)	1 per 1000 persons	1 per 1000 persons (1 to 2 per 1000 persons)	0 (0 to 1 more)	Low

Outcome	Intervention	Comparator	Odds ratio	Baseline risk	Risk if treated	Risk difference	Certainty
**End-stage kidney disease**	GLP-1 receptor agonists	Standard treatments	0.83 (0.75 to 0.92)	2 per 1000 persons	2 per 1000 persons (2 to 2 per 1000 persons)	0 (0 to 0)	Moderate
**End-stage kidney disease**	GLP-1 receptor agonists	Sulfonylureas	1.22 (0.67 to 2.24)	1 per 1000 persons	1 per 1000 persons (1 to 2 per 1000 persons)	0 (0 to 1 more)	Low
**End-stage kidney disease**	GLP-1 receptor agonists	Thiazolidinediones	1.21 (0.64 to 2.26)	1 per 1000 persons	1 per 1000 persons (1 to 2 per 1000 persons)	0 (0 to 1 more)	Low
**End-stage kidney disease**	GLP-1 receptor agonists	Tirzepatide	1.23 (0.17 to 8.80)	1 per 1000 persons	1 per 1000 persons (0 to 9 per 1000 persons)	0 (1 fewer to 8 more)	Low
**End-stage kidney disease**	Metformin	Non-steroidal MRAs	1.93 (0.43 to 8.71)	2 per 1000 persons	4 per 1000 persons (1 to 17 per 1000 persons)	2 more (1 fewer to 15 more)	Very low
**End-stage kidney disease**	Metformin	SGLT2 inhibitors	2.65 (0.59 to 11.96)	1 per 1000 persons	3 per 1000 persons (1 to 12 per 1000 persons)	2 more (0 to 11 more)	Very low
**End-stage kidney disease**	Metformin	Standard treatments	1.61 (0.36 to 7.24)	2 per 1000 persons	3 per 1000 persons (1 to 14 per 1000 persons)	1 more (1 fewer to 12 more)	Very low
**End-stage kidney disease**	Metformin	Sulfonylureas	2.36 (0.47 to 11.91)	1 per 1000 persons	2 per 1000 persons (0 to 12 per 1000 persons)	1 more (1 fewer to 11 more)	Very low
**End-stage kidney disease**	Metformin	Thiazolidinediones	2.33 (0.46 to 11.83)	1 per 1000 persons	2 per 1000 persons (0 to 12 per 1000 persons)	1 more (1 fewer to 11 more)	Very low
**End-stage kidney disease**	Metformin	Tirzepatide	2.38 (0.20 to 28.34)	1 per 1000 persons	2 per 1000 persons (0 to 28 per 1000 persons)	1 more (1 fewer to 27 more)	Very low
**End-stage kidney disease**	Non-steroidal MRAs	SGLT2 inhibitors	1.37 (1.19 to 1.58)	1 per 1000 persons	1 per 1000 persons (1 to 2 per 1000 persons)	0 (0 to 1 more)	Low
**End-stage kidney disease**	Non-steroidal MRAs	Standard treatments	0.83 (0.75 to 0.92)	2 per 1000 persons	2 per 1000 persons (2 to 2 per 1000 persons)	0 (0 to 0)	Moderate
**End-stage kidney disease**	Non-steroidal MRAs	Sulfonylureas	1.22 (0.67 to 2.24)	1 per 1000 persons	1 per 1000 persons (1 to 2 per 1000 persons)	0 (0 to 1 more)	Low
**End-stage kidney disease**	Non-steroidal MRAs	Thiazolidinediones	1.21 (0.64 to 2.26)	1 per 1000 persons	1 per 1000 persons (1 to 2 per 1000 persons)	0 (0 to 1 more)	Low
**End-stage kidney disease**	Non-steroidal MRAs	Tirzepatide	1.23 (0.17 to 8.85)	1 per 1000 persons	1 per 1000 persons (0 to 9 per 1000 persons)	0 (1 fewer to 8 more)	Low

Outcome	Intervention	Comparator	Odds ratio	Baseline risk	Risk if treated	Risk difference	Certainty
**End-stage kidney disease**	SGLT2 inhibitors	Standard treatments	0.61 (0.55 to 0.67)	2 per 1000 persons	1 per 1000 persons (1 to 1 per 1000 persons)	1 fewer (1 fewer to 1 fewer)	Moderate
**End-stage kidney disease**	SGLT2 inhibitors	Sulfonylureas	0.89 (0.48 to 1.63)	1 per 1000 persons	1 per 1000 persons (0 to 2 per 1000 persons)	0 (1 fewer to 1 more)	Low
**End-stage kidney disease**	SGLT2 inhibitors	Thiazolidinediones	0.88 (0.47 to 1.64)	1 per 1000 persons	1 per 1000 persons (0 to 2 per 1000 persons)	0 (1 fewer to 1 more)	Low
**End-stage kidney disease**	SGLT2 inhibitors	Tirzepatide	0.90 (0.13 to 6.45)	1 per 1000 persons	1 per 1000 persons (0 to 6 per 1000 persons)	0 (1 fewer to 5 more)	Low
**End-stage kidney disease**	Sulfonylureas	Standard treatments	0.68 (0.37 to 1.24)	2 per 1000 persons	1 per 1000 persons (1 to 2 per 1000 persons)	1 fewer (1 fewer to 0)	Low
**End-stage kidney disease**	Sulfonylureas	Thiazolidinediones	0.99 (0.82 to 1.18)	1 per 1000 persons	1 per 1000 persons (1 to 1 per 1000 persons)	0 (0 to 0)	Low
**End-stage kidney disease**	Sulfonylureas	Tirzepatide	1.01 (0.13 to 7.90)	1 per 1000 persons	1 per 1000 persons (0 to 8 per 1000 persons)	0 (1 fewer to 7 more)	Low
**End-stage kidney disease**	Thiazolidinediones	Standard treatments	0.69 (0.37 to 1.28)	2 per 1000 persons	1 per 1000 persons (1 to 3 per 1000 persons)	1 fewer (1 fewer to 1 more)	Low
**End-stage kidney disease**	Thiazolidinediones	Tirzepatide	1.02 (0.13 to 8.05)	1 per 1000 persons	1 per 1000 persons (0 to 8 per 1000 persons)	0 (1 fewer to 7 more)	Low
**End-stage kidney disease**	Tirzepatide	Standard treatments	0.68 (0.09 to 4.84)	2 per 1000 persons	1 per 1000 persons (0 to 10 per 1000 persons)	1 fewer (2 fewer to 8 more)	Low
**End-stage kidney disease**	Bolus insulin	Basal insulin	2.13 (0.08 to 50.00)	2 per 1000 persons	4 per 1000 persons (0 to 91 per 1000 persons)	2 more (2 fewer to 89 more)	Very low
**End-stage kidney disease**	DPP-4 inhibitors	Basal insulin	0.87 (0.45 to 1.69)	2 per 1000 persons	2 per 1000 persons (1 to 3 per 1000 persons)	0 (1 fewer to 1 more)	Low
**End-stage kidney disease**	GLP-1 receptor agonists	Basal insulin	0.69 (0.36 to 1.33)	2 per 1000 persons	1 per 1000 persons (1 to 3 per 1000 persons)	1 fewer (1 fewer to 1 more)	Low
**End-stage kidney disease**	Metformin	Basal insulin	1.35 (0.26 to 7.14)	2 per 1000 persons	3 per 1000 persons (1 to 14 per 1000 persons)	1 more (1 fewer to 12 more)	Very low
**End-stage kidney disease**	Non-steroidal MRAs	Basal insulin	0.69 (0.36 to 1.35)	2 per 1000 persons	1 per 1000 persons (1 to 3 per 1000 persons)	1 fewer (1 fewer to 1 more)	Low

Outcome	Intervention	Comparator	Odds ratio	Baseline risk	Risk if treated	Risk difference	Certainty
**End-stage kidney disease**	SGLT2 inhibitors	Basal insulin	0.51 (0.26 to 0.98)	2 per 1000 persons	1 per 1000 persons (1 to 2 per 1000 persons)	1 fewer (1 fewer to 0)	Low
**End-stage kidney disease**	Standard treatments	Basal insulin	0.83 (0.43 to 1.61)	2 per 1000 persons	2 per 1000 persons (1 to 3 per 1000 persons)	0 (1 fewer to 1 more)	Low
**End-stage kidney disease**	Sulfonylureas	Basal insulin	0.57 (0.23 to 1.39)	2 per 1000 persons	1 per 1000 persons (0 to 3 per 1000 persons)	1 fewer (2 fewer to 1 more)	Low
**End-stage kidney disease**	Thiazolidinediones	Basal insulin	0.58 (0.23 to 1.43)	2 per 1000 persons	1 per 1000 persons (0 to 3 per 1000 persons)	1 fewer (2 fewer to 1 more)	Low
**End-stage kidney disease**	Tirzepatide	Basal insulin	0.56 (0.08 to 3.85)	2 per 1000 persons	1 per 1000 persons (0 to 8 per 1000 persons)	1 fewer (2 fewer to 6 more)	Moderate
**End-stage kidney disease**	DPP-4 inhibitors	Bolus insulin	0.41 (0.02 to 10.00)	5 per 1000 persons	2 per 1000 persons (0 to 48 per 1000 persons)	3 fewer (5 fewer to 43 more)	Very low
**End-stage kidney disease**	GLP-1 receptor agonists	Bolus insulin	0.33 (0.01 to 8.33)	5 per 1000 persons	2 per 1000 persons (0 to 40 per 1000 persons)	3 fewer (5 fewer to 35 more)	Very low
**End-stage kidney disease**	Metformin	Bolus insulin	0.63 (0.02 to 20.00)	5 per 1000 persons	3 per 1000 persons (0 to 91 per 1000 persons)	2 fewer (5 fewer to 86 more)	Very low
**End-stage kidney disease**	Non-steroidal MRAs	Bolus insulin	0.33 (0.01 to 8.33)	5 per 1000 persons	2 per 1000 persons (0 to 40 per 1000 persons)	3 fewer (5 fewer to 35 more)	Very low
**End-stage kidney disease**	SGLT2 inhibitors	Bolus insulin	0.24 (0.01 to 5.88)	5 per 1000 persons	1 per 1000 persons (0 to 29 per 1000 persons)	4 fewer (5 fewer to 24 more)	Very low
**End-stage kidney disease**	Standard treatments	Bolus insulin	0.39 (0.02 to 10.00)	5 per 1000 persons	2 per 1000 persons (0 to 48 per 1000 persons)	3 fewer (5 fewer to 43 more)	Very low
**End-stage kidney disease**	Sulfonylureas	Bolus insulin	0.27 (0.01 to 7.14)	5 per 1000 persons	1 per 1000 persons (0 to 35 per 1000 persons)	4 fewer (5 fewer to 30 more)	Very low
**End-stage kidney disease**	Thiazolidinediones	Bolus insulin	0.27 (0.01 to 7.14)	5 per 1000 persons	1 per 1000 persons (0 to 35 per 1000 persons)	4 fewer (5 fewer to 30 more)	Very low
**End-stage kidney disease**	Tirzepatide	Bolus insulin	0.27 (0.01 to 11.11)	5 per 1000 persons	1 per 1000 persons (0 to 53 per 1000 persons)	4 fewer (5 fewer to 48 more)	Very low
**End-stage kidney disease**	GLP-1 receptor agonists	DPP-4 inhibitors	0.80 (0.69 to 0.93)	2 per 1000 persons	2 per 1000 persons (1 to 2 per 1000 persons)	0 (1 fewer to 0)	Low

Outcome	Intervention	Comparator	Odds ratio	Baseline risk	Risk if treated	Risk difference	Certainty
**End-stage kidney disease**	Metformin	DPP-4 inhibitors	1.56 (0.34 to 7.14)	2 per 1000 persons	3 per 1000 persons (1 to 14 per 1000 persons)	1 more (1 fewer to 12 more)	Very low
**End-stage kidney disease**	Non-steroidal MRAs	DPP-4 inhibitors	0.81 (0.69 to 0.93)	2 per 1000 persons	2 per 1000 persons (1 to 2 per 1000 persons)	0 (1 fewer to 0)	Low
**End-stage kidney disease**	SGLT2 inhibitors	DPP-4 inhibitors	0.58 (0.50 to 0.68)	2 per 1000 persons	1 per 1000 persons (1 to 1 per 1000 persons)	1 fewer (1 fewer to 1 fewer)	Low
**End-stage kidney disease**	Standard treatments	DPP-4 inhibitors	0.96 (0.86 to 1.08)	2 per 1000 persons	2 per 1000 persons (2 to 2 per 1000 persons)	0 (0 to 0)	Moderate
**End-stage kidney disease**	Sulfonylureas	DPP-4 inhibitors	0.66 (0.36 to 1.19)	2 per 1000 persons	1 per 1000 persons (1 to 2 per 1000 persons)	1 fewer (1 fewer to 0)	Moderate
**End-stage kidney disease**	Thiazolidinediones	DPP-4 inhibitors	0.67 (0.36 to 1.23)	2 per 1000 persons	1 per 1000 persons (1 to 2 per 1000 persons)	1 fewer (1 fewer to 0)	Low
**End-stage kidney disease**	Tirzepatide	DPP-4 inhibitors	0.65 (0.09 to 4.76)	2 per 1000 persons	1 per 1000 persons (0 to 9 per 1000 persons)	1 fewer (2 fewer to 7 more)	Low
**End-stage kidney disease**	Metformin	GLP-1 receptor agonists	1.92 (0.43 to 9.09)	2 per 1000 persons	4 per 1000 persons (1 to 18 per 1000 persons)	2 more (1 fewer to 16 more)	Very low
**End-stage kidney disease**	Non-steroidal MRAs	GLP-1 receptor agonists	1.00 (0.87 to 1.15)	2 per 1000 persons	2 per 1000 persons (2 to 2 per 1000 persons)	0 (0 to 0)	Low
**End-stage kidney disease**	SGLT2 inhibitors	GLP-1 receptor agonists	0.73 (0.63 to 0.84)	2 per 1000 persons	1 per 1000 persons (1 to 2 per 1000 persons)	1 fewer (1 fewer to 0)	Low
**End-stage kidney disease**	Standard treatments	GLP-1 receptor agonists	1.20 (1.09 to 1.33)	2 per 1000 persons	2 per 1000 persons (2 to 3 per 1000 persons)	0 (0 to 1 more)	Moderate
**End-stage kidney disease**	Sulfonylureas	GLP-1 receptor agonists	0.82 (0.45 to 1.49)	2 per 1000 persons	2 per 1000 persons (1 to 3 per 1000 persons)	0 (1 fewer to 1 more)	Low
**End-stage kidney disease**	Thiazolidinediones	GLP-1 receptor agonists	0.83 (0.44 to 1.56)	2 per 1000 persons	2 per 1000 persons (1 to 3 per 1000 persons)	0 (1 fewer to 1 more)	Low
**End-stage kidney disease**	Tirzepatide	GLP-1 receptor agonists	0.81 (0.11 to 5.88)	2 per 1000 persons	2 per 1000 persons (0 to 12 per 1000 persons)	0 (2 fewer to 10 more)	Low
**End-stage kidney disease**	Non-steroidal MRAs	Metformin	0.52 (0.11 to 2.33)	3 per 1000 persons	2 per 1000 persons (0 to 7 per 1000 persons)	1 fewer (3 fewer to 4 more)	Low

Outcome	Intervention	Comparator	Odds ratio	Baseline risk	Risk if treated	Risk difference	Certainty
**End-stage kidney disease**	SGLT2 inhibitors	Metformin	0.38 (0.08 to 1.69)	3 per 1000 persons	1 per 1000 persons (0 to 5 per 1000 persons)	2 fewer (3 fewer to 2 more)	Low
**End-stage kidney disease**	Standard treatments	Metformin	0.62 (0.14 to 2.78)	3 per 1000 persons	2 per 1000 persons (0 to 8 per 1000 persons)	1 fewer (3 fewer to 5 more)	Low
**End-stage kidney disease**	Sulfonylureas	Metformin	0.42 (0.08 to 2.13)	3 per 1000 persons	1 per 1000 persons (0 to 6 per 1000 persons)	2 fewer (3 fewer to 3 more)	Low
**End-stage kidney disease**	Thiazolidinediones	Metformin	0.43 (0.08 to 2.17)	3 per 1000 persons	1 per 1000 persons (0 to 6 per 1000 persons)	2 fewer (3 fewer to 3 more)	Low
**End-stage kidney disease**	Tirzepatide	Metformin	0.42 (0.04 to 5.00)	3 per 1000 persons	1 per 1000 persons (0 to 15 per 1000 persons)	2 fewer (3 fewer to 12 more)	Very low
**End-stage kidney disease**	SGLT2 inhibitors	Non-steroidal MRAs	0.73 (0.63 to 0.84)	2 per 1000 persons	1 per 1000 persons (1 to 2 per 1000 persons)	1 fewer (1 fewer to 0)	Low
**End-stage kidney disease**	Standard treatments	Non-steroidal MRAs	1.20 (1.09 to 1.33)	2 per 1000 persons	2 per 1000 persons (2 to 3 per 1000 persons)	0 (0 to 1 more)	Moderate
**End-stage kidney disease**	Sulfonylureas	Non-steroidal MRAs	0.82 (0.45 to 1.49)	2 per 1000 persons	2 per 1000 persons (1 to 3 per 1000 persons)	0 (1 fewer to 1 more)	Low
**End-stage kidney disease**	Thiazolidinediones	Non-steroidal MRAs	0.83 (0.44 to 1.56)	2 per 1000 persons	2 per 1000 persons (1 to 3 per 1000 persons)	0 (1 fewer to 1 more)	Low
**End-stage kidney disease**	Tirzepatide	Non-steroidal MRAs	0.81 (0.11 to 5.88)	2 per 1000 persons	2 per 1000 persons (0 to 12 per 1000 persons)	0 (2 fewer to 10 more)	Low
**End-stage kidney disease**	Standard treatments	SGLT2 inhibitors	1.64 (1.49 to 1.82)	1 per 1000 persons	2 per 1000 persons (1 to 2 per 1000 persons)	1 more (0 to 1 more)	Moderate
**End-stage kidney disease**	Sulfonylureas	SGLT2 inhibitors	1.12 (0.61 to 2.08)	1 per 1000 persons	1 per 1000 persons (1 to 2 per 1000 persons)	0 (0 to 1 more)	Low
**End-stage kidney disease**	Thiazolidinediones	SGLT2 inhibitors	1.14 (0.61 to 2.13)	1 per 1000 persons	1 per 1000 persons (1 to 2 per 1000 persons)	0 (0 to 1 more)	Low
**End-stage kidney disease**	Tirzepatide	SGLT2 inhibitors	1.11 (0.16 to 7.69)	1 per 1000 persons	1 per 1000 persons (0 to 8 per 1000 persons)	0 (1 fewer to 7 more)	Low
**End-stage kidney disease**	Standard treatments	Sulfonylureas	1.47 (0.81 to 2.70)	1 per 1000 persons	1 per 1000 persons (1 to 3 per 1000 persons)	0 (0 to 2 more)	Low

Outcome	Intervention	Comparator	Odds ratio	Baseline risk	Risk if treated	Risk difference	Certainty
**End-stage kidney disease**	Thiazolidinediones	Sulfonylureas	1.01 (0.85 to 1.22)	1 per 1000 persons	1 per 1000 persons (1 to 1 per 1000 persons)	0 (0 to 0)	Low
**End-stage kidney disease**	Tirzepatide	Sulfonylureas	0.99 (0.13 to 7.69)	1 per 1000 persons	1 per 1000 persons (0 to 8 per 1000 persons)	0 (1 fewer to 7 more)	Low
**End-stage kidney disease**	Standard treatments	Thiazolidinediones	1.45 (0.78 to 2.70)	1 per 1000 persons	1 per 1000 persons (1 to 3 per 1000 persons)	0 (0 to 2 more)	Low
**End-stage kidney disease**	Tirzepatide	Thiazolidinediones	0.98 (0.12 to 7.69)	1 per 1000 persons	1 per 1000 persons (0 to 8 per 1000 persons)	0 (1 fewer to 7 more)	Low
**End-stage kidney disease**	Standard treatments	Tirzepatide	1.47 (0.21 to 11.11)	1 per 1000 persons	1 per 1000 persons (0 to 11 per 1000 persons)	0 (1 fewer to 10 more)	Low
**Severe hypoglycaemia**	Alpha-glucosidase inhibitors	Basal insulin	0.54 (0.13 to 2.33)	69 per 1000 persons	38 per 1000 persons (10 to 147 per 1000 persons)	31 fewer (59 fewer to 78 more)	Very low
**Severe hypoglycaemia**	Alpha-glucosidase inhibitors	Basal-Bolus insulin	0.26 (0.03 to 2.14)	133 per 1000 persons	38 per 1000 persons (5 to 247 per 1000 persons)	95 fewer (128 fewer to 114 more)	Very low
**Severe hypoglycaemia**	Alpha-glucosidase inhibitors	Bolus insulin	0.53 (0.11 to 2.52)	71 per 1000 persons	39 per 1000 persons (8 to 161 per 1000 persons)	32 fewer (63 fewer to 90 more)	Very low
**Severe hypoglycaemia**	Alpha-glucosidase inhibitors	DPP-4 inhibitors	1.17 (0.28 to 4.90)	33 per 1000 persons	38 per 1000 persons (9 to 143 per 1000 persons)	5 more (24 fewer to 110 more)	Very low
**Severe hypoglycaemia**	Alpha-glucosidase inhibitors	GLP-1 receptor agonists	1.33 (0.32 to 5.58)	29 per 1000 persons	38 per 1000 persons (9 to 143 per 1000 persons)	9 more (20 fewer to 114 more)	Very low
**Severe hypoglycaemia**	Alpha-glucosidase inhibitors	Meglitinides	0.40 (0.07 to 2.52)	90 per 1000 persons	38 per 1000 persons (7 to 200 per 1000 persons)	52 fewer (83 fewer to 110 more)	Very low
**Severe hypoglycaemia**	Alpha-glucosidase inhibitors	Metformin	0.75 (0.16 to 3.58)	51 per 1000 persons	39 per 1000 persons (9 to 161 per 1000 persons)	12 fewer (42 fewer to 110 more)	Very low
**Severe hypoglycaemia**	Alpha-glucosidase inhibitors	Non-steroidal MRAs	2.03 (0.46 to 8.98)	19 per 1000 persons	38 per 1000 persons (9 to 148 per 1000 persons)	19 more (10 fewer to 129 more)	Low
**Severe hypoglycaemia**	Alpha-glucosidase inhibitors	SGLT2 inhibitors	1.45 (0.34 to 6.07)	27 per 1000 persons	39 per 1000 persons (9 to 144 per 1000 persons)	12 more (18 fewer to 117 more)	Very low
**Severe hypoglycaemia**	Alpha-glucosidase inhibitors	Standard treatments	1.30 (0.31 to 5.43)	30 per 1000 persons	39 per 1000 persons (9 to 144 per 1000 persons)	9 more (21 fewer to 114 more)	Low

Outcome	Intervention	Comparator	Odds ratio	Baseline risk	Risk if treated	Risk difference	Certainty
**Severe hypoglycaemia**	Alpha-glucosidase inhibitors	Sulfonylureas	0.25 (0.06 to 1.06)	139 per 1000 persons	39 per 1000 persons (10 to 146 per 1000 persons)	100 fewer (129 fewer to 7 more)	Low
**Severe hypoglycaemia**	Alpha-glucosidase inhibitors	Thiazolidinediones	0.91 (0.21 to 4.01)	42 per 1000 persons	38 per 1000 persons (9 to 150 per 1000 persons)	4 fewer (33 fewer to 108 more)	Very low
**Severe hypoglycaemia**	Alpha-glucosidase inhibitors	Tirzepatide	1.15 (0.20 to 6.56)	34 per 1000 persons	39 per 1000 persons (7 to 188 per 1000 persons)	5 more (27 fewer to 154 more)	Very low
**Severe hypoglycaemia**	Basal insulin	Basal-Bolus insulin	0.48 (0.11 to 2.22)	133 per 1000 persons	69 per 1000 persons (17 to 254 per 1000 persons)	64 fewer (116 fewer to 121 more)	Very low
**Severe hypoglycaemia**	Basal insulin	Bolus insulin	0.97 (0.49 to 1.91)	71 per 1000 persons	69 per 1000 persons (36 to 127 per 1000 persons)	2 fewer (35 fewer to 56 more)	Very low
**Severe hypoglycaemia**	Basal insulin	DPP-4 inhibitors	2.14 (1.62 to 2.85)	33 per 1000 persons	68 per 1000 persons (52 to 89 per 1000 persons)	35 more (19 more to 56 more)	Moderate
**Severe hypoglycaemia**	Basal insulin	GLP-1 receptor agonists	2.44 (1.88 to 3.17)	29 per 1000 persons	68 per 1000 persons (53 to 86 per 1000 persons)	39 more (24 more to 57 more)	Moderate
**Severe hypoglycaemia**	Basal insulin	Meglitinides	0.74 (0.22 to 2.55)	90 per 1000 persons	68 per 1000 persons (21 to 201 per 1000 persons)	22 fewer (69 fewer to 111 more)	Very low
**Severe hypoglycaemia**	Basal insulin	Metformin	1.37 (0.67 to 2.80)	51 per 1000 persons	69 per 1000 persons (35 to 131 per 1000 persons)	18 more (16 fewer to 80 more)	Very low
**Severe hypoglycaemia**	Basal insulin	Non-steroidal MRAs	3.72 (2.28 to 6.08)	19 per 1000 persons	67 per 1000 persons (42 to 105 per 1000 persons)	48 more (23 more to 86 more)	Moderate
**Severe hypoglycaemia**	Basal insulin	SGLT2 inhibitors	2.66 (1.97 to 3.58)	27 per 1000 persons	69 per 1000 persons (52 to 90 per 1000 persons)	42 more (25 more to 63 more)	Moderate
**Severe hypoglycaemia**	Basal insulin	Standard treatments	2.38 (1.82 to 3.12)	30 per 1000 persons	69 per 1000 persons (53 to 88 per 1000 persons)	39 more (23 more to 58 more)	Moderate
**Severe hypoglycaemia**	Basal insulin	Sulfonylureas	0.46 (0.32 to 0.66)	139 per 1000 persons	69 per 1000 persons (49 to 96 per 1000 persons)	70 fewer (90 fewer to 43 fewer)	Moderate
**Severe hypoglycaemia**	Basal insulin	Thiazolidinediones	1.67 (1.07 to 2.63)	42 per 1000 persons	68 per 1000 persons (45 to 103 per 1000 persons)	26 more (3 more to 61 more)	Moderate
**Severe hypoglycaemia**	Basal insulin	Tirzepatide	2.12 (0.81 to 5.56)	34 per 1000 persons	69 per 1000 persons (28 to 164 per 1000 persons)	35 more (6 fewer to 130 more)	Moderate

Outcome	Intervention	Comparator	Odds ratio	Baseline risk	Risk if treated	Risk difference	Certainty
**Severe hypoglycaemia**	Basal-Bolus insulin	Bolus insulin	2.01 (0.38 to 10.53)	71 per 1000 persons	133 per 1000 persons (28 to 446 per 1000 persons)	62 more (43 fewer to 375 more)	Very low
**Severe hypoglycaemia**	Basal-Bolus insulin	DPP-4 inhibitors	4.44 (0.95 to 20.70)	33 per 1000 persons	132 per 1000 persons (31 to 414 per 1000 persons)	99 more (2 fewer to 381 more)	Low
**Severe hypoglycaemia**	Basal-Bolus insulin	GLP-1 receptor agonists	5.06 (1.09 to 23.49)	29 per 1000 persons	131 per 1000 persons (32 to 412 per 1000 persons)	102 more (3 more to 383 more)	Low
**Severe hypoglycaemia**	Basal-Bolus insulin	Meglitinides	1.54 (0.22 to 10.84)	90 per 1000 persons	132 per 1000 persons (21 to 517 per 1000 persons)	42 more (69 fewer to 427 more)	Very low
**Severe hypoglycaemia**	Basal-Bolus insulin	Metformin	2.85 (0.54 to 15.16)	51 per 1000 persons	133 per 1000 persons (28 to 449 per 1000 persons)	82 more (23 fewer to 398 more)	Very low
**Severe hypoglycaemia**	Basal-Bolus insulin	Non-steroidal MRAs	7.71 (1.57 to 37.81)	19 per 1000 persons	130 per 1000 persons (30 to 423 per 1000 persons)	111 more (11 more to 404 more)	Moderate
**Severe hypoglycaemia**	Basal-Bolus insulin	SGLT2 inhibitors	5.50 (1.18 to 25.70)	27 per 1000 persons	132 per 1000 persons (32 to 416 per 1000 persons)	105 more (5 more to 389 more)	Moderate
**Severe hypoglycaemia**	Basal-Bolus insulin	Standard treatments	4.94 (1.06 to 22.96)	30 per 1000 persons	133 per 1000 persons (32 to 415 per 1000 persons)	103 more (2 more to 385 more)	Moderate
**Severe hypoglycaemia**	Basal-Bolus insulin	Sulfonylureas	0.95 (0.20 to 4.50)	139 per 1000 persons	133 per 1000 persons (31 to 421 per 1000 persons)	6 fewer (108 fewer to 282 more)	Very low
**Severe hypoglycaemia**	Basal-Bolus insulin	Thiazolidinediones	3.47 (0.72 to 16.84)	42 per 1000 persons	132 per 1000 persons (31 to 425 per 1000 persons)	90 more (11 fewer to 383 more)	Very low
**Severe hypoglycaemia**	Basal-Bolus insulin	Tirzepatide	4.39 (0.72 to 26.62)	34 per 1000 persons	134 per 1000 persons (25 to 484 per 1000 persons)	100 more (9 fewer to 450 more)	Low
**Severe hypoglycaemia**	Bolus insulin	DPP-4 inhibitors	2.21 (1.17 to 4.20)	33 per 1000 persons	70 per 1000 persons (38 to 125 per 1000 persons)	37 more (5 more to 92 more)	Low
**Severe hypoglycaemia**	Bolus insulin	GLP-1 receptor agonists	2.52 (1.35 to 4.72)	29 per 1000 persons	70 per 1000 persons (39 to 124 per 1000 persons)	41 more (10 more to 95 more)	Low
**Severe hypoglycaemia**	Bolus insulin	Meglitinides	0.77 (0.20 to 2.99)	90 per 1000 persons	71 per 1000 persons (19 to 228 per 1000 persons)	19 fewer (71 fewer to 138 more)	Very low
**Severe hypoglycaemia**	Bolus insulin	Metformin	1.42 (0.57 to 3.54)	51 per 1000 persons	71 per 1000 persons (30 to 160 per 1000 persons)	20 more (21 fewer to 109 more)	Very low

Outcome	Intervention	Comparator	Odds ratio	Baseline risk	Risk if treated	Risk difference	Certainty
**Severe hypoglycaemia**	Bolus insulin	Non-steroidal MRAs	3.84 (1.81 to 8.16)	19 per 1000 persons	69 per 1000 persons (34 to 136 per 1000 persons)	50 more (15 more to 117 more)	Low
**Severe hypoglycaemia**	Bolus insulin	SGLT2 inhibitors	2.74 (1.44 to 5.22)	27 per 1000 persons	71 per 1000 persons (38 to 127 per 1000 persons)	44 more (11 more to 100 more)	Moderate
**Severe hypoglycaemia**	Bolus insulin	Standard treatments	2.46 (1.31 to 4.63)	30 per 1000 persons	71 per 1000 persons (39 to 125 per 1000 persons)	41 more (9 more to 95 more)	Moderate
**Severe hypoglycaemia**	Bolus insulin	Sulfonylureas	0.47 (0.24 to 0.94)	139 per 1000 persons	71 per 1000 persons (37 to 132 per 1000 persons)	68 fewer (102 fewer to 7 fewer)	Moderate
**Severe hypoglycaemia**	Bolus insulin	Thiazolidinediones	1.73 (0.82 to 3.62)	42 per 1000 persons	70 per 1000 persons (35 to 137 per 1000 persons)	28 more (7 fewer to 95 more)	Very low
**Severe hypoglycaemia**	Bolus insulin	Tirzepatide	2.19 (0.68 to 7.03)	34 per 1000 persons	72 per 1000 persons (23 to 198 per 1000 persons)	38 more (11 fewer to 164 more)	Very low
**Severe hypoglycaemia**	DPP-4 inhibitors	GLP-1 receptor agonists	1.14 (1.00 to 1.30)	29 per 1000 persons	33 per 1000 persons (29 to 37 per 1000 persons)	4 more (0 to 8 more)	Moderate
**Severe hypoglycaemia**	DPP-4 inhibitors	Meglitinides	0.35 (0.10 to 1.16)	90 per 1000 persons	33 per 1000 persons (10 to 103 per 1000 persons)	57 fewer (80 fewer to 13 more)	Very low
**Severe hypoglycaemia**	DPP-4 inhibitors	Metformin	0.64 (0.33 to 1.25)	51 per 1000 persons	33 per 1000 persons (17 to 63 per 1000 persons)	18 fewer (34 fewer to 12 more)	Very low
**Severe hypoglycaemia**	DPP-4 inhibitors	Non-steroidal MRAs	1.74 (1.14 to 2.65)	19 per 1000 persons	33 per 1000 persons (22 to 49 per 1000 persons)	14 more (3 more to 30 more)	Moderate
**Severe hypoglycaemia**	DPP-4 inhibitors	SGLT2 inhibitors	1.24 (1.05 to 1.46)	27 per 1000 persons	33 per 1000 persons (28 to 39 per 1000 persons)	6 more (1 more to 12 more)	Low
**Severe hypoglycaemia**	DPP-4 inhibitors	Standard treatments	1.11 (1.00 to 1.23)	30 per 1000 persons	33 per 1000 persons (30 to 37 per 1000 persons)	3 more (0 to 7 more)	Moderate
**Severe hypoglycaemia**	DPP-4 inhibitors	Sulfonylureas	0.21 (0.16 to 0.29)	139 per 1000 persons	33 per 1000 persons (25 to 45 per 1000 persons)	106 fewer (114 fewer to 94 fewer)	High
**Severe hypoglycaemia**	DPP-4 inhibitors	Thiazolidinediones	0.78 (0.52 to 1.16)	42 per 1000 persons	33 per 1000 persons (22 to 48 per 1000 persons)	9 fewer (20 fewer to 6 more)	Low
**Severe hypoglycaemia**	DPP-4 inhibitors	Tirzepatide	0.99 (0.37 to 2.66)	34 per 1000 persons	34 per 1000 persons (13 to 86 per 1000 persons)	0 (21 fewer to 52 more)	Very low

Outcome	Intervention	Comparator	Odds ratio	Baseline risk	Risk if treated	Risk difference	Certainty
**Severe hypoglycaemia**	GLP-1 receptor agonists	Meglitinides	0.30 (0.09 to 1.02)	90 per 1000 persons	29 per 1000 persons (9 to 92 per 1000 persons)	61 fewer (81 fewer to 2 more)	Low
**Severe hypoglycaemia**	GLP-1 receptor agonists	Metformin	0.56 (0.29 to 1.10)	51 per 1000 persons	29 per 1000 persons (15 to 56 per 1000 persons)	22 fewer (36 fewer to 5 more)	Low
**Severe hypoglycaemia**	GLP-1 receptor agonists	Non-steroidal MRAs	1.52 (1.00 to 2.31)	19 per 1000 persons	29 per 1000 persons (19 to 43 per 1000 persons)	10 more (0 to 24 more)	Moderate
**Severe hypoglycaemia**	GLP-1 receptor agonists	SGLT2 inhibitors	1.09 (0.93 to 1.27)	27 per 1000 persons	29 per 1000 persons (25 to 34 per 1000 persons)	2 more (2 fewer to 7 more)	Moderate
**Severe hypoglycaemia**	GLP-1 receptor agonists	Standard treatments	0.98 (0.90 to 1.06)	30 per 1000 persons	29 per 1000 persons (27 to 32 per 1000 persons)	1 fewer (3 fewer to 2 more)	High
**Severe hypoglycaemia**	GLP-1 receptor agonists	Sulfonylureas	0.19 (0.14 to 0.25)	139 per 1000 persons	30 per 1000 persons (22 to 39 per 1000 persons)	109 fewer (117 fewer to 100 fewer)	Very low
**Severe hypoglycaemia**	GLP-1 receptor agonists	Thiazolidinediones	0.69 (0.46 to 1.02)	42 per 1000 persons	29 per 1000 persons (20 to 43 per 1000 persons)	13 fewer (22 fewer to 1 more)	Low
**Severe hypoglycaemia**	GLP-1 receptor agonists	Tirzepatide	0.87 (0.32 to 2.32)	34 per 1000 persons	30 per 1000 persons (11 to 75 per 1000 persons)	4 fewer (23 fewer to 41 more)	Very low
**Severe hypoglycaemia**	Meglitinides	Metformin	1.85 (0.61 to 5.63)	51 per 1000 persons	90 per 1000 persons (32 to 232 per 1000 persons)	39 more (19 fewer to 181 more)	Low
**Severe hypoglycaemia**	Meglitinides	Non-steroidal MRAs	5.01 (1.40 to 17.96)	19 per 1000 persons	88 per 1000 persons (26 to 258 per 1000 persons)	69 more (7 more to 239 more)	Moderate
**Severe hypoglycaemia**	Meglitinides	SGLT2 inhibitors	3.58 (1.06 to 12.02)	27 per 1000 persons	90 per 1000 persons (29 to 250 per 1000 persons)	63 more (2 more to 223 more)	Moderate
**Severe hypoglycaemia**	Meglitinides	Standard treatments	3.21 (0.96 to 10.75)	30 per 1000 persons	90 per 1000 persons (29 to 250 per 1000 persons)	60 more (1 fewer to 220 more)	Low
**Severe hypoglycaemia**	Meglitinides	Sulfonylureas	0.62 (0.18 to 2.08)	139 per 1000 persons	91 per 1000 persons (28 to 251 per 1000 persons)	48 fewer (111 fewer to 112 more)	Very low
**Severe hypoglycaemia**	Meglitinides	Thiazolidinediones	2.26 (0.64 to 7.99)	42 per 1000 persons	90 per 1000 persons (27 to 259 per 1000 persons)	48 more (15 fewer to 217 more)	Very low
**Severe hypoglycaemia**	Meglitinides	Tirzepatide	2.85 (0.60 to 13.55)	34 per 1000 persons	91 per 1000 persons (21 to 323 per 1000 persons)	57 more (13 fewer to 289 more)	Very low

Outcome	Intervention	Comparator	Odds ratio	Baseline risk	Risk if treated	Risk difference	Certainty
**Severe hypoglycaemia**	Metformin	Non-steroidal MRAs	2.71 (1.24 to 5.90)	19 per 1000 persons	50 per 1000 persons (23 to 103 per 1000 persons)	31 more (4 more to 84 more)	Low
**Severe hypoglycaemia**	Metformin	SGLT2 inhibitors	1.93 (0.99 to 3.78)	27 per 1000 persons	51 per 1000 persons (27 to 95 per 1000 persons)	24 more (0 to 68 more)	Moderate
**Severe hypoglycaemia**	Metformin	Standard treatments	1.73 (0.89 to 3.37)	30 per 1000 persons	51 per 1000 persons (27 to 94 per 1000 persons)	21 more (3 fewer to 64 more)	Low
**Severe hypoglycaemia**	Metformin	Sulfonylureas	0.33 (0.17 to 0.67)	139 per 1000 persons	51 per 1000 persons (27 to 98 per 1000 persons)	88 fewer (112 fewer to 41 fewer)	Moderate
**Severe hypoglycaemia**	Metformin	Thiazolidinediones	1.22 (0.57 to 2.61)	42 per 1000 persons	51 per 1000 persons (24 to 103 per 1000 persons)	9 more (18 fewer to 61 more)	Very low
**Severe hypoglycaemia**	Metformin	Tirzepatide	1.54 (0.47 to 5.06)	34 per 1000 persons	51 per 1000 persons (16 to 151 per 1000 persons)	17 more (18 fewer to 117 more)	Very low
**Severe hypoglycaemia**	Non-steroidal MRAs	SGLT2 inhibitors	0.71 (0.46 to 1.10)	27 per 1000 persons	19 per 1000 persons (13 to 30 per 1000 persons)	8 fewer (14 fewer to 3 more)	Low
**Severe hypoglycaemia**	Non-steroidal MRAs	Standard treatments	0.64 (0.43 to 0.96)	30 per 1000 persons	19 per 1000 persons (13 to 29 per 1000 persons)	11 fewer (17 fewer to 1 fewer)	Moderate
**Severe hypoglycaemia**	Non-steroidal MRAs	Sulfonylureas	0.12 (0.07 to 0.20)	139 per 1000 persons	19 per 1000 persons (11 to 31 per 1000 persons)	120 fewer (128 fewer to 108 fewer)	Moderate
**Severe hypoglycaemia**	Non-steroidal MRAs	Thiazolidinediones	0.45 (0.26 to 0.79)	42 per 1000 persons	19 per 1000 persons (11 to 33 per 1000 persons)	23 fewer (31 fewer to 9 fewer)	Moderate
**Severe hypoglycaemia**	Non-steroidal MRAs	Tirzepatide	0.57 (0.20 to 1.66)	34 per 1000 persons	20 per 1000 persons (7 to 55 per 1000 persons)	14 fewer (27 fewer to 21 more)	Very low
**Severe hypoglycaemia**	SGLT2 inhibitors	Standard treatments	0.90 (0.79 to 1.02)	30 per 1000 persons	27 per 1000 persons (24 to 31 per 1000 persons)	3 fewer (6 fewer to 1 more)	High
**Severe hypoglycaemia**	SGLT2 inhibitors	Sulfonylureas	0.17 (0.13 to 0.24)	139 per 1000 persons	27 per 1000 persons (21 to 37 per 1000 persons)	112 fewer (118 fewer to 102 fewer)	Moderate
**Severe hypoglycaemia**	SGLT2 inhibitors	Thiazolidinediones	0.63 (0.42 to 0.95)	42 per 1000 persons	27 per 1000 persons (18 to 40 per 1000 persons)	15 fewer (24 fewer to 2 fewer)	Moderate
**Severe hypoglycaemia**	SGLT2 inhibitors	Tirzepatide	0.80 (0.30 to 2.16)	34 per 1000 persons	27 per 1000 persons (10 to 71 per 1000 persons)	7 fewer (24 fewer to 37 more)	Very low

Outcome	Intervention	Comparator	Odds ratio	Baseline risk	Risk if treated	Risk difference	Certainty
**Severe hypoglycaemia**	Sulfonylureas	Standard treatments	5.22 (3.88 to 7.01)	30 per 1000 persons	139 per 1000 persons (107 to 178 per 1000 persons)	109 more (77 more to 148 more)	Moderate
**Severe hypoglycaemia**	Sulfonylureas	Thiazolidinediones	3.66 (2.31 to 5.80)	42 per 1000 persons	138 per 1000 persons (92 to 203 per 1000 persons)	96 more (50 more to 161 more)	Moderate
**Severe hypoglycaemia**	Sulfonylureas	Tirzepatide	4.64 (1.67 to 12.86)	34 per 1000 persons	140 per 1000 persons (56 to 312 per 1000 persons)	106 more (22 more to 278 more)	Moderate
**Severe hypoglycaemia**	Thiazolidinediones	Standard treatments	1.42 (0.97 to 2.10)	30 per 1000 persons	42 per 1000 persons (29 to 61 per 1000 persons)	12 more (1 fewer to 31 more)	Low
**Severe hypoglycaemia**	Thiazolidinediones	Tirzepatide	1.27 (0.44 to 3.63)	34 per 1000 persons	43 per 1000 persons (15 to 113 per 1000 persons)	9 more (19 fewer to 79 more)	Very low
**Severe hypoglycaemia**	Tirzepatide	Standard treatments	1.13 (0.42 to 3.02)	30 per 1000 persons	34 per 1000 persons (13 to 85 per 1000 persons)	4 more (17 fewer to 55 more)	Very low
**Severe hypoglycaemia**	Basal insulin	Alpha-glucosidase inhibitors	1.85 (0.43 to 7.69)	39 per 1000 persons	70 per 1000 persons (17 to 238 per 1000 persons)	31 more (22 fewer to 199 more)	Very low
**Severe hypoglycaemia**	Basal-Bolus insulin	Alpha-glucosidase inhibitors	3.85 (0.47 to 33.33)	39 per 1000 persons	135 per 1000 persons (19 to 575 per 1000 persons)	96 more (20 fewer to 536 more)	Very low
**Severe hypoglycaemia**	Bolus insulin	Alpha-glucosidase inhibitors	1.89 (0.40 to 9.09)	39 per 1000 persons	71 per 1000 persons (16 to 270 per 1000 persons)	32 more (23 fewer to 231 more)	Very low
**Severe hypoglycaemia**	DPP-4 inhibitors	Alpha-glucosidase inhibitors	0.85 (0.20 to 3.57)	39 per 1000 persons	34 per 1000 persons (8 to 127 per 1000 persons)	5 fewer (31 fewer to 88 more)	Very low
**Severe hypoglycaemia**	GLP-1 receptor agonists	Alpha-glucosidase inhibitors	0.75 (0.18 to 3.12)	39 per 1000 persons	30 per 1000 persons (7 to 113 per 1000 persons)	9 fewer (32 fewer to 74 more)	Very low
**Severe hypoglycaemia**	Meglitinides	Alpha-glucosidase inhibitors	2.50 (0.40 to 14.29)	39 per 1000 persons	92 per 1000 persons (16 to 367 per 1000 persons)	53 more (23 fewer to 328 more)	Very low
**Severe hypoglycaemia**	Metformin	Alpha-glucosidase inhibitors	1.33 (0.28 to 6.25)	39 per 1000 persons	51 per 1000 persons (11 to 202 per 1000 persons)	12 more (28 fewer to 163 more)	Very low
**Severe hypoglycaemia**	Non-steroidal MRAs	Alpha-glucosidase inhibitors	0.49 (0.11 to 2.17)	39 per 1000 persons	20 per 1000 persons (4 to 81 per 1000 persons)	19 fewer (35 fewer to 42 more)	Very low
**Severe hypoglycaemia**	SGLT2 inhibitors	Alpha-glucosidase inhibitors	0.69 (0.16 to 2.94)	39 per 1000 persons	27 per 1000 persons (7 to 107 per 1000 persons)	12 fewer (32 fewer to 68 more)	Very low

Outcome	Intervention	Comparator	Odds ratio	Baseline risk	Risk if treated	Risk difference	Certainty
**Severe hypoglycaemia**	Standard treatments	Alpha-glucosidase inhibitors	0.77 (0.18 to 3.23)	39 per 1000 persons	30 per 1000 persons (7 to 116 per 1000 persons)	9 fewer (32 fewer to 77 more)	Low
**Severe hypoglycaemia**	Sulfonylureas	Alpha-glucosidase inhibitors	4.00 (0.94 to 16.67)	39 per 1000 persons	140 per 1000 persons (37 to 403 per 1000 persons)	101 more (2 fewer to 364 more)	Low
**Severe hypoglycaemia**	Thiazolidinediones	Alpha-glucosidase inhibitors	1.10 (0.25 to 4.76)	39 per 1000 persons	43 per 1000 persons (10 to 162 per 1000 persons)	4 more (29 fewer to 123 more)	Very low
**Severe hypoglycaemia**	Tirzepatide	Alpha-glucosidase inhibitors	0.87 (0.15 to 5.00)	39 per 1000 persons	34 per 1000 persons (6 to 169 per 1000 persons)	5 fewer (33 fewer to 130 more)	Very low
**Severe hypoglycaemia**	Basal-Bolus insulin	Basal insulin	2.08 (0.45 to 9.09)	69 per 1000 persons	134 per 1000 persons (32 to 403 per 1000 persons)	65 more (37 fewer to 334 more)	Very low
**Severe hypoglycaemia**	Bolus insulin	Basal insulin	1.03 (0.52 to 2.04)	69 per 1000 persons	71 per 1000 persons (37 to 131 per 1000 persons)	2 more (32 fewer to 62 more)	Very low
**Severe hypoglycaemia**	DPP-4 inhibitors	Basal insulin	0.47 (0.35 to 0.62)	69 per 1000 persons	33 per 1000 persons (25 to 44 per 1000 persons)	36 fewer (44 fewer to 25 fewer)	Moderate
**Severe hypoglycaemia**	GLP-1 receptor agonists	Basal insulin	0.41 (0.32 to 0.53)	69 per 1000 persons	29 per 1000 persons (23 to 38 per 1000 persons)	40 fewer (46 fewer to 31 fewer)	Moderate
**Severe hypoglycaemia**	Meglitinides	Basal insulin	1.35 (0.39 to 4.55)	69 per 1000 persons	91 per 1000 persons (28 to 252 per 1000 persons)	22 more (41 fewer to 183 more)	Very low
**Severe hypoglycaemia**	Metformin	Basal insulin	0.73 (0.36 to 1.49)	69 per 1000 persons	51 per 1000 persons (26 to 100 per 1000 persons)	18 fewer (43 fewer to 31 more)	Very low
**Severe hypoglycaemia**	Non-steroidal MRAs	Basal insulin	0.27 (0.16 to 0.44)	69 per 1000 persons	20 per 1000 persons (12 to 31 per 1000 persons)	49 fewer (57 fewer to 38 fewer)	Moderate
**Severe hypoglycaemia**	SGLT2 inhibitors	Basal insulin	0.38 (0.28 to 0.51)	69 per 1000 persons	27 per 1000 persons (20 to 36 per 1000 persons)	42 fewer (49 fewer to 33 fewer)	Moderate
**Severe hypoglycaemia**	Standard treatments	Basal insulin	0.42 (0.32 to 0.55)	69 per 1000 persons	30 per 1000 persons (23 to 39 per 1000 persons)	39 fewer (46 fewer to 30 fewer)	Moderate
**Severe hypoglycaemia**	Sulfonylureas	Basal insulin	2.17 (1.52 to 3.12)	69 per 1000 persons	139 per 1000 persons (101 to 188 per 1000 persons)	70 more (32 more to 119 more)	Moderate
**Severe hypoglycaemia**	Thiazolidinediones	Basal insulin	0.60 (0.38 to 0.93)	69 per 1000 persons	42 per 1000 persons (27 to 65 per 1000 persons)	27 fewer (42 fewer to 4 fewer)	Moderate

Outcome	Intervention	Comparator	Odds ratio	Baseline risk	Risk if treated	Risk difference	Certainty
**Severe hypoglycaemia**	Tirzepatide	Basal insulin	0.47 (0.18 to 1.23)	69 per 1000 persons	34 per 1000 persons (13 to 84 per 1000 persons)	35 fewer (56 fewer to 15 more)	Low
**Severe hypoglycaemia**	Bolus insulin	Basal-Bolus insulin	0.50 (0.09 to 2.63)	133 per 1000 persons	71 per 1000 persons (14 to 288 per 1000 persons)	62 fewer (119 fewer to 155 more)	Very low
**Severe hypoglycaemia**	DPP-4 inhibitors	Basal-Bolus insulin	0.23 (0.05 to 1.05)	133 per 1000 persons	33 per 1000 persons (7 to 139 per 1000 persons)	100 fewer (126 fewer to 6 more)	Low
**Severe hypoglycaemia**	GLP-1 receptor agonists	Basal-Bolus insulin	0.20 (0.04 to 0.92)	133 per 1000 persons	29 per 1000 persons (6 to 123 per 1000 persons)	104 fewer (127 fewer to 10 fewer)	Low
**Severe hypoglycaemia**	Meglitinides	Basal-Bolus insulin	0.65 (0.09 to 4.55)	133 per 1000 persons	91 per 1000 persons (14 to 411 per 1000 persons)	42 fewer (119 fewer to 278 more)	Very low
**Severe hypoglycaemia**	Metformin	Basal-Bolus insulin	0.35 (0.07 to 1.85)	133 per 1000 persons	51 per 1000 persons (10 to 221 per 1000 persons)	82 fewer (123 fewer to 88 more)	Very low
**Severe hypoglycaemia**	Non-steroidal MRAs	Basal-Bolus insulin	0.13 (0.03 to 0.64)	133 per 1000 persons	20 per 1000 persons (4 to 89 per 1000 persons)	113 fewer (129 fewer to 44 fewer)	Moderate
**Severe hypoglycaemia**	SGLT2 inhibitors	Basal-Bolus insulin	0.18 (0.04 to 0.85)	133 per 1000 persons	27 per 1000 persons (6 to 115 per 1000 persons)	106 fewer (127 fewer to 18 fewer)	Moderate
**Severe hypoglycaemia**	Standard treatments	Basal-Bolus insulin	0.20 (0.04 to 0.94)	133 per 1000 persons	30 per 1000 persons (7 to 126 per 1000 persons)	103 fewer (126 fewer to 7 fewer)	Moderate
**Severe hypoglycaemia**	Sulfonylureas	Basal-Bolus insulin	1.05 (0.22 to 5.00)	133 per 1000 persons	139 per 1000 persons (33 to 434 per 1000 persons)	6 more (100 fewer to 301 more)	Very low
**Severe hypoglycaemia**	Thiazolidinediones	Basal-Bolus insulin	0.29 (0.06 to 1.39)	133 per 1000 persons	42 per 1000 persons (9 to 176 per 1000 persons)	91 fewer (124 fewer to 43 more)	Very low
**Severe hypoglycaemia**	Tirzepatide	Basal-Bolus insulin	0.23 (0.04 to 1.39)	133 per 1000 persons	34 per 1000 persons (6 to 176 per 1000 persons)	99 fewer (127 fewer to 43 more)	Very low
**Severe hypoglycaemia**	DPP-4 inhibitors	Bolus insulin	0.45 (0.24 to 0.85)	71 per 1000 persons	33 per 1000 persons (18 to 61 per 1000 persons)	38 fewer (53 fewer to 10 fewer)	Low
**Severe hypoglycaemia**	GLP-1 receptor agonists	Bolus insulin	0.40 (0.21 to 0.74)	71 per 1000 persons	29 per 1000 persons (16 to 54 per 1000 persons)	42 fewer (55 fewer to 17 fewer)	Low
**Severe hypoglycaemia**	Meglitinides	Bolus insulin	1.30 (0.33 to 5.00)	71 per 1000 persons	90 per 1000 persons (25 to 276 per 1000 persons)	19 more (46 fewer to 205 more)	Very low

Outcome	Intervention	Comparator	Odds ratio	Baseline risk	Risk if treated	Risk difference	Certainty
**Severe hypoglycaemia**	Metformin	Bolus insulin	0.70 (0.28 to 1.75)	71 per 1000 persons	51 per 1000 persons (21 to 118 per 1000 persons)	20 fewer (50 fewer to 47 more)	Very low
**Severe hypoglycaemia**	Non-steroidal MRAs	Bolus insulin	0.26 (0.12 to 0.55)	71 per 1000 persons	20 per 1000 persons (9 to 41 per 1000 persons)	51 fewer (62 fewer to 30 fewer)	Low
**Severe hypoglycaemia**	SGLT2 inhibitors	Bolus insulin	0.36 (0.19 to 0.69)	71 per 1000 persons	27 per 1000 persons (14 to 50 per 1000 persons)	44 fewer (57 fewer to 21 fewer)	Moderate
**Severe hypoglycaemia**	Standard treatments	Bolus insulin	0.41 (0.22 to 0.76)	71 per 1000 persons	30 per 1000 persons (16 to 55 per 1000 persons)	41 fewer (55 fewer to 16 fewer)	Moderate
**Severe hypoglycaemia**	Sulfonylureas	Bolus insulin	2.13 (1.06 to 4.17)	71 per 1000 persons	140 per 1000 persons (75 to 242 per 1000 persons)	69 more (4 more to 171 more)	Moderate
**Severe hypoglycaemia**	Thiazolidinediones	Bolus insulin	0.58 (0.28 to 1.22)	71 per 1000 persons	42 per 1000 persons (21 to 85 per 1000 persons)	29 fewer (50 fewer to 14 more)	Very low
**Severe hypoglycaemia**	Tirzepatide	Bolus insulin	0.46 (0.14 to 1.47)	71 per 1000 persons	34 per 1000 persons (11 to 101 per 1000 persons)	37 fewer (60 fewer to 30 more)	Very low
**Severe hypoglycaemia**	GLP-1 receptor agonists	DPP-4 inhibitors	0.88 (0.77 to 1.00)	33 per 1000 persons	29 per 1000 persons (26 to 33 per 1000 persons)	4 fewer (7 fewer to 0)	Moderate
**Severe hypoglycaemia**	Meglitinides	DPP-4 inhibitors	2.86 (0.86 to 10.00)	33 per 1000 persons	89 per 1000 persons (29 to 254 per 1000 persons)	56 more (4 fewer to 221 more)	Low
**Severe hypoglycaemia**	Metformin	DPP-4 inhibitors	1.56 (0.80 to 3.03)	33 per 1000 persons	51 per 1000 persons (27 to 94 per 1000 persons)	18 more (6 fewer to 61 more)	Very low
**Severe hypoglycaemia**	Non-steroidal MRAs	DPP-4 inhibitors	0.57 (0.38 to 0.88)	33 per 1000 persons	19 per 1000 persons (13 to 29 per 1000 persons)	14 fewer (20 fewer to 4 fewer)	Moderate
**Severe hypoglycaemia**	SGLT2 inhibitors	DPP-4 inhibitors	0.81 (0.68 to 0.95)	33 per 1000 persons	27 per 1000 persons (23 to 31 per 1000 persons)	6 fewer (10 fewer to 2 fewer)	Moderate
**Severe hypoglycaemia**	Standard treatments	DPP-4 inhibitors	0.90 (0.81 to 1.00)	33 per 1000 persons	30 per 1000 persons (27 to 33 per 1000 persons)	3 fewer (6 fewer to 0)	Moderate
**Severe hypoglycaemia**	Sulfonylureas	DPP-4 inhibitors	4.76 (3.45 to 6.25)	33 per 1000 persons	140 per 1000 persons (105 to 176 per 1000 persons)	107 more (72 more to 143 more)	High
**Severe hypoglycaemia**	Thiazolidinediones	DPP-4 inhibitors	1.28 (0.86 to 1.92)	33 per 1000 persons	42 per 1000 persons (29 to 62 per 1000 persons)	9 more (4 fewer to 29 more)	Low

Outcome	Intervention	Comparator	Odds ratio	Baseline risk	Risk if treated	Risk difference	Certainty
**Severe hypoglycaemia**	Tirzepatide	DPP-4 inhibitors	1.01 (0.38 to 2.70)	33 per 1000 persons	33 per 1000 persons (13 to 84 per 1000 persons)	0 (20 fewer to 51 more)	Very low
**Severe hypoglycaemia**	Meglitinides	GLP-1 receptor agonists	3.33 (0.98 to 11.11)	29 per 1000 persons	91 per 1000 persons (28 to 249 per 1000 persons)	62 more (1 fewer to 220 more)	Low
**Severe hypoglycaemia**	Metformin	GLP-1 receptor agonists	1.79 (0.91 to 3.45)	29 per 1000 persons	51 per 1000 persons (26 to 93 per 1000 persons)	22 more (3 fewer to 64 more)	Low
**Severe hypoglycaemia**	Non-steroidal MRAs	GLP-1 receptor agonists	0.66 (0.43 to 1.00)	29 per 1000 persons	19 per 1000 persons (13 to 29 per 1000 persons)	10 fewer (16 fewer to 0)	Moderate
**Severe hypoglycaemia**	SGLT2 inhibitors	GLP-1 receptor agonists	0.92 (0.79 to 1.08)	29 per 1000 persons	27 per 1000 persons (23 to 31 per 1000 persons)	2 fewer (6 fewer to 2 more)	Moderate
**Severe hypoglycaemia**	Standard treatments	GLP-1 receptor agonists	1.02 (0.94 to 1.11)	29 per 1000 persons	30 per 1000 persons (27 to 32 per 1000 persons)	1 more (2 fewer to 3 more)	High
**Severe hypoglycaemia**	Sulfonylureas	GLP-1 receptor agonists	5.26 (4.00 to 7.14)	29 per 1000 persons	136 per 1000 persons (107 to 176 per 1000 persons)	107 more (78 more to 147 more)	Very low
**Severe hypoglycaemia**	Thiazolidinediones	GLP-1 receptor agonists	1.45 (0.98 to 2.17)	29 per 1000 persons	41 per 1000 persons (28 to 61 per 1000 persons)	12 more (1 fewer to 32 more)	Low
**Severe hypoglycaemia**	Tirzepatide	GLP-1 receptor agonists	1.15 (0.43 to 3.12)	29 per 1000 persons	33 per 1000 persons (13 to 85 per 1000 persons)	4 more (16 fewer to 56 more)	Very low
**Severe hypoglycaemia**	Metformin	Meglitinides	0.54 (0.18 to 1.64)	90 per 1000 persons	51 per 1000 persons (17 to 140 per 1000 persons)	39 fewer (73 fewer to 50 more)	Low
**Severe hypoglycaemia**	Non-steroidal MRAs	Meglitinides	0.20 (0.06 to 0.71)	90 per 1000 persons	19 per 1000 persons (5 to 66 per 1000 persons)	71 fewer (85 fewer to 24 fewer)	Moderate
**Severe hypoglycaemia**	SGLT2 inhibitors	Meglitinides	0.28 (0.08 to 0.94)	90 per 1000 persons	27 per 1000 persons (8 to 85 per 1000 persons)	63 fewer (82 fewer to 5 fewer)	Moderate
**Severe hypoglycaemia**	Standard treatments	Meglitinides	0.31 (0.09 to 1.04)	90 per 1000 persons	30 per 1000 persons (9 to 93 per 1000 persons)	60 fewer (81 fewer to 3 more)	Low
**Severe hypoglycaemia**	Sulfonylureas	Meglitinides	1.61 (0.48 to 5.56)	90 per 1000 persons	138 per 1000 persons (45 to 355 per 1000 persons)	48 more (45 fewer to 265 more)	Very low
**Severe hypoglycaemia**	Thiazolidinediones	Meglitinides	0.44 (0.13 to 1.56)	90 per 1000 persons	42 per 1000 persons (12 to 134 per 1000 persons)	48 fewer (78 fewer to 44 more)	Very low

Outcome	Intervention	Comparator	Odds ratio	Baseline risk	Risk if treated	Risk difference	Certainty
**Severe hypoglycaemia**	Tirzepatide	Meglitinides	0.35 (0.07 to 1.67)	90 per 1000 persons	34 per 1000 persons (7 to 142 per 1000 persons)	56 fewer (83 fewer to 52 more)	Very low
**Severe hypoglycaemia**	Non-steroidal MRAs	Metformin	0.37 (0.17 to 0.81)	51 per 1000 persons	19 per 1000 persons (9 to 42 per 1000 persons)	32 fewer (42 fewer to 9 fewer)	Low
**Severe hypoglycaemia**	SGLT2 inhibitors	Metformin	0.52 (0.26 to 1.01)	51 per 1000 persons	27 per 1000 persons (14 to 51 per 1000 persons)	24 fewer (37 fewer to 0)	Moderate
**Severe hypoglycaemia**	Standard treatments	Metformin	0.58 (0.30 to 1.12)	51 per 1000 persons	30 per 1000 persons (16 to 57 per 1000 persons)	21 fewer (35 fewer to 6 more)	Low
**Severe hypoglycaemia**	Sulfonylureas	Metformin	3.03 (1.49 to 5.88)	51 per 1000 persons	140 per 1000 persons (74 to 240 per 1000 persons)	89 more (23 more to 189 more)	Moderate
**Severe hypoglycaemia**	Thiazolidinediones	Metformin	0.82 (0.38 to 1.75)	51 per 1000 persons	42 per 1000 persons (20 to 86 per 1000 persons)	9 fewer (31 fewer to 35 more)	Very low
**Severe hypoglycaemia**	Tirzepatide	Metformin	0.65 (0.20 to 2.13)	51 per 1000 persons	34 per 1000 persons (11 to 103 per 1000 persons)	17 fewer (40 fewer to 52 more)	Very low
**Severe hypoglycaemia**	SGLT2 inhibitors	Non-steroidal MRAs	1.41 (0.91 to 2.17)	19 per 1000 persons	27 per 1000 persons (17 to 40 per 1000 persons)	8 more (2 fewer to 21 more)	Low
**Severe hypoglycaemia**	Standard treatments	Non-steroidal MRAs	1.56 (1.04 to 2.33)	19 per 1000 persons	29 per 1000 persons (20 to 43 per 1000 persons)	10 more (1 more to 24 more)	Moderate
**Severe hypoglycaemia**	Sulfonylureas	Non-steroidal MRAs	8.33 (5.00 to 14.29)	19 per 1000 persons	139 per 1000 persons (88 to 217 per 1000 persons)	120 more (69 more to 198 more)	Moderate
**Severe hypoglycaemia**	Thiazolidinediones	Non-steroidal MRAs	2.22 (1.27 to 3.85)	19 per 1000 persons	41 per 1000 persons (24 to 69 per 1000 persons)	22 more (5 more to 50 more)	Moderate
**Severe hypoglycaemia**	Tirzepatide	Non-steroidal MRAs	1.75 (0.60 to 5.00)	19 per 1000 persons	33 per 1000 persons (12 to 88 per 1000 persons)	14 more (7 fewer to 69 more)	Low
**Severe hypoglycaemia**	Standard treatments	SGLT2 inhibitors	1.11 (0.98 to 1.27)	27 per 1000 persons	30 per 1000 persons (26 to 34 per 1000 persons)	3 more (1 fewer to 7 more)	High
**Severe hypoglycaemia**	Sulfonylureas	SGLT2 inhibitors	5.88 (4.17 to 7.69)	27 per 1000 persons	140 per 1000 persons (104 to 176 per 1000 persons)	113 more (77 more to 149 more)	Moderate
**Severe hypoglycaemia**	Thiazolidinediones	SGLT2 inhibitors	1.59 (1.05 to 2.38)	27 per 1000 persons	42 per 1000 persons (28 to 62 per 1000 persons)	15 more (1 more to 35 more)	Moderate

Outcome	Intervention	Comparator	Odds ratio	Baseline risk	Risk if treated	Risk difference	Certainty
**Severe hypoglycaemia**	Tirzepatide	SGLT2 inhibitors	1.25 (0.46 to 3.33)	27 per 1000 persons	34 per 1000 persons (13 to 85 per 1000 persons)	7 more (14 fewer to 58 more)	Very low
**Severe hypoglycaemia**	Standard treatments	Sulfonylureas	0.19 (0.14 to 0.26)	139 per 1000 persons	30 per 1000 persons (23 to 40 per 1000 persons)	109 fewer (116 fewer to 99 fewer)	Moderate
**Severe hypoglycaemia**	Thiazolidinediones	Sulfonylureas	0.27 (0.17 to 0.43)	139 per 1000 persons	42 per 1000 persons (27 to 65 per 1000 persons)	97 fewer (112 fewer to 74 fewer)	Moderate
**Severe hypoglycaemia**	Tirzepatide	Sulfonylureas	0.22 (0.08 to 0.60)	139 per 1000 persons	34 per 1000 persons (12 to 88 per 1000 persons)	105 fewer (127 fewer to 51 fewer)	Moderate
**Severe hypoglycaemia**	Standard treatments	Thiazolidinediones	0.70 (0.48 to 1.03)	42 per 1000 persons	30 per 1000 persons (20 to 43 per 1000 persons)	12 fewer (22 fewer to 1 more)	Low
**Severe hypoglycaemia**	Tirzepatide	Thiazolidinediones	0.79 (0.28 to 2.27)	42 per 1000 persons	33 per 1000 persons (12 to 91 per 1000 persons)	9 fewer (30 fewer to 49 more)	Very low
**Severe hypoglycaemia**	Standard treatments	Tirzepatide	0.88 (0.33 to 2.38)	34 per 1000 persons	30 per 1000 persons (12 to 77 per 1000 persons)	4 fewer (22 fewer to 43 more)	Very low
**Severe gastrointestinal events**	Alpha-glucosidase inhibitors	Basal insulin	8.74 (0.58 to 131.74)	18 per 1000 persons	138 per 1000 persons (11 to 707 per 1000 persons)	120 more (7 fewer to 689 more)	Low
**Severe gastrointestinal events**	Alpha-glucosidase inhibitors	Bolus insulin	1.03 (0.05 to 20.56)	135 per 1000 persons	138 per 1000 persons (8 to 762 per 1000 persons)	3 more (127 fewer to 627 more)	Very low
**Severe gastrointestinal events**	Alpha-glucosidase inhibitors	DPP-4 inhibitors	2.96 (0.24 to 36.24)	51 per 1000 persons	137 per 1000 persons (13 to 661 per 1000 persons)	86 more (38 fewer to 610 more)	Very low
**Severe gastrointestinal events**	Alpha-glucosidase inhibitors	GLP-1 receptor agonists	1.72 (0.15 to 19.74)	85 per 1000 persons	138 per 1000 persons (14 to 647 per 1000 persons)	53 more (71 fewer to 562 more)	Very low
**Severe gastrointestinal events**	Alpha-glucosidase inhibitors	Metformin	1.53 (0.19 to 12.17)	95 per 1000 persons	138 per 1000 persons (20 to 561 per 1000 persons)	43 more (75 fewer to 466 more)	Very low
**Severe gastrointestinal events**	Alpha-glucosidase inhibitors	Standard treatments	3.40 (0.30 to 38.15)	45 per 1000 persons	138 per 1000 persons (14 to 643 per 1000 persons)	93 more (31 fewer to 598 more)	Very low
**Severe gastrointestinal events**	Alpha-glucosidase inhibitors	Sulfonylureas	3.43 (0.28 to 42.63)	45 per 1000 persons	139 per 1000 persons (13 to 668 per 1000 persons)	94 more (32 fewer to 623 more)	Very low
**Severe gastrointestinal events**	Alpha-glucosidase inhibitors	Thiazolidinediones	2.38 (0.22 to 25.43)	63 per 1000 persons	138 per 1000 persons (15 to 631 per 1000 persons)	75 more (48 fewer to 568 more)	Very low

Outcome	Intervention	Comparator	Odds ratio	Baseline risk	Risk if treated	Risk difference	Certainty
**Severe gastrointestinal events**	Alpha-glucosidase inhibitors	Tirzepatide	0.74 (0.06 to 9.69)	178 per 1000 persons	138 per 1000 persons (13 to 677 per 1000 persons)	40 fewer (165 fewer to 499 more)	Very low
**Severe gastrointestinal events**	Basal insulin	Bolus insulin	0.12 (0.01 to 0.98)	135 per 1000 persons	18 per 1000 persons (2 to 133 per 1000 persons)	117 fewer (133 fewer to 2 fewer)	Low
**Severe gastrointestinal events**	Basal insulin	DPP-4 inhibitors	0.34 (0.09 to 1.34)	51 per 1000 persons	18 per 1000 persons (5 to 67 per 1000 persons)	33 fewer (46 fewer to 16 more)	Very low
**Severe gastrointestinal events**	Basal insulin	GLP-1 receptor agonists	0.20 (0.06 to 0.67)	85 per 1000 persons	18 per 1000 persons (6 to 59 per 1000 persons)	67 fewer (79 fewer to 26 fewer)	Moderate
**Severe gastrointestinal events**	Basal insulin	Metformin	0.18 (0.03 to 1.01)	95 per 1000 persons	19 per 1000 persons (3 to 96 per 1000 persons)	76 fewer (92 fewer to 1 more)	Low
**Severe gastrointestinal events**	Basal insulin	Standard treatments	0.39 (0.11 to 1.36)	45 per 1000 persons	18 per 1000 persons (5 to 60 per 1000 persons)	27 fewer (40 fewer to 15 more)	Very low
**Severe gastrointestinal events**	Basal insulin	Sulfonylureas	0.39 (0.07 to 2.30)	45 per 1000 persons	18 per 1000 persons (3 to 98 per 1000 persons)	27 fewer (42 fewer to 53 more)	Very low
**Severe gastrointestinal events**	Basal insulin	Thiazolidinediones	0.27 (0.06 to 1.16)	63 per 1000 persons	18 per 1000 persons (4 to 72 per 1000 persons)	45 fewer (59 fewer to 9 more)	Low
**Severe gastrointestinal events**	Basal insulin	Tirzepatide	0.08 (0.02 to 0.30)	178 per 1000 persons	17 per 1000 persons (4 to 61 per 1000 persons)	161 fewer (174 fewer to 117 fewer)	High
**Severe gastrointestinal events**	Bolus insulin	DPP-4 inhibitors	2.88 (0.43 to 19.34)	51 per 1000 persons	134 per 1000 persons (23 to 510 per 1000 persons)	83 more (28 fewer to 459 more)	Very low
**Severe gastrointestinal events**	Bolus insulin	GLP-1 receptor agonists	1.67 (0.29 to 9.47)	85 per 1000 persons	134 per 1000 persons (26 to 468 per 1000 persons)	49 more (59 fewer to 383 more)	Very low
**Severe gastrointestinal events**	Bolus insulin	Metformin	1.48 (0.17 to 12.84)	95 per 1000 persons	134 per 1000 persons (18 to 574 per 1000 persons)	39 more (77 fewer to 479 more)	Very low
**Severe gastrointestinal events**	Bolus insulin	Standard treatments	3.30 (0.56 to 19.35)	45 per 1000 persons	135 per 1000 persons (26 to 477 per 1000 persons)	90 more (19 fewer to 432 more)	Very low
**Severe gastrointestinal events**	Bolus insulin	Sulfonylureas	3.33 (0.37 to 29.63)	45 per 1000 persons	136 per 1000 persons (17 to 583 per 1000 persons)	91 more (28 fewer to 538 more)	Very low
**Severe gastrointestinal events**	Bolus insulin	Thiazolidinediones	2.31 (0.34 to 15.78)	63 per 1000 persons	134 per 1000 persons (22 to 515 per 1000 persons)	71 more (41 fewer to 452 more)	Very low

Outcome	Intervention	Comparator	Odds ratio	Baseline risk	Risk if treated	Risk difference	Certainty
**Severe gastrointestinal events**	Bolus insulin	Tirzepatide	0.72 (0.10 to 4.97)	178 per 1000 persons	135 per 1000 persons (21 to 518 per 1000 persons)	43 fewer (157 fewer to 340 more)	Very low
**Severe gastrointestinal events**	DPP-4 inhibitors	GLP-1 receptor agonists	0.58 (0.26 to 1.28)	85 per 1000 persons	51 per 1000 persons (24 to 106 per 1000 persons)	34 fewer (61 fewer to 21 more)	Very low
**Severe gastrointestinal events**	DPP-4 inhibitors	Metformin	0.52 (0.13 to 2.10)	95 per 1000 persons	52 per 1000 persons (13 to 181 per 1000 persons)	43 fewer (82 fewer to 86 more)	Very low
**Severe gastrointestinal events**	DPP-4 inhibitors	Standard treatments	1.15 (0.52 to 2.55)	45 per 1000 persons	51 per 1000 persons (24 to 107 per 1000 persons)	6 more (21 fewer to 62 more)	Very low
**Severe gastrointestinal events**	DPP-4 inhibitors	Sulfonylureas	1.16 (0.31 to 4.32)	45 per 1000 persons	52 per 1000 persons (14 to 169 per 1000 persons)	7 more (31 fewer to 124 more)	Very low
**Severe gastrointestinal events**	DPP-4 inhibitors	Thiazolidinediones	0.80 (0.28 to 2.29)	63 per 1000 persons	51 per 1000 persons (18 to 133 per 1000 persons)	12 fewer (45 fewer to 70 more)	Very low
**Severe gastrointestinal events**	DPP-4 inhibitors	Tirzepatide	0.25 (0.08 to 0.78)	178 per 1000 persons	51 per 1000 persons (17 to 144 per 1000 persons)	127 fewer (161 fewer to 34 fewer)	Moderate
**Severe gastrointestinal events**	GLP-1 receptor agonists	Metformin	0.89 (0.25 to 3.21)	95 per 1000 persons	85 per 1000 persons (26 to 252 per 1000 persons)	10 fewer (69 fewer to 157 more)	Very low
**Severe gastrointestinal events**	GLP-1 receptor agonists	Standard treatments	1.97 (1.39 to 2.80)	45 per 1000 persons	85 per 1000 persons (61 to 117 per 1000 persons)	40 more (16 more to 72 more)	Moderate
**Severe gastrointestinal events**	GLP-1 receptor agonists	Sulfonylureas	1.99 (0.53 to 7.54)	45 per 1000 persons	86 per 1000 persons (24 to 262 per 1000 persons)	41 more (21 fewer to 217 more)	Very low
**Severe gastrointestinal events**	GLP-1 receptor agonists	Thiazolidinediones	1.38 (0.61 to 3.15)	63 per 1000 persons	85 per 1000 persons (39 to 175 per 1000 persons)	22 more (24 fewer to 112 more)	Very low
**Severe gastrointestinal events**	GLP-1 receptor agonists	Tirzepatide	0.43 (0.18 to 1.01)	178 per 1000 persons	85 per 1000 persons (38 to 179 per 1000 persons)	93 fewer (140 fewer to 1 more)	Moderate
**Severe gastrointestinal events**	Metformin	Standard treatments	2.22 (0.64 to 7.71)	45 per 1000 persons	95 per 1000 persons (29 to 266 per 1000 persons)	50 more (16 fewer to 221 more)	Very low
**Severe gastrointestinal events**	Metformin	Sulfonylureas	2.24 (0.53 to 9.39)	45 per 1000 persons	95 per 1000 persons (24 to 307 per 1000 persons)	50 more (21 fewer to 262 more)	Low
**Severe gastrointestinal events**	Metformin	Thiazolidinediones	1.56 (0.50 to 4.88)	63 per 1000 persons	95 per 1000 persons (33 to 247 per 1000 persons)	32 more (30 fewer to 184 more)	Very low

Outcome	Intervention	Comparator	Odds ratio	Baseline risk	Risk if treated	Risk difference	Certainty
**Severe gastrointestinal events**	Metformin	Tirzepatide	0.48 (0.11 to 2.21)	178 per 1000 persons	94 per 1000 persons (23 to 324 per 1000 persons)	84 fewer (155 fewer to 146 more)	Very low
**Severe gastrointestinal events**	Sulfonylureas	Standard treatments	0.99 (0.27 to 3.68)	45 per 1000 persons	45 per 1000 persons (13 to 148 per 1000 persons)	0 (32 fewer to 103 more)	Very low
**Severe gastrointestinal events**	Sulfonylureas	Thiazolidinediones	0.70 (0.19 to 2.53)	63 per 1000 persons	45 per 1000 persons (13 to 145 per 1000 persons)	18 fewer (50 fewer to 82 more)	Low
**Severe gastrointestinal events**	Sulfonylureas	Tirzepatide	0.22 (0.05 to 1.03)	178 per 1000 persons	45 per 1000 persons (11 to 182 per 1000 persons)	133 fewer (167 fewer to 4 more)	Low
**Severe gastrointestinal events**	Thiazolidinediones	Standard treatments	1.43 (0.67 to 3.03)	45 per 1000 persons	63 per 1000 persons (31 to 125 per 1000 persons)	18 more (14 fewer to 80 more)	Low
**Severe gastrointestinal events**	Thiazolidinediones	Tirzepatide	0.31 (0.10 to 0.99)	178 per 1000 persons	63 per 1000 persons (21 to 177 per 1000 persons)	115 fewer (157 fewer to 1 fewer)	Moderate
**Severe gastrointestinal events**	Tirzepatide	Standard treatments	4.59 (1.89 to 11.14)	45 per 1000 persons	178 per 1000 persons (82 to 344 per 1000 persons)	133 more (37 more to 299 more)	Moderate
**Severe gastrointestinal events**	Basal insulin	Alpha-glucosidase inhibitors	0.11 (0.01 to 1.72)	138 per 1000 persons	18 per 1000 persons (1 to 216 per 1000 persons)	120 fewer (137 fewer to 78 more)	Very low
**Severe gastrointestinal events**	Bolus insulin	Alpha-glucosidase inhibitors	0.97 (0.05 to 20.00)	138 per 1000 persons	135 per 1000 persons (8 to 762 per 1000 persons)	3 fewer (130 fewer to 624 more)	Very low
**Severe gastrointestinal events**	DPP-4 inhibitors	Alpha-glucosidase inhibitors	0.34 (0.03 to 4.17)	138 per 1000 persons	51 per 1000 persons (4 to 400 per 1000 persons)	87 fewer (134 fewer to 262 more)	Very low
**Severe gastrointestinal events**	GLP-1 receptor agonists	Alpha-glucosidase inhibitors	0.58 (0.05 to 6.67)	138 per 1000 persons	85 per 1000 persons (8 to 516 per 1000 persons)	53 fewer (130 fewer to 378 more)	Very low
**Severe gastrointestinal events**	Metformin	Alpha-glucosidase inhibitors	0.65 (0.08 to 5.26)	138 per 1000 persons	95 per 1000 persons (13 to 457 per 1000 persons)	43 fewer (125 fewer to 319 more)	Very low
**Severe gastrointestinal events**	Standard treatments	Alpha-glucosidase inhibitors	0.29 (0.03 to 3.33)	138 per 1000 persons	45 per 1000 persons (4 to 348 per 1000 persons)	93 fewer (134 fewer to 210 more)	Very low
**Severe gastrointestinal events**	Sulfonylureas	Alpha-glucosidase inhibitors	0.29 (0.02 to 3.57)	138 per 1000 persons	45 per 1000 persons (4 to 364 per 1000 persons)	93 fewer (134 fewer to 226 more)	Very low
**Severe gastrointestinal events**	Thiazolidinediones	Alpha-glucosidase inhibitors	0.42 (0.04 to 4.55)	138 per 1000 persons	63 per 1000 persons (6 to 421 per 1000 persons)	75 fewer (132 fewer to 283 more)	Very low

Outcome	Intervention	Comparator	Odds ratio	Baseline risk	Risk if treated	Risk difference	Certainty
**Severe gastrointestinal events**	Tirzepatide	Alpha-glucosidase inhibitors	1.35 (0.10 to 16.67)	138 per 1000 persons	178 per 1000 persons (16 to 727 per 1000 persons)	40 more (122 fewer to 589 more)	Very low
**Severe gastrointestinal events**	Bolus insulin	Basal insulin	8.33 (1.02 to 100.00)	18 per 1000 persons	133 per 1000 persons (18 to 647 per 1000 persons)	115 more (0 to 629 more)	Low
**Severe gastrointestinal events**	DPP-4 inhibitors	Basal insulin	2.94 (0.75 to 11.11)	18 per 1000 persons	51 per 1000 persons (13 to 169 per 1000 persons)	33 more (5 fewer to 151 more)	Very low
**Severe gastrointestinal events**	GLP-1 receptor agonists	Basal insulin	5.00 (1.49 to 16.67)	18 per 1000 persons	84 per 1000 persons (27 to 234 per 1000 persons)	66 more (9 more to 216 more)	Moderate
**Severe gastrointestinal events**	Metformin	Basal insulin	5.56 (0.99 to 33.33)	18 per 1000 persons	92 per 1000 persons (18 to 379 per 1000 persons)	74 more (0 to 361 more)	Moderate
**Severe gastrointestinal events**	Standard treatments	Basal insulin	2.56 (0.74 to 9.09)	18 per 1000 persons	45 per 1000 persons (13 to 143 per 1000 persons)	27 more (5 fewer to 125 more)	Low
**Severe gastrointestinal events**	Sulfonylureas	Basal insulin	2.56 (0.43 to 14.29)	18 per 1000 persons	45 per 1000 persons (8 to 208 per 1000 persons)	27 more (10 fewer to 190 more)	Low
**Severe gastrointestinal events**	Thiazolidinediones	Basal insulin	3.70 (0.86 to 16.67)	18 per 1000 persons	64 per 1000 persons (16 to 234 per 1000 persons)	46 more (2 fewer to 216 more)	Low
**Severe gastrointestinal events**	Tirzepatide	Basal insulin	12.50 (3.33 to 50.00)	18 per 1000 persons	186 per 1000 persons (58 to 478 per 1000 persons)	168 more (40 more to 460 more)	High
**Severe gastrointestinal events**	DPP-4 inhibitors	Bolus insulin	0.35 (0.05 to 2.33)	135 per 1000 persons	51 per 1000 persons (8 to 266 per 1000 persons)	84 fewer (127 fewer to 131 more)	Very low
**Severe gastrointestinal events**	GLP-1 receptor agonists	Bolus insulin	0.60 (0.11 to 3.45)	135 per 1000 persons	85 per 1000 persons (16 to 350 per 1000 persons)	50 fewer (119 fewer to 215 more)	Very low
**Severe gastrointestinal events**	Metformin	Bolus insulin	0.68 (0.08 to 5.88)	135 per 1000 persons	95 per 1000 persons (12 to 479 per 1000 persons)	40 fewer (123 fewer to 344 more)	Very low
**Severe gastrointestinal events**	Standard treatments	Bolus insulin	0.30 (0.05 to 1.79)	135 per 1000 persons	45 per 1000 persons (8 to 218 per 1000 persons)	90 fewer (127 fewer to 83 more)	Very low
**Severe gastrointestinal events**	Sulfonylureas	Bolus insulin	0.30 (0.03 to 2.70)	135 per 1000 persons	45 per 1000 persons (5 to 297 per 1000 persons)	90 fewer (130 fewer to 162 more)	Very low
**Severe gastrointestinal events**	Thiazolidinediones	Bolus insulin	0.43 (0.06 to 2.94)	135 per 1000 persons	63 per 1000 persons (10 to 315 per 1000 persons)	72 fewer (125 fewer to 180 more)	Very low

Outcome	Intervention	Comparator	Odds ratio	Baseline risk	Risk if treated	Risk difference	Certainty
**Severe gastrointestinal events**	Tirzepatide	Bolus insulin	1.39 (0.20 to 10.00)	135 per 1000 persons	178 per 1000 persons (30 to 609 per 1000 persons)	43 more (105 fewer to 474 more)	Very low
**Severe gastrointestinal events**	GLP-1 receptor agonists	DPP-4 inhibitors	1.72 (0.78 to 3.85)	51 per 1000 persons	85 per 1000 persons (40 to 171 per 1000 persons)	34 more (11 fewer to 120 more)	Very low
**Severe gastrointestinal events**	Metformin	DPP-4 inhibitors	1.92 (0.48 to 7.69)	51 per 1000 persons	94 per 1000 persons (25 to 292 per 1000 persons)	43 more (26 fewer to 241 more)	Very low
**Severe gastrointestinal events**	Standard treatments	DPP-4 inhibitors	0.87 (0.39 to 1.92)	51 per 1000 persons	45 per 1000 persons (21 to 94 per 1000 persons)	6 fewer (30 fewer to 43 more)	Very low
**Severe gastrointestinal events**	Sulfonylureas	DPP-4 inhibitors	0.86 (0.23 to 3.23)	51 per 1000 persons	44 per 1000 persons (12 to 148 per 1000 persons)	7 fewer (39 fewer to 97 more)	Very low
**Severe gastrointestinal events**	Thiazolidinediones	DPP-4 inhibitors	1.25 (0.44 to 3.57)	51 per 1000 persons	63 per 1000 persons (23 to 161 per 1000 persons)	12 more (28 fewer to 110 more)	Very low
**Severe gastrointestinal events**	Tirzepatide	DPP-4 inhibitors	4.00 (1.28 to 12.50)	51 per 1000 persons	177 per 1000 persons (64 to 402 per 1000 persons)	126 more (13 more to 351 more)	Moderate
**Severe gastrointestinal events**	Metformin	GLP-1 receptor agonists	1.12 (0.31 to 4.00)	85 per 1000 persons	95 per 1000 persons (28 to 271 per 1000 persons)	10 more (57 fewer to 186 more)	Very low
**Severe gastrointestinal events**	Standard treatments	GLP-1 receptor agonists	0.51 (0.36 to 0.72)	85 per 1000 persons	45 per 1000 persons (32 to 63 per 1000 persons)	40 fewer (53 fewer to 22 fewer)	Moderate
**Severe gastrointestinal events**	Sulfonylureas	GLP-1 receptor agonists	0.50 (0.13 to 1.89)	85 per 1000 persons	45 per 1000 persons (12 to 149 per 1000 persons)	40 fewer (73 fewer to 64 more)	Very low
**Severe gastrointestinal events**	Thiazolidinediones	GLP-1 receptor agonists	0.72 (0.32 to 1.64)	85 per 1000 persons	63 per 1000 persons (29 to 132 per 1000 persons)	22 fewer (56 fewer to 47 more)	Very low
**Severe gastrointestinal events**	Tirzepatide	GLP-1 receptor agonists	2.33 (0.99 to 5.56)	85 per 1000 persons	178 per 1000 persons (84 to 340 per 1000 persons)	93 more (1 fewer to 255 more)	Moderate
**Severe gastrointestinal events**	Standard treatments	Metformin	0.45 (0.13 to 1.56)	95 per 1000 persons	45 per 1000 persons (13 to 141 per 1000 persons)	50 fewer (82 fewer to 46 more)	Very low
**Severe gastrointestinal events**	Sulfonylureas	Metformin	0.45 (0.11 to 1.89)	95 per 1000 persons	45 per 1000 persons (11 to 165 per 1000 persons)	50 fewer (84 fewer to 70 more)	Low
**Severe gastrointestinal events**	Thiazolidinediones	Metformin	0.64 (0.20 to 2.00)	95 per 1000 persons	63 per 1000 persons (21 to 174 per 1000 persons)	32 fewer (74 fewer to 79 more)	Very low

Outcome	Intervention	Comparator	Odds ratio	Baseline risk	Risk if treated	Risk difference	Certainty
**Severe gastrointestinal events**	Tirzepatide	Metformin	2.08 (0.45 to 9.09)	95 per 1000 persons	179 per 1000 persons (45 to 488 per 1000 persons)	84 more (50 fewer to 393 more)	Very low
**Severe gastrointestinal events**	Standard treatments	Sulfonylureas	1.01 (0.27 to 3.70)	45 per 1000 persons	45 per 1000 persons (13 to 149 per 1000 persons)	0 (32 fewer to 104 more)	Very low
**Severe gastrointestinal events**	Thiazolidinediones	Sulfonylureas	1.43 (0.40 to 5.26)	45 per 1000 persons	63 per 1000 persons (18 to 199 per 1000 persons)	18 more (27 fewer to 154 more)	Low
**Severe gastrointestinal events**	Tirzepatide	Sulfonylureas	4.55 (0.97 to 20.00)	45 per 1000 persons	176 per 1000 persons (44 to 485 per 1000 persons)	131 more (1 fewer to 440 more)	Low
**Severe gastrointestinal events**	Standard treatments	Thiazolidinediones	0.70 (0.33 to 1.49)	63 per 1000 persons	45 per 1000 persons (22 to 91 per 1000 persons)	18 fewer (41 fewer to 28 more)	Low
**Severe gastrointestinal events**	Tirzepatide	Thiazolidinediones	3.23 (1.01 to 10.00)	63 per 1000 persons	178 per 1000 persons (64 to 402 per 1000 persons)	115 more (1 more to 339 more)	Moderate
**Severe gastrointestinal events**	Standard treatments	Tirzepatide	0.22 (0.09 to 0.53)	178 per 1000 persons	45 per 1000 persons (19 to 103 per 1000 persons)	133 fewer (159 fewer to 75 fewer)	Moderate
**Genital infection**	DPP-4 inhibitors	GLP-1 receptor agonists	1.03 (0.47 to 2.23)	52 per 1000 persons	53 per 1000 persons (25 to 109 per 1000 persons)	1 more (27 fewer to 57 more)	Very low
**Genital infection**	DPP-4 inhibitors	Metformin	0.55 (0.26 to 1.16)	93 per 1000 persons	53 per 1000 persons (26 to 106 per 1000 persons)	40 fewer (67 fewer to 13 more)	Very low
**Genital infection**	DPP-4 inhibitors	SGLT2 inhibitors	0.22 (0.14 to 0.33)	206 per 1000 persons	54 per 1000 persons (35 to 79 per 1000 persons)	152 fewer (171 fewer to 127 fewer)	Moderate
**Genital infection**	DPP-4 inhibitors	Standard treatments	0.71 (0.46 to 1.10)	73 per 1000 persons	53 per 1000 persons (35 to 80 per 1000 persons)	20 fewer (38 fewer to 7 more)	Low
**Genital infection**	DPP-4 inhibitors	Sulfonylureas	1.36 (0.80 to 2.33)	39 per 1000 persons	52 per 1000 persons (31 to 86 per 1000 persons)	13 more (8 fewer to 47 more)	Low
**Genital infection**	DPP-4 inhibitors	Thiazolidinediones	0.07 (0.00 to 1.72)	461 per 1000 persons	56 per 1000 persons (0 to 595 per 1000 persons)	405 fewer (461 fewer to 134 more)	Very low
**Genital infection**	GLP-1 receptor agonists	Metformin	0.53 (0.22 to 1.31)	93 per 1000 persons	52 per 1000 persons (22 to 118 per 1000 persons)	41 fewer (71 fewer to 25 more)	Very low
**Genital infection**	GLP-1 receptor agonists	SGLT2 inhibitors	0.21 (0.11 to 0.40)	206 per 1000 persons	52 per 1000 persons (28 to 94 per 1000 persons)	154 fewer (178 fewer to 112 fewer)	Moderate

Outcome	Intervention	Comparator	Odds ratio	Baseline risk	Risk if treated	Risk difference	Certainty
**Genital infection**	GLP-1 receptor agonists	Standard treatments	0.69 (0.36 to 1.34)	73 per 1000 persons	52 per 1000 persons (28 to 95 per 1000 persons)	21 fewer (45 fewer to 22 more)	Very low
**Genital infection**	GLP-1 receptor agonists	Sulfonylureas	1.33 (0.64 to 2.76)	39 per 1000 persons	51 per 1000 persons (25 to 101 per 1000 persons)	12 more (14 fewer to 62 more)	Very low
**Genital infection**	GLP-1 receptor agonists	Thiazolidinediones	0.06 (0.00 to 1.73)	461 per 1000 persons	49 per 1000 persons (0 to 597 per 1000 persons)	412 fewer (461 fewer to 136 more)	Very low
**Genital infection**	Metformin	SGLT2 inhibitors	0.39 (0.21 to 0.73)	206 per 1000 persons	92 per 1000 persons (52 to 159 per 1000 persons)	114 fewer (154 fewer to 47 fewer)	High
**Genital infection**	Metformin	Standard treatments	1.30 (0.69 to 2.45)	73 per 1000 persons	93 per 1000 persons (52 to 162 per 1000 persons)	20 more (21 fewer to 89 more)	Very low
**Genital infection**	Metformin	Sulfonylureas	2.49 (1.24 to 5.04)	39 per 1000 persons	92 per 1000 persons (48 to 170 per 1000 persons)	53 more (9 more to 131 more)	High
**Genital infection**	Metformin	Thiazolidinediones	0.12 (0.00 to 3.24)	461 per 1000 persons	93 per 1000 persons (0 to 735 per 1000 persons)	368 fewer (461 fewer to 274 more)	Low
**Genital infection**	SGLT2 inhibitors	Standard treatments	3.30 (2.88 to 3.78)	73 per 1000 persons	206 per 1000 persons (185 to 229 per 1000 persons)	133 more (112 more to 156 more)	High
**Genital infection**	SGLT2 inhibitors	Sulfonylureas	6.32 (4.53 to 8.82)	39 per 1000 persons	204 per 1000 persons (155 to 264 per 1000 persons)	165 more (116 more to 225 more)	High
**Genital infection**	SGLT2 inhibitors	Thiazolidinediones	0.30 (0.01 to 7.75)	461 per 1000 persons	204 per 1000 persons (8 to 869 per 1000 persons)	257 fewer (453 fewer to 408 more)	Low
**Genital infection**	Sulfonylureas	Standard treatments	0.52 (0.36 to 0.75)	73 per 1000 persons	39 per 1000 persons (28 to 56 per 1000 persons)	34 fewer (45 fewer to 17 fewer)	Low
**Genital infection**	Sulfonylureas	Thiazolidinediones	0.05 (0.00 to 1.25)	461 per 1000 persons	41 per 1000 persons (0 to 517 per 1000 persons)	420 fewer (461 fewer to 56 more)	Low
**Genital infection**	Thiazolidinediones	Standard treatments	10.85 (0.42 to 276.91)	73 per 1000 persons	461 per 1000 persons (32 to 956 per 1000 persons)	388 more (41 fewer to 883 more)	Very low
**Genital infection**	GLP-1 receptor agonists	DPP-4 inhibitors	0.97 (0.45 to 2.13)	53 per 1000 persons	52 per 1000 persons (24 to 106 per 1000 persons)	1 fewer (29 fewer to 53 more)	Very low
**Genital infection**	Metformin	DPP-4 inhibitors	1.82 (0.86 to 3.85)	53 per 1000 persons	92 per 1000 persons (46 to 177 per 1000 persons)	39 more (7 fewer to 124 more)	Low

Outcome	Intervention	Comparator	Odds ratio	Baseline risk	Risk if treated	Risk difference	Certainty
**Genital infection**	SGLT2 inhibitors	DPP-4 inhibitors	4.55 (3.03 to 7.14)	53 per 1000 persons	203 per 1000 persons (145 to 286 per 1000 persons)	150 more (92 more to 233 more)	Moderate
**Genital infection**	Standard treatments	DPP-4 inhibitors	1.41 (0.91 to 2.17)	53 per 1000 persons	73 per 1000 persons (48 to 108 per 1000 persons)	20 more (5 fewer to 55 more)	Low
**Genital infection**	Sulfonylureas	DPP-4 inhibitors	0.74 (0.43 to 1.25)	53 per 1000 persons	40 per 1000 persons (23 to 65 per 1000 persons)	13 fewer (30 fewer to 12 more)	Very low
**Genital infection**	Thiazolidinediones	DPP-4 inhibitors	14.29 (0.58 to Inf)	53 per 1000 persons	444 per 1000 persons (32 to NaN per 1000 persons)	391 more (21 fewer to NA)	Moderate
**Genital infection**	Metformin	GLP-1 receptor agonists	1.89 (0.76 to 4.55)	52 per 1000 persons	94 per 1000 persons (40 to 200 per 1000 persons)	42 more (12 fewer to 148 more)	Very low
**Genital infection**	SGLT2 inhibitors	GLP-1 receptor agonists	4.76 (2.50 to 9.09)	52 per 1000 persons	207 per 1000 persons (121 to 333 per 1000 persons)	155 more (69 more to 281 more)	Moderate
**Genital infection**	Standard treatments	GLP-1 receptor agonists	1.45 (0.75 to 2.78)	52 per 1000 persons	74 per 1000 persons (39 to 132 per 1000 persons)	22 more (13 fewer to 80 more)	Very low
**Genital infection**	Sulfonylureas	GLP-1 receptor agonists	0.75 (0.36 to 1.56)	52 per 1000 persons	40 per 1000 persons (19 to 79 per 1000 persons)	12 fewer (33 fewer to 27 more)	Very low
**Genital infection**	Thiazolidinediones	GLP-1 receptor agonists	16.67 (0.58 to Inf)	52 per 1000 persons	478 per 1000 persons (31 to NaN per 1000 persons)	426 more (21 fewer to NA)	Moderate
**Genital infection**	SGLT2 inhibitors	Metformin	2.56 (1.37 to 4.76)	93 per 1000 persons	208 per 1000 persons (123 to 328 per 1000 persons)	115 more (30 more to 235 more)	High
**Genital infection**	Standard treatments	Metformin	0.77 (0.41 to 1.45)	93 per 1000 persons	73 per 1000 persons (40 to 129 per 1000 persons)	20 fewer (53 fewer to 36 more)	Very low
**Genital infection**	Sulfonylureas	Metformin	0.40 (0.20 to 0.81)	93 per 1000 persons	40 per 1000 persons (20 to 76 per 1000 persons)	53 fewer (73 fewer to 17 fewer)	High
**Genital infection**	Thiazolidinediones	Metformin	8.33 (0.31 to Inf)	93 per 1000 persons	461 per 1000 persons (31 to NaN per 1000 persons)	368 more (62 fewer to NA)	High
**Genital infection**	Standard treatments	SGLT2 inhibitors	0.30 (0.26 to 0.35)	206 per 1000 persons	73 per 1000 persons (64 to 83 per 1000 persons)	133 fewer (142 fewer to 123 fewer)	High
**Genital infection**	Sulfonylureas	SGLT2 inhibitors	0.16 (0.11 to 0.22)	206 per 1000 persons	39 per 1000 persons (29 to 54 per 1000 persons)	167 fewer (177 fewer to 152 fewer)	High

Outcome	Intervention	Comparator	Odds ratio	Baseline risk	Risk if treated	Risk difference	Certainty
**Genital infection**	Thiazolidinediones	SGLT2 inhibitors	3.33 (0.13 to 100.00)	206 per 1000 persons	464 per 1000 persons (32 to 963 per 1000 persons)	258 more (174 fewer to 757 more)	Low
**Genital infection**	Standard treatments	Sulfonylureas	1.92 (1.33 to 2.78)	39 per 1000 persons	72 per 1000 persons (51 to 101 per 1000 persons)	33 more (12 more to 62 more)	Low
**Genital infection**	Thiazolidinediones	Sulfonylureas	20.00 (0.80 to Inf)	39 per 1000 persons	448 per 1000 persons (31 to NaN per 1000 persons)	409 more (8 fewer to NA)	High
**Genital infection**	Standard treatments	Thiazolidinediones	0.09 (0.00 to 2.38)	461 per 1000 persons	73 per 1000 persons (3 to 671 per 1000 persons)	388 fewer (458 fewer to 210 more)	Very low
**Amputation**	DPP-4 inhibitors	GLP-1 receptor agonists	1.28 (0.12 to 13.54)	7 per 1000 persons	9 per 1000 persons (1 to 87 per 1000 persons)	2 more (6 fewer to 80 more)	Low
**Amputation**	DPP-4 inhibitors	Non-steroidal MRAs	0.97 (0.37 to 2.55)	9 per 1000 persons	9 per 1000 persons (3 to 23 per 1000 persons)	0 (6 fewer to 14 more)	Low
**Amputation**	DPP-4 inhibitors	SGLT2 inhibitors	0.71 (0.39 to 1.31)	13 per 1000 persons	9 per 1000 persons (5 to 17 per 1000 persons)	4 fewer (8 fewer to 4 more)	Moderate
**Amputation**	DPP-4 inhibitors	Standard treatments	0.91 (0.51 to 1.60)	10 per 1000 persons	9 per 1000 persons (5 to 16 per 1000 persons)	1 fewer (5 fewer to 6 more)	High
**Amputation**	DPP-4 inhibitors	Thiazolidinediones	1.24 (0.54 to 2.83)	7 per 1000 persons	9 per 1000 persons (4 to 20 per 1000 persons)	2 more (3 fewer to 13 more)	Low
**Amputation**	GLP-1 receptor agonists	Non-steroidal MRAs	0.76 (0.07 to 8.48)	9 per 1000 persons	7 per 1000 persons (1 to 72 per 1000 persons)	2 fewer (8 fewer to 63 more)	Low
**Amputation**	GLP-1 receptor agonists	SGLT2 inhibitors	0.55 (0.06 to 5.52)	13 per 1000 persons	7 per 1000 persons (1 to 68 per 1000 persons)	6 fewer (12 fewer to 55 more)	Very low
**Amputation**	GLP-1 receptor agonists	Standard treatments	0.71 (0.07 to 6.95)	10 per 1000 persons	7 per 1000 persons (1 to 66 per 1000 persons)	3 fewer (9 fewer to 56 more)	Moderate
**Amputation**	GLP-1 receptor agonists	Thiazolidinediones	0.96 (0.09 to 10.26)	7 per 1000 persons	7 per 1000 persons (1 to 67 per 1000 persons)	0 (6 fewer to 60 more)	Low
**Amputation**	Non-steroidal MRAs	SGLT2 inhibitors	0.73 (0.32 to 1.65)	13 per 1000 persons	10 per 1000 persons (4 to 21 per 1000 persons)	3 fewer (9 fewer to 8 more)	Moderate
**Amputation**	Non-steroidal MRAs	Standard treatments	0.93 (0.43 to 2.03)	10 per 1000 persons	9 per 1000 persons (4 to 20 per 1000 persons)	1 fewer (6 fewer to 10 more)	Moderate

Outcome	Intervention	Comparator	Odds ratio	Baseline risk	Risk if treated	Risk difference	Certainty
**Amputation**	Non-steroidal MRAs	Thiazolidinediones	1.27 (0.48 to 3.40)	7 per 1000 persons	9 per 1000 persons (3 to 23 per 1000 persons)	2 more (4 fewer to 16 more)	Low
**Amputation**	SGLT2 inhibitors	Standard treatments	1.27 (1.01 to 1.61)	10 per 1000 persons	13 per 1000 persons (10 to 16 per 1000 persons)	3 more (0 to 6 more)	Moderate
**Amputation**	SGLT2 inhibitors	Thiazolidinediones	1.74 (0.91 to 3.31)	7 per 1000 persons	12 per 1000 persons (6 to 23 per 1000 persons)	5 more (1 fewer to 16 more)	Low
**Amputation**	Thiazolidinediones	Standard treatments	0.73 (0.40 to 1.33)	10 per 1000 persons	7 per 1000 persons (4 to 13 per 1000 persons)	3 fewer (6 fewer to 3 more)	Moderate
**Amputation**	GLP-1 receptor agonists	DPP-4 inhibitors	0.78 (0.07 to 8.33)	9 per 1000 persons	7 per 1000 persons (1 to 70 per 1000 persons)	2 fewer (8 fewer to 61 more)	Low
**Amputation**	Non-steroidal MRAs	DPP-4 inhibitors	1.03 (0.39 to 2.70)	9 per 1000 persons	9 per 1000 persons (4 to 24 per 1000 persons)	0 (5 fewer to 15 more)	Low
**Amputation**	SGLT2 inhibitors	DPP-4 inhibitors	1.41 (0.76 to 2.56)	9 per 1000 persons	13 per 1000 persons (7 to 23 per 1000 persons)	4 more (2 fewer to 14 more)	Low
**Amputation**	Standard treatments	DPP-4 inhibitors	1.10 (0.62 to 1.96)	9 per 1000 persons	10 per 1000 persons (6 to 17 per 1000 persons)	1 more (3 fewer to 8 more)	High
**Amputation**	Thiazolidinediones	DPP-4 inhibitors	0.81 (0.35 to 1.85)	9 per 1000 persons	7 per 1000 persons (3 to 17 per 1000 persons)	2 fewer (6 fewer to 8 more)	Moderate
**Amputation**	Non-steroidal MRAs	GLP-1 receptor agonists	1.32 (0.12 to 14.29)	7 per 1000 persons	9 per 1000 persons (1 to 91 per 1000 persons)	2 more (6 fewer to 84 more)	Low
**Amputation**	SGLT2 inhibitors	GLP-1 receptor agonists	1.82 (0.18 to 16.67)	7 per 1000 persons	13 per 1000 persons (1 to 105 per 1000 persons)	6 more (6 fewer to 98 more)	Low
**Amputation**	Standard treatments	GLP-1 receptor agonists	1.41 (0.14 to 14.29)	7 per 1000 persons	10 per 1000 persons (1 to 91 per 1000 persons)	3 more (6 fewer to 84 more)	Moderate
**Amputation**	Thiazolidinediones	GLP-1 receptor agonists	1.04 (0.10 to 11.11)	7 per 1000 persons	7 per 1000 persons (1 to 73 per 1000 persons)	0 (6 fewer to 66 more)	Low
**Amputation**	SGLT2 inhibitors	Non-steroidal MRAs	1.37 (0.61 to 3.12)	9 per 1000 persons	12 per 1000 persons (5 to 28 per 1000 persons)	3 more (4 fewer to 19 more)	Low
**Amputation**	Standard treatments	Non-steroidal MRAs	1.08 (0.49 to 2.33)	9 per 1000 persons	10 per 1000 persons (4 to 21 per 1000 persons)	1 more (5 fewer to 12 more)	Low

Outcome	Intervention	Comparator	Odds ratio	Baseline risk	Risk if treated	Risk difference	Certainty
**Amputation**	Thiazolidinediones	Non-steroidal MRAs	0.79 (0.29 to 2.08)	9 per 1000 persons	7 per 1000 persons (3 to 19 per 1000 persons)	2 fewer (6 fewer to 10 more)	Moderate
**Amputation**	Standard treatments	SGLT2 inhibitors	0.79 (0.62 to 0.99)	13 per 1000 persons	10 per 1000 persons (8 to 13 per 1000 persons)	3 fewer (5 fewer to 0)	Moderate
**Amputation**	Thiazolidinediones	SGLT2 inhibitors	0.57 (0.30 to 1.10)	13 per 1000 persons	8 per 1000 persons (4 to 14 per 1000 persons)	5 fewer (9 fewer to 1 more)	Moderate
**Amputation**	Standard treatments	Thiazolidinediones	1.37 (0.75 to 2.50)	7 per 1000 persons	10 per 1000 persons (5 to 17 per 1000 persons)	3 more (2 fewer to 10 more)	Moderate
**Ketoacidosis due to diabetes**	Alpha-glucosidase inhibitors	DPP-4 inhibitors	0.49 (0.01 to 19.75)	2 per 1000 persons	1 per 1000 persons (0 to 38 per 1000 persons)	1 fewer (2 fewer to 36 more)	Very low
**Ketoacidosis due to diabetes**	Alpha-glucosidase inhibitors	GLP-1 receptor agonists	0.38 (0.01 to 16.39)	2 per 1000 persons	1 per 1000 persons (0 to 32 per 1000 persons)	1 fewer (2 fewer to 30 more)	Low
**Ketoacidosis due to diabetes**	Alpha-glucosidase inhibitors	Meglitinides	1.01 (0.02 to 51.04)	1 per 1000 persons	1 per 1000 persons (0 to 49 per 1000 persons)	0 (1 fewer to 48 more)	Very low
**Ketoacidosis due to diabetes**	Alpha-glucosidase inhibitors	Metformin	0.29 (0.00 to 39.24)	3 per 1000 persons	1 per 1000 persons (0 to 106 per 1000 persons)	2 fewer (3 fewer to 103 more)	Low
**Ketoacidosis due to diabetes**	Alpha-glucosidase inhibitors	Non-steroidal MRAs	0.58 (0.01 to 26.32)	1 per 1000 persons	1 per 1000 persons (0 to 26 per 1000 persons)	0 (1 fewer to 25 more)	Very low
**Ketoacidosis due to diabetes**	Alpha-glucosidase inhibitors	SGLT2 inhibitors	0.19 (0.00 to 7.97)	4 per 1000 persons	1 per 1000 persons (0 to 31 per 1000 persons)	3 fewer (4 fewer to 27 more)	Very low
**Ketoacidosis due to diabetes**	Alpha-glucosidase inhibitors	Standard treatments	0.40 (0.01 to 16.47)	2 per 1000 persons	1 per 1000 persons (0 to 32 per 1000 persons)	1 fewer (2 fewer to 30 more)	Very low
**Ketoacidosis due to diabetes**	Alpha-glucosidase inhibitors	Sulfonylureas	0.67 (0.03 to 16.50)	1 per 1000 persons	1 per 1000 persons (0 to 16 per 1000 persons)	0 (1 fewer to 15 more)	Very low
**Ketoacidosis due to diabetes**	DPP-4 inhibitors	GLP-1 receptor agonists	0.78 (0.32 to 1.89)	2 per 1000 persons	2 per 1000 persons (1 to 4 per 1000 persons)	0 (1 fewer to 2 more)	Moderate
**Ketoacidosis due to diabetes**	DPP-4 inhibitors	Meglitinides	2.08 (0.05 to 84.81)	1 per 1000 persons	2 per 1000 persons (0 to 78 per 1000 persons)	1 more (1 fewer to 77 more)	Very low
**Ketoacidosis due to diabetes**	DPP-4 inhibitors	Metformin	0.59 (0.02 to 15.88)	3 per 1000 persons	2 per 1000 persons (0 to 46 per 1000 persons)	1 fewer (3 fewer to 43 more)	Low

Outcome	Intervention	Comparator	Odds ratio	Baseline risk	Risk if treated	Risk difference	Certainty
**Ketoacidosis due to diabetes**	DPP-4 inhibitors	Non-steroidal MRAs	1.19 (0.41 to 3.46)	1 per 1000 persons	1 per 1000 persons (0 to 3 per 1000 persons)	0 (1 fewer to 2 more)	Moderate
**Ketoacidosis due to diabetes**	DPP-4 inhibitors	SGLT2 inhibitors	0.39 (0.19 to 0.83)	4 per 1000 persons	2 per 1000 persons (1 to 3 per 1000 persons)	2 fewer (3 fewer to 1 fewer)	Moderate
**Ketoacidosis due to diabetes**	DPP-4 inhibitors	Standard treatments	0.82 (0.41 to 1.61)	2 per 1000 persons	2 per 1000 persons (1 to 3 per 1000 persons)	0 (1 fewer to 1 more)	Moderate
**Ketoacidosis due to diabetes**	DPP-4 inhibitors	Sulfonylureas	1.38 (0.21 to 8.94)	1 per 1000 persons	1 per 1000 persons (0 to 9 per 1000 persons)	0 (1 fewer to 8 more)	Moderate
**Ketoacidosis due to diabetes**	GLP-1 receptor agonists	Meglitinides	2.69 (0.06 to 116.94)	1 per 1000 persons	3 per 1000 persons (0 to 105 per 1000 persons)	2 more (1 fewer to 104 more)	Low
**Ketoacidosis due to diabetes**	GLP-1 receptor agonists	Metformin	0.76 (0.03 to 20.25)	3 per 1000 persons	2 per 1000 persons (0 to 57 per 1000 persons)	1 fewer (3 fewer to 54 more)	Low
**Ketoacidosis due to diabetes**	GLP-1 receptor agonists	Non-steroidal MRAs	1.54 (0.55 to 4.26)	1 per 1000 persons	2 per 1000 persons (1 to 4 per 1000 persons)	1 more (0 to 3 more)	Moderate
**Ketoacidosis due to diabetes**	GLP-1 receptor agonists	SGLT2 inhibitors	0.51 (0.25 to 1.02)	4 per 1000 persons	2 per 1000 persons (1 to 4 per 1000 persons)	2 fewer (3 fewer to 0)	Moderate
**Ketoacidosis due to diabetes**	GLP-1 receptor agonists	Standard treatments	1.05 (0.58 to 1.92)	2 per 1000 persons	2 per 1000 persons (1 to 4 per 1000 persons)	0 (1 fewer to 2 more)	High
**Ketoacidosis due to diabetes**	GLP-1 receptor agonists	Sulfonylureas	1.78 (0.24 to 13.13)	1 per 1000 persons	2 per 1000 persons (0 to 13 per 1000 persons)	1 more (1 fewer to 12 more)	Low
**Ketoacidosis due to diabetes**	Meglitinides	Metformin	0.28 (0.00 to 38.81)	3 per 1000 persons	1 per 1000 persons (0 to 105 per 1000 persons)	2 fewer (3 fewer to 102 more)	Low
**Ketoacidosis due to diabetes**	Meglitinides	Non-steroidal MRAs	0.57 (0.01 to 26.04)	1 per 1000 persons	1 per 1000 persons (0 to 25 per 1000 persons)	0 (1 fewer to 24 more)	Very low
**Ketoacidosis due to diabetes**	Meglitinides	SGLT2 inhibitors	0.19 (0.00 to 7.89)	4 per 1000 persons	1 per 1000 persons (0 to 31 per 1000 persons)	3 fewer (4 fewer to 27 more)	Very low
**Ketoacidosis due to diabetes**	Meglitinides	Standard treatments	0.39 (0.01 to 16.29)	2 per 1000 persons	1 per 1000 persons (0 to 32 per 1000 persons)	1 fewer (2 fewer to 30 more)	Very low
**Ketoacidosis due to diabetes**	Meglitinides	Sulfonylureas	0.66 (0.03 to 16.32)	1 per 1000 persons	1 per 1000 persons (0 to 16 per 1000 persons)	0 (1 fewer to 15 more)	Very low

Outcome	Intervention	Comparator	Odds ratio	Baseline risk	Risk if treated	Risk difference	Certainty
**Ketoacidosis due to diabetes**	Metformin	Non-steroidal MRAs	2.02 (0.07 to 56.23)	1 per 1000 persons	2 per 1000 persons (0 to 53 per 1000 persons)	1 more (1 fewer to 52 more)	Low
**Ketoacidosis due to diabetes**	Metformin	SGLT2 inhibitors	0.67 (0.03 to 16.41)	4 per 1000 persons	3 per 1000 persons (0 to 62 per 1000 persons)	1 fewer (4 fewer to 58 more)	Moderate
**Ketoacidosis due to diabetes**	Metformin	Standard treatments	1.38 (0.05 to 34.70)	2 per 1000 persons	3 per 1000 persons (0 to 65 per 1000 persons)	1 more (2 fewer to 63 more)	Low
**Ketoacidosis due to diabetes**	Metformin	Sulfonylureas	2.34 (0.06 to 97.87)	1 per 1000 persons	2 per 1000 persons (0 to 89 per 1000 persons)	1 more (1 fewer to 88 more)	Low
**Ketoacidosis due to diabetes**	Non-steroidal MRAs	SGLT2 inhibitors	0.33 (0.13 to 0.81)	4 per 1000 persons	1 per 1000 persons (1 to 3 per 1000 persons)	3 fewer (3 fewer to 1 fewer)	Moderate
**Ketoacidosis due to diabetes**	Non-steroidal MRAs	Standard treatments	0.68 (0.30 to 1.56)	2 per 1000 persons	1 per 1000 persons (1 to 3 per 1000 persons)	1 fewer (1 fewer to 1 more)	Moderate
**Ketoacidosis due to diabetes**	Non-steroidal MRAs	Sulfonylureas	1.16 (0.15 to 9.29)	1 per 1000 persons	1 per 1000 persons (0 to 9 per 1000 persons)	0 (1 fewer to 8 more)	Moderate
**Ketoacidosis due to diabetes**	SGLT2 inhibitors	Standard treatments	2.07 (1.44 to 2.98)	2 per 1000 persons	4 per 1000 persons (3 to 6 per 1000 persons)	2 more (1 more to 4 more)	High
**Ketoacidosis due to diabetes**	SGLT2 inhibitors	Sulfonylureas	3.51 (0.52 to 23.87)	1 per 1000 persons	4 per 1000 persons (1 to 23 per 1000 persons)	3 more (0 to 22 more)	Low
**Ketoacidosis due to diabetes**	Sulfonylureas	Standard treatments	0.59 (0.09 to 3.98)	2 per 1000 persons	1 per 1000 persons (0 to 8 per 1000 persons)	1 fewer (2 fewer to 6 more)	Moderate
**Ketoacidosis due to diabetes**	DPP-4 inhibitors	Alpha-glucosidase inhibitors	2.04 (0.05 to 100.00)	1 per 1000 persons	2 per 1000 persons (0 to 91 per 1000 persons)	1 more (1 fewer to 90 more)	Very low
**Ketoacidosis due to diabetes**	GLP-1 receptor agonists	Alpha-glucosidase inhibitors	2.63 (0.06 to 100.00)	1 per 1000 persons	3 per 1000 persons (0 to 91 per 1000 persons)	2 more (1 fewer to 90 more)	Low
**Ketoacidosis due to diabetes**	Meglitinides	Alpha-glucosidase inhibitors	0.99 (0.02 to 50.00)	1 per 1000 persons	1 per 1000 persons (0 to 48 per 1000 persons)	0 (1 fewer to 47 more)	Very low
**Ketoacidosis due to diabetes**	Metformin	Alpha-glucosidase inhibitors	3.45 (0.03 to Inf)	1 per 1000 persons	3 per 1000 persons (0 to NaN per 1000 persons)	2 more (1 fewer to NA)	Moderate
**Ketoacidosis due to diabetes**	Non-steroidal MRAs	Alpha-glucosidase inhibitors	1.72 (0.04 to 100.00)	1 per 1000 persons	2 per 1000 persons (0 to 91 per 1000 persons)	1 more (1 fewer to 90 more)	Very low

Outcome	Intervention	Comparator	Odds ratio	Baseline risk	Risk if treated	Risk difference	Certainty
**Ketoacidosis due to diabetes**	SGLT2 inhibitors	Alpha-glucosidase inhibitors	5.26 (0.13 to Inf)	1 per 1000 persons	5 per 1000 persons (0 to NaN per 1000 persons)	4 more (1 fewer to NA)	Low
**Ketoacidosis due to diabetes**	Standard treatments	Alpha-glucosidase inhibitors	2.50 (0.06 to 100.00)	1 per 1000 persons	2 per 1000 persons (0 to 91 per 1000 persons)	1 more (1 fewer to 90 more)	Very low
**Ketoacidosis due to diabetes**	Sulfonylureas	Alpha-glucosidase inhibitors	1.49 (0.06 to 33.33)	1 per 1000 persons	1 per 1000 persons (0 to 32 per 1000 persons)	0 (1 fewer to 31 more)	Very low
**Ketoacidosis due to diabetes**	GLP-1 receptor agonists	DPP-4 inhibitors	1.28 (0.53 to 3.12)	2 per 1000 persons	3 per 1000 persons (1 to 6 per 1000 persons)	1 more (1 fewer to 4 more)	Moderate
**Ketoacidosis due to diabetes**	Meglitinides	DPP-4 inhibitors	0.48 (0.01 to 20.00)	2 per 1000 persons	1 per 1000 persons (0 to 39 per 1000 persons)	1 fewer (2 fewer to 37 more)	Very low
**Ketoacidosis due to diabetes**	Metformin	DPP-4 inhibitors	1.69 (0.06 to 50.00)	2 per 1000 persons	3 per 1000 persons (0 to 91 per 1000 persons)	1 more (2 fewer to 89 more)	Low
**Ketoacidosis due to diabetes**	Non-steroidal MRAs	DPP-4 inhibitors	0.84 (0.29 to 2.44)	2 per 1000 persons	2 per 1000 persons (1 to 5 per 1000 persons)	0 (1 fewer to 3 more)	Moderate
**Ketoacidosis due to diabetes**	SGLT2 inhibitors	DPP-4 inhibitors	2.56 (1.20 to 5.26)	2 per 1000 persons	5 per 1000 persons (2 to 10 per 1000 persons)	3 more (0 to 8 more)	Moderate
**Ketoacidosis due to diabetes**	Standard treatments	DPP-4 inhibitors	1.22 (0.62 to 2.44)	2 per 1000 persons	2 per 1000 persons (1 to 5 per 1000 persons)	0 (1 fewer to 3 more)	Moderate
**Ketoacidosis due to diabetes**	Sulfonylureas	DPP-4 inhibitors	0.72 (0.11 to 4.76)	2 per 1000 persons	1 per 1000 persons (0 to 9 per 1000 persons)	1 fewer (2 fewer to 7 more)	Moderate
**Ketoacidosis due to diabetes**	Meglitinides	GLP-1 receptor agonists	0.37 (0.01 to 16.67)	2 per 1000 persons	1 per 1000 persons (0 to 32 per 1000 persons)	1 fewer (2 fewer to 30 more)	Low
**Ketoacidosis due to diabetes**	Metformin	GLP-1 receptor agonists	1.32 (0.05 to 33.33)	2 per 1000 persons	3 per 1000 persons (0 to 63 per 1000 persons)	1 more (2 fewer to 61 more)	Low
**Ketoacidosis due to diabetes**	Non-steroidal MRAs	GLP-1 receptor agonists	0.65 (0.23 to 1.82)	2 per 1000 persons	1 per 1000 persons (0 to 4 per 1000 persons)	1 fewer (2 fewer to 2 more)	Moderate
**Ketoacidosis due to diabetes**	SGLT2 inhibitors	GLP-1 receptor agonists	1.96 (0.98 to 4.00)	2 per 1000 persons	4 per 1000 persons (2 to 8 per 1000 persons)	2 more (0 to 6 more)	Moderate
**Ketoacidosis due to diabetes**	Standard treatments	GLP-1 receptor agonists	0.95 (0.52 to 1.72)	2 per 1000 persons	2 per 1000 persons (1 to 3 per 1000 persons)	0 (1 fewer to 1 more)	High

Outcome	Intervention	Comparator	Odds ratio	Baseline risk	Risk if treated	Risk difference	Certainty
**Ketoacidosis due to diabetes**	Sulfonylureas	GLP-1 receptor agonists	0.56 (0.08 to 4.17)	2 per 1000 persons	1 per 1000 persons (0 to 8 per 1000 persons)	1 fewer (2 fewer to 6 more)	Moderate
**Ketoacidosis due to diabetes**	Metformin	Meglitinides	3.57 (0.03 to Inf)	1 per 1000 persons	4 per 1000 persons (0 to NaN per 1000 persons)	3 more (1 fewer to NA)	Moderate
**Ketoacidosis due to diabetes**	Non-steroidal MRAs	Meglitinides	1.75 (0.04 to 100.00)	1 per 1000 persons	2 per 1000 persons (0 to 91 per 1000 persons)	1 more (1 fewer to 90 more)	Very low
**Ketoacidosis due to diabetes**	SGLT2 inhibitors	Meglitinides	5.26 (0.13 to Inf)	1 per 1000 persons	5 per 1000 persons (0 to NaN per 1000 persons)	4 more (1 fewer to NA)	Low
**Ketoacidosis due to diabetes**	Standard treatments	Meglitinides	2.56 (0.06 to 100.00)	1 per 1000 persons	3 per 1000 persons (0 to 91 per 1000 persons)	2 more (1 fewer to 90 more)	Very low
**Ketoacidosis due to diabetes**	Sulfonylureas	Meglitinides	1.52 (0.06 to 33.33)	1 per 1000 persons	2 per 1000 persons (0 to 32 per 1000 persons)	1 more (1 fewer to 31 more)	Very low
**Ketoacidosis due to diabetes**	Non-steroidal MRAs	Metformin	0.50 (0.02 to 14.29)	3 per 1000 persons	1 per 1000 persons (0 to 41 per 1000 persons)	2 fewer (3 fewer to 38 more)	Low
**Ketoacidosis due to diabetes**	SGLT2 inhibitors	Metformin	1.49 (0.06 to 33.33)	3 per 1000 persons	4 per 1000 persons (0 to 91 per 1000 persons)	1 more (3 fewer to 88 more)	Moderate
**Ketoacidosis due to diabetes**	Standard treatments	Metformin	0.72 (0.03 to 20.00)	3 per 1000 persons	2 per 1000 persons (0 to 57 per 1000 persons)	1 fewer (3 fewer to 54 more)	Low
**Ketoacidosis due to diabetes**	Sulfonylureas	Metformin	0.43 (0.01 to 16.67)	3 per 1000 persons	1 per 1000 persons (0 to 48 per 1000 persons)	2 fewer (3 fewer to 45 more)	Low
**Ketoacidosis due to diabetes**	SGLT2 inhibitors	Non-steroidal MRAs	3.03 (1.23 to 7.69)	1 per 1000 persons	3 per 1000 persons (1 to 8 per 1000 persons)	2 more (0 to 7 more)	Moderate
**Ketoacidosis due to diabetes**	Standard treatments	Non-steroidal MRAs	1.47 (0.64 to 3.33)	1 per 1000 persons	1 per 1000 persons (1 to 3 per 1000 persons)	0 (0 to 2 more)	Moderate
**Ketoacidosis due to diabetes**	Sulfonylureas	Non-steroidal MRAs	0.86 (0.11 to 6.67)	1 per 1000 persons	1 per 1000 persons (0 to 7 per 1000 persons)	0 (1 fewer to 6 more)	Moderate
**Ketoacidosis due to diabetes**	Standard treatments	SGLT2 inhibitors	0.48 (0.34 to 0.69)	4 per 1000 persons	2 per 1000 persons (1 to 3 per 1000 persons)	2 fewer (3 fewer to 1 fewer)	High
**Ketoacidosis due to diabetes**	Sulfonylureas	SGLT2 inhibitors	0.28 (0.04 to 1.92)	4 per 1000 persons	1 per 1000 persons (0 to 8 per 1000 persons)	3 fewer (4 fewer to 4 more)	Moderate

**Outcome**	**Intervention**	**Comparator**	**Odds ratio**	**Baseline risk**	**Risk if treated**	**Risk difference**	**Certainty**
**Ketoacidosis due to diabetes**	Standard treatments	Sulfonylureas	1.69 (0.25 to 11.11)	1 per 1000 persons	2 per 1000 persons (0 to 11 per 1000 persons)	1 more (1 fewer to 10 more)	Moderate

6.2 In patients with more than 3 cardiovascular risk factors

Outcome	Intervention	Comparator	Odds ratio	Baseline risk	Risk if treated	Risk difference	Certainty
**All-cause death**	Alpha-glucosidase inhibitors	Basal insulin	0.81 (0.26 to 2.47)	76 per 1000 persons	62 per 1000 persons (21 to 169 per 1000 persons)	14 fewer (55 fewer to 93 more)	Very low
**All-cause death**	Alpha-glucosidase inhibitors	Basal-Bolus insulin	1.12 (0.19 to 6.74)	56 per 1000 persons	62 per 1000 persons (11 to 286 per 1000 persons)	6 more (45 fewer to 230 more)	Very low
**All-cause death**	Alpha-glucosidase inhibitors	Bolus insulin	1.85 (0.37 to 9.23)	35 per 1000 persons	63 per 1000 persons (13 to 251 per 1000 persons)	28 more (22 fewer to 216 more)	Very low
**All-cause death**	Alpha-glucosidase inhibitors	DPP-4 inhibitors	0.88 (0.30 to 2.58)	71 per 1000 persons	63 per 1000 persons (22 to 165 per 1000 persons)	8 fewer (49 fewer to 94 more)	Very low
**All-cause death**	Alpha-glucosidase inhibitors	GLP-1 receptor agonists	1.01 (0.34 to 2.98)	62 per 1000 persons	63 per 1000 persons (22 to 165 per 1000 persons)	1 more (40 fewer to 103 more)	Very low
**All-cause death**	Alpha-glucosidase inhibitors	Meglitinides	0.56 (0.13 to 2.48)	106 per 1000 persons	62 per 1000 persons (15 to 227 per 1000 persons)	44 fewer (91 fewer to 121 more)	Very low
**All-cause death**	Alpha-glucosidase inhibitors	Metformin	1.06 (0.36 to 3.18)	59 per 1000 persons	62 per 1000 persons (22 to 166 per 1000 persons)	3 more (37 fewer to 107 more)	Very low
**All-cause death**	Alpha-glucosidase inhibitors	Non-steroidal MRAs	1.00 (0.34 to 2.95)	63 per 1000 persons	63 per 1000 persons (22 to 166 per 1000 persons)	0 (41 fewer to 103 more)	Very low
**All-cause death**	Alpha-glucosidase inhibitors	SGLT2 inhibitors	1.01 (0.34 to 2.96)	62 per 1000 persons	63 per 1000 persons (22 to 164 per 1000 persons)	1 more (40 fewer to 102 more)	Very low
**All-cause death**	Alpha-glucosidase inhibitors	Standard treatments	0.89 (0.30 to 2.61)	70 per 1000 persons	63 per 1000 persons (22 to 164 per 1000 persons)	7 fewer (48 fewer to 94 more)	Very low
**All-cause death**	Alpha-glucosidase inhibitors	Sulfonylureas	0.81 (0.27 to 2.37)	76 per 1000 persons	62 per 1000 persons (22 to 163 per 1000 persons)	14 fewer (54 fewer to 87 more)	Very low
**All-cause death**	Alpha-glucosidase inhibitors	Thiazolidinediones	0.93 (0.32 to 2.76)	67 per 1000 persons	63 per 1000 persons (22 to 165 per 1000 persons)	4 fewer (45 fewer to 98 more)	Very low
**All-cause death**	Alpha-glucosidase inhibitors	Tirzepatide	1.07 (0.32 to 3.57)	59 per 1000 persons	63 per 1000 persons (20 to 183 per 1000 persons)	4 more (39 fewer to 124 more)	Very low
**All-cause death**	Basal insulin	Basal-Bolus insulin	1.39 (0.33 to 5.92)	56 per 1000 persons	76 per 1000 persons (19 to 260 per 1000 persons)	20 more (37 fewer to 204 more)	Very low

Outcome	Intervention	Comparator	Odds ratio	Baseline risk	Risk if treated	Risk difference	Certainty
**All-cause death**	Basal insulin	Bolus insulin	2.28 (0.67 to 7.75)	35 per 1000 persons	76 per 1000 persons (24 to 219 per 1000 persons)	41 more (11 fewer to 184 more)	Very low
**All-cause death**	Basal insulin	DPP-4 inhibitors	1.08 (0.80 to 1.47)	71 per 1000 persons	76 per 1000 persons (58 to 101 per 1000 persons)	5 more (13 fewer to 30 more)	Very low
**All-cause death**	Basal insulin	GLP-1 receptor agonists	1.25 (0.93 to 1.70)	62 per 1000 persons	76 per 1000 persons (58 to 101 per 1000 persons)	14 more (4 fewer to 39 more)	Low
**All-cause death**	Basal insulin	Meglitinides	0.69 (0.22 to 2.24)	106 per 1000 persons	76 per 1000 persons (25 to 210 per 1000 persons)	30 fewer (81 fewer to 104 more)	Very low
**All-cause death**	Basal insulin	Metformin	1.31 (0.91 to 1.90)	59 per 1000 persons	76 per 1000 persons (54 to 106 per 1000 persons)	17 more (5 fewer to 47 more)	Low
**All-cause death**	Basal insulin	Non-steroidal MRAs	1.24 (0.89 to 1.71)	63 per 1000 persons	77 per 1000 persons (56 to 103 per 1000 persons)	14 more (7 fewer to 40 more)	Low
**All-cause death**	Basal insulin	SGLT2 inhibitors	1.24 (0.91 to 1.70)	62 per 1000 persons	76 per 1000 persons (57 to 101 per 1000 persons)	14 more (5 fewer to 39 more)	Low
**All-cause death**	Basal insulin	Standard treatments	1.10 (0.81 to 1.49)	70 per 1000 persons	76 per 1000 persons (57 to 101 per 1000 persons)	6 more (13 fewer to 31 more)	Very low
**All-cause death**	Basal insulin	Sulfonylureas	1.00 (0.73 to 1.36)	76 per 1000 persons	76 per 1000 persons (57 to 101 per 1000 persons)	0 (19 fewer to 25 more)	Low
**All-cause death**	Basal insulin	Thiazolidinediones	1.15 (0.83 to 1.60)	67 per 1000 persons	76 per 1000 persons (56 to 103 per 1000 persons)	9 more (11 fewer to 36 more)	Very low
**All-cause death**	Basal insulin	Tirzepatide	1.32 (0.82 to 2.13)	59 per 1000 persons	76 per 1000 persons (49 to 118 per 1000 persons)	17 more (10 fewer to 59 more)	Moderate
**All-cause death**	Basal-Bolus insulin	Bolus insulin	1.64 (0.25 to 10.61)	35 per 1000 persons	56 per 1000 persons (9 to 278 per 1000 persons)	21 more (26 fewer to 243 more)	Very low
**All-cause death**	Basal-Bolus insulin	DPP-4 inhibitors	0.78 (0.19 to 3.28)	71 per 1000 persons	56 per 1000 persons (14 to 200 per 1000 persons)	15 fewer (57 fewer to 129 more)	Very low
**All-cause death**	Basal-Bolus insulin	GLP-1 receptor agonists	0.90 (0.22 to 3.78)	62 per 1000 persons	56 per 1000 persons (14 to 200 per 1000 persons)	6 fewer (48 fewer to 138 more)	Very low
**All-cause death**	Basal-Bolus insulin	Meglitinides	0.50 (0.08 to 3.11)	106 per 1000 persons	56 per 1000 persons (9 to 269 per 1000 persons)	50 fewer (97 fewer to 163 more)	Very low

Outcome	Intervention	Comparator	Odds ratio	Baseline risk	Risk if treated	Risk difference	Certainty
**All-cause death**	Basal-Bolus insulin	Metformin	0.95 (0.22 to 4.04)	59 per 1000 persons	56 per 1000 persons (14 to 202 per 1000 persons)	3 fewer (45 fewer to 143 more)	Very low
**All-cause death**	Basal-Bolus insulin	Non-steroidal MRAs	0.89 (0.21 to 3.75)	63 per 1000 persons	56 per 1000 persons (14 to 201 per 1000 persons)	7 fewer (49 fewer to 138 more)	Very low
**All-cause death**	Basal-Bolus insulin	SGLT2 inhibitors	0.90 (0.21 to 3.76)	62 per 1000 persons	56 per 1000 persons (14 to 199 per 1000 persons)	6 fewer (48 fewer to 137 more)	Very low
**All-cause death**	Basal-Bolus insulin	Standard treatments	0.79 (0.19 to 3.32)	70 per 1000 persons	56 per 1000 persons (14 to 200 per 1000 persons)	14 fewer (56 fewer to 130 more)	Very low
**All-cause death**	Basal-Bolus insulin	Sulfonylureas	0.72 (0.17 to 3.02)	76 per 1000 persons	56 per 1000 persons (14 to 199 per 1000 persons)	20 fewer (62 fewer to 123 more)	Very low
**All-cause death**	Basal-Bolus insulin	Thiazolidinediones	0.83 (0.20 to 3.51)	67 per 1000 persons	56 per 1000 persons (14 to 201 per 1000 persons)	11 fewer (53 fewer to 134 more)	Very low
**All-cause death**	Basal-Bolus insulin	Tirzepatide	0.95 (0.21 to 4.35)	59 per 1000 persons	56 per 1000 persons (13 to 214 per 1000 persons)	3 fewer (46 fewer to 155 more)	Very low
**All-cause death**	Bolus insulin	DPP-4 inhibitors	0.47 (0.14 to 1.57)	71 per 1000 persons	35 per 1000 persons (11 to 107 per 1000 persons)	36 fewer (60 fewer to 36 more)	Very low
**All-cause death**	Bolus insulin	GLP-1 receptor agonists	0.55 (0.17 to 1.81)	62 per 1000 persons	35 per 1000 persons (11 to 107 per 1000 persons)	27 fewer (51 fewer to 45 more)	Very low
**All-cause death**	Bolus insulin	Meglitinides	0.30 (0.06 to 1.58)	106 per 1000 persons	34 per 1000 persons (7 to 158 per 1000 persons)	72 fewer (99 fewer to 52 more)	Very low
**All-cause death**	Bolus insulin	Metformin	0.58 (0.17 to 1.94)	59 per 1000 persons	35 per 1000 persons (11 to 108 per 1000 persons)	24 fewer (48 fewer to 49 more)	Very low
**All-cause death**	Bolus insulin	Non-steroidal MRAs	0.54 (0.16 to 1.80)	63 per 1000 persons	35 per 1000 persons (11 to 108 per 1000 persons)	28 fewer (52 fewer to 45 more)	Very low
**All-cause death**	Bolus insulin	SGLT2 inhibitors	0.54 (0.16 to 1.81)	62 per 1000 persons	34 per 1000 persons (10 to 107 per 1000 persons)	28 fewer (52 fewer to 45 more)	Very low
**All-cause death**	Bolus insulin	Standard treatments	0.48 (0.15 to 1.59)	70 per 1000 persons	35 per 1000 persons (11 to 107 per 1000 persons)	35 fewer (59 fewer to 37 more)	Very low
**All-cause death**	Bolus insulin	Sulfonylureas	0.44 (0.13 to 1.45)	76 per 1000 persons	35 per 1000 persons (11 to 107 per 1000 persons)	41 fewer (65 fewer to 31 more)	Very low

Outcome	Intervention	Comparator	Odds ratio	Baseline risk	Risk if treated	Risk difference	Certainty
**All-cause death**	Bolus insulin	Thiazolidinediones	0.51 (0.15 to 1.68)	67 per 1000 persons	35 per 1000 persons (11 to 108 per 1000 persons)	32 fewer (56 fewer to 41 more)	Very low
**All-cause death**	Bolus insulin	Tirzepatide	0.58 (0.16 to 2.13)	59 per 1000 persons	35 per 1000 persons (10 to 118 per 1000 persons)	24 fewer (49 fewer to 59 more)	Very low
**All-cause death**	DPP-4 inhibitors	GLP-1 receptor agonists	1.16 (1.06 to 1.26)	62 per 1000 persons	71 per 1000 persons (65 to 77 per 1000 persons)	9 more (3 more to 15 more)	Low
**All-cause death**	DPP-4 inhibitors	Meglitinides	0.64 (0.21 to 1.99)	106 per 1000 persons	71 per 1000 persons (24 to 191 per 1000 persons)	35 fewer (82 fewer to 85 more)	Very low
**All-cause death**	DPP-4 inhibitors	Metformin	1.21 (0.97 to 1.52)	59 per 1000 persons	71 per 1000 persons (57 to 87 per 1000 persons)	12 more (2 fewer to 28 more)	Low
**All-cause death**	DPP-4 inhibitors	Non-steroidal MRAs	1.14 (0.99 to 1.31)	63 per 1000 persons	71 per 1000 persons (62 to 81 per 1000 persons)	8 more (1 fewer to 18 more)	Low
**All-cause death**	DPP-4 inhibitors	SGLT2 inhibitors	1.15 (1.04 to 1.26)	62 per 1000 persons	71 per 1000 persons (64 to 77 per 1000 persons)	9 more (2 more to 15 more)	Low
**All-cause death**	DPP-4 inhibitors	Standard treatments	1.01 (0.95 to 1.08)	70 per 1000 persons	71 per 1000 persons (67 to 75 per 1000 persons)	1 more (3 fewer to 5 more)	Moderate
**All-cause death**	DPP-4 inhibitors	Sulfonylureas	0.92 (0.81 to 1.04)	76 per 1000 persons	70 per 1000 persons (62 to 79 per 1000 persons)	6 fewer (14 fewer to 3 more)	Low
**All-cause death**	DPP-4 inhibitors	Thiazolidinediones	1.06 (0.92 to 1.23)	67 per 1000 persons	71 per 1000 persons (62 to 81 per 1000 persons)	4 more (5 fewer to 14 more)	Low
**All-cause death**	DPP-4 inhibitors	Tirzepatide	1.22 (0.70 to 2.11)	59 per 1000 persons	71 per 1000 persons (42 to 117 per 1000 persons)	12 more (17 fewer to 58 more)	Very low
**All-cause death**	GLP-1 receptor agonists	Meglitinides	0.55 (0.18 to 1.73)	106 per 1000 persons	61 per 1000 persons (21 to 170 per 1000 persons)	45 fewer (85 fewer to 64 more)	Very low
**All-cause death**	GLP-1 receptor agonists	Metformin	1.05 (0.84 to 1.31)	59 per 1000 persons	62 per 1000 persons (50 to 76 per 1000 persons)	3 more (9 fewer to 17 more)	Low
**All-cause death**	GLP-1 receptor agonists	Non-steroidal MRAs	0.99 (0.86 to 1.13)	63 per 1000 persons	62 per 1000 persons (55 to 71 per 1000 persons)	1 fewer (8 fewer to 8 more)	Moderate
**All-cause death**	GLP-1 receptor agonists	SGLT2 inhibitors	0.99 (0.91 to 1.08)	62 per 1000 persons	61 per 1000 persons (57 to 67 per 1000 persons)	1 fewer (5 fewer to 5 more)	Moderate

Outcome	Intervention	Comparator	Odds ratio	Baseline risk	Risk if treated	Risk difference	Certainty
**All-cause death**	GLP-1 receptor agonists	Standard treatments	0.88 (0.82 to 0.93)	70 per 1000 persons	62 per 1000 persons (58 to 65 per 1000 persons)	8 fewer (12 fewer to 5 fewer)	Moderate
**All-cause death**	GLP-1 receptor agonists	Sulfonylureas	0.79 (0.69 to 0.92)	76 per 1000 persons	61 per 1000 persons (54 to 70 per 1000 persons)	15 fewer (22 fewer to 6 fewer)	Moderate
**All-cause death**	GLP-1 receptor agonists	Thiazolidinediones	0.92 (0.80 to 1.07)	67 per 1000 persons	62 per 1000 persons (54 to 71 per 1000 persons)	5 fewer (13 fewer to 4 more)	Low
**All-cause death**	GLP-1 receptor agonists	Tirzepatide	1.05 (0.61 to 1.82)	59 per 1000 persons	62 per 1000 persons (37 to 102 per 1000 persons)	3 more (22 fewer to 43 more)	Very low
**All-cause death**	Meglitinides	Metformin	1.89 (0.60 to 5.97)	59 per 1000 persons	106 per 1000 persons (36 to 272 per 1000 persons)	47 more (23 fewer to 213 more)	Very low
**All-cause death**	Meglitinides	Non-steroidal MRAs	1.78 (0.57 to 5.57)	63 per 1000 persons	107 per 1000 persons (37 to 272 per 1000 persons)	44 more (26 fewer to 209 more)	Very low
**All-cause death**	Meglitinides	SGLT2 inhibitors	1.79 (0.58 to 5.58)	62 per 1000 persons	106 per 1000 persons (37 to 269 per 1000 persons)	44 more (25 fewer to 207 more)	Very low
**All-cause death**	Meglitinides	Standard treatments	1.58 (0.51 to 4.92)	70 per 1000 persons	106 per 1000 persons (37 to 270 per 1000 persons)	36 more (33 fewer to 200 more)	Very low
**All-cause death**	Meglitinides	Sulfonylureas	1.43 (0.46 to 4.45)	76 per 1000 persons	105 per 1000 persons (36 to 268 per 1000 persons)	29 more (40 fewer to 192 more)	Very low
**All-cause death**	Meglitinides	Thiazolidinediones	1.66 (0.53 to 5.19)	67 per 1000 persons	107 per 1000 persons (37 to 272 per 1000 persons)	40 more (30 fewer to 205 more)	Very low
**All-cause death**	Meglitinides	Tirzepatide	1.90 (0.54 to 6.68)	59 per 1000 persons	106 per 1000 persons (33 to 295 per 1000 persons)	47 more (26 fewer to 236 more)	Very low
**All-cause death**	Metformin	Non-steroidal MRAs	0.94 (0.73 to 1.20)	63 per 1000 persons	59 per 1000 persons (47 to 75 per 1000 persons)	4 fewer (16 fewer to 12 more)	Very low
**All-cause death**	Metformin	SGLT2 inhibitors	0.95 (0.76 to 1.19)	62 per 1000 persons	59 per 1000 persons (48 to 73 per 1000 persons)	3 fewer (14 fewer to 11 more)	Very low
**All-cause death**	Metformin	Standard treatments	0.84 (0.67 to 1.04)	70 per 1000 persons	59 per 1000 persons (48 to 73 per 1000 persons)	11 fewer (22 fewer to 3 more)	Low
**All-cause death**	Metformin	Sulfonylureas	0.76 (0.60 to 0.96)	76 per 1000 persons	59 per 1000 persons (47 to 73 per 1000 persons)	17 fewer (29 fewer to 3 fewer)	Moderate

Outcome	Intervention	Comparator	Odds ratio	Baseline risk	Risk if treated	Risk difference	Certainty
**All-cause death**	Metformin	Thiazolidinediones	0.88 (0.69 to 1.12)	67 per 1000 persons	59 per 1000 persons (47 to 74 per 1000 persons)	8 fewer (20 fewer to 7 more)	Low
**All-cause death**	Metformin	Tirzepatide	1.00 (0.56 to 1.81)	59 per 1000 persons	59 per 1000 persons (34 to 102 per 1000 persons)	0 (25 fewer to 43 more)	Very low
**All-cause death**	Non-steroidal MRAs	SGLT2 inhibitors	1.01 (0.88 to 1.15)	62 per 1000 persons	63 per 1000 persons (55 to 71 per 1000 persons)	1 more (7 fewer to 9 more)	Moderate
**All-cause death**	Non-steroidal MRAs	Standard treatments	0.89 (0.79 to 1.00)	70 per 1000 persons	63 per 1000 persons (56 to 70 per 1000 persons)	7 fewer (14 fewer to 0)	Low
**All-cause death**	Non-steroidal MRAs	Sulfonylureas	0.81 (0.67 to 0.96)	76 per 1000 persons	62 per 1000 persons (52 to 73 per 1000 persons)	14 fewer (24 fewer to 3 fewer)	Moderate
**All-cause death**	Non-steroidal MRAs	Thiazolidinediones	0.93 (0.78 to 1.12)	67 per 1000 persons	63 per 1000 persons (53 to 74 per 1000 persons)	4 fewer (14 fewer to 7 more)	Low
**All-cause death**	Non-steroidal MRAs	Tirzepatide	1.07 (0.61 to 1.87)	59 per 1000 persons	63 per 1000 persons (37 to 105 per 1000 persons)	4 more (22 fewer to 46 more)	Very low
**All-cause death**	SGLT2 inhibitors	Standard treatments	0.88 (0.83 to 0.94)	70 per 1000 persons	62 per 1000 persons (59 to 66 per 1000 persons)	8 fewer (11 fewer to 4 fewer)	Moderate
**All-cause death**	SGLT2 inhibitors	Sulfonylureas	0.80 (0.69 to 0.93)	76 per 1000 persons	62 per 1000 persons (54 to 71 per 1000 persons)	14 fewer (22 fewer to 5 fewer)	Moderate
**All-cause death**	SGLT2 inhibitors	Thiazolidinediones	0.93 (0.80 to 1.08)	67 per 1000 persons	63 per 1000 persons (54 to 72 per 1000 persons)	4 fewer (13 fewer to 5 more)	Low
**All-cause death**	SGLT2 inhibitors	Tirzepatide	1.06 (0.61 to 1.84)	59 per 1000 persons	62 per 1000 persons (37 to 103 per 1000 persons)	3 more (22 fewer to 44 more)	Very low
**All-cause death**	Sulfonylureas	Standard treatments	1.10 (0.97 to 1.26)	70 per 1000 persons	76 per 1000 persons (68 to 87 per 1000 persons)	6 more (2 fewer to 17 more)	Low
**All-cause death**	Sulfonylureas	Thiazolidinediones	1.16 (0.98 to 1.37)	67 per 1000 persons	77 per 1000 persons (66 to 90 per 1000 persons)	10 more (1 fewer to 23 more)	Low
**All-cause death**	Sulfonylureas	Tirzepatide	1.32 (0.76 to 2.31)	59 per 1000 persons	76 per 1000 persons (45 to 127 per 1000 persons)	17 more (14 fewer to 68 more)	Very low
**All-cause death**	Thiazolidinediones	Standard treatments	0.95 (0.83 to 1.09)	70 per 1000 persons	67 per 1000 persons (59 to 76 per 1000 persons)	3 fewer (11 fewer to 6 more)	Low

Outcome	Intervention	Comparator	Odds ratio	Baseline risk	Risk if treated	Risk difference	Certainty
**All-cause death**	Thiazolidinediones	Tirzepatide	1.14 (0.65 to 2.01)	59 per 1000 persons	67 per 1000 persons (39 to 112 per 1000 persons)	8 more (20 fewer to 53 more)	Very low
**All-cause death**	Tirzepatide	Standard treatments	0.83 (0.48 to 1.44)	70 per 1000 persons	59 per 1000 persons (35 to 98 per 1000 persons)	11 fewer (35 fewer to 28 more)	Very low
**All-cause death**	Basal insulin	Alpha-glucosidase inhibitors	1.23 (0.40 to 3.85)	63 per 1000 persons	77 per 1000 persons (26 to 205 per 1000 persons)	14 more (37 fewer to 142 more)	Very low
**All-cause death**	Basal-Bolus insulin	Alpha-glucosidase inhibitors	0.89 (0.15 to 5.26)	63 per 1000 persons	57 per 1000 persons (10 to 261 per 1000 persons)	6 fewer (53 fewer to 198 more)	Very low
**All-cause death**	Bolus insulin	Alpha-glucosidase inhibitors	0.54 (0.11 to 2.70)	63 per 1000 persons	35 per 1000 persons (7 to 154 per 1000 persons)	28 fewer (56 fewer to 91 more)	Very low
**All-cause death**	DPP-4 inhibitors	Alpha-glucosidase inhibitors	1.14 (0.39 to 3.33)	63 per 1000 persons	71 per 1000 persons (25 to 183 per 1000 persons)	8 more (38 fewer to 120 more)	Very low
**All-cause death**	GLP-1 receptor agonists	Alpha-glucosidase inhibitors	0.99 (0.34 to 2.94)	63 per 1000 persons	62 per 1000 persons (22 to 165 per 1000 persons)	1 fewer (41 fewer to 102 more)	Very low
**All-cause death**	Meglitinides	Alpha-glucosidase inhibitors	1.79 (0.40 to 7.69)	63 per 1000 persons	107 per 1000 persons (26 to 341 per 1000 persons)	44 more (37 fewer to 278 more)	Very low
**All-cause death**	Metformin	Alpha-glucosidase inhibitors	0.94 (0.31 to 2.78)	63 per 1000 persons	60 per 1000 persons (21 to 157 per 1000 persons)	3 fewer (42 fewer to 94 more)	Very low
**All-cause death**	Non-steroidal MRAs	Alpha-glucosidase inhibitors	1.00 (0.34 to 2.94)	63 per 1000 persons	63 per 1000 persons (22 to 165 per 1000 persons)	0 (41 fewer to 102 more)	Very low
**All-cause death**	SGLT2 inhibitors	Alpha-glucosidase inhibitors	0.99 (0.34 to 2.94)	63 per 1000 persons	62 per 1000 persons (22 to 165 per 1000 persons)	1 fewer (41 fewer to 102 more)	Very low
**All-cause death**	Standard treatments	Alpha-glucosidase inhibitors	1.12 (0.38 to 3.33)	63 per 1000 persons	70 per 1000 persons (25 to 183 per 1000 persons)	7 more (38 fewer to 120 more)	Very low
**All-cause death**	Sulfonylureas	Alpha-glucosidase inhibitors	1.23 (0.42 to 3.70)	63 per 1000 persons	77 per 1000 persons (28 to 199 per 1000 persons)	14 more (35 fewer to 136 more)	Very low
**All-cause death**	Thiazolidinediones	Alpha-glucosidase inhibitors	1.08 (0.36 to 3.12)	63 per 1000 persons	67 per 1000 persons (24 to 174 per 1000 persons)	4 more (39 fewer to 111 more)	Very low
**All-cause death**	Tirzepatide	Alpha-glucosidase inhibitors	0.93 (0.28 to 3.12)	63 per 1000 persons	59 per 1000 persons (18 to 174 per 1000 persons)	4 fewer (45 fewer to 111 more)	Very low

Outcome	Intervention	Comparator	Odds ratio	Baseline risk	Risk if treated	Risk difference	Certainty
**All-cause death**	Basal-Bolus insulin	Basal insulin	0.72 (0.17 to 3.03)	76 per 1000 persons	56 per 1000 persons (14 to 200 per 1000 persons)	20 fewer (62 fewer to 124 more)	Very low
**All-cause death**	Bolus insulin	Basal insulin	0.44 (0.13 to 1.49)	76 per 1000 persons	35 per 1000 persons (11 to 109 per 1000 persons)	41 fewer (65 fewer to 33 more)	Very low
**All-cause death**	DPP-4 inhibitors	Basal insulin	0.93 (0.68 to 1.25)	76 per 1000 persons	71 per 1000 persons (53 to 93 per 1000 persons)	5 fewer (23 fewer to 17 more)	Very low
**All-cause death**	GLP-1 receptor agonists	Basal insulin	0.80 (0.59 to 1.08)	76 per 1000 persons	62 per 1000 persons (46 to 81 per 1000 persons)	14 fewer (30 fewer to 5 more)	Low
**All-cause death**	Meglitinides	Basal insulin	1.45 (0.45 to 4.55)	76 per 1000 persons	107 per 1000 persons (35 to 272 per 1000 persons)	31 more (41 fewer to 196 more)	Very low
**All-cause death**	Metformin	Basal insulin	0.76 (0.53 to 1.10)	76 per 1000 persons	59 per 1000 persons (41 to 83 per 1000 persons)	17 fewer (35 fewer to 7 more)	Low
**All-cause death**	Non-steroidal MRAs	Basal insulin	0.81 (0.58 to 1.12)	76 per 1000 persons	62 per 1000 persons (46 to 85 per 1000 persons)	14 fewer (30 fewer to 9 more)	Low
**All-cause death**	SGLT2 inhibitors	Basal insulin	0.81 (0.59 to 1.10)	76 per 1000 persons	62 per 1000 persons (46 to 83 per 1000 persons)	14 fewer (30 fewer to 7 more)	Low
**All-cause death**	Standard treatments	Basal insulin	0.91 (0.67 to 1.23)	76 per 1000 persons	70 per 1000 persons (52 to 92 per 1000 persons)	6 fewer (24 fewer to 16 more)	Very low
**All-cause death**	Sulfonylureas	Basal insulin	1.00 (0.74 to 1.37)	76 per 1000 persons	76 per 1000 persons (57 to 101 per 1000 persons)	0 (19 fewer to 25 more)	Low
**All-cause death**	Thiazolidinediones	Basal insulin	0.87 (0.62 to 1.20)	76 per 1000 persons	67 per 1000 persons (49 to 90 per 1000 persons)	9 fewer (27 fewer to 14 more)	Very low
**All-cause death**	Tirzepatide	Basal insulin	0.76 (0.47 to 1.22)	76 per 1000 persons	59 per 1000 persons (37 to 91 per 1000 persons)	17 fewer (39 fewer to 15 more)	Low
**All-cause death**	Bolus insulin	Basal-Bolus insulin	0.61 (0.09 to 4.00)	56 per 1000 persons	35 per 1000 persons (6 to 192 per 1000 persons)	21 fewer (50 fewer to 136 more)	Very low
**All-cause death**	DPP-4 inhibitors	Basal-Bolus insulin	1.28 (0.30 to 5.26)	56 per 1000 persons	71 per 1000 persons (18 to 238 per 1000 persons)	15 more (38 fewer to 182 more)	Very low
**All-cause death**	GLP-1 receptor agonists	Basal-Bolus insulin	1.11 (0.26 to 4.55)	56 per 1000 persons	62 per 1000 persons (15 to 212 per 1000 persons)	6 more (41 fewer to 156 more)	Very low

Outcome	Intervention	Comparator	Odds ratio	Baseline risk	Risk if treated	Risk difference	Certainty
**All-cause death**	Meglitinides	Basal-Bolus insulin	2.00 (0.32 to 12.50)	56 per 1000 persons	106 per 1000 persons (19 to 426 per 1000 persons)	50 more (37 fewer to 370 more)	Very low
**All-cause death**	Metformin	Basal-Bolus insulin	1.05 (0.25 to 4.55)	56 per 1000 persons	59 per 1000 persons (14 to 212 per 1000 persons)	3 more (42 fewer to 156 more)	Very low
**All-cause death**	Non-steroidal MRAs	Basal-Bolus insulin	1.12 (0.27 to 4.76)	56 per 1000 persons	62 per 1000 persons (16 to 220 per 1000 persons)	6 more (40 fewer to 164 more)	Very low
**All-cause death**	SGLT2 inhibitors	Basal-Bolus insulin	1.11 (0.27 to 4.76)	56 per 1000 persons	62 per 1000 persons (16 to 220 per 1000 persons)	6 more (40 fewer to 164 more)	Very low
**All-cause death**	Standard treatments	Basal-Bolus insulin	1.27 (0.30 to 5.26)	56 per 1000 persons	70 per 1000 persons (18 to 238 per 1000 persons)	14 more (38 fewer to 182 more)	Very low
**All-cause death**	Sulfonylureas	Basal-Bolus insulin	1.39 (0.33 to 5.88)	56 per 1000 persons	76 per 1000 persons (19 to 259 per 1000 persons)	20 more (37 fewer to 203 more)	Very low
**All-cause death**	Thiazolidinediones	Basal-Bolus insulin	1.20 (0.28 to 5.00)	56 per 1000 persons	67 per 1000 persons (17 to 229 per 1000 persons)	11 more (39 fewer to 173 more)	Very low
**All-cause death**	Tirzepatide	Basal-Bolus insulin	1.05 (0.23 to 4.76)	56 per 1000 persons	59 per 1000 persons (13 to 220 per 1000 persons)	3 more (43 fewer to 164 more)	Very low
**All-cause death**	DPP-4 inhibitors	Bolus insulin	2.13 (0.64 to 7.14)	35 per 1000 persons	72 per 1000 persons (23 to 206 per 1000 persons)	37 more (12 fewer to 171 more)	Very low
**All-cause death**	GLP-1 receptor agonists	Bolus insulin	1.82 (0.55 to 5.88)	35 per 1000 persons	62 per 1000 persons (20 to 176 per 1000 persons)	27 more (15 fewer to 141 more)	Very low
**All-cause death**	Meglitinides	Bolus insulin	3.33 (0.63 to 16.67)	35 per 1000 persons	108 per 1000 persons (22 to 377 per 1000 persons)	73 more (13 fewer to 342 more)	Very low
**All-cause death**	Metformin	Bolus insulin	1.72 (0.52 to 5.88)	35 per 1000 persons	59 per 1000 persons (18 to 176 per 1000 persons)	24 more (17 fewer to 141 more)	Very low
**All-cause death**	Non-steroidal MRAs	Bolus insulin	1.85 (0.56 to 6.25)	35 per 1000 persons	63 per 1000 persons (20 to 185 per 1000 persons)	28 more (15 fewer to 150 more)	Very low
**All-cause death**	SGLT2 inhibitors	Bolus insulin	1.85 (0.55 to 6.25)	35 per 1000 persons	63 per 1000 persons (20 to 185 per 1000 persons)	28 more (15 fewer to 150 more)	Very low
**All-cause death**	Standard treatments	Bolus insulin	2.08 (0.63 to 6.67)	35 per 1000 persons	70 per 1000 persons (22 to 195 per 1000 persons)	35 more (13 fewer to 160 more)	Very low

Outcome	Intervention	Comparator	Odds ratio	Baseline risk	Risk if treated	Risk difference	Certainty
**All-cause death**	Sulfonylureas	Bolus insulin	2.27 (0.69 to 7.69)	35 per 1000 persons	76 per 1000 persons (24 to 218 per 1000 persons)	41 more (11 fewer to 183 more)	Very low
**All-cause death**	Thiazolidinediones	Bolus insulin	1.96 (0.60 to 6.67)	35 per 1000 persons	66 per 1000 persons (21 to 195 per 1000 persons)	31 more (14 fewer to 160 more)	Very low
**All-cause death**	Tirzepatide	Bolus insulin	1.72 (0.47 to 6.25)	35 per 1000 persons	59 per 1000 persons (17 to 185 per 1000 persons)	24 more (18 fewer to 150 more)	Very low
**All-cause death**	GLP-1 receptor agonists	DPP-4 inhibitors	0.86 (0.79 to 0.94)	71 per 1000 persons	62 per 1000 persons (57 to 67 per 1000 persons)	9 fewer (14 fewer to 4 fewer)	Low
**All-cause death**	Meglitinides	DPP-4 inhibitors	1.56 (0.50 to 4.76)	71 per 1000 persons	107 per 1000 persons (37 to 267 per 1000 persons)	36 more (34 fewer to 196 more)	Very low
**All-cause death**	Metformin	DPP-4 inhibitors	0.83 (0.66 to 1.03)	71 per 1000 persons	59 per 1000 persons (48 to 73 per 1000 persons)	12 fewer (23 fewer to 2 more)	Low
**All-cause death**	Non-steroidal MRAs	DPP-4 inhibitors	0.88 (0.76 to 1.01)	71 per 1000 persons	63 per 1000 persons (55 to 72 per 1000 persons)	8 fewer (16 fewer to 1 more)	Low
**All-cause death**	SGLT2 inhibitors	DPP-4 inhibitors	0.87 (0.79 to 0.96)	71 per 1000 persons	62 per 1000 persons (57 to 68 per 1000 persons)	9 fewer (14 fewer to 3 fewer)	Low
**All-cause death**	Standard treatments	DPP-4 inhibitors	0.99 (0.93 to 1.05)	71 per 1000 persons	70 per 1000 persons (66 to 74 per 1000 persons)	1 fewer (5 fewer to 3 more)	Moderate
**All-cause death**	Sulfonylureas	DPP-4 inhibitors	1.09 (0.96 to 1.23)	71 per 1000 persons	77 per 1000 persons (68 to 86 per 1000 persons)	6 more (3 fewer to 15 more)	Low
**All-cause death**	Thiazolidinediones	DPP-4 inhibitors	0.94 (0.81 to 1.09)	71 per 1000 persons	67 per 1000 persons (59 to 77 per 1000 persons)	4 fewer (12 fewer to 6 more)	Low
**All-cause death**	Tirzepatide	DPP-4 inhibitors	0.82 (0.47 to 1.43)	71 per 1000 persons	59 per 1000 persons (35 to 98 per 1000 persons)	12 fewer (36 fewer to 27 more)	Very low
**All-cause death**	Meglitinides	GLP-1 receptor agonists	1.82 (0.58 to 5.56)	62 per 1000 persons	107 per 1000 persons (37 to 269 per 1000 persons)	45 more (25 fewer to 207 more)	Very low
**All-cause death**	Metformin	GLP-1 receptor agonists	0.95 (0.76 to 1.19)	62 per 1000 persons	59 per 1000 persons (48 to 73 per 1000 persons)	3 fewer (14 fewer to 11 more)	Very low
**All-cause death**	Non-steroidal MRAs	GLP-1 receptor agonists	1.01 (0.88 to 1.16)	62 per 1000 persons	63 per 1000 persons (55 to 71 per 1000 persons)	1 more (7 fewer to 9 more)	Moderate

Outcome	Intervention	Comparator	Odds ratio	Baseline risk	Risk if treated	Risk difference	Certainty
**All-cause death**	SGLT2 inhibitors	GLP-1 receptor agonists	1.01 (0.93 to 1.10)	62 per 1000 persons	63 per 1000 persons (58 to 68 per 1000 persons)	1 more (4 fewer to 6 more)	Moderate
**All-cause death**	Standard treatments	GLP-1 receptor agonists	1.14 (1.08 to 1.22)	62 per 1000 persons	70 per 1000 persons (66 to 75 per 1000 persons)	8 more (4 more to 13 more)	Moderate
**All-cause death**	Sulfonylureas	GLP-1 receptor agonists	1.27 (1.09 to 1.45)	62 per 1000 persons	77 per 1000 persons (67 to 87 per 1000 persons)	15 more (5 more to 25 more)	Moderate
**All-cause death**	Thiazolidinediones	GLP-1 receptor agonists	1.09 (0.93 to 1.25)	62 per 1000 persons	67 per 1000 persons (58 to 76 per 1000 persons)	5 more (4 fewer to 14 more)	Low
**All-cause death**	Tirzepatide	GLP-1 receptor agonists	0.95 (0.55 to 1.64)	62 per 1000 persons	59 per 1000 persons (35 to 98 per 1000 persons)	3 fewer (27 fewer to 36 more)	Very low
**All-cause death**	Metformin	Meglitinides	0.53 (0.17 to 1.67)	106 per 1000 persons	59 per 1000 persons (19 to 165 per 1000 persons)	47 fewer (87 fewer to 59 more)	Very low
**All-cause death**	Non-steroidal MRAs	Meglitinides	0.56 (0.18 to 1.75)	106 per 1000 persons	62 per 1000 persons (21 to 172 per 1000 persons)	44 fewer (85 fewer to 66 more)	Very low
**All-cause death**	SGLT2 inhibitors	Meglitinides	0.56 (0.18 to 1.72)	106 per 1000 persons	62 per 1000 persons (21 to 170 per 1000 persons)	44 fewer (85 fewer to 64 more)	Very low
**All-cause death**	Standard treatments	Meglitinides	0.63 (0.20 to 1.96)	106 per 1000 persons	70 per 1000 persons (24 to 189 per 1000 persons)	36 fewer (82 fewer to 83 more)	Very low
**All-cause death**	Sulfonylureas	Meglitinides	0.70 (0.22 to 2.17)	106 per 1000 persons	77 per 1000 persons (26 to 205 per 1000 persons)	29 fewer (80 fewer to 99 more)	Very low
**All-cause death**	Thiazolidinediones	Meglitinides	0.60 (0.19 to 1.89)	106 per 1000 persons	67 per 1000 persons (22 to 183 per 1000 persons)	39 fewer (84 fewer to 77 more)	Very low
**All-cause death**	Tirzepatide	Meglitinides	0.53 (0.15 to 1.85)	106 per 1000 persons	59 per 1000 persons (17 to 180 per 1000 persons)	47 fewer (89 fewer to 74 more)	Very low
**All-cause death**	Non-steroidal MRAs	Metformin	1.06 (0.83 to 1.37)	59 per 1000 persons	63 per 1000 persons (50 to 79 per 1000 persons)	4 more (9 fewer to 20 more)	Low
**All-cause death**	SGLT2 inhibitors	Metformin	1.05 (0.84 to 1.32)	59 per 1000 persons	62 per 1000 persons (50 to 76 per 1000 persons)	3 more (9 fewer to 17 more)	Low
**All-cause death**	Standard treatments	Metformin	1.19 (0.96 to 1.49)	59 per 1000 persons	69 per 1000 persons (57 to 86 per 1000 persons)	10 more (2 fewer to 27 more)	Low

Outcome	Intervention	Comparator	Odds ratio	Baseline risk	Risk if treated	Risk difference	Certainty
**All-cause death**	Sulfonylureas	Metformin	1.32 (1.04 to 1.67)	59 per 1000 persons	76 per 1000 persons (61 to 95 per 1000 persons)	17 more (2 more to 36 more)	Moderate
**All-cause death**	Thiazolidinediones	Metformin	1.14 (0.89 to 1.45)	59 per 1000 persons	67 per 1000 persons (53 to 83 per 1000 persons)	8 more (6 fewer to 24 more)	Low
**All-cause death**	Tirzepatide	Metformin	1.00 (0.55 to 1.79)	59 per 1000 persons	59 per 1000 persons (33 to 101 per 1000 persons)	0 (26 fewer to 42 more)	Very low
**All-cause death**	SGLT2 inhibitors	Non-steroidal MRAs	0.99 (0.87 to 1.14)	63 per 1000 persons	62 per 1000 persons (55 to 71 per 1000 persons)	1 fewer (8 fewer to 8 more)	Moderate
**All-cause death**	Standard treatments	Non-steroidal MRAs	1.12 (1.00 to 1.27)	63 per 1000 persons	70 per 1000 persons (63 to 78 per 1000 persons)	7 more (0 to 15 more)	Low
**All-cause death**	Sulfonylureas	Non-steroidal MRAs	1.23 (1.04 to 1.49)	63 per 1000 persons	77 per 1000 persons (65 to 91 per 1000 persons)	14 more (2 more to 28 more)	Moderate
**All-cause death**	Thiazolidinediones	Non-steroidal MRAs	1.08 (0.89 to 1.28)	63 per 1000 persons	67 per 1000 persons (57 to 79 per 1000 persons)	4 more (6 fewer to 16 more)	Low
**All-cause death**	Tirzepatide	Non-steroidal MRAs	0.93 (0.53 to 1.64)	63 per 1000 persons	59 per 1000 persons (35 to 99 per 1000 persons)	4 fewer (28 fewer to 36 more)	Very low
**All-cause death**	Standard treatments	SGLT2 inhibitors	1.14 (1.06 to 1.20)	62 per 1000 persons	70 per 1000 persons (66 to 74 per 1000 persons)	8 more (4 more to 12 more)	Moderate
**All-cause death**	Sulfonylureas	SGLT2 inhibitors	1.25 (1.08 to 1.45)	62 per 1000 persons	76 per 1000 persons (66 to 87 per 1000 persons)	14 more (4 more to 25 more)	Moderate
**All-cause death**	Thiazolidinediones	SGLT2 inhibitors	1.08 (0.93 to 1.25)	62 per 1000 persons	66 per 1000 persons (58 to 76 per 1000 persons)	4 more (4 fewer to 14 more)	Low
**All-cause death**	Tirzepatide	SGLT2 inhibitors	0.94 (0.54 to 1.64)	62 per 1000 persons	59 per 1000 persons (35 to 98 per 1000 persons)	3 fewer (27 fewer to 36 more)	Very low
**All-cause death**	Standard treatments	Sulfonylureas	0.91 (0.79 to 1.03)	76 per 1000 persons	70 per 1000 persons (61 to 78 per 1000 persons)	6 fewer (15 fewer to 2 more)	Low
**All-cause death**	Thiazolidinediones	Sulfonylureas	0.86 (0.73 to 1.02)	76 per 1000 persons	66 per 1000 persons (57 to 77 per 1000 persons)	10 fewer (19 fewer to 1 more)	Low
**All-cause death**	Tirzepatide	Sulfonylureas	0.76 (0.43 to 1.32)	76 per 1000 persons	59 per 1000 persons (34 to 98 per 1000 persons)	17 fewer (42 fewer to 22 more)	Very low

Outcome	Intervention	Comparator	Odds ratio	Baseline risk	Risk if treated	Risk difference	Certainty
**All-cause death**	Standard treatments	Thiazolidinediones	1.05 (0.92 to 1.20)	67 per 1000 persons	70 per 1000 persons (62 to 80 per 1000 persons)	3 more (5 fewer to 13 more)	Low
**All-cause death**	Tirzepatide	Thiazolidinediones	0.88 (0.50 to 1.54)	67 per 1000 persons	59 per 1000 persons (34 to 99 per 1000 persons)	8 fewer (33 fewer to 32 more)	Very low
**All-cause death**	Standard treatments	Tirzepatide	1.20 (0.69 to 2.08)	59 per 1000 persons	70 per 1000 persons (42 to 116 per 1000 persons)	11 more (17 fewer to 57 more)	Very low
**Cardiovascular death**	Alpha-glucosidase inhibitors	Basal insulin	0.77 (0.15 to 3.88)	58 per 1000 persons	45 per 1000 persons (9 to 193 per 1000 persons)	13 fewer (49 fewer to 135 more)	Very low
**Cardiovascular death**	Alpha-glucosidase inhibitors	Basal-Bolus insulin	0.45 (0.03 to 7.04)	97 per 1000 persons	46 per 1000 persons (3 to 431 per 1000 persons)	51 fewer (94 fewer to 334 more)	Very low
**Cardiovascular death**	Alpha-glucosidase inhibitors	Bolus insulin	0.94 (0.06 to 14.87)	48 per 1000 persons	45 per 1000 persons (3 to 428 per 1000 persons)	3 fewer (45 fewer to 380 more)	Very low
**Cardiovascular death**	Alpha-glucosidase inhibitors	DPP-4 inhibitors	0.99 (0.21 to 4.71)	46 per 1000 persons	46 per 1000 persons (10 to 185 per 1000 persons)	0 (36 fewer to 139 more)	Very low
**Cardiovascular death**	Alpha-glucosidase inhibitors	GLP-1 receptor agonists	1.14 (0.24 to 5.40)	40 per 1000 persons	45 per 1000 persons (10 to 184 per 1000 persons)	5 more (30 fewer to 144 more)	Very low
**Cardiovascular death**	Alpha-glucosidase inhibitors	Meglitinides	1.55 (0.15 to 16.03)	30 per 1000 persons	46 per 1000 persons (5 to 331 per 1000 persons)	16 more (25 fewer to 301 more)	Very low
**Cardiovascular death**	Alpha-glucosidase inhibitors	Metformin	1.05 (0.19 to 5.69)	44 per 1000 persons	46 per 1000 persons (9 to 208 per 1000 persons)	2 more (35 fewer to 164 more)	Very low
**Cardiovascular death**	Alpha-glucosidase inhibitors	Non-steroidal MRAs	1.13 (0.24 to 5.40)	41 per 1000 persons	46 per 1000 persons (10 to 188 per 1000 persons)	5 more (31 fewer to 147 more)	Very low
**Cardiovascular death**	Alpha-glucosidase inhibitors	SGLT2 inhibitors	1.15 (0.24 to 5.44)	40 per 1000 persons	46 per 1000 persons (10 to 185 per 1000 persons)	6 more (30 fewer to 145 more)	Very low
**Cardiovascular death**	Alpha-glucosidase inhibitors	Standard treatments	0.99 (0.21 to 4.70)	46 per 1000 persons	46 per 1000 persons (10 to 185 per 1000 persons)	0 (36 fewer to 139 more)	Very low
**Cardiovascular death**	Alpha-glucosidase inhibitors	Sulfonylureas	0.98 (0.21 to 4.67)	46 per 1000 persons	45 per 1000 persons (10 to 184 per 1000 persons)	1 fewer (36 fewer to 138 more)	Very low
**Cardiovascular death**	Alpha-glucosidase inhibitors	Thiazolidinediones	1.07 (0.22 to 5.12)	43 per 1000 persons	46 per 1000 persons (10 to 187 per 1000 persons)	3 more (33 fewer to 144 more)	Very low

Outcome	Intervention	Comparator	Odds ratio	Baseline risk	Risk if treated	Risk difference	Certainty
**Cardiovascular death**	Alpha-glucosidase inhibitors	Tirzepatide	1.00 (0.15 to 6.50)	46 per 1000 persons	46 per 1000 persons (7 to 239 per 1000 persons)	0 (39 fewer to 193 more)	Very low
**Cardiovascular death**	Basal insulin	Basal-Bolus insulin	0.58 (0.06 to 5.88)	97 per 1000 persons	59 per 1000 persons (6 to 387 per 1000 persons)	38 fewer (91 fewer to 290 more)	Very low
**Cardiovascular death**	Basal insulin	Bolus insulin	1.22 (0.13 to 11.90)	48 per 1000 persons	58 per 1000 persons (7 to 375 per 1000 persons)	10 more (41 fewer to 327 more)	Very low
**Cardiovascular death**	Basal insulin	DPP-4 inhibitors	1.28 (0.83 to 1.99)	46 per 1000 persons	58 per 1000 persons (38 to 88 per 1000 persons)	12 more (8 fewer to 42 more)	Low
**Cardiovascular death**	Basal insulin	GLP-1 receptor agonists	1.47 (0.95 to 2.29)	40 per 1000 persons	58 per 1000 persons (38 to 87 per 1000 persons)	18 more (2 fewer to 47 more)	Low
**Cardiovascular death**	Basal insulin	Meglitinides	2.00 (0.33 to 12.12)	30 per 1000 persons	58 per 1000 persons (10 to 273 per 1000 persons)	28 more (20 fewer to 243 more)	Very low
**Cardiovascular death**	Basal insulin	Metformin	1.35 (0.61 to 3.03)	44 per 1000 persons	58 per 1000 persons (27 to 122 per 1000 persons)	14 more (17 fewer to 78 more)	Very low
**Cardiovascular death**	Basal insulin	Non-steroidal MRAs	1.46 (0.92 to 2.33)	41 per 1000 persons	59 per 1000 persons (38 to 91 per 1000 persons)	18 more (3 fewer to 50 more)	Low
**Cardiovascular death**	Basal insulin	SGLT2 inhibitors	1.48 (0.95 to 2.32)	40 per 1000 persons	58 per 1000 persons (38 to 88 per 1000 persons)	18 more (2 fewer to 48 more)	Low
**Cardiovascular death**	Basal insulin	Standard treatments	1.28 (0.83 to 1.99)	46 per 1000 persons	58 per 1000 persons (38 to 88 per 1000 persons)	12 more (8 fewer to 42 more)	Low
**Cardiovascular death**	Basal insulin	Sulfonylureas	1.27 (0.80 to 2.01)	46 per 1000 persons	58 per 1000 persons (37 to 88 per 1000 persons)	12 more (9 fewer to 42 more)	Low
**Cardiovascular death**	Basal insulin	Thiazolidinediones	1.38 (0.86 to 2.22)	43 per 1000 persons	58 per 1000 persons (37 to 91 per 1000 persons)	15 more (6 fewer to 48 more)	Low
**Cardiovascular death**	Basal insulin	Tirzepatide	1.29 (0.48 to 3.43)	46 per 1000 persons	59 per 1000 persons (23 to 142 per 1000 persons)	13 more (23 fewer to 96 more)	Low
**Cardiovascular death**	Basal-Bolus insulin	Bolus insulin	2.12 (0.08 to 53.34)	48 per 1000 persons	97 per 1000 persons (4 to 729 per 1000 persons)	49 more (44 fewer to 681 more)	Very low
**Cardiovascular death**	Basal-Bolus insulin	DPP-4 inhibitors	2.23 (0.23 to 21.94)	46 per 1000 persons	97 per 1000 persons (11 to 514 per 1000 persons)	51 more (35 fewer to 468 more)	Very low

Outcome	Intervention	Comparator	Odds ratio	Baseline risk	Risk if treated	Risk difference	Certainty
**Cardiovascular death**	Basal-Bolus insulin	GLP-1 receptor agonists	2.56 (0.26 to 25.15)	40 per 1000 persons	96 per 1000 persons (11 to 512 per 1000 persons)	56 more (29 fewer to 472 more)	Very low
**Cardiovascular death**	Basal-Bolus insulin	Meglitinides	3.48 (0.20 to 61.62)	30 per 1000 persons	97 per 1000 persons (6 to 656 per 1000 persons)	67 more (24 fewer to 626 more)	Very low
**Cardiovascular death**	Basal-Bolus insulin	Metformin	2.35 (0.22 to 25.43)	44 per 1000 persons	98 per 1000 persons (10 to 539 per 1000 persons)	54 more (34 fewer to 495 more)	Very low
**Cardiovascular death**	Basal-Bolus insulin	Non-steroidal MRAs	2.55 (0.26 to 25.14)	41 per 1000 persons	98 per 1000 persons (11 to 518 per 1000 persons)	57 more (30 fewer to 477 more)	Very low
**Cardiovascular death**	Basal-Bolus insulin	SGLT2 inhibitors	2.58 (0.26 to 25.38)	40 per 1000 persons	97 per 1000 persons (11 to 514 per 1000 persons)	57 more (29 fewer to 474 more)	Very low
**Cardiovascular death**	Basal-Bolus insulin	Standard treatments	2.23 (0.23 to 21.92)	46 per 1000 persons	97 per 1000 persons (11 to 514 per 1000 persons)	51 more (35 fewer to 468 more)	Very low
**Cardiovascular death**	Basal-Bolus insulin	Sulfonylureas	2.21 (0.22 to 21.71)	46 per 1000 persons	96 per 1000 persons (10 to 511 per 1000 persons)	50 more (36 fewer to 465 more)	Very low
**Cardiovascular death**	Basal-Bolus insulin	Thiazolidinediones	2.40 (0.24 to 23.79)	43 per 1000 persons	97 per 1000 persons (11 to 517 per 1000 persons)	54 more (32 fewer to 474 more)	Very low
**Cardiovascular death**	Basal-Bolus insulin	Tirzepatide	2.24 (0.18 to 27.63)	46 per 1000 persons	97 per 1000 persons (9 to 571 per 1000 persons)	51 more (37 fewer to 525 more)	Very low
**Cardiovascular death**	Bolus insulin	DPP-4 inhibitors	1.05 (0.11 to 10.27)	46 per 1000 persons	48 per 1000 persons (5 to 331 per 1000 persons)	2 more (41 fewer to 285 more)	Very low
**Cardiovascular death**	Bolus insulin	GLP-1 receptor agonists	1.21 (0.12 to 11.76)	40 per 1000 persons	48 per 1000 persons (5 to 329 per 1000 persons)	8 more (35 fewer to 289 more)	Very low
**Cardiovascular death**	Bolus insulin	Meglitinides	1.64 (0.09 to 28.95)	30 per 1000 persons	48 per 1000 persons (3 to 472 per 1000 persons)	18 more (27 fewer to 442 more)	Very low
**Cardiovascular death**	Bolus insulin	Metformin	1.11 (0.10 to 11.94)	44 per 1000 persons	49 per 1000 persons (5 to 355 per 1000 persons)	5 more (39 fewer to 311 more)	Very low
**Cardiovascular death**	Bolus insulin	Non-steroidal MRAs	1.20 (0.12 to 11.76)	41 per 1000 persons	49 per 1000 persons (5 to 335 per 1000 persons)	8 more (36 fewer to 294 more)	Very low
**Cardiovascular death**	Bolus insulin	SGLT2 inhibitors	1.22 (0.12 to 11.88)	40 per 1000 persons	48 per 1000 persons (5 to 331 per 1000 persons)	8 more (35 fewer to 291 more)	Very low

Outcome	Intervention	Comparator	Odds ratio	Baseline risk	Risk if treated	Risk difference	Certainty
**Cardiovascular death**	Bolus insulin	Standard treatments	1.05 (0.11 to 10.26)	46 per 1000 persons	48 per 1000 persons (5 to 331 per 1000 persons)	2 more (41 fewer to 285 more)	Very low
**Cardiovascular death**	Bolus insulin	Sulfonylureas	1.04 (0.11 to 10.22)	46 per 1000 persons	48 per 1000 persons (5 to 330 per 1000 persons)	2 more (41 fewer to 284 more)	Very low
**Cardiovascular death**	Bolus insulin	Thiazolidinediones	1.13 (0.12 to 11.14)	43 per 1000 persons	48 per 1000 persons (5 to 334 per 1000 persons)	5 more (38 fewer to 291 more)	Very low
**Cardiovascular death**	Bolus insulin	Tirzepatide	1.06 (0.09 to 12.53)	46 per 1000 persons	49 per 1000 persons (4 to 377 per 1000 persons)	3 more (42 fewer to 331 more)	Very low
**Cardiovascular death**	DPP-4 inhibitors	GLP-1 receptor agonists	1.15 (1.02 to 1.28)	40 per 1000 persons	46 per 1000 persons (41 to 51 per 1000 persons)	6 more (1 more to 11 more)	Low
**Cardiovascular death**	DPP-4 inhibitors	Meglitinides	1.56 (0.27 to 8.97)	30 per 1000 persons	46 per 1000 persons (8 to 217 per 1000 persons)	16 more (22 fewer to 187 more)	Very low
**Cardiovascular death**	DPP-4 inhibitors	Metformin	1.05 (0.53 to 2.09)	44 per 1000 persons	46 per 1000 persons (24 to 88 per 1000 persons)	2 more (20 fewer to 44 more)	Very low
**Cardiovascular death**	DPP-4 inhibitors	Non-steroidal MRAs	1.14 (0.96 to 1.36)	41 per 1000 persons	46 per 1000 persons (39 to 55 per 1000 persons)	5 more (2 fewer to 14 more)	Low
**Cardiovascular death**	DPP-4 inhibitors	SGLT2 inhibitors	1.16 (1.03 to 1.30)	40 per 1000 persons	46 per 1000 persons (41 to 51 per 1000 persons)	6 more (1 more to 11 more)	Low
**Cardiovascular death**	DPP-4 inhibitors	Standard treatments	1.00 (0.92 to 1.09)	46 per 1000 persons	46 per 1000 persons (42 to 50 per 1000 persons)	0 (4 fewer to 4 more)	Moderate
**Cardiovascular death**	DPP-4 inhibitors	Sulfonylureas	0.99 (0.82 to 1.19)	46 per 1000 persons	46 per 1000 persons (38 to 54 per 1000 persons)	0 (8 fewer to 8 more)	High
**Cardiovascular death**	DPP-4 inhibitors	Thiazolidinediones	1.08 (0.88 to 1.32)	43 per 1000 persons	46 per 1000 persons (38 to 56 per 1000 persons)	3 more (5 fewer to 13 more)	Low
**Cardiovascular death**	DPP-4 inhibitors	Tirzepatide	1.00 (0.35 to 2.87)	46 per 1000 persons	46 per 1000 persons (17 to 122 per 1000 persons)	0 (29 fewer to 76 more)	Very low
**Cardiovascular death**	GLP-1 receptor agonists	Meglitinides	1.36 (0.24 to 7.83)	30 per 1000 persons	40 per 1000 persons (7 to 195 per 1000 persons)	10 more (23 fewer to 165 more)	Very low
**Cardiovascular death**	GLP-1 receptor agonists	Metformin	0.92 (0.46 to 1.83)	44 per 1000 persons	41 per 1000 persons (21 to 78 per 1000 persons)	3 fewer (23 fewer to 34 more)	Very low

Outcome	Intervention	Comparator	Odds ratio	Baseline risk	Risk if treated	Risk difference	Certainty
**Cardiovascular death**	GLP-1 receptor agonists	Non-steroidal MRAs	0.99 (0.84 to 1.18)	41 per 1000 persons	41 per 1000 persons (35 to 48 per 1000 persons)	0 (6 fewer to 7 more)	Moderate
**Cardiovascular death**	GLP-1 receptor agonists	SGLT2 inhibitors	1.01 (0.90 to 1.13)	40 per 1000 persons	40 per 1000 persons (36 to 45 per 1000 persons)	0 (4 fewer to 5 more)	Moderate
**Cardiovascular death**	GLP-1 receptor agonists	Standard treatments	0.87 (0.81 to 0.94)	46 per 1000 persons	40 per 1000 persons (38 to 43 per 1000 persons)	6 fewer (8 fewer to 3 fewer)	High
**Cardiovascular death**	GLP-1 receptor agonists	Sulfonylureas	0.86 (0.70 to 1.07)	46 per 1000 persons	40 per 1000 persons (33 to 49 per 1000 persons)	6 fewer (13 fewer to 3 more)	Low
**Cardiovascular death**	GLP-1 receptor agonists	Thiazolidinediones	0.94 (0.77 to 1.15)	43 per 1000 persons	41 per 1000 persons (33 to 49 per 1000 persons)	2 fewer (10 fewer to 6 more)	Moderate
**Cardiovascular death**	GLP-1 receptor agonists	Tirzepatide	0.88 (0.31 to 2.50)	46 per 1000 persons	41 per 1000 persons (15 to 108 per 1000 persons)	5 fewer (31 fewer to 62 more)	Very low
**Cardiovascular death**	Meglitinides	Metformin	0.68 (0.11 to 4.15)	44 per 1000 persons	30 per 1000 persons (5 to 160 per 1000 persons)	14 fewer (39 fewer to 116 more)	Very low
**Cardiovascular death**	Meglitinides	Non-steroidal MRAs	0.73 (0.13 to 4.24)	41 per 1000 persons	30 per 1000 persons (6 to 153 per 1000 persons)	11 fewer (35 fewer to 112 more)	Very low
**Cardiovascular death**	Meglitinides	SGLT2 inhibitors	0.74 (0.13 to 4.28)	40 per 1000 persons	30 per 1000 persons (5 to 151 per 1000 persons)	10 fewer (35 fewer to 111 more)	Very low
**Cardiovascular death**	Meglitinides	Standard treatments	0.64 (0.11 to 3.69)	46 per 1000 persons	30 per 1000 persons (5 to 151 per 1000 persons)	16 fewer (41 fewer to 105 more)	Very low
**Cardiovascular death**	Meglitinides	Sulfonylureas	0.64 (0.11 to 3.65)	46 per 1000 persons	30 per 1000 persons (5 to 150 per 1000 persons)	16 fewer (41 fewer to 104 more)	Very low
**Cardiovascular death**	Meglitinides	Thiazolidinediones	0.69 (0.12 to 3.99)	43 per 1000 persons	30 per 1000 persons (5 to 152 per 1000 persons)	13 fewer (38 fewer to 109 more)	Very low
**Cardiovascular death**	Meglitinides	Tirzepatide	0.64 (0.08 to 4.95)	46 per 1000 persons	30 per 1000 persons (4 to 193 per 1000 persons)	16 fewer (42 fewer to 147 more)	Very low
**Cardiovascular death**	Metformin	Non-steroidal MRAs	1.08 (0.54 to 2.18)	41 per 1000 persons	44 per 1000 persons (23 to 85 per 1000 persons)	3 more (18 fewer to 44 more)	Very low
**Cardiovascular death**	Metformin	SGLT2 inhibitors	1.10 (0.55 to 2.18)	40 per 1000 persons	44 per 1000 persons (22 to 83 per 1000 persons)	4 more (18 fewer to 43 more)	Very low

Outcome	Intervention	Comparator	Odds ratio	Baseline risk	Risk if treated	Risk difference	Certainty
**Cardiovascular death**	Metformin	Standard treatments	0.95 (0.48 to 1.88)	46 per 1000 persons	44 per 1000 persons (23 to 83 per 1000 persons)	2 fewer (23 fewer to 37 more)	Very low
**Cardiovascular death**	Metformin	Sulfonylureas	0.94 (0.48 to 1.85)	46 per 1000 persons	43 per 1000 persons (23 to 82 per 1000 persons)	3 fewer (23 fewer to 36 more)	Very low
**Cardiovascular death**	Metformin	Thiazolidinediones	1.02 (0.51 to 2.05)	43 per 1000 persons	44 per 1000 persons (22 to 84 per 1000 persons)	1 more (21 fewer to 41 more)	Very low
**Cardiovascular death**	Metformin	Tirzepatide	0.95 (0.27 to 3.32)	46 per 1000 persons	44 per 1000 persons (13 to 138 per 1000 persons)	2 fewer (33 fewer to 92 more)	Very low
**Cardiovascular death**	Non-steroidal MRAs	SGLT2 inhibitors	1.01 (0.85 to 1.21)	40 per 1000 persons	40 per 1000 persons (34 to 48 per 1000 persons)	0 (6 fewer to 8 more)	Moderate
**Cardiovascular death**	Non-steroidal MRAs	Standard treatments	0.88 (0.75 to 1.02)	46 per 1000 persons	41 per 1000 persons (35 to 47 per 1000 persons)	5 fewer (11 fewer to 1 more)	Low
**Cardiovascular death**	Non-steroidal MRAs	Sulfonylureas	0.87 (0.67 to 1.12)	46 per 1000 persons	40 per 1000 persons (31 to 51 per 1000 persons)	6 fewer (15 fewer to 5 more)	Low
**Cardiovascular death**	Non-steroidal MRAs	Thiazolidinediones	0.94 (0.74 to 1.20)	43 per 1000 persons	41 per 1000 persons (32 to 51 per 1000 persons)	2 fewer (11 fewer to 8 more)	Low
**Cardiovascular death**	Non-steroidal MRAs	Tirzepatide	0.88 (0.30 to 2.55)	46 per 1000 persons	41 per 1000 persons (14 to 109 per 1000 persons)	5 fewer (32 fewer to 63 more)	Very low
**Cardiovascular death**	SGLT2 inhibitors	Standard treatments	0.86 (0.80 to 0.94)	46 per 1000 persons	40 per 1000 persons (37 to 43 per 1000 persons)	6 fewer (9 fewer to 3 fewer)	High
**Cardiovascular death**	SGLT2 inhibitors	Sulfonylureas	0.86 (0.69 to 1.06)	46 per 1000 persons	40 per 1000 persons (32 to 49 per 1000 persons)	6 fewer (14 fewer to 3 more)	Low
**Cardiovascular death**	SGLT2 inhibitors	Thiazolidinediones	0.93 (0.76 to 1.14)	43 per 1000 persons	40 per 1000 persons (33 to 49 per 1000 persons)	3 fewer (10 fewer to 6 more)	Moderate
**Cardiovascular death**	SGLT2 inhibitors	Tirzepatide	0.87 (0.30 to 2.49)	46 per 1000 persons	40 per 1000 persons (14 to 107 per 1000 persons)	6 fewer (32 fewer to 61 more)	Very low
**Cardiovascular death**	Sulfonylureas	Standard treatments	1.01 (0.83 to 1.23)	46 per 1000 persons	46 per 1000 persons (38 to 56 per 1000 persons)	0 (8 fewer to 10 more)	Moderate
**Cardiovascular death**	Sulfonylureas	Thiazolidinediones	1.09 (0.84 to 1.41)	43 per 1000 persons	47 per 1000 persons (36 to 60 per 1000 persons)	4 more (7 fewer to 17 more)	Low

Outcome	Intervention	Comparator	Odds ratio	Baseline risk	Risk if treated	Risk difference	Certainty
**Cardiovascular death**	Sulfonylureas	Tirzepatide	1.01 (0.35 to 2.93)	46 per 1000 persons	46 per 1000 persons (17 to 124 per 1000 persons)	0 (29 fewer to 78 more)	Very low
**Cardiovascular death**	Thiazolidinediones	Standard treatments	0.93 (0.77 to 1.12)	46 per 1000 persons	43 per 1000 persons (36 to 51 per 1000 persons)	3 fewer (10 fewer to 5 more)	Moderate
**Cardiovascular death**	Thiazolidinediones	Tirzepatide	0.93 (0.32 to 2.71)	46 per 1000 persons	43 per 1000 persons (15 to 116 per 1000 persons)	3 fewer (31 fewer to 70 more)	Very low
**Cardiovascular death**	Tirzepatide	Standard treatments	1.00 (0.35 to 2.85)	46 per 1000 persons	46 per 1000 persons (17 to 121 per 1000 persons)	0 (29 fewer to 75 more)	Very low
**Cardiovascular death**	Basal insulin	Alpha-glucosidase inhibitors	1.30 (0.26 to 6.67)	46 per 1000 persons	59 per 1000 persons (12 to 243 per 1000 persons)	13 more (34 fewer to 197 more)	Very low
**Cardiovascular death**	Basal-Bolus insulin	Alpha-glucosidase inhibitors	2.22 (0.14 to 33.33)	46 per 1000 persons	97 per 1000 persons (7 to 616 per 1000 persons)	51 more (39 fewer to 570 more)	Very low
**Cardiovascular death**	Bolus insulin	Alpha-glucosidase inhibitors	1.06 (0.07 to 16.67)	46 per 1000 persons	49 per 1000 persons (3 to 446 per 1000 persons)	3 more (43 fewer to 400 more)	Very low
**Cardiovascular death**	DPP-4 inhibitors	Alpha-glucosidase inhibitors	1.01 (0.21 to 4.76)	46 per 1000 persons	46 per 1000 persons (10 to 187 per 1000 persons)	0 (36 fewer to 141 more)	Very low
**Cardiovascular death**	GLP-1 receptor agonists	Alpha-glucosidase inhibitors	0.88 (0.19 to 4.17)	46 per 1000 persons	41 per 1000 persons (9 to 167 per 1000 persons)	5 fewer (37 fewer to 121 more)	Very low
**Cardiovascular death**	Meglitinides	Alpha-glucosidase inhibitors	0.65 (0.06 to 6.67)	46 per 1000 persons	30 per 1000 persons (3 to 243 per 1000 persons)	16 fewer (43 fewer to 197 more)	Very low
**Cardiovascular death**	Metformin	Alpha-glucosidase inhibitors	0.95 (0.18 to 5.26)	46 per 1000 persons	44 per 1000 persons (8 to 202 per 1000 persons)	2 fewer (38 fewer to 156 more)	Very low
**Cardiovascular death**	Non-steroidal MRAs	Alpha-glucosidase inhibitors	0.88 (0.19 to 4.17)	46 per 1000 persons	41 per 1000 persons (9 to 167 per 1000 persons)	5 fewer (37 fewer to 121 more)	Very low
**Cardiovascular death**	SGLT2 inhibitors	Alpha-glucosidase inhibitors	0.87 (0.18 to 4.17)	46 per 1000 persons	40 per 1000 persons (9 to 167 per 1000 persons)	6 fewer (37 fewer to 121 more)	Very low
**Cardiovascular death**	Standard treatments	Alpha-glucosidase inhibitors	1.01 (0.21 to 4.76)	46 per 1000 persons	46 per 1000 persons (10 to 187 per 1000 persons)	0 (36 fewer to 141 more)	Very low
**Cardiovascular death**	Sulfonylureas	Alpha-glucosidase inhibitors	1.02 (0.21 to 4.76)	46 per 1000 persons	47 per 1000 persons (10 to 187 per 1000 persons)	1 more (36 fewer to 141 more)	Very low

Outcome	Intervention	Comparator	Odds ratio	Baseline risk	Risk if treated	Risk difference	Certainty
**Cardiovascular death**	Thiazolidinediones	Alpha-glucosidase inhibitors	0.93 (0.20 to 4.55)	46 per 1000 persons	43 per 1000 persons (9 to 180 per 1000 persons)	3 fewer (37 fewer to 134 more)	Very low
**Cardiovascular death**	Tirzepatide	Alpha-glucosidase inhibitors	1.00 (0.15 to 6.67)	46 per 1000 persons	46 per 1000 persons (7 to 243 per 1000 persons)	0 (39 fewer to 197 more)	Very low
**Cardiovascular death**	Basal-Bolus insulin	Basal insulin	1.72 (0.17 to 16.67)	58 per 1000 persons	96 per 1000 persons (10 to 506 per 1000 persons)	38 more (48 fewer to 448 more)	Very low
**Cardiovascular death**	Bolus insulin	Basal insulin	0.82 (0.08 to 7.69)	58 per 1000 persons	48 per 1000 persons (5 to 321 per 1000 persons)	10 fewer (53 fewer to 263 more)	Very low
**Cardiovascular death**	DPP-4 inhibitors	Basal insulin	0.78 (0.50 to 1.20)	58 per 1000 persons	46 per 1000 persons (30 to 69 per 1000 persons)	12 fewer (28 fewer to 11 more)	Very low
**Cardiovascular death**	GLP-1 receptor agonists	Basal insulin	0.68 (0.44 to 1.05)	58 per 1000 persons	40 per 1000 persons (26 to 61 per 1000 persons)	18 fewer (32 fewer to 3 more)	Low
**Cardiovascular death**	Meglitinides	Basal insulin	0.50 (0.08 to 3.03)	58 per 1000 persons	30 per 1000 persons (5 to 157 per 1000 persons)	28 fewer (53 fewer to 99 more)	Very low
**Cardiovascular death**	Metformin	Basal insulin	0.74 (0.33 to 1.64)	58 per 1000 persons	44 per 1000 persons (20 to 92 per 1000 persons)	14 fewer (38 fewer to 34 more)	Very low
**Cardiovascular death**	Non-steroidal MRAs	Basal insulin	0.68 (0.43 to 1.09)	58 per 1000 persons	40 per 1000 persons (26 to 63 per 1000 persons)	18 fewer (32 fewer to 5 more)	Low
**Cardiovascular death**	SGLT2 inhibitors	Basal insulin	0.68 (0.43 to 1.05)	58 per 1000 persons	40 per 1000 persons (26 to 61 per 1000 persons)	18 fewer (32 fewer to 3 more)	Low
**Cardiovascular death**	Standard treatments	Basal insulin	0.78 (0.50 to 1.20)	58 per 1000 persons	46 per 1000 persons (30 to 69 per 1000 persons)	12 fewer (28 fewer to 11 more)	Very low
**Cardiovascular death**	Sulfonylureas	Basal insulin	0.79 (0.50 to 1.25)	58 per 1000 persons	46 per 1000 persons (30 to 71 per 1000 persons)	12 fewer (28 fewer to 13 more)	Very low
**Cardiovascular death**	Thiazolidinediones	Basal insulin	0.72 (0.45 to 1.16)	58 per 1000 persons	43 per 1000 persons (27 to 67 per 1000 persons)	15 fewer (31 fewer to 9 more)	Low
**Cardiovascular death**	Tirzepatide	Basal insulin	0.78 (0.29 to 2.08)	58 per 1000 persons	46 per 1000 persons (18 to 114 per 1000 persons)	12 fewer (40 fewer to 56 more)	Low
**Cardiovascular death**	Bolus insulin	Basal-Bolus insulin	0.47 (0.02 to 12.50)	97 per 1000 persons	48 per 1000 persons (2 to 573 per 1000 persons)	49 fewer (95 fewer to 476 more)	Very low

Outcome	Intervention	Comparator	Odds ratio	Baseline risk	Risk if treated	Risk difference	Certainty
**Cardiovascular death**	DPP-4 inhibitors	Basal-Bolus insulin	0.45 (0.05 to 4.35)	97 per 1000 persons	46 per 1000 persons (5 to 318 per 1000 persons)	51 fewer (92 fewer to 221 more)	Very low
**Cardiovascular death**	GLP-1 receptor agonists	Basal-Bolus insulin	0.39 (0.04 to 3.85)	97 per 1000 persons	40 per 1000 persons (4 to 292 per 1000 persons)	57 fewer (93 fewer to 195 more)	Very low
**Cardiovascular death**	Meglitinides	Basal-Bolus insulin	0.29 (0.02 to 5.00)	97 per 1000 persons	30 per 1000 persons (2 to 349 per 1000 persons)	67 fewer (95 fewer to 252 more)	Very low
**Cardiovascular death**	Metformin	Basal-Bolus insulin	0.43 (0.04 to 4.55)	97 per 1000 persons	44 per 1000 persons (4 to 328 per 1000 persons)	53 fewer (93 fewer to 231 more)	Very low
**Cardiovascular death**	Non-steroidal MRAs	Basal-Bolus insulin	0.39 (0.04 to 3.85)	97 per 1000 persons	40 per 1000 persons (4 to 292 per 1000 persons)	57 fewer (93 fewer to 195 more)	Very low
**Cardiovascular death**	SGLT2 inhibitors	Basal-Bolus insulin	0.39 (0.04 to 3.85)	97 per 1000 persons	40 per 1000 persons (4 to 292 per 1000 persons)	57 fewer (93 fewer to 195 more)	Very low
**Cardiovascular death**	Standard treatments	Basal-Bolus insulin	0.45 (0.05 to 4.35)	97 per 1000 persons	46 per 1000 persons (5 to 318 per 1000 persons)	51 fewer (92 fewer to 221 more)	Very low
**Cardiovascular death**	Sulfonylureas	Basal-Bolus insulin	0.45 (0.05 to 4.55)	97 per 1000 persons	46 per 1000 persons (5 to 328 per 1000 persons)	51 fewer (92 fewer to 231 more)	Very low
**Cardiovascular death**	Thiazolidinediones	Basal-Bolus insulin	0.42 (0.04 to 4.17)	97 per 1000 persons	43 per 1000 persons (4 to 309 per 1000 persons)	54 fewer (93 fewer to 212 more)	Very low
**Cardiovascular death**	Tirzepatide	Basal-Bolus insulin	0.45 (0.04 to 5.56)	97 per 1000 persons	46 per 1000 persons (4 to 374 per 1000 persons)	51 fewer (93 fewer to 277 more)	Very low
**Cardiovascular death**	DPP-4 inhibitors	Bolus insulin	0.95 (0.10 to 9.09)	48 per 1000 persons	46 per 1000 persons (5 to 314 per 1000 persons)	2 fewer (43 fewer to 266 more)	Very low
**Cardiovascular death**	GLP-1 receptor agonists	Bolus insulin	0.83 (0.09 to 8.33)	48 per 1000 persons	40 per 1000 persons (4 to 296 per 1000 persons)	8 fewer (44 fewer to 248 more)	Very low
**Cardiovascular death**	Meglitinides	Bolus insulin	0.61 (0.03 to 11.11)	48 per 1000 persons	30 per 1000 persons (2 to 359 per 1000 persons)	18 fewer (46 fewer to 311 more)	Very low
**Cardiovascular death**	Metformin	Bolus insulin	0.90 (0.08 to 10.00)	48 per 1000 persons	43 per 1000 persons (4 to 335 per 1000 persons)	5 fewer (44 fewer to 287 more)	Very low
**Cardiovascular death**	Non-steroidal MRAs	Bolus insulin	0.83 (0.09 to 8.33)	48 per 1000 persons	40 per 1000 persons (4 to 296 per 1000 persons)	8 fewer (44 fewer to 248 more)	Very low

Outcome	Intervention	Comparator	Odds ratio	Baseline risk	Risk if treated	Risk difference	Certainty
**Cardiovascular death**	SGLT2 inhibitors	Bolus insulin	0.82 (0.08 to 8.33)	48 per 1000 persons	40 per 1000 persons (4 to 296 per 1000 persons)	8 fewer (44 fewer to 248 more)	Very low
**Cardiovascular death**	Standard treatments	Bolus insulin	0.95 (0.10 to 9.09)	48 per 1000 persons	46 per 1000 persons (5 to 314 per 1000 persons)	2 fewer (43 fewer to 266 more)	Very low
**Cardiovascular death**	Sulfonylureas	Bolus insulin	0.96 (0.10 to 9.09)	48 per 1000 persons	46 per 1000 persons (5 to 314 per 1000 persons)	2 fewer (43 fewer to 266 more)	Very low
**Cardiovascular death**	Thiazolidinediones	Bolus insulin	0.88 (0.09 to 8.33)	48 per 1000 persons	43 per 1000 persons (5 to 296 per 1000 persons)	5 fewer (43 fewer to 248 more)	Very low
**Cardiovascular death**	Tirzepatide	Bolus insulin	0.94 (0.08 to 11.11)	48 per 1000 persons	45 per 1000 persons (4 to 359 per 1000 persons)	3 fewer (44 fewer to 311 more)	Very low
**Cardiovascular death**	GLP-1 receptor agonists	DPP-4 inhibitors	0.87 (0.78 to 0.98)	46 per 1000 persons	40 per 1000 persons (36 to 45 per 1000 persons)	6 fewer (10 fewer to 1 fewer)	Moderate
**Cardiovascular death**	Meglitinides	DPP-4 inhibitors	0.64 (0.11 to 3.70)	46 per 1000 persons	30 per 1000 persons (5 to 152 per 1000 persons)	16 fewer (41 fewer to 106 more)	Very low
**Cardiovascular death**	Metformin	DPP-4 inhibitors	0.95 (0.48 to 1.89)	46 per 1000 persons	44 per 1000 persons (23 to 83 per 1000 persons)	2 fewer (23 fewer to 37 more)	Very low
**Cardiovascular death**	Non-steroidal MRAs	DPP-4 inhibitors	0.88 (0.74 to 1.04)	46 per 1000 persons	41 per 1000 persons (34 to 48 per 1000 persons)	5 fewer (12 fewer to 2 more)	Low
**Cardiovascular death**	SGLT2 inhibitors	DPP-4 inhibitors	0.86 (0.77 to 0.97)	46 per 1000 persons	40 per 1000 persons (36 to 45 per 1000 persons)	6 fewer (10 fewer to 1 fewer)	Moderate
**Cardiovascular death**	Standard treatments	DPP-4 inhibitors	1.00 (0.92 to 1.09)	46 per 1000 persons	46 per 1000 persons (42 to 50 per 1000 persons)	0 (4 fewer to 4 more)	Moderate
**Cardiovascular death**	Sulfonylureas	DPP-4 inhibitors	1.01 (0.84 to 1.22)	46 per 1000 persons	46 per 1000 persons (39 to 56 per 1000 persons)	0 (7 fewer to 10 more)	High
**Cardiovascular death**	Thiazolidinediones	DPP-4 inhibitors	0.93 (0.76 to 1.14)	46 per 1000 persons	43 per 1000 persons (35 to 52 per 1000 persons)	3 fewer (11 fewer to 6 more)	Low
**Cardiovascular death**	Tirzepatide	DPP-4 inhibitors	1.00 (0.35 to 2.86)	46 per 1000 persons	46 per 1000 persons (17 to 121 per 1000 persons)	0 (29 fewer to 75 more)	Very low
**Cardiovascular death**	Meglitinides	GLP-1 receptor agonists	0.74 (0.13 to 4.17)	40 per 1000 persons	30 per 1000 persons (5 to 148 per 1000 persons)	10 fewer (35 fewer to 108 more)	Very low

Outcome	Intervention	Comparator	Odds ratio	Baseline risk	Risk if treated	Risk difference	Certainty
**Cardiovascular death**	Metformin	GLP-1 receptor agonists	1.09 (0.55 to 2.17)	40 per 1000 persons	43 per 1000 persons (22 to 83 per 1000 persons)	3 more (18 fewer to 43 more)	Very low
**Cardiovascular death**	Non-steroidal MRAs	GLP-1 receptor agonists	1.01 (0.85 to 1.19)	40 per 1000 persons	40 per 1000 persons (34 to 47 per 1000 persons)	0 (6 fewer to 7 more)	Moderate
**Cardiovascular death**	SGLT2 inhibitors	GLP-1 receptor agonists	0.99 (0.88 to 1.11)	40 per 1000 persons	40 per 1000 persons (36 to 44 per 1000 persons)	0 (4 fewer to 4 more)	Moderate
**Cardiovascular death**	Standard treatments	GLP-1 receptor agonists	1.15 (1.06 to 1.23)	40 per 1000 persons	46 per 1000 persons (42 to 49 per 1000 persons)	6 more (2 more to 9 more)	High
**Cardiovascular death**	Sulfonylureas	GLP-1 receptor agonists	1.16 (0.93 to 1.43)	40 per 1000 persons	46 per 1000 persons (37 to 56 per 1000 persons)	6 more (3 fewer to 16 more)	Low
**Cardiovascular death**	Thiazolidinediones	GLP-1 receptor agonists	1.06 (0.87 to 1.30)	40 per 1000 persons	42 per 1000 persons (35 to 51 per 1000 persons)	2 more (5 fewer to 11 more)	Low
**Cardiovascular death**	Tirzepatide	GLP-1 receptor agonists	1.14 (0.40 to 3.23)	40 per 1000 persons	45 per 1000 persons (16 to 118 per 1000 persons)	5 more (24 fewer to 78 more)	Very low
**Cardiovascular death**	Metformin	Meglitinides	1.47 (0.24 to 9.09)	30 per 1000 persons	44 per 1000 persons (7 to 219 per 1000 persons)	14 more (23 fewer to 189 more)	Very low
**Cardiovascular death**	Non-steroidal MRAs	Meglitinides	1.37 (0.24 to 7.69)	30 per 1000 persons	41 per 1000 persons (7 to 192 per 1000 persons)	11 more (23 fewer to 162 more)	Very low
**Cardiovascular death**	SGLT2 inhibitors	Meglitinides	1.35 (0.23 to 7.69)	30 per 1000 persons	40 per 1000 persons (7 to 192 per 1000 persons)	10 more (23 fewer to 162 more)	Very low
**Cardiovascular death**	Standard treatments	Meglitinides	1.56 (0.27 to 9.09)	30 per 1000 persons	46 per 1000 persons (8 to 219 per 1000 persons)	16 more (22 fewer to 189 more)	Very low
**Cardiovascular death**	Sulfonylureas	Meglitinides	1.56 (0.27 to 9.09)	30 per 1000 persons	46 per 1000 persons (8 to 219 per 1000 persons)	16 more (22 fewer to 189 more)	Very low
**Cardiovascular death**	Thiazolidinediones	Meglitinides	1.45 (0.25 to 8.33)	30 per 1000 persons	43 per 1000 persons (8 to 205 per 1000 persons)	13 more (22 fewer to 175 more)	Very low
**Cardiovascular death**	Tirzepatide	Meglitinides	1.56 (0.20 to 12.50)	30 per 1000 persons	46 per 1000 persons (6 to 279 per 1000 persons)	16 more (24 fewer to 249 more)	Very low
**Cardiovascular death**	Non-steroidal MRAs	Metformin	0.93 (0.46 to 1.85)	44 per 1000 persons	41 per 1000 persons (21 to 79 per 1000 persons)	3 fewer (23 fewer to 35 more)	Very low

Outcome	Intervention	Comparator	Odds ratio	Baseline risk	Risk if treated	Risk difference	Certainty
**Cardiovascular death**	SGLT2 inhibitors	Metformin	0.91 (0.46 to 1.82)	44 per 1000 persons	40 per 1000 persons (21 to 77 per 1000 persons)	4 fewer (23 fewer to 33 more)	Very low
**Cardiovascular death**	Standard treatments	Metformin	1.05 (0.53 to 2.08)	44 per 1000 persons	46 per 1000 persons (24 to 87 per 1000 persons)	2 more (20 fewer to 43 more)	Very low
**Cardiovascular death**	Sulfonylureas	Metformin	1.06 (0.54 to 2.08)	44 per 1000 persons	47 per 1000 persons (24 to 87 per 1000 persons)	3 more (20 fewer to 43 more)	Very low
**Cardiovascular death**	Thiazolidinediones	Metformin	0.98 (0.49 to 1.96)	44 per 1000 persons	43 per 1000 persons (22 to 83 per 1000 persons)	1 fewer (22 fewer to 39 more)	Very low
**Cardiovascular death**	Tirzepatide	Metformin	1.05 (0.30 to 3.70)	44 per 1000 persons	46 per 1000 persons (14 to 146 per 1000 persons)	2 more (30 fewer to 102 more)	Very low
**Cardiovascular death**	SGLT2 inhibitors	Non-steroidal MRAs	0.99 (0.83 to 1.18)	41 per 1000 persons	41 per 1000 persons (34 to 48 per 1000 persons)	0 (7 fewer to 7 more)	Moderate
**Cardiovascular death**	Standard treatments	Non-steroidal MRAs	1.14 (0.98 to 1.33)	41 per 1000 persons	46 per 1000 persons (40 to 54 per 1000 persons)	5 more (1 fewer to 13 more)	Low
**Cardiovascular death**	Sulfonylureas	Non-steroidal MRAs	1.15 (0.89 to 1.49)	41 per 1000 persons	47 per 1000 persons (37 to 60 per 1000 persons)	6 more (4 fewer to 19 more)	Low
**Cardiovascular death**	Thiazolidinediones	Non-steroidal MRAs	1.06 (0.83 to 1.35)	41 per 1000 persons	44 per 1000 persons (34 to 55 per 1000 persons)	3 more (7 fewer to 14 more)	Low
**Cardiovascular death**	Tirzepatide	Non-steroidal MRAs	1.14 (0.39 to 3.33)	41 per 1000 persons	46 per 1000 persons (16 to 125 per 1000 persons)	5 more (25 fewer to 84 more)	Very low
**Cardiovascular death**	Standard treatments	SGLT2 inhibitors	1.16 (1.06 to 1.25)	40 per 1000 persons	46 per 1000 persons (42 to 50 per 1000 persons)	6 more (2 more to 10 more)	High
**Cardiovascular death**	Sulfonylureas	SGLT2 inhibitors	1.16 (0.94 to 1.45)	40 per 1000 persons	46 per 1000 persons (38 to 57 per 1000 persons)	6 more (2 fewer to 17 more)	Low
**Cardiovascular death**	Thiazolidinediones	SGLT2 inhibitors	1.08 (0.88 to 1.32)	40 per 1000 persons	43 per 1000 persons (35 to 52 per 1000 persons)	3 more (5 fewer to 12 more)	Low
**Cardiovascular death**	Tirzepatide	SGLT2 inhibitors	1.15 (0.40 to 3.33)	40 per 1000 persons	46 per 1000 persons (16 to 122 per 1000 persons)	6 more (24 fewer to 82 more)	Very low
**Cardiovascular death**	Standard treatments	Sulfonylureas	0.99 (0.81 to 1.20)	46 per 1000 persons	46 per 1000 persons (38 to 55 per 1000 persons)	0 (8 fewer to 9 more)	Moderate

Outcome	Intervention	Comparator	Odds ratio	Baseline risk	Risk if treated	Risk difference	Certainty
**Cardiovascular death**	Thiazolidinediones	Sulfonylureas	0.92 (0.71 to 1.19)	46 per 1000 persons	42 per 1000 persons (33 to 54 per 1000 persons)	4 fewer (13 fewer to 8 more)	Low
**Cardiovascular death**	Tirzepatide	Sulfonylureas	0.99 (0.34 to 2.86)	46 per 1000 persons	46 per 1000 persons (16 to 121 per 1000 persons)	0 (30 fewer to 75 more)	Very low
**Cardiovascular death**	Standard treatments	Thiazolidinediones	1.08 (0.89 to 1.30)	43 per 1000 persons	46 per 1000 persons (39 to 55 per 1000 persons)	3 more (4 fewer to 12 more)	Low
**Cardiovascular death**	Tirzepatide	Thiazolidinediones	1.08 (0.37 to 3.12)	43 per 1000 persons	46 per 1000 persons (16 to 123 per 1000 persons)	3 more (27 fewer to 80 more)	Very low
**Cardiovascular death**	Standard treatments	Tirzepatide	1.00 (0.35 to 2.86)	46 per 1000 persons	46 per 1000 persons (17 to 121 per 1000 persons)	0 (29 fewer to 75 more)	Very low
**Non-fatal myocardial infarction**	Alpha-glucosidase inhibitors	Basal insulin	0.33 (0.05 to 2.28)	57 per 1000 persons	20 per 1000 persons (3 to 121 per 1000 persons)	37 fewer (54 fewer to 64 more)	Very low
**Non-fatal myocardial infarction**	Alpha-glucosidase inhibitors	Basal-Bolus insulin	1.00 (0.05 to 18.12)	20 per 1000 persons	20 per 1000 persons (1 to 270 per 1000 persons)	0 (19 fewer to 250 more)	Very low
**Non-fatal myocardial infarction**	Alpha-glucosidase inhibitors	Bolus insulin	0.28 (0.03 to 2.21)	68 per 1000 persons	20 per 1000 persons (2 to 139 per 1000 persons)	48 fewer (66 fewer to 71 more)	Very low
**Non-fatal myocardial infarction**	Alpha-glucosidase inhibitors	DPP-4 inhibitors	0.32 (0.06 to 1.90)	59 per 1000 persons	20 per 1000 persons (4 to 106 per 1000 persons)	39 fewer (55 fewer to 47 more)	Very low
**Non-fatal myocardial infarction**	Alpha-glucosidase inhibitors	GLP-1 receptor agonists	0.36 (0.06 to 2.10)	53 per 1000 persons	20 per 1000 persons (3 to 105 per 1000 persons)	33 fewer (50 fewer to 52 more)	Very low
**Non-fatal myocardial infarction**	Alpha-glucosidase inhibitors	Meglitinides	1.16 (0.10 to 12.83)	17 per 1000 persons	20 per 1000 persons (2 to 182 per 1000 persons)	3 more (15 fewer to 165 more)	Very low
**Non-fatal myocardial infarction**	Alpha-glucosidase inhibitors	Metformin	0.38 (0.06 to 2.27)	50 per 1000 persons	20 per 1000 persons (3 to 107 per 1000 persons)	30 fewer (47 fewer to 57 more)	Very low
**Non-fatal myocardial infarction**	Alpha-glucosidase inhibitors	Non-steroidal MRAs	0.36 (0.06 to 2.13)	53 per 1000 persons	20 per 1000 persons (3 to 107 per 1000 persons)	33 fewer (50 fewer to 54 more)	Very low
**Non-fatal myocardial infarction**	Alpha-glucosidase inhibitors	SGLT2 inhibitors	0.36 (0.06 to 2.14)	53 per 1000 persons	20 per 1000 persons (3 to 107 per 1000 persons)	33 fewer (50 fewer to 54 more)	Very low
**Non-fatal myocardial infarction**	Alpha-glucosidase inhibitors	Standard treatments	0.33 (0.06 to 1.92)	58 per 1000 persons	20 per 1000 persons (4 to 106 per 1000 persons)	38 fewer (54 fewer to 48 more)	Very low

Outcome	Intervention	Comparator	Odds ratio	Baseline risk	Risk if treated	Risk difference	Certainty
**Non-fatal myocardial infarction**	Alpha-glucosidase inhibitors	Sulfonylureas	0.33 (0.06 to 1.92)	58 per 1000 persons	20 per 1000 persons (4 to 106 per 1000 persons)	38 fewer (54 fewer to 48 more)	Very low
**Non-fatal myocardial infarction**	Alpha-glucosidase inhibitors	Thiazolidinediones	0.34 (0.06 to 2.00)	56 per 1000 persons	20 per 1000 persons (4 to 106 per 1000 persons)	36 fewer (52 fewer to 50 more)	Very low
**Non-fatal myocardial infarction**	Alpha-glucosidase inhibitors	Tirzepatide	0.48 (0.03 to 7.88)	41 per 1000 persons	20 per 1000 persons (1 to 252 per 1000 persons)	21 fewer (40 fewer to 211 more)	Very low
**Non-fatal myocardial infarction**	Basal insulin	Basal-Bolus insulin	2.98 (0.30 to 29.67)	20 per 1000 persons	57 per 1000 persons (6 to 377 per 1000 persons)	37 more (14 fewer to 357 more)	Very low
**Non-fatal myocardial infarction**	Basal insulin	Bolus insulin	0.83 (0.28 to 2.50)	68 per 1000 persons	57 per 1000 persons (20 to 154 per 1000 persons)	11 fewer (48 fewer to 86 more)	Very low
**Non-fatal myocardial infarction**	Basal insulin	DPP-4 inhibitors	0.97 (0.46 to 2.05)	59 per 1000 persons	57 per 1000 persons (28 to 114 per 1000 persons)	2 fewer (31 fewer to 55 more)	Very low
**Non-fatal myocardial infarction**	Basal insulin	GLP-1 receptor agonists	1.07 (0.51 to 2.25)	53 per 1000 persons	57 per 1000 persons (28 to 112 per 1000 persons)	4 more (25 fewer to 59 more)	Very low
**Non-fatal myocardial infarction**	Basal insulin	Meglitinides	3.47 (0.53 to 22.89)	17 per 1000 persons	57 per 1000 persons (9 to 284 per 1000 persons)	40 more (8 fewer to 267 more)	Low
**Non-fatal myocardial infarction**	Basal insulin	Metformin	1.14 (0.52 to 2.49)	50 per 1000 persons	57 per 1000 persons (27 to 116 per 1000 persons)	7 more (23 fewer to 66 more)	Very low
**Non-fatal myocardial infarction**	Basal insulin	Non-steroidal MRAs	1.08 (0.50 to 2.33)	53 per 1000 persons	57 per 1000 persons (27 to 115 per 1000 persons)	4 more (26 fewer to 62 more)	Very low
**Non-fatal myocardial infarction**	Basal insulin	SGLT2 inhibitors	1.09 (0.52 to 2.31)	53 per 1000 persons	57 per 1000 persons (28 to 114 per 1000 persons)	4 more (25 fewer to 61 more)	Very low
**Non-fatal myocardial infarction**	Basal insulin	Standard treatments	0.98 (0.47 to 2.06)	58 per 1000 persons	57 per 1000 persons (28 to 113 per 1000 persons)	1 fewer (30 fewer to 55 more)	Very low
**Non-fatal myocardial infarction**	Basal insulin	Sulfonylureas	0.98 (0.45 to 2.10)	58 per 1000 persons	57 per 1000 persons (27 to 114 per 1000 persons)	1 fewer (31 fewer to 56 more)	Very low
**Non-fatal myocardial infarction**	Basal insulin	Thiazolidinediones	1.01 (0.47 to 2.17)	56 per 1000 persons	57 per 1000 persons (27 to 114 per 1000 persons)	1 more (29 fewer to 58 more)	Very low
**Non-fatal myocardial infarction**	Basal insulin	Tirzepatide	1.42 (0.14 to 14.22)	41 per 1000 persons	57 per 1000 persons (6 to 378 per 1000 persons)	16 more (35 fewer to 337 more)	Very low

Outcome	Intervention	Comparator	Odds ratio	Baseline risk	Risk if treated	Risk difference	Certainty
**Non-fatal myocardial infarction**	Basal-Bolus insulin	Bolus insulin	0.28 (0.02 to 3.35)	68 per 1000 persons	20 per 1000 persons (1 to 196 per 1000 persons)	48 fewer (67 fewer to 128 more)	Very low
**Non-fatal myocardial infarction**	Basal-Bolus insulin	DPP-4 inhibitors	0.32 (0.03 to 3.24)	59 per 1000 persons	20 per 1000 persons (2 to 169 per 1000 persons)	39 fewer (57 fewer to 110 more)	Very low
**Non-fatal myocardial infarction**	Basal-Bolus insulin	GLP-1 receptor agonists	0.36 (0.04 to 3.57)	53 per 1000 persons	20 per 1000 persons (2 to 167 per 1000 persons)	33 fewer (51 fewer to 114 more)	Very low
**Non-fatal myocardial infarction**	Basal-Bolus insulin	Meglitinides	1.16 (0.07 to 20.69)	17 per 1000 persons	20 per 1000 persons (1 to 264 per 1000 persons)	3 more (16 fewer to 247 more)	Very low
**Non-fatal myocardial infarction**	Basal-Bolus insulin	Metformin	0.38 (0.04 to 3.85)	50 per 1000 persons	20 per 1000 persons (2 to 168 per 1000 persons)	30 fewer (48 fewer to 118 more)	Very low
**Non-fatal myocardial infarction**	Basal-Bolus insulin	Non-steroidal MRAs	0.36 (0.04 to 3.62)	53 per 1000 persons	20 per 1000 persons (2 to 168 per 1000 persons)	33 fewer (51 fewer to 115 more)	Very low
**Non-fatal myocardial infarction**	Basal-Bolus insulin	SGLT2 inhibitors	0.37 (0.04 to 3.65)	53 per 1000 persons	20 per 1000 persons (2 to 170 per 1000 persons)	33 fewer (51 fewer to 117 more)	Very low
**Non-fatal myocardial infarction**	Basal-Bolus insulin	Standard treatments	0.33 (0.03 to 3.27)	58 per 1000 persons	20 per 1000 persons (2 to 168 per 1000 persons)	38 fewer (56 fewer to 110 more)	Very low
**Non-fatal myocardial infarction**	Basal-Bolus insulin	Sulfonylureas	0.33 (0.03 to 3.28)	58 per 1000 persons	20 per 1000 persons (2 to 168 per 1000 persons)	38 fewer (56 fewer to 110 more)	Very low
**Non-fatal myocardial infarction**	Basal-Bolus insulin	Thiazolidinediones	0.34 (0.03 to 3.40)	56 per 1000 persons	20 per 1000 persons (2 to 168 per 1000 persons)	36 fewer (54 fewer to 112 more)	Very low
**Non-fatal myocardial infarction**	Basal-Bolus insulin	Tirzepatide	0.48 (0.02 to 11.31)	41 per 1000 persons	20 per 1000 persons (1 to 326 per 1000 persons)	21 fewer (40 fewer to 285 more)	Very low
**Non-fatal myocardial infarction**	Bolus insulin	DPP-4 inhibitors	1.17 (0.39 to 3.48)	59 per 1000 persons	68 per 1000 persons (24 to 179 per 1000 persons)	9 more (35 fewer to 120 more)	Very low
**Non-fatal myocardial infarction**	Bolus insulin	GLP-1 receptor agonists	1.29 (0.44 to 3.82)	53 per 1000 persons	67 per 1000 persons (24 to 176 per 1000 persons)	14 more (29 fewer to 123 more)	Very low
**Non-fatal myocardial infarction**	Bolus insulin	Meglitinides	4.19 (0.54 to 32.39)	17 per 1000 persons	68 per 1000 persons (9 to 359 per 1000 persons)	51 more (8 fewer to 342 more)	Very low
**Non-fatal myocardial infarction**	Bolus insulin	Metformin	1.37 (0.45 to 4.18)	50 per 1000 persons	67 per 1000 persons (23 to 180 per 1000 persons)	17 more (27 fewer to 130 more)	Very low

Outcome	Intervention	Comparator	Odds ratio	Baseline risk	Risk if treated	Risk difference	Certainty
**Non-fatal myocardial infarction**	Bolus insulin	Non-steroidal MRAs	1.30 (0.43 to 3.92)	53 per 1000 persons	68 per 1000 persons (23 to 180 per 1000 persons)	15 more (30 fewer to 127 more)	Very low
**Non-fatal myocardial infarction**	Bolus insulin	SGLT2 inhibitors	1.32 (0.44 to 3.92)	53 per 1000 persons	69 per 1000 persons (24 to 180 per 1000 persons)	16 more (29 fewer to 127 more)	Very low
**Non-fatal myocardial infarction**	Bolus insulin	Standard treatments	1.18 (0.40 to 3.50)	58 per 1000 persons	68 per 1000 persons (24 to 177 per 1000 persons)	10 more (34 fewer to 119 more)	Very low
**Non-fatal myocardial infarction**	Bolus insulin	Sulfonylureas	1.18 (0.39 to 3.55)	58 per 1000 persons	68 per 1000 persons (23 to 179 per 1000 persons)	10 more (35 fewer to 121 more)	Very low
**Non-fatal myocardial infarction**	Bolus insulin	Thiazolidinediones	1.22 (0.41 to 3.67)	56 per 1000 persons	67 per 1000 persons (24 to 179 per 1000 persons)	11 more (32 fewer to 123 more)	Very low
**Non-fatal myocardial infarction**	Bolus insulin	Tirzepatide	1.72 (0.15 to 19.58)	41 per 1000 persons	68 per 1000 persons (6 to 456 per 1000 persons)	27 more (35 fewer to 415 more)	Very low
**Non-fatal myocardial infarction**	DPP-4 inhibitors	GLP-1 receptor agonists	1.11 (0.99 to 1.24)	53 per 1000 persons	58 per 1000 persons (52 to 65 per 1000 persons)	5 more (1 fewer to 12 more)	Low
**Non-fatal myocardial infarction**	DPP-4 inhibitors	Meglitinides	3.58 (0.63 to 20.28)	17 per 1000 persons	58 per 1000 persons (11 to 260 per 1000 persons)	41 more (6 fewer to 243 more)	Low
**Non-fatal myocardial infarction**	DPP-4 inhibitors	Metformin	1.18 (0.91 to 1.51)	50 per 1000 persons	58 per 1000 persons (46 to 74 per 1000 persons)	8 more (4 fewer to 24 more)	Low
**Non-fatal myocardial infarction**	DPP-4 inhibitors	Non-steroidal MRAs	1.11 (0.88 to 1.39)	53 per 1000 persons	58 per 1000 persons (47 to 72 per 1000 persons)	5 more (6 fewer to 19 more)	Low
**Non-fatal myocardial infarction**	DPP-4 inhibitors	SGLT2 inhibitors	1.13 (0.99 to 1.28)	53 per 1000 persons	59 per 1000 persons (52 to 67 per 1000 persons)	6 more (1 fewer to 14 more)	Low
**Non-fatal myocardial infarction**	DPP-4 inhibitors	Standard treatments	1.01 (0.92 to 1.11)	58 per 1000 persons	59 per 1000 persons (54 to 64 per 1000 persons)	1 more (4 fewer to 6 more)	Moderate
**Non-fatal myocardial infarction**	DPP-4 inhibitors	Sulfonylureas	1.01 (0.84 to 1.21)	58 per 1000 persons	59 per 1000 persons (49 to 69 per 1000 persons)	1 more (9 fewer to 11 more)	Moderate
**Non-fatal myocardial infarction**	DPP-4 inhibitors	Thiazolidinediones	1.04 (0.87 to 1.26)	56 per 1000 persons	58 per 1000 persons (49 to 70 per 1000 persons)	2 more (7 fewer to 14 more)	Low
**Non-fatal myocardial infarction**	DPP-4 inhibitors	Tirzepatide	1.47 (0.17 to 13.01)	41 per 1000 persons	59 per 1000 persons (7 to 357 per 1000 persons)	18 more (34 fewer to 316 more)	Very low

Outcome	Intervention	Comparator	Odds ratio	Baseline risk	Risk if treated	Risk difference	Certainty
**Non-fatal myocardial infarction**	GLP-1 receptor agonists	Meglitinides	3.24 (0.57 to 18.37)	17 per 1000 persons	53 per 1000 persons (10 to 241 per 1000 persons)	36 more (7 fewer to 224 more)	Low
**Non-fatal myocardial infarction**	GLP-1 receptor agonists	Metformin	1.06 (0.83 to 1.36)	50 per 1000 persons	53 per 1000 persons (42 to 67 per 1000 persons)	3 more (8 fewer to 17 more)	Low
**Non-fatal myocardial infarction**	GLP-1 receptor agonists	Non-steroidal MRAs	1.00 (0.80 to 1.25)	53 per 1000 persons	53 per 1000 persons (43 to 65 per 1000 persons)	0 (10 fewer to 12 more)	Low
**Non-fatal myocardial infarction**	GLP-1 receptor agonists	SGLT2 inhibitors	1.02 (0.91 to 1.14)	53 per 1000 persons	54 per 1000 persons (48 to 60 per 1000 persons)	1 more (5 fewer to 7 more)	Moderate
**Non-fatal myocardial infarction**	GLP-1 receptor agonists	Standard treatments	0.91 (0.85 to 0.98)	58 per 1000 persons	53 per 1000 persons (50 to 57 per 1000 persons)	5 fewer (8 fewer to 1 fewer)	High
**Non-fatal myocardial infarction**	GLP-1 receptor agonists	Sulfonylureas	0.91 (0.74 to 1.12)	58 per 1000 persons	53 per 1000 persons (44 to 65 per 1000 persons)	5 fewer (14 fewer to 7 more)	Low
**Non-fatal myocardial infarction**	GLP-1 receptor agonists	Thiazolidinediones	0.95 (0.79 to 1.14)	56 per 1000 persons	53 per 1000 persons (45 to 63 per 1000 persons)	3 fewer (11 fewer to 7 more)	Low
**Non-fatal myocardial infarction**	GLP-1 receptor agonists	Tirzepatide	1.33 (0.15 to 11.74)	41 per 1000 persons	54 per 1000 persons (6 to 334 per 1000 persons)	13 more (35 fewer to 293 more)	Low
**Non-fatal myocardial infarction**	Meglitinides	Metformin	0.33 (0.06 to 1.89)	50 per 1000 persons	17 per 1000 persons (3 to 90 per 1000 persons)	33 fewer (47 fewer to 40 more)	Very low
**Non-fatal myocardial infarction**	Meglitinides	Non-steroidal MRAs	0.31 (0.05 to 1.78)	53 per 1000 persons	17 per 1000 persons (3 to 91 per 1000 persons)	36 fewer (50 fewer to 38 more)	Very low
**Non-fatal myocardial infarction**	Meglitinides	SGLT2 inhibitors	0.31 (0.06 to 1.79)	53 per 1000 persons	17 per 1000 persons (3 to 91 per 1000 persons)	36 fewer (50 fewer to 38 more)	Very low
**Non-fatal myocardial infarction**	Meglitinides	Standard treatments	0.28 (0.05 to 1.60)	58 per 1000 persons	17 per 1000 persons (3 to 90 per 1000 persons)	41 fewer (55 fewer to 32 more)	Very low
**Non-fatal myocardial infarction**	Meglitinides	Sulfonylureas	0.28 (0.05 to 1.59)	58 per 1000 persons	17 per 1000 persons (3 to 89 per 1000 persons)	41 fewer (55 fewer to 31 more)	Very low
**Non-fatal myocardial infarction**	Meglitinides	Thiazolidinediones	0.29 (0.05 to 1.66)	56 per 1000 persons	17 per 1000 persons (3 to 90 per 1000 persons)	39 fewer (53 fewer to 34 more)	Very low
**Non-fatal myocardial infarction**	Meglitinides	Tirzepatide	0.41 (0.03 to 6.64)	41 per 1000 persons	17 per 1000 persons (1 to 221 per 1000 persons)	24 fewer (40 fewer to 180 more)	Very low

Outcome	Intervention	Comparator	Odds ratio	Baseline risk	Risk if treated	Risk difference	Certainty
**Non-fatal myocardial infarction**	Metformin	Non-steroidal MRAs	0.94 (0.69 to 1.30)	53 per 1000 persons	50 per 1000 persons (37 to 68 per 1000 persons)	3 fewer (16 fewer to 15 more)	Very low
**Non-fatal myocardial infarction**	Metformin	SGLT2 inhibitors	0.96 (0.74 to 1.24)	53 per 1000 persons	51 per 1000 persons (40 to 65 per 1000 persons)	2 fewer (13 fewer to 12 more)	Very low
**Non-fatal myocardial infarction**	Metformin	Standard treatments	0.86 (0.68 to 1.09)	58 per 1000 persons	50 per 1000 persons (40 to 63 per 1000 persons)	8 fewer (18 fewer to 5 more)	Low
**Non-fatal myocardial infarction**	Metformin	Sulfonylureas	0.86 (0.64 to 1.14)	58 per 1000 persons	50 per 1000 persons (38 to 66 per 1000 persons)	8 fewer (20 fewer to 8 more)	Low
**Non-fatal myocardial infarction**	Metformin	Thiazolidinediones	0.89 (0.67 to 1.17)	56 per 1000 persons	50 per 1000 persons (38 to 65 per 1000 persons)	6 fewer (18 fewer to 9 more)	Low
**Non-fatal myocardial infarction**	Metformin	Tirzepatide	1.25 (0.14 to 11.19)	41 per 1000 persons	51 per 1000 persons (6 to 324 per 1000 persons)	10 more (35 fewer to 283 more)	Very low
**Non-fatal myocardial infarction**	Non-steroidal MRAs	SGLT2 inhibitors	1.02 (0.81 to 1.27)	53 per 1000 persons	54 per 1000 persons (43 to 66 per 1000 persons)	1 more (10 fewer to 13 more)	Low
**Non-fatal myocardial infarction**	Non-steroidal MRAs	Standard treatments	0.91 (0.74 to 1.12)	58 per 1000 persons	53 per 1000 persons (44 to 65 per 1000 persons)	5 fewer (14 fewer to 7 more)	Low
**Non-fatal myocardial infarction**	Non-steroidal MRAs	Sulfonylureas	0.91 (0.68 to 1.21)	58 per 1000 persons	53 per 1000 persons (40 to 69 per 1000 persons)	5 fewer (18 fewer to 11 more)	Very low
**Non-fatal myocardial infarction**	Non-steroidal MRAs	Thiazolidinediones	0.94 (0.72 to 1.24)	56 per 1000 persons	53 per 1000 persons (41 to 69 per 1000 persons)	3 fewer (15 fewer to 13 more)	Very low
**Non-fatal myocardial infarction**	Non-steroidal MRAs	Tirzepatide	1.32 (0.15 to 11.83)	41 per 1000 persons	53 per 1000 persons (6 to 336 per 1000 persons)	12 more (35 fewer to 295 more)	Very low
**Non-fatal myocardial infarction**	SGLT2 inhibitors	Standard treatments	0.90 (0.82 to 0.98)	58 per 1000 persons	53 per 1000 persons (48 to 57 per 1000 persons)	5 fewer (10 fewer to 1 fewer)	High
**Non-fatal myocardial infarction**	SGLT2 inhibitors	Sulfonylureas	0.89 (0.72 to 1.10)	58 per 1000 persons	52 per 1000 persons (42 to 63 per 1000 persons)	6 fewer (16 fewer to 5 more)	Low
**Non-fatal myocardial infarction**	SGLT2 inhibitors	Thiazolidinediones	0.93 (0.76 to 1.12)	56 per 1000 persons	52 per 1000 persons (43 to 62 per 1000 persons)	4 fewer (13 fewer to 6 more)	Low
**Non-fatal myocardial infarction**	SGLT2 inhibitors	Tirzepatide	1.30 (0.15 to 11.55)	41 per 1000 persons	53 per 1000 persons (6 to 331 per 1000 persons)	12 more (35 fewer to 290 more)	Very low

Outcome	Intervention	Comparator	Odds ratio	Baseline risk	Risk if treated	Risk difference	Certainty
**Non-fatal myocardial infarction**	Sulfonylureas	Standard treatments	1.00 (0.83 to 1.22)	58 per 1000 persons	58 per 1000 persons (49 to 70 per 1000 persons)	0 (9 fewer to 12 more)	Low
**Non-fatal myocardial infarction**	Sulfonylureas	Thiazolidinediones	1.04 (0.83 to 1.30)	56 per 1000 persons	58 per 1000 persons (47 to 72 per 1000 persons)	2 more (9 fewer to 16 more)	Low
**Non-fatal myocardial infarction**	Sulfonylureas	Tirzepatide	1.46 (0.16 to 13.00)	41 per 1000 persons	59 per 1000 persons (7 to 357 per 1000 persons)	18 more (34 fewer to 316 more)	Very low
**Non-fatal myocardial infarction**	Thiazolidinediones	Standard treatments	0.97 (0.81 to 1.15)	58 per 1000 persons	56 per 1000 persons (48 to 66 per 1000 persons)	2 fewer (10 fewer to 8 more)	Moderate
**Non-fatal myocardial infarction**	Thiazolidinediones	Tirzepatide	1.40 (0.16 to 12.51)	41 per 1000 persons	56 per 1000 persons (7 to 348 per 1000 persons)	15 more (34 fewer to 307 more)	Very low
**Non-fatal myocardial infarction**	Tirzepatide	Standard treatments	0.69 (0.08 to 6.10)	58 per 1000 persons	41 per 1000 persons (5 to 273 per 1000 persons)	17 fewer (53 fewer to 215 more)	Very low
**Non-fatal myocardial infarction**	Basal insulin	Alpha-glucosidase inhibitors	3.03 (0.44 to 20.00)	20 per 1000 persons	58 per 1000 persons (9 to 290 per 1000 persons)	38 more (11 fewer to 270 more)	Very low
**Non-fatal myocardial infarction**	Basal-Bolus insulin	Alpha-glucosidase inhibitors	1.00 (0.06 to 20.00)	20 per 1000 persons	20 per 1000 persons (1 to 290 per 1000 persons)	0 (19 fewer to 270 more)	Very low
**Non-fatal myocardial infarction**	Bolus insulin	Alpha-glucosidase inhibitors	3.57 (0.45 to 33.33)	20 per 1000 persons	68 per 1000 persons (9 to 405 per 1000 persons)	48 more (11 fewer to 385 more)	Very low
**Non-fatal myocardial infarction**	DPP-4 inhibitors	Alpha-glucosidase inhibitors	3.12 (0.53 to 16.67)	20 per 1000 persons	60 per 1000 persons (11 to 254 per 1000 persons)	40 more (9 fewer to 234 more)	Very low
**Non-fatal myocardial infarction**	GLP-1 receptor agonists	Alpha-glucosidase inhibitors	2.78 (0.48 to 16.67)	20 per 1000 persons	54 per 1000 persons (10 to 254 per 1000 persons)	34 more (10 fewer to 234 more)	Low
**Non-fatal myocardial infarction**	Meglitinides	Alpha-glucosidase inhibitors	0.86 (0.08 to 10.00)	20 per 1000 persons	17 per 1000 persons (2 to 169 per 1000 persons)	3 fewer (18 fewer to 149 more)	Very low
**Non-fatal myocardial infarction**	Metformin	Alpha-glucosidase inhibitors	2.63 (0.44 to 16.67)	20 per 1000 persons	51 per 1000 persons (9 to 254 per 1000 persons)	31 more (11 fewer to 234 more)	Very low
**Non-fatal myocardial infarction**	Non-steroidal MRAs	Alpha-glucosidase inhibitors	2.78 (0.47 to 16.67)	20 per 1000 persons	54 per 1000 persons (9 to 254 per 1000 persons)	34 more (11 fewer to 234 more)	Very low
**Non-fatal myocardial infarction**	SGLT2 inhibitors	Alpha-glucosidase inhibitors	2.78 (0.47 to 16.67)	20 per 1000 persons	54 per 1000 persons (9 to 254 per 1000 persons)	34 more (11 fewer to 234 more)	Very low

Outcome	Intervention	Comparator	Odds ratio	Baseline risk	Risk if treated	Risk difference	Certainty
**Non-fatal myocardial infarction**	Standard treatments	Alpha-glucosidase inhibitors	3.03 (0.52 to 16.67)	20 per 1000 persons	58 per 1000 persons (11 to 254 per 1000 persons)	38 more (9 fewer to 234 more)	Low
**Non-fatal myocardial infarction**	Sulfonylureas	Alpha-glucosidase inhibitors	3.03 (0.52 to 16.67)	20 per 1000 persons	58 per 1000 persons (11 to 254 per 1000 persons)	38 more (9 fewer to 234 more)	Low
**Non-fatal myocardial infarction**	Thiazolidinediones	Alpha-glucosidase inhibitors	2.94 (0.50 to 16.67)	20 per 1000 persons	57 per 1000 persons (10 to 254 per 1000 persons)	37 more (10 fewer to 234 more)	Very low
**Non-fatal myocardial infarction**	Tirzepatide	Alpha-glucosidase inhibitors	2.08 (0.13 to 33.33)	20 per 1000 persons	41 per 1000 persons (3 to 405 per 1000 persons)	21 more (17 fewer to 385 more)	Very low
**Non-fatal myocardial infarction**	Basal-Bolus insulin	Basal insulin	0.34 (0.03 to 3.33)	57 per 1000 persons	20 per 1000 persons (2 to 168 per 1000 persons)	37 fewer (55 fewer to 111 more)	Very low
**Non-fatal myocardial infarction**	Bolus insulin	Basal insulin	1.20 (0.40 to 3.57)	57 per 1000 persons	68 per 1000 persons (24 to 178 per 1000 persons)	11 more (33 fewer to 121 more)	Very low
**Non-fatal myocardial infarction**	DPP-4 inhibitors	Basal insulin	1.03 (0.49 to 2.17)	57 per 1000 persons	59 per 1000 persons (29 to 116 per 1000 persons)	2 more (28 fewer to 59 more)	Very low
**Non-fatal myocardial infarction**	GLP-1 receptor agonists	Basal insulin	0.93 (0.44 to 1.96)	57 per 1000 persons	53 per 1000 persons (26 to 106 per 1000 persons)	4 fewer (31 fewer to 49 more)	Very low
**Non-fatal myocardial infarction**	Meglitinides	Basal insulin	0.29 (0.04 to 1.89)	57 per 1000 persons	17 per 1000 persons (3 to 102 per 1000 persons)	40 fewer (54 fewer to 45 more)	Very low
**Non-fatal myocardial infarction**	Metformin	Basal insulin	0.88 (0.40 to 1.92)	57 per 1000 persons	50 per 1000 persons (24 to 104 per 1000 persons)	7 fewer (33 fewer to 47 more)	Very low
**Non-fatal myocardial infarction**	Non-steroidal MRAs	Basal insulin	0.93 (0.43 to 2.00)	57 per 1000 persons	53 per 1000 persons (25 to 108 per 1000 persons)	4 fewer (32 fewer to 51 more)	Very low
**Non-fatal myocardial infarction**	SGLT2 inhibitors	Basal insulin	0.92 (0.43 to 1.92)	57 per 1000 persons	53 per 1000 persons (25 to 104 per 1000 persons)	4 fewer (32 fewer to 47 more)	Very low
**Non-fatal myocardial infarction**	Standard treatments	Basal insulin	1.02 (0.49 to 2.13)	57 per 1000 persons	58 per 1000 persons (29 to 114 per 1000 persons)	1 more (28 fewer to 57 more)	Very low
**Non-fatal myocardial infarction**	Sulfonylureas	Basal insulin	1.02 (0.48 to 2.22)	57 per 1000 persons	58 per 1000 persons (28 to 118 per 1000 persons)	1 more (29 fewer to 61 more)	Very low
**Non-fatal myocardial infarction**	Thiazolidinediones	Basal insulin	0.99 (0.46 to 2.13)	57 per 1000 persons	56 per 1000 persons (27 to 114 per 1000 persons)	1 fewer (30 fewer to 57 more)	Very low

Outcome	Intervention	Comparator	Odds ratio	Baseline risk	Risk if treated	Risk difference	Certainty
**Non-fatal myocardial infarction**	Tirzepatide	Basal insulin	0.70 (0.07 to 7.14)	57 per 1000 persons	41 per 1000 persons (4 to 302 per 1000 persons)	16 fewer (53 fewer to 245 more)	Very low
**Non-fatal myocardial infarction**	Bolus insulin	Basal-Bolus insulin	3.57 (0.30 to 50.00)	20 per 1000 persons	68 per 1000 persons (6 to 505 per 1000 persons)	48 more (14 fewer to 485 more)	Very low
**Non-fatal myocardial infarction**	DPP-4 inhibitors	Basal-Bolus insulin	3.12 (0.31 to 33.33)	20 per 1000 persons	60 per 1000 persons (6 to 405 per 1000 persons)	40 more (14 fewer to 385 more)	Very low
**Non-fatal myocardial infarction**	GLP-1 receptor agonists	Basal-Bolus insulin	2.78 (0.28 to 25.00)	20 per 1000 persons	54 per 1000 persons (6 to 338 per 1000 persons)	34 more (14 fewer to 318 more)	Very low
**Non-fatal myocardial infarction**	Meglitinides	Basal-Bolus insulin	0.86 (0.05 to 14.29)	20 per 1000 persons	17 per 1000 persons (1 to 226 per 1000 persons)	3 fewer (19 fewer to 206 more)	Very low
**Non-fatal myocardial infarction**	Metformin	Basal-Bolus insulin	2.63 (0.26 to 25.00)	20 per 1000 persons	51 per 1000 persons (5 to 338 per 1000 persons)	31 more (15 fewer to 318 more)	Very low
**Non-fatal myocardial infarction**	Non-steroidal MRAs	Basal-Bolus insulin	2.78 (0.28 to 25.00)	20 per 1000 persons	54 per 1000 persons (6 to 338 per 1000 persons)	34 more (14 fewer to 318 more)	Very low
**Non-fatal myocardial infarction**	SGLT2 inhibitors	Basal-Bolus insulin	2.70 (0.27 to 25.00)	20 per 1000 persons	52 per 1000 persons (6 to 338 per 1000 persons)	32 more (14 fewer to 318 more)	Very low
**Non-fatal myocardial infarction**	Standard treatments	Basal-Bolus insulin	3.03 (0.31 to 33.33)	20 per 1000 persons	58 per 1000 persons (6 to 405 per 1000 persons)	38 more (14 fewer to 385 more)	Very low
**Non-fatal myocardial infarction**	Sulfonylureas	Basal-Bolus insulin	3.03 (0.30 to 33.33)	20 per 1000 persons	58 per 1000 persons (6 to 405 per 1000 persons)	38 more (14 fewer to 385 more)	Very low
**Non-fatal myocardial infarction**	Thiazolidinediones	Basal-Bolus insulin	2.94 (0.29 to 33.33)	20 per 1000 persons	57 per 1000 persons (6 to 405 per 1000 persons)	37 more (14 fewer to 385 more)	Very low
**Non-fatal myocardial infarction**	Tirzepatide	Basal-Bolus insulin	2.08 (0.09 to 50.00)	20 per 1000 persons	41 per 1000 persons (2 to 505 per 1000 persons)	21 more (18 fewer to 485 more)	Very low
**Non-fatal myocardial infarction**	DPP-4 inhibitors	Bolus insulin	0.85 (0.29 to 2.56)	68 per 1000 persons	59 per 1000 persons (21 to 158 per 1000 persons)	9 fewer (47 fewer to 90 more)	Very low
**Non-fatal myocardial infarction**	GLP-1 receptor agonists	Bolus insulin	0.78 (0.26 to 2.27)	68 per 1000 persons	54 per 1000 persons (19 to 142 per 1000 persons)	14 fewer (49 fewer to 74 more)	Very low
**Non-fatal myocardial infarction**	Meglitinides	Bolus insulin	0.24 (0.03 to 1.85)	68 per 1000 persons	17 per 1000 persons (2 to 119 per 1000 persons)	51 fewer (66 fewer to 51 more)	Very low

Outcome	Intervention	Comparator	Odds ratio	Baseline risk	Risk if treated	Risk difference	Certainty
**Non-fatal myocardial infarction**	Metformin	Bolus insulin	0.73 (0.24 to 2.22)	68 per 1000 persons	51 per 1000 persons (17 to 140 per 1000 persons)	17 fewer (51 fewer to 72 more)	Very low
**Non-fatal myocardial infarction**	Non-steroidal MRAs	Bolus insulin	0.77 (0.26 to 2.33)	68 per 1000 persons	53 per 1000 persons (18 to 145 per 1000 persons)	15 fewer (50 fewer to 77 more)	Very low
**Non-fatal myocardial infarction**	SGLT2 inhibitors	Bolus insulin	0.76 (0.26 to 2.27)	68 per 1000 persons	52 per 1000 persons (18 to 142 per 1000 persons)	16 fewer (50 fewer to 74 more)	Very low
**Non-fatal myocardial infarction**	Standard treatments	Bolus insulin	0.85 (0.29 to 2.50)	68 per 1000 persons	58 per 1000 persons (20 to 154 per 1000 persons)	10 fewer (48 fewer to 86 more)	Very low
**Non-fatal myocardial infarction**	Sulfonylureas	Bolus insulin	0.85 (0.28 to 2.56)	68 per 1000 persons	58 per 1000 persons (20 to 158 per 1000 persons)	10 fewer (48 fewer to 90 more)	Very low
**Non-fatal myocardial infarction**	Thiazolidinediones	Bolus insulin	0.82 (0.27 to 2.44)	68 per 1000 persons	56 per 1000 persons (19 to 151 per 1000 persons)	12 fewer (49 fewer to 83 more)	Very low
**Non-fatal myocardial infarction**	Tirzepatide	Bolus insulin	0.58 (0.05 to 6.67)	68 per 1000 persons	41 per 1000 persons (4 to 327 per 1000 persons)	27 fewer (64 fewer to 259 more)	Very low
**Non-fatal myocardial infarction**	GLP-1 receptor agonists	DPP-4 inhibitors	0.90 (0.81 to 1.01)	59 per 1000 persons	53 per 1000 persons (48 to 60 per 1000 persons)	6 fewer (11 fewer to 1 more)	Low
**Non-fatal myocardial infarction**	Meglitinides	DPP-4 inhibitors	0.28 (0.05 to 1.59)	59 per 1000 persons	17 per 1000 persons (3 to 91 per 1000 persons)	42 fewer (56 fewer to 32 more)	Very low
**Non-fatal myocardial infarction**	Metformin	DPP-4 inhibitors	0.85 (0.66 to 1.10)	59 per 1000 persons	50 per 1000 persons (40 to 64 per 1000 persons)	9 fewer (19 fewer to 5 more)	Low
**Non-fatal myocardial infarction**	Non-steroidal MRAs	DPP-4 inhibitors	0.90 (0.72 to 1.14)	59 per 1000 persons	53 per 1000 persons (43 to 67 per 1000 persons)	6 fewer (16 fewer to 8 more)	Low
**Non-fatal myocardial infarction**	SGLT2 inhibitors	DPP-4 inhibitors	0.88 (0.78 to 1.01)	59 per 1000 persons	53 per 1000 persons (47 to 60 per 1000 persons)	6 fewer (12 fewer to 1 more)	Low
**Non-fatal myocardial infarction**	Standard treatments	DPP-4 inhibitors	0.99 (0.90 to 1.09)	59 per 1000 persons	58 per 1000 persons (53 to 64 per 1000 persons)	1 fewer (6 fewer to 5 more)	Moderate
**Non-fatal myocardial infarction**	Sulfonylureas	DPP-4 inhibitors	0.99 (0.83 to 1.19)	59 per 1000 persons	58 per 1000 persons (49 to 69 per 1000 persons)	1 fewer (10 fewer to 10 more)	High
**Non-fatal myocardial infarction**	Thiazolidinediones	DPP-4 inhibitors	0.96 (0.79 to 1.15)	59 per 1000 persons	57 per 1000 persons (47 to 67 per 1000 persons)	2 fewer (12 fewer to 8 more)	Low

Outcome	Intervention	Comparator	Odds ratio	Baseline risk	Risk if treated	Risk difference	Certainty
**Non-fatal myocardial infarction**	Tirzepatide	DPP-4 inhibitors	0.68 (0.08 to 5.88)	59 per 1000 persons	41 per 1000 persons (5 to 269 per 1000 persons)	18 fewer (54 fewer to 210 more)	Very low
**Non-fatal myocardial infarction**	Meglitinides	GLP-1 receptor agonists	0.31 (0.05 to 1.75)	53 per 1000 persons	17 per 1000 persons (3 to 89 per 1000 persons)	36 fewer (50 fewer to 36 more)	Very low
**Non-fatal myocardial infarction**	Metformin	GLP-1 receptor agonists	0.94 (0.74 to 1.20)	53 per 1000 persons	50 per 1000 persons (40 to 63 per 1000 persons)	3 fewer (13 fewer to 10 more)	Low
**Non-fatal myocardial infarction**	Non-steroidal MRAs	GLP-1 receptor agonists	1.00 (0.80 to 1.25)	53 per 1000 persons	53 per 1000 persons (43 to 65 per 1000 persons)	0 (10 fewer to 12 more)	Low
**Non-fatal myocardial infarction**	SGLT2 inhibitors	GLP-1 receptor agonists	0.98 (0.88 to 1.10)	53 per 1000 persons	52 per 1000 persons (47 to 58 per 1000 persons)	1 fewer (6 fewer to 5 more)	Moderate
**Non-fatal myocardial infarction**	Standard treatments	GLP-1 receptor agonists	1.10 (1.02 to 1.18)	53 per 1000 persons	58 per 1000 persons (54 to 62 per 1000 persons)	5 more (1 more to 9 more)	High
**Non-fatal myocardial infarction**	Sulfonylureas	GLP-1 receptor agonists	1.10 (0.89 to 1.35)	53 per 1000 persons	58 per 1000 persons (48 to 70 per 1000 persons)	5 more (5 fewer to 17 more)	Low
**Non-fatal myocardial infarction**	Thiazolidinediones	GLP-1 receptor agonists	1.05 (0.88 to 1.27)	53 per 1000 persons	56 per 1000 persons (47 to 66 per 1000 persons)	3 more (6 fewer to 13 more)	Low
**Non-fatal myocardial infarction**	Tirzepatide	GLP-1 receptor agonists	0.75 (0.09 to 6.67)	53 per 1000 persons	40 per 1000 persons (5 to 272 per 1000 persons)	13 fewer (48 fewer to 219 more)	Low
**Non-fatal myocardial infarction**	Metformin	Meglitinides	3.03 (0.53 to 16.67)	17 per 1000 persons	50 per 1000 persons (9 to 224 per 1000 persons)	33 more (8 fewer to 207 more)	Low
**Non-fatal myocardial infarction**	Non-steroidal MRAs	Meglitinides	3.23 (0.56 to 20.00)	17 per 1000 persons	53 per 1000 persons (10 to 257 per 1000 persons)	36 more (7 fewer to 240 more)	Very low
**Non-fatal myocardial infarction**	SGLT2 inhibitors	Meglitinides	3.23 (0.56 to 16.67)	17 per 1000 persons	53 per 1000 persons (10 to 224 per 1000 persons)	36 more (7 fewer to 207 more)	Low
**Non-fatal myocardial infarction**	Standard treatments	Meglitinides	3.57 (0.62 to 20.00)	17 per 1000 persons	58 per 1000 persons (11 to 257 per 1000 persons)	41 more (6 fewer to 240 more)	Low
**Non-fatal myocardial infarction**	Sulfonylureas	Meglitinides	3.57 (0.63 to 20.00)	17 per 1000 persons	58 per 1000 persons (11 to 257 per 1000 persons)	41 more (6 fewer to 240 more)	Low
**Non-fatal myocardial infarction**	Thiazolidinediones	Meglitinides	3.45 (0.60 to 20.00)	17 per 1000 persons	56 per 1000 persons (10 to 257 per 1000 persons)	39 more (7 fewer to 240 more)	Very low

Outcome	Intervention	Comparator	Odds ratio	Baseline risk	Risk if treated	Risk difference	Certainty
**Non-fatal myocardial infarction**	Tirzepatide	Meglitinides	2.44 (0.15 to 33.33)	17 per 1000 persons	40 per 1000 persons (3 to 366 per 1000 persons)	23 more (14 fewer to 349 more)	Very low
**Non-fatal myocardial infarction**	Non-steroidal MRAs	Metformin	1.06 (0.77 to 1.45)	50 per 1000 persons	53 per 1000 persons (39 to 71 per 1000 persons)	3 more (11 fewer to 21 more)	Very low
**Non-fatal myocardial infarction**	SGLT2 inhibitors	Metformin	1.04 (0.81 to 1.35)	50 per 1000 persons	52 per 1000 persons (41 to 66 per 1000 persons)	2 more (9 fewer to 16 more)	Low
**Non-fatal myocardial infarction**	Standard treatments	Metformin	1.16 (0.92 to 1.47)	50 per 1000 persons	58 per 1000 persons (46 to 72 per 1000 persons)	8 more (4 fewer to 22 more)	Low
**Non-fatal myocardial infarction**	Sulfonylureas	Metformin	1.16 (0.88 to 1.56)	50 per 1000 persons	58 per 1000 persons (44 to 76 per 1000 persons)	8 more (6 fewer to 26 more)	Low
**Non-fatal myocardial infarction**	Thiazolidinediones	Metformin	1.12 (0.85 to 1.49)	50 per 1000 persons	56 per 1000 persons (43 to 73 per 1000 persons)	6 more (7 fewer to 23 more)	Low
**Non-fatal myocardial infarction**	Tirzepatide	Metformin	0.80 (0.09 to 7.14)	50 per 1000 persons	40 per 1000 persons (5 to 273 per 1000 persons)	10 fewer (45 fewer to 223 more)	Very low
**Non-fatal myocardial infarction**	SGLT2 inhibitors	Non-steroidal MRAs	0.98 (0.79 to 1.23)	53 per 1000 persons	52 per 1000 persons (42 to 65 per 1000 persons)	1 fewer (11 fewer to 12 more)	Very low
**Non-fatal myocardial infarction**	Standard treatments	Non-steroidal MRAs	1.10 (0.89 to 1.35)	53 per 1000 persons	58 per 1000 persons (48 to 70 per 1000 persons)	5 more (5 fewer to 17 more)	Low
**Non-fatal myocardial infarction**	Sulfonylureas	Non-steroidal MRAs	1.10 (0.83 to 1.47)	53 per 1000 persons	58 per 1000 persons (44 to 76 per 1000 persons)	5 more (9 fewer to 23 more)	Low
**Non-fatal myocardial infarction**	Thiazolidinediones	Non-steroidal MRAs	1.06 (0.81 to 1.39)	53 per 1000 persons	56 per 1000 persons (43 to 72 per 1000 persons)	3 more (10 fewer to 19 more)	Low
**Non-fatal myocardial infarction**	Tirzepatide	Non-steroidal MRAs	0.76 (0.08 to 6.67)	53 per 1000 persons	41 per 1000 persons (5 to 272 per 1000 persons)	12 fewer (48 fewer to 219 more)	Very low
**Non-fatal myocardial infarction**	Standard treatments	SGLT2 inhibitors	1.11 (1.02 to 1.22)	53 per 1000 persons	59 per 1000 persons (54 to 64 per 1000 persons)	6 more (1 more to 11 more)	Moderate
**Non-fatal myocardial infarction**	Sulfonylureas	SGLT2 inhibitors	1.12 (0.91 to 1.39)	53 per 1000 persons	59 per 1000 persons (48 to 72 per 1000 persons)	6 more (5 fewer to 19 more)	Low
**Non-fatal myocardial infarction**	Thiazolidinediones	SGLT2 inhibitors	1.08 (0.89 to 1.32)	53 per 1000 persons	57 per 1000 persons (48 to 69 per 1000 persons)	4 more (5 fewer to 16 more)	Low

Outcome	Intervention	Comparator	Odds ratio	Baseline risk	Risk if treated	Risk difference	Certainty
**Non-fatal myocardial infarction**	Tirzepatide	SGLT2 inhibitors	0.77 (0.09 to 6.67)	53 per 1000 persons	41 per 1000 persons (5 to 272 per 1000 persons)	12 fewer (48 fewer to 219 more)	Very low
**Non-fatal myocardial infarction**	Standard treatments	Sulfonylureas	1.00 (0.82 to 1.20)	58 per 1000 persons	58 per 1000 persons (48 to 69 per 1000 persons)	0 (10 fewer to 11 more)	Low
**Non-fatal myocardial infarction**	Thiazolidinediones	Sulfonylureas	0.96 (0.77 to 1.20)	58 per 1000 persons	56 per 1000 persons (45 to 69 per 1000 persons)	2 fewer (13 fewer to 11 more)	Very low
**Non-fatal myocardial infarction**	Tirzepatide	Sulfonylureas	0.68 (0.08 to 6.25)	58 per 1000 persons	40 per 1000 persons (5 to 278 per 1000 persons)	18 fewer (53 fewer to 220 more)	Very low
**Non-fatal myocardial infarction**	Standard treatments	Thiazolidinediones	1.03 (0.87 to 1.23)	56 per 1000 persons	58 per 1000 persons (49 to 68 per 1000 persons)	2 more (7 fewer to 12 more)	Low
**Non-fatal myocardial infarction**	Tirzepatide	Thiazolidinediones	0.71 (0.08 to 6.25)	56 per 1000 persons	41 per 1000 persons (5 to 270 per 1000 persons)	15 fewer (51 fewer to 214 more)	Very low
**Non-fatal myocardial infarction**	Standard treatments	Tirzepatide	1.45 (0.16 to 12.50)	41 per 1000 persons	58 per 1000 persons (7 to 348 per 1000 persons)	17 more (34 fewer to 307 more)	Very low
**Non-fatal stroke**	Alpha-glucosidase inhibitors	Basal insulin	12.42 (0.88 to 176.12)	45 per 1000 persons	369 per 1000 persons (40 to 892 per 1000 persons)	324 more (5 fewer to 847 more)	Low
**Non-fatal stroke**	Alpha-glucosidase inhibitors	Basal-Bolus insulin	16.34 (0.76 to 350.38)	34 per 1000 persons	365 per 1000 persons (26 to 925 per 1000 persons)	331 more (8 fewer to 891 more)	Very low
**Non-fatal stroke**	Alpha-glucosidase inhibitors	Bolus insulin	11.00 (0.54 to 222.94)	50 per 1000 persons	367 per 1000 persons (28 to 921 per 1000 persons)	317 more (22 fewer to 871 more)	Very low
**Non-fatal stroke**	Alpha-glucosidase inhibitors	DPP-4 inhibitors	10.41 (0.84 to 128.53)	53 per 1000 persons	368 per 1000 persons (45 to 878 per 1000 persons)	315 more (8 fewer to 825 more)	Very low
**Non-fatal stroke**	Alpha-glucosidase inhibitors	GLP-1 receptor agonists	11.08 (0.90 to 137.09)	50 per 1000 persons	368 per 1000 persons (45 to 878 per 1000 persons)	318 more (5 fewer to 828 more)	Low
**Non-fatal stroke**	Alpha-glucosidase inhibitors	Meglitinides	5.53 (0.45 to 67.89)	95 per 1000 persons	367 per 1000 persons (45 to 877 per 1000 persons)	272 more (50 fewer to 782 more)	Very low
**Non-fatal stroke**	Alpha-glucosidase inhibitors	Metformin	9.73 (0.78 to 121.84)	56 per 1000 persons	366 per 1000 persons (44 to 878 per 1000 persons)	310 more (12 fewer to 822 more)	Very low
**Non-fatal stroke**	Alpha-glucosidase inhibitors	Non-steroidal MRAs	9.46 (0.76 to 117.72)	58 per 1000 persons	368 per 1000 persons (45 to 879 per 1000 persons)	310 more (13 fewer to 821 more)	Very low

Outcome	Intervention	Comparator	Odds ratio	Baseline risk	Risk if treated	Risk difference	Certainty
**Non-fatal stroke**	Alpha-glucosidase inhibitors	SGLT2 inhibitors	9.52 (0.77 to 117.89)	57 per 1000 persons	365 per 1000 persons (44 to 877 per 1000 persons)	308 more (13 fewer to 820 more)	Very low
**Non-fatal stroke**	Alpha-glucosidase inhibitors	Standard treatments	9.44 (0.76 to 116.58)	58 per 1000 persons	368 per 1000 persons (45 to 878 per 1000 persons)	310 more (13 fewer to 820 more)	Very low
**Non-fatal stroke**	Alpha-glucosidase inhibitors	Sulfonylureas	8.97 (0.73 to 110.10)	61 per 1000 persons	368 per 1000 persons (45 to 877 per 1000 persons)	307 more (16 fewer to 816 more)	Very low
**Non-fatal stroke**	Alpha-glucosidase inhibitors	Thiazolidinediones	11.12 (0.90 to 137.86)	50 per 1000 persons	369 per 1000 persons (45 to 879 per 1000 persons)	319 more (5 fewer to 829 more)	Very low
**Non-fatal stroke**	Basal insulin	Basal-Bolus insulin	1.32 (0.23 to 7.61)	34 per 1000 persons	44 per 1000 persons (8 to 211 per 1000 persons)	10 more (26 fewer to 177 more)	Very low
**Non-fatal stroke**	Basal insulin	Bolus insulin	0.89 (0.14 to 5.66)	50 per 1000 persons	45 per 1000 persons (7 to 230 per 1000 persons)	5 fewer (43 fewer to 180 more)	Very low
**Non-fatal stroke**	Basal insulin	DPP-4 inhibitors	0.84 (0.36 to 1.97)	53 per 1000 persons	45 per 1000 persons (20 to 99 per 1000 persons)	8 fewer (33 fewer to 46 more)	Very low
**Non-fatal stroke**	Basal insulin	GLP-1 receptor agonists	0.89 (0.38 to 2.07)	50 per 1000 persons	45 per 1000 persons (20 to 98 per 1000 persons)	5 fewer (30 fewer to 48 more)	Very low
**Non-fatal stroke**	Basal insulin	Meglitinides	0.45 (0.06 to 3.56)	95 per 1000 persons	45 per 1000 persons (6 to 272 per 1000 persons)	50 fewer (89 fewer to 177 more)	Very low
**Non-fatal stroke**	Basal insulin	Metformin	0.78 (0.32 to 1.93)	56 per 1000 persons	44 per 1000 persons (19 to 103 per 1000 persons)	12 fewer (37 fewer to 47 more)	Very low
**Non-fatal stroke**	Basal insulin	Non-steroidal MRAs	0.76 (0.32 to 1.82)	58 per 1000 persons	45 per 1000 persons (19 to 101 per 1000 persons)	13 fewer (39 fewer to 43 more)	Very low
**Non-fatal stroke**	Basal insulin	SGLT2 inhibitors	0.77 (0.33 to 1.80)	57 per 1000 persons	44 per 1000 persons (20 to 98 per 1000 persons)	13 fewer (37 fewer to 41 more)	Very low
**Non-fatal stroke**	Basal insulin	Standard treatments	0.76 (0.33 to 1.77)	58 per 1000 persons	45 per 1000 persons (20 to 98 per 1000 persons)	13 fewer (38 fewer to 40 more)	Very low
**Non-fatal stroke**	Basal insulin	Sulfonylureas	0.72 (0.30 to 1.73)	61 per 1000 persons	45 per 1000 persons (19 to 101 per 1000 persons)	16 fewer (42 fewer to 40 more)	Very low
**Non-fatal stroke**	Basal insulin	Thiazolidinediones	0.90 (0.38 to 2.13)	50 per 1000 persons	45 per 1000 persons (20 to 101 per 1000 persons)	5 fewer (30 fewer to 51 more)	Very low

Outcome	Intervention	Comparator	Odds ratio	Baseline risk	Risk if treated	Risk difference	Certainty
**Non-fatal stroke**	Basal-Bolus insulin	Bolus insulin	0.67 (0.06 to 7.50)	50 per 1000 persons	34 per 1000 persons (3 to 283 per 1000 persons)	16 fewer (47 fewer to 233 more)	Very low
**Non-fatal stroke**	Basal-Bolus insulin	DPP-4 inhibitors	0.64 (0.11 to 3.71)	53 per 1000 persons	35 per 1000 persons (6 to 172 per 1000 persons)	18 fewer (47 fewer to 119 more)	Very low
**Non-fatal stroke**	Basal-Bolus insulin	GLP-1 receptor agonists	0.68 (0.12 to 3.92)	50 per 1000 persons	35 per 1000 persons (6 to 171 per 1000 persons)	15 fewer (44 fewer to 121 more)	Very low
**Non-fatal stroke**	Basal-Bolus insulin	Meglitinides	0.34 (0.03 to 4.50)	95 per 1000 persons	34 per 1000 persons (3 to 321 per 1000 persons)	61 fewer (92 fewer to 226 more)	Very low
**Non-fatal stroke**	Basal-Bolus insulin	Metformin	0.60 (0.10 to 3.54)	56 per 1000 persons	34 per 1000 persons (6 to 174 per 1000 persons)	22 fewer (50 fewer to 118 more)	Very low
**Non-fatal stroke**	Basal-Bolus insulin	Non-steroidal MRAs	0.58 (0.10 to 3.39)	58 per 1000 persons	34 per 1000 persons (6 to 173 per 1000 persons)	24 fewer (52 fewer to 115 more)	Very low
**Non-fatal stroke**	Basal-Bolus insulin	SGLT2 inhibitors	0.58 (0.10 to 3.39)	57 per 1000 persons	34 per 1000 persons (6 to 170 per 1000 persons)	23 fewer (51 fewer to 113 more)	Very low
**Non-fatal stroke**	Basal-Bolus insulin	Standard treatments	0.58 (0.10 to 3.35)	58 per 1000 persons	34 per 1000 persons (6 to 171 per 1000 persons)	24 fewer (52 fewer to 113 more)	Very low
**Non-fatal stroke**	Basal-Bolus insulin	Sulfonylureas	0.55 (0.09 to 3.22)	61 per 1000 persons	34 per 1000 persons (6 to 173 per 1000 persons)	27 fewer (55 fewer to 112 more)	Very low
**Non-fatal stroke**	Basal-Bolus insulin	Thiazolidinediones	0.68 (0.12 to 3.96)	50 per 1000 persons	35 per 1000 persons (6 to 172 per 1000 persons)	15 fewer (44 fewer to 122 more)	Very low
**Non-fatal stroke**	Bolus insulin	DPP-4 inhibitors	0.95 (0.18 to 4.97)	53 per 1000 persons	50 per 1000 persons (10 to 218 per 1000 persons)	3 fewer (43 fewer to 165 more)	Very low
**Non-fatal stroke**	Bolus insulin	GLP-1 receptor agonists	1.01 (0.19 to 5.24)	50 per 1000 persons	50 per 1000 persons (10 to 216 per 1000 persons)	0 (40 fewer to 166 more)	Very low
**Non-fatal stroke**	Bolus insulin	Meglitinides	0.50 (0.04 to 6.24)	95 per 1000 persons	50 per 1000 persons (4 to 396 per 1000 persons)	45 fewer (91 fewer to 301 more)	Very low
**Non-fatal stroke**	Bolus insulin	Metformin	0.88 (0.16 to 4.76)	56 per 1000 persons	50 per 1000 persons (9 to 220 per 1000 persons)	6 fewer (47 fewer to 164 more)	Very low
**Non-fatal stroke**	Bolus insulin	Non-steroidal MRAs	0.86 (0.16 to 4.54)	58 per 1000 persons	50 per 1000 persons (10 to 218 per 1000 persons)	8 fewer (48 fewer to 160 more)	Very low

Outcome	Intervention	Comparator	Odds ratio	Baseline risk	Risk if treated	Risk difference	Certainty
**Non-fatal stroke**	Bolus insulin	SGLT2 inhibitors	0.87 (0.16 to 4.54)	57 per 1000 persons	50 per 1000 persons (10 to 215 per 1000 persons)	7 fewer (47 fewer to 158 more)	Very low
**Non-fatal stroke**	Bolus insulin	Standard treatments	0.86 (0.16 to 4.48)	58 per 1000 persons	50 per 1000 persons (10 to 216 per 1000 persons)	8 fewer (48 fewer to 158 more)	Very low
**Non-fatal stroke**	Bolus insulin	Sulfonylureas	0.82 (0.15 to 4.33)	61 per 1000 persons	51 per 1000 persons (10 to 220 per 1000 persons)	10 fewer (51 fewer to 159 more)	Very low
**Non-fatal stroke**	Bolus insulin	Thiazolidinediones	1.01 (0.19 to 5.34)	50 per 1000 persons	50 per 1000 persons (10 to 219 per 1000 persons)	0 (40 fewer to 169 more)	Very low
**Non-fatal stroke**	DPP-4 inhibitors	GLP-1 receptor agonists	1.06 (0.91 to 1.25)	50 per 1000 persons	53 per 1000 persons (46 to 62 per 1000 persons)	3 more (4 fewer to 12 more)	Low
**Non-fatal stroke**	DPP-4 inhibitors	Meglitinides	0.53 (0.08 to 3.55)	95 per 1000 persons	53 per 1000 persons (8 to 271 per 1000 persons)	42 fewer (87 fewer to 176 more)	Very low
**Non-fatal stroke**	DPP-4 inhibitors	Metformin	0.93 (0.67 to 1.29)	56 per 1000 persons	52 per 1000 persons (38 to 71 per 1000 persons)	4 fewer (18 fewer to 15 more)	Very low
**Non-fatal stroke**	DPP-4 inhibitors	Non-steroidal MRAs	0.91 (0.72 to 1.15)	58 per 1000 persons	53 per 1000 persons (42 to 66 per 1000 persons)	5 fewer (16 fewer to 8 more)	Low
**Non-fatal stroke**	DPP-4 inhibitors	SGLT2 inhibitors	0.91 (0.77 to 1.08)	57 per 1000 persons	52 per 1000 persons (44 to 61 per 1000 persons)	5 fewer (13 fewer to 4 more)	Low
**Non-fatal stroke**	DPP-4 inhibitors	Standard treatments	0.91 (0.80 to 1.03)	58 per 1000 persons	53 per 1000 persons (47 to 60 per 1000 persons)	5 fewer (11 fewer to 2 more)	Low
**Non-fatal stroke**	DPP-4 inhibitors	Sulfonylureas	0.86 (0.69 to 1.07)	61 per 1000 persons	53 per 1000 persons (43 to 65 per 1000 persons)	8 fewer (18 fewer to 4 more)	Moderate
**Non-fatal stroke**	DPP-4 inhibitors	Thiazolidinediones	1.07 (0.85 to 1.33)	50 per 1000 persons	53 per 1000 persons (43 to 65 per 1000 persons)	3 more (7 fewer to 15 more)	Very low
**Non-fatal stroke**	GLP-1 receptor agonists	Meglitinides	0.50 (0.07 to 3.35)	95 per 1000 persons	50 per 1000 persons (7 to 260 per 1000 persons)	45 fewer (88 fewer to 165 more)	Very low
**Non-fatal stroke**	GLP-1 receptor agonists	Metformin	0.88 (0.63 to 1.22)	56 per 1000 persons	50 per 1000 persons (36 to 67 per 1000 persons)	6 fewer (20 fewer to 11 more)	Very low
**Non-fatal stroke**	GLP-1 receptor agonists	Non-steroidal MRAs	0.85 (0.68 to 1.07)	58 per 1000 persons	50 per 1000 persons (40 to 62 per 1000 persons)	8 fewer (18 fewer to 4 more)	Low

Outcome	Intervention	Comparator	Odds ratio	Baseline risk	Risk if treated	Risk difference	Certainty
**Non-fatal stroke**	GLP-1 receptor agonists	SGLT2 inhibitors	0.86 (0.74 to 1.00)	57 per 1000 persons	49 per 1000 persons (43 to 57 per 1000 persons)	8 fewer (14 fewer to 0)	Low
**Non-fatal stroke**	GLP-1 receptor agonists	Standard treatments	0.85 (0.77 to 0.94)	58 per 1000 persons	50 per 1000 persons (45 to 55 per 1000 persons)	8 fewer (13 fewer to 3 fewer)	Moderate
**Non-fatal stroke**	GLP-1 receptor agonists	Sulfonylureas	0.81 (0.63 to 1.04)	61 per 1000 persons	50 per 1000 persons (39 to 63 per 1000 persons)	11 fewer (22 fewer to 2 more)	Low
**Non-fatal stroke**	GLP-1 receptor agonists	Thiazolidinediones	1.00 (0.80 to 1.25)	50 per 1000 persons	50 per 1000 persons (40 to 62 per 1000 persons)	0 (10 fewer to 12 more)	Low
**Non-fatal stroke**	Meglitinides	Metformin	1.76 (0.26 to 11.98)	56 per 1000 persons	95 per 1000 persons (15 to 415 per 1000 persons)	39 more (41 fewer to 359 more)	Very low
**Non-fatal stroke**	Meglitinides	Non-steroidal MRAs	1.71 (0.25 to 11.54)	58 per 1000 persons	95 per 1000 persons (15 to 415 per 1000 persons)	37 more (43 fewer to 357 more)	Very low
**Non-fatal stroke**	Meglitinides	SGLT2 inhibitors	1.72 (0.26 to 11.53)	57 per 1000 persons	94 per 1000 persons (15 to 411 per 1000 persons)	37 more (42 fewer to 354 more)	Very low
**Non-fatal stroke**	Meglitinides	Standard treatments	1.71 (0.26 to 11.40)	58 per 1000 persons	95 per 1000 persons (16 to 412 per 1000 persons)	37 more (42 fewer to 354 more)	Very low
**Non-fatal stroke**	Meglitinides	Sulfonylureas	1.62 (0.24 to 10.79)	61 per 1000 persons	95 per 1000 persons (15 to 412 per 1000 persons)	34 more (46 fewer to 351 more)	Very low
**Non-fatal stroke**	Meglitinides	Thiazolidinediones	2.01 (0.30 to 13.51)	50 per 1000 persons	96 per 1000 persons (16 to 416 per 1000 persons)	46 more (34 fewer to 366 more)	Very low
**Non-fatal stroke**	Metformin	Non-steroidal MRAs	0.97 (0.67 to 1.41)	58 per 1000 persons	56 per 1000 persons (40 to 80 per 1000 persons)	2 fewer (18 fewer to 22 more)	Very low
**Non-fatal stroke**	Metformin	SGLT2 inhibitors	0.98 (0.70 to 1.37)	57 per 1000 persons	56 per 1000 persons (41 to 76 per 1000 persons)	1 fewer (16 fewer to 19 more)	Very low
**Non-fatal stroke**	Metformin	Standard treatments	0.97 (0.71 to 1.33)	58 per 1000 persons	56 per 1000 persons (42 to 76 per 1000 persons)	2 fewer (16 fewer to 18 more)	Very low
**Non-fatal stroke**	Metformin	Sulfonylureas	0.92 (0.66 to 1.30)	61 per 1000 persons	56 per 1000 persons (41 to 78 per 1000 persons)	5 fewer (20 fewer to 17 more)	Very low
**Non-fatal stroke**	Metformin	Thiazolidinediones	1.14 (0.81 to 1.61)	50 per 1000 persons	57 per 1000 persons (41 to 78 per 1000 persons)	7 more (9 fewer to 28 more)	Very low

Outcome	Intervention	Comparator	Odds ratio	Baseline risk	Risk if treated	Risk difference	Certainty
**Non-fatal stroke**	Non-steroidal MRAs	SGLT2 inhibitors	1.01 (0.80 to 1.27)	57 per 1000 persons	58 per 1000 persons (46 to 71 per 1000 persons)	1 more (11 fewer to 14 more)	Very low
**Non-fatal stroke**	Non-steroidal MRAs	Standard treatments	1.00 (0.82 to 1.22)	58 per 1000 persons	58 per 1000 persons (48 to 70 per 1000 persons)	0 (10 fewer to 12 more)	Low
**Non-fatal stroke**	Non-steroidal MRAs	Sulfonylureas	0.95 (0.70 to 1.29)	61 per 1000 persons	58 per 1000 persons (43 to 77 per 1000 persons)	3 fewer (18 fewer to 16 more)	Very low
**Non-fatal stroke**	Non-steroidal MRAs	Thiazolidinediones	1.18 (0.89 to 1.56)	50 per 1000 persons	58 per 1000 persons (45 to 76 per 1000 persons)	8 more (5 fewer to 26 more)	Very low
**Non-fatal stroke**	SGLT2 inhibitors	Standard treatments	0.99 (0.88 to 1.11)	58 per 1000 persons	57 per 1000 persons (51 to 64 per 1000 persons)	1 fewer (7 fewer to 6 more)	High
**Non-fatal stroke**	SGLT2 inhibitors	Sulfonylureas	0.94 (0.73 to 1.21)	61 per 1000 persons	58 per 1000 persons (45 to 73 per 1000 persons)	3 fewer (16 fewer to 12 more)	Very low
**Non-fatal stroke**	SGLT2 inhibitors	Thiazolidinediones	1.17 (0.93 to 1.47)	50 per 1000 persons	58 per 1000 persons (47 to 72 per 1000 persons)	8 more (3 fewer to 22 more)	Low
**Non-fatal stroke**	Sulfonylureas	Standard treatments	1.05 (0.84 to 1.32)	58 per 1000 persons	61 per 1000 persons (49 to 75 per 1000 persons)	3 more (9 fewer to 17 more)	Low
**Non-fatal stroke**	Sulfonylureas	Thiazolidinediones	1.24 (0.96 to 1.61)	50 per 1000 persons	61 per 1000 persons (48 to 78 per 1000 persons)	11 more (2 fewer to 28 more)	Low
**Non-fatal stroke**	Thiazolidinediones	Standard treatments	0.85 (0.70 to 1.03)	58 per 1000 persons	50 per 1000 persons (41 to 60 per 1000 persons)	8 fewer (17 fewer to 2 more)	Very low
**Non-fatal stroke**	Basal insulin	Alpha-glucosidase inhibitors	0.08 (0.01 to 1.14)	368 per 1000 persons	45 per 1000 persons (3 to 398 per 1000 persons)	323 fewer (365 fewer to 30 more)	Very low
**Non-fatal stroke**	Basal-Bolus insulin	Alpha-glucosidase inhibitors	0.06 (0.00 to 1.32)	368 per 1000 persons	34 per 1000 persons (2 to 434 per 1000 persons)	334 fewer (366 fewer to 66 more)	Very low
**Non-fatal stroke**	Bolus insulin	Alpha-glucosidase inhibitors	0.09 (0.00 to 1.85)	368 per 1000 persons	50 per 1000 persons (3 to 519 per 1000 persons)	318 fewer (365 fewer to 151 more)	Very low
**Non-fatal stroke**	DPP-4 inhibitors	Alpha-glucosidase inhibitors	0.10 (0.01 to 1.19)	368 per 1000 persons	53 per 1000 persons (5 to 409 per 1000 persons)	315 fewer (363 fewer to 41 more)	Very low
**Non-fatal stroke**	GLP-1 receptor agonists	Alpha-glucosidase inhibitors	0.09 (0.01 to 1.11)	368 per 1000 persons	50 per 1000 persons (4 to 393 per 1000 persons)	318 fewer (364 fewer to 25 more)	Very low

Outcome	Intervention	Comparator	Odds ratio	Baseline risk	Risk if treated	Risk difference	Certainty
**Non-fatal stroke**	Meglitinides	Alpha-glucosidase inhibitors	0.18 (0.01 to 2.22)	368 per 1000 persons	95 per 1000 persons (9 to 564 per 1000 persons)	273 fewer (359 fewer to 196 more)	Very low
**Non-fatal stroke**	Metformin	Alpha-glucosidase inhibitors	0.10 (0.01 to 1.28)	368 per 1000 persons	56 per 1000 persons (5 to 427 per 1000 persons)	312 fewer (363 fewer to 59 more)	Very low
**Non-fatal stroke**	Non-steroidal MRAs	Alpha-glucosidase inhibitors	0.11 (0.01 to 1.32)	368 per 1000 persons	58 per 1000 persons (5 to 434 per 1000 persons)	310 fewer (363 fewer to 66 more)	Very low
**Non-fatal stroke**	SGLT2 inhibitors	Alpha-glucosidase inhibitors	0.11 (0.01 to 1.30)	368 per 1000 persons	58 per 1000 persons (5 to 431 per 1000 persons)	310 fewer (363 fewer to 63 more)	Very low
**Non-fatal stroke**	Standard treatments	Alpha-glucosidase inhibitors	0.11 (0.01 to 1.32)	368 per 1000 persons	58 per 1000 persons (5 to 434 per 1000 persons)	310 fewer (363 fewer to 66 more)	Very low
**Non-fatal stroke**	Sulfonylureas	Alpha-glucosidase inhibitors	0.11 (0.01 to 1.37)	368 per 1000 persons	61 per 1000 persons (5 to 444 per 1000 persons)	307 fewer (363 fewer to 76 more)	Very low
**Non-fatal stroke**	Thiazolidinediones	Alpha-glucosidase inhibitors	0.09 (0.01 to 1.11)	368 per 1000 persons	50 per 1000 persons (4 to 393 per 1000 persons)	318 fewer (364 fewer to 25 more)	Very low
**Non-fatal stroke**	Basal-Bolus insulin	Basal insulin	0.76 (0.13 to 4.35)	45 per 1000 persons	34 per 1000 persons (6 to 170 per 1000 persons)	11 fewer (39 fewer to 125 more)	Very low
**Non-fatal stroke**	Bolus insulin	Basal insulin	1.12 (0.18 to 7.14)	45 per 1000 persons	50 per 1000 persons (8 to 252 per 1000 persons)	5 more (37 fewer to 207 more)	Very low
**Non-fatal stroke**	DPP-4 inhibitors	Basal insulin	1.19 (0.51 to 2.78)	45 per 1000 persons	53 per 1000 persons (23 to 116 per 1000 persons)	8 more (22 fewer to 71 more)	Very low
**Non-fatal stroke**	GLP-1 receptor agonists	Basal insulin	1.12 (0.48 to 2.63)	45 per 1000 persons	50 per 1000 persons (22 to 110 per 1000 persons)	5 more (23 fewer to 65 more)	Very low
**Non-fatal stroke**	Meglitinides	Basal insulin	2.22 (0.28 to 16.67)	45 per 1000 persons	95 per 1000 persons (13 to 440 per 1000 persons)	50 more (32 fewer to 395 more)	Very low
**Non-fatal stroke**	Metformin	Basal insulin	1.28 (0.52 to 3.12)	45 per 1000 persons	57 per 1000 persons (24 to 128 per 1000 persons)	12 more (21 fewer to 83 more)	Very low
**Non-fatal stroke**	Non-steroidal MRAs	Basal insulin	1.32 (0.55 to 3.12)	45 per 1000 persons	58 per 1000 persons (25 to 128 per 1000 persons)	13 more (20 fewer to 83 more)	Very low
**Non-fatal stroke**	SGLT2 inhibitors	Basal insulin	1.30 (0.56 to 3.03)	45 per 1000 persons	58 per 1000 persons (26 to 125 per 1000 persons)	13 more (19 fewer to 80 more)	Very low

Outcome	Intervention	Comparator	Odds ratio	Baseline risk	Risk if treated	Risk difference	Certainty
**Non-fatal stroke**	Standard treatments	Basal insulin	1.32 (0.56 to 3.03)	45 per 1000 persons	58 per 1000 persons (26 to 125 per 1000 persons)	13 more (19 fewer to 80 more)	Very low
**Non-fatal stroke**	Sulfonylureas	Basal insulin	1.39 (0.58 to 3.33)	45 per 1000 persons	61 per 1000 persons (27 to 136 per 1000 persons)	16 more (18 fewer to 91 more)	Very low
**Non-fatal stroke**	Thiazolidinediones	Basal insulin	1.11 (0.47 to 2.63)	45 per 1000 persons	50 per 1000 persons (22 to 110 per 1000 persons)	5 more (23 fewer to 65 more)	Very low
**Non-fatal stroke**	Bolus insulin	Basal-Bolus insulin	1.49 (0.13 to 16.67)	34 per 1000 persons	50 per 1000 persons (5 to 370 per 1000 persons)	16 more (29 fewer to 336 more)	Very low
**Non-fatal stroke**	DPP-4 inhibitors	Basal-Bolus insulin	1.56 (0.27 to 9.09)	34 per 1000 persons	52 per 1000 persons (9 to 242 per 1000 persons)	18 more (25 fewer to 208 more)	Very low
**Non-fatal stroke**	GLP-1 receptor agonists	Basal-Bolus insulin	1.47 (0.26 to 8.33)	34 per 1000 persons	49 per 1000 persons (9 to 227 per 1000 persons)	15 more (25 fewer to 193 more)	Very low
**Non-fatal stroke**	Meglitinides	Basal-Bolus insulin	2.94 (0.22 to 33.33)	34 per 1000 persons	94 per 1000 persons (8 to 540 per 1000 persons)	60 more (26 fewer to 506 more)	Very low
**Non-fatal stroke**	Metformin	Basal-Bolus insulin	1.67 (0.28 to 10.00)	34 per 1000 persons	55 per 1000 persons (10 to 260 per 1000 persons)	21 more (24 fewer to 226 more)	Very low
**Non-fatal stroke**	Non-steroidal MRAs	Basal-Bolus insulin	1.72 (0.29 to 10.00)	34 per 1000 persons	57 per 1000 persons (10 to 260 per 1000 persons)	23 more (24 fewer to 226 more)	Very low
**Non-fatal stroke**	SGLT2 inhibitors	Basal-Bolus insulin	1.72 (0.29 to 10.00)	34 per 1000 persons	57 per 1000 persons (10 to 260 per 1000 persons)	23 more (24 fewer to 226 more)	Very low
**Non-fatal stroke**	Standard treatments	Basal-Bolus insulin	1.72 (0.30 to 10.00)	34 per 1000 persons	57 per 1000 persons (10 to 260 per 1000 persons)	23 more (24 fewer to 226 more)	Very low
**Non-fatal stroke**	Sulfonylureas	Basal-Bolus insulin	1.82 (0.31 to 11.11)	34 per 1000 persons	60 per 1000 persons (11 to 281 per 1000 persons)	26 more (23 fewer to 247 more)	Very low
**Non-fatal stroke**	Thiazolidinediones	Basal-Bolus insulin	1.47 (0.25 to 8.33)	34 per 1000 persons	49 per 1000 persons (9 to 227 per 1000 persons)	15 more (25 fewer to 193 more)	Very low
**Non-fatal stroke**	DPP-4 inhibitors	Bolus insulin	1.05 (0.20 to 5.56)	50 per 1000 persons	52 per 1000 persons (10 to 226 per 1000 persons)	2 more (40 fewer to 176 more)	Very low
**Non-fatal stroke**	GLP-1 receptor agonists	Bolus insulin	0.99 (0.19 to 5.26)	50 per 1000 persons	50 per 1000 persons (10 to 217 per 1000 persons)	0 (40 fewer to 167 more)	Very low

Outcome	Intervention	Comparator	Odds ratio	Baseline risk	Risk if treated	Risk difference	Certainty
**Non-fatal stroke**	Meglitinides	Bolus insulin	2.00 (0.16 to 25.00)	50 per 1000 persons	95 per 1000 persons (8 to 568 per 1000 persons)	45 more (42 fewer to 518 more)	Very low
**Non-fatal stroke**	Metformin	Bolus insulin	1.14 (0.21 to 6.25)	50 per 1000 persons	56 per 1000 persons (11 to 248 per 1000 persons)	6 more (39 fewer to 198 more)	Very low
**Non-fatal stroke**	Non-steroidal MRAs	Bolus insulin	1.16 (0.22 to 6.25)	50 per 1000 persons	58 per 1000 persons (11 to 248 per 1000 persons)	8 more (39 fewer to 198 more)	Very low
**Non-fatal stroke**	SGLT2 inhibitors	Bolus insulin	1.15 (0.22 to 6.25)	50 per 1000 persons	57 per 1000 persons (11 to 248 per 1000 persons)	7 more (39 fewer to 198 more)	Very low
**Non-fatal stroke**	Standard treatments	Bolus insulin	1.16 (0.22 to 6.25)	50 per 1000 persons	58 per 1000 persons (12 to 248 per 1000 persons)	8 more (38 fewer to 198 more)	Very low
**Non-fatal stroke**	Sulfonylureas	Bolus insulin	1.22 (0.23 to 6.67)	50 per 1000 persons	60 per 1000 persons (12 to 260 per 1000 persons)	10 more (38 fewer to 210 more)	Very low
**Non-fatal stroke**	Thiazolidinediones	Bolus insulin	0.99 (0.19 to 5.26)	50 per 1000 persons	50 per 1000 persons (10 to 217 per 1000 persons)	0 (40 fewer to 167 more)	Very low
**Non-fatal stroke**	GLP-1 receptor agonists	DPP-4 inhibitors	0.94 (0.80 to 1.10)	53 per 1000 persons	50 per 1000 persons (43 to 58 per 1000 persons)	3 fewer (10 fewer to 5 more)	Moderate
**Non-fatal stroke**	Meglitinides	DPP-4 inhibitors	1.89 (0.28 to 12.50)	53 per 1000 persons	96 per 1000 persons (16 to 412 per 1000 persons)	43 more (37 fewer to 359 more)	Very low
**Non-fatal stroke**	Metformin	DPP-4 inhibitors	1.08 (0.78 to 1.49)	53 per 1000 persons	57 per 1000 persons (42 to 77 per 1000 persons)	4 more (11 fewer to 24 more)	Very low
**Non-fatal stroke**	Non-steroidal MRAs	DPP-4 inhibitors	1.10 (0.87 to 1.39)	53 per 1000 persons	58 per 1000 persons (46 to 72 per 1000 persons)	5 more (7 fewer to 19 more)	Low
**Non-fatal stroke**	SGLT2 inhibitors	DPP-4 inhibitors	1.10 (0.93 to 1.30)	53 per 1000 persons	58 per 1000 persons (49 to 68 per 1000 persons)	5 more (4 fewer to 15 more)	Low
**Non-fatal stroke**	Standard treatments	DPP-4 inhibitors	1.10 (0.97 to 1.25)	53 per 1000 persons	58 per 1000 persons (52 to 65 per 1000 persons)	5 more (1 fewer to 12 more)	Low
**Non-fatal stroke**	Sulfonylureas	DPP-4 inhibitors	1.16 (0.93 to 1.45)	53 per 1000 persons	61 per 1000 persons (50 to 75 per 1000 persons)	8 more (3 fewer to 22 more)	Moderate
**Non-fatal stroke**	Thiazolidinediones	DPP-4 inhibitors	0.93 (0.75 to 1.18)	53 per 1000 persons	50 per 1000 persons (40 to 62 per 1000 persons)	3 fewer (13 fewer to 9 more)	Very low

Outcome	Intervention	Comparator	Odds ratio	Baseline risk	Risk if treated	Risk difference	Certainty
**Non-fatal stroke**	Meglitinides	GLP-1 receptor agonists	2.00 (0.30 to 14.29)	50 per 1000 persons	95 per 1000 persons (15 to 429 per 1000 persons)	45 more (35 fewer to 379 more)	Very low
**Non-fatal stroke**	Metformin	GLP-1 receptor agonists	1.14 (0.82 to 1.59)	50 per 1000 persons	56 per 1000 persons (41 to 77 per 1000 persons)	6 more (9 fewer to 27 more)	Low
**Non-fatal stroke**	Non-steroidal MRAs	GLP-1 receptor agonists	1.18 (0.93 to 1.47)	50 per 1000 persons	58 per 1000 persons (47 to 72 per 1000 persons)	8 more (3 fewer to 22 more)	Low
**Non-fatal stroke**	SGLT2 inhibitors	GLP-1 receptor agonists	1.16 (1.00 to 1.35)	50 per 1000 persons	58 per 1000 persons (50 to 66 per 1000 persons)	8 more (0 to 16 more)	Low
**Non-fatal stroke**	Standard treatments	GLP-1 receptor agonists	1.18 (1.06 to 1.30)	50 per 1000 persons	58 per 1000 persons (53 to 64 per 1000 persons)	8 more (3 more to 14 more)	Moderate
**Non-fatal stroke**	Sulfonylureas	GLP-1 receptor agonists	1.23 (0.96 to 1.59)	50 per 1000 persons	61 per 1000 persons (48 to 77 per 1000 persons)	11 more (2 fewer to 27 more)	Low
**Non-fatal stroke**	Thiazolidinediones	GLP-1 receptor agonists	1.00 (0.80 to 1.25)	50 per 1000 persons	50 per 1000 persons (40 to 62 per 1000 persons)	0 (10 fewer to 12 more)	Low
**Non-fatal stroke**	Metformin	Meglitinides	0.57 (0.08 to 3.85)	95 per 1000 persons	56 per 1000 persons (9 to 288 per 1000 persons)	39 fewer (86 fewer to 193 more)	Very low
**Non-fatal stroke**	Non-steroidal MRAs	Meglitinides	0.58 (0.09 to 4.00)	95 per 1000 persons	58 per 1000 persons (9 to 296 per 1000 persons)	37 fewer (86 fewer to 201 more)	Very low
**Non-fatal stroke**	SGLT2 inhibitors	Meglitinides	0.58 (0.09 to 3.85)	95 per 1000 persons	58 per 1000 persons (9 to 288 per 1000 persons)	37 fewer (86 fewer to 193 more)	Very low
**Non-fatal stroke**	Standard treatments	Meglitinides	0.58 (0.09 to 3.85)	95 per 1000 persons	58 per 1000 persons (9 to 288 per 1000 persons)	37 fewer (86 fewer to 193 more)	Very low
**Non-fatal stroke**	Sulfonylureas	Meglitinides	0.62 (0.09 to 4.17)	95 per 1000 persons	61 per 1000 persons (10 to 304 per 1000 persons)	34 fewer (85 fewer to 209 more)	Very low
**Non-fatal stroke**	Thiazolidinediones	Meglitinides	0.50 (0.07 to 3.33)	95 per 1000 persons	50 per 1000 persons (8 to 259 per 1000 persons)	45 fewer (87 fewer to 164 more)	Very low
**Non-fatal stroke**	Non-steroidal MRAs	Metformin	1.03 (0.71 to 1.49)	56 per 1000 persons	58 per 1000 persons (40 to 81 per 1000 persons)	2 more (16 fewer to 25 more)	Very low
**Non-fatal stroke**	SGLT2 inhibitors	Metformin	1.02 (0.73 to 1.43)	56 per 1000 persons	57 per 1000 persons (42 to 78 per 1000 persons)	1 more (14 fewer to 22 more)	Very low

Outcome	Intervention	Comparator	Odds ratio	Baseline risk	Risk if treated	Risk difference	Certainty
**Non-fatal stroke**	Standard treatments	Metformin	1.03 (0.75 to 1.41)	56 per 1000 persons	58 per 1000 persons (43 to 77 per 1000 persons)	2 more (13 fewer to 21 more)	Very low
**Non-fatal stroke**	Sulfonylureas	Metformin	1.09 (0.77 to 1.52)	56 per 1000 persons	61 per 1000 persons (44 to 82 per 1000 persons)	5 more (12 fewer to 26 more)	Very low
**Non-fatal stroke**	Thiazolidinediones	Metformin	0.88 (0.62 to 1.23)	56 per 1000 persons	49 per 1000 persons (36 to 68 per 1000 persons)	7 fewer (20 fewer to 12 more)	Very low
**Non-fatal stroke**	SGLT2 inhibitors	Non-steroidal MRAs	0.99 (0.79 to 1.25)	58 per 1000 persons	57 per 1000 persons (46 to 71 per 1000 persons)	1 fewer (12 fewer to 13 more)	Very low
**Non-fatal stroke**	Standard treatments	Non-steroidal MRAs	1.00 (0.82 to 1.22)	58 per 1000 persons	58 per 1000 persons (48 to 70 per 1000 persons)	0 (10 fewer to 12 more)	Low
**Non-fatal stroke**	Sulfonylureas	Non-steroidal MRAs	1.05 (0.78 to 1.43)	58 per 1000 persons	61 per 1000 persons (46 to 81 per 1000 persons)	3 more (12 fewer to 23 more)	Very low
**Non-fatal stroke**	Thiazolidinediones	Non-steroidal MRAs	0.85 (0.64 to 1.12)	58 per 1000 persons	50 per 1000 persons (38 to 65 per 1000 persons)	8 fewer (20 fewer to 7 more)	Very low
**Non-fatal stroke**	Standard treatments	SGLT2 inhibitors	1.01 (0.90 to 1.14)	57 per 1000 persons	58 per 1000 persons (52 to 64 per 1000 persons)	1 more (5 fewer to 7 more)	High
**Non-fatal stroke**	Sulfonylureas	SGLT2 inhibitors	1.06 (0.83 to 1.37)	57 per 1000 persons	60 per 1000 persons (48 to 76 per 1000 persons)	3 more (9 fewer to 19 more)	Low
**Non-fatal stroke**	Thiazolidinediones	SGLT2 inhibitors	0.85 (0.68 to 1.08)	57 per 1000 persons	49 per 1000 persons (39 to 61 per 1000 persons)	8 fewer (18 fewer to 4 more)	Low
**Non-fatal stroke**	Standard treatments	Sulfonylureas	0.95 (0.76 to 1.19)	61 per 1000 persons	58 per 1000 persons (47 to 72 per 1000 persons)	3 fewer (14 fewer to 11 more)	Very low
**Non-fatal stroke**	Thiazolidinediones	Sulfonylureas	0.81 (0.62 to 1.04)	61 per 1000 persons	50 per 1000 persons (39 to 63 per 1000 persons)	11 fewer (22 fewer to 2 more)	Low
**Non-fatal stroke**	Standard treatments	Thiazolidinediones	1.18 (0.97 to 1.43)	50 per 1000 persons	58 per 1000 persons (49 to 70 per 1000 persons)	8 more (1 fewer to 20 more)	Very low
**Hospitalisation for heart failure**	Alpha-glucosidase inhibitors	Basal insulin	3.47 (0.13 to 90.34)	28 per 1000 persons	91 per 1000 persons (4 to 722 per 1000 persons)	63 more (24 fewer to 694 more)	Very low
**Hospitalisation for heart failure**	Alpha-glucosidase inhibitors	Bolus insulin	5.05 (0.10 to 262.01)	19 per 1000 persons	89 per 1000 persons (2 to 835 per 1000 persons)	70 more (17 fewer to 816 more)	Very low

Outcome	Intervention	Comparator	Odds ratio	Baseline risk	Risk if treated	Risk difference	Certainty
**Hospitalisation for heart failure**	Alpha-glucosidase inhibitors	DPP-4 inhibitors	3.09 (0.12 to 78.44)	31 per 1000 persons	90 per 1000 persons (4 to 715 per 1000 persons)	59 more (27 fewer to 684 more)	Very low
**Hospitalisation for heart failure**	Alpha-glucosidase inhibitors	GLP-1 receptor agonists	3.59 (0.14 to 91.17)	27 per 1000 persons	91 per 1000 persons (4 to 717 per 1000 persons)	64 more (23 fewer to 690 more)	Very low
**Hospitalisation for heart failure**	Alpha-glucosidase inhibitors	Metformin	2.25 (0.06 to 84.00)	43 per 1000 persons	92 per 1000 persons (3 to 791 per 1000 persons)	49 more (40 fewer to 748 more)	Very low
**Hospitalisation for heart failure**	Alpha-glucosidase inhibitors	Non-steroidal MRAs	4.18 (0.16 to 106.46)	24 per 1000 persons	93 per 1000 persons (4 to 724 per 1000 persons)	69 more (20 fewer to 700 more)	Very low
**Hospitalisation for heart failure**	Alpha-glucosidase inhibitors	SGLT2 inhibitors	4.93 (0.19 to 125.08)	20 per 1000 persons	91 per 1000 persons (4 to 719 per 1000 persons)	71 more (16 fewer to 699 more)	Very low
**Hospitalisation for heart failure**	Alpha-glucosidase inhibitors	Standard treatments	3.25 (0.13 to 82.49)	30 per 1000 persons	91 per 1000 persons (4 to 718 per 1000 persons)	61 more (26 fewer to 688 more)	Very low
**Hospitalisation for heart failure**	Alpha-glucosidase inhibitors	Sulfonylureas	3.30 (0.13 to 84.25)	30 per 1000 persons	93 per 1000 persons (4 to 723 per 1000 persons)	63 more (26 fewer to 693 more)	Very low
**Hospitalisation for heart failure**	Alpha-glucosidase inhibitors	Thiazolidinediones	2.11 (0.08 to 53.77)	45 per 1000 persons	90 per 1000 persons (4 to 717 per 1000 persons)	45 more (41 fewer to 672 more)	Very low
**Hospitalisation for heart failure**	Alpha-glucosidase inhibitors	Tirzepatide	5.18 (0.16 to 171.40)	19 per 1000 persons	91 per 1000 persons (3 to 769 per 1000 persons)	72 more (16 fewer to 750 more)	Very low
**Hospitalisation for heart failure**	Basal insulin	Bolus insulin	1.46 (0.15 to 14.61)	19 per 1000 persons	27 per 1000 persons (3 to 221 per 1000 persons)	8 more (16 fewer to 202 more)	Very low
**Hospitalisation for heart failure**	Basal insulin	DPP-4 inhibitors	0.89 (0.59 to 1.36)	31 per 1000 persons	28 per 1000 persons (19 to 42 per 1000 persons)	3 fewer (12 fewer to 11 more)	Low
**Hospitalisation for heart failure**	Basal insulin	GLP-1 receptor agonists	1.04 (0.68 to 1.58)	27 per 1000 persons	28 per 1000 persons (19 to 42 per 1000 persons)	1 more (8 fewer to 15 more)	Low
**Hospitalisation for heart failure**	Basal insulin	Metformin	0.65 (0.12 to 3.48)	43 per 1000 persons	28 per 1000 persons (5 to 135 per 1000 persons)	15 fewer (38 fewer to 92 more)	Very low
**Hospitalisation for heart failure**	Basal insulin	Non-steroidal MRAs	1.21 (0.77 to 1.90)	24 per 1000 persons	29 per 1000 persons (19 to 45 per 1000 persons)	5 more (5 fewer to 21 more)	Low
**Hospitalisation for heart failure**	Basal insulin	SGLT2 inhibitors	1.42 (0.92 to 2.19)	20 per 1000 persons	28 per 1000 persons (18 to 43 per 1000 persons)	8 more (2 fewer to 23 more)	Low

Outcome	Intervention	Comparator	Odds ratio	Baseline risk	Risk if treated	Risk difference	Certainty
**Hospitalisation for heart failure**	Basal insulin	Standard treatments	0.94 (0.62 to 1.43)	30 per 1000 persons	28 per 1000 persons (19 to 42 per 1000 persons)	2 fewer (11 fewer to 12 more)	Very low
**Hospitalisation for heart failure**	Basal insulin	Sulfonylureas	0.95 (0.62 to 1.47)	30 per 1000 persons	29 per 1000 persons (19 to 43 per 1000 persons)	1 fewer (11 fewer to 13 more)	Low
**Hospitalisation for heart failure**	Basal insulin	Thiazolidinediones	0.61 (0.38 to 0.96)	45 per 1000 persons	28 per 1000 persons (18 to 43 per 1000 persons)	17 fewer (27 fewer to 2 fewer)	Moderate
**Hospitalisation for heart failure**	Basal insulin	Tirzepatide	1.50 (0.42 to 5.32)	19 per 1000 persons	28 per 1000 persons (8 to 93 per 1000 persons)	9 more (11 fewer to 74 more)	Low
**Hospitalisation for heart failure**	Bolus insulin	DPP-4 inhibitors	0.61 (0.06 to 5.92)	31 per 1000 persons	19 per 1000 persons (2 to 159 per 1000 persons)	12 fewer (29 fewer to 128 more)	Very low
**Hospitalisation for heart failure**	Bolus insulin	GLP-1 receptor agonists	0.71 (0.07 to 6.85)	27 per 1000 persons	19 per 1000 persons (2 to 160 per 1000 persons)	8 fewer (25 fewer to 133 more)	Very low
**Hospitalisation for heart failure**	Bolus insulin	Metformin	0.45 (0.03 to 7.25)	43 per 1000 persons	20 per 1000 persons (1 to 246 per 1000 persons)	23 fewer (42 fewer to 203 more)	Very low
**Hospitalisation for heart failure**	Bolus insulin	Non-steroidal MRAs	0.83 (0.09 to 8.04)	24 per 1000 persons	20 per 1000 persons (2 to 165 per 1000 persons)	4 fewer (22 fewer to 141 more)	Very low
**Hospitalisation for heart failure**	Bolus insulin	SGLT2 inhibitors	0.98 (0.10 to 9.43)	20 per 1000 persons	20 per 1000 persons (2 to 161 per 1000 persons)	0 (18 fewer to 141 more)	Very low
**Hospitalisation for heart failure**	Bolus insulin	Standard treatments	0.64 (0.07 to 6.22)	30 per 1000 persons	19 per 1000 persons (2 to 161 per 1000 persons)	11 fewer (28 fewer to 131 more)	Very low
**Hospitalisation for heart failure**	Bolus insulin	Sulfonylureas	0.65 (0.07 to 6.37)	30 per 1000 persons	20 per 1000 persons (2 to 165 per 1000 persons)	10 fewer (28 fewer to 135 more)	Very low
**Hospitalisation for heart failure**	Bolus insulin	Thiazolidinediones	0.42 (0.04 to 4.06)	45 per 1000 persons	19 per 1000 persons (2 to 161 per 1000 persons)	26 fewer (43 fewer to 116 more)	Very low
**Hospitalisation for heart failure**	Bolus insulin	Tirzepatide	1.03 (0.07 to 14.25)	19 per 1000 persons	20 per 1000 persons (1 to 216 per 1000 persons)	1 more (18 fewer to 197 more)	Very low
**Hospitalisation for heart failure**	DPP-4 inhibitors	GLP-1 receptor agonists	1.16 (1.02 to 1.33)	27 per 1000 persons	31 per 1000 persons (28 to 36 per 1000 persons)	4 more (1 more to 9 more)	Moderate
**Hospitalisation for heart failure**	DPP-4 inhibitors	Metformin	0.73 (0.14 to 3.72)	43 per 1000 persons	32 per 1000 persons (6 to 143 per 1000 persons)	11 fewer (37 fewer to 100 more)	Very low

Outcome	Intervention	Comparator	Odds ratio	Baseline risk	Risk if treated	Risk difference	Certainty
**Hospitalisation for heart failure**	DPP-4 inhibitors	Non-steroidal MRAs	1.35 (1.11 to 1.65)	24 per 1000 persons	32 per 1000 persons (27 to 39 per 1000 persons)	8 more (3 more to 15 more)	Low
**Hospitalisation for heart failure**	DPP-4 inhibitors	SGLT2 inhibitors	1.59 (1.39 to 1.83)	20 per 1000 persons	31 per 1000 persons (28 to 36 per 1000 persons)	11 more (8 more to 16 more)	Moderate
**Hospitalisation for heart failure**	DPP-4 inhibitors	Standard treatments	1.05 (0.95 to 1.16)	30 per 1000 persons	31 per 1000 persons (29 to 35 per 1000 persons)	1 more (1 fewer to 5 more)	Moderate
**Hospitalisation for heart failure**	DPP-4 inhibitors	Sulfonylureas	1.07 (0.86 to 1.32)	30 per 1000 persons	32 per 1000 persons (26 to 39 per 1000 persons)	2 more (4 fewer to 9 more)	High
**Hospitalisation for heart failure**	DPP-4 inhibitors	Thiazolidinediones	0.68 (0.55 to 0.85)	45 per 1000 persons	31 per 1000 persons (25 to 39 per 1000 persons)	14 fewer (20 fewer to 6 fewer)	Moderate
**Hospitalisation for heart failure**	DPP-4 inhibitors	Tirzepatide	1.68 (0.44 to 6.38)	19 per 1000 persons	32 per 1000 persons (8 to 110 per 1000 persons)	13 more (11 fewer to 91 more)	Very low
**Hospitalisation for heart failure**	GLP-1 receptor agonists	Metformin	0.63 (0.12 to 3.19)	43 per 1000 persons	28 per 1000 persons (5 to 125 per 1000 persons)	15 fewer (38 fewer to 82 more)	Low
**Hospitalisation for heart failure**	GLP-1 receptor agonists	Non-steroidal MRAs	1.16 (0.96 to 1.41)	24 per 1000 persons	28 per 1000 persons (23 to 34 per 1000 persons)	4 more (1 fewer to 10 more)	Moderate
**Hospitalisation for heart failure**	GLP-1 receptor agonists	SGLT2 inhibitors	1.37 (1.20 to 1.56)	20 per 1000 persons	27 per 1000 persons (24 to 31 per 1000 persons)	7 more (4 more to 11 more)	Low
**Hospitalisation for heart failure**	GLP-1 receptor agonists	Standard treatments	0.91 (0.83 to 0.99)	30 per 1000 persons	27 per 1000 persons (25 to 30 per 1000 persons)	3 fewer (5 fewer to 0)	Moderate
**Hospitalisation for heart failure**	GLP-1 receptor agonists	Sulfonylureas	0.92 (0.72 to 1.16)	30 per 1000 persons	28 per 1000 persons (22 to 35 per 1000 persons)	2 fewer (8 fewer to 5 more)	Moderate
**Hospitalisation for heart failure**	GLP-1 receptor agonists	Thiazolidinediones	0.59 (0.47 to 0.73)	45 per 1000 persons	27 per 1000 persons (22 to 33 per 1000 persons)	18 fewer (23 fewer to 12 fewer)	Moderate
**Hospitalisation for heart failure**	GLP-1 receptor agonists	Tirzepatide	1.44 (0.38 to 5.50)	19 per 1000 persons	27 per 1000 persons (7 to 96 per 1000 persons)	8 more (12 fewer to 77 more)	Very low
**Hospitalisation for heart failure**	Metformin	Non-steroidal MRAs	1.86 (0.36 to 9.54)	24 per 1000 persons	44 per 1000 persons (9 to 190 per 1000 persons)	20 more (15 fewer to 166 more)	Very low
**Hospitalisation for heart failure**	Metformin	SGLT2 inhibitors	2.19 (0.43 to 11.16)	20 per 1000 persons	43 per 1000 persons (9 to 186 per 1000 persons)	23 more (11 fewer to 166 more)	Very low

Outcome	Intervention	Comparator	Odds ratio	Baseline risk	Risk if treated	Risk difference	Certainty
**Hospitalisation for heart failure**	Metformin	Standard treatments	1.45 (0.28 to 7.36)	30 per 1000 persons	43 per 1000 persons (9 to 185 per 1000 persons)	13 more (21 fewer to 155 more)	Very low
**Hospitalisation for heart failure**	Metformin	Sulfonylureas	1.47 (0.28 to 7.57)	30 per 1000 persons	43 per 1000 persons (9 to 190 per 1000 persons)	13 more (21 fewer to 160 more)	Very low
**Hospitalisation for heart failure**	Metformin	Thiazolidinediones	0.94 (0.18 to 4.81)	45 per 1000 persons	42 per 1000 persons (8 to 185 per 1000 persons)	3 fewer (37 fewer to 140 more)	Very low
**Hospitalisation for heart failure**	Metformin	Tirzepatide	2.30 (0.28 to 18.91)	19 per 1000 persons	43 per 1000 persons (5 to 268 per 1000 persons)	24 more (14 fewer to 249 more)	Very low
**Hospitalisation for heart failure**	Non-steroidal MRAs	SGLT2 inhibitors	1.18 (0.97 to 1.43)	20 per 1000 persons	24 per 1000 persons (19 to 28 per 1000 persons)	4 more (1 fewer to 8 more)	Moderate
**Hospitalisation for heart failure**	Non-steroidal MRAs	Standard treatments	0.78 (0.66 to 0.92)	30 per 1000 persons	24 per 1000 persons (20 to 28 per 1000 persons)	6 fewer (10 fewer to 2 fewer)	Moderate
**Hospitalisation for heart failure**	Non-steroidal MRAs	Sulfonylureas	0.79 (0.60 to 1.04)	30 per 1000 persons	24 per 1000 persons (18 to 31 per 1000 persons)	6 fewer (12 fewer to 1 more)	Low
**Hospitalisation for heart failure**	Non-steroidal MRAs	Thiazolidinediones	0.50 (0.39 to 0.65)	45 per 1000 persons	23 per 1000 persons (18 to 30 per 1000 persons)	22 fewer (27 fewer to 15 fewer)	Moderate
**Hospitalisation for heart failure**	Non-steroidal MRAs	Tirzepatide	1.24 (0.32 to 4.77)	19 per 1000 persons	23 per 1000 persons (6 to 85 per 1000 persons)	4 more (13 fewer to 66 more)	Very low
**Hospitalisation for heart failure**	SGLT2 inhibitors	Standard treatments	0.66 (0.60 to 0.73)	30 per 1000 persons	20 per 1000 persons (18 to 22 per 1000 persons)	10 fewer (12 fewer to 8 fewer)	High
**Hospitalisation for heart failure**	SGLT2 inhibitors	Sulfonylureas	0.67 (0.53 to 0.85)	30 per 1000 persons	20 per 1000 persons (16 to 26 per 1000 persons)	10 fewer (14 fewer to 4 fewer)	Moderate
**Hospitalisation for heart failure**	SGLT2 inhibitors	Thiazolidinediones	0.43 (0.34 to 0.53)	45 per 1000 persons	20 per 1000 persons (16 to 24 per 1000 persons)	25 fewer (29 fewer to 21 fewer)	Moderate
**Hospitalisation for heart failure**	SGLT2 inhibitors	Tirzepatide	1.05 (0.28 to 4.02)	19 per 1000 persons	20 per 1000 persons (5 to 72 per 1000 persons)	1 more (14 fewer to 53 more)	Very low
**Hospitalisation for heart failure**	Sulfonylureas	Standard treatments	0.99 (0.79 to 1.23)	30 per 1000 persons	30 per 1000 persons (24 to 37 per 1000 persons)	0 (6 fewer to 7 more)	Moderate
**Hospitalisation for heart failure**	Sulfonylureas	Thiazolidinediones	0.64 (0.48 to 0.85)	45 per 1000 persons	29 per 1000 persons (22 to 39 per 1000 persons)	16 fewer (23 fewer to 6 fewer)	Moderate

Outcome	Intervention	Comparator	Odds ratio	Baseline risk	Risk if treated	Risk difference	Certainty
**Hospitalisation for heart failure**	Sulfonylureas	Tirzepatide	1.57 (0.41 to 6.01)	19 per 1000 persons	30 per 1000 persons (8 to 104 per 1000 persons)	11 more (11 fewer to 85 more)	Very low
**Hospitalisation for heart failure**	Thiazolidinediones	Standard treatments	1.54 (1.27 to 1.88)	30 per 1000 persons	45 per 1000 persons (38 to 55 per 1000 persons)	15 more (8 more to 25 more)	Moderate
**Hospitalisation for heart failure**	Thiazolidinediones	Tirzepatide	2.46 (0.64 to 9.48)	19 per 1000 persons	45 per 1000 persons (12 to 155 per 1000 persons)	26 more (7 fewer to 136 more)	Low
**Hospitalisation for heart failure**	Tirzepatide	Standard treatments	0.63 (0.16 to 2.39)	30 per 1000 persons	19 per 1000 persons (5 to 69 per 1000 persons)	11 fewer (25 fewer to 39 more)	Very low
**Hospitalisation for heart failure**	Basal insulin	Alpha-glucosidase inhibitors	0.29 (0.01 to 7.69)	91 per 1000 persons	28 per 1000 persons (1 to 435 per 1000 persons)	63 fewer (90 fewer to 344 more)	Very low
**Hospitalisation for heart failure**	Bolus insulin	Alpha-glucosidase inhibitors	0.20 (0.00 to 10.00)	91 per 1000 persons	19 per 1000 persons (0 to 500 per 1000 persons)	72 fewer (91 fewer to 409 more)	Very low
**Hospitalisation for heart failure**	DPP-4 inhibitors	Alpha-glucosidase inhibitors	0.32 (0.01 to 8.33)	91 per 1000 persons	31 per 1000 persons (1 to 455 per 1000 persons)	60 fewer (90 fewer to 364 more)	Very low
**Hospitalisation for heart failure**	GLP-1 receptor agonists	Alpha-glucosidase inhibitors	0.28 (0.01 to 7.14)	91 per 1000 persons	27 per 1000 persons (1 to 417 per 1000 persons)	64 fewer (90 fewer to 326 more)	Very low
**Hospitalisation for heart failure**	Metformin	Alpha-glucosidase inhibitors	0.44 (0.01 to 16.67)	91 per 1000 persons	43 per 1000 persons (1 to 625 per 1000 persons)	48 fewer (90 fewer to 534 more)	Very low
**Hospitalisation for heart failure**	Non-steroidal MRAs	Alpha-glucosidase inhibitors	0.24 (0.01 to 6.25)	91 per 1000 persons	23 per 1000 persons (1 to 385 per 1000 persons)	68 fewer (90 fewer to 294 more)	Very low
**Hospitalisation for heart failure**	SGLT2 inhibitors	Alpha-glucosidase inhibitors	0.20 (0.01 to 5.26)	91 per 1000 persons	20 per 1000 persons (1 to 345 per 1000 persons)	71 fewer (90 fewer to 254 more)	Very low
**Hospitalisation for heart failure**	Standard treatments	Alpha-glucosidase inhibitors	0.31 (0.01 to 7.69)	91 per 1000 persons	30 per 1000 persons (1 to 435 per 1000 persons)	61 fewer (90 fewer to 344 more)	Very low
**Hospitalisation for heart failure**	Sulfonylureas	Alpha-glucosidase inhibitors	0.30 (0.01 to 7.69)	91 per 1000 persons	29 per 1000 persons (1 to 435 per 1000 persons)	62 fewer (90 fewer to 344 more)	Very low
**Hospitalisation for heart failure**	Thiazolidinediones	Alpha-glucosidase inhibitors	0.47 (0.02 to 12.50)	91 per 1000 persons	45 per 1000 persons (2 to 556 per 1000 persons)	46 fewer (89 fewer to 465 more)	Very low
**Hospitalisation for heart failure**	Tirzepatide	Alpha-glucosidase inhibitors	0.19 (0.01 to 6.25)	91 per 1000 persons	19 per 1000 persons (1 to 385 per 1000 persons)	72 fewer (90 fewer to 294 more)	Very low

Outcome	Intervention	Comparator	Odds ratio	Baseline risk	Risk if treated	Risk difference	Certainty
**Hospitalisation for heart failure**	Bolus insulin	Basal insulin	0.68 (0.07 to 6.67)	28 per 1000 persons	19 per 1000 persons (2 to 161 per 1000 persons)	9 fewer (26 fewer to 133 more)	Very low
**Hospitalisation for heart failure**	DPP-4 inhibitors	Basal insulin	1.12 (0.74 to 1.69)	28 per 1000 persons	31 per 1000 persons (21 to 47 per 1000 persons)	3 more (7 fewer to 19 more)	Moderate
**Hospitalisation for heart failure**	GLP-1 receptor agonists	Basal insulin	0.96 (0.63 to 1.47)	28 per 1000 persons	27 per 1000 persons (18 to 41 per 1000 persons)	1 fewer (10 fewer to 13 more)	Low
**Hospitalisation for heart failure**	Metformin	Basal insulin	1.54 (0.29 to 8.33)	28 per 1000 persons	42 per 1000 persons (8 to 194 per 1000 persons)	14 more (20 fewer to 166 more)	Very low
**Hospitalisation for heart failure**	Non-steroidal MRAs	Basal insulin	0.83 (0.53 to 1.30)	28 per 1000 persons	23 per 1000 persons (15 to 36 per 1000 persons)	5 fewer (13 fewer to 8 more)	Low
**Hospitalisation for heart failure**	SGLT2 inhibitors	Basal insulin	0.70 (0.46 to 1.09)	28 per 1000 persons	20 per 1000 persons (13 to 30 per 1000 persons)	8 fewer (15 fewer to 2 more)	Low
**Hospitalisation for heart failure**	Standard treatments	Basal insulin	1.06 (0.70 to 1.61)	28 per 1000 persons	30 per 1000 persons (20 to 44 per 1000 persons)	2 more (8 fewer to 16 more)	Low
**Hospitalisation for heart failure**	Sulfonylureas	Basal insulin	1.05 (0.68 to 1.61)	28 per 1000 persons	29 per 1000 persons (19 to 44 per 1000 persons)	1 more (9 fewer to 16 more)	Moderate
**Hospitalisation for heart failure**	Thiazolidinediones	Basal insulin	1.64 (1.04 to 2.63)	28 per 1000 persons	45 per 1000 persons (29 to 70 per 1000 persons)	17 more (1 more to 42 more)	Moderate
**Hospitalisation for heart failure**	Tirzepatide	Basal insulin	0.67 (0.19 to 2.38)	28 per 1000 persons	19 per 1000 persons (5 to 64 per 1000 persons)	9 fewer (23 fewer to 36 more)	Low
**Hospitalisation for heart failure**	DPP-4 inhibitors	Bolus insulin	1.64 (0.17 to 16.67)	19 per 1000 persons	31 per 1000 persons (3 to 244 per 1000 persons)	12 more (16 fewer to 225 more)	Very low
**Hospitalisation for heart failure**	GLP-1 receptor agonists	Bolus insulin	1.41 (0.15 to 14.29)	19 per 1000 persons	27 per 1000 persons (3 to 217 per 1000 persons)	8 more (16 fewer to 198 more)	Very low
**Hospitalisation for heart failure**	Metformin	Bolus insulin	2.22 (0.14 to 33.33)	19 per 1000 persons	41 per 1000 persons (3 to 392 per 1000 persons)	22 more (16 fewer to 373 more)	Very low
**Hospitalisation for heart failure**	Non-steroidal MRAs	Bolus insulin	1.20 (0.12 to 11.11)	19 per 1000 persons	23 per 1000 persons (2 to 177 per 1000 persons)	4 more (17 fewer to 158 more)	Very low
**Hospitalisation for heart failure**	SGLT2 inhibitors	Bolus insulin	1.02 (0.11 to 10.00)	19 per 1000 persons	19 per 1000 persons (2 to 162 per 1000 persons)	0 (17 fewer to 143 more)	Very low

Outcome	Intervention	Comparator	Odds ratio	Baseline risk	Risk if treated	Risk difference	Certainty
**Hospitalisation for heart failure**	Standard treatments	Bolus insulin	1.56 (0.16 to 14.29)	19 per 1000 persons	29 per 1000 persons (3 to 217 per 1000 persons)	10 more (16 fewer to 198 more)	Very low
**Hospitalisation for heart failure**	Sulfonylureas	Bolus insulin	1.54 (0.16 to 14.29)	19 per 1000 persons	29 per 1000 persons (3 to 217 per 1000 persons)	10 more (16 fewer to 198 more)	Very low
**Hospitalisation for heart failure**	Thiazolidinediones	Bolus insulin	2.38 (0.25 to 25.00)	19 per 1000 persons	44 per 1000 persons (5 to 326 per 1000 persons)	25 more (14 fewer to 307 more)	Very low
**Hospitalisation for heart failure**	Tirzepatide	Bolus insulin	0.97 (0.07 to 14.29)	19 per 1000 persons	18 per 1000 persons (1 to 217 per 1000 persons)	1 fewer (18 fewer to 198 more)	Very low
**Hospitalisation for heart failure**	GLP-1 receptor agonists	DPP-4 inhibitors	0.86 (0.75 to 0.98)	31 per 1000 persons	27 per 1000 persons (23 to 30 per 1000 persons)	4 fewer (8 fewer to 1 fewer)	Moderate
**Hospitalisation for heart failure**	Metformin	DPP-4 inhibitors	1.37 (0.27 to 7.14)	31 per 1000 persons	42 per 1000 persons (9 to 186 per 1000 persons)	11 more (22 fewer to 155 more)	Very low
**Hospitalisation for heart failure**	Non-steroidal MRAs	DPP-4 inhibitors	0.74 (0.61 to 0.90)	31 per 1000 persons	23 per 1000 persons (19 to 28 per 1000 persons)	8 fewer (12 fewer to 3 fewer)	Low
**Hospitalisation for heart failure**	SGLT2 inhibitors	DPP-4 inhibitors	0.63 (0.55 to 0.72)	31 per 1000 persons	20 per 1000 persons (17 to 22 per 1000 persons)	11 fewer (14 fewer to 9 fewer)	Moderate
**Hospitalisation for heart failure**	Standard treatments	DPP-4 inhibitors	0.95 (0.86 to 1.05)	31 per 1000 persons	30 per 1000 persons (27 to 33 per 1000 persons)	1 fewer (4 fewer to 2 more)	Moderate
**Hospitalisation for heart failure**	Sulfonylureas	DPP-4 inhibitors	0.93 (0.76 to 1.16)	31 per 1000 persons	29 per 1000 persons (24 to 36 per 1000 persons)	2 fewer (7 fewer to 5 more)	High
**Hospitalisation for heart failure**	Thiazolidinediones	DPP-4 inhibitors	1.47 (1.18 to 1.82)	31 per 1000 persons	45 per 1000 persons (36 to 55 per 1000 persons)	14 more (5 more to 24 more)	Moderate
**Hospitalisation for heart failure**	Tirzepatide	DPP-4 inhibitors	0.60 (0.16 to 2.27)	31 per 1000 persons	19 per 1000 persons (5 to 68 per 1000 persons)	12 fewer (26 fewer to 37 more)	Very low
**Hospitalisation for heart failure**	Metformin	GLP-1 receptor agonists	1.59 (0.31 to 8.33)	27 per 1000 persons	42 per 1000 persons (9 to 188 per 1000 persons)	15 more (18 fewer to 161 more)	Low
**Hospitalisation for heart failure**	Non-steroidal MRAs	GLP-1 receptor agonists	0.86 (0.71 to 1.04)	27 per 1000 persons	23 per 1000 persons (19 to 28 per 1000 persons)	4 fewer (8 fewer to 1 more)	Moderate
**Hospitalisation for heart failure**	SGLT2 inhibitors	GLP-1 receptor agonists	0.73 (0.64 to 0.83)	27 per 1000 persons	20 per 1000 persons (17 to 23 per 1000 persons)	7 fewer (10 fewer to 4 fewer)	Moderate

Outcome	Intervention	Comparator	Odds ratio	Baseline risk	Risk if treated	Risk difference	Certainty
**Hospitalisation for heart failure**	Standard treatments	GLP-1 receptor agonists	1.10 (1.01 to 1.20)	27 per 1000 persons	30 per 1000 persons (27 to 32 per 1000 persons)	3 more (0 to 5 more)	Moderate
**Hospitalisation for heart failure**	Sulfonylureas	GLP-1 receptor agonists	1.09 (0.86 to 1.39)	27 per 1000 persons	29 per 1000 persons (23 to 37 per 1000 persons)	2 more (4 fewer to 10 more)	Moderate
**Hospitalisation for heart failure**	Thiazolidinediones	GLP-1 receptor agonists	1.69 (1.37 to 2.13)	27 per 1000 persons	45 per 1000 persons (37 to 56 per 1000 persons)	18 more (10 more to 29 more)	Moderate
**Hospitalisation for heart failure**	Tirzepatide	GLP-1 receptor agonists	0.69 (0.18 to 2.63)	27 per 1000 persons	19 per 1000 persons (5 to 68 per 1000 persons)	8 fewer (22 fewer to 41 more)	Very low
**Hospitalisation for heart failure**	Non-steroidal MRAs	Metformin	0.54 (0.10 to 2.78)	43 per 1000 persons	24 per 1000 persons (5 to 111 per 1000 persons)	19 fewer (38 fewer to 68 more)	Very low
**Hospitalisation for heart failure**	SGLT2 inhibitors	Metformin	0.46 (0.09 to 2.33)	43 per 1000 persons	20 per 1000 persons (4 to 95 per 1000 persons)	23 fewer (39 fewer to 52 more)	Very low
**Hospitalisation for heart failure**	Standard treatments	Metformin	0.69 (0.14 to 3.57)	43 per 1000 persons	30 per 1000 persons (6 to 138 per 1000 persons)	13 fewer (37 fewer to 95 more)	Very low
**Hospitalisation for heart failure**	Sulfonylureas	Metformin	0.68 (0.13 to 3.57)	43 per 1000 persons	30 per 1000 persons (6 to 138 per 1000 persons)	13 fewer (37 fewer to 95 more)	Very low
**Hospitalisation for heart failure**	Thiazolidinediones	Metformin	1.06 (0.21 to 5.56)	43 per 1000 persons	46 per 1000 persons (9 to 200 per 1000 persons)	3 more (34 fewer to 157 more)	Very low
**Hospitalisation for heart failure**	Tirzepatide	Metformin	0.43 (0.05 to 3.57)	43 per 1000 persons	19 per 1000 persons (2 to 138 per 1000 persons)	24 fewer (41 fewer to 95 more)	Very low
**Hospitalisation for heart failure**	SGLT2 inhibitors	Non-steroidal MRAs	0.85 (0.70 to 1.03)	24 per 1000 persons	20 per 1000 persons (17 to 25 per 1000 persons)	4 fewer (7 fewer to 1 more)	Moderate
**Hospitalisation for heart failure**	Standard treatments	Non-steroidal MRAs	1.28 (1.09 to 1.52)	24 per 1000 persons	31 per 1000 persons (26 to 36 per 1000 persons)	7 more (2 more to 12 more)	Low
**Hospitalisation for heart failure**	Sulfonylureas	Non-steroidal MRAs	1.27 (0.96 to 1.67)	24 per 1000 persons	30 per 1000 persons (23 to 39 per 1000 persons)	6 more (1 fewer to 15 more)	Low
**Hospitalisation for heart failure**	Thiazolidinediones	Non-steroidal MRAs	2.00 (1.54 to 2.56)	24 per 1000 persons	47 per 1000 persons (36 to 59 per 1000 persons)	23 more (12 more to 35 more)	Moderate
**Hospitalisation for heart failure**	Tirzepatide	Non-steroidal MRAs	0.81 (0.21 to 3.12)	24 per 1000 persons	19 per 1000 persons (5 to 71 per 1000 persons)	5 fewer (19 fewer to 47 more)	Very low

Outcome	Intervention	Comparator	Odds ratio	Baseline risk	Risk if treated	Risk difference	Certainty
**Hospitalisation for heart failure**	Standard treatments	SGLT2 inhibitors	1.52 (1.37 to 1.67)	20 per 1000 persons	30 per 1000 persons (27 to 33 per 1000 persons)	10 more (7 more to 13 more)	High
**Hospitalisation for heart failure**	Sulfonylureas	SGLT2 inhibitors	1.49 (1.18 to 1.89)	20 per 1000 persons	30 per 1000 persons (23 to 37 per 1000 persons)	10 more (3 more to 17 more)	Moderate
**Hospitalisation for heart failure**	Thiazolidinediones	SGLT2 inhibitors	2.33 (1.89 to 2.94)	20 per 1000 persons	45 per 1000 persons (37 to 57 per 1000 persons)	25 more (17 more to 37 more)	Moderate
**Hospitalisation for heart failure**	Tirzepatide	SGLT2 inhibitors	0.95 (0.25 to 3.57)	20 per 1000 persons	19 per 1000 persons (5 to 68 per 1000 persons)	1 fewer (15 fewer to 48 more)	Very low
**Hospitalisation for heart failure**	Standard treatments	Sulfonylureas	1.01 (0.81 to 1.27)	30 per 1000 persons	30 per 1000 persons (25 to 38 per 1000 persons)	0 (5 fewer to 8 more)	Moderate
**Hospitalisation for heart failure**	Thiazolidinediones	Sulfonylureas	1.56 (1.18 to 2.08)	30 per 1000 persons	46 per 1000 persons (35 to 61 per 1000 persons)	16 more (5 more to 31 more)	Moderate
**Hospitalisation for heart failure**	Tirzepatide	Sulfonylureas	0.64 (0.17 to 2.44)	30 per 1000 persons	19 per 1000 persons (5 to 70 per 1000 persons)	11 fewer (25 fewer to 40 more)	Very low
**Hospitalisation for heart failure**	Standard treatments	Thiazolidinediones	0.65 (0.53 to 0.79)	45 per 1000 persons	30 per 1000 persons (24 to 36 per 1000 persons)	15 fewer (21 fewer to 9 fewer)	Moderate
**Hospitalisation for heart failure**	Tirzepatide	Thiazolidinediones	0.41 (0.11 to 1.56)	45 per 1000 persons	19 per 1000 persons (5 to 69 per 1000 persons)	26 fewer (40 fewer to 24 more)	Very low
**Hospitalisation for heart failure**	Standard treatments	Tirzepatide	1.59 (0.42 to 6.25)	19 per 1000 persons	30 per 1000 persons (8 to 108 per 1000 persons)	11 more (11 fewer to 89 more)	Very low
**End-stage kidney disease**	Basal insulin	Bolus insulin	0.47 (0.02 to 12.36)	25 per 1000 persons	12 per 1000 persons (1 to 241 per 1000 persons)	13 fewer (24 fewer to 216 more)	Very low
**End-stage kidney disease**	Basal insulin	DPP-4 inhibitors	1.15 (0.59 to 2.24)	10 per 1000 persons	11 per 1000 persons (6 to 22 per 1000 persons)	1 more (4 fewer to 12 more)	Very low
**End-stage kidney disease**	Basal insulin	GLP-1 receptor agonists	1.44 (0.75 to 2.74)	8 per 1000 persons	11 per 1000 persons (6 to 22 per 1000 persons)	3 more (2 fewer to 14 more)	Very low
**End-stage kidney disease**	Basal insulin	Metformin	0.74 (0.14 to 3.83)	16 per 1000 persons	12 per 1000 persons (2 to 59 per 1000 persons)	4 fewer (14 fewer to 43 more)	Very low
**End-stage kidney disease**	Basal insulin	Non-steroidal MRAs	1.44 (0.74 to 2.78)	8 per 1000 persons	11 per 1000 persons (6 to 22 per 1000 persons)	3 more (2 fewer to 14 more)	Very low

Outcome	Intervention	Comparator	Odds ratio	Baseline risk	Risk if treated	Risk difference	Certainty
**End-stage kidney disease**	Basal insulin	SGLT2 inhibitors	1.97 (1.02 to 3.82)	6 per 1000 persons	12 per 1000 persons (6 to 23 per 1000 persons)	6 more (0 to 17 more)	Very low
**End-stage kidney disease**	Basal insulin	Standard treatments	1.20 (0.62 to 2.30)	10 per 1000 persons	12 per 1000 persons (6 to 23 per 1000 persons)	2 more (4 fewer to 13 more)	Very low
**End-stage kidney disease**	Basal insulin	Sulfonylureas	1.75 (0.72 to 4.26)	7 per 1000 persons	12 per 1000 persons (5 to 29 per 1000 persons)	5 more (2 fewer to 22 more)	Very low
**End-stage kidney disease**	Basal insulin	Thiazolidinediones	1.73 (0.70 to 4.26)	7 per 1000 persons	12 per 1000 persons (5 to 29 per 1000 persons)	5 more (2 fewer to 22 more)	Very low
**End-stage kidney disease**	Basal insulin	Tirzepatide	1.77 (0.26 to 12.27)	7 per 1000 persons	12 per 1000 persons (2 to 80 per 1000 persons)	5 more (5 fewer to 73 more)	Low
**End-stage kidney disease**	Bolus insulin	DPP-4 inhibitors	2.45 (0.10 to 60.68)	10 per 1000 persons	24 per 1000 persons (1 to 380 per 1000 persons)	14 more (9 fewer to 370 more)	Very low
**End-stage kidney disease**	Bolus insulin	GLP-1 receptor agonists	3.05 (0.12 to 75.27)	8 per 1000 persons	24 per 1000 persons (1 to 378 per 1000 persons)	16 more (7 fewer to 370 more)	Very low
**End-stage kidney disease**	Bolus insulin	Metformin	1.58 (0.05 to 54.60)	16 per 1000 persons	25 per 1000 persons (1 to 470 per 1000 persons)	9 more (15 fewer to 454 more)	Very low
**End-stage kidney disease**	Bolus insulin	Non-steroidal MRAs	3.05 (0.12 to 75.50)	8 per 1000 persons	24 per 1000 persons (1 to 378 per 1000 persons)	16 more (7 fewer to 370 more)	Very low
**End-stage kidney disease**	Bolus insulin	SGLT2 inhibitors	4.19 (0.17 to 103.67)	6 per 1000 persons	25 per 1000 persons (1 to 385 per 1000 persons)	19 more (5 fewer to 379 more)	Very low
**End-stage kidney disease**	Bolus insulin	Standard treatments	2.55 (0.10 to 62.86)	10 per 1000 persons	25 per 1000 persons (1 to 388 per 1000 persons)	15 more (9 fewer to 378 more)	Very low
**End-stage kidney disease**	Bolus insulin	Sulfonylureas	3.73 (0.14 to 97.39)	7 per 1000 persons	26 per 1000 persons (1 to 407 per 1000 persons)	19 more (6 fewer to 400 more)	Very low
**End-stage kidney disease**	Bolus insulin	Thiazolidinediones	3.68 (0.14 to 96.44)	7 per 1000 persons	25 per 1000 persons (1 to 405 per 1000 persons)	18 more (6 fewer to 398 more)	Very low
**End-stage kidney disease**	Bolus insulin	Tirzepatide	3.77 (0.09 to 161.65)	7 per 1000 persons	26 per 1000 persons (1 to 533 per 1000 persons)	19 more (6 fewer to 526 more)	Very low
**End-stage kidney disease**	DPP-4 inhibitors	GLP-1 receptor agonists	1.25 (1.07 to 1.45)	8 per 1000 persons	10 per 1000 persons (9 to 12 per 1000 persons)	2 more (1 more to 4 more)	Low

Outcome	Intervention	Comparator	Odds ratio	Baseline risk	Risk if treated	Risk difference	Certainty
**End-stage kidney disease**	DPP-4 inhibitors	Metformin	0.64 (0.14 to 2.91)	16 per 1000 persons	10 per 1000 persons (2 to 45 per 1000 persons)	6 fewer (14 fewer to 29 more)	Very low
**End-stage kidney disease**	DPP-4 inhibitors	Non-steroidal MRAs	1.24 (1.07 to 1.45)	8 per 1000 persons	10 per 1000 persons (9 to 12 per 1000 persons)	2 more (1 more to 4 more)	Low
**End-stage kidney disease**	DPP-4 inhibitors	SGLT2 inhibitors	1.71 (1.47 to 1.99)	6 per 1000 persons	10 per 1000 persons (9 to 12 per 1000 persons)	4 more (3 more to 6 more)	Low
**End-stage kidney disease**	DPP-4 inhibitors	Standard treatments	1.04 (0.93 to 1.16)	10 per 1000 persons	10 per 1000 persons (9 to 12 per 1000 persons)	0 (1 fewer to 2 more)	Moderate
**End-stage kidney disease**	DPP-4 inhibitors	Sulfonylureas	1.52 (0.84 to 2.75)	7 per 1000 persons	11 per 1000 persons (6 to 19 per 1000 persons)	4 more (1 fewer to 12 more)	Low
**End-stage kidney disease**	DPP-4 inhibitors	Thiazolidinediones	1.50 (0.81 to 2.77)	7 per 1000 persons	10 per 1000 persons (6 to 19 per 1000 persons)	3 more (1 fewer to 12 more)	Very low
**End-stage kidney disease**	DPP-4 inhibitors	Tirzepatide	1.54 (0.21 to 11.02)	7 per 1000 persons	11 per 1000 persons (1 to 72 per 1000 persons)	4 more (6 fewer to 65 more)	Very low
**End-stage kidney disease**	GLP-1 receptor agonists	Metformin	0.52 (0.11 to 2.34)	16 per 1000 persons	8 per 1000 persons (2 to 37 per 1000 persons)	8 fewer (14 fewer to 21 more)	Very low
**End-stage kidney disease**	GLP-1 receptor agonists	Non-steroidal MRAs	1.00 (0.87 to 1.15)	8 per 1000 persons	8 per 1000 persons (7 to 9 per 1000 persons)	0 (1 fewer to 1 more)	Low
**End-stage kidney disease**	GLP-1 receptor agonists	SGLT2 inhibitors	1.37 (1.19 to 1.59)	6 per 1000 persons	8 per 1000 persons (7 to 10 per 1000 persons)	2 more (1 more to 4 more)	Low
**End-stage kidney disease**	GLP-1 receptor agonists	Standard treatments	0.83 (0.75 to 0.92)	10 per 1000 persons	8 per 1000 persons (8 to 9 per 1000 persons)	2 fewer (2 fewer to 1 fewer)	Moderate
**End-stage kidney disease**	GLP-1 receptor agonists	Sulfonylureas	1.22 (0.67 to 2.24)	7 per 1000 persons	9 per 1000 persons (5 to 16 per 1000 persons)	2 more (2 fewer to 9 more)	Low
**End-stage kidney disease**	GLP-1 receptor agonists	Thiazolidinediones	1.21 (0.64 to 2.26)	7 per 1000 persons	8 per 1000 persons (4 to 16 per 1000 persons)	1 more (3 fewer to 9 more)	Low
**End-stage kidney disease**	GLP-1 receptor agonists	Tirzepatide	1.23 (0.17 to 8.80)	7 per 1000 persons	9 per 1000 persons (1 to 58 per 1000 persons)	2 more (6 fewer to 51 more)	Very low
**End-stage kidney disease**	Metformin	Non-steroidal MRAs	1.93 (0.43 to 8.71)	8 per 1000 persons	15 per 1000 persons (3 to 66 per 1000 persons)	7 more (5 fewer to 58 more)	Very low

Outcome	Intervention	Comparator	Odds ratio	Baseline risk	Risk if treated	Risk difference	Certainty
**End-stage kidney disease**	Metformin	SGLT2 inhibitors	2.65 (0.59 to 11.96)	6 per 1000 persons	16 per 1000 persons (4 to 67 per 1000 persons)	10 more (2 fewer to 61 more)	Very low
**End-stage kidney disease**	Metformin	Standard treatments	1.61 (0.36 to 7.24)	10 per 1000 persons	16 per 1000 persons (4 to 68 per 1000 persons)	6 more (6 fewer to 58 more)	Very low
**End-stage kidney disease**	Metformin	Sulfonylureas	2.36 (0.47 to 11.91)	7 per 1000 persons	16 per 1000 persons (3 to 77 per 1000 persons)	9 more (4 fewer to 70 more)	Very low
**End-stage kidney disease**	Metformin	Thiazolidinediones	2.33 (0.46 to 11.83)	7 per 1000 persons	16 per 1000 persons (3 to 77 per 1000 persons)	9 more (4 fewer to 70 more)	Very low
**End-stage kidney disease**	Metformin	Tirzepatide	2.38 (0.20 to 28.34)	7 per 1000 persons	17 per 1000 persons (1 to 167 per 1000 persons)	10 more (6 fewer to 160 more)	Very low
**End-stage kidney disease**	Non-steroidal MRAs	SGLT2 inhibitors	1.37 (1.19 to 1.58)	6 per 1000 persons	8 per 1000 persons (7 to 9 per 1000 persons)	2 more (1 more to 3 more)	Low
**End-stage kidney disease**	Non-steroidal MRAs	Standard treatments	0.83 (0.75 to 0.92)	10 per 1000 persons	8 per 1000 persons (8 to 9 per 1000 persons)	2 fewer (2 fewer to 1 fewer)	Moderate
**End-stage kidney disease**	Non-steroidal MRAs	Sulfonylureas	1.22 (0.67 to 2.24)	7 per 1000 persons	9 per 1000 persons (5 to 16 per 1000 persons)	2 more (2 fewer to 9 more)	Low
**End-stage kidney disease**	Non-steroidal MRAs	Thiazolidinediones	1.21 (0.64 to 2.26)	7 per 1000 persons	8 per 1000 persons (4 to 16 per 1000 persons)	1 more (3 fewer to 9 more)	Low
**End-stage kidney disease**	Non-steroidal MRAs	Tirzepatide	1.23 (0.17 to 8.85)	7 per 1000 persons	9 per 1000 persons (1 to 59 per 1000 persons)	2 more (6 fewer to 52 more)	Very low
**End-stage kidney disease**	SGLT2 inhibitors	Standard treatments	0.61 (0.55 to 0.67)	10 per 1000 persons	6 per 1000 persons (6 to 7 per 1000 persons)	4 fewer (4 fewer to 3 fewer)	Moderate
**End-stage kidney disease**	SGLT2 inhibitors	Sulfonylureas	0.89 (0.48 to 1.63)	7 per 1000 persons	6 per 1000 persons (3 to 11 per 1000 persons)	1 fewer (4 fewer to 4 more)	Low
**End-stage kidney disease**	SGLT2 inhibitors	Thiazolidinediones	0.88 (0.47 to 1.64)	7 per 1000 persons	6 per 1000 persons (3 to 11 per 1000 persons)	1 fewer (4 fewer to 4 more)	Low
**End-stage kidney disease**	SGLT2 inhibitors	Tirzepatide	0.90 (0.13 to 6.45)	7 per 1000 persons	6 per 1000 persons (1 to 43 per 1000 persons)	1 fewer (6 fewer to 36 more)	Very low
**End-stage kidney disease**	Sulfonylureas	Standard treatments	0.68 (0.37 to 1.24)	10 per 1000 persons	7 per 1000 persons (4 to 12 per 1000 persons)	3 fewer (6 fewer to 2 more)	Low

Outcome	Intervention	Comparator	Odds ratio	Baseline risk	Risk if treated	Risk difference	Certainty
**End-stage kidney disease**	Sulfonylureas	Thiazolidinediones	0.99 (0.82 to 1.18)	7 per 1000 persons	7 per 1000 persons (6 to 8 per 1000 persons)	0 (1 fewer to 1 more)	Low
**End-stage kidney disease**	Sulfonylureas	Tirzepatide	1.01 (0.13 to 7.90)	7 per 1000 persons	7 per 1000 persons (1 to 53 per 1000 persons)	0 (6 fewer to 46 more)	Very low
**End-stage kidney disease**	Thiazolidinediones	Standard treatments	0.69 (0.37 to 1.28)	10 per 1000 persons	7 per 1000 persons (4 to 13 per 1000 persons)	3 fewer (6 fewer to 3 more)	Low
**End-stage kidney disease**	Thiazolidinediones	Tirzepatide	1.02 (0.13 to 8.05)	7 per 1000 persons	7 per 1000 persons (1 to 54 per 1000 persons)	0 (6 fewer to 47 more)	Very low
**End-stage kidney disease**	Tirzepatide	Standard treatments	0.68 (0.09 to 4.84)	10 per 1000 persons	7 per 1000 persons (1 to 47 per 1000 persons)	3 fewer (9 fewer to 37 more)	Very low
**End-stage kidney disease**	Bolus insulin	Basal insulin	2.13 (0.08 to 50.00)	12 per 1000 persons	25 per 1000 persons (1 to 378 per 1000 persons)	13 more (11 fewer to 366 more)	Very low
**End-stage kidney disease**	DPP-4 inhibitors	Basal insulin	0.87 (0.45 to 1.69)	12 per 1000 persons	10 per 1000 persons (5 to 20 per 1000 persons)	2 fewer (7 fewer to 8 more)	Low
**End-stage kidney disease**	GLP-1 receptor agonists	Basal insulin	0.69 (0.36 to 1.33)	12 per 1000 persons	8 per 1000 persons (4 to 16 per 1000 persons)	4 fewer (8 fewer to 4 more)	Low
**End-stage kidney disease**	Metformin	Basal insulin	1.35 (0.26 to 7.14)	12 per 1000 persons	16 per 1000 persons (3 to 80 per 1000 persons)	4 more (9 fewer to 68 more)	Very low
**End-stage kidney disease**	Non-steroidal MRAs	Basal insulin	0.69 (0.36 to 1.35)	12 per 1000 persons	8 per 1000 persons (4 to 16 per 1000 persons)	4 fewer (8 fewer to 4 more)	Low
**End-stage kidney disease**	SGLT2 inhibitors	Basal insulin	0.51 (0.26 to 0.98)	12 per 1000 persons	6 per 1000 persons (3 to 12 per 1000 persons)	6 fewer (9 fewer to 0)	Low
**End-stage kidney disease**	Standard treatments	Basal insulin	0.83 (0.43 to 1.61)	12 per 1000 persons	10 per 1000 persons (5 to 19 per 1000 persons)	2 fewer (7 fewer to 7 more)	Low
**End-stage kidney disease**	Sulfonylureas	Basal insulin	0.57 (0.23 to 1.39)	12 per 1000 persons	7 per 1000 persons (3 to 17 per 1000 persons)	5 fewer (9 fewer to 5 more)	Low
**End-stage kidney disease**	Thiazolidinediones	Basal insulin	0.58 (0.23 to 1.43)	12 per 1000 persons	7 per 1000 persons (3 to 17 per 1000 persons)	5 fewer (9 fewer to 5 more)	Low
**End-stage kidney disease**	Tirzepatide	Basal insulin	0.56 (0.08 to 3.85)	12 per 1000 persons	7 per 1000 persons (1 to 45 per 1000 persons)	5 fewer (11 fewer to 33 more)	Very low

Outcome	Intervention	Comparator	Odds ratio	Baseline risk	Risk if treated	Risk difference	Certainty
**End-stage kidney disease**	DPP-4 inhibitors	Bolus insulin	0.41 (0.02 to 10.00)	25 per 1000 persons	10 per 1000 persons (0 to 204 per 1000 persons)	15 fewer (25 fewer to 179 more)	Very low
**End-stage kidney disease**	GLP-1 receptor agonists	Bolus insulin	0.33 (0.01 to 8.33)	25 per 1000 persons	8 per 1000 persons (0 to 176 per 1000 persons)	17 fewer (25 fewer to 151 more)	Very low
**End-stage kidney disease**	Metformin	Bolus insulin	0.63 (0.02 to 20.00)	25 per 1000 persons	16 per 1000 persons (0 to 339 per 1000 persons)	9 fewer (25 fewer to 314 more)	Very low
**End-stage kidney disease**	Non-steroidal MRAs	Bolus insulin	0.33 (0.01 to 8.33)	25 per 1000 persons	8 per 1000 persons (0 to 176 per 1000 persons)	17 fewer (25 fewer to 151 more)	Very low
**End-stage kidney disease**	SGLT2 inhibitors	Bolus insulin	0.24 (0.01 to 5.88)	25 per 1000 persons	6 per 1000 persons (0 to 131 per 1000 persons)	19 fewer (25 fewer to 106 more)	Very low
**End-stage kidney disease**	Standard treatments	Bolus insulin	0.39 (0.02 to 10.00)	25 per 1000 persons	10 per 1000 persons (0 to 204 per 1000 persons)	15 fewer (25 fewer to 179 more)	Very low
**End-stage kidney disease**	Sulfonylureas	Bolus insulin	0.27 (0.01 to 7.14)	25 per 1000 persons	7 per 1000 persons (0 to 155 per 1000 persons)	18 fewer (25 fewer to 130 more)	Very low
**End-stage kidney disease**	Thiazolidinediones	Bolus insulin	0.27 (0.01 to 7.14)	25 per 1000 persons	7 per 1000 persons (0 to 155 per 1000 persons)	18 fewer (25 fewer to 130 more)	Very low
**End-stage kidney disease**	Tirzepatide	Bolus insulin	0.27 (0.01 to 11.11)	25 per 1000 persons	7 per 1000 persons (0 to 222 per 1000 persons)	18 fewer (25 fewer to 197 more)	Very low
**End-stage kidney disease**	GLP-1 receptor agonists	DPP-4 inhibitors	0.80 (0.69 to 0.93)	10 per 1000 persons	8 per 1000 persons (7 to 9 per 1000 persons)	2 fewer (3 fewer to 1 fewer)	Low
**End-stage kidney disease**	Metformin	DPP-4 inhibitors	1.56 (0.34 to 7.14)	10 per 1000 persons	16 per 1000 persons (3 to 67 per 1000 persons)	6 more (7 fewer to 57 more)	Very low
**End-stage kidney disease**	Non-steroidal MRAs	DPP-4 inhibitors	0.81 (0.69 to 0.93)	10 per 1000 persons	8 per 1000 persons (7 to 9 per 1000 persons)	2 fewer (3 fewer to 1 fewer)	Low
**End-stage kidney disease**	SGLT2 inhibitors	DPP-4 inhibitors	0.58 (0.50 to 0.68)	10 per 1000 persons	6 per 1000 persons (5 to 7 per 1000 persons)	4 fewer (5 fewer to 3 fewer)	Low
**End-stage kidney disease**	Standard treatments	DPP-4 inhibitors	0.96 (0.86 to 1.08)	10 per 1000 persons	10 per 1000 persons (9 to 11 per 1000 persons)	0 (1 fewer to 1 more)	Moderate
**End-stage kidney disease**	Sulfonylureas	DPP-4 inhibitors	0.66 (0.36 to 1.19)	10 per 1000 persons	7 per 1000 persons (4 to 12 per 1000 persons)	3 fewer (6 fewer to 2 more)	Moderate

Outcome	Intervention	Comparator	Odds ratio	Baseline risk	Risk if treated	Risk difference	Certainty
**End-stage kidney disease**	Thiazolidinediones	DPP-4 inhibitors	0.67 (0.36 to 1.23)	10 per 1000 persons	7 per 1000 persons (4 to 12 per 1000 persons)	3 fewer (6 fewer to 2 more)	Low
**End-stage kidney disease**	Tirzepatide	DPP-4 inhibitors	0.65 (0.09 to 4.76)	10 per 1000 persons	7 per 1000 persons (1 to 46 per 1000 persons)	3 fewer (9 fewer to 36 more)	Very low
**End-stage kidney disease**	Metformin	GLP-1 receptor agonists	1.92 (0.43 to 9.09)	8 per 1000 persons	15 per 1000 persons (3 to 68 per 1000 persons)	7 more (5 fewer to 60 more)	Very low
**End-stage kidney disease**	Non-steroidal MRAs	GLP-1 receptor agonists	1.00 (0.87 to 1.15)	8 per 1000 persons	8 per 1000 persons (7 to 9 per 1000 persons)	0 (1 fewer to 1 more)	Low
**End-stage kidney disease**	SGLT2 inhibitors	GLP-1 receptor agonists	0.73 (0.63 to 0.84)	8 per 1000 persons	6 per 1000 persons (5 to 7 per 1000 persons)	2 fewer (3 fewer to 1 fewer)	Low
**End-stage kidney disease**	Standard treatments	GLP-1 receptor agonists	1.20 (1.09 to 1.33)	8 per 1000 persons	10 per 1000 persons (9 to 11 per 1000 persons)	2 more (1 more to 3 more)	Moderate
**End-stage kidney disease**	Sulfonylureas	GLP-1 receptor agonists	0.82 (0.45 to 1.49)	8 per 1000 persons	7 per 1000 persons (4 to 12 per 1000 persons)	1 fewer (4 fewer to 4 more)	Low
**End-stage kidney disease**	Thiazolidinediones	GLP-1 receptor agonists	0.83 (0.44 to 1.56)	8 per 1000 persons	7 per 1000 persons (4 to 12 per 1000 persons)	1 fewer (4 fewer to 4 more)	Low
**End-stage kidney disease**	Tirzepatide	GLP-1 receptor agonists	0.81 (0.11 to 5.88)	8 per 1000 persons	7 per 1000 persons (1 to 45 per 1000 persons)	1 fewer (7 fewer to 37 more)	Very low
**End-stage kidney disease**	Non-steroidal MRAs	Metformin	0.52 (0.11 to 2.33)	16 per 1000 persons	8 per 1000 persons (2 to 36 per 1000 persons)	8 fewer (14 fewer to 20 more)	Very low
**End-stage kidney disease**	SGLT2 inhibitors	Metformin	0.38 (0.08 to 1.69)	16 per 1000 persons	6 per 1000 persons (1 to 27 per 1000 persons)	10 fewer (15 fewer to 11 more)	Very low
**End-stage kidney disease**	Standard treatments	Metformin	0.62 (0.14 to 2.78)	16 per 1000 persons	10 per 1000 persons (2 to 43 per 1000 persons)	6 fewer (14 fewer to 27 more)	Very low
**End-stage kidney disease**	Sulfonylureas	Metformin	0.42 (0.08 to 2.13)	16 per 1000 persons	7 per 1000 persons (1 to 33 per 1000 persons)	9 fewer (15 fewer to 17 more)	Very low
**End-stage kidney disease**	Thiazolidinediones	Metformin	0.43 (0.08 to 2.17)	16 per 1000 persons	7 per 1000 persons (1 to 34 per 1000 persons)	9 fewer (15 fewer to 18 more)	Very low
**End-stage kidney disease**	Tirzepatide	Metformin	0.42 (0.04 to 5.00)	16 per 1000 persons	7 per 1000 persons (1 to 75 per 1000 persons)	9 fewer (15 fewer to 59 more)	Very low

Outcome	Intervention	Comparator	Odds ratio	Baseline risk	Risk if treated	Risk difference	Certainty
**End-stage kidney disease**	SGLT2 inhibitors	Non-steroidal MRAs	0.73 (0.63 to 0.84)	8 per 1000 persons	6 per 1000 persons (5 to 7 per 1000 persons)	2 fewer (3 fewer to 1 fewer)	Low
**End-stage kidney disease**	Standard treatments	Non-steroidal MRAs	1.20 (1.09 to 1.33)	8 per 1000 persons	10 per 1000 persons (9 to 11 per 1000 persons)	2 more (1 more to 3 more)	Moderate
**End-stage kidney disease**	Sulfonylureas	Non-steroidal MRAs	0.82 (0.45 to 1.49)	8 per 1000 persons	7 per 1000 persons (4 to 12 per 1000 persons)	1 fewer (4 fewer to 4 more)	Low
**End-stage kidney disease**	Thiazolidinediones	Non-steroidal MRAs	0.83 (0.44 to 1.56)	8 per 1000 persons	7 per 1000 persons (4 to 12 per 1000 persons)	1 fewer (4 fewer to 4 more)	Low
**End-stage kidney disease**	Tirzepatide	Non-steroidal MRAs	0.81 (0.11 to 5.88)	8 per 1000 persons	7 per 1000 persons (1 to 45 per 1000 persons)	1 fewer (7 fewer to 37 more)	Very low
**End-stage kidney disease**	Standard treatments	SGLT2 inhibitors	1.64 (1.49 to 1.82)	6 per 1000 persons	10 per 1000 persons (9 to 11 per 1000 persons)	4 more (3 more to 5 more)	Moderate
**End-stage kidney disease**	Sulfonylureas	SGLT2 inhibitors	1.12 (0.61 to 2.08)	6 per 1000 persons	7 per 1000 persons (4 to 12 per 1000 persons)	1 more (2 fewer to 6 more)	Low
**End-stage kidney disease**	Thiazolidinediones	SGLT2 inhibitors	1.14 (0.61 to 2.13)	6 per 1000 persons	7 per 1000 persons (4 to 13 per 1000 persons)	1 more (2 fewer to 7 more)	Low
**End-stage kidney disease**	Tirzepatide	SGLT2 inhibitors	1.11 (0.16 to 7.69)	6 per 1000 persons	7 per 1000 persons (1 to 44 per 1000 persons)	1 more (5 fewer to 38 more)	Very low
**End-stage kidney disease**	Standard treatments	Sulfonylureas	1.47 (0.81 to 2.70)	7 per 1000 persons	10 per 1000 persons (6 to 19 per 1000 persons)	3 more (1 fewer to 12 more)	Very low
**End-stage kidney disease**	Thiazolidinediones	Sulfonylureas	1.01 (0.85 to 1.22)	7 per 1000 persons	7 per 1000 persons (6 to 9 per 1000 persons)	0 (1 fewer to 2 more)	Low
**End-stage kidney disease**	Tirzepatide	Sulfonylureas	0.99 (0.13 to 7.69)	7 per 1000 persons	7 per 1000 persons (1 to 51 per 1000 persons)	0 (6 fewer to 44 more)	Very low
**End-stage kidney disease**	Standard treatments	Thiazolidinediones	1.45 (0.78 to 2.70)	7 per 1000 persons	10 per 1000 persons (5 to 19 per 1000 persons)	3 more (2 fewer to 12 more)	Very low
**End-stage kidney disease**	Tirzepatide	Thiazolidinediones	0.98 (0.12 to 7.69)	7 per 1000 persons	7 per 1000 persons (1 to 51 per 1000 persons)	0 (6 fewer to 44 more)	Very low
**End-stage kidney disease**	Standard treatments	Tirzepatide	1.47 (0.21 to 11.11)	7 per 1000 persons	10 per 1000 persons (1 to 73 per 1000 persons)	3 more (6 fewer to 66 more)	Very low

Outcome	Intervention	Comparator	Odds ratio	Baseline risk	Risk if treated	Risk difference	Certainty
**Severe hypoglycaemia**	Alpha-glucosidase inhibitors	Basal insulin	0.54 (0.13 to 2.33)	69 per 1000 persons	38 per 1000 persons (10 to 147 per 1000 persons)	31 fewer (59 fewer to 78 more)	Very low
**Severe hypoglycaemia**	Alpha-glucosidase inhibitors	Basal-Bolus insulin	0.26 (0.03 to 2.14)	133 per 1000 persons	38 per 1000 persons (5 to 247 per 1000 persons)	95 fewer (128 fewer to 114 more)	Very low
**Severe hypoglycaemia**	Alpha-glucosidase inhibitors	Bolus insulin	0.53 (0.11 to 2.52)	71 per 1000 persons	39 per 1000 persons (8 to 161 per 1000 persons)	32 fewer (63 fewer to 90 more)	Very low
**Severe hypoglycaemia**	Alpha-glucosidase inhibitors	DPP-4 inhibitors	1.17 (0.28 to 4.90)	33 per 1000 persons	38 per 1000 persons (9 to 143 per 1000 persons)	5 more (24 fewer to 110 more)	Very low
**Severe hypoglycaemia**	Alpha-glucosidase inhibitors	GLP-1 receptor agonists	1.33 (0.32 to 5.58)	29 per 1000 persons	38 per 1000 persons (9 to 143 per 1000 persons)	9 more (20 fewer to 114 more)	Very low
**Severe hypoglycaemia**	Alpha-glucosidase inhibitors	Meglitinides	0.40 (0.07 to 2.52)	90 per 1000 persons	38 per 1000 persons (7 to 200 per 1000 persons)	52 fewer (83 fewer to 110 more)	Very low
**Severe hypoglycaemia**	Alpha-glucosidase inhibitors	Metformin	0.75 (0.16 to 3.58)	51 per 1000 persons	39 per 1000 persons (9 to 161 per 1000 persons)	12 fewer (42 fewer to 110 more)	Very low
**Severe hypoglycaemia**	Alpha-glucosidase inhibitors	Non-steroidal MRAs	2.03 (0.46 to 8.98)	19 per 1000 persons	38 per 1000 persons (9 to 148 per 1000 persons)	19 more (10 fewer to 129 more)	Low
**Severe hypoglycaemia**	Alpha-glucosidase inhibitors	SGLT2 inhibitors	1.45 (0.34 to 6.07)	27 per 1000 persons	39 per 1000 persons (9 to 144 per 1000 persons)	12 more (18 fewer to 117 more)	Very low
**Severe hypoglycaemia**	Alpha-glucosidase inhibitors	Standard treatments	1.30 (0.31 to 5.43)	30 per 1000 persons	39 per 1000 persons (9 to 144 per 1000 persons)	9 more (21 fewer to 114 more)	Low
**Severe hypoglycaemia**	Alpha-glucosidase inhibitors	Sulfonylureas	0.25 (0.06 to 1.06)	139 per 1000 persons	39 per 1000 persons (10 to 146 per 1000 persons)	100 fewer (129 fewer to 7 more)	Low
**Severe hypoglycaemia**	Alpha-glucosidase inhibitors	Thiazolidinediones	0.91 (0.21 to 4.01)	42 per 1000 persons	38 per 1000 persons (9 to 150 per 1000 persons)	4 fewer (33 fewer to 108 more)	Very low
**Severe hypoglycaemia**	Alpha-glucosidase inhibitors	Tirzepatide	1.15 (0.20 to 6.56)	34 per 1000 persons	39 per 1000 persons (7 to 188 per 1000 persons)	5 more (27 fewer to 154 more)	Very low
**Severe hypoglycaemia**	Basal insulin	Basal-Bolus insulin	0.48 (0.11 to 2.22)	133 per 1000 persons	69 per 1000 persons (17 to 254 per 1000 persons)	64 fewer (116 fewer to 121 more)	Very low
**Severe hypoglycaemia**	Basal insulin	Bolus insulin	0.97 (0.49 to 1.91)	71 per 1000 persons	69 per 1000 persons (36 to 127 per 1000 persons)	2 fewer (35 fewer to 56 more)	Very low

Outcome	Intervention	Comparator	Odds ratio	Baseline risk	Risk if treated	Risk difference	Certainty
**Severe hypoglycaemia**	Basal insulin	DPP-4 inhibitors	2.14 (1.62 to 2.85)	33 per 1000 persons	68 per 1000 persons (52 to 89 per 1000 persons)	35 more (19 more to 56 more)	Moderate
**Severe hypoglycaemia**	Basal insulin	GLP-1 receptor agonists	2.44 (1.88 to 3.17)	29 per 1000 persons	68 per 1000 persons (53 to 86 per 1000 persons)	39 more (24 more to 57 more)	Moderate
**Severe hypoglycaemia**	Basal insulin	Meglitinides	0.74 (0.22 to 2.55)	90 per 1000 persons	68 per 1000 persons (21 to 201 per 1000 persons)	22 fewer (69 fewer to 111 more)	Very low
**Severe hypoglycaemia**	Basal insulin	Metformin	1.37 (0.67 to 2.80)	51 per 1000 persons	69 per 1000 persons (35 to 131 per 1000 persons)	18 more (16 fewer to 80 more)	Very low
**Severe hypoglycaemia**	Basal insulin	Non-steroidal MRAs	3.72 (2.28 to 6.08)	19 per 1000 persons	67 per 1000 persons (42 to 105 per 1000 persons)	48 more (23 more to 86 more)	Moderate
**Severe hypoglycaemia**	Basal insulin	SGLT2 inhibitors	2.66 (1.97 to 3.58)	27 per 1000 persons	69 per 1000 persons (52 to 90 per 1000 persons)	42 more (25 more to 63 more)	Moderate
**Severe hypoglycaemia**	Basal insulin	Standard treatments	2.38 (1.82 to 3.12)	30 per 1000 persons	69 per 1000 persons (53 to 88 per 1000 persons)	39 more (23 more to 58 more)	Moderate
**Severe hypoglycaemia**	Basal insulin	Sulfonylureas	0.46 (0.32 to 0.66)	139 per 1000 persons	69 per 1000 persons (49 to 96 per 1000 persons)	70 fewer (90 fewer to 43 fewer)	Moderate
**Severe hypoglycaemia**	Basal insulin	Thiazolidinediones	1.67 (1.07 to 2.63)	42 per 1000 persons	68 per 1000 persons (45 to 103 per 1000 persons)	26 more (3 more to 61 more)	Moderate
**Severe hypoglycaemia**	Basal insulin	Tirzepatide	2.12 (0.81 to 5.56)	34 per 1000 persons	69 per 1000 persons (28 to 164 per 1000 persons)	35 more (6 fewer to 130 more)	Moderate
**Severe hypoglycaemia**	Basal-Bolus insulin	Bolus insulin	2.01 (0.38 to 10.53)	71 per 1000 persons	133 per 1000 persons (28 to 446 per 1000 persons)	62 more (43 fewer to 375 more)	Very low
**Severe hypoglycaemia**	Basal-Bolus insulin	DPP-4 inhibitors	4.44 (0.95 to 20.70)	33 per 1000 persons	132 per 1000 persons (31 to 414 per 1000 persons)	99 more (2 fewer to 381 more)	Low
**Severe hypoglycaemia**	Basal-Bolus insulin	GLP-1 receptor agonists	5.06 (1.09 to 23.49)	29 per 1000 persons	131 per 1000 persons (32 to 412 per 1000 persons)	102 more (3 more to 383 more)	Low
**Severe hypoglycaemia**	Basal-Bolus insulin	Meglitinides	1.54 (0.22 to 10.84)	90 per 1000 persons	132 per 1000 persons (21 to 517 per 1000 persons)	42 more (69 fewer to 427 more)	Very low
**Severe hypoglycaemia**	Basal-Bolus insulin	Metformin	2.85 (0.54 to 15.16)	51 per 1000 persons	133 per 1000 persons (28 to 449 per 1000 persons)	82 more (23 fewer to 398 more)	Very low

Outcome	Intervention	Comparator	Odds ratio	Baseline risk	Risk if treated	Risk difference	Certainty
**Severe hypoglycaemia**	Basal-Bolus insulin	Non-steroidal MRAs	7.71 (1.57 to 37.81)	19 per 1000 persons	130 per 1000 persons (30 to 423 per 1000 persons)	111 more (11 more to 404 more)	Moderate
**Severe hypoglycaemia**	Basal-Bolus insulin	SGLT2 inhibitors	5.50 (1.18 to 25.70)	27 per 1000 persons	132 per 1000 persons (32 to 416 per 1000 persons)	105 more (5 more to 389 more)	Moderate
**Severe hypoglycaemia**	Basal-Bolus insulin	Standard treatments	4.94 (1.06 to 22.96)	30 per 1000 persons	133 per 1000 persons (32 to 415 per 1000 persons)	103 more (2 more to 385 more)	Moderate
**Severe hypoglycaemia**	Basal-Bolus insulin	Sulfonylureas	0.95 (0.20 to 4.50)	139 per 1000 persons	133 per 1000 persons (31 to 421 per 1000 persons)	6 fewer (108 fewer to 282 more)	Very low
**Severe hypoglycaemia**	Basal-Bolus insulin	Thiazolidinediones	3.47 (0.72 to 16.84)	42 per 1000 persons	132 per 1000 persons (31 to 425 per 1000 persons)	90 more (11 fewer to 383 more)	Very low
**Severe hypoglycaemia**	Basal-Bolus insulin	Tirzepatide	4.39 (0.72 to 26.62)	34 per 1000 persons	134 per 1000 persons (25 to 484 per 1000 persons)	100 more (9 fewer to 450 more)	Low
**Severe hypoglycaemia**	Bolus insulin	DPP-4 inhibitors	2.21 (1.17 to 4.20)	33 per 1000 persons	70 per 1000 persons (38 to 125 per 1000 persons)	37 more (5 more to 92 more)	Low
**Severe hypoglycaemia**	Bolus insulin	GLP-1 receptor agonists	2.52 (1.35 to 4.72)	29 per 1000 persons	70 per 1000 persons (39 to 124 per 1000 persons)	41 more (10 more to 95 more)	Low
**Severe hypoglycaemia**	Bolus insulin	Meglitinides	0.77 (0.20 to 2.99)	90 per 1000 persons	71 per 1000 persons (19 to 228 per 1000 persons)	19 fewer (71 fewer to 138 more)	Very low
**Severe hypoglycaemia**	Bolus insulin	Metformin	1.42 (0.57 to 3.54)	51 per 1000 persons	71 per 1000 persons (30 to 160 per 1000 persons)	20 more (21 fewer to 109 more)	Very low
**Severe hypoglycaemia**	Bolus insulin	Non-steroidal MRAs	3.84 (1.81 to 8.16)	19 per 1000 persons	69 per 1000 persons (34 to 136 per 1000 persons)	50 more (15 more to 117 more)	Low
**Severe hypoglycaemia**	Bolus insulin	SGLT2 inhibitors	2.74 (1.44 to 5.22)	27 per 1000 persons	71 per 1000 persons (38 to 127 per 1000 persons)	44 more (11 more to 100 more)	Moderate
**Severe hypoglycaemia**	Bolus insulin	Standard treatments	2.46 (1.31 to 4.63)	30 per 1000 persons	71 per 1000 persons (39 to 125 per 1000 persons)	41 more (9 more to 95 more)	Moderate
**Severe hypoglycaemia**	Bolus insulin	Sulfonylureas	0.47 (0.24 to 0.94)	139 per 1000 persons	71 per 1000 persons (37 to 132 per 1000 persons)	68 fewer (102 fewer to 7 fewer)	Moderate
**Severe hypoglycaemia**	Bolus insulin	Thiazolidinediones	1.73 (0.82 to 3.62)	42 per 1000 persons	70 per 1000 persons (35 to 137 per 1000 persons)	28 more (7 fewer to 95 more)	Very low

Outcome	Intervention	Comparator	Odds ratio	Baseline risk	Risk if treated	Risk difference	Certainty
**Severe hypoglycaemia**	Bolus insulin	Tirzepatide	2.19 (0.68 to 7.03)	34 per 1000 persons	72 per 1000 persons (23 to 198 per 1000 persons)	38 more (11 fewer to 164 more)	Very low
**Severe hypoglycaemia**	DPP-4 inhibitors	GLP-1 receptor agonists	1.14 (1.00 to 1.30)	29 per 1000 persons	33 per 1000 persons (29 to 37 per 1000 persons)	4 more (0 to 8 more)	Moderate
**Severe hypoglycaemia**	DPP-4 inhibitors	Meglitinides	0.35 (0.10 to 1.16)	90 per 1000 persons	33 per 1000 persons (10 to 103 per 1000 persons)	57 fewer (80 fewer to 13 more)	Very low
**Severe hypoglycaemia**	DPP-4 inhibitors	Metformin	0.64 (0.33 to 1.25)	51 per 1000 persons	33 per 1000 persons (17 to 63 per 1000 persons)	18 fewer (34 fewer to 12 more)	Very low
**Severe hypoglycaemia**	DPP-4 inhibitors	Non-steroidal MRAs	1.74 (1.14 to 2.65)	19 per 1000 persons	33 per 1000 persons (22 to 49 per 1000 persons)	14 more (3 more to 30 more)	Moderate
**Severe hypoglycaemia**	DPP-4 inhibitors	SGLT2 inhibitors	1.24 (1.05 to 1.46)	27 per 1000 persons	33 per 1000 persons (28 to 39 per 1000 persons)	6 more (1 more to 12 more)	Low
**Severe hypoglycaemia**	DPP-4 inhibitors	Standard treatments	1.11 (1.00 to 1.23)	30 per 1000 persons	33 per 1000 persons (30 to 37 per 1000 persons)	3 more (0 to 7 more)	Moderate
**Severe hypoglycaemia**	DPP-4 inhibitors	Sulfonylureas	0.21 (0.16 to 0.29)	139 per 1000 persons	33 per 1000 persons (25 to 45 per 1000 persons)	106 fewer (114 fewer to 94 fewer)	High
**Severe hypoglycaemia**	DPP-4 inhibitors	Thiazolidinediones	0.78 (0.52 to 1.16)	42 per 1000 persons	33 per 1000 persons (22 to 48 per 1000 persons)	9 fewer (20 fewer to 6 more)	Low
**Severe hypoglycaemia**	DPP-4 inhibitors	Tirzepatide	0.99 (0.37 to 2.66)	34 per 1000 persons	34 per 1000 persons (13 to 86 per 1000 persons)	0 (21 fewer to 52 more)	Very low
**Severe hypoglycaemia**	GLP-1 receptor agonists	Meglitinides	0.30 (0.09 to 1.02)	90 per 1000 persons	29 per 1000 persons (9 to 92 per 1000 persons)	61 fewer (81 fewer to 2 more)	Low
**Severe hypoglycaemia**	GLP-1 receptor agonists	Metformin	0.56 (0.29 to 1.10)	51 per 1000 persons	29 per 1000 persons (15 to 56 per 1000 persons)	22 fewer (36 fewer to 5 more)	Low
**Severe hypoglycaemia**	GLP-1 receptor agonists	Non-steroidal MRAs	1.52 (1.00 to 2.31)	19 per 1000 persons	29 per 1000 persons (19 to 43 per 1000 persons)	10 more (0 to 24 more)	Moderate
**Severe hypoglycaemia**	GLP-1 receptor agonists	SGLT2 inhibitors	1.09 (0.93 to 1.27)	27 per 1000 persons	29 per 1000 persons (25 to 34 per 1000 persons)	2 more (2 fewer to 7 more)	Moderate
**Severe hypoglycaemia**	GLP-1 receptor agonists	Standard treatments	0.98 (0.90 to 1.06)	30 per 1000 persons	29 per 1000 persons (27 to 32 per 1000 persons)	1 fewer (3 fewer to 2 more)	High

Outcome	Intervention	Comparator	Odds ratio	Baseline risk	Risk if treated	Risk difference	Certainty
**Severe hypoglycaemia**	GLP-1 receptor agonists	Sulfonylureas	0.19 (0.14 to 0.25)	139 per 1000 persons	30 per 1000 persons (22 to 39 per 1000 persons)	109 fewer (117 fewer to 100 fewer)	Very low
**Severe hypoglycaemia**	GLP-1 receptor agonists	Thiazolidinediones	0.69 (0.46 to 1.02)	42 per 1000 persons	29 per 1000 persons (20 to 43 per 1000 persons)	13 fewer (22 fewer to 1 more)	Low
**Severe hypoglycaemia**	GLP-1 receptor agonists	Tirzepatide	0.87 (0.32 to 2.32)	34 per 1000 persons	30 per 1000 persons (11 to 75 per 1000 persons)	4 fewer (23 fewer to 41 more)	Very low
**Severe hypoglycaemia**	Meglitinides	Metformin	1.85 (0.61 to 5.63)	51 per 1000 persons	90 per 1000 persons (32 to 232 per 1000 persons)	39 more (19 fewer to 181 more)	Low
**Severe hypoglycaemia**	Meglitinides	Non-steroidal MRAs	5.01 (1.40 to 17.96)	19 per 1000 persons	88 per 1000 persons (26 to 258 per 1000 persons)	69 more (7 more to 239 more)	Moderate
**Severe hypoglycaemia**	Meglitinides	SGLT2 inhibitors	3.58 (1.06 to 12.02)	27 per 1000 persons	90 per 1000 persons (29 to 250 per 1000 persons)	63 more (2 more to 223 more)	Moderate
**Severe hypoglycaemia**	Meglitinides	Standard treatments	3.21 (0.96 to 10.75)	30 per 1000 persons	90 per 1000 persons (29 to 250 per 1000 persons)	60 more (1 fewer to 220 more)	Low
**Severe hypoglycaemia**	Meglitinides	Sulfonylureas	0.62 (0.18 to 2.08)	139 per 1000 persons	91 per 1000 persons (28 to 251 per 1000 persons)	48 fewer (111 fewer to 112 more)	Very low
**Severe hypoglycaemia**	Meglitinides	Thiazolidinediones	2.26 (0.64 to 7.99)	42 per 1000 persons	90 per 1000 persons (27 to 259 per 1000 persons)	48 more (15 fewer to 217 more)	Very low
**Severe hypoglycaemia**	Meglitinides	Tirzepatide	2.85 (0.60 to 13.55)	34 per 1000 persons	91 per 1000 persons (21 to 323 per 1000 persons)	57 more (13 fewer to 289 more)	Very low
**Severe hypoglycaemia**	Metformin	Non-steroidal MRAs	2.71 (1.24 to 5.90)	19 per 1000 persons	50 per 1000 persons (23 to 103 per 1000 persons)	31 more (4 more to 84 more)	Low
**Severe hypoglycaemia**	Metformin	SGLT2 inhibitors	1.93 (0.99 to 3.78)	27 per 1000 persons	51 per 1000 persons (27 to 95 per 1000 persons)	24 more (0 to 68 more)	Moderate
**Severe hypoglycaemia**	Metformin	Standard treatments	1.73 (0.89 to 3.37)	30 per 1000 persons	51 per 1000 persons (27 to 94 per 1000 persons)	21 more (3 fewer to 64 more)	Low
**Severe hypoglycaemia**	Metformin	Sulfonylureas	0.33 (0.17 to 0.67)	139 per 1000 persons	51 per 1000 persons (27 to 98 per 1000 persons)	88 fewer (112 fewer to 41 fewer)	Moderate
**Severe hypoglycaemia**	Metformin	Thiazolidinediones	1.22 (0.57 to 2.61)	42 per 1000 persons	51 per 1000 persons (24 to 103 per 1000 persons)	9 more (18 fewer to 61 more)	Very low

Outcome	Intervention	Comparator	Odds ratio	Baseline risk	Risk if treated	Risk difference	Certainty
**Severe hypoglycaemia**	Metformin	Tirzepatide	1.54 (0.47 to 5.06)	34 per 1000 persons	51 per 1000 persons (16 to 151 per 1000 persons)	17 more (18 fewer to 117 more)	Very low
**Severe hypoglycaemia**	Non-steroidal MRAs	SGLT2 inhibitors	0.71 (0.46 to 1.10)	27 per 1000 persons	19 per 1000 persons (13 to 30 per 1000 persons)	8 fewer (14 fewer to 3 more)	Low
**Severe hypoglycaemia**	Non-steroidal MRAs	Standard treatments	0.64 (0.43 to 0.96)	30 per 1000 persons	19 per 1000 persons (13 to 29 per 1000 persons)	11 fewer (17 fewer to 1 fewer)	Moderate
**Severe hypoglycaemia**	Non-steroidal MRAs	Sulfonylureas	0.12 (0.07 to 0.20)	139 per 1000 persons	19 per 1000 persons (11 to 31 per 1000 persons)	120 fewer (128 fewer to 108 fewer)	Moderate
**Severe hypoglycaemia**	Non-steroidal MRAs	Thiazolidinediones	0.45 (0.26 to 0.79)	42 per 1000 persons	19 per 1000 persons (11 to 33 per 1000 persons)	23 fewer (31 fewer to 9 fewer)	Moderate
**Severe hypoglycaemia**	Non-steroidal MRAs	Tirzepatide	0.57 (0.20 to 1.66)	34 per 1000 persons	20 per 1000 persons (7 to 55 per 1000 persons)	14 fewer (27 fewer to 21 more)	Very low
**Severe hypoglycaemia**	SGLT2 inhibitors	Standard treatments	0.90 (0.79 to 1.02)	30 per 1000 persons	27 per 1000 persons (24 to 31 per 1000 persons)	3 fewer (6 fewer to 1 more)	High
**Severe hypoglycaemia**	SGLT2 inhibitors	Sulfonylureas	0.17 (0.13 to 0.24)	139 per 1000 persons	27 per 1000 persons (21 to 37 per 1000 persons)	112 fewer (118 fewer to 102 fewer)	Moderate
**Severe hypoglycaemia**	SGLT2 inhibitors	Thiazolidinediones	0.63 (0.42 to 0.95)	42 per 1000 persons	27 per 1000 persons (18 to 40 per 1000 persons)	15 fewer (24 fewer to 2 fewer)	Moderate
**Severe hypoglycaemia**	SGLT2 inhibitors	Tirzepatide	0.80 (0.30 to 2.16)	34 per 1000 persons	27 per 1000 persons (10 to 71 per 1000 persons)	7 fewer (24 fewer to 37 more)	Very low
**Severe hypoglycaemia**	Sulfonylureas	Standard treatments	5.22 (3.88 to 7.01)	30 per 1000 persons	139 per 1000 persons (107 to 178 per 1000 persons)	109 more (77 more to 148 more)	Moderate
**Severe hypoglycaemia**	Sulfonylureas	Thiazolidinediones	3.66 (2.31 to 5.80)	42 per 1000 persons	138 per 1000 persons (92 to 203 per 1000 persons)	96 more (50 more to 161 more)	Moderate
**Severe hypoglycaemia**	Sulfonylureas	Tirzepatide	4.64 (1.67 to 12.86)	34 per 1000 persons	140 per 1000 persons (56 to 312 per 1000 persons)	106 more (22 more to 278 more)	Moderate
**Severe hypoglycaemia**	Thiazolidinediones	Standard treatments	1.42 (0.97 to 2.10)	30 per 1000 persons	42 per 1000 persons (29 to 61 per 1000 persons)	12 more (1 fewer to 31 more)	Low
**Severe hypoglycaemia**	Thiazolidinediones	Tirzepatide	1.27 (0.44 to 3.63)	34 per 1000 persons	43 per 1000 persons (15 to 113 per 1000 persons)	9 more (19 fewer to 79 more)	Very low

Outcome	Intervention	Comparator	Odds ratio	Baseline risk	Risk if treated	Risk difference	Certainty
**Severe hypoglycaemia**	Tirzepatide	Standard treatments	1.13 (0.42 to 3.02)	30 per 1000 persons	34 per 1000 persons (13 to 85 per 1000 persons)	4 more (17 fewer to 55 more)	Very low
**Severe hypoglycaemia**	Basal insulin	Alpha-glucosidase inhibitors	1.85 (0.43 to 7.69)	39 per 1000 persons	70 per 1000 persons (17 to 238 per 1000 persons)	31 more (22 fewer to 199 more)	Very low
**Severe hypoglycaemia**	Basal-Bolus insulin	Alpha-glucosidase inhibitors	3.85 (0.47 to 33.33)	39 per 1000 persons	135 per 1000 persons (19 to 575 per 1000 persons)	96 more (20 fewer to 536 more)	Very low
**Severe hypoglycaemia**	Bolus insulin	Alpha-glucosidase inhibitors	1.89 (0.40 to 9.09)	39 per 1000 persons	71 per 1000 persons (16 to 270 per 1000 persons)	32 more (23 fewer to 231 more)	Very low
**Severe hypoglycaemia**	DPP-4 inhibitors	Alpha-glucosidase inhibitors	0.85 (0.20 to 3.57)	39 per 1000 persons	34 per 1000 persons (8 to 127 per 1000 persons)	5 fewer (31 fewer to 88 more)	Very low
**Severe hypoglycaemia**	GLP-1 receptor agonists	Alpha-glucosidase inhibitors	0.75 (0.18 to 3.12)	39 per 1000 persons	30 per 1000 persons (7 to 113 per 1000 persons)	9 fewer (32 fewer to 74 more)	Very low
**Severe hypoglycaemia**	Meglitinides	Alpha-glucosidase inhibitors	2.50 (0.40 to 14.29)	39 per 1000 persons	92 per 1000 persons (16 to 367 per 1000 persons)	53 more (23 fewer to 328 more)	Very low
**Severe hypoglycaemia**	Metformin	Alpha-glucosidase inhibitors	1.33 (0.28 to 6.25)	39 per 1000 persons	51 per 1000 persons (11 to 202 per 1000 persons)	12 more (28 fewer to 163 more)	Very low
**Severe hypoglycaemia**	Non-steroidal MRAs	Alpha-glucosidase inhibitors	0.49 (0.11 to 2.17)	39 per 1000 persons	20 per 1000 persons (4 to 81 per 1000 persons)	19 fewer (35 fewer to 42 more)	Very low
**Severe hypoglycaemia**	SGLT2 inhibitors	Alpha-glucosidase inhibitors	0.69 (0.16 to 2.94)	39 per 1000 persons	27 per 1000 persons (7 to 107 per 1000 persons)	12 fewer (32 fewer to 68 more)	Very low
**Severe hypoglycaemia**	Standard treatments	Alpha-glucosidase inhibitors	0.77 (0.18 to 3.23)	39 per 1000 persons	30 per 1000 persons (7 to 116 per 1000 persons)	9 fewer (32 fewer to 77 more)	Low
**Severe hypoglycaemia**	Sulfonylureas	Alpha-glucosidase inhibitors	4.00 (0.94 to 16.67)	39 per 1000 persons	140 per 1000 persons (37 to 403 per 1000 persons)	101 more (2 fewer to 364 more)	Low
**Severe hypoglycaemia**	Thiazolidinediones	Alpha-glucosidase inhibitors	1.10 (0.25 to 4.76)	39 per 1000 persons	43 per 1000 persons (10 to 162 per 1000 persons)	4 more (29 fewer to 123 more)	Very low
**Severe hypoglycaemia**	Tirzepatide	Alpha-glucosidase inhibitors	0.87 (0.15 to 5.00)	39 per 1000 persons	34 per 1000 persons (6 to 169 per 1000 persons)	5 fewer (33 fewer to 130 more)	Very low
**Severe hypoglycaemia**	Basal-Bolus insulin	Basal insulin	2.08 (0.45 to 9.09)	69 per 1000 persons	134 per 1000 persons (32 to 403 per 1000 persons)	65 more (37 fewer to 334 more)	Very low

Outcome	Intervention	Comparator	Odds ratio	Baseline risk	Risk if treated	Risk difference	Certainty
**Severe hypoglycaemia**	Bolus insulin	Basal insulin	1.03 (0.52 to 2.04)	69 per 1000 persons	71 per 1000 persons (37 to 131 per 1000 persons)	2 more (32 fewer to 62 more)	Very low
**Severe hypoglycaemia**	DPP-4 inhibitors	Basal insulin	0.47 (0.35 to 0.62)	69 per 1000 persons	33 per 1000 persons (25 to 44 per 1000 persons)	36 fewer (44 fewer to 25 fewer)	Moderate
**Severe hypoglycaemia**	GLP-1 receptor agonists	Basal insulin	0.41 (0.32 to 0.53)	69 per 1000 persons	29 per 1000 persons (23 to 38 per 1000 persons)	40 fewer (46 fewer to 31 fewer)	Moderate
**Severe hypoglycaemia**	Meglitinides	Basal insulin	1.35 (0.39 to 4.55)	69 per 1000 persons	91 per 1000 persons (28 to 252 per 1000 persons)	22 more (41 fewer to 183 more)	Very low
**Severe hypoglycaemia**	Metformin	Basal insulin	0.73 (0.36 to 1.49)	69 per 1000 persons	51 per 1000 persons (26 to 100 per 1000 persons)	18 fewer (43 fewer to 31 more)	Very low
**Severe hypoglycaemia**	Non-steroidal MRAs	Basal insulin	0.27 (0.16 to 0.44)	69 per 1000 persons	20 per 1000 persons (12 to 31 per 1000 persons)	49 fewer (57 fewer to 38 fewer)	Moderate
**Severe hypoglycaemia**	SGLT2 inhibitors	Basal insulin	0.38 (0.28 to 0.51)	69 per 1000 persons	27 per 1000 persons (20 to 36 per 1000 persons)	42 fewer (49 fewer to 33 fewer)	Moderate
**Severe hypoglycaemia**	Standard treatments	Basal insulin	0.42 (0.32 to 0.55)	69 per 1000 persons	30 per 1000 persons (23 to 39 per 1000 persons)	39 fewer (46 fewer to 30 fewer)	Moderate
**Severe hypoglycaemia**	Sulfonylureas	Basal insulin	2.17 (1.52 to 3.12)	69 per 1000 persons	139 per 1000 persons (101 to 188 per 1000 persons)	70 more (32 more to 119 more)	Moderate
**Severe hypoglycaemia**	Thiazolidinediones	Basal insulin	0.60 (0.38 to 0.93)	69 per 1000 persons	42 per 1000 persons (27 to 65 per 1000 persons)	27 fewer (42 fewer to 4 fewer)	Moderate
**Severe hypoglycaemia**	Tirzepatide	Basal insulin	0.47 (0.18 to 1.23)	69 per 1000 persons	34 per 1000 persons (13 to 84 per 1000 persons)	35 fewer (56 fewer to 15 more)	Low
**Severe hypoglycaemia**	Bolus insulin	Basal-Bolus insulin	0.50 (0.09 to 2.63)	133 per 1000 persons	71 per 1000 persons (14 to 288 per 1000 persons)	62 fewer (119 fewer to 155 more)	Very low
**Severe hypoglycaemia**	DPP-4 inhibitors	Basal-Bolus insulin	0.23 (0.05 to 1.05)	133 per 1000 persons	33 per 1000 persons (7 to 139 per 1000 persons)	100 fewer (126 fewer to 6 more)	Low
**Severe hypoglycaemia**	GLP-1 receptor agonists	Basal-Bolus insulin	0.20 (0.04 to 0.92)	133 per 1000 persons	29 per 1000 persons (6 to 123 per 1000 persons)	104 fewer (127 fewer to 10 fewer)	Low
**Severe hypoglycaemia**	Meglitinides	Basal-Bolus insulin	0.65 (0.09 to 4.55)	133 per 1000 persons	91 per 1000 persons (14 to 411 per 1000 persons)	42 fewer (119 fewer to 278 more)	Very low

Outcome	Intervention	Comparator	Odds ratio	Baseline risk	Risk if treated	Risk difference	Certainty
**Severe hypoglycaemia**	Metformin	Basal-Bolus insulin	0.35 (0.07 to 1.85)	133 per 1000 persons	51 per 1000 persons (10 to 221 per 1000 persons)	82 fewer (123 fewer to 88 more)	Very low
**Severe hypoglycaemia**	Non-steroidal MRAs	Basal-Bolus insulin	0.13 (0.03 to 0.64)	133 per 1000 persons	20 per 1000 persons (4 to 89 per 1000 persons)	113 fewer (129 fewer to 44 fewer)	Moderate
**Severe hypoglycaemia**	SGLT2 inhibitors	Basal-Bolus insulin	0.18 (0.04 to 0.85)	133 per 1000 persons	27 per 1000 persons (6 to 115 per 1000 persons)	106 fewer (127 fewer to 18 fewer)	Moderate
**Severe hypoglycaemia**	Standard treatments	Basal-Bolus insulin	0.20 (0.04 to 0.94)	133 per 1000 persons	30 per 1000 persons (7 to 126 per 1000 persons)	103 fewer (126 fewer to 7 fewer)	Moderate
**Severe hypoglycaemia**	Sulfonylureas	Basal-Bolus insulin	1.05 (0.22 to 5.00)	133 per 1000 persons	139 per 1000 persons (33 to 434 per 1000 persons)	6 more (100 fewer to 301 more)	Very low
**Severe hypoglycaemia**	Thiazolidinediones	Basal-Bolus insulin	0.29 (0.06 to 1.39)	133 per 1000 persons	42 per 1000 persons (9 to 176 per 1000 persons)	91 fewer (124 fewer to 43 more)	Very low
**Severe hypoglycaemia**	Tirzepatide	Basal-Bolus insulin	0.23 (0.04 to 1.39)	133 per 1000 persons	34 per 1000 persons (6 to 176 per 1000 persons)	99 fewer (127 fewer to 43 more)	Very low
**Severe hypoglycaemia**	DPP-4 inhibitors	Bolus insulin	0.45 (0.24 to 0.85)	71 per 1000 persons	33 per 1000 persons (18 to 61 per 1000 persons)	38 fewer (53 fewer to 10 fewer)	Low
**Severe hypoglycaemia**	GLP-1 receptor agonists	Bolus insulin	0.40 (0.21 to 0.74)	71 per 1000 persons	29 per 1000 persons (16 to 54 per 1000 persons)	42 fewer (55 fewer to 17 fewer)	Low
**Severe hypoglycaemia**	Meglitinides	Bolus insulin	1.30 (0.33 to 5.00)	71 per 1000 persons	90 per 1000 persons (25 to 276 per 1000 persons)	19 more (46 fewer to 205 more)	Very low
**Severe hypoglycaemia**	Metformin	Bolus insulin	0.70 (0.28 to 1.75)	71 per 1000 persons	51 per 1000 persons (21 to 118 per 1000 persons)	20 fewer (50 fewer to 47 more)	Very low
**Severe hypoglycaemia**	Non-steroidal MRAs	Bolus insulin	0.26 (0.12 to 0.55)	71 per 1000 persons	20 per 1000 persons (9 to 41 per 1000 persons)	51 fewer (62 fewer to 30 fewer)	Low
**Severe hypoglycaemia**	SGLT2 inhibitors	Bolus insulin	0.36 (0.19 to 0.69)	71 per 1000 persons	27 per 1000 persons (14 to 50 per 1000 persons)	44 fewer (57 fewer to 21 fewer)	Moderate
**Severe hypoglycaemia**	Standard treatments	Bolus insulin	0.41 (0.22 to 0.76)	71 per 1000 persons	30 per 1000 persons (16 to 55 per 1000 persons)	41 fewer (55 fewer to 16 fewer)	Moderate
**Severe hypoglycaemia**	Sulfonylureas	Bolus insulin	2.13 (1.06 to 4.17)	71 per 1000 persons	140 per 1000 persons (75 to 242 per 1000 persons)	69 more (4 more to 171 more)	Moderate

Outcome	Intervention	Comparator	Odds ratio	Baseline risk	Risk if treated	Risk difference	Certainty
**Severe hypoglycaemia**	Thiazolidinediones	Bolus insulin	0.58 (0.28 to 1.22)	71 per 1000 persons	42 per 1000 persons (21 to 85 per 1000 persons)	29 fewer (50 fewer to 14 more)	Very low
**Severe hypoglycaemia**	Tirzepatide	Bolus insulin	0.46 (0.14 to 1.47)	71 per 1000 persons	34 per 1000 persons (11 to 101 per 1000 persons)	37 fewer (60 fewer to 30 more)	Very low
**Severe hypoglycaemia**	GLP-1 receptor agonists	DPP-4 inhibitors	0.88 (0.77 to 1.00)	33 per 1000 persons	29 per 1000 persons (26 to 33 per 1000 persons)	4 fewer (7 fewer to 0)	Moderate
**Severe hypoglycaemia**	Meglitinides	DPP-4 inhibitors	2.86 (0.86 to 10.00)	33 per 1000 persons	89 per 1000 persons (29 to 254 per 1000 persons)	56 more (4 fewer to 221 more)	Low
**Severe hypoglycaemia**	Metformin	DPP-4 inhibitors	1.56 (0.80 to 3.03)	33 per 1000 persons	51 per 1000 persons (27 to 94 per 1000 persons)	18 more (6 fewer to 61 more)	Very low
**Severe hypoglycaemia**	Non-steroidal MRAs	DPP-4 inhibitors	0.57 (0.38 to 0.88)	33 per 1000 persons	19 per 1000 persons (13 to 29 per 1000 persons)	14 fewer (20 fewer to 4 fewer)	Moderate
**Severe hypoglycaemia**	SGLT2 inhibitors	DPP-4 inhibitors	0.81 (0.68 to 0.95)	33 per 1000 persons	27 per 1000 persons (23 to 31 per 1000 persons)	6 fewer (10 fewer to 2 fewer)	Moderate
**Severe hypoglycaemia**	Standard treatments	DPP-4 inhibitors	0.90 (0.81 to 1.00)	33 per 1000 persons	30 per 1000 persons (27 to 33 per 1000 persons)	3 fewer (6 fewer to 0)	Moderate
**Severe hypoglycaemia**	Sulfonylureas	DPP-4 inhibitors	4.76 (3.45 to 6.25)	33 per 1000 persons	140 per 1000 persons (105 to 176 per 1000 persons)	107 more (72 more to 143 more)	High
**Severe hypoglycaemia**	Thiazolidinediones	DPP-4 inhibitors	1.28 (0.86 to 1.92)	33 per 1000 persons	42 per 1000 persons (29 to 62 per 1000 persons)	9 more (4 fewer to 29 more)	Low
**Severe hypoglycaemia**	Tirzepatide	DPP-4 inhibitors	1.01 (0.38 to 2.70)	33 per 1000 persons	33 per 1000 persons (13 to 84 per 1000 persons)	0 (20 fewer to 51 more)	Very low
**Severe hypoglycaemia**	Meglitinides	GLP-1 receptor agonists	3.33 (0.98 to 11.11)	29 per 1000 persons	91 per 1000 persons (28 to 249 per 1000 persons)	62 more (1 fewer to 220 more)	Low
**Severe hypoglycaemia**	Metformin	GLP-1 receptor agonists	1.79 (0.91 to 3.45)	29 per 1000 persons	51 per 1000 persons (26 to 93 per 1000 persons)	22 more (3 fewer to 64 more)	Low
**Severe hypoglycaemia**	Non-steroidal MRAs	GLP-1 receptor agonists	0.66 (0.43 to 1.00)	29 per 1000 persons	19 per 1000 persons (13 to 29 per 1000 persons)	10 fewer (16 fewer to 0)	Moderate
**Severe hypoglycaemia**	SGLT2 inhibitors	GLP-1 receptor agonists	0.92 (0.79 to 1.08)	29 per 1000 persons	27 per 1000 persons (23 to 31 per 1000 persons)	2 fewer (6 fewer to 2 more)	Moderate

Outcome	Intervention	Comparator	Odds ratio	Baseline risk	Risk if treated	Risk difference	Certainty
**Severe hypoglycaemia**	Standard treatments	GLP-1 receptor agonists	1.02 (0.94 to 1.11)	29 per 1000 persons	30 per 1000 persons (27 to 32 per 1000 persons)	1 more (2 fewer to 3 more)	High
**Severe hypoglycaemia**	Sulfonylureas	GLP-1 receptor agonists	5.26 (4.00 to 7.14)	29 per 1000 persons	136 per 1000 persons (107 to 176 per 1000 persons)	107 more (78 more to 147 more)	Very low
**Severe hypoglycaemia**	Thiazolidinediones	GLP-1 receptor agonists	1.45 (0.98 to 2.17)	29 per 1000 persons	41 per 1000 persons (28 to 61 per 1000 persons)	12 more (1 fewer to 32 more)	Low
**Severe hypoglycaemia**	Tirzepatide	GLP-1 receptor agonists	1.15 (0.43 to 3.12)	29 per 1000 persons	33 per 1000 persons (13 to 85 per 1000 persons)	4 more (16 fewer to 56 more)	Very low
**Severe hypoglycaemia**	Metformin	Meglitinides	0.54 (0.18 to 1.64)	90 per 1000 persons	51 per 1000 persons (17 to 140 per 1000 persons)	39 fewer (73 fewer to 50 more)	Low
**Severe hypoglycaemia**	Non-steroidal MRAs	Meglitinides	0.20 (0.06 to 0.71)	90 per 1000 persons	19 per 1000 persons (5 to 66 per 1000 persons)	71 fewer (85 fewer to 24 fewer)	Moderate
**Severe hypoglycaemia**	SGLT2 inhibitors	Meglitinides	0.28 (0.08 to 0.94)	90 per 1000 persons	27 per 1000 persons (8 to 85 per 1000 persons)	63 fewer (82 fewer to 5 fewer)	Moderate
**Severe hypoglycaemia**	Standard treatments	Meglitinides	0.31 (0.09 to 1.04)	90 per 1000 persons	30 per 1000 persons (9 to 93 per 1000 persons)	60 fewer (81 fewer to 3 more)	Low
**Severe hypoglycaemia**	Sulfonylureas	Meglitinides	1.61 (0.48 to 5.56)	90 per 1000 persons	138 per 1000 persons (45 to 355 per 1000 persons)	48 more (45 fewer to 265 more)	Very low
**Severe hypoglycaemia**	Thiazolidinediones	Meglitinides	0.44 (0.13 to 1.56)	90 per 1000 persons	42 per 1000 persons (12 to 134 per 1000 persons)	48 fewer (78 fewer to 44 more)	Very low
**Severe hypoglycaemia**	Tirzepatide	Meglitinides	0.35 (0.07 to 1.67)	90 per 1000 persons	34 per 1000 persons (7 to 142 per 1000 persons)	56 fewer (83 fewer to 52 more)	Very low
**Severe hypoglycaemia**	Non-steroidal MRAs	Metformin	0.37 (0.17 to 0.81)	51 per 1000 persons	19 per 1000 persons (9 to 42 per 1000 persons)	32 fewer (42 fewer to 9 fewer)	Low
**Severe hypoglycaemia**	SGLT2 inhibitors	Metformin	0.52 (0.26 to 1.01)	51 per 1000 persons	27 per 1000 persons (14 to 51 per 1000 persons)	24 fewer (37 fewer to 0)	Moderate
**Severe hypoglycaemia**	Standard treatments	Metformin	0.58 (0.30 to 1.12)	51 per 1000 persons	30 per 1000 persons (16 to 57 per 1000 persons)	21 fewer (35 fewer to 6 more)	Low
**Severe hypoglycaemia**	Sulfonylureas	Metformin	3.03 (1.49 to 5.88)	51 per 1000 persons	140 per 1000 persons (74 to 240 per 1000 persons)	89 more (23 more to 189 more)	Moderate

Outcome	Intervention	Comparator	Odds ratio	Baseline risk	Risk if treated	Risk difference	Certainty
**Severe hypoglycaemia**	Thiazolidinediones	Metformin	0.82 (0.38 to 1.75)	51 per 1000 persons	42 per 1000 persons (20 to 86 per 1000 persons)	9 fewer (31 fewer to 35 more)	Very low
**Severe hypoglycaemia**	Tirzepatide	Metformin	0.65 (0.20 to 2.13)	51 per 1000 persons	34 per 1000 persons (11 to 103 per 1000 persons)	17 fewer (40 fewer to 52 more)	Very low
**Severe hypoglycaemia**	SGLT2 inhibitors	Non-steroidal MRAs	1.41 (0.91 to 2.17)	19 per 1000 persons	27 per 1000 persons (17 to 40 per 1000 persons)	8 more (2 fewer to 21 more)	Low
**Severe hypoglycaemia**	Standard treatments	Non-steroidal MRAs	1.56 (1.04 to 2.33)	19 per 1000 persons	29 per 1000 persons (20 to 43 per 1000 persons)	10 more (1 more to 24 more)	Moderate
**Severe hypoglycaemia**	Sulfonylureas	Non-steroidal MRAs	8.33 (5.00 to 14.29)	19 per 1000 persons	139 per 1000 persons (88 to 217 per 1000 persons)	120 more (69 more to 198 more)	Moderate
**Severe hypoglycaemia**	Thiazolidinediones	Non-steroidal MRAs	2.22 (1.27 to 3.85)	19 per 1000 persons	41 per 1000 persons (24 to 69 per 1000 persons)	22 more (5 more to 50 more)	Moderate
**Severe hypoglycaemia**	Tirzepatide	Non-steroidal MRAs	1.75 (0.60 to 5.00)	19 per 1000 persons	33 per 1000 persons (12 to 88 per 1000 persons)	14 more (7 fewer to 69 more)	Low
**Severe hypoglycaemia**	Standard treatments	SGLT2 inhibitors	1.11 (0.98 to 1.27)	27 per 1000 persons	30 per 1000 persons (26 to 34 per 1000 persons)	3 more (1 fewer to 7 more)	High
**Severe hypoglycaemia**	Sulfonylureas	SGLT2 inhibitors	5.88 (4.17 to 7.69)	27 per 1000 persons	140 per 1000 persons (104 to 176 per 1000 persons)	113 more (77 more to 149 more)	Moderate
**Severe hypoglycaemia**	Thiazolidinediones	SGLT2 inhibitors	1.59 (1.05 to 2.38)	27 per 1000 persons	42 per 1000 persons (28 to 62 per 1000 persons)	15 more (1 more to 35 more)	Moderate
**Severe hypoglycaemia**	Tirzepatide	SGLT2 inhibitors	1.25 (0.46 to 3.33)	27 per 1000 persons	34 per 1000 persons (13 to 85 per 1000 persons)	7 more (14 fewer to 58 more)	Very low
**Severe hypoglycaemia**	Standard treatments	Sulfonylureas	0.19 (0.14 to 0.26)	139 per 1000 persons	30 per 1000 persons (23 to 40 per 1000 persons)	109 fewer (116 fewer to 99 fewer)	Moderate
**Severe hypoglycaemia**	Thiazolidinediones	Sulfonylureas	0.27 (0.17 to 0.43)	139 per 1000 persons	42 per 1000 persons (27 to 65 per 1000 persons)	97 fewer (112 fewer to 74 fewer)	Moderate
**Severe hypoglycaemia**	Tirzepatide	Sulfonylureas	0.22 (0.08 to 0.60)	139 per 1000 persons	34 per 1000 persons (12 to 88 per 1000 persons)	105 fewer (127 fewer to 51 fewer)	Moderate
**Severe hypoglycaemia**	Standard treatments	Thiazolidinediones	0.70 (0.48 to 1.03)	42 per 1000 persons	30 per 1000 persons (20 to 43 per 1000 persons)	12 fewer (22 fewer to 1 more)	Low

Outcome	Intervention	Comparator	Odds ratio	Baseline risk	Risk if treated	Risk difference	Certainty
**Severe hypoglycaemia**	Tirzepatide	Thiazolidinediones	0.79 (0.28 to 2.27)	42 per 1000 persons	33 per 1000 persons (12 to 91 per 1000 persons)	9 fewer (30 fewer to 49 more)	Very low
**Severe hypoglycaemia**	Standard treatments	Tirzepatide	0.88 (0.33 to 2.38)	34 per 1000 persons	30 per 1000 persons (12 to 77 per 1000 persons)	4 fewer (22 fewer to 43 more)	Very low
**Severe gastrointestinal events**	Alpha-glucosidase inhibitors	Basal insulin	8.74 (0.58 to 131.74)	18 per 1000 persons	138 per 1000 persons (11 to 707 per 1000 persons)	120 more (7 fewer to 689 more)	Low
**Severe gastrointestinal events**	Alpha-glucosidase inhibitors	Bolus insulin	1.03 (0.05 to 20.56)	135 per 1000 persons	138 per 1000 persons (8 to 762 per 1000 persons)	3 more (127 fewer to 627 more)	Very low
**Severe gastrointestinal events**	Alpha-glucosidase inhibitors	DPP-4 inhibitors	2.96 (0.24 to 36.24)	51 per 1000 persons	137 per 1000 persons (13 to 661 per 1000 persons)	86 more (38 fewer to 610 more)	Very low
**Severe gastrointestinal events**	Alpha-glucosidase inhibitors	GLP-1 receptor agonists	1.72 (0.15 to 19.74)	85 per 1000 persons	138 per 1000 persons (14 to 647 per 1000 persons)	53 more (71 fewer to 562 more)	Very low
**Severe gastrointestinal events**	Alpha-glucosidase inhibitors	Metformin	1.53 (0.19 to 12.17)	95 per 1000 persons	138 per 1000 persons (20 to 561 per 1000 persons)	43 more (75 fewer to 466 more)	Very low
**Severe gastrointestinal events**	Alpha-glucosidase inhibitors	Standard treatments	3.40 (0.30 to 38.15)	45 per 1000 persons	138 per 1000 persons (14 to 643 per 1000 persons)	93 more (31 fewer to 598 more)	Very low
**Severe gastrointestinal events**	Alpha-glucosidase inhibitors	Sulfonylureas	3.43 (0.28 to 42.63)	45 per 1000 persons	139 per 1000 persons (13 to 668 per 1000 persons)	94 more (32 fewer to 623 more)	Very low
**Severe gastrointestinal events**	Alpha-glucosidase inhibitors	Thiazolidinediones	2.38 (0.22 to 25.43)	63 per 1000 persons	138 per 1000 persons (15 to 631 per 1000 persons)	75 more (48 fewer to 568 more)	Very low
**Severe gastrointestinal events**	Alpha-glucosidase inhibitors	Tirzepatide	0.74 (0.06 to 9.69)	178 per 1000 persons	138 per 1000 persons (13 to 677 per 1000 persons)	40 fewer (165 fewer to 499 more)	Very low
**Severe gastrointestinal events**	Basal insulin	Bolus insulin	0.12 (0.01 to 0.98)	135 per 1000 persons	18 per 1000 persons (2 to 133 per 1000 persons)	117 fewer (133 fewer to 2 fewer)	Low
**Severe gastrointestinal events**	Basal insulin	DPP-4 inhibitors	0.34 (0.09 to 1.34)	51 per 1000 persons	18 per 1000 persons (5 to 67 per 1000 persons)	33 fewer (46 fewer to 16 more)	Very low
**Severe gastrointestinal events**	Basal insulin	GLP-1 receptor agonists	0.20 (0.06 to 0.67)	85 per 1000 persons	18 per 1000 persons (6 to 59 per 1000 persons)	67 fewer (79 fewer to 26 fewer)	Moderate
**Severe gastrointestinal events**	Basal insulin	Metformin	0.18 (0.03 to 1.01)	95 per 1000 persons	19 per 1000 persons (3 to 96 per 1000 persons)	76 fewer (92 fewer to 1 more)	Low

Outcome	Intervention	Comparator	Odds ratio	Baseline risk	Risk if treated	Risk difference	Certainty
**Severe gastrointestinal events**	Basal insulin	Standard treatments	0.39 (0.11 to 1.36)	45 per 1000 persons	18 per 1000 persons (5 to 60 per 1000 persons)	27 fewer (40 fewer to 15 more)	Very low
**Severe gastrointestinal events**	Basal insulin	Sulfonylureas	0.39 (0.07 to 2.30)	45 per 1000 persons	18 per 1000 persons (3 to 98 per 1000 persons)	27 fewer (42 fewer to 53 more)	Very low
**Severe gastrointestinal events**	Basal insulin	Thiazolidinediones	0.27 (0.06 to 1.16)	63 per 1000 persons	18 per 1000 persons (4 to 72 per 1000 persons)	45 fewer (59 fewer to 9 more)	Low
**Severe gastrointestinal events**	Basal insulin	Tirzepatide	0.08 (0.02 to 0.30)	178 per 1000 persons	17 per 1000 persons (4 to 61 per 1000 persons)	161 fewer (174 fewer to 117 fewer)	High
**Severe gastrointestinal events**	Bolus insulin	DPP-4 inhibitors	2.88 (0.43 to 19.34)	51 per 1000 persons	134 per 1000 persons (23 to 510 per 1000 persons)	83 more (28 fewer to 459 more)	Very low
**Severe gastrointestinal events**	Bolus insulin	GLP-1 receptor agonists	1.67 (0.29 to 9.47)	85 per 1000 persons	134 per 1000 persons (26 to 468 per 1000 persons)	49 more (59 fewer to 383 more)	Very low
**Severe gastrointestinal events**	Bolus insulin	Metformin	1.48 (0.17 to 12.84)	95 per 1000 persons	134 per 1000 persons (18 to 574 per 1000 persons)	39 more (77 fewer to 479 more)	Very low
**Severe gastrointestinal events**	Bolus insulin	Standard treatments	3.30 (0.56 to 19.35)	45 per 1000 persons	135 per 1000 persons (26 to 477 per 1000 persons)	90 more (19 fewer to 432 more)	Very low
**Severe gastrointestinal events**	Bolus insulin	Sulfonylureas	3.33 (0.37 to 29.63)	45 per 1000 persons	136 per 1000 persons (17 to 583 per 1000 persons)	91 more (28 fewer to 538 more)	Very low
**Severe gastrointestinal events**	Bolus insulin	Thiazolidinediones	2.31 (0.34 to 15.78)	63 per 1000 persons	134 per 1000 persons (22 to 515 per 1000 persons)	71 more (41 fewer to 452 more)	Very low
**Severe gastrointestinal events**	Bolus insulin	Tirzepatide	0.72 (0.10 to 4.97)	178 per 1000 persons	135 per 1000 persons (21 to 518 per 1000 persons)	43 fewer (157 fewer to 340 more)	Very low
**Severe gastrointestinal events**	DPP-4 inhibitors	GLP-1 receptor agonists	0.58 (0.26 to 1.28)	85 per 1000 persons	51 per 1000 persons (24 to 106 per 1000 persons)	34 fewer (61 fewer to 21 more)	Very low
**Severe gastrointestinal events**	DPP-4 inhibitors	Metformin	0.52 (0.13 to 2.10)	95 per 1000 persons	52 per 1000 persons (13 to 181 per 1000 persons)	43 fewer (82 fewer to 86 more)	Very low
**Severe gastrointestinal events**	DPP-4 inhibitors	Standard treatments	1.15 (0.52 to 2.55)	45 per 1000 persons	51 per 1000 persons (24 to 107 per 1000 persons)	6 more (21 fewer to 62 more)	Very low
**Severe gastrointestinal events**	DPP-4 inhibitors	Sulfonylureas	1.16 (0.31 to 4.32)	45 per 1000 persons	52 per 1000 persons (14 to 169 per 1000 persons)	7 more (31 fewer to 124 more)	Very low

Outcome	Intervention	Comparator	Odds ratio	Baseline risk	Risk if treated	Risk difference	Certainty
**Severe gastrointestinal events**	DPP-4 inhibitors	Thiazolidinediones	0.80 (0.28 to 2.29)	63 per 1000 persons	51 per 1000 persons (18 to 133 per 1000 persons)	12 fewer (45 fewer to 70 more)	Very low
**Severe gastrointestinal events**	DPP-4 inhibitors	Tirzepatide	0.25 (0.08 to 0.78)	178 per 1000 persons	51 per 1000 persons (17 to 144 per 1000 persons)	127 fewer (161 fewer to 34 fewer)	Moderate
**Severe gastrointestinal events**	GLP-1 receptor agonists	Metformin	0.89 (0.25 to 3.21)	95 per 1000 persons	85 per 1000 persons (26 to 252 per 1000 persons)	10 fewer (69 fewer to 157 more)	Very low
**Severe gastrointestinal events**	GLP-1 receptor agonists	Standard treatments	1.97 (1.39 to 2.80)	45 per 1000 persons	85 per 1000 persons (61 to 117 per 1000 persons)	40 more (16 more to 72 more)	Moderate
**Severe gastrointestinal events**	GLP-1 receptor agonists	Sulfonylureas	1.99 (0.53 to 7.54)	45 per 1000 persons	86 per 1000 persons (24 to 262 per 1000 persons)	41 more (21 fewer to 217 more)	Very low
**Severe gastrointestinal events**	GLP-1 receptor agonists	Thiazolidinediones	1.38 (0.61 to 3.15)	63 per 1000 persons	85 per 1000 persons (39 to 175 per 1000 persons)	22 more (24 fewer to 112 more)	Very low
**Severe gastrointestinal events**	GLP-1 receptor agonists	Tirzepatide	0.43 (0.18 to 1.01)	178 per 1000 persons	85 per 1000 persons (38 to 179 per 1000 persons)	93 fewer (140 fewer to 1 more)	Moderate
**Severe gastrointestinal events**	Metformin	Standard treatments	2.22 (0.64 to 7.71)	45 per 1000 persons	95 per 1000 persons (29 to 266 per 1000 persons)	50 more (16 fewer to 221 more)	Very low
**Severe gastrointestinal events**	Metformin	Sulfonylureas	2.24 (0.53 to 9.39)	45 per 1000 persons	95 per 1000 persons (24 to 307 per 1000 persons)	50 more (21 fewer to 262 more)	Low
**Severe gastrointestinal events**	Metformin	Thiazolidinediones	1.56 (0.50 to 4.88)	63 per 1000 persons	95 per 1000 persons (33 to 247 per 1000 persons)	32 more (30 fewer to 184 more)	Very low
**Severe gastrointestinal events**	Metformin	Tirzepatide	0.48 (0.11 to 2.21)	178 per 1000 persons	94 per 1000 persons (23 to 324 per 1000 persons)	84 fewer (155 fewer to 146 more)	Very low
**Severe gastrointestinal events**	Sulfonylureas	Standard treatments	0.99 (0.27 to 3.68)	45 per 1000 persons	45 per 1000 persons (13 to 148 per 1000 persons)	0 (32 fewer to 103 more)	Very low
**Severe gastrointestinal events**	Sulfonylureas	Thiazolidinediones	0.70 (0.19 to 2.53)	63 per 1000 persons	45 per 1000 persons (13 to 145 per 1000 persons)	18 fewer (50 fewer to 82 more)	Low
**Severe gastrointestinal events**	Sulfonylureas	Tirzepatide	0.22 (0.05 to 1.03)	178 per 1000 persons	45 per 1000 persons (11 to 182 per 1000 persons)	133 fewer (167 fewer to 4 more)	Low
**Severe gastrointestinal events**	Thiazolidinediones	Standard treatments	1.43 (0.67 to 3.03)	45 per 1000 persons	63 per 1000 persons (31 to 125 per 1000 persons)	18 more (14 fewer to 80 more)	Low

Outcome	Intervention	Comparator	Odds ratio	Baseline risk	Risk if treated	Risk difference	Certainty
**Severe gastrointestinal events**	Thiazolidinediones	Tirzepatide	0.31 (0.10 to 0.99)	178 per 1000 persons	63 per 1000 persons (21 to 177 per 1000 persons)	115 fewer (157 fewer to 1 fewer)	Moderate
**Severe gastrointestinal events**	Tirzepatide	Standard treatments	4.59 (1.89 to 11.14)	45 per 1000 persons	178 per 1000 persons (82 to 344 per 1000 persons)	133 more (37 more to 299 more)	Moderate
**Severe gastrointestinal events**	Basal insulin	Alpha-glucosidase inhibitors	0.11 (0.01 to 1.72)	138 per 1000 persons	18 per 1000 persons (1 to 216 per 1000 persons)	120 fewer (137 fewer to 78 more)	Very low
**Severe gastrointestinal events**	Bolus insulin	Alpha-glucosidase inhibitors	0.97 (0.05 to 20.00)	138 per 1000 persons	135 per 1000 persons (8 to 762 per 1000 persons)	3 fewer (130 fewer to 624 more)	Very low
**Severe gastrointestinal events**	DPP-4 inhibitors	Alpha-glucosidase inhibitors	0.34 (0.03 to 4.17)	138 per 1000 persons	51 per 1000 persons (4 to 400 per 1000 persons)	87 fewer (134 fewer to 262 more)	Very low
**Severe gastrointestinal events**	GLP-1 receptor agonists	Alpha-glucosidase inhibitors	0.58 (0.05 to 6.67)	138 per 1000 persons	85 per 1000 persons (8 to 516 per 1000 persons)	53 fewer (130 fewer to 378 more)	Very low
**Severe gastrointestinal events**	Metformin	Alpha-glucosidase inhibitors	0.65 (0.08 to 5.26)	138 per 1000 persons	95 per 1000 persons (13 to 457 per 1000 persons)	43 fewer (125 fewer to 319 more)	Very low
**Severe gastrointestinal events**	Standard treatments	Alpha-glucosidase inhibitors	0.29 (0.03 to 3.33)	138 per 1000 persons	45 per 1000 persons (4 to 348 per 1000 persons)	93 fewer (134 fewer to 210 more)	Very low
**Severe gastrointestinal events**	Sulfonylureas	Alpha-glucosidase inhibitors	0.29 (0.02 to 3.57)	138 per 1000 persons	45 per 1000 persons (4 to 364 per 1000 persons)	93 fewer (134 fewer to 226 more)	Very low
**Severe gastrointestinal events**	Thiazolidinediones	Alpha-glucosidase inhibitors	0.42 (0.04 to 4.55)	138 per 1000 persons	63 per 1000 persons (6 to 421 per 1000 persons)	75 fewer (132 fewer to 283 more)	Very low
**Severe gastrointestinal events**	Tirzepatide	Alpha-glucosidase inhibitors	1.35 (0.10 to 16.67)	138 per 1000 persons	178 per 1000 persons (16 to 727 per 1000 persons)	40 more (122 fewer to 589 more)	Very low
**Severe gastrointestinal events**	Bolus insulin	Basal insulin	8.33 (1.02 to 100.00)	18 per 1000 persons	133 per 1000 persons (18 to 647 per 1000 persons)	115 more (0 to 629 more)	Low
**Severe gastrointestinal events**	DPP-4 inhibitors	Basal insulin	2.94 (0.75 to 11.11)	18 per 1000 persons	51 per 1000 persons (13 to 169 per 1000 persons)	33 more (5 fewer to 151 more)	Very low
**Severe gastrointestinal events**	GLP-1 receptor agonists	Basal insulin	5.00 (1.49 to 16.67)	18 per 1000 persons	84 per 1000 persons (27 to 234 per 1000 persons)	66 more (9 more to 216 more)	Moderate
**Severe gastrointestinal events**	Metformin	Basal insulin	5.56 (0.99 to 33.33)	18 per 1000 persons	92 per 1000 persons (18 to 379 per 1000 persons)	74 more (0 to 361 more)	Moderate

Outcome	Intervention	Comparator	Odds ratio	Baseline risk	Risk if treated	Risk difference	Certainty
**Severe gastrointestinal events**	Standard treatments	Basal insulin	2.56 (0.74 to 9.09)	18 per 1000 persons	45 per 1000 persons (13 to 143 per 1000 persons)	27 more (5 fewer to 125 more)	Low
**Severe gastrointestinal events**	Sulfonylureas	Basal insulin	2.56 (0.43 to 14.29)	18 per 1000 persons	45 per 1000 persons (8 to 208 per 1000 persons)	27 more (10 fewer to 190 more)	Low
**Severe gastrointestinal events**	Thiazolidinediones	Basal insulin	3.70 (0.86 to 16.67)	18 per 1000 persons	64 per 1000 persons (16 to 234 per 1000 persons)	46 more (2 fewer to 216 more)	Low
**Severe gastrointestinal events**	Tirzepatide	Basal insulin	12.50 (3.33 to 50.00)	18 per 1000 persons	186 per 1000 persons (58 to 478 per 1000 persons)	168 more (40 more to 460 more)	High
**Severe gastrointestinal events**	DPP-4 inhibitors	Bolus insulin	0.35 (0.05 to 2.33)	135 per 1000 persons	51 per 1000 persons (8 to 266 per 1000 persons)	84 fewer (127 fewer to 131 more)	Very low
**Severe gastrointestinal events**	GLP-1 receptor agonists	Bolus insulin	0.60 (0.11 to 3.45)	135 per 1000 persons	85 per 1000 persons (16 to 350 per 1000 persons)	50 fewer (119 fewer to 215 more)	Very low
**Severe gastrointestinal events**	Metformin	Bolus insulin	0.68 (0.08 to 5.88)	135 per 1000 persons	95 per 1000 persons (12 to 479 per 1000 persons)	40 fewer (123 fewer to 344 more)	Very low
**Severe gastrointestinal events**	Standard treatments	Bolus insulin	0.30 (0.05 to 1.79)	135 per 1000 persons	45 per 1000 persons (8 to 218 per 1000 persons)	90 fewer (127 fewer to 83 more)	Very low
**Severe gastrointestinal events**	Sulfonylureas	Bolus insulin	0.30 (0.03 to 2.70)	135 per 1000 persons	45 per 1000 persons (5 to 297 per 1000 persons)	90 fewer (130 fewer to 162 more)	Very low
**Severe gastrointestinal events**	Thiazolidinediones	Bolus insulin	0.43 (0.06 to 2.94)	135 per 1000 persons	63 per 1000 persons (10 to 315 per 1000 persons)	72 fewer (125 fewer to 180 more)	Very low
**Severe gastrointestinal events**	Tirzepatide	Bolus insulin	1.39 (0.20 to 10.00)	135 per 1000 persons	178 per 1000 persons (30 to 609 per 1000 persons)	43 more (105 fewer to 474 more)	Very low
**Severe gastrointestinal events**	GLP-1 receptor agonists	DPP-4 inhibitors	1.72 (0.78 to 3.85)	51 per 1000 persons	85 per 1000 persons (40 to 171 per 1000 persons)	34 more (11 fewer to 120 more)	Very low
**Severe gastrointestinal events**	Metformin	DPP-4 inhibitors	1.92 (0.48 to 7.69)	51 per 1000 persons	94 per 1000 persons (25 to 292 per 1000 persons)	43 more (26 fewer to 241 more)	Very low
**Severe gastrointestinal events**	Standard treatments	DPP-4 inhibitors	0.87 (0.39 to 1.92)	51 per 1000 persons	45 per 1000 persons (21 to 94 per 1000 persons)	6 fewer (30 fewer to 43 more)	Very low
**Severe gastrointestinal events**	Sulfonylureas	DPP-4 inhibitors	0.86 (0.23 to 3.23)	51 per 1000 persons	44 per 1000 persons (12 to 148 per 1000 persons)	7 fewer (39 fewer to 97 more)	Very low

Outcome	Intervention	Comparator	Odds ratio	Baseline risk	Risk if treated	Risk difference	Certainty
**Severe gastrointestinal events**	Thiazolidinediones	DPP-4 inhibitors	1.25 (0.44 to 3.57)	51 per 1000 persons	63 per 1000 persons (23 to 161 per 1000 persons)	12 more (28 fewer to 110 more)	Very low
**Severe gastrointestinal events**	Tirzepatide	DPP-4 inhibitors	4.00 (1.28 to 12.50)	51 per 1000 persons	177 per 1000 persons (64 to 402 per 1000 persons)	126 more (13 more to 351 more)	Moderate
**Severe gastrointestinal events**	Metformin	GLP-1 receptor agonists	1.12 (0.31 to 4.00)	85 per 1000 persons	95 per 1000 persons (28 to 271 per 1000 persons)	10 more (57 fewer to 186 more)	Very low
**Severe gastrointestinal events**	Standard treatments	GLP-1 receptor agonists	0.51 (0.36 to 0.72)	85 per 1000 persons	45 per 1000 persons (32 to 63 per 1000 persons)	40 fewer (53 fewer to 22 fewer)	Moderate
**Severe gastrointestinal events**	Sulfonylureas	GLP-1 receptor agonists	0.50 (0.13 to 1.89)	85 per 1000 persons	45 per 1000 persons (12 to 149 per 1000 persons)	40 fewer (73 fewer to 64 more)	Very low
**Severe gastrointestinal events**	Thiazolidinediones	GLP-1 receptor agonists	0.72 (0.32 to 1.64)	85 per 1000 persons	63 per 1000 persons (29 to 132 per 1000 persons)	22 fewer (56 fewer to 47 more)	Very low
**Severe gastrointestinal events**	Tirzepatide	GLP-1 receptor agonists	2.33 (0.99 to 5.56)	85 per 1000 persons	178 per 1000 persons (84 to 340 per 1000 persons)	93 more (1 fewer to 255 more)	Moderate
**Severe gastrointestinal events**	Standard treatments	Metformin	0.45 (0.13 to 1.56)	95 per 1000 persons	45 per 1000 persons (13 to 141 per 1000 persons)	50 fewer (82 fewer to 46 more)	Very low
**Severe gastrointestinal events**	Sulfonylureas	Metformin	0.45 (0.11 to 1.89)	95 per 1000 persons	45 per 1000 persons (11 to 165 per 1000 persons)	50 fewer (84 fewer to 70 more)	Low
**Severe gastrointestinal events**	Thiazolidinediones	Metformin	0.64 (0.20 to 2.00)	95 per 1000 persons	63 per 1000 persons (21 to 174 per 1000 persons)	32 fewer (74 fewer to 79 more)	Very low
**Severe gastrointestinal events**	Tirzepatide	Metformin	2.08 (0.45 to 9.09)	95 per 1000 persons	179 per 1000 persons (45 to 488 per 1000 persons)	84 more (50 fewer to 393 more)	Very low
**Severe gastrointestinal events**	Standard treatments	Sulfonylureas	1.01 (0.27 to 3.70)	45 per 1000 persons	45 per 1000 persons (13 to 149 per 1000 persons)	0 (32 fewer to 104 more)	Very low
**Severe gastrointestinal events**	Thiazolidinediones	Sulfonylureas	1.43 (0.40 to 5.26)	45 per 1000 persons	63 per 1000 persons (18 to 199 per 1000 persons)	18 more (27 fewer to 154 more)	Low
**Severe gastrointestinal events**	Tirzepatide	Sulfonylureas	4.55 (0.97 to 20.00)	45 per 1000 persons	176 per 1000 persons (44 to 485 per 1000 persons)	131 more (1 fewer to 440 more)	Low
**Severe gastrointestinal events**	Standard treatments	Thiazolidinediones	0.70 (0.33 to 1.49)	63 per 1000 persons	45 per 1000 persons (22 to 91 per 1000 persons)	18 fewer (41 fewer to 28 more)	Low

Outcome	Intervention	Comparator	Odds ratio	Baseline risk	Risk if treated	Risk difference	Certainty
**Severe gastrointestinal events**	Tirzepatide	Thiazolidinediones	3.23 (1.01 to 10.00)	63 per 1000 persons	178 per 1000 persons (64 to 402 per 1000 persons)	115 more (1 more to 339 more)	Moderate
**Severe gastrointestinal events**	Standard treatments	Tirzepatide	0.22 (0.09 to 0.53)	178 per 1000 persons	45 per 1000 persons (19 to 103 per 1000 persons)	133 fewer (159 fewer to 75 fewer)	Moderate
**Genital infection**	DPP-4 inhibitors	GLP-1 receptor agonists	1.03 (0.47 to 2.23)	52 per 1000 persons	53 per 1000 persons (25 to 109 per 1000 persons)	1 more (27 fewer to 57 more)	Very low
**Genital infection**	DPP-4 inhibitors	Metformin	0.55 (0.26 to 1.16)	93 per 1000 persons	53 per 1000 persons (26 to 106 per 1000 persons)	40 fewer (67 fewer to 13 more)	Very low
**Genital infection**	DPP-4 inhibitors	SGLT2 inhibitors	0.22 (0.14 to 0.33)	206 per 1000 persons	54 per 1000 persons (35 to 79 per 1000 persons)	152 fewer (171 fewer to 127 fewer)	Moderate
**Genital infection**	DPP-4 inhibitors	Standard treatments	0.71 (0.46 to 1.10)	73 per 1000 persons	53 per 1000 persons (35 to 80 per 1000 persons)	20 fewer (38 fewer to 7 more)	Low
**Genital infection**	DPP-4 inhibitors	Sulfonylureas	1.36 (0.80 to 2.33)	39 per 1000 persons	52 per 1000 persons (31 to 86 per 1000 persons)	13 more (8 fewer to 47 more)	Low
**Genital infection**	DPP-4 inhibitors	Thiazolidinediones	0.07 (0.00 to 1.72)	461 per 1000 persons	56 per 1000 persons (0 to 595 per 1000 persons)	405 fewer (461 fewer to 134 more)	Very low
**Genital infection**	GLP-1 receptor agonists	Metformin	0.53 (0.22 to 1.31)	93 per 1000 persons	52 per 1000 persons (22 to 118 per 1000 persons)	41 fewer (71 fewer to 25 more)	Very low
**Genital infection**	GLP-1 receptor agonists	SGLT2 inhibitors	0.21 (0.11 to 0.40)	206 per 1000 persons	52 per 1000 persons (28 to 94 per 1000 persons)	154 fewer (178 fewer to 112 fewer)	Moderate
**Genital infection**	GLP-1 receptor agonists	Standard treatments	0.69 (0.36 to 1.34)	73 per 1000 persons	52 per 1000 persons (28 to 95 per 1000 persons)	21 fewer (45 fewer to 22 more)	Very low
**Genital infection**	GLP-1 receptor agonists	Sulfonylureas	1.33 (0.64 to 2.76)	39 per 1000 persons	51 per 1000 persons (25 to 101 per 1000 persons)	12 more (14 fewer to 62 more)	Very low
**Genital infection**	GLP-1 receptor agonists	Thiazolidinediones	0.06 (0.00 to 1.73)	461 per 1000 persons	49 per 1000 persons (0 to 597 per 1000 persons)	412 fewer (461 fewer to 136 more)	Very low
**Genital infection**	Metformin	SGLT2 inhibitors	0.39 (0.21 to 0.73)	206 per 1000 persons	92 per 1000 persons (52 to 159 per 1000 persons)	114 fewer (154 fewer to 47 fewer)	High
**Genital infection**	Metformin	Standard treatments	1.30 (0.69 to 2.45)	73 per 1000 persons	93 per 1000 persons (52 to 162 per 1000 persons)	20 more (21 fewer to 89 more)	Very low

Outcome	Intervention	Comparator	Odds ratio	Baseline risk	Risk if treated	Risk difference	Certainty
**Genital infection**	Metformin	Sulfonylureas	2.49 (1.24 to 5.04)	39 per 1000 persons	92 per 1000 persons (48 to 170 per 1000 persons)	53 more (9 more to 131 more)	High
**Genital infection**	Metformin	Thiazolidinediones	0.12 (0.00 to 3.24)	461 per 1000 persons	93 per 1000 persons (0 to 735 per 1000 persons)	368 fewer (461 fewer to 274 more)	Low
**Genital infection**	SGLT2 inhibitors	Standard treatments	3.30 (2.88 to 3.78)	73 per 1000 persons	206 per 1000 persons (185 to 229 per 1000 persons)	133 more (112 more to 156 more)	High
**Genital infection**	SGLT2 inhibitors	Sulfonylureas	6.32 (4.53 to 8.82)	39 per 1000 persons	204 per 1000 persons (155 to 264 per 1000 persons)	165 more (116 more to 225 more)	High
**Genital infection**	SGLT2 inhibitors	Thiazolidinediones	0.30 (0.01 to 7.75)	461 per 1000 persons	204 per 1000 persons (8 to 869 per 1000 persons)	257 fewer (453 fewer to 408 more)	Low
**Genital infection**	Sulfonylureas	Standard treatments	0.52 (0.36 to 0.75)	73 per 1000 persons	39 per 1000 persons (28 to 56 per 1000 persons)	34 fewer (45 fewer to 17 fewer)	Low
**Genital infection**	Sulfonylureas	Thiazolidinediones	0.05 (0.00 to 1.25)	461 per 1000 persons	41 per 1000 persons (0 to 517 per 1000 persons)	420 fewer (461 fewer to 56 more)	Low
**Genital infection**	Thiazolidinediones	Standard treatments	10.85 (0.42 to 276.91)	73 per 1000 persons	461 per 1000 persons (32 to 956 per 1000 persons)	388 more (41 fewer to 883 more)	Very low
**Genital infection**	GLP-1 receptor agonists	DPP-4 inhibitors	0.97 (0.45 to 2.13)	53 per 1000 persons	52 per 1000 persons (24 to 106 per 1000 persons)	1 fewer (29 fewer to 53 more)	Very low
**Genital infection**	Metformin	DPP-4 inhibitors	1.82 (0.86 to 3.85)	53 per 1000 persons	92 per 1000 persons (46 to 177 per 1000 persons)	39 more (7 fewer to 124 more)	Low
**Genital infection**	SGLT2 inhibitors	DPP-4 inhibitors	4.55 (3.03 to 7.14)	53 per 1000 persons	203 per 1000 persons (145 to 286 per 1000 persons)	150 more (92 more to 233 more)	Moderate
**Genital infection**	Standard treatments	DPP-4 inhibitors	1.41 (0.91 to 2.17)	53 per 1000 persons	73 per 1000 persons (48 to 108 per 1000 persons)	20 more (5 fewer to 55 more)	Low
**Genital infection**	Sulfonylureas	DPP-4 inhibitors	0.74 (0.43 to 1.25)	53 per 1000 persons	40 per 1000 persons (23 to 65 per 1000 persons)	13 fewer (30 fewer to 12 more)	Very low
**Genital infection**	Thiazolidinediones	DPP-4 inhibitors	14.29 (0.58 to Inf)	53 per 1000 persons	444 per 1000 persons (32 to NaN per 1000 persons)	391 more (21 fewer to NA)	Moderate
**Genital infection**	Metformin	GLP-1 receptor agonists	1.89 (0.76 to 4.55)	52 per 1000 persons	94 per 1000 persons (40 to 200 per 1000 persons)	42 more (12 fewer to 148 more)	Very low

Outcome	Intervention	Comparator	Odds ratio	Baseline risk	Risk if treated	Risk difference	Certainty
**Genital infection**	SGLT2 inhibitors	GLP-1 receptor agonists	4.76 (2.50 to 9.09)	52 per 1000 persons	207 per 1000 persons (121 to 333 per 1000 persons)	155 more (69 more to 281 more)	Moderate
**Genital infection**	Standard treatments	GLP-1 receptor agonists	1.45 (0.75 to 2.78)	52 per 1000 persons	74 per 1000 persons (39 to 132 per 1000 persons)	22 more (13 fewer to 80 more)	Very low
**Genital infection**	Sulfonylureas	GLP-1 receptor agonists	0.75 (0.36 to 1.56)	52 per 1000 persons	40 per 1000 persons (19 to 79 per 1000 persons)	12 fewer (33 fewer to 27 more)	Very low
**Genital infection**	Thiazolidinediones	GLP-1 receptor agonists	16.67 (0.58 to Inf)	52 per 1000 persons	478 per 1000 persons (31 to NaN per 1000 persons)	426 more (21 fewer to NA)	Moderate
**Genital infection**	SGLT2 inhibitors	Metformin	2.56 (1.37 to 4.76)	93 per 1000 persons	208 per 1000 persons (123 to 328 per 1000 persons)	115 more (30 more to 235 more)	High
**Genital infection**	Standard treatments	Metformin	0.77 (0.41 to 1.45)	93 per 1000 persons	73 per 1000 persons (40 to 129 per 1000 persons)	20 fewer (53 fewer to 36 more)	Very low
**Genital infection**	Sulfonylureas	Metformin	0.40 (0.20 to 0.81)	93 per 1000 persons	40 per 1000 persons (20 to 76 per 1000 persons)	53 fewer (73 fewer to 17 fewer)	High
**Genital infection**	Thiazolidinediones	Metformin	8.33 (0.31 to Inf)	93 per 1000 persons	461 per 1000 persons (31 to NaN per 1000 persons)	368 more (62 fewer to NA)	High
**Genital infection**	Standard treatments	SGLT2 inhibitors	0.30 (0.26 to 0.35)	206 per 1000 persons	73 per 1000 persons (64 to 83 per 1000 persons)	133 fewer (142 fewer to 123 fewer)	High
**Genital infection**	Sulfonylureas	SGLT2 inhibitors	0.16 (0.11 to 0.22)	206 per 1000 persons	39 per 1000 persons (29 to 54 per 1000 persons)	167 fewer (177 fewer to 152 fewer)	High
**Genital infection**	Thiazolidinediones	SGLT2 inhibitors	3.33 (0.13 to 100.00)	206 per 1000 persons	464 per 1000 persons (32 to 963 per 1000 persons)	258 more (174 fewer to 757 more)	Low
**Genital infection**	Standard treatments	Sulfonylureas	1.92 (1.33 to 2.78)	39 per 1000 persons	72 per 1000 persons (51 to 101 per 1000 persons)	33 more (12 more to 62 more)	Low
**Genital infection**	Thiazolidinediones	Sulfonylureas	20.00 (0.80 to Inf)	39 per 1000 persons	448 per 1000 persons (31 to NaN per 1000 persons)	409 more (8 fewer to NA)	High
**Genital infection**	Standard treatments	Thiazolidinediones	0.09 (0.00 to 2.38)	461 per 1000 persons	73 per 1000 persons (3 to 671 per 1000 persons)	388 fewer (458 fewer to 210 more)	Very low
**Amputation**	DPP-4 inhibitors	GLP-1 receptor agonists	1.28 (0.12 to 13.54)	7 per 1000 persons	9 per 1000 persons (1 to 87 per 1000 persons)	2 more (6 fewer to 80 more)	Low

Outcome	Intervention	Comparator	Odds ratio	Baseline risk	Risk if treated	Risk difference	Certainty
**Amputation**	DPP-4 inhibitors	Non-steroidal MRAs	0.97 (0.37 to 2.55)	9 per 1000 persons	9 per 1000 persons (3 to 23 per 1000 persons)	0 (6 fewer to 14 more)	Low
**Amputation**	DPP-4 inhibitors	SGLT2 inhibitors	0.71 (0.39 to 1.31)	13 per 1000 persons	9 per 1000 persons (5 to 17 per 1000 persons)	4 fewer (8 fewer to 4 more)	Moderate
**Amputation**	DPP-4 inhibitors	Standard treatments	0.91 (0.51 to 1.60)	10 per 1000 persons	9 per 1000 persons (5 to 16 per 1000 persons)	1 fewer (5 fewer to 6 more)	High
**Amputation**	DPP-4 inhibitors	Thiazolidinediones	1.24 (0.54 to 2.83)	7 per 1000 persons	9 per 1000 persons (4 to 20 per 1000 persons)	2 more (3 fewer to 13 more)	Low
**Amputation**	GLP-1 receptor agonists	Non-steroidal MRAs	0.76 (0.07 to 8.48)	9 per 1000 persons	7 per 1000 persons (1 to 72 per 1000 persons)	2 fewer (8 fewer to 63 more)	Low
**Amputation**	GLP-1 receptor agonists	SGLT2 inhibitors	0.55 (0.06 to 5.52)	13 per 1000 persons	7 per 1000 persons (1 to 68 per 1000 persons)	6 fewer (12 fewer to 55 more)	Very low
**Amputation**	GLP-1 receptor agonists	Standard treatments	0.71 (0.07 to 6.95)	10 per 1000 persons	7 per 1000 persons (1 to 66 per 1000 persons)	3 fewer (9 fewer to 56 more)	Moderate
**Amputation**	GLP-1 receptor agonists	Thiazolidinediones	0.96 (0.09 to 10.26)	7 per 1000 persons	7 per 1000 persons (1 to 67 per 1000 persons)	0 (6 fewer to 60 more)	Low
**Amputation**	Non-steroidal MRAs	SGLT2 inhibitors	0.73 (0.32 to 1.65)	13 per 1000 persons	10 per 1000 persons (4 to 21 per 1000 persons)	3 fewer (9 fewer to 8 more)	Moderate
**Amputation**	Non-steroidal MRAs	Standard treatments	0.93 (0.43 to 2.03)	10 per 1000 persons	9 per 1000 persons (4 to 20 per 1000 persons)	1 fewer (6 fewer to 10 more)	Moderate
**Amputation**	Non-steroidal MRAs	Thiazolidinediones	1.27 (0.48 to 3.40)	7 per 1000 persons	9 per 1000 persons (3 to 23 per 1000 persons)	2 more (4 fewer to 16 more)	Low
**Amputation**	SGLT2 inhibitors	Standard treatments	1.27 (1.01 to 1.61)	10 per 1000 persons	13 per 1000 persons (10 to 16 per 1000 persons)	3 more (0 to 6 more)	Moderate
**Amputation**	SGLT2 inhibitors	Thiazolidinediones	1.74 (0.91 to 3.31)	7 per 1000 persons	12 per 1000 persons (6 to 23 per 1000 persons)	5 more (1 fewer to 16 more)	Low
**Amputation**	Thiazolidinediones	Standard treatments	0.73 (0.40 to 1.33)	10 per 1000 persons	7 per 1000 persons (4 to 13 per 1000 persons)	3 fewer (6 fewer to 3 more)	Moderate
**Amputation**	GLP-1 receptor agonists	DPP-4 inhibitors	0.78 (0.07 to 8.33)	9 per 1000 persons	7 per 1000 persons (1 to 70 per 1000 persons)	2 fewer (8 fewer to 61 more)	Low

Outcome	Intervention	Comparator	Odds ratio	Baseline risk	Risk if treated	Risk difference	Certainty
**Amputation**	Non-steroidal MRAs	DPP-4 inhibitors	1.03 (0.39 to 2.70)	9 per 1000 persons	9 per 1000 persons (4 to 24 per 1000 persons)	0 (5 fewer to 15 more)	Low
**Amputation**	SGLT2 inhibitors	DPP-4 inhibitors	1.41 (0.76 to 2.56)	9 per 1000 persons	13 per 1000 persons (7 to 23 per 1000 persons)	4 more (2 fewer to 14 more)	Low
**Amputation**	Standard treatments	DPP-4 inhibitors	1.10 (0.62 to 1.96)	9 per 1000 persons	10 per 1000 persons (6 to 17 per 1000 persons)	1 more (3 fewer to 8 more)	High
**Amputation**	Thiazolidinediones	DPP-4 inhibitors	0.81 (0.35 to 1.85)	9 per 1000 persons	7 per 1000 persons (3 to 17 per 1000 persons)	2 fewer (6 fewer to 8 more)	Moderate
**Amputation**	Non-steroidal MRAs	GLP-1 receptor agonists	1.32 (0.12 to 14.29)	7 per 1000 persons	9 per 1000 persons (1 to 91 per 1000 persons)	2 more (6 fewer to 84 more)	Low
**Amputation**	SGLT2 inhibitors	GLP-1 receptor agonists	1.82 (0.18 to 16.67)	7 per 1000 persons	13 per 1000 persons (1 to 105 per 1000 persons)	6 more (6 fewer to 98 more)	Low
**Amputation**	Standard treatments	GLP-1 receptor agonists	1.41 (0.14 to 14.29)	7 per 1000 persons	10 per 1000 persons (1 to 91 per 1000 persons)	3 more (6 fewer to 84 more)	Moderate
**Amputation**	Thiazolidinediones	GLP-1 receptor agonists	1.04 (0.10 to 11.11)	7 per 1000 persons	7 per 1000 persons (1 to 73 per 1000 persons)	0 (6 fewer to 66 more)	Low
**Amputation**	SGLT2 inhibitors	Non-steroidal MRAs	1.37 (0.61 to 3.12)	9 per 1000 persons	12 per 1000 persons (5 to 28 per 1000 persons)	3 more (4 fewer to 19 more)	Low
**Amputation**	Standard treatments	Non-steroidal MRAs	1.08 (0.49 to 2.33)	9 per 1000 persons	10 per 1000 persons (4 to 21 per 1000 persons)	1 more (5 fewer to 12 more)	Low
**Amputation**	Thiazolidinediones	Non-steroidal MRAs	0.79 (0.29 to 2.08)	9 per 1000 persons	7 per 1000 persons (3 to 19 per 1000 persons)	2 fewer (6 fewer to 10 more)	Moderate
**Amputation**	Standard treatments	SGLT2 inhibitors	0.79 (0.62 to 0.99)	13 per 1000 persons	10 per 1000 persons (8 to 13 per 1000 persons)	3 fewer (5 fewer to 0)	Moderate
**Amputation**	Thiazolidinediones	SGLT2 inhibitors	0.57 (0.30 to 1.10)	13 per 1000 persons	8 per 1000 persons (4 to 14 per 1000 persons)	5 fewer (9 fewer to 1 more)	Moderate
**Amputation**	Standard treatments	Thiazolidinediones	1.37 (0.75 to 2.50)	7 per 1000 persons	10 per 1000 persons (5 to 17 per 1000 persons)	3 more (2 fewer to 10 more)	Moderate
**Ketoacidosis due to diabetes**	Alpha-glucosidase inhibitors	DPP-4 inhibitors	0.49 (0.01 to 19.75)	2 per 1000 persons	1 per 1000 persons (0 to 38 per 1000 persons)	1 fewer (2 fewer to 36 more)	Very low

Outcome	Intervention	Comparator	Odds ratio	Baseline risk	Risk if treated	Risk difference	Certainty
**Ketoacidosis due to diabetes**	Alpha-glucosidase inhibitors	GLP-1 receptor agonists	0.38 (0.01 to 16.39)	2 per 1000 persons	1 per 1000 persons (0 to 32 per 1000 persons)	1 fewer (2 fewer to 30 more)	Low
**Ketoacidosis due to diabetes**	Alpha-glucosidase inhibitors	Meglitinides	1.01 (0.02 to 51.04)	1 per 1000 persons	1 per 1000 persons (0 to 49 per 1000 persons)	0 (1 fewer to 48 more)	Very low
**Ketoacidosis due to diabetes**	Alpha-glucosidase inhibitors	Metformin	0.29 (0.00 to 39.24)	3 per 1000 persons	1 per 1000 persons (0 to 106 per 1000 persons)	2 fewer (3 fewer to 103 more)	Low
**Ketoacidosis due to diabetes**	Alpha-glucosidase inhibitors	Non-steroidal MRAs	0.58 (0.01 to 26.32)	1 per 1000 persons	1 per 1000 persons (0 to 26 per 1000 persons)	0 (1 fewer to 25 more)	Very low
**Ketoacidosis due to diabetes**	Alpha-glucosidase inhibitors	SGLT2 inhibitors	0.19 (0.00 to 7.97)	4 per 1000 persons	1 per 1000 persons (0 to 31 per 1000 persons)	3 fewer (4 fewer to 27 more)	Very low
**Ketoacidosis due to diabetes**	Alpha-glucosidase inhibitors	Standard treatments	0.40 (0.01 to 16.47)	2 per 1000 persons	1 per 1000 persons (0 to 32 per 1000 persons)	1 fewer (2 fewer to 30 more)	Very low
**Ketoacidosis due to diabetes**	Alpha-glucosidase inhibitors	Sulfonylureas	0.67 (0.03 to 16.50)	1 per 1000 persons	1 per 1000 persons (0 to 16 per 1000 persons)	0 (1 fewer to 15 more)	Very low
**Ketoacidosis due to diabetes**	DPP-4 inhibitors	GLP-1 receptor agonists	0.78 (0.32 to 1.89)	2 per 1000 persons	2 per 1000 persons (1 to 4 per 1000 persons)	0 (1 fewer to 2 more)	Moderate
[truncated: 481,181 more chars]
